# Brain tissue electrical conductivity as a promising biomarker for dementia assessment using MRI

**DOI:** 10.1002/alz.70270

**Published:** 2025-06-23

**Authors:** Jiayue Chu, Junye Yao, Zhenghao Li, Jun Li, Yuyao Zhang, Chunlei Liu, Hongjian He, Binyin Li, Hongjiang Wei

**Affiliations:** ^1^ School of Biomedical Engineering Shanghai Jiao Tong University Shanghai China; ^2^ Center for Brain Imaging Science and Technology Zhejiang University Hangzhou China; ^3^ College of Biomedical Engineering and Instrument Science Zhejiang University Hangzhou China; ^4^ School of Information Science and Technology ShanghaiTech University Shanghai China; ^5^ Department of Electrical Engineering and Computer Sciences University of California Berkeley California USA; ^6^ School of Physics Zhejiang University Hangzhou China; ^7^ State Key Laboratory of Brain‐Machine Intelligence Zhejiang University Hangzhou China; ^8^ Department of Neurology & Institute of Neurology Ruijin Hospital affiliated with Shanghai Jiao Tong University School of Medicine Shanghai China; ^9^ Clinical Neuroscience Center Ruijin Hospital LuWan Branch Shanghai Jiao Tong University School of Medicine Shanghai China; ^10^ National Engineering Research Center of Advanced Magnetic Resonance Technologies for Diagnosis and Therapy (NERC‐AMRT) Shanghai Jiao Tong University Shanghai China

**Keywords:** dementia, electrical conductivity, MRI, positron emission tomography, PET, protein aggregation

## Abstract

**INTRODUCTION:**

Dementia, particularly Alzheimer's disease, involves cognitive decline linked to amyloid beta (Aβ) and tau protein aggregation. Magnetic resonance imaging (MRI)‐based brain tissue conductivity, which increases in dementia, may serve as a non‐invasive biomarker for protein aggregation. We investigate the relationship between MRI‐based brain electrical conductivity, protein aggregation, cognition, and gene expression.

**METHODS:**

Brain conductivity maps were reconstructed and correlated with PET protein signals, cognitive performance, and plasma protein levels. The diagnostic potential of conductivity for dementia was assessed, and transcriptomic analysis using the Allen Human Brain Atlas elucidated the underlying biological processes.

**RESULTS:**

Increased brain conductivity was associated with Aβ and tau aggregation in specific brain regions, cognitive decline, and plasma protein levels. Conductivity also improved dementia discrimination performance, and higher gene expression related to ion transport, cellular development, and signaling pathways was observed.

**DISCUSSION:**

Brain electrical conductivity shows promise as a biomarker for dementia, correlating with protein aggregation and relevant cellular processes.

**Highlights:**

- Brain tissue conductivity correlates with Aβ and tau aggregation in dementia.
- Brain tissue conductivity correlates with cognitive scores and GMV.
- CSF conductivity correlates with plasma protein levels.
- Combining conductivity with GMV improves dementia diagnosis accuracy.
- Gene expression in ion processes, cell development, and signaling links to conductivity.

## 1 BACKGROUND

Alzheimer's disease (AD) is a progressive neurodegenerative disorder characterized by brain deterioration, leading to cognitive decline and dementia.^1^ Protein aggregation, mainly involving amyloid beta (Aβ) and tau plaques, is a key pathological factor in the development of AD,^2^, ^3^ initiating toxic processes such as oxidative stress, neuroinflammation, ion imbalance, and iron deposition, which result in neuronal dysfunction and cell death.^4^, ^5^, ^6^

In clinical practice, Aβ‐positron emission computed tomography (PET) and tau‐PET are the primary methods for assessing the risk and diagnosis of AD and its progression, with Aβ‐PET being particularly emphasized. However, these techniques have limitations, including exposure to ionizing radiation, limited resolution, and high costs.^7^, ^8^, ^9^ Alternatively, magnetic resonance imaging (MRI) offers a non‐invasive and cost‐effective approach to investigating brain alterations in AD. It detects the downstream effects of protein aggregation through various contrast mechanisms. Previous studies have explored the interaction between protein aggregation using PET and iron deposition using MRI,^10^, ^11^ and the results indicate that there is a certain degree of correlation between the two.^12^, ^13^ However, the interpretation of MRI signals in AD remains complex, as they may reflect multiple pathological processes, including but not limited to protein aggregation. Further research is needed to better understand how different MRI‐derived parameters, including electrical conductivity, relate to the complex pathophysiology of AD and its various biomarkers.

AD is associated with impaired glutamate clearance and reduced Na^+^/K^+^ ATPase levels, leading to a cellular ion imbalance. Previous studies reported significantly elevated Na^+^/K^+^ levels in certain cortical regions of AD samples compared to controls, potentially induced by protein aggregation.^14^ Recent research has shown that brain tissue conductivity measured by MRI is significantly higher in dementia patients than in healthy individuals and those with mild cognitive impairment (MCI).^15^ Moreover, brain tissue conductivity is negatively correlated with the Mini‐Mental State Examination (MMSE) cognitive score. It is hypothesized that a major underlying mechanism may involve protein aggregation in the brains of AD patients, leading to increased concentrations of Na^+^, K^+^, and Ca^2+^ ions, which in turn raise tissue conductivity.^5^, ^16^ This suggests a potential link between brain tissue conductivity and protein aggregation, making conductivity a possible indicator of protein aggregation in the brain. However, the relationship between brain tissue conductivity and protein aggregation has not yet been thoroughly investigated.

This study primarily investigated the relationship between brain tissue conductivity and PET protein signals, cognition, and peripheral plasma protein levels. We focused on (1) determining whether the regions with abnormal conductivity in dementia aligned spatially with areas of protein (Aβ, tau) aggregation; (2) examining the correlation between conductivity and PET protein signals, as well as plasma protein levels; and (3) assessing the effectiveness of electrical conductivity in discriminating dementia. To further understand the molecular mechanisms underlying these pathologies, we analyzed gene expression using transcriptomic data from the Allen Human Brain Atlas.^17^ Using partial least squares (PLS) regression and Gene Ontology (GO) tools,^18^, ^19^, ^20^ we identified biological processes associated with this transcriptomic profile and evaluated the correlation between gene expression, cortical protein aggregation, and tissue conductivity in dementia. This analysis provides insights into the gene expression profiles that may predispose specific cortical regions to increased protein aggregation and tissue conductivity, potentially contributing to neurodegeneration.

## 2 METHODS

The study received approval from the ethics committee of Ruijin Hospital, Shanghai Jiao Tong University School of Medicine, China. Written informed consent was obtained from all participants or their caregivers after they were fully informed about the procedures involved. We confirm that the study adhered to the ethical standards of the Declaration of Helsinki and its subsequent amendments. The study is registered with ClinicalTrials.gov under the identifier NCT05623124.

### 2.1 Participants and study design

Data were obtained from the Ruijin Neurobank of Alzheimer's Disease and Dementia (RJNB‐D) cohort study, an ongoing prospective observational study focused on neuroimaging to enhance our understanding of the risk factors, causes, and clinical outcomes of dementia. The criteria for dementia and MCI were determined by clinical features measured by the MMSE (Chinese version).^21^

The methodology approach of this study is illustrated in Figure S1. We included 97 participants based on the availability and sufficient quality of T1‐weighted (T1w) images, gradient‐recalled echo (GRE) from MRI, and PET scans. All participants were assessed by MMSE. The MRI, PET, and cognitive assessments were conducted during visits within a 2‐week timeframe.^22^ All imaging sessions were performed under resting‐state conditions. The participants were categorized into three groups based on their cognitive performance: 41 with cognitively normal controls (CN, MMSE > 28), 25 with MCI (23 ≤ MMSE ≤ 28), and 31 with dementia (Dem, MMSE < 23). Participants were further categorized based on the negativity (N) or positivity (P) of their Aβ imaging results: 32 with CN_N (CN with Aβ negative), nine with CN_P (CN with Aβ positive), 12 with MCI_N (MCI with Aβ negative), 13 with MCI_P (MCI with Aβ positive), six with Dem_N (Dem with Aβ negative), and 25 with Dem_P (Dem with Aβ positive). Specifically, participants classified as MCI_P and Dem_P are considered to exhibit pathology associated with AD, followed by the research criteria proposed by the National Institute on Aging (NIA)–Alzheimer's Association (AA) (2011) workgroups.^23^

In addition to MRI and PET scans, plasma protein levels were also extracted from 70 out of the 97 participants. Peripheral whole blood was collected from each participant, and plasma was separated and stored at −80°C prior to analysis. The single molecule array (Simoa) technique was employed to measure the levels of Aβ40, Aβ42, glial fibrillary acidic protein (GFAP), neurofilament light (NFL), and phosphorylated‐tau‐181 (pTau‐181) in the plasma. Plasma pTau‐181 was specifically measured using the Simoa pTau‐181 kit, while the Simoa N4PE Advantage kit was used to assess plasma levels of Aβ42, Aβ40, GFAP, and NFL. All procedures were standardized according to the manufacturer's instructions provided with each kit.

**RESEARCH IN CONTEXT**

1. **Systematic review**: The authors reviewed literature from sources like PubMed and recent abstracts. Brain conductivity has emerged as a potential MRI‐based biomarker for dementia, though its ability to detect protein aggregation, specifically Aβ and tau, has not been explored. The review includes references supporting the hypothesis that brain tissue conductivity is associated with cognitive decline and protein aggregation.
2. **Interpretation**: The findings show a significant link between increased brain tissue conductivity and protein aggregation (Aβ and tau) in dementia, correlating with cognitive decline and gene expression changes. This indicates that brain tissue conductivity could be a non‐invasive biomarker for detecting and monitoring protein aggregation in AD, providing insights into the mechanisms of neurodegeneration.
3. **Future directions**: Further research should investigate the causal relationship between protein aggregation‐induced ion changes and tissue conductivity, develop advanced MRI techniques, and integrate conductivity with other biomarkers for early Alzheimer's detection.

### 2.2 Acquisition of MRI and PET images

MRI data were acquired using a 3T scanner (uMR 890, United Imaging Healthcare) equipped with a dedicated 64‐channel head coil. We collected three‐dimensional (3D) T1w and multi‐echo GRE images. The T1w structural images were acquired with a 3D fast spoiled gradient‐echo sequence, with 0.5 mm isotropic voxels (field of view: 220 × 240 mm^2^, 360 sagittal slices, repetition time [TR]/inversion time [TI] = 7.5 ms/1100 ms, echo time [TE] = 3.4 ms, flip angle = 7°). The multi‐echo GRE acquisition parameters were as follows: field of view: 230 × 230 mm^2^, matrix = 224 × 224, slice thickness = 3 mm, 46 axial slices, TR = 35 ms, TE1/ΔTE/TE6 = 2.5/4.3/24.0 ms, flip angle = 15°.

PET scans were performed on a 3T whole‐body PET/MRI scanner (uPMR 790, United Imaging Healthcare). Participants received an intravenous injection of ^18^F florbetapir (AV45) at an average dose of 3.7 MBq/kg body weight to visualize Aβ. Static AV45‐PET data were acquired in sinogram mode 50 min after injection. Additionally, the participants underwent static ^18^F MK‐6240 PET scans to visualize tau. The PET scans were scheduled at least 1 week apart from each other. PET standardized uptake value ratios (SUVRs) were globally normalized to the mean uptake in a cerebellar gray matter (reference) region.

### 2.3 Reconstruction of tissue conductivity image

The phase‐based conductivity maps were reconstructed from B1 phase maps derived from multi‐echo GRE (mGRE) data.^24^, ^25^, ^26^ To estimate the B1 phase images (phase at TE = 0 ms), spatial phase unwrapping was applied to the mGRE phase images at each TE.^27^ The unwrapped phase images were then aligned, and a linear fit was performed to model the phase evolution across different TEs. This linear fitting process yielded B1 phase images, which served as the basis for subsequent conductivity mapping.

The conductivity maps were generated by solving a convection‐reaction partial differential equation (PDE) using a 2D finite‐difference method based on B1 phase images.^28^, ^29^ This computational process was implemented in custom software developed in the C programming language (https://eptlib.github.io/methods/ept‐convreact).^30^ To enhance the stability and accuracy of the PDE solution, a Savitzky‐Golay (SG) filter^31^ with a kernel size of 2 × 2 × 2 was applied. Additionally, the first three echo phase images were used to estimate the B1 phase maps. To further reduce noise and improve robustness, a median filter with a kernel size of 5 × 5 × 5 was applied to the B1 phase images. The reconstructed conductivity maps were subsequently utilized for voxel‐based and region‐of‐interest (ROI) analyses.

### 2.4 Voxel‐based analysis

Individual T1w images were registered to their corresponding GRE magnitude, Aβ‐PET, and tau‐PET images using an affine method with Advanced Normalization Tools (ANTs).^32^ Individual T1w images were also normalized to the Montreal Neurological Institute (MNI) space atlas using the SyN in ANTs. The tissue conductivity, Aβ‐PET, and tau‐PET images were subsequently normalized to MNI space using the inverse affine transformation matrix and the SyN deformation field. All conductivity maps and PET images were smoothed using a 6‐mm full width at half maximum (FWHM) Gaussian kernel. Then voxel‐wise group analysis was done between CN and dementia groups using two‐sample *t*‐tests for images of each modality, with age as a covariate. Furthermore, voxel‐wise Pearson's correlation analysis was performed to identify the brain areas showing a significant correlation between tissue conductivity and PET intensities. For Aβ‐PET, we focused on the ROIs most typically with Aβ aggregation in AD, that is, middle frontal cortex (MFC), posterior cingulate cortex (PCC), precentral cortex, and postcentral cortex.^33^, ^34^ For tau‐PET, we extracted the regions of orbital frontal cortex (OFC), superior temporal cortex (STC), middle temporal cortex (MTC), inferior temporal cortex (ITC), PCC and precuneus cortex.^34^ The mean SUVR values of PET images and the ages of the participants were used to generate the design matrix. FSL randomization was used for all the voxel‐wise analyses using the default 5000 permutations. *P *< .05 with threshold‐free cluster enhancement (TFCE) correction was considered statistically significant. BrainNet Viewer was used to visualize data on cortical surfaces.^35^

### 2.5 Region‐of‐interest analysis

ROI analysis were further performed to more intuitively present the tissue conductivity abnormalities in brain regions and the individual values in regions associated with protein aggregation. Specifically, ROIs were selected from the Desikan‐Killiany‐Tourville‐labeled Atlas,^36^, ^37^ including the MFC, OFC, precentral cortex, postcentral cortex, PCC, STC, MTC, ITC, precuneus cortex, insula cortex, and cerebrospinal fluid (CSF). The T1w atlas was registered to the individual T1w images and aligned with the tissue conductivity, Aβ‐PET, and tau‐PET maps using ANTs. The regional brain volumes of all ROIs were calculated by Freesurfer 7.4^38^ and were used as the covariates in all ROI analyses. Between‐group comparisons were made by two‐sample *t*‐tests. For all participants, correlation analyses were conducted to examine the relationships between mean conductivity signal intensity and PET signals (Aβ‐PET and tau‐PET) in gray matter regions, as well as MMSE scores and plasma protein levels, including Aβ42/Aβ40,^39^ pTau‐181,^40^ GFAP,^41^, ^42^ and NFL.^43^ To investigate potential diagnostic status associations with primary outcomes, correlation analyses across CN, MCI, and Dem were performed, despite the reduced statistical power resulting from smaller sample sizes. For brain regions demonstrating robust associations (OFC, precentral cortex, PCC, and precuneus cortex), secondary analyses were conducted across six refined subgroups (CN_N, CN_P, MCI_N, MCI_P, Dem_N, and Dem_P) stratified by amyloid status (N/P).

### 2.6 Diagnostic performance evaluation

Logistic regression and Receiver Operating Characteristic (ROC) curves were used to assess the ability to distinguish CN, MCI, and dementia using tissue conductivity, gray matter volume (GMV), and a combination of the two signals. ROC analysis was performed for four ROIs: PCC, OFC, precuneus cortex, and precentral cortex. The area under the ROC curve (AUC) was calculated as an evaluation index. The logistic regression and the ROC analyses were performed using Python.

### 2.7 Relating gene expression and brain conductivity and PET images

Gene expression data were obtained from the Allen Human Brain Atlas microarray transcriptomic data and projected onto the Desikan‐Killiany‐Tourville‐labeled cortical Atlas using the “abagen” toolkit.^17^, ^44^ A total of 148 regions defined by the atlas labels were included in the analysis, excluding the bilateral ctx_Unknown and ctx_Medial_wall regions. Genes with expression levels above a 50% background threshold were selected. Regional expression levels for each gene were compiled into a 148 × 15,633 regional transcription matrix. The association between the difference maps of tissue conductivity, Aβ‐PET, and tau‐PET in dementia versus CN and gene expression levels was investigated separately using PLS analysis.^18^

PLS regression is a multivariate statistical method that combines dimension reduction and linear regression, similar to principal component analysis. It extracts components from the predictor matrix X (the 148 × 15,633 matrix of regional mRNA measurements for 15,633 genes) that have maximum covariance with Y (three 148 × 1 vectors denoting regional conductivity or protein‐PET signal differences). The second PLS component (PLS2) was used to weigh and rank the gene predictor variables. To test the null hypothesis that PLS2 explained no more variance in Y than would be expected by chance due to autocorrelation of the neuroimaging data, 10,000 permutations based on sphere‐projection rotations of the cortical map were performed.^45^ Bootstrapping was then implemented to estimate the variability of each gene's weight on PLS2, with the ratio of a gene's weight to its bootstrapped standard error used to assess its impact on PLS2. All PLS and bootstrapping analyses were performed using MATLAB (R2019b, https://www.mathworks.com/products/matlab.html).

To mitigate the winner's curse bias, each gene's weight was tested against the hypothesis of zero weight. A false discovery rate inverse quantile transformation correction was applied using R version 4.3.1.^46^ Only genes that survived this correction at *Q *< 0.05 were included in the enrichment analyses.

### 2.8 Gene Ontology analysis

To conduct a GO enrichment analysis, the g:Profiler toolset was utilized on the significant positively and negatively weighted genes identified by PLS2.^19^ To ensure the robustness of the results, we applied a filter to the list of GO terms, retaining only those that demonstrated significant enrichment with a corrected *p* value of < .01 using the g:SCS algorithm. Additionally, we excluded terms associated with more than 2500 genes, as they were considered too broad.^18^ To reduce and visualize the GO terms, we employed the REViGO tool, which is based on semantic similarity.^47^

## 3 RESULTS

This study included a total of 97 participants, consisting of 31 individuals with dementia (mean age ± SD: 71.0 ± 9.3 years; 16 females), 25 with MCI (mean age ± SD: 71.0 ± 6.6 years; 11 females), and 41 CN individuals (mean age ± SD: 70.0 ± 7.1 years; 25 females). Detailed demographic and clinical characteristics are provided in Table 1.

**TABLE 1:** Demographic and clinical characteristics of study cohort.

		Group					
Parameter	Total	CN	MCI	Dem	*P* value (CN‐MCI)	*P* value (CN‐Dem)	*P* value (MCI‐Dem)
No. participants (F/M)	97 (52/45)	41 (25/16)	25 (11/14)	31 (16/15)	.21	.48	.60
No. Aβ PET (P/N)	97 (47/50)	41 (9/32)	25 (13/12)	31 (25/6)			
No. tau PET (P/N)	76 (48/38)	31 (10/21)	19 (8/11)	26 (20/6)			
Age (years)	70.30 ±7.72	70.00 ±7.10	71.00 ±6.60	71.00 ±9.30	.84	.72	.98
Education (years)	11.90 ±4.18	12.00 ±1.25	13.00 ±4.80	11.00 ±4.50	.45	.68	.15
MMSE score	24.18 ±6.02	28.68 ±1.13	25.88 ±1.94	16.83 ±5.04	.002	<.001	<.001

*Note*: The significance of between‐group difference in age was examined by two‐sample *t*‐test, and the significance of difference in sex was examined by chi‐squared test.

Abbreviations: CN, cognitively normal controls; Dem, dementia; F, female; M, male; MCI, mild cognitive impairment; MMSE, Mini‐Mental State Examination; N, negative on positron emission tomography (PET) signal assessed by a neurologist and a radiologist; P, positive on PET signal assessed by a neurologist and a radiologist.

### 3.1 Relationship between tissue conductivity and protein‐PET images

**FIGURE 1:**
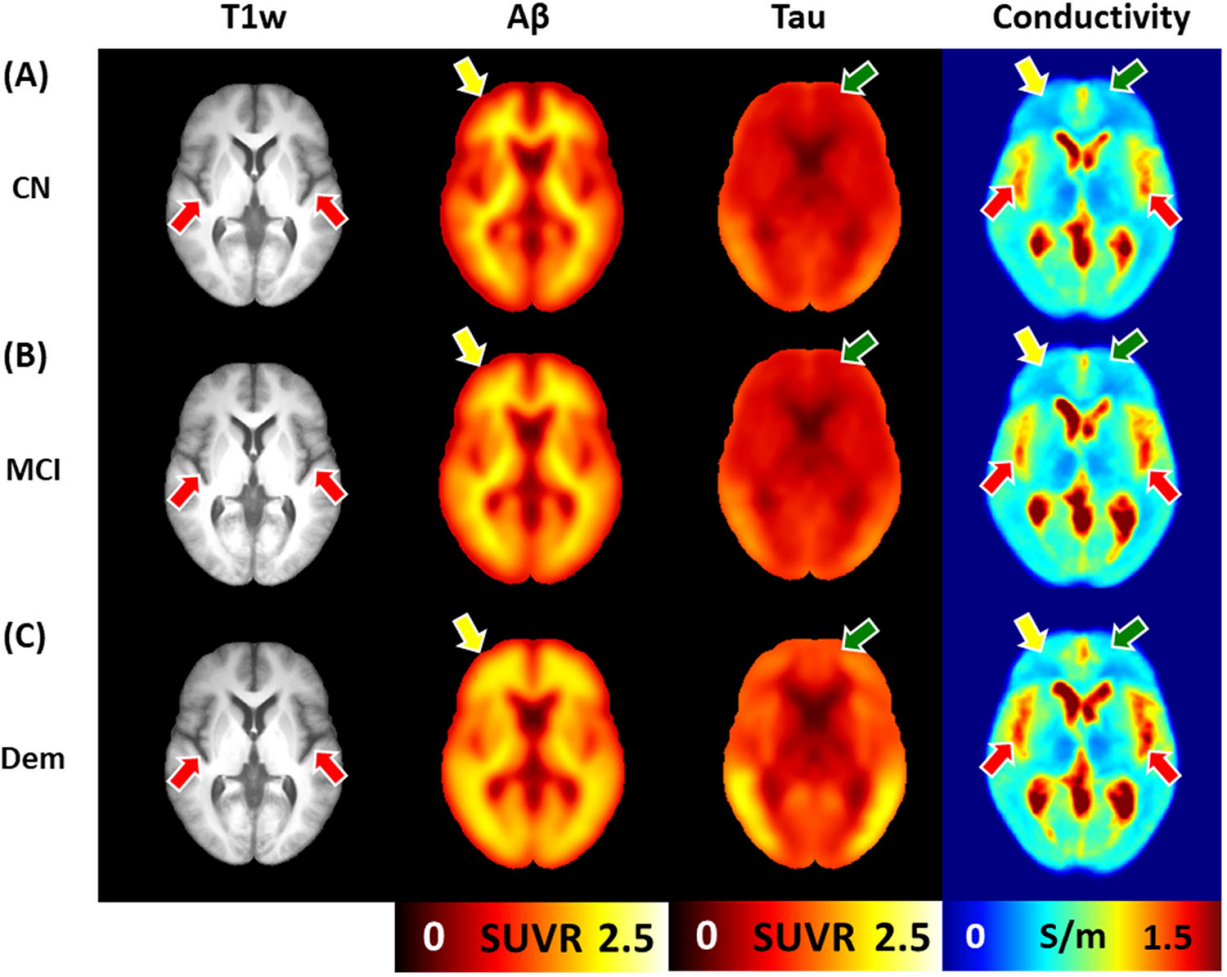
Representative slices from group average images of different modalities in MNI space for CN, MCI, and Dem groups. (A) CN group‐average image. (B) MCI group‐average image. (C) Dem group‐average image. Arrows highlight key regions of increased brain conductivity associated with brain atrophy and cerebrospinal fluid (CSF) expansion (red, bilateral), elevated Aβ signal (yellow), and higher tau signal (green). Aβ, amyloid beta; CN, cognitively normal controls; Dem, dementia; MCI, mild cognitive impairment; S/m, Siemens per meter; SUVR, standardized uptake value ratio.

**FIGURE 2:**
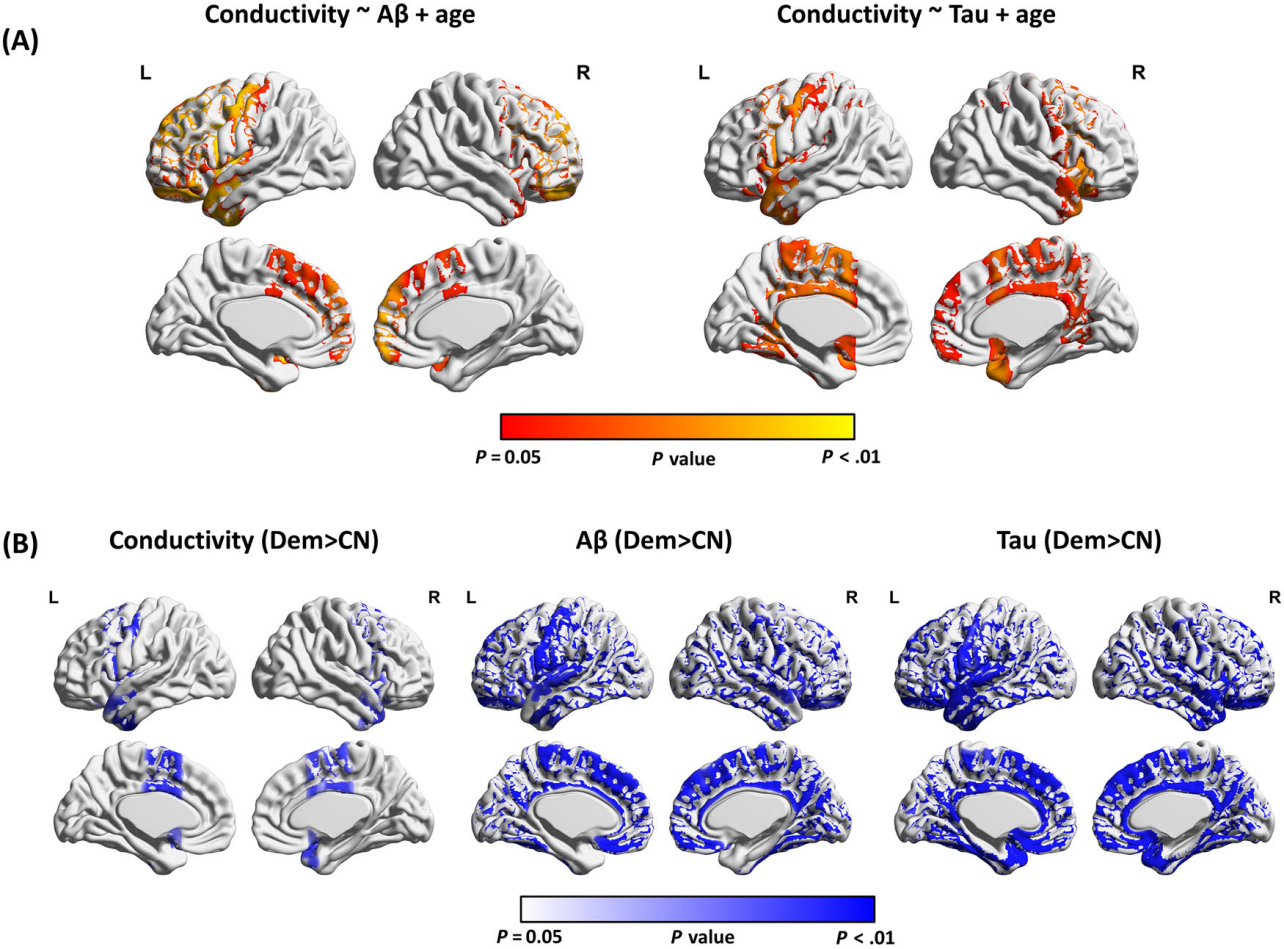
Voxel‐based analysis of relationship between brain tissue conductivity and protein aggregation. (A) Brain regions showing significant correlations between conductivity and Aβ‐PET (left) and tau‐PET (right) after adjusting for age (*p *< .05, TFCE corrected). (B) Brain regions with significant difference between dementia and CN in conductivity (left), Aβ‐PET (middle), and tau‐PET (right) after adjusting for age (*P *< .05, TFCE corrected). Aβ, amyloid beta; CN, cognitively normal controls; Dem, dementia; MCI, mild cognitive impairment; PET, positron emission tomography; TFCE, threshold‐free cluster enhancement.

Figure 1 displays the average images of different modalities, highlighting the Aβ‐PET, tau‐PET, and increase in conductivity signals observed in various brain regions. The between‐group comparison revealed significantly higher tissue conductivity in the regions of frontal, temporal, cingulate, and insula cortices in the participants with dementia compared to the CN participants (*p *< .05, TFCE corrected) (Figure 2). Voxel‐based analysis further indicated that tissue conductivity was significantly correlated with amyloid in the regions of frontal, temporal, precentral, postcentral, cingulate, and insula cortices (*p *< .05, TFCE corrected). Additionally, tissue conductivity was correlated with tau in the regions of frontal, temporal, precentral, postcentral, cingulate, precuneus, and insula cortices (*p *< .05, TFCE corrected).

**FIGURE 3:**
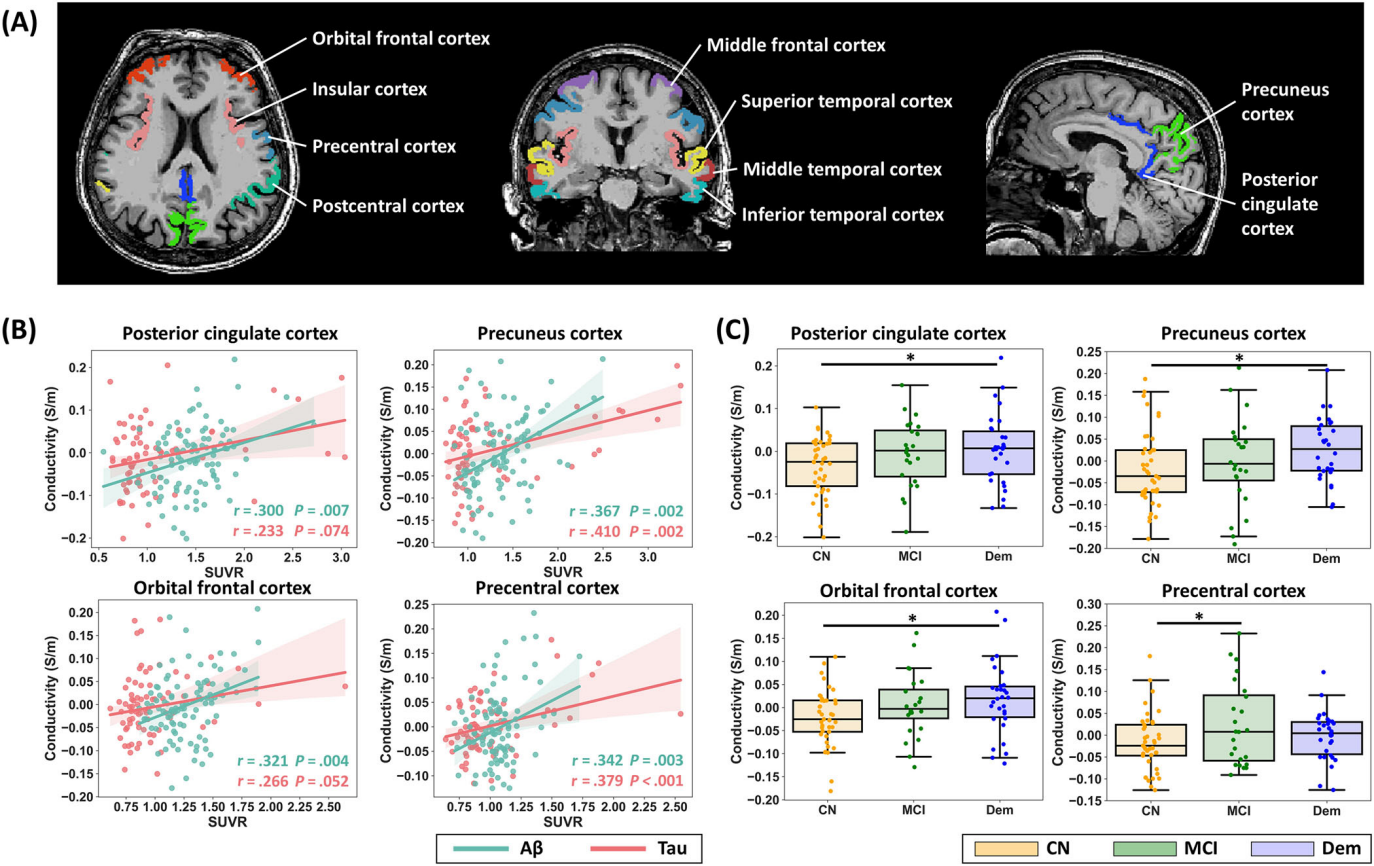
ROI analysis of the relationship between brain tissue conductivity and protein aggregation with regional brain volume as the covariate. (A) Regions included in the correlation analysis. (B) Regional correlations between conductivity and SUVR values. (C) Conductivity levels of different regions in the three participant groups. *p < .05. CN, cognitively normal controls; Dem, dementia; MCI, mild cognitive impairment; ROI, region of interest; SUVR, standardized uptake value ratio.

ROI analysis confirmed the correlation between tissue conductivity and protein aggregation in the brain. As shown in Figure 3B and Table S1, tissue conductivity was correlated with amyloid in the regions of the OFC, precuneus cortex, and PCC (corrected *p *< .05). Tissue conductivity was correlated with tau deposits in the MFC, precuneus cortex, and precentral cortex (corrected *p *< .05).

Based on the diagnosis status, our ROI analyses demonstrated distinct spatial patterns of tissue conductivity correlations with amyloid and tau pathology across different diagnosis statuses (Tables S2–S4). Specifically, amyloid‐conductivity associations were predominantly localized to the precuneus cortex, PCC, precentral cortex, and postcentral cortex in MCI patients, while concentrating in the MFC, PCC, and precentral cortex in Dem patients. In contrast, tau‐conductivity correlations exhibited stronger linkages in the precentral cortex of MCI patients, with predominant associations observed in the precuneus cortex and PCC of Dem patients. Further stratification based on amyloid P/N demonstrated that these significant correlations were primarily concentrated in MCI_P and Dem_P subgroups (Table S5).

Between‐group comparisons showed that tissue conductivity was higher in the regions of OFC, PCC, and precuneus cortex in the participants with dementia compared to CN (Figure 3C). In addition, Figure 3C demonstrates notable group differences between MCI and CN participants in the precentral cortex.

### 3.2 Abnormality in tissue conductivity associated with cognition

**FIGURE 4:**
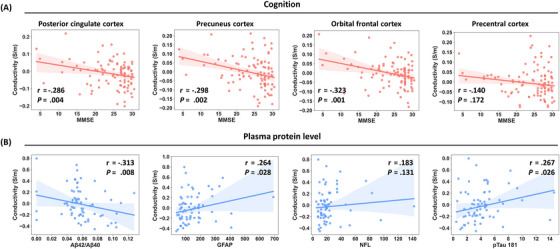
Relationship between electrical conductivity and cognition and plasma protein levels, with regional brain volume as covariate. (A) Relationship between brain tissue conductivity and MMSE scores. (B) Relationship between CSF conductivity and plasma protein levels. CSF, cerebral spinal fluid; GFAP, glial fibrillary acidic protein; MMSE, Mini‐Mental State Examination; NFL, neurofilament light; pTau‐181, phosphorylated‐tau‐181.

The study also explored the relationships between tissue conductivity and cognitive performance with regional brain volume as the covariate. As shown in Figure 4A and Table S6, tissue conductivity was negatively correlated with cognitive performance, as measured by MMSE, in various regions including MFC, OFC, precuneus cortex, and PCC (corrected *p *< .05). In addition, subgroup analysis revealed that the significant correlation between tissue conductivity and MMSE scores was predominantly observed in the MCI and Dem groups, particularly within the MCI_P subgroup (Tables S7–S10).

### 3.3 Relationship between CSF conductivity and blood proteins

The relationship between CSF conductivity and peripheral blood proteins was further examined. As shown in Figure 4B and Table S11, the CSF conductivity was negatively correlated with Aβ42/Aβ40 and positively correlated with pTau‐181 and GFAP extracted from peripheral blood (*p *< .05). The relationship between conductivity in gray matter regions and blood protein levels is also presented in Table S11. In gray matter, few significant correlations were found between conductivity and plasma protein levels. The subgroup analysis revealed that significant correlations were primarily observed in the Dem and MCI_P groups in the CSF region (Tables S12 and S13).

### 3.4 Diagnostic performance

**FIGURE 5:**
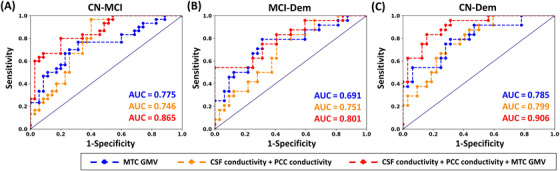
Area under Receiver Operating Characteristic curve for discrimination of dementia using electrical conductivity and gray matter volumes. (A) Discrimination between CN and MCI. (B) Discrimination between MCI and dementia. (C) Discrimination between CN and dementia. AUC, area under curve; CN, cognitively normal controls; CSF, cerebral spinal fluid; Dem, dementia; GMV, gray matter volume; MCI, mild cognitive impairment; MTC, middle temporal cortex; PCC, posterior cingulate cortex.

The AUCs were calculated to assess the discrimination power between CN, MCI, and dementia, using conductivity and GMV signals of the ROIs (Table S14). When using GMV in the MTC alone or conductivity measures in the CSF and PCC individually, the AUCs were less than 0.8 for classifying CN, MCI, and dementia. However, combining GMV in the MTC with conductivity measures in both CSF and PCC enhanced the AUC to 0.8 or even above 0.9 (Figure 5).

### 3.5 Relating tissue conductivity in dementia to variation in gene expression patterns

**FIGURE 6:**
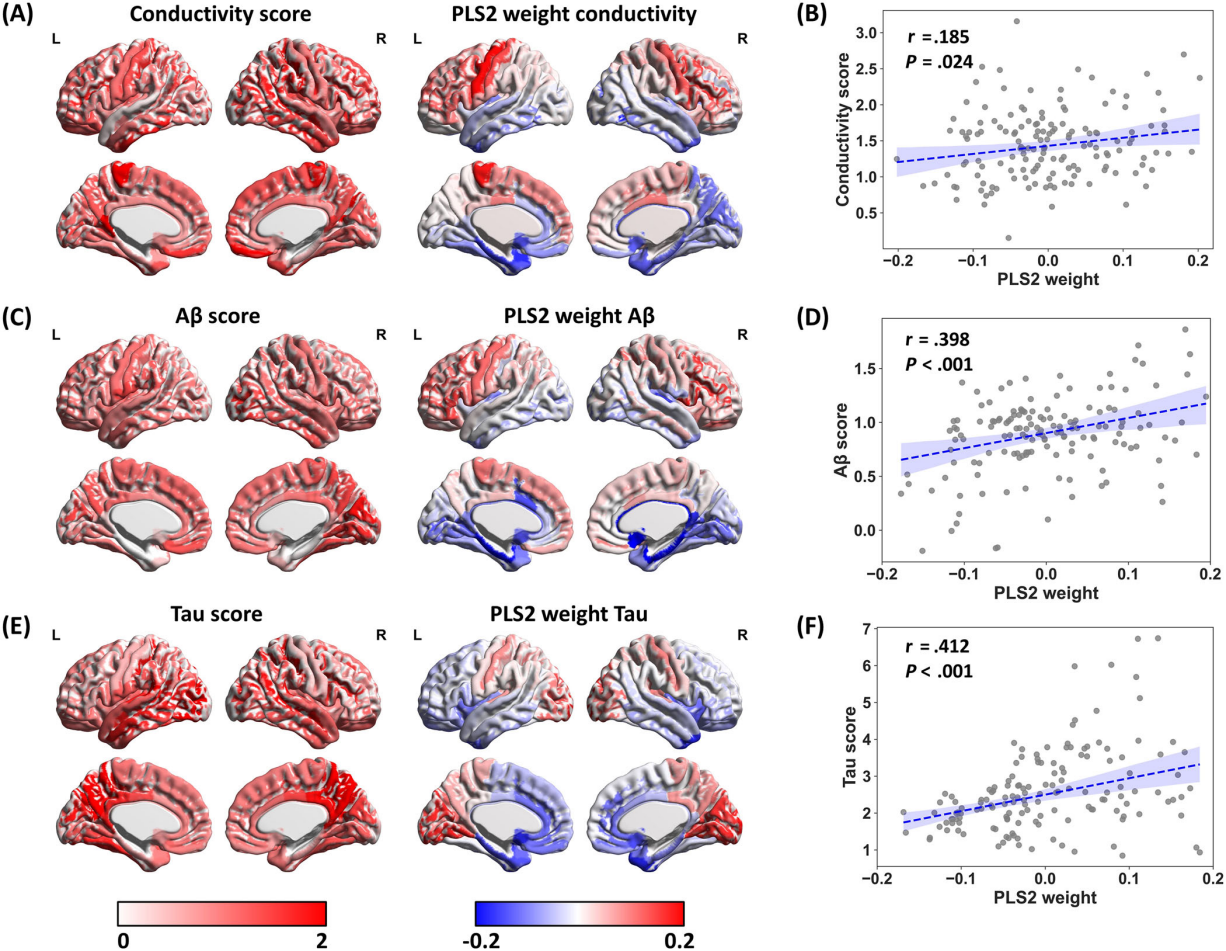
Cortical profiles of PLS regression results. (A) Conductivity, (C) Aβ, and (E) tau difference maps between dementia and CN show spatial patterns similar to the regionally weighted sum of gene expression scores defined by PLS2. Positive correlations between regional PLS2 scores and (B) conductivity, (D) Aβ, and (F) tau difference. In the scatterplot, each point represents one of 148 cortical regions. The results imply that genes with higher weights on PLS2 also exhibited greater expression levels in cortical regions with increased signal differences. Aβ, amyloid beta; CN, cognitively normal controls; PLS, partial least squares.

PLS regression was used to identify gene expression patterns that correlated with the anatomical distribution of brain tissue conductivity, Aβ, and tau. The results revealed that PLS2 accounted for most of the variance observed in the conductivity difference map for these variables. The gene expression weights associated with PLS2 exhibited a positive correlation with tissue conductivity (*r *= 0.185, *p *= .024, confidence interval [CI] = [−2.020, −0.141]), Aβ (*r *= 0.0.398, *p *< .001, CI = [0.923, 2.240]), and tau (*r *= 0.412, *p *< .001, CI = [3.068, 7.102]) (Figure 6), suggesting that genes assigned higher weights on PLS2 also exhibited higher expression levels in cortical regions with increased conductivity, Aβ, and tau. The spatial profile of PLS2 weightings matched that of the conductivity difference map (Figure 6), particularly in the frontal, parietal, and cingulate cortices. Consequently, PLS2 was used to rank and select significantly weighted genes, resulting in a set of significantly upweighted genes (*Q *< 0.001). The complete set of PLS2 gene weights and associated statistics are provided for conductivity (Table S15), Aβ (Table S16), and tau (Table S17).

**FIGURE 7:**
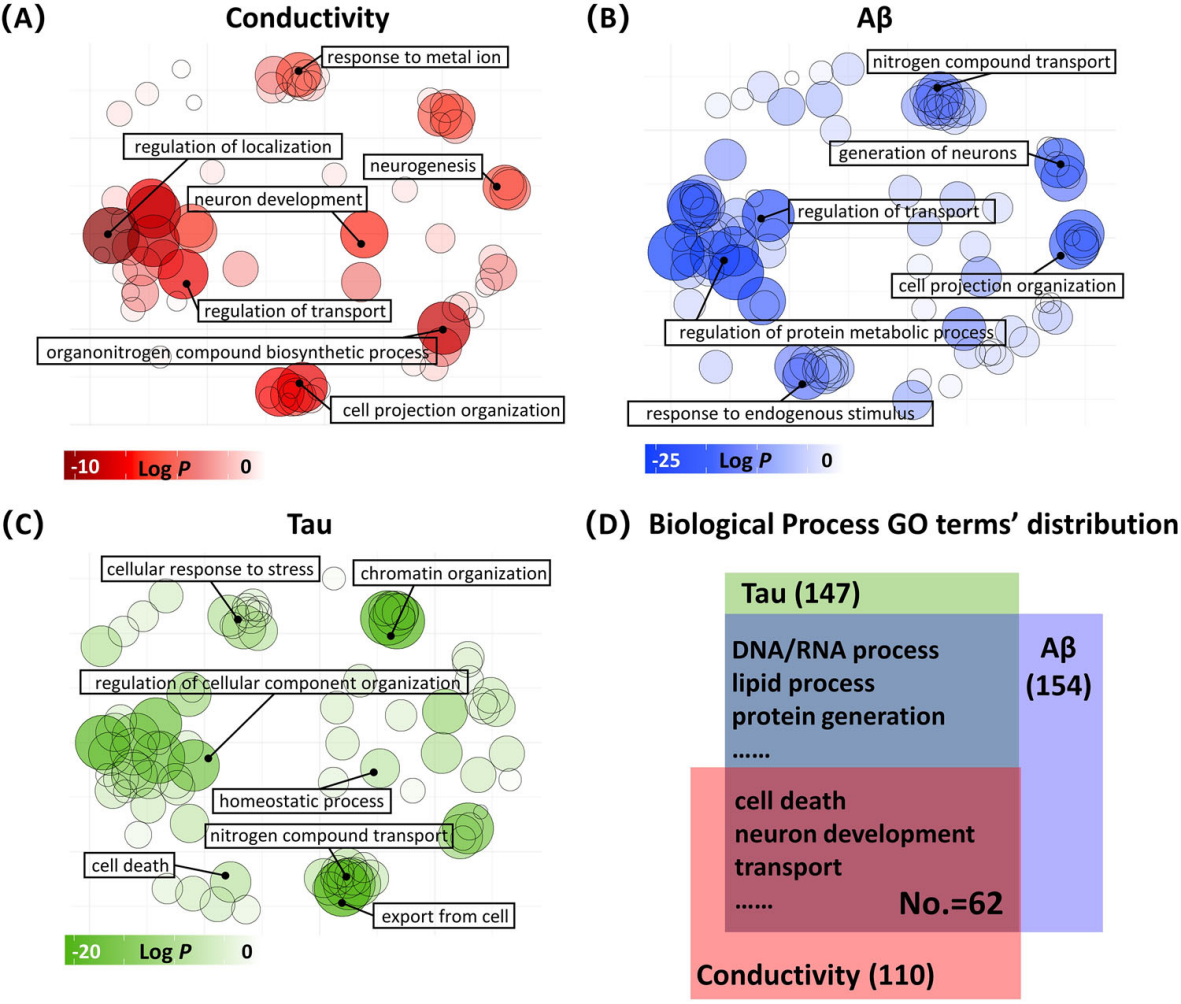
Enrichment analyses of genes associated with brain tissue conductivity in dementia. (A) Conductivity‐related GO terms. (B) Aβ aggregation‐related GO terms. (C) Tau aggregation‐related GO terms. These GO terms for biological processes were significantly enriched in genes with higher weights defined by PLS2. The terms are plotted in semantic space, with more similar terms clustered together. Non‐redundant GO terms significant at g:SCS‐corrected *p *< .001 are shown, with larger, darker circles indicating greater significance. (D) Overlap of GO terms for biological processes across the three categories, with specific terms highlighted. Aβ, amyloid beta; GO, Gene Ontology; PLS, partial least squares.

GO analyses revealed that upweighted genes were associated with specific biological processes. For conductivity, enriched GO terms included ion metabolism and transport (e.g., response to metal ion, regulation of transport) and neuronal development (e.g., neurogenesis) (Figure 7A). For Aβ, enriched terms related to transport processes (e.g., nitrogen compound transport) and neuronal organization (e.g., cell projection organization) (Figure 7B). For tau, enriched terms included cellular stress responses (e.g., cellular response to stress) and transport regulation (e.g., nitrogen compound transport) (Figure 7C). Overlaps between conductivity and protein‐related genes were primarily linked to cell death, neuron development, and transport (Figure 7D). Full GO results are provided in Tables S18–S20. Control analyses confirmed that these associations were not driven by spatial autocorrelation or gene‐gene co‐expression (Tables S21–S23).

## 4 DISCUSSION

This study unveiled a novel and significant association, for the first time, between brain electrical conductivity imaging and protein aggregation, revealing the potential of conductivity imaging to provide insights into brain protein aggregation. Our analysis demonstrated a correlation between conductivity and protein aggregation as well as plasma protein levels, suggesting that conductivity imaging can offer valuable information about protein aggregation in the brain. These associations exhibited disease‐stage specificity, with significant correlations predominantly observed in MCI and Dem groups but absent in cognitively normal individuals, suggesting dynamic interactions between conductivity alterations and neurodegenerative progression. Additionally, brain electrical conductivity, combined with GMV, enhances the diagnostic accuracy for dementia. GO analysis further suggests that abnormalities in tissue conductivity may be linked to microscopic processes such as iron metabolism, cellular development and organization, and cell communication and signaling.

Tissue conductivity, as an electrical property of tissue, is influenced by changes in tissue composition, ion concentration, and other factors.^48^, ^49^ Tissue conductivity can be obtained through post‐processing of magnetic resonance images.^28^, ^50^ Previous studies showed that electrical conductivity increased in malignant tumors such as breast cancer and brain glioma.^51^, ^52^, ^53^ Animal studies have shown that injecting protein aggregates into the brain can lead to disruption of Na^+^/K^+^ ATPase activity, suggesting that the increase in brain tissue conductivity is likely closely associated with protein aggregation.^14^ Recently, Park et al. found that brain tissue conductivity was elevated in AD patients, likely due to increased concentrations of intracellular and extracellular ions from protein aggregation.^15^ In this study, we also demonstrated that tissue conductivity in multiple brain regions was negatively correlated with cognitive function as measured by MMSE scores. These findings further indicate a link between tissue conductivity and cognition decline caused by dementia.

Our results indicate that abnormal tissue conductivity in dementia patients exhibits a spatial similarity to protein aggregation, with higher conductivity correlating with increased Aβ and tau signals in brain areas. Notably, we observed a striking convergence of amyloid deposition, tau accumulation, and abnormal tissue conductivity in many key regions, such as the precuneus cortex, PCC, OFC, and precentral cortex. These robust associations were particularly pronounced in both MCI and Dem groups, with the strongest effects observed in MCI_P and Dem_P subgroups characterized by AD‐related pathology. This aligns with previous findings of elevated PET protein signals in dementia.^54^ The cortical areas that exhibit significant correlations serve as pivotal substrates essential for higher‐level cognitive functions, encompassing executive capabilities, language processing, memory retention, and attentional mechanisms.^55^ The observed increase in brain tissue conductivity observed in dementia patients may be attributed to elevated levels of metallic ions (e.g., Na^+^, K^+^, Ca^2+^),^5^, ^16^ which result from the misfolding and aggregation of Aβ proteins, disrupting homeostasis and causing an imbalance in sodium and potassium ions.^56^, ^57^ We also observed correlations between CSF conductivity and peripheral plasma biomarkers for dementia, including Aβ42/Aβ40,^39^ pTau‐181,^40^ GFAP,^41^, ^42^ and NFL.^43^ These correlations suggest a relationship between plasma biomarkers and elevated ions in CSF in dementia patients.^58^ These findings suggest that brain electrical conductivity measured by MRI is closely related to protein abnormalities in dementia and has potential as a clinical alternative to protein aggregation PET for assessment.

The ROC curve results indicate that using GMV in the MTC or conductivity measures in the CSF and PCC individually provides limited discrimination between CN, MCI, and dementia, with AUC values below 0.8. However, combining these measures significantly enhances diagnostic accuracy, with AUC values rising higher. This suggests that a multimodal approach, integrating both GMV and tissue conductivity, offers a more robust biomarker for distinguishing between stages of cognitive impairment.^15^ The improved AUC underscores the complementary nature of structural and conductivity measures, providing a more comprehensive understanding of the underlying pathophysiology of dementia.

GO analysis suggests that brain tissue conductivity abnormalities are related to microscopic processes such as ion and molecular processes, cellular development and organization, and cell communication and signaling. Abnormal ion concentrations reflected by changes in tissue conductivity are involved in the pathological processes of dementia development. Previous studies showed that disrupted ion concentrations could lead to ion imbalance in neuron and glial cells, affecting transport, ion conductance, and intercellular signaling, associated with neuronal dysfunction and cell death initiation.^5^, ^59^, ^60^ Additionally, genes associated with conductivity, Aβ, and tau in dementia are involved in transport processes and cellular organization and development. Our findings indicate that protein aggregation is linked to abnormal gene expression involved in mitochondrial respiration, protein synthesis, dendrite, axon, and neuron organization, neuronal structure and synaptic function, and lipid metabolism in dementia.^61^, ^62^, ^63^, ^64^ This finding indicates a potential association between ionic dysregulation (reflected by conductivity changes) and protein aggregation in dementia. It implies that disruptions in ion transport, cellular development, and organization could play a critical role in the accumulation of Aβ and tau, leading to neurodegeneration. Further mechanistic studies are needed to confirm the underlying mechanisms responsible for these associations.

## 5 LIMITATIONS

This study has several limitations. First, although we have identified a correlation between brain tissue conductivity, protein aggregation, and plasma protein levels, the hypothesis that increased tissue conductivity in dementia results from ion concentration changes due to protein aggregation remains speculative. Future studies are needed to clarify the direct relationship between tissue conductivity and protein‐induced ion concentration changes. Second, using the global MMSE score as a cognitive assessment tool has limitations, as it lacks sensitivity to specific cognitive domains such as executive function, memory, and language. Future work could correlate conductivity measures with more detailed cognitive tests targeting these domains to better understand the relationship between tissue conductivity and specific cognitive impairments. Third, we used gene expression data from the Allen Human Brain Atlas, which includes individuals without neurological or psychiatric disorders. Additionally, the GRE sequence used in this study had a voxel size of 1 × 1 × 3 mm^3^, which might have introduced partial volume effects in the axial direction. Future studies should employ higher‐resolution imaging protocols to minimize such effects and improve the precision of conductivity images. Consequently, the spatial expression profiles reflect normal variability rather than changes associated with dementia. Despite these limitations, the study provides valuable preliminary data establishing the relationship between brain tissue conductivity and protein aggregation, contributing to the advancement of MRI‐based tools in dementia research and clinical practice.

## 6 CONCLUSIONS

This study revealed a strong relationship between brain tissue conductivity, brain protein aggregation, and peripheral protein levels in plasma. Incorporating brain electrical conductivity improves the accuracy of dementia diagnosis, demonstrating its potential as a clinical indicator of brain protein aggregation. Additionally, the findings suggest that abnormal tissue conductivity in dementia is linked to microscopic processes, particularly those involving ion and molecular activities, cellular development and organization, and cell communication and signaling. This deepens our understanding of the pathological mechanisms underlying these conductivity changes.

## AUTHOR CONTRIBUTIONS

Jiayue Chu: Conceptualization, software, formal analysis, investigation, writing – original draft, writing – review & editing, and visualization. Junye Yao: Software and formal analysis. Zhenghao Li: Software, formal analysis, and visualization. Jun Li: Conceptualization, writing – original draft, and funding acquisition. Yuyao Zhang: Resources and supervision. Chunlei Liu: Conceptualization, writing – review & editing, and supervision. Hongjian He: Writing – review & editing, supervision, and funding acquisition. Binyin Li: Resources, funding acquisition, and supervision. Hongjiang Wei: Conceptualization, writing – review & editing, supervision, project administration, and funding acquisition. All authors read and provided comments on an earlier draft and approved the final manuscript.

## CONFLICT OF INTEREST STATEMENT

The authors declare no conflicts of interest. Author disclosures are available in the Supporting information.

## Supporting information



Supporting Information

Supporting Information

Supporting Information

Supporting Information

Supporting Information

Supporting Information

Supporting Information

Supporting Information

Supporting Information

Supporting Information

Supporting Information

Supporting Information
