# Supplementary material for: Brain tissue electrical conductivity as a promising biomarker for dementia assessment using MRI
Source: Alzheimers Dement. 2025 Jun 23;21(6):e70270. doi: 10.1002/alz.70270 (PMC12185248; doi:10.1002/alz.70270)
Supplement: Supplementary file 1 — Supporting Information [file ALZ-21-e70270-s008.docx]

**Supplementary Tables S1-S14**

**Supplementary Table S1. Regional correlation between brain tissue conductivity and Aβ-PET SUVR or tau-PET SUVR with regional brain volume as the covariate.**

|  | **Aβ-PET SUVR** | | | | **Tau-PET SUVR** | | | | | |
| --- | --- | --- | --- | --- | --- | --- | --- | --- | --- | --- |
|  | Pearson’s *r* | *P*-value | corrected *P* | CI | | Pearson’s *r* | *P*-value | corrected *P* | CI |  |
| **Middle Frontal Cortex** | 0.144 | 0.164 | 0.328 | [-0.067,0.024] | | 0.297 | 0.010 | 0.032 | [-0.035,0.053] |  |
| **Orbital Frontal Cortex** | 0.321 | 0.001 | 0.004 | [0.039,0.115] | | 0.266 | 0.021 | 0.052 | [-0.007,0.091] |  |
| **Insula Cortex** | -0.058 | 0.574 | 0.957 | [-0.018,0.061] | | 0.240 | 0.038 | 0.076 | [0.015,0.067] |  |
| **Precuneus Cortex** | 0.367 | <0.001 | 0.002 | [0.043,0.095] | | 0.410 | <0.001 | 0.002 | [0.024,0.077] |  |
| **Posterior Cingulate Cortex** | 0.300 | 0.003 | 0.007 | [0.032,0.082] | | 0.233 | 0.045 | 0.074 | [0.011,0.075] |  |
| **Precentral Cortex** | 0.342 | <0.001 | 0.003 | [0.035,0.103] | | 0.379 | <0.001 | <0.001 | [0.029,0.078] |  |
| **Postcentral Cortex** | 0.065 | 0.533 | 0.761 | [-0.034,0.063] | | 0.092 | 0.433 | 0.619 | [-0.021,0.032] |  |
| **Superior Temporal Cortex** | -0.015 | 0.888 | 0.888 | [-0.015,0.086] | | 0.110 | 0.349 | 0.436 | [-0.011,0.056] |  |
| **Middle Temporal Cortex** | -0.036 | 0.733 | 0.915 | [-0.036,0.028] | | 0.019 | 0.874 | 0.970 | [-0.016,0.019] |  |
| **Inferior Temporal Cortex** | 0.034 | 0.746 | 0.828 | [-0.033,0.054] | | -0.085 | 0.467 | 0.467 | [-0.019,0.012] |  |

*Notes*. PET, positron emission computed tomography; SUVR, standardized uptake value ratio; CI, confidence interval.

**Supplementary Table S2. Regional correlation between brain tissue conductivity and Aβ-PET SUVR or tau-PET SUVR with regional brain volume as the covariate in cognitively normal controls.**

|  | **Aβ-PET SUVR** | | | | **Tau-PET SUVR** | | | | | |
| --- | --- | --- | --- | --- | --- | --- | --- | --- | --- | --- |
|  | Pearson’s *r* | *P*-value | corrected *P* | CI | | Pearson’s *r* | *P*-value | corrected *P* | CI |  |
| **Middle Frontal Cortex** | 0.173 | 0.278 | 1.000 | [-0.094,0.237] | | 0.023 | 0.900 | 0.900 | [-0.098,0.011] |  |
| **Orbital Frontal Cortex** | 0.091 | 0.572 | 0.954 | [-0.013,0.182] | | -0.262 | 0.154 | 0.385 | [-0.014,0.086] |  |
| **Insula Cortex** | 0.124 | 0.438 | 1.000 | [-0.012,0.209] | | -0.263 | 0.153 | 0.510 | [-0.380,0.008] |  |
| **Precuneus Cortex** | 0.112 | 0.486 | 0.971 | [-0.071,0.112] | | 0.0484 | 0.796 | 0.884 | [-0.016,0.084] |  |
| **Posterior Cingulate Cortex** | 0.049 | 0.759 | 0.843 | [-0.066,0.090] | | 0.0778 | 0.678 | 0.846 | [-0.012,0.103] |  |
| **Precentral Cortex** | 0.067 | 0.678 | 0.847 | [-0.072,0.271] | | 0.168 | 0.365 | 0.607 | [-0.080,0.093] |  |
| **Postcentral Cortex** | -0.131 | 0.413 | 1.000 | [-0.107,0.049] | | 0.297 | 0.103 | 0.517 | [-0.014,0.119] |  |
| **Superior Temporal Cortex** | -0.173 | 0.2780 | 1.000 | [-0.341,-0.007] | | -0.213 | 0.249 | 0.498 | [-0.295,-0.001] |  |
| **Middle Temporal Cortex** | -0.011 | 0.943 | 0.943 | [-0.091,0.001] | | -0.334 | 0.066 | 0.660 | [-0.115,0.002] |  |
| **Inferior Temporal Cortex** | -0.083 | 0.606 | 0.866 | [-1.09,0.002] | | -0.127 | 0.497 | 0.710 | [-0.091,0.047] |  |

*Notes*. PET, positron emission computed tomography; SUVR, standardized uptake value ratio; CI, confidence interval.

**Supplementary Table S3. Regional correlation between brain tissue conductivity and Aβ-PET SUVR or tau-PET SUVR with regional brain volume as the covariate in mild cognitive impairment.**

|  | **Aβ-PET SUVR** | | | | **Tau-PET SUVR** | | | | | |
| --- | --- | --- | --- | --- | --- | --- | --- | --- | --- | --- |
|  | Pearson’s *r* | *P*-value | corrected *P* | CI | | Pearson’s *r* | *P*-value | corrected *P* | CI |  |
| **Middle Frontal Cortex** | -0.237 | 0.265 | 0.443 | [-0.362,-0.001] | | 0.356 | 0.029 | 0.097 | [-0.081,0.005] |  |
| **Orbital Frontal Cortex** | 0.274 | 0.184 | 0.368 | [-0.038,0.104] | | 0.195 | 0.424 | 0.707 | [-0.158,0.027] |  |
| **Insula Cortex** | -0.007 | 0.616 | 0.770 | [-0.041,0.113] | | -0.105 | 0.668 | 0.835 | [-0.279,0.002] |  |
| **Precuneus Cortex** | 0.571 | 0.001 | 0.018 | [0.058,0.228] | | 0.505 | 0.017 | 0.086 | [0.009,0.073] |  |
| **Posterior Cingulate Cortex** | 0.352 | 0.014 | 0.035 | [-0.010,0.133] | | -0.087 | 0.721 | 0.801 | [-0.064,0.063] |  |
| **Precentral Cortex** | 0.444 | 0.003 | 0.017 | [0.034,0.186] | | 0.690 | 0.001 | 0.011 | [0.093,0.210] |  |
| **Postcentral Cortex** | 0.383 | 0.004 | 0.014 | [-0.054,0.144] | | -0.277 | 0.250 | 0.501 | [-0.150,-0.002] |  |
| **Superior Temporal Cortex** | -0.099 | 0.644 | 0.716 | [-0.239,0.005] | | 0.0543 | 0.824 | 0.824 | [-0.010,0.160] |  |
| **Middle Temporal Cortex** | -0.173 | 0.416 | 0.594 | [-0.048,0.046] | | -0.4695 | 0.042 | 0.106 | [-0.070,-0.001] |  |
| **Inferior Temporal Cortex** | -0.001 | 0.998 | 0.998 | [-0.042,0.123] | | 0.110 | 0.652 | 0.931 | [-0.027,0.0318] |  |

*Notes*. PET, positron emission computed tomography; SUVR, standardized uptake value ratio; CI, confidence interval.

**Supplementary Table S4. Regional correlation between brain tissue conductivity and Aβ-PET SUVR or tau-PET SUVR with regional brain volume as the covariate in dementia.**

|  | **Aβ-PET SUVR** | | | | **Tau-PET SUVR** | | | | | |
| --- | --- | --- | --- | --- | --- | --- | --- | --- | --- | --- |
|  | Pearson’s *r* | *P*-value | corrected *P* | CI | | Pearson’s *r* | *P*-value | corrected *P* | CI |  |
| **Middle Frontal Cortex** | 0.360 | 0.013 | 0.045 | [-0.108,0.239] | | 0.330 | 0.107 | 0.178 | [-0.054,0.076] |  |
| **Orbital Frontal Cortex** | 0.256 | 0.163 | 0.326 | [-0.046,0.208] | | 0.444 | 0.025 | 0.065 | [-0.014,0.116] |  |
| **Insula Cortex** | 0.242 | 0.196 | 0.327 | [-0.207,0.328] | | 0.321 | 0.043 | 0.086 | [-0.289,0.007] |  |
| **Precuneus Cortex** | 0.295 | 0.106 | 0.266 | [-0.033,0.150] | | 0.522 | 0.007 | 0.036 | [0.012,0.073] |  |
| **Posterior Cingulate Cortex** | 0.482 | 0.003 | 0.040 | [0.049,0.167] | | 0.524 | 0.001 | 0.011 | [0.018,0.102] |  |
| **Precentral Cortex** | 0.451 | 0.007 | 0.039 | [0.032,0.224] | | 0.471 | 0.017 | 0.057 | [0.012,0.110] |  |
| **Postcentral Cortex** | 0.112 | 0.552 | 0.691 | [-0.070,0.716] | | 0.027 | 0.895 | 0.895 | [-0.031,0.030] |  |
| **Superior Temporal Cortex** | 0.204 | 0.279 | 0.399 | [-0.129,0.031] | | 0.199 | 0.339 | 0.423 | [-0.025,0.001] |  |
| **Middle Temporal Cortex** | -0.034 | 0.858 | 0.953 | [-0.080,0.043] | | 0.103 | 0.623 | 0.693 | [-0.021,0.035] |  |
| **Inferior Temporal Cortex** | 0.030 | 0.872 | 0.872 | [-0.073,0.064] | | -0.219 | 0.290 | 0.415 | [-0.038,0.014] |  |

*Notes*. PET, positron emission computed tomography; SUVR, standardized uptake value ratio; CI, confidence interval.

**Supplementary Table S5. Regional correlation between brain tissue conductivity and Aβ-PET SUVR with regional brain volume as the covariate based on six subgroups.**

|  |  | Pearson’s *r* | *P*-value | corrected *P* | CI |
| --- | --- | --- | --- | --- | --- |
| **CN_N** | **Orbital Frontal Cortex** | -0.132 | 0.473 | 0.945 | [-0.176,0.126] |
|  | **Precuneus Cortex** | 0.075 | 0.684 | 0.684 | [-0.160,0.197] |
|  | **Posterior Cingulate Cortex** | 0.118 | 0.519 | 0.691 | [-0.089,0.124] |
|  | **Precentral Cortex** | 0.175 | 0.339 | 1.000 | [-0.096,0.271] |
| **CN_P** | **Orbital Frontal Cortex** | 0.756 | 0.018 | 0.073 | [0.070,0.553] |
|  | **Precuneus Cortex** | 0.536 | 0.137 | 0.274 | [-0.058,0.347] |
|  | **Posterior Cingulate Cortex** | 0.188 | 0.628 | 0.628 | [-0.144,0.223] |
|  | **Precentral Cortex** | 0.399 | 0.288 | 0.383 | [-0.485,1.405] |
| **MCI_N** | **Orbital Frontal Cortex** | -0.139 | 0.667 | 0.889 | [-0.231,0.154] |
|  | **Precuneus Cortex** | 0.273 | 0.391 | 1.000 | [-0.238,0.346] |
|  | **Posterior Cingulate Cortex** | 0.112 | 0.729 | 0.729 | [-0.322,0.237] |
|  | **Precentral Cortex** | 0.152 | 0.637 | 1.000 | [-0.362,0.564] |
| **MCI_P** | **Orbital Frontal Cortex** | 0.302 | 0.316 | 0.315 | [-0.107,0.263] |
|  | **Precuneus Cortex** | 0.705 | 0.007 | 0.014 | [0.064,0.324] |
|  | **Posterior Cingulate Cortex** | 0.709 | 0.007 | 0.026 | [0.025,0.183] |
|  | **Precentral Cortex** | 0.590 | 0.034 | 0.045 | [0.029,0.613] |
| **Dem_N** | **Orbital Frontal Cortex** | -0.698 | 0.123 | 0.490 | [-0.741,0.129] |
|  | **Precuneus Cortex** | 0.371 | 0.468 | 0.624 | [-0.453,0.434] |
|  | **Posterior Cingulate Cortex** | 0.263 | 0.614 | 0.614 | [-0.426,0.634] |
|  | **Precentral Cortex** | -0.486 | 0.329 | 0.657 | [-0.774,0.346] |
| **Dem_P** | **Orbital Frontal Cortex** | 0.234 | 0.261 | 0.260 | [-0.080,0.253] |
|  | **Precuneus Cortex** | 0.402 | 0.046 | 0.061 | [-0.013,0.197] |
|  | **Posterior Cingulate Cortex** | 0.529 | 0.007 | 0.026 | [0.053,0.295] |
|  | **Precentral Cortex** | 0.483 | 0.014 | 0.028 | [0.029,0.239] |

*Notes*. CN_N, cognitively normal controls with Aβ negative; CN_P, cognitively normal controls with Aβ positive; MCI_N, mild cognitive impairment with Aβ negative; MCI_P, mild cognitive impairment with Aβ positive; Dem_N, dementia with Aβ negative; Dem_P, dementia with Aβ positive; CI, confidence interval.

**Supplementary Table S6. Regional correlation between brain tissue conductivity and mini‐mental state examination with regional brain volume as the covariate.**

|  | Pearson’s *r* | *P*-value | corrected *P* | CI |
| --- | --- | --- | --- | --- |
| **Middle Frontal Cortex** | -0.346 | 0.001 | 0.005 | [-0.015,-0.005] |
| **Orbital Frontal Cortex** | -0.323 | 0.001 | 0.005 | [-0.006,-0.002] |
| **Insula Cortex** | -0.185 | 0.072 | 0.113 | [-0.007,0.000] |
| **Precuneus Cortex** | -0.298 | 0.002 | 0.007 | [-0.007,-0.001] |
| **Posterior Cingulate Cortex** | -0.286 | 0.004 | 0.011 | [-0.006, -0.001] |
| **Precentral Cortex** | -0.139 | 0.172 | 0.210 | [-0.004,0.001] |
| **Postcentral Cortex** | -0.217 | 0.034 | 0.068 | [-0.002,0.000] |
| **Superior Temporal Cortex** | -0.152 | 0.141 | 0.193 | [-0.001,0.001] |
| **Middle Temporal Cortex** | -0.214 | 0.037 | 0.069 | [-0.002,0.000] |
| **Inferior Temporal Cortex** | 0.026 | 0.801 | 0.801 | [-0.001,0.002] |
| **Cerebral Spinal Fluid** | -0.117 | 0.337 | 0.370 | [-0.018,0.002] |

*Notes*. CI, confidence interval.

**Supplementary Table S7. Regional correlation between brain tissue conductivity and mini‐mental state examination with regional brain volume as the covariate in cognitively normal controls.**

|  | Pearson’s *r* | *P*-value | corrected *P* | CI |
| --- | --- | --- | --- | --- |
| **Middle Frontal Cortex** | -0.141 | 0.379 | 1.000 | [-0.049,0.023] |
| **Orbital Frontal Cortex** | -0.007 | 0.963 | 1..000 | [-0.021,0.021] |
| **Insula Cortex** | 0.025 | 0.875 | 1.000 | [-0.029,0.039] |
| **Precuneus Cortex** | -0.144 | 0.366 | 1.000 | [-0.038,0.020] |
| **Posterior Cingulate Cortex** | -0.114 | 0.478 | 1.000 | [-0.029, 0.016] |
| **Precentral Cortex** | 0.054 | 0.734 | 1.000 | [-0.017,0.028] |
| **Postcentral Cortex** | -0.100 | 0.533 | 1.000 | [-0.018,0.007] |
| **Superior Temporal Cortex** | 0.079 | 0.623 | 1.000 | [-0.035,0.077] |
| **Middle Temporal Cortex** | 0.028 | 0.861 | 1.000 | [-0.010,0.012] |
| **Inferior Temporal Cortex** | -0.005 | 0.973 | 0.973 | [-0.015,0.002] |
| **Cerebral Spinal Fluid** | 0.101 | 0.525 | 1.000 | [-0.027,0.701] |

*Notes*. CI, confidence interval.

**Supplementary Table S8. Regional correlation between brain tissue conductivity and mini‐mental state examination with regional brain volume as the covariate in mild cognitive impairment.**

|  | Pearson’s *r* | *P*-value | corrected *P* | CI |
| --- | --- | --- | --- | --- |
| **Middle Frontal Cortex** | -0.515 | 0.009 | 0.099 | [-0.129,-0.001] |
| **Orbital Frontal Cortex** | -0.194 | 0.361 | 0.397 | [-0.037,0.014] |
| **Insula Cortex** | -0.336 | 0.107 | 0.235 | [-0.072,0.007] |
| **Precuneus Cortex** | -0.426 | 0.037 | 0.136 | [-0.073,-0.002] |
| **Posterior Cingulate Cortex** | -0.440 | 0.031 | 0.170 | [-0.056,0.000] |
| **Precentral Cortex** | -0.208 | 0.328 | 0.402 | [-0.055,0.019] |
| **Postcentral Cortex** | -0.223 | 0.293 | 0.403 | [-0.026,0.008] |
| **Superior Temporal Cortex** | -0.123 | 0.563 | 0.563 | [-0.055,0.039] |
| **Middle Temporal Cortex** | -0.239 | 0.259 | 0.408 | [-0.018,0.002] |
| **Inferior Temporal Cortex** | -0.372 | 0.072 | 0.198 | [-0.031,0.004] |
| **Cerebral Spinal Fluid** | -0.253 | 0.231 | 0.423 | [-0.026,0.006] |

*Notes*. CI, confidence interval.

**Supplementary Table S9. Regional correlation between brain tissue conductivity and mini‐mental state examination with regional brain volume as the covariate in dementia.**

|  | Pearson’s *r* | *P*-value | corrected *P* | CI |
| --- | --- | --- | --- | --- |
| **Middle Frontal Cortex** | -0.507 | 0.004 | 0.044 | [-0.022,-0.004] |
| **Orbital Frontal Cortex** | -0.396 | 0.024 | 0.132 | [-0.010,0.001] |
| **Insula Cortex** | -0.159 | 0.399 | 0.487 | [-0.011,0.005] |
| **Precuneus Cortex** | -0.302 | 0.092 | 0.168 | [-0.008,0.001] |
| **Posterior Cingulate Cortex** | -0.258 | 0.153 | 0.205 | [-0.009,0.001] |
| **Precentral Cortex** | -0.364 | 0.041 | 0.090 | [-0.007,0.000] |
| **Postcentral Cortex** | -0.337 | 0.038 | 0.113 | [-0.003,0.001] |
| **Superior Temporal Cortex** | -0.375 | 0.041 | 0.113 | [-0.019,0.002] |
| **Middle Temporal Cortex** | -0.281 | 0.131 | 0.210 | [-0.004,0.001] |
| **Inferior Temporal Cortex** | -0.053 | 0.780 | 0.858 | [-0.003,0.002] |
| **Cerebral Spinal Fluid** | 0.200 | 0.988 | 0.988 | [-0.412,0.415] |

*Notes*. CI, confidence interval.

**Supplementary Table S10. Regional correlation between brain tissue conductivity and mini‐mental state examination with regional brain volume as the covariate based on six subgroups.**

|  |  | Pearson’s *r* | *P*-value | corrected *P* | CI |
| --- | --- | --- | --- | --- | --- |
| **CN_N** | **Orbital Frontal Cortex** | -0.112 | 0.541 | 0.721 | [-0.022,0.016] |
|  | **Precuneus Cortex** | -0.169 | 0.355 | 0.709 | [-0.046,0.022] |
|  | **Posterior Cingulate Cortex** | -0.200 | 0.272 | 1.000 | [-0.038,0.016] |
|  | **Precentral Cortex** | -0.079 | 0.669 | 0.669 | [-0.024,0.025] |
| **CN_P** | **Orbital Frontal Cortex** | 0.226 | 0.557 | 0.743 | [-0.075,0.127] |
|  | **Precuneus Cortex** | 0.151 | 0.697 | 0.697 | [-0.065,0.084] |
|  | **Posterior Cingulate Cortex** | 0.605 | 0.084 | 0.337 | [-0.026,0.071] |
|  | **Precentral Cortex** | 0.571 | 0.107 | 0.214 | [-0.032,0.114] |
| **MCI_N** | **Orbital Frontal Cortex** | -0.191 | 0.551 | 1.000 | [-0.030,0.029] |
|  | **Precuneus Cortex** | -0.028 | 0.930 | 0.930 | [-0.049,0.051] |
|  | **Posterior Cingulate Cortex** | -0.497 | 0.101 | 0.402 | [-0.076,0.018] |
|  | **Precentral Cortex** | -0.167 | 0.603 | 0.804 | [-0.064,0.039] |
| **MCI_P** | **Orbital Frontal Cortex** | -0.259 | 0.392 | 0.522 | [-0.065,0.027] |
|  | **Precuneus Cortex** | -0.714 | 0.006 | 0.024 | [-0.115,-0.008] |
|  | **Posterior Cingulate Cortex** | -0.384 | 0.194 | 0.388 | [-0.066,0.016] |
|  | **Precentral Cortex** | -0.171 | 0.576 | 0.576 | [-0.079,0.047] |
| **Dem_N** | **Orbital Frontal Cortex** | 0.543 | 0.266 | 0.532 | [-0.033,0.062] |
|  | **Precuneus Cortex** | 0.365 | 0.476 | 0.634 | [-0.034, 0.060] |
|  | **Posterior Cingulate Cortex** | 0.285 | 0.585 | 0.585 | [-0.048,0.074] |
|  | **Precentral Cortex** | 0.702 | 0.120 | 0.48 | [-0.006,0.034] |
| **Dem_P** | **Orbital Frontal Cortex** | -0.379 | 0.062 | 0.082 | [-0.010, 0.000] |
|  | **Precuneus Cortex** | -0.443 | 0.027 | 0.054 | [-0.010,-0.001] |
|  | **Posterior Cingulate Cortex** | -0.312 | 0.129 | 0.129 | [-0.010,0.001] |
|  | **Precentral Cortex** | -0.451 | 0.024 | 0.096 | [-0.008,-0.001] |

*Notes*. CN_N, cognitively normal controls with Aβ negative; CN_P, cognitively normal controls with Aβ positive; MCI_N, mild cognitive impairment with Aβ negative; MCI_P, mild cognitive impairment with Aβ positive; Dem_N, dementia with Aβ negative; Dem_P, dementia with Aβ positive; CI, confidence interval.

**Supplementary Table S11. Regional correlation between brain tissue conductivity and plasma protein level with regional brain volume as the covariate.**

|  |  | Pearson’s *r* | *P*-value | corrected *P* | CI |
| --- | --- | --- | --- | --- | --- |
| **Aβ42/Aβ40** | **Middle Frontal Cortex** | -0.147 | 0.233 | 0.427 | [-2.111,0.523] |
|  | **Orbital Frontal Cortex** | 0.029 | 0.812 | 1.000 | [-0.686,0.677] |
|  | **Insula Cortex** | 0.207 | 0.123 | 0.338 | [0.164,2.178] |
|  | **Precuneus Cortex** | -0.206 | 0.088 | 0.323 | [-1.111,0.574] |
|  | **Posterior Cingulate Cortex** | 0.018 | 0.883 | 1.000 | [-0.726,0.834] |
|  | **Precentral Cortex** | -0.077 | 0.527 | 0.829 | [-0.877,0.442] |
|  | **Postcentral Cortex** | -0.015 | 0.905 | 1.000 | [-0.398,0.353] |
|  | **Superior Temporal Cortex** | 0.281 | 0.051 | 0.281 | [0.230,2.808] |
|  | **Middle Temporal Cortex** | 0.175 | 0.156 | 0.343 | [-0.083,0.509] |
|  | **Inferior Temporal Cortex** | -0.017 | 0.893 | 1.000 | [-0.453,0.396] |
|  | **Cerebral Spinal Fluid** | -0.313 | 0.008 | 0.088 | [-3.754,-0.483] |
| **Glial Fibrillary Acidic Protein** | **Middle Frontal Cortex** | 0.238 | 0.052 | 0.572 | [-0.000,0.001] |
|  | **Orbital Frontal Cortex** | 0.045 | 0.707 | 1.000 | [-0.000,0.000] |
|  | **Insula Cortex** | 0.159 | 0.198 | 0.594 | [-0.000,0.000] |
|  | **Precuneus Cortex** | 0.195 | 0.107 | 0.590 | [-0.000,0.000] |
|  | **Posterior Cingulate Cortex** | 0.095 | 0.433 | 0.974 | [-0.000,0.001] |
|  | **Precentral Cortex** | 0.262 | 0.029 | 0.319 | [0.000,0.001] |
|  | **Postcentral Cortex** | -0.109 | 0.379 | 0.799 | [-0.000, 0.000] |
|  | **Superior Temporal Cortex** | 0.021 | 0.867 | 1.000 | [-0.000, 0.000] |
|  | **Middle Temporal Cortex** | 0.127 | 0.307 | 0.676 | [-0.000,0.000] |
|  | **Inferior Temporal Cortex** | -0.044 | 0.724 | 1.000 | [-0.000, 0.000] |
|  | **Cerebral Spinal Fluid** | 0.264 | 0.028 | 0.308 | [-0.000,0.001] |
| **Neurofilament Light** | **Middle Frontal Cortex** | 0.290 | 0.027 | 0.297 | [-0.001, 0.002] |
|  | **Orbital Frontal Cortex** | 0.133 | 0.274 | 0.603 | [-0.000,0.001] |
|  | **Insula Cortex** | 0.098 | 0.430 | 0.932 | [-0.001, 0.001] |
|  | **Precuneus Cortex** | 0.087 | 0.473 | 1.000 | [-0.000,0.001] |
|  | **Posterior Cingulate Cortex** | 0.053 | 0.662 | 1.000 | [-0.001,0.001] |
|  | **Precentral Cortex** | 0.177 | 0.144 | 0.432 | [-0.000,0.001] |
|  | **Postcentral Cortex** | -0.116 | 0.351 | 0.777 | [-0.000, 0.000] |
|  | **Superior Temporal Cortex** | 0.095 | 0.443 | 0.974 | [-0.001, 0.002] |
|  | **Middle Temporal Cortex** | 0.190 | 0.124 | 0.341 | [-0.000, 0.001] |
|  | **Inferior Temporal Cortex** | -0.026 | 0.832 | 1.000 | [-0.001, 0.000] |
|  | **Cerebral Spinal Fluid** | 0.184 | 0.131 | 0.393 | [-0.002,0.004] |
| **Phosphorylated-Tau-181** | **Middle Frontal Cortex** | 0.183 | 0.120 | 0.420 | [-0.004, 0.020] |
|  | **Orbital Frontal Cortex** | 0.439 | 0.094 | 0.414 | [-0.004,0.009] |
|  | **Insula Cortex** | 0.090 | 0.467 | 0.514 | [-0.011, 0.008] |
|  | **Precuneus Cortex** | 0.181 | 0.135 | 0.424 | [-0.001,0.013] |
|  | **Posterior Cingulate Cortex** | 0.214 | 0.076 | 0.418 | [-0.001,0.013] |
|  | **Precentral Cortex** | 0.179 | 0.139 | 0.417 | [-0.001,0.010] |
|  | **Postcentral Cortex** | 0.163 | 0.188 | 0.439 | [-0.001, 0.006] |
|  | **Superior Temporal Cortex** | 0.036 | 0.772 | 0.849 | [-0.010, 0.014] |
|  | **Middle Temporal Cortex** | 0.111 | 0.369 | 0.507 | [-0.001, 0.004] |
|  | **Inferior Temporal Cortex** | -0.159 | 0.200 | 0.440 | [-0.006, 0.002] |
|  | **Cerebral Spinal Fluid** | 0.267 | 0.026 | 0.286 | [-0.006,0.043] |

*Notes*. CI, confidence interval.

**Supplementary Table S12. Regional correlation between cerebral spinal fluid conductivity and plasma protein level with regional brain volume as the covariate based on diagnosis status.**

|  |  | Pearson’s *r* | *P*-value | CI |
| --- | --- | --- | --- | --- |
| **Aβ42/Aβ40** | **CN** | -0.357 | 0.158 | [-9.950,2.147] |
|  | **MCI** | -0.353 | 0.059 | [-6.280,0.322] |
|  | **Dem** | -0.187 | 0.393 | [-8.148,4.438] |
| **Glial Fibrillary Acidic Protein** | **CN** | -0.046 | 0.859 | [-0.002,0.002] |
|  | **MCI** | 0.153 | 0.427 | [-0.001,0.002] |
|  | **Dem** | 0.462 | 0.026 | [0.000,0.001] |
| **Neurofilament Light** | **CN** | -0.151 | 0.560 | [-0.032,0.010] |
|  | **MCI** | -0.102 | 0.595 | [-0.011,0.006] |
|  | **Dem** | 0.497 | 0.016 | [-0.002,0.004] |
| **Phosphorylated-Tau-181** | **CN** | 0.231 | 0.373 | [-0.102,0.102] |
|  | **MCI** | 0.176 | 0.358 | [-0.032,0.054] |
|  | **Dem** | 0.429 | 0.041 | [-0.015,0.054] |

*Notes*. CI, confidence interval.

**Supplementary Table S13. Regional correlation between cerebral fluid conductivity and plasma protein level with regional brain volume as the covariate based on six subgroups.**

|  |  | Pearson’s *r* | *P*-value | corrected *P* | CI |
| --- | --- | --- | --- | --- | --- |
| **Aβ42/Aβ40** | **CN_N** | -0.263 | 0.262 | 0.393 | [-6.147,1.781] |
|  | **CN_P** | 0.183 | 0.692 | 0.692 | [-37.100,51.533] |
|  | **MCI_N** | -0.501 | 0.139 | 0.324 | [-14.000,-2.011] |
|  | **MCI_P** | -0.837 | 0.018 | 0.111 | [-11.082,1.874] |
|  | **Dem_N** | -0.649 | 0.162 | 0.324 | [-23.299,5.538] |
|  | **Dem_P** | -0.157 | 0.518 | 0.622 | [-6.987,5.479] |
| **Glial Fibrillary Acidic Protein** | **CN_N** | -0.142 | 0.547 | 0.716 | [-0.003,0.002] |
|  | **CN_P** | -0.152 | 0.744 | 0.744 | [-0.003,0.003] |
|  | **MCI_N** | -0.415 | 0.232 | 0.464 | [0.000,0.003] |
|  | **MCI_P** | 0.821 | 0.023 | 0.138 | [-0.004,0.001] |
|  | **Dem_N** | 0.274 | 0.597 | 0.716 | [-0.001,0.002] |
|  | **Dem_P** | 0.315 | 0.187 | 0.464 | [-0.001, 0.002] |
| **Neurofilament Light** | **CN_N** | -0.374 | 0.103 | 0.309 | [-0.019,0.008] |
|  | **CN_P** | -0.285 | 0.534 | 0.534 | [-0.051,0.034] |
|  | **MCI_N** | -0.323 | 0.361 | 0.541 | [0.002,0.0518] |
|  | **MCI_P** | 0.892 | 0.006 | 0.036 | [-0.018,0.007] |
|  | **Dem_N** | 0.428 | 0.396 | 0.475 | [-0.007,0.015] |
|  | **Dem_P** | 0.361 | 0.128 | 0.256 | [-0.004,0.003] |
| **Phosphorylated-Tau-181** | **CN_N** | 0.236 | 0.316 | 0.632 | [-0.051,0.088] |
|  | **CN_P** | -0.180 | 0.698 | 0.698 | [-0.297,0.215] |
|  | **MCI_N** | 0.428 | 0.337 | 0.505 | [-0.168, 0.233] |
|  | **MCI_P** | 0.382 | 0.275 | 0.825 | [-0.033,0.101] |
|  | **Dem_N** | -0.314 | 0.544 | 0.652 | [-0.074,0.085] |
|  | **Dem_P** | 0.401 | 0.088 | 0.528 | [-0.029,0.064] |

*Notes*. CN_N, cognitively normal controls with Aβ negative; CN_P, cognitively normal controls with Aβ positive; MCI_N, mild cognitive impairment with Aβ negative; MCI_P, mild cognitive impairment with Aβ positive; Dem_N, dementia with Aβ negative; Dem_P, dementia with Aβ positive; CI, confidence interval.

**Supplementary Table S14. Area under receiver operating characteristic (ROC) curve to evaluate the addition of conductivity values to the gray matter volume values for group classification.**

|  | **CN-MCI** | **MCI-Dem** | **CN-Dem** |
| --- | --- | --- | --- |
| **MTC GMV** | 0.775 | 0.691 | 0.785 |
| **CSF conductivity + Precuneus conductivity** | 0.721 | 0.654 | 0.801 |
| **CSF conductivity + OFC conductivity** | 0.764 | 0.610 | 0.720 |
| **CSF conductivity + Precentral conductivity** | 0.773 | 0.507 | 0.698 |
| **CSF conductivity + Precuneus conductivity + MTC GMV** | 0.852 | 0.731 | 0.862 |
| **CSF conductivity + OFC conductivity + MTC GMV** | 0.822 | 0.702 | 0.754 |
| **CSF conductivity + Precentral conductivity+ MTC GMV** | 0.791 | 0.633 | 0.796 |

*Notes*. CN, cognitively normal controls; Dem, dementia; CSF, cerebral spinal fluid; GMV, gray matter volume; MCI, mild cognitive impairment; MTC, middle temporal cortex; OFC, orbital frontal cortex.
