# Supplementary material for: Brain tissue electrical conductivity as a promising biomarker for dementia assessment using MRI
Source: Alzheimers Dement. 2025 Jun 23;21(6):e70270. doi: 10.1002/alz.70270 (PMC12185248; doi:10.1002/alz.70270)
Supplement: Supplementary file 2 — Supporting Information [file ALZ-21-e70270-s010.docx]

**Table S15.** The complete set of PLS2 gene weights and associated statistics for conductivity difference between Dementia and cognitively normal participants.

| **geneIDs** | **geneIDX** | **zscores** | **pvals** | **adj.pvals** | **significant** |
| --- | --- | --- | --- | --- | --- |
| SHD | 12208 | 6.056471 | 1.39E-09 | 7.25E-06 | TRUE |
| NIPAL2 | 12809 | 5.992403 | 2.07E-09 | 8.08E-06 | TRUE |
| KCNC1 | 12915 | 5.843662 | 5.11E-09 | 1.41E-05 | TRUE |
| EPB41 | 14436 | 5.657165 | 1.54E-08 | 1.95E-05 | TRUE |
| CCDC39 | 3627 | 5.647755 | 1.63E-08 | 1.95E-05 | TRUE |
| LOC101926935 | 4112 | 5.620896 | 1.90E-08 | 2.12E-05 | TRUE |
| SLC38A2 | 7289 | 5.580218 | 2.40E-08 | 2.21E-05 | TRUE |
| PCDH7 | 500 | 5.542962 | 2.97E-08 | 2.32E-05 | TRUE |
| PPARGC1A | 1320 | 5.530609 | 3.19E-08 | 2.32E-05 | TRUE |
| PDZD8 | 9517 | 5.525421 | 3.29E-08 | 2.32E-05 | TRUE |
| C1RL | 14541 | 5.517799 | 3.43E-08 | 2.32E-05 | TRUE |
| SYT2 | 2820 | 5.473932 | 4.40E-08 | 2.75E-05 | TRUE |
| CUX1 | 11436 | 5.441048 | 5.30E-08 | 3.18E-05 | TRUE |
| SLC25A37 | 5920 | 5.385392 | 7.23E-08 | 4.04E-05 | TRUE |
| PTPN2 | 9608 | 5.351465 | 8.72E-08 | 4.26E-05 | TRUE |
| GPLD1 | 2541 | 5.295333 | 1.19E-07 | 4.89E-05 | TRUE |
| GPAT3 | 14995 | 5.268016 | 1.38E-07 | 5.39E-05 | TRUE |
| PPP1R16B | 12113 | 5.252514 | 1.50E-07 | 5.60E-05 | TRUE |
| FAM13B | 601 | 5.251047 | 1.51E-07 | 5.60E-05 | TRUE |
| RORB | 3413 | 5.247676 | 1.54E-07 | 5.60E-05 | TRUE |
| TTC39B | 9823 | 5.243572 | 1.57E-07 | 5.60E-05 | TRUE |
| RBBP4 | 9900 | 5.225217 | 1.74E-07 | 5.85E-05 | TRUE |
| USP45 | 10885 | 5.223308 | 1.76E-07 | 5.85E-05 | TRUE |
| VWC2 | 7434 | 5.20979 | 1.89E-07 | 6.05E-05 | TRUE |
| OIP5-AS1 | 1530 | 5.208743 | 1.90E-07 | 6.05E-05 | TRUE |
| EYA4 | 11608 | 5.196044 | 2.04E-07 | 6.09E-05 | TRUE |
| SERTAD4-AS1 | 5056 | 5.172244 | 2.31E-07 | 6.50E-05 | TRUE |
| IFNLR1 | 6660 | 5.155501 | 2.53E-07 | 6.70E-05 | TRUE |
| SORCS1 | 7960 | 5.123659 | 3.00E-07 | 7.32E-05 | TRUE |
| EEF2K | 6332 | 5.095613 | 3.48E-07 | 7.96E-05 | TRUE |
| NOM1 | 13333 | 5.084691 | 3.68E-07 | 7.96E-05 | TRUE |
| HMCES | 586 | 5.082513 | 3.72E-07 | 7.96E-05 | TRUE |
| LINC01296 | 5135 | 5.078313 | 3.81E-07 | 7.96E-05 | TRUE |
| MIR29B2CHG | 9626 | 5.071348 | 3.95E-07 | 8.02E-05 | TRUE |
| SNHG1 | 4233 | 5.048388 | 4.46E-07 | 8.29E-05 | TRUE |
| TAS2R30 | 6914 | 5.043949 | 4.56E-07 | 8.29E-05 | TRUE |
| MIR600HG | 6408 | 5.037531 | 4.72E-07 | 8.47E-05 | TRUE |
| FER1L4 | 3009 | 5.028466 | 4.94E-07 | 8.78E-05 | TRUE |
| STARD9 | 8028 | 5.018766 | 5.20E-07 | 9.01E-05 | TRUE |
| ST3GAL5 | 6127 | 5.017074 | 5.25E-07 | 9.01E-05 | TRUE |
| TRIP11 | 4329 | 4.991698 | 5.99E-07 | 9.76E-05 | TRUE |
| ITPKC | 7571 | 4.991514 | 5.99E-07 | 9.76E-05 | TRUE |
| SKIDA1 | 9952 | 4.985326 | 6.19E-07 | 9.87E-05 | TRUE |
| SLC38A11 | 4947 | 4.974185 | 6.55E-07 | 0.000102 | TRUE |
| ZNF268 | 4051 | 4.970888 | 6.66E-07 | 0.000103 | TRUE |
| CTSC | 12091 | 4.968829 | 6.74E-07 | 0.000103 | TRUE |
| CDH7 | 10640 | 4.941882 | 7.74E-07 | 0.000112 | TRUE |
| ANKRD20A4-ANKRD20A20P | 5284 | 4.892609 | 9.95E-07 | 0.000132 | TRUE |
| TDRD3 | 13405 | 4.890747 | 1.00E-06 | 0.000132 | TRUE |
| ANKS6 | 4920 | 4.88844 | 1.02E-06 | 0.000132 | TRUE |
| PIK3CB | 8413 | 4.87147 | 1.11E-06 | 0.00014 | TRUE |
| PANX2 | 1644 | 4.86258 | 1.16E-06 | 0.000143 | TRUE |
| ZNF362 | 5379 | 4.846077 | 1.26E-06 | 0.00015 | TRUE |
| FGD5 | 8964 | 4.828646 | 1.37E-06 | 0.000157 | TRUE |
| COQ10B | 13997 | 4.825304 | 1.40E-06 | 0.000157 | TRUE |
| ANKRD20A8P | 40 | 4.801857 | 1.57E-06 | 0.000173 | TRUE |
| SLC25A36 | 4033 | 4.801071 | 1.58E-06 | 0.000173 | TRUE |
| RTKN2 | 8324 | 4.793476 | 1.64E-06 | 0.000176 | TRUE |
| CHI3L1 | 11864 | 4.793152 | 1.64E-06 | 0.000176 | TRUE |
| TUBD1 | 154 | 4.786571 | 1.70E-06 | 0.000177 | TRUE |
| HSPA4L | 14941 | 4.78528 | 1.71E-06 | 0.000177 | TRUE |
| HS3ST1 | 12447 | 4.778911 | 1.76E-06 | 0.000179 | TRUE |
| EEPD1 | 6347 | 4.770766 | 1.84E-06 | 0.000181 | TRUE |
| PLCD4 | 10787 | 4.765701 | 1.88E-06 | 0.000181 | TRUE |
| SMIM3 | 11650 | 4.763918 | 1.90E-06 | 0.000181 | TRUE |
| IDE | 7407 | 4.758054 | 1.95E-06 | 0.000183 | TRUE |
| TENM4 | 9635 | 4.753984 | 1.99E-06 | 0.000183 | TRUE |
| ASB13 | 4449 | 4.749585 | 2.04E-06 | 0.000183 | TRUE |
| SLC25A12 | 10585 | 4.748759 | 2.05E-06 | 0.000183 | TRUE |
| TCHP | 12592 | 4.747053 | 2.06E-06 | 0.000183 | TRUE |
| LINC00515 | 4242 | 4.739923 | 2.14E-06 | 0.000186 | TRUE |
| FASTKD1 | 3209 | 4.739908 | 2.14E-06 | 0.000186 | TRUE |
| USP8 | 14047 | 4.734325 | 2.20E-06 | 0.00019 | TRUE |
| KCNA1 | 9249 | 4.727451 | 2.27E-06 | 0.000193 | TRUE |
| GTF2H2B | 10957 | 4.724422 | 2.31E-06 | 0.000193 | TRUE |
| SERTAD4 | 12451 | 4.724357 | 2.31E-06 | 0.000193 | TRUE |
| PTPRD-AS1 | 13137 | 4.719496 | 2.36E-06 | 0.000197 | TRUE |
| TDRD1 | 2688 | 4.710621 | 2.47E-06 | 0.000202 | TRUE |
| AHI1 | 9638 | 4.691142 | 2.72E-06 | 0.000219 | TRUE |
| KBTBD2 | 15358 | 4.67211 | 2.98E-06 | 0.000237 | TRUE |
| DNAAF2 | 8639 | 4.661752 | 3.14E-06 | 0.000245 | TRUE |
| LOC729737 | 6952 | 4.660537 | 3.15E-06 | 0.000245 | TRUE |
| XKR6 | 6995 | 4.655284 | 3.24E-06 | 0.00025 | TRUE |
| FLT3 | 15242 | 4.654794 | 3.24E-06 | 0.00025 | TRUE |
| FMN1 | 10653 | 4.652402 | 3.28E-06 | 0.000251 | TRUE |
| OVGP1 | 13095 | 4.630644 | 3.65E-06 | 0.000274 | TRUE |
| FAM20A | 11818 | 4.628409 | 3.68E-06 | 0.000276 | TRUE |
| KCNQ5 | 8670 | 4.625596 | 3.74E-06 | 0.000277 | TRUE |
| EPB41L1 | 7081 | 4.625254 | 3.74E-06 | 0.000277 | TRUE |
| LIN28B | 5416 | 4.606717 | 4.09E-06 | 0.000298 | TRUE |
| FAM71E1 | 12238 | 4.604631 | 4.13E-06 | 0.000298 | TRUE |
| LYSMD4 | 4892 | 4.590187 | 4.43E-06 | 0.00031 | TRUE |
| IFFO2 | 5710 | 4.589024 | 4.45E-06 | 0.000311 | TRUE |
| WDR19 | 11031 | 4.585734 | 4.52E-06 | 0.000313 | TRUE |
| SEMA4C | 5069 | 4.582244 | 4.60E-06 | 0.000314 | TRUE |
| UPP1 | 7525 | 4.554605 | 5.25E-06 | 0.000348 | TRUE |
| EIF4E1B | 6243 | 4.551596 | 5.32E-06 | 0.000348 | TRUE |
| STRN | 2551 | 4.550666 | 5.35E-06 | 0.000348 | TRUE |
| ATP4A | 11980 | 4.548641 | 5.40E-06 | 0.00035 | TRUE |
| LIN9 | 3684 | 4.545867 | 5.47E-06 | 0.000352 | TRUE |
| AMMECR1 | 567 | 4.541899 | 5.57E-06 | 0.000356 | TRUE |
| EXOG | 14056 | 4.539553 | 5.64E-06 | 0.000358 | TRUE |
| FRAT2 | 7747 | 4.533372 | 5.80E-06 | 0.000363 | TRUE |
| L2HGDH | 6117 | 4.530153 | 5.89E-06 | 0.000366 | TRUE |
| SLC4A8 | 7919 | 4.528288 | 5.95E-06 | 0.000366 | TRUE |
| PIM3 | 3977 | 4.526693 | 5.99E-06 | 0.000366 | TRUE |
| ECM1 | 5205 | 4.526492 | 6.00E-06 | 0.000366 | TRUE |
| SNX12 | 14243 | 4.523631 | 6.08E-06 | 0.000369 | TRUE |
| DCP1A | 5233 | 4.523385 | 6.09E-06 | 0.000369 | TRUE |
| GUSBP4 | 12779 | 4.517584 | 6.25E-06 | 0.000375 | TRUE |
| NT5M | 825 | 4.517546 | 6.26E-06 | 0.000375 | TRUE |
| LINC01102 | 4163 | 4.516842 | 6.28E-06 | 0.000375 | TRUE |
| ZDHHC2 | 1022 | 4.509081 | 6.51E-06 | 0.000387 | TRUE |
| NR3C2 | 974 | 4.507284 | 6.57E-06 | 0.000389 | TRUE |
| SEMA6D | 14023 | 4.494696 | 6.97E-06 | 0.000402 | TRUE |
| ACVR1C | 10874 | 4.492645 | 7.03E-06 | 0.000404 | TRUE |
| GIGYF1 | 6942 | 4.488361 | 7.18E-06 | 0.000411 | TRUE |
| POU2F2 | 13370 | 4.472966 | 7.71E-06 | 0.000435 | TRUE |
| GLIPR1 | 10427 | 4.470291 | 7.81E-06 | 0.000439 | TRUE |
| HTR1F | 11477 | 4.467843 | 7.90E-06 | 0.00044 | TRUE |
| LOC220729 | 2149 | 4.464829 | 8.01E-06 | 0.000441 | TRUE |
| TDRD6 | 812 | 4.451766 | 8.52E-06 | 0.000464 | TRUE |
| GPCPD1 | 11512 | 4.449369 | 8.61E-06 | 0.000466 | TRUE |
| TRANK1 | 14506 | 4.437642 | 9.09E-06 | 0.000487 | TRUE |
| PHYH | 3985 | 4.436486 | 9.14E-06 | 0.000487 | TRUE |
| PEG3 | 6133 | 4.433757 | 9.26E-06 | 0.000488 | TRUE |
| USP49 | 252 | 4.432942 | 9.30E-06 | 0.000488 | TRUE |
| NR2C1 | 3769 | 4.430032 | 9.42E-06 | 0.000488 | TRUE |
| C4orf33 | 7032 | 4.429335 | 9.45E-06 | 0.000488 | TRUE |
| LAG3 | 11944 | 4.421446 | 9.80E-06 | 0.000501 | TRUE |
| ITM2A | 4599 | 4.419611 | 9.89E-06 | 0.000502 | TRUE |
| FAM110A | 2304 | 4.414298 | 1.01E-05 | 0.000513 | TRUE |
| ATXN7 | 5177 | 4.405753 | 1.05E-05 | 0.000528 | TRUE |
| SNTG1 | 13283 | 4.397143 | 1.10E-05 | 0.000544 | TRUE |
| LYST | 14032 | 4.389124 | 1.14E-05 | 0.000557 | TRUE |
| ATP2B2 | 4558 | 4.388398 | 1.14E-05 | 0.000557 | TRUE |
| PRDM11 | 13148 | 4.387195 | 1.15E-05 | 0.000557 | TRUE |
| VAMP1 | 5873 | 4.383792 | 1.17E-05 | 0.000557 | TRUE |
| RAD54B | 7978 | 4.383309 | 1.17E-05 | 0.000557 | TRUE |
| ETS2 | 13644 | 4.382194 | 1.17E-05 | 0.000557 | TRUE |
| DNAAF5 | 14288 | 4.374402 | 1.22E-05 | 0.000565 | TRUE |
| LINC00476 | 8937 | 4.373124 | 1.22E-05 | 0.000565 | TRUE |
| CA13 | 3900 | 4.365937 | 1.27E-05 | 0.00058 | TRUE |
| PLCXD1 | 3508 | 4.364799 | 1.27E-05 | 0.000582 | TRUE |
| GUCA2B | 6242 | 4.363225 | 1.28E-05 | 0.000583 | TRUE |
| PANK1 | 8887 | 4.351065 | 1.35E-05 | 0.000606 | TRUE |
| KCNK12 | 3020 | 4.336498 | 1.45E-05 | 0.000634 | TRUE |
| IGLL1 | 605 | 4.331047 | 1.48E-05 | 0.000647 | TRUE |
| ANK1 | 6151 | 4.330943 | 1.48E-05 | 0.000647 | TRUE |
| RASSF4 | 11777 | 4.329208 | 1.50E-05 | 0.00065 | TRUE |
| ZMAT4 | 14850 | 4.326284 | 1.52E-05 | 0.000655 | TRUE |
| RWDD1 | 6663 | 4.324152 | 1.53E-05 | 0.000659 | TRUE |
| TMEM132E | 1142 | 4.320595 | 1.56E-05 | 0.000665 | TRUE |
| ST3GAL2 | 10859 | 4.319549 | 1.56E-05 | 0.000665 | TRUE |
| COX7A1 | 1255 | 4.318991 | 1.57E-05 | 0.000665 | TRUE |
| ESRRA | 11289 | 4.318883 | 1.57E-05 | 0.000665 | TRUE |
| GPHN | 14830 | 4.296959 | 1.73E-05 | 0.00072 | TRUE |
| CCDC57 | 7709 | 4.293538 | 1.76E-05 | 0.000722 | TRUE |
| AGO3 | 8906 | 4.293372 | 1.76E-05 | 0.000722 | TRUE |
| KCNH1 | 3594 | 4.286568 | 1.81E-05 | 0.000732 | TRUE |
| ARNTL | 3163 | 4.286097 | 1.82E-05 | 0.000732 | TRUE |
| MYBPHL | 3994 | 4.28355 | 1.84E-05 | 0.000737 | TRUE |
| TRMT10A | 8718 | 4.279748 | 1.87E-05 | 0.000744 | TRUE |
| BHLHE40 | 15174 | 4.274073 | 1.92E-05 | 0.000754 | TRUE |
| PDCD6IP | 6075 | 4.268939 | 1.96E-05 | 0.000764 | TRUE |
| DDX55 | 5861 | 4.26317 | 2.02E-05 | 0.000776 | TRUE |
| SOCS5 | 15359 | 4.26218 | 2.02E-05 | 0.000776 | TRUE |
| TPTE2P6 | 7612 | 4.260924 | 2.04E-05 | 0.000778 | TRUE |
| BICRAL | 12407 | 4.258982 | 2.05E-05 | 0.000782 | TRUE |
| JAK2 | 4874 | 4.258423 | 2.06E-05 | 0.000782 | TRUE |
| LOC101928087 | 7088 | 4.245855 | 2.18E-05 | 0.000811 | TRUE |
| PDCD7 | 10262 | 4.241661 | 2.22E-05 | 0.00082 | TRUE |
| KCNAB3 | 6540 | 4.23566 | 2.28E-05 | 0.000838 | TRUE |
| KCNJ9 | 11113 | 4.230641 | 2.33E-05 | 0.000852 | TRUE |
| SYNE4 | 4642 | 4.229875 | 2.34E-05 | 0.000852 | TRUE |
| NAALAD2 | 7235 | 4.228799 | 2.35E-05 | 0.000853 | TRUE |
| LIAS | 8936 | 4.226211 | 2.38E-05 | 0.000856 | TRUE |
| SCN1B | 9815 | 4.223647 | 2.40E-05 | 0.000864 | TRUE |
| RALB | 4361 | 4.221193 | 2.43E-05 | 0.000865 | TRUE |
| FAM126B | 1700 | 4.218429 | 2.46E-05 | 0.000873 | TRUE |
| STPG1 | 15615 | 4.218146 | 2.46E-05 | 0.000873 | TRUE |
| FGF7 | 12810 | 4.212743 | 2.52E-05 | 0.000886 | TRUE |
| WDR97 | 1969 | 4.211342 | 2.54E-05 | 0.00089 | TRUE |
| CELF2 | 5811 | 4.203623 | 2.63E-05 | 0.000911 | TRUE |
| KALRN | 6208 | 4.200651 | 2.66E-05 | 0.000916 | TRUE |
| TBR1 | 6108 | 4.193563 | 2.75E-05 | 0.000941 | TRUE |
| CTTNBP2 | 7585 | 4.192879 | 2.75E-05 | 0.000941 | TRUE |
| TNRC18 | 10239 | 4.192425 | 2.76E-05 | 0.000941 | TRUE |
| ZNF34 | 15319 | 4.185288 | 2.85E-05 | 0.000966 | TRUE |
| METTL15 | 2410 | 4.184457 | 2.86E-05 | 0.000967 | TRUE |
| TMEM132C | 8379 | 4.177477 | 2.95E-05 | 0.00099 | TRUE |
| ICA1 | 11152 | 4.176801 | 2.96E-05 | 0.00099 | TRUE |
| TACC1 | 9358 | 4.169374 | 3.05E-05 | 0.001014 | TRUE |
| MTUS2 | 9831 | 4.167241 | 3.08E-05 | 0.001021 | TRUE |
| MIR124-2HG | 5194 | 4.160803 | 3.17E-05 | 0.001036 | TRUE |
| LINC00999 | 9827 | 4.158545 | 3.20E-05 | 0.001041 | TRUE |
| HYDIN2 | 11156 | 4.157327 | 3.22E-05 | 0.001042 | TRUE |
| CHIC1 | 7438 | 4.155221 | 3.25E-05 | 0.001045 | TRUE |
| ARHGEF7 | 14933 | 4.154521 | 3.26E-05 | 0.001045 | TRUE |
| HR | 6308 | 4.145852 | 3.39E-05 | 0.001074 | TRUE |
| IPW | 2507 | 4.14475 | 3.40E-05 | 0.001077 | TRUE |
| ZZEF1 | 8950 | 4.142836 | 3.43E-05 | 0.001083 | TRUE |
| BEND6 | 4432 | 4.142478 | 3.44E-05 | 0.001083 | TRUE |
| THAP10 | 15187 | 4.14068 | 3.46E-05 | 0.001085 | TRUE |
| ZCCHC8 | 7995 | 4.140543 | 3.46E-05 | 0.001085 | TRUE |
| LINC01158 | 496 | 4.135218 | 3.55E-05 | 0.001104 | TRUE |
| NFIX | 13154 | 4.131147 | 3.61E-05 | 0.001118 | TRUE |
| MCTS1 | 11776 | 4.130044 | 3.63E-05 | 0.001121 | TRUE |
| SMIM13 | 1009 | 4.129061 | 3.64E-05 | 0.001123 | TRUE |
| PLXNA4 | 12114 | 4.12809 | 3.66E-05 | 0.001126 | TRUE |
| DHX35 | 9901 | 4.12723 | 3.67E-05 | 0.001128 | TRUE |
| B4GALT6 | 10183 | 4.118389 | 3.82E-05 | 0.001153 | TRUE |
| COL7A1 | 6593 | 4.118229 | 3.82E-05 | 0.001153 | TRUE |
| PRICKLE1 | 13926 | 4.111513 | 3.93E-05 | 0.001175 | TRUE |
| ANKRD33B | 7250 | 4.110982 | 3.94E-05 | 0.001175 | TRUE |
| NHLRC2 | 13082 | 4.105891 | 4.03E-05 | 0.001198 | TRUE |
| ZNF629 | 10599 | 4.102551 | 4.09E-05 | 0.001212 | TRUE |
| KIAA1551 | 10931 | 4.101064 | 4.11E-05 | 0.001215 | TRUE |
| NDUFS1 | 3945 | 4.098455 | 4.16E-05 | 0.001227 | TRUE |
| CREBRF | 4634 | 4.092405 | 4.27E-05 | 0.001252 | TRUE |
| LIN52 | 13749 | 4.087848 | 4.35E-05 | 0.001271 | TRUE |
| KBTBD3 | 1690 | 4.085176 | 4.40E-05 | 0.001279 | TRUE |
| KCNS2 | 1069 | 4.084444 | 4.42E-05 | 0.001279 | TRUE |
| HSPBAP1 | 14962 | 4.081502 | 4.47E-05 | 0.001286 | TRUE |
| RET | 12725 | 4.081134 | 4.48E-05 | 0.001286 | TRUE |
| SACM1L | 1915 | 4.080544 | 4.49E-05 | 0.001286 | TRUE |
| GPR161 | 7944 | 4.080059 | 4.50E-05 | 0.001287 | TRUE |
| GCC2 | 13562 | 4.076752 | 4.57E-05 | 0.0013 | TRUE |
| MAGI2-AS3 | 1117 | 4.072892 | 4.64E-05 | 0.001317 | TRUE |
| ANO5 | 4645 | 4.071405 | 4.67E-05 | 0.001321 | TRUE |
| NAT8L | 12044 | 4.07027 | 4.70E-05 | 0.001325 | TRUE |
| CCDC58 | 4377 | 4.066983 | 4.76E-05 | 0.001337 | TRUE |
| SCLT1 | 4212 | 4.064752 | 4.81E-05 | 0.001342 | TRUE |
| ERCC4 | 802 | 4.0646 | 4.81E-05 | 0.001342 | TRUE |
| KLF12 | 8768 | 4.05821 | 4.95E-05 | 0.001373 | TRUE |
| ADCY3 | 9825 | 4.056229 | 4.99E-05 | 0.001378 | TRUE |
| ALDH1A3 | 3949 | 4.041796 | 5.30E-05 | 0.001447 | TRUE |
| TFB2M | 15086 | 4.03225 | 5.52E-05 | 0.001486 | TRUE |
| SCARB2 | 7441 | 4.02868 | 5.61E-05 | 0.001504 | TRUE |
| EPB41L3 | 15364 | 4.026205 | 5.67E-05 | 0.001517 | TRUE |
| EIF5A2 | 806 | 4.024028 | 5.72E-05 | 0.001529 | TRUE |
| SLC44A5 | 2720 | 4.021022 | 5.79E-05 | 0.001543 | TRUE |
| NEIL1 | 14562 | 4.015765 | 5.93E-05 | 0.00157 | TRUE |
| ST3GAL6 | 5852 | 4.014062 | 5.97E-05 | 0.001577 | TRUE |
| LINC00599 | 9921 | 4.013279 | 5.99E-05 | 0.001577 | TRUE |
| CADPS2 | 13105 | 4.010288 | 6.06E-05 | 0.001588 | TRUE |
| RHBDL3 | 1746 | 4.009342 | 6.09E-05 | 0.001592 | TRUE |
| LOC100289230 | 769 | 4.005734 | 6.18E-05 | 0.001611 | TRUE |
| PPM1H | 14996 | 4.003638 | 6.24E-05 | 0.001614 | TRUE |
| ZNF445 | 11263 | 4.001305 | 6.30E-05 | 0.001625 | TRUE |
| SLC6A6 | 8726 | 3.99984 | 6.34E-05 | 0.001632 | TRUE |
| SEPHS1 | 13680 | 3.997175 | 6.41E-05 | 0.001646 | TRUE |
| AGO1 | 341 | 3.989986 | 6.61E-05 | 0.001693 | TRUE |
| C16orf74 | 6444 | 3.989079 | 6.63E-05 | 0.001693 | TRUE |
| LMBR1 | 6263 | 3.988439 | 6.65E-05 | 0.001693 | TRUE |
| SLC5A6 | 7311 | 3.987334 | 6.68E-05 | 0.001695 | TRUE |
| SYCP2 | 10783 | 3.98388 | 6.78E-05 | 0.00171 | TRUE |
| FRAT1 | 13187 | 3.982316 | 6.82E-05 | 0.001713 | TRUE |
| PAX8-AS1 | 10834 | 3.982287 | 6.83E-05 | 0.001713 | TRUE |
| MFHAS1 | 1628 | 3.977492 | 6.96E-05 | 0.001731 | TRUE |
| CCND2-AS1 | 343 | 3.977254 | 6.97E-05 | 0.001731 | TRUE |
| RPRD2 | 9797 | 3.972655 | 7.11E-05 | 0.001758 | TRUE |
| SLC17A6 | 13014 | 3.971335 | 7.15E-05 | 0.001765 | TRUE |
| ZBTB16 | 3277 | 3.969361 | 7.21E-05 | 0.001774 | TRUE |
| MCF2L2 | 6002 | 3.967666 | 7.26E-05 | 0.001784 | TRUE |
| SORBS1 | 10351 | 3.966905 | 7.28E-05 | 0.001786 | TRUE |
| PPP1R15B | 11786 | 3.966395 | 7.30E-05 | 0.001786 | TRUE |
| PJVK | 12541 | 3.961129 | 7.46E-05 | 0.001811 | TRUE |
| TLE2 | 7258 | 3.958305 | 7.55E-05 | 0.001824 | TRUE |
| VAV3 | 14663 | 3.953164 | 7.71E-05 | 0.001855 | TRUE |
| NFATC3 | 12026 | 3.951296 | 7.77E-05 | 0.001867 | TRUE |
| EDEM1 | 420 | 3.949805 | 7.82E-05 | 0.001875 | TRUE |
| CDS1 | 7580 | 3.94677 | 7.92E-05 | 0.001893 | TRUE |
| RALGAPA2 | 4464 | 3.940804 | 8.12E-05 | 0.001931 | TRUE |
| USP13 | 15488 | 3.940273 | 8.14E-05 | 0.001931 | TRUE |
| FAM131B | 15090 | 3.939401 | 8.17E-05 | 0.001933 | TRUE |
| GFM1 | 9467 | 3.935745 | 8.29E-05 | 0.001953 | TRUE |
| LOC400682 | 4314 | 3.933745 | 8.36E-05 | 0.001963 | TRUE |
| ENTPD4 | 14822 | 3.932155 | 8.42E-05 | 0.001973 | TRUE |
| RMND5A | 6973 | 3.930496 | 8.48E-05 | 0.001982 | TRUE |
| OXNAD1 | 4837 | 3.928443 | 8.55E-05 | 0.001991 | TRUE |
| FSTL1 | 13072 | 3.927263 | 8.59E-05 | 0.001995 | TRUE |
| TMTC4 | 3382 | 3.927016 | 8.60E-05 | 0.001995 | TRUE |
| PXYLP1 | 12429 | 3.923615 | 8.72E-05 | 0.002008 | TRUE |
| MIR4697HG | 6387 | 3.915719 | 9.01E-05 | 0.002061 | TRUE |
| IDI2-AS1 | 8719 | 3.915576 | 9.02E-05 | 0.002061 | TRUE |
| FAM135B | 1735 | 3.914481 | 9.06E-05 | 0.002064 | TRUE |
| ATP8B2 | 3598 | 3.914209 | 9.07E-05 | 0.002064 | TRUE |
| DUSP22 | 9913 | 3.913313 | 9.10E-05 | 0.002066 | TRUE |
| ZNF714 | 9646 | 3.909749 | 9.24E-05 | 0.002088 | TRUE |
| KCNT1 | 4746 | 3.901486 | 9.56E-05 | 0.002141 | TRUE |
| PDIA5 | 10796 | 3.894095 | 9.86E-05 | 0.002198 | TRUE |
| PLAGL2 | 14050 | 3.891972 | 9.94E-05 | 0.002208 | TRUE |
| WIPF3 | 14397 | 3.88854 | 0.000101 | 0.002225 | TRUE |
| LCA5 | 1390 | 3.888518 | 0.000101 | 0.002225 | TRUE |
| MID2 | 4116 | 3.888371 | 0.000101 | 0.002225 | TRUE |
| SRRM4 | 14429 | 3.887501 | 0.000101 | 0.00223 | TRUE |
| LOC100506124 | 1802 | 3.886043 | 0.000102 | 0.00224 | TRUE |
| GLS2 | 8681 | 3.883495 | 0.000103 | 0.002261 | TRUE |
| TMEM223 | 5511 | 3.878899 | 0.000105 | 0.002294 | TRUE |
| ESCO1 | 2225 | 3.876161 | 0.000106 | 0.002317 | TRUE |
| TOP1P2 | 13088 | 3.87491 | 0.000107 | 0.002326 | TRUE |
| ZNF662 | 13357 | 3.871201 | 0.000108 | 0.002355 | TRUE |
| CCNO | 9148 | 3.871131 | 0.000108 | 0.002355 | TRUE |
| VWA8 | 9160 | 3.869492 | 0.000109 | 0.002363 | TRUE |
| OTUD7A | 7346 | 3.865233 | 0.000111 | 0.002396 | TRUE |
| ARHGEF17 | 13800 | 3.862622 | 0.000112 | 0.002409 | TRUE |
| HDAC7 | 3266 | 3.861278 | 0.000113 | 0.002419 | TRUE |
| ZBTB7A | 11272 | 3.854324 | 0.000116 | 0.002468 | TRUE |
| MET | 14162 | 3.851997 | 0.000117 | 0.002481 | TRUE |
| MTAP | 10329 | 3.851212 | 0.000118 | 0.002481 | TRUE |
| MICB | 7773 | 3.851188 | 0.000118 | 0.002481 | TRUE |
| TIFA | 8115 | 3.850425 | 0.000118 | 0.002481 | TRUE |
| NRDE2 | 11438 | 3.846687 | 0.00012 | 0.002506 | TRUE |
| NR1D1 | 12544 | 3.846285 | 0.00012 | 0.002506 | TRUE |
| DUSP8 | 9288 | 3.843197 | 0.000121 | 0.002527 | TRUE |
| CDK13 | 5543 | 3.834578 | 0.000126 | 0.002598 | TRUE |
| TRMT2B | 6349 | 3.833736 | 0.000126 | 0.002603 | TRUE |
| TSPAN13 | 14161 | 3.83119 | 0.000128 | 0.002623 | TRUE |
| AMD1 | 6286 | 3.828902 | 0.000129 | 0.002644 | TRUE |
| KMT2E | 4453 | 3.827694 | 0.000129 | 0.002654 | TRUE |
| RRM2B | 3081 | 3.825147 | 0.000131 | 0.002674 | TRUE |
| PLEKHH3 | 8261 | 3.824729 | 0.000131 | 0.002675 | TRUE |
| H1F0 | 1542 | 3.822897 | 0.000132 | 0.002687 | TRUE |
| VCPIP1 | 13878 | 3.822724 | 0.000132 | 0.002687 | TRUE |
| LRRK2 | 603 | 3.819468 | 0.000134 | 0.002715 | TRUE |
| DDX3X | 2496 | 3.815656 | 0.000136 | 0.00275 | TRUE |
| PLK5 | 11847 | 3.812811 | 0.000137 | 0.002775 | TRUE |
| GNRH1 | 13260 | 3.809758 | 0.000139 | 0.002802 | TRUE |
| CNTN5 | 9935 | 3.808646 | 0.00014 | 0.002802 | TRUE |
| KIF9-AS1 | 12395 | 3.808634 | 0.00014 | 0.002802 | TRUE |
| TPCN1 | 5170 | 3.807851 | 0.00014 | 0.002806 | TRUE |
| ZNF502 | 10308 | 3.806321 | 0.000141 | 0.00282 | TRUE |
| JMY | 9035 | 3.804018 | 0.000142 | 0.002832 | TRUE |
| TNIP1 | 9085 | 3.801923 | 0.000144 | 0.002852 | TRUE |
| OR7E91P | 374 | 3.801574 | 0.000144 | 0.002852 | TRUE |
| MKNK2 | 4976 | 3.800052 | 0.000145 | 0.002866 | TRUE |
| STAT4 | 9576 | 3.794905 | 0.000148 | 0.002908 | TRUE |
| RCCD1 | 7171 | 3.790553 | 0.00015 | 0.002934 | TRUE |
| MLLT10 | 9263 | 3.788971 | 0.000151 | 0.002949 | TRUE |
| CCDC126 | 1398 | 3.782755 | 0.000155 | 0.003011 | TRUE |
| PRC1 | 5006 | 3.782627 | 0.000155 | 0.003011 | TRUE |
| CAPN15 | 6351 | 3.779734 | 0.000157 | 0.003032 | TRUE |
| ADCY9 | 11845 | 3.779319 | 0.000157 | 0.003032 | TRUE |
| PRR16 | 1724 | 3.778969 | 0.000157 | 0.003032 | TRUE |
| ATG16L1 | 13173 | 3.778965 | 0.000157 | 0.003032 | TRUE |
| CMPK1 | 5404 | 3.778498 | 0.000158 | 0.003032 | TRUE |
| CLEC16A | 4483 | 3.776343 | 0.000159 | 0.003043 | TRUE |
| LRRC49 | 7991 | 3.776212 | 0.000159 | 0.003043 | TRUE |
| DPY19L2P4 | 15185 | 3.775565 | 0.00016 | 0.003044 | TRUE |
| WDR90 | 13152 | 3.775514 | 0.00016 | 0.003044 | TRUE |
| DENND1B | 8013 | 3.773765 | 0.000161 | 0.003058 | TRUE |
| COG2 | 10598 | 3.772241 | 0.000162 | 0.003069 | TRUE |
| FBXO21 | 6235 | 3.771554 | 0.000162 | 0.003074 | TRUE |
| FAM222B | 3737 | 3.77062 | 0.000163 | 0.003082 | TRUE |
| CAST | 10161 | 3.769305 | 0.000164 | 0.003095 | TRUE |
| BMS1P2 | 7414 | 3.764822 | 0.000167 | 0.003137 | TRUE |
| TWNK | 3859 | 3.764744 | 0.000167 | 0.003137 | TRUE |
| XPA | 13826 | 3.74986 | 0.000177 | 0.003292 | TRUE |
| CCDC138 | 7533 | 3.746349 | 0.000179 | 0.003327 | TRUE |
| CTIF | 13769 | 3.745717 | 0.00018 | 0.003332 | TRUE |
| DPY19L2P1 | 11649 | 3.744723 | 0.000181 | 0.003337 | TRUE |
| TTC39A | 6138 | 3.744445 | 0.000181 | 0.003337 | TRUE |
| TAS2R43 | 175 | 3.737915 | 0.000186 | 0.003402 | TRUE |
| PIGL | 6410 | 3.736025 | 0.000187 | 0.003422 | TRUE |
| ERO1B | 1379 | 3.731847 | 0.00019 | 0.003455 | TRUE |
| ZNF562 | 13206 | 3.731536 | 0.00019 | 0.003456 | TRUE |
| ST8SIA1 | 2770 | 3.72891 | 0.000192 | 0.003482 | TRUE |
| ZRANB1 | 10333 | 3.727819 | 0.000193 | 0.003491 | TRUE |
| XPO4 | 5559 | 3.727308 | 0.000194 | 0.003494 | TRUE |
| TBC1D30 | 7000 | 3.726096 | 0.000194 | 0.003504 | TRUE |
| MDN1 | 14324 | 3.714569 | 0.000204 | 0.003636 | TRUE |
| CNST | 9369 | 3.714432 | 0.000204 | 0.003636 | TRUE |
| PRKCB | 4415 | 3.714303 | 0.000204 | 0.003636 | TRUE |
| PECR | 1004 | 3.71095 | 0.000206 | 0.003668 | TRUE |
| NCOR2 | 8549 | 3.710006 | 0.000207 | 0.00367 | TRUE |
| DNMT3A | 10998 | 3.70979 | 0.000207 | 0.00367 | TRUE |
| GFRA2 | 14297 | 3.707604 | 0.000209 | 0.003688 | TRUE |
| RORA | 6744 | 3.700183 | 0.000215 | 0.003772 | TRUE |
| VPS39 | 4351 | 3.698421 | 0.000217 | 0.003794 | TRUE |
| TXLNG | 2321 | 3.697398 | 0.000218 | 0.003805 | TRUE |
| DLG5 | 1699 | 3.697038 | 0.000218 | 0.003806 | TRUE |
| PLAGL1 | 4474 | 3.695456 | 0.000219 | 0.003817 | TRUE |
| SNHG14 | 11767 | 3.690502 | 0.000224 | 0.00387 | TRUE |
| LYPD5 | 5965 | 3.688591 | 0.000225 | 0.003882 | TRUE |
| LAT | 8730 | 3.686069 | 0.000228 | 0.003913 | TRUE |
| IQUB | 4607 | 3.686001 | 0.000228 | 0.003913 | TRUE |
| SLC16A7 | 72 | 3.68564 | 0.000228 | 0.003915 | TRUE |
| TIAM1 | 13419 | 3.683065 | 0.00023 | 0.003941 | TRUE |
| ZIM2 | 10974 | 3.682148 | 0.000231 | 0.003947 | TRUE |
| PLCB1 | 15428 | 3.680089 | 0.000233 | 0.003973 | TRUE |
| LINC01637 | 2262 | 3.679827 | 0.000233 | 0.003973 | TRUE |
| HERC3 | 6919 | 3.674414 | 0.000238 | 0.004042 | TRUE |
| CEP152 | 6735 | 3.671946 | 0.000241 | 0.004073 | TRUE |
| KRTCAP3 | 430 | 3.670954 | 0.000242 | 0.004084 | TRUE |
| GDPD1 | 3571 | 3.670564 | 0.000242 | 0.004086 | TRUE |
| CCNL1 | 10255 | 3.666951 | 0.000245 | 0.004113 | TRUE |
| NEB | 6796 | 3.664998 | 0.000247 | 0.00414 | TRUE |
| RNF144A | 11701 | 3.662517 | 0.00025 | 0.004154 | TRUE |
| ITGB1 | 1438 | 3.659561 | 0.000253 | 0.004187 | TRUE |
| LRRC37A4P | 2660 | 3.659438 | 0.000253 | 0.004187 | TRUE |
| OXSR1 | 6560 | 3.659351 | 0.000253 | 0.004187 | TRUE |
| MRPL33 | 6753 | 3.657049 | 0.000255 | 0.004212 | TRUE |
| FAM216A | 11439 | 3.655781 | 0.000256 | 0.004224 | TRUE |
| OSBPL6 | 12846 | 3.653817 | 0.000258 | 0.004246 | TRUE |
| VCPKMT | 9816 | 3.653007 | 0.000259 | 0.004252 | TRUE |
| ARID2 | 5539 | 3.645956 | 0.000266 | 0.004324 | TRUE |
| CDH12 | 12549 | 3.645186 | 0.000267 | 0.004324 | TRUE |
| VN1R1 | 4524 | 3.64494 | 0.000267 | 0.004324 | TRUE |
| CARD9 | 3939 | 3.644578 | 0.000268 | 0.004325 | TRUE |
| DUBR | 8859 | 3.64242 | 0.00027 | 0.004353 | TRUE |
| ACYP2 | 8742 | 3.641326 | 0.000271 | 0.004362 | TRUE |
| RFPL2 | 3265 | 3.639084 | 0.000274 | 0.004392 | TRUE |
| P2RX6 | 9346 | 3.637171 | 0.000276 | 0.004406 | TRUE |
| PDP2 | 12007 | 3.634658 | 0.000278 | 0.00444 | TRUE |
| COL27A1 | 8573 | 3.625879 | 0.000288 | 0.004566 | TRUE |
| ZBED6 | 9597 | 3.624783 | 0.000289 | 0.004575 | TRUE |
| TAF1C | 6224 | 3.624571 | 0.000289 | 0.004575 | TRUE |
| BCO2 | 1016 | 3.622999 | 0.000291 | 0.004598 | TRUE |
| NCOA3 | 4372 | 3.62148 | 0.000293 | 0.004621 | TRUE |
| ACSL6 | 11549 | 3.620773 | 0.000294 | 0.004624 | TRUE |
| TAS2R5 | 10147 | 3.619915 | 0.000295 | 0.00463 | TRUE |
| TFAM | 10855 | 3.619413 | 0.000295 | 0.00463 | TRUE |
| FAM78B | 6564 | 3.616468 | 0.000299 | 0.004669 | TRUE |
| ATP2B3 | 11271 | 3.614718 | 0.000301 | 0.004696 | TRUE |
| ZNF557 | 9519 | 3.613815 | 0.000302 | 0.004704 | TRUE |
| MCF2L | 2276 | 3.611824 | 0.000304 | 0.00472 | TRUE |
| IFIT5 | 6594 | 3.610581 | 0.000306 | 0.004738 | TRUE |
| SORBS3 | 13919 | 3.610198 | 0.000306 | 0.00474 | TRUE |
| UBAP1L | 6346 | 3.608879 | 0.000308 | 0.00476 | TRUE |
| LOC652276 | 1237 | 3.597771 | 0.000321 | 0.004914 | TRUE |
| HSPB6 | 6104 | 3.59602 | 0.000323 | 0.004938 | TRUE |
| GLCCI1 | 13145 | 3.595269 | 0.000324 | 0.004942 | TRUE |
| IL1RAP | 12822 | 3.594264 | 0.000325 | 0.004957 | TRUE |
| USP28 | 14487 | 3.591845 | 0.000328 | 0.004993 | TRUE |
| FBXO45 | 5967 | 3.591059 | 0.000329 | 0.005003 | TRUE |
| CORO6 | 12385 | 3.589762 | 0.000331 | 0.005013 | TRUE |
| SIKE1 | 4700 | 3.589554 | 0.000331 | 0.005013 | TRUE |
| CEP97 | 1493 | 3.589272 | 0.000332 | 0.005013 | TRUE |
| SNRNP35 | 2619 | 3.588873 | 0.000332 | 0.005016 | TRUE |
| UBQLN4 | 2292 | 3.587728 | 0.000334 | 0.005034 | TRUE |
| PRKCA | 14519 | 3.584491 | 0.000338 | 0.005087 | TRUE |
| ZADH2 | 9256 | 3.580478 | 0.000343 | 0.005127 | TRUE |
| GOT2 | 14852 | 3.580336 | 0.000343 | 0.005127 | TRUE |
| GPR158 | 724 | 3.580228 | 0.000343 | 0.005127 | TRUE |
| SLCO4A1 | 6146 | 3.580148 | 0.000343 | 0.005127 | TRUE |
| VSTM2A | 2598 | 3.579946 | 0.000344 | 0.005127 | TRUE |
| STARD5 | 3642 | 3.579937 | 0.000344 | 0.005127 | TRUE |
| MC1R | 15250 | 3.577745 | 0.000347 | 0.005165 | TRUE |
| PDE4A | 3601 | 3.576512 | 0.000348 | 0.005174 | TRUE |
| ESRRG | 12491 | 3.559967 | 0.000371 | 0.005444 | TRUE |
| CDR2L | 14464 | 3.558809 | 0.000373 | 0.005455 | TRUE |
| DTWD2 | 13337 | 3.55566 | 0.000377 | 0.005488 | TRUE |
| REPIN1 | 6089 | 3.554694 | 0.000378 | 0.005503 | TRUE |
| JAKMIP3 | 12096 | 3.552932 | 0.000381 | 0.005535 | TRUE |
| TOGARAM1 | 9092 | 3.549944 | 0.000385 | 0.005583 | TRUE |
| ELMOD3 | 13688 | 3.549272 | 0.000386 | 0.005586 | TRUE |
| CROCCP2 | 14943 | 3.549073 | 0.000387 | 0.005586 | TRUE |
| SMCO4 | 6488 | 3.542925 | 0.000396 | 0.005696 | TRUE |
| HOMEZ | 4554 | 3.536925 | 0.000405 | 0.005811 | TRUE |
| TCF7L2 | 4379 | 3.535673 | 0.000407 | 0.005834 | TRUE |
| SS18L1 | 5530 | 3.532089 | 0.000412 | 0.005897 | TRUE |
| ATRNL1 | 6911 | 3.531708 | 0.000413 | 0.005898 | TRUE |
| JDP2 | 5040 | 3.531264 | 0.000414 | 0.005898 | TRUE |
| SPIDR | 14473 | 3.531138 | 0.000414 | 0.005898 | TRUE |
| SLC25A41 | 2455 | 3.530453 | 0.000415 | 0.005898 | TRUE |
| GFPT2 | 10799 | 3.529813 | 0.000416 | 0.005903 | TRUE |
| TMEM255B | 7052 | 3.529639 | 0.000416 | 0.005903 | TRUE |
| YARS | 6456 | 3.528431 | 0.000418 | 0.005925 | TRUE |
| PATE2 | 14136 | 3.525445 | 0.000423 | 0.00596 | TRUE |
| PLXDC1 | 9420 | 3.523881 | 0.000425 | 0.005989 | TRUE |
| KREMEN1 | 6039 | 3.519825 | 0.000432 | 0.006071 | TRUE |
| KCNK10 | 3303 | 3.519278 | 0.000433 | 0.006077 | TRUE |
| UBA3 | 8940 | 3.516653 | 0.000437 | 0.006127 | TRUE |
| SCRT1 | 14569 | 3.516221 | 0.000438 | 0.006132 | TRUE |
| GNAS | 9195 | 3.514668 | 0.00044 | 0.006162 | TRUE |
| LOC100131289 | 15571 | 3.51224 | 0.000444 | 0.006193 | TRUE |
| SUZ12 | 13195 | 3.511224 | 0.000446 | 0.006198 | TRUE |
| DPP6 | 13914 | 3.509555 | 0.000449 | 0.006226 | TRUE |
| PCSK1 | 12850 | 3.50857 | 0.000451 | 0.006244 | TRUE |
| ZNF365 | 7721 | 3.507551 | 0.000452 | 0.006254 | TRUE |
| ELL | 1014 | 3.506286 | 0.000454 | 0.00627 | TRUE |
| LXN | 15079 | 3.50306 | 0.00046 | 0.006335 | TRUE |
| MINDY3 | 9956 | 3.501674 | 0.000462 | 0.006361 | TRUE |
| FBN1 | 14825 | 3.500041 | 0.000465 | 0.00639 | TRUE |
| RASD2 | 2416 | 3.499495 | 0.000466 | 0.006398 | TRUE |
| SH3BGRL | 8713 | 3.496172 | 0.000472 | 0.006455 | TRUE |
| WFDC2 | 1233 | 3.494442 | 0.000475 | 0.006492 | TRUE |
| QRFPR | 4550 | 3.491404 | 0.00048 | 0.00656 | TRUE |
| TNFRSF25 | 15485 | 3.488197 | 0.000486 | 0.006602 | TRUE |
| CHRD | 10914 | 3.488078 | 0.000487 | 0.006602 | TRUE |
| CLGN | 3892 | 3.486477 | 0.000489 | 0.006636 | TRUE |
| KLHL21 | 5515 | 3.484672 | 0.000493 | 0.006671 | TRUE |
| KCNC3 | 7511 | 3.484595 | 0.000493 | 0.006671 | TRUE |
| PELI3 | 6447 | 3.480676 | 0.0005 | 0.006746 | TRUE |
| ACAN | 6412 | 3.480461 | 0.000501 | 0.006746 | TRUE |
| FAM78A | 10798 | 3.473039 | 0.000515 | 0.006894 | TRUE |
| CEP85L | 5344 | 3.472514 | 0.000516 | 0.006901 | TRUE |
| UBFD1 | 5886 | 3.471674 | 0.000517 | 0.006917 | TRUE |
| CAMTA2 | 4799 | 3.470973 | 0.000519 | 0.006926 | TRUE |
| STS | 11892 | 3.469863 | 0.000521 | 0.00694 | TRUE |
| SGK494 | 7402 | 3.46945 | 0.000522 | 0.006942 | TRUE |
| CREBZF | 4143 | 3.46897 | 0.000522 | 0.006945 | TRUE |
| SCAF11 | 14919 | 3.467066 | 0.000526 | 0.006975 | TRUE |
| POU3F2 | 11738 | 3.466965 | 0.000526 | 0.006975 | TRUE |
| NOXO1 | 3196 | 3.466906 | 0.000526 | 0.006975 | TRUE |
| SATB1 | 5514 | 3.465227 | 0.00053 | 0.007001 | TRUE |
| YBEY | 6788 | 3.464833 | 0.000531 | 0.007005 | TRUE |
| CACNA2D2 | 3465 | 3.462902 | 0.000534 | 0.007034 | TRUE |
| PDE7A | 8866 | 3.462038 | 0.000536 | 0.007049 | TRUE |
| SOGA3 | 13366 | 3.460068 | 0.00054 | 0.007089 | TRUE |
| CAB39 | 12183 | 3.45662 | 0.000547 | 0.007162 | TRUE |
| TMEM38A | 12211 | 3.451768 | 0.000557 | 0.007267 | TRUE |
| TSTD2 | 15217 | 3.45017 | 0.00056 | 0.007305 | TRUE |
| SCN4B | 10303 | 3.449752 | 0.000561 | 0.00731 | TRUE |
| APOL2 | 5429 | 3.446644 | 0.000568 | 0.007364 | TRUE |
| LOC441455 | 7252 | 3.44597 | 0.000569 | 0.007376 | TRUE |
| SRARP | 6928 | 3.445684 | 0.00057 | 0.007378 | TRUE |
| INSM2 | 12275 | 3.443346 | 0.000575 | 0.007429 | TRUE |
| EYA3 | 8036 | 3.442991 | 0.000575 | 0.007433 | TRUE |
| OGDHL | 13215 | 3.441075 | 0.000579 | 0.00748 | TRUE |
| SLITRK3 | 7930 | 3.440837 | 0.00058 | 0.00748 | TRUE |
| AP5M1 | 3486 | 3.439856 | 0.000582 | 0.007486 | TRUE |
| TMCC2 | 64 | 3.439718 | 0.000582 | 0.007486 | TRUE |
| LINC00641 | 1096 | 3.43779 | 0.000586 | 0.007528 | TRUE |
| ORAOV1 | 2313 | 3.434046 | 0.000595 | 0.007613 | TRUE |
| MBNL2 | 5565 | 3.433746 | 0.000595 | 0.007616 | TRUE |
| ZNF573 | 1087 | 3.43146 | 0.0006 | 0.007649 | TRUE |
| PRAG1 | 9642 | 3.430711 | 0.000602 | 0.007658 | TRUE |
| TCHH | 8963 | 3.429862 | 0.000604 | 0.007675 | TRUE |
| SUMF2 | 2197 | 3.429385 | 0.000605 | 0.007683 | TRUE |
| CHD1 | 9105 | 3.427687 | 0.000609 | 0.007724 | TRUE |
| UPF2 | 7347 | 3.426648 | 0.000611 | 0.007743 | TRUE |
| EMX1 | 2238 | 3.426604 | 0.000611 | 0.007743 | TRUE |
| SERPINB9 | 3746 | 3.426254 | 0.000612 | 0.007746 | TRUE |
| PPP6R3 | 15530 | 3.423246 | 0.000619 | 0.00782 | TRUE |
| TMPO | 11221 | 3.422742 | 0.00062 | 0.007828 | TRUE |
| LOC100294145 | 3255 | 3.418225 | 0.00063 | 0.007934 | TRUE |
| GALR1 | 14004 | 3.417938 | 0.000631 | 0.007936 | TRUE |
| ZNF771 | 1993 | 3.416415 | 0.000635 | 0.007974 | TRUE |
| MGARP | 15224 | 3.415242 | 0.000637 | 0.007995 | TRUE |
| ARHGEF12 | 9908 | 3.413555 | 0.000641 | 0.008032 | TRUE |
| RBL2 | 6655 | 3.413172 | 0.000642 | 0.008037 | TRUE |
| RMI1 | 10782 | 3.411845 | 0.000645 | 0.008063 | TRUE |
| KBTBD12 | 13273 | 3.409873 | 0.00065 | 0.008093 | TRUE |
| ATXN3 | 4311 | 3.40698 | 0.000657 | 0.008143 | TRUE |
| GOLGA8A | 10068 | 3.405351 | 0.000661 | 0.00816 | TRUE |
| TNF | 2873 | 3.403765 | 0.000665 | 0.008181 | TRUE |
| MTBP | 2038 | 3.402128 | 0.000669 | 0.008224 | TRUE |
| CASP8AP2 | 435 | 3.401591 | 0.00067 | 0.008234 | TRUE |
| TCTEX1D1 | 10225 | 3.400193 | 0.000673 | 0.008256 | TRUE |
| DOT1L | 1018 | 3.399975 | 0.000674 | 0.008257 | TRUE |
| TRIM58 | 8018 | 3.39577 | 0.000684 | 0.008365 | TRUE |
| LOC100240734 | 12119 | 3.394371 | 0.000688 | 0.008401 | TRUE |
| PLEKHM2 | 7748 | 3.393692 | 0.00069 | 0.008408 | TRUE |
| CHST12 | 7192 | 3.392165 | 0.000693 | 0.00843 | TRUE |
| TCERG1 | 6269 | 3.389028 | 0.000701 | 0.0085 | TRUE |
| LOC100287497 | 15523 | 3.387298 | 0.000706 | 0.008541 | TRUE |
| PCDHGB4 | 1376 | 3.385714 | 0.00071 | 0.008577 | TRUE |
| H6PD | 14039 | 3.384964 | 0.000712 | 0.008594 | TRUE |
| RGPD3 | 11085 | 3.384103 | 0.000714 | 0.008607 | TRUE |
| TMEM229B | 12999 | 3.383265 | 0.000716 | 0.008627 | TRUE |
| MIRLET7BHG | 14626 | 3.375706 | 0.000736 | 0.008793 | TRUE |
| DCBLD2 | 15056 | 3.375363 | 0.000737 | 0.008794 | TRUE |
| MAP9 | 6947 | 3.374577 | 0.000739 | 0.008809 | TRUE |
| RBMS1 | 12154 | 3.367409 | 0.000759 | 0.008993 | TRUE |
| RSAD2 | 5742 | 3.366526 | 0.000761 | 0.009002 | TRUE |
| TNKS | 7137 | 3.365995 | 0.000763 | 0.009012 | TRUE |
| LRRC4 | 8771 | 3.365288 | 0.000765 | 0.009021 | TRUE |
| PPFIA4 | 14145 | 3.362115 | 0.000773 | 0.009092 | TRUE |
| BACH2 | 6113 | 3.361874 | 0.000774 | 0.009093 | TRUE |
| C9orf72 | 5049 | 3.358685 | 0.000783 | 0.009164 | TRUE |
| GALE | 13571 | 3.358417 | 0.000784 | 0.009166 | TRUE |
| MTPAP | 6503 | 3.354224 | 0.000796 | 0.009257 | TRUE |
| EXOC3 | 9258 | 3.35002 | 0.000808 | 0.009337 | TRUE |
| TXLNGY | 10193 | 3.349986 | 0.000808 | 0.009337 | TRUE |
| FAM217B | 15595 | 3.349155 | 0.000811 | 0.009352 | TRUE |
| DSCC1 | 611 | 3.34741 | 0.000816 | 0.009397 | TRUE |
| C3orf58 | 1697 | 3.346253 | 0.000819 | 0.009429 | TRUE |
| PLEKHM1 | 5938 | 3.34581 | 0.00082 | 0.009435 | TRUE |
| PIN4P1 | 7622 | 3.344357 | 0.000825 | 0.00945 | TRUE |
| STARD10 | 1088 | 3.34401 | 0.000826 | 0.00945 | TRUE |
| RERGL | 3223 | 3.341904 | 0.000832 | 0.009495 | TRUE |
| AGO4 | 1744 | 3.340801 | 0.000835 | 0.009518 | TRUE |
| KRIT1 | 4475 | 3.337889 | 0.000844 | 0.009591 | TRUE |
| LOC729218 | 14351 | 3.333778 | 0.000857 | 0.009691 | TRUE |
| BRSK2 | 7988 | 3.333272 | 0.000858 | 0.009702 | TRUE |
| KIF27 | 3856 | 3.331963 | 0.000862 | 0.009727 | TRUE |
| FAM210B | 7722 | 3.327833 | 0.000875 | 0.009818 | TRUE |
| WDR33 | 4637 | 3.327631 | 0.000876 | 0.009818 | TRUE |
| FLJ42627 | 3237 | 3.327587 | 0.000876 | 0.009818 | TRUE |
| AK2 | 13902 | 3.325969 | 0.000881 | 0.009828 | TRUE |
| SNAP25 | 4606 | 3.325881 | 0.000881 | 0.009828 | TRUE |
| PI4K2A | 4738 | 3.325686 | 0.000882 | 0.009828 | TRUE |
| RELL2 | 4237 | 3.323164 | 0.00089 | 0.009896 | TRUE |
| THOC1 | 14344 | 3.322523 | 0.000892 | 0.009911 | TRUE |
| DDX19B | 2387 | 3.322349 | 0.000893 | 0.009911 | TRUE |
| LY6G5B | 2642 | 3.32213 | 0.000893 | 0.009912 | TRUE |
| RBFOX2 | 2703 | 3.320404 | 0.000899 | 0.009945 | TRUE |
| HECW1 | 13056 | 3.320184 | 0.0009 | 0.009946 | TRUE |
| ZC3H12C | 3950 | 3.319121 | 0.000903 | 0.009977 | TRUE |
| GK | 14571 | 3.318198 | 0.000906 | 0.010002 | TRUE |
| SPAG16 | 3997 | 3.313725 | 0.000921 | 0.010121 | TRUE |
| SMARCC1 | 12705 | 3.31243 | 0.000925 | 0.010139 | TRUE |
| SOAT1 | 7315 | 3.311619 | 0.000928 | 0.010162 | TRUE |
| TMEM204 | 1317 | 3.310559 | 0.000931 | 0.010186 | TRUE |
| FAM3C | 13203 | 3.308942 | 0.000936 | 0.010215 | TRUE |
| GSDMB | 1366 | 3.304705 | 0.000951 | 0.010341 | TRUE |
| ZMYM2 | 12821 | 3.302875 | 0.000957 | 0.010389 | TRUE |
| MIR9-3HG | 4317 | 3.301568 | 0.000961 | 0.010431 | TRUE |
| CCDC136 | 4086 | 3.301032 | 0.000963 | 0.010443 | TRUE |
| SSBP3 | 10735 | 3.299617 | 0.000968 | 0.010469 | TRUE |
| SEMA5B | 2309 | 3.299554 | 0.000968 | 0.010469 | TRUE |
| NDUFAF7 | 11591 | 3.299055 | 0.00097 | 0.010481 | TRUE |
| ARHGEF18 | 11102 | 3.29863 | 0.000972 | 0.010489 | TRUE |
| IQCE | 7526 | 3.294861 | 0.000985 | 0.010599 | TRUE |
| PLEKHA6 | 574 | 3.294754 | 0.000985 | 0.010599 | TRUE |
| KLHDC1 | 13409 | 3.293098 | 0.000991 | 0.010638 | TRUE |
| TRIP4 | 14353 | 3.292939 | 0.000991 | 0.010638 | TRUE |
| SV2C | 12912 | 3.292038 | 0.000995 | 0.010657 | TRUE |
| ZSCAN9 | 1155 | 3.290227 | 0.001001 | 0.010711 | TRUE |
| NRF1 | 223 | 3.289156 | 0.001005 | 0.010723 | TRUE |
| CLEC4M | 13816 | 3.28718 | 0.001012 | 0.010756 | TRUE |
| TMEM39A | 5148 | 3.287145 | 0.001012 | 0.010756 | TRUE |
| MYOZ3 | 9551 | 3.285063 | 0.00102 | 0.010814 | TRUE |
| NBR1 | 15100 | 3.283716 | 0.001024 | 0.010851 | TRUE |
| REV1 | 10802 | 3.282527 | 0.001029 | 0.010875 | TRUE |
| CYB5R4 | 2991 | 3.281076 | 0.001034 | 0.010916 | TRUE |
| PAXIP1 | 6031 | 3.279347 | 0.00104 | 0.010953 | TRUE |
| B3GALNT2 | 9374 | 3.278089 | 0.001045 | 0.010971 | TRUE |
| BRWD3 | 1589 | 3.277502 | 0.001047 | 0.010981 | TRUE |
| LOC101928673 | 15433 | 3.276847 | 0.00105 | 0.010992 | TRUE |
| ATP1A4 | 4069 | 3.275178 | 0.001056 | 0.011049 | TRUE |
| CPNE9 | 8468 | 3.272794 | 0.001065 | 0.011136 | TRUE |
| PCNX2 | 7830 | 3.2725 | 0.001066 | 0.011138 | TRUE |
| ITGB3BP | 11625 | 3.271645 | 0.001069 | 0.011158 | TRUE |
| NUPL2 | 6927 | 3.271238 | 0.001071 | 0.011167 | TRUE |
| DNAJC25 | 12118 | 3.270279 | 0.001074 | 0.011196 | TRUE |
| LOC285074 | 1613 | 3.269857 | 0.001076 | 0.011196 | TRUE |
| CKMT1B | 10151 | 3.269747 | 0.001076 | 0.011196 | TRUE |
| SEMA7A | 8176 | 3.268852 | 0.00108 | 0.011197 | TRUE |
| MOGAT2 | 7077 | 3.268749 | 0.00108 | 0.011197 | TRUE |
| LOC100130950 | 3109 | 3.268501 | 0.001081 | 0.011197 | TRUE |
| STRBP | 11997 | 3.26843 | 0.001081 | 0.011197 | TRUE |
| LINC00473 | 14600 | 3.268334 | 0.001082 | 0.011197 | TRUE |
| FZD4 | 13307 | 3.266147 | 0.00109 | 0.01125 | TRUE |
| SFXN1 | 2554 | 3.265413 | 0.001093 | 0.011257 | TRUE |
| TMEM30A | 13155 | 3.265061 | 0.001094 | 0.011263 | TRUE |
| ZFYVE9 | 12368 | 3.257713 | 0.001123 | 0.011491 | TRUE |
| MIAT | 3040 | 3.256381 | 0.001128 | 0.01153 | TRUE |
| FBXL4 | 7355 | 3.256185 | 0.001129 | 0.01153 | TRUE |
| TTC21B | 5864 | 3.254266 | 0.001137 | 0.011586 | TRUE |
| RGPD4 | 4320 | 3.254001 | 0.001138 | 0.011589 | TRUE |
| KDM7A | 6641 | 3.250777 | 0.001151 | 0.011676 | TRUE |
| E2F4 | 14430 | 3.249189 | 0.001157 | 0.011718 | TRUE |
| ABTB2 | 15261 | 3.248848 | 0.001159 | 0.011725 | TRUE |
| SLC9A1 | 12836 | 3.248459 | 0.00116 | 0.011733 | TRUE |
| GPR180 | 9738 | 3.248085 | 0.001162 | 0.011741 | TRUE |
| LRRFIP1 | 5905 | 3.246484 | 0.001168 | 0.011792 | TRUE |
| BTBD8 | 12868 | 3.242676 | 0.001184 | 0.01192 | TRUE |
| CAAP1 | 6395 | 3.242449 | 0.001185 | 0.011922 | TRUE |
| ZNF786 | 3340 | 3.240378 | 0.001194 | 0.011985 | TRUE |
| INTS8 | 259 | 3.240198 | 0.001194 | 0.011985 | TRUE |
| NAA50 | 7633 | 3.238271 | 0.001203 | 0.012059 | TRUE |
| GSE1 | 6167 | 3.237886 | 0.001204 | 0.012067 | TRUE |
| NUP205 | 6087 | 3.236727 | 0.001209 | 0.012109 | TRUE |
| POLR2C | 5822 | 3.235758 | 0.001213 | 0.012134 | TRUE |
| NCAPD2 | 6453 | 3.235245 | 0.001215 | 0.012147 | TRUE |
| CS | 152 | 3.233863 | 0.001221 | 0.012184 | TRUE |
| LMLN | 7381 | 3.230477 | 0.001236 | 0.012304 | TRUE |
| STAG1 | 13386 | 3.230461 | 0.001236 | 0.012304 | TRUE |
| NFIC | 7638 | 3.227361 | 0.001249 | 0.012411 | TRUE |
| WNT3 | 1622 | 3.227189 | 0.00125 | 0.012411 | TRUE |
| ANKS1A | 9424 | 3.226845 | 0.001252 | 0.012411 | TRUE |
| DGCR9 | 5952 | 3.224757 | 0.001261 | 0.012475 | TRUE |
| LINC00667 | 15618 | 3.224348 | 0.001263 | 0.012485 | TRUE |
| ANKRD29 | 14568 | 3.22143 | 0.001276 | 0.012574 | TRUE |
| ZNF718 | 10792 | 3.22139 | 0.001276 | 0.012574 | TRUE |
| KCNB1 | 13257 | 3.2208 | 0.001278 | 0.012577 | TRUE |
| ZNF670 | 12045 | 3.218252 | 0.00129 | 0.012665 | TRUE |
| BTBD11 | 2043 | 3.216867 | 0.001296 | 0.012677 | TRUE |
| CDC42BPA | 4676 | 3.216269 | 0.001299 | 0.012677 | TRUE |
| ATG2B | 3030 | 3.216242 | 0.001299 | 0.012677 | TRUE |
| ARID3B | 9480 | 3.21613 | 0.001299 | 0.012677 | TRUE |
| ZYX | 8623 | 3.215654 | 0.001301 | 0.012677 | TRUE |
| IGF1R | 8415 | 3.214039 | 0.001309 | 0.012724 | TRUE |
| KHDC4 | 13111 | 3.213718 | 0.00131 | 0.012725 | TRUE |
| RNF208 | 6451 | 3.21322 | 0.001313 | 0.012737 | TRUE |
| SLC16A6 | 12253 | 3.212163 | 0.001317 | 0.01276 | TRUE |
| SCN1A | 4337 | 3.211999 | 0.001318 | 0.01276 | TRUE |
| FAM153A | 5065 | 3.210197 | 0.001326 | 0.012816 | TRUE |
| PIKFYVE | 11159 | 3.209225 | 0.001331 | 0.012844 | TRUE |
| ELOVL4 | 188 | 3.207945 | 0.001337 | 0.012885 | TRUE |
| EGFR | 14901 | 3.20619 | 0.001345 | 0.012952 | TRUE |
| KDM5C | 7319 | 3.20609 | 0.001346 | 0.012952 | TRUE |
| MAST4 | 10280 | 3.204804 | 0.001352 | 0.013002 | TRUE |
| XIRP1 | 8848 | 3.203545 | 0.001357 | 0.013051 | TRUE |
| KCND3 | 270 | 3.202273 | 0.001363 | 0.013101 | TRUE |
| ZFYVE28 | 11053 | 3.202082 | 0.001364 | 0.013102 | TRUE |
| PDE12 | 4095 | 3.199557 | 0.001376 | 0.013184 | TRUE |
| ONECUT2 | 14711 | 3.19773 | 0.001385 | 0.013228 | TRUE |
| TBC1D8 | 9611 | 3.195937 | 0.001394 | 0.013276 | TRUE |
| COL5A1 | 1080 | 3.193547 | 0.001405 | 0.013331 | TRUE |
| PIM1 | 361 | 3.192641 | 0.00141 | 0.013342 | TRUE |
| LINC-PINT | 11054 | 3.192035 | 0.001413 | 0.013354 | TRUE |
| ZMAT3 | 5501 | 3.191571 | 0.001415 | 0.013354 | TRUE |
| HDAC4 | 15189 | 3.191093 | 0.001417 | 0.013364 | TRUE |
| XYLT2 | 12526 | 3.190259 | 0.001421 | 0.013395 | TRUE |
| ST3GAL6-AS1 | 1318 | 3.188029 | 0.001432 | 0.01347 | TRUE |
| FRMD5 | 8491 | 3.187945 | 0.001433 | 0.01347 | TRUE |
| TRIM26 | 14319 | 3.187542 | 0.001435 | 0.01348 | TRUE |
| BRWD1 | 14015 | 3.183793 | 0.001454 | 0.013616 | TRUE |
| C20orf196 | 2967 | 3.183774 | 0.001454 | 0.013616 | TRUE |
| KCNA2 | 11450 | 3.183188 | 0.001457 | 0.013636 | TRUE |
| XKR9 | 6892 | 3.182414 | 0.001461 | 0.013664 | TRUE |
| NKX3-1 | 12755 | 3.181539 | 0.001465 | 0.013683 | TRUE |
| SYN3 | 6333 | 3.181492 | 0.001465 | 0.013683 | TRUE |
| LINC00965 | 7437 | 3.180153 | 0.001472 | 0.013738 | TRUE |
| GAS2 | 12322 | 3.179522 | 0.001475 | 0.01376 | TRUE |
| PLAT | 2324 | 3.177056 | 0.001488 | 0.013838 | TRUE |
| CENPJ | 11579 | 3.176927 | 0.001488 | 0.013838 | TRUE |
| SORT1 | 9494 | 3.172909 | 0.001509 | 0.013977 | TRUE |
| MBL1P | 9777 | 3.172309 | 0.001512 | 0.013981 | TRUE |
| CEP44 | 11217 | 3.167933 | 0.001535 | 0.014135 | TRUE |
| ITPR1 | 13815 | 3.163232 | 0.00156 | 0.014323 | TRUE |
| SLC2A11 | 15312 | 3.16257 | 0.001564 | 0.014347 | TRUE |
| DCAF13P3 | 10628 | 3.157521 | 0.001591 | 0.014572 | TRUE |
| PALB2 | 604 | 3.15691 | 0.001595 | 0.014586 | TRUE |
| CACNA1C | 485 | 3.155915 | 0.0016 | 0.014627 | TRUE |
| THBS3 | 13692 | 3.155457 | 0.001602 | 0.014636 | TRUE |
| ARHGAP39 | 6576 | 3.152776 | 0.001617 | 0.014718 | TRUE |
| STOX2 | 11658 | 3.152743 | 0.001617 | 0.014718 | TRUE |
| GNB5 | 990 | 3.15001 | 0.001633 | 0.01483 | TRUE |
| TMEM178A | 14102 | 3.149563 | 0.001635 | 0.014845 | TRUE |
| ZBTB21 | 892 | 3.14196 | 0.001678 | 0.015183 | TRUE |
| PTX3 | 8728 | 3.139078 | 0.001695 | 0.015277 | TRUE |
| ZNF487 | 12084 | 3.13901 | 0.001695 | 0.015277 | TRUE |
| OSBP2 | 2647 | 3.138581 | 0.001698 | 0.015277 | TRUE |
| ARIH2 | 4639 | 3.137982 | 0.001701 | 0.015284 | TRUE |
| CPSF1 | 8560 | 3.134772 | 0.00172 | 0.015384 | TRUE |
| ZNF234 | 13726 | 3.134722 | 0.00172 | 0.015384 | TRUE |
| PDP1 | 9862 | 3.134064 | 0.001724 | 0.01541 | TRUE |
| ZC3H7B | 1482 | 3.132715 | 0.001732 | 0.015454 | TRUE |
| TPP2 | 7873 | 3.132514 | 0.001733 | 0.015456 | TRUE |
| MAGEL2 | 1533 | 3.129755 | 0.00175 | 0.015575 | TRUE |
| RNF19B | 14775 | 3.129204 | 0.001753 | 0.015587 | TRUE |
| FLYWCH1 | 6429 | 3.127856 | 0.001761 | 0.015632 | TRUE |
| METTL21A | 1705 | 3.127048 | 0.001766 | 0.015666 | TRUE |
| UIMC1 | 11121 | 3.124642 | 0.00178 | 0.015759 | TRUE |
| DNAJC28 | 11048 | 3.121546 | 0.001799 | 0.01589 | TRUE |
| PDPR | 7337 | 3.118477 | 0.001818 | 0.016029 | TRUE |
| ZNF385A | 3650 | 3.118262 | 0.001819 | 0.016031 | TRUE |
| CEP120 | 342 | 3.117227 | 0.001826 | 0.016079 | TRUE |
| HBP1 | 9338 | 3.114587 | 0.001842 | 0.016187 | TRUE |
| SZT2 | 8751 | 3.113767 | 0.001847 | 0.016214 | TRUE |
| RNF25 | 7237 | 3.109475 | 0.001874 | 0.016414 | TRUE |
| HES6 | 3250 | 3.108962 | 0.001877 | 0.016434 | TRUE |
| SEC31B | 7636 | 3.107756 | 0.001885 | 0.016491 | TRUE |
| HERC2P9 | 15411 | 3.10733 | 0.001888 | 0.016491 | TRUE |
| ZNF654 | 568 | 3.107266 | 0.001888 | 0.016491 | TRUE |
| TRAF3IP2-AS1 | 4705 | 3.106385 | 0.001894 | 0.016521 | TRUE |
| FIGN | 2990 | 3.10636 | 0.001894 | 0.016521 | TRUE |
| HUNK | 8903 | 3.106234 | 0.001895 | 0.016521 | TRUE |
| CNKSR2 | 15230 | 3.10283 | 0.001917 | 0.016687 | TRUE |
| DNA2 | 12859 | 3.102786 | 0.001917 | 0.016687 | TRUE |
| ZNF774 | 196 | 3.101925 | 0.001923 | 0.016717 | TRUE |
| MED13L | 7380 | 3.098039 | 0.001948 | 0.016882 | TRUE |
| RCC2 | 4448 | 3.098034 | 0.001948 | 0.016882 | TRUE |
| CACNB4 | 6479 | 3.092448 | 0.001985 | 0.017117 | TRUE |
| PRDM2 | 7993 | 3.091987 | 0.001988 | 0.017133 | TRUE |
| RAPGEF2 | 12242 | 3.091181 | 0.001994 | 0.017153 | TRUE |
| DCUN1D1 | 1855 | 3.089742 | 0.002003 | 0.017214 | TRUE |
| NCEH1 | 12469 | 3.089523 | 0.002005 | 0.017214 | TRUE |
| KCNC4 | 11203 | 3.087082 | 0.002021 | 0.017288 | TRUE |
| HPS5 | 977 | 3.08689 | 0.002023 | 0.017288 | TRUE |
| RSC1A1 | 8283 | 3.085035 | 0.002035 | 0.017371 | TRUE |
| FGF9 | 3909 | 3.085017 | 0.002035 | 0.017371 | TRUE |
| RGL2 | 7789 | 3.08497 | 0.002036 | 0.017371 | TRUE |
| ANKRD18CP | 2058 | 3.084715 | 0.002037 | 0.017377 | TRUE |
| TTBK2 | 9230 | 3.083395 | 0.002047 | 0.017397 | TRUE |
| TNRC6A | 9857 | 3.080771 | 0.002065 | 0.017523 | TRUE |
| MEIKIN | 10958 | 3.07839 | 0.002081 | 0.01765 | TRUE |
| LDB2 | 14046 | 3.076853 | 0.002092 | 0.017697 | TRUE |
| KDM5D | 107 | 3.0724 | 0.002123 | 0.017915 | TRUE |
| COL6A1 | 7866 | 3.070446 | 0.002137 | 0.018007 | TRUE |
| ELMO3 | 314 | 3.070067 | 0.00214 | 0.018007 | TRUE |
| ATP6AP1L | 1656 | 3.068594 | 0.002151 | 0.018081 | TRUE |
| POU6F1 | 3454 | 3.068063 | 0.002155 | 0.018094 | TRUE |
| EFR3A | 5157 | 3.067974 | 0.002155 | 0.018094 | TRUE |
| TESC | 5894 | 3.066736 | 0.002164 | 0.01813 | TRUE |
| PAK1 | 15429 | 3.066229 | 0.002168 | 0.018138 | TRUE |
| ETV6 | 4627 | 3.065877 | 0.00217 | 0.018138 | TRUE |
| RCAN2 | 11042 | 3.065822 | 0.002171 | 0.018138 | TRUE |
| ATAD1 | 14511 | 3.062248 | 0.002197 | 0.018297 | TRUE |
| CMYA5 | 8996 | 3.060727 | 0.002208 | 0.01836 | TRUE |
| FMR1 | 9890 | 3.059926 | 0.002214 | 0.01836 | TRUE |
| SEC14L5 | 393 | 3.057528 | 0.002232 | 0.018479 | TRUE |
| ZNF333 | 3466 | 3.056791 | 0.002237 | 0.018515 | TRUE |
| TBK1 | 12023 | 3.055241 | 0.002249 | 0.018581 | TRUE |
| EMSY | 900 | 3.054312 | 0.002256 | 0.018612 | TRUE |
| DLG1 | 3624 | 3.054271 | 0.002256 | 0.018612 | TRUE |
| GOLPH3 | 14238 | 3.051763 | 0.002275 | 0.018738 | TRUE |
| SCX | 13512 | 3.050052 | 0.002288 | 0.018809 | TRUE |
| PFKFB2 | 14692 | 3.049833 | 0.00229 | 0.01881 | TRUE |
| CPLX2 | 13896 | 3.048805 | 0.002298 | 0.018834 | TRUE |
| TMEM81 | 14730 | 3.04847 | 0.0023 | 0.018846 | TRUE |
| PTPDC1 | 565 | 3.0422 | 0.002349 | 0.019182 | TRUE |
| MYH7B | 13893 | 3.039371 | 0.002371 | 0.019323 | TRUE |
| RMDN2 | 2023 | 3.038008 | 0.002381 | 0.019377 | TRUE |
| KCTD20 | 1846 | 3.037744 | 0.002384 | 0.019377 | TRUE |
| BCL9 | 4325 | 3.036177 | 0.002396 | 0.01944 | TRUE |
| NUB1 | 12814 | 3.035054 | 0.002405 | 0.01947 | TRUE |
| TP53TG3D | 15543 | 3.034167 | 0.002412 | 0.019517 | TRUE |
| ERMP1 | 3920 | 3.033726 | 0.002416 | 0.019535 | TRUE |
| UBN2 | 3224 | 3.032646 | 0.002424 | 0.019595 | TRUE |
| CDK14 | 8234 | 3.032433 | 0.002426 | 0.019599 | TRUE |
| RAB11FIP2 | 6984 | 3.031939 | 0.00243 | 0.019611 | TRUE |
| ZMYM6 | 344 | 3.031219 | 0.002436 | 0.019617 | TRUE |
| NFKBIA | 9284 | 3.031168 | 0.002436 | 0.019617 | TRUE |
| TMEM35A | 13846 | 3.030914 | 0.002438 | 0.019617 | TRUE |
| TRIM7 | 4560 | 3.027923 | 0.002462 | 0.019773 | TRUE |
| ERICH5 | 14278 | 3.027891 | 0.002463 | 0.019773 | TRUE |
| TOB1-AS1 | 2514 | 3.025672 | 0.002481 | 0.019899 | TRUE |
| FKBP4 | 11646 | 3.02358 | 0.002498 | 0.020009 | TRUE |
| EPB41L5 | 13717 | 3.021744 | 0.002513 | 0.020097 | TRUE |
| SETDB1 | 6920 | 3.018154 | 0.002543 | 0.020285 | TRUE |
| ZNF106 | 329 | 3.016553 | 0.002557 | 0.020354 | TRUE |
| ZNF250 | 13761 | 3.016377 | 0.002558 | 0.020354 | TRUE |
| HCN1 | 5105 | 3.015964 | 0.002562 | 0.020359 | TRUE |
| RSAD1 | 4890 | 3.01242 | 0.002592 | 0.020556 | TRUE |
| NT5DC1 | 9286 | 3.012007 | 0.002595 | 0.020564 | TRUE |
| TAF4B | 2785 | 3.011367 | 0.002601 | 0.020592 | TRUE |
| AKT2 | 8622 | 3.011171 | 0.002602 | 0.020592 | TRUE |
| FOSB | 12027 | 3.010202 | 0.002611 | 0.020623 | TRUE |
| ENPP5 | 13741 | 3.009044 | 0.002621 | 0.020671 | TRUE |
| ZNF385B | 12837 | 3.007496 | 0.002634 | 0.020745 | TRUE |
| DIS3L2 | 6808 | 3.006428 | 0.002643 | 0.020797 | TRUE |
| GSAP | 12366 | 3.004444 | 0.002661 | 0.020912 | TRUE |
| LOC284009 | 5240 | 3.003877 | 0.002666 | 0.02092 | TRUE |
| MPP5 | 10676 | 3.003035 | 0.002673 | 0.020946 | TRUE |
| ACTR1B | 6739 | 2.998771 | 0.002711 | 0.021136 | TRUE |
| ARL2BP | 13942 | 2.998061 | 0.002717 | 0.021174 | TRUE |
| LOC728024 | 11367 | 2.997512 | 0.002722 | 0.021191 | TRUE |
| TRIM37 | 6361 | 2.996757 | 0.002729 | 0.021233 | TRUE |
| ANKFY1 | 14978 | 2.99412 | 0.002752 | 0.021325 | TRUE |
| PYROXD1 | 10123 | 2.993298 | 0.00276 | 0.021369 | TRUE |
| TAF13 | 14346 | 2.99231 | 0.002769 | 0.021423 | TRUE |
| INPP5F | 12360 | 2.991613 | 0.002775 | 0.021455 | TRUE |
| TRIM44 | 12808 | 2.991393 | 0.002777 | 0.02146 | TRUE |
| BCL11A | 7731 | 2.988176 | 0.002806 | 0.021655 | TRUE |
| VCX | 6340 | 2.986583 | 0.002821 | 0.021694 | TRUE |
| MTERF4 | 6855 | 2.98608 | 0.002826 | 0.021719 | TRUE |
| TRAF3IP1 | 15424 | 2.985593 | 0.00283 | 0.021732 | TRUE |
| THSD4 | 3184 | 2.982897 | 0.002855 | 0.021903 | TRUE |
| MIS18BP1 | 14696 | 2.981052 | 0.002873 | 0.021988 | TRUE |
| GLIS1 | 14897 | 2.980949 | 0.002874 | 0.021988 | TRUE |
| OLR1 | 15139 | 2.978918 | 0.002893 | 0.022102 | TRUE |
| BBS1 | 1208 | 2.9787 | 0.002895 | 0.022107 | TRUE |
| LOC727896 | 13605 | 2.978343 | 0.002898 | 0.022122 | TRUE |
| FDX1 | 11830 | 2.977828 | 0.002903 | 0.022149 | TRUE |
| LOC728743 | 9552 | 2.977575 | 0.002905 | 0.022155 | TRUE |
| PPP1R3D | 12637 | 2.977292 | 0.002908 | 0.022155 | TRUE |
| HCN4 | 5475 | 2.976466 | 0.002916 | 0.022204 | TRUE |
| ZNF37BP | 9595 | 2.975649 | 0.002924 | 0.022243 | TRUE |
| GPR137C | 14267 | 2.974216 | 0.002937 | 0.022332 | TRUE |
| SPECC1L | 12224 | 2.973566 | 0.002944 | 0.022349 | TRUE |
| CLIP4 | 2821 | 2.971856 | 0.00296 | 0.022442 | TRUE |
| RANBP6 | 4718 | 2.971699 | 0.002962 | 0.022442 | TRUE |
| PARD6B | 8105 | 2.969853 | 0.002979 | 0.022545 | TRUE |
| GLRX2 | 8079 | 2.968764 | 0.00299 | 0.022586 | TRUE |
| CDKL1 | 15180 | 2.968724 | 0.00299 | 0.022586 | TRUE |
| NEFM | 6454 | 2.968377 | 0.002994 | 0.022596 | TRUE |
| IPCEF1 | 12359 | 2.966694 | 0.00301 | 0.02269 | TRUE |
| TAS2R45 | 11713 | 2.963778 | 0.003039 | 0.022884 | TRUE |
| LRCH1 | 5032 | 2.958693 | 0.003089 | 0.023194 | TRUE |
| FKBP11 | 2806 | 2.9586 | 0.00309 | 0.023194 | TRUE |
| AKT3 | 14914 | 2.955694 | 0.00312 | 0.023368 | TRUE |
| BMS1P4 | 2711 | 2.954811 | 0.003129 | 0.023393 | TRUE |
| PAG1 | 12508 | 2.951689 | 0.00316 | 0.023538 | TRUE |
| IP6K2 | 891 | 2.949111 | 0.003187 | 0.023668 | TRUE |
| DAZAP1 | 4249 | 2.947827 | 0.0032 | 0.023755 | TRUE |
| METTL24 | 9421 | 2.947609 | 0.003202 | 0.023757 | TRUE |
| SLC35A4 | 4664 | 2.945655 | 0.003223 | 0.023855 | TRUE |
| FGF7P6 | 4002 | 2.943807 | 0.003242 | 0.023921 | TRUE |
| GFOD1 | 3001 | 2.943589 | 0.003244 | 0.023923 | TRUE |
| FAM133DP | 2239 | 2.942392 | 0.003257 | 0.023972 | TRUE |
| B3GNT9 | 11728 | 2.942234 | 0.003259 | 0.023972 | TRUE |
| ZBTB4 | 789 | 2.939128 | 0.003291 | 0.024145 | TRUE |
| MICU3 | 4536 | 2.937664 | 0.003307 | 0.024237 | TRUE |
| SLC25A40 | 12057 | 2.935612 | 0.003329 | 0.024364 | TRUE |
| UBLCP1 | 12922 | 2.935211 | 0.003333 | 0.02437 | TRUE |
| ESYT2 | 12531 | 2.935127 | 0.003334 | 0.02437 | TRUE |
| LRRC8C | 1566 | 2.935099 | 0.003334 | 0.02437 | TRUE |
| ABCB10 | 8473 | 2.934913 | 0.003336 | 0.024373 | TRUE |
| AP5B1 | 5034 | 2.934136 | 0.003345 | 0.024423 | TRUE |
| KDM2A | 510 | 2.933102 | 0.003356 | 0.02447 | TRUE |
| ABLIM2 | 13677 | 2.932699 | 0.00336 | 0.024485 | TRUE |
| ATG13 | 2521 | 2.93232 | 0.003364 | 0.024485 | TRUE |
| KITLG | 1216 | 2.931362 | 0.003375 | 0.024523 | TRUE |
| SLC6A10P | 2041 | 2.931099 | 0.003378 | 0.024523 | TRUE |
| USP16 | 15195 | 2.930766 | 0.003381 | 0.024529 | TRUE |
| KIF5B | 3434 | 2.927869 | 0.003413 | 0.024686 | TRUE |
| CYP2E1 | 7605 | 2.927754 | 0.003414 | 0.024686 | TRUE |
| INPP5A | 5342 | 2.926982 | 0.003423 | 0.024714 | TRUE |
| SETD6 | 7382 | 2.926051 | 0.003433 | 0.024773 | TRUE |
| PCNT | 9745 | 2.925872 | 0.003435 | 0.024773 | TRUE |
| C12orf49 | 9545 | 2.925817 | 0.003436 | 0.024773 | TRUE |
| AIFM3 | 11195 | 2.924915 | 0.003446 | 0.024799 | TRUE |
| NF2 | 10350 | 2.924588 | 0.003449 | 0.024814 | TRUE |
| TBCEL | 3525 | 2.922779 | 0.003469 | 0.024912 | TRUE |
| FAR2 | 12050 | 2.921704 | 0.003481 | 0.024976 | TRUE |
| SLC38A6 | 14905 | 2.920676 | 0.003493 | 0.025047 | TRUE |
| SFI1 | 14703 | 2.920127 | 0.003499 | 0.025064 | TRUE |
| ATAD2 | 7006 | 2.920023 | 0.0035 | 0.025064 | TRUE |
| TMEM120B | 6376 | 2.919195 | 0.003509 | 0.025108 | TRUE |
| ARMT1 | 5796 | 2.91815 | 0.003521 | 0.025181 | TRUE |
| MADCAM1 | 15315 | 2.917116 | 0.003533 | 0.02523 | TRUE |
| SLC35E1 | 8153 | 2.913992 | 0.003568 | 0.025449 | TRUE |
| EPHB6 | 2364 | 2.913825 | 0.00357 | 0.025451 | TRUE |
| RHOQ | 307 | 2.912551 | 0.003585 | 0.025523 | TRUE |
| LAPTM4B | 8106 | 2.910042 | 0.003614 | 0.025657 | TRUE |
| KPNA5 | 10445 | 2.910038 | 0.003614 | 0.025657 | TRUE |
| GTF2A1 | 6957 | 2.910029 | 0.003614 | 0.025657 | TRUE |
| COL19A1 | 6997 | 2.909233 | 0.003623 | 0.025667 | TRUE |
| VMP1 | 36 | 2.908386 | 0.003633 | 0.025711 | TRUE |
| FZD6 | 13028 | 2.907508 | 0.003643 | 0.02576 | TRUE |
| CCNI | 9075 | 2.907306 | 0.003646 | 0.025765 | TRUE |
| KNG1 | 14476 | 2.906577 | 0.003654 | 0.02579 | TRUE |
| MAP3K6 | 10158 | 2.904264 | 0.003681 | 0.025946 | TRUE |
| ZNF251 | 12188 | 2.903067 | 0.003695 | 0.02601 | TRUE |
| RICTOR | 9049 | 2.901531 | 0.003713 | 0.02608 | TRUE |
| CCSER1 | 3035 | 2.901525 | 0.003714 | 0.02608 | TRUE |
| RLF | 2626 | 2.899937 | 0.003732 | 0.026183 | TRUE |
| RASIP1 | 15259 | 2.896551 | 0.003773 | 0.02639 | TRUE |
| NCOA4 | 4076 | 2.896109 | 0.003778 | 0.026415 | TRUE |
| SEPHS2 | 10928 | 2.89363 | 0.003808 | 0.026597 | TRUE |
| DDHD1 | 12967 | 2.893535 | 0.003809 | 0.026597 | TRUE |
| PPARD | 857 | 2.892043 | 0.003827 | 0.026688 | TRUE |
| CTNNB1 | 3801 | 2.890991 | 0.00384 | 0.026754 | TRUE |
| MLLT3 | 8874 | 2.888834 | 0.003867 | 0.026902 | TRUE |
| ZNF25 | 10714 | 2.888607 | 0.00387 | 0.026909 | TRUE |
| SIRT4 | 2437 | 2.88634 | 0.003898 | 0.027056 | TRUE |
| MROH5 | 6239 | 2.882554 | 0.003945 | 0.027262 | TRUE |
| CEP192 | 13553 | 2.881077 | 0.003963 | 0.027378 | TRUE |
| RNF157 | 3296 | 2.880879 | 0.003966 | 0.027383 | TRUE |
| P3H3 | 13560 | 2.879215 | 0.003987 | 0.027481 | TRUE |
| CFAP69 | 7148 | 2.879197 | 0.003987 | 0.027481 | TRUE |
| UHRF1BP1L | 9316 | 2.877874 | 0.004004 | 0.027548 | TRUE |
| WASHC2C | 4552 | 2.877237 | 0.004012 | 0.027592 | TRUE |
| SNX27 | 1217 | 2.873524 | 0.004059 | 0.027839 | TRUE |
| PCDHB16 | 8298 | 2.873444 | 0.00406 | 0.027839 | TRUE |
| ZDHHC20 | 9615 | 2.873163 | 0.004064 | 0.02784 | TRUE |
| DDHD2 | 785 | 2.873058 | 0.004065 | 0.02784 | TRUE |
| PATL1 | 13993 | 2.873024 | 0.004066 | 0.02784 | TRUE |
| C21orf33 | 8574 | 2.872228 | 0.004076 | 0.027885 | TRUE |
| RBFOX3 | 12632 | 2.871873 | 0.00408 | 0.027902 | TRUE |
| CPLX1 | 9763 | 2.871763 | 0.004082 | 0.027902 | TRUE |
| ZNF491 | 6264 | 2.871212 | 0.004089 | 0.027939 | TRUE |
| STAT1 | 10168 | 2.869209 | 0.004115 | 0.028079 | TRUE |
| DENND6A | 9434 | 2.868815 | 0.00412 | 0.02809 | TRUE |
| PARP3 | 13059 | 2.86725 | 0.004141 | 0.028192 | TRUE |
| RSPO2 | 3411 | 2.865367 | 0.004165 | 0.028336 | TRUE |
| ALX3 | 15057 | 2.864286 | 0.00418 | 0.02842 | TRUE |
| CHCHD3 | 489 | 2.863928 | 0.004184 | 0.028421 | TRUE |
| YTHDF3 | 8716 | 2.86387 | 0.004185 | 0.028421 | TRUE |
| SUN1 | 15069 | 2.863644 | 0.004188 | 0.028423 | TRUE |
| SHROOM2 | 15212 | 2.861811 | 0.004212 | 0.028519 | TRUE |
| MSL2 | 4652 | 2.860499 | 0.00423 | 0.028625 | TRUE |
| NBPF3 | 10145 | 2.859583 | 0.004242 | 0.028672 | TRUE |
| NBPF1 | 13984 | 2.859414 | 0.004244 | 0.028672 | TRUE |
| PLOD2 | 14750 | 2.857346 | 0.004272 | 0.028774 | TRUE |
| NREP | 10306 | 2.855851 | 0.004292 | 0.028891 | TRUE |
| KIAA1841 | 10223 | 2.85547 | 0.004297 | 0.028895 | TRUE |
| LRPAP1 | 178 | 2.853284 | 0.004327 | 0.029044 | TRUE |
| SECISBP2 | 12207 | 2.853029 | 0.00433 | 0.029055 | TRUE |
| LINC00889 | 12904 | 2.852645 | 0.004336 | 0.029065 | TRUE |
| SAMD14 | 10240 | 2.851956 | 0.004345 | 0.029116 | TRUE |
| COX20 | 3675 | 2.851351 | 0.004353 | 0.029146 | TRUE |
| PPP2R5D | 14810 | 2.84925 | 0.004382 | 0.029274 | TRUE |
| RAB40AL | 13192 | 2.849144 | 0.004384 | 0.029274 | TRUE |
| CHD6 | 7787 | 2.847394 | 0.004408 | 0.02941 | TRUE |
| MTHFD2L | 14908 | 2.846771 | 0.004417 | 0.029432 | TRUE |
| SPINK2 | 1012 | 2.846496 | 0.00442 | 0.029443 | TRUE |
| PDE7B | 12294 | 2.846067 | 0.004426 | 0.029457 | TRUE |
| LRRFIP2 | 15030 | 2.845948 | 0.004428 | 0.029457 | TRUE |
| RPL13P5 | 4471 | 2.845706 | 0.004431 | 0.029466 | TRUE |
| ZNF8 | 2569 | 2.845103 | 0.00444 | 0.029509 | TRUE |
| FBXO32 | 2184 | 2.843693 | 0.004459 | 0.029615 | TRUE |
| ABHD3 | 6130 | 2.842936 | 0.00447 | 0.029673 | TRUE |
| EXOC2 | 4004 | 2.842678 | 0.004474 | 0.029684 | TRUE |
| LMTK2 | 4222 | 2.837175 | 0.004551 | 0.030124 | TRUE |
| KCNH6 | 14788 | 2.836728 | 0.004558 | 0.03015 | TRUE |
| SLC4A9 | 4100 | 2.836285 | 0.004564 | 0.03015 | TRUE |
| RRN3P3 | 6116 | 2.835906 | 0.00457 | 0.030167 | TRUE |
| ANKRD20A9P | 1313 | 2.835237 | 0.004579 | 0.030205 | TRUE |
| 6-Sep | 10894 | 2.834998 | 0.004583 | 0.030215 | TRUE |
| AEBP2 | 2584 | 2.832253 | 0.004622 | 0.030437 | TRUE |
| YY1AP1 | 12753 | 2.831489 | 0.004633 | 0.030484 | TRUE |
| TSHZ1 | 5007 | 2.831105 | 0.004639 | 0.030484 | TRUE |
| METTL16 | 12829 | 2.830519 | 0.004647 | 0.030513 | TRUE |
| TAF3 | 1683 | 2.827079 | 0.004697 | 0.030765 | TRUE |
| PPP4R1 | 14337 | 2.826056 | 0.004713 | 0.03085 | TRUE |
| LAMA3 | 4162 | 2.824676 | 0.004733 | 0.030958 | TRUE |
| SSH3 | 1962 | 2.82384 | 0.004745 | 0.031025 | TRUE |
| CAMKK1 | 8482 | 2.822619 | 0.004763 | 0.031079 | TRUE |
| ZNF853 | 7686 | 2.820811 | 0.00479 | 0.031228 | TRUE |
| KCNS1 | 2608 | 2.819953 | 0.004803 | 0.03126 | TRUE |
| ASAH2B | 9770 | 2.819006 | 0.004817 | 0.031339 | TRUE |
| CBX5 | 1321 | 2.817196 | 0.004844 | 0.031445 | TRUE |
| PPTC7 | 1852 | 2.817072 | 0.004846 | 0.031445 | TRUE |
| DLGAP1-AS4 | 7132 | 2.816158 | 0.00486 | 0.031494 | TRUE |
| ARID5B | 2198 | 2.81609 | 0.004861 | 0.031494 | TRUE |
| PTPN5 | 7116 | 2.81544 | 0.004871 | 0.031545 | TRUE |
| FAM49B | 13281 | 2.813051 | 0.004907 | 0.031767 | TRUE |
| AMPD3 | 4354 | 2.812535 | 0.004915 | 0.031805 | TRUE |
| GIGYF2 | 3570 | 2.812078 | 0.004922 | 0.031815 | TRUE |
| XRN2 | 9507 | 2.811765 | 0.004927 | 0.031815 | TRUE |
| STAM2 | 286 | 2.811761 | 0.004927 | 0.031815 | TRUE |
| LOC389765 | 9295 | 2.811631 | 0.004929 | 0.031815 | TRUE |
| DEPTOR | 8892 | 2.811135 | 0.004937 | 0.031851 | TRUE |
| LPCAT4 | 320 | 2.810936 | 0.00494 | 0.031852 | TRUE |
| C3orf38 | 2219 | 2.810857 | 0.004941 | 0.031852 | TRUE |
| PIK3CA | 15447 | 2.808614 | 0.004976 | 0.032022 | TRUE |
| USP42 | 9865 | 2.806143 | 0.005014 | 0.032236 | TRUE |
| PRR22 | 9090 | 2.806076 | 0.005015 | 0.032236 | TRUE |
| IMPA1 | 11796 | 2.80556 | 0.005023 | 0.032274 | TRUE |
| EHMT1 | 12740 | 2.803367 | 0.005057 | 0.032415 | TRUE |
| MYNN | 15534 | 2.802852 | 0.005065 | 0.03244 | TRUE |
| ING1 | 5005 | 2.801523 | 0.005086 | 0.032547 | TRUE |
| NUP155 | 12972 | 2.801335 | 0.005089 | 0.032553 | TRUE |
| ANKS3 | 2837 | 2.80048 | 0.005103 | 0.032594 | TRUE |
| MCF2L-AS1 | 6971 | 2.799925 | 0.005111 | 0.032629 | TRUE |
| AFTPH | 10284 | 2.799134 | 0.005124 | 0.032695 | TRUE |
| MASP2 | 4097 | 2.798841 | 0.005129 | 0.032712 | TRUE |
| ANKRD20A11P | 15536 | 2.7973 | 0.005153 | 0.032814 | TRUE |
| DPT | 1378 | 2.797162 | 0.005155 | 0.032815 | TRUE |
| DCUN1D2 | 5014 | 2.796288 | 0.005169 | 0.032877 | TRUE |
| STAG3L5P | 397 | 2.795507 | 0.005182 | 0.032943 | TRUE |
| FAM66B | 13 | 2.794219 | 0.005203 | 0.033033 | TRUE |
| ZFP14 | 2597 | 2.794138 | 0.005204 | 0.033033 | TRUE |
| CYTH3 | 14570 | 2.79375 | 0.00521 | 0.03304 | TRUE |
| SDSL | 11879 | 2.792741 | 0.005226 | 0.033115 | TRUE |
| SYNE1 | 3322 | 2.792645 | 0.005228 | 0.033115 | TRUE |
| LINC02210 | 12833 | 2.790972 | 0.005255 | 0.03326 | TRUE |
| ZNF638 | 6477 | 2.788868 | 0.005289 | 0.033449 | TRUE |
| UBE2O | 5799 | 2.786019 | 0.005336 | 0.03369 | TRUE |
| CDYL | 14502 | 2.783372 | 0.00538 | 0.033912 | TRUE |
| SYT15 | 15072 | 2.781908 | 0.005404 | 0.034014 | TRUE |
| SLFN5 | 2237 | 2.780305 | 0.005431 | 0.034082 | TRUE |
| SETD2 | 2870 | 2.780091 | 0.005434 | 0.034091 | TRUE |
| ARC | 6951 | 2.779083 | 0.005451 | 0.034174 | TRUE |
| CAMK1D | 810 | 2.779048 | 0.005452 | 0.034174 | TRUE |
| ZNF16 | 7950 | 2.778526 | 0.005461 | 0.034176 | TRUE |
| MSRB3 | 4729 | 2.778378 | 0.005463 | 0.034176 | TRUE |
| FBLIM1 | 14988 | 2.777662 | 0.005475 | 0.034237 | TRUE |
| NAP1L1 | 15148 | 2.775574 | 0.00551 | 0.03443 | TRUE |
| CA7 | 12512 | 2.774964 | 0.005521 | 0.034477 | TRUE |
| RIMKLA | 7184 | 2.774086 | 0.005536 | 0.034513 | TRUE |
| CLN6 | 13933 | 2.774065 | 0.005536 | 0.034513 | TRUE |
| JAM2 | 14601 | 2.774012 | 0.005537 | 0.034513 | TRUE |
| RNF168 | 12340 | 2.773466 | 0.005546 | 0.03454 | TRUE |
| EML6 | 2392 | 2.771566 | 0.005579 | 0.034691 | TRUE |
| MOK | 33 | 2.770383 | 0.005599 | 0.034789 | TRUE |
| SYMPK | 13818 | 2.769583 | 0.005613 | 0.034835 | TRUE |
| LUZP1 | 6555 | 2.769568 | 0.005613 | 0.034835 | TRUE |
| MAT2B | 3941 | 2.769158 | 0.00562 | 0.034851 | TRUE |
| TRPC3 | 1274 | 2.768389 | 0.005633 | 0.034897 | TRUE |
| CRISPLD1 | 6545 | 2.768343 | 0.005634 | 0.034897 | TRUE |
| DUSP4 | 2290 | 2.76725 | 0.005653 | 0.034976 | TRUE |
| CEP63 | 537 | 2.767169 | 0.005655 | 0.034976 | TRUE |
| ZNF586 | 12499 | 2.767086 | 0.005656 | 0.034976 | TRUE |
| LINC00938 | 7121 | 2.765415 | 0.005685 | 0.035087 | TRUE |
| CEP41 | 11014 | 2.764785 | 0.005696 | 0.035134 | TRUE |
| LRIG2 | 1133 | 2.764609 | 0.005699 | 0.035134 | TRUE |
| OSGEPL1 | 10241 | 2.764458 | 0.005702 | 0.035134 | TRUE |
| MROH8 | 14670 | 2.764422 | 0.005702 | 0.035134 | TRUE |
| ZBTB1 | 14140 | 2.764215 | 0.005706 | 0.035134 | TRUE |
| ZNF252P | 1968 | 2.764202 | 0.005706 | 0.035134 | TRUE |
| TPMT | 14976 | 2.763901 | 0.005711 | 0.035153 | TRUE |
| NEAT1 | 14372 | 2.759058 | 0.005797 | 0.03558 | TRUE |
| PWAR5 | 3506 | 2.758244 | 0.005811 | 0.035655 | TRUE |
| BRIX1 | 11547 | 2.757434 | 0.005826 | 0.035705 | TRUE |
| RIF1 | 14521 | 2.757395 | 0.005826 | 0.035705 | TRUE |
| NEK1 | 14953 | 2.756177 | 0.005848 | 0.035796 | TRUE |
| PTCHD4 | 6886 | 2.755794 | 0.005855 | 0.035824 | TRUE |
| ACADSB | 6639 | 2.752226 | 0.005919 | 0.036146 | TRUE |
| CCDC88A | 13099 | 2.750197 | 0.005956 | 0.036314 | TRUE |
| TARDBP | 7998 | 2.748557 | 0.005986 | 0.036454 | TRUE |
| RERE | 12997 | 2.748394 | 0.005989 | 0.036458 | TRUE |
| MOGAT1 | 5679 | 2.747781 | 0.006 | 0.036497 | TRUE |
| SMC2 | 12590 | 2.747225 | 0.00601 | 0.036545 | TRUE |
| TRIM9 | 14336 | 2.744629 | 0.006058 | 0.036821 | TRUE |
| VIRMA | 9398 | 2.740186 | 0.00614 | 0.037232 | TRUE |
| MRS2 | 3339 | 2.739387 | 0.006155 | 0.037287 | TRUE |
| TAS2R14 | 7142 | 2.739348 | 0.006156 | 0.037287 | TRUE |
| CLMN | 13091 | 2.738187 | 0.006178 | 0.037332 | TRUE |
| LGR6 | 13278 | 2.737125 | 0.006198 | 0.037424 | TRUE |
| POGLUT1 | 15040 | 2.735613 | 0.006226 | 0.037582 | TRUE |
| JAK1 | 5201 | 2.735162 | 0.006235 | 0.037598 | TRUE |
| TMX3 | 12037 | 2.735092 | 0.006236 | 0.037598 | TRUE |
| TRERF1 | 12265 | 2.731004 | 0.006314 | 0.038009 | TRUE |
| KLHL3 | 3403 | 2.729669 | 0.00634 | 0.03809 | TRUE |
| EMBP1 | 3665 | 2.729351 | 0.006346 | 0.038112 | TRUE |
| CACNG7 | 818 | 2.728353 | 0.006365 | 0.038154 | TRUE |
| PDXDC2P-NPIPB14P | 8251 | 2.725178 | 0.006427 | 0.038494 | TRUE |
| PFAS | 2232 | 2.724581 | 0.006438 | 0.038534 | TRUE |
| CNNM2 | 15127 | 2.721563 | 0.006497 | 0.038783 | TRUE |
| PENK | 11004 | 2.721272 | 0.006503 | 0.038788 | TRUE |
| DMKN | 1729 | 2.715529 | 0.006617 | 0.039347 | TRUE |
| TUBA8 | 12095 | 2.715043 | 0.006627 | 0.03939 | TRUE |
| STK36 | 8672 | 2.713495 | 0.006658 | 0.039559 | TRUE |
| TTC39C | 11967 | 2.712947 | 0.006669 | 0.039599 | TRUE |
| MYO5C | 10057 | 2.712784 | 0.006672 | 0.039599 | TRUE |
| PPP2CA | 13437 | 2.712239 | 0.006683 | 0.039637 | TRUE |
| RAPGEFL1 | 6234 | 2.711668 | 0.006695 | 0.039673 | TRUE |
| JARID2 | 15073 | 2.710561 | 0.006717 | 0.039767 | TRUE |
| FAM169A | 3867 | 2.708769 | 0.006753 | 0.03993 | TRUE |
| SRFBP1 | 14219 | 2.707951 | 0.00677 | 0.040004 | TRUE |
| DPP8 | 2640 | 2.705236 | 0.006826 | 0.040235 | TRUE |
| OR7E47P | 13546 | 2.704359 | 0.006844 | 0.040311 | TRUE |
| SIPA1L3 | 5985 | 2.702572 | 0.006881 | 0.040513 | TRUE |
| KLHL24 | 6835 | 2.700861 | 0.006916 | 0.040661 | TRUE |
| ZC3H18 | 1498 | 2.700494 | 0.006924 | 0.040691 | TRUE |
| ZNF587 | 6268 | 2.699404 | 0.006946 | 0.040789 | TRUE |
| ASPRV1 | 10160 | 2.698899 | 0.006957 | 0.040822 | TRUE |
| RIPK1 | 6366 | 2.698674 | 0.006962 | 0.040822 | TRUE |
| TADA2A | 12356 | 2.698529 | 0.006965 | 0.040824 | TRUE |
| MDM2 | 10129 | 2.697282 | 0.006991 | 0.040947 | TRUE |
| GGA2 | 14133 | 2.696546 | 0.007006 | 0.041007 | TRUE |
| N4BP1 | 11792 | 2.694741 | 0.007044 | 0.041169 | TRUE |
| ASAP2 | 9698 | 2.694732 | 0.007045 | 0.041169 | TRUE |
| FKBP5 | 1786 | 2.693966 | 0.007061 | 0.041248 | TRUE |
| HMGB2 | 13151 | 2.693701 | 0.007066 | 0.041266 | TRUE |
| IMPAD1 | 727 | 2.691846 | 0.007106 | 0.041449 | TRUE |
| GLMN | 7945 | 2.689736 | 0.007151 | 0.04163 | TRUE |
| PCDHGA3 | 7496 | 2.689543 | 0.007155 | 0.04163 | TRUE |
| PRO1804 | 757 | 2.689448 | 0.007157 | 0.04163 | TRUE |
| HIST1H1D | 10002 | 2.687302 | 0.007203 | 0.041815 | TRUE |
| DCLRE1C | 4760 | 2.686798 | 0.007214 | 0.041847 | TRUE |
| BCOR | 12226 | 2.686263 | 0.007226 | 0.041883 | TRUE |
| LATS1 | 11275 | 2.686061 | 0.00723 | 0.041893 | TRUE |
| HSD11B1 | 11697 | 2.684714 | 0.007259 | 0.042046 | TRUE |
| SLC24A2 | 6487 | 2.682108 | 0.007316 | 0.042266 | TRUE |
| FLRT2 | 12995 | 2.680659 | 0.007348 | 0.042386 | TRUE |
| SRSF4 | 9974 | 2.68023 | 0.007357 | 0.042425 | TRUE |
| NR3C1 | 10520 | 2.679433 | 0.007375 | 0.04246 | TRUE |
| LARP4 | 8863 | 2.679111 | 0.007382 | 0.04246 | TRUE |
| TEPSIN | 11207 | 2.677571 | 0.007416 | 0.042611 | TRUE |
| CHFR | 15133 | 2.677531 | 0.007417 | 0.042611 | TRUE |
| ZBTB47 | 9954 | 2.677093 | 0.007426 | 0.042639 | TRUE |
| TENM2 | 12841 | 2.67707 | 0.007427 | 0.042639 | TRUE |
| AGO2 | 9528 | 2.676602 | 0.007437 | 0.04268 | TRUE |
| GNL3L | 9730 | 2.676501 | 0.00744 | 0.04268 | TRUE |
| SERAC1 | 12722 | 2.673678 | 0.007502 | 0.042946 | TRUE |
| PARGP1 | 10999 | 2.671906 | 0.007542 | 0.043142 | TRUE |
| OSTF1 | 6774 | 2.670983 | 0.007563 | 0.04322 | TRUE |
| LEPROTL1 | 6604 | 2.670931 | 0.007564 | 0.04322 | TRUE |
| SLC25A5 | 15412 | 2.670082 | 0.007583 | 0.043293 | TRUE |
| NTNG1 | 10212 | 2.669993 | 0.007585 | 0.043293 | TRUE |
| HEATR1 | 808 | 2.66878 | 0.007613 | 0.043418 | TRUE |
| KLHL34 | 5982 | 2.66862 | 0.007616 | 0.043423 | TRUE |
| UBE2W | 6409 | 2.668315 | 0.007623 | 0.043447 | TRUE |
| LOC100132249 | 8448 | 2.668189 | 0.007626 | 0.043447 | TRUE |
| ANP32A | 7140 | 2.665547 | 0.007686 | 0.043758 | TRUE |
| NDUFAF6 | 11998 | 2.663843 | 0.007725 | 0.043965 | TRUE |
| L3HYPDH | 11622 | 2.662832 | 0.007749 | 0.044001 | TRUE |
| POP1 | 7130 | 2.662396 | 0.007759 | 0.044004 | TRUE |
| LANCL1 | 9002 | 2.662181 | 0.007764 | 0.044004 | TRUE |
| HNRNPL | 2327 | 2.662068 | 0.007766 | 0.044004 | TRUE |
| CASC3 | 6759 | 2.661899 | 0.00777 | 0.044004 | TRUE |
| HNRNPK | 4493 | 2.6612 | 0.007786 | 0.044028 | TRUE |
| DEFB131A | 10266 | 2.660285 | 0.007807 | 0.044095 | TRUE |
| TYW1B | 13194 | 2.659244 | 0.007832 | 0.044183 | TRUE |
| MTG1 | 12543 | 2.658953 | 0.007838 | 0.04419 | TRUE |
| GABRA4 | 14762 | 2.658268 | 0.007854 | 0.044263 | TRUE |
| BCL3 | 2887 | 2.656713 | 0.007891 | 0.04442 | TRUE |
| RNF217 | 546 | 2.655287 | 0.007924 | 0.04456 | TRUE |
| CEP295 | 7122 | 2.654924 | 0.007933 | 0.044592 | TRUE |
| RPGR | 2297 | 2.651728 | 0.008008 | 0.044936 | TRUE |
| LOC643406 | 14214 | 2.64784 | 0.008101 | 0.045374 | TRUE |
| ZBED9 | 2300 | 2.647033 | 0.00812 | 0.045466 | TRUE |
| ST6GALNAC1 | 8734 | 2.646581 | 0.008131 | 0.045511 | TRUE |
| SDHAF2 | 6228 | 2.645454 | 0.008158 | 0.045646 | TRUE |
| THSD7A | 1042 | 2.644092 | 0.008191 | 0.04579 | TRUE |
| ANKZF1 | 6080 | 2.642093 | 0.00824 | 0.046003 | TRUE |
| ZSWIM4 | 4461 | 2.639063 | 0.008314 | 0.046367 | TRUE |
| PDE4B | 5436 | 2.638623 | 0.008324 | 0.0464 | TRUE |
| GREB1L | 12631 | 2.638072 | 0.008338 | 0.046453 | TRUE |
| ZBED1 | 3837 | 2.636429 | 0.008378 | 0.046656 | TRUE |
| DCPS | 2234 | 2.636351 | 0.00838 | 0.046656 | TRUE |
| MBD3 | 616 | 2.636096 | 0.008387 | 0.046674 | TRUE |
| C3orf18 | 12461 | 2.635638 | 0.008398 | 0.046704 | TRUE |
| INSIG2 | 15243 | 2.634215 | 0.008433 | 0.046867 | TRUE |
| EMILIN3 | 5897 | 2.632684 | 0.008471 | 0.047012 | TRUE |
| AKAP13 | 13962 | 2.631428 | 0.008503 | 0.047136 | TRUE |
| ZNF273 | 14124 | 2.630909 | 0.008516 | 0.047188 | TRUE |
| KCTD16 | 5225 | 2.628866 | 0.008567 | 0.047375 | TRUE |
| CEP95 | 5753 | 2.626338 | 0.008631 | 0.047668 | TRUE |
| LINC01128 | 9432 | 2.625794 | 0.008645 | 0.047703 | TRUE |
| NXPE3 | 13280 | 2.624699 | 0.008673 | 0.047772 | TRUE |
| FOSL2 | 4248 | 2.623538 | 0.008702 | 0.047885 | TRUE |
| SFSWAP | 5706 | 2.622231 | 0.008736 | 0.048035 | TRUE |
| LINC02145 | 1360 | 2.622065 | 0.00874 | 0.048042 | TRUE |
| SLC25A16 | 508 | 2.621766 | 0.008748 | 0.04805 | TRUE |
| KCTD18 | 7567 | 2.620702 | 0.008775 | 0.048183 | TRUE |
| MLXIP | 8637 | 2.619463 | 0.008807 | 0.048316 | TRUE |
| MIA3 | 1283 | 2.619454 | 0.008807 | 0.048316 | TRUE |
| SDHC | 10622 | 2.619402 | 0.008808 | 0.048316 | TRUE |
| NMT2 | 13645 | 2.618056 | 0.008843 | 0.048439 | TRUE |
| ITFG2 | 11154 | 2.617343 | 0.008862 | 0.048524 | TRUE |
| USP4 | 9842 | 2.616885 | 0.008874 | 0.048569 | TRUE |
| FBXL20 | 14741 | 2.615231 | 0.008917 | 0.04875 | TRUE |
| TRMT11 | 7740 | 2.615224 | 0.008917 | 0.04875 | TRUE |
| MRPS36 | 12934 | 2.615159 | 0.008919 | 0.04875 | TRUE |
| CRIPAK | 8033 | 2.614423 | 0.008938 | 0.048838 | TRUE |
| CNOT6L | 7303 | 2.613794 | 0.008954 | 0.048911 | TRUE |
| NFKB1 | 13415 | 2.611636 | 0.009011 | 0.049115 | TRUE |
| MARF1 | 8228 | 2.611541 | 0.009014 | 0.049115 | TRUE |
| ADAM22 | 13031 | 2.611412 | 0.009017 | 0.049116 | TRUE |
| EML2 | 10385 | 2.611277 | 0.00902 | 0.049118 | TRUE |
| MIB2 | 299 | 2.609618 | 0.009064 | 0.049339 | TRUE |
| INA | 950 | 2.608409 | 0.009096 | 0.04948 | TRUE |
| ITIH5 | 8457 | 2.607755 | 0.009114 | 0.049557 | TRUE |
| PPP3CC | 12593 | 2.607312 | 0.009126 | 0.0496 | TRUE |
| OTULIN | 12112 | 2.606228 | 0.009155 | 0.049698 | TRUE |
| GSTT1 | 988 | 2.605108 | 0.009185 | 0.049789 | TRUE |
| HS6ST2 | 12975 | 2.605087 | 0.009185 | 0.049789 | TRUE |
| LRRC34 | 12254 | 2.604263 | 0.009207 | 0.04986 | TRUE |
| WAPL | 12365 | 2.604242 | 0.009208 | 0.04986 | TRUE |
| LPGAT1 | 7300 | 2.603471 | 0.009229 | 0.049955 | TRUE |
| RAB37 | 15119 | 2.603219 | 0.009235 | 0.049974 | TRUE |
| NTN5 | 8703 | 2.602695 | 0.009249 | 0.050016 | FALSE |
| ZNF195 | 5473 | 2.601145 | 0.009291 | 0.050215 | FALSE |
| ASAH2 | 11007 | 2.601097 | 0.009293 | 0.050215 | FALSE |
| TRRAP | 7383 | 2.598521 | 0.009363 | 0.05054 | FALSE |
| TBC1D19 | 12700 | 2.598491 | 0.009363 | 0.05054 | FALSE |
| FAM8A1 | 10025 | 2.598273 | 0.009369 | 0.050542 | FALSE |
| RPE | 3397 | 2.598005 | 0.009377 | 0.050547 | FALSE |
| ZNF470 | 13724 | 2.598002 | 0.009377 | 0.050547 | FALSE |
| MSTO2P | 9879 | 2.595054 | 0.009458 | 0.05093 | FALSE |
| ZNF395 | 7613 | 2.59483 | 0.009464 | 0.050943 | FALSE |
| WIPI1 | 9565 | 2.59383 | 0.009491 | 0.051059 | FALSE |
| CHD8 | 14895 | 2.593479 | 0.009501 | 0.051059 | FALSE |
| MX1 | 3210 | 2.589984 | 0.009598 | 0.051465 | FALSE |
| ZNF143 | 9098 | 2.589922 | 0.0096 | 0.051465 | FALSE |
| MCPH1 | 38 | 2.589128 | 0.009622 | 0.051567 | FALSE |
| FRMPD4 | 8154 | 2.588994 | 0.009626 | 0.051569 | FALSE |
| ZNF767P | 12650 | 2.588577 | 0.009637 | 0.051614 | FALSE |
| CDR2 | 9237 | 2.586682 | 0.00969 | 0.051845 | FALSE |
| RGS7 | 14307 | 2.586488 | 0.009696 | 0.051857 | FALSE |
| CBLB | 3745 | 2.581879 | 0.009826 | 0.052364 | FALSE |
| NPIPA8 | 9678 | 2.581832 | 0.009828 | 0.052364 | FALSE |
| RRP12 | 5561 | 2.580187 | 0.009875 | 0.05248 | FALSE |
| MEF2A | 3767 | 2.580141 | 0.009876 | 0.05248 | FALSE |
| CAPN7 | 5768 | 2.57948 | 0.009895 | 0.052543 | FALSE |
| ADAM9 | 8327 | 2.579183 | 0.009903 | 0.052553 | FALSE |
| ZNF304 | 5593 | 2.57726 | 0.009959 | 0.052792 | FALSE |
| HERPUD1 | 15367 | 2.575369 | 0.010013 | 0.052974 | FALSE |
| STAU2 | 7650 | 2.575255 | 0.010017 | 0.052974 | FALSE |
| SLC25A17 | 11149 | 2.574425 | 0.010041 | 0.053083 | FALSE |
| KDM4A | 2789 | 2.574206 | 0.010047 | 0.053099 | FALSE |
| DPY19L4 | 8419 | 2.573689 | 0.010062 | 0.053142 | FALSE |
| FAM118A | 7869 | 2.57208 | 0.010109 | 0.053337 | FALSE |
| CCDC25 | 5405 | 2.57207 | 0.010109 | 0.053337 | FALSE |
| EMB | 3720 | 2.570787 | 0.010147 | 0.053463 | FALSE |
| UBE2D1 | 1207 | 2.570222 | 0.010163 | 0.053532 | FALSE |
| HNRNPU | 5341 | 2.569529 | 0.010184 | 0.053621 | FALSE |
| PVR | 13683 | 2.56926 | 0.010192 | 0.053645 | FALSE |
| TMEM127 | 9232 | 2.568851 | 0.010204 | 0.05369 | FALSE |
| SLC35B4 | 11463 | 2.568636 | 0.01021 | 0.053705 | FALSE |
| TMF1 | 5621 | 2.56794 | 0.01023 | 0.053759 | FALSE |
| PGGT1B | 1868 | 2.567827 | 0.010234 | 0.053759 | FALSE |
| ACSF3 | 979 | 2.566511 | 0.010273 | 0.053945 | FALSE |
| LRFN5 | 11730 | 2.565408 | 0.010305 | 0.054026 | FALSE |
| PIK3C3 | 43 | 2.56415 | 0.010343 | 0.054186 | FALSE |
| SV2A | 13804 | 2.563442 | 0.010364 | 0.054224 | FALSE |
| NRBF2 | 8699 | 2.563209 | 0.010371 | 0.054226 | FALSE |
| ADGRL2 | 7896 | 2.563198 | 0.010371 | 0.054226 | FALSE |
| KRBA1 | 4562 | 2.562632 | 0.010388 | 0.054296 | FALSE |
| ZBTB43 | 6590 | 2.56223 | 0.0104 | 0.054322 | FALSE |
| IRS1 | 10877 | 2.561725 | 0.010415 | 0.054383 | FALSE |
| DPY19L1P1 | 15632 | 2.559189 | 0.010492 | 0.054709 | FALSE |
| EML5 | 9805 | 2.558744 | 0.010505 | 0.05476 | FALSE |
| KNDC1 | 9877 | 2.558289 | 0.010519 | 0.054814 | FALSE |
| RND1 | 4832 | 2.556578 | 0.010571 | 0.055011 | FALSE |
| NPAT | 10699 | 2.556162 | 0.010583 | 0.055058 | FALSE |
| METTL3 | 6918 | 2.554114 | 0.010646 | 0.055346 | FALSE |
| SLC12A6 | 4851 | 2.553518 | 0.010664 | 0.055412 | FALSE |
| DPH3 | 7546 | 2.553467 | 0.010666 | 0.055412 | FALSE |
| FADS6 | 8572 | 2.553002 | 0.01068 | 0.05545 | FALSE |
| BTBD3 | 9607 | 2.551597 | 0.010723 | 0.055655 | FALSE |
| ING3 | 13149 | 2.549031 | 0.010802 | 0.055992 | FALSE |
| MAGI3 | 10816 | 2.547739 | 0.010842 | 0.056125 | FALSE |
| SULT4A1 | 13053 | 2.547417 | 0.010852 | 0.05614 | FALSE |
| GRAMD1B | 15258 | 2.545397 | 0.010915 | 0.056428 | FALSE |
| ZNF398 | 14928 | 2.544684 | 0.010938 | 0.056525 | FALSE |
| NFAT5 | 13153 | 2.544379 | 0.010947 | 0.056547 | FALSE |
| HSF4 | 15619 | 2.543021 | 0.01099 | 0.056682 | FALSE |
| CLVS1 | 12173 | 2.542908 | 0.010993 | 0.056682 | FALSE |
| ARAP2 | 3179 | 2.542427 | 0.011009 | 0.056741 | FALSE |
| RDH5 | 8076 | 2.541694 | 0.011032 | 0.056842 | FALSE |
| CCDC88B | 13229 | 2.54038 | 0.011073 | 0.056999 | FALSE |
| PLA2G5 | 14078 | 2.539403 | 0.011104 | 0.05714 | FALSE |
| UBE2Q2 | 12200 | 2.5389 | 0.01112 | 0.057166 | FALSE |
| PPIEL | 972 | 2.538116 | 0.011145 | 0.057256 | FALSE |
| PACS2 | 15284 | 2.537403 | 0.011168 | 0.057336 | FALSE |
| RALGAPA1 | 8242 | 2.53581 | 0.011219 | 0.057566 | FALSE |
| LOC100288203 | 9739 | 2.535685 | 0.011223 | 0.057566 | FALSE |
| MICA | 6601 | 2.534238 | 0.011269 | 0.057742 | FALSE |
| FGF17 | 15505 | 2.533606 | 0.01129 | 0.05779 | FALSE |
| LTN1 | 7891 | 2.533271 | 0.0113 | 0.057826 | FALSE |
| DYM | 2630 | 2.533038 | 0.011308 | 0.057846 | FALSE |
| PIP4P2 | 11656 | 2.531576 | 0.011355 | 0.058011 | FALSE |
| SETD7 | 851 | 2.531365 | 0.011362 | 0.058027 | FALSE |
| LZTS3 | 10188 | 2.531149 | 0.011369 | 0.058044 | FALSE |
| PCGF5 | 14404 | 2.530517 | 0.011389 | 0.05813 | FALSE |
| CNTRL | 14176 | 2.530357 | 0.011395 | 0.058137 | FALSE |
| EPN3 | 21 | 2.528307 | 0.011461 | 0.058387 | FALSE |
| SLC9A5 | 8291 | 2.528191 | 0.011465 | 0.058387 | FALSE |
| LMAN1 | 15390 | 2.52819 | 0.011465 | 0.058387 | FALSE |
| NKX6-3 | 7957 | 2.528168 | 0.011466 | 0.058387 | FALSE |
| GABRG2 | 11673 | 2.526475 | 0.011521 | 0.058573 | FALSE |
| BCAT1 | 8580 | 2.52545 | 0.011555 | 0.058712 | FALSE |
| KCNA5 | 1921 | 2.524697 | 0.01158 | 0.058813 | FALSE |
| MAP3K3 | 9902 | 2.52207 | 0.011667 | 0.059169 | FALSE |
| RFX5 | 13786 | 2.521633 | 0.011681 | 0.059194 | FALSE |
| SLC39A14 | 7660 | 2.520942 | 0.011704 | 0.059271 | FALSE |
| AASDH | 9298 | 2.520654 | 0.011714 | 0.059301 | FALSE |
| NEXN | 4421 | 2.520182 | 0.011729 | 0.059316 | FALSE |
| KLF9 | 6637 | 2.520106 | 0.011732 | 0.059316 | FALSE |
| CAVIN4 | 564 | 2.517524 | 0.011818 | 0.059695 | FALSE |
| CD99L2 | 12929 | 2.517097 | 0.011833 | 0.059748 | FALSE |
| TRMT9B | 50 | 2.516936 | 0.011838 | 0.059756 | FALSE |
| YIPF4 | 5232 | 2.515613 | 0.011883 | 0.059945 | FALSE |
| ATP13A3 | 12660 | 2.514789 | 0.01191 | 0.060028 | FALSE |
| TTC28 | 7051 | 2.514675 | 0.011914 | 0.060028 | FALSE |
| VPS54 | 551 | 2.513647 | 0.011949 | 0.060141 | FALSE |
| PSMD12 | 14929 | 2.513016 | 0.01197 | 0.06023 | FALSE |
| RARB | 7284 | 2.512724 | 0.01198 | 0.060241 | FALSE |
| PDCL3P4 | 7468 | 2.512721 | 0.01198 | 0.060241 | FALSE |
| FAM81A | 2898 | 2.511193 | 0.012032 | 0.060483 | FALSE |
| ATF6B | 14974 | 2.510909 | 0.012042 | 0.060512 | FALSE |
| HNRNPH1 | 6175 | 2.510777 | 0.012047 | 0.060515 | FALSE |
| GLRX3 | 15147 | 2.510631 | 0.012052 | 0.060521 | FALSE |
| SIAH1 | 12074 | 2.51033 | 0.012062 | 0.060537 | FALSE |
| ATP11B | 10171 | 2.510311 | 0.012062 | 0.060537 | FALSE |
| PPARGC1B | 7727 | 2.509201 | 0.0121 | 0.060674 | FALSE |
| MED1 | 8702 | 2.509168 | 0.012102 | 0.060674 | FALSE |
| SDHAF3 | 6370 | 2.508021 | 0.012141 | 0.060794 | FALSE |
| IL17RC | 13686 | 2.505846 | 0.012216 | 0.061062 | FALSE |
| RBM7 | 13144 | 2.499231 | 0.012446 | 0.061887 | FALSE |
| SAR1B | 854 | 2.498089 | 0.012486 | 0.062013 | FALSE |
| ZFP3 | 215 | 2.497717 | 0.0125 | 0.062014 | FALSE |
| OR2AK2 | 14065 | 2.494086 | 0.012628 | 0.062397 | FALSE |
| INSR | 14025 | 2.493891 | 0.012635 | 0.062397 | FALSE |
| KANSL1 | 6986 | 2.493695 | 0.012642 | 0.062397 | FALSE |
| LOC100507507 | 7304 | 2.493372 | 0.012654 | 0.062397 | FALSE |
| FIZ1 | 3693 | 2.493313 | 0.012656 | 0.062397 | FALSE |
| ZNF354C | 2979 | 2.493287 | 0.012657 | 0.062397 | FALSE |
| ASB10 | 12712 | 2.492572 | 0.012682 | 0.062454 | FALSE |
| JMJD1C | 2049 | 2.492516 | 0.012684 | 0.062454 | FALSE |
| BMPER | 805 | 2.491763 | 0.012711 | 0.062547 | FALSE |
| STX6 | 10902 | 2.49121 | 0.012731 | 0.062613 | FALSE |
| ZKSCAN8 | 11705 | 2.489611 | 0.012788 | 0.062828 | FALSE |
| MAPK10 | 7981 | 2.489474 | 0.012793 | 0.062833 | FALSE |
| SLC12A7 | 2675 | 2.489182 | 0.012804 | 0.062865 | FALSE |
| RBM48 | 12363 | 2.488479 | 0.012829 | 0.062949 | FALSE |
| NEK4 | 78 | 2.486761 | 0.012891 | 0.063139 | FALSE |
| SLC8A1 | 10694 | 2.486741 | 0.012892 | 0.063139 | FALSE |
| IL17RA | 1290 | 2.486445 | 0.012903 | 0.063172 | FALSE |
| ADM | 2786 | 2.486137 | 0.012914 | 0.063207 | FALSE |
| ABCA9 | 5052 | 2.48593 | 0.012921 | 0.063224 | FALSE |
| IFRD2 | 13204 | 2.485461 | 0.012938 | 0.063248 | FALSE |
| IGLL3P | 15436 | 2.484901 | 0.012959 | 0.063277 | FALSE |
| ABCC8 | 12022 | 2.483483 | 0.01301 | 0.063501 | FALSE |
| SORL1 | 1334 | 2.482705 | 0.013039 | 0.063538 | FALSE |
| INTS4P1 | 9140 | 2.482432 | 0.013049 | 0.063549 | FALSE |
| ATP10A | 5066 | 2.481342 | 0.013089 | 0.063704 | FALSE |
| ZNF500 | 15124 | 2.477465 | 0.013232 | 0.064341 | FALSE |
| SRPK1 | 10272 | 2.475889 | 0.01329 | 0.064541 | FALSE |
| IKZF4 | 14926 | 2.475426 | 0.013308 | 0.064589 | FALSE |
| ZNF287 | 2400 | 2.475076 | 0.013321 | 0.0646 | FALSE |
| KIAA0355 | 5764 | 2.475003 | 0.013324 | 0.0646 | FALSE |
| SDHAF4 | 10889 | 2.474727 | 0.013334 | 0.0646 | FALSE |
| ZNF280D | 6991 | 2.471551 | 0.013453 | 0.065071 | FALSE |
| CSKMT | 5127 | 2.471344 | 0.013461 | 0.065083 | FALSE |
| NUDT5 | 13574 | 2.470573 | 0.01349 | 0.065142 | FALSE |
| C11orf54 | 9367 | 2.470495 | 0.013493 | 0.065142 | FALSE |
| MITF | 10197 | 2.469566 | 0.013528 | 0.065231 | FALSE |
| CCDC84 | 2771 | 2.469303 | 0.013538 | 0.065259 | FALSE |
| SMCHD1 | 3314 | 2.467737 | 0.013597 | 0.065463 | FALSE |
| MKX | 6596 | 2.467089 | 0.013622 | 0.065522 | FALSE |
| AGAP5 | 6777 | 2.466499 | 0.013644 | 0.06559 | FALSE |
| ATL2 | 48 | 2.466265 | 0.013653 | 0.065613 | FALSE |
| EBF4 | 15352 | 2.466045 | 0.013661 | 0.065633 | FALSE |
| CHML | 2308 | 2.464683 | 0.013713 | 0.065843 | FALSE |
| PRDM5 | 9957 | 2.464678 | 0.013714 | 0.065843 | FALSE |
| PIGC | 12298 | 2.461177 | 0.013848 | 0.066448 | FALSE |
| BICD2 | 3710 | 2.460657 | 0.013868 | 0.066484 | FALSE |
| LINC01106 | 15516 | 2.460488 | 0.013875 | 0.066495 | FALSE |
| SPTSSB | 13089 | 2.45974 | 0.013904 | 0.066613 | FALSE |
| NIPA1 | 2994 | 2.457604 | 0.013987 | 0.06696 | FALSE |
| RRNAD1 | 3437 | 2.457431 | 0.013993 | 0.06696 | FALSE |
| GCSHP3 | 13503 | 2.455911 | 0.014053 | 0.067117 | FALSE |
| PKD1 | 8619 | 2.455894 | 0.014053 | 0.067117 | FALSE |
| KDM2B | 8543 | 2.455824 | 0.014056 | 0.067117 | FALSE |
| SCN8A | 7240 | 2.455001 | 0.014088 | 0.06725 | FALSE |
| PRIM1 | 15157 | 2.454543 | 0.014106 | 0.067315 | FALSE |
| CYP2U1 | 10626 | 2.454003 | 0.014128 | 0.067375 | FALSE |
| ITPK1 | 4902 | 2.453754 | 0.014137 | 0.067397 | FALSE |
| EEF1AKMT2 | 12036 | 2.453308 | 0.014155 | 0.067423 | FALSE |
| GART | 909 | 2.452935 | 0.01417 | 0.067434 | FALSE |
| BLZF1 | 641 | 2.452811 | 0.014174 | 0.067434 | FALSE |
| UBR2 | 7750 | 2.451688 | 0.014219 | 0.067625 | FALSE |
| USF2 | 6276 | 2.45128 | 0.014235 | 0.06766 | FALSE |
| ZKSCAN7 | 8595 | 2.450564 | 0.014263 | 0.067753 | FALSE |
| ZNF146 | 15631 | 2.450487 | 0.014266 | 0.067753 | FALSE |
| SRPK2 | 10637 | 2.450269 | 0.014275 | 0.067753 | FALSE |
| SLC19A2 | 15290 | 2.450242 | 0.014276 | 0.067753 | FALSE |
| SYNDIG1 | 15440 | 2.448273 | 0.014354 | 0.068059 | FALSE |
| PGBD2 | 8423 | 2.446958 | 0.014407 | 0.068207 | FALSE |
| PNLDC1 | 15324 | 2.446284 | 0.014434 | 0.068265 | FALSE |
| ZNF292 | 725 | 2.446217 | 0.014436 | 0.068265 | FALSE |
| MCM7 | 11731 | 2.445433 | 0.014468 | 0.068372 | FALSE |
| RELT | 3596 | 2.444931 | 0.014488 | 0.068447 | FALSE |
| DNM1 | 15585 | 2.444358 | 0.014511 | 0.068514 | FALSE |
| UBE4A | 3885 | 2.444098 | 0.014521 | 0.068543 | FALSE |
| TGFBR1 | 4771 | 2.443786 | 0.014534 | 0.068582 | FALSE |
| PCDHB14 | 7366 | 2.443161 | 0.014559 | 0.06868 | FALSE |
| TPTE | 1684 | 2.443027 | 0.014565 | 0.068683 | FALSE |
| CAMK2B | 11285 | 2.442924 | 0.014569 | 0.068683 | FALSE |
| HERC2P2 | 5570 | 2.442678 | 0.014579 | 0.06871 | FALSE |
| TTLL11 | 6673 | 2.442188 | 0.014599 | 0.068761 | FALSE |
| CEPT1 | 12440 | 2.441628 | 0.014621 | 0.068847 | FALSE |
| TIMM23B | 13002 | 2.438594 | 0.014745 | 0.069261 | FALSE |
| LOC441155 | 4380 | 2.434507 | 0.014912 | 0.069859 | FALSE |
| CEP170 | 7767 | 2.433714 | 0.014945 | 0.069971 | FALSE |
| TAP1 | 6798 | 2.432943 | 0.014977 | 0.070099 | FALSE |
| DDX11 | 11897 | 2.432668 | 0.014988 | 0.070131 | FALSE |
| MAML1 | 9394 | 2.432491 | 0.014995 | 0.070144 | FALSE |
| ABCA6 | 3311 | 2.432249 | 0.015005 | 0.070144 | FALSE |
| SLC35A5 | 4335 | 2.432168 | 0.015009 | 0.070144 | FALSE |
| NANOG | 10083 | 2.43112 | 0.015052 | 0.070305 | FALSE |
| PPIL3 | 542 | 2.431015 | 0.015057 | 0.070305 | FALSE |
| TNRC6B | 14090 | 2.430422 | 0.015081 | 0.070378 | FALSE |
| FCHO2 | 3597 | 2.430313 | 0.015086 | 0.070378 | FALSE |
| PDS5A | 10984 | 2.428779 | 0.01515 | 0.070613 | FALSE |
| RAB11FIP3 | 722 | 2.428312 | 0.015169 | 0.070683 | FALSE |
| AMER1 | 13373 | 2.427963 | 0.015184 | 0.070726 | FALSE |
| SKIL | 12751 | 2.427875 | 0.015188 | 0.070726 | FALSE |
| KCNAB1 | 7401 | 2.427223 | 0.015215 | 0.070832 | FALSE |
| PURA | 6463 | 2.426658 | 0.015239 | 0.070921 | FALSE |
| C5orf24 | 8748 | 2.426307 | 0.015253 | 0.070969 | FALSE |
| CRIP3 | 2428 | 2.425478 | 0.015288 | 0.071089 | FALSE |
| TMEM267 | 2667 | 2.425083 | 0.015305 | 0.071145 | FALSE |
| SCAMP1 | 1110 | 2.421849 | 0.015442 | 0.071567 | FALSE |
| SCAMP5 | 12903 | 2.42175 | 0.015446 | 0.071567 | FALSE |
| ACVR1 | 10916 | 2.420915 | 0.015481 | 0.071689 | FALSE |
| KDM1A | 2989 | 2.418647 | 0.015578 | 0.072088 | FALSE |
| ZFPM2 | 8097 | 2.416779 | 0.015659 | 0.072337 | FALSE |
| ROCK1 | 11678 | 2.41628 | 0.01568 | 0.072394 | FALSE |
| ZNF182 | 12475 | 2.415518 | 0.015713 | 0.072484 | FALSE |
| INTS6-AS1 | 11919 | 2.415504 | 0.015713 | 0.072484 | FALSE |
| PVRIG | 12240 | 2.414357 | 0.015763 | 0.072691 | FALSE |
| PAXBP1 | 12669 | 2.414087 | 0.015775 | 0.072702 | FALSE |
| POMT2 | 4636 | 2.413941 | 0.015781 | 0.07271 | FALSE |
| MUM1 | 578 | 2.413604 | 0.015796 | 0.072756 | FALSE |
| LOC642929 | 10895 | 2.41289 | 0.015827 | 0.072855 | FALSE |
| MRPS30 | 208 | 2.409813 | 0.015961 | 0.073365 | FALSE |
| HECA | 10583 | 2.40927 | 0.015984 | 0.073452 | FALSE |
| IMPA2 | 10264 | 2.407042 | 0.016082 | 0.073794 | FALSE |
| STIM2 | 7001 | 2.406475 | 0.016107 | 0.073855 | FALSE |
| SLC25A51P1 | 5110 | 2.406309 | 0.016115 | 0.073855 | FALSE |
| FEM1C | 11143 | 2.404903 | 0.016177 | 0.074118 | FALSE |
| RAPGEF6 | 1798 | 2.4031 | 0.016257 | 0.074337 | FALSE |
| PRMT8 | 1819 | 2.403077 | 0.016258 | 0.074337 | FALSE |
| GMCL1 | 12835 | 2.402598 | 0.016279 | 0.074391 | FALSE |
| DBR1 | 5960 | 2.402146 | 0.016299 | 0.074461 | FALSE |
| KIF5A | 15080 | 2.401168 | 0.016343 | 0.074638 | FALSE |
| ZNF652 | 8557 | 2.399948 | 0.016397 | 0.074866 | FALSE |
| GTF2IRD1 | 6250 | 2.398424 | 0.016466 | 0.075065 | FALSE |
| SEC24B | 11411 | 2.397435 | 0.01651 | 0.075154 | FALSE |
| ZNF615 | 1928 | 2.397364 | 0.016514 | 0.075154 | FALSE |
| WWP1 | 13587 | 2.397064 | 0.016527 | 0.07516 | FALSE |
| LGI2 | 14157 | 2.397035 | 0.016528 | 0.07516 | FALSE |
| HAUS3 | 1896 | 2.39606 | 0.016572 | 0.075247 | FALSE |
| FAM76B | 7947 | 2.39545 | 0.0166 | 0.075326 | FALSE |
| DHX33 | 9390 | 2.395358 | 0.016604 | 0.075326 | FALSE |
| HDAC8 | 3872 | 2.392912 | 0.016715 | 0.075786 | FALSE |
| ST7L | 6140 | 2.39262 | 0.016729 | 0.075824 | FALSE |
| HAPLN4 | 15246 | 2.392177 | 0.016749 | 0.075894 | FALSE |
| ABCB7 | 5185 | 2.391705 | 0.01677 | 0.075947 | FALSE |
| SESN1 | 32 | 2.391369 | 0.016786 | 0.075995 | FALSE |
| STXBP5L | 2317 | 2.391191 | 0.016794 | 0.07601 | FALSE |
| BIRC6 | 10201 | 2.391075 | 0.016799 | 0.076012 | FALSE |
| CWF19L1 | 3583 | 2.38836 | 0.016924 | 0.076478 | FALSE |
| ARHGAP21 | 11717 | 2.388207 | 0.016931 | 0.076478 | FALSE |
| EPHA7 | 14951 | 2.388193 | 0.016931 | 0.076478 | FALSE |
| GOLGA6L10 | 695 | 2.387954 | 0.016942 | 0.076505 | FALSE |
| WDR4 | 8003 | 2.387092 | 0.016982 | 0.076663 | FALSE |
| SPNS2 | 11791 | 2.385785 | 0.017043 | 0.076891 | FALSE |
| C6orf47 | 3236 | 2.385394 | 0.017061 | 0.076943 | FALSE |
| THUMPD2 | 752 | 2.385326 | 0.017064 | 0.076943 | FALSE |
| SETD1A | 15213 | 2.381782 | 0.017229 | 0.077469 | FALSE |
| CEP76 | 12003 | 2.381756 | 0.01723 | 0.077469 | FALSE |
| PASK | 10581 | 2.380131 | 0.017306 | 0.077767 | FALSE |
| KCNMA1 | 14083 | 2.379177 | 0.017351 | 0.077928 | FALSE |
| NRG2 | 9618 | 2.379157 | 0.017352 | 0.077928 | FALSE |
| PMS2P5 | 8761 | 2.377945 | 0.017409 | 0.07814 | FALSE |
| NOP14 | 11779 | 2.377184 | 0.017445 | 0.078279 | FALSE |
| KAT8 | 4368 | 2.376534 | 0.017476 | 0.078349 | FALSE |
| RASGRF1 | 13146 | 2.37622 | 0.017491 | 0.078394 | FALSE |
| LNX1 | 15522 | 2.37599 | 0.017502 | 0.078398 | FALSE |
| EXD2 | 9285 | 2.374816 | 0.017558 | 0.078535 | FALSE |
| MECOM | 15612 | 2.374464 | 0.017574 | 0.078555 | FALSE |
| ACTN4 | 6352 | 2.372583 | 0.017664 | 0.078831 | FALSE |
| MIB1 | 15206 | 2.372449 | 0.017671 | 0.078831 | FALSE |
| FNDC4 | 14818 | 2.372336 | 0.017676 | 0.078831 | FALSE |
| ADGRA2 | 6386 | 2.372268 | 0.017679 | 0.078831 | FALSE |
| RNF180 | 13388 | 2.37096 | 0.017742 | 0.07902 | FALSE |
| SRSF1 | 15165 | 2.370128 | 0.017782 | 0.079153 | FALSE |
| MYO9A | 12539 | 2.369297 | 0.017822 | 0.079308 | FALSE |
| WDR75 | 8858 | 2.368694 | 0.017851 | 0.07937 | FALSE |
| API5 | 108 | 2.367431 | 0.017912 | 0.079574 | FALSE |
| ZNF644 | 975 | 2.36726 | 0.01792 | 0.079588 | FALSE |
| DEXI | 3514 | 2.367029 | 0.017932 | 0.079615 | FALSE |
| SYT12 | 9017 | 2.366421 | 0.017961 | 0.079704 | FALSE |
| ITSN2 | 6254 | 2.365358 | 0.018013 | 0.079862 | FALSE |
| CDKN1B | 4272 | 2.363625 | 0.018097 | 0.080122 | FALSE |
| RFT1 | 10307 | 2.363469 | 0.018105 | 0.080125 | FALSE |
| ARID1B | 2269 | 2.363314 | 0.018112 | 0.080125 | FALSE |
| VSIG10 | 2361 | 2.363149 | 0.01812 | 0.080125 | FALSE |
| RCOR3 | 9867 | 2.362932 | 0.018131 | 0.080125 | FALSE |
| ABRACL | 13406 | 2.36277 | 0.018139 | 0.080126 | FALSE |
| VPS41 | 14714 | 2.361789 | 0.018187 | 0.080316 | FALSE |
| NUDT15 | 2563 | 2.360799 | 0.018236 | 0.080485 | FALSE |
| LOC155060 | 14496 | 2.358085 | 0.018369 | 0.080916 | FALSE |
| ZNF280A | 8223 | 2.355864 | 0.01848 | 0.08127 | FALSE |
| CHGA | 4567 | 2.35564 | 0.018491 | 0.08129 | FALSE |
| TRIM52 | 90 | 2.354925 | 0.018526 | 0.081378 | FALSE |
| MAP1LC3B | 14980 | 2.354348 | 0.018555 | 0.081459 | FALSE |
| PLEKHA8 | 1238 | 2.354013 | 0.018572 | 0.081509 | FALSE |
| SNX25 | 5988 | 2.353284 | 0.018608 | 0.08161 | FALSE |
| ZNF223 | 7798 | 2.352724 | 0.018636 | 0.0817 | FALSE |
| HAUS8 | 15540 | 2.352461 | 0.01865 | 0.081735 | FALSE |
| SLC46A3 | 10382 | 2.352262 | 0.01866 | 0.081756 | FALSE |
| EPS15 | 5498 | 2.351905 | 0.018678 | 0.081789 | FALSE |
| LOXL2 | 10339 | 2.35175 | 0.018685 | 0.0818 | FALSE |
| PVALB | 14242 | 2.350928 | 0.018727 | 0.081958 | FALSE |
| UBE2Q2P1 | 12530 | 2.350582 | 0.018744 | 0.081966 | FALSE |
| FNDC5 | 13259 | 2.350578 | 0.018744 | 0.081966 | FALSE |
| PAFAH1B2 | 7278 | 2.348423 | 0.018853 | 0.08235 | FALSE |
| EPB41L2 | 14035 | 2.347377 | 0.018906 | 0.082484 | FALSE |
| RNF138 | 2851 | 2.346106 | 0.018971 | 0.082656 | FALSE |
| MRRF | 15349 | 2.346015 | 0.018975 | 0.082656 | FALSE |
| UNC13C | 8738 | 2.345974 | 0.018977 | 0.082656 | FALSE |
| BRSK1 | 15205 | 2.343289 | 0.019115 | 0.083144 | FALSE |
| PIP5KL1 | 11002 | 2.341029 | 0.019231 | 0.083532 | FALSE |
| RCSD1 | 13892 | 2.340902 | 0.019237 | 0.083532 | FALSE |
| CCNB1IP1 | 8460 | 2.340895 | 0.019238 | 0.083532 | FALSE |
| ZFX | 13414 | 2.340801 | 0.019242 | 0.083532 | FALSE |
| UPF3A | 4244 | 2.340738 | 0.019246 | 0.083532 | FALSE |
| COBLL1 | 661 | 2.340721 | 0.019247 | 0.083532 | FALSE |
| DCP1B | 12853 | 2.340585 | 0.019254 | 0.083539 | FALSE |
| ISG20L2 | 8958 | 2.33986 | 0.019291 | 0.083632 | FALSE |
| FBXO33 | 3512 | 2.33832 | 0.019371 | 0.083931 | FALSE |
| SDR16C5 | 6662 | 2.33762 | 0.019407 | 0.084018 | FALSE |
| TPRKB | 6169 | 2.33638 | 0.019471 | 0.084204 | FALSE |
| FAM13A | 13814 | 2.335576 | 0.019513 | 0.084326 | FALSE |
| ZNF766 | 2037 | 2.334964 | 0.019545 | 0.084407 | FALSE |
| ASPDH | 4829 | 2.334823 | 0.019553 | 0.084413 | FALSE |
| TGDS | 9669 | 2.334727 | 0.019558 | 0.084413 | FALSE |
| STAR | 11499 | 2.333639 | 0.019615 | 0.084598 | FALSE |
| CCL27 | 2669 | 2.333601 | 0.019617 | 0.084598 | FALSE |
| CRTC3 | 9139 | 2.333402 | 0.019627 | 0.084619 | FALSE |
| FAM43A | 14006 | 2.332412 | 0.019679 | 0.084797 | FALSE |
| FSTL4 | 1481 | 2.330025 | 0.019805 | 0.085268 | FALSE |
| NAP1L3 | 11795 | 2.329301 | 0.019843 | 0.085386 | FALSE |
| CMPK2 | 14776 | 2.329162 | 0.01985 | 0.085394 | FALSE |
| ANKRD37 | 3435 | 2.328901 | 0.019864 | 0.08543 | FALSE |
| SLC47A1 | 7843 | 2.328313 | 0.019895 | 0.085501 | FALSE |
| MPP7 | 13102 | 2.32817 | 0.019903 | 0.085501 | FALSE |
| AUTS2 | 7128 | 2.328117 | 0.019906 | 0.085501 | FALSE |
| POGZ | 12886 | 2.328074 | 0.019908 | 0.085501 | FALSE |
| CORO7 | 9477 | 2.327747 | 0.019926 | 0.085542 | FALSE |
| WDR37 | 14163 | 2.327522 | 0.019937 | 0.085542 | FALSE |
| CCDC174 | 12647 | 2.32636 | 0.019999 | 0.085775 | FALSE |
| LRPPRC | 2833 | 2.326189 | 0.020008 | 0.085776 | FALSE |
| TAS2R19 | 9672 | 2.324891 | 0.020078 | 0.086017 | FALSE |
| SEC62 | 7624 | 2.324047 | 0.020123 | 0.086153 | FALSE |
| ZNF230 | 11245 | 2.323657 | 0.020144 | 0.086153 | FALSE |
| SKA2 | 772 | 2.323479 | 0.020153 | 0.086153 | FALSE |
| NUP93 | 11095 | 2.32336 | 0.02016 | 0.086155 | FALSE |
| ITGA11 | 6557 | 2.323262 | 0.020165 | 0.086155 | FALSE |
| CAMK2G | 6303 | 2.322097 | 0.020228 | 0.086399 | FALSE |
| EXTL2 | 10723 | 2.320914 | 0.020291 | 0.08652 | FALSE |
| RASSF8 | 12760 | 2.320547 | 0.020311 | 0.08652 | FALSE |
| EPM2A | 3617 | 2.320261 | 0.020327 | 0.086538 | FALSE |
| PTPN14 | 5081 | 2.319676 | 0.020358 | 0.086602 | FALSE |
| ALKBH5 | 3207 | 2.319486 | 0.020369 | 0.086622 | FALSE |
| NIPBL | 11474 | 2.318668 | 0.020413 | 0.086764 | FALSE |
| UBAP1 | 14693 | 2.318282 | 0.020434 | 0.086773 | FALSE |
| JADE1 | 11254 | 2.318219 | 0.020437 | 0.086773 | FALSE |
| FAM206A | 8772 | 2.31773 | 0.020464 | 0.086862 | FALSE |
| RILP | 7879 | 2.31756 | 0.020473 | 0.086872 | FALSE |
| GRK2 | 9534 | 2.317208 | 0.020492 | 0.086912 | FALSE |
| PLEKHF1 | 9292 | 2.316747 | 0.020518 | 0.086995 | FALSE |
| DDI2 | 15118 | 2.316435 | 0.020535 | 0.08702 | FALSE |
| LOC389834 | 15075 | 2.315166 | 0.020604 | 0.087266 | FALSE |
| NAAA | 13937 | 2.314277 | 0.020653 | 0.08743 | FALSE |
| ZBTB34 | 10825 | 2.314157 | 0.020659 | 0.08743 | FALSE |
| KSR2 | 2924 | 2.312783 | 0.020735 | 0.087711 | FALSE |
| DLGAP1 | 788 | 2.3122 | 0.020767 | 0.087776 | FALSE |
| LINC01061 | 13990 | 2.312154 | 0.020769 | 0.087776 | FALSE |
| MAP3K9 | 14904 | 2.31071 | 0.020849 | 0.088053 | FALSE |
| C2CD3 | 9382 | 2.310559 | 0.020857 | 0.088053 | FALSE |
| GID8 | 7738 | 2.309837 | 0.020897 | 0.088198 | FALSE |
| FAM106A | 555 | 2.309283 | 0.020928 | 0.08828 | FALSE |
| SENP1 | 7637 | 2.308528 | 0.02097 | 0.088433 | FALSE |
| ARID4B | 8965 | 2.307901 | 0.021005 | 0.088556 | FALSE |
| C17orf75 | 10966 | 2.307593 | 0.021022 | 0.088604 | FALSE |
| TSEN2 | 4025 | 2.301528 | 0.021362 | 0.089579 | FALSE |
| NUDT8 | 15399 | 2.301417 | 0.021368 | 0.089581 | FALSE |
| MCF2 | 246 | 2.301168 | 0.021382 | 0.089616 | FALSE |
| ZC3H4 | 8632 | 2.299297 | 0.021488 | 0.089925 | FALSE |
| SLC35F6 | 12309 | 2.299254 | 0.021491 | 0.089925 | FALSE |
| 6-Mar | 14642 | 2.299074 | 0.021501 | 0.089944 | FALSE |
| CCDC144CP | 1950 | 2.298703 | 0.021522 | 0.090008 | FALSE |
| NAB1 | 14399 | 2.297966 | 0.021564 | 0.090105 | FALSE |
| CPPED1 | 14391 | 2.29791 | 0.021567 | 0.090105 | FALSE |
| PPP1R10 | 12830 | 2.297806 | 0.021573 | 0.090105 | FALSE |
| CCNB1 | 9199 | 2.297789 | 0.021574 | 0.090105 | FALSE |
| GALNT13 | 9314 | 2.297005 | 0.021618 | 0.090268 | FALSE |
| 12-Sep | 15254 | 2.296792 | 0.021631 | 0.090294 | FALSE |
| PCNX4 | 9915 | 2.296095 | 0.02167 | 0.090412 | FALSE |
| UST | 15397 | 2.295864 | 0.021684 | 0.090443 | FALSE |
| MTFR1 | 12066 | 2.295283 | 0.021717 | 0.090558 | FALSE |
| HDX | 5538 | 2.29359 | 0.021814 | 0.090906 | FALSE |
| ALDH1B1 | 2426 | 2.293525 | 0.021818 | 0.090906 | FALSE |
| IGDCC3 | 7702 | 2.293323 | 0.021829 | 0.09093 | FALSE |
| TBCE | 8650 | 2.290115 | 0.022015 | 0.091531 | FALSE |
| TBC1D4 | 6155 | 2.288743 | 0.022094 | 0.091764 | FALSE |
| SLC39A13 | 2398 | 2.286331 | 0.022235 | 0.092226 | FALSE |
| C1orf109 | 5560 | 2.286048 | 0.022251 | 0.09227 | FALSE |
| LIN7C | 2573 | 2.285305 | 0.022295 | 0.092376 | FALSE |
| CCDC171 | 5351 | 2.285207 | 0.022301 | 0.092376 | FALSE |
| ZCCHC3 | 4254 | 2.284993 | 0.022313 | 0.092403 | FALSE |
| ZNF616 | 9475 | 2.284814 | 0.022324 | 0.092405 | FALSE |
| SLC50A1 | 7754 | 2.284146 | 0.022363 | 0.092456 | FALSE |
| WFIKKN1 | 6033 | 2.283492 | 0.022401 | 0.092523 | FALSE |
| UNC79 | 11612 | 2.282472 | 0.022461 | 0.092688 | FALSE |
| LOC100286906 | 3032 | 2.282413 | 0.022465 | 0.092688 | FALSE |
| AURKA | 7713 | 2.281998 | 0.022489 | 0.092724 | FALSE |
| POMC | 10446 | 2.281963 | 0.022492 | 0.092724 | FALSE |
| MRPS30-DT | 7372 | 2.281857 | 0.022498 | 0.092726 | FALSE |
| VEZF1 | 7673 | 2.28163 | 0.022511 | 0.092742 | FALSE |
| KIAA0753 | 10896 | 2.281589 | 0.022514 | 0.092742 | FALSE |
| ZNF180 | 10285 | 2.281385 | 0.022526 | 0.092752 | FALSE |
| MED12 | 100 | 2.281348 | 0.022528 | 0.092752 | FALSE |
| OSBPL9 | 6556 | 2.281215 | 0.022536 | 0.09276 | FALSE |
| ATXN1 | 12289 | 2.280576 | 0.022574 | 0.092842 | FALSE |
| PHF21B | 6219 | 2.27948 | 0.022639 | 0.093036 | FALSE |
| UNKL | 12198 | 2.278496 | 0.022697 | 0.093227 | FALSE |
| KYAT1 | 5484 | 2.277617 | 0.022749 | 0.093393 | FALSE |
| URB2 | 12741 | 2.2774 | 0.022762 | 0.093406 | FALSE |
| MGST2 | 4596 | 2.276793 | 0.022799 | 0.093472 | FALSE |
| SRXN1 | 6201 | 2.276379 | 0.022823 | 0.093538 | FALSE |
| BSN | 4986 | 2.276326 | 0.022827 | 0.093538 | FALSE |
| TAS2R4 | 2834 | 2.27514 | 0.022898 | 0.093731 | FALSE |
| NR1D2 | 1926 | 2.274997 | 0.022906 | 0.093741 | FALSE |
| PCTP | 3478 | 2.274879 | 0.022913 | 0.093746 | FALSE |
| ADAD2 | 4133 | 2.274078 | 0.022961 | 0.093893 | FALSE |
| BEND7 | 14204 | 2.27272 | 0.023043 | 0.094154 | FALSE |
| GTPBP1 | 15074 | 2.271485 | 0.023118 | 0.09436 | FALSE |
| TPPP | 11872 | 2.270628 | 0.02317 | 0.094421 | FALSE |
| SENP7 | 2567 | 2.270537 | 0.023175 | 0.094421 | FALSE |
| LRRC3 | 1059 | 2.269073 | 0.023264 | 0.09466 | FALSE |
| SMARCD2 | 6003 | 2.26754 | 0.023357 | 0.094991 | FALSE |
| ZNF592 | 3498 | 2.267222 | 0.023377 | 0.095045 | FALSE |
| GGH | 7363 | 2.266362 | 0.023429 | 0.09516 | FALSE |
| LMNB2 | 4748 | 2.266259 | 0.023436 | 0.09516 | FALSE |
| COIL | 13305 | 2.265699 | 0.02347 | 0.095275 | FALSE |
| CUL5 | 1571 | 2.264301 | 0.023556 | 0.095549 | FALSE |
| IRF3 | 7774 | 2.264101 | 0.023568 | 0.095574 | FALSE |
| FAM104B | 15248 | 2.263578 | 0.0236 | 0.095679 | FALSE |
| SMC5 | 3212 | 2.262929 | 0.02364 | 0.095792 | FALSE |
| ZNF284 | 2695 | 2.262811 | 0.023647 | 0.095797 | FALSE |
| JRK | 7490 | 2.262106 | 0.023691 | 0.095898 | FALSE |
| ATG16L2 | 12179 | 2.261794 | 0.02371 | 0.095949 | FALSE |
| TSPYL5 | 8117 | 2.261178 | 0.023748 | 0.096056 | FALSE |
| MATR3 | 6846 | 2.260548 | 0.023787 | 0.096164 | FALSE |
| RRS1 | 8967 | 2.260138 | 0.023813 | 0.096217 | FALSE |
| LINC00265 | 6330 | 2.257629 | 0.023969 | 0.096773 | FALSE |
| GOPC | 11467 | 2.257409 | 0.023983 | 0.096778 | FALSE |
| SLC39A8 | 14305 | 2.256654 | 0.02403 | 0.096919 | FALSE |
| IL17RE | 9558 | 2.256025 | 0.024069 | 0.097052 | FALSE |
| WASHC4 | 2685 | 2.255102 | 0.024127 | 0.09721 | FALSE |
| SLC11A2 | 4226 | 2.254329 | 0.024175 | 0.097327 | FALSE |
| SIRT5 | 2700 | 2.254171 | 0.024185 | 0.097327 | FALSE |
| RHOT1 | 3912 | 2.254043 | 0.024193 | 0.097328 | FALSE |
| P3H1 | 9653 | 2.253786 | 0.02421 | 0.097368 | FALSE |
| SLC45A1 | 8372 | 2.252578 | 0.024286 | 0.097588 | FALSE |
| SEC24C | 3013 | 2.252546 | 0.024288 | 0.097588 | FALSE |
| ODF2L | 14072 | 2.252383 | 0.024298 | 0.097588 | FALSE |
| RHO | 6372 | 2.251018 | 0.024384 | 0.097844 | FALSE |
| RMC1 | 3599 | 2.249776 | 0.024463 | 0.098009 | FALSE |
| TRAF7 | 8006 | 2.249344 | 0.024491 | 0.098094 | FALSE |
| ZNF507 | 7288 | 2.247639 | 0.024599 | 0.09844 | FALSE |
| ATM | 10332 | 2.244238 | 0.024817 | 0.099148 | FALSE |
| SPAG4 | 1750 | 2.242441 | 0.024933 | 0.099453 | FALSE |
| ARAF | 11783 | 2.242361 | 0.024938 | 0.099453 | FALSE |
| MPP1 | 8296 | 2.24067 | 0.025047 | 0.099839 | FALSE |
| RPSA | 11103 | 2.240446 | 0.025062 | 0.099871 | FALSE |
| ZNF280B | 13361 | 2.239072 | 0.025151 | 0.100125 | FALSE |
| PDIK1L | 894 | 2.237419 | 0.025259 | 0.100477 | FALSE |
| PPP2R2D | 3626 | 2.237194 | 0.025274 | 0.10051 | FALSE |
| RSRP1 | 1409 | 2.233907 | 0.025489 | 0.101244 | FALSE |
| ASB8 | 8081 | 2.233882 | 0.025491 | 0.101244 | FALSE |
| ZNF785 | 11829 | 2.231228 | 0.025666 | 0.101603 | FALSE |
| HIST1H4A | 2039 | 2.231172 | 0.02567 | 0.101603 | FALSE |
| SMCO2 | 4929 | 2.231138 | 0.025672 | 0.101603 | FALSE |
| NPHP4 | 7485 | 2.230705 | 0.025701 | 0.10169 | FALSE |
| RASGRP2 | 1887 | 2.230404 | 0.025721 | 0.101692 | FALSE |
| CDK6 | 759 | 2.229778 | 0.025762 | 0.101781 | FALSE |
| LINC00652 | 15316 | 2.229027 | 0.025812 | 0.101951 | FALSE |
| PRICKLE4 | 10921 | 2.227981 | 0.025882 | 0.102123 | FALSE |
| ZBTB8A | 14846 | 2.227641 | 0.025904 | 0.102186 | FALSE |
| ZDHHC13 | 3330 | 2.226622 | 0.025973 | 0.102275 | FALSE |
| FANCI | 1614 | 2.226572 | 0.025976 | 0.102275 | FALSE |
| NOC2L | 9091 | 2.226524 | 0.025979 | 0.102275 | FALSE |
| FUBP3 | 12297 | 2.223738 | 0.026166 | 0.102907 | FALSE |
| XYLB | 11959 | 2.223357 | 0.026192 | 0.102944 | FALSE |
| RALGPS1 | 9613 | 2.223264 | 0.026198 | 0.102944 | FALSE |
| SEC14L1 | 4678 | 2.222595 | 0.026243 | 0.10308 | FALSE |
| WDR55 | 588 | 2.221947 | 0.026287 | 0.1032 | FALSE |
| RBM12B | 4247 | 2.221194 | 0.026338 | 0.103322 | FALSE |
| NEFH | 3174 | 2.219652 | 0.026442 | 0.103654 | FALSE |
| MPC1 | 4484 | 2.218517 | 0.02652 | 0.103879 | FALSE |
| TMCC1 | 8651 | 2.218016 | 0.026554 | 0.103987 | FALSE |
| PRKAR2A | 8000 | 2.217491 | 0.02659 | 0.104101 | FALSE |
| PARG | 10830 | 2.216905 | 0.02663 | 0.104231 | FALSE |
| KDF1 | 10069 | 2.216027 | 0.02669 | 0.10443 | FALSE |
| PDCD11 | 3680 | 2.215869 | 0.0267 | 0.10443 | FALSE |
| UBE2QL1 | 9792 | 2.21548 | 0.026727 | 0.104482 | FALSE |
| NVL | 14959 | 2.215216 | 0.026745 | 0.104527 | FALSE |
| KLHL20 | 10848 | 2.214618 | 0.026786 | 0.104605 | FALSE |
| TANK | 7951 | 2.214532 | 0.026792 | 0.104605 | FALSE |
| CHMP7 | 2623 | 2.214477 | 0.026796 | 0.104605 | FALSE |
| SOHLH1 | 4369 | 2.213655 | 0.026853 | 0.104737 | FALSE |
| CNOT11 | 3166 | 2.213158 | 0.026887 | 0.104844 | FALSE |
| KIAA0391 | 6440 | 2.212269 | 0.026948 | 0.10496 | FALSE |
| FOXP2 | 3996 | 2.212236 | 0.02695 | 0.10496 | FALSE |
| IWS1 | 15219 | 2.212047 | 0.026963 | 0.10496 | FALSE |
| RFPL1 | 3995 | 2.211792 | 0.026981 | 0.104997 | FALSE |
| CALCOCO1 | 13823 | 2.211715 | 0.026986 | 0.104997 | FALSE |
| DNASE1L2 | 2903 | 2.211598 | 0.026994 | 0.105002 | FALSE |
| PKD1P6-NPIPP1 | 4517 | 2.210298 | 0.027084 | 0.105281 | FALSE |
| TBC1D16 | 13395 | 2.210212 | 0.02709 | 0.105281 | FALSE |
| APIP | 8397 | 2.210102 | 0.027098 | 0.105281 | FALSE |
| DOPEY2 | 8323 | 2.210009 | 0.027105 | 0.105281 | FALSE |
| ATF6 | 14534 | 2.209982 | 0.027106 | 0.105281 | FALSE |
| NELFA | 12473 | 2.209837 | 0.027116 | 0.105294 | FALSE |
| MPHOSPH9 | 11844 | 2.20842 | 0.027215 | 0.105624 | FALSE |
| ZFHX2 | 1045 | 2.20794 | 0.027248 | 0.105682 | FALSE |
| ZCCHC6 | 5126 | 2.206384 | 0.027357 | 0.105991 | FALSE |
| ZC3H11B | 9761 | 2.204324 | 0.027502 | 0.106419 | FALSE |
| CTCF | 11990 | 2.203411 | 0.027566 | 0.106588 | FALSE |
| AMACR | 11480 | 2.203295 | 0.027574 | 0.106593 | FALSE |
| LEO1 | 6172 | 2.202381 | 0.027638 | 0.106815 | FALSE |
| CLCN4 | 14824 | 2.201338 | 0.027712 | 0.107022 | FALSE |
| NEURL3 | 4270 | 2.200599 | 0.027764 | 0.10717 | FALSE |
| UCHL5 | 6105 | 2.200394 | 0.027779 | 0.107174 | FALSE |
| FAP | 7940 | 2.199885 | 0.027815 | 0.107287 | FALSE |
| GSK3B | 7916 | 2.197479 | 0.027986 | 0.107814 | FALSE |
| PPP1R3E | 15176 | 2.196735 | 0.028039 | 0.107939 | FALSE |
| HNRNPA1L2 | 13678 | 2.196022 | 0.02809 | 0.108082 | FALSE |
| ZNF37A | 7129 | 2.195914 | 0.028098 | 0.108085 | FALSE |
| ZNF780B | 2566 | 2.195812 | 0.028105 | 0.108087 | FALSE |
| ADSSL1 | 14038 | 2.195074 | 0.028158 | 0.108263 | FALSE |
| UBE4B | 12008 | 2.193459 | 0.028274 | 0.108549 | FALSE |
| GABPA | 738 | 2.192878 | 0.028316 | 0.108657 | FALSE |
| NGEF | 7892 | 2.191636 | 0.028406 | 0.108862 | FALSE |
| CERK | 15117 | 2.191627 | 0.028406 | 0.108862 | FALSE |
| DVL2 | 6565 | 2.191365 | 0.028425 | 0.108862 | FALSE |
| TGS1 | 6311 | 2.190079 | 0.028519 | 0.109112 | FALSE |
| COX19 | 13210 | 2.189919 | 0.02853 | 0.109129 | FALSE |
| FYTTD1 | 4313 | 2.189061 | 0.028592 | 0.109204 | FALSE |
| ZNF136 | 11524 | 2.188902 | 0.028604 | 0.109204 | FALSE |
| CDC42EP3 | 179 | 2.18888 | 0.028606 | 0.109204 | FALSE |
| CTSK | 10397 | 2.18875 | 0.028615 | 0.109214 | FALSE |
| DNAJC5G | 949 | 2.188253 | 0.028651 | 0.109245 | FALSE |
| HAAO | 9079 | 2.187727 | 0.028689 | 0.109358 | FALSE |
| SLC35E3 | 8541 | 2.187602 | 0.028699 | 0.109358 | FALSE |
| C7orf57 | 4080 | 2.187511 | 0.028705 | 0.109358 | FALSE |
| MAP3K10 | 9997 | 2.187221 | 0.028726 | 0.109398 | FALSE |
| TANC1 | 1945 | 2.186982 | 0.028744 | 0.109438 | FALSE |
| MORC2-AS1 | 1380 | 2.186477 | 0.028781 | 0.109525 | FALSE |
| ZBTB26 | 12375 | 2.186044 | 0.028812 | 0.109566 | FALSE |
| PREPL | 13408 | 2.184964 | 0.028891 | 0.109785 | FALSE |
| ZNF3 | 5224 | 2.184522 | 0.028924 | 0.109856 | FALSE |
| GABPB2 | 15034 | 2.184055 | 0.028958 | 0.10996 | FALSE |
| FSBP | 2818 | 2.183808 | 0.028976 | 0.109999 | FALSE |
| FAM135A | 3713 | 2.183637 | 0.028989 | 0.109999 | FALSE |
| NKIRAS1 | 10592 | 2.183628 | 0.02899 | 0.109999 | FALSE |
| DLGAP2 | 11801 | 2.183415 | 0.029005 | 0.110031 | FALSE |
| ARHGAP27P1 | 4822 | 2.183193 | 0.029022 | 0.11004 | FALSE |
| RAB40A | 4797 | 2.182854 | 0.029047 | 0.110108 | FALSE |
| CNNM1 | 8037 | 2.182196 | 0.029095 | 0.110238 | FALSE |
| ZNF385D | 3805 | 2.181844 | 0.029121 | 0.110283 | FALSE |
| SLC31A1 | 1948 | 2.181181 | 0.02917 | 0.110415 | FALSE |
| ABHD17B | 13103 | 2.180566 | 0.029216 | 0.110561 | FALSE |
| HTATIP2 | 7475 | 2.180426 | 0.029226 | 0.110573 | FALSE |
| ERRFI1 | 11835 | 2.178331 | 0.029381 | 0.111119 | FALSE |
| GOLGA1 | 796 | 2.178185 | 0.029392 | 0.111122 | FALSE |
| DNAJC4 | 8440 | 2.177764 | 0.029424 | 0.11115 | FALSE |
| USP36 | 10029 | 2.177738 | 0.029426 | 0.11115 | FALSE |
| INPP5E | 10207 | 2.17759 | 0.029437 | 0.111155 | FALSE |
| TMEM161B-AS1 | 7504 | 2.177338 | 0.029455 | 0.111199 | FALSE |
| LRRC37A3 | 1785 | 2.175535 | 0.02959 | 0.1116 | FALSE |
| USP38 | 14402 | 2.173739 | 0.029725 | 0.111973 | FALSE |
| NFKBIZ | 2212 | 2.172379 | 0.029827 | 0.11225 | FALSE |
| PRRT3 | 5715 | 2.171794 | 0.029871 | 0.112335 | FALSE |
| MBD2 | 14041 | 2.170444 | 0.029973 | 0.112637 | FALSE |
| INTS9 | 10415 | 2.169697 | 0.03003 | 0.112806 | FALSE |
| ZBTB38 | 11912 | 2.168652 | 0.030109 | 0.112958 | FALSE |
| PLAU | 541 | 2.168315 | 0.030135 | 0.113 | FALSE |
| RAB9B | 5893 | 2.167846 | 0.03017 | 0.113079 | FALSE |
| USP33 | 6073 | 2.167747 | 0.030178 | 0.11308 | FALSE |
| MTMR2 | 5817 | 2.167182 | 0.030221 | 0.11316 | FALSE |
| VAMP4 | 1227 | 2.166864 | 0.030245 | 0.11317 | FALSE |
| IRAK1 | 2609 | 2.166609 | 0.030265 | 0.113216 | FALSE |
| PEPD | 9577 | 2.165923 | 0.030317 | 0.113357 | FALSE |
| ATP6V1E2 | 2394 | 2.165804 | 0.030326 | 0.113364 | FALSE |
| MTRF1 | 13309 | 2.164926 | 0.030393 | 0.113534 | FALSE |
| TDP2 | 13131 | 2.16443 | 0.030431 | 0.113649 | FALSE |
| C18orf25 | 4003 | 2.164186 | 0.03045 | 0.113664 | FALSE |
| ANKRD26 | 14329 | 2.163925 | 0.03047 | 0.113712 | FALSE |
| CTNNAL1 | 8729 | 2.163801 | 0.03048 | 0.11372 | FALSE |
| AP1S2 | 4520 | 2.163232 | 0.030523 | 0.113856 | FALSE |
| LINS1 | 1091 | 2.161539 | 0.030654 | 0.11428 | FALSE |
| SNCAIP | 6344 | 2.160467 | 0.030737 | 0.114379 | FALSE |
| G0S2 | 10000 | 2.160294 | 0.03075 | 0.114401 | FALSE |
| SPTB | 3019 | 2.159518 | 0.03081 | 0.114543 | FALSE |
| PER3 | 8470 | 2.156454 | 0.031048 | 0.115278 | FALSE |
| ANKRD13A | 4821 | 2.156392 | 0.031053 | 0.115278 | FALSE |
| REV3L | 4494 | 2.156313 | 0.031059 | 0.115278 | FALSE |
| PLEKHM3 | 8286 | 2.155966 | 0.031086 | 0.115323 | FALSE |
| LINC00963 | 9343 | 2.155174 | 0.031148 | 0.115404 | FALSE |
| RASSF8-AS1 | 11561 | 2.154648 | 0.031189 | 0.115486 | FALSE |
| SGPP2 | 684 | 2.15442 | 0.031207 | 0.115525 | FALSE |
| GAS7 | 831 | 2.153596 | 0.031272 | 0.11571 | FALSE |
| CPEB3 | 6134 | 2.153465 | 0.031282 | 0.11572 | FALSE |
| COL13A1 | 12353 | 2.15319 | 0.031304 | 0.115773 | FALSE |
| RAB3GAP2 | 14323 | 2.152885 | 0.031328 | 0.115834 | FALSE |
| DIP2A | 1421 | 2.15189 | 0.031406 | 0.116069 | FALSE |
| SUSD5 | 12645 | 2.150474 | 0.031518 | 0.116454 | FALSE |
| SSFA2 | 1987 | 2.149521 | 0.031593 | 0.11665 | FALSE |
| ZNF641 | 10298 | 2.149175 | 0.031621 | 0.116681 | FALSE |
| HEXIM1 | 2906 | 2.149132 | 0.031624 | 0.116681 | FALSE |
| TSPAN1 | 11441 | 2.147325 | 0.031767 | 0.1171 | FALSE |
| KLC4 | 2638 | 2.147197 | 0.031778 | 0.11711 | FALSE |
| NUMA1 | 726 | 2.146969 | 0.031796 | 0.117121 | FALSE |
| HECTD1 | 12130 | 2.145715 | 0.031896 | 0.117407 | FALSE |
| KIAA0513 | 6427 | 2.145565 | 0.031908 | 0.117423 | FALSE |
| ZNF490 | 12748 | 2.143526 | 0.032071 | 0.117913 | FALSE |
| H2AFY2 | 9061 | 2.143086 | 0.032106 | 0.117987 | FALSE |
| IQCN | 14423 | 2.142979 | 0.032115 | 0.117991 | FALSE |
| DGCR8 | 12267 | 2.142637 | 0.032142 | 0.118064 | FALSE |
| C7orf25 | 13369 | 2.14232 | 0.032168 | 0.118104 | FALSE |
| PCF11 | 10133 | 2.14101 | 0.032273 | 0.118434 | FALSE |
| RBPJ | 15441 | 2.14082 | 0.032289 | 0.118447 | FALSE |
| NIPAL3 | 7610 | 2.140353 | 0.032326 | 0.118505 | FALSE |
| PPIP5K2 | 5751 | 2.137591 | 0.03255 | 0.119169 | FALSE |
| ZCCHC9 | 7123 | 2.137257 | 0.032577 | 0.119241 | FALSE |
| ZNF41 | 11915 | 2.137062 | 0.032593 | 0.119271 | FALSE |
| OLFML2A | 2017 | 2.136861 | 0.032609 | 0.119295 | FALSE |
| PIK3R4 | 2108 | 2.135937 | 0.032685 | 0.119485 | FALSE |
| CIT | 8471 | 2.134968 | 0.032764 | 0.119671 | FALSE |
| RBAK | 13353 | 2.133813 | 0.032858 | 0.119926 | FALSE |
| KDM5A | 1771 | 2.131604 | 0.033039 | 0.120482 | FALSE |
| ATP5F1C | 14478 | 2.131175 | 0.033075 | 0.120526 | FALSE |
| HNRNPA3 | 71 | 2.129919 | 0.033178 | 0.120785 | FALSE |
| RIPPLY2 | 558 | 2.129845 | 0.033184 | 0.120785 | FALSE |
| ZNF773 | 758 | 2.129395 | 0.033222 | 0.120863 | FALSE |
| AAK1 | 9553 | 2.129305 | 0.033229 | 0.120863 | FALSE |
| C2CD5 | 8525 | 2.129084 | 0.033247 | 0.120891 | FALSE |
| MARVELD1 | 7948 | 2.129025 | 0.033252 | 0.120891 | FALSE |
| AMT | 15159 | 2.128612 | 0.033286 | 0.120987 | FALSE |
| FUT11 | 9590 | 2.128417 | 0.033303 | 0.121018 | FALSE |
| DCAF8L2 | 12866 | 2.125794 | 0.03352 | 0.121708 | FALSE |
| EIF4G3 | 13513 | 2.125703 | 0.033528 | 0.121708 | FALSE |
| HNRNPA0 | 7804 | 2.125662 | 0.033531 | 0.121708 | FALSE |
| LRR1 | 991 | 2.124641 | 0.033617 | 0.121932 | FALSE |
| MED21 | 470 | 2.124176 | 0.033655 | 0.121976 | FALSE |
| EWSR1 | 5756 | 2.123418 | 0.033719 | 0.122068 | FALSE |
| DNAJA3 | 13796 | 2.123352 | 0.033724 | 0.122068 | FALSE |
| DENND4C | 7220 | 2.122968 | 0.033757 | 0.122156 | FALSE |
| ZNF429 | 2885 | 2.122154 | 0.033825 | 0.122262 | FALSE |
| DNAJB4 | 1322 | 2.121821 | 0.033853 | 0.122307 | FALSE |
| DEK | 2380 | 2.121069 | 0.033916 | 0.122448 | FALSE |
| PLEKHA5 | 14428 | 2.120542 | 0.03396 | 0.122525 | FALSE |
| RERG | 1659 | 2.120345 | 0.033977 | 0.122557 | FALSE |
| MFAP3L | 2560 | 2.119839 | 0.03402 | 0.122626 | FALSE |
| GNAL | 2051 | 2.11838 | 0.034143 | 0.122914 | FALSE |
| PAIP2 | 2203 | 2.118223 | 0.034156 | 0.12292 | FALSE |
| MTERF2 | 13094 | 2.118054 | 0.03417 | 0.122943 | FALSE |
| HSPB9 | 15326 | 2.116892 | 0.034269 | 0.123128 | FALSE |
| TRMT61B | 13023 | 2.116891 | 0.034269 | 0.123128 | FALSE |
| KCNE5 | 8676 | 2.116092 | 0.034337 | 0.123315 | FALSE |
| CCZ1 | 14248 | 2.115187 | 0.034414 | 0.123563 | FALSE |
| DPH7 | 899 | 2.114945 | 0.034435 | 0.123609 | FALSE |
| ZBED5 | 322 | 2.113298 | 0.034575 | 0.124 | FALSE |
| IGF2BP3 | 9305 | 2.112559 | 0.034639 | 0.124198 | FALSE |
| IER5L | 10560 | 2.111439 | 0.034735 | 0.124486 | FALSE |
| SPOP | 3043 | 2.110986 | 0.034774 | 0.124511 | FALSE |
| RAB11FIP5 | 1025 | 2.110812 | 0.034788 | 0.124511 | FALSE |
| PPP3CA | 6497 | 2.110805 | 0.034789 | 0.124511 | FALSE |
| KCNJ14 | 13100 | 2.110801 | 0.034789 | 0.124511 | FALSE |
| CORO2A | 15241 | 2.110578 | 0.034809 | 0.124522 | FALSE |
| CEP350 | 6055 | 2.109515 | 0.0349 | 0.124793 | FALSE |
| MTCL1 | 6963 | 2.109285 | 0.03492 | 0.124822 | FALSE |
| BCAS4 | 12213 | 2.108405 | 0.034996 | 0.125021 | FALSE |
| GLE1 | 6851 | 2.107995 | 0.035031 | 0.125119 | FALSE |
| VPS11 | 15049 | 2.107158 | 0.035104 | 0.125292 | FALSE |
| FAM120B | 2299 | 2.106968 | 0.03512 | 0.125294 | FALSE |
| ZNF200 | 11196 | 2.106685 | 0.035145 | 0.125353 | FALSE |
| ULK1 | 6272 | 2.106279 | 0.03518 | 0.125364 | FALSE |
| LURAP1L | 4587 | 2.106068 | 0.035198 | 0.1254 | FALSE |
| EIF4EBP2 | 11233 | 2.104884 | 0.035301 | 0.12571 | FALSE |
| ARHGEF10L | 1346 | 2.103916 | 0.035386 | 0.125982 | FALSE |
| SHPRH | 10778 | 2.102659 | 0.035496 | 0.126258 | FALSE |
| CREBBP | 6480 | 2.10238 | 0.03552 | 0.126293 | FALSE |
| PPP4R3B | 9570 | 2.102362 | 0.035522 | 0.126293 | FALSE |
| SKI | 14287 | 2.101636 | 0.035585 | 0.126461 | FALSE |
| PSMD6 | 9893 | 2.101545 | 0.035593 | 0.126461 | FALSE |
| PPP2R2C | 14844 | 2.100587 | 0.035677 | 0.126702 | FALSE |
| ULK3 | 10292 | 2.100111 | 0.035719 | 0.126744 | FALSE |
| BAIAP2-AS1 | 9076 | 2.100037 | 0.035726 | 0.126744 | FALSE |
| DIS3 | 466 | 2.098968 | 0.03582 | 0.126897 | FALSE |
| TAF8 | 6541 | 2.098762 | 0.035838 | 0.126897 | FALSE |
| HTR4 | 15173 | 2.09863 | 0.03585 | 0.126897 | FALSE |
| HELZ | 12776 | 2.098579 | 0.035854 | 0.126897 | FALSE |
| TMEM266 | 4150 | 2.097478 | 0.035951 | 0.127158 | FALSE |
| ZNF212 | 10898 | 2.09747 | 0.035952 | 0.127158 | FALSE |
| TMA16 | 12507 | 2.097332 | 0.035964 | 0.127172 | FALSE |
| PKD2L2 | 8140 | 2.096061 | 0.036077 | 0.127452 | FALSE |
| PROX1 | 10301 | 2.09526 | 0.036148 | 0.127545 | FALSE |
| FAM208B | 12178 | 2.095223 | 0.036151 | 0.127545 | FALSE |
| PPFIBP1 | 7411 | 2.094981 | 0.036173 | 0.127563 | FALSE |
| PHF2 | 1586 | 2.094752 | 0.036193 | 0.127606 | FALSE |
| ARL13B | 5213 | 2.094193 | 0.036243 | 0.127692 | FALSE |
| ANKRD16 | 4443 | 2.09315 | 0.036336 | 0.127965 | FALSE |
| FES | 5163 | 2.091925 | 0.036445 | 0.128322 | FALSE |
| ZFP91 | 15201 | 2.091384 | 0.036494 | 0.128377 | FALSE |
| GK5 | 9934 | 2.091113 | 0.036518 | 0.128433 | FALSE |
| DCAF16 | 6745 | 2.090481 | 0.036575 | 0.128511 | FALSE |
| ITCH | 15222 | 2.090382 | 0.036583 | 0.128511 | FALSE |
| BPTF | 10963 | 2.089864 | 0.03663 | 0.128625 | FALSE |
| KLRA1P | 2461 | 2.089767 | 0.036639 | 0.128626 | FALSE |
| CYLD | 10400 | 2.088903 | 0.036716 | 0.128841 | FALSE |
| LOC401320 | 5558 | 2.088208 | 0.036779 | 0.129025 | FALSE |
| AGK | 13747 | 2.087798 | 0.036816 | 0.129102 | FALSE |
| HNRNPUL2 | 12223 | 2.086904 | 0.036897 | 0.129295 | FALSE |
| GGT1 | 10904 | 2.086486 | 0.036935 | 0.129375 | FALSE |
| ABCD2 | 8949 | 2.085479 | 0.037026 | 0.12965 | FALSE |
| KLHL32 | 645 | 2.085436 | 0.03703 | 0.12965 | FALSE |
| FUNDC1 | 7642 | 2.084666 | 0.0371 | 0.129836 | FALSE |
| KRT10 | 6517 | 2.083889 | 0.03717 | 0.129967 | FALSE |
| MTOR | 5179 | 2.083719 | 0.037186 | 0.129974 | FALSE |
| KIT | 980 | 2.083684 | 0.037189 | 0.129974 | FALSE |
| RNF113A | 5758 | 2.083262 | 0.037227 | 0.130079 | FALSE |
| CA5B | 6549 | 2.083096 | 0.037242 | 0.130103 | FALSE |
| SAV1 | 12765 | 2.082752 | 0.037274 | 0.130184 | FALSE |
| ZNF781 | 8073 | 2.080729 | 0.037459 | 0.1308 | FALSE |
| MFAP3 | 12936 | 2.080172 | 0.03751 | 0.130949 | FALSE |
| PTAR1 | 8923 | 2.080024 | 0.037523 | 0.130967 | FALSE |
| TRPM7 | 13597 | 2.079854 | 0.037539 | 0.130992 | FALSE |
| TUG1 | 9051 | 2.079732 | 0.03755 | 0.131002 | FALSE |
| TRNP1 | 535 | 2.079552 | 0.037567 | 0.131011 | FALSE |
| CA12 | 14555 | 2.079083 | 0.03761 | 0.131122 | FALSE |
| ZNF44 | 1998 | 2.078837 | 0.037632 | 0.13113 | FALSE |
| POU6F2 | 14151 | 2.077835 | 0.037725 | 0.131376 | FALSE |
| HYDIN | 2180 | 2.076887 | 0.037812 | 0.131604 | FALSE |
| ZNF276 | 3531 | 2.076853 | 0.037815 | 0.131604 | FALSE |
| ZNF322 | 3815 | 2.076295 | 0.037867 | 0.131695 | FALSE |
| GRK3 | 9070 | 2.075992 | 0.037895 | 0.131763 | FALSE |
| FGF14 | 1907 | 2.075617 | 0.037929 | 0.131808 | FALSE |
| CACNA1E | 6847 | 2.07557 | 0.037934 | 0.131808 | FALSE |
| LINC00266-1 | 218 | 2.07549 | 0.037941 | 0.131808 | FALSE |
| ZNF142 | 325 | 2.075318 | 0.037957 | 0.131834 | FALSE |
| TEX261 | 15272 | 2.07407 | 0.038073 | 0.132086 | FALSE |
| MCM8 | 13186 | 2.073988 | 0.03808 | 0.132086 | FALSE |
| STARD13 | 12937 | 2.073698 | 0.038107 | 0.13215 | FALSE |
| ANKRD18B | 12049 | 2.073075 | 0.038165 | 0.132254 | FALSE |
| PHC2 | 5617 | 2.072185 | 0.038248 | 0.132433 | FALSE |
| FLT1 | 11576 | 2.071564 | 0.038306 | 0.132566 | FALSE |
| EBAG9 | 6993 | 2.0715 | 0.038312 | 0.132566 | FALSE |
| UBL7-AS1 | 9482 | 2.07125 | 0.038335 | 0.132572 | FALSE |
| SPOPL | 5740 | 2.071118 | 0.038348 | 0.132572 | FALSE |
| HOMER2 | 14153 | 2.071 | 0.038359 | 0.132581 | FALSE |
| FAM193B | 8030 | 2.070668 | 0.03839 | 0.132659 | FALSE |
| ATAD2B | 14374 | 2.070221 | 0.038432 | 0.132686 | FALSE |
| MST1R | 683 | 2.069915 | 0.03846 | 0.132756 | FALSE |
| TMEM69 | 6193 | 2.069225 | 0.038525 | 0.132949 | FALSE |
| RHOBTB2 | 2296 | 2.068722 | 0.038572 | 0.133054 | FALSE |
| CEMIP | 14536 | 2.068287 | 0.038613 | 0.133136 | FALSE |
| LGI3 | 13676 | 2.068165 | 0.038625 | 0.133146 | FALSE |
| TNPO1 | 4410 | 2.067802 | 0.038659 | 0.133183 | FALSE |
| PRKAB1 | 8380 | 2.067778 | 0.038661 | 0.133183 | FALSE |
| ADCY8 | 7444 | 2.066276 | 0.038802 | 0.133612 | FALSE |
| NOS2 | 1889 | 2.066108 | 0.038818 | 0.133637 | FALSE |
| IL6ST | 8233 | 2.064509 | 0.038969 | 0.13404 | FALSE |
| HENMT1 | 12140 | 2.064326 | 0.038987 | 0.13407 | FALSE |
| G3BP1 | 6814 | 2.063488 | 0.039066 | 0.134313 | FALSE |
| AKAP10 | 12767 | 2.060883 | 0.039314 | 0.135047 | FALSE |
| STH | 14098 | 2.060099 | 0.039389 | 0.135274 | FALSE |
| ANKRD42 | 12854 | 2.05898 | 0.039496 | 0.13553 | FALSE |
| SLC12A8 | 5264 | 2.058958 | 0.039498 | 0.13553 | FALSE |
| WHRN | 11303 | 2.057369 | 0.039651 | 0.135875 | FALSE |
| GATAD1 | 14530 | 2.057005 | 0.039686 | 0.135935 | FALSE |
| UBE2H | 15167 | 2.056335 | 0.03975 | 0.136096 | FALSE |
| GGT7 | 11453 | 2.054755 | 0.039903 | 0.136465 | FALSE |
| PPP4R2 | 9740 | 2.054677 | 0.03991 | 0.136465 | FALSE |
| MYC | 8812 | 2.053459 | 0.040028 | 0.136748 | FALSE |
| FTSJ3 | 6355 | 2.053355 | 0.040038 | 0.136749 | FALSE |
| NOL10 | 2310 | 2.053277 | 0.040046 | 0.136749 | FALSE |
| PEX1 | 3891 | 2.053018 | 0.040071 | 0.13675 | FALSE |
| TAS2R50 | 11895 | 2.052898 | 0.040082 | 0.13675 | FALSE |
| NTM | 896 | 2.052765 | 0.040095 | 0.13675 | FALSE |
| PILRA | 419 | 2.052756 | 0.040096 | 0.13675 | FALSE |
| GADD45A | 6475 | 2.052731 | 0.040099 | 0.13675 | FALSE |
| PDPN | 6144 | 2.052563 | 0.040115 | 0.136764 | FALSE |
| RNF212 | 6651 | 2.052443 | 0.040127 | 0.136764 | FALSE |
| ISPD | 11827 | 2.052227 | 0.040148 | 0.136768 | FALSE |
| PHACTR4 | 9397 | 2.050555 | 0.04031 | 0.137202 | FALSE |
| FBLN7 | 3430 | 2.04958 | 0.040405 | 0.13736 | FALSE |
| TAF2 | 15334 | 2.049541 | 0.040409 | 0.13736 | FALSE |
| MARK3 | 14769 | 2.048345 | 0.040526 | 0.137654 | FALSE |
| CLK2P1 | 5443 | 2.046912 | 0.040667 | 0.138025 | FALSE |
| AFF1 | 8452 | 2.046666 | 0.040691 | 0.138051 | FALSE |
| PHKA2 | 14662 | 2.046655 | 0.040692 | 0.138051 | FALSE |
| BRINP1 | 4193 | 2.045247 | 0.040831 | 0.138341 | FALSE |
| HIBADH | 10887 | 2.044706 | 0.040884 | 0.138401 | FALSE |
| RABGEF1 | 4758 | 2.043877 | 0.040966 | 0.138558 | FALSE |
| AMBRA1 | 5390 | 2.043096 | 0.041043 | 0.138746 | FALSE |
| CLASP2 | 1100 | 2.042999 | 0.041053 | 0.138746 | FALSE |
| ZNF257 | 7570 | 2.042956 | 0.041057 | 0.138746 | FALSE |
| AP4E1 | 6940 | 2.042379 | 0.041114 | 0.13891 | FALSE |
| BHLHE41 | 94 | 2.041376 | 0.041213 | 0.139187 | FALSE |
| SLC45A4 | 8266 | 2.04105 | 0.041246 | 0.139217 | FALSE |
| CCP110 | 7030 | 2.039638 | 0.041386 | 0.139619 | FALSE |
| STAG3L4 | 8805 | 2.038945 | 0.041456 | 0.139759 | FALSE |
| FAM13A-AS1 | 7097 | 2.038872 | 0.041463 | 0.139759 | FALSE |
| NDUFC1 | 1417 | 2.038799 | 0.04147 | 0.139759 | FALSE |
| TMEM170B | 12535 | 2.038774 | 0.041473 | 0.139759 | FALSE |
| TXNDC15 | 6530 | 2.037776 | 0.041572 | 0.140065 | FALSE |
| BOLA2-SMG1P6 | 15251 | 2.035551 | 0.041795 | 0.140688 | FALSE |
| UXS1 | 12344 | 2.035146 | 0.041836 | 0.140711 | FALSE |
| ZKSCAN5 | 12842 | 2.03494 | 0.041857 | 0.140751 | FALSE |
| IL6R | 15479 | 2.033627 | 0.041989 | 0.141134 | FALSE |
| ZCCHC2 | 14283 | 2.033222 | 0.04203 | 0.141211 | FALSE |
| TMEM145 | 15535 | 2.032796 | 0.042073 | 0.141326 | FALSE |
| NKTR | 10933 | 2.031801 | 0.042174 | 0.141512 | FALSE |
| BAK1 | 11726 | 2.031046 | 0.04225 | 0.141738 | FALSE |
| STAM | 11073 | 2.029213 | 0.042437 | 0.142258 | FALSE |
| TRPM3 | 15116 | 2.028659 | 0.042493 | 0.142342 | FALSE |
| CCDC144B | 3944 | 2.028649 | 0.042494 | 0.142342 | FALSE |
| NOLC1 | 2170 | 2.028321 | 0.042527 | 0.142423 | FALSE |
| MOGAT3 | 9010 | 2.027503 | 0.042611 | 0.142672 | FALSE |
| STAMBP | 4841 | 2.025782 | 0.042787 | 0.143113 | FALSE |
| ZFP82 | 11171 | 2.025769 | 0.042788 | 0.143113 | FALSE |
| PKNOX2 | 6343 | 2.02466 | 0.042902 | 0.143318 | FALSE |
| MYO19 | 15532 | 2.024174 | 0.042952 | 0.143446 | FALSE |
| ZNF324B | 6602 | 2.02407 | 0.042963 | 0.143451 | FALSE |
| SLC3A1 | 12657 | 2.023635 | 0.043008 | 0.143509 | FALSE |
| WFDC10A | 5188 | 2.023321 | 0.04304 | 0.143545 | FALSE |
| VCX2 | 6321 | 2.02316 | 0.043057 | 0.143545 | FALSE |
| MAP1LC3B2 | 2042 | 2.023124 | 0.04306 | 0.143545 | FALSE |
| STX1B | 4026 | 2.023061 | 0.043067 | 0.143545 | FALSE |
| ADAMTS5 | 9452 | 2.022827 | 0.043091 | 0.143572 | FALSE |
| KIAA0586 | 5928 | 2.022268 | 0.043149 | 0.14363 | FALSE |
| SNRNP48 | 6737 | 2.022196 | 0.043156 | 0.14363 | FALSE |
| TARSL2 | 8189 | 2.022124 | 0.043164 | 0.14363 | FALSE |
| LAMP5 | 11504 | 2.021932 | 0.043183 | 0.143664 | FALSE |
| MON2 | 11286 | 2.021436 | 0.043235 | 0.143745 | FALSE |
| STAC2 | 8282 | 2.021259 | 0.043253 | 0.143775 | FALSE |
| IPO7 | 6384 | 2.020357 | 0.043346 | 0.144024 | FALSE |
| ZNF655 | 15198 | 2.019871 | 0.043397 | 0.1441 | FALSE |
| C21orf62-AS1 | 10070 | 2.018107 | 0.04358 | 0.144494 | FALSE |
| TUBGCP3 | 11784 | 2.017117 | 0.043683 | 0.144744 | FALSE |
| UTRN | 9824 | 2.016819 | 0.043714 | 0.144785 | FALSE |
| RANGAP1 | 5320 | 2.016349 | 0.043763 | 0.144887 | FALSE |
| ANTXR1 | 1304 | 2.015952 | 0.043805 | 0.144935 | FALSE |
| TFDP1 | 3193 | 2.015682 | 0.043833 | 0.144995 | FALSE |
| DPY19L2 | 11698 | 2.014493 | 0.043958 | 0.145315 | FALSE |
| PAN2 | 1867 | 2.013459 | 0.044066 | 0.145643 | FALSE |
| APPBP2 | 5385 | 2.012737 | 0.044142 | 0.145832 | FALSE |
| SLFN11 | 2636 | 2.012453 | 0.044172 | 0.1459 | FALSE |
| RBM4B | 8290 | 2.012242 | 0.044194 | 0.145942 | FALSE |
| AGAP11 | 5335 | 2.01189 | 0.044232 | 0.146034 | FALSE |
| ZNF768 | 7110 | 2.011723 | 0.044249 | 0.146059 | FALSE |
| MBP | 5869 | 2.011642 | 0.044258 | 0.146059 | FALSE |
| EPC2 | 15260 | 2.011326 | 0.044291 | 0.146107 | FALSE |
| MTR | 13026 | 2.01032 | 0.044397 | 0.146427 | FALSE |
| LOC100132057 | 3187 | 2.010174 | 0.044413 | 0.146447 | FALSE |
| SPAG8 | 6425 | 2.009134 | 0.044523 | 0.146748 | FALSE |
| DHX40 | 11015 | 2.008823 | 0.044556 | 0.146814 | FALSE |
| DIDO1 | 6001 | 2.008769 | 0.044562 | 0.146814 | FALSE |
| RABEP2 | 3911 | 2.008237 | 0.044618 | 0.146891 | FALSE |
| PREP | 10501 | 2.00819 | 0.044623 | 0.146891 | FALSE |
| NAGPA | 12941 | 2.008006 | 0.044643 | 0.146891 | FALSE |
| WDPCP | 6733 | 2.007753 | 0.04467 | 0.146891 | FALSE |
| FGD5-AS1 | 15132 | 2.007396 | 0.044708 | 0.146985 | FALSE |
| N4BP2L1 | 5311 | 2.006917 | 0.044759 | 0.147121 | FALSE |
| RIMS2 | 9648 | 2.005849 | 0.044872 | 0.147392 | FALSE |
| SACS | 8835 | 2.005587 | 0.0449 | 0.147433 | FALSE |
| CLIC2 | 14257 | 2.005316 | 0.044929 | 0.147497 | FALSE |
| EFNA5 | 7491 | 2.003938 | 0.045077 | 0.14795 | FALSE |
| IRF2BP2 | 3682 | 2.002873 | 0.045191 | 0.14815 | FALSE |
| DNAJC22 | 6547 | 2.002838 | 0.045195 | 0.14815 | FALSE |
| TRAK2 | 3309 | 2.002375 | 0.045244 | 0.14822 | FALSE |
| ARRDC3 | 8621 | 2.002283 | 0.045254 | 0.148221 | FALSE |
| WASHC2A | 6035 | 2.00171 | 0.045316 | 0.148392 | FALSE |
| PUM2 | 4328 | 2.0015 | 0.045339 | 0.148435 | FALSE |
| CCDC181 | 14116 | 2.000244 | 0.045474 | 0.148787 | FALSE |
| MYT1L | 1767 | 2.000239 | 0.045474 | 0.148787 | FALSE |
| GPR149 | 10629 | 2.000132 | 0.045486 | 0.148793 | FALSE |
| OPN3 | 9501 | 1.999962 | 0.045504 | 0.148822 | FALSE |
| DCAF10 | 4993 | 1.997996 | 0.045717 | 0.149299 | FALSE |
| CEP250 | 5143 | 1.996476 | 0.045882 | 0.149682 | FALSE |
| NPR3 | 9937 | 1.995003 | 0.046043 | 0.150147 | FALSE |
| CSNK1G3 | 8287 | 1.994903 | 0.046053 | 0.150147 | FALSE |
| RAD54L2 | 243 | 1.994375 | 0.046111 | 0.150264 | FALSE |
| UEVLD | 14504 | 1.993142 | 0.046246 | 0.150554 | FALSE |
| ZBTB5 | 7099 | 1.992756 | 0.046288 | 0.150629 | FALSE |
| PRDM4 | 3579 | 1.99167 | 0.046407 | 0.150985 | FALSE |
| ATP8A2 | 3903 | 1.991132 | 0.046466 | 0.151146 | FALSE |
| PCDHB11 | 85 | 1.990565 | 0.046529 | 0.151266 | FALSE |
| GOLT1B | 396 | 1.990387 | 0.046548 | 0.151287 | FALSE |
| BCL7B | 1061 | 1.990236 | 0.046565 | 0.151309 | FALSE |
| LEF1 | 6443 | 1.990064 | 0.046584 | 0.15134 | FALSE |
| PCID2 | 10482 | 1.988406 | 0.046767 | 0.15165 | FALSE |
| METTL2A | 2323 | 1.987811 | 0.046833 | 0.151801 | FALSE |
| FAAH2 | 4990 | 1.987597 | 0.046856 | 0.151846 | FALSE |
| CISD2 | 8538 | 1.987034 | 0.046919 | 0.152016 | FALSE |
| FOXN3 | 6339 | 1.986675 | 0.046958 | 0.152085 | FALSE |
| TANC2 | 7627 | 1.986623 | 0.046964 | 0.152085 | FALSE |
| AFG3L2 | 13468 | 1.985551 | 0.047083 | 0.15239 | FALSE |
| SP1 | 12189 | 1.985468 | 0.047092 | 0.15239 | FALSE |
| PPIL2 | 14909 | 1.9836 | 0.0473 | 0.152936 | FALSE |
| EIF4E | 6182 | 1.982916 | 0.047377 | 0.153152 | FALSE |
| TMEM19 | 9491 | 1.982815 | 0.047388 | 0.153157 | FALSE |
| NIFK | 5557 | 1.981931 | 0.047487 | 0.153363 | FALSE |
| GLB1 | 15481 | 1.981919 | 0.047488 | 0.153363 | FALSE |
| ZNF503 | 5771 | 1.981894 | 0.047491 | 0.153363 | FALSE |
| TTC14 | 2549 | 1.981004 | 0.047591 | 0.153653 | FALSE |
| MFSD8 | 15088 | 1.980632 | 0.047633 | 0.153724 | FALSE |
| SAAL1 | 8016 | 1.980029 | 0.0477 | 0.153847 | FALSE |
| WWP2 | 10323 | 1.979393 | 0.047772 | 0.153946 | FALSE |
| CASP3 | 12290 | 1.979392 | 0.047772 | 0.153946 | FALSE |
| PAPOLG | 9845 | 1.979358 | 0.047776 | 0.153946 | FALSE |
| HERC2P11 | 2305 | 1.979264 | 0.047786 | 0.153946 | FALSE |
| SOCS4 | 3990 | 1.979233 | 0.04779 | 0.153946 | FALSE |
| EMC3-AS1 | 6107 | 1.978338 | 0.047891 | 0.154175 | FALSE |
| SLC6A8 | 9863 | 1.978027 | 0.047926 | 0.154244 | FALSE |
| MPHOSPH8 | 5744 | 1.977467 | 0.047989 | 0.154301 | FALSE |
| EGR1 | 9505 | 1.976948 | 0.048048 | 0.154457 | FALSE |
| DTX2P1-UPK3BP1-PMS2P11 | 9536 | 1.976598 | 0.048087 | 0.154553 | FALSE |
| HNRNPF | 9401 | 1.975737 | 0.048185 | 0.154803 | FALSE |
| SMOC2 | 6983 | 1.975481 | 0.048214 | 0.154841 | FALSE |
| ASB7 | 2520 | 1.975457 | 0.048216 | 0.154841 | FALSE |
| PLXNA2 | 640 | 1.974772 | 0.048294 | 0.154963 | FALSE |
| CCDC146 | 4243 | 1.974234 | 0.048355 | 0.155064 | FALSE |
| PLEKHM1P1 | 10461 | 1.973393 | 0.048451 | 0.155253 | FALSE |
| PDCL | 9717 | 1.973327 | 0.048458 | 0.155253 | FALSE |
| CCAR2 | 8349 | 1.973279 | 0.048464 | 0.155253 | FALSE |
| EIF2AK1 | 14456 | 1.972126 | 0.048595 | 0.15561 | FALSE |
| ZNRD1ASP | 207 | 1.971294 | 0.04869 | 0.155883 | FALSE |
| PKD2 | 121 | 1.969866 | 0.048854 | 0.15631 | FALSE |
| RBM26-AS1 | 9377 | 1.969196 | 0.048931 | 0.15646 | FALSE |
| PITRM1 | 7954 | 1.969195 | 0.048931 | 0.15646 | FALSE |
| MIA2 | 3046 | 1.967195 | 0.049161 | 0.157097 | FALSE |
| GATAD2B | 234 | 1.966513 | 0.049239 | 0.157222 | FALSE |
| ELOVL7 | 12538 | 1.966182 | 0.049278 | 0.15728 | FALSE |
| KIAA1109 | 1195 | 1.964403 | 0.049483 | 0.15773 | FALSE |
| HAR1A | 3178 | 1.964178 | 0.049509 | 0.15773 | FALSE |
| ZNF620 | 2028 | 1.962823 | 0.049667 | 0.158006 | FALSE |
| CNTFR | 10651 | 1.962424 | 0.049713 | 0.15806 | FALSE |
| RAB14 | 9006 | 1.961816 | 0.049784 | 0.158125 | FALSE |
| HYLS1 | 6295 | 1.961785 | 0.049788 | 0.158125 | FALSE |
| CTNNA1 | 12218 | 1.961653 | 0.049803 | 0.158125 | FALSE |
| PPWD1 | 15256 | 1.961631 | 0.049805 | 0.158125 | FALSE |
| TRPM2 | 15089 | 1.961514 | 0.049819 | 0.158136 | FALSE |
| SPART | 1836 | 1.961146 | 0.049862 | 0.15824 | FALSE |
| LONRF3 | 5220 | 1.960679 | 0.049916 | 0.158381 | FALSE |
| AMH | 14142 | 1.960292 | 0.049962 | 0.15848 | FALSE |
| SGPL1 | 13843 | 1.959199 | 0.050089 | 0.158801 | FALSE |
| NFATC2IP | 6509 | 1.958877 | 0.050127 | 0.158841 | FALSE |
| NUDT12 | 4241 | 1.958283 | 0.050197 | 0.158948 | FALSE |
| MSL3 | 13845 | 1.957847 | 0.050248 | 0.159078 | FALSE |
| NT5C2 | 10828 | 1.957269 | 0.050316 | 0.159228 | FALSE |
| INO80 | 12277 | 1.955856 | 0.050482 | 0.159624 | FALSE |
| ALKBH8 | 7102 | 1.954403 | 0.050654 | 0.159887 | FALSE |
| SLC12A5 | 14717 | 1.954287 | 0.050667 | 0.159887 | FALSE |
| DDX23 | 11417 | 1.952398 | 0.050891 | 0.160496 | FALSE |
| FNBP4 | 4733 | 1.951818 | 0.05096 | 0.160588 | FALSE |
| SIAH2 | 964 | 1.951806 | 0.050961 | 0.160588 | FALSE |
| GNL1 | 15125 | 1.950982 | 0.051059 | 0.16086 | FALSE |
| DPY19L2P2 | 8335 | 1.950906 | 0.051068 | 0.16086 | FALSE |
| SPIB | 12078 | 1.950644 | 0.051099 | 0.160916 | FALSE |
| RIMS4 | 12654 | 1.950583 | 0.051107 | 0.160916 | FALSE |
| PAX8 | 74 | 1.949787 | 0.051202 | 0.161118 | FALSE |
| TM9SF3 | 10544 | 1.949114 | 0.051282 | 0.161306 | FALSE |
| DGKI | 15610 | 1.948105 | 0.051402 | 0.161603 | FALSE |
| ULK4 | 707 | 1.947177 | 0.051514 | 0.161904 | FALSE |
| SPEN | 15487 | 1.946896 | 0.051547 | 0.161945 | FALSE |
| ABL2 | 14811 | 1.946218 | 0.051629 | 0.162135 | FALSE |
| GCFC2 | 13665 | 1.945493 | 0.051716 | 0.162311 | FALSE |
| CCDC91 | 9479 | 1.944474 | 0.051838 | 0.1626 | FALSE |
| GJC1 | 12286 | 1.943997 | 0.051896 | 0.16274 | FALSE |
| SHMT1 | 13501 | 1.943273 | 0.051983 | 0.162954 | FALSE |
| SNED1 | 8229 | 1.94125 | 0.052228 | 0.16359 | FALSE |
| RPRML | 2950 | 1.941078 | 0.052249 | 0.163597 | FALSE |
| SLC7A5 | 14284 | 1.941061 | 0.052251 | 0.163597 | FALSE |
| DUS1L | 1581 | 1.940294 | 0.052344 | 0.163855 | FALSE |
| ARIH1 | 15274 | 1.939747 | 0.05241 | 0.164031 | FALSE |
| SCAI | 14463 | 1.93937 | 0.052456 | 0.164141 | FALSE |
| KANSL2 | 14606 | 1.939144 | 0.052484 | 0.164194 | FALSE |
| BTN2A2 | 3603 | 1.9387 | 0.052538 | 0.164331 | FALSE |
| DNAJC18 | 15449 | 1.938208 | 0.052598 | 0.164485 | FALSE |
| IFNAR2 | 11838 | 1.93788 | 0.052638 | 0.164578 | FALSE |
| ZMYM4 | 2370 | 1.937735 | 0.052656 | 0.1646 | FALSE |
| HEATR3 | 7706 | 1.936829 | 0.052766 | 0.16488 | FALSE |
| DPY19L2P3 | 11609 | 1.936339 | 0.052826 | 0.165004 | FALSE |
| LHX4-AS1 | 7090 | 1.936333 | 0.052827 | 0.165004 | FALSE |
| PRSS56 | 14884 | 1.935888 | 0.052881 | 0.165141 | FALSE |
| RPS6KB2 | 9650 | 1.93484 | 0.05301 | 0.165509 | FALSE |
| SHANK3 | 9666 | 1.934444 | 0.053059 | 0.165628 | FALSE |
| TTI2 | 1383 | 1.934083 | 0.053103 | 0.1657 | FALSE |
| COX10 | 3795 | 1.932793 | 0.053262 | 0.166096 | FALSE |
| NAP1L2 | 10563 | 1.931726 | 0.053393 | 0.166473 | FALSE |
| ZNF492 | 12849 | 1.931574 | 0.053412 | 0.166497 | FALSE |
| ZDHHC18 | 4723 | 1.931245 | 0.053453 | 0.166559 | FALSE |
| ABCA5 | 15257 | 1.930955 | 0.053489 | 0.166638 | FALSE |
| SATB2 | 15402 | 1.930215 | 0.05358 | 0.166808 | FALSE |
| SLC17A5 | 7177 | 1.928824 | 0.053753 | 0.167127 | FALSE |
| STAG2 | 919 | 1.92833 | 0.053814 | 0.167285 | FALSE |
| ZNF660 | 3540 | 1.92811 | 0.053841 | 0.167301 | FALSE |
| GRK5 | 6173 | 1.928034 | 0.053851 | 0.167301 | FALSE |
| C12orf54 | 9072 | 1.927945 | 0.053862 | 0.167301 | FALSE |
| PURB | 14757 | 1.927856 | 0.053873 | 0.167302 | FALSE |
| ZMYND8 | 7233 | 1.927666 | 0.053897 | 0.16732 | FALSE |
| SLCO3A1 | 6345 | 1.927551 | 0.053911 | 0.16732 | FALSE |
| ZBTB40 | 3840 | 1.926553 | 0.054035 | 0.167462 | FALSE |
| ABHD6 | 1008 | 1.925899 | 0.054117 | 0.16748 | FALSE |
| MED23 | 12884 | 1.925785 | 0.054131 | 0.16748 | FALSE |
| SNX1 | 14509 | 1.924913 | 0.05424 | 0.167676 | FALSE |
| CD209 | 8072 | 1.922996 | 0.054481 | 0.168319 | FALSE |
| CEP104 | 10953 | 1.922762 | 0.05451 | 0.168343 | FALSE |
| SLC39A7 | 10994 | 1.922573 | 0.054534 | 0.16835 | FALSE |
| ATP2A2 | 1000 | 1.922198 | 0.054581 | 0.168396 | FALSE |
| TRAPPC10 | 13294 | 1.921658 | 0.054649 | 0.168572 | FALSE |
| RADIL | 3130 | 1.920941 | 0.054739 | 0.168784 | FALSE |
| SLC35F3 | 6484 | 1.920722 | 0.054767 | 0.168789 | FALSE |
| GABRB2 | 7153 | 1.920632 | 0.054778 | 0.168789 | FALSE |
| LIN7A | 15531 | 1.919622 | 0.054906 | 0.169064 | FALSE |
| EHMT2 | 8746 | 1.919056 | 0.054977 | 0.169218 | FALSE |
| THAP2 | 14634 | 1.918885 | 0.054999 | 0.169218 | FALSE |
| TGFBRAP1 | 7399 | 1.918003 | 0.055111 | 0.169529 | FALSE |
| N4BP2 | 4971 | 1.917906 | 0.055123 | 0.169533 | FALSE |
| TRIM33 | 231 | 1.917571 | 0.055165 | 0.16963 | FALSE |
| SMC1A | 866 | 1.915748 | 0.055397 | 0.170109 | FALSE |
| BET1 | 13070 | 1.914133 | 0.055603 | 0.170528 | FALSE |
| TAS2R31 | 4964 | 1.910904 | 0.056017 | 0.171641 | FALSE |
| DENND4A | 1919 | 1.910206 | 0.056107 | 0.171882 | FALSE |
| GRIN2A | 7440 | 1.910043 | 0.056128 | 0.171913 | FALSE |
| NUP153 | 3301 | 1.90865 | 0.056307 | 0.172409 | FALSE |
| TNNC2 | 12209 | 1.908616 | 0.056312 | 0.172409 | FALSE |
| ADAM20 | 14679 | 1.908356 | 0.056345 | 0.172444 | FALSE |
| CRTAP | 13143 | 1.908258 | 0.056358 | 0.172448 | FALSE |
| UGDH | 5294 | 1.908175 | 0.056369 | 0.172448 | FALSE |
| PPP1R3F | 5371 | 1.907926 | 0.056401 | 0.172513 | FALSE |
| PAQR8 | 15415 | 1.90737 | 0.056473 | 0.172699 | FALSE |
| ZNF317 | 11003 | 1.907168 | 0.056499 | 0.172745 | FALSE |
| PRKAB2 | 7146 | 1.906795 | 0.056547 | 0.172849 | FALSE |
| RYR2 | 14538 | 1.906734 | 0.056555 | 0.172849 | FALSE |
| ITGA9-AS1 | 3380 | 1.906237 | 0.056619 | 0.172945 | FALSE |
| ARSG | 15323 | 1.906095 | 0.056638 | 0.172967 | FALSE |
| NCOA2 | 5370 | 1.905552 | 0.056708 | 0.173081 | FALSE |
| SLC9A3R2 | 7138 | 1.90532 | 0.056738 | 0.173118 | FALSE |
| MPZL3 | 5395 | 1.905288 | 0.056743 | 0.173118 | FALSE |
| HKR1 | 7553 | 1.905049 | 0.056774 | 0.173158 | FALSE |
| EIF2S3 | 8802 | 1.905018 | 0.056778 | 0.173158 | FALSE |
| FLRT3 | 3826 | 1.903919 | 0.056921 | 0.173437 | FALSE |
| GRSF1 | 15314 | 1.903123 | 0.057024 | 0.173605 | FALSE |
| LAMP2 | 4364 | 1.902729 | 0.057076 | 0.173694 | FALSE |
| TSC22D1 | 8920 | 1.902457 | 0.057111 | 0.173735 | FALSE |
| LAMA4 | 14936 | 1.90216 | 0.05715 | 0.173819 | FALSE |
| AKAP17A | 12828 | 1.901506 | 0.057236 | 0.174011 | FALSE |
| ZBTB7C | 7296 | 1.900478 | 0.05737 | 0.174319 | FALSE |
| TRABD | 6533 | 1.899267 | 0.057529 | 0.1747 | FALSE |
| HIPK1 | 12170 | 1.899008 | 0.057563 | 0.174736 | FALSE |
| MAFB | 4172 | 1.898906 | 0.057577 | 0.174743 | FALSE |
| CSPP1 | 18 | 1.89815 | 0.057676 | 0.174943 | FALSE |
| ACLY | 15126 | 1.897323 | 0.057785 | 0.175239 | FALSE |
| RAD52 | 14931 | 1.895133 | 0.058075 | 0.175912 | FALSE |
| OGFOD3 | 15368 | 1.893807 | 0.058251 | 0.176342 | FALSE |
| MAP2K3 | 445 | 1.892328 | 0.058447 | 0.176801 | FALSE |
| DNAJC16 | 6097 | 1.892108 | 0.058477 | 0.176814 | FALSE |
| CRLS1 | 7013 | 1.892 | 0.058491 | 0.176814 | FALSE |
| KIAA0895L | 338 | 1.891759 | 0.058523 | 0.176814 | FALSE |
| ZFP69B | 6697 | 1.8917 | 0.058531 | 0.176814 | FALSE |
| USP9Y | 2153 | 1.890605 | 0.058677 | 0.177015 | FALSE |
| FBXO46 | 3115 | 1.890524 | 0.058688 | 0.177015 | FALSE |
| MYBBP1A | 8711 | 1.890375 | 0.058708 | 0.177041 | FALSE |
| BAHCC1 | 14537 | 1.890235 | 0.058727 | 0.177063 | FALSE |
| DRD1 | 15289 | 1.88894 | 0.0589 | 0.177444 | FALSE |
| C8orf31 | 15607 | 1.888865 | 0.05891 | 0.177444 | FALSE |
| CGN | 14221 | 1.888698 | 0.058932 | 0.177444 | FALSE |
| GCLM | 5529 | 1.88839 | 0.058974 | 0.177534 | FALSE |
| CKMT2-AS1 | 1104 | 1.887747 | 0.05906 | 0.177735 | FALSE |
| G3BP2 | 12958 | 1.887723 | 0.059063 | 0.177735 | FALSE |
| PSMC5 | 13810 | 1.887534 | 0.059089 | 0.177743 | FALSE |
| ADAM15 | 11832 | 1.887314 | 0.059118 | 0.177798 | FALSE |
| PCDHB2 | 3258 | 1.886866 | 0.059178 | 0.177876 | FALSE |
| CUL1 | 7631 | 1.886105 | 0.059281 | 0.178084 | FALSE |
| CLASRP | 13284 | 1.886098 | 0.059282 | 0.178084 | FALSE |
| ZNHIT6 | 14913 | 1.884868 | 0.059448 | 0.178462 | FALSE |
| TRIM32 | 8244 | 1.884828 | 0.059453 | 0.178462 | FALSE |
| ARL4A | 11605 | 1.884732 | 0.059466 | 0.178467 | FALSE |
| ZNF543 | 13646 | 1.884286 | 0.059526 | 0.178613 | FALSE |
| TP53INP2 | 3044 | 1.883903 | 0.059578 | 0.178666 | FALSE |
| NAA15 | 1377 | 1.88287 | 0.059718 | 0.178982 | FALSE |
| NSRP1 | 6907 | 1.88268 | 0.059744 | 0.179025 | FALSE |
| CSTF2 | 5531 | 1.881522 | 0.059901 | 0.179359 | FALSE |
| KIAA1324L | 7162 | 1.879995 | 0.060109 | 0.179706 | FALSE |
| ODR4 | 5391 | 1.878291 | 0.060341 | 0.180282 | FALSE |
| ZNF32 | 6577 | 1.878213 | 0.060352 | 0.180282 | FALSE |
| EOGT | 10759 | 1.877992 | 0.060382 | 0.180282 | FALSE |
| RAI1 | 12909 | 1.877576 | 0.060439 | 0.180383 | FALSE |
| CARNMT1 | 3752 | 1.876441 | 0.060595 | 0.180675 | FALSE |
| KRT222 | 76 | 1.876144 | 0.060636 | 0.180754 | FALSE |
| MAP3K20 | 1280 | 1.875252 | 0.060758 | 0.181024 | FALSE |
| ADRB1 | 3468 | 1.875032 | 0.060788 | 0.181061 | FALSE |
| ATP1A3 | 8011 | 1.874992 | 0.060794 | 0.181061 | FALSE |
| STXBP1 | 2842 | 1.874796 | 0.060821 | 0.181107 | FALSE |
| CELSR2 | 5936 | 1.874026 | 0.060927 | 0.181319 | FALSE |
| ZNF595 | 10383 | 1.873567 | 0.06099 | 0.181473 | FALSE |
| CSNK1G1 | 9903 | 1.873459 | 0.061005 | 0.181483 | FALSE |
| TMEM169 | 469 | 1.873034 | 0.061064 | 0.181528 | FALSE |
| UBE3C | 11736 | 1.872929 | 0.061078 | 0.181528 | FALSE |
| ZNRF2P1 | 5655 | 1.87237 | 0.061155 | 0.181654 | FALSE |
| YLPM1 | 4933 | 1.871203 | 0.061317 | 0.18203 | FALSE |
| IPO5 | 3860 | 1.870912 | 0.061357 | 0.18209 | FALSE |
| EVI5 | 6174 | 1.870888 | 0.061361 | 0.18209 | FALSE |
| ZNF621 | 5887 | 1.870232 | 0.061452 | 0.182325 | FALSE |
| ANKMY2 | 15515 | 1.868505 | 0.061692 | 0.18283 | FALSE |
| CCDC130 | 9201 | 1.868187 | 0.061736 | 0.182857 | FALSE |
| BBS7 | 999 | 1.867299 | 0.06186 | 0.183119 | FALSE |
| PCDHB9 | 10992 | 1.865782 | 0.062072 | 0.183496 | FALSE |
| AR | 559 | 1.865648 | 0.062091 | 0.183496 | FALSE |
| JOSD1 | 4792 | 1.865465 | 0.062116 | 0.183496 | FALSE |
| TMEM229A | 5442 | 1.864383 | 0.062268 | 0.18391 | FALSE |
| PPM1D | 9476 | 1.863897 | 0.062336 | 0.184077 | FALSE |
| PTPN21 | 7716 | 1.863538 | 0.062387 | 0.184171 | FALSE |
| STX16 | 658 | 1.863502 | 0.062392 | 0.184171 | FALSE |
| RHOBTB1 | 12687 | 1.863304 | 0.06242 | 0.184183 | FALSE |
| NCK2 | 520 | 1.862306 | 0.06256 | 0.184528 | FALSE |
| AMY2A | 14527 | 1.861493 | 0.062675 | 0.184692 | FALSE |
| C9orf3 | 7218 | 1.860951 | 0.062751 | 0.184883 | FALSE |
| EIF4A2 | 14957 | 1.860574 | 0.062804 | 0.185005 | FALSE |
| RTEL1 | 4255 | 1.859734 | 0.062923 | 0.185207 | FALSE |
| TBC1D8B | 10960 | 1.859523 | 0.062953 | 0.185207 | FALSE |
| PACSIN2 | 7870 | 1.859502 | 0.062956 | 0.185207 | FALSE |
| LINC01105 | 10742 | 1.859128 | 0.063009 | 0.185266 | FALSE |
| ZNF404 | 4876 | 1.859111 | 0.063011 | 0.185266 | FALSE |
| F2RL1 | 13675 | 1.858476 | 0.063101 | 0.185456 | FALSE |
| CBWD1 | 8432 | 1.857867 | 0.063188 | 0.18561 | FALSE |
| LIMK1 | 2401 | 1.857627 | 0.063222 | 0.185675 | FALSE |
| STK35 | 14495 | 1.853353 | 0.063832 | 0.187185 | FALSE |
| LCE2A | 12174 | 1.852182 | 0.064 | 0.187573 | FALSE |
| PUS1 | 9005 | 1.852124 | 0.064008 | 0.187573 | FALSE |
| ARHGAP9 | 2855 | 1.851994 | 0.064027 | 0.187573 | FALSE |
| RBM15 | 1629 | 1.851927 | 0.064036 | 0.187573 | FALSE |
| ZNF772 | 6328 | 1.850978 | 0.064173 | 0.187903 | FALSE |
| FBXO22 | 1189 | 1.850636 | 0.064222 | 0.18798 | FALSE |
| MRGPRF | 11179 | 1.849839 | 0.064337 | 0.188215 | FALSE |
| HCG4 | 13129 | 1.84982 | 0.064339 | 0.188215 | FALSE |
| RPEL1 | 1343 | 1.84864 | 0.06451 | 0.18855 | FALSE |
| GPR85 | 4666 | 1.848609 | 0.064514 | 0.18855 | FALSE |
| SRSF11 | 9555 | 1.848303 | 0.064559 | 0.188644 | FALSE |
| PSMG2 | 1695 | 1.846642 | 0.064799 | 0.189064 | FALSE |
| NELL1 | 5840 | 1.845756 | 0.064928 | 0.189368 | FALSE |
| MRPL48 | 2532 | 1.845339 | 0.064988 | 0.18951 | FALSE |
| THEM4 | 2938 | 1.845062 | 0.065029 | 0.189592 | FALSE |
| EP300 | 8377 | 1.844541 | 0.065104 | 0.189666 | FALSE |
| HIST1H1A | 6323 | 1.844509 | 0.065109 | 0.189666 | FALSE |
| FAM98B | 524 | 1.84447 | 0.065115 | 0.189666 | FALSE |
| ZNF318 | 3610 | 1.844271 | 0.065144 | 0.189715 | FALSE |
| ZNF653 | 5372 | 1.844128 | 0.065164 | 0.18974 | FALSE |
| PEX26 | 721 | 1.843606 | 0.065241 | 0.189876 | FALSE |
| ZNF17 | 6850 | 1.842911 | 0.065342 | 0.190045 | FALSE |
| IGIP | 10951 | 1.84274 | 0.065367 | 0.190082 | FALSE |
| TRIM13 | 12525 | 1.842605 | 0.065387 | 0.190104 | FALSE |
| MIR497HG | 12028 | 1.842494 | 0.065403 | 0.190116 | FALSE |
| STRADA | 15039 | 1.84211 | 0.065459 | 0.190244 | FALSE |
| MALAT1 | 5549 | 1.841731 | 0.065515 | 0.190299 | FALSE |
| SPDYE1 | 10016 | 1.840894 | 0.065637 | 0.190517 | FALSE |
| AMOTL1 | 3387 | 1.840501 | 0.065695 | 0.190517 | FALSE |
| ARHGAP32 | 8068 | 1.840457 | 0.065701 | 0.190517 | FALSE |
| EPC1 | 7238 | 1.837281 | 0.066168 | 0.191448 | FALSE |
| ABHD5 | 9350 | 1.837183 | 0.066183 | 0.191448 | FALSE |
| ARMCX4 | 11029 | 1.834902 | 0.06652 | 0.192214 | FALSE |
| GNG11 | 3635 | 1.83484 | 0.066529 | 0.192214 | FALSE |
| PHTF1 | 203 | 1.834833 | 0.06653 | 0.192214 | FALSE |
| NEK10 | 3331 | 1.832807 | 0.066831 | 0.192881 | FALSE |
| C1orf174 | 13631 | 1.832552 | 0.066869 | 0.192908 | FALSE |
| OR7E24 | 11833 | 1.832246 | 0.066915 | 0.192947 | FALSE |
| DSCAML1 | 6650 | 1.832239 | 0.066916 | 0.192947 | FALSE |
| PCGF6 | 8550 | 1.832169 | 0.066926 | 0.192947 | FALSE |
| LSMEM1 | 13848 | 1.832018 | 0.066949 | 0.192947 | FALSE |
| TDG | 9970 | 1.832006 | 0.066951 | 0.192947 | FALSE |
| STON1 | 9718 | 1.830142 | 0.067229 | 0.193435 | FALSE |
| ARL9 | 10769 | 1.82874 | 0.067439 | 0.193872 | FALSE |
| KCNIP2 | 934 | 1.828523 | 0.067471 | 0.193928 | FALSE |
| EXOSC6 | 11772 | 1.827374 | 0.067644 | 0.194352 | FALSE |
| NSUN4 | 4029 | 1.827062 | 0.06769 | 0.194432 | FALSE |
| GPR108 | 951 | 1.827024 | 0.067696 | 0.194432 | FALSE |
| SYCP2L | 6212 | 1.825529 | 0.067921 | 0.195007 | FALSE |
| NECAB3 | 15295 | 1.825285 | 0.067958 | 0.195077 | FALSE |
| CWC22 | 10324 | 1.825031 | 0.067996 | 0.195129 | FALSE |
| PROSER1 | 14389 | 1.824998 | 0.068001 | 0.195129 | FALSE |
| C2orf27A | 4717 | 1.824684 | 0.068049 | 0.195194 | FALSE |
| KLC2 | 1261 | 1.824432 | 0.068087 | 0.195231 | FALSE |
| MFSD14C | 1805 | 1.82396 | 0.068158 | 0.195296 | FALSE |
| TAF12 | 2147 | 1.823951 | 0.068159 | 0.195296 | FALSE |
| TMEM249 | 7538 | 1.823586 | 0.068215 | 0.19531 | FALSE |
| SENP6 | 8453 | 1.823586 | 0.068215 | 0.19531 | FALSE |
| GPATCH8 | 14380 | 1.823578 | 0.068216 | 0.19531 | FALSE |
| GOLGA6L17P | 3376 | 1.823425 | 0.068239 | 0.19531 | FALSE |
| GOLGA4 | 1917 | 1.823206 | 0.068272 | 0.195342 | FALSE |
| FBXO30 | 7646 | 1.823185 | 0.068275 | 0.195342 | FALSE |
| LEMD3 | 5044 | 1.823035 | 0.068298 | 0.195371 | FALSE |
| METTL18 | 13766 | 1.822134 | 0.068435 | 0.195619 | FALSE |
| KCTD3 | 6206 | 1.821926 | 0.068466 | 0.195673 | FALSE |
| CHSY1 | 14949 | 1.82173 | 0.068496 | 0.195722 | FALSE |
| TIA1 | 6518 | 1.821387 | 0.068548 | 0.195831 | FALSE |
| PCMTD1 | 540 | 1.821301 | 0.068561 | 0.195831 | FALSE |
| POLR1B | 10189 | 1.821232 | 0.068572 | 0.195831 | FALSE |
| FANCC | 15245 | 1.8208 | 0.068637 | 0.195983 | FALSE |
| TPRXL | 14610 | 1.819702 | 0.068804 | 0.196317 | FALSE |
| KIAA0754 | 7795 | 1.81959 | 0.068821 | 0.19633 | FALSE |
| PPP4R3A | 15016 | 1.819504 | 0.068835 | 0.196331 | FALSE |
| C5orf56 | 4530 | 1.819037 | 0.068906 | 0.196427 | FALSE |
| ARHGAP26 | 11451 | 1.818109 | 0.069047 | 0.196723 | FALSE |
| KIZ | 150 | 1.817763 | 0.0691 | 0.196781 | FALSE |
| SLC33A1 | 7275 | 1.817335 | 0.069166 | 0.196917 | FALSE |
| SCARA3 | 10943 | 1.817061 | 0.069208 | 0.196964 | FALSE |
| CITED2 | 4271 | 1.816079 | 0.069358 | 0.197321 | FALSE |
| MANSC1 | 10636 | 1.815818 | 0.069398 | 0.197363 | FALSE |
| ELF2 | 4805 | 1.815058 | 0.069515 | 0.197614 | FALSE |
| SRSF5 | 14012 | 1.814995 | 0.069525 | 0.197614 | FALSE |
| GON7 | 6378 | 1.814681 | 0.069573 | 0.197664 | FALSE |
| NDUFAF4 | 1652 | 1.814634 | 0.06958 | 0.197664 | FALSE |
| DOCK4 | 9702 | 1.814516 | 0.069598 | 0.197669 | FALSE |
| TRIO | 2351 | 1.813501 | 0.069755 | 0.198001 | FALSE |
| RTL5 | 8656 | 1.813478 | 0.069758 | 0.198001 | FALSE |
| MOCS1 | 15629 | 1.812856 | 0.069854 | 0.19819 | FALSE |
| SNAPC3 | 3335 | 1.811759 | 0.070023 | 0.198563 | FALSE |
| MRPL1 | 3374 | 1.810833 | 0.070167 | 0.198861 | FALSE |
| ZSCAN23 | 7487 | 1.81059 | 0.070204 | 0.1989 | FALSE |
| LINC01089 | 384 | 1.81058 | 0.070206 | 0.1989 | FALSE |
| CABIN1 | 14154 | 1.81037 | 0.070238 | 0.198956 | FALSE |
| SUDS3 | 12895 | 1.810127 | 0.070276 | 0.199026 | FALSE |
| PTGIS | 11409 | 1.809417 | 0.070386 | 0.19925 | FALSE |
| COTL1 | 11050 | 1.808296 | 0.07056 | 0.199543 | FALSE |
| ZFAT | 1773 | 1.808116 | 0.070588 | 0.199586 | FALSE |
| VASH2 | 8564 | 1.807385 | 0.070702 | 0.199871 | FALSE |
| MICAL2 | 11115 | 1.807092 | 0.070748 | 0.199928 | FALSE |
| EDRF1 | 10513 | 1.806832 | 0.070788 | 0.199966 | FALSE |
| LRP12 | 7762 | 1.806759 | 0.0708 | 0.199966 | FALSE |
| ANKRD45 | 5520 | 1.806031 | 0.070913 | 0.200179 | FALSE |
| EEF2 | 505 | 1.805845 | 0.070943 | 0.200205 | FALSE |
| CACNG8 | 571 | 1.805623 | 0.070977 | 0.200241 | FALSE |
| ARFGEF2 | 1895 | 1.805225 | 0.071039 | 0.200288 | FALSE |
| LOC100507642 | 4867 | 1.804038 | 0.071225 | 0.200697 | FALSE |
| MLH3 | 12127 | 1.802717 | 0.071433 | 0.201136 | FALSE |
| SPIRE2 | 4209 | 1.802134 | 0.071524 | 0.201358 | FALSE |
| ARHGAP27 | 12991 | 1.80192 | 0.071558 | 0.201416 | FALSE |
| EIF2D | 11954 | 1.80155 | 0.071616 | 0.201544 | FALSE |
| ELAVL1 | 1458 | 1.801318 | 0.071653 | 0.20161 | FALSE |
| PGBD4 | 6916 | 1.800857 | 0.071725 | 0.201779 | FALSE |
| CBR4 | 3493 | 1.800634 | 0.071761 | 0.201831 | FALSE |
| FAM161A | 8001 | 1.800461 | 0.071788 | 0.201831 | FALSE |
| PAQR9 | 9430 | 1.800412 | 0.071796 | 0.201831 | FALSE |
| WDR70 | 12237 | 1.799115 | 0.072 | 0.202298 | FALSE |
| ZNF566 | 14738 | 1.798683 | 0.072069 | 0.20238 | FALSE |
| ZNF165 | 6731 | 1.798175 | 0.072149 | 0.202534 | FALSE |
| TET1 | 13122 | 1.797891 | 0.072194 | 0.202587 | FALSE |
| GANC | 2889 | 1.797751 | 0.072216 | 0.202613 | FALSE |
| PPIG | 10801 | 1.797343 | 0.072281 | 0.202722 | FALSE |
| SPG11 | 13140 | 1.796584 | 0.072402 | 0.202953 | FALSE |
| ZNF565 | 13590 | 1.796579 | 0.072402 | 0.202953 | FALSE |
| EMCN | 13049 | 1.795935 | 0.072505 | 0.203131 | FALSE |
| ZNF436-AS1 | 15520 | 1.795753 | 0.072534 | 0.203139 | FALSE |
| MTRF1L | 7286 | 1.79536 | 0.072596 | 0.203197 | FALSE |
| MASTL | 667 | 1.795299 | 0.072606 | 0.203197 | FALSE |
| GUCD1 | 14356 | 1.794773 | 0.07269 | 0.203365 | FALSE |
| MTIF2 | 11172 | 1.794115 | 0.072795 | 0.203578 | FALSE |
| PCAT19 | 2348 | 1.793823 | 0.072841 | 0.203636 | FALSE |
| CCNE1 | 8334 | 1.793674 | 0.072865 | 0.203666 | FALSE |
| MGC27345 | 15027 | 1.79355 | 0.072885 | 0.203685 | FALSE |
| CDK5RAP2 | 3705 | 1.791786 | 0.073167 | 0.204291 | FALSE |
| NMD3 | 15172 | 1.791375 | 0.073233 | 0.204438 | FALSE |
| NRN1 | 1007 | 1.790843 | 0.073318 | 0.204581 | FALSE |
| SLC26A6 | 8112 | 1.790811 | 0.073324 | 0.204581 | FALSE |
| NDRG3 | 487 | 1.79021 | 0.07342 | 0.204777 | FALSE |
| PES1 | 15587 | 1.78994 | 0.073464 | 0.204862 | FALSE |
| PDHX | 9253 | 1.78898 | 0.073618 | 0.205073 | FALSE |
| SLC34A3 | 12243 | 1.788618 | 0.073676 | 0.205163 | FALSE |
| ZNF135 | 8074 | 1.7883 | 0.073728 | 0.205232 | FALSE |
| HUS1 | 13525 | 1.787104 | 0.073921 | 0.20566 | FALSE |
| PTPN4 | 9389 | 1.786912 | 0.073952 | 0.205709 | FALSE |
| LHFPL4 | 8002 | 1.786756 | 0.073977 | 0.205743 | FALSE |
| PDSS1 | 1082 | 1.785219 | 0.074226 | 0.206376 | FALSE |
| NUP107 | 8795 | 1.784892 | 0.074279 | 0.206472 | FALSE |
| ACAP2 | 14759 | 1.784409 | 0.074357 | 0.20658 | FALSE |
| SPAG5 | 9423 | 1.783981 | 0.074427 | 0.2067 | FALSE |
| ASCL1 | 4333 | 1.78365 | 0.074481 | 0.206776 | FALSE |
| LOC100506990 | 13141 | 1.782452 | 0.074676 | 0.20717 | FALSE |
| ZNF783 | 15288 | 1.781865 | 0.074771 | 0.207328 | FALSE |
| IRAK1BP1 | 8760 | 1.781859 | 0.074772 | 0.207328 | FALSE |
| ARL5B | 4353 | 1.781509 | 0.074829 | 0.207449 | FALSE |
| CCAR1 | 12638 | 1.781152 | 0.074888 | 0.207469 | FALSE |
| ANKS1B | 6603 | 1.781141 | 0.074889 | 0.207469 | FALSE |
| GSTCD | 14608 | 1.780868 | 0.074934 | 0.207519 | FALSE |
| PAM | 7867 | 1.780781 | 0.074948 | 0.207521 | FALSE |
| ZNF596 | 13396 | 1.780587 | 0.07498 | 0.207572 | FALSE |
| CLDN15 | 13538 | 1.780234 | 0.075038 | 0.207622 | FALSE |
| EVA1C | 12581 | 1.779339 | 0.075184 | 0.20788 | FALSE |
| ZNF711 | 12634 | 1.778657 | 0.075296 | 0.208027 | FALSE |
| ZNF841 | 9171 | 1.778446 | 0.075331 | 0.208064 | FALSE |
| UNC80 | 1737 | 1.777417 | 0.0755 | 0.208421 | FALSE |
| GALT | 7693 | 1.776908 | 0.075583 | 0.208541 | FALSE |
| CLVS2 | 10665 | 1.776672 | 0.075622 | 0.208547 | FALSE |
| ZNF736 | 4798 | 1.776652 | 0.075626 | 0.208547 | FALSE |
| KIN | 14225 | 1.775261 | 0.075855 | 0.209078 | FALSE |
| ARCN1 | 1872 | 1.77516 | 0.075871 | 0.209078 | FALSE |
| PCDHB13 | 14754 | 1.774786 | 0.075933 | 0.209174 | FALSE |
| UTY | 7994 | 1.773739 | 0.076106 | 0.209467 | FALSE |
| MEF2C | 13517 | 1.773465 | 0.076152 | 0.209469 | FALSE |
| FBRS | 12058 | 1.773413 | 0.07616 | 0.209469 | FALSE |
| FGR | 1551 | 1.772816 | 0.076259 | 0.209703 | FALSE |
| CRYGA | 15567 | 1.772722 | 0.076275 | 0.209709 | FALSE |
| RWDD2B | 4118 | 1.771079 | 0.076548 | 0.210422 | FALSE |
| CD46 | 8822 | 1.770665 | 0.076616 | 0.210537 | FALSE |
| LOC100505938 | 6063 | 1.770159 | 0.076701 | 0.210655 | FALSE |
| FASTKD5 | 5805 | 1.770074 | 0.076715 | 0.210655 | FALSE |
| SLC25A34 | 15393 | 1.770003 | 0.076727 | 0.210655 | FALSE |
| KCTD8 | 9488 | 1.76905 | 0.076886 | 0.210933 | FALSE |
| PMM2 | 6613 | 1.768738 | 0.076938 | 0.210974 | FALSE |
| EPHA3 | 7796 | 1.768283 | 0.077014 | 0.211072 | FALSE |
| FAM214A | 87 | 1.767714 | 0.077109 | 0.211295 | FALSE |
| AP1G1 | 3319 | 1.767499 | 0.077145 | 0.211357 | FALSE |
| AARS | 5347 | 1.767293 | 0.077179 | 0.211408 | FALSE |
| TBC1D1 | 13636 | 1.767227 | 0.07719 | 0.211408 | FALSE |
| LRP8 | 4394 | 1.765515 | 0.077477 | 0.211971 | FALSE |
| 14-Sep | 5182 | 1.764445 | 0.077657 | 0.212269 | FALSE |
| CEP135 | 2071 | 1.763847 | 0.077758 | 0.212461 | FALSE |
| LINC00597 | 8325 | 1.763364 | 0.077839 | 0.212515 | FALSE |
| KLHL15 | 1457 | 1.762824 | 0.07793 | 0.212726 | FALSE |
| KCNAB2 | 8292 | 1.762702 | 0.077951 | 0.212745 | FALSE |
| HNRNPKP3 | 6678 | 1.762274 | 0.078023 | 0.212868 | FALSE |
| ACTR5 | 7708 | 1.758152 | 0.078722 | 0.214512 | FALSE |
| AHSA2P | 12550 | 1.757859 | 0.078772 | 0.21461 | FALSE |
| VPS13B | 2957 | 1.757507 | 0.078831 | 0.214699 | FALSE |
| LINC00869 | 2513 | 1.756859 | 0.078942 | 0.214925 | FALSE |
| BCL2L2 | 5383 | 1.756282 | 0.07904 | 0.215118 | FALSE |
| PABPC1L2B | 4960 | 1.755822 | 0.079119 | 0.215232 | FALSE |
| SH3BP5L | 10425 | 1.755794 | 0.079124 | 0.215232 | FALSE |
| ANKH | 5494 | 1.755317 | 0.079205 | 0.215389 | FALSE |
| ZBTB14 | 1686 | 1.754642 | 0.079321 | 0.215655 | FALSE |
| PDE10A | 5552 | 1.754558 | 0.079335 | 0.215657 | FALSE |
| MAP3K21 | 2824 | 1.753812 | 0.079463 | 0.215967 | FALSE |
| PAGR1 | 7213 | 1.752904 | 0.079618 | 0.216315 | FALSE |
| RALGDS | 14922 | 1.752435 | 0.079699 | 0.216496 | FALSE |
| TPTE2P5 | 13385 | 1.751869 | 0.079796 | 0.216698 | FALSE |
| WDFY3 | 11968 | 1.751764 | 0.079814 | 0.216698 | FALSE |
| TOX2 | 814 | 1.751367 | 0.079883 | 0.216758 | FALSE |
| ZKSCAN3 | 11094 | 1.75131 | 0.079893 | 0.216758 | FALSE |
| C17orf80 | 494 | 1.751001 | 0.079946 | 0.216865 | FALSE |
| ZNF320 | 3590 | 1.750823 | 0.079976 | 0.21691 | FALSE |
| UBP1 | 3913 | 1.750461 | 0.080039 | 0.217042 | FALSE |
| FAM69A | 4378 | 1.750071 | 0.080106 | 0.217149 | FALSE |
| CACTIN | 8263 | 1.747492 | 0.080552 | 0.21813 | FALSE |
| PMS2CL | 8391 | 1.746316 | 0.080756 | 0.218532 | FALSE |
| TBC1D2B | 5532 | 1.74579 | 0.080847 | 0.218704 | FALSE |
| RSBN1 | 15435 | 1.745349 | 0.080924 | 0.218873 | FALSE |
| RCL1 | 13965 | 1.745021 | 0.080981 | 0.21899 | FALSE |
| SLC6A15 | 2407 | 1.74494 | 0.080995 | 0.21899 | FALSE |
| KIF5C | 2254 | 1.743416 | 0.081261 | 0.219518 | FALSE |
| CAVIN2 | 10915 | 1.742026 | 0.081504 | 0.220061 | FALSE |
| PARM1 | 644 | 1.741757 | 0.081551 | 0.22015 | FALSE |
| CDON | 3670 | 1.741454 | 0.081604 | 0.220255 | FALSE |
| GZF1 | 3931 | 1.74094 | 0.081694 | 0.220422 | FALSE |
| NRIP3 | 14427 | 1.740496 | 0.081772 | 0.220594 | FALSE |
| ZNF181 | 15169 | 1.740214 | 0.081821 | 0.220597 | FALSE |
| MROH6 | 11126 | 1.740167 | 0.08183 | 0.220597 | FALSE |
| POLA1 | 7333 | 1.739638 | 0.081923 | 0.220707 | FALSE |
| TMOD2 | 6665 | 1.739534 | 0.081941 | 0.220707 | FALSE |
| FASTKD3 | 3181 | 1.738989 | 0.082037 | 0.220892 | FALSE |
| TMX4 | 14678 | 1.738983 | 0.082038 | 0.220892 | FALSE |
| SMAP1 | 7857 | 1.738777 | 0.082074 | 0.220951 | FALSE |
| TAS2R10 | 9252 | 1.73832 | 0.082154 | 0.22113 | FALSE |
| GAB1 | 10805 | 1.737436 | 0.08231 | 0.221435 | FALSE |
| DDB2 | 13999 | 1.737257 | 0.082342 | 0.221481 | FALSE |
| BBS10 | 5818 | 1.737142 | 0.082362 | 0.221498 | FALSE |
| PRRT4 | 7699 | 1.736916 | 0.082402 | 0.221567 | FALSE |
| FAM155A | 4115 | 1.736424 | 0.082489 | 0.22169 | FALSE |
| CYP20A1 | 2916 | 1.736416 | 0.08249 | 0.22169 | FALSE |
| TTC5 | 13989 | 1.736209 | 0.082527 | 0.22175 | FALSE |
| ZBTB8OS | 12652 | 1.735734 | 0.082611 | 0.221915 | FALSE |
| EPHB2 | 2764 | 1.734925 | 0.082754 | 0.22217 | FALSE |
| CHAF1A | 4966 | 1.734563 | 0.082818 | 0.222266 | FALSE |
| MTERF3 | 4077 | 1.734283 | 0.082868 | 0.222348 | FALSE |
| DR1 | 8509 | 1.733398 | 0.083025 | 0.222591 | FALSE |
| SMAD4 | 1295 | 1.732931 | 0.083108 | 0.222775 | FALSE |
| LEAP2 | 9875 | 1.732552 | 0.083175 | 0.22288 | FALSE |
| RAP2C-AS1 | 9027 | 1.732252 | 0.083229 | 0.222911 | FALSE |
| TMEM268 | 14715 | 1.732246 | 0.08323 | 0.222911 | FALSE |
| TOP1P1 | 9610 | 1.731783 | 0.083312 | 0.223055 | FALSE |
| MYMX | 4089 | 1.731412 | 0.083378 | 0.223093 | FALSE |
| BCL10 | 3828 | 1.730445 | 0.083551 | 0.223426 | FALSE |
| ZNF717 | 2406 | 1.729656 | 0.083692 | 0.223765 | FALSE |
| BICDL1 | 6423 | 1.728138 | 0.083963 | 0.224338 | FALSE |
| STRIP2 | 13567 | 1.727881 | 0.08401 | 0.224416 | FALSE |
| MAP1A | 3552 | 1.727815 | 0.084021 | 0.224416 | FALSE |
| SPARCL1 | 8668 | 1.727242 | 0.084124 | 0.224597 | FALSE |
| SNRK | 15211 | 1.727207 | 0.08413 | 0.224597 | FALSE |
| ZNF443 | 13669 | 1.726435 | 0.084269 | 0.224847 | FALSE |
| MTF1 | 13218 | 1.726233 | 0.084306 | 0.224906 | FALSE |
| NUMB | 7084 | 1.724839 | 0.084556 | 0.225288 | FALSE |
| MAP3K13 | 2235 | 1.724809 | 0.084562 | 0.225288 | FALSE |
| CFL2 | 14591 | 1.724783 | 0.084567 | 0.225288 | FALSE |
| PCM1 | 4663 | 1.724683 | 0.084585 | 0.225288 | FALSE |
| ASH1L | 10835 | 1.724641 | 0.084592 | 0.225288 | FALSE |
| GLRB | 12110 | 1.724638 | 0.084593 | 0.225288 | FALSE |
| TJAP1 | 201 | 1.722104 | 0.085051 | 0.226314 | FALSE |
| CPOX | 15045 | 1.721623 | 0.085138 | 0.226349 | FALSE |
| SLC7A6 | 13945 | 1.721137 | 0.085226 | 0.226507 | FALSE |
| ZNF22 | 5590 | 1.720986 | 0.085253 | 0.226507 | FALSE |
| TMEM192 | 5544 | 1.720263 | 0.085385 | 0.226694 | FALSE |
| CDKN3 | 12951 | 1.720198 | 0.085396 | 0.226694 | FALSE |
| NUP54 | 8128 | 1.720065 | 0.085421 | 0.22672 | FALSE |
| KIAA1107 | 3651 | 1.719298 | 0.08556 | 0.226975 | FALSE |
| RBM39 | 6161 | 1.718643 | 0.085679 | 0.227252 | FALSE |
| TMEM265 | 10056 | 1.718043 | 0.085789 | 0.227427 | FALSE |
| CTSZ | 3897 | 1.716824 | 0.086011 | 0.227824 | FALSE |
| LOC645513 | 3347 | 1.716354 | 0.086097 | 0.227967 | FALSE |
| MIS18A | 128 | 1.716132 | 0.086138 | 0.22797 | FALSE |
| OTUD6B-AS1 | 13319 | 1.71537 | 0.086277 | 0.228219 | FALSE |
| DAZAP2 | 2699 | 1.714853 | 0.086372 | 0.228431 | FALSE |
| GSPT1 | 1725 | 1.71462 | 0.086415 | 0.228505 | FALSE |
| ABHD18 | 13121 | 1.714243 | 0.086484 | 0.22865 | FALSE |
| LOC100128398 | 15321 | 1.713888 | 0.086549 | 0.228745 | FALSE |
| ERO1A | 3868 | 1.713737 | 0.086577 | 0.228779 | FALSE |
| HSD17B11 | 960 | 1.712058 | 0.086886 | 0.229324 | FALSE |
| DCLK1 | 9352 | 1.711613 | 0.086968 | 0.229502 | FALSE |
| MAP2K7 | 10734 | 1.711121 | 0.087059 | 0.229703 | FALSE |
| FAM35BP | 1395 | 1.710968 | 0.087087 | 0.229739 | FALSE |
| AP1G2 | 14677 | 1.7104 | 0.087192 | 0.229938 | FALSE |
| ANKRD20A12P | 15131 | 1.709666 | 0.087328 | 0.230218 | FALSE |
| CNNM3 | 7623 | 1.70868 | 0.08751 | 0.230578 | FALSE |
| PMS2P3 | 10693 | 1.708594 | 0.087526 | 0.230578 | FALSE |
| EIF4E3 | 2871 | 1.70828 | 0.087584 | 0.230662 | FALSE |
| HERPUD2 | 4486 | 1.707914 | 0.087652 | 0.230801 | FALSE |
| UNC119B | 17 | 1.707823 | 0.087669 | 0.230807 | FALSE |
| SPATA7 | 14333 | 1.705534 | 0.088095 | 0.231543 | FALSE |
| EPM2AIP1 | 11708 | 1.705509 | 0.0881 | 0.231543 | FALSE |
| PNPT1 | 5145 | 1.705445 | 0.088111 | 0.231543 | FALSE |
| CCNI2 | 11611 | 1.704378 | 0.08831 | 0.231949 | FALSE |
| SNX21 | 1256 | 1.703162 | 0.088538 | 0.232366 | FALSE |
| CBX7 | 9078 | 1.703015 | 0.088565 | 0.232381 | FALSE |
| SNX20 | 4853 | 1.7026 | 0.088643 | 0.232509 | FALSE |
| TIGD7 | 13175 | 1.702428 | 0.088675 | 0.232531 | FALSE |
| BMS1P1 | 2872 | 1.702313 | 0.088697 | 0.232533 | FALSE |
| LOC100190986 | 13910 | 1.701551 | 0.08884 | 0.232791 | FALSE |
| CNOT1 | 11913 | 1.70131 | 0.088885 | 0.23287 | FALSE |
| GGACT | 2166 | 1.700683 | 0.089003 | 0.233061 | FALSE |
| PNMA2 | 8418 | 1.700201 | 0.089093 | 0.233212 | FALSE |
| ASAP1-IT1 | 15215 | 1.700139 | 0.089105 | 0.233212 | FALSE |
| MAPK6 | 11840 | 1.69968 | 0.089191 | 0.233321 | FALSE |
| NEUROG3 | 11960 | 1.698037 | 0.089501 | 0.233935 | FALSE |
| FOXRED1 | 1166 | 1.697895 | 0.089528 | 0.233966 | FALSE |
| ELOA | 12576 | 1.697187 | 0.089661 | 0.234206 | FALSE |
| MGAT3 | 11739 | 1.696859 | 0.089723 | 0.234321 | FALSE |
| OMG | 9734 | 1.69567 | 0.089948 | 0.23483 | FALSE |
| LTB | 9154 | 1.695107 | 0.090055 | 0.23499 | FALSE |
| SLC6A7 | 144 | 1.695043 | 0.090067 | 0.23499 | FALSE |
| B4GALT4 | 14590 | 1.694646 | 0.090143 | 0.235141 | FALSE |
| KLHL42 | 7859 | 1.694326 | 0.090203 | 0.23526 | FALSE |
| FAM21EP | 4339 | 1.693624 | 0.090337 | 0.235529 | FALSE |
| SERTAD2 | 8749 | 1.692777 | 0.090498 | 0.235754 | FALSE |
| ZNF444 | 438 | 1.692626 | 0.090527 | 0.235754 | FALSE |
| REL | 428 | 1.692321 | 0.090585 | 0.235754 | FALSE |
| LOC100506476 | 8354 | 1.692321 | 0.090585 | 0.235754 | FALSE |
| ARL6IP6 | 11842 | 1.692224 | 0.090603 | 0.235754 | FALSE |
| SPEF2 | 5486 | 1.69222 | 0.090604 | 0.235754 | FALSE |
| CLDN9 | 9150 | 1.691837 | 0.090677 | 0.235866 | FALSE |
| FAM199X | 14768 | 1.691569 | 0.090728 | 0.23592 | FALSE |
| ZNF383 | 3625 | 1.691078 | 0.090822 | 0.236086 | FALSE |
| CEBPG | 13973 | 1.689944 | 0.091039 | 0.236451 | FALSE |
| NAPA | 12628 | 1.689868 | 0.091053 | 0.236451 | FALSE |
| GIN1 | 938 | 1.689573 | 0.09111 | 0.236471 | FALSE |
| PRMT3 | 12255 | 1.689526 | 0.091119 | 0.236471 | FALSE |
| ZNF394 | 12432 | 1.689432 | 0.091137 | 0.236471 | FALSE |
| VPS13C | 10086 | 1.688561 | 0.091304 | 0.236787 | FALSE |
| HRH2 | 6253 | 1.687477 | 0.091512 | 0.237208 | FALSE |
| C5orf22 | 2717 | 1.687307 | 0.091544 | 0.23721 | FALSE |
| REEP3 | 11242 | 1.687236 | 0.091558 | 0.23721 | FALSE |
| CCDC115 | 6280 | 1.687063 | 0.091591 | 0.237257 | FALSE |
| OSBPL5 | 15244 | 1.68631 | 0.091736 | 0.237494 | FALSE |
| TCTN2 | 11043 | 1.685415 | 0.091909 | 0.237803 | FALSE |
| BAX | 5029 | 1.684242 | 0.092135 | 0.238343 | FALSE |
| SNAP91 | 7763 | 1.684175 | 0.092148 | 0.238343 | FALSE |
| MRPL34 | 3880 | 1.683227 | 0.092331 | 0.238639 | FALSE |
| PTPN12 | 14893 | 1.682678 | 0.092437 | 0.238816 | FALSE |
| FEN1 | 6449 | 1.682514 | 0.092469 | 0.238858 | FALSE |
| SLC2A3 | 1163 | 1.681828 | 0.092602 | 0.239145 | FALSE |
| DUSP7 | 621 | 1.681587 | 0.092649 | 0.239145 | FALSE |
| HECW2 | 11460 | 1.681548 | 0.092657 | 0.239145 | FALSE |
| RNF114 | 9469 | 1.681247 | 0.092715 | 0.239194 | FALSE |
| PCBP2 | 6287 | 1.681187 | 0.092727 | 0.239194 | FALSE |
| SPEG | 5917 | 1.681134 | 0.092737 | 0.239194 | FALSE |
| MAT2A | 5535 | 1.68088 | 0.092786 | 0.239282 | FALSE |
| ZNF862 | 4079 | 1.680636 | 0.092834 | 0.239365 | FALSE |
| STK40 | 612 | 1.680022 | 0.092953 | 0.239556 | FALSE |
| CHD9 | 14500 | 1.67994 | 0.092969 | 0.239556 | FALSE |
| MGC16025 | 12167 | 1.67963 | 0.093029 | 0.239672 | FALSE |
| SPACA6 | 9943 | 1.67747 | 0.093451 | 0.240559 | FALSE |
| GTF2I | 9007 | 1.677232 | 0.093497 | 0.240599 | FALSE |
| CMBL | 13078 | 1.67683 | 0.093576 | 0.240683 | FALSE |
| NDUFB5 | 14019 | 1.676262 | 0.093687 | 0.240929 | FALSE |
| DCAF17 | 10879 | 1.675114 | 0.093912 | 0.241468 | FALSE |
| MYO9B | 7124 | 1.674768 | 0.09398 | 0.241603 | FALSE |
| FBXO16 | 12772 | 1.673775 | 0.094175 | 0.242025 | FALSE |
| TMEM250 | 10751 | 1.671933 | 0.094538 | 0.242757 | FALSE |
| RPAP2 | 1187 | 1.670964 | 0.094729 | 0.243128 | FALSE |
| DMPK | 9554 | 1.670676 | 0.094786 | 0.243189 | FALSE |
| 7-Mar | 15416 | 1.670608 | 0.094799 | 0.243189 | FALSE |
| NAA40 | 10988 | 1.669804 | 0.094958 | 0.243557 | FALSE |
| HEATR6 | 7710 | 1.668206 | 0.095275 | 0.244249 | FALSE |
| ITGB1BP2 | 14911 | 1.667999 | 0.095316 | 0.244274 | FALSE |
| CRABP1 | 8222 | 1.6678 | 0.095355 | 0.244306 | FALSE |
| INPP5B | 12201 | 1.66753 | 0.095409 | 0.244327 | FALSE |
| VIPR2 | 11425 | 1.667502 | 0.095415 | 0.244327 | FALSE |
| SETBP1 | 3655 | 1.666821 | 0.09555 | 0.244574 | FALSE |
| ZSCAN21 | 6760 | 1.666646 | 0.095585 | 0.244578 | FALSE |
| MAD2L1 | 9752 | 1.666616 | 0.095591 | 0.244578 | FALSE |
| GAPVD1 | 1365 | 1.666385 | 0.095637 | 0.244644 | FALSE |
| FBXL12 | 9283 | 1.666328 | 0.095648 | 0.244644 | FALSE |
| CACNA2D3 | 1244 | 1.665439 | 0.095825 | 0.244937 | FALSE |
| SH3BGRL2 | 4298 | 1.665301 | 0.095853 | 0.244967 | FALSE |
| CABP1 | 6441 | 1.664565 | 0.096 | 0.245263 | FALSE |
| TOP3A | 13928 | 1.6642 | 0.096072 | 0.245409 | FALSE |
| NKX2-8 | 9556 | 1.663836 | 0.096145 | 0.245482 | FALSE |
| CCDC65 | 9365 | 1.663787 | 0.096155 | 0.245482 | FALSE |
| FAM86HP | 9894 | 1.6635 | 0.096212 | 0.245482 | FALSE |
| ALDH8A1 | 14579 | 1.663301 | 0.096252 | 0.245482 | FALSE |
| NAT10 | 5113 | 1.663156 | 0.096281 | 0.245501 | FALSE |
| PIAS2 | 4987 | 1.662027 | 0.096507 | 0.246037 | FALSE |
| PDCD10 | 6019 | 1.661712 | 0.096571 | 0.246158 | FALSE |
| EBF1 | 7447 | 1.661576 | 0.096598 | 0.246187 | FALSE |
| XRN1 | 9138 | 1.660791 | 0.096755 | 0.246428 | FALSE |
| SLC43A2 | 14321 | 1.660713 | 0.096771 | 0.246428 | FALSE |
| KCTD9 | 847 | 1.657986 | 0.09732 | 0.247383 | FALSE |
| DCBLD1 | 14488 | 1.657471 | 0.097424 | 0.247608 | FALSE |
| NOP53 | 9385 | 1.656761 | 0.097568 | 0.247851 | FALSE |
| ZNF598 | 6071 | 1.656325 | 0.097656 | 0.247995 | FALSE |
| ZNF782 | 632 | 1.656013 | 0.097719 | 0.248002 | FALSE |
| SLC7A8 | 2724 | 1.655841 | 0.097754 | 0.248042 | FALSE |
| TAPBP | 2509 | 1.654998 | 0.097925 | 0.248355 | FALSE |
| GPR22 | 12233 | 1.654805 | 0.097964 | 0.248414 | FALSE |
| DDX3Y | 8774 | 1.6545 | 0.098026 | 0.248531 | FALSE |
| RSRC1 | 5357 | 1.654297 | 0.098067 | 0.248554 | FALSE |
| TAS2R13 | 11962 | 1.654224 | 0.098082 | 0.248554 | FALSE |
| PCP4 | 1973 | 1.653344 | 0.098261 | 0.248924 | FALSE |
| TAF1 | 7158 | 1.653227 | 0.098285 | 0.248944 | FALSE |
| FAM57B | 14993 | 1.65218 | 0.098498 | 0.249282 | FALSE |
| MATN4 | 4419 | 1.651552 | 0.098626 | 0.249526 | FALSE |
| LOC105378663 | 431 | 1.650764 | 0.098787 | 0.249874 | FALSE |
| RAP2A | 5463 | 1.650721 | 0.098796 | 0.249874 | FALSE |
| INPP4B | 12040 | 1.649844 | 0.098975 | 0.250156 | FALSE |
| MYO1E | 5115 | 1.649788 | 0.098986 | 0.250156 | FALSE |
| DIAPH1 | 4434 | 1.649543 | 0.099036 | 0.2502 | FALSE |
| CALML4 | 11191 | 1.648764 | 0.099196 | 0.250522 | FALSE |
| KCTD1 | 6177 | 1.648309 | 0.099289 | 0.250717 | FALSE |
| SESTD1 | 488 | 1.648017 | 0.099349 | 0.250787 | FALSE |
| PPM1E | 7340 | 1.647601 | 0.099435 | 0.250962 | FALSE |
| PRPF39 | 12229 | 1.647406 | 0.099475 | 0.251008 | FALSE |
| NXF1 | 8186 | 1.647047 | 0.099548 | 0.251128 | FALSE |
| CYP1B1-AS1 | 12663 | 1.645884 | 0.099788 | 0.25165 | FALSE |
| RBBP6 | 14842 | 1.644863 | 0.099998 | 0.2521 | FALSE |
| GPATCH1 | 3688 | 1.644653 | 0.100041 | 0.252168 | FALSE |
| CDK19 | 7813 | 1.64367 | 0.100244 | 0.252639 | FALSE |
| FGFR2 | 10587 | 1.643451 | 0.10029 | 0.252672 | FALSE |
| ZNF823 | 6514 | 1.642283 | 0.100531 | 0.25308 | FALSE |
| WDSUB1 | 4408 | 1.642264 | 0.100535 | 0.25308 | FALSE |
| CNIH1 | 13873 | 1.642072 | 0.100575 | 0.25308 | FALSE |
| LOC100133331 | 2637 | 1.642068 | 0.100576 | 0.25308 | FALSE |
| TMEM243 | 7809 | 1.641969 | 0.100596 | 0.25308 | FALSE |
| H2AFX | 2617 | 1.641964 | 0.100597 | 0.25308 | FALSE |
| OSBPL1A | 4338 | 1.641539 | 0.100686 | 0.253229 | FALSE |
| NACC2 | 10737 | 1.640552 | 0.10089 | 0.253572 | FALSE |
| ZNF330 | 4083 | 1.640453 | 0.100911 | 0.253583 | FALSE |
| TBL1Y | 3219 | 1.639933 | 0.101019 | 0.253692 | FALSE |
| TRMT1L | 4057 | 1.639595 | 0.101089 | 0.253787 | FALSE |
| ECHDC2 | 15310 | 1.639394 | 0.101131 | 0.253851 | FALSE |
| HAUS6 | 10979 | 1.638987 | 0.101216 | 0.254023 | FALSE |
| RAB30 | 12851 | 1.638702 | 0.101275 | 0.254085 | FALSE |
| ZKSCAN4 | 3719 | 1.638634 | 0.10129 | 0.254085 | FALSE |
| TSPAN9 | 1456 | 1.638093 | 0.101402 | 0.254205 | FALSE |
| CNOT10 | 6434 | 1.636241 | 0.101789 | 0.255093 | FALSE |
| PLEKHG1 | 2236 | 1.635806 | 0.10188 | 0.255227 | FALSE |
| ACTR2 | 282 | 1.635674 | 0.101908 | 0.255227 | FALSE |
| PRPS2 | 15096 | 1.635518 | 0.101941 | 0.255268 | FALSE |
| NFYC | 14771 | 1.635255 | 0.101996 | 0.255324 | FALSE |
| LMO7 | 994 | 1.634646 | 0.102123 | 0.255562 | FALSE |
| MINPP1 | 14930 | 1.633913 | 0.102277 | 0.255783 | FALSE |
| KPNA6 | 15511 | 1.633539 | 0.102356 | 0.255938 | FALSE |
| HOOK3 | 7687 | 1.632203 | 0.102637 | 0.256518 | FALSE |
| ABHD17A | 8832 | 1.631499 | 0.102785 | 0.256791 | FALSE |
| ACOT9 | 5752 | 1.631416 | 0.102803 | 0.256791 | FALSE |
| ATG4B | 623 | 1.630819 | 0.102929 | 0.257001 | FALSE |
| AGAP1 | 14455 | 1.629801 | 0.103144 | 0.257332 | FALSE |
| THBS2 | 6124 | 1.62843 | 0.103434 | 0.257913 | FALSE |
| ZNF510 | 7877 | 1.627918 | 0.103542 | 0.258121 | FALSE |
| KMT5B | 9818 | 1.627761 | 0.103576 | 0.258129 | FALSE |
| RUNDC3B | 2083 | 1.627691 | 0.10359 | 0.258129 | FALSE |
| LMBRD2 | 6210 | 1.627669 | 0.103595 | 0.258129 | FALSE |
| DAGLB | 137 | 1.627497 | 0.103632 | 0.258179 | FALSE |
| CNDP1 | 8740 | 1.626938 | 0.10375 | 0.258433 | FALSE |
| LOC100129917 | 13897 | 1.626758 | 0.103789 | 0.258446 | FALSE |
| SNX9 | 14166 | 1.626345 | 0.103876 | 0.258624 | FALSE |
| NPTN | 7584 | 1.625838 | 0.103984 | 0.258851 | FALSE |
| CCNJ | 14414 | 1.625477 | 0.104061 | 0.259001 | FALSE |
| ESYT1 | 9864 | 1.624162 | 0.104341 | 0.259575 | FALSE |
| TMEM87A | 7422 | 1.623835 | 0.104411 | 0.259666 | FALSE |
| CAMK1 | 10198 | 1.623343 | 0.104516 | 0.259844 | FALSE |
| PXDN | 5063 | 1.623248 | 0.104536 | 0.259853 | FALSE |
| SYNGR1 | 1140 | 1.622995 | 0.10459 | 0.259915 | FALSE |
| FNIP1 | 8792 | 1.622854 | 0.104621 | 0.259939 | FALSE |
| FAM221A | 11276 | 1.622755 | 0.104642 | 0.25995 | FALSE |
| GTF2IP20 | 14627 | 1.622676 | 0.104659 | 0.259951 | FALSE |
| ALDH1A1 | 14149 | 1.621415 | 0.104929 | 0.260497 | FALSE |
| TIAL1 | 4395 | 1.620943 | 0.10503 | 0.260665 | FALSE |
| POLK | 15465 | 1.620806 | 0.105059 | 0.260697 | FALSE |
| NOP2 | 11124 | 1.620386 | 0.105149 | 0.260819 | FALSE |
| DSP | 7335 | 1.620266 | 0.105175 | 0.260819 | FALSE |
| RBM17 | 14841 | 1.619424 | 0.105356 | 0.261226 | FALSE |
| CDR1 | 10755 | 1.618329 | 0.105592 | 0.261532 | FALSE |
| ST8SIA5 | 7749 | 1.618307 | 0.105596 | 0.261532 | FALSE |
| ADNP2 | 11894 | 1.617529 | 0.105764 | 0.261794 | FALSE |
| ARAP3 | 10348 | 1.61735 | 0.105803 | 0.261794 | FALSE |
| PCDHB8 | 1881 | 1.617174 | 0.105841 | 0.261847 | FALSE |
| DIRAS1 | 4860 | 1.61688 | 0.105904 | 0.261962 | FALSE |
| ZNF639 | 3500 | 1.61612 | 0.106068 | 0.262327 | FALSE |
| SH3BP1 | 4566 | 1.615091 | 0.106291 | 0.262805 | FALSE |
| PPP3CB | 15624 | 1.614733 | 0.106369 | 0.262805 | FALSE |
| FAM198B | 9941 | 1.614629 | 0.106391 | 0.262805 | FALSE |
| SPSB1 | 9269 | 1.614623 | 0.106392 | 0.262805 | FALSE |
| APPL1 | 15439 | 1.614248 | 0.106474 | 0.262872 | FALSE |
| CEP290 | 15192 | 1.614126 | 0.1065 | 0.262895 | FALSE |
| ZFPM1 | 12015 | 1.613006 | 0.106743 | 0.263288 | FALSE |
| ZW10 | 13184 | 1.612225 | 0.106913 | 0.263623 | FALSE |
| UPF1 | 8870 | 1.612073 | 0.106946 | 0.263642 | FALSE |
| GNB3 | 9020 | 1.612003 | 0.106961 | 0.263642 | FALSE |
| AKAP7 | 4740 | 1.611879 | 0.106988 | 0.263642 | FALSE |
| POU3F1 | 9069 | 1.610843 | 0.107214 | 0.26399 | FALSE |
| PTTG2 | 2796 | 1.609947 | 0.107409 | 0.264315 | FALSE |
| SYS1 | 14834 | 1.609929 | 0.107413 | 0.264315 | FALSE |
| FAN1 | 14704 | 1.609624 | 0.10748 | 0.264437 | FALSE |
| TLE1 | 15554 | 1.608389 | 0.10775 | 0.264893 | FALSE |
| CMTR2 | 12594 | 1.608276 | 0.107775 | 0.264912 | FALSE |
| ZBTB39 | 8844 | 1.608166 | 0.107799 | 0.26493 | FALSE |
| PHF21A | 10638 | 1.607781 | 0.107883 | 0.265012 | FALSE |
| KCNIP4 | 15396 | 1.606988 | 0.108057 | 0.265384 | FALSE |
| ZNF536 | 11916 | 1.606726 | 0.108114 | 0.265455 | FALSE |
| ELP1 | 8061 | 1.60632 | 0.108204 | 0.265627 | FALSE |
| CLK3 | 7 | 1.603471 | 0.108831 | 0.26692 | FALSE |
| GTDC1 | 7815 | 1.603219 | 0.108886 | 0.266984 | FALSE |
| LOC494141 | 12195 | 1.602365 | 0.109075 | 0.267268 | FALSE |
| ARL4C | 7920 | 1.601398 | 0.109289 | 0.26757 | FALSE |
| PIGZ | 2122 | 1.601251 | 0.109321 | 0.26757 | FALSE |
| NBPF22P | 6873 | 1.601188 | 0.109335 | 0.26757 | FALSE |
| ZNF358 | 10035 | 1.601111 | 0.109352 | 0.26757 | FALSE |
| ZNF791 | 908 | 1.599718 | 0.109661 | 0.268172 | FALSE |
| ZNF567 | 5824 | 1.599692 | 0.109667 | 0.268172 | FALSE |
| RBM22 | 4547 | 1.598837 | 0.109857 | 0.268559 | FALSE |
| TSNAX | 11850 | 1.598434 | 0.109946 | 0.268687 | FALSE |
| ARHGEF3 | 15164 | 1.597779 | 0.110092 | 0.268835 | FALSE |
| ATP6V0A2 | 11138 | 1.597667 | 0.110117 | 0.268852 | FALSE |
| GDAP2 | 13791 | 1.596836 | 0.110302 | 0.269113 | FALSE |
| ALDH3A2 | 14179 | 1.596744 | 0.110323 | 0.269113 | FALSE |
| CPSF7 | 6996 | 1.596558 | 0.110364 | 0.269148 | FALSE |
| ISL1 | 1808 | 1.596308 | 0.11042 | 0.269175 | FALSE |
| CYP26B1 | 1880 | 1.596302 | 0.110421 | 0.269175 | FALSE |
| LOC283788 | 15572 | 1.596197 | 0.110445 | 0.26919 | FALSE |
| GATAD2A | 15318 | 1.595508 | 0.110599 | 0.269324 | FALSE |
| CHD2 | 12308 | 1.595343 | 0.110636 | 0.269324 | FALSE |
| DBF4 | 15545 | 1.595333 | 0.110638 | 0.269324 | FALSE |
| HIST1H4E | 9081 | 1.594622 | 0.110797 | 0.269585 | FALSE |
| YPEL4 | 15264 | 1.593884 | 0.110962 | 0.269819 | FALSE |
| MORC3 | 9164 | 1.593472 | 0.111054 | 0.269978 | FALSE |
| TRA2B | 6627 | 1.593362 | 0.111079 | 0.269978 | FALSE |
| TMCO3 | 14335 | 1.593161 | 0.111124 | 0.270002 | FALSE |
| DNAJC11 | 272 | 1.592987 | 0.111163 | 0.270002 | FALSE |
| SPIRE1 | 10785 | 1.592855 | 0.111193 | 0.270002 | FALSE |
| FAM182B | 1955 | 1.592028 | 0.111378 | 0.270369 | FALSE |
| CDKN2C | 5138 | 1.591509 | 0.111495 | 0.270521 | FALSE |
| SPHK1 | 6247 | 1.591446 | 0.111509 | 0.270521 | FALSE |
| NEDD9 | 3150 | 1.591442 | 0.11151 | 0.270521 | FALSE |
| RRAD | 10818 | 1.591085 | 0.11159 | 0.270674 | FALSE |
| ZBTB33 | 5161 | 1.590881 | 0.111636 | 0.270696 | FALSE |
| PLCB4 | 13860 | 1.590789 | 0.111657 | 0.270696 | FALSE |
| PHC3 | 4301 | 1.589383 | 0.111974 | 0.271322 | FALSE |
| ADAP1 | 4764 | 1.587851 | 0.11232 | 0.271853 | FALSE |
| LOC641746 | 10927 | 1.586566 | 0.112611 | 0.272473 | FALSE |
| ACER3 | 8837 | 1.586389 | 0.112651 | 0.272486 | FALSE |
| GMPR2 | 6836 | 1.586039 | 0.11273 | 0.272635 | FALSE |
| TNFAIP1 | 9489 | 1.585835 | 0.112777 | 0.272705 | FALSE |
| ZNF770 | 26 | 1.58543 | 0.112869 | 0.272885 | FALSE |
| ZNF396 | 9228 | 1.585183 | 0.112925 | 0.272979 | FALSE |
| SIRT1 | 9086 | 1.584174 | 0.113154 | 0.273369 | FALSE |
| KLF13 | 1777 | 1.584049 | 0.113183 | 0.27339 | FALSE |
| LOC100133091 | 5464 | 1.58396 | 0.113203 | 0.273397 | FALSE |
| TRIM67 | 12266 | 1.583697 | 0.113263 | 0.273499 | FALSE |
| NCOR1P1 | 13594 | 1.582658 | 0.113499 | 0.273902 | FALSE |
| TGM1 | 15035 | 1.582346 | 0.113571 | 0.274031 | FALSE |
| ZNF664 | 6079 | 1.581181 | 0.113837 | 0.274554 | FALSE |
| CNTNAP1 | 2244 | 1.580617 | 0.113966 | 0.274815 | FALSE |
| CADM4 | 15175 | 1.579815 | 0.114149 | 0.275215 | FALSE |
| NFYA | 11034 | 1.57972 | 0.114171 | 0.275225 | FALSE |
| ADAM20P1 | 4922 | 1.579405 | 0.114243 | 0.275308 | FALSE |
| VPS53 | 2022 | 1.578182 | 0.114524 | 0.275786 | FALSE |
| ALMS1P1 | 13516 | 1.577922 | 0.114584 | 0.275879 | FALSE |
| PSME4 | 7047 | 1.577437 | 0.114695 | 0.276105 | FALSE |
| KDSR | 7393 | 1.577298 | 0.114727 | 0.27614 | FALSE |
| TMEM97 | 765 | 1.576931 | 0.114811 | 0.276273 | FALSE |
| ITPRIPL1 | 14981 | 1.576903 | 0.114818 | 0.276273 | FALSE |
| FKBP8 | 11718 | 1.576462 | 0.114919 | 0.276475 | FALSE |
| CSMD1 | 840 | 1.576105 | 0.115002 | 0.276545 | FALSE |
| PDS5B | 4812 | 1.575788 | 0.115075 | 0.276665 | FALSE |
| USP47 | 6226 | 1.575736 | 0.115087 | 0.276665 | FALSE |
| NAPEPLD | 9244 | 1.575321 | 0.115182 | 0.276724 | FALSE |
| PLA2R1 | 15154 | 1.574984 | 0.11526 | 0.276856 | FALSE |
| FAM209A | 5913 | 1.574496 | 0.115373 | 0.276972 | FALSE |
| IL15RA | 674 | 1.574297 | 0.115419 | 0.277037 | FALSE |
| LNP1 | 734 | 1.573652 | 0.115568 | 0.277267 | FALSE |
| DAPK1-IT1 | 15351 | 1.573459 | 0.115613 | 0.277332 | FALSE |
| ELAVL4 | 2501 | 1.573243 | 0.115663 | 0.277347 | FALSE |
| SLC25A32 | 15196 | 1.572914 | 0.115739 | 0.277354 | FALSE |
| BHLHE22 | 7764 | 1.572882 | 0.115746 | 0.277354 | FALSE |
| NGRN | 4015 | 1.572597 | 0.115812 | 0.27747 | FALSE |
| ZNF630 | 15501 | 1.572367 | 0.115865 | 0.277546 | FALSE |
| MYO15A | 15426 | 1.572306 | 0.11588 | 0.277546 | FALSE |
| FAM118B | 3106 | 1.571659 | 0.11603 | 0.277821 | FALSE |
| APOOL | 1911 | 1.57076 | 0.116238 | 0.278156 | FALSE |
| NPPB | 2641 | 1.57065 | 0.116264 | 0.278164 | FALSE |
| MOB1A | 5863 | 1.569494 | 0.116533 | 0.278556 | FALSE |
| TH | 13413 | 1.56938 | 0.116559 | 0.278577 | FALSE |
| TTC25 | 12799 | 1.569276 | 0.116584 | 0.278592 | FALSE |
| TMEM41B | 1573 | 1.568773 | 0.116701 | 0.278745 | FALSE |
| KATNAL2 | 12070 | 1.56829 | 0.116813 | 0.278901 | FALSE |
| VPS36 | 13020 | 1.568089 | 0.11686 | 0.278955 | FALSE |
| RUFY1 | 7103 | 1.567877 | 0.11691 | 0.279031 | FALSE |
| SLC4A11 | 255 | 1.567683 | 0.116955 | 0.279072 | FALSE |
| ATMIN | 190 | 1.567535 | 0.11699 | 0.279094 | FALSE |
| RASSF5 | 12402 | 1.566648 | 0.117197 | 0.279461 | FALSE |
| BTAF1 | 3238 | 1.566646 | 0.117197 | 0.279461 | FALSE |
| GCNA | 14635 | 1.566109 | 0.117323 | 0.279718 | FALSE |
| SPIN3 | 7801 | 1.565621 | 0.117437 | 0.279948 | FALSE |
| LOC100507387 | 8505 | 1.565102 | 0.117559 | 0.280169 | FALSE |
| ISLR | 8542 | 1.563269 | 0.117989 | 0.280911 | FALSE |
| SLC35B3 | 1093 | 1.56321 | 0.118003 | 0.280911 | FALSE |
| TUBE1 | 6136 | 1.562924 | 0.11807 | 0.281029 | FALSE |
| DUT | 7397 | 1.562613 | 0.118144 | 0.28116 | FALSE |
| MIOS | 1330 | 1.56094 | 0.118538 | 0.281884 | FALSE |
| C1orf162 | 11454 | 1.559442 | 0.118892 | 0.282639 | FALSE |
| BRCA1 | 10067 | 1.559176 | 0.118955 | 0.282703 | FALSE |
| MST1P2 | 11249 | 1.55895 | 0.119008 | 0.282738 | FALSE |
| RNF220 | 782 | 1.558856 | 0.11903 | 0.282738 | FALSE |
| UVRAG | 3195 | 1.558808 | 0.119042 | 0.282738 | FALSE |
| TTLL4 | 15020 | 1.557962 | 0.119242 | 0.283128 | FALSE |
| NIPA2 | 9723 | 1.557661 | 0.119314 | 0.283254 | FALSE |
| MDC1 | 10810 | 1.556698 | 0.119542 | 0.283539 | FALSE |
| PCNX1 | 9134 | 1.556044 | 0.119698 | 0.283821 | FALSE |
| ARL5A | 7832 | 1.555763 | 0.119764 | 0.283909 | FALSE |
| CCNT1 | 6988 | 1.555077 | 0.119928 | 0.284199 | FALSE |
| PCGF1 | 15369 | 1.554992 | 0.119948 | 0.284199 | FALSE |
| TAF1D | 11449 | 1.553987 | 0.120188 | 0.284681 | FALSE |
| KIF16B | 14925 | 1.553826 | 0.120226 | 0.284685 | FALSE |
| LINC02012 | 5762 | 1.553283 | 0.120356 | 0.284949 | FALSE |
| RPUSD2 | 791 | 1.553052 | 0.120411 | 0.285012 | FALSE |
| SPHK2 | 1605 | 1.553019 | 0.120419 | 0.285012 | FALSE |
| CEND1 | 4531 | 1.552781 | 0.120475 | 0.285073 | FALSE |
| ZBED4 | 7127 | 1.552718 | 0.120491 | 0.285073 | FALSE |
| CKAP2 | 2045 | 1.552399 | 0.120567 | 0.28519 | FALSE |
| SLAIN2 | 1716 | 1.551683 | 0.120738 | 0.285509 | FALSE |
| TTC30B | 10731 | 1.551082 | 0.120882 | 0.28572 | FALSE |
| LDB1 | 10027 | 1.550026 | 0.121135 | 0.286232 | FALSE |
| PCDHB12 | 9919 | 1.548219 | 0.12157 | 0.287041 | FALSE |
| E4F1 | 5966 | 1.547926 | 0.12164 | 0.287077 | FALSE |
| ELMOD1 | 8150 | 1.546642 | 0.12195 | 0.287721 | FALSE |
| KAZALD1 | 8410 | 1.546085 | 0.122084 | 0.28798 | FALSE |
| XPR1 | 2602 | 1.546035 | 0.122096 | 0.28798 | FALSE |
| ABCC1 | 5167 | 1.54573 | 0.12217 | 0.28811 | FALSE |
| CNEP1R1 | 14877 | 1.545341 | 0.122264 | 0.288246 | FALSE |
| TCEA1 | 15281 | 1.545338 | 0.122265 | 0.288246 | FALSE |
| NOL3 | 7135 | 1.54518 | 0.122303 | 0.288293 | FALSE |
| SAP30L | 9821 | 1.545033 | 0.122338 | 0.288333 | FALSE |
| CLMP | 14061 | 1.544306 | 0.122514 | 0.288661 | FALSE |
| TOX | 9511 | 1.54402 | 0.122583 | 0.288781 | FALSE |
| SIK3 | 5317 | 1.543503 | 0.122709 | 0.288989 | FALSE |
| WDR20 | 4020 | 1.542929 | 0.122848 | 0.289229 | FALSE |
| UBR5 | 8801 | 1.541252 | 0.123255 | 0.290058 | FALSE |
| SSH1 | 10108 | 1.541004 | 0.123316 | 0.290112 | FALSE |
| TTC16 | 11828 | 1.540663 | 0.123399 | 0.290186 | FALSE |
| SEC31A | 706 | 1.540618 | 0.12341 | 0.290186 | FALSE |
| MKLN1 | 3332 | 1.54057 | 0.123421 | 0.290186 | FALSE |
| CAPS2 | 3766 | 1.540306 | 0.123486 | 0.290294 | FALSE |
| REPS1 | 12965 | 1.539062 | 0.123789 | 0.290765 | FALSE |
| METTL17 | 14347 | 1.538146 | 0.124013 | 0.291133 | FALSE |
| UPF3B | 12626 | 1.537399 | 0.124196 | 0.291321 | FALSE |
| SYT11 | 5141 | 1.537371 | 0.124202 | 0.291321 | FALSE |
| BPGM | 14020 | 1.537173 | 0.124251 | 0.291391 | FALSE |
| CTPS2 | 6959 | 1.536312 | 0.124462 | 0.291752 | FALSE |
| ABCC5 | 2941 | 1.536241 | 0.124479 | 0.291752 | FALSE |
| ERCC8 | 12142 | 1.535931 | 0.124555 | 0.291886 | FALSE |
| MTDH | 15345 | 1.535629 | 0.124629 | 0.292016 | FALSE |
| GGT3P | 13854 | 1.535307 | 0.124708 | 0.292114 | FALSE |
| NCS1 | 1226 | 1.534822 | 0.124828 | 0.292349 | FALSE |
| AARS2 | 10506 | 1.534706 | 0.124856 | 0.292372 | FALSE |
| PRMT9 | 8314 | 1.53366 | 0.125113 | 0.292887 | FALSE |
| LSM12 | 1692 | 1.533173 | 0.125233 | 0.293123 | FALSE |
| KATNAL1 | 4609 | 1.53274 | 0.12534 | 0.293178 | FALSE |
| LOC653080 | 1679 | 1.531186 | 0.125723 | 0.293963 | FALSE |
| RNF216 | 3898 | 1.53095 | 0.125782 | 0.294055 | FALSE |
| SPDYE5 | 5575 | 1.530633 | 0.12586 | 0.294062 | FALSE |
| C8orf37 | 13831 | 1.530341 | 0.125932 | 0.294099 | FALSE |
| CYP4B1 | 10394 | 1.530041 | 0.126007 | 0.294141 | FALSE |
| ZNF214 | 3538 | 1.527668 | 0.126595 | 0.295294 | FALSE |
| NANP | 5038 | 1.527278 | 0.126692 | 0.295388 | FALSE |
| SREK1 | 2493 | 1.526966 | 0.126769 | 0.29548 | FALSE |
| ANXA6 | 2270 | 1.525874 | 0.127041 | 0.295893 | FALSE |
| NSMAF | 15042 | 1.525099 | 0.127234 | 0.296191 | FALSE |
| ARHGEF11 | 7301 | 1.525053 | 0.127246 | 0.296191 | FALSE |
| NLGN1 | 1352 | 1.524775 | 0.127315 | 0.296267 | FALSE |
| ALKBH4 | 13325 | 1.52458 | 0.127364 | 0.296336 | FALSE |
| 4-Sep | 13339 | 1.524049 | 0.127496 | 0.2966 | FALSE |
| EIF1B | 1275 | 1.523635 | 0.1276 | 0.296797 | FALSE |
| COL11A2 | 7714 | 1.523103 | 0.127733 | 0.297018 | FALSE |
| PHLDB2 | 46 | 1.522818 | 0.127804 | 0.297139 | FALSE |
| COQ2 | 15355 | 1.5225 | 0.127884 | 0.297236 | FALSE |
| ZNF225 | 4903 | 1.521922 | 0.128029 | 0.297484 | FALSE |
| ANKHD1 | 11841 | 1.521542 | 0.128124 | 0.297661 | FALSE |
| SPINK8 | 104 | 1.520831 | 0.128302 | 0.298012 | FALSE |
| DRP2 | 881 | 1.520408 | 0.128408 | 0.298145 | FALSE |
| GNL3 | 4880 | 1.520044 | 0.1285 | 0.298212 | FALSE |
| LOC105374727 | 8976 | 1.519936 | 0.128527 | 0.298212 | FALSE |
| APBA2 | 10114 | 1.519913 | 0.128533 | 0.298212 | FALSE |
| SFTPA1 | 830 | 1.519205 | 0.128711 | 0.298537 | FALSE |
| NEURL2 | 6544 | 1.518252 | 0.128951 | 0.299049 | FALSE |
| G2E3 | 6029 | 1.51778 | 0.12907 | 0.299237 | FALSE |
| OGDH | 2214 | 1.517702 | 0.12909 | 0.299237 | FALSE |
| ATXN7L3 | 7201 | 1.517453 | 0.129152 | 0.299294 | FALSE |
| ERN1 | 3087 | 1.516996 | 0.129268 | 0.299384 | FALSE |
| PRPF3 | 6319 | 1.516879 | 0.129297 | 0.299408 | FALSE |
| MLPH | 9544 | 1.516099 | 0.129494 | 0.299576 | FALSE |
| SOD2 | 3469 | 1.516083 | 0.129498 | 0.299576 | FALSE |
| CLPX | 465 | 1.515122 | 0.129742 | 0.299992 | FALSE |
| LUC7L | 9347 | 1.514598 | 0.129874 | 0.300175 | FALSE |
| FAM149B1 | 4302 | 1.51452 | 0.129894 | 0.300175 | FALSE |
| STAMBPL1 | 2947 | 1.514436 | 0.129915 | 0.300175 | FALSE |
| CNTLN | 2096 | 1.514348 | 0.129938 | 0.300179 | FALSE |
| BSCL2 | 219 | 1.514266 | 0.129958 | 0.300183 | FALSE |
| SPEF1 | 942 | 1.513033 | 0.130271 | 0.30079 | FALSE |
| STK4-AS1 | 13376 | 1.512917 | 0.130301 | 0.30079 | FALSE |
| LHX1 | 12617 | 1.512886 | 0.130309 | 0.30079 | FALSE |
| ZNF407 | 9481 | 1.512205 | 0.130482 | 0.301081 | FALSE |
| SLC38A9 | 2130 | 1.51212 | 0.130503 | 0.301086 | FALSE |
| CSGALNACT2 | 4897 | 1.511843 | 0.130574 | 0.30116 | FALSE |
| MRPL45 | 14182 | 1.510195 | 0.130994 | 0.30195 | FALSE |
| KDM4C | 6072 | 1.509794 | 0.131096 | 0.302074 | FALSE |
| LINC01137 | 14713 | 1.509756 | 0.131106 | 0.302074 | FALSE |
| CXorf56 | 3882 | 1.509655 | 0.131131 | 0.302089 | FALSE |
| PPM1B | 14349 | 1.509483 | 0.131175 | 0.302101 | FALSE |
| SRSF7 | 10001 | 1.508896 | 0.131325 | 0.302313 | FALSE |
| ZNF283 | 4731 | 1.508182 | 0.131508 | 0.302644 | FALSE |
| TOMM70 | 15076 | 1.508008 | 0.131552 | 0.302702 | FALSE |
| DXO | 12094 | 1.507915 | 0.131576 | 0.302713 | FALSE |
| KRTAP4-12 | 7540 | 1.506523 | 0.131933 | 0.303406 | FALSE |
| EDIL3 | 10353 | 1.506056 | 0.132053 | 0.30363 | FALSE |
| RELA | 8224 | 1.505595 | 0.132171 | 0.303851 | FALSE |
| NFXL1 | 6164 | 1.50553 | 0.132188 | 0.303851 | FALSE |
| LETM2 | 6416 | 1.505446 | 0.132209 | 0.303856 | FALSE |
| SUGT1P3 | 12205 | 1.504927 | 0.132343 | 0.304118 | FALSE |
| EIF4G2 | 12496 | 1.504755 | 0.132387 | 0.304175 | FALSE |
| ZNF132 | 3156 | 1.504506 | 0.132451 | 0.304278 | FALSE |
| ZNF74 | 10912 | 1.504237 | 0.13252 | 0.304368 | FALSE |
| ELAVL2 | 4807 | 1.504126 | 0.132549 | 0.304368 | FALSE |
| ZNF749 | 11695 | 1.503352 | 0.132748 | 0.304707 | FALSE |
| ALG1 | 4457 | 1.503326 | 0.132755 | 0.304707 | FALSE |
| ADPRM | 5124 | 1.502367 | 0.133002 | 0.305095 | FALSE |
| MAP3K7 | 4904 | 1.502261 | 0.13303 | 0.305113 | FALSE |
| CCR10 | 1994 | 1.501122 | 0.133324 | 0.30552 | FALSE |
| CHST4 | 15466 | 1.500778 | 0.133413 | 0.305679 | FALSE |
| CNOT8 | 6334 | 1.500178 | 0.133568 | 0.305845 | FALSE |
| SNHG7 | 105 | 1.50012 | 0.133583 | 0.305845 | FALSE |
| ANAPC10 | 3021 | 1.49996 | 0.133625 | 0.305895 | FALSE |
| UBN1 | 8177 | 1.499881 | 0.133645 | 0.305897 | FALSE |
| RP9P | 10679 | 1.499592 | 0.13372 | 0.305979 | FALSE |
| DGLUCY | 3760 | 1.499208 | 0.13382 | 0.306117 | FALSE |
| THEMIS | 6609 | 1.498332 | 0.134047 | 0.306547 | FALSE |
| RAB30-AS1 | 9647 | 1.497644 | 0.134226 | 0.306866 | FALSE |
| ZNF845 | 4158 | 1.496718 | 0.134467 | 0.307327 | FALSE |
| MRPL42 | 6478 | 1.495939 | 0.13467 | 0.307533 | FALSE |
| DVL3 | 3919 | 1.495675 | 0.134738 | 0.307588 | FALSE |
| MTUS1 | 593 | 1.495337 | 0.134827 | 0.307745 | FALSE |
| KIF15 | 6787 | 1.494576 | 0.135025 | 0.308063 | FALSE |
| PLCL1 | 12601 | 1.494293 | 0.135099 | 0.308187 | FALSE |
| SH3BP2 | 560 | 1.493882 | 0.135206 | 0.30837 | FALSE |
| COX8C | 14961 | 1.493163 | 0.135395 | 0.308591 | FALSE |
| COPB2 | 1865 | 1.491298 | 0.135883 | 0.309369 | FALSE |
| IMPA1P1 | 6559 | 1.491284 | 0.135887 | 0.309369 | FALSE |
| ANTXR2 | 5234 | 1.491185 | 0.135913 | 0.309369 | FALSE |
| DUSP11 | 11283 | 1.490533 | 0.136084 | 0.309666 | FALSE |
| LINC01011 | 8501 | 1.489947 | 0.136238 | 0.309901 | FALSE |
| COPA | 15578 | 1.489914 | 0.136247 | 0.309901 | FALSE |
| RPTOR | 10906 | 1.489089 | 0.136464 | 0.310176 | FALSE |
| HERC1 | 6858 | 1.489079 | 0.136467 | 0.310176 | FALSE |
| ZNF441 | 8867 | 1.489036 | 0.136478 | 0.310176 | FALSE |
| OR2L8 | 8431 | 1.488353 | 0.136658 | 0.310474 | FALSE |
| GTF2A2 | 633 | 1.487377 | 0.136915 | 0.310878 | FALSE |
| HMBS | 11128 | 1.485489 | 0.137414 | 0.311661 | FALSE |
| USP19 | 15300 | 1.484861 | 0.137581 | 0.311936 | FALSE |
| N6AMT1 | 11197 | 1.484633 | 0.137641 | 0.312011 | FALSE |
| TMEM150A | 13526 | 1.484585 | 0.137654 | 0.312011 | FALSE |
| ST13 | 15278 | 1.484411 | 0.1377 | 0.312071 | FALSE |
| FAF1 | 15609 | 1.482995 | 0.138076 | 0.312715 | FALSE |
| LRRC38 | 9697 | 1.482896 | 0.138102 | 0.312715 | FALSE |
| ZNF248 | 4128 | 1.482885 | 0.138105 | 0.312715 | FALSE |
| TNFSF4 | 400 | 1.482674 | 0.138161 | 0.312715 | FALSE |
| ZCCHC14 | 12417 | 1.482572 | 0.138188 | 0.312715 | FALSE |
| SGO2 | 10523 | 1.482514 | 0.138204 | 0.312715 | FALSE |
| PLK1 | 11369 | 1.482366 | 0.138243 | 0.312715 | FALSE |
| BRAP | 8919 | 1.482361 | 0.138244 | 0.312715 | FALSE |
| NEBL | 2769 | 1.482104 | 0.138313 | 0.312824 | FALSE |
| ELOVL6 | 10591 | 1.480937 | 0.138623 | 0.313391 | FALSE |
| TAZ | 13981 | 1.480675 | 0.138693 | 0.313504 | FALSE |
| BMS1P20 | 4635 | 1.480331 | 0.138785 | 0.313666 | FALSE |
| OXCT2 | 4510 | 1.479601 | 0.13898 | 0.31397 | FALSE |
| DCTN4 | 1074 | 1.47919 | 0.13909 | 0.314082 | FALSE |
| GNE | 11514 | 1.478216 | 0.13935 | 0.314536 | FALSE |
| TCAIM | 10597 | 1.478212 | 0.139351 | 0.314536 | FALSE |
| ZC3H7A | 662 | 1.477403 | 0.139568 | 0.314934 | FALSE |
| ITGA2B | 8342 | 1.477095 | 0.13965 | 0.315074 | FALSE |
| TBCK | 8252 | 1.476554 | 0.139795 | 0.315265 | FALSE |
| SYT3 | 6158 | 1.475114 | 0.140182 | 0.315927 | FALSE |
| MKRN7P | 15104 | 1.475085 | 0.14019 | 0.315927 | FALSE |
| TMEM88 | 742 | 1.474966 | 0.140222 | 0.315954 | FALSE |
| ECSIT | 3722 | 1.474869 | 0.140248 | 0.315967 | FALSE |
| DYNC1LI1 | 4981 | 1.474749 | 0.14028 | 0.315994 | FALSE |
| CAMSAP3 | 2674 | 1.474648 | 0.140307 | 0.31601 | FALSE |
| ZNF486 | 8582 | 1.47443 | 0.140366 | 0.316051 | FALSE |
| KRTAP9-4 | 9924 | 1.474086 | 0.140458 | 0.316122 | FALSE |
| LOC728485 | 10631 | 1.472669 | 0.14084 | 0.316754 | FALSE |
| CMTR1 | 12567 | 1.472409 | 0.14091 | 0.316821 | FALSE |
| DCLRE1A | 3881 | 1.470874 | 0.141325 | 0.317519 | FALSE |
| LRBA | 5080 | 1.47074 | 0.141361 | 0.317519 | FALSE |
| ACVR1B | 13994 | 1.47032 | 0.141475 | 0.317725 | FALSE |
| ZFYVE19 | 8144 | 1.469446 | 0.141712 | 0.318165 | FALSE |
| PDCL3 | 8773 | 1.469253 | 0.141764 | 0.318237 | FALSE |
| NLK | 9026 | 1.469072 | 0.141813 | 0.318301 | FALSE |
| AKAIN1 | 5002 | 1.468061 | 0.142088 | 0.31878 | FALSE |
| NR1H2 | 3164 | 1.46736 | 0.142278 | 0.318978 | FALSE |
| CRKL | 13191 | 1.46681 | 0.142428 | 0.319176 | FALSE |
| CTTN | 15048 | 1.466186 | 0.142598 | 0.319419 | FALSE |
| COQ3 | 8521 | 1.465988 | 0.142652 | 0.319495 | FALSE |
| ARMC1 | 7028 | 1.465705 | 0.142729 | 0.319576 | FALSE |
| USF1 | 11228 | 1.465406 | 0.14281 | 0.319712 | FALSE |
| GGTLC1 | 11787 | 1.464803 | 0.142975 | 0.319962 | FALSE |
| NDFIP1 | 2698 | 1.464772 | 0.142983 | 0.319962 | FALSE |
| PPFIA1 | 1348 | 1.464393 | 0.143087 | 0.320118 | FALSE |
| PCDHB15 | 11928 | 1.464344 | 0.1431 | 0.320118 | FALSE |
| TRIT1 | 511 | 1.463089 | 0.143443 | 0.320641 | FALSE |
| KRTAP10-6 | 9578 | 1.462518 | 0.143599 | 0.320836 | FALSE |
| ATP6V1G1 | 6357 | 1.46141 | 0.143903 | 0.321376 | FALSE |
| CCDC184 | 6535 | 1.461235 | 0.143951 | 0.321438 | FALSE |
| PAPOLA | 6438 | 1.459259 | 0.144494 | 0.322558 | FALSE |
| SPTBN4 | 9478 | 1.45873 | 0.144639 | 0.322837 | FALSE |
| ATOH8 | 15296 | 1.458009 | 0.144838 | 0.323188 | FALSE |
| LYG1 | 3041 | 1.456616 | 0.145222 | 0.32387 | FALSE |
| MRPS10 | 15228 | 1.45651 | 0.145252 | 0.32387 | FALSE |
| MLXIPL | 9737 | 1.456191 | 0.14534 | 0.323984 | FALSE |
| MNT | 11904 | 1.456022 | 0.145387 | 0.32404 | FALSE |
| KIF2C | 7262 | 1.455612 | 0.1455 | 0.324109 | FALSE |
| UBXN7 | 2752 | 1.455484 | 0.145535 | 0.324109 | FALSE |
| CCDC152 | 13770 | 1.455437 | 0.145548 | 0.324109 | FALSE |
| MAP4K4 | 7316 | 1.455309 | 0.145584 | 0.324111 | FALSE |
| PHF5A | 6168 | 1.454449 | 0.145822 | 0.324503 | FALSE |
| PLEKHO1 | 12301 | 1.454116 | 0.145914 | 0.324662 | FALSE |
| TTC33 | 15434 | 1.453805 | 0.146 | 0.324808 | FALSE |
| FGF12 | 3436 | 1.453603 | 0.146056 | 0.324852 | FALSE |
| METTL25 | 2228 | 1.453346 | 0.146128 | 0.324952 | FALSE |
| TTR | 9351 | 1.451932 | 0.146521 | 0.32566 | FALSE |
| FGFR1OP2 | 8877 | 1.451341 | 0.146685 | 0.32596 | FALSE |
| CBFA2T2 | 15407 | 1.451133 | 0.146743 | 0.326042 | FALSE |
| CABLES2 | 1075 | 1.450829 | 0.146827 | 0.326137 | FALSE |
| C2orf68 | 281 | 1.450202 | 0.147002 | 0.326433 | FALSE |
| OTUD7B | 8192 | 1.448955 | 0.14735 | 0.327019 | FALSE |
| TADA2B | 14649 | 1.448401 | 0.147505 | 0.32727 | FALSE |
| GMPR | 9696 | 1.448138 | 0.147578 | 0.32734 | FALSE |
| BVES | 7357 | 1.447936 | 0.147635 | 0.327419 | FALSE |
| KCNIP3 | 9406 | 1.447714 | 0.147697 | 0.32751 | FALSE |
| GOLGA2P7 | 10270 | 1.447492 | 0.147759 | 0.327569 | FALSE |
| ATP11A | 15311 | 1.44732 | 0.147807 | 0.327569 | FALSE |
| PDE8A | 3910 | 1.445808 | 0.148231 | 0.328275 | FALSE |
| ACE | 3751 | 1.445594 | 0.148291 | 0.328362 | FALSE |
| SMYD2 | 1662 | 1.445427 | 0.148338 | 0.328419 | FALSE |
| MECP2 | 4207 | 1.445005 | 0.148456 | 0.328635 | FALSE |
| NOP9 | 15255 | 1.444527 | 0.148591 | 0.328793 | FALSE |
| LAMC1 | 2661 | 1.44443 | 0.148618 | 0.328806 | FALSE |
| LRP2BP | 14966 | 1.441869 | 0.149339 | 0.330169 | FALSE |
| QSER1 | 1742 | 1.441011 | 0.149582 | 0.330564 | FALSE |
| CCDC137 | 15353 | 1.439936 | 0.149886 | 0.330916 | FALSE |
| KLHDC2 | 13216 | 1.439848 | 0.14991 | 0.330916 | FALSE |
| FRMD8 | 11091 | 1.439702 | 0.149952 | 0.330927 | FALSE |
| KIFC2 | 11211 | 1.439682 | 0.149957 | 0.330927 | FALSE |
| SPATA6 | 2812 | 1.439558 | 0.149992 | 0.330957 | FALSE |
| ATG4A | 11592 | 1.439085 | 0.150126 | 0.331113 | FALSE |
| ZBTB25 | 4466 | 1.438663 | 0.150246 | 0.331283 | FALSE |
| BCCIP | 13098 | 1.438097 | 0.150407 | 0.33159 | FALSE |
| TATDN3 | 5415 | 1.437597 | 0.150548 | 0.331856 | FALSE |
| C16orf45 | 5243 | 1.437242 | 0.150649 | 0.331985 | FALSE |
| KCNN4 | 3677 | 1.436249 | 0.150932 | 0.332422 | FALSE |
| ALDH18A1 | 15562 | 1.436017 | 0.150998 | 0.332498 | FALSE |
| SECISBP2L | 15559 | 1.435455 | 0.151157 | 0.33273 | FALSE |
| MPV17L | 7928 | 1.434987 | 0.151291 | 0.332976 | FALSE |
| LRRC14 | 3816 | 1.434642 | 0.151389 | 0.333059 | FALSE |
| NCAPD3 | 1335 | 1.43463 | 0.151393 | 0.333059 | FALSE |
| KLHL9 | 14345 | 1.434098 | 0.151544 | 0.333305 | FALSE |
| SP4 | 12737 | 1.434089 | 0.151547 | 0.333305 | FALSE |
| TCFL5 | 13266 | 1.433506 | 0.151713 | 0.33353 | FALSE |
| SRSF6 | 10784 | 1.433407 | 0.151742 | 0.333546 | FALSE |
| ATPAF2 | 3327 | 1.432931 | 0.151878 | 0.333714 | FALSE |
| RPS6KC1 | 9513 | 1.432757 | 0.151927 | 0.333714 | FALSE |
| RAB5C | 1204 | 1.432723 | 0.151937 | 0.333714 | FALSE |
| MIS12 | 10271 | 1.432691 | 0.151946 | 0.333714 | FALSE |
| MCL1 | 1925 | 1.43185 | 0.152187 | 0.334129 | FALSE |
| SPECC1 | 14992 | 1.431559 | 0.15227 | 0.334207 | FALSE |
| SLC35D1 | 3786 | 1.431532 | 0.152278 | 0.334207 | FALSE |
| ARHGAP44 | 4533 | 1.43096 | 0.152442 | 0.334511 | FALSE |
| GMPS | 11132 | 1.430872 | 0.152467 | 0.334511 | FALSE |
| MDH1B | 4391 | 1.430273 | 0.152639 | 0.334717 | FALSE |
| RAD54L | 7868 | 1.429798 | 0.152775 | 0.33488 | FALSE |
| RBL1 | 15462 | 1.429647 | 0.152818 | 0.33488 | FALSE |
| POLG2 | 7354 | 1.427987 | 0.153296 | 0.335828 | FALSE |
| UBQLN1 | 13015 | 1.427724 | 0.153371 | 0.335853 | FALSE |
| SLC5A4 | 14424 | 1.427685 | 0.153383 | 0.335853 | FALSE |
| C1D | 1387 | 1.427532 | 0.153427 | 0.335853 | FALSE |
| GRPEL2 | 3925 | 1.427426 | 0.153457 | 0.335853 | FALSE |
| LOC101927550 | 15111 | 1.426563 | 0.153706 | 0.336303 | FALSE |
| C1orf229 | 13911 | 1.4259 | 0.153897 | 0.336618 | FALSE |
| SSR1 | 15580 | 1.425402 | 0.154041 | 0.3368 | FALSE |
| MTMR11 | 13460 | 1.425164 | 0.15411 | 0.336904 | FALSE |
| MRPL27 | 15003 | 1.424742 | 0.154232 | 0.337064 | FALSE |
| MCM9 | 3285 | 1.424653 | 0.154258 | 0.337064 | FALSE |
| CDPF1 | 9520 | 1.424126 | 0.15441 | 0.337324 | FALSE |
| SLC38A1 | 1541 | 1.423811 | 0.154501 | 0.337476 | FALSE |
| NBEAL2 | 14835 | 1.42361 | 0.154559 | 0.337496 | FALSE |
| PNPLA8 | 8861 | 1.422448 | 0.154896 | 0.33815 | FALSE |
| NRSN1 | 15163 | 1.422151 | 0.154982 | 0.338291 | FALSE |
| LOC150776 | 4735 | 1.421654 | 0.155127 | 0.338511 | FALSE |
| HEG1 | 13372 | 1.419488 | 0.155757 | 0.339521 | FALSE |
| DLC1 | 2430 | 1.419467 | 0.155763 | 0.339521 | FALSE |
| DMTF1 | 4451 | 1.419191 | 0.155843 | 0.339563 | FALSE |
| NIPSNAP3B | 11837 | 1.418969 | 0.155908 | 0.339618 | FALSE |
| SETD3 | 11994 | 1.418941 | 0.155916 | 0.339618 | FALSE |
| C9orf64 | 5510 | 1.418561 | 0.156027 | 0.339765 | FALSE |
| HIST1H4B | 2620 | 1.416863 | 0.156523 | 0.340371 | FALSE |
| ZSCAN31 | 12219 | 1.416244 | 0.156704 | 0.34067 | FALSE |
| HNRNPM | 482 | 1.416127 | 0.156738 | 0.340697 | FALSE |
| CEP164 | 206 | 1.415625 | 0.156885 | 0.340921 | FALSE |
| SMAD5 | 2307 | 1.415502 | 0.156921 | 0.340952 | FALSE |
| UBE2G1 | 5321 | 1.414863 | 0.157109 | 0.341217 | FALSE |
| SYVN1 | 8685 | 1.413919 | 0.157386 | 0.341629 | FALSE |
| CCDC121 | 11219 | 1.413593 | 0.157481 | 0.341789 | FALSE |
| MZF1 | 12318 | 1.412074 | 0.157928 | 0.342521 | FALSE |
| KIF9 | 8329 | 1.411131 | 0.158206 | 0.342981 | FALSE |
| HELLS | 15391 | 1.410907 | 0.158272 | 0.342986 | FALSE |
| NDUFAF5 | 13455 | 1.4109 | 0.158274 | 0.342986 | FALSE |
| FRG1JP | 1949 | 1.410167 | 0.15849 | 0.343368 | FALSE |
| SLC36A4 | 2393 | 1.410153 | 0.158495 | 0.343368 | FALSE |
| GEN1 | 12900 | 1.409995 | 0.158541 | 0.343374 | FALSE |
| P2RX4 | 7417 | 1.409626 | 0.15865 | 0.343524 | FALSE |
| ACBD3 | 14096 | 1.409537 | 0.158676 | 0.343524 | FALSE |
| CCDC144NL | 6512 | 1.408699 | 0.158924 | 0.343918 | FALSE |
| NUP98 | 9143 | 1.408028 | 0.159123 | 0.344252 | FALSE |
| TTC17 | 3444 | 1.407105 | 0.159396 | 0.34461 | FALSE |
| GREB1 | 441 | 1.407098 | 0.159398 | 0.34461 | FALSE |
| TMEM140 | 7874 | 1.406674 | 0.159524 | 0.34477 | FALSE |
| KAT6B | 6275 | 1.406623 | 0.159539 | 0.34477 | FALSE |
| EIF2A | 12175 | 1.406261 | 0.159647 | 0.344861 | FALSE |
| RNF145 | 4435 | 1.405605 | 0.159841 | 0.345082 | FALSE |
| ZNF540 | 7143 | 1.405007 | 0.160019 | 0.345276 | FALSE |
| NPIPB15 | 6882 | 1.404986 | 0.160025 | 0.345276 | FALSE |
| RSPH3 | 12445 | 1.404454 | 0.160184 | 0.345496 | FALSE |
| DYNC2H1 | 3674 | 1.402979 | 0.160623 | 0.346144 | FALSE |
| IFRD1 | 5425 | 1.402881 | 0.160652 | 0.346144 | FALSE |
| TNS1 | 11410 | 1.401385 | 0.161099 | 0.346858 | FALSE |
| RIN2 | 10884 | 1.400899 | 0.161244 | 0.347017 | FALSE |
| SDHA | 8503 | 1.40067 | 0.161313 | 0.347053 | FALSE |
| IREB2 | 9506 | 1.400374 | 0.161401 | 0.347164 | FALSE |
| ANO6 | 7652 | 1.400087 | 0.161487 | 0.347198 | FALSE |
| DIP2B | 13165 | 1.399896 | 0.161544 | 0.347222 | FALSE |
| MYO18A | 3727 | 1.399522 | 0.161657 | 0.347278 | FALSE |
| ZSCAN29 | 5912 | 1.398639 | 0.161921 | 0.347604 | FALSE |
| OGT | 8070 | 1.398605 | 0.161931 | 0.347604 | FALSE |
| NMNAT2 | 12664 | 1.398407 | 0.161991 | 0.347619 | FALSE |
| ATP1A2 | 9264 | 1.39825 | 0.162038 | 0.347672 | FALSE |
| TAF5L | 9349 | 1.397795 | 0.162175 | 0.34787 | FALSE |
| TAF6 | 12129 | 1.397323 | 0.162316 | 0.348126 | FALSE |
| ACSL3 | 965 | 1.396814 | 0.16247 | 0.348322 | FALSE |
| UBL3 | 703 | 1.396811 | 0.16247 | 0.348322 | FALSE |
| GOLGA2P5 | 3186 | 1.396798 | 0.162474 | 0.348322 | FALSE |
| PHF8 | 14240 | 1.396417 | 0.162589 | 0.34852 | FALSE |
| RBM28 | 4127 | 1.395521 | 0.162859 | 0.348983 | FALSE |
| PPP1R3B | 14343 | 1.395342 | 0.162913 | 0.349 | FALSE |
| SMG7 | 11000 | 1.39467 | 0.163115 | 0.349313 | FALSE |
| LPAR1 | 1494 | 1.394116 | 0.163283 | 0.349623 | FALSE |
| EDC4 | 3921 | 1.393894 | 0.16335 | 0.349719 | FALSE |
| ZNF555 | 4546 | 1.393806 | 0.163376 | 0.349728 | FALSE |
| KHNYN | 2618 | 1.393262 | 0.163541 | 0.349936 | FALSE |
| XPOT | 14795 | 1.392223 | 0.163855 | 0.350295 | FALSE |
| FAM185A | 7507 | 1.392189 | 0.163865 | 0.350295 | FALSE |
| PRB4 | 391 | 1.391958 | 0.163935 | 0.350348 | FALSE |
| BCL7A | 13789 | 1.391765 | 0.163994 | 0.350425 | FALSE |
| LNX2 | 6980 | 1.391417 | 0.164099 | 0.350486 | FALSE |
| CFHR3 | 15216 | 1.39085 | 0.164271 | 0.350778 | FALSE |
| UBOX5 | 8930 | 1.39055 | 0.164362 | 0.350826 | FALSE |
| NONO | 15401 | 1.390361 | 0.164419 | 0.350826 | FALSE |
| RBKS | 2409 | 1.390333 | 0.164428 | 0.350826 | FALSE |
| RSBN1L | 10404 | 1.389988 | 0.164533 | 0.350953 | FALSE |
| NUP35 | 3461 | 1.389531 | 0.164671 | 0.351154 | FALSE |
| CCNC | 14955 | 1.389324 | 0.164734 | 0.351175 | FALSE |
| SUPT16H | 3864 | 1.389304 | 0.16474 | 0.351175 | FALSE |
| ZNF710 | 14666 | 1.389213 | 0.164768 | 0.351175 | FALSE |
| MAATS1 | 10862 | 1.389203 | 0.164771 | 0.351175 | FALSE |
| SLC6A1 | 13941 | 1.388795 | 0.164895 | 0.351301 | FALSE |
| ZNF30 | 9841 | 1.388778 | 0.1649 | 0.351301 | FALSE |
| PTPRE | 12374 | 1.388712 | 0.16492 | 0.351301 | FALSE |
| CTTNBP2NL | 4260 | 1.388548 | 0.16497 | 0.351312 | FALSE |
| RNF144B | 367 | 1.387421 | 0.165313 | 0.35185 | FALSE |
| RRP1B | 12724 | 1.387274 | 0.165358 | 0.35185 | FALSE |
| MAP7 | 12342 | 1.387145 | 0.165398 | 0.351886 | FALSE |
| TSHZ3 | 13454 | 1.387023 | 0.165435 | 0.351917 | FALSE |
| PARP12 | 5274 | 1.386939 | 0.16546 | 0.351924 | FALSE |
| CHN2 | 9064 | 1.386502 | 0.165594 | 0.352112 | FALSE |
| CHRM2 | 6290 | 1.386404 | 0.165624 | 0.352127 | FALSE |
| ATF1 | 13161 | 1.385305 | 0.165959 | 0.352638 | FALSE |
| KDELC1 | 5162 | 1.38525 | 0.165976 | 0.352638 | FALSE |
| MAP2K6 | 10172 | 1.385156 | 0.166005 | 0.35265 | FALSE |
| MYH10 | 9379 | 1.384182 | 0.166303 | 0.353154 | FALSE |
| FAM91A1 | 12547 | 1.383182 | 0.166609 | 0.353572 | FALSE |
| MCU | 5043 | 1.383148 | 0.166619 | 0.353572 | FALSE |
| JCAD | 3067 | 1.383036 | 0.166654 | 0.353597 | FALSE |
| HCCS | 2905 | 1.382479 | 0.166825 | 0.353826 | FALSE |
| LYPLA1 | 3275 | 1.381849 | 0.167018 | 0.354177 | FALSE |
| ZNF518A | 5564 | 1.381405 | 0.167154 | 0.354291 | FALSE |
| KBTBD8 | 9630 | 1.381357 | 0.167169 | 0.354291 | FALSE |
| SLC36A1 | 15001 | 1.38106 | 0.167261 | 0.354403 | FALSE |
| STEAP2 | 13996 | 1.380852 | 0.167324 | 0.35449 | FALSE |
| ZC3H14 | 10840 | 1.380662 | 0.167383 | 0.354518 | FALSE |
| CENPC | 3162 | 1.380396 | 0.167465 | 0.354595 | FALSE |
| C6orf136 | 6215 | 1.380024 | 0.167579 | 0.35479 | FALSE |
| DFFB | 2490 | 1.378775 | 0.167964 | 0.35546 | FALSE |
| DHX57 | 9733 | 1.378537 | 0.168038 | 0.355567 | FALSE |
| VASP | 11447 | 1.377033 | 0.168502 | 0.356357 | FALSE |
| GHRLOS | 3299 | 1.376318 | 0.168723 | 0.356632 | FALSE |
| HNRNPA2B1 | 7166 | 1.37594 | 0.16884 | 0.356735 | FALSE |
| ANKRD40 | 12479 | 1.37538 | 0.169014 | 0.357003 | FALSE |
| QTRT2 | 15232 | 1.375255 | 0.169052 | 0.357003 | FALSE |
| USP3 | 9910 | 1.375235 | 0.169059 | 0.357003 | FALSE |
| ADO | 13841 | 1.374915 | 0.169158 | 0.357116 | FALSE |
| LOC100507599 | 4121 | 1.374363 | 0.169329 | 0.357333 | FALSE |
| GVINP1 | 13060 | 1.374244 | 0.169366 | 0.357362 | FALSE |
| ZMYM5 | 4978 | 1.373965 | 0.169453 | 0.357472 | FALSE |
| ALDH5A1 | 429 | 1.37393 | 0.169463 | 0.357472 | FALSE |
| NUP210 | 580 | 1.373675 | 0.169543 | 0.35759 | FALSE |
| ATP1A1 | 6860 | 1.373579 | 0.169572 | 0.357605 | FALSE |
| SAXO2 | 1473 | 1.373134 | 0.169711 | 0.357783 | FALSE |
| LOC341056 | 9929 | 1.37304 | 0.16974 | 0.357783 | FALSE |
| ZNF606 | 13052 | 1.373013 | 0.169748 | 0.357783 | FALSE |
| MAVS | 13367 | 1.372915 | 0.169779 | 0.357799 | FALSE |
| KHDRBS2 | 7279 | 1.371636 | 0.170177 | 0.358589 | FALSE |
| SLC39A9 | 14770 | 1.371118 | 0.170338 | 0.358833 | FALSE |
| ZNF692 | 6195 | 1.370847 | 0.170423 | 0.358914 | FALSE |
| FAM160B1 | 14218 | 1.370516 | 0.170526 | 0.359059 | FALSE |
| AKAP1 | 2267 | 1.369604 | 0.170811 | 0.359495 | FALSE |
| RND2 | 12444 | 1.369594 | 0.170814 | 0.359495 | FALSE |
| TMED7 | 13849 | 1.369322 | 0.170899 | 0.359582 | FALSE |
| TPD52L1 | 2194 | 1.369314 | 0.170901 | 0.359582 | FALSE |
| NOB1 | 14728 | 1.367874 | 0.171352 | 0.360324 | FALSE |
| SLC22A15 | 2772 | 1.367745 | 0.171392 | 0.360324 | FALSE |
| UGGT2 | 12172 | 1.367546 | 0.171454 | 0.360386 | FALSE |
| PTPRCAP | 15470 | 1.367398 | 0.171501 | 0.360386 | FALSE |
| ACP6 | 11594 | 1.367361 | 0.171512 | 0.360386 | FALSE |
| UTP23 | 9429 | 1.367233 | 0.171552 | 0.360419 | FALSE |
| ZNF483 | 5122 | 1.366365 | 0.171824 | 0.360825 | FALSE |
| CECR7 | 2948 | 1.366287 | 0.171849 | 0.360825 | FALSE |
| INTS11 | 8741 | 1.365408 | 0.172125 | 0.361312 | FALSE |
| BUB3 | 2530 | 1.365348 | 0.172144 | 0.361312 | FALSE |
| C2orf92 | 12697 | 1.365182 | 0.172196 | 0.361312 | FALSE |
| FBXO36 | 1231 | 1.365142 | 0.172208 | 0.361312 | FALSE |
| RBBP5 | 1196 | 1.36501 | 0.17225 | 0.36135 | FALSE |
| AZGP1 | 5766 | 1.364429 | 0.172433 | 0.361588 | FALSE |
| SRP68 | 6194 | 1.364329 | 0.172464 | 0.361605 | FALSE |
| TRHDE | 10091 | 1.364137 | 0.172524 | 0.361684 | FALSE |
| PLCG1 | 13276 | 1.363976 | 0.172575 | 0.361741 | FALSE |
| TMEM86B | 3633 | 1.363376 | 0.172764 | 0.361993 | FALSE |
| ZNF300P1 | 1067 | 1.36315 | 0.172835 | 0.362044 | FALSE |
| ANK3 | 9101 | 1.36113 | 0.173473 | 0.363087 | FALSE |
| MEAF6 | 11785 | 1.361013 | 0.17351 | 0.363116 | FALSE |
| KIF25 | 3504 | 1.36081 | 0.173574 | 0.363201 | FALSE |
| ZNF232 | 913 | 1.359879 | 0.173868 | 0.363672 | FALSE |
| HAUS4 | 1230 | 1.359797 | 0.173894 | 0.363677 | FALSE |
| IRF2 | 5353 | 1.359279 | 0.174058 | 0.363839 | FALSE |
| USP34 | 15605 | 1.358715 | 0.174237 | 0.36415 | FALSE |
| FAM19A5 | 6715 | 1.358467 | 0.174316 | 0.364266 | FALSE |
| MYL12A | 7615 | 1.35827 | 0.174378 | 0.364348 | FALSE |
| PTPRD | 13125 | 1.357636 | 0.174579 | 0.364671 | FALSE |
| COL9A3 | 9440 | 1.357232 | 0.174708 | 0.364795 | FALSE |
| ROCK2 | 13959 | 1.357172 | 0.174727 | 0.364795 | FALSE |
| HIRA | 10878 | 1.356983 | 0.174787 | 0.364795 | FALSE |
| SLC19A3 | 15208 | 1.356934 | 0.174802 | 0.364795 | FALSE |
| SLC22A23 | 15110 | 1.356647 | 0.174893 | 0.364937 | FALSE |
| SLC25A15 | 2918 | 1.356025 | 0.175091 | 0.365009 | FALSE |
| ZNF236 | 12284 | 1.355792 | 0.175165 | 0.365045 | FALSE |
| ZNF425 | 4937 | 1.35521 | 0.175351 | 0.365237 | FALSE |
| HPN | 9381 | 1.353919 | 0.175762 | 0.365871 | FALSE |
| STARD8 | 5384 | 1.353746 | 0.175817 | 0.365882 | FALSE |
| TAB3 | 15140 | 1.353584 | 0.175869 | 0.365882 | FALSE |
| ATPAF1 | 9447 | 1.353568 | 0.175874 | 0.365882 | FALSE |
| RPIA | 9780 | 1.352039 | 0.176363 | 0.366731 | FALSE |
| VPS13D | 7736 | 1.351384 | 0.176572 | 0.367062 | FALSE |
| CDC40 | 1827 | 1.351243 | 0.176618 | 0.367065 | FALSE |
| LINC01003 | 12435 | 1.351159 | 0.176645 | 0.367072 | FALSE |
| LRRN3 | 5102 | 1.351077 | 0.176671 | 0.367078 | FALSE |
| OSBPL2 | 3386 | 1.35071 | 0.176788 | 0.367273 | FALSE |
| ELK4 | 1595 | 1.350374 | 0.176896 | 0.367399 | FALSE |
| HLF | 1111 | 1.350095 | 0.176986 | 0.367488 | FALSE |
| ZC3HC1 | 15608 | 1.349724 | 0.177105 | 0.367641 | FALSE |
| ALG3 | 15209 | 1.349718 | 0.177106 | 0.367641 | FALSE |
| RNPC3 | 15240 | 1.349466 | 0.177187 | 0.367711 | FALSE |
| SLC7A5P2 | 13402 | 1.348916 | 0.177364 | 0.36798 | FALSE |
| ZNF740 | 4969 | 1.348302 | 0.177561 | 0.368292 | FALSE |
| CAMTA1 | 3328 | 1.34794 | 0.177678 | 0.368435 | FALSE |
| ANP32D | 6481 | 1.347271 | 0.177893 | 0.368784 | FALSE |
| IER2 | 3018 | 1.345425 | 0.178488 | 0.369675 | FALSE |
| LINC00680 | 618 | 1.345424 | 0.178488 | 0.369675 | FALSE |
| LPAR2 | 7514 | 1.345243 | 0.178547 | 0.369698 | FALSE |
| CCNT2 | 14843 | 1.344667 | 0.178733 | 0.370034 | FALSE |
| SLC20A1 | 15200 | 1.344141 | 0.178903 | 0.370337 | FALSE |
| UHRF1 | 3702 | 1.343587 | 0.179082 | 0.37057 | FALSE |
| FAM133CP | 6782 | 1.343503 | 0.179109 | 0.37057 | FALSE |
| GALNT3 | 600 | 1.343324 | 0.179167 | 0.37057 | FALSE |
| PAPD4 | 11469 | 1.343307 | 0.179173 | 0.37057 | FALSE |
| GPR146 | 7207 | 1.34328 | 0.179181 | 0.37057 | FALSE |
| UBE2CP5 | 7922 | 1.343028 | 0.179263 | 0.37069 | FALSE |
| TMEM14A | 12380 | 1.342479 | 0.179441 | 0.370894 | FALSE |
| SMIM37 | 13903 | 1.342432 | 0.179456 | 0.370894 | FALSE |
| SOX15 | 7427 | 1.342431 | 0.179456 | 0.370894 | FALSE |
| SMG1 | 10886 | 1.342167 | 0.179542 | 0.371022 | FALSE |
| GSR | 10744 | 1.341099 | 0.179888 | 0.371688 | FALSE |
| DEGS1 | 2302 | 1.340887 | 0.179957 | 0.371781 | FALSE |
| YY2 | 4574 | 1.340174 | 0.180189 | 0.372063 | FALSE |
| PAQR3 | 9619 | 1.339883 | 0.180283 | 0.372209 | FALSE |
| LAMTOR3 | 13620 | 1.339466 | 0.180419 | 0.372384 | FALSE |
| C5orf51 | 199 | 1.339403 | 0.18044 | 0.372384 | FALSE |
| ING4 | 2876 | 1.33887 | 0.180613 | 0.372693 | FALSE |
| TLK2 | 10146 | 1.338345 | 0.180784 | 0.372997 | FALSE |
| KIAA0895 | 9328 | 1.336845 | 0.181273 | 0.373756 | FALSE |
| KIAA1958 | 3776 | 1.336705 | 0.181319 | 0.373756 | FALSE |
| FAM208A | 5471 | 1.335759 | 0.181628 | 0.374205 | FALSE |
| KTN1-AS1 | 11180 | 1.335647 | 0.181665 | 0.374205 | FALSE |
| PIK3C2A | 9522 | 1.335597 | 0.181681 | 0.374205 | FALSE |
| EFHD2 | 112 | 1.335222 | 0.181804 | 0.374359 | FALSE |
| ZNF415 | 14175 | 1.334852 | 0.181925 | 0.374472 | FALSE |
| DCAF1 | 14328 | 1.334712 | 0.181971 | 0.37449 | FALSE |
| FBXO9 | 13606 | 1.33459 | 0.182011 | 0.37449 | FALSE |
| PXN | 10097 | 1.334494 | 0.182042 | 0.374494 | FALSE |
| RCBTB1 | 12906 | 1.333948 | 0.182221 | 0.374725 | FALSE |
| BSDC1 | 6402 | 1.333405 | 0.182399 | 0.374992 | FALSE |
| GFRA1 | 5827 | 1.332945 | 0.18255 | 0.375253 | FALSE |
| ADK | 2306 | 1.332215 | 0.18279 | 0.375598 | FALSE |
| CRYGS | 15317 | 1.332016 | 0.182855 | 0.375604 | FALSE |
| SLC10A7 | 8826 | 1.331986 | 0.182865 | 0.375604 | FALSE |
| C16orf72 | 11569 | 1.331757 | 0.18294 | 0.37571 | FALSE |
| DICER1 | 13787 | 1.331633 | 0.182981 | 0.375744 | FALSE |
| KIAA1211L | 7715 | 1.331405 | 0.183056 | 0.375849 | FALSE |
| ADAMTS13 | 823 | 1.331273 | 0.183099 | 0.375888 | FALSE |
| RNF44 | 7520 | 1.331083 | 0.183162 | 0.375918 | FALSE |
| FAM160B2 | 5603 | 1.330463 | 0.183366 | 0.37609 | FALSE |
| ZNF23 | 14986 | 1.330191 | 0.183455 | 0.376224 | FALSE |
| PPP1CB | 9003 | 1.329947 | 0.183536 | 0.37629 | FALSE |
| ZNF366 | 7783 | 1.329662 | 0.18363 | 0.376342 | FALSE |
| NEU4 | 11989 | 1.329526 | 0.183674 | 0.376373 | FALSE |
| ZBTB11 | 1006 | 1.328868 | 0.183892 | 0.376674 | FALSE |
| PPME1 | 1736 | 1.327762 | 0.184257 | 0.37708 | FALSE |
| CDC42EP1 | 14364 | 1.326999 | 0.184509 | 0.377419 | FALSE |
| ZNF512 | 12883 | 1.326832 | 0.184564 | 0.377419 | FALSE |
| ZBBX | 5618 | 1.326818 | 0.184569 | 0.377419 | FALSE |
| HDAC1 | 10149 | 1.326611 | 0.184637 | 0.377477 | FALSE |
| PLD1 | 7986 | 1.326586 | 0.184646 | 0.377477 | FALSE |
| ANKRD34C | 516 | 1.326237 | 0.184761 | 0.377664 | FALSE |
| ZNF75A | 14799 | 1.325909 | 0.18487 | 0.377787 | FALSE |
| HERC6 | 9386 | 1.32572 | 0.184932 | 0.377866 | FALSE |
| HDAC3 | 5319 | 1.325232 | 0.185094 | 0.378097 | FALSE |
| IFIH1 | 7386 | 1.324498 | 0.185338 | 0.378397 | FALSE |
| RNF115 | 9127 | 1.324415 | 0.185365 | 0.378404 | FALSE |
| MAP1B | 5112 | 1.324113 | 0.185466 | 0.378491 | FALSE |
| DUSP1 | 2753 | 1.324067 | 0.185481 | 0.378491 | FALSE |
| TRAP1 | 3754 | 1.323946 | 0.185521 | 0.378524 | FALSE |
| TMEM165 | 7020 | 1.323819 | 0.185563 | 0.37856 | FALSE |
| RPS6KB1 | 15207 | 1.322891 | 0.185872 | 0.37914 | FALSE |
| TMEM14B | 5757 | 1.322373 | 0.186044 | 0.379393 | FALSE |
| ATG4D | 3138 | 1.322087 | 0.186139 | 0.379532 | FALSE |
| SRRM1 | 6459 | 1.322022 | 0.186161 | 0.379532 | FALSE |
| PTCD3 | 7676 | 1.32175 | 0.186251 | 0.379591 | FALSE |
| LRRC45 | 2649 | 1.321292 | 0.186404 | 0.379757 | FALSE |
| ZNF454 | 8428 | 1.320017 | 0.186829 | 0.380448 | FALSE |
| DAPK3 | 688 | 1.319859 | 0.186882 | 0.380506 | FALSE |
| ZNF224 | 14589 | 1.319486 | 0.187007 | 0.380611 | FALSE |
| ACOX1 | 1478 | 1.31794 | 0.187524 | 0.381614 | FALSE |
| DSCAM | 1369 | 1.31722 | 0.187765 | 0.381978 | FALSE |
| DFFA | 14523 | 1.317187 | 0.187776 | 0.381978 | FALSE |
| HELQ | 5417 | 1.316403 | 0.188039 | 0.382264 | FALSE |
| MSANTD4 | 5556 | 1.316214 | 0.188102 | 0.382343 | FALSE |
| QPCT | 7189 | 1.315503 | 0.188341 | 0.382743 | FALSE |
| AAAS | 9483 | 1.315482 | 0.188348 | 0.382743 | FALSE |
| RCN3 | 9726 | 1.313961 | 0.188859 | 0.383433 | FALSE |
| PMS2P9 | 610 | 1.313823 | 0.188906 | 0.383478 | FALSE |
| LYNX1 | 13793 | 1.313334 | 0.18907 | 0.383719 | FALSE |
| ZSWIM5 | 15302 | 1.312775 | 0.189259 | 0.383928 | FALSE |
| ZNF625 | 5898 | 1.3127 | 0.189284 | 0.383928 | FALSE |
| SPOCK1 | 9236 | 1.312654 | 0.1893 | 0.383928 | FALSE |
| CHD1L | 14609 | 1.31251 | 0.189348 | 0.383977 | FALSE |
| RP9 | 15266 | 1.31163 | 0.189645 | 0.38438 | FALSE |
| PRDM1 | 8709 | 1.311341 | 0.189743 | 0.384528 | FALSE |
| SAFB2 | 13923 | 1.310988 | 0.189862 | 0.38462 | FALSE |
| MUC20 | 4099 | 1.310555 | 0.190008 | 0.384767 | FALSE |
| CTHRC1 | 2040 | 1.310234 | 0.190117 | 0.384807 | FALSE |
| PMS2P7 | 6591 | 1.309018 | 0.190528 | 0.38537 | FALSE |
| 7-Sep | 8913 | 1.30847 | 0.190714 | 0.385596 | FALSE |
| GPBP1L1 | 9405 | 1.30819 | 0.190809 | 0.385689 | FALSE |
| MBTPS2 | 4053 | 1.30758 | 0.191016 | 0.386007 | FALSE |
| ELOVL5 | 5566 | 1.307424 | 0.191069 | 0.386064 | FALSE |
| DSE | 10180 | 1.307269 | 0.191121 | 0.386071 | FALSE |
| PERP | 10645 | 1.306535 | 0.191371 | 0.386524 | FALSE |
| ZNF709 | 5288 | 1.306232 | 0.191474 | 0.38664 | FALSE |
| PHF12 | 392 | 1.306221 | 0.191477 | 0.38664 | FALSE |
| NPR2 | 15346 | 1.305924 | 0.191578 | 0.386794 | FALSE |
| ZNF689 | 119 | 1.305463 | 0.191735 | 0.387003 | FALSE |
| UBAC1 | 3317 | 1.305402 | 0.191756 | 0.387003 | FALSE |
| PHYHIPL | 91 | 1.304722 | 0.191988 | 0.38732 | FALSE |
| TBC1D27P | 8917 | 1.304371 | 0.192107 | 0.387511 | FALSE |
| NFE2L2 | 4766 | 1.304225 | 0.192157 | 0.38756 | FALSE |
| AZI2 | 10649 | 1.303816 | 0.192296 | 0.387743 | FALSE |
| RBBP8 | 10841 | 1.303741 | 0.192322 | 0.387744 | FALSE |
| ANKRD18DP | 8467 | 1.303543 | 0.192389 | 0.387771 | FALSE |
| TAF1L | 10405 | 1.303512 | 0.1924 | 0.387771 | FALSE |
| PDCD4 | 10196 | 1.303474 | 0.192413 | 0.387771 | FALSE |
| ZNF397 | 9799 | 1.303023 | 0.192567 | 0.387988 | FALSE |
| CRY1 | 6656 | 1.302877 | 0.192617 | 0.388013 | FALSE |
| RELL1 | 6318 | 1.302841 | 0.192629 | 0.388013 | FALSE |
| SPRY2 | 473 | 1.302584 | 0.192717 | 0.38814 | FALSE |
| VIPR1 | 9881 | 1.301992 | 0.192919 | 0.388447 | FALSE |
| LOC100233156 | 4713 | 1.301496 | 0.193089 | 0.388652 | FALSE |
| PHTF2 | 6852 | 1.301476 | 0.193096 | 0.388652 | FALSE |
| RPH3A | 8845 | 1.300818 | 0.193321 | 0.389056 | FALSE |
| ATRAID | 2104 | 1.299832 | 0.193659 | 0.389635 | FALSE |
| AARD | 6371 | 1.299315 | 0.193836 | 0.389886 | FALSE |
| CLIP1 | 10297 | 1.29925 | 0.193858 | 0.389886 | FALSE |
| GLDN | 12548 | 1.299048 | 0.193927 | 0.389975 | FALSE |
| RAB40C | 9137 | 1.298789 | 0.194016 | 0.390067 | FALSE |
| BRAF | 2960 | 1.298471 | 0.194126 | 0.390073 | FALSE |
| DESI2 | 15628 | 1.298095 | 0.194255 | 0.390209 | FALSE |
| CGGBP1 | 13277 | 1.297931 | 0.194311 | 0.390245 | FALSE |
| EHD2 | 14060 | 1.297799 | 0.194356 | 0.390286 | FALSE |
| CYTH1 | 7367 | 1.297426 | 0.194485 | 0.390423 | FALSE |
| LINC00632 | 2511 | 1.297389 | 0.194497 | 0.390423 | FALSE |
| PCSK7 | 2034 | 1.297382 | 0.1945 | 0.390423 | FALSE |
| NOXA1 | 7493 | 1.296956 | 0.194646 | 0.390598 | FALSE |
| PAPD7 | 13258 | 1.29672 | 0.194728 | 0.390667 | FALSE |
| PLSCR1 | 836 | 1.296666 | 0.194746 | 0.390667 | FALSE |
| MRPL58 | 9785 | 1.296437 | 0.194825 | 0.390775 | FALSE |
| TIRAP | 5414 | 1.29592 | 0.195003 | 0.391082 | FALSE |
| ADGRL4 | 6770 | 1.295844 | 0.195029 | 0.391084 | FALSE |
| PXK | 14557 | 1.2952 | 0.195251 | 0.391429 | FALSE |
| ZNF101 | 12285 | 1.293635 | 0.195792 | 0.39206 | FALSE |
| ZNF141 | 3281 | 1.293258 | 0.195922 | 0.39227 | FALSE |
| THOC5 | 2942 | 1.291508 | 0.196528 | 0.393282 | FALSE |
| ZHX3 | 6460 | 1.290847 | 0.196757 | 0.39341 | FALSE |
| MSANTD3 | 4420 | 1.290643 | 0.196827 | 0.393479 | FALSE |
| COPB1 | 10026 | 1.290194 | 0.196983 | 0.39369 | FALSE |
| TUBGCP5 | 12672 | 1.289125 | 0.197355 | 0.394281 | FALSE |
| DGCR2 | 14544 | 1.288972 | 0.197408 | 0.394336 | FALSE |
| ORMDL1 | 735 | 1.288723 | 0.197494 | 0.394431 | FALSE |
| PCDHB7 | 3895 | 1.28869 | 0.197506 | 0.394431 | FALSE |
| ZNF532 | 8317 | 1.288025 | 0.197737 | 0.394592 | FALSE |
| NFX1 | 9074 | 1.288023 | 0.197738 | 0.394592 | FALSE |
| MAP3K1 | 10265 | 1.287436 | 0.197942 | 0.394826 | FALSE |
| CUL3 | 3690 | 1.286962 | 0.198108 | 0.395078 | FALSE |
| COQ10A | 5525 | 1.286386 | 0.198308 | 0.395428 | FALSE |
| LOC105377590 | 3774 | 1.285245 | 0.198707 | 0.39602 | FALSE |
| KLHL4 | 7943 | 1.28495 | 0.19881 | 0.396074 | FALSE |
| UBAC2 | 2415 | 1.284829 | 0.198852 | 0.396108 | FALSE |
| KCNJ11 | 595 | 1.283356 | 0.199367 | 0.396842 | FALSE |
| ANXA3 | 6374 | 1.283291 | 0.19939 | 0.396842 | FALSE |
| ACADM | 2216 | 1.283275 | 0.199396 | 0.396842 | FALSE |
| ANAPC7 | 15343 | 1.283145 | 0.199441 | 0.396842 | FALSE |
| OLFM1 | 3681 | 1.283122 | 0.199449 | 0.396842 | FALSE |
| HNRNPDL | 2032 | 1.282736 | 0.199585 | 0.397061 | FALSE |
| TRDMT1 | 11169 | 1.282279 | 0.199745 | 0.397329 | FALSE |
| CIC | 123 | 1.281977 | 0.199851 | 0.397489 | FALSE |
| CFAP53 | 14944 | 1.281348 | 0.200071 | 0.397777 | FALSE |
| QDPR | 1634 | 1.280766 | 0.200276 | 0.398132 | FALSE |
| NEK9 | 7907 | 1.279675 | 0.200659 | 0.398626 | FALSE |
| ZSCAN26 | 10038 | 1.279624 | 0.200677 | 0.398626 | FALSE |
| GON4L | 12871 | 1.279134 | 0.20085 | 0.398766 | FALSE |
| TDRP | 7758 | 1.278897 | 0.200933 | 0.398881 | FALSE |
| ZNF787 | 14431 | 1.278516 | 0.201068 | 0.399097 | FALSE |
| TOP1 | 11270 | 1.277637 | 0.201377 | 0.399509 | FALSE |
| CDK9 | 7543 | 1.277233 | 0.20152 | 0.399741 | FALSE |
| UGP2 | 2595 | 1.277023 | 0.201594 | 0.399838 | FALSE |
| ZNF776 | 563 | 1.27691 | 0.201634 | 0.399866 | FALSE |
| ZNF493 | 7639 | 1.275817 | 0.20202 | 0.40053 | FALSE |
| NCDN | 11208 | 1.274086 | 0.202633 | 0.40144 | FALSE |
| BMS1 | 3205 | 1.273718 | 0.202763 | 0.401495 | FALSE |
| HELB | 10820 | 1.273542 | 0.202826 | 0.401567 | FALSE |
| WHAMMP2 | 1180 | 1.273399 | 0.202876 | 0.401589 | FALSE |
| TCOF1 | 8819 | 1.273366 | 0.202888 | 0.401589 | FALSE |
| RUNX2 | 1609 | 1.273057 | 0.202998 | 0.401755 | FALSE |
| SLC32A1 | 6771 | 1.272409 | 0.203228 | 0.401961 | FALSE |
| SLC25A13 | 13659 | 1.27232 | 0.203259 | 0.401961 | FALSE |
| GABPB1-AS1 | 6204 | 1.272263 | 0.20328 | 0.401961 | FALSE |
| TMEM99 | 12326 | 1.271985 | 0.203378 | 0.401961 | FALSE |
| SLC35G5 | 5446 | 1.271786 | 0.203449 | 0.402038 | FALSE |
| EFL1P1 | 15360 | 1.270587 | 0.203876 | 0.402779 | FALSE |
| HYOU1 | 11595 | 1.270495 | 0.203908 | 0.402792 | FALSE |
| GPSM2 | 10055 | 1.269786 | 0.204161 | 0.403189 | FALSE |
| CREBL2 | 9566 | 1.269229 | 0.204359 | 0.403429 | FALSE |
| VRK2 | 51 | 1.268461 | 0.204633 | 0.403918 | FALSE |
| PCDHB17P | 11291 | 1.268327 | 0.204681 | 0.403928 | FALSE |
| INHBA | 8476 | 1.267915 | 0.204828 | 0.403984 | FALSE |
| CCNG1 | 7860 | 1.26776 | 0.204884 | 0.403984 | FALSE |
| SAMHD1 | 7707 | 1.267717 | 0.204899 | 0.403984 | FALSE |
| ARL17B | 15044 | 1.267589 | 0.204945 | 0.404023 | FALSE |
| MCM3AP-AS1 | 5318 | 1.266752 | 0.205244 | 0.404409 | FALSE |
| PHRF1 | 5439 | 1.265722 | 0.205613 | 0.404896 | FALSE |
| SLC22A5 | 9013 | 1.265699 | 0.205621 | 0.404896 | FALSE |
| SH3PXD2A | 14249 | 1.265612 | 0.205652 | 0.404907 | FALSE |
| ZNF777 | 4997 | 1.263637 | 0.20636 | 0.405994 | FALSE |
| HIGD1B | 3392 | 1.263202 | 0.206517 | 0.40621 | FALSE |
| NDC1 | 14881 | 1.26259 | 0.206737 | 0.406428 | FALSE |
| FRYL | 7063 | 1.262459 | 0.206784 | 0.406469 | FALSE |
| LOC101928068 | 7183 | 1.262279 | 0.206848 | 0.406539 | FALSE |
| INIP | 15004 | 1.262216 | 0.206871 | 0.406539 | FALSE |
| SCAF4 | 5506 | 1.261137 | 0.207259 | 0.407251 | FALSE |
| FAM171A1 | 4869 | 1.260605 | 0.207451 | 0.407428 | FALSE |
| PDXK | 3442 | 1.260525 | 0.20748 | 0.407428 | FALSE |
| RBM25 | 14396 | 1.260411 | 0.207521 | 0.407458 | FALSE |
| ANKRD52 | 8788 | 1.259778 | 0.207749 | 0.407841 | FALSE |
| SFR1 | 2445 | 1.259243 | 0.207943 | 0.407859 | FALSE |
| AP1S3 | 12276 | 1.258256 | 0.208299 | 0.408358 | FALSE |
| LOC100133050 | 3092 | 1.258254 | 0.2083 | 0.408358 | FALSE |
| GTF3C2 | 13232 | 1.258201 | 0.208319 | 0.408358 | FALSE |
| ALMS1 | 9410 | 1.257114 | 0.208712 | 0.408975 | FALSE |
| THOC2 | 14871 | 1.256907 | 0.208787 | 0.40907 | FALSE |
| SRPRA | 12596 | 1.25675 | 0.208844 | 0.40913 | FALSE |
| DNAJC21 | 3600 | 1.256032 | 0.209104 | 0.409384 | FALSE |
| WRAP73 | 4585 | 1.255944 | 0.209136 | 0.409395 | FALSE |
| TMX2 | 8161 | 1.255458 | 0.209313 | 0.409637 | FALSE |
| WDR60 | 8673 | 1.255161 | 0.20942 | 0.409695 | FALSE |
| ARPIN | 9814 | 1.255062 | 0.209456 | 0.409695 | FALSE |
| CCDC186 | 2783 | 1.254205 | 0.209768 | 0.410066 | FALSE |
| XXYLT1 | 11797 | 1.253753 | 0.209932 | 0.410284 | FALSE |
| ARMC9 | 10340 | 1.253592 | 0.20999 | 0.410347 | FALSE |
| DZANK1 | 1890 | 1.252529 | 0.210377 | 0.411001 | FALSE |
| RNASEL | 14733 | 1.252045 | 0.210553 | 0.411191 | FALSE |
| ABHD12 | 12805 | 1.251911 | 0.210602 | 0.411235 | FALSE |
| STX8 | 7111 | 1.251577 | 0.210724 | 0.41137 | FALSE |
| ARFGAP1 | 1397 | 1.250917 | 0.210965 | 0.411788 | FALSE |
| FCF1 | 2258 | 1.24964 | 0.211431 | 0.412647 | FALSE |
| PCP4L1 | 2662 | 1.249463 | 0.211496 | 0.412722 | FALSE |
| ZNF154 | 1920 | 1.248723 | 0.211766 | 0.413095 | FALSE |
| FOPNL | 7587 | 1.248221 | 0.21195 | 0.413366 | FALSE |
| ZNF880 | 3836 | 1.247789 | 0.212108 | 0.413607 | FALSE |
| CENPBD1 | 2325 | 1.246164 | 0.212704 | 0.414562 | FALSE |
| RBM8A | 5619 | 1.245908 | 0.212798 | 0.414634 | FALSE |
| POMT1 | 11950 | 1.245847 | 0.212821 | 0.414634 | FALSE |
| ETV5 | 7503 | 1.245464 | 0.212961 | 0.414805 | FALSE |
| TBX1 | 12587 | 1.244719 | 0.213235 | 0.415131 | FALSE |
| GRAP | 11033 | 1.244353 | 0.21337 | 0.415182 | FALSE |
| SPPL2A | 15010 | 1.244186 | 0.213431 | 0.415182 | FALSE |
| FAR1 | 3862 | 1.244178 | 0.213434 | 0.415182 | FALSE |
| IGFBPL1 | 10561 | 1.244142 | 0.213447 | 0.415182 | FALSE |
| PIP5K1A | 454 | 1.244057 | 0.213479 | 0.415192 | FALSE |
| TMEM214 | 11181 | 1.243809 | 0.21357 | 0.415318 | FALSE |
| SIPA1L2 | 14543 | 1.243326 | 0.213748 | 0.415612 | FALSE |
| PYGM | 14621 | 1.243011 | 0.213864 | 0.415786 | FALSE |
| SETX | 12314 | 1.242176 | 0.214172 | 0.416085 | FALSE |
| CACNG2 | 8938 | 1.24216 | 0.214178 | 0.416085 | FALSE |
| C2orf72 | 5434 | 1.241886 | 0.214279 | 0.416126 | FALSE |
| RAB6B | 8511 | 1.24176 | 0.214325 | 0.416165 | FALSE |
| YTHDC2 | 9722 | 1.241194 | 0.214534 | 0.416416 | FALSE |
| ZC3H6 | 11214 | 1.240647 | 0.214736 | 0.416704 | FALSE |
| LDB3 | 13397 | 1.240291 | 0.214868 | 0.416784 | FALSE |
| IQCH-AS1 | 12769 | 1.240135 | 0.214925 | 0.416813 | FALSE |
| LETM1 | 8412 | 1.238881 | 0.21539 | 0.417506 | FALSE |
| AIFM1 | 6949 | 1.238781 | 0.215427 | 0.417526 | FALSE |
| PAQR4 | 4425 | 1.238597 | 0.215495 | 0.417606 | FALSE |
| RACGAP1 | 7865 | 1.237691 | 0.215831 | 0.41805 | FALSE |
| FOCAD | 79 | 1.237473 | 0.215912 | 0.418155 | FALSE |
| ASPSCR1 | 10073 | 1.236643 | 0.21622 | 0.418648 | FALSE |
| PIDD1 | 1229 | 1.236314 | 0.216342 | 0.418771 | FALSE |
| NOVA1 | 11616 | 1.236256 | 0.216363 | 0.418771 | FALSE |
| MAD1L1 | 1882 | 1.235729 | 0.216559 | 0.418971 | FALSE |
| BRD1 | 10113 | 1.235583 | 0.216614 | 0.418971 | FALSE |
| FAM168A | 15526 | 1.235565 | 0.21662 | 0.418971 | FALSE |
| FBXL17 | 12630 | 1.233539 | 0.217375 | 0.419979 | FALSE |
| LOC441601 | 3718 | 1.233053 | 0.217556 | 0.420194 | FALSE |
| PWARSN | 3518 | 1.232652 | 0.217706 | 0.420426 | FALSE |
| ASF1A | 8680 | 1.232214 | 0.217869 | 0.420591 | FALSE |
| CEP57 | 2535 | 1.231149 | 0.218267 | 0.421188 | FALSE |
| ANAPC4 | 11619 | 1.229884 | 0.218741 | 0.421857 | FALSE |
| CCDC18-AS1 | 13548 | 1.229507 | 0.218882 | 0.422025 | FALSE |
| TXLNA | 4595 | 1.228947 | 0.219092 | 0.422378 | FALSE |
| SLC25A51 | 14839 | 1.228329 | 0.219323 | 0.422732 | FALSE |
| DST | 11979 | 1.22829 | 0.219338 | 0.422732 | FALSE |
| BLMH | 9766 | 1.228241 | 0.219357 | 0.422732 | FALSE |
| SLC12A2 | 14607 | 1.228045 | 0.21943 | 0.422821 | FALSE |
| LRP11 | 14296 | 1.227636 | 0.219584 | 0.423065 | FALSE |
| RASA2 | 8303 | 1.227198 | 0.219748 | 0.42319 | FALSE |
| DYRK1A | 1043 | 1.227175 | 0.219757 | 0.42319 | FALSE |
| ISOC2 | 8472 | 1.226969 | 0.219834 | 0.423287 | FALSE |
| ATAD3C | 5373 | 1.226788 | 0.219902 | 0.423314 | FALSE |
| STAT3 | 10890 | 1.226661 | 0.21995 | 0.423354 | FALSE |
| UTP14C | 11566 | 1.22387 | 0.221001 | 0.424959 | FALSE |
| ADAM10 | 2311 | 1.223398 | 0.221179 | 0.425249 | FALSE |
| ATF2 | 15525 | 1.223001 | 0.221329 | 0.425485 | FALSE |
| ATP1B1 | 6468 | 1.222715 | 0.221437 | 0.425562 | FALSE |
| SYBU | 2961 | 1.222496 | 0.22152 | 0.425562 | FALSE |
| GTF3C3 | 12419 | 1.222253 | 0.221612 | 0.425562 | FALSE |
| NUAK1 | 15473 | 1.222247 | 0.221614 | 0.425562 | FALSE |
| BARD1 | 2151 | 1.222246 | 0.221615 | 0.425562 | FALSE |
| SMG6 | 14339 | 1.221927 | 0.221735 | 0.425615 | FALSE |
| PPM1L | 12092 | 1.220621 | 0.22223 | 0.426135 | FALSE |
| GUSB | 3671 | 1.219738 | 0.222564 | 0.4266 | FALSE |
| ANKRD13C | 9503 | 1.219332 | 0.222718 | 0.426723 | FALSE |
| ATP1B3 | 10577 | 1.219267 | 0.222743 | 0.426723 | FALSE |
| LOC100128361 | 4085 | 1.219083 | 0.222813 | 0.426745 | FALSE |
| CFH | 6642 | 1.21901 | 0.22284 | 0.426745 | FALSE |
| TRMT13 | 10997 | 1.21889 | 0.222886 | 0.426745 | FALSE |
| PMS2P4 | 10023 | 1.218754 | 0.222938 | 0.426792 | FALSE |
| CLCN2 | 5481 | 1.218321 | 0.223102 | 0.427054 | FALSE |
| CNTNAP5 | 304 | 1.217076 | 0.223575 | 0.427654 | FALSE |
| TARS2 | 11626 | 1.216864 | 0.223656 | 0.42766 | FALSE |
| NPEPPS | 11222 | 1.216678 | 0.223727 | 0.427727 | FALSE |
| FAM86C2P | 2945 | 1.216555 | 0.223774 | 0.427764 | FALSE |
| HBEGF | 6747 | 1.21644 | 0.223817 | 0.427795 | FALSE |
| SUV39H2 | 6337 | 1.216252 | 0.223889 | 0.42788 | FALSE |
| SHISA8 | 12668 | 1.216173 | 0.223919 | 0.427885 | FALSE |
| BRICD5 | 7092 | 1.215803 | 0.22406 | 0.428102 | FALSE |
| LOC100270746 | 3395 | 1.215587 | 0.224142 | 0.428181 | FALSE |
| DLL1 | 7136 | 1.2153 | 0.224252 | 0.428259 | FALSE |
| IVNS1ABP | 4669 | 1.215085 | 0.224334 | 0.428363 | FALSE |
| REEP6 | 939 | 1.213067 | 0.225104 | 0.429415 | FALSE |
| FAM167B | 1820 | 1.212796 | 0.225208 | 0.429508 | FALSE |
| HIST1H4I | 12423 | 1.212326 | 0.225388 | 0.429774 | FALSE |
| PRSS16 | 15107 | 1.212239 | 0.225421 | 0.429774 | FALSE |
| CHORDC1 | 5656 | 1.212128 | 0.225463 | 0.429774 | FALSE |
| ZNF426 | 5091 | 1.212071 | 0.225485 | 0.429774 | FALSE |
| RALYL | 14719 | 1.211715 | 0.225621 | 0.42997 | FALSE |
| NUP58 | 324 | 1.20918 | 0.226594 | 0.431466 | FALSE |
| SKP1P2 | 12826 | 1.208843 | 0.226723 | 0.431636 | FALSE |
| KIAA1324 | 5259 | 1.20864 | 0.226801 | 0.431666 | FALSE |
| LINC01481 | 4926 | 1.20862 | 0.226809 | 0.431666 | FALSE |
| RARG | 6785 | 1.207871 | 0.227097 | 0.431929 | FALSE |
| MAP2K5 | 14614 | 1.207757 | 0.227141 | 0.431929 | FALSE |
| BBS5 | 13918 | 1.207569 | 0.227213 | 0.431962 | FALSE |
| GRIPAP1 | 13107 | 1.206812 | 0.227505 | 0.432301 | FALSE |
| POLR3A | 7458 | 1.206245 | 0.227723 | 0.432511 | FALSE |
| AFDN | 8666 | 1.205975 | 0.227827 | 0.432656 | FALSE |
| RAVER1 | 6123 | 1.205594 | 0.227974 | 0.432882 | FALSE |
| UCK2 | 9395 | 1.205284 | 0.228094 | 0.432905 | FALSE |
| HLTF | 10185 | 1.204751 | 0.228299 | 0.433197 | FALSE |
| EIF4ENIF1 | 1660 | 1.204734 | 0.228306 | 0.433197 | FALSE |
| INTS2 | 15009 | 1.204373 | 0.228445 | 0.433356 | FALSE |
| CDC37 | 7484 | 1.204049 | 0.228571 | 0.433541 | FALSE |
| EDN3 | 4643 | 1.203776 | 0.228676 | 0.433689 | FALSE |
| FASTK | 13488 | 1.203666 | 0.228719 | 0.43371 | FALSE |
| DEPDC1 | 6543 | 1.202547 | 0.229152 | 0.434342 | FALSE |
| FH | 14923 | 1.202526 | 0.22916 | 0.434342 | FALSE |
| ZNF235 | 13943 | 1.202202 | 0.229285 | 0.434527 | FALSE |
| FARP2 | 1689 | 1.201837 | 0.229427 | 0.434658 | FALSE |
| CACNA1A | 10579 | 1.201809 | 0.229438 | 0.434658 | FALSE |
| EMC1 | 8886 | 1.201304 | 0.229633 | 0.434976 | FALSE |
| MRM1 | 19 | 1.200674 | 0.229878 | 0.435346 | FALSE |
| ZNF541 | 13342 | 1.200511 | 0.229941 | 0.435346 | FALSE |
| VGLL4 | 3747 | 1.200506 | 0.229943 | 0.435346 | FALSE |
| SMAD7 | 5673 | 1.200435 | 0.22997 | 0.435346 | FALSE |
| MAMDC4 | 4239 | 1.20037 | 0.229996 | 0.435346 | FALSE |
| SLC15A4 | 4194 | 1.200238 | 0.230047 | 0.43539 | FALSE |
| ABCA7 | 10156 | 1.199322 | 0.230403 | 0.435905 | FALSE |
| PDGFD | 10923 | 1.199095 | 0.230491 | 0.435932 | FALSE |
| PCDH12 | 13417 | 1.199071 | 0.2305 | 0.435932 | FALSE |
| SLC22A18AS | 7245 | 1.198895 | 0.230569 | 0.436008 | FALSE |
| FANCG | 7621 | 1.198805 | 0.230604 | 0.436022 | FALSE |
| RFWD3 | 11443 | 1.198425 | 0.230752 | 0.436249 | FALSE |
| FZD5 | 5295 | 1.197888 | 0.230961 | 0.436503 | FALSE |
| ZNF548 | 209 | 1.197831 | 0.230983 | 0.436503 | FALSE |
| GRM8 | 11937 | 1.197721 | 0.231026 | 0.436503 | FALSE |
| POM121L12 | 2646 | 1.196651 | 0.231443 | 0.437132 | FALSE |
| CD300LD | 12024 | 1.196552 | 0.231481 | 0.437152 | FALSE |
| FBXL6 | 6505 | 1.196321 | 0.231571 | 0.437217 | FALSE |
| CNOT6 | 15286 | 1.195667 | 0.231827 | 0.437487 | FALSE |
| CDKN2AIP | 7632 | 1.195301 | 0.231969 | 0.437685 | FALSE |
| PGBD1 | 2046 | 1.194974 | 0.232097 | 0.437839 | FALSE |
| WHAMMP3 | 9652 | 1.194466 | 0.232296 | 0.43812 | FALSE |
| SLC16A14 | 12428 | 1.19445 | 0.232302 | 0.43812 | FALSE |
| PHF6 | 4825 | 1.193769 | 0.232568 | 0.438517 | FALSE |
| PATJ | 9959 | 1.1927 | 0.232987 | 0.4392 | FALSE |
| DDX31 | 4165 | 1.191781 | 0.233347 | 0.439667 | FALSE |
| CD59 | 2907 | 1.191268 | 0.233548 | 0.439887 | FALSE |
| KMT2A | 14969 | 1.190566 | 0.233824 | 0.440204 | FALSE |
| ADAM23 | 15498 | 1.189772 | 0.234136 | 0.440675 | FALSE |
| PER1 | 11037 | 1.189036 | 0.234426 | 0.441141 | FALSE |
| MXD4 | 6278 | 1.188999 | 0.23444 | 0.441141 | FALSE |
| MED18 | 2866 | 1.18818 | 0.234763 | 0.441582 | FALSE |
| NEIL2 | 5946 | 1.188097 | 0.234795 | 0.441582 | FALSE |
| LARP1B | 8382 | 1.187432 | 0.235057 | 0.441975 | FALSE |
| COL4A4 | 12098 | 1.187371 | 0.235081 | 0.441975 | FALSE |
| IQSEC1 | 1361 | 1.187108 | 0.235185 | 0.442018 | FALSE |
| POGK | 15098 | 1.1865 | 0.235425 | 0.442302 | FALSE |
| TMC7 | 8082 | 1.186427 | 0.235454 | 0.442303 | FALSE |
| DESI1 | 114 | 1.185755 | 0.235719 | 0.442642 | FALSE |
| AQP11 | 7151 | 1.185265 | 0.235913 | 0.442953 | FALSE |
| ZNF112 | 13739 | 1.185009 | 0.236014 | 0.443019 | FALSE |
| TK2 | 5424 | 1.184866 | 0.23607 | 0.443019 | FALSE |
| TXNRD2 | 12006 | 1.184817 | 0.23609 | 0.443019 | FALSE |
| OR2L13 | 15509 | 1.184572 | 0.236187 | 0.443095 | FALSE |
| CLDN3 | 14945 | 1.183921 | 0.236444 | 0.443413 | FALSE |
| EDNRA | 10120 | 1.18371 | 0.236528 | 0.443468 | FALSE |
| GPR162 | 2326 | 1.182781 | 0.236896 | 0.443974 | FALSE |
| C9orf85 | 3749 | 1.182743 | 0.236911 | 0.443974 | FALSE |
| RBFOX1 | 14279 | 1.182576 | 0.236977 | 0.443991 | FALSE |
| SLC22A9 | 7599 | 1.182314 | 0.237081 | 0.44408 | FALSE |
| CDK12 | 10935 | 1.1822 | 0.237126 | 0.444092 | FALSE |
| TAF1B | 15271 | 1.182122 | 0.237157 | 0.444092 | FALSE |
| TMED2 | 9227 | 1.182083 | 0.237173 | 0.444092 | FALSE |
| EIF2B5 | 8778 | 1.180815 | 0.237676 | 0.444715 | FALSE |
| FOXK1 | 9602 | 1.180428 | 0.23783 | 0.444949 | FALSE |
| ZNF732 | 10586 | 1.180208 | 0.237917 | 0.44506 | FALSE |
| IQSEC3 | 5956 | 1.180024 | 0.237991 | 0.445143 | FALSE |
| MAGI2 | 1612 | 1.179703 | 0.238118 | 0.445254 | FALSE |
| ZNF605 | 14531 | 1.179571 | 0.238171 | 0.445267 | FALSE |
| LOC105274304 | 8860 | 1.179432 | 0.238226 | 0.445278 | FALSE |
| TPM4 | 6766 | 1.179413 | 0.238234 | 0.445278 | FALSE |
| NIPBL-AS1 | 13246 | 1.179295 | 0.238281 | 0.445313 | FALSE |
| MREG | 828 | 1.179102 | 0.238358 | 0.445403 | FALSE |
| PPIC | 3629 | 1.177873 | 0.238847 | 0.446022 | FALSE |
| OFD1 | 12656 | 1.17727 | 0.239088 | 0.446234 | FALSE |
| ICK | 4315 | 1.175938 | 0.23962 | 0.44712 | FALSE |
| ICE2 | 317 | 1.175396 | 0.239836 | 0.447418 | FALSE |
| CUX2 | 3991 | 1.175122 | 0.239946 | 0.44753 | FALSE |
| POU3F4 | 2202 | 1.174848 | 0.240056 | 0.447666 | FALSE |
| PIGN | 5033 | 1.174722 | 0.240106 | 0.447679 | FALSE |
| LDHAL6A | 13723 | 1.173053 | 0.240775 | 0.448579 | FALSE |
| FAM95A | 11891 | 1.172725 | 0.240906 | 0.448705 | FALSE |
| M6PR | 7768 | 1.172598 | 0.240957 | 0.448705 | FALSE |
| NBEAL1 | 6989 | 1.172204 | 0.241115 | 0.44886 | FALSE |
| TMED8 | 59 | 1.172177 | 0.241126 | 0.44886 | FALSE |
| ZC3H10 | 5745 | 1.171984 | 0.241203 | 0.44895 | FALSE |
| TPRN | 11984 | 1.171306 | 0.241476 | 0.44935 | FALSE |
| TRIM39 | 1740 | 1.17114 | 0.241543 | 0.449421 | FALSE |
| DNAJC9 | 549 | 1.171042 | 0.241582 | 0.449441 | FALSE |
| DECR1 | 12600 | 1.170183 | 0.241927 | 0.449816 | FALSE |
| HIPK3 | 1405 | 1.169642 | 0.242145 | 0.450113 | FALSE |
| BID | 5879 | 1.169122 | 0.242354 | 0.450396 | FALSE |
| RFXAP | 8851 | 1.169122 | 0.242354 | 0.450396 | FALSE |
| RBM15B | 9971 | 1.168581 | 0.242572 | 0.45064 | FALSE |
| BRD9 | 189 | 1.167884 | 0.242854 | 0.451047 | FALSE |
| CHRNA2 | 12834 | 1.166967 | 0.243224 | 0.451581 | FALSE |
| KDELR2 | 2031 | 1.16618 | 0.243542 | 0.45206 | FALSE |
| PEAK1 | 3740 | 1.164601 | 0.244181 | 0.452862 | FALSE |
| CYCSP52 | 1107 | 1.164303 | 0.244301 | 0.452929 | FALSE |
| LMO2 | 138 | 1.164175 | 0.244353 | 0.452929 | FALSE |
| MYH9 | 10669 | 1.164088 | 0.244388 | 0.452929 | FALSE |
| CDK8 | 15252 | 1.16406 | 0.2444 | 0.452929 | FALSE |
| RNF146 | 2729 | 1.163625 | 0.244576 | 0.453124 | FALSE |
| FAM90A1 | 4132 | 1.163372 | 0.244679 | 0.45326 | FALSE |
| ZDHHC3 | 8360 | 1.163071 | 0.244801 | 0.453432 | FALSE |
| HTR5A | 11104 | 1.162795 | 0.244913 | 0.453586 | FALSE |
| CORO2B | 2570 | 1.162704 | 0.24495 | 0.453601 | FALSE |
| FCRLB | 2230 | 1.162181 | 0.245162 | 0.453779 | FALSE |
| ZEB1 | 12511 | 1.161994 | 0.245238 | 0.453823 | FALSE |
| LARGE2 | 13341 | 1.16198 | 0.245244 | 0.453823 | FALSE |
| CCZ1P-OR7E38P | 13428 | 1.161186 | 0.245566 | 0.454234 | FALSE |
| DEPDC5 | 10089 | 1.161104 | 0.2456 | 0.454234 | FALSE |
| LINC01578 | 8884 | 1.161098 | 0.245602 | 0.454234 | FALSE |
| EIF3E | 84 | 1.158248 | 0.246763 | 0.455825 | FALSE |
| HSD11B2 | 4018 | 1.158075 | 0.246833 | 0.455901 | FALSE |
| KRI1 | 9502 | 1.157901 | 0.246904 | 0.455978 | FALSE |
| TNK2 | 4437 | 1.157272 | 0.247161 | 0.456291 | FALSE |
| DHFR2 | 2255 | 1.156465 | 0.247491 | 0.456691 | FALSE |
| SP3 | 7436 | 1.156294 | 0.247561 | 0.456705 | FALSE |
| KANK3 | 783 | 1.156085 | 0.247646 | 0.456755 | FALSE |
| ASTN2 | 15544 | 1.155122 | 0.24804 | 0.45732 | FALSE |
| PAPD5 | 5831 | 1.154816 | 0.248166 | 0.457497 | FALSE |
| WDR35 | 4765 | 1.15412 | 0.248451 | 0.457915 | FALSE |
| SUN2 | 6554 | 1.153594 | 0.248667 | 0.458258 | FALSE |
| SLC41A1 | 5659 | 1.15313 | 0.248857 | 0.458447 | FALSE |
| LOC100506282 | 15347 | 1.152749 | 0.249013 | 0.458601 | FALSE |
| UTP14A | 4806 | 1.152712 | 0.249029 | 0.458601 | FALSE |
| TWSG1 | 12184 | 1.152592 | 0.249078 | 0.458638 | FALSE |
| CDH26 | 8309 | 1.152402 | 0.249156 | 0.458723 | FALSE |
| KRTAP5-9 | 7112 | 1.152337 | 0.249183 | 0.458723 | FALSE |
| FECH | 7864 | 1.152181 | 0.249247 | 0.458786 | FALSE |
| METAP1D | 15113 | 1.151502 | 0.249526 | 0.459084 | FALSE |
| ZNF70 | 14968 | 1.151307 | 0.249606 | 0.459123 | FALSE |
| HDGF | 3141 | 1.151066 | 0.249705 | 0.459143 | FALSE |
| DHTKD1 | 15061 | 1.151037 | 0.249717 | 0.459143 | FALSE |
| CD82 | 15052 | 1.150995 | 0.249734 | 0.459143 | FALSE |
| ZFP41 | 2893 | 1.150736 | 0.249841 | 0.459285 | FALSE |
| STAG3L2 | 6803 | 1.150566 | 0.249911 | 0.45936 | FALSE |
| PHKA1 | 14300 | 1.150295 | 0.250022 | 0.45938 | FALSE |
| PNPO | 2025 | 1.150183 | 0.250069 | 0.45938 | FALSE |
| PCDHA11 | 5367 | 1.15003 | 0.250132 | 0.459441 | FALSE |
| KRBA2 | 12615 | 1.148847 | 0.250619 | 0.460175 | FALSE |
| RNASEH2B | 14390 | 1.148591 | 0.250725 | 0.460261 | FALSE |
| DBP | 13163 | 1.147883 | 0.251017 | 0.460743 | FALSE |
| PCNA | 8519 | 1.147081 | 0.251348 | 0.461253 | FALSE |
| LENG8 | 7737 | 1.147067 | 0.251354 | 0.461253 | FALSE |
| CDCA8 | 3715 | 1.146744 | 0.251487 | 0.461444 | FALSE |
| ADAM19 | 8439 | 1.146163 | 0.251728 | 0.461831 | FALSE |
| GABPB1 | 200 | 1.145722 | 0.25191 | 0.462003 | FALSE |
| SERTAD3 | 2861 | 1.145431 | 0.252031 | 0.462146 | FALSE |
| MAP2 | 5160 | 1.14539 | 0.252048 | 0.462146 | FALSE |
| ZFY | 11550 | 1.145317 | 0.252078 | 0.462148 | FALSE |
| LOC100506548 | 12330 | 1.144986 | 0.252215 | 0.462236 | FALSE |
| HIVEP2 | 14807 | 1.144795 | 0.252294 | 0.462327 | FALSE |
| FMNL2 | 8364 | 1.14448 | 0.252425 | 0.462512 | FALSE |
| SRF | 3791 | 1.144314 | 0.252493 | 0.462584 | FALSE |
| BCL6B | 11182 | 1.142745 | 0.253144 | 0.463659 | FALSE |
| ACAP3 | 346 | 1.142463 | 0.253262 | 0.463665 | FALSE |
| HSPB2 | 13767 | 1.142297 | 0.253331 | 0.463737 | FALSE |
| PKP2 | 14405 | 1.142154 | 0.25339 | 0.463754 | FALSE |
| ZNF764 | 5705 | 1.141857 | 0.253513 | 0.463909 | FALSE |
| TFEB | 6120 | 1.141586 | 0.253626 | 0.464007 | FALSE |
| TRIOBP | 2480 | 1.141414 | 0.253698 | 0.46405 | FALSE |
| MTRR | 813 | 1.140655 | 0.254014 | 0.464444 | FALSE |
| POLR1A | 5730 | 1.139686 | 0.254417 | 0.465028 | FALSE |
| CENPV | 10642 | 1.139582 | 0.25446 | 0.465043 | FALSE |
| GUSBP3 | 3812 | 1.139314 | 0.254572 | 0.465146 | FALSE |
| ZCCHC10 | 7645 | 1.13906 | 0.254678 | 0.465224 | FALSE |
| TMEM80 | 8842 | 1.137481 | 0.255337 | 0.466196 | FALSE |
| ZHX1 | 13882 | 1.137277 | 0.255423 | 0.466196 | FALSE |
| UNK | 4387 | 1.137214 | 0.255449 | 0.466196 | FALSE |
| TRAPPC6B | 11552 | 1.136912 | 0.255575 | 0.466372 | FALSE |
| VPS50 | 13521 | 1.136833 | 0.255608 | 0.466377 | FALSE |
| ACAD11 | 3099 | 1.136641 | 0.255688 | 0.466399 | FALSE |
| GRIP2 | 4350 | 1.13659 | 0.25571 | 0.466399 | FALSE |
| GALK2 | 15099 | 1.136292 | 0.255834 | 0.466572 | FALSE |
| KCNV2 | 7967 | 1.135954 | 0.255976 | 0.466721 | FALSE |
| WDR36 | 10254 | 1.135317 | 0.256243 | 0.467044 | FALSE |
| LRRC59 | 1092 | 1.1349 | 0.256417 | 0.467224 | FALSE |
| BTG1 | 10273 | 1.134802 | 0.256458 | 0.467224 | FALSE |
| ZDHHC17 | 5412 | 1.133844 | 0.25686 | 0.467612 | FALSE |
| AFG3L1P | 13096 | 1.133829 | 0.256866 | 0.467612 | FALSE |
| IFI44L | 6495 | 1.133811 | 0.256874 | 0.467612 | FALSE |
| MUTYH | 9955 | 1.133789 | 0.256883 | 0.467612 | FALSE |
| ESM1 | 3827 | 1.133717 | 0.256913 | 0.467613 | FALSE |
| RBM41 | 4331 | 1.132984 | 0.257221 | 0.468064 | FALSE |
| SENP8 | 12856 | 1.132804 | 0.257297 | 0.468147 | FALSE |
| DNAH1 | 2621 | 1.132367 | 0.25748 | 0.468263 | FALSE |
| MAPK8 | 2758 | 1.13228 | 0.257517 | 0.468275 | FALSE |
| DSCR9 | 11030 | 1.132161 | 0.257567 | 0.468297 | FALSE |
| TRIM41 | 4658 | 1.1321 | 0.257592 | 0.468297 | FALSE |
| FAM84A | 5545 | 1.131966 | 0.257649 | 0.468297 | FALSE |
| TRIM23 | 1076 | 1.131728 | 0.257749 | 0.468424 | FALSE |
| ARNT | 8151 | 1.131334 | 0.257915 | 0.468508 | FALSE |
| PAICS | 13101 | 1.130384 | 0.258314 | 0.469016 | FALSE |
| WDYHV1 | 15158 | 1.129967 | 0.25849 | 0.469256 | FALSE |
| BUD23 | 8304 | 1.129857 | 0.258536 | 0.469256 | FALSE |
| ACBD5 | 12750 | 1.129561 | 0.258661 | 0.469428 | FALSE |
| GALNT6 | 9518 | 1.129269 | 0.258784 | 0.469587 | FALSE |
| EPHX4 | 9663 | 1.129164 | 0.258829 | 0.469587 | FALSE |
| DNM1P46 | 7640 | 1.128801 | 0.258982 | 0.469628 | FALSE |
| NARS2 | 553 | 1.128121 | 0.259269 | 0.470094 | FALSE |
| PCDHB5 | 14082 | 1.127734 | 0.259432 | 0.470281 | FALSE |
| WWC3 | 6542 | 1.1275 | 0.259531 | 0.470351 | FALSE |
| ZNF618 | 3447 | 1.126894 | 0.259787 | 0.470645 | FALSE |
| LRRC6 | 14215 | 1.126174 | 0.260092 | 0.470973 | FALSE |
| KRTAP5-2 | 6844 | 1.126034 | 0.260151 | 0.470975 | FALSE |
| THAP5 | 319 | 1.125518 | 0.26037 | 0.471161 | FALSE |
| TRMU | 916 | 1.125381 | 0.260428 | 0.471211 | FALSE |
| TAF15 | 6834 | 1.125084 | 0.260553 | 0.471275 | FALSE |
| THUMPD1 | 13473 | 1.124637 | 0.260743 | 0.471478 | FALSE |
| SHQ1 | 13193 | 1.124606 | 0.260756 | 0.471478 | FALSE |
| PLCL2 | 7712 | 1.124521 | 0.260792 | 0.471489 | FALSE |
| RIPPLY3 | 6956 | 1.124361 | 0.26086 | 0.471557 | FALSE |
| ERG | 1768 | 1.123783 | 0.261105 | 0.471891 | FALSE |
| ABCG1 | 5152 | 1.123678 | 0.26115 | 0.471917 | FALSE |
| RAD51C | 15376 | 1.122569 | 0.261621 | 0.472658 | FALSE |
| HACD2 | 1965 | 1.122498 | 0.261651 | 0.472658 | FALSE |
| TBC1D3P5 | 1927 | 1.121841 | 0.26193 | 0.472944 | FALSE |
| ABHD11 | 10672 | 1.121375 | 0.262128 | 0.473193 | FALSE |
| ADCY1 | 8306 | 1.120415 | 0.262537 | 0.473712 | FALSE |
| TMEM201 | 14421 | 1.120089 | 0.262676 | 0.473908 | FALSE |
| ANKRD9 | 10440 | 1.119494 | 0.262929 | 0.474256 | FALSE |
| FAM162B | 998 | 1.118971 | 0.263153 | 0.474549 | FALSE |
| RINT1 | 4327 | 1.118786 | 0.263231 | 0.474636 | FALSE |
| TPT1-AS1 | 8520 | 1.118544 | 0.263335 | 0.474658 | FALSE |
| SRRM3 | 15529 | 1.118358 | 0.263414 | 0.474747 | FALSE |
| SLC35C1 | 7369 | 1.118177 | 0.263491 | 0.474777 | FALSE |
| MTFP1 | 4179 | 1.117173 | 0.26392 | 0.475277 | FALSE |
| ALG6 | 15549 | 1.116965 | 0.264009 | 0.475321 | FALSE |
| PABPC1L2A | 12532 | 1.1169 | 0.264037 | 0.475321 | FALSE |
| CCNE2 | 5449 | 1.116316 | 0.264287 | 0.475662 | FALSE |
| GPR19 | 12890 | 1.115081 | 0.264816 | 0.476517 | FALSE |
| CADM2 | 14492 | 1.115064 | 0.264823 | 0.476517 | FALSE |
| JMJD6 | 6421 | 1.11486 | 0.26491 | 0.476619 | FALSE |
| MIDN | 15553 | 1.11476 | 0.264953 | 0.476621 | FALSE |
| NMRK1 | 1633 | 1.114715 | 0.264973 | 0.476621 | FALSE |
| OSGIN2 | 63 | 1.1145 | 0.265065 | 0.476732 | FALSE |
| TUBGCP2 | 13323 | 1.113752 | 0.265386 | 0.477145 | FALSE |
| EBLN2 | 2995 | 1.113671 | 0.26542 | 0.477152 | FALSE |
| CSNK1D | 10192 | 1.112812 | 0.265789 | 0.477515 | FALSE |
| SH3KBP1 | 1277 | 1.112703 | 0.265836 | 0.477515 | FALSE |
| DSTYK | 10152 | 1.112574 | 0.265891 | 0.477518 | FALSE |
| ZNF839 | 12838 | 1.112557 | 0.265899 | 0.477518 | FALSE |
| TSEN15 | 7618 | 1.112198 | 0.266053 | 0.477576 | FALSE |
| MBTPS1 | 7095 | 1.111542 | 0.266335 | 0.477972 | FALSE |
| TRIM46 | 13108 | 1.111182 | 0.26649 | 0.478141 | FALSE |
| CD34 | 5996 | 1.111059 | 0.266543 | 0.478181 | FALSE |
| CCER2 | 2128 | 1.110914 | 0.266605 | 0.478238 | FALSE |
| OR2L3 | 11506 | 1.1105 | 0.266784 | 0.478309 | FALSE |
| BRF1 | 9762 | 1.110466 | 0.266798 | 0.478309 | FALSE |
| GK3P | 10037 | 1.110244 | 0.266894 | 0.478371 | FALSE |
| ZNF189 | 13510 | 1.109352 | 0.267278 | 0.478786 | FALSE |
| CELF1 | 8318 | 1.109183 | 0.267351 | 0.478861 | FALSE |
| TMEM167B | 648 | 1.109038 | 0.267414 | 0.478919 | FALSE |
| LOC729159 | 8447 | 1.108836 | 0.267501 | 0.478965 | FALSE |
| REP15 | 10909 | 1.108462 | 0.267662 | 0.479144 | FALSE |
| RGCC | 10283 | 1.108239 | 0.267759 | 0.479149 | FALSE |
| TMEM121B | 14444 | 1.108226 | 0.267764 | 0.479149 | FALSE |
| PHLPP1 | 15519 | 1.106975 | 0.268305 | 0.479799 | FALSE |
| NPY2R | 3047 | 1.106794 | 0.268383 | 0.479799 | FALSE |
| C4orf3 | 9808 | 1.106193 | 0.268643 | 0.48013 | FALSE |
| SVIP | 14866 | 1.105975 | 0.268737 | 0.480206 | FALSE |
| MACF1 | 11670 | 1.105888 | 0.268775 | 0.480206 | FALSE |
| LOXHD1 | 8504 | 1.105882 | 0.268778 | 0.480206 | FALSE |
| LOC105370333 | 5444 | 1.105749 | 0.268835 | 0.480254 | FALSE |
| ZBTB24 | 2195 | 1.105424 | 0.268976 | 0.48034 | FALSE |
| WWC2 | 8579 | 1.105311 | 0.269025 | 0.480373 | FALSE |
| GUSBP10 | 14498 | 1.105132 | 0.269102 | 0.480457 | FALSE |
| LRIG3 | 7470 | 1.104982 | 0.269167 | 0.480518 | FALSE |
| ZNF99 | 4487 | 1.104581 | 0.269341 | 0.480728 | FALSE |
| ABCD3 | 12927 | 1.103979 | 0.269602 | 0.481014 | FALSE |
| C20orf96 | 1239 | 1.10397 | 0.269606 | 0.481014 | FALSE |
| MYLK | 14008 | 1.103106 | 0.269981 | 0.481353 | FALSE |
| NOMO1 | 4371 | 1.102147 | 0.270398 | 0.481812 | FALSE |
| CEP68 | 6283 | 1.100645 | 0.271051 | 0.482516 | FALSE |
| ZNF830 | 4573 | 1.100626 | 0.271059 | 0.482516 | FALSE |
| KRTAP10-2 | 2715 | 1.100558 | 0.271089 | 0.482516 | FALSE |
| CD9 | 6249 | 1.099648 | 0.271486 | 0.483001 | FALSE |
| ZCCHC11 | 7696 | 1.099335 | 0.271622 | 0.483047 | FALSE |
| ZNF610 | 2114 | 1.099248 | 0.27166 | 0.483047 | FALSE |
| HS3ST5 | 10738 | 1.099235 | 0.271666 | 0.483047 | FALSE |
| COL12A1 | 5323 | 1.099155 | 0.2717 | 0.483054 | FALSE |
| USP53 | 15438 | 1.098925 | 0.271801 | 0.483122 | FALSE |
| FUNDC2 | 12686 | 1.098833 | 0.271841 | 0.483139 | FALSE |
| TTI1 | 14915 | 1.098344 | 0.272054 | 0.483463 | FALSE |
| HACD3 | 4674 | 1.098104 | 0.272159 | 0.483594 | FALSE |
| YOD1 | 1703 | 1.097802 | 0.272291 | 0.483774 | FALSE |
| PCGF2 | 11412 | 1.097523 | 0.272413 | 0.483922 | FALSE |
| DAB2IP | 12982 | 1.097469 | 0.272436 | 0.483922 | FALSE |
| TTC30A | 13033 | 1.096417 | 0.272896 | 0.484323 | FALSE |
| PWWP2A | 10540 | 1.096386 | 0.27291 | 0.484323 | FALSE |
| ANAPC16 | 14368 | 1.096137 | 0.273019 | 0.48446 | FALSE |
| ID2 | 9162 | 1.095897 | 0.273124 | 0.484538 | FALSE |
| POU2F1 | 12973 | 1.095558 | 0.273272 | 0.484691 | FALSE |
| MED17 | 3239 | 1.095329 | 0.273373 | 0.484761 | FALSE |
| SLC38A3 | 2090 | 1.095255 | 0.273405 | 0.484761 | FALSE |
| WNT2B | 13085 | 1.094521 | 0.273727 | 0.485276 | FALSE |
| FUT4 | 14575 | 1.094033 | 0.273941 | 0.485546 | FALSE |
| SNRPB | 4537 | 1.093735 | 0.274071 | 0.485575 | FALSE |
| BMPR1A | 13009 | 1.093657 | 0.274105 | 0.485575 | FALSE |
| ATRX | 6692 | 1.09357 | 0.274144 | 0.485575 | FALSE |
| KEAP1 | 14299 | 1.092985 | 0.2744 | 0.48592 | FALSE |
| WDR27 | 4977 | 1.092464 | 0.274629 | 0.486238 | FALSE |
| CEP170P1 | 15437 | 1.092435 | 0.274642 | 0.486238 | FALSE |
| USP24 | 13045 | 1.091826 | 0.27491 | 0.486546 | FALSE |
| C9orf147 | 1783 | 1.091659 | 0.274983 | 0.486621 | FALSE |
| INTS14 | 1947 | 1.091305 | 0.275139 | 0.486736 | FALSE |
| PTGS2 | 4516 | 1.091285 | 0.275147 | 0.486736 | FALSE |
| PLXNA3 | 2362 | 1.091165 | 0.2752 | 0.486736 | FALSE |
| SOGA1 | 12225 | 1.091086 | 0.275235 | 0.486736 | FALSE |
| POLR3D | 6415 | 1.090032 | 0.275699 | 0.487392 | FALSE |
| ZNF432 | 7771 | 1.089689 | 0.27585 | 0.487442 | FALSE |
| FKBP3 | 839 | 1.089613 | 0.275884 | 0.487442 | FALSE |
| ATP10D | 13176 | 1.089377 | 0.275988 | 0.487571 | FALSE |
| ERC2-IT1 | 14960 | 1.089041 | 0.276136 | 0.487778 | FALSE |
| DHX16 | 2286 | 1.088889 | 0.276203 | 0.487788 | FALSE |
| LTK | 9104 | 1.088753 | 0.276263 | 0.487788 | FALSE |
| DENND5B | 1210 | 1.088676 | 0.276297 | 0.487788 | FALSE |
| SORD2P | 11046 | 1.088454 | 0.276395 | 0.487788 | FALSE |
| ASTE1 | 4417 | 1.088368 | 0.276433 | 0.487788 | FALSE |
| LINC01596 | 13222 | 1.08832 | 0.276454 | 0.487788 | FALSE |
| TRIM65 | 8512 | 1.087825 | 0.276672 | 0.488064 | FALSE |
| KIAA1656 | 13835 | 1.087146 | 0.276972 | 0.488397 | FALSE |
| ZNF547 | 9614 | 1.087051 | 0.277014 | 0.488397 | FALSE |
| PRKACB | 8326 | 1.087031 | 0.277023 | 0.488397 | FALSE |
| MAPT | 1514 | 1.086973 | 0.277049 | 0.488397 | FALSE |
| TTC4 | 13799 | 1.086613 | 0.277208 | 0.488622 | FALSE |
| DLST | 589 | 1.086452 | 0.277279 | 0.488638 | FALSE |
| RNF169 | 13025 | 1.086186 | 0.277397 | 0.48879 | FALSE |
| FZD10 | 2643 | 1.085676 | 0.277622 | 0.489022 | FALSE |
| SLC35A1 | 11198 | 1.085281 | 0.277797 | 0.489275 | FALSE |
| THAP1 | 4259 | 1.085173 | 0.277845 | 0.489304 | FALSE |
| LOC644762 | 3361 | 1.084599 | 0.278099 | 0.489697 | FALSE |
| VILL | 3657 | 1.084048 | 0.278344 | 0.490016 | FALSE |
| LINC00115 | 6879 | 1.083691 | 0.278502 | 0.490185 | FALSE |
| DDX52 | 2884 | 1.082833 | 0.278883 | 0.490689 | FALSE |
| LEMD2 | 7946 | 1.082525 | 0.279019 | 0.490874 | FALSE |
| SCAND2P | 10918 | 1.082095 | 0.27921 | 0.4911 | FALSE |
| EDEM3 | 2055 | 1.082007 | 0.279249 | 0.491113 | FALSE |
| ISY1 | 6306 | 1.081503 | 0.279473 | 0.491452 | FALSE |
| APC2 | 7435 | 1.080847 | 0.279765 | 0.491744 | FALSE |
| ANP32AP1 | 5386 | 1.079385 | 0.280416 | 0.4925 | FALSE |
| WDFY3-AS2 | 1761 | 1.078787 | 0.280683 | 0.49286 | FALSE |
| SLC14A1 | 801 | 1.0787 | 0.280721 | 0.49286 | FALSE |
| IDH1 | 10529 | 1.078659 | 0.28074 | 0.49286 | FALSE |
| PSPC1 | 9255 | 1.078643 | 0.280747 | 0.49286 | FALSE |
| FEZ1 | 57 | 1.07852 | 0.280802 | 0.492901 | FALSE |
| CCNK | 2360 | 1.078369 | 0.280869 | 0.492964 | FALSE |
| FAM69C | 4007 | 1.078234 | 0.280929 | 0.493014 | FALSE |
| MAPRE3 | 13456 | 1.077987 | 0.28104 | 0.493152 | FALSE |
| KLHL18 | 12641 | 1.076999 | 0.281481 | 0.493704 | FALSE |
| ZDHHC5 | 5472 | 1.076415 | 0.281742 | 0.494107 | FALSE |
| PIGU | 12264 | 1.076055 | 0.281903 | 0.49421 | FALSE |
| MATN3 | 3357 | 1.075242 | 0.282266 | 0.494749 | FALSE |
| CRTC2 | 4654 | 1.074567 | 0.282569 | 0.495067 | FALSE |
| ZNF700 | 2141 | 1.074554 | 0.282574 | 0.495067 | FALSE |
| PPA2 | 14388 | 1.074442 | 0.282625 | 0.4951 | FALSE |
| AK9 | 4889 | 1.074309 | 0.282684 | 0.495149 | FALSE |
| ROR1 | 7160 | 1.073616 | 0.282995 | 0.495547 | FALSE |
| ATP6AP2 | 382 | 1.073584 | 0.283009 | 0.495547 | FALSE |
| SOX21 | 2795 | 1.073519 | 0.283038 | 0.495547 | FALSE |
| PFN4 | 515 | 1.07233 | 0.283572 | 0.496298 | FALSE |
| SLC9A8 | 9220 | 1.071898 | 0.283766 | 0.496487 | FALSE |
| PLEC | 13622 | 1.070992 | 0.284173 | 0.496986 | FALSE |
| TEX2 | 11117 | 1.07048 | 0.284403 | 0.49723 | FALSE |
| GSTK1 | 7666 | 1.070268 | 0.284499 | 0.497324 | FALSE |
| ABCG4 | 8739 | 1.070025 | 0.284608 | 0.497362 | FALSE |
| FLT4 | 6257 | 1.070008 | 0.284616 | 0.497362 | FALSE |
| PLPP1 | 5615 | 1.069317 | 0.284927 | 0.497683 | FALSE |
| UBE2J2 | 9248 | 1.068725 | 0.285194 | 0.497984 | FALSE |
| ANKRD13D | 7682 | 1.068343 | 0.285366 | 0.498171 | FALSE |
| PRPF38A | 10677 | 1.067697 | 0.285657 | 0.498513 | FALSE |
| SCRN1 | 8203 | 1.067447 | 0.28577 | 0.498654 | FALSE |
| NIFK-AS1 | 12029 | 1.06684 | 0.286044 | 0.499027 | FALSE |
| MORF4L2 | 2073 | 1.066833 | 0.286047 | 0.499027 | FALSE |
| PIGX | 554 | 1.066506 | 0.286195 | 0.499173 | FALSE |
| METTL14 | 7231 | 1.066431 | 0.286229 | 0.499176 | FALSE |
| FAM122A | 8080 | 1.066267 | 0.286303 | 0.499194 | FALSE |
| ZNF192P1 | 1706 | 1.065659 | 0.286578 | 0.499458 | FALSE |
| ZNF674-AS1 | 11560 | 1.065649 | 0.286582 | 0.499458 | FALSE |
| BIRC2 | 2318 | 1.064815 | 0.28696 | 0.500004 | FALSE |
| ECD | 11226 | 1.064223 | 0.287228 | 0.500248 | FALSE |
| DDX6 | 6513 | 1.063768 | 0.287434 | 0.500496 | FALSE |
| RTN2 | 4195 | 1.06356 | 0.287528 | 0.500538 | FALSE |
| MSANTD2 | 4709 | 1.063502 | 0.287554 | 0.500538 | FALSE |
| HAPLN2 | 6373 | 1.063445 | 0.28758 | 0.500538 | FALSE |
| MICAL3 | 7339 | 1.062675 | 0.287929 | 0.500812 | FALSE |
| LRRC28 | 13147 | 1.062545 | 0.287988 | 0.500812 | FALSE |
| PHAX | 12562 | 1.062497 | 0.28801 | 0.500812 | FALSE |
| ACVRL1 | 15322 | 1.062435 | 0.288038 | 0.500812 | FALSE |
| ZNF460 | 10567 | 1.061381 | 0.288517 | 0.501321 | FALSE |
| TPD52L2 | 5548 | 1.060819 | 0.288772 | 0.501486 | FALSE |
| HACL1 | 2092 | 1.060518 | 0.288909 | 0.501612 | FALSE |
| SCARF1 | 3464 | 1.058989 | 0.289605 | 0.502541 | FALSE |
| CCDC43 | 8786 | 1.057932 | 0.290086 | 0.503085 | FALSE |
| B3GALT1 | 9474 | 1.057864 | 0.290117 | 0.503085 | FALSE |
| MEF2D | 14808 | 1.057807 | 0.290143 | 0.503085 | FALSE |
| GOSR1 | 15590 | 1.055796 | 0.291061 | 0.504341 | FALSE |
| VWDE | 13765 | 1.055629 | 0.291138 | 0.504417 | FALSE |
| HAUS2 | 7739 | 1.054962 | 0.291443 | 0.504817 | FALSE |
| YY1 | 835 | 1.054791 | 0.291521 | 0.504857 | FALSE |
| SLX4IP | 3173 | 1.054632 | 0.291594 | 0.504927 | FALSE |
| ZFAND1 | 11699 | 1.053248 | 0.292227 | 0.5058 | FALSE |
| ZNF763 | 4431 | 1.05302 | 0.292332 | 0.505869 | FALSE |
| ZBTB17 | 14251 | 1.052803 | 0.292431 | 0.505985 | FALSE |
| IPP | 12557 | 1.05208 | 0.292763 | 0.506391 | FALSE |
| SGK3 | 6978 | 1.051274 | 0.293133 | 0.506919 | FALSE |
| AXIN1 | 14184 | 1.050205 | 0.293624 | 0.50743 | FALSE |
| ZNF138 | 15513 | 1.050145 | 0.293651 | 0.50743 | FALSE |
| USP54 | 12766 | 1.050033 | 0.293703 | 0.507456 | FALSE |
| HACE1 | 12158 | 1.04965 | 0.293879 | 0.507543 | FALSE |
| CWF19L2 | 9378 | 1.049345 | 0.294019 | 0.507607 | FALSE |
| ZXDC | 14939 | 1.049278 | 0.29405 | 0.507607 | FALSE |
| LINC02018 | 1410 | 1.048966 | 0.294194 | 0.507742 | FALSE |
| UBE2D3 | 13205 | 1.048857 | 0.294244 | 0.507773 | FALSE |
| MGAT1 | 4884 | 1.048603 | 0.294361 | 0.507917 | FALSE |
| PTGR1 | 312 | 1.048535 | 0.294392 | 0.507917 | FALSE |
| NHEJ1 | 4610 | 1.048142 | 0.294573 | 0.508117 | FALSE |
| PKN2 | 14903 | 1.047915 | 0.294678 | 0.508185 | FALSE |
| PPIP5K1 | 13737 | 1.047642 | 0.294804 | 0.508346 | FALSE |
| ORC5 | 9546 | 1.046748 | 0.295216 | 0.508945 | FALSE |
| ADRA1D | 11702 | 1.046559 | 0.295303 | 0.509037 | FALSE |
| MAPKBP1 | 3455 | 1.04635 | 0.295399 | 0.509037 | FALSE |
| AP2B1 | 10995 | 1.046148 | 0.295493 | 0.509141 | FALSE |
| ZNF496 | 15210 | 1.045096 | 0.295979 | 0.509624 | FALSE |
| ABRAXAS2 | 20 | 1.045047 | 0.296001 | 0.509624 | FALSE |
| ADAL | 10989 | 1.043805 | 0.296576 | 0.510332 | FALSE |
| CYB5B | 14515 | 1.043598 | 0.296671 | 0.510365 | FALSE |
| ELMO1 | 2193 | 1.04356 | 0.296689 | 0.510365 | FALSE |
| NELFCD | 15103 | 1.043372 | 0.296776 | 0.510396 | FALSE |
| ACSS2 | 10897 | 1.043109 | 0.296898 | 0.510549 | FALSE |
| CDK18 | 1621 | 1.042579 | 0.297143 | 0.510812 | FALSE |
| KCNJ2 | 15403 | 1.041965 | 0.297428 | 0.510899 | FALSE |
| PARP11 | 15339 | 1.041727 | 0.297538 | 0.510976 | FALSE |
| ZNF121 | 14640 | 1.041311 | 0.297731 | 0.511195 | FALSE |
| DAXX | 1118 | 1.040419 | 0.298145 | 0.511794 | FALSE |
| WSB1 | 9754 | 1.040183 | 0.298255 | 0.511926 | FALSE |
| VPS26A | 3158 | 1.040076 | 0.298305 | 0.511955 | FALSE |
| FAM225B | 6599 | 1.03949 | 0.298577 | 0.512265 | FALSE |
| ZNF696 | 13729 | 1.039475 | 0.298584 | 0.512265 | FALSE |
| LRRTM3 | 3404 | 1.03904 | 0.298786 | 0.512553 | FALSE |
| SORCS3 | 5616 | 1.038825 | 0.298886 | 0.512615 | FALSE |
| DCAF8 | 1675 | 1.038728 | 0.298931 | 0.512636 | FALSE |
| EPG5 | 4482 | 1.038442 | 0.299064 | 0.512696 | FALSE |
| CAPN3 | 5430 | 1.038187 | 0.299183 | 0.512843 | FALSE |
| DYNLL2 | 7583 | 1.036419 | 0.300007 | 0.514142 | FALSE |
| MALT1 | 15276 | 1.036262 | 0.30008 | 0.514195 | FALSE |
| KRT8 | 6465 | 1.036041 | 0.300183 | 0.514226 | FALSE |
| PEX5L | 954 | 1.035961 | 0.30022 | 0.514226 | FALSE |
| TXNDC9 | 369 | 1.035613 | 0.300383 | 0.514448 | FALSE |
| KRTAP12-3 | 8398 | 1.035465 | 0.300452 | 0.51451 | FALSE |
| CDK11A | 501 | 1.035123 | 0.300611 | 0.514727 | FALSE |
| LARS | 8587 | 1.034998 | 0.30067 | 0.514771 | FALSE |
| ZBTB2 | 13777 | 1.034319 | 0.300987 | 0.515201 | FALSE |
| MCPH1-AS1 | 15332 | 1.033907 | 0.30118 | 0.515369 | FALSE |
| SLC16A1 | 13904 | 1.033895 | 0.301185 | 0.515369 | FALSE |
| MFGE8 | 14159 | 1.033847 | 0.301208 | 0.515369 | FALSE |
| LAMB2P1 | 3197 | 1.033827 | 0.301217 | 0.515369 | FALSE |
| ZFAND4 | 15150 | 1.03334 | 0.301445 | 0.515477 | FALSE |
| TOP3B | 8559 | 1.03289 | 0.301655 | 0.515552 | FALSE |
| E2F6 | 2664 | 1.032752 | 0.30172 | 0.515552 | FALSE |
| UNC5C | 11553 | 1.032678 | 0.301755 | 0.515555 | FALSE |
| PCDH9 | 6539 | 1.032101 | 0.302025 | 0.515843 | FALSE |
| MACO1 | 492 | 1.032065 | 0.302042 | 0.515843 | FALSE |
| LDLRAP1 | 456 | 1.03191 | 0.302114 | 0.515888 | FALSE |
| EAF2 | 7677 | 1.030973 | 0.302553 | 0.516422 | FALSE |
| AHCYL2 | 591 | 1.030746 | 0.30266 | 0.516422 | FALSE |
| CCDC151 | 12962 | 1.030542 | 0.302756 | 0.516475 | FALSE |
| PCNX3 | 5413 | 1.02997 | 0.303024 | 0.516708 | FALSE |
| TNRC6C | 4479 | 1.029608 | 0.303194 | 0.516848 | FALSE |
| BCAS1 | 1912 | 1.029603 | 0.303196 | 0.516848 | FALSE |
| POLR3B | 777 | 1.029405 | 0.303289 | 0.516906 | FALSE |
| ZNF213 | 6838 | 1.029225 | 0.303374 | 0.516966 | FALSE |
| C3orf35 | 15168 | 1.028902 | 0.303526 | 0.517168 | FALSE |
| MBD6 | 14080 | 1.028759 | 0.303593 | 0.51717 | FALSE |
| BBS9 | 11788 | 1.028214 | 0.303849 | 0.517494 | FALSE |
| SNIP1 | 34 | 1.027948 | 0.303974 | 0.51765 | FALSE |
| MCRS1 | 7743 | 1.027625 | 0.304126 | 0.517853 | FALSE |
| TMEM251 | 9004 | 1.026927 | 0.304455 | 0.518265 | FALSE |
| TMEM14C | 10075 | 1.026193 | 0.304801 | 0.518733 | FALSE |
| MRPS26 | 3658 | 1.026109 | 0.30484 | 0.518733 | FALSE |
| MED11 | 7725 | 1.026104 | 0.304843 | 0.518733 | FALSE |
| TOE1 | 8024 | 1.025681 | 0.305042 | 0.519016 | FALSE |
| LOC730183 | 13466 | 1.025506 | 0.305124 | 0.5191 | FALSE |
| GOLGA8IP | 7576 | 1.025239 | 0.30525 | 0.519201 | FALSE |
| KIAA1549L | 8219 | 1.023939 | 0.305864 | 0.519962 | FALSE |
| UBE2D4 | 13727 | 1.023665 | 0.305994 | 0.520126 | FALSE |
| KCNH4 | 8058 | 1.02345 | 0.306095 | 0.520242 | FALSE |
| MAP4 | 11144 | 1.023052 | 0.306283 | 0.520422 | FALSE |
| TECTA | 10378 | 1.022888 | 0.306361 | 0.520422 | FALSE |
| HABP4 | 6435 | 1.022874 | 0.306367 | 0.520422 | FALSE |
| NFYB | 12171 | 1.022364 | 0.306609 | 0.520662 | FALSE |
| TSPAN12 | 15471 | 1.021881 | 0.306837 | 0.520937 | FALSE |
| FITM2 | 10398 | 1.021388 | 0.307071 | 0.521212 | FALSE |
| TLDC1 | 12186 | 1.020714 | 0.30739 | 0.52159 | FALSE |
| EZH2 | 1639 | 1.019828 | 0.30781 | 0.522135 | FALSE |
| PTPN3 | 10564 | 1.019269 | 0.308075 | 0.522471 | FALSE |
| CLCN3 | 11293 | 1.018552 | 0.308416 | 0.522822 | FALSE |
| PEX16 | 1099 | 1.018251 | 0.308559 | 0.523007 | FALSE |
| MTG2 | 2819 | 1.018097 | 0.308632 | 0.523061 | FALSE |
| ZNF345 | 890 | 1.017834 | 0.308757 | 0.523065 | FALSE |
| ZNF417 | 8908 | 1.017828 | 0.30876 | 0.523065 | FALSE |
| FPGT | 9348 | 1.017722 | 0.30881 | 0.523093 | FALSE |
| SCGB1C2 | 9251 | 1.017407 | 0.30896 | 0.52329 | FALSE |
| PRKCE | 11639 | 1.016817 | 0.30924 | 0.523595 | FALSE |
| UBXN2A | 12763 | 1.016735 | 0.30928 | 0.523605 | FALSE |
| TESPA1 | 1174 | 1.016323 | 0.309476 | 0.523648 | FALSE |
| KAT6A | 15468 | 1.016266 | 0.309503 | 0.523648 | FALSE |
| FAT3 | 7298 | 1.016217 | 0.309526 | 0.523648 | FALSE |
| ATR | 15363 | 1.015937 | 0.309659 | 0.523662 | FALSE |
| EIF2S1 | 2460 | 1.015627 | 0.309807 | 0.523761 | FALSE |
| FAM53B | 9515 | 1.01504 | 0.310087 | 0.524177 | FALSE |
| ZNF410 | 1976 | 1.013629 | 0.31076 | 0.525182 | FALSE |
| WDR91 | 9077 | 1.013474 | 0.310834 | 0.525182 | FALSE |
| ILKAP | 10670 | 1.013441 | 0.31085 | 0.525182 | FALSE |
| AFF4 | 14681 | 1.013365 | 0.310886 | 0.525187 | FALSE |
| BLOC1S6 | 11858 | 1.01311 | 0.311008 | 0.525223 | FALSE |
| PACSIN3 | 8168 | 1.013064 | 0.31103 | 0.525223 | FALSE |
| WASHC1 | 13870 | 1.012856 | 0.311129 | 0.525251 | FALSE |
| CLK4 | 945 | 1.012793 | 0.311159 | 0.525251 | FALSE |
| MRPL16 | 3883 | 1.012479 | 0.311309 | 0.525408 | FALSE |
| USP22 | 522 | 1.012408 | 0.311343 | 0.525408 | FALSE |
| MTHFR | 8351 | 1.012308 | 0.311391 | 0.525408 | FALSE |
| OCEL1 | 12011 | 1.012247 | 0.31142 | 0.525408 | FALSE |
| MRPL19 | 10883 | 1.011538 | 0.311759 | 0.525923 | FALSE |
| CAV1 | 11131 | 1.010985 | 0.312024 | 0.526175 | FALSE |
| UQCRB | 10253 | 1.010305 | 0.312349 | 0.526463 | FALSE |
| HIVEP3 | 4286 | 1.010236 | 0.312382 | 0.526463 | FALSE |
| FKBP9 | 4111 | 1.009856 | 0.312564 | 0.526585 | FALSE |
| FBXO28 | 12911 | 1.009793 | 0.312594 | 0.526585 | FALSE |
| HNRNPLL | 7906 | 1.009653 | 0.312662 | 0.526593 | FALSE |
| ARHGEF33 | 8570 | 1.008699 | 0.313119 | 0.52725 | FALSE |
| PMEPA1 | 12761 | 1.008162 | 0.313377 | 0.527355 | FALSE |
| SERPING1 | 10499 | 1.008154 | 0.313381 | 0.527355 | FALSE |
| PCDHB10 | 13881 | 1.008097 | 0.313408 | 0.527355 | FALSE |
| C15orf41 | 15478 | 1.007693 | 0.313602 | 0.527608 | FALSE |
| GIMAP7 | 8075 | 1.007603 | 0.313645 | 0.527624 | FALSE |
| BTBD7 | 15342 | 1.00709 | 0.313892 | 0.527964 | FALSE |
| TTC12 | 15008 | 1.007042 | 0.313915 | 0.527964 | FALSE |
| KAZN | 9967 | 1.006871 | 0.313997 | 0.527975 | FALSE |
| SNAP23 | 13502 | 1.006818 | 0.314022 | 0.527975 | FALSE |
| SRGAP2 | 2001 | 1.006442 | 0.314203 | 0.528082 | FALSE |
| GORASP1 | 10648 | 1.006157 | 0.31434 | 0.528154 | FALSE |
| MUS81 | 5503 | 1.005936 | 0.314446 | 0.528154 | FALSE |
| COPS2 | 3405 | 1.005876 | 0.314475 | 0.528154 | FALSE |
| RABL6 | 11422 | 1.005844 | 0.314491 | 0.528154 | FALSE |
| ZNF778 | 5331 | 1.005372 | 0.314718 | 0.528405 | FALSE |
| AMER3 | 9930 | 1.005005 | 0.314894 | 0.528621 | FALSE |
| CSRP1 | 1862 | 1.003926 | 0.315414 | 0.529347 | FALSE |
| GUSBP11 | 12341 | 1.003616 | 0.315564 | 0.529397 | FALSE |
| PIP4K2B | 11420 | 1.002494 | 0.316105 | 0.529711 | FALSE |
| MIPEP | 3481 | 1.001478 | 0.316596 | 0.530306 | FALSE |
| ZNF550 | 14141 | 1.001384 | 0.316641 | 0.530325 | FALSE |
| KIAA1614 | 9600 | 1.000901 | 0.316875 | 0.530594 | FALSE |
| GCNT4 | 6420 | 1.000558 | 0.317041 | 0.530766 | FALSE |
| CAPRIN1 | 10875 | 0.999956 | 0.317332 | 0.53118 | FALSE |
| PEX12 | 7450 | 0.999814 | 0.317401 | 0.531198 | FALSE |
| PHF19 | 356 | 0.999713 | 0.317449 | 0.531223 | FALSE |
| TIAM2 | 13320 | 0.999066 | 0.317763 | 0.531587 | FALSE |
| ZC3H13 | 8526 | 0.999054 | 0.317769 | 0.531587 | FALSE |
| ACIN1 | 9495 | 0.998983 | 0.317803 | 0.531587 | FALSE |
| VHL | 15136 | 0.998749 | 0.317916 | 0.53172 | FALSE |
| SULT1A2 | 3632 | 0.998194 | 0.318185 | 0.532113 | FALSE |
| TIMELESS | 5245 | 0.997961 | 0.318298 | 0.532245 | FALSE |
| USP2 | 2220 | 0.99766 | 0.318444 | 0.532375 | FALSE |
| EXOC6B | 10109 | 0.99723 | 0.318653 | 0.532553 | FALSE |
| ANO8 | 11874 | 0.997088 | 0.318722 | 0.532611 | FALSE |
| ADAMTS14 | 1279 | 0.996857 | 0.318834 | 0.532691 | FALSE |
| SERPINB6 | 13515 | 0.996772 | 0.318875 | 0.532691 | FALSE |
| LRCH3 | 784 | 0.996697 | 0.318912 | 0.532691 | FALSE |
| ALG10 | 8426 | 0.996639 | 0.31894 | 0.532691 | FALSE |
| FAM215A | 4794 | 0.996045 | 0.319228 | 0.533059 | FALSE |
| RNF214 | 6923 | 0.995512 | 0.319487 | 0.533342 | FALSE |
| ALS2 | 9083 | 0.995501 | 0.319493 | 0.533342 | FALSE |
| KIAA2026 | 8399 | 0.995485 | 0.3195 | 0.533342 | FALSE |
| RANBP1 | 287 | 0.995282 | 0.319599 | 0.533378 | FALSE |
| LYRM7 | 12312 | 0.994552 | 0.319954 | 0.533583 | FALSE |
| SMIM10L1 | 5337 | 0.994304 | 0.320075 | 0.533638 | FALSE |
| SESN2 | 7810 | 0.994279 | 0.320087 | 0.533638 | FALSE |
| SLC38A5 | 8201 | 0.994127 | 0.320161 | 0.533657 | FALSE |
| TICAM1 | 15517 | 0.993981 | 0.320232 | 0.533657 | FALSE |
| ELAVL3 | 14411 | 0.993879 | 0.320282 | 0.533657 | FALSE |
| DYRK4 | 14479 | 0.993862 | 0.32029 | 0.533657 | FALSE |
| TMEM178B | 15527 | 0.993769 | 0.320335 | 0.533657 | FALSE |
| FAM133A | 14765 | 0.99276 | 0.320827 | 0.534025 | FALSE |
| PRLHR | 5613 | 0.99253 | 0.320939 | 0.53409 | FALSE |
| TMEM67 | 13573 | 0.992269 | 0.321066 | 0.534188 | FALSE |
| PRPF18 | 5790 | 0.991448 | 0.321467 | 0.53465 | FALSE |
| SS18 | 15413 | 0.991188 | 0.321594 | 0.53465 | FALSE |
| GPATCH2L | 3592 | 0.991114 | 0.32163 | 0.53465 | FALSE |
| ARFGAP2 | 7849 | 0.991071 | 0.321651 | 0.53465 | FALSE |
| RLIM | 15233 | 0.991041 | 0.321666 | 0.53465 | FALSE |
| NXNL2 | 13836 | 0.991032 | 0.32167 | 0.53465 | FALSE |
| TJP1 | 390 | 0.990747 | 0.321809 | 0.534669 | FALSE |
| SLTM | 14010 | 0.990216 | 0.322069 | 0.534789 | FALSE |
| PARP16 | 10210 | 0.990197 | 0.322078 | 0.534789 | FALSE |
| RB1CC1 | 3865 | 0.989954 | 0.322197 | 0.534873 | FALSE |
| MPDU1 | 14051 | 0.989768 | 0.322288 | 0.534967 | FALSE |
| GIMAP6 | 4273 | 0.989383 | 0.322476 | 0.535223 | FALSE |
| ZNF468 | 15427 | 0.989276 | 0.322528 | 0.535238 | FALSE |
| SLC27A1 | 6921 | 0.989002 | 0.322662 | 0.535238 | FALSE |
| NUDT19 | 15092 | 0.988938 | 0.322693 | 0.535238 | FALSE |
| SLC22A7 | 14167 | 0.988932 | 0.322696 | 0.535238 | FALSE |
| LEFTY1 | 15504 | 0.988867 | 0.322728 | 0.535238 | FALSE |
| HDHD3 | 11869 | 0.988839 | 0.322742 | 0.535238 | FALSE |
| GM2A | 7681 | 0.988805 | 0.322759 | 0.535238 | FALSE |
| MACROD1 | 8704 | 0.988606 | 0.322856 | 0.535286 | FALSE |
| ANKRD49 | 15218 | 0.988098 | 0.323105 | 0.535576 | FALSE |
| NOP58 | 7835 | 0.987828 | 0.323237 | 0.535576 | FALSE |
| NKAPP1 | 11185 | 0.987648 | 0.323325 | 0.535666 | FALSE |
| MAGEE1 | 13879 | 0.987234 | 0.323528 | 0.535931 | FALSE |
| ARFRP1 | 2467 | 0.987181 | 0.323554 | 0.535931 | FALSE |
| COPZ2 | 1301 | 0.986762 | 0.323759 | 0.536158 | FALSE |
| CLCN6 | 14062 | 0.986545 | 0.323866 | 0.536187 | FALSE |
| GTF2B | 7049 | 0.986206 | 0.324032 | 0.536382 | FALSE |
| PIK3R1 | 6016 | 0.985859 | 0.324202 | 0.536593 | FALSE |
| PIK3IP1 | 874 | 0.985806 | 0.324228 | 0.536593 | FALSE |
| XRCC5 | 8552 | 0.985362 | 0.324446 | 0.536798 | FALSE |
| LRRC8D | 12131 | 0.985335 | 0.32446 | 0.536798 | FALSE |
| RCAN1 | 6064 | 0.985256 | 0.324498 | 0.536798 | FALSE |
| SLC4A10 | 7834 | 0.98505 | 0.3246 | 0.53681 | FALSE |
| RBM6 | 10263 | 0.984348 | 0.324944 | 0.537096 | FALSE |
| MYO5A | 2751 | 0.984206 | 0.325014 | 0.537107 | FALSE |
| ADD1 | 4064 | 0.982962 | 0.325626 | 0.537744 | FALSE |
| SIN3A | 3203 | 0.982948 | 0.325633 | 0.537744 | FALSE |
| PCDHGB7 | 15051 | 0.982814 | 0.325699 | 0.537775 | FALSE |
| SDE2 | 12521 | 0.982524 | 0.325842 | 0.537954 | FALSE |
| PHACTR1 | 3199 | 0.982147 | 0.326027 | 0.538203 | FALSE |
| PDE1C | 12468 | 0.981991 | 0.326104 | 0.538273 | FALSE |
| CARD14 | 10721 | 0.981317 | 0.326436 | 0.538637 | FALSE |
| ERCC6L2 | 9487 | 0.980294 | 0.326941 | 0.538987 | FALSE |
| IRF2BP1 | 4297 | 0.980275 | 0.32695 | 0.538987 | FALSE |
| ZNF805 | 7534 | 0.979743 | 0.327213 | 0.539255 | FALSE |
| FN3K | 2312 | 0.979736 | 0.327216 | 0.539255 | FALSE |
| ZNF324 | 10144 | 0.979611 | 0.327278 | 0.5393 | FALSE |
| ZNF33A | 10173 | 0.979083 | 0.327539 | 0.539559 | FALSE |
| AKAP12 | 8409 | 0.978927 | 0.327616 | 0.539572 | FALSE |
| ZNF76 | 10804 | 0.978697 | 0.32773 | 0.539693 | FALSE |
| MOCS3 | 4409 | 0.97863 | 0.327763 | 0.539693 | FALSE |
| CRYGD | 5716 | 0.978499 | 0.327828 | 0.539693 | FALSE |
| PIBF1 | 1419 | 0.978324 | 0.327914 | 0.539779 | FALSE |
| RRP8 | 9409 | 0.977951 | 0.328099 | 0.539845 | FALSE |
| BOLA3 | 11876 | 0.977864 | 0.328142 | 0.539845 | FALSE |
| FER | 2523 | 0.977237 | 0.328452 | 0.539946 | FALSE |
| GNA11 | 7306 | 0.977231 | 0.328455 | 0.539946 | FALSE |
| CNBP | 8256 | 0.976646 | 0.328744 | 0.540039 | FALSE |
| ARHGEF2 | 5793 | 0.976287 | 0.328922 | 0.540187 | FALSE |
| GGA3 | 12079 | 0.976039 | 0.329045 | 0.54023 | FALSE |
| LYSMD1 | 3254 | 0.976031 | 0.329049 | 0.54023 | FALSE |
| KRTAP5-8 | 7965 | 0.975982 | 0.329073 | 0.54023 | FALSE |
| ITSN1 | 12957 | 0.975956 | 0.329086 | 0.54023 | FALSE |
| NUDCD2 | 12169 | 0.975775 | 0.329176 | 0.54032 | FALSE |
| LRP1 | 9598 | 0.974864 | 0.329628 | 0.540834 | FALSE |
| DHX15 | 4695 | 0.973425 | 0.330342 | 0.541683 | FALSE |
| RAB7B | 2440 | 0.973192 | 0.330458 | 0.541798 | FALSE |
| AJM1 | 14605 | 0.97304 | 0.330533 | 0.541832 | FALSE |
| PTPRT | 1966 | 0.97206 | 0.331021 | 0.542266 | FALSE |
| MAN2A2 | 12463 | 0.971887 | 0.331107 | 0.542307 | FALSE |
| RAB4A | 14507 | 0.971584 | 0.331258 | 0.542484 | FALSE |
| PLEKHA1 | 5547 | 0.971295 | 0.331401 | 0.542662 | FALSE |
| MYEF2 | 6129 | 0.970945 | 0.331576 | 0.542777 | FALSE |
| EPHB1 | 2565 | 0.970368 | 0.331863 | 0.542963 | FALSE |
| GRWD1 | 4614 | 0.970081 | 0.332006 | 0.543084 | FALSE |
| COX17 | 15477 | 0.969691 | 0.332201 | 0.543174 | FALSE |
| LOC100129034 | 11816 | 0.969435 | 0.332328 | 0.543326 | FALSE |
| RFC5 | 7956 | 0.968973 | 0.332559 | 0.543533 | FALSE |
| COX18 | 4804 | 0.968865 | 0.332613 | 0.543564 | FALSE |
| TRIM66 | 14724 | 0.968722 | 0.332684 | 0.543624 | FALSE |
| NCBP3 | 6054 | 0.9676 | 0.333244 | 0.544295 | FALSE |
| KLHL33 | 13315 | 0.967551 | 0.333269 | 0.544295 | FALSE |
| XBP1 | 8597 | 0.967478 | 0.333305 | 0.544297 | FALSE |
| GNAI3 | 13453 | 0.967008 | 0.33354 | 0.544297 | FALSE |
| CDC25B | 11432 | 0.966991 | 0.333549 | 0.544297 | FALSE |
| HIST4H4 | 4252 | 0.966361 | 0.333864 | 0.544692 | FALSE |
| NRXN1 | 6854 | 0.966198 | 0.333945 | 0.544692 | FALSE |
| TSPYL4 | 14101 | 0.966158 | 0.333965 | 0.544692 | FALSE |
| PBX3 | 6889 | 0.965966 | 0.334061 | 0.544718 | FALSE |
| ABAT | 2721 | 0.965345 | 0.334372 | 0.545072 | FALSE |
| CHM | 7232 | 0.964145 | 0.334973 | 0.545807 | FALSE |
| DNMBP | 8817 | 0.963726 | 0.335183 | 0.545939 | FALSE |
| ANKRD23 | 797 | 0.961968 | 0.336066 | 0.546832 | FALSE |
| EARS2 | 8843 | 0.961665 | 0.336218 | 0.546997 | FALSE |
| RPS6KA3 | 5980 | 0.96061 | 0.336748 | 0.547702 | FALSE |
| ZNF266 | 11617 | 0.960517 | 0.336795 | 0.547708 | FALSE |
| JPH3 | 12692 | 0.959829 | 0.337141 | 0.548214 | FALSE |
| PTPN13 | 12762 | 0.959685 | 0.337214 | 0.548275 | FALSE |
| PDE4DIPP1 | 13913 | 0.959275 | 0.33742 | 0.54855 | FALSE |
| MATK | 15012 | 0.959209 | 0.337453 | 0.54855 | FALSE |
| NSMCE4A | 1640 | 0.958056 | 0.338035 | 0.549381 | FALSE |
| ZNF256 | 6368 | 0.957943 | 0.338092 | 0.549416 | FALSE |
| RBMS3 | 14030 | 0.957567 | 0.338281 | 0.549496 | FALSE |
| P4HTM | 14755 | 0.956929 | 0.338603 | 0.54979 | FALSE |
| HEATR4 | 11452 | 0.956528 | 0.338806 | 0.549948 | FALSE |
| VAX2 | 6183 | 0.956353 | 0.338894 | 0.550034 | FALSE |
| CLN5 | 10687 | 0.955888 | 0.339129 | 0.550301 | FALSE |
| MFSD2A | 13812 | 0.955547 | 0.339301 | 0.55048 | FALSE |
| RPS6KA2 | 14950 | 0.955379 | 0.339386 | 0.55049 | FALSE |
| VPS13A | 10950 | 0.955111 | 0.339522 | 0.550636 | FALSE |
| TNFSF10 | 9084 | 0.954589 | 0.339786 | 0.550966 | FALSE |
| METTL4 | 1278 | 0.954009 | 0.340079 | 0.551271 | FALSE |
| ZBTB18 | 6327 | 0.953902 | 0.340133 | 0.551302 | FALSE |
| STK38L | 10661 | 0.953271 | 0.340453 | 0.551697 | FALSE |
| UTP3 | 6725 | 0.95238 | 0.340904 | 0.552243 | FALSE |
| MRPS31 | 453 | 0.952337 | 0.340926 | 0.552243 | FALSE |
| SAMD12 | 185 | 0.952216 | 0.340987 | 0.552285 | FALSE |
| PTK2 | 6500 | 0.95195 | 0.341122 | 0.552446 | FALSE |
| ZNF430 | 767 | 0.951844 | 0.341176 | 0.552476 | FALSE |
| CPEB4 | 7822 | 0.951704 | 0.341247 | 0.552534 | FALSE |
| RFPL3 | 3406 | 0.950987 | 0.341611 | 0.552951 | FALSE |
| TMEM242 | 8429 | 0.95062 | 0.341797 | 0.553064 | FALSE |
| JAM3 | 5729 | 0.950571 | 0.341822 | 0.553064 | FALSE |
| ZBTB10 | 9586 | 0.950005 | 0.34211 | 0.553358 | FALSE |
| PDLIM2 | 9422 | 0.949734 | 0.342247 | 0.553508 | FALSE |
| PRMT7 | 372 | 0.949589 | 0.342321 | 0.553528 | FALSE |
| MTMR4 | 6090 | 0.94901 | 0.342616 | 0.553858 | FALSE |
| PPL | 11961 | 0.948928 | 0.342657 | 0.553858 | FALSE |
| PTPN1 | 8243 | 0.948632 | 0.342808 | 0.55389 | FALSE |
| CKS2 | 855 | 0.948586 | 0.342831 | 0.55389 | FALSE |
| AVP | 10715 | 0.948306 | 0.342974 | 0.553895 | FALSE |
| RGMA | 10336 | 0.948165 | 0.343045 | 0.553954 | FALSE |
| BTN2A3P | 8869 | 0.947937 | 0.343162 | 0.554057 | FALSE |
| FKBP7 | 13598 | 0.947601 | 0.343333 | 0.554132 | FALSE |
| PBRM1 | 8769 | 0.947508 | 0.34338 | 0.554151 | FALSE |
| KIAA0319L | 11822 | 0.947369 | 0.343451 | 0.554208 | FALSE |
| KATNBL1 | 7412 | 0.94728 | 0.343496 | 0.554224 | FALSE |
| ZFP37 | 15483 | 0.94686 | 0.34371 | 0.554486 | FALSE |
| LYAR | 14563 | 0.946412 | 0.343938 | 0.554651 | FALSE |
| GPR150 | 7075 | 0.946009 | 0.344144 | 0.554899 | FALSE |
| SMYD4 | 8722 | 0.945558 | 0.344374 | 0.555139 | FALSE |
| CD274 | 10248 | 0.944727 | 0.344798 | 0.555435 | FALSE |
| FAM189B | 14815 | 0.944694 | 0.344815 | 0.555435 | FALSE |
| TMCO1 | 11018 | 0.943765 | 0.34529 | 0.555998 | FALSE |
| EIF2AK3 | 14566 | 0.943731 | 0.345307 | 0.555998 | FALSE |
| SPRY3 | 2568 | 0.943305 | 0.345525 | 0.556181 | FALSE |
| CBY1 | 5093 | 0.943266 | 0.345545 | 0.556181 | FALSE |
| ZNF302 | 4750 | 0.943089 | 0.345635 | 0.556183 | FALSE |
| RAB35 | 11931 | 0.942808 | 0.345779 | 0.5563 | FALSE |
| POLB | 4555 | 0.94273 | 0.345819 | 0.556307 | FALSE |
| ZNF343 | 14787 | 0.942467 | 0.345954 | 0.556409 | FALSE |
| TRPM4 | 7552 | 0.942265 | 0.346057 | 0.556496 | FALSE |
| PER2 | 2217 | 0.942222 | 0.346079 | 0.556496 | FALSE |
| MYCBPAP | 1818 | 0.94156 | 0.346418 | 0.556926 | FALSE |
| AMMECR1L | 9584 | 0.941268 | 0.346568 | 0.557102 | FALSE |
| TMEM56 | 14526 | 0.940782 | 0.346817 | 0.557395 | FALSE |
| RASL11A | 9340 | 0.940289 | 0.347069 | 0.557726 | FALSE |
| NUP43 | 3645 | 0.940242 | 0.347093 | 0.557726 | FALSE |
| STAT5B | 569 | 0.940029 | 0.347203 | 0.557738 | FALSE |
| RBM27 | 11751 | 0.940019 | 0.347208 | 0.557738 | FALSE |
| FICD | 497 | 0.939919 | 0.347259 | 0.557761 | FALSE |
| CCR9 | 12372 | 0.939304 | 0.347575 | 0.55804 | FALSE |
| DCAF12 | 3263 | 0.938695 | 0.347887 | 0.558427 | FALSE |
| C2orf49 | 14392 | 0.937463 | 0.34852 | 0.559058 | FALSE |
| PRKD3 | 11366 | 0.937331 | 0.348588 | 0.559058 | FALSE |
| RNF6 | 412 | 0.937299 | 0.348605 | 0.559058 | FALSE |
| SDHD | 1373 | 0.936956 | 0.348781 | 0.55923 | FALSE |
| HSD17B4 | 10905 | 0.936749 | 0.348888 | 0.559344 | FALSE |
| MTF2 | 12678 | 0.936207 | 0.349167 | 0.559676 | FALSE |
| MERTK | 2491 | 0.935658 | 0.349449 | 0.560072 | FALSE |
| ST6GAL2 | 1190 | 0.935451 | 0.349556 | 0.560135 | FALSE |
| PHF3 | 1169 | 0.935348 | 0.349609 | 0.560135 | FALSE |
| FAM193A | 1350 | 0.934692 | 0.349947 | 0.560491 | FALSE |
| RECQL | 13576 | 0.934664 | 0.349961 | 0.560491 | FALSE |
| CDK11B | 13899 | 0.934223 | 0.350189 | 0.560579 | FALSE |
| MRPL39 | 10028 | 0.934216 | 0.350192 | 0.560579 | FALSE |
| CLOCK | 4931 | 0.933987 | 0.350311 | 0.560705 | FALSE |
| PNISR | 3914 | 0.933645 | 0.350487 | 0.56079 | FALSE |
| GHDC | 5709 | 0.933586 | 0.350517 | 0.56079 | FALSE |
| TTC37 | 4527 | 0.933362 | 0.350633 | 0.560801 | FALSE |
| ZNF658B | 7814 | 0.933315 | 0.350657 | 0.560801 | FALSE |
| HBS1L | 1511 | 0.933028 | 0.350806 | 0.560923 | FALSE |
| CCDC59 | 12795 | 0.932664 | 0.350993 | 0.561048 | FALSE |
| WDR47 | 7371 | 0.93264 | 0.351006 | 0.561048 | FALSE |
| FAM228A | 6483 | 0.9326 | 0.351027 | 0.561048 | FALSE |
| KRTAP4-11 | 8807 | 0.932148 | 0.35126 | 0.561352 | FALSE |
| PRRT2 | 11223 | 0.931304 | 0.351696 | 0.561715 | FALSE |
| PRKAG2 | 1090 | 0.931221 | 0.351739 | 0.561716 | FALSE |
| TOMM40 | 395 | 0.931164 | 0.351769 | 0.561716 | FALSE |
| ZNF92 | 6520 | 0.930604 | 0.352058 | 0.562121 | FALSE |
| GARS | 9993 | 0.930309 | 0.352211 | 0.562173 | FALSE |
| SOS1 | 6633 | 0.930263 | 0.352235 | 0.562173 | FALSE |
| G6PD | 8536 | 0.929246 | 0.352762 | 0.562899 | FALSE |
| PDIA6 | 5646 | 0.928918 | 0.352932 | 0.563027 | FALSE |
| OR7E156P | 14042 | 0.928883 | 0.35295 | 0.563027 | FALSE |
| ALOX12P2 | 7292 | 0.928466 | 0.353166 | 0.563199 | FALSE |
| RPS6KL1 | 2784 | 0.928093 | 0.353359 | 0.563393 | FALSE |
| HIRIP3 | 3192 | 0.927908 | 0.353455 | 0.563488 | FALSE |
| TASP1 | 1475 | 0.927501 | 0.353666 | 0.563768 | FALSE |
| CIR1 | 4823 | 0.926973 | 0.353941 | 0.564089 | FALSE |
| DUSP5 | 8652 | 0.926475 | 0.354199 | 0.564329 | FALSE |
| PPM1A | 4810 | 0.92629 | 0.354295 | 0.564407 | FALSE |
| IGF2R | 5878 | 0.926134 | 0.354376 | 0.564407 | FALSE |
| TMEM109 | 14376 | 0.925803 | 0.354548 | 0.564483 | FALSE |
| MTMR7 | 7794 | 0.925039 | 0.354946 | 0.564942 | FALSE |
| LINC00537 | 5921 | 0.924694 | 0.355125 | 0.565171 | FALSE |
| INVS | 10696 | 0.924477 | 0.355238 | 0.565246 | FALSE |
| MORC2 | 9973 | 0.923968 | 0.355503 | 0.56536 | FALSE |
| SYNGAP1 | 4288 | 0.923909 | 0.355534 | 0.56536 | FALSE |
| RNFT1 | 14751 | 0.923743 | 0.35562 | 0.565383 | FALSE |
| SNHG21 | 180 | 0.92321 | 0.355898 | 0.565709 | FALSE |
| ANP32B | 8535 | 0.922852 | 0.356084 | 0.565878 | FALSE |
| IFT122 | 9082 | 0.922602 | 0.356215 | 0.565983 | FALSE |
| ZNF570 | 13575 | 0.922171 | 0.356439 | 0.566282 | FALSE |
| SAMD13 | 846 | 0.921947 | 0.356556 | 0.56641 | FALSE |
| RNF213 | 11485 | 0.921361 | 0.356862 | 0.566781 | FALSE |
| FTCD | 7878 | 0.920733 | 0.35719 | 0.567244 | FALSE |
| PLLP | 12362 | 0.919379 | 0.357897 | 0.568223 | FALSE |
| CHST5 | 8566 | 0.919344 | 0.357916 | 0.568223 | FALSE |
| PRRG1 | 9920 | 0.918547 | 0.358333 | 0.568712 | FALSE |
| GRIP1 | 13629 | 0.917781 | 0.358734 | 0.569117 | FALSE |
| LOC643387 | 13363 | 0.917232 | 0.359021 | 0.569458 | FALSE |
| C6orf106 | 9655 | 0.916955 | 0.359166 | 0.569572 | FALSE |
| TMCO6 | 8498 | 0.916704 | 0.359298 | 0.569666 | FALSE |
| LCE3C | 8469 | 0.916505 | 0.359402 | 0.569773 | FALSE |
| ZNF527 | 10752 | 0.915343 | 0.360012 | 0.570432 | FALSE |
| LINC01260 | 4405 | 0.915225 | 0.360074 | 0.570433 | FALSE |
| TBC1D2 | 2576 | 0.915066 | 0.360157 | 0.570451 | FALSE |
| RNF20 | 2835 | 0.914828 | 0.360282 | 0.570589 | FALSE |
| RNF125 | 5428 | 0.914245 | 0.360588 | 0.570917 | FALSE |
| TIMM8A | 8454 | 0.914146 | 0.36064 | 0.570925 | FALSE |
| C1orf112 | 1672 | 0.913746 | 0.36085 | 0.571177 | FALSE |
| WDR43 | 10612 | 0.913635 | 0.360909 | 0.571177 | FALSE |
| LOC440311 | 6815 | 0.913105 | 0.361187 | 0.571502 | FALSE |
| PRKCI | 15570 | 0.912807 | 0.361344 | 0.571617 | FALSE |
| RHOT2 | 13390 | 0.912759 | 0.361369 | 0.571617 | FALSE |
| FBXO4 | 184 | 0.912362 | 0.361578 | 0.571773 | FALSE |
| THNSL1 | 5418 | 0.911912 | 0.361815 | 0.571995 | FALSE |
| CCDC66 | 13350 | 0.911887 | 0.361828 | 0.571995 | FALSE |
| KIF1C | 15275 | 0.911581 | 0.361989 | 0.572091 | FALSE |
| IMPG2 | 2026 | 0.911563 | 0.361999 | 0.572091 | FALSE |
| OTUD4 | 1281 | 0.910493 | 0.362563 | 0.572693 | FALSE |
| ZNF765 | 9172 | 0.910288 | 0.362671 | 0.572739 | FALSE |
| BCL2L11 | 13416 | 0.910273 | 0.362679 | 0.572739 | FALSE |
| NSUN5 | 14514 | 0.910229 | 0.362702 | 0.572739 | FALSE |
| MRPS5 | 8328 | 0.909926 | 0.362862 | 0.572876 | FALSE |
| TNFSF13 | 6716 | 0.909605 | 0.363031 | 0.573085 | FALSE |
| AKR1E2 | 4140 | 0.909304 | 0.36319 | 0.57322 | FALSE |
| LENEP | 5676 | 0.908788 | 0.363462 | 0.573491 | FALSE |
| ANO4 | 15420 | 0.908752 | 0.363481 | 0.573491 | FALSE |
| CUL4B | 8683 | 0.908631 | 0.363545 | 0.573491 | FALSE |
| KLF14 | 9147 | 0.90809 | 0.363831 | 0.573653 | FALSE |
| LRRC26 | 9000 | 0.907811 | 0.363978 | 0.573776 | FALSE |
| LCE2C | 9687 | 0.907776 | 0.363997 | 0.573776 | FALSE |
| FMC1 | 14281 | 0.907611 | 0.364084 | 0.573796 | FALSE |
| DLK1 | 2005 | 0.90757 | 0.364105 | 0.573796 | FALSE |
| PTCD2 | 12864 | 0.907176 | 0.364314 | 0.573951 | FALSE |
| ACTR6 | 15514 | 0.907026 | 0.364393 | 0.57397 | FALSE |
| RAD51-AS1 | 12193 | 0.906636 | 0.364599 | 0.574227 | FALSE |
| ZDHHC11 | 2572 | 0.906517 | 0.364662 | 0.574269 | FALSE |
| SGPP1 | 11548 | 0.905962 | 0.364956 | 0.574673 | FALSE |
| DUSP12 | 11175 | 0.905731 | 0.365078 | 0.574736 | FALSE |
| MTMR1 | 5149 | 0.905701 | 0.365094 | 0.574736 | FALSE |
| PSMD2 | 8727 | 0.905678 | 0.365106 | 0.574736 | FALSE |
| ADARB1 | 12759 | 0.904902 | 0.365517 | 0.57516 | FALSE |
| USH1C | 5455 | 0.904892 | 0.365523 | 0.57516 | FALSE |
| IFT81 | 1081 | 0.904506 | 0.365727 | 0.575424 | FALSE |
| SMIM8 | 10444 | 0.904064 | 0.365961 | 0.575677 | FALSE |
| LIPE | 10908 | 0.903714 | 0.366147 | 0.575911 | FALSE |
| MAU2 | 4648 | 0.903626 | 0.366194 | 0.575926 | FALSE |
| UQCRBP1 | 7757 | 0.903182 | 0.366429 | 0.576123 | FALSE |
| PANX3 | 14916 | 0.901572 | 0.367284 | 0.577235 | FALSE |
| APBB2 | 15338 | 0.900515 | 0.367846 | 0.577998 | FALSE |
| RFX7 | 8182 | 0.900256 | 0.367984 | 0.578044 | FALSE |
| BTN3A3 | 9045 | 0.899477 | 0.368399 | 0.578579 | FALSE |
| DDX59 | 5011 | 0.899379 | 0.368451 | 0.578603 | FALSE |
| LCLAT1 | 5334 | 0.899032 | 0.368636 | 0.578835 | FALSE |
| CD3EAP | 6792 | 0.898519 | 0.368909 | 0.579031 | FALSE |
| ZNF160 | 1154 | 0.898239 | 0.369058 | 0.579079 | FALSE |
| ZNF530 | 15262 | 0.898226 | 0.369065 | 0.579079 | FALSE |
| TAF5 | 4010 | 0.898174 | 0.369093 | 0.579079 | FALSE |
| PRPSAP1 | 13302 | 0.897652 | 0.369371 | 0.57939 | FALSE |
| ESAM | 293 | 0.897061 | 0.369686 | 0.579786 | FALSE |
| FTCDNL1 | 11045 | 0.896598 | 0.369933 | 0.580103 | FALSE |
| CDC73 | 6829 | 0.896474 | 0.37 | 0.580103 | FALSE |
| RANBP9 | 6149 | 0.895927 | 0.370292 | 0.580503 | FALSE |
| ATF4 | 7180 | 0.895085 | 0.370742 | 0.580813 | FALSE |
| SLC46A1 | 3118 | 0.894988 | 0.370794 | 0.580823 | FALSE |
| CEP78 | 10194 | 0.894142 | 0.371246 | 0.581241 | FALSE |
| PDK3 | 12087 | 0.893995 | 0.371325 | 0.581306 | FALSE |
| MCM2 | 14259 | 0.893346 | 0.371672 | 0.581616 | FALSE |
| INTS6L | 6131 | 0.89261 | 0.372066 | 0.582042 | FALSE |
| FAM35A | 11620 | 0.892491 | 0.37213 | 0.582042 | FALSE |
| GTF2IRD1P1 | 13825 | 0.892412 | 0.372172 | 0.582044 | FALSE |
| ANXA5 | 10865 | 0.891947 | 0.372421 | 0.582314 | FALSE |
| REPS2 | 12504 | 0.891888 | 0.372453 | 0.582314 | FALSE |
| SBNO2 | 2369 | 0.891806 | 0.372497 | 0.582324 | FALSE |
| SCAMP4 | 5504 | 0.891393 | 0.372718 | 0.582496 | FALSE |
| SHC3 | 2314 | 0.89114 | 0.372854 | 0.582591 | FALSE |
| RNF185 | 11666 | 0.88997 | 0.373482 | 0.583398 | FALSE |
| KIAA0825 | 10867 | 0.889752 | 0.373599 | 0.583503 | FALSE |
| TAS2R20 | 13649 | 0.889586 | 0.373688 | 0.583503 | FALSE |
| TBX2-AS1 | 10811 | 0.889498 | 0.373735 | 0.583503 | FALSE |
| OSBPL11 | 197 | 0.889477 | 0.373747 | 0.583503 | FALSE |
| LRTM2 | 10509 | 0.889427 | 0.373774 | 0.583503 | FALSE |
| ZNF672 | 10621 | 0.889326 | 0.373828 | 0.58353 | FALSE |
| CLK2 | 1787 | 0.888803 | 0.374109 | 0.583653 | FALSE |
| ZNF511 | 9287 | 0.888395 | 0.374328 | 0.583879 | FALSE |
| SF1 | 14469 | 0.888314 | 0.374372 | 0.583879 | FALSE |
| EED | 13288 | 0.888285 | 0.374387 | 0.583879 | FALSE |
| ZNF699 | 8299 | 0.887703 | 0.374701 | 0.584147 | FALSE |
| CNTN2 | 4500 | 0.887687 | 0.374709 | 0.584147 | FALSE |
| FCHO1 | 5934 | 0.887386 | 0.374871 | 0.584283 | FALSE |
| CRELD1 | 7011 | 0.887317 | 0.374908 | 0.584283 | FALSE |
| SEC16A | 15022 | 0.8869 | 0.375133 | 0.58447 | FALSE |
| KIF6 | 8137 | 0.886885 | 0.375141 | 0.58447 | FALSE |
| CFL1 | 4078 | 0.886494 | 0.375351 | 0.58474 | FALSE |
| ELP2 | 10402 | 0.88614 | 0.375542 | 0.584862 | FALSE |
| REXO4 | 4024 | 0.88532 | 0.375984 | 0.585492 | FALSE |
| CAND1 | 15121 | 0.88516 | 0.37607 | 0.585568 | FALSE |
| SP2 | 4223 | 0.885049 | 0.37613 | 0.585603 | FALSE |
| TSC22D3 | 11802 | 0.884849 | 0.376238 | 0.585713 | FALSE |
| RBMXL1 | 8955 | 0.884779 | 0.376276 | 0.585713 | FALSE |
| KIF4B | 7730 | 0.884269 | 0.376551 | 0.585929 | FALSE |
| HRC | 10683 | 0.884252 | 0.37656 | 0.585929 | FALSE |
| LOC101927765 | 12333 | 0.884244 | 0.376565 | 0.585929 | FALSE |
| MIER3 | 9443 | 0.883946 | 0.376725 | 0.586065 | FALSE |
| C2orf15 | 5888 | 0.883191 | 0.377133 | 0.586429 | FALSE |
| MYLK4 | 1714 | 0.883147 | 0.377157 | 0.586429 | FALSE |
| MDM1 | 7563 | 0.883094 | 0.377185 | 0.586429 | FALSE |
| RFPL1S | 9760 | 0.882884 | 0.377299 | 0.58647 | FALSE |
| DISP1 | 7522 | 0.882875 | 0.377304 | 0.58647 | FALSE |
| DNAJC13 | 9231 | 0.882761 | 0.377365 | 0.586475 | FALSE |
| TNFRSF11B | 9268 | 0.882429 | 0.377545 | 0.586639 | FALSE |
| MTMR6 | 8164 | 0.882427 | 0.377546 | 0.586639 | FALSE |
| CWC25 | 10373 | 0.882348 | 0.377589 | 0.586647 | FALSE |
| RALY | 11023 | 0.882177 | 0.377681 | 0.586732 | FALSE |
| GEMIN5 | 5165 | 0.88188 | 0.377842 | 0.586924 | FALSE |
| RDM1 | 249 | 0.881644 | 0.377969 | 0.587064 | FALSE |
| CFLAR | 11012 | 0.881008 | 0.378313 | 0.587311 | FALSE |
| EXOSC2 | 5662 | 0.881002 | 0.378317 | 0.587311 | FALSE |
| L3MBTL2 | 1731 | 0.880908 | 0.378368 | 0.587331 | FALSE |
| FBXO42 | 3280 | 0.880715 | 0.378472 | 0.587378 | FALSE |
| KCTD13 | 1845 | 0.880355 | 0.378667 | 0.587512 | FALSE |
| MIPOL1 | 6270 | 0.880248 | 0.378725 | 0.587512 | FALSE |
| EFCAB2 | 4728 | 0.880208 | 0.378747 | 0.587512 | FALSE |
| POPDC3 | 274 | 0.880114 | 0.378798 | 0.587533 | FALSE |
| CLEC2L | 1347 | 0.879344 | 0.379215 | 0.588063 | FALSE |
| TBC1D5 | 8665 | 0.878911 | 0.37945 | 0.588358 | FALSE |
| UBR3 | 7620 | 0.878683 | 0.379573 | 0.588385 | FALSE |
| ASXL2 | 10419 | 0.878527 | 0.379658 | 0.588458 | FALSE |
| ZNF519 | 11265 | 0.878147 | 0.379864 | 0.588719 | FALSE |
| ILK | 10098 | 0.877826 | 0.380038 | 0.588931 | FALSE |
| LOC151174 | 13696 | 0.877412 | 0.380263 | 0.589126 | FALSE |
| NDST1 | 8495 | 0.877386 | 0.380277 | 0.589126 | FALSE |
| ANAPC1 | 14426 | 0.875962 | 0.381051 | 0.589875 | FALSE |
| SLC6A12 | 3732 | 0.87594 | 0.381063 | 0.589875 | FALSE |
| UTP15 | 1574 | 0.87441 | 0.381895 | 0.590871 | FALSE |
| POLR3F | 11602 | 0.873743 | 0.382258 | 0.591283 | FALSE |
| HCRTR1 | 3425 | 0.87368 | 0.382293 | 0.591283 | FALSE |
| WDR53 | 6579 | 0.873633 | 0.382318 | 0.591283 | FALSE |
| URGCP | 6607 | 0.873585 | 0.382344 | 0.591283 | FALSE |
| PAPSS2 | 12670 | 0.873574 | 0.38235 | 0.591283 | FALSE |
| C2orf54 | 9537 | 0.87312 | 0.382598 | 0.591549 | FALSE |
| CD2AP | 13200 | 0.872485 | 0.382944 | 0.591857 | FALSE |
| NOA1 | 11942 | 0.872104 | 0.383152 | 0.592091 | FALSE |
| TXNDC16 | 10780 | 0.871848 | 0.383291 | 0.592185 | FALSE |
| C8orf33 | 8099 | 0.87181 | 0.383312 | 0.592185 | FALSE |
| DNAJC8 | 5976 | 0.871616 | 0.383418 | 0.592275 | FALSE |
| STMP1 | 321 | 0.871495 | 0.383484 | 0.592275 | FALSE |
| C12orf57 | 7596 | 0.871034 | 0.383736 | 0.592546 | FALSE |
| KRTAP10-5 | 8253 | 0.870868 | 0.383826 | 0.592549 | FALSE |
| UCN | 8057 | 0.870837 | 0.383843 | 0.592549 | FALSE |
| MELTF-AS1 | 4146 | 0.870802 | 0.383862 | 0.592549 | FALSE |
| MAP3K4 | 9979 | 0.870207 | 0.384187 | 0.592892 | FALSE |
| GZMM | 2213 | 0.869657 | 0.384488 | 0.593239 | FALSE |
| AUH | 6510 | 0.869339 | 0.384662 | 0.593449 | FALSE |
| NOP14-AS1 | 3007 | 0.868539 | 0.385099 | 0.593948 | FALSE |
| BICDL2 | 7281 | 0.868229 | 0.385269 | 0.594089 | FALSE |
| FKBP15 | 5282 | 0.868164 | 0.385305 | 0.594089 | FALSE |
| C21orf91 | 10987 | 0.867602 | 0.385612 | 0.594438 | FALSE |
| IFT52 | 5092 | 0.867542 | 0.385645 | 0.594438 | FALSE |
| GRID1 | 3234 | 0.866887 | 0.386004 | 0.594815 | FALSE |
| CIRBP | 12736 | 0.866307 | 0.386322 | 0.595129 | FALSE |
| TTC21A | 1085 | 0.866167 | 0.386399 | 0.595143 | FALSE |
| HPS1 | 7155 | 0.866082 | 0.386445 | 0.595143 | FALSE |
| DAAM2 | 6970 | 0.866005 | 0.386487 | 0.59515 | FALSE |
| ANKRD20A5P | 10600 | 0.865599 | 0.38671 | 0.595434 | FALSE |
| RNFT2 | 9200 | 0.86477 | 0.387165 | 0.595988 | FALSE |
| METAP2 | 996 | 0.864735 | 0.387184 | 0.595988 | FALSE |
| ZNF600 | 244 | 0.86399 | 0.387593 | 0.596559 | FALSE |
| NNT | 9375 | 0.863878 | 0.387655 | 0.596595 | FALSE |
| FBXO31 | 1197 | 0.86357 | 0.387824 | 0.596738 | FALSE |
| ANKRD36BP1 | 8462 | 0.8633 | 0.387973 | 0.596907 | FALSE |
| POLD3 | 14971 | 0.863017 | 0.388128 | 0.597029 | FALSE |
| TNPO3 | 9087 | 0.86273 | 0.388286 | 0.597096 | FALSE |
| TRIM3 | 7888 | 0.8622 | 0.388577 | 0.597368 | FALSE |
| FAM133B | 11153 | 0.861699 | 0.388853 | 0.597604 | FALSE |
| TACO1 | 14165 | 0.86157 | 0.388924 | 0.597604 | FALSE |
| LIN54 | 14528 | 0.860483 | 0.389523 | 0.598233 | FALSE |
| ZSCAN25 | 3698 | 0.860346 | 0.389598 | 0.598242 | FALSE |
| NAA16 | 10675 | 0.860333 | 0.389606 | 0.598242 | FALSE |
| KLF2 | 4306 | 0.860198 | 0.38968 | 0.598298 | FALSE |
| OSTCP1 | 5859 | 0.860076 | 0.389747 | 0.598342 | FALSE |
| SLC6A17 | 11020 | 0.859442 | 0.390097 | 0.598644 | FALSE |
| TRAM2-AS1 | 10765 | 0.859167 | 0.390248 | 0.598818 | FALSE |
| ZNF446 | 9470 | 0.85876 | 0.390473 | 0.599017 | FALSE |
| FLYWCH2 | 1932 | 0.858335 | 0.390708 | 0.599017 | FALSE |
| PLEKHG4 | 959 | 0.858304 | 0.390725 | 0.599017 | FALSE |
| SRSF8 | 11582 | 0.858237 | 0.390762 | 0.599017 | FALSE |
| KLHL28 | 3861 | 0.857504 | 0.391166 | 0.599403 | FALSE |
| TTTY14 | 12583 | 0.857334 | 0.39126 | 0.599488 | FALSE |
| CERCAM | 8378 | 0.857007 | 0.391441 | 0.599602 | FALSE |
| MYBPC3 | 7569 | 0.85636 | 0.391799 | 0.600018 | FALSE |
| BCL2 | 9158 | 0.85524 | 0.392418 | 0.600673 | FALSE |
| NSUN3 | 1892 | 0.854265 | 0.392958 | 0.601264 | FALSE |
| IFITM3 | 9714 | 0.854046 | 0.39308 | 0.601391 | FALSE |
| CHSY3 | 11167 | 0.853644 | 0.393302 | 0.601592 | FALSE |
| CEBPZOS | 13666 | 0.8536 | 0.393327 | 0.601592 | FALSE |
| IFT88 | 13704 | 0.853164 | 0.393568 | 0.601785 | FALSE |
| FANCB | 11518 | 0.853022 | 0.393647 | 0.601847 | FALSE |
| TDP1 | 4481 | 0.851938 | 0.394249 | 0.602649 | FALSE |
| CRCP | 12505 | 0.851185 | 0.394667 | 0.603007 | FALSE |
| UBAP2L | 2256 | 0.851097 | 0.394715 | 0.603009 | FALSE |
| MYO6 | 4509 | 0.850503 | 0.395045 | 0.603454 | FALSE |
| TOR1AIP2 | 14239 | 0.84999 | 0.395331 | 0.603662 | FALSE |
| INTS6 | 962 | 0.849782 | 0.395446 | 0.603712 | FALSE |
| OSBPL3 | 4932 | 0.849341 | 0.395692 | 0.603851 | FALSE |
| PI4KAP2 | 12777 | 0.849064 | 0.395846 | 0.604027 | FALSE |
| FAF2 | 3813 | 0.848495 | 0.396162 | 0.604451 | FALSE |
| PCYOX1 | 8745 | 0.847989 | 0.396444 | 0.604693 | FALSE |
| PLAA | 9327 | 0.84796 | 0.39646 | 0.604693 | FALSE |
| IFFO1 | 10673 | 0.847769 | 0.396567 | 0.604773 | FALSE |
| TMSB15A | 4367 | 0.846954 | 0.397021 | 0.605229 | FALSE |
| CYCS | 5542 | 0.846842 | 0.397083 | 0.605265 | FALSE |
| EHD3 | 7897 | 0.846507 | 0.39727 | 0.605429 | FALSE |
| PCDH11X | 12002 | 0.846147 | 0.397471 | 0.605429 | FALSE |
| PTH1R | 6987 | 0.846127 | 0.397482 | 0.605429 | FALSE |
| PATE3 | 7120 | 0.846112 | 0.39749 | 0.605429 | FALSE |
| ZGRF1 | 692 | 0.845145 | 0.39803 | 0.605951 | FALSE |
| POM121 | 10087 | 0.845004 | 0.398109 | 0.605951 | FALSE |
| CARS | 15291 | 0.844994 | 0.398114 | 0.605951 | FALSE |
| NSD1 | 4491 | 0.844398 | 0.398447 | 0.60628 | FALSE |
| KBTBD6 | 1986 | 0.843689 | 0.398843 | 0.606824 | FALSE |
| CLUHP3 | 8250 | 0.843507 | 0.398945 | 0.606866 | FALSE |
| GYS1 | 3756 | 0.843501 | 0.398948 | 0.606866 | FALSE |
| TMEM135 | 7539 | 0.843036 | 0.399208 | 0.607143 | FALSE |
| ZDHHC6 | 13956 | 0.84264 | 0.39943 | 0.607362 | FALSE |
| DCC | 7535 | 0.842464 | 0.399528 | 0.607452 | FALSE |
| CARF | 619 | 0.842278 | 0.399632 | 0.607552 | FALSE |
| LRIF1 | 7662 | 0.841965 | 0.399808 | 0.60768 | FALSE |
| SYNRG | 7353 | 0.841913 | 0.399837 | 0.60768 | FALSE |
| ZNF485 | 7982 | 0.841849 | 0.399872 | 0.60768 | FALSE |
| ASGR1 | 14103 | 0.841226 | 0.400221 | 0.607805 | FALSE |
| ABHD13 | 11964 | 0.841216 | 0.400227 | 0.607805 | FALSE |
| MCM4 | 13909 | 0.840656 | 0.400541 | 0.608223 | FALSE |
| DOCK1 | 7206 | 0.840257 | 0.400764 | 0.608503 | FALSE |
| DANCR | 15480 | 0.839992 | 0.400913 | 0.60867 | FALSE |
| CEP57L1 | 5153 | 0.839391 | 0.40125 | 0.609122 | FALSE |
| MINDY1 | 10481 | 0.838497 | 0.401752 | 0.609529 | FALSE |
| LAYN | 8687 | 0.838338 | 0.401841 | 0.609604 | FALSE |
| PLOD1 | 13976 | 0.8382 | 0.401918 | 0.609604 | FALSE |
| STRN3 | 7528 | 0.837675 | 0.402213 | 0.609815 | FALSE |
| PRIMPOL | 7308 | 0.836861 | 0.402671 | 0.610331 | FALSE |
| GPSM3 | 2971 | 0.83629 | 0.402992 | 0.610699 | FALSE |
| IDH3G | 13979 | 0.834708 | 0.403882 | 0.611426 | FALSE |
| MEG8 | 6719 | 0.834685 | 0.403895 | 0.611426 | FALSE |
| CAPN2 | 3329 | 0.834491 | 0.404004 | 0.611426 | FALSE |
| PARN | 11559 | 0.834475 | 0.404013 | 0.611426 | FALSE |
| DLAT | 1933 | 0.833941 | 0.404314 | 0.611639 | FALSE |
| SH2B1 | 3646 | 0.833527 | 0.404548 | 0.61187 | FALSE |
| VMAC | 10359 | 0.833304 | 0.404673 | 0.611972 | FALSE |
| GJC2 | 1916 | 0.833269 | 0.404693 | 0.611972 | FALSE |
| UTP18 | 6096 | 0.832805 | 0.404955 | 0.612308 | FALSE |
| FRMD4B | 6691 | 0.832279 | 0.405251 | 0.612639 | FALSE |
| SLC26A8 | 10195 | 0.831681 | 0.405589 | 0.61309 | FALSE |
| POLR2D | 15145 | 0.831125 | 0.405903 | 0.613292 | FALSE |
| NCAPH | 9303 | 0.829434 | 0.406859 | 0.614415 | FALSE |
| AGL | 14577 | 0.828637 | 0.40731 | 0.614859 | FALSE |
| USP18 | 10747 | 0.827953 | 0.407697 | 0.615265 | FALSE |
| LOC100335030 | 1497 | 0.82762 | 0.407886 | 0.61539 | FALSE |
| FANCM | 9044 | 0.827545 | 0.407928 | 0.61539 | FALSE |
| FAM178B | 15325 | 0.827023 | 0.408224 | 0.615703 | FALSE |
| CASP2 | 5261 | 0.826408 | 0.408573 | 0.615992 | FALSE |
| ARHGEF6 | 9056 | 0.826212 | 0.408684 | 0.6161 | FALSE |
| PCDHA5 | 13715 | 0.825936 | 0.40884 | 0.616248 | FALSE |
| SLC30A5 | 8124 | 0.825453 | 0.409114 | 0.616511 | FALSE |
| SLIT2 | 5226 | 0.825207 | 0.409254 | 0.616622 | FALSE |
| NTPCR | 15184 | 0.825184 | 0.409267 | 0.616622 | FALSE |
| POLR1E | 2157 | 0.824333 | 0.40975 | 0.617112 | FALSE |
| C18orf21 | 1598 | 0.823826 | 0.410038 | 0.617427 | FALSE |
| NOSTRIN | 6436 | 0.823439 | 0.410258 | 0.61764 | FALSE |
| LSR | 8316 | 0.823143 | 0.410427 | 0.617715 | FALSE |
| NCKIPSD | 8899 | 0.823142 | 0.410427 | 0.617715 | FALSE |
| DDX18 | 8289 | 0.822927 | 0.41055 | 0.61778 | FALSE |
| CPD | 15518 | 0.822686 | 0.410687 | 0.617868 | FALSE |
| ADAT1 | 11988 | 0.822384 | 0.410858 | 0.618057 | FALSE |
| BBOF1 | 14525 | 0.822326 | 0.410891 | 0.618057 | FALSE |
| MYBPH | 14470 | 0.822196 | 0.410965 | 0.618109 | FALSE |
| ZDHHC9 | 3634 | 0.822037 | 0.411056 | 0.618185 | FALSE |
| LOC729603 | 5181 | 0.821802 | 0.41119 | 0.618267 | FALSE |
| GTPBP6 | 5489 | 0.821681 | 0.411258 | 0.618312 | FALSE |
| P2RX7 | 4994 | 0.821602 | 0.411303 | 0.61832 | FALSE |
| FAM168B | 12782 | 0.82058 | 0.411886 | 0.619027 | FALSE |
| TCTN3 | 3034 | 0.820318 | 0.412035 | 0.619122 | FALSE |
| AMOTL2 | 1413 | 0.820164 | 0.412123 | 0.619194 | FALSE |
| ZNF678 | 11604 | 0.820048 | 0.412189 | 0.619215 | FALSE |
| CAMK2A | 2099 | 0.820001 | 0.412216 | 0.619215 | FALSE |
| AKT1S1 | 7269 | 0.819486 | 0.412509 | 0.619456 | FALSE |
| MRI1 | 11230 | 0.819296 | 0.412618 | 0.619461 | FALSE |
| CTSA | 614 | 0.819074 | 0.412744 | 0.619532 | FALSE |
| ZNF24 | 13221 | 0.818608 | 0.41301 | 0.619872 | FALSE |
| CCDC50 | 13592 | 0.818 | 0.413357 | 0.620117 | FALSE |
| CYFIP1 | 5601 | 0.817703 | 0.413527 | 0.62029 | FALSE |
| USP46 | 15575 | 0.816873 | 0.414001 | 0.620704 | FALSE |
| RNF121 | 12012 | 0.816739 | 0.414078 | 0.620759 | FALSE |
| WBP4 | 8362 | 0.816411 | 0.414265 | 0.620921 | FALSE |
| LRRC55 | 2274 | 0.816332 | 0.41431 | 0.620929 | FALSE |
| FAM20B | 7949 | 0.815953 | 0.414527 | 0.621049 | FALSE |
| KBTBD4 | 4773 | 0.815924 | 0.414544 | 0.621049 | FALSE |
| CDC37L1 | 14910 | 0.81585 | 0.414586 | 0.621049 | FALSE |
| UGGT1 | 15108 | 0.815792 | 0.414619 | 0.621049 | FALSE |
| TBCC | 6326 | 0.815776 | 0.414628 | 0.621049 | FALSE |
| LRRK1 | 14348 | 0.815637 | 0.414708 | 0.621049 | FALSE |
| NEURL4 | 12272 | 0.815443 | 0.414819 | 0.621073 | FALSE |
| LOC102723753 | 12743 | 0.815432 | 0.414825 | 0.621073 | FALSE |
| USP1 | 2462 | 0.814806 | 0.415183 | 0.621376 | FALSE |
| SAC3D1 | 8041 | 0.814409 | 0.415411 | 0.621448 | FALSE |
| DET1 | 58 | 0.814407 | 0.415412 | 0.621448 | FALSE |
| NUP160 | 9408 | 0.814304 | 0.415471 | 0.621477 | FALSE |
| TOM1L2 | 7467 | 0.813919 | 0.415691 | 0.621688 | FALSE |
| NAPRT | 7165 | 0.813559 | 0.415898 | 0.621788 | FALSE |
| SARM1 | 5948 | 0.813552 | 0.415902 | 0.621788 | FALSE |
| THAP12 | 471 | 0.812615 | 0.416439 | 0.622218 | FALSE |
| RNPS1 | 5338 | 0.812441 | 0.416539 | 0.6223 | FALSE |
| PFKP | 2814 | 0.811825 | 0.416892 | 0.622641 | FALSE |
| NTN4 | 8143 | 0.811766 | 0.416926 | 0.622641 | FALSE |
| MXD1 | 1591 | 0.811629 | 0.417005 | 0.622675 | FALSE |
| PSMA4 | 9471 | 0.811587 | 0.417029 | 0.622675 | FALSE |
| HSPA14 | 969 | 0.811142 | 0.417284 | 0.622883 | FALSE |
| MAIP1 | 2161 | 0.811136 | 0.417288 | 0.622883 | FALSE |
| DLX4 | 1222 | 0.810689 | 0.417544 | 0.623088 | FALSE |
| INTS5 | 1726 | 0.810519 | 0.417642 | 0.623173 | FALSE |
| C6orf15 | 14310 | 0.810409 | 0.417705 | 0.623173 | FALSE |
| RBMS2 | 7243 | 0.810382 | 0.417721 | 0.623173 | FALSE |
| BDH1 | 2816 | 0.810112 | 0.417876 | 0.623345 | FALSE |
| SEPSECS | 11127 | 0.80951 | 0.418222 | 0.623801 | FALSE |
| TCIM | 8568 | 0.809007 | 0.418511 | 0.624054 | FALSE |
| GPN3 | 1829 | 0.808605 | 0.418742 | 0.62434 | FALSE |
| USP15 | 4295 | 0.808442 | 0.418836 | 0.62442 | FALSE |
| MAP6D1 | 251 | 0.808341 | 0.418894 | 0.624421 | FALSE |
| FGF18 | 5760 | 0.808232 | 0.418957 | 0.624421 | FALSE |
| ZNF100 | 5150 | 0.807323 | 0.41948 | 0.624904 | FALSE |
| MYOT | 12458 | 0.805983 | 0.420253 | 0.625875 | FALSE |
| GCSH | 3167 | 0.805372 | 0.420605 | 0.626289 | FALSE |
| DEF6 | 2543 | 0.80492 | 0.420866 | 0.626609 | FALSE |
| GPX7 | 14688 | 0.80485 | 0.420906 | 0.62661 | FALSE |
| ZNF484 | 10849 | 0.804382 | 0.421176 | 0.626892 | FALSE |
| DMTN | 8987 | 0.804147 | 0.421312 | 0.626975 | FALSE |
| DDX56 | 2868 | 0.803384 | 0.421753 | 0.627511 | FALSE |
| SNPH | 1299 | 0.80274 | 0.422125 | 0.627757 | FALSE |
| SRP54 | 7828 | 0.802681 | 0.422159 | 0.627757 | FALSE |
| LINC01000 | 14483 | 0.802581 | 0.422217 | 0.627784 | FALSE |
| SLC2A1 | 12546 | 0.802499 | 0.422264 | 0.627794 | FALSE |
| ZFYVE27 | 4261 | 0.802369 | 0.42234 | 0.627847 | FALSE |
| PPP1R12B | 12537 | 0.80157 | 0.422802 | 0.627999 | FALSE |
| SAFB | 1267 | 0.801567 | 0.422803 | 0.627999 | FALSE |
| SCAP | 11676 | 0.801164 | 0.423037 | 0.628285 | FALSE |
| PSKH1 | 2112 | 0.801028 | 0.423115 | 0.628343 | FALSE |
| SSU72 | 6166 | 0.800735 | 0.423285 | 0.628535 | FALSE |
| NDUFAF2 | 4699 | 0.800418 | 0.423469 | 0.628697 | FALSE |
| IKBKB | 6203 | 0.800408 | 0.423474 | 0.628697 | FALSE |
| SMU1 | 8755 | 0.799558 | 0.423967 | 0.629189 | FALSE |
| C19orf66 | 15512 | 0.799472 | 0.424017 | 0.629203 | FALSE |
| CBWD5 | 6693 | 0.799333 | 0.424097 | 0.629263 | FALSE |
| PRKCSH | 14021 | 0.799244 | 0.424149 | 0.62928 | FALSE |
| KAT2A | 5094 | 0.799023 | 0.424277 | 0.629326 | FALSE |
| MNS1 | 992 | 0.798981 | 0.424301 | 0.629326 | FALSE |
| HHIP-AS1 | 3350 | 0.798821 | 0.424394 | 0.629326 | FALSE |
| OSTM1 | 13859 | 0.798774 | 0.424421 | 0.629326 | FALSE |
| PARP2 | 4469 | 0.798423 | 0.424625 | 0.629508 | FALSE |
| INPP1 | 10450 | 0.798059 | 0.424836 | 0.629762 | FALSE |
| NUP37 | 5447 | 0.797743 | 0.42502 | 0.629974 | FALSE |
| QRICH1 | 14749 | 0.796921 | 0.425497 | 0.630351 | FALSE |
| SLC25A25 | 10868 | 0.796597 | 0.425685 | 0.630422 | FALSE |
| ISX | 11706 | 0.796337 | 0.425836 | 0.630586 | FALSE |
| CAD | 14873 | 0.796055 | 0.426 | 0.630677 | FALSE |
| CSRNP3 | 630 | 0.796023 | 0.426019 | 0.630677 | FALSE |
| MRGBP | 2346 | 0.795762 | 0.42617 | 0.630836 | FALSE |
| MSL1 | 15179 | 0.795699 | 0.426207 | 0.630836 | FALSE |
| SMAD6 | 4397 | 0.795291 | 0.426444 | 0.631128 | FALSE |
| INTS12 | 8554 | 0.794664 | 0.426809 | 0.631428 | FALSE |
| CCDC154 | 4751 | 0.794159 | 0.427103 | 0.631803 | FALSE |
| ZNF674 | 5084 | 0.793668 | 0.427389 | 0.632022 | FALSE |
| HSCB | 10141 | 0.793589 | 0.427435 | 0.632022 | FALSE |
| MGP | 5303 | 0.793549 | 0.427458 | 0.632022 | FALSE |
| C2orf42 | 4532 | 0.793487 | 0.427494 | 0.632022 | FALSE |
| NUDT21 | 14827 | 0.793061 | 0.427742 | 0.632193 | FALSE |
| ZNF546 | 7895 | 0.793012 | 0.427771 | 0.632193 | FALSE |
| BCLAF1 | 11927 | 0.792755 | 0.427921 | 0.632281 | FALSE |
| ABCA11P | 5397 | 0.792695 | 0.427956 | 0.632281 | FALSE |
| ZNF221 | 193 | 0.792671 | 0.42797 | 0.632281 | FALSE |
| UBL7 | 1770 | 0.791053 | 0.428913 | 0.633328 | FALSE |
| RIC3 | 1636 | 0.790859 | 0.429026 | 0.63339 | FALSE |
| ESRRB | 205 | 0.790724 | 0.429105 | 0.633447 | FALSE |
| TSPAN14 | 5180 | 0.790436 | 0.429273 | 0.633582 | FALSE |
| CARD8 | 10346 | 0.790115 | 0.429461 | 0.633792 | FALSE |
| TLX2 | 9660 | 0.789852 | 0.429614 | 0.633959 | FALSE |
| PA2G4P4 | 14660 | 0.789516 | 0.42981 | 0.634134 | FALSE |
| LSM14B | 5527 | 0.789405 | 0.429875 | 0.634143 | FALSE |
| LHFPL2 | 12752 | 0.789272 | 0.429953 | 0.634159 | FALSE |
| HMGXB3 | 1200 | 0.789004 | 0.43011 | 0.634199 | FALSE |
| NLRP3 | 671 | 0.788971 | 0.430129 | 0.634199 | FALSE |
| STX19 | 6752 | 0.78897 | 0.43013 | 0.634199 | FALSE |
| CHST3 | 3313 | 0.788851 | 0.430199 | 0.634199 | FALSE |
| LOC728613 | 6859 | 0.788737 | 0.430266 | 0.634199 | FALSE |
| BZW1 | 3148 | 0.788708 | 0.430283 | 0.634199 | FALSE |
| ARL6IP5 | 8513 | 0.787511 | 0.430983 | 0.635019 | FALSE |
| SPOCD1 | 7825 | 0.786922 | 0.431328 | 0.635348 | FALSE |
| DCTN5 | 2159 | 0.786504 | 0.431572 | 0.635638 | FALSE |
| CXorf38 | 11150 | 0.786347 | 0.431664 | 0.635638 | FALSE |
| DIO3OS | 4565 | 0.786273 | 0.431708 | 0.635638 | FALSE |
| PTPN20 | 7542 | 0.786236 | 0.431729 | 0.635638 | FALSE |
| MAP4K2 | 1837 | 0.785986 | 0.431876 | 0.635656 | FALSE |
| KCNQ1OT1 | 10800 | 0.785939 | 0.431903 | 0.635656 | FALSE |
| CCDC47 | 15354 | 0.785464 | 0.432182 | 0.635946 | FALSE |
| CFAP46 | 6689 | 0.784929 | 0.432495 | 0.636152 | FALSE |
| ROPN1L | 11999 | 0.784879 | 0.432525 | 0.636152 | FALSE |
| LRRC46 | 9542 | 0.784628 | 0.432672 | 0.636189 | FALSE |
| PPP1R14B | 11111 | 0.784306 | 0.432861 | 0.636362 | FALSE |
| ACTR3 | 3375 | 0.784288 | 0.432871 | 0.636362 | FALSE |
| LRRCC1 | 10342 | 0.783984 | 0.433049 | 0.636505 | FALSE |
| SURF6 | 446 | 0.783604 | 0.433273 | 0.636773 | FALSE |
| SUPV3L1 | 10423 | 0.783515 | 0.433325 | 0.636789 | FALSE |
| GOLGA7 | 4054 | 0.783171 | 0.433527 | 0.636951 | FALSE |
| SLC25A5-AS1 | 3000 | 0.78305 | 0.433598 | 0.636951 | FALSE |
| TMEM259 | 6635 | 0.782926 | 0.433671 | 0.636998 | FALSE |
| GCHFR | 6674 | 0.78158 | 0.434461 | 0.63786 | FALSE |
| ATN1 | 359 | 0.78126 | 0.43465 | 0.63791 | FALSE |
| PARD6G | 417 | 0.781245 | 0.434658 | 0.63791 | FALSE |
| PICALM | 15149 | 0.780763 | 0.434942 | 0.638206 | FALSE |
| SND1-IT1 | 7609 | 0.78016 | 0.435297 | 0.63862 | FALSE |
| IFT20 | 10396 | 0.780145 | 0.435306 | 0.63862 | FALSE |
| C9orf129 | 3065 | 0.779654 | 0.435595 | 0.638804 | FALSE |
| GRM7 | 2284 | 0.779424 | 0.43573 | 0.638841 | FALSE |
| PTPRK | 9596 | 0.778789 | 0.436104 | 0.639071 | FALSE |
| ABCG2 | 8981 | 0.778659 | 0.436181 | 0.639124 | FALSE |
| ATG7 | 15277 | 0.77839 | 0.436339 | 0.639296 | FALSE |
| SMURF1 | 8280 | 0.778195 | 0.436454 | 0.639405 | FALSE |
| ARHGAP29 | 6878 | 0.77749 | 0.43687 | 0.639894 | FALSE |
| NUDT9P1 | 13853 | 0.777217 | 0.437031 | 0.640004 | FALSE |
| DUX4 | 10881 | 0.777154 | 0.437068 | 0.640004 | FALSE |
| PRR15 | 10024 | 0.776937 | 0.437196 | 0.640072 | FALSE |
| FBXO38 | 9472 | 0.77646 | 0.437477 | 0.640304 | FALSE |
| GSTM2P1 | 10926 | 0.77639 | 0.437519 | 0.640304 | FALSE |
| LGI1 | 3678 | 0.776318 | 0.437561 | 0.640307 | FALSE |
| DAG1 | 1534 | 0.77541 | 0.438097 | 0.640851 | FALSE |
| SF3B1 | 3257 | 0.774754 | 0.438485 | 0.641186 | FALSE |
| ZNF133 | 6634 | 0.774614 | 0.438568 | 0.641186 | FALSE |
| CKMT1A | 1657 | 0.774606 | 0.438573 | 0.641186 | FALSE |
| ZNF71 | 12033 | 0.77427 | 0.438771 | 0.641417 | FALSE |
| NAPG | 9029 | 0.773827 | 0.439033 | 0.641642 | FALSE |
| DMXL2 | 6324 | 0.773801 | 0.439048 | 0.641642 | FALSE |
| ERI2 | 418 | 0.772968 | 0.439541 | 0.642122 | FALSE |
| DTX3 | 827 | 0.772695 | 0.439703 | 0.642298 | FALSE |
| CCDC134 | 6748 | 0.77258 | 0.439771 | 0.642338 | FALSE |
| TARBP2 | 8038 | 0.772473 | 0.439834 | 0.64237 | FALSE |
| PDCD2 | 9493 | 0.772036 | 0.440093 | 0.642688 | FALSE |
| ALDOC | 1743 | 0.771273 | 0.440545 | 0.643228 | FALSE |
| ANGPTL2 | 602 | 0.770884 | 0.440776 | 0.643464 | FALSE |
| HIST1H1C | 5083 | 0.770841 | 0.440801 | 0.643464 | FALSE |
| AUNIP | 2973 | 0.770722 | 0.440872 | 0.643464 | FALSE |
| RNF216P1 | 11025 | 0.770371 | 0.44108 | 0.643699 | FALSE |
| YWHAZ | 15129 | 0.769339 | 0.441692 | 0.644409 | FALSE |
| PDGFRB | 5803 | 0.76906 | 0.441858 | 0.644409 | FALSE |
| LOC101928433 | 15508 | 0.768898 | 0.441954 | 0.644409 | FALSE |
| CLIP2 | 5974 | 0.768867 | 0.441972 | 0.644409 | FALSE |
| HS6ST3 | 12840 | 0.768767 | 0.442032 | 0.644435 | FALSE |
| TBX3 | 15573 | 0.768618 | 0.44212 | 0.644504 | FALSE |
| EGLN3 | 3787 | 0.768272 | 0.442326 | 0.644684 | FALSE |
| OR7E5P | 7299 | 0.768076 | 0.442442 | 0.644793 | FALSE |
| MAFG-AS1 | 12134 | 0.767746 | 0.442638 | 0.644944 | FALSE |
| HERC2 | 7314 | 0.767445 | 0.442817 | 0.645013 | FALSE |
| RBM33 | 3278 | 0.767412 | 0.442837 | 0.645013 | FALSE |
| NR2F1 | 12832 | 0.766971 | 0.443099 | 0.645269 | FALSE |
| NDUFS2 | 5462 | 0.766465 | 0.4434 | 0.645543 | FALSE |
| RINL | 4034 | 0.766308 | 0.443493 | 0.645543 | FALSE |
| CTDSP2 | 2271 | 0.765938 | 0.443713 | 0.645683 | FALSE |
| FGFR1OP | 7880 | 0.765779 | 0.443808 | 0.64576 | FALSE |
| EFNA1 | 7263 | 0.765683 | 0.443865 | 0.645783 | FALSE |
| OLFM4 | 7568 | 0.765331 | 0.444074 | 0.645908 | FALSE |
| ATP11C | 8092 | 0.765109 | 0.444207 | 0.64604 | FALSE |
| SETD4 | 2179 | 0.764868 | 0.44435 | 0.646188 | FALSE |
| ASAP3 | 8544 | 0.764349 | 0.444659 | 0.64651 | FALSE |
| ANK2 | 3460 | 0.764289 | 0.444695 | 0.64651 | FALSE |
| SLC24A4 | 12923 | 0.764166 | 0.444768 | 0.646556 | FALSE |
| WHAMM | 13427 | 0.762991 | 0.445469 | 0.647093 | FALSE |
| LOC728554 | 982 | 0.762819 | 0.445571 | 0.647149 | FALSE |
| CCDC96 | 5932 | 0.762542 | 0.445737 | 0.647231 | FALSE |
| SPTLC1 | 1718 | 0.762415 | 0.445812 | 0.647231 | FALSE |
| ALDH1L2 | 12104 | 0.761404 | 0.446416 | 0.647867 | FALSE |
| VAMP3 | 8171 | 0.761215 | 0.446529 | 0.647962 | FALSE |
| XPNPEP1 | 9527 | 0.760482 | 0.446967 | 0.648365 | FALSE |
| RAB42 | 12415 | 0.760131 | 0.447176 | 0.648609 | FALSE |
| ACR | 14340 | 0.759897 | 0.447316 | 0.648619 | FALSE |
| CSNK1A1L | 9543 | 0.759772 | 0.447391 | 0.648619 | FALSE |
| ZNF716 | 350 | 0.759337 | 0.447651 | 0.648876 | FALSE |
| ZYG11B | 12246 | 0.758447 | 0.448183 | 0.649467 | FALSE |
| PBDC1 | 10061 | 0.758221 | 0.448319 | 0.649552 | FALSE |
| C15orf61 | 14583 | 0.757921 | 0.448498 | 0.649699 | FALSE |
| PAK2 | 11404 | 0.757264 | 0.448892 | 0.650132 | FALSE |
| LRRTM4 | 5082 | 0.756737 | 0.449207 | 0.650346 | FALSE |
| MFSD14B | 9837 | 0.756434 | 0.449389 | 0.650491 | FALSE |
| EXOSC10 | 8530 | 0.755858 | 0.449734 | 0.65087 | FALSE |
| METTL2B | 7961 | 0.755314 | 0.450061 | 0.651047 | FALSE |
| USP25 | 6849 | 0.754963 | 0.450271 | 0.651245 | FALSE |
| ARMCX3 | 15060 | 0.75494 | 0.450285 | 0.651245 | FALSE |
| ARHGEF10 | 5470 | 0.7547 | 0.450429 | 0.651333 | FALSE |
| RIN1 | 14246 | 0.754418 | 0.450598 | 0.651517 | FALSE |
| FSCN1 | 566 | 0.754121 | 0.450777 | 0.651594 | FALSE |
| CHEK2 | 10045 | 0.753284 | 0.451279 | 0.65193 | FALSE |
| RNF26 | 6587 | 0.753194 | 0.451333 | 0.65193 | FALSE |
| TTBK1 | 10991 | 0.75306 | 0.451414 | 0.651957 | FALSE |
| NPM2 | 4955 | 0.752939 | 0.451487 | 0.651957 | FALSE |
| SRCIN1 | 13700 | 0.752548 | 0.451722 | 0.652116 | FALSE |
| SLC5A3 | 12633 | 0.75227 | 0.451889 | 0.652237 | FALSE |
| CEBPZ | 12086 | 0.751936 | 0.45209 | 0.652466 | FALSE |
| SNAPC2 | 3548 | 0.751332 | 0.452453 | 0.652841 | FALSE |
| IP6K3 | 1970 | 0.75116 | 0.452556 | 0.652899 | FALSE |
| TRA2A | 14672 | 0.750708 | 0.452828 | 0.653111 | FALSE |
| MED30 | 11854 | 0.750506 | 0.45295 | 0.653166 | FALSE |
| UGCG | 1943 | 0.749609 | 0.45349 | 0.653704 | FALSE |
| ZDHHC21 | 10539 | 0.749608 | 0.453491 | 0.653704 | FALSE |
| SH3YL1 | 8831 | 0.749155 | 0.453764 | 0.653979 | FALSE |
| NHS | 4896 | 0.749153 | 0.453765 | 0.653979 | FALSE |
| PAIP1 | 10967 | 0.748956 | 0.453884 | 0.653998 | FALSE |
| TNPO2 | 13268 | 0.748875 | 0.453933 | 0.653998 | FALSE |
| MAGT1 | 4741 | 0.748869 | 0.453936 | 0.653998 | FALSE |
| TMEM45B | 3784 | 0.748784 | 0.453987 | 0.653998 | FALSE |
| TBRG1 | 8973 | 0.748599 | 0.454099 | 0.654042 | FALSE |
| ABCE1 | 1223 | 0.748367 | 0.454239 | 0.654119 | FALSE |
| KARS | 2545 | 0.748112 | 0.454393 | 0.654166 | FALSE |
| SPOCK2 | 2498 | 0.748108 | 0.454395 | 0.654166 | FALSE |
| SPCS2 | 5926 | 0.747906 | 0.454517 | 0.654166 | FALSE |
| HIGD2B | 3845 | 0.74788 | 0.454533 | 0.654166 | FALSE |
| C7orf61 | 3152 | 0.747846 | 0.454553 | 0.654166 | FALSE |
| ANKAR | 7471 | 0.747602 | 0.4547 | 0.654241 | FALSE |
| MAML3 | 5597 | 0.747207 | 0.454939 | 0.654524 | FALSE |
| CCNL2 | 7777 | 0.746723 | 0.455231 | 0.654824 | FALSE |
| THRAP3 | 6872 | 0.746441 | 0.455401 | 0.654971 | FALSE |
| HAGHL | 1799 | 0.746003 | 0.455666 | 0.655208 | FALSE |
| STK11 | 5620 | 0.745555 | 0.455936 | 0.655537 | FALSE |
| NDRG1 | 850 | 0.745112 | 0.456204 | 0.655861 | FALSE |
| STX17 | 538 | 0.744794 | 0.456396 | 0.655897 | FALSE |
| LINC00507 | 5968 | 0.744619 | 0.456502 | 0.655988 | FALSE |
| GBP2 | 13672 | 0.744169 | 0.456774 | 0.656203 | FALSE |
| MGA | 131 | 0.744107 | 0.456812 | 0.656203 | FALSE |
| ZNF107 | 2413 | 0.744025 | 0.456861 | 0.656203 | FALSE |
| OCIAD2 | 2162 | 0.743242 | 0.457335 | 0.656645 | FALSE |
| IFITM4P | 12334 | 0.743164 | 0.457382 | 0.656645 | FALSE |
| ORAI3 | 14954 | 0.742749 | 0.457634 | 0.65683 | FALSE |
| ADARB2 | 13261 | 0.74249 | 0.457791 | 0.656934 | FALSE |
| RHBDL2 | 5587 | 0.74166 | 0.458293 | 0.657422 | FALSE |
| PRKY | 1194 | 0.741644 | 0.458303 | 0.657422 | FALSE |
| GXYLT1 | 15102 | 0.741331 | 0.458493 | 0.657459 | FALSE |
| SCTR | 5979 | 0.741257 | 0.458538 | 0.657463 | FALSE |
| FGF14-AS2 | 14362 | 0.741009 | 0.458688 | 0.657618 | FALSE |
| ATXN7L3B | 4913 | 0.740527 | 0.45898 | 0.657855 | FALSE |
| VEGFC | 9744 | 0.7405 | 0.458997 | 0.657855 | FALSE |
| ALS2CR12 | 4137 | 0.740273 | 0.459134 | 0.657896 | FALSE |
| ANKRD11 | 5400 | 0.739014 | 0.459898 | 0.65887 | FALSE |
| NLGN4X | 10552 | 0.738679 | 0.460102 | 0.658977 | FALSE |
| PSMD13 | 7395 | 0.738624 | 0.460135 | 0.658977 | FALSE |
| H1FNT | 6496 | 0.738518 | 0.4602 | 0.658977 | FALSE |
| LRRC23 | 12968 | 0.738476 | 0.460225 | 0.658977 | FALSE |
| CPEB1 | 14997 | 0.738182 | 0.460404 | 0.659111 | FALSE |
| PCDHA1 | 4304 | 0.737968 | 0.460534 | 0.659177 | FALSE |
| VSTM1 | 14708 | 0.737775 | 0.460651 | 0.659263 | FALSE |
| ZNF48 | 9041 | 0.737698 | 0.460698 | 0.659263 | FALSE |
| CD47 | 2755 | 0.737652 | 0.460726 | 0.659263 | FALSE |
| SLC25A52 | 1286 | 0.737591 | 0.460763 | 0.659263 | FALSE |
| COPS7B | 15564 | 0.737366 | 0.4609 | 0.6593 | FALSE |
| EID3 | 608 | 0.737263 | 0.460962 | 0.659307 | FALSE |
| AGPAT3 | 13722 | 0.736691 | 0.46131 | 0.659684 | FALSE |
| ZNF12 | 1594 | 0.736338 | 0.461525 | 0.659805 | FALSE |
| FRK | 5874 | 0.73631 | 0.461542 | 0.659805 | FALSE |
| TMC6 | 6699 | 0.736263 | 0.461571 | 0.659805 | FALSE |
| ZNF680 | 3499 | 0.736205 | 0.461606 | 0.659805 | FALSE |
| PNMA3 | 15521 | 0.735921 | 0.461779 | 0.659992 | FALSE |
| PTPRH | 6200 | 0.734713 | 0.462514 | 0.66079 | FALSE |
| VPS33A | 14477 | 0.734456 | 0.462671 | 0.66079 | FALSE |
| CD163L1 | 1844 | 0.734448 | 0.462676 | 0.66079 | FALSE |
| ARID4A | 8294 | 0.734057 | 0.462914 | 0.66107 | FALSE |
| UBXN10 | 15455 | 0.7331 | 0.463497 | 0.661619 | FALSE |
| PADI2 | 5763 | 0.73308 | 0.46351 | 0.661619 | FALSE |
| ADA | 12629 | 0.732961 | 0.463582 | 0.661662 | FALSE |
| FKTN | 14358 | 0.732852 | 0.463649 | 0.661672 | FALSE |
| RFC4 | 4269 | 0.73281 | 0.463674 | 0.661672 | FALSE |
| SMARCC2 | 7323 | 0.732358 | 0.46395 | 0.661885 | FALSE |
| TTLL7 | 13441 | 0.732028 | 0.464151 | 0.661991 | FALSE |
| LACTB2 | 11638 | 0.731426 | 0.464519 | 0.662334 | FALSE |
| RBBP9 | 10536 | 0.731426 | 0.464519 | 0.662334 | FALSE |
| SBNO1 | 12073 | 0.731297 | 0.464598 | 0.662385 | FALSE |
| AFMID | 1077 | 0.730757 | 0.464928 | 0.662771 | FALSE |
| SOX7 | 8678 | 0.730715 | 0.464953 | 0.662771 | FALSE |
| CC2D1B | 1288 | 0.730199 | 0.465269 | 0.663098 | FALSE |
| FRS2 | 10111 | 0.730088 | 0.465336 | 0.663098 | FALSE |
| CTPS1 | 6893 | 0.730063 | 0.465352 | 0.663098 | FALSE |
| LOC100130705 | 10942 | 0.729591 | 0.46564 | 0.663333 | FALSE |
| GAS6 | 3804 | 0.729584 | 0.465645 | 0.663333 | FALSE |
| CASP9 | 7348 | 0.729286 | 0.465827 | 0.663533 | FALSE |
| LLGL2 | 11227 | 0.729068 | 0.46596 | 0.663662 | FALSE |
| ZKSCAN1 | 13336 | 0.728768 | 0.466144 | 0.663815 | FALSE |
| TMEM184C | 634 | 0.727869 | 0.466694 | 0.664268 | FALSE |
| CHMP4B | 6103 | 0.727466 | 0.466941 | 0.664383 | FALSE |
| KLF15 | 4970 | 0.727349 | 0.467012 | 0.664383 | FALSE |
| SLC26A1 | 624 | 0.727246 | 0.467075 | 0.664383 | FALSE |
| PPP2R5E | 1272 | 0.727038 | 0.467203 | 0.664383 | FALSE |
| KCNJ10 | 339 | 0.726773 | 0.467365 | 0.664383 | FALSE |
| LSS | 6643 | 0.726723 | 0.467396 | 0.664383 | FALSE |
| SUMO1 | 13932 | 0.726717 | 0.467399 | 0.664383 | FALSE |
| C1QTNF4 | 531 | 0.726418 | 0.467583 | 0.664383 | FALSE |
| OTX1 | 5275 | 0.726366 | 0.467614 | 0.664383 | FALSE |
| UBE2V2 | 7556 | 0.726298 | 0.467656 | 0.664383 | FALSE |
| LCOR | 13435 | 0.726242 | 0.46769 | 0.664383 | FALSE |
| DUS4L | 4612 | 0.726228 | 0.467699 | 0.664383 | FALSE |
| NUTM2B-AS1 | 11189 | 0.725903 | 0.467898 | 0.664578 | FALSE |
| TACC3 | 10893 | 0.725818 | 0.46795 | 0.664578 | FALSE |
| PPP2R2B | 6313 | 0.725727 | 0.468006 | 0.664578 | FALSE |
| FAM95C | 6066 | 0.725541 | 0.46812 | 0.66468 | FALSE |
| DAPK1 | 1182 | 0.724884 | 0.468523 | 0.66501 | FALSE |
| WNK3 | 11896 | 0.724648 | 0.468668 | 0.665137 | FALSE |
| FBXO11 | 1010 | 0.7246 | 0.468697 | 0.665137 | FALSE |
| CYP4V2 | 14516 | 0.724281 | 0.468893 | 0.665308 | FALSE |
| ZNF451 | 3053 | 0.724265 | 0.468903 | 0.665308 | FALSE |
| METTL13 | 9807 | 0.724125 | 0.468989 | 0.665369 | FALSE |
| YME1L1 | 9580 | 0.723303 | 0.469494 | 0.665783 | FALSE |
| PCDHB18P | 11224 | 0.723162 | 0.46958 | 0.66579 | FALSE |
| LRRC4B | 6502 | 0.723131 | 0.469599 | 0.66579 | FALSE |
| ZNF558 | 294 | 0.723004 | 0.469677 | 0.66579 | FALSE |
| EPHA4 | 14977 | 0.722887 | 0.469749 | 0.66579 | FALSE |
| LOC646626 | 10371 | 0.72288 | 0.469754 | 0.66579 | FALSE |
| NOTCH4 | 6238 | 0.722064 | 0.470255 | 0.666379 | FALSE |
| MED6 | 15306 | 0.721656 | 0.470506 | 0.666582 | FALSE |
| MAP10 | 12071 | 0.721283 | 0.470735 | 0.666758 | FALSE |
| SLC31A2 | 3098 | 0.720994 | 0.470913 | 0.666889 | FALSE |
| ITGA6 | 14598 | 0.720664 | 0.471116 | 0.667116 | FALSE |
| GCN1 | 6111 | 0.720139 | 0.471439 | 0.667453 | FALSE |
| KHSRP | 13106 | 0.72002 | 0.471513 | 0.667496 | FALSE |
| KIF11 | 4400 | 0.719537 | 0.47181 | 0.667857 | FALSE |
| TMPRSS2 | 14892 | 0.718791 | 0.47227 | 0.668428 | FALSE |
| CALN1 | 8584 | 0.718743 | 0.472299 | 0.668428 | FALSE |
| KIAA1522 | 14917 | 0.718607 | 0.472383 | 0.668446 | FALSE |
| ZNF184 | 10794 | 0.718426 | 0.472495 | 0.668462 | FALSE |
| RANBP3 | 4495 | 0.718023 | 0.472743 | 0.668753 | FALSE |
| PAIP2B | 4047 | 0.717825 | 0.472865 | 0.668836 | FALSE |
| CHCHD10 | 13684 | 0.71779 | 0.472887 | 0.668836 | FALSE |
| CREB3L4 | 3717 | 0.717379 | 0.47314 | 0.668947 | FALSE |
| TMEM35B | 4414 | 0.717343 | 0.473162 | 0.668947 | FALSE |
| ATG4C | 1184 | 0.717315 | 0.47318 | 0.668947 | FALSE |
| C20orf204 | 6188 | 0.716711 | 0.473552 | 0.669218 | FALSE |
| SKP2 | 713 | 0.716564 | 0.473643 | 0.669223 | FALSE |
| NET1 | 9812 | 0.716189 | 0.473875 | 0.669385 | FALSE |
| IER3 | 4611 | 0.715955 | 0.474019 | 0.669528 | FALSE |
| TRNT1 | 1381 | 0.715805 | 0.474112 | 0.669599 | FALSE |
| TOB1 | 10072 | 0.715607 | 0.474234 | 0.669618 | FALSE |
| RFC1 | 15414 | 0.715601 | 0.474238 | 0.669618 | FALSE |
| PSMA3-AS1 | 7759 | 0.715052 | 0.474577 | 0.669988 | FALSE |
| ZNF876P | 3389 | 0.714916 | 0.474661 | 0.670011 | FALSE |
| TTC7A | 2196 | 0.714742 | 0.474768 | 0.670103 | FALSE |
| TIPRL | 15199 | 0.714611 | 0.474849 | 0.670156 | FALSE |
| WDR45B | 3253 | 0.714096 | 0.475168 | 0.670377 | FALSE |
| ZEB2 | 5989 | 0.714024 | 0.475212 | 0.670377 | FALSE |
| IMPDH2 | 5661 | 0.714011 | 0.47522 | 0.670377 | FALSE |
| AHCTF1 | 9683 | 0.71349 | 0.475543 | 0.67065 | FALSE |
| CENPT | 2109 | 0.713296 | 0.475663 | 0.670759 | FALSE |
| RAB9BP1 | 6566 | 0.71315 | 0.475753 | 0.670765 | FALSE |
| PLEKHG5 | 14048 | 0.712415 | 0.476208 | 0.671204 | FALSE |
| ZFYVE16 | 14272 | 0.711658 | 0.476677 | 0.671765 | FALSE |
| LRRC8A | 3289 | 0.711011 | 0.477077 | 0.672148 | FALSE |
| NSUN5P2 | 7786 | 0.71018 | 0.477593 | 0.672631 | FALSE |
| DIP2C | 11136 | 0.709384 | 0.478086 | 0.672943 | FALSE |
| USP10 | 9561 | 0.70921 | 0.478194 | 0.672993 | FALSE |
| DSTN | 10128 | 0.708814 | 0.47844 | 0.673223 | FALSE |
| NPIPB11 | 10260 | 0.708808 | 0.478444 | 0.673223 | FALSE |
| BAZ1B | 11403 | 0.708654 | 0.478539 | 0.673241 | FALSE |
| ADCY10P1 | 3269 | 0.708649 | 0.478542 | 0.673241 | FALSE |
| TRAF3 | 2246 | 0.708169 | 0.47884 | 0.673599 | FALSE |
| LINC00933 | 10211 | 0.706799 | 0.479691 | 0.674486 | FALSE |
| MICAL1 | 1569 | 0.706704 | 0.47975 | 0.674486 | FALSE |
| SLX4 | 9948 | 0.706669 | 0.479772 | 0.674486 | FALSE |
| SAMD8 | 3830 | 0.706127 | 0.480109 | 0.674717 | FALSE |
| PKP4 | 7068 | 0.705403 | 0.480559 | 0.675228 | FALSE |
| TIE1 | 7113 | 0.705147 | 0.480719 | 0.67533 | FALSE |
| HMGB3P1 | 6128 | 0.704651 | 0.481027 | 0.675582 | FALSE |
| LSM8 | 9065 | 0.703869 | 0.481514 | 0.676144 | FALSE |
| IFITM2 | 12390 | 0.702388 | 0.482437 | 0.677258 | FALSE |
| ZBTB7B | 13817 | 0.70117 | 0.483197 | 0.678263 | FALSE |
| COL6A2 | 14482 | 0.701013 | 0.483295 | 0.67834 | FALSE |
| SIPA1L1 | 5957 | 0.700655 | 0.483518 | 0.678526 | FALSE |
| U2SURP | 1876 | 0.700345 | 0.483712 | 0.678661 | FALSE |
| RYK | 10777 | 0.700299 | 0.483741 | 0.678661 | FALSE |
| LOC401357 | 3321 | 0.698964 | 0.484575 | 0.679526 | FALSE |
| AKT1 | 8506 | 0.698045 | 0.485149 | 0.680026 | FALSE |
| CYTH2 | 6646 | 0.697041 | 0.485777 | 0.680613 | FALSE |
| PITHD1 | 6157 | 0.696914 | 0.485857 | 0.680652 | FALSE |
| CDC5L | 1532 | 0.696736 | 0.485968 | 0.680686 | FALSE |
| MMP15 | 10011 | 0.695133 | 0.486972 | 0.681787 | FALSE |
| SOCS6 | 13995 | 0.694765 | 0.487203 | 0.681874 | FALSE |
| RASSF2 | 7755 | 0.694755 | 0.487209 | 0.681874 | FALSE |
| RFPL3S | 12270 | 0.694611 | 0.487299 | 0.68194 | FALSE |
| POLR3G | 3503 | 0.694093 | 0.487624 | 0.682272 | FALSE |
| IFT172 | 5499 | 0.693912 | 0.487737 | 0.68229 | FALSE |
| ZNF850 | 8120 | 0.69388 | 0.487757 | 0.68229 | FALSE |
| BCORL1 | 4553 | 0.693781 | 0.48782 | 0.68229 | FALSE |
| KPNA1 | 11742 | 0.693724 | 0.487855 | 0.68229 | FALSE |
| NMNAT1 | 12398 | 0.69314 | 0.488222 | 0.68262 | FALSE |
| NSUN2 | 2423 | 0.693041 | 0.488284 | 0.682627 | FALSE |
| CCDC82 | 6213 | 0.692505 | 0.48862 | 0.682994 | FALSE |
| ZNF14 | 11700 | 0.691992 | 0.488942 | 0.683319 | FALSE |
| POLD1 | 9336 | 0.691947 | 0.488971 | 0.683319 | FALSE |
| CD55 | 5068 | 0.691925 | 0.488984 | 0.683319 | FALSE |
| SPOUT1 | 6724 | 0.69084 | 0.489666 | 0.684051 | FALSE |
| WDR81 | 4289 | 0.690506 | 0.489876 | 0.684137 | FALSE |
| MEX3C | 15171 | 0.690172 | 0.490086 | 0.684369 | FALSE |
| ZC3H11A | 9838 | 0.688472 | 0.491156 | 0.685496 | FALSE |
| TM6SF2 | 9927 | 0.688321 | 0.491251 | 0.685506 | FALSE |
| OLIG2 | 15442 | 0.688187 | 0.491335 | 0.685562 | FALSE |
| FBXO27 | 2078 | 0.687458 | 0.491794 | 0.686132 | FALSE |
| MMACHC | 7831 | 0.687387 | 0.491839 | 0.686132 | FALSE |
| PRKAR1A | 9339 | 0.68726 | 0.491919 | 0.686132 | FALSE |
| ZNF43 | 2000 | 0.686324 | 0.492509 | 0.686737 | FALSE |
| ZNF175 | 13607 | 0.686293 | 0.492528 | 0.686737 | FALSE |
| R3HDM2 | 10814 | 0.685944 | 0.492748 | 0.686921 | FALSE |
| CANX | 1341 | 0.685745 | 0.492874 | 0.687035 | FALSE |
| TRIM4 | 1567 | 0.68523 | 0.493199 | 0.687417 | FALSE |
| PALM2 | 14781 | 0.685168 | 0.493238 | 0.687417 | FALSE |
| FUNDC2P2 | 6316 | 0.684761 | 0.493495 | 0.687594 | FALSE |
| CFAP73 | 15377 | 0.684637 | 0.493573 | 0.687635 | FALSE |
| ATXN2 | 15309 | 0.684321 | 0.493772 | 0.687736 | FALSE |
| AURKC | 2929 | 0.682894 | 0.494674 | 0.688684 | FALSE |
| UBA6 | 13564 | 0.682471 | 0.494941 | 0.688934 | FALSE |
| GMEB1 | 14744 | 0.682382 | 0.494997 | 0.688951 | FALSE |
| AIDA | 15373 | 0.681556 | 0.49552 | 0.689556 | FALSE |
| TFB1M | 6871 | 0.681432 | 0.495598 | 0.689603 | FALSE |
| SNX18 | 14085 | 0.680856 | 0.495963 | 0.689987 | FALSE |
| ZNF609 | 6966 | 0.6805 | 0.496188 | 0.690178 | FALSE |
| NCLN | 696 | 0.680203 | 0.496376 | 0.690316 | FALSE |
| SLC44A1 | 4094 | 0.679936 | 0.496545 | 0.690429 | FALSE |
| MAN2C1 | 12263 | 0.67951 | 0.496815 | 0.690681 | FALSE |
| MAP4K5 | 7364 | 0.679033 | 0.497117 | 0.690973 | FALSE |
| KRR1 | 4923 | 0.678728 | 0.49731 | 0.691085 | FALSE |
| DTNA | 6676 | 0.678702 | 0.497327 | 0.691085 | FALSE |
| CDKL2 | 7908 | 0.678414 | 0.497509 | 0.691206 | FALSE |
| PHACTR2 | 9323 | 0.678352 | 0.497549 | 0.691206 | FALSE |
| OPN4 | 6506 | 0.678286 | 0.49759 | 0.691206 | FALSE |
| VWA5B2 | 12128 | 0.678213 | 0.497637 | 0.691209 | FALSE |
| GPN1 | 14401 | 0.677669 | 0.497982 | 0.691626 | FALSE |
| GPR63 | 5257 | 0.677127 | 0.498325 | 0.691878 | FALSE |
| RNF103 | 15236 | 0.676816 | 0.498523 | 0.691878 | FALSE |
| EIF4E2 | 15463 | 0.676763 | 0.498556 | 0.691878 | FALSE |
| TPR | 4608 | 0.676756 | 0.498561 | 0.691878 | FALSE |
| H3.Y | 29 | 0.676484 | 0.498733 | 0.692022 | FALSE |
| CKMT2 | 2629 | 0.676383 | 0.498798 | 0.692022 | FALSE |
| BAZ2B | 6121 | 0.675984 | 0.499051 | 0.69225 | FALSE |
| TNFRSF10A | 12727 | 0.675633 | 0.499274 | 0.692498 | FALSE |
| TMEM171 | 11253 | 0.675133 | 0.499591 | 0.692693 | FALSE |
| PPFIA2 | 981 | 0.67498 | 0.499688 | 0.692766 | FALSE |
| PLPP7 | 15406 | 0.674808 | 0.499798 | 0.692843 | FALSE |
| OR5L2 | 10817 | 0.674523 | 0.499979 | 0.692925 | FALSE |
| VMA21 | 3534 | 0.674521 | 0.49998 | 0.692925 | FALSE |
| ZNF702P | 10930 | 0.673963 | 0.500335 | 0.693319 | FALSE |
| SIRT7 | 6791 | 0.673934 | 0.500353 | 0.693319 | FALSE |
| FAM27E2 | 14481 | 0.673776 | 0.500454 | 0.69337 | FALSE |
| TULP4 | 7575 | 0.673736 | 0.500479 | 0.69337 | FALSE |
| QPCTL | 8293 | 0.673398 | 0.500694 | 0.69359 | FALSE |
| BBX | 8295 | 0.673347 | 0.500727 | 0.69359 | FALSE |
| MCAM | 10558 | 0.673237 | 0.500797 | 0.693626 | FALSE |
| SLK | 10078 | 0.673151 | 0.500851 | 0.693629 | FALSE |
| CSTF2T | 8640 | 0.67276 | 0.5011 | 0.693682 | FALSE |
| FAM19A2 | 14029 | 0.672682 | 0.50115 | 0.693684 | FALSE |
| CLEC2B | 1354 | 0.672206 | 0.501453 | 0.693919 | FALSE |
| GNG7 | 235 | 0.67204 | 0.501558 | 0.693944 | FALSE |
| MGAT5 | 237 | 0.671886 | 0.501656 | 0.693944 | FALSE |
| PROSER3 | 3517 | 0.671744 | 0.501747 | 0.693944 | FALSE |
| LRRC70 | 9215 | 0.671083 | 0.502168 | 0.694233 | FALSE |
| SPRED2 | 6567 | 0.670991 | 0.502226 | 0.694253 | FALSE |
| CDH20 | 9938 | 0.670807 | 0.502343 | 0.694353 | FALSE |
| IER3IP1 | 11691 | 0.670221 | 0.502717 | 0.694685 | FALSE |
| TMEM232 | 12182 | 0.669431 | 0.503221 | 0.695258 | FALSE |
| NUP188 | 14883 | 0.668822 | 0.503609 | 0.695383 | FALSE |
| ADCY6 | 9548 | 0.668781 | 0.503635 | 0.695383 | FALSE |
| LBR | 3414 | 0.668728 | 0.503669 | 0.695383 | FALSE |
| TET2 | 1604 | 0.668579 | 0.503764 | 0.695383 | FALSE |
| GOLGA7B | 11257 | 0.667754 | 0.504291 | 0.695859 | FALSE |
| MAPK14 | 11933 | 0.667696 | 0.504328 | 0.695859 | FALSE |
| GPATCH2 | 3628 | 0.667497 | 0.504455 | 0.695918 | FALSE |
| SCO1 | 2538 | 0.667347 | 0.504551 | 0.695989 | FALSE |
| TYW3 | 6077 | 0.665964 | 0.505434 | 0.696767 | FALSE |
| MPPE1 | 7668 | 0.665884 | 0.505485 | 0.696767 | FALSE |
| BOD1L1 | 8264 | 0.665836 | 0.505516 | 0.696767 | FALSE |
| NFRKB | 14190 | 0.665736 | 0.50558 | 0.696785 | FALSE |
| ZNF208 | 5012 | 0.665676 | 0.505618 | 0.696785 | FALSE |
| DDX19A | 833 | 0.665364 | 0.505818 | 0.696999 | FALSE |
| SERBP1 | 10932 | 0.665069 | 0.506006 | 0.697013 | FALSE |
| TPTE2P1 | 2788 | 0.664716 | 0.506232 | 0.697262 | FALSE |
| RBM19 | 8598 | 0.664486 | 0.506379 | 0.697404 | FALSE |
| TRAF1 | 12339 | 0.664198 | 0.506564 | 0.697596 | FALSE |
| HNRNPUL1 | 11373 | 0.663341 | 0.507112 | 0.697982 | FALSE |
| ZXDB | 13588 | 0.663084 | 0.507277 | 0.698086 | FALSE |
| EXPH5 | 13540 | 0.662472 | 0.507669 | 0.698441 | FALSE |
| RASAL2 | 5356 | 0.662215 | 0.507833 | 0.698589 | FALSE |
| LHB | 1183 | 0.661814 | 0.50809 | 0.698775 | FALSE |
| CNFN | 9461 | 0.661394 | 0.50836 | 0.6989 | FALSE |
| HAUS1 | 7887 | 0.661097 | 0.50855 | 0.699034 | FALSE |
| KCNJ3 | 14611 | 0.661064 | 0.508571 | 0.699034 | FALSE |
| FKBP9P1 | 12478 | 0.661032 | 0.508592 | 0.699034 | FALSE |
| HMGXB4 | 3134 | 0.660722 | 0.508791 | 0.699246 | FALSE |
| BEX5 | 12303 | 0.660388 | 0.509005 | 0.699418 | FALSE |
| HIPK2 | 5652 | 0.660029 | 0.509235 | 0.699673 | FALSE |
| ACBD7 | 5937 | 0.659665 | 0.509469 | 0.699809 | FALSE |
| PCCA | 9161 | 0.659402 | 0.509638 | 0.699878 | FALSE |
| PURG | 14942 | 0.659384 | 0.509649 | 0.699878 | FALSE |
| AMIGO1 | 10356 | 0.659005 | 0.509893 | 0.700099 | FALSE |
| RANBP10 | 7643 | 0.658932 | 0.509939 | 0.700099 | FALSE |
| EZH1 | 9564 | 0.658716 | 0.510078 | 0.700099 | FALSE |
| GRPEL1 | 4727 | 0.658572 | 0.510171 | 0.700114 | FALSE |
| PRPF4 | 12721 | 0.658451 | 0.510248 | 0.700114 | FALSE |
| 11-Mar | 5101 | 0.65836 | 0.510307 | 0.700114 | FALSE |
| ATP2B1 | 53 | 0.658343 | 0.510318 | 0.700114 | FALSE |
| KPNA3 | 8493 | 0.657795 | 0.51067 | 0.700346 | FALSE |
| ZRANB2 | 14406 | 0.657697 | 0.510733 | 0.700346 | FALSE |
| MYO7A | 7329 | 0.657681 | 0.510743 | 0.700346 | FALSE |
| CREB3L2 | 9270 | 0.657506 | 0.510856 | 0.700421 | FALSE |
| PSMC3IP | 10831 | 0.657211 | 0.511045 | 0.700458 | FALSE |
| ZFP28 | 1790 | 0.657048 | 0.51115 | 0.700458 | FALSE |
| SPAG9 | 4229 | 0.657046 | 0.511151 | 0.700458 | FALSE |
| CTSS | 13174 | 0.656085 | 0.511769 | 0.70101 | FALSE |
| SLC13A3 | 570 | 0.655707 | 0.512013 | 0.701118 | FALSE |
| E2F1 | 12552 | 0.655608 | 0.512076 | 0.701118 | FALSE |
| OSBP | 9307 | 0.655542 | 0.512119 | 0.701118 | FALSE |
| MRO | 10705 | 0.655481 | 0.512158 | 0.701118 | FALSE |
| S1PR5 | 5573 | 0.655461 | 0.512171 | 0.701118 | FALSE |
| CLEC11A | 12106 | 0.654975 | 0.512484 | 0.701424 | FALSE |
| RHBDD1 | 10981 | 0.654382 | 0.512866 | 0.701823 | FALSE |
| TFE3 | 13947 | 0.653941 | 0.51315 | 0.702011 | FALSE |
| FAM209B | 5000 | 0.653854 | 0.513206 | 0.702011 | FALSE |
| SPDL1 | 13948 | 0.653738 | 0.513281 | 0.702011 | FALSE |
| PDK1 | 10032 | 0.653623 | 0.513355 | 0.702011 | FALSE |
| SF3A1 | 15491 | 0.653128 | 0.513674 | 0.702191 | FALSE |
| NME5 | 15138 | 0.652525 | 0.514063 | 0.702578 | FALSE |
| MYLK2 | 7131 | 0.652502 | 0.514077 | 0.702578 | FALSE |
| KIF19 | 13335 | 0.65248 | 0.514092 | 0.702578 | FALSE |
| APOL3 | 9241 | 0.652387 | 0.514152 | 0.702599 | FALSE |
| KCNH8 | 4318 | 0.651946 | 0.514436 | 0.702865 | FALSE |
| DDX21 | 4928 | 0.651172 | 0.514935 | 0.70324 | FALSE |
| CCDC93 | 583 | 0.650893 | 0.515116 | 0.703301 | FALSE |
| HIBCH | 6516 | 0.650312 | 0.515491 | 0.703629 | FALSE |
| LINC00950 | 9486 | 0.650038 | 0.515668 | 0.703809 | FALSE |
| EXOC4 | 7209 | 0.649615 | 0.515941 | 0.704059 | FALSE |
| CDH24 | 2145 | 0.649188 | 0.516217 | 0.704191 | FALSE |
| SH3BP4 | 3654 | 0.648972 | 0.516356 | 0.704223 | FALSE |
| PTMA | 9243 | 0.648958 | 0.516366 | 0.704223 | FALSE |
| COA5 | 14618 | 0.648688 | 0.51654 | 0.704303 | FALSE |
| PRKRA | 8864 | 0.648321 | 0.516777 | 0.704453 | FALSE |
| SYNJ2 | 10256 | 0.647935 | 0.517027 | 0.70468 | FALSE |
| SLC25A19 | 9321 | 0.647813 | 0.517106 | 0.704726 | FALSE |
| MFN1 | 10355 | 0.647073 | 0.517585 | 0.704995 | FALSE |
| TRAPPC11 | 1527 | 0.646884 | 0.517707 | 0.704995 | FALSE |
| ALPK1 | 6764 | 0.646849 | 0.51773 | 0.704995 | FALSE |
| PPP2R5B | 10416 | 0.6462 | 0.51815 | 0.705466 | FALSE |
| ADRA1A | 14342 | 0.646025 | 0.518263 | 0.705504 | FALSE |
| ZNF549 | 1800 | 0.645859 | 0.518371 | 0.705559 | FALSE |
| PDCD1 | 5286 | 0.645325 | 0.518717 | 0.705597 | FALSE |
| MED22 | 4644 | 0.645293 | 0.518737 | 0.705597 | FALSE |
| PCDHGB2 | 14302 | 0.644712 | 0.519114 | 0.705986 | FALSE |
| TBC1D32 | 3712 | 0.644428 | 0.519298 | 0.706039 | FALSE |
| YTHDF2 | 8311 | 0.644415 | 0.519306 | 0.706039 | FALSE |
| SREBF2 | 478 | 0.643759 | 0.519732 | 0.706396 | FALSE |
| WNK1 | 3821 | 0.643592 | 0.51984 | 0.706482 | FALSE |
| PTPRJ | 4681 | 0.643191 | 0.5201 | 0.706712 | FALSE |
| CCDC122 | 3128 | 0.642566 | 0.520506 | 0.707202 | FALSE |
| LRRC20 | 2768 | 0.642417 | 0.520602 | 0.707272 | FALSE |
| KLHL11 | 10299 | 0.642293 | 0.520683 | 0.707317 | FALSE |
| PRH2 | 12754 | 0.642107 | 0.520804 | 0.707328 | FALSE |
| EFCAB7 | 6106 | 0.642075 | 0.520824 | 0.707328 | FALSE |
| SLC43A1 | 10252 | 0.641979 | 0.520887 | 0.707351 | FALSE |
| SSB | 12994 | 0.641871 | 0.520957 | 0.707381 | FALSE |
| TNFRSF4 | 13658 | 0.641302 | 0.521326 | 0.707641 | FALSE |
| MSMP | 6616 | 0.641192 | 0.521398 | 0.707676 | FALSE |
| POT1 | 9715 | 0.640957 | 0.521551 | 0.707822 | FALSE |
| TRMT44 | 9387 | 0.640532 | 0.521827 | 0.708085 | FALSE |
| PRKCQ | 3806 | 0.640341 | 0.521951 | 0.708119 | FALSE |
| RAMP3 | 9361 | 0.640259 | 0.522004 | 0.70813 | FALSE |
| SLC22A10 | 12572 | 0.640048 | 0.522141 | 0.708193 | FALSE |
| CA2 | 15063 | 0.639939 | 0.522212 | 0.708212 | FALSE |
| TXNDC11 | 15155 | 0.639704 | 0.522365 | 0.708312 | FALSE |
| PCDH11Y | 1918 | 0.63916 | 0.522719 | 0.708597 | FALSE |
| MAPKAP1 | 8578 | 0.639103 | 0.522756 | 0.708597 | FALSE |
| SELENOO | 11855 | 0.63901 | 0.522816 | 0.708617 | FALSE |
| TM2D1 | 6625 | 0.638531 | 0.523128 | 0.708862 | FALSE |
| OR52K2 | 2983 | 0.638523 | 0.523133 | 0.708862 | FALSE |
| CNOT2 | 4017 | 0.638388 | 0.523221 | 0.70892 | FALSE |
| MAX | 1489 | 0.637879 | 0.523552 | 0.70928 | FALSE |
| TMEM143 | 7918 | 0.637701 | 0.523668 | 0.70928 | FALSE |
| PDZRN3 | 1177 | 0.637505 | 0.523796 | 0.70933 | FALSE |
| DNASE1 | 10464 | 0.637331 | 0.523909 | 0.70937 | FALSE |
| AGFG2 | 14415 | 0.637285 | 0.523939 | 0.70937 | FALSE |
| LINC00167 | 6364 | 0.636881 | 0.524202 | 0.709584 | FALSE |
| LYSMD3 | 4401 | 0.636814 | 0.524246 | 0.709584 | FALSE |
| LINC00844 | 13496 | 0.636728 | 0.524302 | 0.709584 | FALSE |
| APLP2 | 13279 | 0.636719 | 0.524308 | 0.709584 | FALSE |
| RFESD | 10872 | 0.63664 | 0.524359 | 0.709584 | FALSE |
| TIMM21 | 12053 | 0.636032 | 0.524756 | 0.709768 | FALSE |
| JHY | 10934 | 0.635252 | 0.525264 | 0.710228 | FALSE |
| PRR5L | 5594 | 0.634719 | 0.525612 | 0.710432 | FALSE |
| NCOR1 | 13434 | 0.634685 | 0.525634 | 0.710432 | FALSE |
| NEMF | 15398 | 0.634507 | 0.52575 | 0.710432 | FALSE |
| INPPL1 | 6153 | 0.634443 | 0.525792 | 0.710432 | FALSE |
| VPREB1 | 6093 | 0.634249 | 0.525918 | 0.710542 | FALSE |
| ZCCHC4 | 8617 | 0.632719 | 0.526917 | 0.711534 | FALSE |
| ATG10 | 15249 | 0.632189 | 0.527263 | 0.711767 | FALSE |
| KANK4 | 11405 | 0.632163 | 0.52728 | 0.711767 | FALSE |
| DEPP1 | 7608 | 0.632027 | 0.527369 | 0.711826 | FALSE |
| RNF130 | 13656 | 0.631742 | 0.527555 | 0.711972 | FALSE |
| LPIN1 | 1853 | 0.631569 | 0.527669 | 0.711972 | FALSE |
| UBTD1 | 14833 | 0.631282 | 0.527856 | 0.712026 | FALSE |
| RBM45 | 12083 | 0.631242 | 0.527882 | 0.712026 | FALSE |
| CDC7 | 8408 | 0.630721 | 0.528223 | 0.712237 | FALSE |
| CXorf36 | 14604 | 0.630704 | 0.528234 | 0.712237 | FALSE |
| SNX5 | 9835 | 0.630617 | 0.528291 | 0.712237 | FALSE |
| SEL1L | 6981 | 0.630238 | 0.528539 | 0.71242 | FALSE |
| WDR89 | 4605 | 0.629625 | 0.52894 | 0.712717 | FALSE |
| CAMSAP2 | 15225 | 0.629623 | 0.528941 | 0.712717 | FALSE |
| PPT2 | 8194 | 0.629458 | 0.529049 | 0.712801 | FALSE |
| EP400 | 5997 | 0.629139 | 0.529258 | 0.712959 | FALSE |
| RALGAPB | 15109 | 0.628834 | 0.529458 | 0.713167 | FALSE |
| RBP7 | 8134 | 0.627661 | 0.530226 | 0.713845 | FALSE |
| ZNF334 | 1592 | 0.627647 | 0.530235 | 0.713845 | FALSE |
| HHAT | 9034 | 0.627184 | 0.530539 | 0.714192 | FALSE |
| USP32 | 14876 | 0.627052 | 0.530625 | 0.714222 | FALSE |
| PNN | 5546 | 0.626871 | 0.530744 | 0.714222 | FALSE |
| ATXN7L2 | 5675 | 0.626773 | 0.530808 | 0.714247 | FALSE |
| SYNCRIP | 1089 | 0.626394 | 0.531057 | 0.714397 | FALSE |
| C17orf100 | 5469 | 0.626206 | 0.53118 | 0.714422 | FALSE |
| PNMA6E | 5717 | 0.626156 | 0.531213 | 0.714422 | FALSE |
| GPR4 | 10955 | 0.625945 | 0.531351 | 0.714547 | FALSE |
| RXRA | 8461 | 0.625148 | 0.531874 | 0.71498 | FALSE |
| SLC35D2 | 14144 | 0.62474 | 0.532142 | 0.715241 | FALSE |
| SP9 | 14631 | 0.624347 | 0.5324 | 0.715465 | FALSE |
| MAGI1 | 14900 | 0.623745 | 0.532795 | 0.715873 | FALSE |
| NRARP | 5651 | 0.622293 | 0.533749 | 0.716604 | FALSE |
| VEGFA | 12582 | 0.622251 | 0.533777 | 0.716604 | FALSE |
| FAM9B | 12606 | 0.62222 | 0.533797 | 0.716604 | FALSE |
| ADH1C | 2775 | 0.621946 | 0.533977 | 0.716784 | FALSE |
| NUP50 | 2684 | 0.621175 | 0.534484 | 0.717219 | FALSE |
| ZNF813 | 14123 | 0.620921 | 0.534652 | 0.717381 | FALSE |
| KLF3-AS1 | 14597 | 0.620727 | 0.534779 | 0.717491 | FALSE |
| FAM184B | 9372 | 0.620638 | 0.534838 | 0.717508 | FALSE |
| LINC00672 | 15144 | 0.620463 | 0.534953 | 0.717573 | FALSE |
| LPCAT1 | 15410 | 0.620425 | 0.534978 | 0.717573 | FALSE |
| HHATL | 7307 | 0.620241 | 0.535099 | 0.717674 | FALSE |
| MTREX | 10421 | 0.620164 | 0.53515 | 0.71768 | FALSE |
| GPR61 | 1841 | 0.619982 | 0.53527 | 0.717718 | FALSE |
| MAP3K14 | 15112 | 0.618855 | 0.536012 | 0.718462 | FALSE |
| UBXN2B | 5210 | 0.618835 | 0.536025 | 0.718462 | FALSE |
| LOC440040 | 4374 | 0.61879 | 0.536055 | 0.718462 | FALSE |
| PIH1D2 | 5151 | 0.617462 | 0.53693 | 0.719058 | FALSE |
| HCRT | 14366 | 0.617049 | 0.537202 | 0.71926 | FALSE |
| CCDC127 | 947 | 0.616608 | 0.537493 | 0.719588 | FALSE |
| SDC2 | 13490 | 0.616376 | 0.537646 | 0.71967 | FALSE |
| CISH | 1079 | 0.616168 | 0.537784 | 0.719759 | FALSE |
| CNOT9 | 4601 | 0.615726 | 0.538075 | 0.719909 | FALSE |
| LPXN | 9668 | 0.615687 | 0.538101 | 0.719909 | FALSE |
| ZNF614 | 3062 | 0.615406 | 0.538287 | 0.720034 | FALSE |
| HSD17B12 | 10403 | 0.614563 | 0.538843 | 0.720441 | FALSE |
| CSDC2 | 11269 | 0.613543 | 0.539517 | 0.72094 | FALSE |
| TSEN54 | 14475 | 0.613325 | 0.539661 | 0.721026 | FALSE |
| CBR3-AS1 | 2422 | 0.613079 | 0.539824 | 0.721165 | FALSE |
| BCAP29 | 8389 | 0.612644 | 0.540112 | 0.721426 | FALSE |
| ZNRF1 | 10090 | 0.612377 | 0.540288 | 0.7216 | FALSE |
| BIN3 | 13745 | 0.612137 | 0.540447 | 0.721684 | FALSE |
| MOSPD1 | 4691 | 0.612073 | 0.540489 | 0.721684 | FALSE |
| YPEL3 | 15486 | 0.611997 | 0.54054 | 0.721689 | FALSE |
| RASL10B | 2859 | 0.611855 | 0.540634 | 0.721699 | FALSE |
| TOMM34 | 12434 | 0.61135 | 0.540968 | 0.722014 | FALSE |
| EPS15L1 | 159 | 0.61118 | 0.54108 | 0.722103 | FALSE |
| SMPX | 5450 | 0.611089 | 0.541141 | 0.722121 | FALSE |
| NPC1 | 11155 | 0.610919 | 0.541253 | 0.722126 | FALSE |
| A2ML1 | 13039 | 0.610631 | 0.541444 | 0.722218 | FALSE |
| NEGR1 | 15285 | 0.610248 | 0.541698 | 0.722433 | FALSE |
| MID1IP1 | 12304 | 0.609281 | 0.542338 | 0.723132 | FALSE |
| RAPGEF3 | 13663 | 0.608072 | 0.54314 | 0.723649 | FALSE |
| TMEM79 | 15287 | 0.608052 | 0.543153 | 0.723649 | FALSE |
| USP6 | 506 | 0.608034 | 0.543165 | 0.723649 | FALSE |
| PM20D2 | 12354 | 0.607637 | 0.543428 | 0.723931 | FALSE |
| LRP6 | 12247 | 0.607383 | 0.543597 | 0.723931 | FALSE |
| THAP7-AS1 | 14418 | 0.607221 | 0.543704 | 0.723997 | FALSE |
| BICD1 | 15070 | 0.606485 | 0.544193 | 0.724448 | FALSE |
| TBC1D12 | 10021 | 0.606292 | 0.544321 | 0.724448 | FALSE |
| COBL | 15101 | 0.606073 | 0.544466 | 0.72458 | FALSE |
| TEF | 1094 | 0.605886 | 0.54459 | 0.724684 | FALSE |
| ZNF124 | 12210 | 0.605647 | 0.544749 | 0.724771 | FALSE |
| FAM219A | 15451 | 0.60525 | 0.545013 | 0.724998 | FALSE |
| NGDN | 3638 | 0.605042 | 0.545151 | 0.724998 | FALSE |
| PEG3-AS1 | 1879 | 0.604893 | 0.54525 | 0.725029 | FALSE |
| KMT2B | 8007 | 0.604867 | 0.545267 | 0.725029 | FALSE |
| CHD4 | 15106 | 0.604164 | 0.545735 | 0.725538 | FALSE |
| ETS1 | 10094 | 0.604081 | 0.54579 | 0.725538 | FALSE |
| WBP1L | 8811 | 0.60401 | 0.545837 | 0.725539 | FALSE |
| NPFF | 2624 | 0.603235 | 0.546352 | 0.726101 | FALSE |
| GCC1 | 7990 | 0.602801 | 0.546641 | 0.7263 | FALSE |
| LZIC | 14938 | 0.602516 | 0.546831 | 0.726366 | FALSE |
| LOC107984974 | 2261 | 0.601541 | 0.54748 | 0.726981 | FALSE |
| LONP1 | 1423 | 0.601443 | 0.547545 | 0.726993 | FALSE |
| MKNK1 | 7432 | 0.601388 | 0.547582 | 0.726993 | FALSE |
| LINC01963 | 12684 | 0.60115 | 0.54774 | 0.727142 | FALSE |
| ZNF264 | 14484 | 0.600791 | 0.547979 | 0.727381 | FALSE |
| MAPK8IP3 | 10237 | 0.600676 | 0.548056 | 0.727381 | FALSE |
| TM4SF18 | 10289 | 0.600601 | 0.548106 | 0.727381 | FALSE |
| GMDS | 10041 | 0.600535 | 0.54815 | 0.727381 | FALSE |
| MTMR10 | 822 | 0.60053 | 0.548153 | 0.727381 | FALSE |
| MEX3B | 13863 | 0.599989 | 0.548514 | 0.727755 | FALSE |
| MFF | 9946 | 0.599967 | 0.548528 | 0.727755 | FALSE |
| ZMYND11 | 9649 | 0.599401 | 0.548906 | 0.728051 | FALSE |
| DHRS1 | 6705 | 0.598476 | 0.549522 | 0.728641 | FALSE |
| NUDT9 | 12234 | 0.59812 | 0.54976 | 0.728833 | FALSE |
| HTR2A | 11456 | 0.597747 | 0.550009 | 0.728872 | FALSE |
| NAIF1 | 10310 | 0.597639 | 0.550081 | 0.728887 | FALSE |
| SUGP2 | 1813 | 0.597131 | 0.55042 | 0.729252 | FALSE |
| PPFIBP2 | 15425 | 0.596773 | 0.550659 | 0.729252 | FALSE |
| FAM160A1 | 11969 | 0.595585 | 0.551453 | 0.730086 | FALSE |
| TMEM241 | 2556 | 0.595161 | 0.551736 | 0.730282 | FALSE |
| RPP25L | 12420 | 0.595007 | 0.551839 | 0.730302 | FALSE |
| PDE9A | 4919 | 0.593964 | 0.552536 | 0.730944 | FALSE |
| VPS37A | 14921 | 0.593916 | 0.552568 | 0.730944 | FALSE |
| RASA3 | 9354 | 0.593758 | 0.552674 | 0.731022 | FALSE |
| SEMA3B | 1794 | 0.59286 | 0.553275 | 0.731569 | FALSE |
| SMC4 | 1784 | 0.5925 | 0.553516 | 0.731826 | FALSE |
| SFT2D3 | 8005 | 0.592388 | 0.553591 | 0.731863 | FALSE |
| TFAP2E | 8085 | 0.591721 | 0.554037 | 0.732206 | FALSE |
| CDK5RAP3 | 15374 | 0.590958 | 0.554549 | 0.73251 | FALSE |
| KRTAP10-8 | 6818 | 0.590446 | 0.554892 | 0.732807 | FALSE |
| ASAH1 | 7404 | 0.590336 | 0.554965 | 0.732807 | FALSE |
| KDM3A | 12561 | 0.590273 | 0.555008 | 0.732807 | FALSE |
| ZNF561 | 14435 | 0.589777 | 0.55534 | 0.732936 | FALSE |
| GGT5 | 9904 | 0.589528 | 0.555507 | 0.733095 | FALSE |
| SLC4A7 | 14722 | 0.589384 | 0.555604 | 0.733161 | FALSE |
| PUS7 | 6259 | 0.589173 | 0.555745 | 0.733285 | FALSE |
| BTBD17 | 2176 | 0.58849 | 0.556203 | 0.733704 | FALSE |
| THAP9 | 6800 | 0.587733 | 0.556712 | 0.734065 | FALSE |
| DBF4B | 15029 | 0.586965 | 0.557227 | 0.734559 | FALSE |
| GPATCH11 | 12976 | 0.586662 | 0.557431 | 0.734765 | FALSE |
| ABTB1 | 3424 | 0.586402 | 0.557605 | 0.734933 | FALSE |
| ARL6IP1 | 14092 | 0.586072 | 0.557827 | 0.735108 | FALSE |
| PSMD3 | 433 | 0.586065 | 0.557832 | 0.735108 | FALSE |
| AQR | 9861 | 0.585458 | 0.55824 | 0.735426 | FALSE |
| NAXD | 13157 | 0.585199 | 0.558414 | 0.735503 | FALSE |
| RGS5 | 10569 | 0.584744 | 0.55872 | 0.7358 | FALSE |
| ID4 | 13358 | 0.584538 | 0.558858 | 0.73584 | FALSE |
| TRIAP1 | 13811 | 0.584327 | 0.559 | 0.735959 | FALSE |
| ID3 | 2561 | 0.584264 | 0.559043 | 0.735959 | FALSE |
| BACH1 | 14318 | 0.583411 | 0.559617 | 0.736226 | FALSE |
| ZNF35 | 4218 | 0.583263 | 0.559716 | 0.736226 | FALSE |
| HEXB | 3984 | 0.582587 | 0.560171 | 0.736583 | FALSE |
| DUS3L | 10938 | 0.58258 | 0.560176 | 0.736583 | FALSE |
| VPREB3 | 4096 | 0.582238 | 0.560406 | 0.736762 | FALSE |
| ZNF66 | 13875 | 0.581709 | 0.560763 | 0.737168 | FALSE |
| KLHL7 | 7800 | 0.581553 | 0.560868 | 0.737192 | FALSE |
| LOC100506388 | 5821 | 0.581387 | 0.56098 | 0.737267 | FALSE |
| TEDC2 | 11880 | 0.580295 | 0.561716 | 0.737924 | FALSE |
| DTNB | 9539 | 0.579513 | 0.562243 | 0.738555 | FALSE |
| RPSAP52 | 9851 | 0.578637 | 0.562834 | 0.739083 | FALSE |
| MIR22HG | 12639 | 0.578543 | 0.562898 | 0.739104 | FALSE |
| UBXN4 | 7628 | 0.578275 | 0.563078 | 0.739218 | FALSE |
| ZNF137P | 12513 | 0.577303 | 0.563735 | 0.739955 | FALSE |
| MTHFD2 | 9129 | 0.577084 | 0.563883 | 0.740087 | FALSE |
| RAP1GAP2 | 4316 | 0.57546 | 0.56498 | 0.741289 | FALSE |
| GAA | 3016 | 0.575425 | 0.565004 | 0.741289 | FALSE |
| LOC102723566 | 12152 | 0.575355 | 0.565051 | 0.741289 | FALSE |
| SLC9B2 | 13657 | 0.575074 | 0.565241 | 0.741372 | FALSE |
| ORM1 | 13596 | 0.574748 | 0.565462 | 0.741508 | FALSE |
| DHX8 | 7390 | 0.574083 | 0.565912 | 0.741775 | FALSE |
| NEO1 | 8787 | 0.573965 | 0.565991 | 0.741796 | FALSE |
| TFR2 | 109 | 0.573197 | 0.566511 | 0.742145 | FALSE |
| SH3RF3 | 5955 | 0.573168 | 0.566531 | 0.742145 | FALSE |
| F7 | 13987 | 0.572848 | 0.566748 | 0.742145 | FALSE |
| RAB22A | 3541 | 0.572697 | 0.56685 | 0.742145 | FALSE |
| PRKCH | 10368 | 0.572654 | 0.566879 | 0.742145 | FALSE |
| SMARCB1 | 6086 | 0.57262 | 0.566902 | 0.742145 | FALSE |
| MCC | 3066 | 0.572618 | 0.566903 | 0.742145 | FALSE |
| KRT3 | 13381 | 0.572596 | 0.566918 | 0.742145 | FALSE |
| NABP2 | 9290 | 0.57251 | 0.566977 | 0.742145 | FALSE |
| NAA60 | 9402 | 0.572479 | 0.566998 | 0.742145 | FALSE |
| PALM | 5759 | 0.572441 | 0.567023 | 0.742145 | FALSE |
| TRAF6 | 1974 | 0.572336 | 0.567094 | 0.742145 | FALSE |
| COPS3 | 2917 | 0.572298 | 0.56712 | 0.742145 | FALSE |
| CD8B | 15385 | 0.572168 | 0.567208 | 0.742145 | FALSE |
| KLK14 | 8435 | 0.572113 | 0.567245 | 0.742145 | FALSE |
| GLI4 | 14503 | 0.572099 | 0.567255 | 0.742145 | FALSE |
| XKR8 | 4256 | 0.57177 | 0.567478 | 0.742282 | FALSE |
| C12orf29 | 6964 | 0.571752 | 0.56749 | 0.742282 | FALSE |
| TBC1D9B | 6162 | 0.571582 | 0.567605 | 0.742355 | FALSE |
| NUDT6 | 11689 | 0.571491 | 0.567667 | 0.742374 | FALSE |
| MLLT1 | 11843 | 0.571295 | 0.5678 | 0.742454 | FALSE |
| ZNF384 | 8907 | 0.571186 | 0.567874 | 0.742454 | FALSE |
| LOC100506844 | 7852 | 0.57112 | 0.567918 | 0.742454 | FALSE |
| CRAMP1 | 11032 | 0.570864 | 0.568092 | 0.742547 | FALSE |
| TREML1 | 4450 | 0.570805 | 0.568132 | 0.742547 | FALSE |
| AGPAT5 | 4160 | 0.570665 | 0.568227 | 0.742547 | FALSE |
| NRG4 | 3086 | 0.570312 | 0.568466 | 0.742549 | FALSE |
| ZNF440 | 6936 | 0.570112 | 0.568602 | 0.742607 | FALSE |
| NAT9 | 13946 | 0.570037 | 0.568653 | 0.742607 | FALSE |
| DDX5 | 10510 | 0.567835 | 0.570147 | 0.744123 | FALSE |
| NAA25 | 2385 | 0.567218 | 0.570566 | 0.744479 | FALSE |
| ALG2 | 4282 | 0.567153 | 0.57061 | 0.744479 | FALSE |
| ARHGAP35 | 6315 | 0.566593 | 0.570991 | 0.744789 | FALSE |
| ELMO2 | 3096 | 0.566395 | 0.571125 | 0.744903 | FALSE |
| ATXN7L1 | 1979 | 0.56631 | 0.571183 | 0.744916 | FALSE |
| UBL4A | 11110 | 0.566133 | 0.571303 | 0.74501 | FALSE |
| FAM155B | 4102 | 0.5656 | 0.571666 | 0.745359 | FALSE |
| SHROOM4 | 6405 | 0.565206 | 0.571934 | 0.745584 | FALSE |
| BORCS8 | 7100 | 0.564613 | 0.572337 | 0.745985 | FALSE |
| CNIH4 | 11917 | 0.56397 | 0.572775 | 0.746431 | FALSE |
| LIMK2 | 8027 | 0.563081 | 0.57338 | 0.746999 | FALSE |
| SDF4 | 5933 | 0.563049 | 0.573402 | 0.746999 | FALSE |
| ZNF708 | 9370 | 0.562728 | 0.57362 | 0.74716 | FALSE |
| ZNF281 | 1588 | 0.561963 | 0.574141 | 0.747713 | FALSE |
| RSL1D1 | 9794 | 0.561747 | 0.574288 | 0.747843 | FALSE |
| LINC00839 | 1763 | 0.561326 | 0.574575 | 0.748029 | FALSE |
| PEX5 | 7341 | 0.560606 | 0.575066 | 0.748544 | FALSE |
| TRIM21 | 536 | 0.560271 | 0.575295 | 0.748716 | FALSE |
| FAM122B | 13803 | 0.558813 | 0.576289 | 0.749761 | FALSE |
| TGFB2 | 2403 | 0.55854 | 0.576476 | 0.749934 | FALSE |
| ZNFX1 | 2002 | 0.558456 | 0.576533 | 0.749934 | FALSE |
| NAALADL2 | 14783 | 0.558408 | 0.576566 | 0.749934 | FALSE |
| LOC339059 | 5392 | 0.558207 | 0.576703 | 0.750007 | FALSE |
| RNF170 | 702 | 0.558066 | 0.576799 | 0.75005 | FALSE |
| ZNF213-AS1 | 902 | 0.557708 | 0.577044 | 0.750119 | FALSE |
| CENPB | 12714 | 0.557503 | 0.577184 | 0.750238 | FALSE |
| PKDCC | 450 | 0.557128 | 0.57744 | 0.75039 | FALSE |
| SBK1 | 6474 | 0.557121 | 0.577445 | 0.75039 | FALSE |
| HUS1B | 12352 | 0.557024 | 0.577511 | 0.750414 | FALSE |
| MRPL22 | 10582 | 0.556844 | 0.577634 | 0.750511 | FALSE |
| TMEM179 | 4300 | 0.556405 | 0.577934 | 0.750773 | FALSE |
| KNOP1 | 5847 | 0.555923 | 0.578263 | 0.751017 | FALSE |
| PDE3B | 12413 | 0.555835 | 0.578324 | 0.751033 | FALSE |
| KISS1R | 11084 | 0.555305 | 0.578686 | 0.751379 | FALSE |
| LRRC37B | 4176 | 0.55505 | 0.57886 | 0.75151 | FALSE |
| ATXN2L | 14155 | 0.554085 | 0.579521 | 0.752088 | FALSE |
| IMMP1L | 4488 | 0.55385 | 0.579682 | 0.752234 | FALSE |
| PRKAR1B | 5919 | 0.553434 | 0.579966 | 0.752541 | FALSE |
| POLN | 10477 | 0.553155 | 0.580157 | 0.752664 | FALSE |
| MTSS1L | 7379 | 0.552973 | 0.580282 | 0.752702 | FALSE |
| CADM3 | 15050 | 0.552826 | 0.580383 | 0.752702 | FALSE |
| ZNF701 | 8983 | 0.552196 | 0.580814 | 0.753016 | FALSE |
| FOXN2 | 1540 | 0.551877 | 0.581033 | 0.753237 | FALSE |
| GABBR1 | 4870 | 0.551113 | 0.581556 | 0.753666 | FALSE |
| LONRF1 | 6880 | 0.550914 | 0.581693 | 0.753713 | FALSE |
| AAR2 | 14354 | 0.550778 | 0.581786 | 0.753713 | FALSE |
| UNC5A | 2574 | 0.550693 | 0.581844 | 0.753726 | FALSE |
| TTC31 | 8288 | 0.55051 | 0.58197 | 0.753826 | FALSE |
| ITPA | 11665 | 0.550242 | 0.582153 | 0.753857 | FALSE |
| LIPT1 | 733 | 0.550239 | 0.582155 | 0.753857 | FALSE |
| SPATA2 | 8249 | 0.550194 | 0.582186 | 0.753857 | FALSE |
| ALG13 | 7486 | 0.54999 | 0.582326 | 0.753937 | FALSE |
| CNOT4 | 6148 | 0.549713 | 0.582516 | 0.754097 | FALSE |
| SNX19 | 3495 | 0.549307 | 0.582795 | 0.754395 | FALSE |
| MBNL1 | 15453 | 0.549184 | 0.582879 | 0.754397 | FALSE |
| ZNF800 | 14290 | 0.548078 | 0.583638 | 0.75505 | FALSE |
| GPR75 | 4918 | 0.547406 | 0.5841 | 0.755334 | FALSE |
| NAIP | 7421 | 0.547329 | 0.584153 | 0.75534 | FALSE |
| RAD50 | 8669 | 0.546725 | 0.584568 | 0.75556 | FALSE |
| KYAT3 | 8682 | 0.546653 | 0.584617 | 0.75556 | FALSE |
| MBTD1 | 7125 | 0.546522 | 0.584707 | 0.75556 | FALSE |
| ZNF442 | 4049 | 0.546519 | 0.584709 | 0.75556 | FALSE |
| ULBP2 | 4579 | 0.546202 | 0.584927 | 0.755713 | FALSE |
| MOGS | 2793 | 0.546133 | 0.584975 | 0.755713 | FALSE |
| SLC30A9 | 11544 | 0.545933 | 0.585112 | 0.755713 | FALSE |
| LINC00216 | 7648 | 0.545859 | 0.585163 | 0.755713 | FALSE |
| UNC13A | 946 | 0.545808 | 0.585198 | 0.755713 | FALSE |
| FXN | 13855 | 0.545779 | 0.585218 | 0.755713 | FALSE |
| SLC25A27 | 3440 | 0.545713 | 0.585263 | 0.755713 | FALSE |
| NBR2 | 4031 | 0.545439 | 0.585452 | 0.755769 | FALSE |
| PJA2 | 11178 | 0.545267 | 0.58557 | 0.755859 | FALSE |
| TRAM1 | 15450 | 0.545156 | 0.585646 | 0.755896 | FALSE |
| GALC | 1121 | 0.544649 | 0.585995 | 0.756221 | FALSE |
| UBE2NL | 9868 | 0.544387 | 0.586175 | 0.756329 | FALSE |
| YY1P2 | 1601 | 0.544176 | 0.58632 | 0.756391 | FALSE |
| CRTAC1 | 12421 | 0.544176 | 0.58632 | 0.756391 | FALSE |
| NDST2 | 10381 | 0.544056 | 0.586403 | 0.756422 | FALSE |
| ZNF707 | 15596 | 0.543916 | 0.586499 | 0.756422 | FALSE |
| ZNF818P | 14853 | 0.54386 | 0.586538 | 0.756422 | FALSE |
| NFIL3 | 14091 | 0.543551 | 0.58675 | 0.756571 | FALSE |
| CYP1A2 | 12141 | 0.543218 | 0.58698 | 0.756804 | FALSE |
| SDHB | 4398 | 0.542987 | 0.587139 | 0.756873 | FALSE |
| GCAT | 11418 | 0.542892 | 0.587204 | 0.756873 | FALSE |
| GGTLC2 | 4044 | 0.542859 | 0.587227 | 0.756873 | FALSE |
| SGCD | 1935 | 0.542531 | 0.587453 | 0.757102 | FALSE |
| SIAH3 | 14584 | 0.541991 | 0.587825 | 0.75728 | FALSE |
| KAT2B | 13139 | 0.541984 | 0.58783 | 0.75728 | FALSE |
| POLDIP3 | 7959 | 0.541979 | 0.587833 | 0.75728 | FALSE |
| ITGAV | 895 | 0.541789 | 0.587964 | 0.757386 | FALSE |
| PRPF38B | 10328 | 0.54088 | 0.58859 | 0.757943 | FALSE |
| KIAA1257 | 2272 | 0.540611 | 0.588776 | 0.758057 | FALSE |
| ARF6 | 3402 | 0.540464 | 0.588877 | 0.758063 | FALSE |
| THAP9-AS1 | 11287 | 0.540394 | 0.588925 | 0.758063 | FALSE |
| NUP62 | 11078 | 0.540107 | 0.589123 | 0.758142 | FALSE |
| YAE1D1 | 296 | 0.539947 | 0.589234 | 0.75821 | FALSE |
| RAI2 | 11229 | 0.539766 | 0.589358 | 0.758245 | FALSE |
| TOR1AIP1 | 9058 | 0.539291 | 0.589686 | 0.758503 | FALSE |
| NLGN4Y | 6310 | 0.539254 | 0.589712 | 0.758503 | FALSE |
| RASD1 | 15114 | 0.539066 | 0.589841 | 0.758503 | FALSE |
| NAP1L4 | 7157 | 0.539059 | 0.589846 | 0.758503 | FALSE |
| C2orf88 | 14445 | 0.538747 | 0.590061 | 0.758713 | FALSE |
| PRR7 | 11888 | 0.538616 | 0.590152 | 0.758764 | FALSE |
| RAP1A | 8671 | 0.538549 | 0.590198 | 0.758764 | FALSE |
| SYNJ1 | 9985 | 0.537708 | 0.590779 | 0.759385 | FALSE |
| RPS6KA1 | 11684 | 0.537618 | 0.590841 | 0.759389 | FALSE |
| SELENOP | 2273 | 0.537563 | 0.590879 | 0.759389 | FALSE |
| SLC22A17 | 6418 | 0.537078 | 0.591214 | 0.759695 | FALSE |
| THSD1 | 10739 | 0.53677 | 0.591426 | 0.759906 | FALSE |
| FAM71F2 | 3272 | 0.535894 | 0.592032 | 0.760538 | FALSE |
| PDAP1 | 3004 | 0.535255 | 0.592474 | 0.76082 | FALSE |
| CNPY4 | 3824 | 0.534725 | 0.59284 | 0.761044 | FALSE |
| GLS | 6524 | 0.534713 | 0.592848 | 0.761044 | FALSE |
| IPPK | 897 | 0.534559 | 0.592955 | 0.761119 | FALSE |
| PIGG | 6493 | 0.534301 | 0.593133 | 0.761257 | FALSE |
| ATP7B | 11782 | 0.534249 | 0.593169 | 0.761257 | FALSE |
| KCTD14 | 6822 | 0.534062 | 0.593299 | 0.761257 | FALSE |
| KMT2C | 8297 | 0.534051 | 0.593306 | 0.761257 | FALSE |
| BTNL2 | 10347 | 0.533897 | 0.593413 | 0.76128 | FALSE |
| RAB21 | 1225 | 0.533814 | 0.59347 | 0.76128 | FALSE |
| USP40 | 15493 | 0.533554 | 0.59365 | 0.761442 | FALSE |
| ABL1 | 9521 | 0.53342 | 0.593743 | 0.761442 | FALSE |
| TBXA2R | 1516 | 0.533005 | 0.59403 | 0.761748 | FALSE |
| ARL17A | 6925 | 0.53283 | 0.594151 | 0.761841 | FALSE |
| PMS2P1 | 6610 | 0.532732 | 0.594219 | 0.761866 | FALSE |
| SNRPA1 | 6945 | 0.532497 | 0.594382 | 0.761929 | FALSE |
| VCL | 14633 | 0.532486 | 0.594389 | 0.761929 | FALSE |
| DNAJC17 | 2127 | 0.532449 | 0.594415 | 0.761929 | FALSE |
| RNF112 | 11135 | 0.53208 | 0.594671 | 0.762132 | FALSE |
| KCTD11 | 12520 | 0.531937 | 0.59477 | 0.762189 | FALSE |
| CCDC69 | 8593 | 0.531875 | 0.594813 | 0.762189 | FALSE |
| BRD3 | 5030 | 0.531329 | 0.595191 | 0.762302 | FALSE |
| XPO7 | 4240 | 0.531325 | 0.595194 | 0.762302 | FALSE |
| DDX24 | 8331 | 0.53084 | 0.59553 | 0.762608 | FALSE |
| HDHD5-AS1 | 12955 | 0.530522 | 0.59575 | 0.762819 | FALSE |
| TPCN2 | 7403 | 0.53018 | 0.595987 | 0.763006 | FALSE |
| SENP5 | 8881 | 0.52991 | 0.596174 | 0.763122 | FALSE |
| IGFBP6 | 15620 | 0.529797 | 0.596253 | 0.763159 | FALSE |
| CPN2 | 2519 | 0.529638 | 0.596363 | 0.763237 | FALSE |
| SSH2 | 3293 | 0.529515 | 0.596448 | 0.763284 | FALSE |
| FEM1B | 2739 | 0.529305 | 0.596594 | 0.763345 | FALSE |
| DENND1A | 7224 | 0.529083 | 0.596748 | 0.763373 | FALSE |
| RPS13 | 11798 | 0.529033 | 0.596783 | 0.763373 | FALSE |
| C1orf100 | 8889 | 0.528994 | 0.59681 | 0.763373 | FALSE |
| C5AR2 | 10008 | 0.528992 | 0.596811 | 0.763373 | FALSE |
| QRFP | 6139 | 0.528603 | 0.597081 | 0.763656 | FALSE |
| ANKRD18A | 14268 | 0.528466 | 0.597176 | 0.763715 | FALSE |
| IDH3A | 4478 | 0.527935 | 0.597544 | 0.764045 | FALSE |
| HECTD4 | 12625 | 0.527883 | 0.597581 | 0.764045 | FALSE |
| NCBP1 | 9097 | 0.527764 | 0.597663 | 0.764088 | FALSE |
| NKAPD1 | 13404 | 0.527349 | 0.597951 | 0.764331 | FALSE |
| DMRT2 | 8054 | 0.526939 | 0.598236 | 0.764553 | FALSE |
| RSPRY1 | 12716 | 0.526237 | 0.598724 | 0.764693 | FALSE |
| ERP44 | 2508 | 0.525755 | 0.599058 | 0.76493 | FALSE |
| ZNF597 | 14902 | 0.525388 | 0.599313 | 0.765134 | FALSE |
| CCDC7 | 9312 | 0.524912 | 0.599644 | 0.765369 | FALSE |
| FBN3 | 12414 | 0.524777 | 0.599738 | 0.765381 | FALSE |
| CTAGE9 | 13494 | 0.524715 | 0.599781 | 0.765381 | FALSE |
| KLKB1 | 4840 | 0.524533 | 0.599908 | 0.765455 | FALSE |
| CASC4 | 9729 | 0.524463 | 0.599957 | 0.765455 | FALSE |
| SAPCD2 | 7976 | 0.523997 | 0.600281 | 0.765743 | FALSE |
| ZBED3 | 11807 | 0.523845 | 0.600386 | 0.765743 | FALSE |
| FCER2 | 12514 | 0.523718 | 0.600475 | 0.765743 | FALSE |
| TGFBR2 | 8691 | 0.52345 | 0.600661 | 0.765866 | FALSE |
| MSL3P1 | 4914 | 0.523287 | 0.600775 | 0.765866 | FALSE |
| SLC23A2 | 14872 | 0.523225 | 0.600818 | 0.765866 | FALSE |
| TMEM163 | 4228 | 0.522957 | 0.601004 | 0.765966 | FALSE |
| ZNF793-AS1 | 5254 | 0.522901 | 0.601043 | 0.765966 | FALSE |
| SNX29P2 | 5048 | 0.522049 | 0.601636 | 0.766543 | FALSE |
| ANXA7 | 5208 | 0.522039 | 0.601643 | 0.766543 | FALSE |
| PC | 7926 | 0.520941 | 0.602408 | 0.767296 | FALSE |
| XPO5 | 14135 | 0.520909 | 0.60243 | 0.767296 | FALSE |
| STEAP1B | 10182 | 0.520323 | 0.602838 | 0.767691 | FALSE |
| SOSTDC1 | 13348 | 0.520117 | 0.602982 | 0.767797 | FALSE |
| MED9 | 11024 | 0.519458 | 0.603441 | 0.768208 | FALSE |
| PAQR6 | 8494 | 0.51922 | 0.603607 | 0.768357 | FALSE |
| 2-Mar | 11248 | 0.518957 | 0.603791 | 0.768528 | FALSE |
| CASP6 | 6222 | 0.518671 | 0.60399 | 0.768556 | FALSE |
| RIOX1 | 12232 | 0.517974 | 0.604476 | 0.768958 | FALSE |
| TAF6L | 8608 | 0.517518 | 0.604795 | 0.769242 | FALSE |
| SHROOM1 | 9384 | 0.517425 | 0.60486 | 0.769262 | FALSE |
| CDC42SE1 | 6664 | 0.516767 | 0.605319 | 0.769784 | FALSE |
| INF2 | 10337 | 0.516343 | 0.605615 | 0.770013 | FALSE |
| MCCC1 | 5971 | 0.516294 | 0.605649 | 0.770013 | FALSE |
| IQCB1 | 12913 | 0.516156 | 0.605745 | 0.770013 | FALSE |
| ZNF576 | 1587 | 0.515655 | 0.606095 | 0.770301 | FALSE |
| NIM1K | 10127 | 0.515535 | 0.606179 | 0.770301 | FALSE |
| GPR37 | 14394 | 0.515456 | 0.606234 | 0.770301 | FALSE |
| GALNT7 | 13084 | 0.515118 | 0.606471 | 0.770389 | FALSE |
| EIF2AK4 | 9174 | 0.515106 | 0.606479 | 0.770389 | FALSE |
| ZNF77 | 14011 | 0.515098 | 0.606485 | 0.770389 | FALSE |
| FGFR4 | 2949 | 0.514279 | 0.607057 | 0.770786 | FALSE |
| PHKB | 5533 | 0.51418 | 0.607126 | 0.770786 | FALSE |
| POLDIP2 | 10813 | 0.513717 | 0.60745 | 0.770983 | FALSE |
| RNMT | 1483 | 0.513652 | 0.607495 | 0.770983 | FALSE |
| TSHZ2 | 9781 | 0.513561 | 0.607559 | 0.771002 | FALSE |
| TTF2 | 3145 | 0.51349 | 0.607609 | 0.771002 | FALSE |
| HMGB3 | 9362 | 0.513199 | 0.607812 | 0.771182 | FALSE |
| LOC730098 | 720 | 0.513001 | 0.607951 | 0.771248 | FALSE |
| CSNK1G2 | 4145 | 0.512404 | 0.608368 | 0.771653 | FALSE |
| CXorf67 | 4266 | 0.5117 | 0.608861 | 0.77209 | FALSE |
| RIOK3 | 15630 | 0.511621 | 0.608916 | 0.772097 | FALSE |
| ARMC8 | 10633 | 0.511385 | 0.609081 | 0.772176 | FALSE |
| TRIM14 | 1972 | 0.510872 | 0.609441 | 0.772373 | FALSE |
| ZNF688 | 9052 | 0.510824 | 0.609474 | 0.772373 | FALSE |
| RNF219 | 14222 | 0.510816 | 0.60948 | 0.772373 | FALSE |
| SLC18B1 | 7359 | 0.510311 | 0.609834 | 0.772759 | FALSE |
| KIDINS220 | 6101 | 0.509026 | 0.610734 | 0.773513 | FALSE |
| PPP1CC | 7375 | 0.508968 | 0.610775 | 0.773513 | FALSE |
| MEG3 | 13780 | 0.508407 | 0.611168 | 0.773905 | FALSE |
| SLC24A1 | 3155 | 0.508385 | 0.611183 | 0.773905 | FALSE |
| CCND1 | 7894 | 0.508293 | 0.611248 | 0.773924 | FALSE |
| ERBIN | 10077 | 0.507591 | 0.61174 | 0.774359 | FALSE |
| MTFMT | 1543 | 0.507518 | 0.611791 | 0.774361 | FALSE |
| LIF | 10215 | 0.507078 | 0.6121 | 0.774583 | FALSE |
| PCOLCE | 3259 | 0.507056 | 0.612116 | 0.774583 | FALSE |
| SCGB1C1 | 9773 | 0.506802 | 0.612294 | 0.774746 | FALSE |
| SLC7A14 | 1268 | 0.506638 | 0.612409 | 0.774801 | FALSE |
| TCIRG1 | 9153 | 0.506572 | 0.612455 | 0.774801 | FALSE |
| MTERF1 | 9932 | 0.506528 | 0.612486 | 0.774801 | FALSE |
| SCP2 | 3088 | 0.505912 | 0.612918 | 0.775097 | FALSE |
| SPRTN | 6022 | 0.504898 | 0.61363 | 0.775621 | FALSE |
| PGP | 14059 | 0.504595 | 0.613843 | 0.775769 | FALSE |
| PUM1 | 13569 | 0.50459 | 0.613847 | 0.775769 | FALSE |
| HIST1H3A | 11522 | 0.504513 | 0.613901 | 0.775775 | FALSE |
| MAN1B1 | 5003 | 0.504296 | 0.614053 | 0.775842 | FALSE |
| ATP5S | 10169 | 0.50394 | 0.614304 | 0.77597 | FALSE |
| MAFA | 13296 | 0.502471 | 0.615336 | 0.776898 | FALSE |
| HIF1A | 2656 | 0.502338 | 0.61543 | 0.776953 | FALSE |
| TM9SF4 | 3075 | 0.501858 | 0.615767 | 0.777099 | FALSE |
| NALCN | 7634 | 0.501809 | 0.615802 | 0.777099 | FALSE |
| FAM24B | 238 | 0.501808 | 0.615803 | 0.777099 | FALSE |
| CYP2W1 | 673 | 0.50175 | 0.615843 | 0.777099 | FALSE |
| UBA5 | 11694 | 0.50159 | 0.615956 | 0.777116 | FALSE |
| LINC01976 | 10948 | 0.501258 | 0.61619 | 0.777293 | FALSE |
| DDX46 | 3129 | 0.501249 | 0.616196 | 0.777293 | FALSE |
| NCKAP5 | 14705 | 0.500936 | 0.616416 | 0.777508 | FALSE |
| UNC13B | 12702 | 0.500808 | 0.616506 | 0.777559 | FALSE |
| CCDC97 | 9438 | 0.500731 | 0.61656 | 0.777565 | FALSE |
| KIF13B | 7066 | 0.500567 | 0.616676 | 0.777588 | FALSE |
| PLXNB1 | 1638 | 0.500563 | 0.616679 | 0.777588 | FALSE |
| SCOC-AS1 | 1850 | 0.500211 | 0.616927 | 0.777775 | FALSE |
| DCAF13 | 7717 | 0.500211 | 0.616927 | 0.777775 | FALSE |
| ENTPD3 | 7803 | 0.500091 | 0.617011 | 0.777819 | FALSE |
| BAZ2A | 6244 | 0.49994 | 0.617117 | 0.77789 | FALSE |
| FOXH1 | 14585 | 0.499342 | 0.617538 | 0.77823 | FALSE |
| LOC105372990 | 14174 | 0.499199 | 0.617639 | 0.77823 | FALSE |
| SUPT3H | 15122 | 0.49905 | 0.617744 | 0.77823 | FALSE |
| ZNF423 | 15484 | 0.498789 | 0.617928 | 0.778276 | FALSE |
| OLA1 | 15461 | 0.498728 | 0.617971 | 0.778276 | FALSE |
| NDUFA4L2 | 10220 | 0.497474 | 0.618855 | 0.779264 | FALSE |
| FUZ | 14457 | 0.497401 | 0.618906 | 0.779266 | FALSE |
| COPG1 | 1314 | 0.496981 | 0.619202 | 0.779513 | FALSE |
| ZNF526 | 2506 | 0.496836 | 0.619305 | 0.779579 | FALSE |
| FKBPL | 66 | 0.496534 | 0.619518 | 0.779721 | FALSE |
| PTGER2 | 12597 | 0.496043 | 0.619864 | 0.779814 | FALSE |
| PCGF3 | 3359 | 0.495808 | 0.62003 | 0.779872 | FALSE |
| MPPED2 | 13966 | 0.495587 | 0.620186 | 0.779872 | FALSE |
| DPH3P1 | 13498 | 0.495587 | 0.620186 | 0.779872 | FALSE |
| ZFYVE26 | 92 | 0.494354 | 0.621056 | 0.780778 | FALSE |
| UBE2F | 13988 | 0.493612 | 0.62158 | 0.781095 | FALSE |
| DCDC1 | 763 | 0.493577 | 0.621605 | 0.781095 | FALSE |
| MCM3AP | 4154 | 0.493572 | 0.621608 | 0.781095 | FALSE |
| PLD5 | 5079 | 0.492987 | 0.622022 | 0.781426 | FALSE |
| PABPC1 | 5106 | 0.492108 | 0.622643 | 0.781892 | FALSE |
| PTGES3 | 349 | 0.491269 | 0.623236 | 0.782466 | FALSE |
| P4HA1 | 660 | 0.490636 | 0.623684 | 0.782882 | FALSE |
| USP21 | 11234 | 0.490016 | 0.624123 | 0.783184 | FALSE |
| PRCC | 10823 | 0.489289 | 0.624637 | 0.783515 | FALSE |
| CASKIN2 | 1001 | 0.489169 | 0.624722 | 0.783559 | FALSE |
| STK31 | 3190 | 0.487914 | 0.625611 | 0.784422 | FALSE |
| TMEM240 | 3761 | 0.487616 | 0.625822 | 0.784624 | FALSE |
| WDR24 | 4781 | 0.4875 | 0.625904 | 0.784632 | FALSE |
| SUPT20H | 118 | 0.487465 | 0.625929 | 0.784632 | FALSE |
| RRBP1 | 6702 | 0.486991 | 0.626265 | 0.784927 | FALSE |
| MRPS14 | 1284 | 0.486894 | 0.626333 | 0.78495 | FALSE |
| ZC3H8 | 13754 | 0.486345 | 0.626723 | 0.785375 | FALSE |
| DCAF6 | 7199 | 0.485833 | 0.627086 | 0.78556 | FALSE |
| MAP7D2 | 13938 | 0.485782 | 0.627122 | 0.78556 | FALSE |
| FAM163B | 10281 | 0.485024 | 0.627659 | 0.786045 | FALSE |
| HEATR5A | 11071 | 0.484673 | 0.627908 | 0.786231 | FALSE |
| TCF3 | 13253 | 0.484565 | 0.627985 | 0.786264 | FALSE |
| JADE3 | 5924 | 0.484434 | 0.628078 | 0.786289 | FALSE |
| RMDN1 | 13393 | 0.484122 | 0.628299 | 0.786289 | FALSE |
| C6orf120 | 9333 | 0.48367 | 0.62862 | 0.786365 | FALSE |
| IFT43 | 10434 | 0.48348 | 0.628755 | 0.786365 | FALSE |
| HNRNPH3 | 151 | 0.483459 | 0.62877 | 0.786365 | FALSE |
| SLC24A5 | 3183 | 0.483146 | 0.628992 | 0.786465 | FALSE |
| WARS2 | 748 | 0.483139 | 0.628997 | 0.786465 | FALSE |
| CAVIN1 | 12404 | 0.483063 | 0.629051 | 0.786465 | FALSE |
| CDK10 | 2365 | 0.482817 | 0.629226 | 0.78661 | FALSE |
| SEMA3G | 13555 | 0.482758 | 0.629268 | 0.78661 | FALSE |
| LIFR-AS1 | 3527 | 0.482363 | 0.629548 | 0.786897 | FALSE |
| TRPT1 | 984 | 0.482051 | 0.62977 | 0.787085 | FALSE |
| TRMO | 12081 | 0.481515 | 0.630151 | 0.787416 | FALSE |
| NKAP | 3101 | 0.481495 | 0.630165 | 0.787416 | FALSE |
| ZNF724 | 8791 | 0.480954 | 0.630549 | 0.787834 | FALSE |
| TMEM144 | 10003 | 0.480576 | 0.630818 | 0.787988 | FALSE |
| HCFC1 | 6469 | 0.480568 | 0.630824 | 0.787988 | FALSE |
| KLK6 | 3708 | 0.480136 | 0.631131 | 0.788308 | FALSE |
| COQ6 | 15569 | 0.479797 | 0.631372 | 0.788483 | FALSE |
| GADD45B | 5488 | 0.479392 | 0.63166 | 0.788717 | FALSE |
| TIMM22 | 10022 | 0.479171 | 0.631817 | 0.788743 | FALSE |
| QARS | 1015 | 0.478529 | 0.632274 | 0.789105 | FALSE |
| LEPR | 1396 | 0.477912 | 0.632713 | 0.78948 | FALSE |
| ACOT11 | 9639 | 0.47753 | 0.632985 | 0.789678 | FALSE |
| IL10RA | 4569 | 0.477399 | 0.633078 | 0.789731 | FALSE |
| IFI27 | 12662 | 0.477226 | 0.633201 | 0.789822 | FALSE |
| SFXN3 | 9843 | 0.476788 | 0.633513 | 0.790027 | FALSE |
| ERCC2 | 13876 | 0.476752 | 0.633539 | 0.790027 | FALSE |
| RMDN3 | 809 | 0.476695 | 0.633579 | 0.790027 | FALSE |
| GJB1 | 11884 | 0.476609 | 0.633641 | 0.790027 | FALSE |
| FOLH1B | 3264 | 0.476498 | 0.63372 | 0.790027 | FALSE |
| CAPN1 | 10344 | 0.476097 | 0.634005 | 0.790242 | FALSE |
| CTRB2 | 1040 | 0.476043 | 0.634044 | 0.790242 | FALSE |
| IGSF3 | 15308 | 0.474546 | 0.635111 | 0.791206 | FALSE |
| URI1 | 527 | 0.474493 | 0.635148 | 0.791206 | FALSE |
| ZNF821 | 15456 | 0.474438 | 0.635188 | 0.791206 | FALSE |
| ZNF285 | 8206 | 0.473932 | 0.635548 | 0.791461 | FALSE |
| INPP5D | 10150 | 0.473639 | 0.635757 | 0.79162 | FALSE |
| TRIM16L | 14517 | 0.47335 | 0.635963 | 0.791655 | FALSE |
| TNNC1 | 6856 | 0.473187 | 0.63608 | 0.791707 | FALSE |
| APC | 3992 | 0.472397 | 0.636643 | 0.792184 | FALSE |
| CEACAM1 | 164 | 0.472365 | 0.636666 | 0.792184 | FALSE |
| LANCL3 | 11759 | 0.47082 | 0.637769 | 0.793178 | FALSE |
| SMTN | 4109 | 0.470302 | 0.638139 | 0.793512 | FALSE |
| SMS | 14227 | 0.470202 | 0.638211 | 0.793538 | FALSE |
| PPP2R2A | 9455 | 0.469539 | 0.638684 | 0.793788 | FALSE |
| RPF1 | 13169 | 0.469494 | 0.638717 | 0.793788 | FALSE |
| USP6NL | 4878 | 0.469086 | 0.639008 | 0.794087 | FALSE |
| USB1 | 8463 | 0.468823 | 0.639196 | 0.794137 | FALSE |
| ZNF676 | 6363 | 0.468817 | 0.6392 | 0.794137 | FALSE |
| XPO1 | 8445 | 0.468739 | 0.639256 | 0.794142 | FALSE |
| NUDCD3 | 1452 | 0.468415 | 0.639488 | 0.794241 | FALSE |
| AIFM2 | 3220 | 0.467761 | 0.639956 | 0.794759 | FALSE |
| FAM198A | 5828 | 0.46764 | 0.640042 | 0.794803 | FALSE |
| RGS17 | 4407 | 0.467517 | 0.64013 | 0.794849 | FALSE |
| NUDT16 | 6611 | 0.467297 | 0.640287 | 0.794982 | FALSE |
| HEBP2 | 13687 | 0.46706 | 0.640457 | 0.79512 | FALSE |
| LINC00526 | 13570 | 0.466216 | 0.641061 | 0.7955 | FALSE |
| GMEB2 | 6150 | 0.466143 | 0.641113 | 0.795501 | FALSE |
| PINX1 | 11965 | 0.465874 | 0.641306 | 0.795617 | FALSE |
| SMR3A | 9746 | 0.465847 | 0.641325 | 0.795617 | FALSE |
| PRR12 | 14686 | 0.465799 | 0.641359 | 0.795617 | FALSE |
| TTC8 | 6010 | 0.465262 | 0.641744 | 0.795968 | FALSE |
| FBXO24 | 654 | 0.464701 | 0.642146 | 0.79634 | FALSE |
| NLRX1 | 2794 | 0.463199 | 0.643222 | 0.797132 | FALSE |
| SERINC3 | 4842 | 0.463169 | 0.643243 | 0.797132 | FALSE |
| PIP5K1B | 4722 | 0.462427 | 0.643775 | 0.797468 | FALSE |
| CHKA | 13750 | 0.462364 | 0.64382 | 0.797468 | FALSE |
| TSC22D2 | 12249 | 0.462121 | 0.643995 | 0.797621 | FALSE |
| GLYR1 | 4440 | 0.461686 | 0.644307 | 0.797944 | FALSE |
| NAA20 | 13354 | 0.461328 | 0.644563 | 0.798068 | FALSE |
| GTPBP10 | 8969 | 0.461131 | 0.644705 | 0.798068 | FALSE |
| COMMD2 | 11808 | 0.461099 | 0.644728 | 0.798068 | FALSE |
| KRT71 | 13032 | 0.461096 | 0.64473 | 0.798068 | FALSE |
| PMS2 | 10603 | 0.460852 | 0.644905 | 0.798127 | FALSE |
| AP4S1 | 12863 | 0.460792 | 0.644948 | 0.798127 | FALSE |
| PEX13 | 8012 | 0.460768 | 0.644965 | 0.798127 | FALSE |
| RING1 | 3551 | 0.460544 | 0.645126 | 0.798188 | FALSE |
| MKRN2 | 2146 | 0.46025 | 0.645337 | 0.798334 | FALSE |
| PPA1 | 13382 | 0.460144 | 0.645413 | 0.798334 | FALSE |
| DNAJC9-AS1 | 3463 | 0.459632 | 0.64578 | 0.798693 | FALSE |
| TRAPPC12 | 7043 | 0.45871 | 0.646442 | 0.799309 | FALSE |
| C20orf194 | 3523 | 0.458645 | 0.646489 | 0.799309 | FALSE |
| FUS | 5360 | 0.458141 | 0.646851 | 0.799638 | FALSE |
| CALCOCO2 | 1065 | 0.458018 | 0.64694 | 0.799684 | FALSE |
| ETAA1 | 2972 | 0.457182 | 0.64754 | 0.800174 | FALSE |
| ZNF611 | 14638 | 0.456994 | 0.647675 | 0.800219 | FALSE |
| GAD2 | 6853 | 0.456988 | 0.64768 | 0.800219 | FALSE |
| TSC1 | 12031 | 0.456817 | 0.647803 | 0.800308 | FALSE |
| NSMCE3 | 4780 | 0.456715 | 0.647876 | 0.800335 | FALSE |
| H2AFV | 15105 | 0.456464 | 0.648056 | 0.800495 | FALSE |
| SYNJ2BP | 6895 | 0.456342 | 0.648144 | 0.80054 | FALSE |
| CASP16P | 6569 | 0.456251 | 0.648209 | 0.800558 | FALSE |
| SMC6 | 2680 | 0.45578 | 0.648548 | 0.800723 | FALSE |
| CCDC61 | 8015 | 0.455065 | 0.649062 | 0.801168 | FALSE |
| DPY19L1 | 3302 | 0.454945 | 0.649149 | 0.801211 | FALSE |
| ITPR2 | 5221 | 0.454828 | 0.649233 | 0.801252 | FALSE |
| SRSF10 | 4383 | 0.454737 | 0.649298 | 0.80127 | FALSE |
| KCNH3 | 2683 | 0.45411 | 0.64975 | 0.801637 | FALSE |
| WDR5 | 4347 | 0.453329 | 0.650312 | 0.80214 | FALSE |
| C2CD6 | 9067 | 0.453249 | 0.650369 | 0.802148 | FALSE |
| MIGA2 | 4346 | 0.453142 | 0.650446 | 0.80218 | FALSE |
| APOD | 2495 | 0.452255 | 0.651085 | 0.802694 | FALSE |
| BRD2 | 15055 | 0.452208 | 0.651119 | 0.802694 | FALSE |
| ETNK1 | 8735 | 0.452164 | 0.651151 | 0.802694 | FALSE |
| SUGT1P1 | 3838 | 0.452135 | 0.651172 | 0.802694 | FALSE |
| LAMA5 | 14673 | 0.45165 | 0.651521 | 0.803062 | FALSE |
| MED13 | 7331 | 0.451338 | 0.651746 | 0.803275 | FALSE |
| GRAMD2B | 13418 | 0.450489 | 0.652358 | 0.803627 | FALSE |
| ARHGAP17 | 6305 | 0.450389 | 0.65243 | 0.803627 | FALSE |
| CBLN4 | 14304 | 0.450378 | 0.652438 | 0.803627 | FALSE |
| ZNF665 | 9911 | 0.45027 | 0.652516 | 0.803654 | FALSE |
| SIK2 | 3356 | 0.450124 | 0.652621 | 0.803657 | FALSE |
| ENPP2 | 9504 | 0.449983 | 0.652723 | 0.803681 | FALSE |
| ARPC5L | 10630 | 0.449954 | 0.652744 | 0.803681 | FALSE |
| CHMP4C | 12889 | 0.449614 | 0.652989 | 0.803793 | FALSE |
| HNRNPCL1 | 8442 | 0.44938 | 0.653158 | 0.803938 | FALSE |
| RCC1 | 4264 | 0.448863 | 0.65353 | 0.804333 | FALSE |
| FOXL2 | 5251 | 0.448754 | 0.653609 | 0.804367 | FALSE |
| MLYCD | 3300 | 0.4486 | 0.65372 | 0.804415 | FALSE |
| SGIP1 | 1977 | 0.448557 | 0.653751 | 0.804415 | FALSE |
| ZNF574 | 12281 | 0.448293 | 0.653942 | 0.804523 | FALSE |
| HIST1H3F | 8999 | 0.448131 | 0.654059 | 0.804603 | FALSE |
| PDZD11 | 13224 | 0.448031 | 0.654131 | 0.804629 | FALSE |
| LOC100132356 | 93 | 0.447631 | 0.65442 | 0.804846 | FALSE |
| NAB2 | 14466 | 0.447437 | 0.65456 | 0.804846 | FALSE |
| DHODH | 6894 | 0.44739 | 0.654593 | 0.804846 | FALSE |
| PPRC1 | 12268 | 0.447358 | 0.654617 | 0.804846 | FALSE |
| ZNF723 | 5777 | 0.446548 | 0.655201 | 0.805411 | FALSE |
| BROX | 12642 | 0.446508 | 0.65523 | 0.805411 | FALSE |
| VPS9D1 | 4059 | 0.446235 | 0.655428 | 0.805488 | FALSE |
| FRMD4A | 1964 | 0.445514 | 0.655948 | 0.805745 | FALSE |
| PANK4 | 13851 | 0.444093 | 0.656975 | 0.806694 | FALSE |
| NKG7 | 14637 | 0.443745 | 0.657227 | 0.806798 | FALSE |
| ZSCAN32 | 6312 | 0.443732 | 0.657236 | 0.806798 | FALSE |
| MRPS17 | 3110 | 0.443064 | 0.657719 | 0.807138 | FALSE |
| EI24 | 15378 | 0.442685 | 0.657994 | 0.807347 | FALSE |
| DOCK3 | 1626 | 0.442568 | 0.658078 | 0.807388 | FALSE |
| OMA1 | 8895 | 0.442454 | 0.658161 | 0.807391 | FALSE |
| OAT | 15495 | 0.442422 | 0.658184 | 0.807391 | FALSE |
| MTA3 | 1353 | 0.441953 | 0.658523 | 0.807734 | FALSE |
| CPEB2 | 13895 | 0.441892 | 0.658567 | 0.807734 | FALSE |
| TMEM38B | 7532 | 0.441377 | 0.65894 | 0.807959 | FALSE |
| PRELID3B | 10625 | 0.44078 | 0.659372 | 0.808405 | FALSE |
| MIER1 | 15537 | 0.440267 | 0.659744 | 0.808568 | FALSE |
| TMED4 | 5396 | 0.440225 | 0.659774 | 0.808568 | FALSE |
| SPTBN5 | 2968 | 0.440212 | 0.659784 | 0.808568 | FALSE |
| ELP4 | 11493 | 0.440029 | 0.659916 | 0.808568 | FALSE |
| NDE1 | 12080 | 0.439769 | 0.660104 | 0.808731 | FALSE |
| RPRD1B | 14622 | 0.438521 | 0.661009 | 0.809585 | FALSE |
| NASP | 13961 | 0.438141 | 0.661284 | 0.809796 | FALSE |
| RECQL5 | 9062 | 0.437846 | 0.661498 | 0.809874 | FALSE |
| ASXL1 | 12882 | 0.437707 | 0.661599 | 0.809874 | FALSE |
| CPSF3 | 11237 | 0.437433 | 0.661797 | 0.810044 | FALSE |
| RIMS3 | 15294 | 0.437276 | 0.661911 | 0.81012 | FALSE |
| PDPK1 | 9456 | 0.437019 | 0.662098 | 0.810284 | FALSE |
| PRR14 | 5816 | 0.43668 | 0.662343 | 0.810425 | FALSE |
| RPP30 | 1939 | 0.436296 | 0.662622 | 0.810609 | FALSE |
| TMEM235 | 12622 | 0.435662 | 0.663082 | 0.811045 | FALSE |
| SOX13 | 3817 | 0.43468 | 0.663795 | 0.811747 | FALSE |
| IP6K1 | 3653 | 0.434442 | 0.663968 | 0.811747 | FALSE |
| PCBD2 | 9622 | 0.434012 | 0.66428 | 0.811885 | FALSE |
| BASP1-AS1 | 15581 | 0.43393 | 0.664339 | 0.811885 | FALSE |
| EYA1 | 14266 | 0.433912 | 0.664352 | 0.811885 | FALSE |
| CXXC1 | 11752 | 0.433606 | 0.664575 | 0.81194 | FALSE |
| NELFB | 5186 | 0.433581 | 0.664593 | 0.81194 | FALSE |
| MNAT1 | 10321 | 0.433046 | 0.664981 | 0.812351 | FALSE |
| DACT2 | 1414 | 0.432633 | 0.665281 | 0.81259 | FALSE |
| PRKRIP1 | 15182 | 0.432149 | 0.665633 | 0.812893 | FALSE |
| ACTBL2 | 7271 | 0.431985 | 0.665752 | 0.812975 | FALSE |
| USP48 | 5537 | 0.431763 | 0.665914 | 0.813109 | FALSE |
| GTF3A | 3484 | 0.431083 | 0.666408 | 0.813267 | FALSE |
| CSNK1A1P1 | 1219 | 0.429011 | 0.667915 | 0.814216 | FALSE |
| ANGEL2 | 7977 | 0.428941 | 0.667966 | 0.814216 | FALSE |
| SAA2 | 146 | 0.428543 | 0.668256 | 0.814378 | FALSE |
| HIST1H3G | 12241 | 0.427954 | 0.668685 | 0.814647 | FALSE |
| SHTN1 | 15459 | 0.427734 | 0.668845 | 0.814778 | FALSE |
| EMILIN2 | 7415 | 0.427275 | 0.669179 | 0.814852 | FALSE |
| SRC | 1957 | 0.426919 | 0.669438 | 0.814993 | FALSE |
| TEX29 | 7198 | 0.426845 | 0.669492 | 0.814995 | FALSE |
| FAM218A | 3349 | 0.426765 | 0.669551 | 0.815003 | FALSE |
| LOC202181 | 10267 | 0.426454 | 0.669777 | 0.815061 | FALSE |
| SCARNA17 | 12416 | 0.426382 | 0.66983 | 0.815061 | FALSE |
| GNAZ | 9296 | 0.42636 | 0.669846 | 0.815061 | FALSE |
| IQGAP1 | 13189 | 0.426342 | 0.669859 | 0.815061 | FALSE |
| POLL | 917 | 0.425428 | 0.670525 | 0.815554 | FALSE |
| JPX | 14138 | 0.425158 | 0.670721 | 0.815666 | FALSE |
| OXA1L | 4724 | 0.424636 | 0.671102 | 0.815812 | FALSE |
| DEGS2 | 6171 | 0.424636 | 0.671102 | 0.815812 | FALSE |
| PIP4K2A | 15613 | 0.424389 | 0.671282 | 0.815899 | FALSE |
| CCDC9B | 10357 | 0.424274 | 0.671366 | 0.815899 | FALSE |
| TMEM168 | 3922 | 0.424251 | 0.671383 | 0.815899 | FALSE |
| LZTS2 | 11597 | 0.423588 | 0.671866 | 0.816325 | FALSE |
| HDAC2 | 14648 | 0.423544 | 0.671898 | 0.816325 | FALSE |
| RND3 | 373 | 0.423484 | 0.671942 | 0.816325 | FALSE |
| PANK2 | 13037 | 0.423336 | 0.67205 | 0.816335 | FALSE |
| SPP1 | 10854 | 0.42314 | 0.672193 | 0.816376 | FALSE |
| PRAC1 | 12610 | 0.422925 | 0.67235 | 0.816503 | FALSE |
| PDE4D | 269 | 0.422818 | 0.672428 | 0.816535 | FALSE |
| ZFYVE1 | 3982 | 0.422495 | 0.672664 | 0.816611 | FALSE |
| CTAGE7P | 11055 | 0.422426 | 0.672714 | 0.816611 | FALSE |
| KRTAP19-1 | 4063 | 0.422216 | 0.672867 | 0.816688 | FALSE |
| C2orf76 | 3916 | 0.421923 | 0.673081 | 0.81682 | FALSE |
| NEUROD1 | 7152 | 0.421648 | 0.673282 | 0.817 | FALSE |
| LARP6 | 12252 | 0.42141 | 0.673456 | 0.817148 | FALSE |
| THEMIS2 | 4413 | 0.420673 | 0.673994 | 0.817674 | FALSE |
| BMPR1B | 1561 | 0.4205 | 0.67412 | 0.817692 | FALSE |
| ZNF568 | 12778 | 0.420438 | 0.674166 | 0.817692 | FALSE |
| RPP25 | 13000 | 0.419056 | 0.675175 | 0.818535 | FALSE |
| FHL5 | 6482 | 0.418927 | 0.675269 | 0.818586 | FALSE |
| BICC1 | 9661 | 0.41836 | 0.675684 | 0.818835 | FALSE |
| CCM2 | 9318 | 0.418288 | 0.675737 | 0.818835 | FALSE |
| DNAJC7 | 1306 | 0.417484 | 0.676324 | 0.819473 | FALSE |
| ACD | 444 | 0.417184 | 0.676544 | 0.819478 | FALSE |
| HOXD9 | 9670 | 0.41715 | 0.676569 | 0.819478 | FALSE |
| TM7SF3 | 2468 | 0.417085 | 0.676616 | 0.819478 | FALSE |
| KLHL5 | 1235 | 0.41706 | 0.676635 | 0.819478 | FALSE |
| DND1 | 2396 | 0.416586 | 0.676981 | 0.819665 | FALSE |
| GNPTG | 10460 | 0.416496 | 0.677047 | 0.819665 | FALSE |
| NCALD | 6915 | 0.416419 | 0.677103 | 0.819665 | FALSE |
| DEFB118 | 4330 | 0.416014 | 0.6774 | 0.819897 | FALSE |
| LTV1 | 2072 | 0.415782 | 0.67757 | 0.820039 | FALSE |
| DDIT3 | 11885 | 0.415562 | 0.677731 | 0.82017 | FALSE |
| LOC100129138 | 6294 | 0.415297 | 0.677924 | 0.820278 | FALSE |
| LINC00431 | 9149 | 0.414851 | 0.678251 | 0.820425 | FALSE |
| LOC284454 | 9279 | 0.414845 | 0.678255 | 0.820425 | FALSE |
| FLNB | 2178 | 0.414462 | 0.678536 | 0.8207 | FALSE |
| MINDY2 | 2759 | 0.41436 | 0.67861 | 0.820727 | FALSE |
| COASY | 15344 | 0.414121 | 0.678785 | 0.820791 | FALSE |
| CCDC113 | 15370 | 0.413977 | 0.678891 | 0.820812 | FALSE |
| KANSL1L | 14413 | 0.413735 | 0.679068 | 0.8209 | FALSE |
| FAXDC2 | 4476 | 0.413447 | 0.679279 | 0.821028 | FALSE |
| SETMAR | 3262 | 0.413117 | 0.679521 | 0.821201 | FALSE |
| REM2 | 7391 | 0.413036 | 0.67958 | 0.821201 | FALSE |
| SPDYE7P | 7460 | 0.412927 | 0.67966 | 0.821226 | FALSE |
| GTPBP2 | 10363 | 0.412714 | 0.679816 | 0.821226 | FALSE |
| L3MBTL3 | 15270 | 0.412603 | 0.679898 | 0.821226 | FALSE |
| CRB3 | 6946 | 0.412578 | 0.679916 | 0.821226 | FALSE |
| TRPC5 | 15588 | 0.411947 | 0.680378 | 0.821594 | FALSE |
| PPIE | 6948 | 0.41185 | 0.680449 | 0.821616 | FALSE |
| ANO2 | 11280 | 0.411576 | 0.68065 | 0.821795 | FALSE |
| ZNF726 | 3841 | 0.410712 | 0.681284 | 0.822306 | FALSE |
| CLIC4 | 15598 | 0.410598 | 0.681367 | 0.822313 | FALSE |
| TP73-AS1 | 11130 | 0.40955 | 0.682136 | 0.822932 | FALSE |
| LOC646938 | 15472 | 0.40947 | 0.682195 | 0.822932 | FALSE |
| MICU2 | 3377 | 0.409359 | 0.682276 | 0.822932 | FALSE |
| GAB2 | 3963 | 0.408682 | 0.682773 | 0.823339 | FALSE |
| UBE2E1 | 14847 | 0.408612 | 0.682824 | 0.823339 | FALSE |
| RAX2 | 4011 | 0.40836 | 0.683009 | 0.823499 | FALSE |
| ZNF571 | 7656 | 0.407684 | 0.683506 | 0.82397 | FALSE |
| TMEM170A | 8103 | 0.406857 | 0.684113 | 0.824414 | FALSE |
| CBX3 | 15627 | 0.406696 | 0.684231 | 0.824414 | FALSE |
| MSI2 | 1153 | 0.40668 | 0.684243 | 0.824414 | FALSE |
| S100A8 | 5586 | 0.406333 | 0.684498 | 0.824515 | FALSE |
| NUDT22 | 14802 | 0.406239 | 0.684567 | 0.824515 | FALSE |
| NUCKS1 | 14918 | 0.406181 | 0.68461 | 0.824515 | FALSE |
| ERI1 | 6571 | 0.406097 | 0.684671 | 0.824515 | FALSE |
| SOX1 | 4164 | 0.405991 | 0.684749 | 0.824515 | FALSE |
| GIPC1 | 10704 | 0.405784 | 0.684901 | 0.824585 | FALSE |
| AVPI1 | 1086 | 0.405769 | 0.684912 | 0.824585 | FALSE |
| ZNF382 | 11241 | 0.405044 | 0.685445 | 0.824938 | FALSE |
| AQP6 | 12671 | 0.404795 | 0.685628 | 0.824938 | FALSE |
| UBE2L3 | 15552 | 0.404384 | 0.68593 | 0.825175 | FALSE |
| MYADML2 | 8688 | 0.404006 | 0.686208 | 0.825404 | FALSE |
| BRI3BP | 4671 | 0.403861 | 0.686315 | 0.825447 | FALSE |
| EIF3A | 9810 | 0.403177 | 0.686818 | 0.825798 | FALSE |
| RETREG3 | 14247 | 0.402954 | 0.686982 | 0.825868 | FALSE |
| YBX1 | 11358 | 0.402716 | 0.687157 | 0.825999 | FALSE |
| PRSS55 | 7267 | 0.402663 | 0.687196 | 0.825999 | FALSE |
| CLN8 | 3938 | 0.402316 | 0.687451 | 0.826206 | FALSE |
| DUSP5P1 | 13263 | 0.402285 | 0.687474 | 0.826206 | FALSE |
| ANKRD36BP2 | 9025 | 0.401803 | 0.687829 | 0.826505 | FALSE |
| NEK7 | 2650 | 0.401484 | 0.688064 | 0.826597 | FALSE |
| IGFBP2 | 13379 | 0.40099 | 0.688427 | 0.82695 | FALSE |
| NEFL | 4653 | 0.400929 | 0.688472 | 0.82695 | FALSE |
| MRPL50 | 4850 | 0.400653 | 0.688676 | 0.82695 | FALSE |
| PRDM8 | 2357 | 0.400435 | 0.688836 | 0.82708 | FALSE |
| HTT | 13313 | 0.400211 | 0.689001 | 0.827189 | FALSE |
| ISCA1 | 6021 | 0.400167 | 0.689034 | 0.827189 | FALSE |
| AGPS | 13917 | 0.399983 | 0.689169 | 0.827225 | FALSE |
| SERPINH1 | 1885 | 0.399549 | 0.689489 | 0.827301 | FALSE |
| PACRG-AS3 | 11519 | 0.399512 | 0.689516 | 0.827301 | FALSE |
| CHADL | 67 | 0.399485 | 0.689536 | 0.827301 | FALSE |
| ATG14 | 5025 | 0.399466 | 0.68955 | 0.827301 | FALSE |
| HS6ST1 | 484 | 0.399326 | 0.689653 | 0.827301 | FALSE |
| KRTAP19-8 | 15492 | 0.398824 | 0.690023 | 0.827497 | FALSE |
| PRKAA1 | 13652 | 0.398819 | 0.690027 | 0.827497 | FALSE |
| THEM6 | 8118 | 0.398672 | 0.690135 | 0.827558 | FALSE |
| CACNB1 | 15457 | 0.398284 | 0.690421 | 0.827715 | FALSE |
| SNX29 | 4174 | 0.398162 | 0.690511 | 0.827755 | FALSE |
| MTA2 | 10852 | 0.397848 | 0.690742 | 0.8279 | FALSE |
| SLC5A11 | 10119 | 0.397359 | 0.691103 | 0.827976 | FALSE |
| SERINC5 | 11799 | 0.396699 | 0.691589 | 0.828413 | FALSE |
| SMIM20 | 5918 | 0.39643 | 0.691788 | 0.828587 | FALSE |
| CENPBD1P1 | 15267 | 0.396228 | 0.691937 | 0.828702 | FALSE |
| TMLHE | 15542 | 0.396109 | 0.692025 | 0.828705 | FALSE |
| SEPT7P2 | 1147 | 0.396081 | 0.692045 | 0.828705 | FALSE |
| ITPR3 | 14018 | 0.395806 | 0.692248 | 0.828821 | FALSE |
| BMP1 | 12696 | 0.394802 | 0.692989 | 0.829327 | FALSE |
| HIST1H2AE | 11662 | 0.394388 | 0.693295 | 0.829502 | FALSE |
| GHRHR | 12187 | 0.394148 | 0.693472 | 0.829651 | FALSE |
| FAM114A1 | 5237 | 0.393943 | 0.693623 | 0.829768 | FALSE |
| PCSK2 | 14189 | 0.392864 | 0.69442 | 0.83034 | FALSE |
| EPOR | 15134 | 0.392626 | 0.694596 | 0.830443 | FALSE |
| RANBP2 | 7044 | 0.392604 | 0.694612 | 0.830443 | FALSE |
| GAN | 4737 | 0.392414 | 0.694752 | 0.830547 | FALSE |
| FBXL3 | 13509 | 0.391836 | 0.695179 | 0.830676 | FALSE |
| KANK2 | 14067 | 0.391645 | 0.695321 | 0.830737 | FALSE |
| BCL2L10 | 11849 | 0.391295 | 0.695579 | 0.830964 | FALSE |
| LDLRAD4 | 7937 | 0.39051 | 0.696159 | 0.831593 | FALSE |
| POU5F1P3 | 7322 | 0.390387 | 0.69625 | 0.831638 | FALSE |
| PNKD | 14829 | 0.389979 | 0.696552 | 0.831935 | FALSE |
| DDX39A | 6335 | 0.389455 | 0.69694 | 0.832144 | FALSE |
| TPP1 | 7457 | 0.389301 | 0.697053 | 0.832177 | FALSE |
| EGR2 | 3975 | 0.388818 | 0.697411 | 0.832452 | FALSE |
| TMEM114 | 10590 | 0.388512 | 0.697637 | 0.832595 | FALSE |
| IRS2 | 11209 | 0.387173 | 0.698628 | 0.833343 | FALSE |
| HMG20B | 582 | 0.385715 | 0.699708 | 0.834175 | FALSE |
| ANKRD44 | 6648 | 0.385318 | 0.700002 | 0.834334 | FALSE |
| UPK3B | 14193 | 0.384886 | 0.700322 | 0.834588 | FALSE |
| MIXL1 | 750 | 0.384204 | 0.700827 | 0.834944 | FALSE |
| MRPL35 | 7489 | 0.383193 | 0.701577 | 0.835702 | FALSE |
| ZNF69 | 9819 | 0.382632 | 0.701993 | 0.835754 | FALSE |
| ZBTB49 | 3516 | 0.382578 | 0.702033 | 0.835754 | FALSE |
| B3GALT5-AS1 | 10033 | 0.382572 | 0.702037 | 0.835754 | FALSE |
| HIST1H4H | 7480 | 0.382527 | 0.702071 | 0.835754 | FALSE |
| FBXO48 | 3907 | 0.382503 | 0.702088 | 0.835754 | FALSE |
| ZBTB20 | 1084 | 0.382485 | 0.702102 | 0.835754 | FALSE |
| AFAP1L1 | 2342 | 0.381957 | 0.702493 | 0.836001 | FALSE |
| NSMF | 8302 | 0.381905 | 0.702532 | 0.836001 | FALSE |
| DNMT3B | 5649 | 0.381845 | 0.702576 | 0.836001 | FALSE |
| EIF2B3 | 6867 | 0.380629 | 0.703479 | 0.836756 | FALSE |
| DZIP1 | 5281 | 0.380386 | 0.703659 | 0.836907 | FALSE |
| GPR139 | 8563 | 0.380303 | 0.703721 | 0.836916 | FALSE |
| RPN1 | 11764 | 0.380093 | 0.703876 | 0.836991 | FALSE |
| SDR39U1 | 7972 | 0.380074 | 0.70389 | 0.836991 | FALSE |
| GBF1 | 13822 | 0.379989 | 0.703954 | 0.837002 | FALSE |
| IL10RB | 4180 | 0.379376 | 0.704409 | 0.837352 | FALSE |
| DNAJC10 | 8474 | 0.37929 | 0.704473 | 0.837365 | FALSE |
| ZNF668 | 4290 | 0.378342 | 0.705177 | 0.83801 | FALSE |
| ZNF713 | 7524 | 0.377402 | 0.705875 | 0.83871 | FALSE |
| PEX6 | 4352 | 0.377352 | 0.705912 | 0.83871 | FALSE |
| TIGD1 | 3399 | 0.377171 | 0.706047 | 0.83871 | FALSE |
| TERT | 14312 | 0.377124 | 0.706081 | 0.83871 | FALSE |
| UGT8 | 13136 | 0.376982 | 0.706187 | 0.83871 | FALSE |
| BACE2 | 3069 | 0.375628 | 0.707193 | 0.839562 | FALSE |
| IPO9 | 3639 | 0.375573 | 0.707234 | 0.839562 | FALSE |
| ZRSR2 | 3456 | 0.374931 | 0.707712 | 0.839874 | FALSE |
| ANKDD1A | 12956 | 0.373551 | 0.708738 | 0.840766 | FALSE |
| PLCD1 | 15362 | 0.373423 | 0.708834 | 0.840766 | FALSE |
| SLC6A3 | 1847 | 0.373346 | 0.708891 | 0.840766 | FALSE |
| TEKT5 | 14963 | 0.373342 | 0.708894 | 0.840766 | FALSE |
| CDC14B | 3550 | 0.372058 | 0.70985 | 0.841601 | FALSE |
| DDX58 | 3064 | 0.371955 | 0.709926 | 0.841608 | FALSE |
| EGR3 | 12715 | 0.371843 | 0.71001 | 0.841643 | FALSE |
| AP1B1 | 12977 | 0.371315 | 0.710403 | 0.842015 | FALSE |
| ASAP1-IT2 | 8960 | 0.371276 | 0.710432 | 0.842015 | FALSE |
| CCSAP | 8416 | 0.370799 | 0.710787 | 0.842373 | FALSE |
| PPP1R9B | 11140 | 0.370284 | 0.711171 | 0.842763 | FALSE |
| DNAJB14 | 12260 | 0.369718 | 0.711593 | 0.843179 | FALSE |
| C1orf159 | 8900 | 0.36963 | 0.711658 | 0.843179 | FALSE |
| ANKRD36B | 1257 | 0.368906 | 0.712198 | 0.843533 | FALSE |
| SGCB | 13109 | 0.367676 | 0.713115 | 0.844137 | FALSE |
| ITGAE | 10436 | 0.367642 | 0.71314 | 0.844137 | FALSE |
| GPRC5B | 10605 | 0.367206 | 0.713465 | 0.844383 | FALSE |
| ABT1 | 2003 | 0.366869 | 0.713717 | 0.844383 | FALSE |
| CD109 | 5755 | 0.366675 | 0.713861 | 0.844383 | FALSE |
| TOR1B | 4588 | 0.366629 | 0.713896 | 0.844383 | FALSE |
| IGFL3 | 8585 | 0.366611 | 0.713909 | 0.844383 | FALSE |
| TMEM51 | 10786 | 0.366506 | 0.713988 | 0.844383 | FALSE |
| ACO1 | 7915 | 0.366414 | 0.714056 | 0.844383 | FALSE |
| IQCH | 5815 | 0.366396 | 0.71407 | 0.844383 | FALSE |
| EGFR-AS1 | 4943 | 0.366129 | 0.714269 | 0.844514 | FALSE |
| F8A1 | 3896 | 0.364879 | 0.715202 | 0.845042 | FALSE |
| AGT | 3385 | 0.364382 | 0.715573 | 0.845325 | FALSE |
| PBX2 | 2079 | 0.36424 | 0.715679 | 0.84535 | FALSE |
| ADAMTS1 | 10369 | 0.363503 | 0.716229 | 0.84564 | FALSE |
| CCDC71 | 10812 | 0.363444 | 0.716273 | 0.84564 | FALSE |
| PLEKHA3 | 13285 | 0.363422 | 0.71629 | 0.84564 | FALSE |
| SYT7 | 10247 | 0.363404 | 0.716303 | 0.84564 | FALSE |
| BCAP31 | 6761 | 0.362967 | 0.71663 | 0.845896 | FALSE |
| CASC15 | 3379 | 0.362325 | 0.717109 | 0.846279 | FALSE |
| SETDB2 | 7385 | 0.362255 | 0.717161 | 0.846279 | FALSE |
| ERN2 | 14365 | 0.362245 | 0.717169 | 0.846279 | FALSE |
| BTBD6 | 13974 | 0.362015 | 0.717341 | 0.846418 | FALSE |
| RC3H1 | 7820 | 0.360745 | 0.71829 | 0.847282 | FALSE |
| VPS16 | 10978 | 0.360133 | 0.718748 | 0.847638 | FALSE |
| CMTM5 | 6790 | 0.359821 | 0.718981 | 0.847718 | FALSE |
| ZNF575 | 4813 | 0.359795 | 0.719 | 0.847718 | FALSE |
| BAALC | 8363 | 0.359743 | 0.719039 | 0.847718 | FALSE |
| MYSM1 | 590 | 0.359159 | 0.719476 | 0.84792 | FALSE |
| ELK3 | 12516 | 0.359081 | 0.719534 | 0.84792 | FALSE |
| DNAJB11 | 13467 | 0.359079 | 0.719536 | 0.84792 | FALSE |
| PLAUR | 9568 | 0.358347 | 0.720084 | 0.848213 | FALSE |
| IRF9 | 13421 | 0.358312 | 0.72011 | 0.848213 | FALSE |
| RAP2C | 1873 | 0.35757 | 0.720665 | 0.848739 | FALSE |
| GRM4 | 2946 | 0.357472 | 0.720738 | 0.848761 | FALSE |
| LPAR5 | 5885 | 0.357007 | 0.721087 | 0.849043 | FALSE |
| LARP7 | 6369 | 0.356663 | 0.721344 | 0.849282 | FALSE |
| PCYT1A | 55 | 0.356531 | 0.721443 | 0.849335 | FALSE |
| ZNF207 | 129 | 0.355805 | 0.721987 | 0.849591 | FALSE |
| FBXO8 | 10808 | 0.35553 | 0.722193 | 0.849769 | FALSE |
| SLC4A2 | 6638 | 0.355349 | 0.722328 | 0.849865 | FALSE |
| C1orf189 | 11360 | 0.355159 | 0.72247 | 0.849968 | FALSE |
| OLIG1 | 3362 | 0.354882 | 0.722678 | 0.850123 | FALSE |
| FAM96A | 12813 | 0.354772 | 0.72276 | 0.850123 | FALSE |
| SPG21 | 8265 | 0.354766 | 0.722765 | 0.850123 | FALSE |
| ZNF346 | 7592 | 0.354433 | 0.723014 | 0.850231 | FALSE |
| ZNF155 | 2057 | 0.354426 | 0.72302 | 0.850231 | FALSE |
| PCSK6 | 267 | 0.35423 | 0.723167 | 0.850339 | FALSE |
| ENTPD1-AS1 | 15445 | 0.352098 | 0.724765 | 0.851517 | FALSE |
| NEMP1 | 12500 | 0.35195 | 0.724876 | 0.851517 | FALSE |
| SETD5 | 13593 | 0.351751 | 0.725025 | 0.851624 | FALSE |
| TP53AIP1 | 5712 | 0.351656 | 0.725096 | 0.851624 | FALSE |
| RPGRIP1L | 2089 | 0.350939 | 0.725634 | 0.851876 | FALSE |
| SMCR8 | 8712 | 0.350215 | 0.726177 | 0.852341 | FALSE |
| DUSP16 | 5330 | 0.350078 | 0.72628 | 0.852395 | FALSE |
| ZNF131 | 15202 | 0.349838 | 0.72646 | 0.852417 | FALSE |
| TUBGCP6 | 1339 | 0.349407 | 0.726784 | 0.852733 | FALSE |
| CASP7 | 12274 | 0.348814 | 0.727229 | 0.852999 | FALSE |
| AVEN | 7167 | 0.348486 | 0.727475 | 0.853224 | FALSE |
| CRABP2 | 1751 | 0.348352 | 0.727576 | 0.853278 | FALSE |
| ATRN | 8694 | 0.348234 | 0.727664 | 0.853318 | FALSE |
| KIF21A | 4257 | 0.348079 | 0.727781 | 0.853326 | FALSE |
| TDRD7 | 13523 | 0.34739 | 0.728298 | 0.853741 | FALSE |
| MTFR1L | 2605 | 0.347261 | 0.728395 | 0.85379 | FALSE |
| RIPOR1 | 13334 | 0.346279 | 0.729133 | 0.854591 | FALSE |
| SEC14L1P1 | 9233 | 0.345893 | 0.729423 | 0.854867 | FALSE |
| HOXB3 | 6868 | 0.345737 | 0.72954 | 0.85494 | FALSE |
| ZNF681 | 8148 | 0.344739 | 0.730291 | 0.855538 | FALSE |
| EEF1AKMT3 | 3149 | 0.344637 | 0.730367 | 0.855538 | FALSE |
| POMP | 7082 | 0.343544 | 0.731189 | 0.856231 | FALSE |
| PRADC1 | 11173 | 0.343003 | 0.731596 | 0.856515 | FALSE |
| SLC17A9 | 15024 | 0.342119 | 0.732261 | 0.857229 | FALSE |
| RASGEF1B | 335 | 0.341706 | 0.732572 | 0.857465 | FALSE |
| PLPP6 | 4393 | 0.341535 | 0.732701 | 0.857487 | FALSE |
| ZNF582 | 10507 | 0.340456 | 0.733513 | 0.857988 | FALSE |
| RCOR1 | 6147 | 0.339956 | 0.73389 | 0.8583 | FALSE |
| SNX4 | 336 | 0.338614 | 0.734901 | 0.859014 | FALSE |
| ELMOD2 | 9225 | 0.338607 | 0.734906 | 0.859014 | FALSE |
| ENPP7P13 | 3869 | 0.338512 | 0.734977 | 0.859014 | FALSE |
| OXR1 | 7985 | 0.337828 | 0.735493 | 0.859468 | FALSE |
| DUSP10 | 10155 | 0.337617 | 0.735652 | 0.859589 | FALSE |
| NPAS1 | 2645 | 0.337261 | 0.73592 | 0.85971 | FALSE |
| SRGAP2B | 6220 | 0.336643 | 0.736386 | 0.859933 | FALSE |
| SMAP2 | 11628 | 0.336561 | 0.736448 | 0.859941 | FALSE |
| KCNS3 | 12133 | 0.336317 | 0.736632 | 0.860033 | FALSE |
| XAB2 | 6118 | 0.33631 | 0.736637 | 0.860033 | FALSE |
| HNRNPH2 | 10691 | 0.336194 | 0.736725 | 0.860069 | FALSE |
| PLEKHA8P1 | 7266 | 0.336124 | 0.736777 | 0.860069 | FALSE |
| EMC10 | 3214 | 0.335988 | 0.73688 | 0.860124 | FALSE |
| VPS52 | 584 | 0.33585 | 0.736984 | 0.860181 | FALSE |
| LMBR1L | 14489 | 0.335529 | 0.737226 | 0.860271 | FALSE |
| SCYL2 | 1849 | 0.33524 | 0.737444 | 0.860397 | FALSE |
| FRY | 14170 | 0.33513 | 0.737527 | 0.86043 | FALSE |
| SGMS1 | 8031 | 0.334747 | 0.737816 | 0.860559 | FALSE |
| ENTPD7 | 4629 | 0.334429 | 0.738056 | 0.860661 | FALSE |
| TRIM8 | 12986 | 0.332857 | 0.739242 | 0.861681 | FALSE |
| LENG1 | 10827 | 0.332713 | 0.739351 | 0.861681 | FALSE |
| BLOC1S3 | 11546 | 0.332652 | 0.739397 | 0.861681 | FALSE |
| DEFB135 | 6779 | 0.331784 | 0.740052 | 0.862154 | FALSE |
| ANKRD24 | 10975 | 0.331249 | 0.740456 | 0.862367 | FALSE |
| PPP1R8 | 12729 | 0.330762 | 0.740824 | 0.862603 | FALSE |
| FOXP1 | 5626 | 0.330531 | 0.740999 | 0.862742 | FALSE |
| CDH23 | 4082 | 0.330212 | 0.74124 | 0.862958 | FALSE |
| LNPEP | 9330 | 0.33009 | 0.741332 | 0.863001 | FALSE |
| P2RY4 | 5064 | 0.32976 | 0.741581 | 0.863163 | FALSE |
| FAM89B | 10604 | 0.329304 | 0.741926 | 0.863286 | FALSE |
| FAAP100 | 9641 | 0.329036 | 0.742128 | 0.863286 | FALSE |
| FBXL22 | 8310 | 0.328891 | 0.742238 | 0.863295 | FALSE |
| RAD9A | 9066 | 0.328628 | 0.742437 | 0.863295 | FALSE |
| TP73 | 3492 | 0.328499 | 0.742534 | 0.863295 | FALSE |
| ADAM17 | 6137 | 0.328485 | 0.742545 | 0.863295 | FALSE |
| MCM5 | 7219 | 0.328363 | 0.742637 | 0.863295 | FALSE |
| TYK2 | 14891 | 0.328331 | 0.742661 | 0.863295 | FALSE |
| LTBP3 | 1822 | 0.328221 | 0.742745 | 0.863295 | FALSE |
| ERGIC1 | 8762 | 0.32816 | 0.742791 | 0.863295 | FALSE |
| MYOG | 9526 | 0.328113 | 0.742826 | 0.863295 | FALSE |
| INTS13 | 13950 | 0.328074 | 0.742856 | 0.863295 | FALSE |
| FJX1 | 9282 | 0.327929 | 0.742965 | 0.863295 | FALSE |
| IGFLR1 | 5310 | 0.327514 | 0.743279 | 0.863531 | FALSE |
| ERVK13-1 | 1565 | 0.327223 | 0.743499 | 0.863531 | FALSE |
| ATXN1L | 8856 | 0.327116 | 0.74358 | 0.863531 | FALSE |
| ITGA3 | 9450 | 0.327024 | 0.74365 | 0.863531 | FALSE |
| MIR100HG | 13038 | 0.327014 | 0.743657 | 0.863531 | FALSE |
| PELI2 | 13055 | 0.326726 | 0.743875 | 0.863531 | FALSE |
| DMXL1 | 6740 | 0.3266 | 0.74397 | 0.863556 | FALSE |
| LOC100240735 | 10964 | 0.326537 | 0.744018 | 0.863556 | FALSE |
| MYADM | 8281 | 0.326411 | 0.744113 | 0.863602 | FALSE |
| MYO1B | 1392 | 0.326058 | 0.74438 | 0.86372 | FALSE |
| PSD | 5217 | 0.32528 | 0.744969 | 0.864275 | FALSE |
| DNAJC24 | 3270 | 0.325103 | 0.745103 | 0.864329 | FALSE |
| GPR173 | 12055 | 0.324807 | 0.745327 | 0.864434 | FALSE |
| ZSWIM6 | 7425 | 0.324649 | 0.745447 | 0.864453 | FALSE |
| FOXO4 | 865 | 0.32462 | 0.745469 | 0.864453 | FALSE |
| TLN1 | 10758 | 0.324492 | 0.745566 | 0.864453 | FALSE |
| ESRP2 | 5940 | 0.324419 | 0.745621 | 0.864453 | FALSE |
| CAMKK2 | 1611 | 0.324163 | 0.745815 | 0.864614 | FALSE |
| ZNF134 | 14076 | 0.324051 | 0.745899 | 0.864648 | FALSE |
| ABCB1 | 11036 | 0.323601 | 0.74624 | 0.864815 | FALSE |
| CD27 | 11655 | 0.323594 | 0.746245 | 0.864815 | FALSE |
| F11R | 10217 | 0.323569 | 0.746264 | 0.864815 | FALSE |
| PLAG1 | 798 | 0.323275 | 0.746487 | 0.865009 | FALSE |
| DAGLA | 8209 | 0.323002 | 0.746694 | 0.865184 | FALSE |
| PMS1 | 10138 | 0.322746 | 0.746888 | 0.86528 | FALSE |
| CSNK1A1 | 8365 | 0.322666 | 0.746948 | 0.865286 | FALSE |
| HIVEP1 | 5749 | 0.322519 | 0.74706 | 0.865351 | FALSE |
| ELOVL1 | 3656 | 0.3222 | 0.747301 | 0.8654 | FALSE |
| ZCWPW1 | 9783 | 0.321925 | 0.74751 | 0.865409 | FALSE |
| CBLL1 | 3458 | 0.321869 | 0.747552 | 0.865409 | FALSE |
| CAV2 | 13064 | 0.321869 | 0.747552 | 0.865409 | FALSE |
| ZFHX4 | 3661 | 0.321568 | 0.74778 | 0.865461 | FALSE |
| SLC6A16 | 11685 | 0.32127 | 0.748006 | 0.865485 | FALSE |
| UNC13D | 14797 | 0.320994 | 0.748215 | 0.865663 | FALSE |
| POC1B | 13614 | 0.320402 | 0.748664 | 0.865959 | FALSE |
| REST | 2074 | 0.320364 | 0.748692 | 0.865959 | FALSE |
| PGD | 1201 | 0.320139 | 0.748863 | 0.866029 | FALSE |
| C16orf86 | 9120 | 0.319902 | 0.749043 | 0.866042 | FALSE |
| GPBAR1 | 9357 | 0.319854 | 0.749079 | 0.866042 | FALSE |
| SFMBT1 | 13083 | 0.319769 | 0.749143 | 0.866042 | FALSE |
| PARD3 | 9656 | 0.319758 | 0.749152 | 0.866042 | FALSE |
| CCDC51 | 6898 | 0.319635 | 0.749245 | 0.866086 | FALSE |
| LRFN1 | 4703 | 0.3193 | 0.749499 | 0.866152 | FALSE |
| TCEAL4 | 11006 | 0.319267 | 0.749524 | 0.866152 | FALSE |
| CAP2 | 6935 | 0.318912 | 0.749793 | 0.866293 | FALSE |
| HLA-J | 3890 | 0.318673 | 0.749974 | 0.866331 | FALSE |
| LRRC10B | 13580 | 0.318625 | 0.750011 | 0.866331 | FALSE |
| PHF14 | 2860 | 0.318546 | 0.750071 | 0.866336 | FALSE |
| DHX29 | 2839 | 0.318473 | 0.750126 | 0.866336 | FALSE |
| DPF3 | 11457 | 0.317838 | 0.750608 | 0.866764 | FALSE |
| EIF5 | 13660 | 0.316997 | 0.751246 | 0.867181 | FALSE |
| RFTN2 | 9102 | 0.316675 | 0.75149 | 0.867335 | FALSE |
| CEP85 | 639 | 0.316237 | 0.751823 | 0.867526 | FALSE |
| BEST1 | 5031 | 0.315536 | 0.752355 | 0.867948 | FALSE |
| LOC440570 | 2652 | 0.314896 | 0.752841 | 0.868226 | FALSE |
| TRAPPC13 | 620 | 0.314594 | 0.75307 | 0.868244 | FALSE |
| MITD1 | 11466 | 0.314374 | 0.753237 | 0.868325 | FALSE |
| CTAGE11P | 6445 | 0.314212 | 0.75336 | 0.868339 | FALSE |
| CCDC160 | 13389 | 0.313702 | 0.753747 | 0.868721 | FALSE |
| PRCP | 4124 | 0.313369 | 0.754 | 0.868885 | FALSE |
| SYT14 | 1101 | 0.313041 | 0.75425 | 0.868982 | FALSE |
| C10orf90 | 6307 | 0.312804 | 0.75443 | 0.868982 | FALSE |
| EXOC8 | 6636 | 0.312792 | 0.754439 | 0.868982 | FALSE |
| ZNF260 | 2353 | 0.312767 | 0.754458 | 0.868982 | FALSE |
| NBPF10 | 4811 | 0.312745 | 0.754474 | 0.868982 | FALSE |
| FHL1 | 15395 | 0.31231 | 0.754805 | 0.869299 | FALSE |
| SNX13 | 4529 | 0.311494 | 0.755425 | 0.869663 | FALSE |
| SDC1 | 14461 | 0.31106 | 0.755755 | 0.869663 | FALSE |
| TACC2 | 8759 | 0.311012 | 0.755791 | 0.869663 | FALSE |
| MXI1 | 14994 | 0.310867 | 0.755902 | 0.869663 | FALSE |
| KMT5A | 3580 | 0.310668 | 0.756053 | 0.869663 | FALSE |
| TOMM40L | 8420 | 0.309965 | 0.756588 | 0.870006 | FALSE |
| MRPL12 | 14417 | 0.309844 | 0.75668 | 0.870048 | FALSE |
| LGALS8 | 14412 | 0.309452 | 0.756978 | 0.870327 | FALSE |
| PPP3R1 | 4769 | 0.30888 | 0.757413 | 0.870761 | FALSE |
| MSH3 | 5252 | 0.308809 | 0.757467 | 0.870761 | FALSE |
| HCN2 | 11564 | 0.308056 | 0.75804 | 0.871234 | FALSE |
| CLSTN2 | 5018 | 0.308049 | 0.758045 | 0.871234 | FALSE |
| CFAP206 | 10399 | 0.307936 | 0.758131 | 0.871268 | FALSE |
| CYYR1 | 6379 | 0.307285 | 0.758626 | 0.871453 | FALSE |
| UMAD1 | 14896 | 0.307062 | 0.758796 | 0.871584 | FALSE |
| INPP5J | 12377 | 0.306718 | 0.759058 | 0.871757 | FALSE |
| MDH1 | 3505 | 0.30658 | 0.759163 | 0.87176 | FALSE |
| APCS | 15489 | 0.306568 | 0.759172 | 0.87176 | FALSE |
| RXRB | 13673 | 0.306401 | 0.759299 | 0.871794 | FALSE |
| FAM92A | 14510 | 0.306206 | 0.759448 | 0.871884 | FALSE |
| CCZ1B | 15408 | 0.305782 | 0.759771 | 0.871967 | FALSE |
| PLPP2 | 11865 | 0.30571 | 0.759825 | 0.871967 | FALSE |
| PABPC5 | 7667 | 0.305695 | 0.759837 | 0.871967 | FALSE |
| VPS8 | 9213 | 0.305246 | 0.760179 | 0.872219 | FALSE |
| NRBP2 | 14000 | 0.305237 | 0.760186 | 0.872219 | FALSE |
| SMURF2 | 4039 | 0.305116 | 0.760278 | 0.872261 | FALSE |
| DGKZ | 7448 | 0.304754 | 0.760554 | 0.872414 | FALSE |
| ZNF684 | 37 | 0.304607 | 0.760665 | 0.872449 | FALSE |
| FANCF | 7744 | 0.304495 | 0.760751 | 0.87248 | FALSE |
| C1GALT1C1 | 1411 | 0.304334 | 0.760873 | 0.87248 | FALSE |
| ZNF619 | 557 | 0.304279 | 0.760915 | 0.87248 | FALSE |
| ZNF418 | 973 | 0.303832 | 0.761256 | 0.872806 | FALSE |
| EFHD1 | 9533 | 0.303679 | 0.761372 | 0.872843 | FALSE |
| ZNF431 | 2320 | 0.303594 | 0.761437 | 0.872843 | FALSE |
| ZNF7 | 9844 | 0.303292 | 0.761667 | 0.872894 | FALSE |
| ZSWIM8 | 9431 | 0.302906 | 0.761961 | 0.873103 | FALSE |
| AP3M1 | 1691 | 0.302761 | 0.762072 | 0.873166 | FALSE |
| CD226 | 943 | 0.302112 | 0.762567 | 0.873668 | FALSE |
| ZXDA | 2399 | 0.302028 | 0.762631 | 0.873678 | FALSE |
| PHLDB1 | 8944 | 0.301699 | 0.762882 | 0.873901 | FALSE |
| DGKE | 3768 | 0.30135 | 0.763148 | 0.873999 | FALSE |
| MEGF6 | 5781 | 0.301173 | 0.763283 | 0.87404 | FALSE |
| SRP9 | 8051 | 0.300886 | 0.763501 | 0.874227 | FALSE |
| GHSR | 5702 | 0.300479 | 0.763812 | 0.874371 | FALSE |
| ALG10B | 14169 | 0.299932 | 0.764229 | 0.874675 | FALSE |
| ZACN | 15561 | 0.299808 | 0.764324 | 0.874697 | FALSE |
| SAMD5 | 9980 | 0.299721 | 0.76439 | 0.874697 | FALSE |
| CASP4 | 4988 | 0.299603 | 0.76448 | 0.874697 | FALSE |
| ZNF728 | 10245 | 0.299375 | 0.764654 | 0.874697 | FALSE |
| STK33 | 11290 | 0.299321 | 0.764695 | 0.874697 | FALSE |
| C1orf94 | 14338 | 0.299007 | 0.764935 | 0.874907 | FALSE |
| ENKD1 | 9238 | 0.298534 | 0.765296 | 0.875113 | FALSE |
| ZNF57 | 7551 | 0.298477 | 0.765339 | 0.875113 | FALSE |
| ABCF2 | 792 | 0.298279 | 0.76549 | 0.875222 | FALSE |
| ENO1-AS1 | 1429 | 0.297437 | 0.766133 | 0.875572 | FALSE |
| ST3GAL4 | 12358 | 0.296237 | 0.767049 | 0.876235 | FALSE |
| GABRA1 | 10290 | 0.294887 | 0.76808 | 0.877093 | FALSE |
| KANSL3 | 12345 | 0.294771 | 0.768169 | 0.877102 | FALSE |
| DDX28 | 2639 | 0.294582 | 0.768313 | 0.877102 | FALSE |
| ACSL1 | 6180 | 0.293638 | 0.769035 | 0.87767 | FALSE |
| APOL1 | 9426 | 0.29335 | 0.769255 | 0.877846 | FALSE |
| ASAP1 | 10224 | 0.293289 | 0.769301 | 0.877846 | FALSE |
| OTUD6B | 204 | 0.293182 | 0.769383 | 0.877875 | FALSE |
| CD164 | 11125 | 0.293074 | 0.769466 | 0.877905 | FALSE |
| TMEM158 | 13527 | 0.2928 | 0.769675 | 0.877907 | FALSE |
| GLRX | 15379 | 0.292704 | 0.769748 | 0.877907 | FALSE |
| SURF4 | 12653 | 0.292276 | 0.770076 | 0.878152 | FALSE |
| TTC9 | 13500 | 0.291368 | 0.77077 | 0.87856 | FALSE |
| WAS | 4693 | 0.29112 | 0.77096 | 0.878648 | FALSE |
| CRBN | 7154 | 0.290736 | 0.771253 | 0.878656 | FALSE |
| TMEM25 | 2019 | 0.289493 | 0.772204 | 0.879233 | FALSE |
| DDX42 | 8370 | 0.288848 | 0.772698 | 0.879603 | FALSE |
| SBDS | 1291 | 0.288598 | 0.772889 | 0.87961 | FALSE |
| LOC100996385 | 15054 | 0.288414 | 0.77303 | 0.87966 | FALSE |
| RIC1 | 13238 | 0.288258 | 0.773149 | 0.879732 | FALSE |
| CCDC142 | 4211 | 0.287585 | 0.773664 | 0.880126 | FALSE |
| ZNF584 | 15469 | 0.287393 | 0.773811 | 0.880165 | FALSE |
| FOXB1 | 7741 | 0.286953 | 0.774148 | 0.880366 | FALSE |
| ATG5 | 3870 | 0.286868 | 0.774213 | 0.880366 | FALSE |
| ARHGEF37 | 403 | 0.286456 | 0.774529 | 0.880617 | FALSE |
| TFPI | 15234 | 0.286102 | 0.7748 | 0.880724 | FALSE |
| SAMD4B | 8046 | 0.285943 | 0.774922 | 0.880724 | FALSE |
| TNFRSF12A | 7838 | 0.285369 | 0.775361 | 0.881031 | FALSE |
| TARS | 4168 | 0.285177 | 0.775509 | 0.881134 | FALSE |
| SRP72 | 7119 | 0.284869 | 0.775745 | 0.881274 | FALSE |
| TCL1B | 8178 | 0.284541 | 0.775996 | 0.881432 | FALSE |
| ARMC2 | 14690 | 0.284233 | 0.776232 | 0.881534 | FALSE |
| ZNF416 | 10505 | 0.283974 | 0.77643 | 0.881669 | FALSE |
| ANP32C | 3899 | 0.283791 | 0.776571 | 0.881764 | FALSE |
| NUFIP1 | 2582 | 0.282923 | 0.777236 | 0.882327 | FALSE |
| ADAT2 | 5433 | 0.282453 | 0.777596 | 0.88253 | FALSE |
| TXNDC12 | 11362 | 0.282163 | 0.777819 | 0.882608 | FALSE |
| TFAP2C | 1914 | 0.281849 | 0.778059 | 0.882813 | FALSE |
| LINC00506 | 14773 | 0.281564 | 0.778278 | 0.882933 | FALSE |
| SDCCAG3 | 7452 | 0.281528 | 0.778305 | 0.882933 | FALSE |
| CGRRF1 | 4142 | 0.281337 | 0.778452 | 0.882938 | FALSE |
| NARS | 13290 | 0.280997 | 0.778713 | 0.882978 | FALSE |
| ARNTL2 | 7343 | 0.280722 | 0.778924 | 0.883153 | FALSE |
| MTHFSD | 14532 | 0.280455 | 0.779128 | 0.883181 | FALSE |
| PPP2R3A | 4 | 0.28036 | 0.779201 | 0.883181 | FALSE |
| PRTFDC1 | 5734 | 0.280322 | 0.77923 | 0.883181 | FALSE |
| SNX24 | 6458 | 0.279929 | 0.779532 | 0.883394 | FALSE |
| GRIN1 | 5388 | 0.279817 | 0.779618 | 0.883428 | FALSE |
| TMED9 | 11978 | 0.27967 | 0.779731 | 0.883491 | FALSE |
| PPP1R16A | 12370 | 0.279303 | 0.780012 | 0.883566 | FALSE |
| SPATA2L | 8743 | 0.279105 | 0.780164 | 0.883566 | FALSE |
| TRIM61 | 10204 | 0.279027 | 0.780224 | 0.883566 | FALSE |
| TXNRD1 | 3834 | 0.278864 | 0.780349 | 0.883566 | FALSE |
| LOC103091866 | 14823 | 0.278854 | 0.780357 | 0.883566 | FALSE |
| LOC440084 | 8539 | 0.278799 | 0.780399 | 0.883566 | FALSE |
| RNF13 | 3268 | 0.278774 | 0.780418 | 0.883566 | FALSE |
| ZNF534 | 9609 | 0.278663 | 0.780503 | 0.883579 | FALSE |
| PPP1R13B | 9446 | 0.278532 | 0.780604 | 0.883579 | FALSE |
| ADORA1 | 11596 | 0.278485 | 0.78064 | 0.883579 | FALSE |
| LURAP1 | 13664 | 0.27835 | 0.780744 | 0.883614 | FALSE |
| TMEM129 | 13190 | 0.277719 | 0.781228 | 0.883976 | FALSE |
| TBC1D17 | 6805 | 0.277639 | 0.781289 | 0.883976 | FALSE |
| RPA4 | 13262 | 0.276969 | 0.781804 | 0.884247 | FALSE |
| CREB1 | 5236 | 0.276944 | 0.781823 | 0.884247 | FALSE |
| GPR135 | 997 | 0.276519 | 0.782149 | 0.884373 | FALSE |
| EIF1AX | 13925 | 0.275739 | 0.782749 | 0.88485 | FALSE |
| PRKCQ-AS1 | 5994 | 0.275675 | 0.782798 | 0.88485 | FALSE |
| LOC100506544 | 216 | 0.275368 | 0.783034 | 0.885053 | FALSE |
| RNF38 | 14831 | 0.274925 | 0.783374 | 0.885373 | FALSE |
| FBXW11 | 13476 | 0.274183 | 0.783944 | 0.885762 | FALSE |
| SYPL1 | 3344 | 0.27334 | 0.784592 | 0.886237 | FALSE |
| RABGAP1L | 15220 | 0.272882 | 0.784944 | 0.886384 | FALSE |
| MLKL | 9425 | 0.272668 | 0.785108 | 0.886384 | FALSE |
| LRWD1 | 9984 | 0.272638 | 0.785131 | 0.886384 | FALSE |
| CHD7 | 3783 | 0.272608 | 0.785155 | 0.886384 | FALSE |
| FAM13C | 9624 | 0.272585 | 0.785172 | 0.886384 | FALSE |
| TMEM245 | 13378 | 0.272507 | 0.785232 | 0.886384 | FALSE |
| FN1 | 5743 | 0.272239 | 0.785438 | 0.886543 | FALSE |
| ZNF85 | 12364 | 0.272064 | 0.785573 | 0.886543 | FALSE |
| FOXD3 | 2902 | 0.27203 | 0.785599 | 0.886543 | FALSE |
| IFIT2 | 3519 | 0.271898 | 0.7857 | 0.886543 | FALSE |
| ERAP1 | 6472 | 0.271881 | 0.785714 | 0.886543 | FALSE |
| NTM-AS1 | 15265 | 0.271596 | 0.785933 | 0.886646 | FALSE |
| BORCS6 | 1788 | 0.271336 | 0.786133 | 0.88676 | FALSE |
| RTP4 | 8430 | 0.271034 | 0.786365 | 0.886865 | FALSE |
| ZNF337 | 2944 | 0.270999 | 0.786392 | 0.886865 | FALSE |
| MFSD6L | 9572 | 0.270782 | 0.786559 | 0.886946 | FALSE |
| TLNRD1 | 5589 | 0.270648 | 0.786662 | 0.886946 | FALSE |
| OPRL1 | 2221 | 0.270606 | 0.786694 | 0.886946 | FALSE |
| SMG9 | 5782 | 0.270186 | 0.787017 | 0.887118 | FALSE |
| UNC50 | 677 | 0.269628 | 0.787446 | 0.887438 | FALSE |
| MDM4 | 12880 | 0.269501 | 0.787544 | 0.887438 | FALSE |
| LSM11 | 14079 | 0.269444 | 0.787588 | 0.887438 | FALSE |
| NOL8 | 5754 | 0.269374 | 0.787642 | 0.887438 | FALSE |
| LHX2 | 116 | 0.269242 | 0.787743 | 0.887489 | FALSE |
| RGR | 8383 | 0.26897 | 0.787953 | 0.887578 | FALSE |
| LOC102606465 | 3831 | 0.268321 | 0.788452 | 0.887792 | FALSE |
| PPP2R5A | 12474 | 0.268159 | 0.788577 | 0.887792 | FALSE |
| BIVM | 7476 | 0.26785 | 0.788815 | 0.887874 | FALSE |
| DOCK11 | 2293 | 0.266977 | 0.789487 | 0.888237 | FALSE |
| MAPK9 | 9700 | 0.266447 | 0.789895 | 0.888568 | FALSE |
| PDE6D | 4245 | 0.266042 | 0.790207 | 0.88863 | FALSE |
| SAP30 | 13563 | 0.266011 | 0.790231 | 0.88863 | FALSE |
| TK1 | 1248 | 0.266008 | 0.790233 | 0.88863 | FALSE |
| CDC14C | 4462 | 0.266006 | 0.790235 | 0.88863 | FALSE |
| SMIM14 | 15601 | 0.265837 | 0.790365 | 0.888693 | FALSE |
| ASB16 | 3006 | 0.265334 | 0.790752 | 0.888904 | FALSE |
| PCSK4 | 14256 | 0.265321 | 0.790762 | 0.888904 | FALSE |
| ZNF474 | 13104 | 0.265044 | 0.790976 | 0.88908 | FALSE |
| CAND2 | 11848 | 0.264286 | 0.79156 | 0.88948 | FALSE |
| TIGAR | 983 | 0.263635 | 0.792061 | 0.889724 | FALSE |
| SSX2IP | 6743 | 0.262833 | 0.792679 | 0.890227 | FALSE |
| CLPTM1 | 4505 | 0.262509 | 0.792929 | 0.890443 | FALSE |
| PSRC1 | 4656 | 0.261623 | 0.793612 | 0.891018 | FALSE |
| GTF3C4 | 12728 | 0.261361 | 0.793814 | 0.891117 | FALSE |
| CXXC5 | 3310 | 0.261172 | 0.79396 | 0.891215 | FALSE |
| WDFY1 | 5970 | 0.2611 | 0.794015 | 0.891215 | FALSE |
| LOC100130691 | 12014 | 0.260977 | 0.79411 | 0.891257 | FALSE |
| GPAM | 11105 | 0.260853 | 0.794206 | 0.891279 | FALSE |
| DIRAS2 | 2282 | 0.260724 | 0.794305 | 0.891279 | FALSE |
| KIAA1586 | 755 | 0.260659 | 0.794355 | 0.891279 | FALSE |
| SLC7A1 | 4698 | 0.260239 | 0.794679 | 0.891576 | FALSE |
| PODXL | 1105 | 0.260071 | 0.794809 | 0.891622 | FALSE |
| ZNF789 | 8066 | 0.25972 | 0.79508 | 0.891699 | FALSE |
| ABCA2 | 9059 | 0.259653 | 0.795131 | 0.891699 | FALSE |
| FAM3A | 11437 | 0.259094 | 0.795563 | 0.891904 | FALSE |
| FLJ23867 | 14731 | 0.259047 | 0.795599 | 0.891904 | FALSE |
| CXCL12 | 13297 | 0.25884 | 0.795759 | 0.892019 | FALSE |
| CNNM4 | 12701 | 0.258259 | 0.796207 | 0.892457 | FALSE |
| RAPGEF5 | 3873 | 0.258154 | 0.796288 | 0.892484 | FALSE |
| ADGRG5 | 6887 | 0.257646 | 0.79668 | 0.892826 | FALSE |
| PRRC1 | 11893 | 0.257611 | 0.796707 | 0.892826 | FALSE |
| CA14 | 1364 | 0.257023 | 0.797161 | 0.893079 | FALSE |
| SCD5 | 7987 | 0.256632 | 0.797463 | 0.893208 | FALSE |
| PAWR | 6450 | 0.256631 | 0.797464 | 0.893208 | FALSE |
| SMNDC1 | 13759 | 0.255895 | 0.798032 | 0.89367 | FALSE |
| EIF2S3B | 13582 | 0.255723 | 0.798165 | 0.893732 | FALSE |
| LIFR | 11680 | 0.255675 | 0.798202 | 0.893732 | FALSE |
| DENND4B | 4906 | 0.255481 | 0.798352 | 0.893836 | FALSE |
| SPSB3 | 11714 | 0.254933 | 0.798775 | 0.894069 | FALSE |
| RRN3 | 1359 | 0.254834 | 0.798851 | 0.894069 | FALSE |
| LIG4 | 901 | 0.254767 | 0.798903 | 0.894069 | FALSE |
| SYCE1 | 9899 | 0.254438 | 0.799157 | 0.894102 | FALSE |
| FREM1 | 1412 | 0.254386 | 0.799197 | 0.894102 | FALSE |
| C3orf33 | 2993 | 0.253543 | 0.799849 | 0.894551 | FALSE |
| STX18-AS1 | 6109 | 0.253129 | 0.800169 | 0.894845 | FALSE |
| BOLA3-AS1 | 11727 | 0.253035 | 0.800241 | 0.894862 | FALSE |
| TCP11L1 | 3283 | 0.252099 | 0.800965 | 0.895351 | FALSE |
| SHROOM3 | 858 | 0.250758 | 0.802001 | 0.896295 | FALSE |
| NKRF | 6968 | 0.250635 | 0.802096 | 0.896295 | FALSE |
| CES2 | 9709 | 0.250535 | 0.802174 | 0.896318 | FALSE |
| MYRF | 9573 | 0.249663 | 0.802848 | 0.8967 | FALSE |
| CKM | 10763 | 0.249648 | 0.80286 | 0.8967 | FALSE |
| THAP7 | 10426 | 0.249462 | 0.803003 | 0.896796 | FALSE |
| LOC90246 | 9945 | 0.249191 | 0.803213 | 0.896936 | FALSE |
| PFKM | 7325 | 0.249152 | 0.803243 | 0.896936 | FALSE |
| FAM83E | 12926 | 0.249015 | 0.803349 | 0.89699 | FALSE |
| RECK | 8026 | 0.248809 | 0.803509 | 0.897052 | FALSE |
| CEP126 | 9728 | 0.248795 | 0.803519 | 0.897052 | FALSE |
| BFAR | 2335 | 0.248378 | 0.803842 | 0.897348 | FALSE |
| DDX60L | 3988 | 0.248112 | 0.804048 | 0.897503 | FALSE |
| RNF14 | 5328 | 0.246887 | 0.804996 | 0.898124 | FALSE |
| KMT2D | 10064 | 0.246868 | 0.80501 | 0.898124 | FALSE |
| ABCC9 | 8035 | 0.246439 | 0.805342 | 0.898381 | FALSE |
| SVBP | 2137 | 0.246365 | 0.8054 | 0.898381 | FALSE |
| IRAK4 | 1469 | 0.2461 | 0.805605 | 0.898546 | FALSE |
| ASIC3 | 5991 | 0.24585 | 0.805798 | 0.898691 | FALSE |
| FAM222A | 10062 | 0.245645 | 0.805957 | 0.898713 | FALSE |
| LOC441454 | 9923 | 0.244903 | 0.806532 | 0.899131 | FALSE |
| WIPF1 | 5267 | 0.244086 | 0.807164 | 0.899511 | FALSE |
| TM9SF2 | 6122 | 0.244053 | 0.80719 | 0.899511 | FALSE |
| HOMER3 | 12738 | 0.244016 | 0.807218 | 0.899511 | FALSE |
| CCDC92 | 7788 | 0.243859 | 0.80734 | 0.899539 | FALSE |
| TMEM260 | 49 | 0.243836 | 0.807358 | 0.899539 | FALSE |
| ACACA | 10713 | 0.243707 | 0.807458 | 0.899586 | FALSE |
| HMX1 | 8246 | 0.243105 | 0.807924 | 0.899849 | FALSE |
| PABPC4 | 1999 | 0.242955 | 0.80804 | 0.899914 | FALSE |
| GIMAP1 | 718 | 0.242609 | 0.808308 | 0.899966 | FALSE |
| BTN3A1 | 9756 | 0.242276 | 0.808566 | 0.900169 | FALSE |
| DOCK9 | 11555 | 0.24145 | 0.809206 | 0.900727 | FALSE |
| WASHC5 | 10944 | 0.241223 | 0.809382 | 0.900793 | FALSE |
| SUCLA2 | 4575 | 0.241193 | 0.809406 | 0.900793 | FALSE |
| C15orf59 | 15622 | 0.240753 | 0.809747 | 0.901044 | FALSE |
| ZMPSTE24 | 6621 | 0.240473 | 0.809964 | 0.901157 | FALSE |
| SLCO1A2 | 2852 | 0.239636 | 0.810612 | 0.901731 | FALSE |
| APOO | 6526 | 0.239524 | 0.810699 | 0.901731 | FALSE |
| TCF23 | 3943 | 0.239482 | 0.810732 | 0.901731 | FALSE |
| LCN15 | 11063 | 0.239361 | 0.810826 | 0.901731 | FALSE |
| SUGT1 | 9021 | 0.23871 | 0.81133 | 0.902174 | FALSE |
| KRTAP13-1 | 1315 | 0.238557 | 0.811449 | 0.902174 | FALSE |
| PDE6B | 8810 | 0.238453 | 0.81153 | 0.902193 | FALSE |
| GTF2E2 | 1905 | 0.237155 | 0.812537 | 0.903004 | FALSE |
| CTRL | 8679 | 0.237141 | 0.812547 | 0.903004 | FALSE |
| CD22 | 8305 | 0.236124 | 0.813336 | 0.903728 | FALSE |
| RARA | 4463 | 0.235637 | 0.813714 | 0.903915 | FALSE |
| TSN | 11631 | 0.235034 | 0.814182 | 0.904145 | FALSE |
| HAGLR | 2571 | 0.234977 | 0.814227 | 0.904145 | FALSE |
| KIF3B | 125 | 0.234976 | 0.814227 | 0.904145 | FALSE |
| TYSND1 | 7817 | 0.234923 | 0.814268 | 0.904145 | FALSE |
| BTN3A2 | 5509 | 0.234428 | 0.814653 | 0.904379 | FALSE |
| IPO8 | 12797 | 0.234198 | 0.814831 | 0.904513 | FALSE |
| TRIM29 | 9751 | 0.233344 | 0.815494 | 0.905133 | FALSE |
| BEX4 | 9331 | 0.233329 | 0.815506 | 0.905133 | FALSE |
| CFB | 14826 | 0.233162 | 0.815636 | 0.905213 | FALSE |
| SCN2B | 15021 | 0.232992 | 0.815768 | 0.905228 | FALSE |
| TOPORS | 13318 | 0.232775 | 0.815936 | 0.905228 | FALSE |
| PATZ1 | 9459 | 0.232697 | 0.815997 | 0.905228 | FALSE |
| PIK3R2 | 28 | 0.232482 | 0.816164 | 0.905307 | FALSE |
| POC1B-AS1 | 3407 | 0.231945 | 0.816581 | 0.905521 | FALSE |
| NPHP1 | 4499 | 0.231792 | 0.8167 | 0.905521 | FALSE |
| SRRT | 5008 | 0.231751 | 0.816731 | 0.905521 | FALSE |
| PDZD4 | 6696 | 0.231634 | 0.816822 | 0.905521 | FALSE |
| D2HGDH | 3966 | 0.231611 | 0.81684 | 0.905521 | FALSE |
| TSPAN31 | 4819 | 0.231397 | 0.817006 | 0.905641 | FALSE |
| FIGNL1 | 14682 | 0.230935 | 0.817365 | 0.905683 | FALSE |
| MED31 | 5714 | 0.230832 | 0.817445 | 0.905683 | FALSE |
| HIST1H3D | 7239 | 0.230759 | 0.817502 | 0.905683 | FALSE |
| CLP1 | 5674 | 0.230745 | 0.817513 | 0.905683 | FALSE |
| SPTAN1 | 7847 | 0.230657 | 0.817581 | 0.905683 | FALSE |
| APPL2 | 5456 | 0.230603 | 0.817623 | 0.905683 | FALSE |
| NEK8 | 13478 | 0.230324 | 0.81784 | 0.905803 | FALSE |
| CARM1 | 14932 | 0.230314 | 0.817848 | 0.905803 | FALSE |
| WSCD2 | 10532 | 0.230164 | 0.817964 | 0.905868 | FALSE |
| PIP5K1C | 14727 | 0.229827 | 0.818226 | 0.905964 | FALSE |
| ZNF267 | 8753 | 0.229754 | 0.818283 | 0.905964 | FALSE |
| ARHGEF25 | 11279 | 0.229204 | 0.81871 | 0.906309 | FALSE |
| NXF3 | 10435 | 0.228933 | 0.818921 | 0.906414 | FALSE |
| HIST1H1E | 12214 | 0.228753 | 0.819061 | 0.906461 | FALSE |
| CSTF3 | 172 | 0.228217 | 0.819478 | 0.906698 | FALSE |
| LAP3 | 7234 | 0.228192 | 0.819497 | 0.906698 | FALSE |
| HNRNPA1P33 | 13762 | 0.228132 | 0.819544 | 0.906698 | FALSE |
| C12orf4 | 9254 | 0.22799 | 0.819654 | 0.906698 | FALSE |
| CENPQ | 10470 | 0.227873 | 0.819745 | 0.906698 | FALSE |
| LOC100506725 | 11652 | 0.227316 | 0.820178 | 0.90687 | FALSE |
| CNKSR3 | 8540 | 0.227216 | 0.820256 | 0.90687 | FALSE |
| TRAPPC2B | 2084 | 0.227209 | 0.820261 | 0.90687 | FALSE |
| CCDC28B | 15186 | 0.226111 | 0.821115 | 0.907649 | FALSE |
| ORC2 | 6861 | 0.2257 | 0.821435 | 0.907747 | FALSE |
| RBPMS | 8794 | 0.225666 | 0.821461 | 0.907747 | FALSE |
| MPHOSPH10 | 11361 | 0.225433 | 0.821642 | 0.907884 | FALSE |
| AKAP9 | 12948 | 0.225083 | 0.821915 | 0.90812 | FALSE |
| HECTD2 | 3771 | 0.22444 | 0.822415 | 0.908338 | FALSE |
| NF1 | 7195 | 0.223853 | 0.822872 | 0.908664 | FALSE |
| FRG1BP | 9157 | 0.222514 | 0.823914 | 0.909429 | FALSE |
| SIX5 | 308 | 0.222413 | 0.823992 | 0.909452 | FALSE |
| NHLRC3 | 13764 | 0.222314 | 0.824069 | 0.909462 | FALSE |
| EIF2AK2 | 15446 | 0.221739 | 0.824517 | 0.909622 | FALSE |
| CMC4 | 3528 | 0.221645 | 0.82459 | 0.909622 | FALSE |
[truncated: 513,017 more chars]
