# Supplementary material for: Brain tissue electrical conductivity as a promising biomarker for dementia assessment using MRI
Source: Alzheimers Dement. 2025 Jun 23;21(6):e70270. doi: 10.1002/alz.70270 (PMC12185248; doi:10.1002/alz.70270)
Supplement: Supplementary file 3 — Supporting Information [file ALZ-21-e70270-s011.docx]

**Table S16.** The complete set of PLS2 gene weights and associated statistics for Aβ SUVRs difference between Dementia and cognitively normal participants.

| geneIDs | geneIDX | zscores | pvals | adj.pvals | significant |
| --- | --- | --- | --- | --- | --- |
| KLK10 | 14341 | 7.375631 | 1.64E-13 | 1.71E-10 | TRUE |
| PRKCD | 7139 | 7.375236 | 1.64E-13 | 1.71E-10 | TRUE |
| SYT17 | 1554 | 6.883268 | 5.85E-12 | 2.74E-09 | TRUE |
| DOK6 | 3024 | 6.502316 | 7.91E-11 | 2.19E-08 | TRUE |
| UG0898H09 | 3372 | 6.500677 | 8.00E-11 | 2.19E-08 | TRUE |
| IFT22 | 7939 | 6.482085 | 9.05E-11 | 2.36E-08 | TRUE |
| TMSB4X | 1602 | 6.415347 | 1.41E-10 | 3.37E-08 | TRUE |
| RAP2B | 13589 | 6.254689 | 3.98E-10 | 7.50E-08 | TRUE |
| MCUB | 5785 | 6.234349 | 4.54E-10 | 8.25E-08 | TRUE |
| SLIT1 | 12462 | 6.224421 | 4.83E-10 | 8.59E-08 | TRUE |
| RSPH9 | 10941 | 6.146546 | 7.92E-10 | 1.26E-07 | TRUE |
| GRP | 2250 | 6.136355 | 8.44E-10 | 1.29E-07 | TRUE |
| LINC02482 | 6419 | 6.113176 | 9.77E-10 | 1.45E-07 | TRUE |
| PLPPR3 | 2390 | 6.112723 | 9.79E-10 | 1.45E-07 | TRUE |
| LINC00260 | 9309 | 6.070944 | 1.27E-09 | 1.73E-07 | TRUE |
| UCHL3 | 4830 | 6.040218 | 1.54E-09 | 2.04E-07 | TRUE |
| RNF150 | 12381 | 5.987436 | 2.13E-09 | 2.55E-07 | TRUE |
| GNG2 | 10880 | 5.986835 | 2.14E-09 | 2.55E-07 | TRUE |
| PRSS35 | 6292 | 5.921738 | 3.19E-09 | 3.39E-07 | TRUE |
| GLRA2 | 8062 | 5.921634 | 3.19E-09 | 3.39E-07 | TRUE |
| DIRAS3 | 12811 | 5.909608 | 3.43E-09 | 3.57E-07 | TRUE |
| SNHG8 | 11116 | 5.8812 | 4.07E-09 | 4.19E-07 | TRUE |
| MTCH1 | 11981 | 5.855614 | 4.75E-09 | 4.76E-07 | TRUE |
| OST4 | 14254 | 5.790798 | 7.01E-09 | 6.29E-07 | TRUE |
| NUPR2 | 13556 | 5.786589 | 7.18E-09 | 6.38E-07 | TRUE |
| KCNMB4 | 8402 | 5.778788 | 7.52E-09 | 6.61E-07 | TRUE |
| SLA | 13992 | 5.769032 | 7.97E-09 | 6.92E-07 | TRUE |
| RNASEH2C | 8533 | 5.76743 | 8.05E-09 | 6.95E-07 | TRUE |
| WDR66 | 10107 | 5.713762 | 1.11E-08 | 9.00E-07 | TRUE |
| LOC100288911 | 7410 | 5.699758 | 1.20E-08 | 9.52E-07 | TRUE |
| ASIC2 | 14732 | 5.69508 | 1.23E-08 | 9.74E-07 | TRUE |
| PPM1M | 11370 | 5.654791 | 1.56E-08 | 1.13E-06 | TRUE |
| HIST1H2BK | 795 | 5.643893 | 1.66E-08 | 1.19E-06 | TRUE |
| KLHDC8B | 14785 | 5.633375 | 1.77E-08 | 1.26E-06 | TRUE |
| DKFZp779M0652 | 13234 | 5.628203 | 1.82E-08 | 1.28E-06 | TRUE |
| SMARCD3 | 14560 | 5.604641 | 2.09E-08 | 1.44E-06 | TRUE |
| CMSS1 | 10655 | 5.595131 | 2.20E-08 | 1.49E-06 | TRUE |
| GMFB | 3935 | 5.583627 | 2.36E-08 | 1.57E-06 | TRUE |
| SLC4A3 | 11243 | 5.578123 | 2.43E-08 | 1.61E-06 | TRUE |
| CPNE6 | 4934 | 5.572405 | 2.51E-08 | 1.65E-06 | TRUE |
| PID1 | 5942 | 5.570689 | 2.54E-08 | 1.65E-06 | TRUE |
| NNAT | 2288 | 5.568576 | 2.57E-08 | 1.66E-06 | TRUE |
| YPEL1 | 14736 | 5.545172 | 2.94E-08 | 1.84E-06 | TRUE |
| EFCAB1 | 14244 | 5.542711 | 2.98E-08 | 1.85E-06 | TRUE |
| WTIP | 10234 | 5.537367 | 3.07E-08 | 1.90E-06 | TRUE |
| FAM110C | 7305 | 5.531735 | 3.17E-08 | 1.94E-06 | TRUE |
| NECAB2 | 88 | 5.527263 | 3.25E-08 | 1.96E-06 | TRUE |
| COCH | 10789 | 5.51942 | 3.40E-08 | 2.01E-06 | TRUE |
| MMD | 13442 | 5.516268 | 3.46E-08 | 2.04E-06 | TRUE |
| MYL5 | 10486 | 5.478002 | 4.30E-08 | 2.39E-06 | TRUE |
| PTGER4 | 656 | 5.46629 | 4.60E-08 | 2.51E-06 | TRUE |
| NUDT11 | 12283 | 5.458879 | 4.79E-08 | 2.58E-06 | TRUE |
| CIB1 | 271 | 5.452834 | 4.96E-08 | 2.61E-06 | TRUE |
| RTBDN | 5315 | 5.445637 | 5.16E-08 | 2.70E-06 | TRUE |
| CFD | 11298 | 5.440471 | 5.31E-08 | 2.76E-06 | TRUE |
| RTP1 | 12382 | 5.425411 | 5.78E-08 | 2.94E-06 | TRUE |
| GPR68 | 14387 | 5.41238 | 6.22E-08 | 3.14E-06 | TRUE |
| STX17-AS1 | 10615 | 5.387177 | 7.16E-08 | 3.53E-06 | TRUE |
| TSPAN4 | 5929 | 5.370609 | 7.85E-08 | 3.79E-06 | TRUE |
| KCTD12 | 9032 | 5.366243 | 8.04E-08 | 3.86E-06 | TRUE |
| TBC1D26 | 914 | 5.364542 | 8.12E-08 | 3.88E-06 | TRUE |
| CAMKV | 2813 | 5.357311 | 8.45E-08 | 3.98E-06 | TRUE |
| SP5 | 5361 | 5.356755 | 8.47E-08 | 3.98E-06 | TRUE |
| TXNL4A | 8489 | 5.349183 | 8.84E-08 | 4.07E-06 | TRUE |
| LOXL1 | 9048 | 5.34556 | 9.01E-08 | 4.13E-06 | TRUE |
| TMEM176A | 11654 | 5.341955 | 9.19E-08 | 4.17E-06 | TRUE |
| FARP1 | 14748 | 5.323936 | 1.02E-07 | 4.47E-06 | TRUE |
| B3GNT4 | 6236 | 5.323121 | 1.02E-07 | 4.48E-06 | TRUE |
| AP3S1 | 12790 | 5.319353 | 1.04E-07 | 4.56E-06 | TRUE |
| NPTXR | 13670 | 5.301964 | 1.15E-07 | 4.99E-06 | TRUE |
| C1QL3 | 9585 | 5.299053 | 1.16E-07 | 5.04E-06 | TRUE |
| TMEM263 | 12394 | 5.289698 | 1.23E-07 | 5.22E-06 | TRUE |
| PTGER3 | 3420 | 5.285084 | 1.26E-07 | 5.34E-06 | TRUE |
| SVOP | 995 | 5.276391 | 1.32E-07 | 5.52E-06 | TRUE |
| RASGEF1C | 13535 | 5.262728 | 1.42E-07 | 5.83E-06 | TRUE |
| ADGRB3 | 7657 | 5.251168 | 1.51E-07 | 6.14E-06 | TRUE |
| IFI27L2 | 4602 | 5.247487 | 1.54E-07 | 6.24E-06 | TRUE |
| MDGA1 | 10456 | 5.245495 | 1.56E-07 | 6.30E-06 | TRUE |
| WDR6 | 2116 | 5.238125 | 1.62E-07 | 6.50E-06 | TRUE |
| DNAH5 | 3194 | 5.230642 | 1.69E-07 | 6.72E-06 | TRUE |
| FAM71F1 | 9759 | 5.226672 | 1.73E-07 | 6.83E-06 | TRUE |
| LINC00484 | 2301 | 5.220714 | 1.78E-07 | 6.97E-06 | TRUE |
| CEBPA | 442 | 5.171153 | 2.33E-07 | 8.77E-06 | TRUE |
| HTR1A | 4789 | 5.165668 | 2.40E-07 | 8.95E-06 | TRUE |
| PTPRF | 10525 | 5.165437 | 2.40E-07 | 8.95E-06 | TRUE |
| ACOT2 | 8400 | 5.143417 | 2.70E-07 | 9.85E-06 | TRUE |
| TRIM24 | 7658 | 5.140285 | 2.74E-07 | 9.93E-06 | TRUE |
| GPT2 | 8278 | 5.136561 | 2.80E-07 | 1.01E-05 | TRUE |
| NKAIN4 | 1145 | 5.128603 | 2.92E-07 | 1.05E-05 | TRUE |
| BLNK | 13166 | 5.125285 | 2.97E-07 | 1.06E-05 | TRUE |
| LOC441052 | 10624 | 5.123624 | 3.00E-07 | 1.06E-05 | TRUE |
| HPCAL4 | 13951 | 5.1188 | 3.07E-07 | 1.08E-05 | TRUE |
| TDRD9 | 5111 | 5.115075 | 3.14E-07 | 1.10E-05 | TRUE |
| STUM | 2934 | 5.111256 | 3.20E-07 | 1.12E-05 | TRUE |
| CALB2 | 13929 | 5.104588 | 3.32E-07 | 1.14E-05 | TRUE |
| TUNAR | 11952 | 5.100475 | 3.39E-07 | 1.16E-05 | TRUE |
| TRIM22 | 13758 | 5.094517 | 3.50E-07 | 1.19E-05 | TRUE |
| CRIP2 | 8655 | 5.087727 | 3.62E-07 | 1.23E-05 | TRUE |
| LIX1 | 6668 | 5.086719 | 3.64E-07 | 1.23E-05 | TRUE |
| MZT2B | 14694 | 5.086566 | 3.65E-07 | 1.23E-05 | TRUE |
| RGS10 | 11064 | 5.078841 | 3.80E-07 | 1.27E-05 | TRUE |
| VWA5A | 10594 | 5.072584 | 3.92E-07 | 1.31E-05 | TRUE |
| LOC100129291 | 12472 | 5.068788 | 4.00E-07 | 1.33E-05 | TRUE |
| SCN3B | 1521 | 5.066684 | 4.05E-07 | 1.34E-05 | TRUE |
| UCHL1 | 5973 | 5.043846 | 4.56E-07 | 1.48E-05 | TRUE |
| RIC8A | 4169 | 5.040647 | 4.64E-07 | 1.50E-05 | TRUE |
| B9D1 | 15602 | 5.037302 | 4.72E-07 | 1.52E-05 | TRUE |
| DYRK3 | 3068 | 5.034988 | 4.78E-07 | 1.53E-05 | TRUE |
| WDR86 | 3965 | 5.0309 | 4.88E-07 | 1.56E-05 | TRUE |
| ARHGAP28 | 1606 | 5.023772 | 5.07E-07 | 1.59E-05 | TRUE |
| CCDC85C | 1241 | 5.018068 | 5.22E-07 | 1.63E-05 | TRUE |
| KCNN3 | 7147 | 5.017342 | 5.24E-07 | 1.63E-05 | TRUE |
| FAM189A2 | 11336 | 5.015018 | 5.30E-07 | 1.64E-05 | TRUE |
| C5orf49 | 8450 | 5.011721 | 5.39E-07 | 1.66E-05 | TRUE |
| RPS18 | 2615 | 5.008014 | 5.50E-07 | 1.68E-05 | TRUE |
| CENPW | 10030 | 4.991963 | 5.98E-07 | 1.80E-05 | TRUE |
| INTS4 | 2686 | 4.991948 | 5.98E-07 | 1.80E-05 | TRUE |
| SUB1 | 7611 | 4.990999 | 6.01E-07 | 1.80E-05 | TRUE |
| RAB27B | 13079 | 4.988424 | 6.09E-07 | 1.82E-05 | TRUE |
| ACOT8 | 2826 | 4.984895 | 6.20E-07 | 1.85E-05 | TRUE |
| PACSIN1 | 6223 | 4.983696 | 6.24E-07 | 1.85E-05 | TRUE |
| NR4A3 | 3472 | 4.978336 | 6.41E-07 | 1.89E-05 | TRUE |
| NSG2 | 10851 | 4.974687 | 6.54E-07 | 1.92E-05 | TRUE |
| HTR7 | 5871 | 4.969496 | 6.71E-07 | 1.97E-05 | TRUE |
| MAGED1 | 7881 | 4.965309 | 6.86E-07 | 2.00E-05 | TRUE |
| LRP1B | 870 | 4.963597 | 6.92E-07 | 2.01E-05 | TRUE |
| KLK7 | 11445 | 4.937014 | 7.93E-07 | 2.25E-05 | TRUE |
| MYL12B | 5109 | 4.932906 | 8.10E-07 | 2.28E-05 | TRUE |
| ZMAT1 | 10571 | 4.927095 | 8.35E-07 | 2.34E-05 | TRUE |
| TIMM10 | 2231 | 4.926163 | 8.39E-07 | 2.35E-05 | TRUE |
| TIMP4 | 11757 | 4.915128 | 8.87E-07 | 2.45E-05 | TRUE |
| TNFAIP8L2 | 15094 | 4.914456 | 8.90E-07 | 2.45E-05 | TRUE |
| SNRNP27 | 8284 | 4.913453 | 8.95E-07 | 2.46E-05 | TRUE |
| GSS | 762 | 4.910993 | 9.06E-07 | 2.48E-05 | TRUE |
| SLC16A8 | 2265 | 4.907664 | 9.22E-07 | 2.51E-05 | TRUE |
| TMEM130 | 5801 | 4.906816 | 9.26E-07 | 2.52E-05 | TRUE |
| ATP2B4 | 2287 | 4.904423 | 9.37E-07 | 2.54E-05 | TRUE |
| PLB1 | 14370 | 4.903647 | 9.41E-07 | 2.55E-05 | TRUE |
| SSTR1 | 5972 | 4.903178 | 9.43E-07 | 2.55E-05 | TRUE |
| PDE2A | 14206 | 4.897432 | 9.71E-07 | 2.60E-05 | TRUE |
| AHRR | 6586 | 4.897377 | 9.71E-07 | 2.60E-05 | TRUE |
| TMEM108 | 4196 | 4.895451 | 9.81E-07 | 2.62E-05 | TRUE |
| ARMC10 | 2822 | 4.880928 | 1.06E-06 | 2.79E-05 | TRUE |
| WDR54 | 14659 | 4.880316 | 1.06E-06 | 2.79E-05 | TRUE |
| PDGFC | 4979 | 4.879195 | 1.07E-06 | 2.80E-05 | TRUE |
| FYB1 | 2421 | 4.878309 | 1.07E-06 | 2.81E-05 | TRUE |
| TMEM18 | 5568 | 4.876044 | 1.08E-06 | 2.83E-05 | TRUE |
| JMJD4 | 6992 | 4.873862 | 1.09E-06 | 2.86E-05 | TRUE |
| TMEM159 | 1289 | 4.864366 | 1.15E-06 | 2.97E-05 | TRUE |
| FARSB | 1056 | 4.851936 | 1.22E-06 | 3.14E-05 | TRUE |
| ANKRD6 | 7462 | 4.848766 | 1.24E-06 | 3.17E-05 | TRUE |
| JUN | 4424 | 4.845172 | 1.27E-06 | 3.22E-05 | TRUE |
| POLR3GL | 2098 | 4.824767 | 1.40E-06 | 3.48E-05 | TRUE |
| GSKIP | 1357 | 4.819895 | 1.44E-06 | 3.56E-05 | TRUE |
| MZT2A | 12803 | 4.818462 | 1.45E-06 | 3.58E-05 | TRUE |
| SPARC | 598 | 4.817935 | 1.45E-06 | 3.58E-05 | TRUE |
| RASAL3 | 7036 | 4.815556 | 1.47E-06 | 3.62E-05 | TRUE |
| LINC01140 | 12584 | 4.808134 | 1.52E-06 | 3.73E-05 | TRUE |
| FAM181A | 13583 | 4.805396 | 1.54E-06 | 3.77E-05 | TRUE |
| GPNMB | 1608 | 4.803948 | 1.56E-06 | 3.79E-05 | TRUE |
| ADGRG1 | 4787 | 4.801695 | 1.57E-06 | 3.81E-05 | TRUE |
| ZCCHC18 | 1113 | 4.798377 | 1.60E-06 | 3.87E-05 | TRUE |
| PRKCG | 13407 | 4.797714 | 1.60E-06 | 3.88E-05 | TRUE |
| ALOX5 | 3131 | 4.795993 | 1.62E-06 | 3.90E-05 | TRUE |
| PHPT1 | 8499 | 4.781059 | 1.74E-06 | 4.16E-05 | TRUE |
| SELENOW | 13985 | 4.775065 | 1.80E-06 | 4.27E-05 | TRUE |
| EDARADD | 701 | 4.77167 | 1.83E-06 | 4.33E-05 | TRUE |
| PYDC1 | 12559 | 4.76597 | 1.88E-06 | 4.44E-05 | TRUE |
| SH3RF1 | 4544 | 4.759682 | 1.94E-06 | 4.53E-05 | TRUE |
| DCAKD | 10554 | 4.757661 | 1.96E-06 | 4.56E-05 | TRUE |
| PSMB7 | 416 | 4.753516 | 2.00E-06 | 4.64E-05 | TRUE |
| CXorf57 | 14213 | 4.752837 | 2.01E-06 | 4.65E-05 | TRUE |
| CALML3 | 1264 | 4.746898 | 2.07E-06 | 4.75E-05 | TRUE |
| OPRM1 | 14371 | 4.744929 | 2.09E-06 | 4.79E-05 | TRUE |
| SCGN | 133 | 4.743776 | 2.10E-06 | 4.81E-05 | TRUE |
| BATF3 | 2450 | 4.740351 | 2.13E-06 | 4.87E-05 | TRUE |
| PTPRZ1 | 7882 | 4.740345 | 2.13E-06 | 4.87E-05 | TRUE |
| C1S | 10354 | 4.739432 | 2.14E-06 | 4.88E-05 | TRUE |
| ADTRP | 747 | 4.735768 | 2.18E-06 | 4.95E-05 | TRUE |
| MAPK1IP1L | 10617 | 4.730642 | 2.24E-06 | 5.05E-05 | TRUE |
| CCDC167 | 10139 | 4.730043 | 2.24E-06 | 5.06E-05 | TRUE |
| ABLIM3 | 9532 | 4.726884 | 2.28E-06 | 5.12E-05 | TRUE |
| GTF2F2 | 10429 | 4.724191 | 2.31E-06 | 5.18E-05 | TRUE |
| GLOD4 | 6614 | 4.721728 | 2.34E-06 | 5.22E-05 | TRUE |
| CMTM3 | 12591 | 4.714707 | 2.42E-06 | 5.36E-05 | TRUE |
| TG | 10352 | 4.709929 | 2.48E-06 | 5.46E-05 | TRUE |
| NUPR1 | 5394 | 4.707992 | 2.50E-06 | 5.49E-05 | TRUE |
| HGH1 | 4473 | 4.692628 | 2.70E-06 | 5.89E-05 | TRUE |
| POLR2G | 3822 | 4.68642 | 2.78E-06 | 6.05E-05 | TRUE |
| CDH11 | 13429 | 4.676868 | 2.91E-06 | 6.29E-05 | TRUE |
| MYB | 11384 | 4.675574 | 2.93E-06 | 6.32E-05 | TRUE |
| COPS4 | 841 | 4.674477 | 2.95E-06 | 6.33E-05 | TRUE |
| MSANTD1 | 5059 | 4.674441 | 2.95E-06 | 6.33E-05 | TRUE |
| LOC440934 | 4793 | 4.672888 | 2.97E-06 | 6.37E-05 | TRUE |
| CHCHD6 | 2417 | 4.671938 | 2.98E-06 | 6.38E-05 | TRUE |
| RPS14 | 11483 | 4.671841 | 2.99E-06 | 6.38E-05 | TRUE |
| ENO1 | 4340 | 4.666948 | 3.06E-06 | 6.52E-05 | TRUE |
| FXYD6 | 6841 | 4.665483 | 3.08E-06 | 6.55E-05 | TRUE |
| RER1 | 12250 | 4.658416 | 3.19E-06 | 6.74E-05 | TRUE |
| RTL8C | 10856 | 4.657984 | 3.19E-06 | 6.75E-05 | TRUE |
| FABP5P3 | 7443 | 4.654216 | 3.25E-06 | 6.86E-05 | TRUE |
| POLR2L | 3143 | 4.654048 | 3.25E-06 | 6.86E-05 | TRUE |
| RPL10A | 4589 | 4.652187 | 3.28E-06 | 6.91E-05 | TRUE |
| FAM107A | 9466 | 4.648985 | 3.34E-06 | 7.01E-05 | TRUE |
| ERICH6-AS1 | 1897 | 4.648077 | 3.35E-06 | 7.03E-05 | TRUE |
| HTR7P1 | 8785 | 4.641782 | 3.45E-06 | 7.23E-05 | TRUE |
| KCNG1 | 9500 | 4.639124 | 3.50E-06 | 7.30E-05 | TRUE |
| SMIM10L2B | 11583 | 4.637201 | 3.53E-06 | 7.35E-05 | TRUE |
| SMIM29 | 3640 | 4.635813 | 3.56E-06 | 7.39E-05 | TRUE |
| FZD8 | 6786 | 4.635671 | 3.56E-06 | 7.39E-05 | TRUE |
| PKIA | 14970 | 4.634851 | 3.57E-06 | 7.41E-05 | TRUE |
| PYGL | 11803 | 4.630958 | 3.64E-06 | 7.54E-05 | TRUE |
| LRRC3B | 1632 | 4.628552 | 3.68E-06 | 7.61E-05 | TRUE |
| NFKBIE | 5718 | 4.627153 | 3.71E-06 | 7.66E-05 | TRUE |
| NADK2 | 1570 | 4.620417 | 3.83E-06 | 7.85E-05 | TRUE |
| CENPVL3 | 4373 | 4.609682 | 4.03E-06 | 8.21E-05 | TRUE |
| S100A10 | 6199 | 4.609148 | 4.04E-06 | 8.22E-05 | TRUE |
| LSM4 | 14729 | 4.599126 | 4.24E-06 | 8.56E-05 | TRUE |
| RPL41 | 10115 | 4.597108 | 4.28E-06 | 8.61E-05 | TRUE |
| TSPAN2 | 6221 | 4.595554 | 4.32E-06 | 8.66E-05 | TRUE |
| FKBP1B | 8241 | 4.593267 | 4.36E-06 | 8.72E-05 | TRUE |
| TIMM8B | 11914 | 4.589688 | 4.44E-06 | 8.85E-05 | TRUE |
| ME1 | 6298 | 4.58837 | 4.47E-06 | 8.88E-05 | TRUE |
| WFDC1 | 3189 | 4.587419 | 4.49E-06 | 8.91E-05 | TRUE |
| EIF1 | 9053 | 4.585432 | 4.53E-06 | 8.98E-05 | TRUE |
| EMG1 | 210 | 4.583624 | 4.57E-06 | 9.04E-05 | TRUE |
| IGFN1 | 9463 | 4.583281 | 4.58E-06 | 9.05E-05 | TRUE |
| PPP1R1A | 2878 | 4.5827 | 4.59E-06 | 9.06E-05 | TRUE |
| TBCB | 5359 | 4.571234 | 4.85E-06 | 9.51E-05 | TRUE |
| HNRNPC | 12943 | 4.559545 | 5.13E-06 | 0.0001002 | TRUE |
| LOC440434 | 11985 | 4.559256 | 5.13E-06 | 0.0001002 | TRUE |
| ELL3 | 4456 | 4.543415 | 5.54E-06 | 0.0001071 | TRUE |
| ANOS1 | 1128 | 4.539341 | 5.64E-06 | 0.0001086 | TRUE |
| C3 | 2478 | 4.537262 | 5.70E-06 | 0.000109 | TRUE |
| CPT1C | 13634 | 4.528817 | 5.93E-06 | 0.0001128 | TRUE |
| DNAAF4 | 8230 | 4.528203 | 5.95E-06 | 0.0001129 | TRUE |
| TINCR | 12919 | 4.524908 | 6.04E-06 | 0.0001145 | TRUE |
| SH3BP5 | 1370 | 4.523605 | 6.08E-06 | 0.0001151 | TRUE |
| PEMT | 14801 | 4.521225 | 6.15E-06 | 0.0001162 | TRUE |
| HEBP1 | 9126 | 4.518266 | 6.23E-06 | 0.0001177 | TRUE |
| AMIGO2 | 7272 | 4.515962 | 6.30E-06 | 0.0001189 | TRUE |
| KCTD4 | 9987 | 4.513897 | 6.36E-06 | 0.0001199 | TRUE |
| FNBP1L | 11462 | 4.513557 | 6.37E-06 | 0.0001199 | TRUE |
| CPAMD8 | 4185 | 4.510295 | 6.47E-06 | 0.0001213 | TRUE |
| WNT10B | 9266 | 4.506233 | 6.60E-06 | 0.0001235 | TRUE |
| RPS4X | 5212 | 4.504388 | 6.66E-06 | 0.0001242 | TRUE |
| PCDH19 | 14264 | 4.504304 | 6.66E-06 | 0.0001242 | TRUE |
| LINC02217 | 11061 | 4.501763 | 6.74E-06 | 0.0001254 | TRUE |
| LSM7 | 3388 | 4.498434 | 6.85E-06 | 0.0001269 | TRUE |
| STOML1 | 461 | 4.49581 | 6.93E-06 | 0.0001284 | TRUE |
| IL17RD | 10657 | 4.492889 | 7.03E-06 | 0.0001297 | TRUE |
| TKFC | 2627 | 4.491894 | 7.06E-06 | 0.0001301 | TRUE |
| CCKBR | 14395 | 4.491725 | 7.06E-06 | 0.0001301 | TRUE |
| GPC4 | 14625 | 4.488374 | 7.18E-06 | 0.0001318 | TRUE |
| DOC2B | 10601 | 4.48719 | 7.22E-06 | 0.0001324 | TRUE |
| TBCA | 10826 | 4.486655 | 7.24E-06 | 0.0001326 | TRUE |
| THTPA | 7854 | 4.483787 | 7.33E-06 | 0.0001339 | TRUE |
| CDH4 | 15091 | 4.479063 | 7.50E-06 | 0.0001364 | TRUE |
| RAVER2 | 6830 | 4.478954 | 7.50E-06 | 0.0001364 | TRUE |
| SNTB1 | 10015 | 4.478002 | 7.53E-06 | 0.0001368 | TRUE |
| GABRA5 | 1450 | 4.469617 | 7.84E-06 | 0.000141 | TRUE |
| ERFE | 12523 | 4.460981 | 8.16E-06 | 0.0001451 | TRUE |
| MCUR1 | 4511 | 4.460812 | 8.16E-06 | 0.0001451 | TRUE |
| RBP4 | 8624 | 4.460025 | 8.20E-06 | 0.0001454 | TRUE |
| LOC100507516 | 12960 | 4.459545 | 8.21E-06 | 0.0001456 | TRUE |
| MGMT | 5354 | 4.4592 | 8.23E-06 | 0.0001456 | TRUE |
| DDRGK1 | 7518 | 4.455831 | 8.36E-06 | 0.0001473 | TRUE |
| PACRG | 9664 | 4.453515 | 8.45E-06 | 0.0001486 | TRUE |
| SFTPD | 13891 | 4.448191 | 8.66E-06 | 0.0001516 | TRUE |
| LARGE1 | 4139 | 4.44592 | 8.75E-06 | 0.000153 | TRUE |
| ARHGAP4 | 6049 | 4.445607 | 8.76E-06 | 0.0001531 | TRUE |
| P2RY13 | 10493 | 4.436931 | 9.13E-06 | 0.0001589 | TRUE |
| PLPPR4 | 6398 | 4.434424 | 9.23E-06 | 0.0001604 | TRUE |
| PLD6 | 2063 | 4.432446 | 9.32E-06 | 0.0001617 | TRUE |
| TYROBP | 9569 | 4.428532 | 9.49E-06 | 0.0001641 | TRUE |
| SLC16A2 | 4225 | 4.42602 | 9.60E-06 | 0.0001653 | TRUE |
| THBS4 | 8997 | 4.4241 | 9.68E-06 | 0.0001666 | TRUE |
| PLPPR2 | 10031 | 4.41892 | 9.92E-06 | 0.0001702 | TRUE |
| C11orf97 | 4062 | 4.418323 | 9.95E-06 | 0.0001704 | TRUE |
| PRDX6 | 7321 | 4.417972 | 9.96E-06 | 0.0001704 | TRUE |
| ATOX1 | 5273 | 4.411708 | 1.03E-05 | 0.0001745 | TRUE |
| FUOM | 12000 | 4.410438 | 1.03E-05 | 0.0001753 | TRUE |
| HLA-DMB | 10051 | 4.408237 | 1.04E-05 | 0.0001765 | TRUE |
| HCG23 | 821 | 4.406667 | 1.05E-05 | 0.0001776 | TRUE |
| DPCD | 5348 | 4.403076 | 1.07E-05 | 0.00018 | TRUE |
| DRAXIN | 2402 | 4.402655 | 1.07E-05 | 0.0001801 | TRUE |
| CPNE7 | 7021 | 4.402162 | 1.07E-05 | 0.0001802 | TRUE |
| DACH2 | 4214 | 4.401735 | 1.07E-05 | 0.0001803 | TRUE |
| HCST | 14845 | 4.401326 | 1.08E-05 | 0.0001805 | TRUE |
| LSM3 | 14192 | 4.395436 | 1.11E-05 | 0.0001848 | TRUE |
| DBI | 7270 | 4.393876 | 1.11E-05 | 0.0001856 | TRUE |
| SQLE | 3049 | 4.392553 | 1.12E-05 | 0.0001865 | TRUE |
| PIRT | 11051 | 4.390393 | 1.13E-05 | 0.000188 | TRUE |
| TMEM208 | 5482 | 4.383854 | 1.17E-05 | 0.0001925 | TRUE |
| NUDT10 | 14293 | 4.383655 | 1.17E-05 | 0.0001925 | TRUE |
| PDGFRA | 9047 | 4.383265 | 1.17E-05 | 0.0001926 | TRUE |
| DPF1 | 5788 | 4.3826 | 1.17E-05 | 0.000193 | TRUE |
| ZSCAN18 | 3564 | 4.380993 | 1.18E-05 | 0.0001938 | TRUE |
| SPHKAP | 7058 | 4.380794 | 1.18E-05 | 0.0001938 | TRUE |
| FKBP1A | 11347 | 4.377856 | 1.20E-05 | 0.0001961 | TRUE |
| MUM1L1 | 14409 | 4.375133 | 1.21E-05 | 0.000198 | TRUE |
| DDAH1 | 12878 | 4.374895 | 1.21E-05 | 0.000198 | TRUE |
| UBE2L5 | 1021 | 4.374083 | 1.22E-05 | 0.0001984 | TRUE |
| MANF | 8806 | 4.365976 | 1.27E-05 | 0.0002046 | TRUE |
| GRID2 | 12732 | 4.363721 | 1.28E-05 | 0.0002063 | TRUE |
| SHISAL1 | 3607 | 4.363468 | 1.28E-05 | 0.0002063 | TRUE |
| OLFML2B | 12618 | 4.36268 | 1.28E-05 | 0.0002069 | TRUE |
| FZD1 | 3547 | 4.358453 | 1.31E-05 | 0.0002107 | TRUE |
| GLG1 | 4423 | 4.357539 | 1.32E-05 | 0.0002113 | TRUE |
| ADGRA3 | 5993 | 4.357304 | 1.32E-05 | 0.0002113 | TRUE |
| PHYHD1 | 1292 | 4.35396 | 1.34E-05 | 0.0002137 | TRUE |
| LOC728392 | 3692 | 4.353572 | 1.34E-05 | 0.0002139 | TRUE |
| HPCAL1 | 6027 | 4.353247 | 1.34E-05 | 0.000214 | TRUE |
| CRMP1 | 2515 | 4.351719 | 1.35E-05 | 0.000215 | TRUE |
| COG1 | 12556 | 4.349301 | 1.37E-05 | 0.0002168 | TRUE |
| UQCC2 | 7472 | 4.340415 | 1.42E-05 | 0.0002253 | TRUE |
| KXD1 | 12041 | 4.338994 | 1.43E-05 | 0.000226 | TRUE |
| ALDOA | 7692 | 4.335281 | 1.46E-05 | 0.0002295 | TRUE |
| PSORS1C1 | 9869 | 4.335156 | 1.46E-05 | 0.0002295 | TRUE |
| PSMB1 | 11212 | 4.333723 | 1.47E-05 | 0.0002308 | TRUE |
| GDA | 13756 | 4.332313 | 1.48E-05 | 0.0002318 | TRUE |
| PNPLA3 | 9562 | 4.33074 | 1.49E-05 | 0.0002333 | TRUE |
| LCP1 | 2687 | 4.326326 | 1.52E-05 | 0.0002377 | TRUE |
| CMTM8 | 12430 | 4.323861 | 1.53E-05 | 0.0002394 | TRUE |
| C2CD4C | 9784 | 4.323086 | 1.54E-05 | 0.0002398 | TRUE |
| PEA15 | 1997 | 4.322186 | 1.54E-05 | 0.0002406 | TRUE |
| CTNND2 | 11324 | 4.320633 | 1.56E-05 | 0.0002418 | TRUE |
| GTF2H5 | 14420 | 4.318676 | 1.57E-05 | 0.0002437 | TRUE |
| LOC93622 | 4992 | 4.315157 | 1.59E-05 | 0.0002469 | TRUE |
| KRT14 | 7974 | 4.308152 | 1.65E-05 | 0.0002538 | TRUE |
| LINGO1 | 11831 | 4.303013 | 1.68E-05 | 0.0002587 | TRUE |
| ANXA1 | 9727 | 4.299707 | 1.71E-05 | 0.0002616 | TRUE |
| SLC39A4 | 12952 | 4.299036 | 1.72E-05 | 0.0002621 | TRUE |
| CCDC8 | 7342 | 4.29843 | 1.72E-05 | 0.0002623 | TRUE |
| LRRN2 | 11119 | 4.295723 | 1.74E-05 | 0.0002645 | TRUE |
| CDH13 | 13775 | 4.292736 | 1.76E-05 | 0.0002672 | TRUE |
| DCAF5 | 11321 | 4.292665 | 1.77E-05 | 0.0002672 | TRUE |
| GPR34 | 8396 | 4.289816 | 1.79E-05 | 0.0002702 | TRUE |
| C1orf61 | 14084 | 4.288609 | 1.80E-05 | 0.000271 | TRUE |
| SLC26A11 | 3479 | 4.282765 | 1.85E-05 | 0.0002769 | TRUE |
| FTH1 | 3521 | 4.2798 | 1.87E-05 | 0.0002796 | TRUE |
| RPL32 | 7499 | 4.277913 | 1.89E-05 | 0.0002814 | TRUE |
| SIL1 | 11398 | 4.276708 | 1.90E-05 | 0.0002824 | TRUE |
| MAPK3 | 11866 | 4.274843 | 1.91E-05 | 0.0002845 | TRUE |
| STOM | 11068 | 4.2737 | 1.92E-05 | 0.0002855 | TRUE |
| CDKN2D | 12708 | 4.269342 | 1.96E-05 | 0.0002905 | TRUE |
| CRHBP | 11341 | 4.269055 | 1.96E-05 | 0.0002906 | TRUE |
| BPHL | 861 | 4.266014 | 1.99E-05 | 0.0002935 | TRUE |
| PEG10 | 3077 | 4.265011 | 2.00E-05 | 0.0002945 | TRUE |
| RAB36 | 2412 | 4.26349 | 2.01E-05 | 0.0002957 | TRUE |
| ABCC6 | 4701 | 4.261471 | 2.03E-05 | 0.0002978 | TRUE |
| TRAPPC6A | 232 | 4.259507 | 2.05E-05 | 0.0003002 | TRUE |
| CCDC189 | 9905 | 4.258854 | 2.05E-05 | 0.0003008 | TRUE |
| METTL6 | 9912 | 4.25847 | 2.06E-05 | 0.000301 | TRUE |
| ZNF436 | 6110 | 4.257522 | 2.07E-05 | 0.000302 | TRUE |
| RAPGEF4 | 11325 | 4.255246 | 2.09E-05 | 0.0003045 | TRUE |
| FAM120AOS | 10475 | 4.254682 | 2.09E-05 | 0.000305 | TRUE |
| CPLX3 | 13286 | 4.254064 | 2.10E-05 | 0.0003056 | TRUE |
| C12orf75 | 8777 | 4.253446 | 2.11E-05 | 0.0003058 | TRUE |
| FADS2 | 8926 | 4.251474 | 2.12E-05 | 0.000308 | TRUE |
| DYNLL1 | 7406 | 4.249369 | 2.14E-05 | 0.0003103 | TRUE |
| TMSB10 | 9449 | 4.248566 | 2.15E-05 | 0.0003108 | TRUE |
| STIM1 | 14117 | 4.246966 | 2.17E-05 | 0.0003128 | TRUE |
| GNB4 | 11644 | 4.246513 | 2.17E-05 | 0.0003128 | TRUE |
| GRIN3A | 2610 | 4.24591 | 2.18E-05 | 0.0003134 | TRUE |
| HDC | 13671 | 4.24419 | 2.19E-05 | 0.0003149 | TRUE |
| CCBE1 | 7607 | 4.242599 | 2.21E-05 | 0.0003169 | TRUE |
| CHID1 | 10549 | 4.237907 | 2.26E-05 | 0.0003227 | TRUE |
| BAIAP3 | 10081 | 4.236365 | 2.27E-05 | 0.0003242 | TRUE |
| LOC645166 | 2464 | 4.233554 | 2.30E-05 | 0.0003275 | TRUE |
| LUZP2 | 13528 | 4.233323 | 2.30E-05 | 0.0003275 | TRUE |
| RAB3B | 8277 | 4.232413 | 2.31E-05 | 0.0003286 | TRUE |
| TSTD1 | 357 | 4.231622 | 2.32E-05 | 0.0003294 | TRUE |
| SMAD9 | 14956 | 4.227644 | 2.36E-05 | 0.0003338 | TRUE |
| MYH15 | 14706 | 4.227091 | 2.37E-05 | 0.0003341 | TRUE |
| CHRNB1 | 8276 | 4.22696 | 2.37E-05 | 0.0003341 | TRUE |
| TMEM196 | 1531 | 4.22391 | 2.40E-05 | 0.0003375 | TRUE |
| SH3BGRL3 | 11380 | 4.220162 | 2.44E-05 | 0.0003423 | TRUE |
| SSR4 | 1265 | 4.21939 | 2.45E-05 | 0.0003431 | TRUE |
| RPL29P2 | 11711 | 4.219027 | 2.45E-05 | 0.0003431 | TRUE |
| LOC100507351 | 5684 | 4.217265 | 2.47E-05 | 0.0003455 | TRUE |
| SUMO3 | 2358 | 4.217073 | 2.47E-05 | 0.0003455 | TRUE |
| DOC2A | 8125 | 4.215502 | 2.49E-05 | 0.0003471 | TRUE |
| PCDH20 | 13674 | 4.214521 | 2.50E-05 | 0.0003473 | TRUE |
| DNALI1 | 11878 | 4.214178 | 2.51E-05 | 0.0003473 | TRUE |
| ITGB2 | 2389 | 4.213357 | 2.52E-05 | 0.0003478 | TRUE |
| FABP5 | 8032 | 4.208294 | 2.57E-05 | 0.0003553 | TRUE |
| MT1F | 8994 | 4.205806 | 2.60E-05 | 0.0003578 | TRUE |
| ARL3 | 11941 | 4.205735 | 2.60E-05 | 0.0003578 | TRUE |
| SUSD1 | 7091 | 4.203351 | 2.63E-05 | 0.0003603 | TRUE |
| SOX4 | 8275 | 4.200403 | 2.66E-05 | 0.0003646 | TRUE |
| CCR1 | 15337 | 4.198575 | 2.69E-05 | 0.0003661 | TRUE |
| CNR1 | 12150 | 4.19699 | 2.70E-05 | 0.000368 | TRUE |
| LINC00320 | 11312 | 4.196375 | 2.71E-05 | 0.0003687 | TRUE |
| EXOC6 | 2831 | 4.195766 | 2.72E-05 | 0.0003689 | TRUE |
| HSBP1 | 7775 | 4.195248 | 2.73E-05 | 0.0003689 | TRUE |
| FBL | 3876 | 4.194733 | 2.73E-05 | 0.0003694 | TRUE |
| HES1 | 4308 | 4.190735 | 2.78E-05 | 0.000375 | TRUE |
| FAM228B | 1285 | 4.19041 | 2.78E-05 | 0.0003753 | TRUE |
| TEKT2 | 3230 | 4.188096 | 2.81E-05 | 0.0003785 | TRUE |
| LOC101060391 | 11356 | 4.187812 | 2.82E-05 | 0.0003786 | TRUE |
| IGF1 | 13274 | 4.186516 | 2.83E-05 | 0.0003804 | TRUE |
| NBDY | 266 | 4.186231 | 2.84E-05 | 0.0003806 | TRUE |
| TOM1L1 | 8208 | 4.184255 | 2.86E-05 | 0.0003829 | TRUE |
| KCNK2 | 7163 | 4.183573 | 2.87E-05 | 0.0003838 | TRUE |
| PLTP | 5377 | 4.183154 | 2.87E-05 | 0.0003841 | TRUE |
| PNCK | 8451 | 4.181941 | 2.89E-05 | 0.0003859 | TRUE |
| PNMT | 3473 | 4.178942 | 2.93E-05 | 0.00039 | TRUE |
| RYR1 | 14821 | 4.177174 | 2.95E-05 | 0.000392 | TRUE |
| B4GAT1 | 11333 | 4.175758 | 2.97E-05 | 0.0003941 | TRUE |
| HGF | 5340 | 4.172978 | 3.01E-05 | 0.0003986 | TRUE |
| MT1E | 7208 | 4.17268 | 3.01E-05 | 0.0003988 | TRUE |
| TCEA3 | 13753 | 4.172504 | 3.01E-05 | 0.0003988 | TRUE |
| CDO1 | 8388 | 4.168626 | 3.06E-05 | 0.0004045 | TRUE |
| FGGY | 12773 | 4.16813 | 3.07E-05 | 0.0004047 | TRUE |
| FAM117A | 6738 | 4.166846 | 3.09E-05 | 0.0004061 | TRUE |
| CETN2 | 11327 | 4.166294 | 3.10E-05 | 0.0004067 | TRUE |
| TMEM17 | 9909 | 4.164842 | 3.12E-05 | 0.0004086 | TRUE |
| NTSR1 | 8043 | 4.164376 | 3.12E-05 | 0.0004088 | TRUE |
| B3GALT6 | 8129 | 4.164372 | 3.12E-05 | 0.0004088 | TRUE |
| CPNE5 | 4909 | 4.162266 | 3.15E-05 | 0.0004115 | TRUE |
| SMIM10L2A | 9428 | 4.158271 | 3.21E-05 | 0.0004181 | TRUE |
| SCUBE2 | 11067 | 4.157945 | 3.21E-05 | 0.0004183 | TRUE |
| ADRA1B | 1951 | 4.156169 | 3.24E-05 | 0.000421 | TRUE |
| C1QA | 14113 | 4.156131 | 3.24E-05 | 0.000421 | TRUE |
| ST6GALNAC5 | 9725 | 4.155587 | 3.24E-05 | 0.0004213 | TRUE |
| CADM1 | 2830 | 4.154377 | 3.26E-05 | 0.0004232 | TRUE |
| SLIT3 | 1518 | 4.153655 | 3.27E-05 | 0.0004238 | TRUE |
| CNIH2 | 6389 | 4.148482 | 3.35E-05 | 0.0004328 | TRUE |
| CIB2 | 8273 | 4.148147 | 3.35E-05 | 0.000433 | TRUE |
| RPL24 | 10602 | 4.13944 | 3.48E-05 | 0.000448 | TRUE |
| GMIP | 4291 | 4.138526 | 3.50E-05 | 0.0004494 | TRUE |
| KLHL1 | 1220 | 4.138284 | 3.50E-05 | 0.0004495 | TRUE |
| PUSL1 | 2749 | 4.135423 | 3.54E-05 | 0.0004537 | TRUE |
| MGST1 | 4056 | 4.135401 | 3.54E-05 | 0.0004537 | TRUE |
| GMFG | 8784 | 4.134058 | 3.56E-05 | 0.000456 | TRUE |
| SIGIRR | 3575 | 4.131831 | 3.60E-05 | 0.0004594 | TRUE |
| PPDPF | 13380 | 4.127379 | 3.67E-05 | 0.0004679 | TRUE |
| WDFY4 | 5448 | 4.126582 | 3.68E-05 | 0.0004689 | TRUE |
| ONECUT1 | 13545 | 4.124272 | 3.72E-05 | 0.0004723 | TRUE |
| MAP1LC3A | 15032 | 4.123539 | 3.73E-05 | 0.0004733 | TRUE |
| GABARAP | 1263 | 4.119612 | 3.80E-05 | 0.0004796 | TRUE |
| TFPT | 10518 | 4.117324 | 3.83E-05 | 0.0004832 | TRUE |
| TUBB2A | 13058 | 4.113994 | 3.89E-05 | 0.0004884 | TRUE |
| C1orf50 | 6231 | 4.112987 | 3.91E-05 | 0.0004892 | TRUE |
| ADCY2 | 8653 | 4.11207 | 3.92E-05 | 0.0004908 | TRUE |
| RTN4RL2 | 3894 | 4.106796 | 4.01E-05 | 0.0005013 | TRUE |
| C8orf46 | 8371 | 4.105282 | 4.04E-05 | 0.0005038 | TRUE |
| LINC01094 | 7174 | 4.104321 | 4.06E-05 | 0.0005051 | TRUE |
| FAH | 12481 | 4.101694 | 4.10E-05 | 0.0005105 | TRUE |
| DNAJA4 | 923 | 4.098794 | 4.15E-05 | 0.0005149 | TRUE |
| GTF3C6 | 7688 | 4.09726 | 4.18E-05 | 0.0005175 | TRUE |
| RPL39L | 10832 | 4.096494 | 4.19E-05 | 0.0005186 | TRUE |
| TSPAN18 | 5505 | 4.096037 | 4.20E-05 | 0.000519 | TRUE |
| EFHC2 | 12328 | 4.095683 | 4.21E-05 | 0.0005194 | TRUE |
| PLXNC1 | 773 | 4.093218 | 4.25E-05 | 0.0005245 | TRUE |
| CELF5 | 3394 | 4.092477 | 4.27E-05 | 0.0005258 | TRUE |
| MACROD2 | 2121 | 4.091515 | 4.29E-05 | 0.0005275 | TRUE |
| CBFB | 1463 | 4.088464 | 4.34E-05 | 0.0005337 | TRUE |
| FDXR | 2978 | 4.086505 | 4.38E-05 | 0.0005374 | TRUE |
| KLF6 | 13595 | 4.083377 | 4.44E-05 | 0.0005438 | TRUE |
| SPATA33 | 11112 | 4.08125 | 4.48E-05 | 0.000548 | TRUE |
| MEIS3 | 475 | 4.081244 | 4.48E-05 | 0.000548 | TRUE |
| ITGB7 | 1340 | 4.07915 | 4.52E-05 | 0.0005516 | TRUE |
| XKR4 | 5118 | 4.078793 | 4.53E-05 | 0.0005516 | TRUE |
| UBA52 | 12399 | 4.075835 | 4.58E-05 | 0.0005574 | TRUE |
| PCOLCE2 | 4210 | 4.071162 | 4.68E-05 | 0.0005678 | TRUE |
| BTG3 | 4512 | 4.069 | 4.72E-05 | 0.0005726 | TRUE |
| SLC35F2 | 10228 | 4.063481 | 4.83E-05 | 0.0005832 | TRUE |
| OPRK1 | 12146 | 4.060715 | 4.89E-05 | 0.0005892 | TRUE |
| CDC42BPB | 12439 | 4.058985 | 4.93E-05 | 0.0005931 | TRUE |
| SMIM4 | 1252 | 4.055903 | 4.99E-05 | 0.0005997 | TRUE |
| HSPB11 | 12989 | 4.055894 | 4.99E-05 | 0.0005997 | TRUE |
| FAR2P2 | 7903 | 4.050334 | 5.11E-05 | 0.0006117 | TRUE |
| CD63 | 7780 | 4.049228 | 5.14E-05 | 0.0006142 | TRUE |
| NPC2 | 5808 | 4.047662 | 5.17E-05 | 0.0006173 | TRUE |
| MEA1 | 10060 | 4.042583 | 5.29E-05 | 0.0006294 | TRUE |
| LRRC42 | 1122 | 4.042235 | 5.29E-05 | 0.0006295 | TRUE |
| EMP1 | 9418 | 4.041374 | 5.31E-05 | 0.0006311 | TRUE |
| IGSF22 | 13603 | 4.033321 | 5.50E-05 | 0.0006481 | TRUE |
| UBL5 | 1713 | 4.032946 | 5.51E-05 | 0.0006484 | TRUE |
| GNAQ | 10753 | 4.030026 | 5.58E-05 | 0.0006555 | TRUE |
| GNG4 | 9988 | 4.028806 | 5.61E-05 | 0.0006585 | TRUE |
| E2F5 | 3779 | 4.028227 | 5.62E-05 | 0.0006596 | TRUE |
| DHRS2 | 10660 | 4.025442 | 5.69E-05 | 0.0006659 | TRUE |
| FOLR2 | 10717 | 4.023188 | 5.74E-05 | 0.0006703 | TRUE |
| HRAS | 5487 | 4.022601 | 5.76E-05 | 0.0006713 | TRUE |
| SNTG2 | 2186 | 4.02073 | 5.80E-05 | 0.0006759 | TRUE |
| MESP1 | 6359 | 4.020536 | 5.81E-05 | 0.0006759 | TRUE |
| AKIRIN2 | 10516 | 4.014075 | 5.97E-05 | 0.0006926 | TRUE |
| PLCH1 | 11402 | 4.011245 | 6.04E-05 | 0.0006994 | TRUE |
| NEBL-AS1 | 2998 | 4.009635 | 6.08E-05 | 0.0007035 | TRUE |
| TUBA1B | 12159 | 4.009181 | 6.09E-05 | 0.000704 | TRUE |
| BRK1 | 7690 | 4.00795 | 6.12E-05 | 0.0007066 | TRUE |
| TRIM36 | 5085 | 4.005876 | 6.18E-05 | 0.0007118 | TRUE |
| PDCD5 | 10274 | 4.005302 | 6.19E-05 | 0.0007128 | TRUE |
| LOXL3 | 8484 | 4.005147 | 6.20E-05 | 0.0007128 | TRUE |
| DOK1 | 13092 | 4.005032 | 6.20E-05 | 0.0007128 | TRUE |
| DYDC2 | 13352 | 4.003242 | 6.25E-05 | 0.0007177 | TRUE |
| SLC7A4 | 4590 | 4.002396 | 6.27E-05 | 0.0007192 | TRUE |
| LDOC1 | 6237 | 4.000078 | 6.33E-05 | 0.0007247 | TRUE |
| C22orf23 | 8101 | 3.999158 | 6.36E-05 | 0.000727 | TRUE |
| ENC1 | 10242 | 3.996472 | 6.43E-05 | 0.0007337 | TRUE |
| GATB | 15023 | 3.996445 | 6.43E-05 | 0.0007337 | TRUE |
| DHRS9 | 10044 | 3.994036 | 6.50E-05 | 0.0007396 | TRUE |
| VAMP8 | 14178 | 3.993256 | 6.52E-05 | 0.0007415 | TRUE |
| CA10 | 7203 | 3.988833 | 6.64E-05 | 0.0007539 | TRUE |
| GABRA3 | 13461 | 3.988758 | 6.64E-05 | 0.0007539 | TRUE |
| CX3CR1 | 4134 | 3.988644 | 6.65E-05 | 0.0007539 | TRUE |
| TMEM200A | 12983 | 3.987703 | 6.67E-05 | 0.0007563 | TRUE |
| TSEN34 | 1839 | 3.985155 | 6.74E-05 | 0.0007634 | TRUE |
| ISG15 | 10311 | 3.984592 | 6.76E-05 | 0.0007646 | TRUE |
| UNC5B-AS1 | 11170 | 3.983082 | 6.80E-05 | 0.0007678 | TRUE |
| AKR7L | 9272 | 3.982547 | 6.82E-05 | 0.000769 | TRUE |
| FLRT1 | 4321 | 3.982339 | 6.82E-05 | 0.000769 | TRUE |
| TUSC3 | 11531 | 3.982201 | 6.83E-05 | 0.000769 | TRUE |
| NKAIN2 | 10527 | 3.982019 | 6.83E-05 | 0.0007691 | TRUE |
| IL1B | 2580 | 3.98035 | 6.88E-05 | 0.0007728 | TRUE |
| SOWAHA | 13349 | 3.978389 | 6.94E-05 | 0.0007775 | TRUE |
| B3GALNT1 | 1032 | 3.976786 | 6.99E-05 | 0.0007817 | TRUE |
| GRIK2 | 956 | 3.972865 | 7.10E-05 | 0.0007918 | TRUE |
| THRA | 993 | 3.970268 | 7.18E-05 | 0.0007982 | TRUE |
| LINC00900 | 7179 | 3.969562 | 7.20E-05 | 0.0008 | TRUE |
| FREM3 | 6784 | 3.968133 | 7.24E-05 | 0.0008031 | TRUE |
| DNAL4 | 1772 | 3.967297 | 7.27E-05 | 0.0008048 | TRUE |
| AGA | 468 | 3.967082 | 7.28E-05 | 0.000805 | TRUE |
| EIF4EBP1 | 10767 | 3.966144 | 7.30E-05 | 0.0008076 | TRUE |
| 8-Mar | 6424 | 3.961125 | 7.46E-05 | 0.0008213 | TRUE |
| DNAH2 | 5881 | 3.960613 | 7.48E-05 | 0.0008223 | TRUE |
| NLE1 | 7604 | 3.95826 | 7.55E-05 | 0.0008288 | TRUE |
| IPO5P1 | 2319 | 3.957101 | 7.59E-05 | 0.0008317 | TRUE |
| MT2A | 3780 | 3.955868 | 7.63E-05 | 0.0008342 | TRUE |
| ARHGAP36 | 3928 | 3.955013 | 7.65E-05 | 0.0008355 | TRUE |
| GBA | 13912 | 3.951185 | 7.78E-05 | 0.0008466 | TRUE |
| SPACA9 | 1296 | 3.950242 | 7.81E-05 | 0.0008488 | TRUE |
| RPA2 | 5843 | 3.946725 | 7.92E-05 | 0.0008601 | TRUE |
| EDF1 | 6011 | 3.944907 | 7.98E-05 | 0.0008631 | TRUE |
| LOC102724156 | 14700 | 3.94293 | 8.05E-05 | 0.0008684 | TRUE |
| LOC642852 | 11860 | 3.937194 | 8.24E-05 | 0.000887 | TRUE |
| SEMA6B | 149 | 3.936409 | 8.27E-05 | 0.0008885 | TRUE |
| SUSD3 | 5061 | 3.934489 | 8.34E-05 | 0.000894 | TRUE |
| COL21A1 | 11166 | 3.934181 | 8.35E-05 | 0.0008945 | TRUE |
| TCTA | 14779 | 3.932926 | 8.39E-05 | 0.0008967 | TRUE |
| ANO3 | 9291 | 3.932901 | 8.39E-05 | 0.0008967 | TRUE |
| CDC42 | 13306 | 3.930489 | 8.48E-05 | 0.0009028 | TRUE |
| TMEM132A | 12966 | 3.92617 | 8.63E-05 | 0.0009172 | TRUE |
| FOXF2 | 8056 | 3.921867 | 8.79E-05 | 0.0009306 | TRUE |
| APOM | 3753 | 3.920503 | 8.84E-05 | 0.0009327 | TRUE |
| ADAMTS9 | 8403 | 3.918021 | 8.93E-05 | 0.0009411 | TRUE |
| FAM200B | 512 | 3.916778 | 8.97E-05 | 0.0009445 | TRUE |
| MT1L | 1814 | 3.916629 | 8.98E-05 | 0.0009445 | TRUE |
| PTPRA | 3770 | 3.916518 | 8.98E-05 | 0.0009445 | TRUE |
| NPDC1 | 1529 | 3.912929 | 9.12E-05 | 0.0009573 | TRUE |
| RIMBP2 | 4442 | 3.912167 | 9.15E-05 | 0.0009584 | TRUE |
| EDNRB | 12052 | 3.912165 | 9.15E-05 | 0.0009584 | TRUE |
| PCDH17 | 498 | 3.910903 | 9.20E-05 | 0.0009608 | TRUE |
| C1R | 5016 | 3.910753 | 9.20E-05 | 0.0009608 | TRUE |
| COL11A1 | 7501 | 3.910592 | 9.21E-05 | 0.0009608 | TRUE |
| KLF8 | 11244 | 3.908991 | 9.27E-05 | 0.0009666 | TRUE |
| DCLK2 | 5895 | 3.908815 | 9.27E-05 | 0.0009666 | TRUE |
| WDR62 | 4227 | 3.908101 | 9.30E-05 | 0.0009689 | TRUE |
| FAR2P1 | 5269 | 3.906808 | 9.35E-05 | 0.0009734 | TRUE |
| PYCARD | 594 | 3.903469 | 9.48E-05 | 0.0009856 | TRUE |
| AMZ1 | 11951 | 3.900219 | 9.61E-05 | 0.0009983 | TRUE |
| TRAPPC2L | 5880 | 3.899612 | 9.63E-05 | 0.0009989 | TRUE |
| CDH9 | 1793 | 3.899595 | 9.64E-05 | 0.0009989 | TRUE |
| ADAMTS19 | 11026 | 3.899339 | 9.65E-05 | 0.0009993 | TRUE |
| JPT1 | 3078 | 3.898502 | 9.68E-05 | 0.0010021 | TRUE |
| TMEFF2 | 3232 | 3.897349 | 9.73E-05 | 0.0010062 | TRUE |
| VANGL2 | 11137 | 3.896049 | 9.78E-05 | 0.0010096 | TRUE |
| SPPL3 | 4831 | 3.892168 | 9.94E-05 | 0.0010218 | TRUE |
| DOCK8 | 9981 | 3.891476 | 9.96E-05 | 0.0010234 | TRUE |
| FHOD3 | 3144 | 3.89091 | 9.99E-05 | 0.0010247 | TRUE |
| HMGCR | 3904 | 3.890838 | 9.99E-05 | 0.0010247 | TRUE |
| PSMD4 | 12406 | 3.887705 | 0.000101 | 0.0010333 | TRUE |
| ZPR1 | 9603 | 3.88727 | 0.000101 | 0.0010338 | TRUE |
| PLEKHA4 | 3778 | 3.885619 | 0.000102 | 0.0010388 | TRUE |
| LINC01088 | 10844 | 3.882665 | 0.000103 | 0.0010509 | TRUE |
| ITGAM | 14643 | 3.882003 | 0.000104 | 0.001053 | TRUE |
| CTC1 | 13554 | 3.881832 | 0.000104 | 0.0010531 | TRUE |
| SUMF1 | 2148 | 3.881471 | 0.000104 | 0.001054 | TRUE |
| PTS | 8953 | 3.879609 | 0.000105 | 0.00106 | TRUE |
| DPYSL5 | 1123 | 3.878158 | 0.000105 | 0.0010643 | TRUE |
| PDLIM3 | 10821 | 3.876923 | 0.000106 | 0.001069 | TRUE |
| TAC1 | 10709 | 3.874924 | 0.000107 | 0.0010757 | TRUE |
| PTN | 12844 | 3.87187 | 0.000108 | 0.0010865 | TRUE |
| SMOC1 | 11376 | 3.870469 | 0.000109 | 0.0010917 | TRUE |
| SLC7A11 | 11339 | 3.869348 | 0.000109 | 0.0010957 | TRUE |
| TUBA3D | 1568 | 3.868923 | 0.000109 | 0.0010969 | TRUE |
| TTYH1 | 11756 | 3.86749 | 0.00011 | 0.0011005 | TRUE |
| ZNF706 | 3146 | 3.86629 | 0.000111 | 0.0011046 | TRUE |
| LOC100506100 | 9332 | 3.864607 | 0.000111 | 0.0011095 | TRUE |
| FOLR1 | 14987 | 3.863774 | 0.000112 | 0.0011124 | TRUE |
| EGFEM1P | 10596 | 3.861734 | 0.000113 | 0.0011196 | TRUE |
| SLC26A4-AS1 | 14200 | 3.857988 | 0.000114 | 0.0011349 | TRUE |
| RPS19 | 12319 | 3.85796 | 0.000114 | 0.0011349 | TRUE |
| IFT27 | 2168 | 3.857132 | 0.000115 | 0.001138 | TRUE |
| SF3B3 | 8100 | 3.854125 | 0.000116 | 0.0011499 | TRUE |
| SOX9-AS1 | 2429 | 3.853942 | 0.000116 | 0.00115 | TRUE |
| IL13RA2 | 7400 | 3.85275 | 0.000117 | 0.0011542 | TRUE |
| MPV17L2 | 11391 | 3.852337 | 0.000117 | 0.0011547 | TRUE |
| RPL31 | 11139 | 3.851853 | 0.000117 | 0.0011562 | TRUE |
| CABP7 | 273 | 3.851309 | 0.000117 | 0.0011573 | TRUE |
| LDHD | 8394 | 3.849084 | 0.000119 | 0.0011672 | TRUE |
| PRMT2 | 11562 | 3.846876 | 0.00012 | 0.0011746 | TRUE |
| CDC42EP4 | 11523 | 3.846621 | 0.00012 | 0.0011746 | TRUE |
| S100Z | 10635 | 3.846602 | 0.00012 | 0.0011746 | TRUE |
| STYK1 | 13565 | 3.840674 | 0.000123 | 0.0011976 | TRUE |
| LYPD8 | 11381 | 3.839123 | 0.000123 | 0.0012022 | TRUE |
| B3GAT1 | 4068 | 3.838859 | 0.000124 | 0.0012022 | TRUE |
| CHMP1A | 3701 | 3.838844 | 0.000124 | 0.0012022 | TRUE |
| BRD7 | 3276 | 3.838298 | 0.000124 | 0.001203 | TRUE |
| EMC7 | 5748 | 3.836591 | 0.000125 | 0.0012106 | TRUE |
| RASL11B | 2760 | 3.831821 | 0.000127 | 0.0012305 | TRUE |
| SOD1 | 4120 | 3.831128 | 0.000128 | 0.0012332 | TRUE |
| LY86 | 15594 | 3.828258 | 0.000129 | 0.0012446 | TRUE |
| UQCRFS1 | 7212 | 3.827735 | 0.000129 | 0.0012465 | TRUE |
| GPX4 | 2363 | 3.826699 | 0.00013 | 0.001251 | TRUE |
| ARL16 | 14549 | 3.826201 | 0.00013 | 0.0012527 | TRUE |
| SMIM10 | 8174 | 3.825253 | 0.000131 | 0.0012568 | TRUE |
| NPY | 12788 | 3.824827 | 0.000131 | 0.0012577 | TRUE |
| STK10 | 12093 | 3.824761 | 0.000131 | 0.0012577 | TRUE |
| RAB31 | 5154 | 3.82422 | 0.000131 | 0.0012584 | TRUE |
| FABP7 | 10722 | 3.824091 | 0.000131 | 0.0012584 | TRUE |
| OAZ2 | 414 | 3.823886 | 0.000131 | 0.0012584 | TRUE |
| FZR1 | 6152 | 3.82292 | 0.000132 | 0.0012617 | TRUE |
| HES4 | 2537 | 3.822483 | 0.000132 | 0.0012632 | TRUE |
| POLE4 | 3683 | 3.819163 | 0.000134 | 0.0012788 | TRUE |
| ARHGEF28 | 12612 | 3.818449 | 0.000134 | 0.0012809 | TRUE |
| CARD16 | 10556 | 3.816786 | 0.000135 | 0.0012888 | TRUE |
| VAT1L | 11774 | 3.816578 | 0.000135 | 0.0012891 | TRUE |
| C3orf14 | 11567 | 3.816063 | 0.000136 | 0.001291 | TRUE |
| ACVR2A | 2575 | 3.814373 | 0.000137 | 0.0012976 | TRUE |
| VAMP2 | 13558 | 3.814344 | 0.000137 | 0.0012976 | TRUE |
| SNRPD3 | 11677 | 3.814083 | 0.000137 | 0.0012982 | TRUE |
| SEMA4A | 5250 | 3.813212 | 0.000137 | 0.001302 | TRUE |
| EXOC3-AS1 | 10412 | 3.812704 | 0.000137 | 0.0013039 | TRUE |
| ENOX1 | 5910 | 3.812454 | 0.000138 | 0.0013044 | TRUE |
| NALT1 | 6491 | 3.812077 | 0.000138 | 0.0013045 | TRUE |
| ACSM5 | 9789 | 3.811988 | 0.000138 | 0.0013045 | TRUE |
| NECTIN2 | 13304 | 3.811528 | 0.000138 | 0.001306 | TRUE |
| CXXC4 | 3338 | 3.810112 | 0.000139 | 0.0013113 | TRUE |
| RGMB | 4673 | 3.807979 | 0.00014 | 0.0013195 | TRUE |
| LTBP4 | 157 | 3.807498 | 0.00014 | 0.0013212 | TRUE |
| HLA-H | 12005 | 3.805224 | 0.000142 | 0.0013318 | TRUE |
| SFMBT2 | 11465 | 3.804789 | 0.000142 | 0.0013334 | TRUE |
| PTPRC | 14107 | 3.802523 | 0.000143 | 0.0013437 | TRUE |
| MT1X | 3113 | 3.801736 | 0.000144 | 0.0013467 | TRUE |
| NPY1R | 3793 | 3.800398 | 0.000144 | 0.0013531 | TRUE |
| ZC3HAV1 | 11946 | 3.793928 | 0.000148 | 0.0013848 | TRUE |
| CBLN1 | 11120 | 3.790665 | 0.00015 | 0.0013972 | TRUE |
| GSTM2 | 13537 | 3.78896 | 0.000151 | 0.001406 | TRUE |
| PSD3 | 3083 | 3.78596 | 0.000153 | 0.0014214 | TRUE |
| PLCB2 | 370 | 3.785688 | 0.000153 | 0.0014221 | TRUE |
| SSR4P1 | 479 | 3.782833 | 0.000155 | 0.0014351 | TRUE |
| KLF10 | 3415 | 3.781889 | 0.000156 | 0.0014397 | TRUE |
| NUDT18 | 13702 | 3.779304 | 0.000157 | 0.0014513 | TRUE |
| TMEM205 | 3326 | 3.778857 | 0.000158 | 0.0014531 | TRUE |
| SYTL5 | 11987 | 3.776512 | 0.000159 | 0.0014651 | TRUE |
| TMTC1 | 6994 | 3.773415 | 0.000161 | 0.0014799 | TRUE |
| FAM171B | 13703 | 3.772467 | 0.000162 | 0.0014835 | TRUE |
| XYLT1 | 7463 | 3.772374 | 0.000162 | 0.0014835 | TRUE |
| ZMYND15 | 12924 | 3.771125 | 0.000163 | 0.0014901 | TRUE |
| SYNPR | 12570 | 3.770875 | 0.000163 | 0.0014907 | TRUE |
| TUBB6 | 5270 | 3.770683 | 0.000163 | 0.001491 | TRUE |
| EDN1 | 4521 | 3.770196 | 0.000163 | 0.0014927 | TRUE |
| GLDC | 1116 | 3.769849 | 0.000163 | 0.0014933 | TRUE |
| ETNPPL | 103 | 3.766214 | 0.000166 | 0.0015135 | TRUE |
| C20orf27 | 12603 | 3.764503 | 0.000167 | 0.0015215 | TRUE |
| ZNF831 | 5485 | 3.764445 | 0.000167 | 0.0015215 | TRUE |
| GFAP | 1344 | 3.761686 | 0.000169 | 0.0015357 | TRUE |
| BORCS7 | 1269 | 3.757795 | 0.000171 | 0.0015571 | TRUE |
| SRSF12 | 6476 | 3.756556 | 0.000172 | 0.0015621 | TRUE |
| CORO1A | 14280 | 3.754364 | 0.000174 | 0.001574 | TRUE |
| CTGF | 5223 | 3.751862 | 0.000176 | 0.0015862 | TRUE |
| PLA2G15 | 3233 | 3.751855 | 0.000176 | 0.0015862 | TRUE |
| LRMDA | 11260 | 3.749259 | 0.000177 | 0.0016018 | TRUE |
| TPST1 | 9276 | 3.745957 | 0.00018 | 0.0016192 | TRUE |
| ST6GALNAC6 | 1815 | 3.74466 | 0.000181 | 0.0016257 | TRUE |
| SND1 | 8458 | 3.74334 | 0.000182 | 0.0016334 | TRUE |
| PSTPIP1 | 13170 | 3.741772 | 0.000183 | 0.0016408 | TRUE |
| GLP2R | 7418 | 3.740307 | 0.000184 | 0.0016494 | TRUE |
| PIAS3 | 5051 | 3.73844 | 0.000185 | 0.0016604 | TRUE |
| CCDC3 | 245 | 3.737341 | 0.000186 | 0.0016642 | TRUE |
| GPR183 | 4428 | 3.737032 | 0.000186 | 0.0016653 | TRUE |
| PSMD9 | 15237 | 3.730102 | 0.000191 | 0.0017058 | TRUE |
| RPLP1 | 298 | 3.727868 | 0.000193 | 0.0017143 | TRUE |
| FBXO2 | 12046 | 3.723967 | 0.000196 | 0.001738 | TRUE |
| JARID2-AS1 | 9860 | 3.721752 | 0.000198 | 0.0017484 | TRUE |
| TMEM106A | 6666 | 3.721568 | 0.000198 | 0.0017487 | TRUE |
| SHISA4 | 14870 | 3.719907 | 0.000199 | 0.0017592 | TRUE |
| TRIB1 | 13044 | 3.718905 | 0.0002 | 0.0017649 | TRUE |
| SMAD2 | 15432 | 3.718816 | 0.0002 | 0.0017649 | TRUE |
| DACT1 | 9310 | 3.718544 | 0.0002 | 0.0017658 | TRUE |
| F12 | 4070 | 3.717149 | 0.000201 | 0.0017726 | TRUE |
| MAOB | 1019 | 3.713953 | 0.000204 | 0.0017921 | TRUE |
| RAP1B | 11581 | 3.711273 | 0.000206 | 0.0018071 | TRUE |
| RNF5 | 5725 | 3.709954 | 0.000207 | 0.0018135 | TRUE |
| CFAP70 | 7295 | 3.70963 | 0.000208 | 0.0018148 | TRUE |
| NWD2 | 9337 | 3.709062 | 0.000208 | 0.0018178 | TRUE |
| SLC16A9 | 4710 | 3.705965 | 0.000211 | 0.0018351 | TRUE |
| GBAP1 | 9742 | 3.704774 | 0.000212 | 0.0018414 | TRUE |
| ICAM5 | 1242 | 3.704532 | 0.000212 | 0.0018414 | TRUE |
| CRYZ | 12553 | 3.703539 | 0.000213 | 0.0018476 | TRUE |
| ALKAL2 | 7776 | 3.701853 | 0.000214 | 0.0018578 | TRUE |
| RPL36 | 3363 | 3.697376 | 0.000218 | 0.0018853 | TRUE |
| ATP6V1C1 | 3431 | 3.697286 | 0.000218 | 0.0018853 | TRUE |
| SAP30BP | 4267 | 3.696889 | 0.000218 | 0.0018861 | TRUE |
| ZDHHC12 | 5265 | 3.69458 | 0.00022 | 0.0018971 | TRUE |
| RRP7A | 2337 | 3.69164 | 0.000223 | 0.0019161 | TRUE |
| FILIP1 | 12791 | 3.691629 | 0.000223 | 0.0019161 | TRUE |
| TBX19 | 13005 | 3.690905 | 0.000223 | 0.0019194 | TRUE |
| APTR | 3485 | 3.689938 | 0.000224 | 0.0019257 | TRUE |
| EIPR1 | 6025 | 3.68895 | 0.000225 | 0.00193 | TRUE |
| MAOA | 14869 | 3.688168 | 0.000226 | 0.0019321 | TRUE |
| GPR88 | 2920 | 3.688117 | 0.000226 | 0.0019321 | TRUE |
| CHMP2A | 14603 | 3.687422 | 0.000227 | 0.0019352 | TRUE |
| CHRNA3 | 13609 | 3.68484 | 0.000229 | 0.0019539 | TRUE |
| LAMB1 | 13289 | 3.683995 | 0.00023 | 0.001959 | TRUE |
| RIPOR2 | 8781 | 3.683758 | 0.00023 | 0.001959 | TRUE |
| MLH1 | 4534 | 3.681916 | 0.000231 | 0.0019693 | TRUE |
| ARHGAP12 | 9583 | 3.67957 | 0.000234 | 0.0019839 | TRUE |
| RAC2 | 12542 | 3.677591 | 0.000235 | 0.0019971 | TRUE |
| FOXN3-AS1 | 4204 | 3.677212 | 0.000236 | 0.0019986 | TRUE |
| TMEM233 | 139 | 3.675401 | 0.000237 | 0.00201 | TRUE |
| CELF6 | 10716 | 3.67384 | 0.000239 | 0.0020201 | TRUE |
| ADI1 | 13632 | 3.673187 | 0.00024 | 0.0020231 | TRUE |
| BCS1L | 6490 | 3.671641 | 0.000241 | 0.0020332 | TRUE |
| SEZ6 | 1144 | 3.669196 | 0.000243 | 0.0020461 | TRUE |
| IL33 | 3204 | 3.668649 | 0.000244 | 0.0020494 | TRUE |
| PRDX1 | 13872 | 3.666949 | 0.000245 | 0.002062 | TRUE |
| SGSM2 | 1165 | 3.665727 | 0.000247 | 0.0020696 | TRUE |
| UFL1 | 13533 | 3.665493 | 0.000247 | 0.0020704 | TRUE |
| NEPRO | 4105 | 3.663858 | 0.000248 | 0.0020825 | TRUE |
| LMO1 | 14132 | 3.663644 | 0.000249 | 0.0020832 | TRUE |
| KLRC2 | 10487 | 3.663201 | 0.000249 | 0.0020857 | TRUE |
| SEMA5A | 7761 | 3.662597 | 0.00025 | 0.0020883 | TRUE |
| HLA-DRB5 | 2466 | 3.66193 | 0.00025 | 0.0020915 | TRUE |
| SNX7 | 12136 | 3.660462 | 0.000252 | 0.0021002 | TRUE |
| SNCA | 10012 | 3.660101 | 0.000252 | 0.002102 | TRUE |
| BCRP2 | 2616 | 3.658356 | 0.000254 | 0.0021147 | TRUE |
| CACNA1H | 4251 | 3.656408 | 0.000256 | 0.0021291 | TRUE |
| NR2F2 | 3470 | 3.655481 | 0.000257 | 0.0021357 | TRUE |
| FABP6 | 14624 | 3.654587 | 0.000258 | 0.002142 | TRUE |
| VSIG4 | 8893 | 3.654336 | 0.000258 | 0.002143 | TRUE |
| DHX58 | 4312 | 3.653699 | 0.000258 | 0.002146 | TRUE |
| SLC27A3 | 7169 | 3.652609 | 0.00026 | 0.0021517 | TRUE |
| NUDT16L1 | 14228 | 3.651661 | 0.000261 | 0.0021574 | TRUE |
| CFL1P1 | 14840 | 3.650639 | 0.000262 | 0.0021637 | TRUE |
| ALDH9A1 | 11057 | 3.645935 | 0.000266 | 0.0021967 | TRUE |
| SST | 13890 | 3.644654 | 0.000268 | 0.0022054 | TRUE |
| SLC25A26 | 9453 | 3.644144 | 0.000268 | 0.0022086 | TRUE |
| NKAIN3 | 7521 | 3.642048 | 0.00027 | 0.0022243 | TRUE |
| TMEM256 | 15430 | 3.640602 | 0.000272 | 0.0022356 | TRUE |
| STX1A | 7846 | 3.6403 | 0.000272 | 0.0022371 | TRUE |
| PYCR3 | 7561 | 3.634621 | 0.000278 | 0.002281 | TRUE |
| RPL35 | 10453 | 3.634204 | 0.000279 | 0.0022823 | TRUE |
| P2RX5 | 13068 | 3.630596 | 0.000283 | 0.0023092 | TRUE |
| RPL15 | 2701 | 3.630514 | 0.000283 | 0.0023092 | TRUE |
| PIPSL | 3124 | 3.630501 | 0.000283 | 0.0023092 | TRUE |
| PCED1B | 12357 | 3.629993 | 0.000283 | 0.0023125 | TRUE |
| SOX11 | 15560 | 3.625943 | 0.000288 | 0.0023454 | TRUE |
| KIAA0368 | 1669 | 3.624936 | 0.000289 | 0.0023525 | TRUE |
| TEAD1 | 5577 | 3.624605 | 0.000289 | 0.0023539 | TRUE |
| SHC2 | 8424 | 3.622954 | 0.000291 | 0.0023678 | TRUE |
| NEURL1B | 11328 | 3.621748 | 0.000293 | 0.0023751 | TRUE |
| TNIK | 13980 | 3.621374 | 0.000293 | 0.0023773 | TRUE |
| CD33 | 11971 | 3.620225 | 0.000294 | 0.0023855 | TRUE |
| LYPD1 | 6401 | 3.619887 | 0.000295 | 0.0023867 | TRUE |
| HRK | 14205 | 3.619696 | 0.000295 | 0.0023867 | TRUE |
| SURF1 | 14558 | 3.619344 | 0.000295 | 0.0023886 | TRUE |
| ADCYAP1R1 | 5327 | 3.618688 | 0.000296 | 0.0023922 | TRUE |
| ARPP19 | 3421 | 3.617443 | 0.000298 | 0.0024013 | TRUE |
| TMEM128 | 12143 | 3.614104 | 0.000301 | 0.0024287 | TRUE |
| ZFHX3 | 2844 | 3.6138 | 0.000302 | 0.0024303 | TRUE |
| FIBIN | 6008 | 3.610618 | 0.000305 | 0.002456 | TRUE |
| RPS8 | 10869 | 3.608805 | 0.000308 | 0.0024712 | TRUE |
| CALHM6 | 2452 | 3.607993 | 0.000309 | 0.0024776 | TRUE |
| SGSH | 710 | 3.607683 | 0.000309 | 0.0024776 | TRUE |
| RPL28 | 14269 | 3.602655 | 0.000315 | 0.00252 | TRUE |
| ZNF521 | 689 | 3.60059 | 0.000317 | 0.0025336 | TRUE |
| KIF21B | 7373 | 3.600045 | 0.000318 | 0.0025377 | TRUE |
| DISP2 | 862 | 3.599725 | 0.000319 | 0.0025395 | TRUE |
| LYRM9 | 1721 | 3.599545 | 0.000319 | 0.00254 | TRUE |
| ADCY7 | 3605 | 3.598927 | 0.00032 | 0.0025447 | TRUE |
| TUFM | 2802 | 3.598677 | 0.00032 | 0.0025459 | TRUE |
| CYTH4 | 10167 | 3.598382 | 0.00032 | 0.0025462 | TRUE |
| DACT3 | 6573 | 3.59726 | 0.000322 | 0.0025546 | TRUE |
| ALOX5AP | 9121 | 3.594834 | 0.000325 | 0.0025728 | TRUE |
| NOL4 | 3471 | 3.594747 | 0.000325 | 0.0025728 | TRUE |
| MCTP1 | 6191 | 3.594174 | 0.000325 | 0.0025759 | TRUE |
| TBC1D13 | 5854 | 3.592811 | 0.000327 | 0.0025868 | TRUE |
| GSTM3 | 413 | 3.592436 | 0.000328 | 0.0025892 | TRUE |
| FCGRT | 11345 | 3.592167 | 0.000328 | 0.0025905 | TRUE |
| TECPR2 | 11331 | 3.591293 | 0.000329 | 0.0025979 | TRUE |
| TMEM106C | 10472 | 3.589092 | 0.000332 | 0.0026173 | TRUE |
| TEX9 | 14763 | 3.588319 | 0.000333 | 0.0026221 | TRUE |
| HACD4 | 12138 | 3.588218 | 0.000333 | 0.0026221 | TRUE |
| SIGLEC11 | 13449 | 3.587704 | 0.000334 | 0.002626 | TRUE |
| PNP | 1956 | 3.586064 | 0.000336 | 0.0026409 | TRUE |
| POR | 283 | 3.585968 | 0.000336 | 0.0026409 | TRUE |
| BEX1 | 11326 | 3.58396 | 0.000338 | 0.0026586 | TRUE |
| HSD3B7 | 7842 | 3.582762 | 0.00034 | 0.0026668 | TRUE |
| SCG3 | 295 | 3.582377 | 0.00034 | 0.0026694 | TRUE |
| SLC35A2 | 10304 | 3.580281 | 0.000343 | 0.0026878 | TRUE |
| MDK | 12217 | 3.579334 | 0.000344 | 0.0026939 | TRUE |
| FSTL5 | 11295 | 3.576743 | 0.000348 | 0.0027182 | TRUE |
| ARPC3 | 7664 | 3.575577 | 0.000349 | 0.0027288 | TRUE |
| VSTM2L | 12097 | 3.575028 | 0.00035 | 0.0027298 | TRUE |
| GPRIN1 | 2182 | 3.573795 | 0.000352 | 0.0027406 | TRUE |
| HLA-DRA | 2815 | 3.57142 | 0.000355 | 0.0027628 | TRUE |
| HOPX | 3823 | 3.570825 | 0.000356 | 0.0027668 | TRUE |
| DDX54 | 2477 | 3.570752 | 0.000356 | 0.0027668 | TRUE |
| DPYSL4 | 8132 | 3.570417 | 0.000356 | 0.0027679 | TRUE |
| ARHGAP45 | 3443 | 3.568505 | 0.000359 | 0.002784 | TRUE |
| NME4 | 11343 | 3.568026 | 0.00036 | 0.0027864 | TRUE |
| LYRM2 | 3133 | 3.566633 | 0.000362 | 0.0027998 | TRUE |
| ASB1 | 12575 | 3.565551 | 0.000363 | 0.0028086 | TRUE |
| EFEMP2 | 8477 | 3.563201 | 0.000366 | 0.0028297 | TRUE |
| MOB1B | 12792 | 3.558907 | 0.000372 | 0.0028721 | TRUE |
| LOC101927318 | 5365 | 3.558562 | 0.000373 | 0.0028745 | TRUE |
| LRRTM1 | 3948 | 3.557936 | 0.000374 | 0.0028799 | TRUE |
| AKR7A2 | 7273 | 3.556877 | 0.000375 | 0.0028901 | TRUE |
| CSF1R | 14381 | 3.556618 | 0.000376 | 0.0028915 | TRUE |
| PAX6 | 13492 | 3.556181 | 0.000376 | 0.0028949 | TRUE |
| A1BG | 8840 | 3.556045 | 0.000376 | 0.002895 | TRUE |
| BAIAP2 | 3191 | 3.554819 | 0.000378 | 0.0029057 | TRUE |
| EFHB | 4712 | 3.553224 | 0.000381 | 0.0029146 | TRUE |
| CREM | 7808 | 3.552963 | 0.000381 | 0.0029146 | TRUE |
| SLIRP | 13445 | 3.552719 | 0.000381 | 0.0029146 | TRUE |
| UBE2S | 1544 | 3.550573 | 0.000384 | 0.0029342 | TRUE |
| NELFE | 4754 | 3.550147 | 0.000385 | 0.0029375 | TRUE |
| JMJD7 | 817 | 3.549502 | 0.000386 | 0.0029433 | TRUE |
| IDH2 | 8258 | 3.547545 | 0.000389 | 0.0029595 | TRUE |
| RSPH1 | 3581 | 3.547255 | 0.000389 | 0.0029598 | TRUE |
| MBNL3 | 2118 | 3.546136 | 0.000391 | 0.002971 | TRUE |
| BBOX1 | 3227 | 3.545993 | 0.000391 | 0.0029712 | TRUE |
| KLHL35 | 12010 | 3.545656 | 0.000392 | 0.0029721 | TRUE |
| SLC39A12 | 5133 | 3.544705 | 0.000393 | 0.0029781 | TRUE |
| FBLN2 | 9116 | 3.544614 | 0.000393 | 0.0029781 | TRUE |
| BCKDK | 9986 | 3.543856 | 0.000394 | 0.0029852 | TRUE |
| FRA10AC1 | 12453 | 3.542501 | 0.000396 | 0.0029948 | TRUE |
| RPS25 | 9870 | 3.541296 | 0.000398 | 0.003007 | TRUE |
| RGS9 | 3863 | 3.54097 | 0.000399 | 0.0030093 | TRUE |
| ACTR3B | 1232 | 3.536919 | 0.000405 | 0.003047 | TRUE |
| SPRN | 11315 | 3.53639 | 0.000406 | 0.0030516 | TRUE |
| ARFGEF3 | 3679 | 3.536212 | 0.000406 | 0.0030522 | TRUE |
| ABI1 | 11267 | 3.536022 | 0.000406 | 0.003053 | TRUE |
| DIAPH3 | 11806 | 3.534439 | 0.000409 | 0.0030669 | TRUE |
| PPP5D1 | 14093 | 3.531232 | 0.000414 | 0.0030961 | TRUE |
| PTRH1 | 8169 | 3.531172 | 0.000414 | 0.0030961 | TRUE |
| ME3 | 13219 | 3.530163 | 0.000415 | 0.0031064 | TRUE |
| VIT | 2591 | 3.528876 | 0.000417 | 0.00312 | TRUE |
| ADIPOR1 | 8904 | 3.528758 | 0.000418 | 0.00312 | TRUE |
| IL12RB2 | 13908 | 3.528275 | 0.000418 | 0.0031242 | TRUE |
| ZNF544 | 5608 | 3.52785 | 0.000419 | 0.0031277 | TRUE |
| SEL1L3 | 11297 | 3.527473 | 0.00042 | 0.0031302 | TRUE |
| NTNG2 | 11900 | 3.526696 | 0.000421 | 0.0031357 | TRUE |
| NUDT4 | 9793 | 3.526668 | 0.000421 | 0.0031357 | TRUE |
| PPP1R42 | 5563 | 3.52304 | 0.000427 | 0.0031699 | TRUE |
| FYN | 4220 | 3.517347 | 0.000436 | 0.0032295 | TRUE |
| ADRB2 | 8825 | 3.513959 | 0.000441 | 0.0032617 | TRUE |
| ATP6V1H | 5583 | 3.511944 | 0.000445 | 0.0032849 | TRUE |
| TSPAN6 | 3560 | 3.511094 | 0.000446 | 0.0032939 | TRUE |
| KIRREL3 | 693 | 3.508442 | 0.000451 | 0.0033222 | TRUE |
| CD81 | 3400 | 3.507655 | 0.000452 | 0.0033305 | TRUE |
| DTNBP1 | 10658 | 3.506622 | 0.000454 | 0.0033419 | TRUE |
| LAMC3 | 7593 | 3.506452 | 0.000454 | 0.0033424 | TRUE |
| KIAA1024 | 14972 | 3.505374 | 0.000456 | 0.0033544 | TRUE |
| CEP83 | 8690 | 3.503423 | 0.000459 | 0.0033759 | TRUE |
| TGFBI | 13183 | 3.503195 | 0.00046 | 0.0033772 | TRUE |
| PTPRG | 4191 | 3.502482 | 0.000461 | 0.0033847 | TRUE |
| RTL8A | 12102 | 3.501506 | 0.000463 | 0.0033955 | TRUE |
| MT1JP | 1842 | 3.499498 | 0.000466 | 0.0034148 | TRUE |
| LILRB4 | 11307 | 3.49906 | 0.000467 | 0.0034172 | TRUE |
| GGTA1P | 11540 | 3.498867 | 0.000467 | 0.003418 | TRUE |
| GALM | 4655 | 3.498029 | 0.000469 | 0.003427 | TRUE |
| CARD17 | 4817 | 3.497705 | 0.000469 | 0.0034282 | TRUE |
| PRPH2 | 4390 | 3.49734 | 0.00047 | 0.0034308 | TRUE |
| WNT7B | 12492 | 3.497253 | 0.00047 | 0.0034308 | TRUE |
| CD74 | 11342 | 3.496589 | 0.000471 | 0.0034377 | TRUE |
| HEYL | 9826 | 3.494865 | 0.000474 | 0.0034542 | TRUE |
| TMEM164 | 2086 | 3.494123 | 0.000476 | 0.0034604 | TRUE |
| FGD4 | 11811 | 3.494085 | 0.000476 | 0.0034604 | TRUE |
| PELI1 | 3242 | 3.493749 | 0.000476 | 0.0034624 | TRUE |
| ZNRF4 | 9462 | 3.491567 | 0.00048 | 0.0034851 | TRUE |
| PPP1R17 | 2007 | 3.486415 | 0.00049 | 0.0035447 | TRUE |
| TIMP2 | 3609 | 3.485681 | 0.000491 | 0.0035528 | TRUE |
| ACTR10 | 4508 | 3.485549 | 0.000491 | 0.0035529 | TRUE |
| SSUH2 | 11882 | 3.481551 | 0.000499 | 0.0035997 | TRUE |
| B4GALT2 | 12785 | 3.479359 | 0.000503 | 0.0036226 | TRUE |
| PET100 | 3462 | 3.478201 | 0.000505 | 0.0036366 | TRUE |
| SERF2 | 1870 | 3.477901 | 0.000505 | 0.003639 | TRUE |
| MARCKS | 9629 | 3.47588 | 0.000509 | 0.0036615 | TRUE |
| MYH7 | 13198 | 3.472963 | 0.000515 | 0.0036977 | TRUE |
| ISOC1 | 7466 | 3.466539 | 0.000527 | 0.0037685 | TRUE |
| TAF10 | 7241 | 3.465801 | 0.000529 | 0.0037737 | TRUE |
| MYL6 | 6926 | 3.465153 | 0.00053 | 0.0037811 | TRUE |
| IQCA1 | 7508 | 3.46304 | 0.000534 | 0.0038074 | TRUE |
| RPL27 | 1215 | 3.461948 | 0.000536 | 0.0038212 | TRUE |
| TAGLN2 | 3733 | 3.461013 | 0.000538 | 0.0038327 | TRUE |
| PBX4 | 6403 | 3.460187 | 0.00054 | 0.003841 | TRUE |
| MAPKAPK3 | 9344 | 3.45892 | 0.000542 | 0.0038539 | TRUE |
| RPL27A | 7792 | 3.458502 | 0.000543 | 0.0038581 | TRUE |
| APOC1 | 12817 | 3.458205 | 0.000544 | 0.0038597 | TRUE |
| TBC1D24 | 14964 | 3.457926 | 0.000544 | 0.0038597 | TRUE |
| KLHDC8A | 365 | 3.457902 | 0.000544 | 0.0038597 | TRUE |
| LIMS2 | 1464 | 3.457404 | 0.000545 | 0.0038633 | TRUE |
| FAM229B | 476 | 3.457235 | 0.000546 | 0.003864 | TRUE |
| NFATC1 | 12038 | 3.456913 | 0.000546 | 0.0038669 | TRUE |
| SLC8A2 | 2156 | 3.454237 | 0.000552 | 0.0038984 | TRUE |
| TRIB3 | 14615 | 3.453831 | 0.000553 | 0.0039013 | TRUE |
| CACNG4 | 2053 | 3.453675 | 0.000553 | 0.0039013 | TRUE |
| CRELD2 | 4525 | 3.452615 | 0.000555 | 0.003914 | TRUE |
| LRRC56 | 11471 | 3.452249 | 0.000556 | 0.003914 | TRUE |
| IL13RA1 | 14186 | 3.452186 | 0.000556 | 0.003914 | TRUE |
| AHCY | 5908 | 3.451541 | 0.000557 | 0.0039181 | TRUE |
| CYTL1 | 289 | 3.450121 | 0.00056 | 0.0039352 | TRUE |
| TUBB | 730 | 3.449652 | 0.000561 | 0.0039403 | TRUE |
| OTOF | 6005 | 3.448006 | 0.000565 | 0.0039561 | TRUE |
| RNASEH2A | 5410 | 3.44429 | 0.000573 | 0.0040013 | TRUE |
| SLC2A4RG | 8269 | 3.444288 | 0.000573 | 0.0040013 | TRUE |
| GLUD1 | 2119 | 3.443138 | 0.000575 | 0.0040166 | TRUE |
| ADIRF | 8684 | 3.442791 | 0.000576 | 0.0040182 | TRUE |
| OTUB2 | 11629 | 3.442783 | 0.000576 | 0.0040182 | TRUE |
| TRIM27 | 8928 | 3.442215 | 0.000577 | 0.0040249 | TRUE |
| RNF157-AS1 | 9015 | 3.441688 | 0.000578 | 0.0040309 | TRUE |
| PSENEN | 1990 | 3.438908 | 0.000584 | 0.0040689 | TRUE |
| CELF4 | 9994 | 3.43804 | 0.000586 | 0.0040802 | TRUE |
| PALMD | 5087 | 3.436684 | 0.000589 | 0.004097 | TRUE |
| LINC00958 | 7816 | 3.434007 | 0.000595 | 0.0041303 | TRUE |
| OVOL2 | 8692 | 3.431307 | 0.000601 | 0.0041643 | TRUE |
| FDFT1 | 11918 | 3.430615 | 0.000602 | 0.0041731 | TRUE |
| TPPP3 | 13577 | 3.430189 | 0.000603 | 0.0041759 | TRUE |
| FHOD1 | 3929 | 3.429824 | 0.000604 | 0.0041797 | TRUE |
| CACHD1 | 7093 | 3.429574 | 0.000605 | 0.0041817 | TRUE |
| NETO2 | 14462 | 3.427642 | 0.000609 | 0.004206 | TRUE |
| ITM2B | 2663 | 3.427509 | 0.000609 | 0.0042062 | TRUE |
| CARMIL3 | 941 | 3.425685 | 0.000613 | 0.0042308 | TRUE |
| LOC105372480 | 9093 | 3.424334 | 0.000616 | 0.0042481 | TRUE |
| PLSCR4 | 15077 | 3.423229 | 0.000619 | 0.0042598 | TRUE |
| AQP5 | 7980 | 3.42242 | 0.000621 | 0.0042689 | TRUE |
| UNC93B1 | 10330 | 3.422408 | 0.000621 | 0.0042689 | TRUE |
| PLAC9 | 13955 | 3.421813 | 0.000622 | 0.0042722 | TRUE |
| ACSL4 | 2705 | 3.421239 | 0.000623 | 0.0042779 | TRUE |
| LOC729970 | 3522 | 3.419738 | 0.000627 | 0.0042978 | TRUE |
| ACTG1 | 2372 | 3.418194 | 0.00063 | 0.0043128 | TRUE |
| CNTN3 | 4983 | 3.415761 | 0.000636 | 0.004342 | TRUE |
| SULF2 | 1760 | 3.414845 | 0.000638 | 0.0043528 | TRUE |
| ASIP | 15280 | 3.41338 | 0.000642 | 0.0043744 | TRUE |
| SMAD3 | 2681 | 3.410669 | 0.000648 | 0.0044162 | TRUE |
| MAMLD1 | 11374 | 3.410296 | 0.000649 | 0.0044203 | TRUE |
| TBC1D14 | 6837 | 3.409141 | 0.000652 | 0.0044352 | TRUE |
| PSME2 | 3231 | 3.405839 | 0.00066 | 0.0044809 | TRUE |
| RAP1GAP | 2849 | 3.405749 | 0.00066 | 0.0044809 | TRUE |
| ASS1 | 486 | 3.405114 | 0.000661 | 0.0044875 | TRUE |
| C12orf73 | 4275 | 3.404232 | 0.000664 | 0.0045 | TRUE |
| CNMD | 9173 | 3.403813 | 0.000665 | 0.0045037 | TRUE |
| CHDH | 10442 | 3.403775 | 0.000665 | 0.0045037 | TRUE |
| PARVA | 5277 | 3.402956 | 0.000667 | 0.0045124 | TRUE |
| DDAH2 | 8783 | 3.402889 | 0.000667 | 0.0045124 | TRUE |
| MIR7-3HG | 12176 | 3.400873 | 0.000672 | 0.0045342 | TRUE |
| GSG1L | 15193 | 3.400853 | 0.000672 | 0.0045342 | TRUE |
| PDE4DIP | 4930 | 3.400745 | 0.000672 | 0.0045342 | TRUE |
| PTTG1IP | 11657 | 3.400445 | 0.000673 | 0.0045372 | TRUE |
| LPAR6 | 11395 | 3.399938 | 0.000674 | 0.0045437 | TRUE |
| MCM6 | 5826 | 3.399549 | 0.000675 | 0.0045482 | TRUE |
| GSTO2 | 14130 | 3.396632 | 0.000682 | 0.0045839 | TRUE |
| GNG8 | 10803 | 3.394297 | 0.000688 | 0.0046184 | TRUE |
| CHRM4 | 5835 | 3.390708 | 0.000697 | 0.0046673 | TRUE |
| FAM86EP | 5875 | 3.389829 | 0.000699 | 0.0046803 | TRUE |
| TNNT1 | 6917 | 3.387901 | 0.000704 | 0.0047052 | TRUE |
| GNPTAB | 4795 | 3.387541 | 0.000705 | 0.0047094 | TRUE |
| VASN | 3709 | 3.387353 | 0.000706 | 0.0047106 | TRUE |
| TM2D3 | 5641 | 3.386735 | 0.000707 | 0.0047142 | TRUE |
| NUMBL | 7024 | 3.386679 | 0.000707 | 0.0047142 | TRUE |
| UQCR11 | 10614 | 3.385311 | 0.000711 | 0.0047297 | TRUE |
| TNFRSF14 | 2865 | 3.384499 | 0.000713 | 0.0047417 | TRUE |
| TCF7L1 | 5047 | 3.383442 | 0.000716 | 0.0047559 | TRUE |
| SAMD9 | 5299 | 3.382704 | 0.000718 | 0.0047667 | TRUE |
| CXCR4 | 15419 | 3.382108 | 0.000719 | 0.004773 | TRUE |
| NCAN | 145 | 3.37893 | 0.000728 | 0.0048231 | TRUE |
| GABRB1 | 9775 | 3.376846 | 0.000733 | 0.0048487 | TRUE |
| LOC101927420 | 11945 | 3.376387 | 0.000734 | 0.0048528 | TRUE |
| TPM3 | 4657 | 3.376181 | 0.000735 | 0.0048529 | TRUE |
| CYTOR | 12291 | 3.376148 | 0.000735 | 0.0048529 | TRUE |
| RASGEF1A | 9094 | 3.375885 | 0.000736 | 0.0048554 | TRUE |
| TMEM215 | 2056 | 3.375595 | 0.000737 | 0.0048565 | TRUE |
| ALCAM | 12262 | 3.375196 | 0.000738 | 0.0048615 | TRUE |
| FGD3 | 3153 | 3.372615 | 0.000745 | 0.004899 | TRUE |
| SNAP29 | 1491 | 3.372276 | 0.000745 | 0.0049009 | TRUE |
| FN3KRP | 12489 | 3.371865 | 0.000747 | 0.0049041 | TRUE |
| PLIN5 | 6422 | 3.371714 | 0.000747 | 0.0049047 | TRUE |
| TMEM198B | 12448 | 3.371103 | 0.000749 | 0.0049136 | TRUE |
| ZCCHC17 | 5304 | 3.368662 | 0.000755 | 0.0049531 | TRUE |
| TCTE1 | 13022 | 3.367574 | 0.000758 | 0.0049661 | TRUE |
| GALNT14 | 11383 | 3.367428 | 0.000759 | 0.0049661 | TRUE |
| MT1M | 47 | 3.367419 | 0.000759 | 0.0049661 | TRUE |
| UBE2D2 | 15162 | 3.365388 | 0.000764 | 0.0049939 | TRUE |
| EZR | 6583 | 3.364834 | 0.000766 | 0.0049998 | TRUE |
| PCBP3 | 4863 | 3.364691 | 0.000766 | 0.0049998 | TRUE |
| TPT1 | 3667 | 3.363905 | 0.000768 | 0.005012 | TRUE |
| ACTA1 | 2009 | 3.362975 | 0.000771 | 0.0050247 | TRUE |
| NANOS1 | 14211 | 3.360368 | 0.000778 | 0.005066 | TRUE |
| NDP | 820 | 3.358367 | 0.000784 | 0.0050965 | TRUE |
| NUDT2 | 5333 | 3.357376 | 0.000787 | 0.0051126 | TRUE |
| MIF | 11371 | 3.355966 | 0.000791 | 0.0051345 | TRUE |
| ARHGEF40 | 12675 | 3.35527 | 0.000793 | 0.0051453 | TRUE |
| PMVK | 14443 | 3.354378 | 0.000795 | 0.0051534 | TRUE |
| CA11 | 383 | 3.354378 | 0.000795 | 0.0051534 | TRUE |
| TMEM176B | 670 | 3.353779 | 0.000797 | 0.0051624 | TRUE |
| GNB2 | 13668 | 3.351849 | 0.000803 | 0.0051878 | TRUE |
| RASAL1 | 10314 | 3.351121 | 0.000805 | 0.0051993 | TRUE |
| RPS23 | 10559 | 3.348403 | 0.000813 | 0.0052419 | TRUE |
| HMGN2 | 5159 | 3.348132 | 0.000814 | 0.0052448 | TRUE |
| C8orf82 | 5692 | 3.346584 | 0.000818 | 0.005272 | TRUE |
| PNMA8A | 12151 | 3.345362 | 0.000822 | 0.005291 | TRUE |
| CHP1 | 11532 | 3.343422 | 0.000828 | 0.0053218 | TRUE |
| RAB34 | 3765 | 3.343291 | 0.000828 | 0.0053218 | TRUE |
| GPM6A | 14547 | 3.342487 | 0.00083 | 0.0053329 | TRUE |
| ATP2C2 | 6944 | 3.341277 | 0.000834 | 0.0053496 | TRUE |
| PLEKHG4B | 4742 | 3.337672 | 0.000845 | 0.0054084 | TRUE |
| HADH | 13120 | 3.337426 | 0.000846 | 0.005411 | TRUE |
| NBAS | 5823 | 3.335693 | 0.000851 | 0.0054359 | TRUE |
| FKBP14 | 9928 | 3.334789 | 0.000854 | 0.0054514 | TRUE |
| TP53I11 | 7114 | 3.33396 | 0.000856 | 0.0054654 | TRUE |
| CHST9 | 13448 | 3.332799 | 0.00086 | 0.0054838 | TRUE |
| S100A2 | 12019 | 3.332543 | 0.000861 | 0.0054866 | TRUE |
| DOK4 | 5579 | 3.332151 | 0.000862 | 0.0054921 | TRUE |
| SPINT2 | 5045 | 3.331604 | 0.000863 | 0.0055007 | TRUE |
| ALPL | 10853 | 3.330759 | 0.000866 | 0.0055129 | TRUE |
| CNPY2 | 9523 | 3.329657 | 0.00087 | 0.0055258 | TRUE |
| OAZ1 | 2921 | 3.32942 | 0.00087 | 0.0055282 | TRUE |
| CHMP4A | 11458 | 3.328743 | 0.000872 | 0.0055349 | TRUE |
| ELOB | 4541 | 3.328543 | 0.000873 | 0.0055367 | TRUE |
| RPL37A | 4294 | 3.328334 | 0.000874 | 0.0055386 | TRUE |
| SLC29A4 | 7969 | 3.324912 | 0.000884 | 0.005599 | TRUE |
| PLPP4 | 4422 | 3.324809 | 0.000885 | 0.005599 | TRUE |
| PON3 | 12192 | 3.323765 | 0.000888 | 0.0056142 | TRUE |
| FZD9 | 2712 | 3.322784 | 0.000891 | 0.0056231 | TRUE |
| APBB1IP | 11470 | 3.322496 | 0.000892 | 0.0056261 | TRUE |
| APLNR | 4386 | 3.322204 | 0.000893 | 0.0056297 | TRUE |
| ITGAX | 3091 | 3.321558 | 0.000895 | 0.0056405 | TRUE |
| ERC2 | 13536 | 3.318236 | 0.000906 | 0.0057011 | TRUE |
| SOX5 | 10131 | 3.318086 | 0.000906 | 0.0057019 | TRUE |
| WLS | 4948 | 3.317797 | 0.000907 | 0.0057055 | TRUE |
| C1QB | 3782 | 3.316884 | 0.00091 | 0.0057196 | TRUE |
| FAU | 2510 | 3.316537 | 0.000911 | 0.0057221 | TRUE |
| EIF3H | 6562 | 3.316073 | 0.000913 | 0.0057293 | TRUE |
| ERMARD | 779 | 3.315695 | 0.000914 | 0.0057311 | TRUE |
| CYP26A1 | 704 | 3.315469 | 0.000915 | 0.0057325 | TRUE |
| SF3B6 | 9711 | 3.314333 | 0.000919 | 0.0057535 | TRUE |
| PRMT6 | 6629 | 3.313512 | 0.000921 | 0.0057609 | TRUE |
| LINC01547 | 12457 | 3.31287 | 0.000923 | 0.0057675 | TRUE |
| RPS3A | 13751 | 3.312711 | 0.000924 | 0.0057684 | TRUE |
| CRYM | 9050 | 3.312603 | 0.000924 | 0.0057684 | TRUE |
| PBXIP1 | 7328 | 3.312436 | 0.000925 | 0.0057696 | TRUE |
| TSKU | 11397 | 3.311884 | 0.000927 | 0.0057787 | TRUE |
| YWHAH | 8357 | 3.310837 | 0.00093 | 0.0057957 | TRUE |
| LRTOMT | 3748 | 3.31069 | 0.000931 | 0.0057961 | TRUE |
| LST1 | 11765 | 3.310185 | 0.000932 | 0.0058023 | TRUE |
| XRCC6 | 14369 | 3.309208 | 0.000936 | 0.0058179 | TRUE |
| COLEC11 | 1188 | 3.307423 | 0.000942 | 0.0058412 | TRUE |
| TUBB8P12 | 12818 | 3.305686 | 0.000947 | 0.0058705 | TRUE |
| GSTM5 | 8270 | 3.303943 | 0.000953 | 0.0059025 | TRUE |
| SPRY1 | 11753 | 3.303135 | 0.000956 | 0.0059172 | TRUE |
| ZNF219 | 5596 | 3.301907 | 0.00096 | 0.0059384 | TRUE |
| SOWAHD | 14735 | 3.301287 | 0.000962 | 0.0059492 | TRUE |
| RPL10 | 2801 | 3.300754 | 0.000964 | 0.0059579 | TRUE |
| DLL3 | 4186 | 3.300657 | 0.000965 | 0.0059579 | TRUE |
| SCN7A | 5870 | 3.300446 | 0.000965 | 0.00596 | TRUE |
| MINOS1 | 6933 | 3.299511 | 0.000969 | 0.0059775 | TRUE |
| CCL28 | 14263 | 3.297773 | 0.000975 | 0.0060099 | TRUE |
| PTRHD1 | 7327 | 3.297511 | 0.000975 | 0.0060131 | TRUE |
| LRRC36 | 5086 | 3.294609 | 0.000986 | 0.0060612 | TRUE |
| GNA12 | 6605 | 3.293097 | 0.000991 | 0.0060915 | TRUE |
| TSPO | 6950 | 3.292552 | 0.000993 | 0.006101 | TRUE |
| TLR2 | 7187 | 3.292104 | 0.000994 | 0.0061083 | TRUE |
| PGM1 | 5441 | 3.291946 | 0.000995 | 0.0061087 | TRUE |
| HSP90B1 | 1517 | 3.290982 | 0.000998 | 0.0061183 | TRUE |
| MED16 | 14168 | 3.290725 | 0.000999 | 0.0061191 | TRUE |
| HLA-DQB1 | 7037 | 3.289102 | 0.001005 | 0.0061521 | TRUE |
| GPC5 | 2901 | 3.288915 | 0.001006 | 0.0061537 | TRUE |
| 1-Mar | 12280 | 3.287345 | 0.001011 | 0.0061809 | TRUE |
| PPHLN1 | 5193 | 3.286774 | 0.001013 | 0.006191 | TRUE |
| CXADR | 2487 | 3.285699 | 0.001017 | 0.0062123 | TRUE |
| CYP46A1 | 12613 | 3.282315 | 0.00103 | 0.0062799 | TRUE |
| GUCY1A1 | 6013 | 3.281567 | 0.001032 | 0.0062893 | TRUE |
| SRGAP1 | 13795 | 3.280058 | 0.001038 | 0.0063205 | TRUE |
| RNF141 | 1193 | 3.279172 | 0.001041 | 0.0063379 | TRUE |
| ARHGAP6 | 1584 | 3.278744 | 0.001043 | 0.0063426 | TRUE |
| RPL12 | 12317 | 3.277879 | 0.001046 | 0.0063596 | TRUE |
| ATP6V0C | 2787 | 3.276934 | 0.001049 | 0.0063735 | TRUE |
| KLRC1 | 6301 | 3.276464 | 0.001051 | 0.0063816 | TRUE |
| DDN | 7014 | 3.273777 | 0.001061 | 0.0064362 | TRUE |
| RGL3 | 7705 | 3.273557 | 0.001062 | 0.0064362 | TRUE |
| WWTR1 | 9444 | 3.273445 | 0.001062 | 0.0064362 | TRUE |
| RIIAD1 | 13401 | 3.273298 | 0.001063 | 0.0064362 | TRUE |
| ARHGAP18 | 1462 | 3.272615 | 0.001066 | 0.0064491 | TRUE |
| MT1G | 11482 | 3.272369 | 0.001067 | 0.0064523 | TRUE |
| DNAJC12 | 530 | 3.27192 | 0.001068 | 0.006455 | TRUE |
| C1QL1 | 1303 | 3.271525 | 0.00107 | 0.0064588 | TRUE |
| MARCO | 6527 | 3.271445 | 0.00107 | 0.0064588 | TRUE |
| FAM184A | 13518 | 3.271427 | 0.00107 | 0.0064588 | TRUE |
| RNASET2 | 3467 | 3.270863 | 0.001072 | 0.0064692 | TRUE |
| TBC1D10A | 9335 | 3.270369 | 0.001074 | 0.0064742 | TRUE |
| CDH10 | 8475 | 3.27021 | 0.001075 | 0.0064742 | TRUE |
| TP53TG1 | 11863 | 3.268319 | 0.001082 | 0.006506 | TRUE |
| KCNK3 | 3810 | 3.268275 | 0.001082 | 0.006506 | TRUE |
| CCIN | 3353 | 3.263129 | 0.001102 | 0.0066172 | TRUE |
| NOVA2 | 4583 | 3.261655 | 0.001108 | 0.0066446 | TRUE |
| LOC105374546 | 6534 | 3.261322 | 0.001109 | 0.0066498 | TRUE |
| LOC101927752 | 14231 | 3.260858 | 0.001111 | 0.0066582 | TRUE |
| SAMD9L | 2757 | 3.256746 | 0.001127 | 0.0067414 | TRUE |
| ARHGDIG | 13300 | 3.255211 | 0.001133 | 0.0067653 | TRUE |
| RCN1 | 562 | 3.252629 | 0.001143 | 0.0068174 | TRUE |
| ADRA2C | 12062 | 3.250926 | 0.00115 | 0.0068505 | TRUE |
| CYP2R1 | 14043 | 3.248471 | 0.00116 | 0.0068969 | TRUE |
| MRPL20 | 10534 | 3.248031 | 0.001162 | 0.0069038 | TRUE |
| GASAL1 | 12898 | 3.247952 | 0.001162 | 0.0069038 | TRUE |
| FAM241B | 10042 | 3.247857 | 0.001163 | 0.0069038 | TRUE |
| NECAP2 | 236 | 3.245031 | 0.001174 | 0.0069621 | TRUE |
| CRADD | 11379 | 3.243985 | 0.001179 | 0.0069851 | TRUE |
| BCHE | 10766 | 3.242074 | 0.001187 | 0.0070188 | TRUE |
| DRD2 | 3305 | 3.241877 | 0.001187 | 0.007021 | TRUE |
| FCGBP | 3325 | 3.241336 | 0.00119 | 0.0070308 | TRUE |
| SSBP4 | 1597 | 3.241262 | 0.00119 | 0.0070308 | TRUE |
| ARMCX1 | 3799 | 3.240862 | 0.001192 | 0.007038 | TRUE |
| RFK | 11332 | 3.239369 | 0.001198 | 0.0070723 | TRUE |
| DERL2 | 8089 | 3.236897 | 0.001208 | 0.0071211 | TRUE |
| LHX8 | 8138 | 3.236403 | 0.00121 | 0.0071301 | TRUE |
| TIMP1 | 7060 | 3.236152 | 0.001212 | 0.0071336 | TRUE |
| SKAP2 | 11338 | 3.23496 | 0.001217 | 0.0071508 | TRUE |
| RILPL2 | 11141 | 3.234935 | 0.001217 | 0.0071508 | TRUE |
| ARF3 | 615 | 3.234931 | 0.001217 | 0.0071508 | TRUE |
| C16orf58 | 10052 | 3.233255 | 0.001224 | 0.0071847 | TRUE |
| INPP5K | 9445 | 3.233045 | 0.001225 | 0.0071873 | TRUE |
| CLTCL1 | 434 | 3.232862 | 0.001226 | 0.0071878 | TRUE |
| NSMCE1 | 3734 | 3.232699 | 0.001226 | 0.0071878 | TRUE |
| C2CD2L | 5493 | 3.232599 | 0.001227 | 0.0071878 | TRUE |
| FPGS | 14586 | 3.231965 | 0.001229 | 0.007201 | TRUE |
| PTDSS1 | 6680 | 3.231239 | 0.001233 | 0.0072139 | TRUE |
| CNIH3 | 14202 | 3.229436 | 0.00124 | 0.0072569 | TRUE |
| C22orf39 | 4365 | 3.22927 | 0.001241 | 0.0072584 | TRUE |
| TRAF3IP3 | 8898 | 3.228534 | 0.001244 | 0.0072716 | TRUE |
| FGL2 | 13511 | 3.227611 | 0.001248 | 0.0072897 | TRUE |
| PDYN | 10837 | 3.226812 | 0.001252 | 0.0073046 | TRUE |
| LOC101928111 | 3100 | 3.224667 | 0.001261 | 0.0073513 | TRUE |
| ERBB2 | 9547 | 3.223143 | 0.001268 | 0.007385 | TRUE |
| LMO3 | 11200 | 3.222586 | 0.00127 | 0.0073967 | TRUE |
| TSPOAP1 | 5834 | 3.222156 | 0.001272 | 0.0074022 | TRUE |
| TUBA4A | 9096 | 3.221502 | 0.001275 | 0.0074164 | TRUE |
| ADAM28 | 13840 | 3.2198 | 0.001283 | 0.007455 | TRUE |
| GPR143 | 12786 | 3.218542 | 0.001288 | 0.0074821 | TRUE |
| ABCA1 | 1607 | 3.217912 | 0.001291 | 0.0074917 | TRUE |
| TIMM13 | 183 | 3.21786 | 0.001292 | 0.0074917 | TRUE |
| HLA-A | 5633 | 3.216514 | 0.001298 | 0.007513 | TRUE |
| IL34 | 14127 | 3.216084 | 0.0013 | 0.0075187 | TRUE |
| S100A16 | 7492 | 3.214718 | 0.001306 | 0.007549 | TRUE |
| ECH1 | 13779 | 3.213816 | 0.00131 | 0.0075667 | TRUE |
| SYK | 11996 | 3.212188 | 0.001317 | 0.0076017 | TRUE |
| OCA2 | 5567 | 3.212049 | 0.001318 | 0.0076026 | TRUE |
| NETO1 | 5911 | 3.211605 | 0.00132 | 0.0076087 | TRUE |
| IMPACT | 12928 | 3.211189 | 0.001322 | 0.0076141 | TRUE |
| GPC2 | 7150 | 3.210994 | 0.001323 | 0.0076165 | TRUE |
| USE1 | 13916 | 3.20936 | 0.00133 | 0.0076515 | TRUE |
| FIGNL2 | 9651 | 3.20803 | 0.001336 | 0.0076841 | TRUE |
| MIR4435-2HG | 8218 | 3.207278 | 0.00134 | 0.0076957 | TRUE |
| AIF1 | 800 | 3.204934 | 0.001351 | 0.0077502 | TRUE |
| RMI2 | 13065 | 3.203737 | 0.001357 | 0.0077767 | TRUE |
| RAC1 | 9885 | 3.203572 | 0.001357 | 0.0077783 | TRUE |
| DKK3 | 9175 | 3.203374 | 0.001358 | 0.0077808 | TRUE |
| PRDM12 | 14812 | 3.202763 | 0.001361 | 0.0077945 | TRUE |
| MDH2 | 8706 | 3.202459 | 0.001363 | 0.0077999 | TRUE |
| RNF181 | 126 | 3.201778 | 0.001366 | 0.0078121 | TRUE |
| MT1B | 13861 | 3.201041 | 0.001369 | 0.007824 | TRUE |
| PEX19 | 6227 | 3.199426 | 0.001377 | 0.0078508 | TRUE |
| LRP4 | 9131 | 3.197646 | 0.001386 | 0.0078934 | TRUE |
| ATP9A | 10961 | 3.196262 | 0.001392 | 0.0079171 | TRUE |
| PDK2 | 2895 | 3.196162 | 0.001393 | 0.0079171 | TRUE |
| FAM86FP | 3615 | 3.195776 | 0.001395 | 0.0079248 | TRUE |
| NEUROD6 | 4187 | 3.194603 | 0.0014 | 0.0079542 | TRUE |
| SPICE1 | 7672 | 3.193104 | 0.001408 | 0.0079898 | TRUE |
| DSEL | 7344 | 3.191414 | 0.001416 | 0.0080279 | TRUE |
| RIPK2 | 4905 | 3.190577 | 0.00142 | 0.0080454 | TRUE |
| WDR13 | 11273 | 3.189087 | 0.001427 | 0.0080833 | TRUE |
| ROBO1 | 2732 | 3.189009 | 0.001428 | 0.0080833 | TRUE |
| HMGN2P46 | 6430 | 3.187287 | 0.001436 | 0.0081139 | TRUE |
| CAMK2D | 6622 | 3.186676 | 0.001439 | 0.0081281 | TRUE |
| ABHD1 | 4203 | 3.183095 | 0.001457 | 0.0082204 | TRUE |
| GRB14 | 7779 | 3.182013 | 0.001463 | 0.0082453 | TRUE |
| CFAP44 | 9239 | 3.181604 | 0.001465 | 0.008251 | TRUE |
| FAM89A | 278 | 3.180384 | 0.001471 | 0.0082798 | TRUE |
| UFD1 | 11898 | 3.180225 | 0.001472 | 0.0082814 | TRUE |
| NDUFA11 | 14789 | 3.179919 | 0.001473 | 0.0082871 | TRUE |
| ROBO2 | 8916 | 3.179324 | 0.001476 | 0.0082982 | TRUE |
| MAN1B1-AS1 | 3666 | 3.177869 | 0.001484 | 0.0083251 | TRUE |
| PHGR1 | 10511 | 3.177822 | 0.001484 | 0.0083251 | TRUE |
| RPL19 | 3412 | 3.177772 | 0.001484 | 0.0083251 | TRUE |
| CRLF1 | 5363 | 3.177725 | 0.001484 | 0.0083251 | TRUE |
| RABGAP1 | 1485 | 3.177534 | 0.001485 | 0.0083256 | TRUE |
| FANCL | 7924 | 3.176875 | 0.001489 | 0.0083376 | TRUE |
| GPR27 | 4507 | 3.176676 | 0.00149 | 0.0083376 | TRUE |
| NPHS1 | 12902 | 3.176599 | 0.00149 | 0.0083376 | TRUE |
| PSMD1 | 7901 | 3.176276 | 0.001492 | 0.0083426 | TRUE |
| CXADRP2 | 4028 | 3.176146 | 0.001492 | 0.0083426 | TRUE |
| CCDC68 | 5696 | 3.175578 | 0.001495 | 0.008355 | TRUE |
| SCIN | 5690 | 3.175369 | 0.001496 | 0.0083552 | TRUE |
| LILRP2 | 1856 | 3.175365 | 0.001496 | 0.0083552 | TRUE |
| ZFP36L2 | 6975 | 3.174724 | 0.0015 | 0.0083677 | TRUE |
| CLNS1A | 10733 | 3.174368 | 0.001502 | 0.008372 | TRUE |
| PRR29 | 6845 | 3.173825 | 0.001504 | 0.0083847 | TRUE |
| SLC2A12 | 2767 | 3.17293 | 0.001509 | 0.0084047 | TRUE |
| ZSWIM7 | 3215 | 3.172404 | 0.001512 | 0.0084168 | TRUE |
| ADAM7 | 5144 | 3.171706 | 0.001515 | 0.0084314 | TRUE |
| DMWD | 8496 | 3.16978 | 0.001526 | 0.0084841 | TRUE |
| SNRPD2 | 1599 | 3.167934 | 0.001535 | 0.0085291 | TRUE |
| SPATA1 | 4672 | 3.167191 | 0.001539 | 0.0085478 | TRUE |
| DOK5 | 12443 | 3.166996 | 0.00154 | 0.008548 | TRUE |
| RAN | 9599 | 3.166979 | 0.00154 | 0.008548 | TRUE |
| SAP18 | 856 | 3.166203 | 0.001544 | 0.0085648 | TRUE |
| RAB32 | 1756 | 3.162408 | 0.001565 | 0.0086618 | TRUE |
| ARHGDIB | 12332 | 3.162228 | 0.001566 | 0.0086641 | TRUE |
| BASP1 | 2285 | 3.162084 | 0.001566 | 0.0086653 | TRUE |
| KLHL13 | 1596 | 3.161747 | 0.001568 | 0.0086723 | TRUE |
| TREM2 | 826 | 3.161081 | 0.001572 | 0.008686 | TRUE |
| ACADL | 11163 | 3.159966 | 0.001578 | 0.0087132 | TRUE |
| GXYLT2 | 1929 | 3.159328 | 0.001581 | 0.0087248 | TRUE |
| EFEMP1 | 9637 | 3.159275 | 0.001582 | 0.0087248 | TRUE |
| GPRIN2 | 166 | 3.159268 | 0.001582 | 0.0087248 | TRUE |
| SPATA4 | 12847 | 3.15877 | 0.001584 | 0.0087342 | TRUE |
| HYI | 6034 | 3.15832 | 0.001587 | 0.008744 | TRUE |
| STON2 | 776 | 3.156562 | 0.001596 | 0.0087865 | TRUE |
| PHF20L1 | 4005 | 3.156321 | 0.001598 | 0.0087865 | TRUE |
| RPS17 | 2432 | 3.156051 | 0.001599 | 0.0087884 | TRUE |
| PPM1N | 10291 | 3.155918 | 0.0016 | 0.0087884 | TRUE |
| JPH4 | 7003 | 3.155767 | 0.001601 | 0.0087899 | TRUE |
| NDUFA6-AS1 | 13733 | 3.154806 | 0.001606 | 0.0088158 | TRUE |
| GPR6 | 9849 | 3.15349 | 0.001613 | 0.0088463 | TRUE |
| AK5 | 2077 | 3.151932 | 0.001622 | 0.0088874 | TRUE |
| SPATA20 | 8155 | 3.151784 | 0.001623 | 0.0088888 | TRUE |
| KIF2A | 2266 | 3.150703 | 0.001629 | 0.0089186 | TRUE |
| HBM | 8019 | 3.149974 | 0.001633 | 0.0089378 | TRUE |
| UMPS | 14542 | 3.149739 | 0.001634 | 0.0089419 | TRUE |
| MTHFD1 | 10620 | 3.148392 | 0.001642 | 0.0089769 | TRUE |
| SPATC1L | 14393 | 3.14721 | 0.001648 | 0.0090049 | TRUE |
| ASPHD2 | 8643 | 3.147175 | 0.001649 | 0.0090049 | TRUE |
| PRAF2 | 4438 | 3.146888 | 0.00165 | 0.0090105 | TRUE |
| CLDN4 | 9817 | 3.146789 | 0.001651 | 0.0090105 | TRUE |
| EIF6 | 5606 | 3.145143 | 0.00166 | 0.0090487 | TRUE |
| SLC27A2 | 8180 | 3.144719 | 0.001662 | 0.0090555 | TRUE |
| BCAN | 7309 | 3.143223 | 0.001671 | 0.0090956 | TRUE |
| GJA1 | 3668 | 3.142502 | 0.001675 | 0.0091124 | TRUE |
| NIPSNAP3A | 8885 | 3.142478 | 0.001675 | 0.0091124 | TRUE |
| OAF | 561 | 3.142088 | 0.001677 | 0.0091214 | TRUE |
| LAMTOR2 | 15482 | 3.14091 | 0.001684 | 0.0091518 | TRUE |
| TLL1 | 12897 | 3.140301 | 0.001688 | 0.0091676 | TRUE |
| C9orf116 | 15476 | 3.139655 | 0.001691 | 0.0091846 | TRUE |
| MYDGF | 7670 | 3.139555 | 0.001692 | 0.0091846 | TRUE |
| MBLAC2 | 5943 | 3.138966 | 0.001695 | 0.0091967 | TRUE |
| E2F3 | 2264 | 3.138714 | 0.001697 | 0.0091983 | TRUE |
| HSD17B8 | 4281 | 3.137224 | 0.001706 | 0.0092275 | TRUE |
| LRRC73 | 12774 | 3.137074 | 0.001706 | 0.0092275 | TRUE |
| CABYR | 753 | 3.136421 | 0.00171 | 0.0092417 | TRUE |
| SKP1 | 14223 | 3.134295 | 0.001723 | 0.0092928 | TRUE |
| TRMT112 | 12874 | 3.132873 | 0.001731 | 0.0093315 | TRUE |
| TMEM255A | 11118 | 3.132307 | 0.001734 | 0.0093463 | TRUE |
| TXN | 12971 | 3.131238 | 0.001741 | 0.0093772 | TRUE |
| CEP170B | 14644 | 3.130877 | 0.001743 | 0.009385 | TRUE |
| SLC25A18 | 5721 | 3.130791 | 0.001743 | 0.009385 | TRUE |
| PSMA2 | 4472 | 3.130422 | 0.001746 | 0.0093935 | TRUE |
| DUSP18 | 14016 | 3.129463 | 0.001751 | 0.009421 | TRUE |
| AZIN1-AS1 | 5999 | 3.129021 | 0.001754 | 0.009432 | TRUE |
| NAA10 | 4572 | 3.127867 | 0.001761 | 0.0094658 | TRUE |
| MAN1A1 | 1363 | 3.126468 | 0.001769 | 0.0095044 | TRUE |
| HCFC1R1 | 14934 | 3.126067 | 0.001772 | 0.0095141 | TRUE |
| NEDD8 | 11385 | 3.125952 | 0.001772 | 0.0095146 | TRUE |
| KCNQ1 | 10205 | 3.125362 | 0.001776 | 0.0095285 | TRUE |
| PLAC4 | 7482 | 3.124392 | 0.001782 | 0.0095553 | TRUE |
| PIGF | 3955 | 3.123466 | 0.001787 | 0.0095756 | TRUE |
| PRKG1 | 61 | 3.122098 | 0.001796 | 0.0096136 | TRUE |
| NPM1 | 9319 | 3.121733 | 0.001798 | 0.0096222 | TRUE |
| MR1 | 6630 | 3.121248 | 0.001801 | 0.0096348 | TRUE |
| SCPEP1 | 4110 | 3.120316 | 0.001807 | 0.0096587 | TRUE |
| NSMF | 3611 | 3.120145 | 0.001808 | 0.0096602 | TRUE |
| SRM | 11911 | 3.11997 | 0.001809 | 0.0096602 | TRUE |
| CMTM7 | 13292 | 3.117524 | 0.001824 | 0.0097307 | TRUE |
| IDNK | 4284 | 3.116974 | 0.001827 | 0.0097456 | TRUE |
| IQCK | 1664 | 3.115802 | 0.001834 | 0.009781 | TRUE |
| CHRDL1 | 10608 | 3.115215 | 0.001838 | 0.0097972 | TRUE |
| PIFO | 11322 | 3.114729 | 0.001841 | 0.00981 | TRUE |
| LOC105376064 | 14308 | 3.112003 | 0.001858 | 0.0098977 | TRUE |
| LINC01485 | 2877 | 3.110802 | 0.001866 | 0.0099312 | TRUE |
| ANAPC11 | 12867 | 3.109842 | 0.001872 | 0.0099602 | TRUE |
| FBXO44 | 1732 | 3.109649 | 0.001873 | 0.0099607 | TRUE |
| MRPL23 | 12921 | 3.109627 | 0.001873 | 0.0099607 | TRUE |
| SERP1 | 13226 | 3.108072 | 0.001883 | 0.0099985 | TRUE |
| PTCHD1 | 11001 | 3.105844 | 0.001897 | 0.0100675 | TRUE |
| IQCD | 5453 | 3.105685 | 0.001898 | 0.0100675 | TRUE |
| MT3 | 5490 | 3.10567 | 0.001898 | 0.0100675 | TRUE |
| MRPS6 | 1550 | 3.104079 | 0.001909 | 0.0101164 | TRUE |
| TNFAIP8L1 | 11355 | 3.102232 | 0.001921 | 0.0101714 | TRUE |
| PPEF1 | 1646 | 3.101353 | 0.001926 | 0.010198 | TRUE |
| RNASE6 | 13199 | 3.101031 | 0.001928 | 0.0102001 | TRUE |
| ECEL1 | 12545 | 3.100997 | 0.001929 | 0.0102001 | TRUE |
| TMEM234 | 6241 | 3.100176 | 0.001934 | 0.0102215 | TRUE |
| BIN1 | 8638 | 3.099784 | 0.001937 | 0.0102315 | TRUE |
| SORBS2 | 11246 | 3.099404 | 0.001939 | 0.0102412 | TRUE |
| HBZ | 2150 | 3.098191 | 0.001947 | 0.0102742 | TRUE |
| TRIP6 | 12680 | 3.09815 | 0.001947 | 0.0102742 | TRUE |
| ST5 | 10316 | 3.097943 | 0.001949 | 0.010278 | TRUE |
| ETFB | 768 | 3.097403 | 0.001952 | 0.0102898 | TRUE |
| C17orf67 | 7483 | 3.096915 | 0.001955 | 0.0103032 | TRUE |
| MYBPC1 | 10174 | 3.095729 | 0.001963 | 0.010341 | TRUE |
| DGCR6 | 15015 | 3.094931 | 0.001969 | 0.0103604 | TRUE |
| HLA-DOA | 2854 | 3.09331 | 0.001979 | 0.0104066 | TRUE |
| SPACA3 | 73 | 3.09303 | 0.001981 | 0.010411 | TRUE |
| CAPG | 2233 | 3.089814 | 0.002003 | 0.010503 | TRUE |
| FRMD3 | 6115 | 3.08922 | 0.002007 | 0.0105172 | TRUE |
| C12orf45 | 1 | 3.088043 | 0.002015 | 0.0105483 | TRUE |
| EMP3 | 5332 | 3.087601 | 0.002018 | 0.0105605 | TRUE |
| FBXO15 | 8852 | 3.086831 | 0.002023 | 0.0105776 | TRUE |
| ETFRF1 | 1821 | 3.086079 | 0.002028 | 0.0106005 | TRUE |
| H3F3B | 5720 | 3.085458 | 0.002032 | 0.0106191 | TRUE |
| SERPINB2 | 8466 | 3.084575 | 0.002038 | 0.0106436 | TRUE |
| VPS26B | 778 | 3.083299 | 0.002047 | 0.0106822 | TRUE |
| TNIP3 | 1637 | 3.083177 | 0.002048 | 0.010683 | TRUE |
| EEF1AKMT1 | 5903 | 3.082392 | 0.002053 | 0.0106969 | TRUE |
| GRIK4 | 11601 | 3.081056 | 0.002063 | 0.0107343 | TRUE |
| C1orf226 | 8166 | 3.080608 | 0.002066 | 0.0107408 | TRUE |
| EID2B | 4563 | 3.08058 | 0.002066 | 0.0107408 | TRUE |
| CRB1 | 1811 | 3.080464 | 0.002067 | 0.0107414 | TRUE |
| SNRPC | 5685 | 3.080181 | 0.002069 | 0.0107444 | TRUE |
| TAB2 | 6282 | 3.077736 | 0.002086 | 0.0108294 | TRUE |
| TUBB8 | 12901 | 3.077274 | 0.002089 | 0.0108426 | TRUE |
| PLPP3 | 3562 | 3.075822 | 0.002099 | 0.0108812 | TRUE |
| ZNF710-AS1 | 11503 | 3.075818 | 0.002099 | 0.0108812 | TRUE |
| RPS20 | 11459 | 3.075252 | 0.002103 | 0.0108983 | TRUE |
| HILS1 | 6145 | 3.074623 | 0.002108 | 0.010914 | TRUE |
| PUF60 | 7159 | 3.074138 | 0.002111 | 0.0109226 | TRUE |
| ST20 | 5800 | 3.074093 | 0.002111 | 0.0109226 | TRUE |
| ISYNA1 | 11668 | 3.072763 | 0.002121 | 0.0109678 | TRUE |
| CD52 | 7200 | 3.07213 | 0.002125 | 0.0109838 | TRUE |
| EIF3D | 7018 | 3.071786 | 0.002128 | 0.0109892 | TRUE |
| WDR17 | 8724 | 3.071423 | 0.00213 | 0.0109953 | TRUE |
| CHST1 | 4188 | 3.070911 | 0.002134 | 0.0110105 | TRUE |
| NPFFR2 | 5780 | 3.070087 | 0.00214 | 0.0110373 | TRUE |
| GAP43 | 749 | 3.069957 | 0.002141 | 0.0110385 | TRUE |
| HSD17B6 | 15153 | 3.065198 | 0.002175 | 0.011186 | TRUE |
| SWI5 | 11748 | 3.065154 | 0.002176 | 0.011186 | TRUE |
| SBF2 | 7202 | 3.064991 | 0.002177 | 0.0111865 | TRUE |
| FAM160A2 | 10503 | 3.064688 | 0.002179 | 0.0111942 | TRUE |
| DBX2 | 5467 | 3.064412 | 0.002181 | 0.0112008 | TRUE |
| S100A6 | 6682 | 3.063443 | 0.002188 | 0.0112321 | TRUE |
| PLEKHO2 | 13067 | 3.06188 | 0.0022 | 0.0112738 | TRUE |
| CHP2 | 9192 | 3.061367 | 0.002203 | 0.0112849 | TRUE |
| VEZT | 5022 | 3.061092 | 0.002205 | 0.0112887 | TRUE |
| RARRES3 | 14326 | 3.060962 | 0.002206 | 0.0112899 | TRUE |
| NUDT14 | 1179 | 3.060711 | 0.002208 | 0.0112957 | TRUE |
| POMGNT2 | 9156 | 3.060114 | 0.002213 | 0.0113145 | TRUE |
| PARP1 | 12157 | 3.059533 | 0.002217 | 0.0113296 | TRUE |
| GPC6 | 8091 | 3.059518 | 0.002217 | 0.0113296 | TRUE |
| SIDT2 | 2937 | 3.058173 | 0.002227 | 0.0113769 | TRUE |
| EHBP1L1 | 257 | 3.056804 | 0.002237 | 0.0114252 | TRUE |
| DHDH | 4603 | 3.055772 | 0.002245 | 0.0114572 | TRUE |
| CGREF1 | 13806 | 3.05553 | 0.002247 | 0.0114627 | TRUE |
| SV2B | 12533 | 3.052845 | 0.002267 | 0.0115431 | TRUE |
| RTL6 | 7282 | 3.052024 | 0.002273 | 0.011571 | TRUE |
| PSMA5 | 3934 | 3.05138 | 0.002278 | 0.011592 | TRUE |
| RPL13 | 5206 | 3.050038 | 0.002288 | 0.0116364 | TRUE |
| RASGRP1 | 958 | 3.049144 | 0.002295 | 0.0116628 | TRUE |
| DCAF7 | 5242 | 3.048773 | 0.002298 | 0.0116665 | TRUE |
| MVD | 2352 | 3.047774 | 0.002305 | 0.011694 | TRUE |
| HES5 | 5120 | 3.047611 | 0.002307 | 0.0116965 | TRUE |
| GS1-124K5.4 | 15135 | 3.047381 | 0.002308 | 0.0117017 | TRUE |
| HCK | 4753 | 3.04553 | 0.002323 | 0.0117559 | TRUE |
| VAT1 | 323 | 3.045408 | 0.002324 | 0.0117559 | TRUE |
| SEMA3D | 7555 | 3.045055 | 0.002326 | 0.0117582 | TRUE |
| CDKN1A | 77 | 3.04505 | 0.002326 | 0.0117582 | TRUE |
| SH3BGR | 13820 | 3.044958 | 0.002327 | 0.0117582 | TRUE |
| METTL5 | 1492 | 3.04367 | 0.002337 | 0.0118011 | TRUE |
| ALKBH7 | 10443 | 3.042733 | 0.002344 | 0.0118302 | TRUE |
| SIRPA | 14196 | 3.042537 | 0.002346 | 0.0118341 | TRUE |
| SYT10 | 13982 | 3.03957 | 0.002369 | 0.0119359 | TRUE |
| SLC2A10 | 14513 | 3.039317 | 0.002371 | 0.0119382 | TRUE |
| FAM149A | 11755 | 3.038451 | 0.002378 | 0.0119572 | TRUE |
| LINC00937 | 9707 | 3.037454 | 0.002386 | 0.0119891 | TRUE |
| TANGO6 | 14367 | 3.037251 | 0.002387 | 0.0119933 | TRUE |
| PDCD6 | 10208 | 3.036086 | 0.002397 | 0.0120359 | TRUE |
| TMEM136 | 9654 | 3.035776 | 0.002399 | 0.0120444 | TRUE |
| PRKD2 | 6982 | 3.035528 | 0.002401 | 0.0120505 | TRUE |
| LPCAT2 | 3453 | 3.035225 | 0.002404 | 0.0120552 | TRUE |
| SLC27A5 | 7175 | 3.034872 | 0.002406 | 0.0120612 | TRUE |
| CFAP52 | 1741 | 3.033513 | 0.002417 | 0.0121033 | TRUE |
| TST | 2671 | 3.033317 | 0.002419 | 0.0121033 | TRUE |
| COMMD9 | 12088 | 3.033242 | 0.002419 | 0.0121033 | TRUE |
| RPS3 | 8459 | 3.032021 | 0.002429 | 0.0121484 | TRUE |
| GOLT1A | 4571 | 3.027314 | 0.002467 | 0.0123235 | TRUE |
| HLA-F-AS1 | 13331 | 3.026694 | 0.002472 | 0.0123448 | TRUE |
| DNAJC30 | 2746 | 3.025928 | 0.002479 | 0.0123648 | TRUE |
| SLC44A2 | 1904 | 3.025917 | 0.002479 | 0.0123648 | TRUE |
| CELF3 | 13735 | 3.025484 | 0.002482 | 0.0123785 | TRUE |
| GSTP1 | 10515 | 3.023242 | 0.002501 | 0.0124475 | TRUE |
| ANXA11 | 2431 | 3.022268 | 0.002509 | 0.012479 | TRUE |
| AP3B2 | 906 | 3.022069 | 0.002511 | 0.0124832 | TRUE |
| SLC26A4 | 770 | 3.020405 | 0.002524 | 0.01254 | TRUE |
| NRAP | 14710 | 3.019711 | 0.00253 | 0.012562 | TRUE |
| TRAIP | 8662 | 3.019608 | 0.002531 | 0.012562 | TRUE |
| HMGCL | 6810 | 3.018751 | 0.002538 | 0.0125887 | TRUE |
| FAM196A | 9109 | 3.018039 | 0.002544 | 0.0126103 | TRUE |
| NPTX1 | 3878 | 3.016925 | 0.002554 | 0.0126407 | TRUE |
| SERHL2 | 4580 | 3.015011 | 0.00257 | 0.0127128 | TRUE |
| RPL37 | 13651 | 3.013159 | 0.002585 | 0.0127865 | TRUE |
| FAM187A | 3542 | 3.012932 | 0.002587 | 0.012792 | TRUE |
| ADD3 | 4200 | 3.011637 | 0.002598 | 0.0128426 | TRUE |
| FADS3 | 11357 | 3.011434 | 0.0026 | 0.0128454 | TRUE |
| TRAF3IP2 | 12273 | 3.011091 | 0.002603 | 0.0128503 | TRUE |
| RPL29 | 10276 | 3.01005 | 0.002612 | 0.0128819 | TRUE |
| DPH5 | 4854 | 3.009792 | 0.002614 | 0.0128883 | TRUE |
| ANKRD30BP2 | 9753 | 3.009664 | 0.002615 | 0.0128897 | TRUE |
| ERICH3 | 11972 | 3.009551 | 0.002616 | 0.0128904 | TRUE |
| ANKRD54 | 5219 | 3.009023 | 0.002621 | 0.0129026 | TRUE |
| HPGD | 8023 | 3.003097 | 0.002672 | 0.0131338 | TRUE |
| FNDC11 | 81 | 3.00261 | 0.002677 | 0.0131507 | TRUE |
| ARL15 | 1159 | 3.002259 | 0.00268 | 0.0131576 | TRUE |
| PIM2 | 8300 | 3.001698 | 0.002685 | 0.0131746 | TRUE |
| BAG3 | 2611 | 3.001677 | 0.002685 | 0.0131746 | TRUE |
| FZD2 | 12812 | 3.000714 | 0.002693 | 0.0132039 | TRUE |
| SIN3B | 11097 | 3.00046 | 0.002696 | 0.0132107 | TRUE |
| PLAC9P1 | 4815 | 3.000159 | 0.002698 | 0.0132196 | TRUE |
| ZFP36L1 | 13077 | 2.999739 | 0.002702 | 0.0132337 | TRUE |
| CCT7 | 5483 | 2.997684 | 0.00272 | 0.0133108 | TRUE |
| PERM1 | 4287 | 2.996908 | 0.002727 | 0.0133405 | TRUE |
| EML3 | 1545 | 2.996296 | 0.002733 | 0.0133632 | TRUE |
| TFEC | 1500 | 2.994251 | 0.002751 | 0.0134237 | TRUE |
| ENDOU | 3616 | 2.994076 | 0.002753 | 0.0134243 | TRUE |
| VWCE | 4801 | 2.993828 | 0.002755 | 0.0134255 | TRUE |
| HBG1 | 10888 | 2.992987 | 0.002763 | 0.0134584 | TRUE |
| PPP1R32 | 12527 | 2.991563 | 0.002776 | 0.0135087 | TRUE |
| GOLM1 | 9549 | 2.991388 | 0.002777 | 0.0135107 | TRUE |
| ASH2L | 5857 | 2.990435 | 0.002786 | 0.0135418 | TRUE |
| FAM212A | 2438 | 2.988909 | 0.0028 | 0.0135885 | TRUE |
| STRIP1 | 11427 | 2.988144 | 0.002807 | 0.0136183 | TRUE |
| FBXO41 | 11497 | 2.987333 | 0.002814 | 0.013646 | TRUE |
| PRIM2 | 2697 | 2.986834 | 0.002819 | 0.0136599 | TRUE |
| WIF1 | 2289 | 2.983858 | 0.002846 | 0.0137595 | TRUE |
| SLC30A3 | 1710 | 2.983858 | 0.002846 | 0.0137595 | TRUE |
| C2orf69 | 686 | 2.983854 | 0.002846 | 0.0137595 | TRUE |
| P2RY12 | 452 | 2.982244 | 0.002861 | 0.0138192 | TRUE |
| DDOST | 229 | 2.981721 | 0.002866 | 0.0138386 | TRUE |
| EPB41L4A-AS1 | 3612 | 2.981576 | 0.002868 | 0.0138409 | TRUE |
| LIN7B | 11491 | 2.981441 | 0.002869 | 0.0138427 | TRUE |
| POLR2J | 10132 | 2.981338 | 0.00287 | 0.0138431 | TRUE |
| GINS2 | 14208 | 2.978613 | 0.002896 | 0.0139367 | TRUE |
| SHF | 5290 | 2.974585 | 0.002934 | 0.0141036 | TRUE |
| RSU1 | 9465 | 2.973833 | 0.002941 | 0.0141295 | TRUE |
| RPS2P32 | 12827 | 2.973612 | 0.002943 | 0.0141313 | TRUE |
| FAM212B | 10545 | 2.973593 | 0.002943 | 0.0141313 | TRUE |
| CYB5R1 | 7336 | 2.97343 | 0.002945 | 0.0141313 | TRUE |
| PRAM1 | 5553 | 2.973417 | 0.002945 | 0.0141313 | TRUE |
| STOX1 | 926 | 2.973118 | 0.002948 | 0.0141407 | TRUE |
| TNNI3 | 848 | 2.972818 | 0.002951 | 0.0141459 | TRUE |
| SLC29A3 | 3154 | 2.972395 | 0.002955 | 0.0141611 | TRUE |
| RGS19 | 12153 | 2.971732 | 0.002961 | 0.014183 | TRUE |
| KANK1 | 14594 | 2.968746 | 0.00299 | 0.0143015 | TRUE |
| PRDX4 | 9122 | 2.968359 | 0.002994 | 0.0143115 | TRUE |
| TTC38 | 12608 | 2.968302 | 0.002995 | 0.0143115 | TRUE |
| FAM111A | 6900 | 2.967951 | 0.002998 | 0.0143235 | TRUE |
| LOC644189 | 4041 | 2.967256 | 0.003005 | 0.0143515 | TRUE |
| TYRO3 | 2592 | 2.966131 | 0.003016 | 0.0143997 | TRUE |
| AQP4 | 10650 | 2.965295 | 0.003024 | 0.0144345 | TRUE |
| LRRC27 | 8187 | 2.963392 | 0.003043 | 0.0145196 | TRUE |
| TPM3P9 | 9640 | 2.962321 | 0.003053 | 0.0145658 | TRUE |
| NDUFS6 | 12313 | 2.961594 | 0.003061 | 0.0145869 | TRUE |
| MESD | 10929 | 2.960898 | 0.003067 | 0.0146154 | TRUE |
| GNG10 | 13446 | 2.959752 | 0.003079 | 0.0146565 | TRUE |
| MLIP | 1525 | 2.959471 | 0.003082 | 0.0146611 | TRUE |
| SLC1A3 | 7886 | 2.959468 | 0.003082 | 0.0146611 | TRUE |
| MAGIX | 12056 | 2.958782 | 0.003089 | 0.0146893 | TRUE |
| PITPNM2 | 4522 | 2.957971 | 0.003097 | 0.014719 | TRUE |
| POC1A | 1063 | 2.957602 | 0.0031 | 0.0147301 | TRUE |
| DBNL | 5116 | 2.957343 | 0.003103 | 0.0147356 | TRUE |
| DRAIC | 1199 | 2.956714 | 0.003109 | 0.0147579 | TRUE |
| BDNF | 3070 | 2.95594 | 0.003117 | 0.0147893 | TRUE |
| CCDC153 | 1046 | 2.955613 | 0.00312 | 0.0147929 | TRUE |
| NIT2 | 1168 | 2.955601 | 0.003121 | 0.0147929 | TRUE |
| VAV1 | 5156 | 2.955531 | 0.003121 | 0.0147929 | TRUE |
| PON2 | 3730 | 2.954631 | 0.00313 | 0.0148255 | TRUE |
| ARHGAP5-AS1 | 11386 | 2.954234 | 0.003134 | 0.0148356 | TRUE |
| MASP1 | 14617 | 2.953427 | 0.003143 | 0.0148652 | TRUE |
| CYBB | 14382 | 2.953061 | 0.003146 | 0.0148748 | TRUE |
| JAML | 2754 | 2.95197 | 0.003158 | 0.0149121 | TRUE |
| RGS20 | 609 | 2.949925 | 0.003179 | 0.0149779 | TRUE |
| CENPP | 1385 | 2.949678 | 0.003181 | 0.0149832 | TRUE |
| MAF | 4192 | 2.948938 | 0.003189 | 0.0149966 | TRUE |
| IDH1-AS1 | 5909 | 2.948651 | 0.003192 | 0.015006 | TRUE |
| NXN | 10125 | 2.948286 | 0.003195 | 0.0150192 | TRUE |
| PRRG3 | 11076 | 2.947965 | 0.003199 | 0.0150303 | TRUE |
| CERS1 | 4341 | 2.947584 | 0.003203 | 0.0150443 | TRUE |
| CACNG3 | 4600 | 2.947471 | 0.003204 | 0.0150446 | TRUE |
| BTF3P11 | 2864 | 2.947138 | 0.003207 | 0.0150525 | TRUE |
| PGAM5 | 5554 | 2.946899 | 0.00321 | 0.0150551 | TRUE |
| NT5DC2 | 14197 | 2.946491 | 0.003214 | 0.0150644 | TRUE |
| GPD2 | 9612 | 2.94616 | 0.003217 | 0.015073 | TRUE |
| ATP5MF | 8239 | 2.945779 | 0.003221 | 0.015087 | TRUE |
| BTBD9 | 136 | 2.945096 | 0.003229 | 0.0151128 | TRUE |
| TUBB3 | 11527 | 2.944185 | 0.003238 | 0.0151429 | TRUE |
| TMPRSS5 | 8547 | 2.944173 | 0.003238 | 0.0151429 | TRUE |
| PEX11G | 4535 | 2.943864 | 0.003241 | 0.0151535 | TRUE |
| DYNLT1 | 7999 | 2.943129 | 0.003249 | 0.0151759 | TRUE |
| RGS14 | 9897 | 2.941707 | 0.003264 | 0.0152337 | TRUE |
| LOC644936 | 9245 | 2.941182 | 0.00327 | 0.0152488 | TRUE |
| TCAP | 4389 | 2.941053 | 0.003271 | 0.0152506 | TRUE |
| C1orf194 | 7790 | 2.937779 | 0.003306 | 0.0153851 | TRUE |
| CXCL16 | 6154 | 2.937294 | 0.003311 | 0.0153994 | TRUE |
| SMPD1 | 3243 | 2.937165 | 0.003312 | 0.0153994 | TRUE |
| TMEM54 | 2659 | 2.932234 | 0.003365 | 0.0156208 | TRUE |
| PTHLH | 1139 | 2.93223 | 0.003365 | 0.0156208 | TRUE |
| TIMM17B | 10845 | 2.931821 | 0.00337 | 0.0156332 | TRUE |
| SERINC1 | 2829 | 2.931799 | 0.00337 | 0.0156332 | TRUE |
| FCER1G | 9741 | 2.931486 | 0.003373 | 0.0156397 | TRUE |
| PPP2R3C | 9038 | 2.930572 | 0.003383 | 0.0156811 | TRUE |
| MBOAT2 | 3055 | 2.929395 | 0.003396 | 0.015736 | TRUE |
| MLC1 | 2129 | 2.928464 | 0.003406 | 0.0157738 | TRUE |
| CHD3 | 306 | 2.927417 | 0.003418 | 0.0158223 | TRUE |
| VOPP1 | 7182 | 2.927036 | 0.003422 | 0.0158277 | TRUE |
| EYA2 | 6038 | 2.926075 | 0.003433 | 0.0158603 | TRUE |
| AP3M2 | 226 | 2.926027 | 0.003433 | 0.0158603 | TRUE |
| TMED10 | 6061 | 2.925357 | 0.003441 | 0.0158898 | TRUE |
| PRKAA2 | 3763 | 2.924961 | 0.003445 | 0.0159054 | TRUE |
| ALG1L | 8268 | 2.92482 | 0.003447 | 0.0159066 | TRUE |
| PHF13 | 8478 | 2.924754 | 0.003447 | 0.0159066 | TRUE |
| MAP3K19 | 5192 | 2.924655 | 0.003448 | 0.0159069 | TRUE |
| CASP1 | 15160 | 2.92358 | 0.00346 | 0.0159572 | TRUE |
| UHMK1 | 14973 | 2.922763 | 0.003469 | 0.0159945 | TRUE |
| DDX25 | 5060 | 2.921456 | 0.003484 | 0.016057 | TRUE |
| PDLIM5 | 6768 | 2.919124 | 0.00351 | 0.0161681 | TRUE |
| TUBB2B | 1479 | 2.918373 | 0.003519 | 0.0161996 | TRUE |
| AZIN2 | 14160 | 2.918333 | 0.003519 | 0.0161996 | TRUE |
| KIAA1147 | 4582 | 2.917448 | 0.003529 | 0.0162409 | TRUE |
| AQP1 | 6979 | 2.915907 | 0.003547 | 0.0163165 | TRUE |
| STARD3NL | 6797 | 2.914971 | 0.003557 | 0.0163559 | TRUE |
| SLC2A14 | 3952 | 2.914118 | 0.003567 | 0.0163958 | TRUE |
| CCR5 | 1748 | 2.913812 | 0.00357 | 0.0164071 | TRUE |
| SLC44A3 | 10365 | 2.912154 | 0.003589 | 0.0164825 | TRUE |
| NACA | 3244 | 2.908716 | 0.003629 | 0.0166475 | TRUE |
| ACKR3 | 4900 | 2.908492 | 0.003632 | 0.0166507 | TRUE |
| WNT4 | 4515 | 2.908472 | 0.003632 | 0.0166507 | TRUE |
| PDRG1 | 13312 | 2.908301 | 0.003634 | 0.016655 | TRUE |
| LAMTOR4 | 7185 | 2.907977 | 0.003638 | 0.0166673 | TRUE |
| HIST1H2BH | 11413 | 2.906597 | 0.003654 | 0.0167352 | TRUE |
| TRIL | 13641 | 2.906488 | 0.003655 | 0.0167352 | TRUE |
| POLR2J4 | 10258 | 2.906432 | 0.003656 | 0.0167352 | TRUE |
| PLCD3 | 5191 | 2.905495 | 0.003667 | 0.0167755 | TRUE |
| ZFAND3 | 14108 | 2.905212 | 0.00367 | 0.0167858 | TRUE |
| WASHC3 | 4523 | 2.905041 | 0.003672 | 0.01679 | TRUE |
| ECHDC3 | 14874 | 2.904168 | 0.003682 | 0.016832 | TRUE |
| RPLP0P2 | 11906 | 2.903953 | 0.003685 | 0.0168387 | TRUE |
| HSPA8 | 15614 | 2.902803 | 0.003698 | 0.0168957 | TRUE |
| PRPS1 | 3063 | 2.900136 | 0.00373 | 0.0170202 | TRUE |
| CCDC90B | 10553 | 2.899147 | 0.003742 | 0.017069 | TRUE |
| CLIP3 | 6701 | 2.898885 | 0.003745 | 0.0170733 | TRUE |
| SYNGR3 | 14680 | 2.897834 | 0.003757 | 0.0171256 | TRUE |
| ANKRD30B | 7197 | 2.894811 | 0.003794 | 0.017268 | TRUE |
| ABR | 13599 | 2.894699 | 0.003795 | 0.017268 | TRUE |
| SLBP | 1670 | 2.894687 | 0.003795 | 0.017268 | TRUE |
| ARL10 | 15592 | 2.894443 | 0.003798 | 0.0172764 | TRUE |
| GLRA3 | 13019 | 2.894257 | 0.003801 | 0.0172816 | TRUE |
| C6orf52 | 4885 | 2.893736 | 0.003807 | 0.0173053 | TRUE |
| TEK | 14106 | 2.893286 | 0.003812 | 0.0173251 | TRUE |
| BCL11B | 8235 | 2.893048 | 0.003815 | 0.0173332 | TRUE |
| ZDHHC4 | 1677 | 2.891608 | 0.003833 | 0.0173825 | TRUE |
| ZCCHC12 | 898 | 2.891298 | 0.003837 | 0.0173946 | TRUE |
| TRAPPC5 | 6060 | 2.889601 | 0.003857 | 0.0174786 | TRUE |
| TPI1 | 6126 | 2.887547 | 0.003883 | 0.0175738 | TRUE |
| YWHAB | 96 | 2.887432 | 0.003884 | 0.0175741 | TRUE |
| BFSP1 | 14767 | 2.887007 | 0.003889 | 0.0175928 | TRUE |
| CES4A | 1982 | 2.886645 | 0.003894 | 0.017608 | TRUE |
| UBE2A | 11925 | 2.884739 | 0.003917 | 0.0176944 | TRUE |
| DMGDH | 6536 | 2.884405 | 0.003922 | 0.0177081 | TRUE |
| ITPKB | 921 | 2.883736 | 0.00393 | 0.0177406 | TRUE |
| ASXL3 | 5285 | 2.882625 | 0.003944 | 0.017793 | TRUE |
| GUK1 | 1138 | 2.881511 | 0.003958 | 0.0178458 | TRUE |
| GRM3 | 10105 | 2.880427 | 0.003971 | 0.0178944 | TRUE |
| KIAA1191 | 709 | 2.880381 | 0.003972 | 0.0178944 | TRUE |
| LGR4 | 7394 | 2.879255 | 0.003986 | 0.0179326 | TRUE |
| LINC00595 | 2810 | 2.878551 | 0.003995 | 0.0179533 | TRUE |
| CLPSL1 | 12379 | 2.878527 | 0.003995 | 0.0179533 | TRUE |
| CLBA1 | 11809 | 2.877509 | 0.004008 | 0.0180062 | TRUE |
| LRRIQ1 | 10293 | 2.875535 | 0.004033 | 0.0181088 | TRUE |
| AKR7A2P1 | 3563 | 2.873195 | 0.004063 | 0.0182203 | TRUE |
| ARRB2 | 1580 | 2.872641 | 0.004071 | 0.0182399 | TRUE |
| ANGPT1 | 7590 | 2.871726 | 0.004082 | 0.0182707 | TRUE |
| MTMR9LP | 7968 | 2.871337 | 0.004087 | 0.0182848 | TRUE |
| PRR13 | 13906 | 2.871212 | 0.004089 | 0.0182848 | TRUE |
| GLI3 | 1399 | 2.870282 | 0.004101 | 0.0183331 | TRUE |
| ZNF350 | 11851 | 2.870197 | 0.004102 | 0.0183331 | TRUE |
| CCDC77 | 2853 | 2.869686 | 0.004109 | 0.0183575 | TRUE |
| LARP1 | 3408 | 2.868034 | 0.00413 | 0.0184419 | TRUE |
| DENND2C | 10946 | 2.867156 | 0.004142 | 0.0184785 | TRUE |
| LINC02381 | 528 | 2.867025 | 0.004144 | 0.0184809 | TRUE |
| KCTD15 | 5440 | 2.86577 | 0.00416 | 0.018549 | TRUE |
| EFNA2 | 597 | 2.865261 | 0.004167 | 0.0185682 | TRUE |
| PABPC1L | 4767 | 2.864172 | 0.004181 | 0.0186216 | TRUE |
| MPV17 | 5701 | 2.86366 | 0.004188 | 0.0186464 | TRUE |
| THAP8 | 2123 | 2.863284 | 0.004193 | 0.0186535 | TRUE |
| PCBD1 | 6584 | 2.863172 | 0.004194 | 0.0186539 | TRUE |
| RHOA | 517 | 2.86288 | 0.004198 | 0.018659 | TRUE |
| RIT2 | 13074 | 2.862814 | 0.004199 | 0.018659 | TRUE |
| CENPX | 443 | 2.862686 | 0.004201 | 0.0186613 | TRUE |
| GHR | 2154 | 2.858661 | 0.004254 | 0.0188836 | TRUE |
| BEX2 | 6575 | 2.858342 | 0.004259 | 0.0188972 | TRUE |
| PI4KA | 5369 | 2.858179 | 0.004261 | 0.0189016 | TRUE |
| RPS15 | 9764 | 2.857794 | 0.004266 | 0.0189191 | TRUE |
| FAM129A | 8107 | 2.857488 | 0.00427 | 0.018932 | TRUE |
| TBXT | 11354 | 2.855939 | 0.004291 | 0.0190138 | TRUE |
| ARPC2 | 176 | 2.855694 | 0.004294 | 0.0190223 | TRUE |
| LRRC61 | 3419 | 2.855618 | 0.004295 | 0.0190223 | TRUE |
| GAR1 | 14298 | 2.855465 | 0.004297 | 0.0190261 | TRUE |
| BEGAIN | 10377 | 2.855051 | 0.004303 | 0.0190293 | TRUE |
| SNHG19 | 12199 | 2.853685 | 0.004322 | 0.0191005 | TRUE |
| BIRC3 | 12013 | 2.853476 | 0.004324 | 0.0191077 | TRUE |
| TUBA1A | 9014 | 2.852777 | 0.004334 | 0.019139 | TRUE |
| C10orf105 | 3946 | 2.852155 | 0.004342 | 0.019171 | TRUE |
| PTGES | 8592 | 2.849036 | 0.004385 | 0.0193381 | TRUE |
| SNRPB2 | 4258 | 2.848425 | 0.004394 | 0.0193644 | TRUE |
| BSPRY | 4074 | 2.847852 | 0.004402 | 0.0193938 | TRUE |
| SHC1 | 7222 | 2.846526 | 0.00442 | 0.0194693 | TRUE |
| HACD1 | 5189 | 2.845917 | 0.004428 | 0.0194956 | TRUE |
| ADGRL3 | 6522 | 2.845634 | 0.004432 | 0.0195074 | TRUE |
| VIM | 3729 | 2.845274 | 0.004437 | 0.019524 | TRUE |
| SRRD | 4622 | 2.844952 | 0.004442 | 0.0195352 | TRUE |
| FAM105A | 1900 | 2.844604 | 0.004447 | 0.0195431 | TRUE |
| PCK2 | 9856 | 2.844212 | 0.004452 | 0.0195616 | TRUE |
| MZT1 | 12221 | 2.843718 | 0.004459 | 0.0195865 | TRUE |
| ZNF239 | 1052 | 2.842989 | 0.004469 | 0.0196093 | TRUE |
| STARD7 | 9693 | 2.84227 | 0.004479 | 0.0196481 | TRUE |
| LINC01410 | 12599 | 2.839786 | 0.004514 | 0.0197795 | TRUE |
| RBBP7 | 15335 | 2.83899 | 0.004526 | 0.0198234 | TRUE |
| TPI1P2 | 4715 | 2.838175 | 0.004537 | 0.0198629 | TRUE |
| FAM102B | 13602 | 2.83764 | 0.004545 | 0.0198851 | TRUE |
| STK25 | 2185 | 2.836199 | 0.004565 | 0.0199695 | TRUE |
| UQCC3 | 7661 | 2.834868 | 0.004584 | 0.0200416 | TRUE |
| PRSS23 | 7080 | 2.834386 | 0.004591 | 0.0200663 | TRUE |
| FEZF2 | 6592 | 2.833271 | 0.004607 | 0.0201308 | TRUE |
| LOC257396 | 12784 | 2.832424 | 0.00462 | 0.0201786 | TRUE |
| RPL31P11 | 712 | 2.831521 | 0.004633 | 0.02023 | TRUE |
| ARHGDIA | 13519 | 2.831376 | 0.004635 | 0.0202332 | TRUE |
| EMID1 | 2880 | 2.83105 | 0.00464 | 0.0202428 | TRUE |
| SHISA9 | 7285 | 2.8307 | 0.004645 | 0.0202537 | TRUE |
| OPTN | 2015 | 2.827572 | 0.00469 | 0.0204298 | TRUE |
| CD24 | 5382 | 2.826417 | 0.004707 | 0.0204979 | TRUE |
| HBQ1 | 10858 | 2.824708 | 0.004732 | 0.0205881 | TRUE |
| SEC11C | 927 | 2.824654 | 0.004733 | 0.0205881 | TRUE |
| ATP6V0CP3 | 3711 | 2.823795 | 0.004746 | 0.0206319 | TRUE |
| PLXDC2 | 14111 | 2.822378 | 0.004767 | 0.0207037 | TRUE |
| DCAF15 | 3337 | 2.822357 | 0.004767 | 0.0207037 | TRUE |
| ATP5ME | 1702 | 2.822324 | 0.004768 | 0.0207037 | TRUE |
| GABRE | 140 | 2.820884 | 0.004789 | 0.0207853 | TRUE |
| P2RY6 | 12228 | 2.819954 | 0.004803 | 0.0208399 | TRUE |
| NPPA | 8732 | 2.817173 | 0.004845 | 0.0210095 | TRUE |
| PALD1 | 534 | 2.816447 | 0.004856 | 0.0210513 | TRUE |
| LOC101930085 | 2080 | 2.816133 | 0.004861 | 0.021066 | TRUE |
| BAIAP2L2 | 10103 | 2.814854 | 0.00488 | 0.0211266 | TRUE |
| SMARCA4 | 11337 | 2.812688 | 0.004913 | 0.0212519 | TRUE |
| SEC63 | 6620 | 2.812686 | 0.004913 | 0.0212519 | TRUE |
| DNAJC5 | 4690 | 2.811483 | 0.004931 | 0.0213256 | TRUE |
| SCN9A | 8090 | 2.810749 | 0.004943 | 0.0213563 | TRUE |
| SEC11A | 1033 | 2.810664 | 0.004944 | 0.0213563 | TRUE |
| FAHD2A | 1665 | 2.810519 | 0.004946 | 0.0213601 | TRUE |
| FAM120C | 8611 | 2.809571 | 0.004961 | 0.0214112 | TRUE |
| CPE | 3573 | 2.808939 | 0.004971 | 0.0214458 | TRUE |
| SH3PXD2B | 15350 | 2.807809 | 0.004988 | 0.021505 | TRUE |
| PTBP2 | 1070 | 2.805912 | 0.005017 | 0.0216141 | TRUE |
| ZNRF3 | 5280 | 2.80528 | 0.005027 | 0.0216497 | TRUE |
| RAB8B | 8933 | 2.805205 | 0.005028 | 0.0216497 | TRUE |
| SSR2 | 9692 | 2.804188 | 0.005044 | 0.0217121 | TRUE |
| FDPS | 10543 | 2.803119 | 0.005061 | 0.0217722 | TRUE |
| C19orf12 | 8532 | 2.802893 | 0.005065 | 0.0217815 | TRUE |
| HSP90AB1 | 8660 | 2.802691 | 0.005068 | 0.0217891 | TRUE |
| HRASLS | 2780 | 2.802274 | 0.005074 | 0.0218108 | TRUE |
| LYPLAL1 | 3662 | 2.802193 | 0.005076 | 0.0218108 | TRUE |
| SFRP1 | 5698 | 2.801857 | 0.005081 | 0.0218275 | TRUE |
| SLITRK6 | 13706 | 2.801751 | 0.005083 | 0.0218287 | TRUE |
| COPS9 | 12998 | 2.801221 | 0.005091 | 0.0218586 | TRUE |
| OXTR | 5517 | 2.800842 | 0.005097 | 0.0218782 | TRUE |
| LRCH2 | 13650 | 2.800675 | 0.0051 | 0.0218836 | TRUE |
| GLT8D2 | 14024 | 2.800177 | 0.005107 | 0.0219053 | TRUE |
| MRC1 | 14314 | 2.800063 | 0.005109 | 0.0219071 | TRUE |
| PPID | 5256 | 2.799655 | 0.005116 | 0.0219227 | TRUE |
| LGI4 | 4911 | 2.798737 | 0.00513 | 0.0219671 | TRUE |
| RAMP1 | 5802 | 2.797902 | 0.005144 | 0.0220179 | TRUE |
| SLC52A1 | 8236 | 2.796286 | 0.005169 | 0.0221162 | TRUE |
| ZWINT | 15031 | 2.795839 | 0.005177 | 0.0221347 | TRUE |
| RSPO4 | 3546 | 2.795214 | 0.005187 | 0.0221715 | TRUE |
| GEMIN4 | 2881 | 2.793988 | 0.005206 | 0.0222496 | TRUE |
| AEBP1 | 10483 | 2.792779 | 0.005226 | 0.0223086 | TRUE |
| SARDH | 3093 | 2.79216 | 0.005236 | 0.0223391 | TRUE |
| SYN2 | 715 | 2.792042 | 0.005238 | 0.0223404 | TRUE |
| STX12 | 10362 | 2.791553 | 0.005246 | 0.0223566 | TRUE |
| MAFG | 1577 | 2.791426 | 0.005248 | 0.0223569 | TRUE |
| FADS1 | 15626 | 2.791373 | 0.005248 | 0.0223569 | TRUE |
| ASL | 9313 | 2.789895 | 0.005273 | 0.022447 | TRUE |
| RPL36A | 12959 | 2.788936 | 0.005288 | 0.0225074 | TRUE |
| PGAP1 | 9123 | 2.787527 | 0.005311 | 0.0225809 | TRUE |
| ZNF277 | 12162 | 2.787313 | 0.005315 | 0.0225897 | TRUE |
| TERF2IP | 14245 | 2.787049 | 0.005319 | 0.022602 | TRUE |
| ACVR2B | 14448 | 2.786601 | 0.005326 | 0.0226162 | TRUE |
| DHRS12 | 8717 | 2.786581 | 0.005327 | 0.0226162 | TRUE |
| CRACR2B | 10530 | 2.78645 | 0.005329 | 0.0226192 | TRUE |
| ANG | 5704 | 2.78622 | 0.005333 | 0.0226291 | TRUE |
| GPR17 | 13654 | 2.785445 | 0.005345 | 0.0226771 | TRUE |
| ANGPTL6 | 6869 | 2.783527 | 0.005377 | 0.0227745 | TRUE |
| MRVI1 | 4107 | 2.782858 | 0.005388 | 0.0228154 | TRUE |
| LOH12CR2 | 7294 | 2.782612 | 0.005392 | 0.0228265 | TRUE |
| SLC24A3 | 11162 | 2.781999 | 0.005403 | 0.0228635 | TRUE |
| MED24 | 5072 | 2.780034 | 0.005435 | 0.0229836 | TRUE |
| TNFRSF11A | 6284 | 2.778581 | 0.00546 | 0.0230679 | TRUE |
| LOC728715 | 11319 | 2.777827 | 0.005472 | 0.0231028 | TRUE |
| SPATA18 | 1243 | 2.776719 | 0.005491 | 0.0231692 | TRUE |
| IL4I1 | 14999 | 2.776328 | 0.005498 | 0.0231788 | TRUE |
| ATAT1 | 8862 | 2.776321 | 0.005498 | 0.0231788 | TRUE |
| WDR25 | 39 | 2.775407 | 0.005513 | 0.0232315 | TRUE |
| RAB13 | 11317 | 2.774713 | 0.005525 | 0.0232686 | TRUE |
| NHP2 | 4138 | 2.772412 | 0.005564 | 0.0234172 | TRUE |
| TTC9B | 11525 | 2.772379 | 0.005565 | 0.0234172 | TRUE |
| ISLR2 | 12770 | 2.770435 | 0.005598 | 0.0235258 | TRUE |
| SAMD15 | 5829 | 2.768826 | 0.005626 | 0.0236359 | TRUE |
| PSMA6 | 13886 | 2.768652 | 0.005629 | 0.0236422 | TRUE |
| SYT9 | 6881 | 2.767598 | 0.005647 | 0.0236997 | TRUE |
| RORC | 1106 | 2.767487 | 0.005649 | 0.0237014 | TRUE |
| RYR3 | 5062 | 2.767226 | 0.005654 | 0.023714 | TRUE |
| DNAH14 | 415 | 2.766862 | 0.00566 | 0.0237341 | TRUE |
| GGCT | 9898 | 2.766429 | 0.005667 | 0.0237593 | TRUE |
| PHGDH | 888 | 2.766284 | 0.00567 | 0.0237635 | TRUE |
| NPB | 14437 | 2.765966 | 0.005675 | 0.0237803 | TRUE |
| ZNF525 | 7680 | 2.765643 | 0.005681 | 0.0237975 | TRUE |
| SMO | 11793 | 2.76492 | 0.005694 | 0.0238311 | TRUE |
| PMM1 | 10654 | 2.763724 | 0.005715 | 0.0239067 | TRUE |
| RAET1E-AS1 | 2169 | 2.763712 | 0.005715 | 0.0239067 | TRUE |
| A1BG-AS1 | 3723 | 2.762283 | 0.00574 | 0.0240052 | TRUE |
| KCNA3 | 2883 | 2.761941 | 0.005746 | 0.0240232 | TRUE |
| PSMD8 | 7566 | 2.761421 | 0.005755 | 0.0240493 | TRUE |
| LONRF2 | 3306 | 2.760644 | 0.005769 | 0.0240809 | TRUE |
| FRMPD3 | 10959 | 2.760126 | 0.005778 | 0.0241127 | TRUE |
| GCNT1 | 12139 | 2.759658 | 0.005786 | 0.0241408 | TRUE |
| DHCR7 | 8701 | 2.75916 | 0.005795 | 0.0241712 | TRUE |
| NDUFC2 | 5352 | 2.758809 | 0.005801 | 0.0241778 | TRUE |
| SLC25A23 | 8883 | 2.758399 | 0.005809 | 0.0241888 | TRUE |
| MEIG1 | 4858 | 2.756415 | 0.005844 | 0.0243231 | TRUE |
| PGLS | 11762 | 2.754497 | 0.005878 | 0.0244596 | TRUE |
| TMSB4Y | 12105 | 2.754319 | 0.005881 | 0.0244664 | TRUE |
| RPL30 | 11306 | 2.754213 | 0.005883 | 0.0244678 | TRUE |
| FTL | 9028 | 2.754115 | 0.005885 | 0.0244686 | TRUE |
| S100A5 | 7194 | 2.753765 | 0.005891 | 0.0244883 | TRUE |
| TRPV2 | 9088 | 2.752766 | 0.005909 | 0.0245566 | TRUE |
| NR4A2 | 6781 | 2.751761 | 0.005928 | 0.0246255 | TRUE |
| BCR | 2579 | 2.750678 | 0.005947 | 0.0247005 | TRUE |
| HID1 | 4786 | 2.75043 | 0.005952 | 0.0247126 | TRUE |
| FUT9 | 14315 | 2.748284 | 0.005991 | 0.0248684 | TRUE |
| TLR1 | 11086 | 2.747163 | 0.006011 | 0.0249469 | TRUE |
| COX7A2L | 2601 | 2.746494 | 0.006024 | 0.024978 | TRUE |
| ITM2C | 3168 | 2.744943 | 0.006052 | 0.0250697 | TRUE |
| NRCAM | 5421 | 2.743014 | 0.006088 | 0.0252041 | TRUE |
| CTNNBIP1 | 7586 | 2.742836 | 0.006091 | 0.025211 | TRUE |
| HERC5 | 14460 | 2.741719 | 0.006112 | 0.0252635 | TRUE |
| FCGR1B | 13969 | 2.738089 | 0.00618 | 0.0255171 | TRUE |
| ZNF667-AS1 | 4263 | 2.737217 | 0.006196 | 0.0255579 | TRUE |
| PLEKHA7 | 4895 | 2.736431 | 0.006211 | 0.0256055 | TRUE |
| MAP2K1 | 9620 | 2.73585 | 0.006222 | 0.025644 | TRUE |
| HSD17B14 | 3172 | 2.734787 | 0.006242 | 0.0257123 | TRUE |
| MRPL51 | 13329 | 2.734714 | 0.006243 | 0.0257123 | TRUE |
| EEF1G | 5042 | 2.732829 | 0.006279 | 0.0258531 | TRUE |
| MRPL10 | 12646 | 2.731824 | 0.006298 | 0.0259048 | TRUE |
| UROS | 13115 | 2.729799 | 0.006337 | 0.026037 | TRUE |
| SLC25A14 | 3974 | 2.727559 | 0.00638 | 0.0262007 | TRUE |
| ZNF624 | 9376 | 2.726961 | 0.006392 | 0.0262414 | TRUE |
| ACSF2 | 3608 | 2.726369 | 0.006404 | 0.0262816 | TRUE |
| CCDC148 | 11310 | 2.726043 | 0.00641 | 0.0263006 | TRUE |
| KCNMB2 | 6628 | 2.72407 | 0.006448 | 0.0264236 | TRUE |
| RLBP1 | 4238 | 2.723711 | 0.006455 | 0.0264454 | TRUE |
| GABARAPL2 | 3012 | 2.723292 | 0.006463 | 0.026472 | TRUE |
| WIZ | 9704 | 2.723117 | 0.006467 | 0.0264791 | TRUE |
| TOMM7 | 1072 | 2.721738 | 0.006494 | 0.0265618 | TRUE |
| IL3RA | 13387 | 2.721655 | 0.006496 | 0.0265618 | TRUE |
| LCTL | 775 | 2.719829 | 0.006532 | 0.0266906 | TRUE |
| WWC1 | 7550 | 2.719804 | 0.006532 | 0.0266906 | TRUE |
| CLPS | 13960 | 2.719796 | 0.006532 | 0.0266906 | TRUE |
| LY96 | 3218 | 2.719633 | 0.006535 | 0.0266968 | TRUE |
| RPS27A | 8272 | 2.718455 | 0.006559 | 0.026771 | TRUE |
| DNAH12 | 14982 | 2.715803 | 0.006612 | 0.0269583 | TRUE |
| ARHGAP22 | 837 | 2.715481 | 0.006618 | 0.0269775 | TRUE |
| STAB1 | 12144 | 2.715046 | 0.006627 | 0.0269988 | TRUE |
| ALYREF | 4861 | 2.714874 | 0.00663 | 0.0270058 | TRUE |
| HSD11B1L | 4322 | 2.714352 | 0.006641 | 0.0270414 | TRUE |
| NELL2 | 8260 | 2.713178 | 0.006664 | 0.0271162 | TRUE |
| NMT1 | 5306 | 2.71259 | 0.006676 | 0.0271502 | TRUE |
| VEGFB | 10698 | 2.711843 | 0.006691 | 0.0272022 | TRUE |
| CNTN1 | 15555 | 2.708393 | 0.006761 | 0.0274531 | TRUE |
| TSR3 | 5703 | 2.708237 | 0.006764 | 0.0274589 | TRUE |
| TMEM68 | 3260 | 2.707974 | 0.00677 | 0.0274735 | TRUE |
| LHFPL3 | 4624 | 2.705543 | 0.006819 | 0.0276611 | TRUE |
| CD163 | 8602 | 2.704983 | 0.006831 | 0.0277006 | TRUE |
| KLHL2 | 2187 | 2.704689 | 0.006837 | 0.0277179 | TRUE |
| GPR25 | 13181 | 2.702191 | 0.006888 | 0.0279126 | TRUE |
| IFI30 | 1648 | 2.702057 | 0.006891 | 0.0279166 | TRUE |
| MGAT4B | 2224 | 2.699988 | 0.006934 | 0.028069 | TRUE |
| WISP2 | 12464 | 2.699673 | 0.006941 | 0.0280883 | TRUE |
| HSPA6 | 14623 | 2.69887 | 0.006958 | 0.0281416 | TRUE |
| SLC27A6 | 14013 | 2.697859 | 0.006979 | 0.0282053 | TRUE |
| TTPA | 11710 | 2.697362 | 0.006989 | 0.02824 | TRUE |
| PSMB3 | 9960 | 2.697191 | 0.006993 | 0.02824 | TRUE |
| ZNF516 | 13697 | 2.696702 | 0.007003 | 0.0282669 | TRUE |
| EPCAM | 5100 | 2.696079 | 0.007016 | 0.0283125 | TRUE |
| ZBTB44 | 5939 | 2.694412 | 0.007051 | 0.0284179 | TRUE |
| IGSF6 | 12348 | 2.692407 | 0.007094 | 0.0285598 | TRUE |
| WFS1 | 11281 | 2.691712 | 0.007109 | 0.0286032 | TRUE |
| TMEM238 | 9999 | 2.691644 | 0.00711 | 0.0286032 | TRUE |
| ARPC1A | 9992 | 2.691419 | 0.007115 | 0.0286151 | TRUE |
| BLCAP | 3999 | 2.691191 | 0.00712 | 0.0286273 | TRUE |
| FAM131C | 10163 | 2.689728 | 0.007151 | 0.0287457 | TRUE |
| SCAF1 | 10491 | 2.689595 | 0.007154 | 0.0287498 | TRUE |
| RPL36AL | 2891 | 2.689175 | 0.007163 | 0.0287786 | TRUE |
| ZNF229 | 3511 | 2.688643 | 0.007174 | 0.0287967 | TRUE |
| BRINP3 | 262 | 2.688622 | 0.007175 | 0.0287967 | TRUE |
| SLC2A5 | 12437 | 2.688437 | 0.007179 | 0.0288052 | TRUE |
| SLC1A4 | 5984 | 2.688216 | 0.007183 | 0.02881 | TRUE |
| CHMP2B | 13433 | 2.687646 | 0.007196 | 0.0288513 | TRUE |
| TENM1 | 10618 | 2.687292 | 0.007203 | 0.028874 | TRUE |
| CERS4 | 2862 | 2.686928 | 0.007211 | 0.0288912 | TRUE |
| WRB | 4694 | 2.68656 | 0.007219 | 0.0289156 | TRUE |
| GULP1 | 5625 | 2.686067 | 0.00723 | 0.0289509 | TRUE |
| PCSK5 | 3159 | 2.685737 | 0.007237 | 0.0289721 | TRUE |
| MFSD10 | 1114 | 2.685343 | 0.007246 | 0.028994 | TRUE |
| KAT14 | 3304 | 2.685313 | 0.007246 | 0.028994 | TRUE |
| ZDHHC23 | 12051 | 2.684973 | 0.007254 | 0.0290161 | TRUE |
| RPL7 | 328 | 2.683883 | 0.007277 | 0.0291034 | TRUE |
| PLEKHH2 | 9966 | 2.683059 | 0.007295 | 0.0291625 | TRUE |
| TAC3 | 9207 | 2.682949 | 0.007298 | 0.0291625 | TRUE |
| ST6GAL1 | 9925 | 2.681948 | 0.007319 | 0.0292349 | TRUE |
| PDHB | 14747 | 2.680967 | 0.007341 | 0.0293133 | TRUE |
| FBXO17 | 7226 | 2.679754 | 0.007368 | 0.0293987 | TRUE |
| CASTOR1 | 9701 | 2.679464 | 0.007374 | 0.0294151 | TRUE |
| PLD3 | 14580 | 2.678952 | 0.007385 | 0.0294461 | TRUE |
| KLK5 | 3844 | 2.678474 | 0.007396 | 0.0294797 | TRUE |
| MPHOSPH6 | 8725 | 2.678138 | 0.007403 | 0.0295017 | TRUE |
| C19orf70 | 11929 | 2.677751 | 0.007412 | 0.029521 | TRUE |
| ADSL | 7026 | 2.677464 | 0.007418 | 0.0295311 | TRUE |
| RGS22 | 1825 | 2.67677 | 0.007434 | 0.0295772 | TRUE |
| METTL26 | 8104 | 2.676661 | 0.007436 | 0.0295793 | TRUE |
| STK32C | 13270 | 2.675759 | 0.007456 | 0.0296364 | TRUE |
| HLA-DRB1 | 3953 | 2.674636 | 0.007481 | 0.0297132 | TRUE |
| FGFBP3 | 9099 | 2.674398 | 0.007486 | 0.0297267 | TRUE |
| ATOH7 | 1455 | 2.671758 | 0.007546 | 0.0299388 | TRUE |
| CNRIP1 | 685 | 2.671253 | 0.007557 | 0.0299738 | TRUE |
| PTER | 387 | 2.670895 | 0.007565 | 0.0299875 | TRUE |
| INHBB | 6028 | 2.670642 | 0.007571 | 0.0300004 | TRUE |
| TNS3 | 13866 | 2.669569 | 0.007595 | 0.0300888 | TRUE |
| RPS2 | 2547 | 2.66764 | 0.007639 | 0.0302545 | TRUE |
| DNAJA1 | 9830 | 2.665885 | 0.007679 | 0.0303761 | TRUE |
| RPL13AP3 | 1036 | 2.665866 | 0.007679 | 0.0303761 | TRUE |
| UFC1 | 13271 | 2.66372 | 0.007728 | 0.0305396 | TRUE |
| SCGB3A2 | 10772 | 2.663353 | 0.007737 | 0.0305575 | TRUE |
| TXLNB | 5680 | 2.662536 | 0.007755 | 0.0306189 | TRUE |
| WDR1 | 867 | 2.66106 | 0.00779 | 0.03073 | TRUE |
| GALNT17 | 9324 | 2.661033 | 0.00779 | 0.03073 | TRUE |
| SLC5A12 | 11494 | 2.66073 | 0.007797 | 0.0307462 | TRUE |
| GSPT2 | 1034 | 2.660686 | 0.007798 | 0.0307462 | TRUE |
| SHISA5 | 13543 | 2.660132 | 0.007811 | 0.0307891 | TRUE |
| DGCR6L | 3618 | 2.659136 | 0.007834 | 0.0308505 | TRUE |
| CASC10 | 4755 | 2.6588 | 0.007842 | 0.0308574 | TRUE |
| SLC17A8 | 14440 | 2.65773 | 0.007867 | 0.0309391 | TRUE |
| DAP | 11415 | 2.656619 | 0.007893 | 0.0310257 | TRUE |
| ICAM3 | 14796 | 2.65653 | 0.007895 | 0.0310261 | TRUE |
| RHBDF2 | 6273 | 2.655981 | 0.007908 | 0.031061 | TRUE |
| SSC4D | 7651 | 2.653912 | 0.007956 | 0.0311816 | TRUE |
| TMEM181 | 9509 | 2.653749 | 0.00796 | 0.0311888 | TRUE |
| DCUN1D5 | 11388 | 2.651692 | 0.008009 | 0.0313595 | TRUE |
| DCAF4 | 7917 | 2.651653 | 0.00801 | 0.0313595 | TRUE |
| TP53I3 | 8172 | 2.650609 | 0.008035 | 0.0314408 | TRUE |
| HSPB3 | 1733 | 2.65044 | 0.008039 | 0.0314487 | TRUE |
| ALDH7A1 | 1245 | 2.649055 | 0.008072 | 0.0315621 | TRUE |
| TMC8 | 7751 | 2.648755 | 0.008079 | 0.0315822 | TRUE |
| FAM241A | 5497 | 2.648218 | 0.008092 | 0.0316245 | TRUE |
| PSMB10 | 3160 | 2.647153 | 0.008117 | 0.0317005 | TRUE |
| UPP2 | 3222 | 2.645283 | 0.008162 | 0.0318603 | TRUE |
| C8orf34 | 3170 | 2.64482 | 0.008173 | 0.031896 | TRUE |
| ATP6V0D1 | 9100 | 2.644333 | 0.008185 | 0.031934 | TRUE |
| SF3B5 | 10401 | 2.644207 | 0.008188 | 0.0319379 | TRUE |
| STUB1 | 4492 | 2.643134 | 0.008214 | 0.0320233 | TRUE |
| EIF4A3 | 12709 | 2.642597 | 0.008227 | 0.0320661 | TRUE |
| RSPO3 | 11667 | 2.642409 | 0.008232 | 0.0320759 | TRUE |
| CNN3 | 11044 | 2.642276 | 0.008235 | 0.0320805 | TRUE |
| HEY1 | 11301 | 2.641932 | 0.008243 | 0.0321051 | TRUE |
| GRIA1 | 11168 | 2.641736 | 0.008248 | 0.0321157 | TRUE |
| HLA-G | 7423 | 2.639587 | 0.008301 | 0.0322906 | TRUE |
| CDH8 | 2632 | 2.639559 | 0.008301 | 0.0322906 | TRUE |
| HIST1H2BI | 14646 | 2.637502 | 0.008352 | 0.0324628 | TRUE |
| SCRG1 | 10485 | 2.636726 | 0.008371 | 0.0325291 | TRUE |
| NDUFA13 | 12577 | 2.636245 | 0.008383 | 0.0325671 | TRUE |
| LOC644794 | 6930 | 2.635605 | 0.008399 | 0.0326043 | TRUE |
| EMX2OS | 13520 | 2.635167 | 0.00841 | 0.0326302 | TRUE |
| APOPT1 | 2050 | 2.634643 | 0.008423 | 0.0326725 | TRUE |
| CSGALNACT1 | 10006 | 2.63408 | 0.008437 | 0.0327186 | TRUE |
| AK1 | 13171 | 2.630845 | 0.008517 | 0.0329814 | TRUE |
| C19orf57 | 2847 | 2.630773 | 0.008519 | 0.0329814 | TRUE |
| HDDC2 | 10420 | 2.630128 | 0.008535 | 0.0330171 | TRUE |
| ARHGEF26-AS1 | 13486 | 2.629411 | 0.008553 | 0.0330647 | TRUE |
| UBA1 | 2726 | 2.627902 | 0.008591 | 0.0331789 | TRUE |
| NRP2 | 3295 | 2.627357 | 0.008605 | 0.0332144 | TRUE |
| LOC654780 | 8809 | 2.627286 | 0.008607 | 0.0332144 | TRUE |
| TRIM62 | 15067 | 2.626357 | 0.00863 | 0.0332794 | TRUE |
| SLC4A4 | 532 | 2.626321 | 0.008631 | 0.0332794 | TRUE |
| LOC100506125 | 5695 | 2.626285 | 0.008632 | 0.0332794 | TRUE |
| NPM3 | 3409 | 2.625615 | 0.008649 | 0.0333368 | TRUE |
| KRTAP17-1 | 6248 | 2.625489 | 0.008652 | 0.0333369 | TRUE |
| GALNT11 | 7996 | 2.625446 | 0.008654 | 0.0333369 | TRUE |
| ILF3-AS1 | 8836 | 2.6252 | 0.00866 | 0.0333527 | TRUE |
| ALG8 | 15327 | 2.623711 | 0.008698 | 0.0334737 | TRUE |
| ADAMTS8 | 9203 | 2.622842 | 0.00872 | 0.0335138 | TRUE |
| RHOC | 12946 | 2.622164 | 0.008737 | 0.0335604 | TRUE |
| PRICKLE2 | 8321 | 2.620125 | 0.00879 | 0.0337286 | TRUE |
| JUP | 5114 | 2.618764 | 0.008825 | 0.0338468 | TRUE |
| RGS18 | 300 | 2.617428 | 0.00886 | 0.033938 | TRUE |
| DAPL1 | 3743 | 2.616812 | 0.008876 | 0.0339826 | TRUE |
| RPL10L | 9257 | 2.616629 | 0.00888 | 0.0339908 | TRUE |
| DNAJB2 | 11092 | 2.616563 | 0.008882 | 0.0339908 | TRUE |
| HMGCS1 | 7181 | 2.615539 | 0.008909 | 0.0340845 | TRUE |
| FERMT2 | 8404 | 2.614665 | 0.008932 | 0.0341468 | TRUE |
| UBXN6 | 13485 | 2.614327 | 0.00894 | 0.0341722 | TRUE |
| IDS | 14889 | 2.61381 | 0.008954 | 0.0342156 | TRUE |
| C1orf21 | 2936 | 2.612063 | 0.009 | 0.0343741 | TRUE |
| COL5A3 | 11492 | 2.61193 | 0.009003 | 0.0343791 | TRUE |
| NOV | 11157 | 2.610826 | 0.009032 | 0.0344609 | TRUE |
| TNFAIP2 | 12061 | 2.610732 | 0.009035 | 0.0344609 | TRUE |
| ERI3 | 11490 | 2.6107 | 0.009036 | 0.0344609 | TRUE |
| CCDC191 | 14701 | 2.609594 | 0.009065 | 0.0345472 | TRUE |
| RPS21 | 10727 | 2.608679 | 0.009089 | 0.0346229 | TRUE |
| MPEG1 | 3886 | 2.607509 | 0.00912 | 0.034716 | TRUE |
| YWHAG | 6258 | 2.607041 | 0.009133 | 0.0347381 | TRUE |
| RNF7 | 14794 | 2.60689 | 0.009137 | 0.034745 | TRUE |
| RPL23 | 9982 | 2.604448 | 0.009202 | 0.0349511 | TRUE |
| ITGB1BP1 | 11192 | 2.604185 | 0.009209 | 0.0349678 | TRUE |
| PRRX1 | 13480 | 2.603245 | 0.009235 | 0.0350144 | TRUE |
| CORO1C | 11218 | 2.602392 | 0.009258 | 0.0350931 | TRUE |
| DKFZP586I1420 | 15568 | 2.601591 | 0.009279 | 0.0351666 | TRUE |
| LARS2 | 10749 | 2.600882 | 0.009298 | 0.0352223 | TRUE |
| NENF | 11299 | 2.600289 | 0.009315 | 0.0352747 | TRUE |
| TMEM9 | 11947 | 2.599852 | 0.009326 | 0.0353043 | TRUE |
| UBE3A | 5609 | 2.599361 | 0.00934 | 0.0353445 | TRUE |
| SELPLG | 14806 | 2.598664 | 0.009359 | 0.0353992 | TRUE |
| COMMD7 | 12757 | 2.598316 | 0.009368 | 0.03541 | TRUE |
| NPTX2 | 1297 | 2.59776 | 0.009383 | 0.0354582 | TRUE |
| RAD21 | 11065 | 2.597518 | 0.00939 | 0.0354661 | TRUE |
| RARS | 10546 | 2.59685 | 0.009408 | 0.0355264 | TRUE |
| LGALS9C | 2442 | 2.596769 | 0.009411 | 0.0355264 | TRUE |
| TMEM59L | 14207 | 2.594833 | 0.009464 | 0.0357098 | TRUE |
| LINC01993 | 7909 | 2.593325 | 0.009505 | 0.0358322 | TRUE |
| VPS35 | 6271 | 2.59091 | 0.009572 | 0.0360325 | TRUE |
| RGS4 | 163 | 2.590622 | 0.00958 | 0.036054 | TRUE |
| DPM2 | 2931 | 2.590151 | 0.009593 | 0.0360947 | TRUE |
| SELENOK | 9180 | 2.58994 | 0.009599 | 0.036105 | TRUE |
| LIG1 | 5345 | 2.589677 | 0.009607 | 0.0361183 | TRUE |
| TOMM6 | 11568 | 2.587972 | 0.009654 | 0.0362801 | TRUE |
| B4GALT3 | 11049 | 2.585847 | 0.009714 | 0.0364783 | TRUE |
| COL10A1 | 9435 | 2.584546 | 0.009751 | 0.0365965 | TRUE |
| TRIM71 | 13507 | 2.584466 | 0.009753 | 0.0365965 | TRUE |
| HSDL2 | 5204 | 2.584195 | 0.009761 | 0.0366038 | TRUE |
| SNN | 2856 | 2.583994 | 0.009766 | 0.0366049 | TRUE |
| PAFAH1B3 | 5569 | 2.58399 | 0.009766 | 0.0366049 | TRUE |
| HIST1H2AD | 3857 | 2.583482 | 0.009781 | 0.036642 | TRUE |
| RILPL1 | 10317 | 2.583399 | 0.009783 | 0.036642 | TRUE |
| MATN2 | 8604 | 2.582406 | 0.009811 | 0.0367293 | TRUE |
| LINGO3 | 8109 | 2.58097 | 0.009852 | 0.0368648 | TRUE |
| CLPP | 13003 | 2.578968 | 0.00991 | 0.0370353 | TRUE |
| MID1 | 6647 | 2.576821 | 0.009971 | 0.0372186 | TRUE |
| COG5 | 7370 | 2.576762 | 0.009973 | 0.0372186 | TRUE |
| SH2D5 | 12718 | 2.576479 | 0.009981 | 0.0372402 | TRUE |
| NUCB2 | 11712 | 2.574599 | 0.010036 | 0.0373986 | TRUE |
| NDUFA3 | 14675 | 2.574419 | 0.010041 | 0.0374091 | TRUE |
| CRB2 | 12009 | 2.574332 | 0.010043 | 0.0374096 | TRUE |
| 3-Mar | 7900 | 2.572628 | 0.010093 | 0.0375674 | TRUE |
| ZNF883 | 15502 | 2.572087 | 0.010109 | 0.0376173 | TRUE |
| B3GNT5 | 5789 | 2.571429 | 0.010128 | 0.0376739 | TRUE |
| RABAC1 | 14742 | 2.571401 | 0.010129 | 0.0376739 | TRUE |
| TTR | 4749 | 2.570405 | 0.010158 | 0.0377658 | TRUE |
| TBPL1 | 7062 | 2.570388 | 0.010158 | 0.0377658 | TRUE |
| ILF3 | 9800 | 2.57031 | 0.010161 | 0.0377658 | TRUE |
| HEPH | 5944 | 2.569164 | 0.010194 | 0.037882 | TRUE |
| NTSR2 | 5536 | 2.568633 | 0.01021 | 0.037922 | TRUE |
| ORMDL2 | 627 | 2.567821 | 0.010234 | 0.0379839 | TRUE |
| DIO2 | 7793 | 2.567538 | 0.010242 | 0.0380059 | TRUE |
| RSPH14 | 9304 | 2.566776 | 0.010265 | 0.0380624 | TRUE |
| MLNR | 13830 | 2.565888 | 0.010291 | 0.0381328 | TRUE |
| ACTR3C | 11744 | 2.565769 | 0.010295 | 0.0381369 | TRUE |
| PAMR1 | 14313 | 2.565344 | 0.010307 | 0.0381599 | TRUE |
| SCAMP1-AS1 | 1991 | 2.564827 | 0.010323 | 0.0382044 | TRUE |
| GPR107 | 6757 | 2.564113 | 0.010344 | 0.0382649 | TRUE |
| MEX3A | 13474 | 2.562757 | 0.010384 | 0.0383693 | TRUE |
| RBX1 | 7419 | 2.559816 | 0.010473 | 0.0386589 | TRUE |
| CH25H | 675 | 2.558635 | 0.010508 | 0.0387722 | TRUE |
| LINC00467 | 14069 | 2.558264 | 0.01052 | 0.0387953 | TRUE |
| NCAM2 | 12987 | 2.557008 | 0.010558 | 0.0389081 | TRUE |
| ALDH6A1 | 13885 | 2.556913 | 0.010561 | 0.0389096 | TRUE |
| EMC6 | 8390 | 2.556512 | 0.010573 | 0.0389361 | TRUE |
| COX7C | 14320 | 2.555364 | 0.010608 | 0.0390464 | TRUE |
| RAB3GAP1 | 11794 | 2.555088 | 0.010616 | 0.0390681 | TRUE |
| BCAS2 | 2743 | 2.554932 | 0.010621 | 0.0390764 | TRUE |
| MXRA8 | 14559 | 2.554674 | 0.010629 | 0.0390962 | TRUE |
| CNTN6 | 3875 | 2.553744 | 0.010657 | 0.0391745 | TRUE |
| B3GAT2 | 6937 | 2.552112 | 0.010707 | 0.0393479 | TRUE |
| PDXP | 4216 | 2.550408 | 0.01076 | 0.0395221 | TRUE |
| SOCS7 | 4844 | 2.549809 | 0.010778 | 0.0395808 | TRUE |
| RNF2 | 11008 | 2.54911 | 0.0108 | 0.0396462 | TRUE |
| FABP3 | 254 | 2.54907 | 0.010801 | 0.0396462 | TRUE |
| COMMD6 | 9936 | 2.54818 | 0.010829 | 0.0397102 | TRUE |
| TRAPPC2 | 5591 | 2.545813 | 0.010902 | 0.0399523 | TRUE |
| FGFR3 | 15282 | 2.544492 | 0.010944 | 0.0400756 | TRUE |
| CDC42P3 | 10720 | 2.542198 | 0.011016 | 0.0403018 | TRUE |
| ROM1 | 7071 | 2.541922 | 0.011024 | 0.0403242 | TRUE |
| SMYD3 | 6754 | 2.541199 | 0.011047 | 0.0403888 | TRUE |
| ADM5 | 9995 | 2.541027 | 0.011053 | 0.0403992 | TRUE |
| HRH1 | 3365 | 2.540939 | 0.011056 | 0.0403999 | TRUE |
| CD68 | 14233 | 2.540835 | 0.011059 | 0.0404025 | TRUE |
| IGSF1 | 11316 | 2.539751 | 0.011093 | 0.0405185 | TRUE |
| FSCN2 | 4265 | 2.53818 | 0.011143 | 0.0406543 | TRUE |
| SPON2 | 1941 | 2.538173 | 0.011143 | 0.0406543 | TRUE |
| RPS5 | 14766 | 2.537227 | 0.011173 | 0.0407452 | TRUE |
| RAB29 | 8695 | 2.537055 | 0.011179 | 0.0407462 | TRUE |
| DMAC1 | 7368 | 2.536056 | 0.011211 | 0.0408436 | TRUE |
| C12orf76 | 13964 | 2.535925 | 0.011215 | 0.0408493 | TRUE |
| DAB1 | 4344 | 2.535322 | 0.011234 | 0.0408911 | TRUE |
| TMEM42 | 11464 | 2.535163 | 0.01124 | 0.0409002 | TRUE |
| 3-Sep | 9710 | 2.534705 | 0.011254 | 0.0409441 | TRUE |
| SNAPC5 | 1276 | 2.53424 | 0.011269 | 0.0409794 | TRUE |
| ERH | 533 | 2.533076 | 0.011307 | 0.0410995 | TRUE |
| LY9 | 1923 | 2.533052 | 0.011307 | 0.0410995 | TRUE |
| FOXJ3 | 13827 | 2.532856 | 0.011314 | 0.0411129 | TRUE |
| FAM19A1 | 2014 | 2.531934 | 0.011344 | 0.0412116 | TRUE |
| LINC00674 | 2181 | 2.531334 | 0.011363 | 0.041263 | TRUE |
| YJEFN3 | 8444 | 2.531207 | 0.011367 | 0.0412683 | TRUE |
| MSN | 7451 | 2.530585 | 0.011387 | 0.0413224 | TRUE |
| TLL2 | 1124 | 2.52974 | 0.011415 | 0.0413932 | TRUE |
| RRAGB | 11572 | 2.528557 | 0.011453 | 0.0415137 | TRUE |
| UGT2B10 | 11578 | 2.528089 | 0.011469 | 0.0415498 | TRUE |
| CLPB | 4543 | 2.527795 | 0.011478 | 0.041575 | TRUE |
| SLC9A6 | 7253 | 2.527672 | 0.011482 | 0.0415799 | TRUE |
| PPIB | 2612 | 2.525367 | 0.011558 | 0.0418246 | TRUE |
| KCTD17 | 14490 | 2.525192 | 0.011564 | 0.0418358 | TRUE |
| 2-Sep | 14066 | 2.524831 | 0.011575 | 0.0418625 | TRUE |
| PTGER4P2-CDK2AP2P2 | 13071 | 2.524805 | 0.011576 | 0.0418625 | TRUE |
| MPP2 | 13239 | 2.524264 | 0.011594 | 0.0418979 | TRUE |
| ZMYND10 | 6486 | 2.523913 | 0.011606 | 0.0419204 | TRUE |
| SIRT3 | 3132 | 2.522857 | 0.011641 | 0.042027 | TRUE |
| NMNAT3 | 7293 | 2.522592 | 0.011649 | 0.042049 | TRUE |
| ARHGAP1 | 13497 | 2.522281 | 0.01166 | 0.0420668 | TRUE |
| SIGLEC14 | 1236 | 2.5217 | 0.011679 | 0.0421266 | TRUE |
| IL17RB | 11735 | 2.51927 | 0.01176 | 0.0423891 | TRUE |
| DGKB | 9057 | 2.51865 | 0.011781 | 0.0424443 | TRUE |
| CNTNAP3 | 11204 | 2.517958 | 0.011804 | 0.0425179 | TRUE |
| PHACTR3 | 2526 | 2.517702 | 0.011812 | 0.042521 | TRUE |
| GDF10 | 2338 | 2.517689 | 0.011813 | 0.042521 | TRUE |
| LIX1L | 11805 | 2.51743 | 0.011821 | 0.0425366 | TRUE |
| RREB1 | 14331 | 2.516519 | 0.011852 | 0.0426036 | TRUE |
| RCN2 | 8330 | 2.514647 | 0.011915 | 0.0427972 | TRUE |
| NR0B1 | 3061 | 2.51457 | 0.011918 | 0.0427972 | TRUE |
| DARS | 6581 | 2.513727 | 0.011946 | 0.0428832 | TRUE |
| MT1A | 6341 | 2.513169 | 0.011965 | 0.0429215 | TRUE |
| ADH5 | 10774 | 2.512321 | 0.011994 | 0.0430051 | TRUE |
| CDC34 | 7729 | 2.511446 | 0.012024 | 0.0431019 | TRUE |
| LAT2 | 6874 | 2.511147 | 0.012034 | 0.0431286 | TRUE |
| FGF2 | 11350 | 2.510993 | 0.012039 | 0.0431375 | TRUE |
| COMTD1 | 2347 | 2.509846 | 0.012078 | 0.0432481 | TRUE |
| SNAP47 | 4016 | 2.508967 | 0.012108 | 0.043346 | TRUE |
| FAM167A | 11749 | 2.508298 | 0.012131 | 0.0434155 | TRUE |
| LAGE3 | 8135 | 2.508239 | 0.012133 | 0.0434155 | TRUE |
| DHCR24 | 1958 | 2.508104 | 0.012138 | 0.0434222 | TRUE |
| PGRMC1 | 5451 | 2.505667 | 0.012222 | 0.0436926 | TRUE |
| GPSM1 | 6783 | 2.505577 | 0.012225 | 0.0436937 | TRUE |
| SPA17 | 3015 | 2.503549 | 0.012295 | 0.0438947 | TRUE |
| CETN3 | 4802 | 2.502995 | 0.012315 | 0.0439334 | TRUE |
| TMEM47 | 10479 | 2.502282 | 0.01234 | 0.0440119 | TRUE |
| CENPF | 11145 | 2.502142 | 0.012344 | 0.044017 | TRUE |
| CASP5 | 12396 | 2.501786 | 0.012357 | 0.0440335 | TRUE |
| DCTPP1 | 5366 | 2.500032 | 0.012418 | 0.0442319 | TRUE |
| SELENBP1 | 13051 | 2.497924 | 0.012492 | 0.0444755 | TRUE |
| PLCH2 | 4166 | 2.497277 | 0.012515 | 0.0445466 | TRUE |
| FAM207A | 11353 | 2.496569 | 0.01254 | 0.0446255 | TRUE |
| DPYD | 12970 | 2.496328 | 0.012549 | 0.0446457 | TRUE |
| MRAS | 5172 | 2.496037 | 0.012559 | 0.0446721 | TRUE |
| NPNT | 7811 | 2.495584 | 0.012575 | 0.0447069 | TRUE |
| IGFBP5 | 12393 | 2.49547 | 0.012579 | 0.0447069 | TRUE |
| PKIB | 6552 | 2.495438 | 0.01258 | 0.0447069 | TRUE |
| CHMP6 | 10338 | 2.494912 | 0.012599 | 0.0447631 | TRUE |
| DERL3 | 745 | 2.494805 | 0.012603 | 0.0447659 | TRUE |
| NCAPG2 | 13124 | 2.494723 | 0.012606 | 0.0447659 | TRUE |
| MSRB2 | 8401 | 2.493849 | 0.012637 | 0.0448283 | TRUE |
| MFSD12 | 1688 | 2.493163 | 0.012661 | 0.0449026 | TRUE |
| CACUL1 | 13343 | 2.492849 | 0.012672 | 0.0449321 | TRUE |
| ACAD10 | 4865 | 2.492546 | 0.012683 | 0.0449603 | TRUE |
| BABAM2 | 9806 | 2.491402 | 0.012724 | 0.0450849 | TRUE |
| NOP16 | 14316 | 2.490992 | 0.012739 | 0.0451074 | TRUE |
| NORAD | 9274 | 2.490219 | 0.012766 | 0.0451942 | TRUE |
| RPL6 | 3358 | 2.488662 | 0.012822 | 0.0453413 | TRUE |
| CD4 | 13977 | 2.487592 | 0.012861 | 0.0454574 | TRUE |
| ZFP36 | 4357 | 2.487449 | 0.012866 | 0.0454654 | TRUE |
| MOSPD2 | 14384 | 2.486833 | 0.012889 | 0.0455339 | TRUE |
| LACTB | 1273 | 2.486515 | 0.0129 | 0.0455643 | TRUE |
| PLP2 | 14073 | 2.486307 | 0.012908 | 0.0455703 | TRUE |
| BAD | 10054 | 2.486109 | 0.012915 | 0.0455854 | TRUE |
| DERL1 | 13953 | 2.484926 | 0.012958 | 0.0457044 | TRUE |
| PKIG | 1555 | 2.48486 | 0.01296 | 0.0457044 | TRUE |
| CFAP97 | 11535 | 2.484424 | 0.012976 | 0.0457397 | TRUE |
| VBP1 | 4324 | 2.483072 | 0.013025 | 0.0459034 | TRUE |
| APOE | 7684 | 2.482747 | 0.013037 | 0.0459349 | TRUE |
| MED19 | 2336 | 2.482609 | 0.013042 | 0.0459359 | TRUE |
| MORN2 | 10795 | 2.482579 | 0.013044 | 0.0459359 | TRUE |
| POLR1D | 3649 | 2.481846 | 0.01307 | 0.0460137 | TRUE |
| C1orf122 | 849 | 2.481588 | 0.01308 | 0.0460327 | TRUE |
| RPL39 | 8914 | 2.481299 | 0.01309 | 0.0460493 | TRUE |
| HEXA | 1874 | 2.481072 | 0.013099 | 0.0460683 | TRUE |
| CFAP20 | 3558 | 2.48055 | 0.013118 | 0.0461254 | TRUE |
| RRAS2 | 14564 | 2.480329 | 0.013126 | 0.0461335 | TRUE |
| RPS6 | 12261 | 2.480247 | 0.013129 | 0.0461335 | TRUE |
| LOC285097 | 1520 | 2.47991 | 0.013142 | 0.0461641 | TRUE |
| CDKL5 | 14194 | 2.47985 | 0.013144 | 0.0461641 | TRUE |
| L3MBTL4 | 8865 | 2.479589 | 0.013153 | 0.0461772 | TRUE |
| MAPK11 | 9193 | 2.47924 | 0.013166 | 0.0461913 | TRUE |
| DBN1 | 277 | 2.478686 | 0.013187 | 0.0462328 | TRUE |
| PTP4A1 | 11870 | 2.478283 | 0.013202 | 0.0462738 | TRUE |
| CISD3 | 11790 | 2.477469 | 0.013232 | 0.0463691 | TRUE |
| NDUFAF8 | 13807 | 2.477177 | 0.013243 | 0.0463967 | TRUE |
| MCM3 | 13384 | 2.477086 | 0.013246 | 0.0463981 | TRUE |
| CRNDE | 8912 | 2.476879 | 0.013254 | 0.0464146 | TRUE |
| ACBD6 | 8376 | 2.476587 | 0.013265 | 0.0464184 | TRUE |
| DDA1 | 4877 | 2.476581 | 0.013265 | 0.0464184 | TRUE |
| LAPTM5 | 10179 | 2.475959 | 0.013288 | 0.0464823 | TRUE |
| SYTL3 | 10492 | 2.475481 | 0.013306 | 0.0465342 | TRUE |
| ATL1 | 2548 | 2.474917 | 0.013327 | 0.0465973 | TRUE |
| CCT5 | 7936 | 2.472715 | 0.013409 | 0.0468539 | TRUE |
| MSRA | 12235 | 2.472197 | 0.013429 | 0.0469009 | TRUE |
| PHF24 | 6938 | 2.470006 | 0.013511 | 0.0471365 | TRUE |
| CTSO | 13949 | 2.469897 | 0.013515 | 0.0471404 | TRUE |
| GRB10 | 8750 | 2.469663 | 0.013524 | 0.0471585 | TRUE |
| SLC16A5 | 2374 | 2.4696 | 0.013526 | 0.0471585 | TRUE |
| AMZ2P1 | 12494 | 2.468836 | 0.013555 | 0.0472382 | TRUE |
| NAGK | 13967 | 2.46847 | 0.013569 | 0.0472678 | TRUE |
| FITM1 | 8350 | 2.468393 | 0.013572 | 0.0472678 | TRUE |
| ARHGEF26 | 7616 | 2.468347 | 0.013574 | 0.0472678 | TRUE |
| BYSL | 3185 | 2.465635 | 0.013677 | 0.047567 | TRUE |
| DDR1 | 5624 | 2.464612 | 0.013716 | 0.0476924 | TRUE |
| C6orf48 | 11926 | 2.464354 | 0.013726 | 0.0477161 | TRUE |
| CCDC24 | 3848 | 2.463129 | 0.013773 | 0.0478582 | TRUE |
| EEF1B2 | 13113 | 2.462734 | 0.013788 | 0.0478911 | TRUE |
| ZNF648 | 12720 | 2.462723 | 0.013789 | 0.0478911 | TRUE |
| LOC101928370 | 3494 | 2.462266 | 0.013806 | 0.0479202 | TRUE |
| NT5DC3 | 11778 | 2.461949 | 0.013818 | 0.0479287 | TRUE |
| ZC3H15 | 11600 | 2.461884 | 0.013821 | 0.0479287 | TRUE |
| FMO2 | 691 | 2.461244 | 0.013846 | 0.047993 | TRUE |
| FTMT | 7588 | 2.460277 | 0.013883 | 0.0481012 | TRUE |
| SNX3 | 1954 | 2.459866 | 0.013899 | 0.0481457 | TRUE |
| IDI1 | 4965 | 2.456194 | 0.014042 | 0.0485866 | TRUE |
| VSIR | 2954 | 2.45596 | 0.014051 | 0.0486075 | TRUE |
| FCGR2A | 9103 | 2.454899 | 0.014092 | 0.0487297 | TRUE |
| GGNBP1 | 2976 | 2.45472 | 0.014099 | 0.0487431 | TRUE |
| SLC40A1 | 9359 | 2.454438 | 0.01411 | 0.0487706 | TRUE |
| HAVCR2 | 11575 | 2.453166 | 0.01416 | 0.048911 | TRUE |
| MRPS11 | 1719 | 2.45175 | 0.014216 | 0.0490822 | TRUE |
| FAM174B | 5772 | 2.451537 | 0.014225 | 0.0490981 | TRUE |
| BCL7C | 12164 | 2.451216 | 0.014237 | 0.0491225 | TRUE |
| DPYSL2 | 2101 | 2.450438 | 0.014268 | 0.0492071 | TRUE |
| HLA-C | 89 | 2.449534 | 0.014304 | 0.049309 | TRUE |
| FAM53C | 8096 | 2.449397 | 0.01431 | 0.0493169 | TRUE |
| MAGEH1 | 2471 | 2.449083 | 0.014322 | 0.0493383 | TRUE |
| LHFPL6 | 334 | 2.448911 | 0.014329 | 0.0493508 | TRUE |
| KLRG1 | 10388 | 2.447862 | 0.014371 | 0.0494838 | TRUE |
| MAPK7 | 11308 | 2.447635 | 0.01438 | 0.0495041 | TRUE |
| PAXBP1-AS1 | 8199 | 2.446999 | 0.014405 | 0.0495534 | TRUE |
| RASGRP4 | 3249 | 2.446974 | 0.014406 | 0.0495534 | TRUE |
| SLC52A2 | 5399 | 2.446826 | 0.014412 | 0.0495534 | TRUE |
| GYG2 | 7644 | 2.446634 | 0.01442 | 0.0495545 | TRUE |
| OR2W5 | 1316 | 2.446206 | 0.014437 | 0.0496025 | TRUE |
| NAXE | 14441 | 2.445822 | 0.014452 | 0.0496226 | TRUE |
| LTC4S | 5963 | 2.445327 | 0.014472 | 0.0496467 | TRUE |
| VPS37B | 1934 | 2.445307 | 0.014473 | 0.0496467 | TRUE |
| SLC6A19 | 3724 | 2.445251 | 0.014475 | 0.0496467 | TRUE |
| APRT | 8527 | 2.445025 | 0.014484 | 0.0496561 | TRUE |
| CTSH | 12047 | 2.444334 | 0.014512 | 0.0497404 | TRUE |
| PDE1A | 2012 | 2.443187 | 0.014558 | 0.049866 | TRUE |
| KDELR1 | 10243 | 2.442808 | 0.014573 | 0.0499074 | TRUE |
| QSOX1 | 10663 | 2.442282 | 0.014595 | 0.0499474 | TRUE |
| PLCG2 | 14539 | 2.441725 | 0.014617 | 0.0500026 | FALSE |
| CSPG4 | 7214 | 2.441251 | 0.014636 | 0.0500573 | FALSE |
| ACY3 | 7513 | 2.43936 | 0.014713 | 0.0502661 | FALSE |
| CCDC71L | 8387 | 2.43903 | 0.014727 | 0.0503 | FALSE |
| PGM2L1 | 14805 | 2.436003 | 0.014851 | 0.0507008 | FALSE |
| ANKRD19P | 12756 | 2.435615 | 0.014866 | 0.0507441 | FALSE |
| FAM46A | 944 | 2.434958 | 0.014894 | 0.0508141 | FALSE |
| GLO1 | 5927 | 2.433658 | 0.014947 | 0.0509747 | FALSE |
| DUSP23 | 10610 | 2.433026 | 0.014973 | 0.0510421 | FALSE |
| PPP1R26 | 1687 | 2.432927 | 0.014977 | 0.0510444 | FALSE |
| LCAT | 9582 | 2.432826 | 0.014981 | 0.0510475 | FALSE |
| MILR1 | 3147 | 2.431994 | 0.015016 | 0.0511203 | FALSE |
| AK4 | 3556 | 2.43106 | 0.015055 | 0.05123 | FALSE |
| FUT7 | 3188 | 2.429467 | 0.015121 | 0.0514221 | FALSE |
| EMC3 | 14171 | 2.428164 | 0.015175 | 0.0515848 | FALSE |
| CCDC102A | 10230 | 2.427854 | 0.015188 | 0.0516176 | FALSE |
| PTCRA | 1048 | 2.427643 | 0.015197 | 0.0516364 | FALSE |
| SYTL4 | 2858 | 2.427244 | 0.015214 | 0.0516708 | FALSE |
| TERF1 | 305 | 2.42678 | 0.015233 | 0.0517145 | FALSE |
| GAREM2 | 13481 | 2.425356 | 0.015293 | 0.0518953 | FALSE |
| TALDO1 | 655 | 2.425043 | 0.015307 | 0.0519287 | FALSE |
| TVP23A | 2126 | 2.424418 | 0.015333 | 0.0519988 | FALSE |
| TMBIM6 | 13757 | 2.424258 | 0.01534 | 0.0520073 | FALSE |
| CD38 | 3059 | 2.424144 | 0.015345 | 0.0520123 | FALSE |
| CD99 | 406 | 2.422804 | 0.015401 | 0.0521738 | FALSE |
| MPPED1 | 4800 | 2.422754 | 0.015403 | 0.0521738 | FALSE |
| HSPA1A | 11498 | 2.422703 | 0.015406 | 0.0521738 | FALSE |
| BRI3 | 12534 | 2.422187 | 0.015427 | 0.0522367 | FALSE |
| DVL1 | 10730 | 2.420381 | 0.015504 | 0.0524174 | FALSE |
| LINC01736 | 1576 | 2.420007 | 0.01552 | 0.05246 | FALSE |
| PRRC2B | 14497 | 2.41921 | 0.015554 | 0.0525637 | FALSE |
| GIMAP2 | 14292 | 2.4188 | 0.015572 | 0.0526116 | FALSE |
| ARFGAP3 | 2696 | 2.418229 | 0.015596 | 0.0526715 | FALSE |
| PLCE1 | 9828 | 2.417953 | 0.015608 | 0.0527001 | FALSE |
| DENND2A | 12409 | 2.417833 | 0.015613 | 0.0527061 | FALSE |
| FAM86C1 | 4581 | 2.417513 | 0.015627 | 0.052741 | FALSE |
| PPCS | 11737 | 2.417028 | 0.015648 | 0.0528 | FALSE |
| TMEM173 | 13971 | 2.416233 | 0.015682 | 0.052904 | FALSE |
| ANGPTL4 | 11817 | 2.415888 | 0.015697 | 0.0529309 | FALSE |
| KLK12 | 14276 | 2.415832 | 0.015699 | 0.0529309 | FALSE |
| CLEC9A | 5009 | 2.415812 | 0.0157 | 0.0529309 | FALSE |
| DCHS1 | 3987 | 2.415431 | 0.015717 | 0.0529635 | FALSE |
| ACAT2 | 9786 | 2.414994 | 0.015735 | 0.0530156 | FALSE |
| RPL11 | 6681 | 2.414767 | 0.015745 | 0.0530373 | FALSE |
| GABRB3 | 2418 | 2.414297 | 0.015766 | 0.0530943 | FALSE |
| RNF135 | 4790 | 2.413847 | 0.015785 | 0.0531485 | FALSE |
| CCNH | 14653 | 2.413716 | 0.015791 | 0.0531561 | FALSE |
| DNAJB5 | 9587 | 2.413425 | 0.015803 | 0.0531757 | FALSE |
| B3GNT8 | 5683 | 2.412448 | 0.015846 | 0.053284 | FALSE |
| SNHG15 | 786 | 2.411749 | 0.015876 | 0.0533404 | FALSE |
| PPIAL4G | 5307 | 2.411625 | 0.015882 | 0.053347 | FALSE |
| HINT2 | 8677 | 2.411279 | 0.015897 | 0.0533633 | FALSE |
| PDIA3P1 | 2965 | 2.410896 | 0.015913 | 0.0533964 | FALSE |
| MPI | 14752 | 2.410747 | 0.01592 | 0.0534003 | FALSE |
| CDH3 | 9234 | 2.410422 | 0.015934 | 0.0534134 | FALSE |
| BCL2A1 | 4828 | 2.410389 | 0.015936 | 0.0534134 | FALSE |
| DUSP26 | 9989 | 2.410092 | 0.015948 | 0.0534454 | FALSE |
| FPR1 | 1027 | 2.409573 | 0.015971 | 0.0534994 | FALSE |
| NUTF2 | 5638 | 2.409567 | 0.015971 | 0.0534994 | FALSE |
| SPATA24 | 7045 | 2.409317 | 0.015982 | 0.0535246 | FALSE |
| YAF2 | 14425 | 2.409061 | 0.015994 | 0.0535454 | FALSE |
| PSMC1 | 7338 | 2.409019 | 0.015995 | 0.0535454 | FALSE |
| HSPA13 | 14003 | 2.406269 | 0.016116 | 0.0538321 | FALSE |
| GLI1 | 1780 | 2.404263 | 0.016205 | 0.0540735 | FALSE |
| ICE1 | 2144 | 2.403941 | 0.016219 | 0.0541096 | FALSE |
| LYRM1 | 7007 | 2.403477 | 0.01624 | 0.0541401 | FALSE |
| GPC1 | 8924 | 2.403203 | 0.016252 | 0.0541612 | FALSE |
| ACOX2 | 11305 | 2.401184 | 0.016342 | 0.0544146 | FALSE |
| RPL38 | 14592 | 2.401046 | 0.016348 | 0.0544235 | FALSE |
| SCN3A | 2581 | 2.400478 | 0.016374 | 0.0544964 | FALSE |
| ZNF185 | 13431 | 2.398411 | 0.016466 | 0.0547343 | FALSE |
| TMEM30B | 4619 | 2.398332 | 0.01647 | 0.0547343 | FALSE |
| C1GALT1 | 3593 | 2.397896 | 0.01649 | 0.0547655 | FALSE |
| MCRIP1 | 4545 | 2.396616 | 0.016547 | 0.0548988 | FALSE |
| FLJ20021 | 4557 | 2.39625 | 0.016564 | 0.054942 | FALSE |
| ITPRIPL2 | 7215 | 2.396002 | 0.016575 | 0.0549675 | FALSE |
| RIBC1 | 11261 | 2.39561 | 0.016593 | 0.0550106 | FALSE |
| PIANP | 9055 | 2.395559 | 0.016595 | 0.0550106 | FALSE |
| HPGDS | 8123 | 2.395444 | 0.0166 | 0.0550106 | FALSE |
| NLGN3 | 13681 | 2.394825 | 0.016628 | 0.055078 | FALSE |
| ASB5 | 5325 | 2.394068 | 0.016663 | 0.0551529 | FALSE |
| EIF3F | 132 | 2.393442 | 0.016691 | 0.0552238 | FALSE |
| NDUFB8 | 6015 | 2.393028 | 0.01671 | 0.0552657 | FALSE |
| CXCL2 | 7691 | 2.393008 | 0.016711 | 0.0552657 | FALSE |
| SNUPN | 6902 | 2.39261 | 0.016729 | 0.0552813 | FALSE |
| DEFB124 | 7085 | 2.392137 | 0.016751 | 0.0553035 | FALSE |
| KCNH7 | 2061 | 2.391892 | 0.016762 | 0.0553287 | FALSE |
| NAT14 | 14017 | 2.389652 | 0.016864 | 0.0556321 | FALSE |
| KRT31 | 8010 | 2.389418 | 0.016875 | 0.0556558 | FALSE |
| LOC541472 | 2024 | 2.388975 | 0.016895 | 0.0556994 | FALSE |
| RPL4 | 6756 | 2.388583 | 0.016913 | 0.0557471 | FALSE |
| CLEC18B | 389 | 2.38849 | 0.016918 | 0.0557495 | FALSE |
| AKAP5 | 13839 | 2.388135 | 0.016934 | 0.0557843 | FALSE |
| FAM96B | 10806 | 2.388068 | 0.016937 | 0.0557843 | FALSE |
| CPZ | 12695 | 2.387806 | 0.016949 | 0.0558063 | FALSE |
| TLR4 | 14816 | 2.387393 | 0.016968 | 0.0558455 | FALSE |
| IGDCC4 | 9755 | 2.384594 | 0.017098 | 0.0562248 | FALSE |
| CDH22 | 2919 | 2.384486 | 0.017103 | 0.0562295 | FALSE |
| CIDEB | 4952 | 2.384092 | 0.017121 | 0.0562608 | FALSE |
| MRPS18A | 3643 | 2.383794 | 0.017135 | 0.056288 | FALSE |
| WDR77 | 8345 | 2.383686 | 0.01714 | 0.0562927 | FALSE |
| RFC3 | 13744 | 2.383522 | 0.017148 | 0.0562941 | FALSE |
| VCAN | 10374 | 2.381853 | 0.017226 | 0.0565143 | FALSE |
| SMAD1 | 1260 | 2.381559 | 0.01724 | 0.0565475 | FALSE |
| PRPH | 8897 | 2.381221 | 0.017255 | 0.0565876 | FALSE |
| COA6 | 9163 | 2.380946 | 0.017268 | 0.0565951 | FALSE |
| TPX2 | 13356 | 2.38094 | 0.017269 | 0.0565951 | FALSE |
| TTPAL | 10380 | 2.377933 | 0.01741 | 0.057011 | FALSE |
| SNHG20 | 11022 | 2.377684 | 0.017422 | 0.0570369 | FALSE |
| BTC | 1796 | 2.37741 | 0.017435 | 0.057056 | FALSE |
| VWA7 | 457 | 2.376852 | 0.017461 | 0.0571304 | FALSE |
| CTSF | 15375 | 2.376253 | 0.017489 | 0.0572065 | FALSE |
| DRAM1 | 12607 | 2.376207 | 0.017492 | 0.0572065 | FALSE |
| NT5C3A | 514 | 2.375345 | 0.017533 | 0.0573163 | FALSE |
| MYO16 | 8929 | 2.373853 | 0.017604 | 0.0575364 | FALSE |
| RPS9 | 5814 | 2.373026 | 0.017643 | 0.0576412 | FALSE |
| RPL7A | 2747 | 2.372774 | 0.017655 | 0.0576685 | FALSE |
| SERTM1 | 1337 | 2.372615 | 0.017663 | 0.0576813 | FALSE |
| ZNF438 | 13119 | 2.371967 | 0.017694 | 0.0577705 | FALSE |
| LINC00461 | 8635 | 2.370028 | 0.017787 | 0.0580331 | FALSE |
| LMAN2L | 2987 | 2.370007 | 0.017788 | 0.0580331 | FALSE |
| OXCT1 | 5419 | 2.369907 | 0.017793 | 0.0580331 | FALSE |
| POLR2I | 11607 | 2.369643 | 0.017805 | 0.0580516 | FALSE |
| DLEC1 | 5406 | 2.369633 | 0.017806 | 0.0580516 | FALSE |
| PTPRR | 4803 | 2.369376 | 0.017818 | 0.0580798 | FALSE |
| MRPS16 | 7973 | 2.369253 | 0.017824 | 0.058087 | FALSE |
| CDC27 | 3245 | 2.36817 | 0.017876 | 0.0582452 | FALSE |
| TRPC1 | 12426 | 2.367591 | 0.017904 | 0.0583121 | FALSE |
| MFAP4 | 12431 | 2.365305 | 0.018015 | 0.0586489 | FALSE |
| UFM1 | 736 | 2.36495 | 0.018033 | 0.0586807 | FALSE |
| RHBDD2 | 13041 | 2.364493 | 0.018055 | 0.0587409 | FALSE |
| PIN4 | 11320 | 2.363031 | 0.018126 | 0.0589241 | FALSE |
| RPL23AP7 | 13970 | 2.362112 | 0.018171 | 0.0590581 | FALSE |
| LOC344967 | 5648 | 2.361636 | 0.018194 | 0.0591094 | FALSE |
| LAIR1 | 13287 | 2.361428 | 0.018205 | 0.0591209 | FALSE |
| TBXAS1 | 7168 | 2.36141 | 0.018206 | 0.0591209 | FALSE |
| CLEC4A | 2425 | 2.361314 | 0.01821 | 0.0591239 | FALSE |
| SDC4 | 14524 | 2.360791 | 0.018236 | 0.0591828 | FALSE |
| USP30-AS1 | 4881 | 2.360484 | 0.018251 | 0.0591897 | FALSE |
| C19orf38 | 11268 | 2.36044 | 0.018253 | 0.0591897 | FALSE |
| DRC1 | 8240 | 2.360288 | 0.018261 | 0.0592016 | FALSE |
| MGC70870 | 6910 | 2.360206 | 0.018265 | 0.0592025 | FALSE |
| DGKG | 2691 | 2.35767 | 0.01839 | 0.0595467 | FALSE |
| PTPA | 10190 | 2.35749 | 0.018399 | 0.0595549 | FALSE |
| TLR3 | 9060 | 2.357465 | 0.0184 | 0.0595549 | FALSE |
| NUP85 | 12685 | 2.357321 | 0.018407 | 0.0595657 | FALSE |
| DLEU1 | 6706 | 2.356977 | 0.018424 | 0.0595962 | FALSE |
| ADNP-AS1 | 1838 | 2.356891 | 0.018429 | 0.0595977 | FALSE |
| BRF2 | 1747 | 2.356677 | 0.018439 | 0.0596074 | FALSE |
| SCML1 | 4497 | 2.356621 | 0.018442 | 0.0596074 | FALSE |
| UBR7 | 14888 | 2.3566 | 0.018443 | 0.0596074 | FALSE |
| TYW5 | 6755 | 2.355514 | 0.018497 | 0.0597449 | FALSE |
| ELF4 | 15019 | 2.355211 | 0.018512 | 0.0597813 | FALSE |
| HSPA5 | 6302 | 2.355101 | 0.018518 | 0.0597866 | FALSE |
| CKS1B | 5832 | 2.353773 | 0.018584 | 0.0599511 | FALSE |
| GTPBP3 | 4849 | 2.353475 | 0.018599 | 0.0599868 | FALSE |
| OXER1 | 3835 | 2.352501 | 0.018648 | 0.0600857 | FALSE |
| MFSD9 | 10538 | 2.352009 | 0.018672 | 0.0601493 | FALSE |
| BRPF1 | 13736 | 2.351279 | 0.018709 | 0.0602426 | FALSE |
| VAPA | 15170 | 2.35045 | 0.018751 | 0.0603521 | FALSE |
| MAPK8IP1P2 | 14188 | 2.350242 | 0.018761 | 0.0603734 | FALSE |
| CHTOP | 2923 | 2.349993 | 0.018774 | 0.0604014 | FALSE |
| RIDA | 14330 | 2.349915 | 0.018778 | 0.0604016 | FALSE |
| CTBP2 | 14201 | 2.348376 | 0.018855 | 0.0606019 | FALSE |
| KIF1B | 3180 | 2.348012 | 0.018874 | 0.0606362 | FALSE |
| RIT1 | 8529 | 2.347391 | 0.018905 | 0.0607124 | FALSE |
| EFNB2 | 1671 | 2.347217 | 0.018914 | 0.0607283 | FALSE |
| GLIPR2 | 12383 | 2.345876 | 0.018982 | 0.0609276 | FALSE |
| NEXMIF | 8095 | 2.345761 | 0.018988 | 0.0609276 | FALSE |
| TMEM258 | 7863 | 2.34569 | 0.018992 | 0.0609276 | FALSE |
| TLCD1 | 11393 | 2.345423 | 0.019006 | 0.0609588 | FALSE |
| ENKUR | 6767 | 2.344566 | 0.019049 | 0.0610364 | FALSE |
| LOC101927974 | 14576 | 2.344409 | 0.019057 | 0.0610496 | FALSE |
| TMEM147 | 8142 | 2.343751 | 0.019091 | 0.0611324 | FALSE |
| MS4A4A | 5398 | 2.343606 | 0.019098 | 0.0611436 | FALSE |
| ISCA2 | 5640 | 2.342514 | 0.019154 | 0.0612852 | FALSE |
| DAD1 | 14146 | 2.342209 | 0.01917 | 0.0613227 | FALSE |
| ATP8B3 | 3448 | 2.341931 | 0.019184 | 0.0613559 | FALSE |
| PAK3 | 2375 | 2.340817 | 0.019242 | 0.0615267 | FALSE |
| ME2 | 6958 | 2.339978 | 0.019285 | 0.0616366 | FALSE |
| NXPH1 | 13035 | 2.339438 | 0.019313 | 0.0617038 | FALSE |
| C1QTNF2 | 2107 | 2.33838 | 0.019368 | 0.0618535 | FALSE |
| CYB5D2 | 11390 | 2.337978 | 0.019388 | 0.0619074 | FALSE |
| HTR2C | 11769 | 2.337489 | 0.019414 | 0.0619758 | FALSE |
| C2orf80 | 4752 | 2.337017 | 0.019438 | 0.0620362 | FALSE |
| LOC286359 | 9896 | 2.336896 | 0.019445 | 0.0620362 | FALSE |
| STK17B | 2900 | 2.33499 | 0.019544 | 0.0622896 | FALSE |
| SZRD1 | 14241 | 2.333689 | 0.019612 | 0.0624302 | FALSE |
| BLOC1S1 | 386 | 2.333109 | 0.019642 | 0.0625143 | FALSE |
| C8orf58 | 4403 | 2.332688 | 0.019665 | 0.0625719 | FALSE |
| LMCD1 | 15444 | 2.331952 | 0.019703 | 0.0626695 | FALSE |
| IMMT | 6595 | 2.331641 | 0.01972 | 0.0627088 | FALSE |
| GALNT18 | 3422 | 2.330933 | 0.019757 | 0.0627891 | FALSE |
| ADAMTSL3 | 8053 | 2.330681 | 0.01977 | 0.0628058 | FALSE |
| RPL3 | 13888 | 2.330184 | 0.019796 | 0.0628535 | FALSE |
| INSM1 | 1371 | 2.329212 | 0.019848 | 0.0629806 | FALSE |
| SBF1P1 | 6631 | 2.329177 | 0.01985 | 0.0629806 | FALSE |
| DDB1 | 1984 | 2.328867 | 0.019866 | 0.0630081 | FALSE |
| CFAP61 | 426 | 2.328666 | 0.019877 | 0.0630163 | FALSE |
| CLDN10 | 5308 | 2.327705 | 0.019928 | 0.0631652 | FALSE |
| RPL26 | 4908 | 2.326689 | 0.019982 | 0.0633237 | FALSE |
| IL4R | 13211 | 2.326129 | 0.020012 | 0.0634054 | FALSE |
| TADA3 | 9559 | 2.326039 | 0.020016 | 0.0634078 | FALSE |
| ENTPD6 | 1049 | 2.325931 | 0.020022 | 0.0634132 | FALSE |
| COL18A1 | 11009 | 2.325518 | 0.020044 | 0.0634702 | FALSE |
| CMTM4 | 5074 | 2.325372 | 0.020052 | 0.063482 | FALSE |
| PTPRN2 | 12403 | 2.323346 | 0.020161 | 0.0637738 | FALSE |
| HIST2H2AC | 3614 | 2.322423 | 0.02021 | 0.0639049 | FALSE |
| SERPINI2 | 10993 | 2.320556 | 0.020311 | 0.0641657 | FALSE |
| ZNF341 | 12288 | 2.320355 | 0.020322 | 0.0641796 | FALSE |
| ADGRV1 | 12655 | 2.319607 | 0.020362 | 0.0642814 | FALSE |
| ASCL2 | 11930 | 2.317504 | 0.020476 | 0.0645635 | FALSE |
| UBE2N | 12506 | 2.317348 | 0.020485 | 0.0645642 | FALSE |
| FDPSP2 | 13898 | 2.316233 | 0.020546 | 0.0647297 | FALSE |
| SAMD11 | 4235 | 2.315608 | 0.02058 | 0.0648241 | FALSE |
| GATM | 8686 | 2.314911 | 0.020618 | 0.0649268 | FALSE |
| ZSWIM9 | 12569 | 2.314739 | 0.020627 | 0.0649293 | FALSE |
| HLA-F | 3725 | 2.314694 | 0.02063 | 0.0649293 | FALSE |
| SLC15A2 | 1685 | 2.313573 | 0.020691 | 0.0650966 | FALSE |
| CRIM1 | 5026 | 2.312554 | 0.020747 | 0.0652334 | FALSE |
| DIAPH2 | 225 | 2.311447 | 0.020808 | 0.0653858 | FALSE |
| PIN1 | 1132 | 2.311157 | 0.020824 | 0.0654224 | FALSE |
| SEMA4F | 5046 | 2.311084 | 0.020828 | 0.0654224 | FALSE |
| TAAR5 | 5202 | 2.310636 | 0.020853 | 0.0654739 | FALSE |
| ABCC12 | 2260 | 2.309189 | 0.020933 | 0.0656991 | FALSE |
| LOC729683 | 6848 | 2.307666 | 0.021018 | 0.0658985 | FALSE |
| PJA1 | 7506 | 2.306935 | 0.021058 | 0.0659865 | FALSE |
| VCP | 8714 | 2.306603 | 0.021077 | 0.0660313 | FALSE |
| CLDND2 | 14291 | 2.306008 | 0.02111 | 0.0660956 | FALSE |
| TCEAL9 | 1645 | 2.305509 | 0.021138 | 0.0661697 | FALSE |
| DUSP28 | 2857 | 2.304889 | 0.021173 | 0.0662651 | FALSE |
| FAM120A | 4649 | 2.304166 | 0.021213 | 0.0663574 | FALSE |
| ASH1L-AS1 | 7734 | 2.304136 | 0.021215 | 0.0663574 | FALSE |
| METTL1 | 5313 | 2.30372 | 0.021238 | 0.0664172 | FALSE |
| SLC37A4 | 11455 | 2.303148 | 0.021271 | 0.0665044 | FALSE |
| TATDN1 | 7332 | 2.300841 | 0.021401 | 0.066871 | FALSE |
| TTLL1 | 2124 | 2.30071 | 0.021408 | 0.066871 | FALSE |
| CHRM3 | 5200 | 2.300304 | 0.021431 | 0.0669259 | FALSE |
| RPS10 | 3936 | 2.299695 | 0.021466 | 0.0670002 | FALSE |
| COPZ1 | 9645 | 2.29956 | 0.021473 | 0.0670002 | FALSE |
| GRM1 | 5476 | 2.299506 | 0.021476 | 0.0670002 | FALSE |
| DENND1C | 2762 | 2.299215 | 0.021493 | 0.0670383 | FALSE |
| CAPNS1 | 2533 | 2.298939 | 0.021508 | 0.0670641 | FALSE |
| PLPBP | 14906 | 2.29835 | 0.021542 | 0.0671514 | FALSE |
| CPVL | 3792 | 2.298265 | 0.021547 | 0.0671531 | FALSE |
| PSMC3 | 6396 | 2.297399 | 0.021596 | 0.0672665 | FALSE |
| EMX2 | 14229 | 2.296973 | 0.02162 | 0.0673287 | FALSE |
| MRPL28 | 626 | 2.296652 | 0.021639 | 0.067359 | FALSE |
| SLC8B1 | 6471 | 2.296421 | 0.021652 | 0.0673866 | FALSE |
| PNPLA4 | 7629 | 2.296037 | 0.021674 | 0.0674281 | FALSE |
| MTMR8 | 5588 | 2.295839 | 0.021685 | 0.0674499 | FALSE |
| CRYBB2 | 13482 | 2.295344 | 0.021713 | 0.0675007 | FALSE |
| TP53BP1 | 11834 | 2.295327 | 0.021714 | 0.0675007 | FALSE |
| ADGRG6 | 12831 | 2.295125 | 0.021726 | 0.0675233 | FALSE |
| GSTM1 | 2021 | 2.294613 | 0.021755 | 0.067601 | FALSE |
| SH2B3 | 7806 | 2.292976 | 0.021849 | 0.06785 | FALSE |
| NUDT17 | 4514 | 2.292335 | 0.021886 | 0.0679406 | FALSE |
| SOBP | 10165 | 2.292157 | 0.021897 | 0.0679578 | FALSE |
| UBE2Q1 | 4791 | 2.291192 | 0.021952 | 0.0680644 | FALSE |
| OR2Z1 | 13457 | 2.290407 | 0.021998 | 0.0681917 | FALSE |
| LINC01176 | 2135 | 2.290181 | 0.022011 | 0.0682187 | FALSE |
| C1QTNF12 | 12723 | 2.289813 | 0.022032 | 0.0682639 | FALSE |
| SH3D21 | 1711 | 2.289779 | 0.022034 | 0.0682639 | FALSE |
| DDR2 | 8658 | 2.289635 | 0.022042 | 0.0682762 | FALSE |
| TDRKH | 11039 | 2.289385 | 0.022057 | 0.0683076 | FALSE |
| LRRC4C | 920 | 2.289181 | 0.022069 | 0.0683173 | FALSE |
| DDX10 | 2458 | 2.288749 | 0.022094 | 0.0683814 | FALSE |
| COMT | 5731 | 2.286954 | 0.022199 | 0.0686507 | FALSE |
| CHST15 | 13868 | 2.286172 | 0.022244 | 0.0687648 | FALSE |
| KCNG3 | 14317 | 2.286064 | 0.022251 | 0.0687707 | FALSE |
| FYCO1 | 12555 | 2.284886 | 0.02232 | 0.0689431 | FALSE |
| CD200 | 10305 | 2.284454 | 0.022345 | 0.0689941 | FALSE |
| SYPL2 | 7663 | 2.283659 | 0.022392 | 0.0690974 | FALSE |
| MECR | 13299 | 2.283445 | 0.022404 | 0.069109 | FALSE |
| C16orf89 | 9918 | 2.283131 | 0.022423 | 0.0691524 | FALSE |
| SOWAHC | 2434 | 2.282441 | 0.022463 | 0.0692505 | FALSE |
| FUCA1 | 3942 | 2.281951 | 0.022492 | 0.0693259 | FALSE |
| PWP2 | 12064 | 2.280797 | 0.02256 | 0.0695225 | FALSE |
| TRIP12 | 8271 | 2.280631 | 0.02257 | 0.0695391 | FALSE |
| FAM167A-AS1 | 12204 | 2.28039 | 0.022585 | 0.0695684 | FALSE |
| NBEA | 5639 | 2.279557 | 0.022634 | 0.0696857 | FALSE |
| CYP11A1 | 10701 | 2.279528 | 0.022636 | 0.0696857 | FALSE |
| FAM102A | 2992 | 2.279355 | 0.022646 | 0.0697036 | FALSE |
| TTC19 | 8974 | 2.279077 | 0.022662 | 0.0697407 | FALSE |
| SLC1A6 | 12930 | 2.278726 | 0.022683 | 0.0697833 | FALSE |
| ANO7 | 7229 | 2.278694 | 0.022685 | 0.0697833 | FALSE |
| COA4 | 3961 | 2.27761 | 0.02275 | 0.0699131 | FALSE |
| NFIA | 12155 | 2.276958 | 0.022789 | 0.0699777 | FALSE |
| MRPS33 | 13608 | 2.276606 | 0.02281 | 0.0700285 | FALSE |
| ZFP69 | 3513 | 2.276194 | 0.022834 | 0.0700797 | FALSE |
| C9orf131 | 3112 | 2.276177 | 0.022835 | 0.0700797 | FALSE |
| HLA-DRB4 | 10815 | 2.276008 | 0.022846 | 0.070097 | FALSE |
| GDE1 | 424 | 2.274241 | 0.022952 | 0.0703531 | FALSE |
| FERMT1 | 5309 | 2.273842 | 0.022975 | 0.070399 | FALSE |
| HOXD8 | 3177 | 2.273188 | 0.023015 | 0.0705058 | FALSE |
| G6PC | 15431 | 2.272968 | 0.023028 | 0.0705326 | FALSE |
| AMOT | 7070 | 2.272269 | 0.02307 | 0.0706341 | FALSE |
| TNFSF12 | 14595 | 2.271789 | 0.023099 | 0.070709 | FALSE |
| TRPC4 | 987 | 2.271554 | 0.023113 | 0.0707122 | FALSE |
| TAAR8 | 5555 | 2.271547 | 0.023114 | 0.0707122 | FALSE |
| KLRC3 | 10229 | 2.271335 | 0.023127 | 0.0707306 | FALSE |
| SH3GLB1 | 5104 | 2.270859 | 0.023156 | 0.070798 | FALSE |
| RENBP | 11949 | 2.270595 | 0.023172 | 0.070833 | FALSE |
| DOLK | 2004 | 2.270013 | 0.023207 | 0.0709132 | FALSE |
| CHCHD5 | 4959 | 2.269557 | 0.023234 | 0.0709839 | FALSE |
| CGNL1 | 9383 | 2.269238 | 0.023254 | 0.0710292 | FALSE |
| PMP2 | 13611 | 2.267759 | 0.023344 | 0.0712764 | FALSE |
| IGSF23 | 1894 | 2.266301 | 0.023433 | 0.0715203 | FALSE |
| GNG3 | 5837 | 2.266227 | 0.023437 | 0.0715203 | FALSE |
| CYP2J2 | 13790 | 2.26545 | 0.023485 | 0.0716515 | FALSE |
| FGF3 | 12165 | 2.265175 | 0.023502 | 0.071689 | FALSE |
| RIOX2 | 6291 | 2.264326 | 0.023554 | 0.0718199 | FALSE |
| NEK6 | 4887 | 2.263818 | 0.023585 | 0.0719012 | FALSE |
| COL23A1 | 13954 | 2.262693 | 0.023655 | 0.0720653 | FALSE |
| RAB3IP | 1041 | 2.262645 | 0.023658 | 0.0720653 | FALSE |
| SSBP1 | 8025 | 2.262485 | 0.023667 | 0.0720813 | FALSE |
| PTGR2 | 4061 | 2.26215 | 0.023688 | 0.0721162 | FALSE |
| RAB23 | 5995 | 2.260928 | 0.023764 | 0.07229 | FALSE |
| PSMG3-AS1 | 15037 | 2.260577 | 0.023785 | 0.0723421 | FALSE |
| SREK1IP1 | 5017 | 2.260225 | 0.023807 | 0.0723944 | FALSE |
| S100A13 | 11344 | 2.258935 | 0.023887 | 0.0725667 | FALSE |
| ACO2 | 3958 | 2.258854 | 0.023892 | 0.0725667 | FALSE |
| C9orf163 | 4991 | 2.258721 | 0.023901 | 0.0725667 | FALSE |
| CD151 | 9496 | 2.258715 | 0.023901 | 0.0725667 | FALSE |
| LY86-AS1 | 280 | 2.258562 | 0.023911 | 0.0725675 | FALSE |
| ITGB8 | 7376 | 2.256409 | 0.024045 | 0.0729103 | FALSE |
| SULF1 | 6205 | 2.256398 | 0.024046 | 0.0729103 | FALSE |
| EMILIN1 | 5039 | 2.256304 | 0.024052 | 0.0729103 | FALSE |
| ADORA2B | 9366 | 2.25522 | 0.02412 | 0.0730879 | FALSE |
| C17orf58 | 4460 | 2.255054 | 0.02413 | 0.0731053 | FALSE |
| CTXND1 | 6437 | 2.254083 | 0.024191 | 0.0732617 | FALSE |
| SLN | 10101 | 2.253761 | 0.024211 | 0.0733089 | FALSE |
| HEPN1 | 2076 | 2.252149 | 0.024313 | 0.073582 | FALSE |
| MYO1F | 7772 | 2.25198 | 0.024324 | 0.073592 | FALSE |
| LHX2 | 11821 | 2.251789 | 0.024336 | 0.0736136 | FALSE |
| VTI1B | 9326 | 2.251588 | 0.024348 | 0.0736173 | FALSE |
| RASL10A | 13627 | 2.250343 | 0.024427 | 0.0737628 | FALSE |
| ATP5MG | 12894 | 2.249119 | 0.024505 | 0.0739657 | FALSE |
| PDCD2L | 6832 | 2.249062 | 0.024509 | 0.0739657 | FALSE |
| MCFD2 | 6901 | 2.248304 | 0.024557 | 0.0740827 | FALSE |
| PTRH2 | 637 | 2.247847 | 0.024586 | 0.0741337 | FALSE |
| CCL19 | 10447 | 2.247684 | 0.024596 | 0.0741448 | FALSE |
| GRIN2C | 10892 | 2.247385 | 0.024615 | 0.0741682 | FALSE |
| IVD | 14152 | 2.247339 | 0.024618 | 0.0741682 | FALSE |
| PPP1R3G | 13828 | 2.247038 | 0.024638 | 0.0741969 | FALSE |
| SMPDL3A | 3750 | 2.246131 | 0.024696 | 0.0743409 | FALSE |
| GCDH | 353 | 2.246071 | 0.024699 | 0.0743409 | FALSE |
| SP100 | 15181 | 2.245861 | 0.024713 | 0.0743671 | FALSE |
| EFNB1 | 2263 | 2.245649 | 0.024726 | 0.0743936 | FALSE |
| STX2 | 3585 | 2.245061 | 0.024764 | 0.0744928 | FALSE |
| DIRC2 | 13118 | 2.244032 | 0.02483 | 0.0746773 | FALSE |
| PDGFB | 12001 | 2.243551 | 0.024861 | 0.0747273 | FALSE |
| COP1 | 2935 | 2.240127 | 0.025083 | 0.0753637 | FALSE |
| TRPS1 | 9933 | 2.239446 | 0.025127 | 0.0754676 | FALSE |
| PYGO2 | 4151 | 2.23923 | 0.025141 | 0.0754808 | FALSE |
| NAA38 | 455 | 2.238416 | 0.025194 | 0.0756254 | FALSE |
| PPP1R18 | 6232 | 2.238077 | 0.025216 | 0.0756481 | FALSE |
| HAUS7 | 7502 | 2.237593 | 0.025248 | 0.0757283 | FALSE |
| GRAMD1C | 12227 | 2.237378 | 0.025262 | 0.0757515 | FALSE |
| ZCRB1 | 11294 | 2.236654 | 0.025309 | 0.0758583 | FALSE |
| SUCO | 9802 | 2.236422 | 0.025324 | 0.0758583 | FALSE |
| SLC25A22 | 2316 | 2.235115 | 0.02541 | 0.0760834 | FALSE |
| CENPM | 3937 | 2.233432 | 0.02552 | 0.0763417 | FALSE |
| NRDC | 9951 | 2.232565 | 0.025578 | 0.0764714 | FALSE |
| RBFA | 1422 | 2.232552 | 0.025579 | 0.0764714 | FALSE |
| RHBDF1 | 1156 | 2.232246 | 0.025599 | 0.0765171 | FALSE |
| DPH6 | 9355 | 2.23147 | 0.02565 | 0.0766245 | FALSE |
| ZFAS1 | 9364 | 2.231395 | 0.025655 | 0.0766245 | FALSE |
| FIS1 | 2331 | 2.23089 | 0.025688 | 0.0766787 | FALSE |
| FLJ33534 | 12101 | 2.230836 | 0.025692 | 0.0766787 | FALSE |
| SLC15A3 | 10668 | 2.230486 | 0.025715 | 0.0767333 | FALSE |
| EPHA8 | 9822 | 2.230379 | 0.025722 | 0.0767398 | FALSE |
| MED14OS | 7104 | 2.228921 | 0.025819 | 0.0769407 | FALSE |
| CCT6B | 331 | 2.227965 | 0.025883 | 0.0771011 | FALSE |
| DLG2 | 14758 | 2.2266 | 0.025974 | 0.0772696 | FALSE |
| SAR1A | 5387 | 2.226449 | 0.025984 | 0.077285 | FALSE |
| WDR92 | 628 | 2.223375 | 0.026191 | 0.0778396 | FALSE |
| ASB6 | 4734 | 2.222977 | 0.026217 | 0.0778768 | FALSE |
| ARPC5 | 13048 | 2.222971 | 0.026218 | 0.0778768 | FALSE |
| ABHD17C | 1975 | 2.222968 | 0.026218 | 0.0778768 | FALSE |
| SIPA1 | 7853 | 2.222441 | 0.026254 | 0.0779528 | FALSE |
| PNOC | 878 | 2.222016 | 0.026282 | 0.0780164 | FALSE |
| HCLS1 | 5842 | 2.221976 | 0.026285 | 0.0780164 | FALSE |
| MVK | 11634 | 2.22106 | 0.026347 | 0.0781706 | FALSE |
| AAMDC | 347 | 2.220714 | 0.02637 | 0.0782253 | FALSE |
| NAV1 | 716 | 2.219052 | 0.026483 | 0.0785005 | FALSE |
| MYD88 | 666 | 2.218625 | 0.026512 | 0.0785569 | FALSE |
| LINC01750 | 10206 | 2.2182 | 0.026541 | 0.0786128 | FALSE |
| MTSS1 | 2065 | 2.21798 | 0.026556 | 0.0786424 | FALSE |
| CCS | 8411 | 2.216966 | 0.026625 | 0.0787876 | FALSE |
| SYCN | 8915 | 2.21687 | 0.026632 | 0.0787921 | FALSE |
| PRRT1 | 106 | 2.216364 | 0.026667 | 0.0788647 | FALSE |
| EIF3K | 13774 | 2.215873 | 0.0267 | 0.0789342 | FALSE |
| ORC3 | 10968 | 2.215067 | 0.026755 | 0.0790827 | FALSE |
| STXBP3 | 12938 | 2.214593 | 0.026788 | 0.0791639 | FALSE |
| ZDHHC7 | 213 | 2.214216 | 0.026814 | 0.0792255 | FALSE |
| FBXW9 | 12343 | 2.211814 | 0.02698 | 0.0796997 | FALSE |
| HIST1H2BL | 7210 | 2.211575 | 0.026996 | 0.0797335 | FALSE |
| DPYSL3 | 5819 | 2.211009 | 0.027035 | 0.0798341 | FALSE |
| CALY | 11630 | 2.210606 | 0.027063 | 0.0799014 | FALSE |
| STK17A | 8385 | 2.207707 | 0.027265 | 0.0804206 | FALSE |
| SSSCA1-AS1 | 4540 | 2.207524 | 0.027277 | 0.0804279 | FALSE |
| TSACC | 1981 | 2.207367 | 0.027288 | 0.080445 | FALSE |
| NAA11 | 11707 | 2.205226 | 0.027438 | 0.0808409 | FALSE |
| RAD17 | 8531 | 2.205002 | 0.027454 | 0.080872 | FALSE |
| CDHR1 | 14110 | 2.203714 | 0.027544 | 0.0810894 | FALSE |
| SORCS2 | 3866 | 2.20364 | 0.02755 | 0.0810894 | FALSE |
| NIPSNAP1 | 12336 | 2.203583 | 0.027554 | 0.0810894 | FALSE |
| SMIM27 | 14780 | 2.202355 | 0.02764 | 0.0813288 | FALSE |
| RDH11 | 1650 | 2.201028 | 0.027734 | 0.0815894 | FALSE |
| LCMT1 | 12574 | 2.200865 | 0.027746 | 0.081608 | FALSE |
| NDN | 11082 | 2.200538 | 0.027769 | 0.0816608 | FALSE |
| SSSCA1 | 1523 | 2.198967 | 0.02788 | 0.0819579 | FALSE |
| MAD2L2 | 6809 | 2.196924 | 0.028026 | 0.0822931 | FALSE |
| NSFL1C | 15584 | 2.196407 | 0.028063 | 0.0823707 | FALSE |
| RPL35A | 8943 | 2.196179 | 0.028079 | 0.0824031 | FALSE |
| AKR7A3 | 5246 | 2.195384 | 0.028136 | 0.0825391 | FALSE |
| USP5 | 3700 | 2.195205 | 0.028149 | 0.0825613 | FALSE |
| TMEM120A | 11387 | 2.194754 | 0.028181 | 0.0826407 | FALSE |
| SSR3 | 5841 | 2.194601 | 0.028192 | 0.0826419 | FALSE |
| ATF5 | 11377 | 2.194319 | 0.028212 | 0.0826702 | FALSE |
| SSTR2 | 12643 | 2.193778 | 0.028251 | 0.0827512 | FALSE |
| TPTE2P3 | 10711 | 2.193714 | 0.028256 | 0.0827512 | FALSE |
| CABCOCO1 | 5784 | 2.193358 | 0.028282 | 0.0827797 | FALSE |
| PPP1R1B | 7784 | 2.191821 | 0.028392 | 0.0830574 | FALSE |
| RPL34 | 1394 | 2.190998 | 0.028452 | 0.0832142 | FALSE |
| ENPP1 | 13075 | 2.190872 | 0.028461 | 0.0832142 | FALSE |
| COPS6 | 3108 | 2.190859 | 0.028462 | 0.0832142 | FALSE |
| RPLP0 | 5645 | 2.190595 | 0.028481 | 0.0832545 | FALSE |
| USP11 | 481 | 2.190251 | 0.028506 | 0.0833045 | FALSE |
| CALN1 | 14669 | 2.189715 | 0.028545 | 0.0833942 | FALSE |
| ZNF157 | 3052 | 2.189369 | 0.02857 | 0.0834515 | FALSE |
| RACK1 | 12996 | 2.188132 | 0.02866 | 0.0836657 | FALSE |
| ALAS2 | 2707 | 2.187179 | 0.028729 | 0.0838117 | FALSE |
| LRRN1 | 2578 | 2.18626 | 0.028797 | 0.0839726 | FALSE |
| BORCS8-MEF2B | 13458 | 2.185863 | 0.028826 | 0.0840291 | FALSE |
| SQOR | 4190 | 2.185305 | 0.028866 | 0.0841294 | FALSE |
| SLC2A8 | 3050 | 2.184881 | 0.028898 | 0.0842042 | FALSE |
| ATXN10 | 10389 | 2.184737 | 0.028908 | 0.0842108 | FALSE |
| DCAF11 | 2419 | 2.18463 | 0.028916 | 0.0842108 | FALSE |
| NDUFAF1 | 1362 | 2.184418 | 0.028932 | 0.0842404 | FALSE |
| RPL13A | 4087 | 2.184249 | 0.028944 | 0.0842609 | FALSE |
| DTX1 | 15579 | 2.183931 | 0.028967 | 0.0842975 | FALSE |
| PCDH15 | 7053 | 2.18354 | 0.028996 | 0.0843497 | FALSE |
| RPS11 | 12337 | 2.182825 | 0.029049 | 0.0844868 | FALSE |
| RRAGC | 7260 | 2.182599 | 0.029065 | 0.0844868 | FALSE |
| TMEM150B | 3057 | 2.182535 | 0.02907 | 0.0844868 | FALSE |
| NQO1 | 5578 | 2.182533 | 0.02907 | 0.0844868 | FALSE |
| TMEM141 | 9186 | 2.182156 | 0.029098 | 0.0845519 | FALSE |
| MOS | 15062 | 2.180798 | 0.029198 | 0.0847951 | FALSE |
| RAB3C | 6888 | 2.18073 | 0.029203 | 0.0847951 | FALSE |
| LOC102723665 | 10706 | 2.180497 | 0.029221 | 0.0848294 | FALSE |
| SLC30A10 | 6773 | 2.179887 | 0.029266 | 0.0849291 | FALSE |
| DRAP1 | 14561 | 2.179036 | 0.029329 | 0.0850492 | FALSE |
| C1orf54 | 9847 | 2.178862 | 0.029342 | 0.0850709 | FALSE |
| ARRB1 | 8045 | 2.177689 | 0.029429 | 0.0853081 | FALSE |
| RBM24 | 9403 | 2.177203 | 0.029465 | 0.0853973 | FALSE |
| BOP1 | 2463 | 2.176711 | 0.029502 | 0.085472 | FALSE |
| KPNA4 | 2894 | 2.176614 | 0.029509 | 0.0854771 | FALSE |
| LRSAM1 | 7665 | 2.176245 | 0.029537 | 0.0855411 | FALSE |
| RPL5 | 1620 | 2.175971 | 0.029557 | 0.0855846 | FALSE |
| WDR83OS | 7572 | 2.17556 | 0.029588 | 0.0856332 | FALSE |
| PUS7L | 2223 | 2.175041 | 0.029627 | 0.0857227 | FALSE |
| DAP3 | 5687 | 2.174679 | 0.029654 | 0.0857853 | FALSE |
| CROT | 13016 | 2.171197 | 0.029916 | 0.0864631 | FALSE |
| C3AR1 | 12604 | 2.171126 | 0.029922 | 0.0864631 | FALSE |
| LAPTM4A | 11495 | 2.17055 | 0.029965 | 0.0865729 | FALSE |
| HLA-DPA1 | 12726 | 2.169789 | 0.030023 | 0.0867074 | FALSE |
| DNAH9 | 550 | 2.169123 | 0.030073 | 0.0868051 | FALSE |
| AHSG | 6431 | 2.168385 | 0.030129 | 0.0869348 | FALSE |
| TXN2 | 9315 | 2.167347 | 0.030208 | 0.0871306 | FALSE |
| ZNF826P | 5582 | 2.166533 | 0.03027 | 0.0872614 | FALSE |
| DIS3L | 6876 | 2.164895 | 0.030396 | 0.0875879 | FALSE |
| CALHM2 | 268 | 2.163853 | 0.030476 | 0.0877634 | FALSE |
| NAA35 | 10588 | 2.163317 | 0.030517 | 0.0878095 | FALSE |
| NEUROD2 | 3820 | 2.161553 | 0.030653 | 0.0881073 | FALSE |
| ANXA2P1 | 14512 | 2.161525 | 0.030655 | 0.0881073 | FALSE |
| ASTN1 | 5858 | 2.161394 | 0.030665 | 0.0881073 | FALSE |
| BHMT2 | 11372 | 2.161318 | 0.030671 | 0.0881073 | FALSE |
| PRODH | 3825 | 2.160862 | 0.030706 | 0.0881916 | FALSE |
| PIGV | 3850 | 2.16057 | 0.030729 | 0.0882402 | FALSE |
| CC2D2A | 3621 | 2.160318 | 0.030748 | 0.08828 | FALSE |
| CX3CL1 | 10911 | 2.158331 | 0.030902 | 0.088706 | FALSE |
| PNPLA7 | 15002 | 2.158216 | 0.030911 | 0.0887153 | FALSE |
| PAX4 | 14554 | 2.15774 | 0.030948 | 0.0887903 | FALSE |
| ANKRD2 | 8087 | 2.157734 | 0.030949 | 0.0887903 | FALSE |
| MRTO4 | 11704 | 2.156697 | 0.031029 | 0.0889685 | FALSE |
| TMEM53 | 5866 | 2.15653 | 0.031042 | 0.0889735 | FALSE |
| CRLF2 | 14355 | 2.155935 | 0.031089 | 0.0890781 | FALSE |
| TMED1 | 6819 | 2.154052 | 0.031236 | 0.0894839 | FALSE |
| DNAJB1 | 6160 | 2.153868 | 0.031251 | 0.0895089 | FALSE |
| FAM181B | 10757 | 2.153658 | 0.031267 | 0.0895266 | FALSE |
| AVPR2 | 545 | 2.153209 | 0.031302 | 0.089575 | FALSE |
| CUL4A | 8257 | 2.152783 | 0.031336 | 0.089638 | FALSE |
| DOCK9-AS2 | 15143 | 2.152008 | 0.031397 | 0.089796 | FALSE |
| GOLGB1 | 2281 | 2.151932 | 0.031403 | 0.0897967 | FALSE |
| MINCR | 2722 | 2.151195 | 0.031461 | 0.0898971 | FALSE |
| EBNA1BP2 | 1524 | 2.150534 | 0.031513 | 0.0899968 | FALSE |
| B3GNTL1 | 250 | 2.150126 | 0.031545 | 0.0900629 | FALSE |
| FLNA | 7086 | 2.150059 | 0.031551 | 0.0900629 | FALSE |
| TRMT5 | 14520 | 2.150023 | 0.031553 | 0.0900629 | FALSE |
| SCCPDH | 8375 | 2.149366 | 0.031605 | 0.0901784 | FALSE |
| EXOC5 | 599 | 2.148353 | 0.031686 | 0.0903251 | FALSE |
| PLPPR1 | 2719 | 2.148184 | 0.031699 | 0.0903469 | FALSE |
| FAM47E | 8534 | 2.14754 | 0.03175 | 0.0904598 | FALSE |
| DUSP2 | 2725 | 2.147003 | 0.031793 | 0.090565 | FALSE |
| SLURP1 | 11733 | 2.146791 | 0.03181 | 0.0905966 | FALSE |
| SHISA7 | 8522 | 2.145989 | 0.031874 | 0.0907291 | FALSE |
| EPN2 | 9063 | 2.145189 | 0.031938 | 0.0908778 | FALSE |
| COA3 | 9054 | 2.145093 | 0.031945 | 0.0908831 | FALSE |
| PLXNA1 | 4556 | 2.144676 | 0.031979 | 0.0909161 | FALSE |
| GAL3ST4 | 3119 | 2.144469 | 0.031995 | 0.0909258 | FALSE |
| CALD1 | 14407 | 2.143013 | 0.032112 | 0.0912078 | FALSE |
| SLC7A7 | 1134 | 2.14276 | 0.032132 | 0.0912367 | FALSE |
| UBE2L6 | 7797 | 2.141802 | 0.032209 | 0.0914345 | FALSE |
| CD83 | 260 | 2.141508 | 0.032233 | 0.0914851 | FALSE |
| PNO1 | 5654 | 2.141373 | 0.032244 | 0.0914994 | FALSE |
| SCAPER | 8700 | 2.140857 | 0.032286 | 0.0916008 | FALSE |
| LILRA4 | 7255 | 2.140569 | 0.032309 | 0.091644 | FALSE |
| KCNE4 | 153 | 2.140523 | 0.032313 | 0.091644 | FALSE |
| PCDH8 | 1546 | 2.139684 | 0.03238 | 0.0918029 | FALSE |
| SSTR3 | 14210 | 2.139505 | 0.032395 | 0.0918212 | FALSE |
| CASK | 11599 | 2.139432 | 0.032401 | 0.0918212 | FALSE |
| CSH1 | 8946 | 2.139331 | 0.032409 | 0.0918212 | FALSE |
| CAPN12 | 2628 | 2.139314 | 0.03241 | 0.0918212 | FALSE |
| SMOX | 14651 | 2.139067 | 0.03243 | 0.0918612 | FALSE |
| SLC6A16 | 5635 | 2.138838 | 0.032449 | 0.0918804 | FALSE |
| TTC23L | 10134 | 2.138281 | 0.032494 | 0.0919916 | FALSE |
| RARA-AS1 | 8131 | 2.137382 | 0.032567 | 0.0921816 | FALSE |
| B3GALT2 | 15183 | 2.136963 | 0.032601 | 0.0922613 | FALSE |
| PSMB6 | 15005 | 2.135437 | 0.032725 | 0.0925964 | FALSE |
| PAAF1 | 12487 | 2.135007 | 0.03276 | 0.092679 | FALSE |
| COL8A2 | 6548 | 2.134872 | 0.032771 | 0.0926934 | FALSE |
| SRI | 14282 | 2.13399 | 0.032844 | 0.0928459 | FALSE |
| RBM5 | 11346 | 2.133931 | 0.032848 | 0.0928459 | FALSE |
| DRG2 | 5432 | 2.13381 | 0.032858 | 0.0928459 | FALSE |
| OAZ3 | 4345 | 2.133279 | 0.032902 | 0.0929277 | FALSE |
| HSD17B10 | 9674 | 2.132709 | 0.032949 | 0.0930227 | FALSE |
| MRPS25 | 10226 | 2.13142 | 0.033055 | 0.0932579 | FALSE |
| NEDD4 | 2105 | 2.13111 | 0.03308 | 0.0933006 | FALSE |
| SYTL2 | 8446 | 2.131091 | 0.033082 | 0.0933006 | FALSE |
| MYO3A | 14002 | 2.130257 | 0.03315 | 0.0934777 | FALSE |
| LINC02004 | 1448 | 2.129859 | 0.033183 | 0.0935534 | FALSE |
| UCP3 | 12918 | 2.129571 | 0.033207 | 0.0936017 | FALSE |
| SFTA1P | 1515 | 2.129237 | 0.033235 | 0.0936477 | FALSE |
| SNHG18 | 9345 | 2.128291 | 0.033313 | 0.0938345 | FALSE |
| CUEDC1 | 2454 | 2.127572 | 0.033373 | 0.0939855 | FALSE |
| MAML2 | 10690 | 2.127376 | 0.033389 | 0.0940144 | FALSE |
| RPS12 | 2562 | 2.126989 | 0.033421 | 0.094088 | FALSE |
| CERS6 | 12914 | 2.126586 | 0.033454 | 0.0941484 | FALSE |
| TSPAN33 | 11278 | 2.125614 | 0.033535 | 0.0943138 | FALSE |
| ANKRD12 | 9106 | 2.12559 | 0.033537 | 0.0943138 | FALSE |
| PQBP1 | 3139 | 2.125193 | 0.033571 | 0.0943729 | FALSE |
| CREB3 | 6669 | 2.124444 | 0.033633 | 0.0944977 | FALSE |
| TOLLIP | 7291 | 2.124065 | 0.033665 | 0.0945357 | FALSE |
| RNF5P1 | 5572 | 2.123894 | 0.033679 | 0.0945589 | FALSE |
| SEMA3A | 5001 | 2.122361 | 0.033807 | 0.0948684 | FALSE |
| AMN | 12781 | 2.122049 | 0.033834 | 0.0949078 | FALSE |
| FFAR4 | 14665 | 2.121298 | 0.033897 | 0.0950589 | FALSE |
| POLR3H | 7469 | 2.121263 | 0.0339 | 0.0950589 | FALSE |
| CHST6 | 12442 | 2.12077 | 0.033941 | 0.0951412 | FALSE |
| AP1S1 | 9557 | 2.120142 | 0.033994 | 0.0952724 | FALSE |
| VASH1 | 12706 | 2.119909 | 0.034014 | 0.0953104 | FALSE |
| DPP10-AS1 | 12256 | 2.118304 | 0.034149 | 0.0956279 | FALSE |
| RANBP3L | 13618 | 2.118247 | 0.034154 | 0.0956279 | FALSE |
| NABP1 | 1559 | 2.117802 | 0.034192 | 0.0956951 | FALSE |
| ZNF572 | 11613 | 2.117613 | 0.034208 | 0.0957171 | FALSE |
| CRYL1 | 3854 | 2.117327 | 0.034232 | 0.0957678 | FALSE |
| IGFBP7 | 3315 | 2.115331 | 0.034402 | 0.0961908 | FALSE |
| STAU1 | 10124 | 2.11327 | 0.034578 | 0.0966481 | FALSE |
| FAM162A | 2635 | 2.113184 | 0.034585 | 0.0966514 | FALSE |
| DERA | 15083 | 2.11289 | 0.03461 | 0.0967044 | FALSE |
| RPL18A | 13086 | 2.111444 | 0.034734 | 0.0969988 | FALSE |
| CUL2 | 11934 | 2.110388 | 0.034825 | 0.0971934 | FALSE |
| FLJ37035 | 11977 | 2.110344 | 0.034829 | 0.0971934 | FALSE |
| RTTN | 13637 | 2.109304 | 0.034918 | 0.097374 | FALSE |
| ATP6V0A4 | 8707 | 2.109062 | 0.034939 | 0.0973975 | FALSE |
| FXYD5 | 7517 | 2.108449 | 0.034992 | 0.0975103 | FALSE |
| TTC7B | 9155 | 2.107522 | 0.035072 | 0.0976989 | FALSE |
| ANGPT4 | 10996 | 2.106508 | 0.03516 | 0.097874 | FALSE |
| MNDA | 12305 | 2.106363 | 0.035173 | 0.0978916 | FALSE |
| SNAI2 | 7975 | 2.105633 | 0.035236 | 0.0980331 | FALSE |
| TENM3 | 5260 | 2.105231 | 0.035271 | 0.0980955 | FALSE |
| ST8SIA2 | 7473 | 2.104253 | 0.035356 | 0.0982974 | FALSE |
| USP9X | 191 | 2.103697 | 0.035405 | 0.0983798 | FALSE |
| MPL | 15371 | 2.103489 | 0.035423 | 0.0983953 | FALSE |
| SLFN12 | 3201 | 2.103213 | 0.035447 | 0.0984272 | FALSE |
| ZNF846 | 12296 | 2.102694 | 0.035493 | 0.0985182 | FALSE |
| ACPP | 5642 | 2.102305 | 0.035527 | 0.0985602 | FALSE |
| NOP10 | 8616 | 2.102233 | 0.035533 | 0.0985602 | FALSE |
| LBHD1 | 4745 | 2.101894 | 0.035563 | 0.0986202 | FALSE |
| FOXG1 | 3587 | 2.100333 | 0.0357 | 0.0989523 | FALSE |
| LINC01551 | 1661 | 2.100157 | 0.035715 | 0.0989777 | FALSE |
| ADPRHL2 | 9705 | 2.099436 | 0.035778 | 0.0991184 | FALSE |
| RPL7L1 | 1149 | 2.099309 | 0.03579 | 0.0991318 | FALSE |
| ZFP64 | 12338 | 2.098936 | 0.035823 | 0.0991699 | FALSE |
| ADCK2 | 2340 | 2.098713 | 0.035842 | 0.099181 | FALSE |
| SREBF1 | 6793 | 2.098569 | 0.035855 | 0.099181 | FALSE |
| PPP2R3B | 10770 | 2.098472 | 0.035863 | 0.099181 | FALSE |
| KRT86 | 780 | 2.098452 | 0.035865 | 0.099181 | FALSE |
| GNRH2 | 2386 | 2.098388 | 0.035871 | 0.099181 | FALSE |
| SLC39A3 | 4660 | 2.097471 | 0.035952 | 0.0993875 | FALSE |
| SELENOM | 10117 | 2.096679 | 0.036022 | 0.099546 | FALSE |
| CILP2 | 6802 | 2.096587 | 0.03603 | 0.099551 | FALSE |
| SUV39H1 | 3428 | 2.09629 | 0.036056 | 0.0996061 | FALSE |
| DPP10 | 2177 | 2.09565 | 0.036113 | 0.0997296 | FALSE |
| TTC23 | 5810 | 2.095642 | 0.036114 | 0.0997296 | FALSE |
| ZC2HC1A | 2606 | 2.095418 | 0.036134 | 0.0997445 | FALSE |
| CLIC6 | 11056 | 2.095384 | 0.036137 | 0.0997445 | FALSE |
| DEAF1 | 11867 | 2.095007 | 0.03617 | 0.0998149 | FALSE |
| UMOD | 504 | 2.094695 | 0.036198 | 0.0998738 | FALSE |
| GPI | 9176 | 2.09392 | 0.036267 | 0.1000198 | FALSE |
| ACOX3 | 11921 | 2.093694 | 0.036287 | 0.1000491 | FALSE |
| ATP6V0E2 | 10233 | 2.092871 | 0.036361 | 0.1002162 | FALSE |
| ZNF646 | 3246 | 2.092534 | 0.036391 | 0.1002815 | FALSE |
| ALPPL2 | 2102 | 2.092124 | 0.036427 | 0.1003513 | FALSE |
| LOC390705 | 10319 | 2.092107 | 0.036429 | 0.1003513 | FALSE |
| SDF2L1 | 11974 | 2.091735 | 0.036462 | 0.1004076 | FALSE |
| MOB3A | 3324 | 2.091605 | 0.036474 | 0.100422 | FALSE |
| HS3ST4 | 15231 | 2.090805 | 0.036546 | 0.1005308 | FALSE |
| GRASP | 12517 | 2.089898 | 0.036627 | 0.1006995 | FALSE |
| SYP | 13282 | 2.089835 | 0.036633 | 0.1006995 | FALSE |
| CLEC14A | 147 | 2.088694 | 0.036735 | 0.1009639 | FALSE |
| AGPAT4 | 5890 | 2.088279 | 0.036773 | 0.1010417 | FALSE |
| FBXL16 | 263 | 2.088131 | 0.036786 | 0.1010417 | FALSE |
| CSTB | 10917 | 2.088093 | 0.036789 | 0.1010417 | FALSE |
| MUC4 | 5738 | 2.08796 | 0.036801 | 0.1010569 | FALSE |
| C2orf70 | 3342 | 2.087625 | 0.036832 | 0.1011213 | FALSE |
| KIF3A | 2110 | 2.087525 | 0.036841 | 0.1011213 | FALSE |
| FUT10 | 6961 | 2.087485 | 0.036844 | 0.1011213 | FALSE |
| PDIA3 | 6094 | 2.086229 | 0.036958 | 0.1013715 | FALSE |
| KRT81 | 15548 | 2.08619 | 0.036961 | 0.1013715 | FALSE |
| ALG5 | 5496 | 2.085651 | 0.03701 | 0.1014877 | FALSE |
| C6orf89 | 12043 | 2.084698 | 0.037097 | 0.101707 | FALSE |
| PYCR1 | 5923 | 2.084407 | 0.037123 | 0.1017616 | FALSE |
| PTCH1 | 7606 | 2.083971 | 0.037163 | 0.1018237 | FALSE |
| ZBTB48 | 3874 | 2.083943 | 0.037165 | 0.1018237 | FALSE |
| CCK | 12325 | 2.083197 | 0.037233 | 0.1019919 | FALSE |
| MLLT11 | 7875 | 2.082728 | 0.037276 | 0.1020911 | FALSE |
| LINC01554 | 1467 | 2.081726 | 0.037368 | 0.1023133 | FALSE |
| CORO1B | 7453 | 2.081696 | 0.03737 | 0.1023133 | FALSE |
| PAXIP1-AS1 | 9071 | 2.081592 | 0.03738 | 0.1023214 | FALSE |
| EXOC7 | 4274 | 2.080901 | 0.037443 | 0.1024586 | FALSE |
| MUC5AC | 14817 | 2.079329 | 0.037587 | 0.1028041 | FALSE |
| PRDM16 | 9468 | 2.079309 | 0.037589 | 0.1028041 | FALSE |
| SPATA13 | 8591 | 2.078603 | 0.037654 | 0.1029498 | FALSE |
| ACAD9 | 5190 | 2.078586 | 0.037655 | 0.1029498 | FALSE |
| SSPN | 10689 | 2.078103 | 0.0377 | 0.1030353 | FALSE |
| COL16A1 | 10104 | 2.077731 | 0.037734 | 0.1030749 | FALSE |
| SERPINB8 | 652 | 2.077502 | 0.037755 | 0.1031093 | FALSE |
| VLDLR-AS1 | 8605 | 2.077451 | 0.03776 | 0.1031093 | FALSE |
| PPOX | 8551 | 2.07738 | 0.037767 | 0.1031093 | FALSE |
| APEX1 | 3501 | 2.076639 | 0.037835 | 0.1032599 | FALSE |
| PPP1R1C | 3346 | 2.076555 | 0.037843 | 0.1032631 | FALSE |
| TMEM107 | 4714 | 2.076209 | 0.037875 | 0.1033323 | FALSE |
| ANKRD50 | 6074 | 2.075339 | 0.037955 | 0.1035114 | FALSE |
| KIAA1211 | 12222 | 2.075285 | 0.03796 | 0.1035114 | FALSE |
| KRTAP2-1 | 7356 | 2.074824 | 0.038003 | 0.1036098 | FALSE |
| HIST1H2BC | 275 | 2.074482 | 0.038035 | 0.1036601 | FALSE |
| NOL4L | 13642 | 2.07397 | 0.038082 | 0.1037535 | FALSE |
| AKR1A1 | 13718 | 2.07302 | 0.03817 | 0.1039579 | FALSE |
| GLUD2 | 3972 | 2.072886 | 0.038183 | 0.1039737 | FALSE |
| TIMP3 | 13623 | 2.072645 | 0.038205 | 0.1040167 | FALSE |
| RGS1 | 11635 | 2.072376 | 0.03823 | 0.1040306 | FALSE |
| CLIC5 | 5123 | 2.070479 | 0.038408 | 0.1044217 | FALSE |
| MYRIP | 10537 | 2.069722 | 0.038478 | 0.1045649 | FALSE |
| ABCG8 | 4268 | 2.069124 | 0.038534 | 0.104694 | FALSE |
| CINP | 1673 | 2.068995 | 0.038547 | 0.1047087 | FALSE |
| GDPD2 | 13310 | 2.068358 | 0.038606 | 0.1048529 | FALSE |
| DENND6B | 3345 | 2.067084 | 0.038726 | 0.1050872 | FALSE |
| CLU | 11529 | 2.065693 | 0.038857 | 0.1054188 | FALSE |
| PRR7 | 5846 | 2.065646 | 0.038862 | 0.1054188 | FALSE |
| PPIAP30 | 8625 | 2.065528 | 0.038873 | 0.1054307 | FALSE |
| COPRS | 11334 | 2.065265 | 0.038898 | 0.1054799 | FALSE |
| ETFDH | 1755 | 2.064109 | 0.039007 | 0.1057216 | FALSE |
| PI16 | 1298 | 2.063767 | 0.03904 | 0.105748 | FALSE |
| CPNE2 | 12480 | 2.062836 | 0.039128 | 0.1059389 | FALSE |
| COPE | 13062 | 2.062455 | 0.039164 | 0.1059819 | FALSE |
| RPL21P44 | 7334 | 2.061842 | 0.039223 | 0.1061031 | FALSE |
| TP53BP2 | 13927 | 2.059923 | 0.039406 | 0.1065616 | FALSE |
| NFAM1 | 2011 | 2.058921 | 0.039502 | 0.1067709 | FALSE |
| SNHG10 | 1765 | 2.058829 | 0.039511 | 0.1067709 | FALSE |
| ARHGAP20 | 8347 | 2.058704 | 0.039523 | 0.1067848 | FALSE |
| HHIPL1 | 7246 | 2.058448 | 0.039547 | 0.1068327 | FALSE |
| C6orf118 | 6251 | 2.058364 | 0.039555 | 0.106836 | FALSE |
| MAPK1 | 11309 | 2.058143 | 0.039576 | 0.1068708 | FALSE |
| TEX44 | 10177 | 2.057884 | 0.039601 | 0.1068865 | FALSE |
| DCN | 2355 | 2.057684 | 0.03962 | 0.1069199 | FALSE |
| ALDH1L1 | 14471 | 2.056634 | 0.039721 | 0.1071475 | FALSE |
| ATP8A1 | 11414 | 2.055574 | 0.039824 | 0.107355 | FALSE |
| GPRC5C | 10680 | 2.054157 | 0.03996 | 0.1076331 | FALSE |
| AHNAK | 8939 | 2.05372 | 0.040003 | 0.1077285 | FALSE |
| EBLN3P | 11936 | 2.05318 | 0.040055 | 0.1078508 | FALSE |
| PA2G4 | 7938 | 2.053032 | 0.040069 | 0.1078523 | FALSE |
| TRUB1 | 8589 | 2.052184 | 0.040152 | 0.1079995 | FALSE |
| KCNQ1DN | 13464 | 2.05132 | 0.040236 | 0.1082068 | FALSE |
| OSR2 | 13202 | 2.051117 | 0.040256 | 0.1082413 | FALSE |
| TRIM68 | 4021 | 2.050328 | 0.040332 | 0.1083735 | FALSE |
| GIMAP4 | 1430 | 2.05021 | 0.040344 | 0.1083858 | FALSE |
| AFF2 | 1891 | 2.049351 | 0.040428 | 0.1085551 | FALSE |
| UBR4 | 9768 | 2.048606 | 0.040501 | 0.1087321 | FALSE |
| ELFN2 | 6833 | 2.048278 | 0.040533 | 0.1087809 | FALSE |
| APCDD1 | 4526 | 2.048174 | 0.040543 | 0.1087896 | FALSE |
| WASF2 | 1539 | 2.047184 | 0.04064 | 0.1090126 | FALSE |
| FRRS1L | 1170 | 2.04598 | 0.040758 | 0.1092737 | FALSE |
| BRMS1L | 15600 | 2.045425 | 0.040813 | 0.1094015 | FALSE |
| LOC91548 | 14236 | 2.045227 | 0.040832 | 0.109435 | FALSE |
| CCL2 | 3788 | 2.043788 | 0.040974 | 0.1097591 | FALSE |
| CAPNS2 | 2996 | 2.043702 | 0.040983 | 0.1097631 | FALSE |
| TOGARAM2 | 3125 | 2.042504 | 0.041102 | 0.1100053 | FALSE |
| MEDAG | 13475 | 2.041474 | 0.041204 | 0.1102221 | FALSE |
| CLSTN2 | 5474 | 2.041124 | 0.041239 | 0.11028 | FALSE |
| SLC22A3 | 8049 | 2.041114 | 0.041239 | 0.11028 | FALSE |
| RTN4RL1 | 5230 | 2.040938 | 0.041257 | 0.1103079 | FALSE |
| MGEA5 | 5853 | 2.039638 | 0.041386 | 0.1105972 | FALSE |
| SEC23B | 14745 | 2.039062 | 0.041444 | 0.1107128 | FALSE |
| WAC-AS1 | 9277 | 2.03888 | 0.041462 | 0.1107235 | FALSE |
| GPAA1 | 3846 | 2.038782 | 0.041472 | 0.1107307 | FALSE |
| ZNF517 | 11721 | 2.03868 | 0.041482 | 0.110739 | FALSE |
| TCTEX1D2 | 2622 | 2.038603 | 0.04149 | 0.1107406 | FALSE |
| ACTA2 | 13483 | 2.037378 | 0.041612 | 0.1109975 | FALSE |
| GNG12 | 7785 | 2.037054 | 0.041645 | 0.1110405 | FALSE |
| ANKRD33 | 3775 | 2.03674 | 0.041676 | 0.1110773 | FALSE |
| VPS37C | 181 | 2.036633 | 0.041687 | 0.1110773 | FALSE |
| RPS26 | 4073 | 2.036402 | 0.04171 | 0.1110961 | FALSE |
| AMN1 | 6825 | 2.034584 | 0.041893 | 0.1114929 | FALSE |
| LOC101929066 | 1342 | 2.03398 | 0.041954 | 0.1116169 | FALSE |
| TRIM10 | 7992 | 2.033693 | 0.041983 | 0.1116559 | FALSE |
| BEST4 | 9247 | 2.03349 | 0.042003 | 0.1116914 | FALSE |
| MARCKSL1 | 3294 | 2.033324 | 0.04202 | 0.1117169 | FALSE |
| DUSP5P1 | 11300 | 2.032337 | 0.04212 | 0.1119441 | FALSE |
| TUBB4A | 12611 | 2.032104 | 0.042143 | 0.1119686 | FALSE |
| FSIP1 | 1848 | 2.030755 | 0.04228 | 0.1122653 | FALSE |
| C14orf132 | 7510 | 2.030719 | 0.042284 | 0.1122653 | FALSE |
| TTC3 | 547 | 2.03057 | 0.042299 | 0.1122864 | FALSE |
| NUBPL | 2679 | 2.03032 | 0.042324 | 0.1123347 | FALSE |
| IFT74 | 502 | 2.029914 | 0.042365 | 0.112387 | FALSE |
| SDK2 | 7858 | 2.029007 | 0.042458 | 0.1125554 | FALSE |
| SUFU | 3051 | 2.028857 | 0.042473 | 0.1125768 | FALSE |
| EDA | 1803 | 2.02799 | 0.042561 | 0.1127921 | FALSE |
| POLM | 10286 | 2.027911 | 0.042569 | 0.1127943 | FALSE |
| CCDC70 | 5452 | 2.027763 | 0.042584 | 0.1128115 | FALSE |
| SLC7A10 | 12907 | 2.027706 | 0.04259 | 0.1128115 | FALSE |
| SPTY2D1 | 5238 | 2.025423 | 0.042824 | 0.1133245 | FALSE |
| P4HA2 | 2117 | 2.025316 | 0.042835 | 0.1133245 | FALSE |
| CTXN1 | 2329 | 2.025296 | 0.042837 | 0.1133245 | FALSE |
| UBC | 10269 | 2.024976 | 0.04287 | 0.11336 | FALSE |
| CAPS | 11340 | 2.022237 | 0.043152 | 0.1140286 | FALSE |
| ANP32E | 6729 | 2.022042 | 0.043172 | 0.1140501 | FALSE |
| PAOX | 13865 | 2.021837 | 0.043193 | 0.1140668 | FALSE |
| NDUFB10 | 8661 | 2.021792 | 0.043198 | 0.1140668 | FALSE |
| PROP1 | 1898 | 2.021744 | 0.043203 | 0.1140668 | FALSE |
| IPO13 | 6230 | 2.021301 | 0.043249 | 0.1141478 | FALSE |
| CTNS | 198 | 2.021201 | 0.043259 | 0.1141478 | FALSE |
| HMGN1 | 437 | 2.019902 | 0.043394 | 0.1144351 | FALSE |
| COX6A1 | 11259 | 2.019513 | 0.043434 | 0.1145223 | FALSE |
| OR13A1 | 11047 | 2.019172 | 0.043469 | 0.1145963 | FALSE |
| CLEC4G | 11875 | 2.018891 | 0.043499 | 0.1146238 | FALSE |
| AOAH | 10079 | 2.017907 | 0.043601 | 0.1148464 | FALSE |
| TUBA3FP | 3074 | 2.01745 | 0.043649 | 0.1149332 | FALSE |
| DNPEP | 6050 | 2.017104 | 0.043685 | 0.1150088 | FALSE |
| HADHB | 10411 | 2.01673 | 0.043724 | 0.1150922 | FALSE |
| RFXANK | 14565 | 2.016416 | 0.043756 | 0.1151591 | FALSE |
| PSMB5 | 1083 | 2.015483 | 0.043854 | 0.1153771 | FALSE |
| BLVRB | 10413 | 2.015217 | 0.043882 | 0.1154309 | FALSE |
| SIGMAR1 | 7769 | 2.014918 | 0.043913 | 0.1154939 | FALSE |
| PSEN2 | 60 | 2.013383 | 0.044074 | 0.1158591 | FALSE |
| PGAM2 | 13648 | 2.012383 | 0.04418 | 0.1161162 | FALSE |
| MYOM1 | 10498 | 2.011867 | 0.044234 | 0.1162006 | FALSE |
| GADD45GIP1 | 302 | 2.011609 | 0.044261 | 0.1162432 | FALSE |
| HIP1R | 8052 | 2.011572 | 0.044265 | 0.1162432 | FALSE |
| SF3A2 | 11428 | 2.010947 | 0.044331 | 0.1163969 | FALSE |
| CACNA1F | 4001 | 2.010805 | 0.044346 | 0.1164044 | FALSE |
| SMKR1 | 10891 | 2.010779 | 0.044349 | 0.1164044 | FALSE |
| OS9 | 12858 | 2.01029 | 0.044401 | 0.116501 | FALSE |
| FZD7 | 10322 | 2.009998 | 0.044431 | 0.1165625 | FALSE |
| CNTNAP3B | 903 | 2.007631 | 0.044683 | 0.117123 | FALSE |
| NINL | 1526 | 2.007307 | 0.044717 | 0.1171597 | FALSE |
| PPIAL4A | 6985 | 2.007055 | 0.044744 | 0.1171916 | FALSE |
| LIMK2 | 9159 | 2.006451 | 0.044808 | 0.1173147 | FALSE |
| NRXN2 | 14377 | 2.006325 | 0.044822 | 0.1173302 | FALSE |
| RPS24 | 1212 | 2.006017 | 0.044854 | 0.1173965 | FALSE |
| ZNF217 | 10261 | 2.005475 | 0.044912 | 0.1174889 | FALSE |
| REEP1 | 15307 | 2.005155 | 0.044946 | 0.117539 | FALSE |
| GPR153 | 8581 | 2.004902 | 0.044974 | 0.1175883 | FALSE |
| MAPK8IP1 | 4208 | 2.004226 | 0.045046 | 0.1177201 | FALSE |
| OARD1 | 4836 | 2.003915 | 0.045079 | 0.1177678 | FALSE |
| WFIKKN2 | 8954 | 2.003666 | 0.045106 | 0.1178178 | FALSE |
| CD44 | 15623 | 2.003224 | 0.045153 | 0.1179213 | FALSE |
| PRM2 | 8606 | 2.003156 | 0.045161 | 0.1179213 | FALSE |
| APBA1 | 7170 | 2.002985 | 0.045179 | 0.1179495 | FALSE |
| NECTIN3 | 9621 | 2.002128 | 0.045271 | 0.1181504 | FALSE |
| RAB18 | 10181 | 2.000414 | 0.045456 | 0.1185135 | FALSE |
| MS4A7 | 4385 | 1.999723 | 0.04553 | 0.1186684 | FALSE |
| CCDC74B | 6617 | 1.999297 | 0.045576 | 0.1187687 | FALSE |
| DNAJC3-AS1 | 9887 | 1.998609 | 0.045651 | 0.118932 | FALSE |
| UAP1 | 13991 | 1.998577 | 0.045654 | 0.118932 | FALSE |
| KANSL1-AS1 | 575 | 1.99791 | 0.045726 | 0.1190514 | FALSE |
| GPM6B | 8204 | 1.997874 | 0.04573 | 0.1190514 | FALSE |
| GABRG1 | 15499 | 1.997721 | 0.045747 | 0.1190745 | FALSE |
| DCTN3 | 8925 | 1.997476 | 0.045773 | 0.1191239 | FALSE |
| PSPH | 11598 | 1.99698 | 0.045827 | 0.11923 | FALSE |
| EPB41L4B | 6062 | 1.994192 | 0.046131 | 0.1198749 | FALSE |
| MRPL37 | 12881 | 1.994032 | 0.046149 | 0.1198939 | FALSE |
| NME3 | 5272 | 1.99397 | 0.046155 | 0.1198939 | FALSE |
| SELENON | 7732 | 1.993815 | 0.046172 | 0.1198939 | FALSE |
| S100B | 3418 | 1.993602 | 0.046196 | 0.1199228 | FALSE |
| ZMYND12 | 10463 | 1.993283 | 0.04623 | 0.1199935 | FALSE |
| RNF11 | 6891 | 1.992503 | 0.046316 | 0.1201656 | FALSE |
| ARVCF | 1404 | 1.992415 | 0.046326 | 0.1201656 | FALSE |
| FAM32A | 9998 | 1.992226 | 0.046346 | 0.1201809 | FALSE |
| SELENOS | 14099 | 1.992203 | 0.046349 | 0.1201809 | FALSE |
| LOC102723968 | 11496 | 1.992079 | 0.046362 | 0.1201914 | FALSE |
| HMOX1 | 3048 | 1.992026 | 0.046368 | 0.1201914 | FALSE |
| UBE2MP1 | 6677 | 1.991177 | 0.046461 | 0.1203674 | FALSE |
| SPTSSA | 12731 | 1.991173 | 0.046462 | 0.1203674 | FALSE |
| RAB11A | 4349 | 1.989425 | 0.046654 | 0.1207928 | FALSE |
| LOC100130331 | 5518 | 1.98931 | 0.046667 | 0.1208056 | FALSE |
| ERLIN2 | 2665 | 1.987134 | 0.046908 | 0.1213078 | FALSE |
| CORT | 4198 | 1.986923 | 0.046931 | 0.1213482 | FALSE |
| MTHFD1L | 9681 | 1.986672 | 0.046959 | 0.1214001 | FALSE |
| GABRA2 | 4962 | 1.986404 | 0.046988 | 0.1214568 | FALSE |
| GRINA | 15589 | 1.986051 | 0.047028 | 0.121538 | FALSE |
| LINC01315 | 9033 | 1.985089 | 0.047135 | 0.1217807 | FALSE |
| MB21D2 | 11545 | 1.983596 | 0.047301 | 0.1221726 | FALSE |
| C10orf82 | 12775 | 1.983564 | 0.047304 | 0.1221726 | FALSE |
| EXT1 | 6300 | 1.983341 | 0.047329 | 0.1222126 | FALSE |
| CHPF2 | 9716 | 1.982933 | 0.047375 | 0.1222599 | FALSE |
| DYNC1I1 | 5248 | 1.982911 | 0.047377 | 0.1222599 | FALSE |
| PRDX5 | 1795 | 1.982713 | 0.047399 | 0.1222766 | FALSE |
| CCT3 | 5024 | 1.982583 | 0.047414 | 0.1222769 | FALSE |
| ITGB5 | 7009 | 1.982572 | 0.047415 | 0.1222769 | FALSE |
| PXMP4 | 2349 | 1.982293 | 0.047446 | 0.1223312 | FALSE |
| SARNP | 4633 | 1.981724 | 0.04751 | 0.122461 | FALSE |
| MDFIC | 7545 | 1.981546 | 0.04753 | 0.1224922 | FALSE |
| SPSB4 | 11079 | 1.981035 | 0.047587 | 0.1225847 | FALSE |
| FGD2 | 5643 | 1.980598 | 0.047636 | 0.1226725 | FALSE |
| UBE2M | 1801 | 1.980572 | 0.047639 | 0.1226725 | FALSE |
| FLOT2 | 111 | 1.980406 | 0.047658 | 0.1226801 | FALSE |
| CTR9 | 14350 | 1.979638 | 0.047744 | 0.1228754 | FALSE |
| LIMD2 | 14037 | 1.977758 | 0.047956 | 0.1233662 | FALSE |
| PHLPP2 | 10922 | 1.977567 | 0.047978 | 0.1234014 | FALSE |
| RPL18 | 7481 | 1.976173 | 0.048135 | 0.1237457 | FALSE |
| 4-Mar | 12870 | 1.975877 | 0.048169 | 0.123781 | FALSE |
| ZNF93 | 11108 | 1.975866 | 0.04817 | 0.123781 | FALSE |
| ZMYM3 | 3853 | 1.975842 | 0.048173 | 0.123781 | FALSE |
| ELOC | 1893 | 1.975676 | 0.048191 | 0.123809 | FALSE |
| PGAM1P5 | 2384 | 1.975546 | 0.048206 | 0.123823 | FALSE |
| TTN | 6775 | 1.975471 | 0.048215 | 0.123823 | FALSE |
| RPL9 | 12236 | 1.975418 | 0.048221 | 0.123823 | FALSE |
| CREB3L1 | 10484 | 1.974724 | 0.048299 | 0.1239845 | FALSE |
| RELN | 1522 | 1.974533 | 0.048321 | 0.1240198 | FALSE |
| PDGFRL | 8113 | 1.974197 | 0.048359 | 0.1240815 | FALSE |
| DNAJC15 | 13440 | 1.974182 | 0.048361 | 0.1240815 | FALSE |
| C1QC | 7932 | 1.97378 | 0.048407 | 0.1241645 | FALSE |
| FOXRED2 | 3355 | 1.973638 | 0.048423 | 0.1241645 | FALSE |
| ALDH2 | 5211 | 1.973618 | 0.048425 | 0.1241645 | FALSE |
| CAP1 | 3476 | 1.972957 | 0.0485 | 0.1243371 | FALSE |
| RUNX1T1 | 4392 | 1.971837 | 0.048628 | 0.1246033 | FALSE |
| OR14I1 | 12329 | 1.971837 | 0.048628 | 0.1246033 | FALSE |
| PREB | 11856 | 1.971676 | 0.048647 | 0.12463 | FALSE |
| DLEU7 | 117 | 1.971559 | 0.04866 | 0.1246375 | FALSE |
| NWD1 | 11991 | 1.970647 | 0.048764 | 0.1248126 | FALSE |
| RITA1 | 2740 | 1.970564 | 0.048774 | 0.1248126 | FALSE |
| MGC16275 | 14049 | 1.969909 | 0.048849 | 0.1249433 | FALSE |
| C1orf53 | 2890 | 1.969452 | 0.048901 | 0.1250364 | FALSE |
| NHLRC1 | 14383 | 1.968324 | 0.049031 | 0.1253267 | FALSE |
| MRPL55 | 14550 | 1.967414 | 0.049136 | 0.1255328 | FALSE |
| LOC101060091 | 1109 | 1.967333 | 0.049145 | 0.1255361 | FALSE |
| OR2A14 | 11330 | 1.966446 | 0.049247 | 0.1257357 | FALSE |
| AK8 | 9550 | 1.966307 | 0.049263 | 0.1257357 | FALSE |
| PRMT1 | 14258 | 1.965965 | 0.049303 | 0.1257784 | FALSE |
| LSAMP | 8210 | 1.965953 | 0.049304 | 0.1257784 | FALSE |
| SDC3 | 14147 | 1.965845 | 0.049317 | 0.1257897 | FALSE |
| TIMM50 | 2953 | 1.96519 | 0.049392 | 0.1259623 | FALSE |
| DHRSX | 8618 | 1.964952 | 0.04942 | 0.1259812 | FALSE |
| SLC25A48 | 1617 | 1.964471 | 0.049475 | 0.1260923 | FALSE |
| SFN | 11528 | 1.96398 | 0.049532 | 0.1262168 | FALSE |
| SLC39A6 | 7365 | 1.963772 | 0.049557 | 0.1262577 | FALSE |
| TSNARE1 | 11446 | 1.963691 | 0.049566 | 0.1262611 | FALSE |
| HEY2 | 8710 | 1.963312 | 0.04961 | 0.126332 | FALSE |
| HARS2 | 9892 | 1.962532 | 0.049701 | 0.1265216 | FALSE |
| RPS29 | 12460 | 1.962231 | 0.049736 | 0.1265901 | FALSE |
| WSCD1 | 3973 | 1.961178 | 0.049858 | 0.1268817 | FALSE |
| PSMA7 | 11481 | 1.960726 | 0.049911 | 0.1269745 | FALSE |
| FAM177A1 | 9196 | 1.960418 | 0.049947 | 0.1269857 | FALSE |
| LOC100507487 | 4436 | 1.960416 | 0.049947 | 0.1269857 | FALSE |
| C6orf99 | 12796 | 1.960271 | 0.049964 | 0.1269857 | FALSE |
| EPB41L4A | 1073 | 1.960038 | 0.049991 | 0.1270151 | FALSE |
| HIST1H2BM | 1971 | 1.959987 | 0.049997 | 0.1270151 | FALSE |
| DNAH7 | 14556 | 1.959894 | 0.050008 | 0.1270151 | FALSE |
| LIME1 | 9380 | 1.959451 | 0.05006 | 0.127126 | FALSE |
| PCDHGA12 | 6675 | 1.95886 | 0.050129 | 0.1272604 | FALSE |
| GAMT | 6615 | 1.958464 | 0.050176 | 0.1273467 | FALSE |
| PACS1 | 7428 | 1.958431 | 0.050179 | 0.1273467 | FALSE |
| ECI2 | 10576 | 1.957189 | 0.050325 | 0.1276545 | FALSE |
| NR1H3 | 7488 | 1.957079 | 0.050338 | 0.1276666 | FALSE |
| SUPT4H1 | 14273 | 1.956728 | 0.050379 | 0.1277505 | FALSE |
| TFG | 5075 | 1.956614 | 0.050393 | 0.1277638 | FALSE |
| ESPL1 | 1983 | 1.956282 | 0.050432 | 0.1278422 | FALSE |
| TAMM41 | 5678 | 1.955481 | 0.050526 | 0.1280606 | FALSE |
| DTYMK | 5889 | 1.955177 | 0.050562 | 0.1281308 | FALSE |
| ATIC | 8301 | 1.954981 | 0.050585 | 0.1281495 | FALSE |
| ARHGEF15 | 1563 | 1.954836 | 0.050602 | 0.1281495 | FALSE |
| TMEM65 | 14451 | 1.954784 | 0.050609 | 0.1281495 | FALSE |
| PLCXD2 | 7719 | 1.95466 | 0.050623 | 0.1281608 | FALSE |
| PCP2 | 6358 | 1.953902 | 0.050713 | 0.1283285 | FALSE |
| C21orf2 | 4161 | 1.953891 | 0.050714 | 0.1283285 | FALSE |
| DOCK7 | 13214 | 1.953582 | 0.050751 | 0.1284003 | FALSE |
| ADAMTSL2 | 5096 | 1.953024 | 0.050817 | 0.1285003 | FALSE |
| KCNA4 | 11973 | 1.952901 | 0.050831 | 0.1285003 | FALSE |
| LRIG1 | 11060 | 1.952407 | 0.05089 | 0.1286068 | FALSE |
| EPHX1 | 439 | 1.95162 | 0.050983 | 0.1288013 | FALSE |
| CRSP8P | 13069 | 1.949821 | 0.051197 | 0.1292204 | FALSE |
| CD7 | 6932 | 1.949806 | 0.051199 | 0.1292204 | FALSE |
| NDUFB1 | 13915 | 1.949671 | 0.051215 | 0.1292204 | FALSE |
| UQCRQ | 8344 | 1.949127 | 0.05128 | 0.1293633 | FALSE |
| CSF3R | 6762 | 1.948022 | 0.051412 | 0.1295919 | FALSE |
| TRAF4 | 4213 | 1.947728 | 0.051448 | 0.1296388 | FALSE |
| BDH2 | 11311 | 1.947617 | 0.051461 | 0.1296514 | FALSE |
| SNCG | 214 | 1.946917 | 0.051545 | 0.1298209 | FALSE |
| RPL23AP82 | 7149 | 1.946419 | 0.051604 | 0.1299504 | FALSE |
| TMEM95 | 751 | 1.946262 | 0.051623 | 0.1299769 | FALSE |
| LCN12 | 10162 | 1.945996 | 0.051655 | 0.1300364 | FALSE |
| COL8A1 | 2082 | 1.945673 | 0.051694 | 0.1301132 | FALSE |
| PIGQ | 13164 | 1.945049 | 0.051769 | 0.1302752 | FALSE |
| SMIM30 | 12667 | 1.944999 | 0.051775 | 0.1302752 | FALSE |
| KRT17 | 1738 | 1.944867 | 0.051791 | 0.1302943 | FALSE |
| MAFK | 6578 | 1.944473 | 0.051838 | 0.1303728 | FALSE |
| PARD3 | 12528 | 1.94362 | 0.051941 | 0.1305673 | FALSE |
| SEC24D | 7265 | 1.943161 | 0.051997 | 0.1306661 | FALSE |
| ARFIP1 | 5435 | 1.941892 | 0.05215 | 0.1309871 | FALSE |
| POFUT2 | 3027 | 1.941696 | 0.052174 | 0.1310046 | FALSE |
| TRMT12 | 11975 | 1.940738 | 0.05229 | 0.1312541 | FALSE |
| LRRC7 | 7416 | 1.940603 | 0.052306 | 0.1312742 | FALSE |
| GGT8P | 12651 | 1.940531 | 0.052315 | 0.131275 | FALSE |
| RFX2 | 6051 | 1.939978 | 0.052382 | 0.1314014 | FALSE |
| PRSS1 | 10462 | 1.939801 | 0.052404 | 0.1314343 | FALSE |
| PPP4R4 | 10166 | 1.939363 | 0.052457 | 0.1315257 | FALSE |
| CALU | 1557 | 1.939013 | 0.0525 | 0.1315692 | FALSE |
| MFSD2B | 8200 | 1.938705 | 0.052537 | 0.1315941 | FALSE |
| IER5 | 10671 | 1.93869 | 0.052539 | 0.1315941 | FALSE |
| FBXL2 | 1400 | 1.938655 | 0.052543 | 0.1315941 | FALSE |
| IL15 | 4921 | 1.938364 | 0.052579 | 0.1316407 | FALSE |
| TCEAL1 | 2774 | 1.937666 | 0.052664 | 0.1318273 | FALSE |
| HMOX2 | 5719 | 1.937615 | 0.05267 | 0.1318273 | FALSE |
| ROBO3 | 14656 | 1.937515 | 0.052682 | 0.1318368 | FALSE |
| VPS29 | 9895 | 1.937398 | 0.052697 | 0.1318514 | FALSE |
| GARNL3 | 14596 | 1.936074 | 0.052859 | 0.132172 | FALSE |
| ITGB4 | 2748 | 1.93576 | 0.052897 | 0.1322179 | FALSE |
| LOC100049716 | 8918 | 1.935566 | 0.052921 | 0.1322218 | FALSE |
| HSP90AA1 | 4720 | 1.934983 | 0.052992 | 0.1323695 | FALSE |
| POLR3K | 8634 | 1.9342 | 0.053089 | 0.132556 | FALSE |
| HSPB1 | 10562 | 1.933737 | 0.053145 | 0.1326346 | FALSE |
| ZBED6CL | 4366 | 1.933634 | 0.053158 | 0.1326368 | FALSE |
| VKORC1L1 | 13610 | 1.933592 | 0.053163 | 0.1326368 | FALSE |
| USP51 | 7236 | 1.93252 | 0.053295 | 0.1329026 | FALSE |
| ZNF649 | 1477 | 1.93108 | 0.053473 | 0.1333034 | FALSE |
| ALB | 7516 | 1.930633 | 0.053528 | 0.1333562 | FALSE |
| FBLN5 | 2933 | 1.929743 | 0.053639 | 0.1336096 | FALSE |
| FAM138E | 11910 | 1.9294 | 0.053681 | 0.1336942 | FALSE |
| MAST3 | 9132 | 1.929199 | 0.053706 | 0.133735 | FALSE |
| APEH | 13600 | 1.928232 | 0.053826 | 0.1339914 | FALSE |
| ZNF733P | 6178 | 1.927565 | 0.053909 | 0.1341472 | FALSE |
| MYBPC2 | 3555 | 1.926818 | 0.054002 | 0.1343441 | FALSE |
| HS2ST1 | 4131 | 1.925826 | 0.054126 | 0.1346092 | FALSE |
| MSMO1 | 4360 | 1.925505 | 0.054166 | 0.1346661 | FALSE |
| UBE2R2 | 3354 | 1.925343 | 0.054186 | 0.134695 | FALSE |
| PPP1R8 | 9535 | 1.92516 | 0.054209 | 0.1347305 | FALSE |
| EPN1 | 10632 | 1.924973 | 0.054233 | 0.1347672 | FALSE |
| YWHAEP1 | 10390 | 1.92478 | 0.054257 | 0.1347974 | FALSE |
| BTBD1 | 12355 | 1.924738 | 0.054262 | 0.1347974 | FALSE |
| NDUFB2-AS1 | 13685 | 1.924648 | 0.054273 | 0.134804 | FALSE |
| CA4 | 12455 | 1.923713 | 0.054391 | 0.1350263 | FALSE |
| GOLGA3 | 10490 | 1.92356 | 0.05441 | 0.1350354 | FALSE |
| TMEM61 | 6142 | 1.923031 | 0.054476 | 0.1351572 | FALSE |
| HINT1 | 1129 | 1.922343 | 0.054563 | 0.1353288 | FALSE |
| ILF2 | 2133 | 1.921552 | 0.054662 | 0.1354866 | FALSE |
| BBS4 | 7302 | 1.921493 | 0.05467 | 0.1354866 | FALSE |
| ROMO1 | 10088 | 1.921299 | 0.054694 | 0.1355042 | FALSE |
| ENGASE | 6972 | 1.92077 | 0.054761 | 0.1356172 | FALSE |
| LBH | 9184 | 1.920098 | 0.054846 | 0.135772 | FALSE |
| LITAF | 333 | 1.91922 | 0.054956 | 0.1359391 | FALSE |
| ZNRF2 | 9095 | 1.918897 | 0.054997 | 0.1360028 | FALSE |
| PRELP | 2558 | 1.918879 | 0.055 | 0.1360028 | FALSE |
| LINC01664 | 13355 | 1.918773 | 0.055013 | 0.1360144 | FALSE |
| APMAP | 12910 | 1.917168 | 0.055217 | 0.1364745 | FALSE |
| TOMM20 | 2482 | 1.916958 | 0.055243 | 0.1364972 | FALSE |
| ARMCX2 | 13256 | 1.916386 | 0.055316 | 0.1366551 | FALSE |
| RPL34-AS1 | 326 | 1.91568 | 0.055406 | 0.1368124 | FALSE |
| PLA2G7 | 3011 | 1.915402 | 0.055441 | 0.1368783 | FALSE |
| TUBA4B | 2550 | 1.915145 | 0.055474 | 0.1369375 | FALSE |
| NDUFS5 | 739 | 1.914649 | 0.055537 | 0.137072 | FALSE |
| OR2W3 | 4027 | 1.914074 | 0.055611 | 0.1372018 | FALSE |
| TMEM182 | 5667 | 1.914066 | 0.055612 | 0.1372018 | FALSE |
| SLAMF7 | 5571 | 1.91357 | 0.055675 | 0.1373039 | FALSE |
| MYL6B | 220 | 1.913335 | 0.055705 | 0.1373563 | FALSE |
| MORN4 | 1512 | 1.91326 | 0.055715 | 0.1373583 | FALSE |
| MYCNOS | 9011 | 1.912502 | 0.055812 | 0.1375759 | FALSE |
| SOX2 | 8414 | 1.912246 | 0.055845 | 0.1376346 | FALSE |
| UBB | 8417 | 1.910076 | 0.056123 | 0.138235 | FALSE |
| DNPH1 | 1861 | 1.909979 | 0.056136 | 0.138244 | FALSE |
| RAB33A | 12367 | 1.909341 | 0.056218 | 0.1384028 | FALSE |
| RPL8 | 11534 | 1.908167 | 0.05637 | 0.1387321 | FALSE |
| TRAPPC9 | 11942 | 1.907649 | 0.056437 | 0.1388532 | FALSE |
| MAP7D1 | 10947 | 1.905997 | 0.056651 | 0.1393139 | FALSE |
| RNF165 | 1247 | 1.905741 | 0.056684 | 0.1393737 | FALSE |
| UBQLN2 | 11674 | 1.905613 | 0.0567 | 0.1393927 | FALSE |
| EXOSC7 | 8225 | 1.905132 | 0.056763 | 0.1395243 | FALSE |
| TGFBR3 | 7778 | 1.904822 | 0.056803 | 0.1396014 | FALSE |
| ARHGAP31 | 12681 | 1.903647 | 0.056956 | 0.1399114 | FALSE |
| LRRC8B | 7893 | 1.903514 | 0.056974 | 0.139932 | FALSE |
| TAS1R1 | 2713 | 1.903376 | 0.056992 | 0.1399542 | FALSE |
| NISCH | 14737 | 1.903209 | 0.057013 | 0.1399583 | FALSE |
| RNF222 | 2457 | 1.903118 | 0.057025 | 0.1399583 | FALSE |
| NES | 4279 | 1.903072 | 0.057031 | 0.1399583 | FALSE |
| DECR2 | 7072 | 1.903046 | 0.057035 | 0.1399583 | FALSE |
| GNAI2 | 1505 | 1.901693 | 0.057211 | 0.1403176 | FALSE |
| FAM163A | 11148 | 1.90159 | 0.057225 | 0.1403286 | FALSE |
| PSME1 | 714 | 1.900889 | 0.057317 | 0.1405096 | FALSE |
| GJA8 | 8583 | 1.900811 | 0.057327 | 0.1405126 | FALSE |
| DYRK1B | 13647 | 1.900256 | 0.0574 | 0.1406316 | FALSE |
| DOCK10 | 4181 | 1.899847 | 0.057453 | 0.1407122 | FALSE |
| RUNDC3A | 14885 | 1.899594 | 0.057486 | 0.1407498 | FALSE |
| BEND3 | 3410 | 1.899315 | 0.057523 | 0.1408171 | FALSE |
| RPS7 | 5214 | 1.898228 | 0.057666 | 0.141123 | FALSE |
| PTGDS | 56 | 1.897824 | 0.057719 | 0.1411961 | FALSE |
| LEF1-AS1 | 6404 | 1.89773 | 0.057732 | 0.1411961 | FALSE |
| ULK2 | 6828 | 1.897466 | 0.057766 | 0.1412581 | FALSE |
| CPNE3 | 9205 | 1.896034 | 0.057956 | 0.1416762 | FALSE |
| NEK3 | 1282 | 1.895948 | 0.057967 | 0.1416818 | FALSE |
| TAL2 | 7439 | 1.895351 | 0.058046 | 0.1418305 | FALSE |
| SMIM10L2B-AS1 | 1753 | 1.895071 | 0.058083 | 0.141899 | FALSE |
| LRRC2 | 1749 | 1.89431 | 0.058184 | 0.142101 | FALSE |
| ZNF737 | 4363 | 1.893784 | 0.058254 | 0.1422493 | FALSE |
| PRDX2 | 9341 | 1.893547 | 0.058285 | 0.1422914 | FALSE |
| GPR156 | 7107 | 1.893354 | 0.058311 | 0.1422968 | FALSE |
| GPR155 | 14684 | 1.891869 | 0.058508 | 0.142693 | FALSE |
| NEURL1 | 12693 | 1.89003 | 0.058754 | 0.1432024 | FALSE |
| SUZ12P1 | 12993 | 1.889878 | 0.058774 | 0.1432187 | FALSE |
| NCKAP1 | 13479 | 1.889843 | 0.058779 | 0.1432187 | FALSE |
| GDF5OS | 14552 | 1.888869 | 0.058909 | 0.1434918 | FALSE |
| CDAN1 | 385 | 1.888262 | 0.058991 | 0.1436453 | FALSE |
| NDUFB7 | 13783 | 1.887808 | 0.059052 | 0.1437489 | FALSE |
| KIF1BP | 10048 | 1.886383 | 0.059243 | 0.1441257 | FALSE |
| TMEM187 | 12025 | 1.88542 | 0.059373 | 0.144374 | FALSE |
| ECHS1 | 6397 | 1.884795 | 0.059458 | 0.1445567 | FALSE |
| ENOSF1 | 8855 | 1.884466 | 0.059502 | 0.1445942 | FALSE |
| TLR9 | 12486 | 1.884458 | 0.059503 | 0.1445942 | FALSE |
| VSIG10L | 11659 | 1.884407 | 0.05951 | 0.1445942 | FALSE |
| LRRC57 | 1658 | 1.883366 | 0.059651 | 0.1448913 | FALSE |
| OCRL | 7527 | 1.883082 | 0.059689 | 0.1449623 | FALSE |
| SIX3 | 5227 | 1.881159 | 0.05995 | 0.1455058 | FALSE |
| CALB1 | 11416 | 1.88066 | 0.060018 | 0.1456254 | FALSE |
| YAP1 | 11686 | 1.880532 | 0.060036 | 0.145645 | FALSE |
| NDOR1 | 8664 | 1.879823 | 0.060132 | 0.1458018 | FALSE |
| WDFY2 | 3979 | 1.87925 | 0.06021 | 0.1459558 | FALSE |
| C1orf198 | 961 | 1.878664 | 0.06029 | 0.1461137 | FALSE |
| AGAP3 | 4126 | 1.878636 | 0.060294 | 0.1461137 | FALSE |
| RPL19P12 | 11329 | 1.878547 | 0.060306 | 0.1461184 | FALSE |
| TMEM37 | 13833 | 1.878485 | 0.060315 | 0.1461184 | FALSE |
| CCDC112 | 5445 | 1.878133 | 0.060363 | 0.1462123 | FALSE |
| FIBCD1 | 4938 | 1.877288 | 0.060479 | 0.1464471 | FALSE |
| P3H2 | 8481 | 1.875742 | 0.060691 | 0.1469171 | FALSE |
| LOC105373876 | 3288 | 1.875618 | 0.060708 | 0.1469205 | FALSE |
| TSHB | 8834 | 1.875589 | 0.060712 | 0.1469205 | FALSE |
| MRPL18 | 14131 | 1.875428 | 0.060734 | 0.1469513 | FALSE |
| RXYLT1 | 11225 | 1.875342 | 0.060746 | 0.1469572 | FALSE |
| SLC39A11 | 7034 | 1.875151 | 0.060772 | 0.1469752 | FALSE |
| TMEM101 | 1218 | 1.874217 | 0.060901 | 0.1472406 | FALSE |
| HIST1H2BD | 11478 | 1.874114 | 0.060915 | 0.1472522 | FALSE |
| COX7A2 | 2208 | 1.873289 | 0.061028 | 0.1474816 | FALSE |
| MIF-AS1 | 3566 | 1.872576 | 0.061127 | 0.1476055 | FALSE |
| TAC4 | 6840 | 1.872218 | 0.061176 | 0.1477022 | FALSE |
| PRSS3 | 4899 | 1.872133 | 0.061188 | 0.1477077 | FALSE |
| A2M | 14726 | 1.871431 | 0.061285 | 0.1479194 | FALSE |
| MOXD1 | 14772 | 1.870711 | 0.061385 | 0.1481374 | FALSE |
| GTF2IRD2 | 2486 | 1.870562 | 0.061406 | 0.1481512 | FALSE |
| PMF1 | 14119 | 1.870273 | 0.061446 | 0.1481921 | FALSE |
| C2orf73 | 11693 | 1.868929 | 0.061633 | 0.148527 | FALSE |
| ESD | 9914 | 1.868865 | 0.061642 | 0.148527 | FALSE |
| AGAP2 | 4355 | 1.868123 | 0.061745 | 0.1486843 | FALSE |
| MRGPRD | 3474 | 1.868035 | 0.061757 | 0.1486909 | FALSE |
| PPP1CA | 8932 | 1.867758 | 0.061796 | 0.148761 | FALSE |
| HNRNPR | 4224 | 1.8675 | 0.061832 | 0.1487974 | FALSE |
| UBXN8 | 2657 | 1.867131 | 0.061883 | 0.1488341 | FALSE |
| RTL10 | 12794 | 1.867044 | 0.061895 | 0.1488404 | FALSE |
| MEIS2 | 14 | 1.866026 | 0.062038 | 0.1491367 | FALSE |
| ATE1 | 13231 | 1.865833 | 0.062065 | 0.1491787 | FALSE |
| SEMA3C | 766 | 1.864679 | 0.062226 | 0.1495443 | FALSE |
| RAB24 | 8088 | 1.864337 | 0.062274 | 0.1496366 | FALSE |
| HOXB7 | 10093 | 1.863439 | 0.062401 | 0.1498936 | FALSE |
| SPRR2C | 14459 | 1.863031 | 0.062458 | 0.1499903 | FALSE |
| ORC4 | 11351 | 1.862963 | 0.062467 | 0.1499903 | FALSE |
| PSMC6 | 4035 | 1.862835 | 0.062485 | 0.1499932 | FALSE |
| CALM3 | 2488 | 1.861358 | 0.062694 | 0.1503466 | FALSE |
| TTYH3 | 7442 | 1.861349 | 0.062695 | 0.1503466 | FALSE |
| ZNF26 | 9736 | 1.860029 | 0.062881 | 0.1507642 | FALSE |
| METTL7A | 13255 | 1.858277 | 0.06313 | 0.1512698 | FALSE |
| ANXA2 | 6314 | 1.856987 | 0.063313 | 0.1516427 | FALSE |
| EIF3J | 5807 | 1.856436 | 0.063391 | 0.1518073 | FALSE |
| EFCAB11 | 8758 | 1.855995 | 0.063454 | 0.1519113 | FALSE |
| PIK3R5 | 3323 | 1.855862 | 0.063473 | 0.1519334 | FALSE |
| OR5B17 | 7560 | 1.855614 | 0.063509 | 0.1519948 | FALSE |
| PTDSS2 | 6053 | 1.854963 | 0.063602 | 0.1521706 | FALSE |
| C11orf95 | 13160 | 1.854451 | 0.063675 | 0.1523223 | FALSE |
| PARP14 | 6905 | 1.854024 | 0.063736 | 0.1524217 | FALSE |
| AMPD2 | 7377 | 1.853789 | 0.063769 | 0.1524555 | FALSE |
| SERPINA1 | 8114 | 1.852651 | 0.063932 | 0.1527786 | FALSE |
| SLCO1C1 | 11101 | 1.852635 | 0.063935 | 0.1527786 | FALSE |
| VLDLR | 14403 | 1.852606 | 0.063939 | 0.1527786 | FALSE |
| RETREG1 | 8871 | 1.852272 | 0.063987 | 0.1528584 | FALSE |
| TRIM35 | 3855 | 1.852003 | 0.064025 | 0.1529004 | FALSE |
| UPK2 | 12299 | 1.851947 | 0.064033 | 0.1529004 | FALSE |
| CCDC28A | 12316 | 1.851945 | 0.064034 | 0.1529004 | FALSE |
| HAGH | 5019 | 1.851539 | 0.064092 | 0.1530163 | FALSE |
| FCGR3A | 4939 | 1.851423 | 0.064109 | 0.1530328 | FALSE |
| RB1 | 10882 | 1.850924 | 0.06418 | 0.1531274 | FALSE |
| C19orf25 | 9871 | 1.850695 | 0.064213 | 0.1531658 | FALSE |
| TUBA1C | 10740 | 1.849722 | 0.064354 | 0.1534768 | FALSE |
| PVT1 | 5268 | 1.848981 | 0.064461 | 0.1537083 | FALSE |
| JADE2 | 1901 | 1.848506 | 0.064529 | 0.1538485 | FALSE |
| TMBIM1 | 425 | 1.847611 | 0.064659 | 0.1540787 | FALSE |
| INPP4A | 14820 | 1.847566 | 0.064665 | 0.1540787 | FALSE |
| GALR3 | 3208 | 1.847106 | 0.064732 | 0.1541904 | FALSE |
| NXPH4 | 1151 | 1.846433 | 0.064829 | 0.1543994 | FALSE |
| SEC14L4 | 9917 | 1.84599 | 0.064894 | 0.1545054 | FALSE |
| TRABD2A | 330 | 1.845608 | 0.064949 | 0.1545427 | FALSE |
| LGALS14 | 14089 | 1.84555 | 0.064958 | 0.1545427 | FALSE |
| PLEKHB1 | 4103 | 1.845543 | 0.064959 | 0.1545427 | FALSE |
| TMEM219 | 9439 | 1.84547 | 0.064969 | 0.154544 | FALSE |
| DICER1-AS1 | 8561 | 1.845308 | 0.064993 | 0.1545765 | FALSE |
| PTPN23 | 6778 | 1.844215 | 0.065152 | 0.1549077 | FALSE |
| USP27X-AS1 | 12509 | 1.844118 | 0.065166 | 0.1549178 | FALSE |
| CNDP2 | 6360 | 1.843804 | 0.065212 | 0.155003 | FALSE |
| SASH1 | 13505 | 1.842059 | 0.065467 | 0.155585 | FALSE |
| MAP7D3 | 2658 | 1.841052 | 0.065614 | 0.1558716 | FALSE |
| LMF1 | 10565 | 1.840987 | 0.065623 | 0.1558716 | FALSE |
| RUBCN | 8039 | 1.840181 | 0.065742 | 0.1560307 | FALSE |
| ZHX2 | 10496 | 1.839494 | 0.065843 | 0.1562174 | FALSE |
| PCDHGC3 | 9433 | 1.837153 | 0.066187 | 0.1569688 | FALSE |
| SBF1 | 2018 | 1.837139 | 0.066189 | 0.1569688 | FALSE |
| C9orf16 | 12369 | 1.83704 | 0.066204 | 0.1569797 | FALSE |
| GPR182 | 11081 | 1.836914 | 0.066223 | 0.157 | FALSE |
| AIMP2 | 11970 | 1.836383 | 0.066301 | 0.1571383 | FALSE |
| SYNPO2L | 12963 | 1.836015 | 0.066355 | 0.1572434 | FALSE |
| SLITRK1 | 14857 | 1.835726 | 0.066398 | 0.1573208 | FALSE |
| SPSB2 | 2060 | 1.834958 | 0.066512 | 0.1575446 | FALSE |
| AP2S1 | 10954 | 1.83484 | 0.066529 | 0.1575601 | FALSE |
| FAM151B | 13821 | 1.834183 | 0.066627 | 0.1577192 | FALSE |
| SSTR4 | 10246 | 1.834074 | 0.066643 | 0.1577265 | FALSE |
| GPR119 | 13714 | 1.834026 | 0.06665 | 0.1577265 | FALSE |
| TLCD2 | 4382 | 1.833876 | 0.066672 | 0.1577554 | FALSE |
| ATP6V0E1 | 799 | 1.833197 | 0.066773 | 0.1579462 | FALSE |
| TM7SF2 | 3759 | 1.832824 | 0.066829 | 0.1580535 | FALSE |
| PIGO | 3549 | 1.831556 | 0.067018 | 0.1584762 | FALSE |
| NUDT7 | 11902 | 1.831287 | 0.067058 | 0.1585471 | FALSE |
| OR10G2 | 5528 | 1.83114 | 0.06708 | 0.158575 | FALSE |
| CHAT | 5562 | 1.830549 | 0.067168 | 0.1587472 | FALSE |
| BTG2 | 11889 | 1.830428 | 0.067186 | 0.1587543 | FALSE |
| DLG3 | 12798 | 1.830225 | 0.067216 | 0.1588019 | FALSE |
| ZNF83 | 14761 | 1.830028 | 0.067246 | 0.1588235 | FALSE |
| SCRN3 | 3676 | 1.829129 | 0.06738 | 0.1590932 | FALSE |
| TMIGD3 | 7653 | 1.827745 | 0.067588 | 0.159535 | FALSE |
| ENOX2 | 4065 | 1.827315 | 0.067652 | 0.1596634 | FALSE |
| SLC1A2 | 8961 | 1.826951 | 0.067707 | 0.1597443 | FALSE |
| PGPEP1 | 2781 | 1.826766 | 0.067735 | 0.1597858 | FALSE |
| KRT78 | 11743 | 1.826464 | 0.06778 | 0.1598448 | FALSE |
| SNX2 | 10703 | 1.826103 | 0.067835 | 0.1599293 | FALSE |
| BLOC1S2 | 9144 | 1.82609 | 0.067837 | 0.1599293 | FALSE |
| VPS37D | 10822 | 1.825349 | 0.067948 | 0.1601443 | FALSE |
| IGSF9 | 11098 | 1.824667 | 0.068051 | 0.1603627 | FALSE |
| LFNG | 5599 | 1.824299 | 0.068107 | 0.1604368 | FALSE |
| TROAP | 6428 | 1.823606 | 0.068212 | 0.160627 | FALSE |
| PGAP2 | 13709 | 1.823579 | 0.068216 | 0.160627 | FALSE |
| AGBL3 | 8708 | 1.823517 | 0.068225 | 0.160627 | FALSE |
| HMGCLL1 | 2010 | 1.823417 | 0.06824 | 0.1606385 | FALSE |
| QKI | 15556 | 1.822337 | 0.068404 | 0.1608623 | FALSE |
| RCHY1 | 11687 | 1.822248 | 0.068417 | 0.1608623 | FALSE |
| SUMO2 | 1205 | 1.822246 | 0.068418 | 0.1608623 | FALSE |
| VSX1 | 9218 | 1.8218 | 0.068485 | 0.1609972 | FALSE |
| LINC00886 | 11516 | 1.8215 | 0.068531 | 0.1610558 | FALSE |
| EMC8 | 1556 | 1.820491 | 0.068684 | 0.1613625 | FALSE |
| SNHG16 | 14009 | 1.819465 | 0.068841 | 0.1616368 | FALSE |
| LCE1A | 1345 | 1.819414 | 0.068848 | 0.1616368 | FALSE |
| LOC105373383 | 4246 | 1.819342 | 0.068859 | 0.1616368 | FALSE |
| HIF3A | 4761 | 1.818714 | 0.068955 | 0.1618338 | FALSE |
| MAP6 | 9848 | 1.818559 | 0.068979 | 0.1618583 | FALSE |
| SSC5D | 4594 | 1.81851 | 0.068986 | 0.1618583 | FALSE |
| FOXO1 | 10489 | 1.818433 | 0.068998 | 0.1618616 | FALSE |
| RNF139 | 11302 | 1.818179 | 0.069037 | 0.1619119 | FALSE |
| NDUFA6 | 6385 | 1.818157 | 0.06904 | 0.1619119 | FALSE |
| POP4 | 12147 | 1.818027 | 0.06906 | 0.1619342 | FALSE |
| EIF3M | 14582 | 1.817839 | 0.069089 | 0.1619773 | FALSE |
| FAM49A | 3487 | 1.817649 | 0.069118 | 0.1620211 | FALSE |
| WDR49 | 12220 | 1.816722 | 0.06926 | 0.1623293 | FALSE |
| SCO2 | 4359 | 1.816328 | 0.06932 | 0.1624465 | FALSE |
| GRIK5 | 10417 | 1.815958 | 0.069377 | 0.1625551 | FALSE |
| SAT1 | 9185 | 1.815694 | 0.069417 | 0.1626257 | FALSE |
| FNDC9 | 3685 | 1.814941 | 0.069533 | 0.1628618 | FALSE |
| C16orf95 | 14187 | 1.814272 | 0.069636 | 0.1630643 | FALSE |
| INTU | 5235 | 1.813309 | 0.069784 | 0.1633627 | FALSE |
| AAED1 | 15038 | 1.812904 | 0.069847 | 0.1634599 | FALSE |
| ARL8A | 10541 | 1.81129 | 0.070096 | 0.1639944 | FALSE |
| STMN4 | 4023 | 1.811089 | 0.070127 | 0.1640426 | FALSE |
| MYCN | 2106 | 1.810657 | 0.070194 | 0.1641499 | FALSE |
| PFKL | 6751 | 1.810566 | 0.070208 | 0.1641583 | FALSE |
| IKBIP | 8853 | 1.810102 | 0.07028 | 0.1642773 | FALSE |
| PKP3 | 1791 | 1.809341 | 0.070398 | 0.1644803 | FALSE |
| MYL3 | 14778 | 1.809187 | 0.070422 | 0.1644891 | FALSE |
| HCN3 | 12674 | 1.808948 | 0.070459 | 0.1645181 | FALSE |
| KIAA0319 | 15576 | 1.808896 | 0.070467 | 0.1645181 | FALSE |
| ZNF263 | 1590 | 1.80839 | 0.070546 | 0.1646448 | FALSE |
| CCDC120 | 910 | 1.808276 | 0.070564 | 0.1646448 | FALSE |
| BEX3 | 8274 | 1.806804 | 0.070793 | 0.1651551 | FALSE |
| PCDH10 | 11621 | 1.806562 | 0.070831 | 0.165211 | FALSE |
| RAPSN | 3140 | 1.806515 | 0.070838 | 0.165211 | FALSE |
| OGFR | 13177 | 1.806418 | 0.070853 | 0.1652217 | FALSE |
| EIF5B | 7942 | 1.806203 | 0.070887 | 0.1652753 | FALSE |
| ARHGEF19 | 15506 | 1.805625 | 0.070977 | 0.1654611 | FALSE |
| LEPROT | 11109 | 1.805492 | 0.070998 | 0.1654849 | FALSE |
| MX2 | 7413 | 1.805135 | 0.071054 | 0.1655904 | FALSE |
| KIF1A | 11729 | 1.804512 | 0.071151 | 0.1657929 | FALSE |
| LHX3 | 4941 | 1.803227 | 0.071353 | 0.1662377 | FALSE |
| RNH1 | 12949 | 1.80251 | 0.071465 | 0.1664009 | FALSE |
| MDFI | 6684 | 1.801665 | 0.071598 | 0.1665863 | FALSE |
| RUSC1-AS1 | 3989 | 1.801404 | 0.071639 | 0.1666571 | FALSE |
| SERPINF1 | 12515 | 1.80015 | 0.071837 | 0.1670329 | FALSE |
| SLC25A1 | 13090 | 1.799067 | 0.072008 | 0.1673658 | FALSE |
| PHF10 | 9834 | 1.798494 | 0.072099 | 0.1675267 | FALSE |
| CYB561A3 | 8565 | 1.797834 | 0.072203 | 0.1677198 | FALSE |
| HEMK1 | 7042 | 1.797645 | 0.072233 | 0.1677558 | FALSE |
| AZIN1 | 5119 | 1.797601 | 0.07224 | 0.1677558 | FALSE |
| NR2E1 | 6052 | 1.797159 | 0.07231 | 0.1678438 | FALSE |
| NFIB | 1536 | 1.796944 | 0.072345 | 0.1678981 | FALSE |
| YBX2 | 407 | 1.796693 | 0.072384 | 0.1679408 | FALSE |
| NPY5R | 6009 | 1.79661 | 0.072398 | 0.1679464 | FALSE |
| MRPS21 | 13011 | 1.796395 | 0.072432 | 0.1680008 | FALSE |
| ADA2 | 7244 | 1.796228 | 0.072458 | 0.1680374 | FALSE |
| RALA | 11557 | 1.795785 | 0.072529 | 0.1681758 | FALSE |
| PTGIR | 12126 | 1.795666 | 0.072548 | 0.1681802 | FALSE |
| AP2M1 | 11660 | 1.794984 | 0.072656 | 0.1683581 | FALSE |
| OR2A2 | 4835 | 1.794953 | 0.072661 | 0.1683581 | FALSE |
| CDK5 | 1583 | 1.794595 | 0.072718 | 0.1684654 | FALSE |
| NAV3 | 130 | 1.794353 | 0.072757 | 0.1685298 | FALSE |
| NME1 | 14071 | 1.793019 | 0.07297 | 0.1689482 | FALSE |
| PIK3CD | 10728 | 1.792671 | 0.073026 | 0.169027 | FALSE |
| UBE2G2 | 354 | 1.792578 | 0.07304 | 0.1690364 | FALSE |
| RGL1 | 12422 | 1.792466 | 0.073058 | 0.1690529 | FALSE |
| LOC339803 | 9329 | 1.791349 | 0.073237 | 0.1693917 | FALSE |
| CXCL6 | 2359 | 1.791179 | 0.073265 | 0.1694297 | FALSE |
| ADHFE1 | 7118 | 1.790849 | 0.073318 | 0.1695271 | FALSE |
| ZNF449 | 11862 | 1.790018 | 0.073451 | 0.1697856 | FALSE |
| MEGF11 | 345 | 1.789795 | 0.073487 | 0.1698433 | FALSE |
| FAM213A | 3602 | 1.789337 | 0.073561 | 0.1699634 | FALSE |
| GFM2 | 1910 | 1.786299 | 0.074051 | 0.1709641 | FALSE |
| PIP4P1 | 12035 | 1.786247 | 0.074059 | 0.1709641 | FALSE |
| B4GALT1 | 12793 | 1.785751 | 0.07414 | 0.1710782 | FALSE |
| ZNF226 | 11643 | 1.785739 | 0.074142 | 0.1710782 | FALSE |
| DDX49 | 7055 | 1.785534 | 0.074175 | 0.1711078 | FALSE |
| LOC400710 | 12405 | 1.785472 | 0.074185 | 0.1711078 | FALSE |
| CEACAM20 | 9191 | 1.784397 | 0.074359 | 0.1714791 | FALSE |
| RPL21 | 13394 | 1.784146 | 0.0744 | 0.1715225 | FALSE |
| CCDC86 | 4762 | 1.783732 | 0.074467 | 0.1716523 | FALSE |
| CD84 | 8833 | 1.783299 | 0.074538 | 0.171764 | FALSE |
| LYPD3 | 3247 | 1.783138 | 0.074564 | 0.171799 | FALSE |
| CIP2A | 9772 | 1.78246 | 0.074674 | 0.1719774 | FALSE |
| IRF5 | 2036 | 1.780953 | 0.07492 | 0.1724958 | FALSE |
| NCOA1 | 3509 | 1.780945 | 0.074921 | 0.1724958 | FALSE |
| LCNL1 | 7038 | 1.78083 | 0.07494 | 0.1725137 | FALSE |
| RPL23AP32 | 45 | 1.780272 | 0.075031 | 0.1726474 | FALSE |
| CYB5A | 11734 | 1.779496 | 0.075158 | 0.1728634 | FALSE |
| LRFN3 | 6309 | 1.778362 | 0.075344 | 0.173189 | FALSE |
| CHRNB2 | 1167 | 1.778192 | 0.075372 | 0.173189 | FALSE |
| KRBOX4 | 6730 | 1.777691 | 0.075455 | 0.1732851 | FALSE |
| ADAP2 | 13693 | 1.777579 | 0.075473 | 0.1732851 | FALSE |
| TOR2A | 1329 | 1.777491 | 0.075487 | 0.1732887 | FALSE |
| FUK | 679 | 1.777383 | 0.075505 | 0.173304 | FALSE |
| UHRF2 | 8992 | 1.777294 | 0.07552 | 0.1733121 | FALSE |
| HLA-J | 1447 | 1.777024 | 0.075564 | 0.1733777 | FALSE |
| LOC101929122 | 7069 | 1.776536 | 0.075645 | 0.1734649 | FALSE |
| EIF1AD | 10095 | 1.77652 | 0.075647 | 0.1734649 | FALSE |
| GIPR | 7054 | 1.774977 | 0.075902 | 0.1739585 | FALSE |
| AMDHD1 | 15474 | 1.774881 | 0.075918 | 0.1739693 | FALSE |
| DKKL1 | 4303 | 1.774609 | 0.075962 | 0.1740468 | FALSE |
| TMEM220 | 9117 | 1.774232 | 0.076025 | 0.174164 | FALSE |
| FKBP10 | 13784 | 1.773596 | 0.07613 | 0.1743593 | FALSE |
| WDR7 | 11433 | 1.773011 | 0.076227 | 0.1744892 | FALSE |
| CDC14A | 9363 | 1.772786 | 0.076264 | 0.1745079 | FALSE |
| NPPC | 10647 | 1.771018 | 0.076558 | 0.1751283 | FALSE |
| SH2D6 | 15417 | 1.770759 | 0.076601 | 0.1751756 | FALSE |
| DGKH | 1011 | 1.770582 | 0.07663 | 0.1752173 | FALSE |
| NEK11 | 11231 | 1.770348 | 0.076669 | 0.1752557 | FALSE |
| ATP5IF1 | 4285 | 1.770282 | 0.07668 | 0.1752557 | FALSE |
| ZZZ3 | 12075 | 1.770178 | 0.076697 | 0.1752685 | FALSE |
| C1orf220 | 14454 | 1.770069 | 0.076716 | 0.1752844 | FALSE |
| PTPN18 | 8984 | 1.769575 | 0.076798 | 0.1754161 | FALSE |
| KRTAP20-1 | 13159 | 1.769521 | 0.076807 | 0.1754161 | FALSE |
| RDH16 | 5050 | 1.768895 | 0.076911 | 0.1756109 | FALSE |
| TMEM94 | 2113 | 1.768875 | 0.076915 | 0.1756109 | FALSE |
| RFX1 | 13901 | 1.768247 | 0.07702 | 0.1758063 | FALSE |
| NYNRIN | 8675 | 1.767629 | 0.077123 | 0.1759578 | FALSE |
| L3MBTL1 | 4684 | 1.767256 | 0.077185 | 0.1760745 | FALSE |
| SLC12A4 | 10458 | 1.767063 | 0.077218 | 0.1761163 | FALSE |
| HIST2H2BE | 3906 | 1.767012 | 0.077226 | 0.1761163 | FALSE |
| LINC00663 | 5358 | 1.766472 | 0.077317 | 0.1762938 | FALSE |
| CXCL3 | 13080 | 1.765746 | 0.077438 | 0.1764974 | FALSE |
| RUBCNL | 4770 | 1.765136 | 0.077541 | 0.1766167 | FALSE |
| NMI | 6896 | 1.765009 | 0.077562 | 0.1766242 | FALSE |
| FAM131A | 9659 | 1.764944 | 0.077573 | 0.1766242 | FALSE |
| TRIP10 | 6047 | 1.764325 | 0.077677 | 0.1768354 | FALSE |
| CSF2RA | 1488 | 1.763038 | 0.077894 | 0.1772258 | FALSE |
| IL27 | 15500 | 1.762815 | 0.077932 | 0.1772855 | FALSE |
| OTOS | 3696 | 1.761898 | 0.078087 | 0.1775861 | FALSE |
| UTP11 | 13272 | 1.761824 | 0.078099 | 0.1775887 | FALSE |
| LOC439933 | 14053 | 1.761661 | 0.078127 | 0.1776255 | FALSE |
| TMEM9B | 2446 | 1.761466 | 0.07816 | 0.1776746 | FALSE |
| 9-Sep | 9211 | 1.760841 | 0.078265 | 0.1778375 | FALSE |
| LDHC | 3569 | 1.760713 | 0.078287 | 0.1778604 | FALSE |
| IFNL2 | 13013 | 1.760647 | 0.078298 | 0.1778604 | FALSE |
| C9orf106 | 5500 | 1.760574 | 0.078311 | 0.1778626 | FALSE |
| NCAM1 | 5058 | 1.760054 | 0.078399 | 0.1780369 | FALSE |
| ACOT13 | 2999 | 1.759769 | 0.078447 | 0.1781207 | FALSE |
| SEC61A2 | 9977 | 1.759635 | 0.07847 | 0.1781207 | FALSE |
| CD58 | 9396 | 1.759505 | 0.078492 | 0.1781279 | FALSE |
| RBM3 | 7056 | 1.759482 | 0.078496 | 0.1781279 | FALSE |
| MAN2B2 | 1676 | 1.759308 | 0.078525 | 0.178169 | FALSE |
| MKL2 | 7541 | 1.759115 | 0.078558 | 0.1782175 | FALSE |
| LAMA1 | 3630 | 1.758546 | 0.078655 | 0.1783592 | FALSE |
| COL4A2 | 14777 | 1.758276 | 0.078701 | 0.1784115 | FALSE |
| CDKN2AIPNL | 8824 | 1.757951 | 0.078756 | 0.1784824 | FALSE |
| CMKLR1 | 3839 | 1.757891 | 0.078766 | 0.1784824 | FALSE |
| GRN | 14260 | 1.756478 | 0.079007 | 0.179002 | FALSE |
| CCNA1 | 4613 | 1.75452 | 0.079341 | 0.179708 | FALSE |
| ZNF818P | 10684 | 1.754355 | 0.07937 | 0.179746 | FALSE |
| ACTB | 2678 | 1.753786 | 0.079467 | 0.1798675 | FALSE |
| SMARCD1 | 4702 | 1.753712 | 0.07948 | 0.1798675 | FALSE |
| SERPINB1 | 9829 | 1.753706 | 0.079481 | 0.1798675 | FALSE |
| URB1-AS1 | 462 | 1.752249 | 0.079731 | 0.1802769 | FALSE |
| RPS15A | 6574 | 1.751524 | 0.079856 | 0.1804806 | FALSE |
| S100A4 | 13640 | 1.750972 | 0.079951 | 0.1806692 | FALSE |
| SHANK1 | 3772 | 1.750594 | 0.080016 | 0.1807903 | FALSE |
| MOV10 | 7188 | 1.750424 | 0.080045 | 0.1808089 | FALSE |
| GDAP1L1 | 6135 | 1.750412 | 0.080047 | 0.1808089 | FALSE |
| PRSS8 | 9216 | 1.74923 | 0.080251 | 0.1812436 | FALSE |
| ALDH4A1 | 6390 | 1.748524 | 0.080373 | 0.181493 | FALSE |
| PRSS3P2 | 10178 | 1.748063 | 0.080453 | 0.181647 | FALSE |
| ACTN1 | 95 | 1.747714 | 0.080514 | 0.1817572 | FALSE |
| NMB | 882 | 1.746291 | 0.08076 | 0.1822092 | FALSE |
| POM121L1P | 12215 | 1.74604 | 0.080804 | 0.1822207 | FALSE |
| SLC3A2 | 4490 | 1.745975 | 0.080815 | 0.1822207 | FALSE |
| POLR2A | 12950 | 1.745926 | 0.080824 | 0.1822207 | FALSE |
| TCEAL5 | 12602 | 1.745341 | 0.080926 | 0.1824171 | FALSE |
| PTBP3 | 13142 | 1.745291 | 0.080934 | 0.1824171 | FALSE |
| GRIK3 | 292 | 1.744935 | 0.080996 | 0.1825304 | FALSE |
| SEC61G | 3084 | 1.744707 | 0.081036 | 0.1825672 | FALSE |
| FAM187B | 8674 | 1.744442 | 0.081082 | 0.1826154 | FALSE |
| DDX20 | 2229 | 1.744383 | 0.081092 | 0.1826154 | FALSE |
| GEMIN8P4 | 14075 | 1.743754 | 0.081202 | 0.1828097 | FALSE |
| ANKDD1A | 3707 | 1.743409 | 0.081262 | 0.1828926 | FALSE |
| HADHA | 14198 | 1.743233 | 0.081293 | 0.1829279 | FALSE |
| LINC00982 | 1333 | 1.743185 | 0.081301 | 0.1829279 | FALSE |
| C7 | 1305 | 1.742747 | 0.081378 | 0.1830737 | FALSE |
| OBSCN | 6375 | 1.742323 | 0.081452 | 0.1831878 | FALSE |
| HLA-DQB2 | 15093 | 1.7422 | 0.081473 | 0.1832098 | FALSE |
| TSTA3 | 4292 | 1.74204 | 0.081501 | 0.1832464 | FALSE |
| TOX3 | 5804 | 1.741809 | 0.081542 | 0.1833109 | FALSE |
| P2RX2 | 4726 | 1.741459 | 0.081603 | 0.1834223 | FALSE |
| EIF3IP1 | 523 | 1.741134 | 0.08166 | 0.1834975 | FALSE |
| SLC39A10 | 790 | 1.740571 | 0.081759 | 0.1836665 | FALSE |
| CEP89 | 9958 | 1.740299 | 0.081807 | 0.1837473 | FALSE |
| CTNNA2 | 650 | 1.740176 | 0.081828 | 0.1837694 | FALSE |
| HTR3B | 1053 | 1.740082 | 0.081845 | 0.1837801 | FALSE |
| TRH | 8425 | 1.739954 | 0.081867 | 0.1838042 | FALSE |
| KRT85 | 5855 | 1.739471 | 0.081952 | 0.1839154 | FALSE |
| MAB21L2 | 1616 | 1.739268 | 0.081988 | 0.1839691 | FALSE |
| TPRG1L | 2888 | 1.739061 | 0.082024 | 0.1840244 | FALSE |
| YDJC | 1119 | 1.738513 | 0.08212 | 0.1841878 | FALSE |
| PSAT1 | 15331 | 1.738199 | 0.082176 | 0.1842854 | FALSE |
| CXADRP3 | 15025 | 1.737652 | 0.082272 | 0.1844582 | FALSE |
| DOPEY1 | 8170 | 1.737174 | 0.082356 | 0.1846112 | FALSE |
| TAB1 | 3798 | 1.736925 | 0.0824 | 0.1846832 | FALSE |
| RHOD | 11389 | 1.7366 | 0.082458 | 0.1847826 | FALSE |
| RAB11FIP4 | 2668 | 1.73654 | 0.082468 | 0.1847826 | FALSE |
| PGAP3 | 9922 | 1.735582 | 0.082638 | 0.185003 | FALSE |
| H2AFJ | 8340 | 1.735335 | 0.082681 | 0.1850744 | FALSE |
| PRPSAP2 | 13081 | 1.735032 | 0.082735 | 0.185168 | FALSE |
| RPL13AP6 | 14026 | 1.734452 | 0.082838 | 0.1852919 | FALSE |
| BPNT1 | 14055 | 1.73407 | 0.082906 | 0.1854123 | FALSE |
| EIF3L | 807 | 1.733881 | 0.082939 | 0.1854123 | FALSE |
| CD8A | 8952 | 1.733212 | 0.083058 | 0.1855983 | FALSE |
| RDH10 | 9293 | 1.732578 | 0.083171 | 0.185797 | FALSE |
| SLC35B1 | 3393 | 1.732388 | 0.083205 | 0.185846 | FALSE |
| MPDZ | 12605 | 1.731888 | 0.083294 | 0.1860182 | FALSE |
| KLK15 | 11096 | 1.731345 | 0.08339 | 0.1861811 | FALSE |
| RETNLB | 14057 | 1.730815 | 0.083485 | 0.1863655 | FALSE |
| SELENOI | 6742 | 1.730684 | 0.083508 | 0.186391 | FALSE |
| SFRP2 | 9939 | 1.729903 | 0.083648 | 0.1866757 | FALSE |
| RPS6KA4 | 9769 | 1.729066 | 0.083797 | 0.1869183 | FALSE |
| RETN | 7287 | 1.728894 | 0.083828 | 0.1869183 | FALSE |
| HIST1H2BE | 649 | 1.728446 | 0.083908 | 0.1870705 | FALSE |
| CHMP1B | 14433 | 1.728334 | 0.083928 | 0.1870886 | FALSE |
| PLAC8L1 | 12424 | 1.728099 | 0.08397 | 0.1871307 | FALSE |
| PINK1-AS | 1028 | 1.72721 | 0.08413 | 0.1873776 | FALSE |
| CERS5 | 6274 | 1.72699 | 0.084169 | 0.1874122 | FALSE |
| CHCHD1 | 754 | 1.72679 | 0.084205 | 0.1874655 | FALSE |
| UBA7 | 6229 | 1.724817 | 0.08456 | 0.1881286 | FALSE |
| SPNS3 | 8839 | 1.724734 | 0.084575 | 0.1881286 | FALSE |
| KRTAP22-1 | 12034 | 1.724545 | 0.08461 | 0.1881776 | FALSE |
| KIF22 | 5216 | 1.72246 | 0.084986 | 0.1889348 | FALSE |
| SMCR5 | 7039 | 1.722387 | 0.084999 | 0.1889373 | FALSE |
| MUC6 | 4012 | 1.721906 | 0.085087 | 0.1890772 | FALSE |
| UROD | 9691 | 1.721606 | 0.085141 | 0.1891442 | FALSE |
| TMEM91 | 11846 | 1.721334 | 0.08519 | 0.1892269 | FALSE |
| CFAP161 | 15298 | 1.721096 | 0.085233 | 0.189269 | FALSE |
| FAM43B | 6198 | 1.719907 | 0.085449 | 0.1896203 | FALSE |
| ABHD12B | 14185 | 1.719891 | 0.085452 | 0.1896203 | FALSE |
| ZNF418 | 1202 | 1.718751 | 0.08566 | 0.1899728 | FALSE |
| LOC145694 | 3271 | 1.7176 | 0.08587 | 0.1903303 | FALSE |
| C11orf1 | 102 | 1.717505 | 0.085887 | 0.1903417 | FALSE |
| LINC00319 | 13935 | 1.716914 | 0.085995 | 0.1905269 | FALSE |
| PLG | 285 | 1.716716 | 0.086031 | 0.1905801 | FALSE |
| ATF3 | 2044 | 1.71637 | 0.086094 | 0.1906727 | FALSE |
| EPHA5 | 4236 | 1.713084 | 0.086697 | 0.1917566 | FALSE |
| PLEKHN1 | 7498 | 1.71296 | 0.08672 | 0.1917799 | FALSE |
| KAAG1 | 5197 | 1.712779 | 0.086753 | 0.1918264 | FALSE |
| GPS2 | 12978 | 1.712467 | 0.086811 | 0.1918991 | FALSE |
| GALNT16 | 12412 | 1.711183 | 0.087047 | 0.192368 | FALSE |
| AIM2 | 459 | 1.710741 | 0.087129 | 0.1925211 | FALSE |
| USP35 | 5291 | 1.709902 | 0.087284 | 0.1927885 | FALSE |
| CDH16 | 8594 | 1.709886 | 0.087287 | 0.1927885 | FALSE |
| TMEM174 | 12456 | 1.709379 | 0.087381 | 0.1929684 | FALSE |
| ACADVL | 11401 | 1.709056 | 0.087441 | 0.1930732 | FALSE |
| HMHB1 | 14289 | 1.708899 | 0.08747 | 0.1930829 | FALSE |
| KPTN | 12302 | 1.708529 | 0.087538 | 0.193207 | FALSE |
| KLC1 | 3971 | 1.707193 | 0.087786 | 0.1935903 | FALSE |
| RGS8 | 5623 | 1.707069 | 0.087809 | 0.1936138 | FALSE |
| THBS1 | 8571 | 1.706952 | 0.087831 | 0.1936344 | FALSE |
| PPP1R9A | 12573 | 1.705617 | 0.088079 | 0.1941274 | FALSE |
| MUC12 | 10473 | 1.705471 | 0.088107 | 0.19416 | FALSE |
| EML4 | 2268 | 1.705057 | 0.088184 | 0.1943026 | FALSE |
| C1QL2 | 11716 | 1.70455 | 0.088278 | 0.1944836 | FALSE |
| HLA-DMA | 1510 | 1.704159 | 0.088351 | 0.1946171 | FALSE |
| SLC4A11 | 4090 | 1.703892 | 0.088401 | 0.1946995 | FALSE |
| KCNJ12 | 8339 | 1.703277 | 0.088516 | 0.194872 | FALSE |
| CHRNA6 | 2451 | 1.703273 | 0.088517 | 0.194872 | FALSE |
| ERC1 | 2081 | 1.70177 | 0.088798 | 0.1953267 | FALSE |
| MORN5 | 7837 | 1.701241 | 0.088898 | 0.19549 | FALSE |
| CDC16 | 10547 | 1.700764 | 0.088987 | 0.195639 | FALSE |
| CD3D | 14209 | 1.700561 | 0.089025 | 0.1956883 | FALSE |
| GAS8-AS1 | 613 | 1.699445 | 0.089235 | 0.1960119 | FALSE |
| GATD1 | 7500 | 1.698507 | 0.089412 | 0.1963174 | FALSE |
| GLTPD2 | 697 | 1.698119 | 0.089485 | 0.1964505 | FALSE |
| WDR26 | 9858 | 1.697688 | 0.089567 | 0.1966015 | FALSE |
| METRNL | 635 | 1.697486 | 0.089605 | 0.1966576 | FALSE |
| STBD1 | 3080 | 1.697118 | 0.089674 | 0.1967043 | FALSE |
| DCTD | 9427 | 1.697117 | 0.089675 | 0.1967043 | FALSE |
| C6orf226 | 11349 | 1.697107 | 0.089676 | 0.1967043 | FALSE |
| ACCS | 1715 | 1.696494 | 0.089792 | 0.1968757 | FALSE |
| UBE2E2 | 5078 | 1.696056 | 0.089875 | 0.1970023 | FALSE |
| TCTE3 | 2029 | 1.694637 | 0.090144 | 0.1974259 | FALSE |
| FTHL17 | 6717 | 1.693754 | 0.090312 | 0.1976854 | FALSE |
| KYNU | 2807 | 1.692618 | 0.090528 | 0.1980447 | FALSE |
| PRB1 | 4924 | 1.692368 | 0.090576 | 0.1981212 | FALSE |
| KIAA0100 | 744 | 1.691541 | 0.090734 | 0.1984106 | FALSE |
| TNR | 8766 | 1.691301 | 0.090779 | 0.1984552 | FALSE |
| CHST14 | 14105 | 1.691029 | 0.090831 | 0.198541 | FALSE |
| HEXIM2 | 625 | 1.690624 | 0.090909 | 0.1986531 | FALSE |
| CCEPR | 3888 | 1.690561 | 0.090921 | 0.1986531 | FALSE |
| URB1 | 12251 | 1.690415 | 0.090949 | 0.1986863 | FALSE |
| BAMBI | 9301 | 1.690272 | 0.090976 | 0.1986905 | FALSE |
| NCR2 | 8004 | 1.689427 | 0.091138 | 0.1989881 | FALSE |
| METAP1 | 4388 | 1.689109 | 0.091199 | 0.1990933 | FALSE |
| FAM171A2 | 8558 | 1.688482 | 0.091319 | 0.1993278 | FALSE |
| EBP | 8644 | 1.687431 | 0.091521 | 0.1996494 | FALSE |
| RHCE | 7161 | 1.687382 | 0.09153 | 0.1996494 | FALSE |
| ANKRA2 | 5666 | 1.686339 | 0.091731 | 0.2000591 | FALSE |
| SIGLEC15 | 10550 | 1.686184 | 0.09176 | 0.2000962 | FALSE |
| WNT11 | 9809 | 1.684798 | 0.092028 | 0.2006372 | FALSE |
| PALLD | 1308 | 1.684764 | 0.092034 | 0.2006372 | FALSE |
| CMIP | 4772 | 1.683784 | 0.092223 | 0.2009938 | FALSE |
| MRFAP1 | 5778 | 1.683629 | 0.092253 | 0.2010311 | FALSE |
| TCL6 | 6865 | 1.683242 | 0.092328 | 0.2011161 | FALSE |
| MYRFL | 7326 | 1.683228 | 0.092331 | 0.2011161 | FALSE |
| SEM1 | 10866 | 1.683051 | 0.092365 | 0.2011626 | FALSE |
| GPR137B | 9300 | 1.68253 | 0.092466 | 0.2013543 | FALSE |
| MLF1 | 6159 | 1.682369 | 0.092497 | 0.2013662 | FALSE |
| MIIP | 4539 | 1.682169 | 0.092536 | 0.2013945 | FALSE |
| TMEM230 | 8341 | 1.681727 | 0.092622 | 0.2015249 | FALSE |
| ATP9B | 6036 | 1.681503 | 0.092665 | 0.2015914 | FALSE |
| GCLC | 3429 | 1.681221 | 0.09272 | 0.2016824 | FALSE |
| DPRX | 9018 | 1.680822 | 0.092797 | 0.2017949 | FALSE |
| ACTG2 | 8776 | 1.680744 | 0.092813 | 0.2017997 | FALSE |
| KRTAP8-1 | 1547 | 1.680615 | 0.092838 | 0.2018262 | FALSE |
| HIST1H2AC | 13359 | 1.680005 | 0.092956 | 0.202056 | FALSE |
| NDUFAF3 | 7040 | 1.67897 | 0.093158 | 0.2024325 | FALSE |
| REC8 | 3297 | 1.678916 | 0.093168 | 0.2024325 | FALSE |
| AKR1C3 | 7505 | 1.6782 | 0.093308 | 0.2026796 | FALSE |
| PUS3 | 10555 | 1.678027 | 0.093342 | 0.2027212 | FALSE |
| CDYL2 | 2653 | 1.677969 | 0.093353 | 0.2027212 | FALSE |
| HVCN1 | 6558 | 1.676248 | 0.09369 | 0.2033389 | FALSE |
| SALL1 | 7223 | 1.67587 | 0.093764 | 0.2034713 | FALSE |
| SLC25A53 | 13076 | 1.675438 | 0.093848 | 0.2036268 | FALSE |
| MRPL43 | 11190 | 1.674402 | 0.094052 | 0.2039678 | FALSE |
| CTH | 4106 | 1.67438 | 0.094056 | 0.2039678 | FALSE |
| UBE3D | 2912 | 1.674195 | 0.094092 | 0.2039678 | FALSE |
| EIF4G1 | 3622 | 1.674121 | 0.094107 | 0.2039678 | FALSE |
| SULT2B1 | 5027 | 1.674047 | 0.094121 | 0.2039678 | FALSE |
| NACA2 | 8775 | 1.674039 | 0.094123 | 0.2039678 | FALSE |
| HPCA | 1995 | 1.673771 | 0.094176 | 0.2040403 | FALSE |
| TNKS2 | 7597 | 1.673736 | 0.094182 | 0.2040403 | FALSE |
| LOC101927811 | 2782 | 1.672843 | 0.094358 | 0.2043077 | FALSE |
| DNAJC2 | 1050 | 1.67242 | 0.094442 | 0.2044032 | FALSE |
| TMEM179B | 2199 | 1.672089 | 0.094507 | 0.2044878 | FALSE |
| SEC61B | 8255 | 1.672006 | 0.094523 | 0.2044914 | FALSE |
| MYT1 | 5253 | 1.671948 | 0.094535 | 0.2044914 | FALSE |
| CHAD | 13889 | 1.671693 | 0.094585 | 0.2045719 | FALSE |
| SHC3 | 8152 | 1.670844 | 0.094752 | 0.2048494 | FALSE |
| ZNF319 | 3659 | 1.670194 | 0.094881 | 0.2050704 | FALSE |
| ASMTL | 3704 | 1.669479 | 0.095022 | 0.2053168 | FALSE |
| NTAN1 | 2977 | 1.669419 | 0.095034 | 0.2053168 | FALSE |
| KRTAP19-2 | 1406 | 1.669189 | 0.09508 | 0.2053868 | FALSE |
| PUM3 | 2736 | 1.66863 | 0.095191 | 0.2055978 | FALSE |
| NKX2-3 | 4968 | 1.668199 | 0.095276 | 0.205754 | FALSE |
| HIST1H2BO | 8158 | 1.667573 | 0.095401 | 0.2059371 | FALSE |
| ZNF844 | 2378 | 1.667258 | 0.095463 | 0.2059899 | FALSE |
| SEC23IP | 12981 | 1.667258 | 0.095463 | 0.2059899 | FALSE |
| TOMM5 | 6679 | 1.667251 | 0.095465 | 0.2059899 | FALSE |
| CC2D2B | 9008 | 1.666961 | 0.095522 | 0.2060858 | FALSE |
| TSG101 | 5231 | 1.666687 | 0.095577 | 0.206175 | FALSE |
| ARHGAP27P1-BPTFP1-KPNA2P3 | 14578 | 1.666541 | 0.095606 | 0.2062092 | FALSE |
| C2orf81 | 10154 | 1.666423 | 0.095629 | 0.2062314 | FALSE |
| ENAH | 8333 | 1.665694 | 0.095774 | 0.2064875 | FALSE |
| MISP3 | 315 | 1.664859 | 0.095941 | 0.2068144 | FALSE |
| C1orf158 | 525 | 1.664384 | 0.096036 | 0.2069038 | FALSE |
| SOX8 | 3072 | 1.664328 | 0.096047 | 0.2069038 | FALSE |
| NDUFA2 | 12679 | 1.66392 | 0.096128 | 0.2070509 | FALSE |
| RPS27 | 14453 | 1.663661 | 0.09618 | 0.2071339 | FALSE |
| AGT | 211 | 1.663245 | 0.096263 | 0.2072845 | FALSE |
| TTL | 8821 | 1.663071 | 0.096298 | 0.2073024 | FALSE |
| DYNC1H1 | 7041 | 1.662731 | 0.096366 | 0.2074204 | FALSE |
| AASS | 7048 | 1.662105 | 0.096492 | 0.2076618 | FALSE |
| RNLS | 3491 | 1.661571 | 0.096599 | 0.2078636 | FALSE |
| PAK4 | 13869 | 1.661379 | 0.096637 | 0.2079179 | FALSE |
| GBP3 | 4459 | 1.661072 | 0.096699 | 0.2079647 | FALSE |
| HTATSF1 | 5699 | 1.660295 | 0.096855 | 0.2082145 | FALSE |
| KRTAP10-9 | 4399 | 1.658735 | 0.097169 | 0.2087462 | FALSE |
| TAAR2 | 3660 | 1.658374 | 0.097242 | 0.2088739 | FALSE |
| ELAC2 | 13346 | 1.658096 | 0.097298 | 0.2089113 | FALSE |
| GRIA2 | 3423 | 1.658094 | 0.097298 | 0.2089113 | FALSE |
| OAS1 | 5174 | 1.658089 | 0.0973 | 0.2089113 | FALSE |
| DZIP3 | 4623 | 1.657611 | 0.097396 | 0.2090324 | FALSE |
| PAPLN | 10376 | 1.657516 | 0.097415 | 0.2090449 | FALSE |
| PLBD1 | 1519 | 1.656904 | 0.097539 | 0.2092816 | FALSE |
| SLC35G6 | 2278 | 1.656542 | 0.097612 | 0.2094099 | FALSE |
| CSE1L | 4046 | 1.656376 | 0.097646 | 0.2094245 | FALSE |
| MAGOHB | 1307 | 1.656171 | 0.097687 | 0.2094848 | FALSE |
| ZNF275 | 12588 | 1.655626 | 0.097798 | 0.2096352 | FALSE |
| ATP6V1A | 3028 | 1.654971 | 0.09793 | 0.2098911 | FALSE |
| KCMF1 | 12589 | 1.654708 | 0.097984 | 0.2099479 | FALSE |
| PQLC1 | 1782 | 1.654253 | 0.098076 | 0.2100794 | FALSE |
| PPIA | 10085 | 1.654141 | 0.098099 | 0.2100794 | FALSE |
| TGFBR3L | 13920 | 1.653972 | 0.098133 | 0.2101036 | FALSE |
| EIF1AY | 12666 | 1.653635 | 0.098202 | 0.2101326 | FALSE |
| TEDC1 | 5350 | 1.65363 | 0.098203 | 0.2101326 | FALSE |
| FKRP | 13708 | 1.652581 | 0.098416 | 0.2105282 | FALSE |
| APAF1 | 432 | 1.651497 | 0.098637 | 0.2109147 | FALSE |
| KIRREL2 | 13368 | 1.651496 | 0.098637 | 0.2109147 | FALSE |
| BMP7 | 8508 | 1.650867 | 0.098766 | 0.2111315 | FALSE |
| SMCO3 | 11520 | 1.650145 | 0.098913 | 0.211418 | FALSE |
| VAV2 | 1407 | 1.649579 | 0.099029 | 0.2115787 | FALSE |
| C2orf50 | 13712 | 1.649357 | 0.099075 | 0.2116469 | FALSE |
| RHOBTB3 | 5604 | 1.648811 | 0.099186 | 0.2118279 | FALSE |
| MAEA | 3031 | 1.648548 | 0.09924 | 0.2119141 | FALSE |
| AKAP11 | 10700 | 1.648388 | 0.099273 | 0.2119552 | FALSE |
| ADARB2-AS1 | 3951 | 1.647431 | 0.099469 | 0.2122739 | FALSE |
| PLK3 | 14890 | 1.64733 | 0.09949 | 0.2122739 | FALSE |
| OR10V1 | 1883 | 1.647069 | 0.099544 | 0.2123461 | FALSE |
| LYN | 192 | 1.646106 | 0.099742 | 0.2126658 | FALSE |
| HOXB-AS3 | 13921 | 1.645953 | 0.099773 | 0.212704 | FALSE |
| ANHX | 12180 | 1.645382 | 0.099891 | 0.2128967 | FALSE |
| NMUR1 | 5023 | 1.644583 | 0.100056 | 0.2131065 | FALSE |
| COMP | 2334 | 1.644566 | 0.100059 | 0.2131065 | FALSE |
| VSTM4 | 11541 | 1.644285 | 0.100117 | 0.2131755 | FALSE |
| CH17-340M24.3 | 2557 | 1.644084 | 0.100159 | 0.2132335 | FALSE |
| LIMA1 | 9206 | 1.644021 | 0.100172 | 0.2132335 | FALSE |
| TLX1 | 12279 | 1.643574 | 0.100264 | 0.213343 | FALSE |
| CYP1B1 | 1418 | 1.64288 | 0.100408 | 0.2136193 | FALSE |
| SPAST | 6032 | 1.642378 | 0.100512 | 0.2137606 | FALSE |
| UXT | 9778 | 1.642348 | 0.100518 | 0.2137606 | FALSE |
| PLS3 | 11352 | 1.642295 | 0.100529 | 0.2137606 | FALSE |
| PTH2 | 12216 | 1.641789 | 0.100634 | 0.213939 | FALSE |
| HPS4 | 8122 | 1.641284 | 0.100738 | 0.2140898 | FALSE |
| TMEM231 | 378 | 1.64115 | 0.100766 | 0.2141198 | FALSE |
| PSMF1 | 11146 | 1.640845 | 0.10083 | 0.2142252 | FALSE |
| CCDC85A | 13679 | 1.64055 | 0.100891 | 0.2142998 | FALSE |
| CFAP97D1 | 13365 | 1.640544 | 0.100892 | 0.2142998 | FALSE |
| EVX1 | 14237 | 1.63922 | 0.101167 | 0.2147427 | FALSE |
| R3HCC1 | 5856 | 1.638343 | 0.10135 | 0.2150204 | FALSE |
| HK2 | 507 | 1.637742 | 0.101476 | 0.2152465 | FALSE |
| PREX2 | 7221 | 1.637371 | 0.101553 | 0.2153816 | FALSE |
| MHENCR | 2988 | 1.637234 | 0.101582 | 0.215413 | FALSE |
| TBATA | 12635 | 1.636118 | 0.101815 | 0.2158492 | FALSE |
| NSDHL | 1466 | 1.635159 | 0.102016 | 0.2162163 | FALSE |
| DKK4 | 2405 | 1.634411 | 0.102173 | 0.2164608 | FALSE |
| PLPPR5 | 3082 | 1.63431 | 0.102194 | 0.2164763 | FALSE |
| CASTOR2 | 1051 | 1.634069 | 0.102244 | 0.2165248 | FALSE |
| DAB2 | 9947 | 1.633586 | 0.102346 | 0.2166516 | FALSE |
| NIN | 6741 | 1.633325 | 0.102401 | 0.2167384 | FALSE |
| IQCJ | 3235 | 1.633178 | 0.102432 | 0.2167549 | FALSE |
| IBA57 | 4954 | 1.633156 | 0.102436 | 0.2167549 | FALSE |
| GPR132 | 12746 | 1.632608 | 0.102551 | 0.2169401 | FALSE |
| MANBAL | 8626 | 1.632026 | 0.102674 | 0.2171699 | FALSE |
| SYDE1 | 10652 | 1.631599 | 0.102764 | 0.2173308 | FALSE |
| DOCK9 | 6132 | 1.630694 | 0.102955 | 0.2177051 | FALSE |
| ZMAT2 | 9114 | 1.630137 | 0.103073 | 0.2178949 | FALSE |
| C9orf78 | 5477 | 1.629683 | 0.103169 | 0.2180603 | FALSE |
| EHD1 | 11199 | 1.629635 | 0.103179 | 0.2180603 | FALSE |
| GEM | 10227 | 1.628167 | 0.103489 | 0.2185458 | FALSE |
| RPAIN | 15033 | 1.628052 | 0.103514 | 0.2185458 | FALSE |
| PLXNB1 | 13006 | 1.626755 | 0.103789 | 0.2190544 | FALSE |
| LRP10 | 10809 | 1.626525 | 0.103838 | 0.2191279 | FALSE |
| LOC101928424 | 12565 | 1.625984 | 0.103953 | 0.2193115 | FALSE |
| RGN | 5795 | 1.625414 | 0.104074 | 0.2195082 | FALSE |
| CIPC | 10106 | 1.624992 | 0.104164 | 0.2196385 | FALSE |
| SULT1C4 | 12257 | 1.624797 | 0.104206 | 0.2196736 | FALSE |
| SESN3 | 13601 | 1.624782 | 0.104209 | 0.2196736 | FALSE |
| C4orf19 | 14122 | 1.624713 | 0.104224 | 0.219675 | FALSE |
| AIG1 | 11558 | 1.624288 | 0.104314 | 0.2198364 | FALSE |
| RHOJ | 6708 | 1.624019 | 0.104372 | 0.219846 | FALSE |
| IVL | 2672 | 1.624014 | 0.104373 | 0.219846 | FALSE |
| SMYD5 | 9224 | 1.624003 | 0.104375 | 0.219846 | FALSE |
| TGOLN2 | 9240 | 1.623278 | 0.10453 | 0.2201101 | FALSE |
| LRRC47 | 12048 | 1.622973 | 0.104595 | 0.2201907 | FALSE |
| FDX1L | 12484 | 1.622848 | 0.104622 | 0.2202173 | FALSE |
| MON1B | 14800 | 1.622328 | 0.104733 | 0.2203328 | FALSE |
| AGPAT1 | 6749 | 1.621676 | 0.104873 | 0.2205888 | FALSE |
| SEMG2 | 11 | 1.620667 | 0.105089 | 0.2209627 | FALSE |
| FAM213B | 10644 | 1.620343 | 0.105159 | 0.2210792 | FALSE |
| KRTAP19-5 | 6095 | 1.620139 | 0.105202 | 0.2211415 | FALSE |
| AANAT | 9214 | 1.619574 | 0.105324 | 0.2213119 | FALSE |
| CCDC6 | 15301 | 1.61895 | 0.105458 | 0.2215299 | FALSE |
| GINS3 | 4833 | 1.618448 | 0.105566 | 0.2216973 | FALSE |
| FCN3 | 2172 | 1.618259 | 0.105607 | 0.2217133 | FALSE |
| CCL22 | 5930 | 1.618249 | 0.105609 | 0.2217133 | FALSE |
| PXDC1 | 8055 | 1.618111 | 0.105639 | 0.2217203 | FALSE |
| GDI1 | 1416 | 1.617874 | 0.10569 | 0.2217485 | FALSE |
| GCK | 10176 | 1.617471 | 0.105777 | 0.221901 | FALSE |
| KRTAP5-10 | 6929 | 1.61676 | 0.10593 | 0.2221633 | FALSE |
| GPBP1 | 11647 | 1.616388 | 0.10601 | 0.2222366 | FALSE |
| YIF1A | 4888 | 1.615353 | 0.106234 | 0.222587 | FALSE |
| GPD1 | 4625 | 1.615351 | 0.106235 | 0.222587 | FALSE |
| ATP1B2 | 8159 | 1.615291 | 0.106248 | 0.222587 | FALSE |
| HIST1H2APS1 | 3908 | 1.615233 | 0.10626 | 0.222587 | FALSE |
| ZNF814 | 4775 | 1.613838 | 0.106563 | 0.2230708 | FALSE |
| NDUFB4 | 1654 | 1.611729 | 0.107021 | 0.2238847 | FALSE |
| TLX1NB | 9275 | 1.611602 | 0.107049 | 0.2238847 | FALSE |
| TMOD3 | 7697 | 1.611588 | 0.107052 | 0.2238847 | FALSE |
| BHLHE23 | 11252 | 1.611068 | 0.107165 | 0.2240916 | FALSE |
| DPEP3 | 15267 | 1.610808 | 0.107222 | 0.2241502 | FALSE |
| KCNT2 | 5173 | 1.610171 | 0.107361 | 0.2244106 | FALSE |
| SPPL2C | 10295 | 1.609989 | 0.1074 | 0.2244355 | FALSE |
| CHERP | 13360 | 1.609791 | 0.107443 | 0.2244691 | FALSE |
| PBLD | 14661 | 1.609714 | 0.10746 | 0.2244691 | FALSE |
| CD2 | 10455 | 1.609573 | 0.107491 | 0.2245035 | FALSE |
| RWDD4 | 10627 | 1.60939 | 0.107531 | 0.2245486 | FALSE |
| FAM86B3P | 6026 | 1.609289 | 0.107553 | 0.2245486 | FALSE |
| NRL | 3686 | 1.609277 | 0.107556 | 0.2245486 | FALSE |
| C5 | 7701 | 1.608958 | 0.107626 | 0.2246642 | FALSE |
| TRIM17 | 10969 | 1.608499 | 0.107726 | 0.2247864 | FALSE |
| WIPI2 | 10100 | 1.608144 | 0.107804 | 0.2248805 | FALSE |
| C5orf30 | 13760 | 1.608116 | 0.10781 | 0.2248805 | FALSE |
| RAPH1 | 3742 | 1.60617 | 0.108237 | 0.2256384 | FALSE |
| FAM83H | 5783 | 1.605917 | 0.108292 | 0.2256956 | FALSE |
| HRASLS5 | 12704 | 1.605503 | 0.108383 | 0.2258235 | FALSE |
| KBTBD6 | 5349 | 1.605338 | 0.108419 | 0.225869 | FALSE |
| TSPAN17 | 10474 | 1.605261 | 0.108436 | 0.2258742 | FALSE |
| LOC105378853 | 1440 | 1.604919 | 0.108512 | 0.2260008 | FALSE |
| GTPBP4 | 8195 | 1.604679 | 0.108564 | 0.2260808 | FALSE |
| FAM84B | 13447 | 1.603369 | 0.108853 | 0.2265312 | FALSE |
| CCDC78 | 13398 | 1.603302 | 0.108868 | 0.2265318 | FALSE |
| ZNF622 | 7449 | 1.603067 | 0.10892 | 0.2265973 | FALSE |
| SIDT1 | 5576 | 1.603028 | 0.108928 | 0.2265973 | FALSE |
| LSG1 | 355 | 1.602844 | 0.108969 | 0.2266401 | FALSE |
| KPNA2 | 7242 | 1.602738 | 0.108993 | 0.2266401 | FALSE |
| COMMD1 | 14947 | 1.60233 | 0.109083 | 0.2267672 | FALSE |
| IL17REL | 6181 | 1.602123 | 0.109128 | 0.2268321 | FALSE |
| CAVIN3 | 8029 | 1.601907 | 0.109176 | 0.2269012 | FALSE |
| SRD5A3 | 5813 | 1.601305 | 0.109309 | 0.2271177 | FALSE |
| CGAS | 7649 | 1.600767 | 0.109429 | 0.2272445 | FALSE |
| MYADM | 1722 | 1.600632 | 0.109458 | 0.2272764 | FALSE |
| THAP6 | 11814 | 1.599966 | 0.109606 | 0.2275528 | FALSE |
| ZNHIT3 | 13838 | 1.597313 | 0.110196 | 0.2285787 | FALSE |
| OSBPL8 | 6801 | 1.597034 | 0.110258 | 0.2286635 | FALSE |
| CLDN14 | 15151 | 1.595794 | 0.110535 | 0.2290853 | FALSE |
| MMRN2 | 7964 | 1.5948 | 0.110757 | 0.2294765 | FALSE |
| RBM43 | 4253 | 1.594687 | 0.110782 | 0.2294765 | FALSE |
| SYCE1L | 10452 | 1.594555 | 0.110812 | 0.2294768 | FALSE |
| FBXL14 | 4621 | 1.594349 | 0.110858 | 0.2295419 | FALSE |
| CTRB1 | 2827 | 1.593619 | 0.111021 | 0.22985 | FALSE |
| CHCHD2 | 1471 | 1.593419 | 0.111066 | 0.2299124 | FALSE |
| SNX22 | 6924 | 1.591561 | 0.111483 | 0.2305622 | FALSE |
| HDLBP | 5411 | 1.59035 | 0.111756 | 0.2310647 | FALSE |
| TMTC3 | 7089 | 1.590132 | 0.111805 | 0.2311357 | FALSE |
| C1QTNF3 | 5935 | 1.590062 | 0.111821 | 0.2311378 | FALSE |
| PIH1D2 | 9996 | 1.589972 | 0.111841 | 0.2311492 | FALSE |
| LIPA | 276 | 1.58876 | 0.112115 | 0.2316225 | FALSE |
| IRX5 | 5723 | 1.587999 | 0.112287 | 0.2319029 | FALSE |
| SPIN4 | 9289 | 1.587965 | 0.112294 | 0.2319029 | FALSE |
| SPEM1 | 9689 | 1.587897 | 0.11231 | 0.2319029 | FALSE |
| C15orf39 | 8407 | 1.587273 | 0.112451 | 0.2321293 | FALSE |
| SEC13 | 13771 | 1.587181 | 0.112472 | 0.2321293 | FALSE |
| TOP1MT | 4972 | 1.58715 | 0.112479 | 0.2321293 | FALSE |
| NCSTN | 8888 | 1.58679 | 0.11256 | 0.2322669 | FALSE |
| FUT6 | 7683 | 1.586604 | 0.112602 | 0.2322925 | FALSE |
| CC2D1A | 15566 | 1.586535 | 0.112618 | 0.2322941 | FALSE |
| ZFAND6 | 9721 | 1.586449 | 0.112637 | 0.2322997 | FALSE |
| ADAM8 | 8779 | 1.586392 | 0.11265 | 0.2322997 | FALSE |
| ERGIC3 | 11537 | 1.586214 | 0.112691 | 0.2323471 | FALSE |
| CALM2 | 3526 | 1.586114 | 0.112713 | 0.2323471 | FALSE |
| MLEC | 14620 | 1.586094 | 0.112718 | 0.2323471 | FALSE |
| SYCE3 | 852 | 1.585456 | 0.112863 | 0.2325842 | FALSE |
| LDHA | 13798 | 1.584843 | 0.113002 | 0.2328271 | FALSE |
| SLC5A2 | 13716 | 1.584806 | 0.11301 | 0.2328271 | FALSE |
| LINC01792 | 12551 | 1.584452 | 0.113091 | 0.2329008 | FALSE |
| SFXN5 | 12483 | 1.583739 | 0.113253 | 0.2331102 | FALSE |
| KIFC3 | 11501 | 1.583632 | 0.113277 | 0.2331102 | FALSE |
| ABCA3 | 11758 | 1.583612 | 0.113282 | 0.2331102 | FALSE |
| ANKRD65 | 11909 | 1.582468 | 0.113543 | 0.2335545 | FALSE |
| TJP3 | 6823 | 1.582327 | 0.113575 | 0.2335899 | FALSE |
| WSB2 | 4776 | 1.581932 | 0.113665 | 0.2337139 | FALSE |
| FUCA2 | 576 | 1.581262 | 0.113818 | 0.2339362 | FALSE |
| RTRAF | 2420 | 1.57939 | 0.114247 | 0.234601 | FALSE |
| TMEM199 | 8185 | 1.578424 | 0.114468 | 0.2350156 | FALSE |
| NOL7 | 14529 | 1.578379 | 0.114479 | 0.2350156 | FALSE |
| JUNB | 7883 | 1.57763 | 0.114651 | 0.2353379 | FALSE |
| LOC101926934 | 9265 | 1.576611 | 0.114885 | 0.2357572 | FALSE |
| CARHSP1 | 10864 | 1.576131 | 0.114996 | 0.2358602 | FALSE |
| CHAF1B | 10435 | 1.576036 | 0.115018 | 0.2358742 | FALSE |
| MRPL24 | 5986 | 1.57386 | 0.11552 | 0.23678 | FALSE |
| HLA-DPB2 | 14143 | 1.57369 | 0.115559 | 0.2368296 | FALSE |
| PLA2G2D | 7791 | 1.573381 | 0.115631 | 0.2368296 | FALSE |
| PRPF31 | 14274 | 1.57331 | 0.115647 | 0.2368296 | FALSE |
| CYP4Z1 | 14334 | 1.573297 | 0.11565 | 0.2368296 | FALSE |
| DDT | 5761 | 1.573297 | 0.11565 | 0.2368296 | FALSE |
| METTL9 | 13504 | 1.573053 | 0.115707 | 0.2369143 | FALSE |
| SRSF2 | 4030 | 1.572896 | 0.115743 | 0.2369577 | FALSE |
| CYB561 | 3502 | 1.57199 | 0.115953 | 0.2373565 | FALSE |
| AMFR | 5055 | 1.571661 | 0.116029 | 0.2374816 | FALSE |
| PIH1D1 | 1472 | 1.571065 | 0.116168 | 0.2377336 | FALSE |
| ARF4 | 3889 | 1.570917 | 0.116202 | 0.2377729 | FALSE |
| LINC02361 | 4416 | 1.570443 | 0.116312 | 0.2378707 | FALSE |
| C16orf70 | 6348 | 1.570384 | 0.116326 | 0.2378707 | FALSE |
| VPS25 | 15330 | 1.570099 | 0.116392 | 0.2379751 | FALSE |
| TBC1D10B | 13930 | 1.569246 | 0.116591 | 0.2382098 | FALSE |
| TMC4 | 15053 | 1.568525 | 0.116759 | 0.2384439 | FALSE |
| WDR45B | 7933 | 1.568346 | 0.1168 | 0.238498 | FALSE |
| EME2 | 31 | 1.568164 | 0.116843 | 0.2385535 | FALSE |
| GAS5 | 15621 | 1.567753 | 0.116939 | 0.2387182 | FALSE |
| RTP5 | 12258 | 1.567599 | 0.116975 | 0.2387604 | FALSE |
| SCNN1B | 10973 | 1.567248 | 0.117057 | 0.2388342 | FALSE |
| RAB15 | 8064 | 1.567152 | 0.117079 | 0.2388488 | FALSE |
| SPRYD4 | 10121 | 1.566609 | 0.117206 | 0.2390454 | FALSE |
| RCBTB2 | 2479 | 1.566315 | 0.117275 | 0.2390921 | FALSE |
| KRT17P5 | 9593 | 1.565994 | 0.11735 | 0.2391829 | FALSE |
| OR2B11 | 4808 | 1.564585 | 0.11768 | 0.239731 | FALSE |
| RBM4 | 6528 | 1.563997 | 0.117818 | 0.2399496 | FALSE |
| PHOX2A | 14709 | 1.563917 | 0.117837 | 0.2399566 | FALSE |
| SLC38A10 | 13963 | 1.563476 | 0.117941 | 0.240105 | FALSE |
| ABCB6 | 194 | 1.56319 | 0.118008 | 0.2402106 | FALSE |
| BDKRB2 | 224 | 1.563095 | 0.11803 | 0.2402248 | FALSE |
| SNTA1 | 8696 | 1.56129 | 0.118455 | 0.2409645 | FALSE |
| ZNF512B | 6704 | 1.560353 | 0.118676 | 0.2413516 | FALSE |
| LYL1 | 989 | 1.56011 | 0.118734 | 0.2414369 | FALSE |
| SLC17A7 | 5860 | 1.559739 | 0.118822 | 0.2415838 | FALSE |
| DPY19L3 | 1425 | 1.558733 | 0.11906 | 0.2419715 | FALSE |
| NICN1 | 10457 | 1.558627 | 0.119085 | 0.2419715 | FALSE |
| HERC4 | 6812 | 1.558418 | 0.119134 | 0.2419715 | FALSE |
| STRAP | 12789 | 1.558179 | 0.119191 | 0.2420512 | FALSE |
| BMP2 | 3524 | 1.557406 | 0.119374 | 0.2423605 | FALSE |
| NDUFA10 | 8315 | 1.557224 | 0.119417 | 0.2424167 | FALSE |
| GAB4 | 4857 | 1.555709 | 0.119777 | 0.2430341 | FALSE |
| HIST1H2BF | 14887 | 1.555617 | 0.119799 | 0.2430341 | FALSE |
| ZNF613 | 13043 | 1.55533 | 0.119867 | 0.2431411 | FALSE |
| SINHCAF | 9360 | 1.555034 | 0.119938 | 0.2432525 | FALSE |
| FOXF1 | 5882 | 1.554815 | 0.11999 | 0.2432636 | FALSE |
| HSPH1 | 13225 | 1.554349 | 0.120101 | 0.2434256 | FALSE |
| ICAM4 | 27 | 1.552254 | 0.120601 | 0.2443129 | FALSE |
| MUL1 | 15177 | 1.551346 | 0.120819 | 0.2447214 | FALSE |
| ODF3L1 | 9873 | 1.551201 | 0.120854 | 0.2447601 | FALSE |
| CCDC170 | 14505 | 1.550823 | 0.120944 | 0.2449118 | FALSE |
| SLC8A3 | 1374 | 1.550326 | 0.121063 | 0.2451214 | FALSE |
| GPKOW | 8449 | 1.55011 | 0.121115 | 0.2451692 | FALSE |
| GLIS2 | 6962 | 1.549441 | 0.121276 | 0.2454562 | FALSE |
| ACP1 | 10924 | 1.548762 | 0.121439 | 0.2457229 | FALSE |
| MGRN1 | 2247 | 1.547817 | 0.121666 | 0.2460238 | FALSE |
| JUND | 10245 | 1.547693 | 0.121696 | 0.2460524 | FALSE |
| LINC02550 | 10017 | 1.547297 | 0.121792 | 0.2462135 | FALSE |
| GRAMD4 | 12376 | 1.547176 | 0.121821 | 0.2462406 | FALSE |
| MROH7 | 3567 | 1.546887 | 0.12189 | 0.2463496 | FALSE |
| NMRAL1 | 2485 | 1.546137 | 0.122071 | 0.2465971 | FALSE |
| STIP1 | 15078 | 1.546118 | 0.122076 | 0.2465971 | FALSE |
| GSTA4 | 2682 | 1.545377 | 0.122255 | 0.246895 | FALSE |
| SLC17A4 | 5403 | 1.544995 | 0.122347 | 0.2470177 | FALSE |
| ACAA1 | 8050 | 1.544586 | 0.122446 | 0.2471644 | FALSE |
| SKAP1 | 13012 | 1.544564 | 0.122452 | 0.2471644 | FALSE |
| WFDC3 | 5700 | 1.54435 | 0.122504 | 0.2472371 | FALSE |
| AOC3 | 774 | 1.544266 | 0.122524 | 0.2472462 | FALSE |
| SLC22A12 | 15066 | 1.544188 | 0.122543 | 0.2472524 | FALSE |
| COX6A2 | 11645 | 1.543985 | 0.122592 | 0.2472637 | FALSE |
| GNB1L | 7360 | 1.543605 | 0.122684 | 0.2473831 | FALSE |
| SLC52A3 | 5077 | 1.543594 | 0.122687 | 0.2473831 | FALSE |
| GLB1L | 9874 | 1.542631 | 0.12292 | 0.2477264 | FALSE |
| SUGCT | 8828 | 1.542454 | 0.122963 | 0.2477811 | FALSE |
| CD300C | 9204 | 1.541722 | 0.123141 | 0.2480755 | FALSE |
| COG6 | 13617 | 1.541238 | 0.123259 | 0.2482486 | FALSE |
| MTMR3 | 3543 | 1.541133 | 0.123284 | 0.2482681 | FALSE |
| NIPSNAP2 | 12408 | 1.540968 | 0.123325 | 0.248287 | FALSE |
| UCN2 | 10836 | 1.540964 | 0.123326 | 0.248287 | FALSE |
| MAGEF1 | 859 | 1.539809 | 0.123607 | 0.2487894 | FALSE |
| C3orf62 | 15464 | 1.539469 | 0.12369 | 0.2488922 | FALSE |
| VANGL1 | 13813 | 1.539346 | 0.12372 | 0.2489184 | FALSE |
| GPR176 | 15018 | 1.539055 | 0.123791 | 0.2489835 | FALSE |
| APOL4 | 1804 | 1.539022 | 0.123799 | 0.2489835 | FALSE |
| IER3 | 7387 | 1.537067 | 0.124277 | 0.249687 | FALSE |
| ACTG1P4 | 3427 | 1.536967 | 0.124301 | 0.249687 | FALSE |
| GS1-124K5.11 | 5775 | 1.536893 | 0.12432 | 0.249687 | FALSE |
| GPS1 | 6068 | 1.536873 | 0.124324 | 0.249687 | FALSE |
| POMGNT1 | 731 | 1.536779 | 0.124347 | 0.2497012 | FALSE |
| NID2 | 794 | 1.536425 | 0.124434 | 0.2498432 | FALSE |
| SLC9A3R1 | 9690 | 1.535929 | 0.124556 | 0.2500553 | FALSE |
| GPHB5 | 3757 | 1.535423 | 0.12468 | 0.2502724 | FALSE |
| ACAA2 | 2171 | 1.534916 | 0.124804 | 0.250458 | FALSE |
| SGTA | 2542 | 1.534825 | 0.124827 | 0.2504708 | FALSE |
| AMZ2 | 1480 | 1.534745 | 0.124846 | 0.2504781 | FALSE |
| EFR3B | 5195 | 1.534477 | 0.124912 | 0.250578 | FALSE |
| PNKP | 11723 | 1.534406 | 0.12493 | 0.2505809 | FALSE |
| EGLN1 | 3887 | 1.533731 | 0.125096 | 0.2508174 | FALSE |
| CRAT | 7228 | 1.533588 | 0.125131 | 0.2508558 | FALSE |
| RSRC2 | 8629 | 1.532426 | 0.125417 | 0.2513653 | FALSE |
| MRGPRX4 | 10839 | 1.532342 | 0.125438 | 0.2513746 | FALSE |
| LRCOL1 | 6909 | 1.531829 | 0.125565 | 0.2515002 | FALSE |
| ENO4 | 4122 | 1.531777 | 0.125577 | 0.2515002 | FALSE |
| DNAJB6 | 1980 | 1.531762 | 0.125581 | 0.2515002 | FALSE |
| G6PC3 | 7929 | 1.531101 | 0.125744 | 0.2517626 | FALSE |
| CRYZL2P | 12925 | 1.530946 | 0.125783 | 0.2518071 | FALSE |
| ACSBG1 | 7647 | 1.530685 | 0.125847 | 0.251904 | FALSE |
| S1PR1 | 4942 | 1.530583 | 0.125872 | 0.2519222 | FALSE |
| MMGT1 | 11479 | 1.530375 | 0.125924 | 0.2519929 | FALSE |
| ATP5PB | 11771 | 1.530072 | 0.125999 | 0.2520829 | FALSE |
| ARHGAP5 | 9589 | 1.530063 | 0.126001 | 0.2520829 | FALSE |
| HIGD1A | 9242 | 1.529988 | 0.12602 | 0.2520878 | FALSE |
| SYNPO | 9916 | 1.529843 | 0.126056 | 0.252103 | FALSE |
| CEP72 | 13864 | 1.528458 | 0.126399 | 0.25262 | FALSE |
| CELA2B | 11648 | 1.528088 | 0.126491 | 0.2527389 | FALSE |
| LOC389895 | 4982 | 1.527484 | 0.126641 | 0.2530063 | FALSE |
| BCAR1 | 13832 | 1.527354 | 0.126673 | 0.2530385 | FALSE |
| FAM122C | 10438 | 1.527099 | 0.126736 | 0.2531004 | FALSE |
| MELTF | 9592 | 1.526459 | 0.126896 | 0.253386 | FALSE |
| C1orf210 | 7479 | 1.526281 | 0.12694 | 0.2534211 | FALSE |
| LINC01672 | 9490 | 1.525816 | 0.127056 | 0.2536085 | FALSE |
| TUBB4B | 3790 | 1.524996 | 0.12726 | 0.2539192 | FALSE |
| FAM126A | 5848 | 1.52488 | 0.127289 | 0.2539445 | FALSE |
| PQLC3 | 12783 | 1.524578 | 0.127364 | 0.2539976 | FALSE |
| HEPACAM | 4827 | 1.52433 | 0.127426 | 0.2540887 | FALSE |
| PSMB4 | 11396 | 1.522531 | 0.127876 | 0.254888 | FALSE |
| TOR3A | 14655 | 1.522304 | 0.127933 | 0.2549688 | FALSE |
| PIEZO1 | 3286 | 1.520705 | 0.128334 | 0.2555724 | FALSE |
| DIRC1 | 12160 | 1.52 | 0.128511 | 0.2558599 | FALSE |
| BOLA1 | 7704 | 1.519839 | 0.128551 | 0.2558853 | FALSE |
| NME7 | 168 | 1.519761 | 0.128571 | 0.2558853 | FALSE |
| TMEM52 | 13626 | 1.519754 | 0.128573 | 0.2558853 | FALSE |
| KRTAP6-2 | 212 | 1.519581 | 0.128616 | 0.2559393 | FALSE |
| C10orf95 | 9068 | 1.517933 | 0.129031 | 0.2566997 | FALSE |
| DYTN | 1384 | 1.517639 | 0.129105 | 0.2567818 | FALSE |
| CDK15 | 3097 | 1.517416 | 0.129162 | 0.2568258 | FALSE |
| GJA4 | 14052 | 1.517356 | 0.129177 | 0.2568258 | FALSE |
| OSCAR | 14087 | 1.516907 | 0.12929 | 0.2570185 | FALSE |
| DIEXF | 6439 | 1.51656 | 0.129378 | 0.25716 | FALSE |
| SAA1 | 11038 | 1.516192 | 0.129471 | 0.2572794 | FALSE |
| SDS | 8927 | 1.515738 | 0.129586 | 0.2574421 | FALSE |
| USP43 | 8110 | 1.515568 | 0.129629 | 0.2574621 | FALSE |
| FRMD1 | 1708 | 1.514999 | 0.129773 | 0.2576565 | FALSE |
| USP39 | 966 | 1.514967 | 0.129781 | 0.2576565 | FALSE |
| LINC00475 | 12892 | 1.51464 | 0.129864 | 0.2577323 | FALSE |
| TMEM70 | 10334 | 1.513758 | 0.130087 | 0.2580295 | FALSE |
| CCR7 | 3851 | 1.513724 | 0.130096 | 0.2580295 | FALSE |
| XIAP | 5271 | 1.513298 | 0.130204 | 0.2581784 | FALSE |
| CYB561D1 | 13720 | 1.512937 | 0.130296 | 0.2582947 | FALSE |
| IZUMO4 | 2100 | 1.512242 | 0.130472 | 0.2585793 | FALSE |
| CAMLG | 7840 | 1.511932 | 0.130551 | 0.2586675 | FALSE |
| SNX33 | 5884 | 1.511872 | 0.130566 | 0.2586675 | FALSE |
| AK3 | 10502 | 1.511479 | 0.130666 | 0.2588328 | FALSE |
| HDGFL3 | 12196 | 1.51114 | 0.130753 | 0.2588948 | FALSE |
| MFAP1 | 6252 | 1.511096 | 0.130764 | 0.2588948 | FALSE |
| TSPAN10 | 4234 | 1.51068 | 0.13087 | 0.2590063 | FALSE |
| HIST2H2BC | 8196 | 1.510431 | 0.130933 | 0.2590991 | FALSE |
| MRPL13 | 3475 | 1.510029 | 0.131036 | 0.2592137 | FALSE |
| LUC7L3 | 8078 | 1.508869 | 0.131332 | 0.259691 | FALSE |
| C2CD4B | 1952 | 1.508578 | 0.131407 | 0.2598053 | FALSE |
| ATL3 | 14486 | 1.508456 | 0.131438 | 0.2598341 | FALSE |
| CSF1 | 2869 | 1.508271 | 0.131485 | 0.2598948 | FALSE |
| AGBL2 | 10807 | 1.507862 | 0.13159 | 0.2600688 | FALSE |
| SLC19A1 | 14379 | 1.507326 | 0.131727 | 0.2602743 | FALSE |
| SPTBN1 | 13568 | 1.506849 | 0.131849 | 0.26045 | FALSE |
| TMEM35B | 8610 | 1.506523 | 0.131933 | 0.2605822 | FALSE |
| MIR99AHG | 13625 | 1.506203 | 0.132015 | 0.2607114 | FALSE |
| COX6C | 11720 | 1.505575 | 0.132176 | 0.2609639 | FALSE |
| ZIC5 | 6338 | 1.505455 | 0.132207 | 0.2609918 | FALSE |
| C11orf65 | 410 | 1.505196 | 0.132274 | 0.2610902 | FALSE |
| PLEKHB2 | 15503 | 1.504957 | 0.132335 | 0.2611785 | FALSE |
| ANO10 | 11672 | 1.504481 | 0.132458 | 0.2613258 | FALSE |
| CFAP74 | 2540 | 1.504473 | 0.13246 | 0.2613258 | FALSE |
| IRGC | 4688 | 1.504152 | 0.132542 | 0.2614304 | FALSE |
| LAMC2 | 4141 | 1.504073 | 0.132563 | 0.2614304 | FALSE |
| EN2 | 9259 | 1.503964 | 0.132591 | 0.2614304 | FALSE |
| ZIC4 | 13167 | 1.503941 | 0.132597 | 0.2614304 | FALSE |
| TRIM72 | 8901 | 1.503795 | 0.132634 | 0.2614716 | FALSE |
| ABI3 | 9001 | 1.502783 | 0.132895 | 0.2619198 | FALSE |
| PHLDB3 | 864 | 1.50259 | 0.132945 | 0.2619849 | FALSE |
| BICRA | 6331 | 1.502332 | 0.133011 | 0.2620831 | FALSE |
| STRN4 | 14756 | 1.501807 | 0.133147 | 0.2623171 | FALSE |
| TMEM117 | 4446 | 1.501658 | 0.133185 | 0.2623599 | FALSE |
| NACAD | 13939 | 1.501187 | 0.133307 | 0.2625666 | FALSE |
| NPC1L1 | 491 | 1.500907 | 0.13338 | 0.2626762 | FALSE |
| KCNK1 | 2882 | 1.499413 | 0.133767 | 0.2633386 | FALSE |
| WNT7A | 15550 | 1.498252 | 0.134068 | 0.2637626 | FALSE |
| SPEM2 | 10575 | 1.497938 | 0.134149 | 0.2638265 | FALSE |
| CCT2 | 5865 | 1.49758 | 0.134242 | 0.2639375 | FALSE |
| GAST | 398 | 1.497526 | 0.134256 | 0.2639375 | FALSE |
| OLFM3 | 14674 | 1.497451 | 0.134276 | 0.2639426 | FALSE |
| MPP3 | 4936 | 1.497234 | 0.134332 | 0.2639872 | FALSE |
| RSF1 | 10066 | 1.497029 | 0.134386 | 0.2640588 | FALSE |
| FAM201A | 1039 | 1.496401 | 0.134549 | 0.2643468 | FALSE |
| REST | 3002 | 1.495531 | 0.134776 | 0.2647257 | FALSE |
| OR2M7 | 15582 | 1.49499 | 0.134917 | 0.2649696 | FALSE |
| PUM1 | 4725 | 1.49478 | 0.134972 | 0.2650107 | FALSE |
| FAM110B | 12293 | 1.494624 | 0.135013 | 0.2650247 | FALSE |
| C3orf67 | 13551 | 1.494606 | 0.135017 | 0.2650247 | FALSE |
| TYMP | 173 | 1.494365 | 0.13508 | 0.2650903 | FALSE |
| HIST1H2BN | 4862 | 1.494258 | 0.135108 | 0.2651119 | FALSE |
| TMED3 | 1615 | 1.494 | 0.135176 | 0.2652109 | FALSE |
| DNAJC19 | 4814 | 1.49392 | 0.135197 | 0.2652172 | FALSE |
| HMGCS2 | 653 | 1.492952 | 0.13545 | 0.2656154 | FALSE |
| ZFHX4-AS1 | 23 | 1.49254 | 0.135558 | 0.2657788 | FALSE |
| LAMB2 | 3954 | 1.491511 | 0.135827 | 0.2662224 | FALSE |
| LOR | 1415 | 1.491241 | 0.135898 | 0.2663278 | FALSE |
| CNPY1 | 1508 | 1.491074 | 0.135942 | 0.2663803 | FALSE |
| SERPINA6 | 279 | 1.490894 | 0.135989 | 0.2664395 | FALSE |
| MBOAT7 | 871 | 1.490089 | 0.136201 | 0.2668205 | FALSE |
| ADGRB2 | 4280 | 1.489482 | 0.13636 | 0.2670772 | FALSE |
| SNHG5 | 14357 | 1.489431 | 0.136374 | 0.2670772 | FALSE |
| ATP6V0B | 1655 | 1.488986 | 0.136491 | 0.2672268 | FALSE |
| CEACAM19 | 8374 | 1.488976 | 0.136494 | 0.2672268 | FALSE |
| FBXO6 | 5305 | 1.48853 | 0.136611 | 0.2674233 | FALSE |
| KPNB1 | 3998 | 1.487756 | 0.136815 | 0.2677557 | FALSE |
| BOD1 | 5255 | 1.487537 | 0.136873 | 0.2678353 | FALSE |
| AP5S1 | 11176 | 1.48715 | 0.136975 | 0.2680017 | FALSE |
| CD19 | 13530 | 1.486991 | 0.137017 | 0.2680503 | FALSE |
| SH3GLB2 | 5693 | 1.486787 | 0.137071 | 0.2681221 | FALSE |
| ZFYVE21 | 11147 | 1.485497 | 0.137412 | 0.2687093 | FALSE |
| ZNF469 | 5241 | 1.48487 | 0.137578 | 0.268946 | FALSE |
| C8G | 4429 | 1.484631 | 0.137642 | 0.2690025 | FALSE |
| CCNQ | 6367 | 1.483889 | 0.137838 | 0.2693534 | FALSE |
| PROKR1 | 99 | 1.483666 | 0.137898 | 0.2693898 | FALSE |
| OR1D5 | 230 | 1.483624 | 0.137909 | 0.2693898 | FALSE |
| GPR52 | 2342 | 1.483277 | 0.138001 | 0.2695024 | FALSE |
| C1orf52 | 643 | 1.483089 | 0.138051 | 0.2695663 | FALSE |
| OSCP1 | 14786 | 1.483017 | 0.13807 | 0.26957 | FALSE |
| COL4A1 | 11296 | 1.482466 | 0.138216 | 0.2697885 | FALSE |
| LOC100506271 | 663 | 1.482259 | 0.138271 | 0.2698286 | FALSE |
| ZNHIT1 | 5121 | 1.482027 | 0.138333 | 0.2699153 | FALSE |
| ADGRF5 | 3284 | 1.481945 | 0.138355 | 0.2699242 | FALSE |
| LPIN2 | 3672 | 1.481749 | 0.138407 | 0.2699923 | FALSE |
| FAM172A | 10053 | 1.481526 | 0.138466 | 0.2700641 | FALSE |
| MMAA | 6521 | 1.481442 | 0.138489 | 0.2700641 | FALSE |
| ITGA5 | 2341 | 1.481416 | 0.138496 | 0.2700641 | FALSE |
| REXO2 | 10439 | 1.480945 | 0.138621 | 0.270255 | FALSE |
| PAFAH2 | 1878 | 1.480854 | 0.138645 | 0.270255 | FALSE |
| RHEBL1 | 5300 | 1.480775 | 0.138667 | 0.2702623 | FALSE |
| RBM42 | 11313 | 1.480431 | 0.138758 | 0.2703561 | FALSE |
| PFDN2 | 543 | 1.479993 | 0.138875 | 0.2705002 | FALSE |
| SAMD10 | 9923 | 1.479491 | 0.139009 | 0.2707275 | FALSE |
| GRK6 | 5987 | 1.478919 | 0.139162 | 0.2709239 | FALSE |
| DKFZP434K028 | 3497 | 1.478026 | 0.139401 | 0.2713213 | FALSE |
| HSPBP1 | 4961 | 1.477886 | 0.139438 | 0.2713605 | FALSE |
| LY6G5C | 13788 | 1.477638 | 0.139505 | 0.2714559 | FALSE |
| ARHGAP23 | 4632 | 1.474436 | 0.140364 | 0.2729242 | FALSE |
| 2-Mar | 12879 | 1.474372 | 0.140381 | 0.2729242 | FALSE |
| DHODH | 12137 | 1.474302 | 0.1404 | 0.2729269 | FALSE |
| TGFB3 | 10047 | 1.472722 | 0.140826 | 0.2736597 | FALSE |
| SLF2 | 4907 | 1.469633 | 0.141661 | 0.2748311 | FALSE |
| STK26 | 1071 | 1.469281 | 0.141757 | 0.2749524 | FALSE |
| NACC1 | 11663 | 1.469203 | 0.141778 | 0.2749524 | FALSE |
| SPATA32 | 4384 | 1.469128 | 0.141798 | 0.2749524 | FALSE |
| NCF1C | 6806 | 1.469078 | 0.141812 | 0.2749524 | FALSE |
| TWISTNB | 1946 | 1.468841 | 0.141876 | 0.2750241 | FALSE |
| LINC00302 | 7012 | 1.468812 | 0.141884 | 0.2750241 | FALSE |
| HES7 | 1603 | 1.468662 | 0.141924 | 0.2750689 | FALSE |
| BRMS1 | 12823 | 1.467651 | 0.142199 | 0.2754644 | FALSE |
| ZNF579 | 9229 | 1.467371 | 0.142275 | 0.2755777 | FALSE |
| SLPI | 12747 | 1.466788 | 0.142434 | 0.2758507 | FALSE |
| LOC729732 | 3441 | 1.466548 | 0.142499 | 0.275943 | FALSE |
| LINC00174 | 1503 | 1.466417 | 0.142535 | 0.2759779 | FALSE |
| SKIV2L | 8020 | 1.464933 | 0.142939 | 0.2765897 | FALSE |
| DDIT4L | 115 | 1.464705 | 0.143001 | 0.2766759 | FALSE |
| ZSCAN5A | 1432 | 1.464333 | 0.143103 | 0.2768049 | FALSE |
| BEST2 | 8436 | 1.464005 | 0.143193 | 0.2769087 | FALSE |
| SNHG12 | 1641 | 1.463642 | 0.143292 | 0.2770662 | FALSE |
| CYB5R3 | 6658 | 1.463484 | 0.143335 | 0.2771155 | FALSE |
| PPIL6 | 2345 | 1.46326 | 0.143396 | 0.2771996 | FALSE |
| LANCL2 | 15552 | 1.463043 | 0.143456 | 0.2772801 | FALSE |
| ZNF691 | 5825 | 1.46286 | 0.143506 | 0.2773083 | FALSE |
| C6orf201 | 11122 | 1.462555 | 0.143589 | 0.2774011 | FALSE |
| HIKESHI | 11070 | 1.461747 | 0.143811 | 0.2776915 | FALSE |
| LBX2-AS1 | 4519 | 1.461532 | 0.14387 | 0.277771 | FALSE |
| PRSS36 | 10535 | 1.461274 | 0.14394 | 0.2778733 | FALSE |
| TPM2 | 10517 | 1.461037 | 0.144005 | 0.2779445 | FALSE |
| KCNN2 | 8051 | 1.46101 | 0.144013 | 0.2779445 | FALSE |
| PRR35 | 6857 | 1.460725 | 0.144091 | 0.2780612 | FALSE |
| TSFM | 5355 | 1.460565 | 0.144135 | 0.2781116 | FALSE |
| LSP1 | 8139 | 1.460361 | 0.144191 | 0.2781854 | FALSE |
| HIST1H2AB | 5287 | 1.460294 | 0.144209 | 0.2781866 | FALSE |
| HRH4 | 10788 | 1.459758 | 0.144357 | 0.2784364 | FALSE |
| ENY2 | 7454 | 1.459543 | 0.144416 | 0.278516 | FALSE |
| B4GALT5 | 5644 | 1.459442 | 0.144443 | 0.2785352 | FALSE |
| SMARCA1 | 440 | 1.458809 | 0.144618 | 0.2788024 | FALSE |
| NPAP1 | 8633 | 1.458744 | 0.144636 | 0.2788025 | FALSE |
| PCYOX1L | 5239 | 1.458531 | 0.144694 | 0.2788812 | FALSE |
| C21orf58 | 10236 | 1.458047 | 0.144828 | 0.2790509 | FALSE |
| MARK4 | 3176 | 1.458017 | 0.144836 | 0.2790509 | FALSE |
| ABHD4 | 8093 | 1.457884 | 0.144873 | 0.2790871 | FALSE |
| IFI16 | 6589 | 1.457801 | 0.144895 | 0.2790968 | FALSE |
| PDLIM7 | 1401 | 1.457027 | 0.145109 | 0.279361 | FALSE |
| HTRA1 | 5941 | 1.45698 | 0.145122 | 0.279361 | FALSE |
| PPIL4 | 241 | 1.456609 | 0.145224 | 0.2795238 | FALSE |
| ENPP7 | 7019 | 1.455884 | 0.145425 | 0.2798061 | FALSE |
| KRT83 | 5095 | 1.45565 | 0.145489 | 0.2798617 | FALSE |
| LINC00909 | 8823 | 1.455537 | 0.145521 | 0.2798874 | FALSE |
| SBDSP1 | 8367 | 1.453862 | 0.145985 | 0.280671 | FALSE |
| RAB40B | 10521 | 1.452877 | 0.146258 | 0.2808833 | FALSE |
| TLR5 | 8935 | 1.452826 | 0.146272 | 0.2808833 | FALSE |
| ZMIZ2 | 368 | 1.452544 | 0.14635 | 0.2809791 | FALSE |
| GINM1 | 6866 | 1.452517 | 0.146358 | 0.2809791 | FALSE |
| NDUFB6 | 15382 | 1.452305 | 0.146417 | 0.2810577 | FALSE |
| MGST3 | 6795 | 1.452228 | 0.146438 | 0.2810643 | FALSE |
| C3orf70 | 11940 | 1.451837 | 0.146547 | 0.2812384 | FALSE |
| CHODL | 5724 | 1.451586 | 0.146617 | 0.2813379 | FALSE |
| NT5C3B | 8942 | 1.451389 | 0.146672 | 0.2814085 | FALSE |
| OLMALINC | 3117 | 1.451142 | 0.14674 | 0.2814694 | FALSE |
| CLCNKA | 4309 | 1.451113 | 0.146748 | 0.2814694 | FALSE |
| FBXL19 | 8217 | 1.451081 | 0.146757 | 0.2814694 | FALSE |
| PNPLA1 | 1549 | 1.450753 | 0.146849 | 0.2815937 | FALSE |
| ATP2C1 | 928 | 1.450719 | 0.146858 | 0.2815937 | FALSE |
| SCG2 | 4864 | 1.450157 | 0.147015 | 0.2817705 | FALSE |
| TAF9B | 6214 | 1.450102 | 0.14703 | 0.2817705 | FALSE |
| RPS7P5 | 14400 | 1.45 | 0.147059 | 0.2817705 | FALSE |
| CYP4F8 | 9540 | 1.449417 | 0.147221 | 0.2820476 | FALSE |
| DIO3 | 13579 | 1.448853 | 0.147379 | 0.2822455 | FALSE |
| MRPL14 | 2939 | 1.448343 | 0.147521 | 0.2824838 | FALSE |
| CMAS | 10191 | 1.447639 | 0.147718 | 0.2827916 | FALSE |
| MRGPRE | 889 | 1.44676 | 0.147964 | 0.2831587 | FALSE |
| CD109 | 7067 | 1.446436 | 0.148055 | 0.2832631 | FALSE |
| STMN2 | 6304 | 1.446237 | 0.148111 | 0.2833352 | FALSE |
| GFRA3 | 3056 | 1.445516 | 0.148313 | 0.2836324 | FALSE |
| ZNF569 | 14849 | 1.445395 | 0.148347 | 0.2836324 | FALSE |
| HIST2H2AB | 764 | 1.445383 | 0.14835 | 0.2836324 | FALSE |
| CDHR3 | 7059 | 1.445133 | 0.148421 | 0.2836848 | FALSE |
| TYRP1 | 7389 | 1.443675 | 0.14883 | 0.2843987 | FALSE |
| CENPU | 11800 | 1.443537 | 0.148869 | 0.2844276 | FALSE |
| OR10C1 | 665 | 1.443492 | 0.148882 | 0.2844276 | FALSE |
| ALS2CL | 5004 | 1.443213 | 0.14896 | 0.2845429 | FALSE |
| NRN1L | 11444 | 1.44188 | 0.149336 | 0.2851561 | FALSE |
| APLN | 10566 | 1.441395 | 0.149473 | 0.2853478 | FALSE |
| CAMK4 | 265 | 1.441186 | 0.149532 | 0.2854256 | FALSE |
| LYZL1 | 1625 | 1.440793 | 0.149643 | 0.2856027 | FALSE |
| PARD3B | 7548 | 1.440487 | 0.14973 | 0.285698 | FALSE |
| SLF1 | 2095 | 1.439954 | 0.14988 | 0.285815 | FALSE |
| PCDHGC5 | 6202 | 1.439947 | 0.149882 | 0.285815 | FALSE |
| ERAP2 | 12843 | 1.439312 | 0.150062 | 0.286088 | FALSE |
| KCNMB1 | 5922 | 1.439146 | 0.150109 | 0.2861427 | FALSE |
| CIB3 | 14657 | 1.438785 | 0.150211 | 0.2862679 | FALSE |
| CUTA | 14022 | 1.438557 | 0.150276 | 0.2863562 | FALSE |
| IL2RB | 2332 | 1.437462 | 0.150587 | 0.2869132 | FALSE |
| POLR2K | 6711 | 1.436738 | 0.150792 | 0.2872001 | FALSE |
| FABP1 | 8879 | 1.435775 | 0.151066 | 0.2875678 | FALSE |
| HDAC9 | 6908 | 1.435304 | 0.1512 | 0.2877669 | FALSE |
| 11-Sep | 1649 | 1.43506 | 0.15127 | 0.2878641 | FALSE |
| CSRP2 | 13905 | 1.434988 | 0.151291 | 0.2878681 | FALSE |
| ASB18 | 2970 | 1.434222 | 0.151509 | 0.2882486 | FALSE |
| ZNF667 | 8267 | 1.433386 | 0.151748 | 0.2886011 | FALSE |
| TMEM209 | 381 | 1.433089 | 0.151832 | 0.2886884 | FALSE |
| CD226 | 2069 | 1.432878 | 0.151893 | 0.2887328 | FALSE |
| PRR5 | 1781 | 1.432338 | 0.152047 | 0.2888885 | FALSE |
| UACA | 7530 | 1.431423 | 0.152309 | 0.2892526 | FALSE |
| LRFN2 | 3510 | 1.431281 | 0.15235 | 0.2892849 | FALSE |
| C3orf22 | 13532 | 1.430718 | 0.152511 | 0.2895561 | FALSE |
| TOMM20L | 1807 | 1.43028 | 0.152637 | 0.2897243 | FALSE |
| KIAA1143 | 5600 | 1.429984 | 0.152722 | 0.2898066 | FALSE |
| MORC4 | 12891 | 1.429393 | 0.152891 | 0.2900315 | FALSE |
| PYGO1 | 12598 | 1.429303 | 0.152917 | 0.2900454 | FALSE |
| CES1 | 8830 | 1.428884 | 0.153038 | 0.2902132 | FALSE |
| MORN3 | 6903 | 1.428866 | 0.153043 | 0.2902132 | FALSE |
| DOK7 | 7426 | 1.428482 | 0.153153 | 0.2903873 | FALSE |
| IFNL3 | 12586 | 1.42796 | 0.153303 | 0.2905664 | FALSE |
| CFDP1 | 3038 | 1.426666 | 0.153676 | 0.2911318 | FALSE |
| BARX1 | 14088 | 1.426121 | 0.153833 | 0.2913591 | FALSE |
| RPS14P3 | 9110 | 1.425811 | 0.153923 | 0.2914579 | FALSE |
| LINC01506 | 4946 | 1.425136 | 0.154118 | 0.2917001 | FALSE |
| KCNF1 | 10049 | 1.424949 | 0.154172 | 0.2917528 | FALSE |
| OLFML3 | 4999 | 1.423841 | 0.154493 | 0.2922887 | FALSE |
| MED15 | 1258 | 1.423289 | 0.154652 | 0.2925558 | FALSE |
| YWHAQ | 10431 | 1.4225 | 0.154881 | 0.2928473 | FALSE |
| TBC1D20 | 10607 | 1.421367 | 0.15521 | 0.2933623 | FALSE |
| NTRK3 | 5423 | 1.420672 | 0.155412 | 0.2936731 | FALSE |
| STAG3 | 15591 | 1.420553 | 0.155447 | 0.2937031 | FALSE |
| CABP4 | 3352 | 1.419926 | 0.155629 | 0.2939828 | FALSE |
| GAL3ST1 | 5495 | 1.419915 | 0.155632 | 0.2939828 | FALSE |
| SCMH1 | 1355 | 1.419129 | 0.155861 | 0.2943442 | FALSE |
| FOXA3 | 948 | 1.418665 | 0.155997 | 0.2945286 | FALSE |
| PLA1A | 6763 | 1.418486 | 0.156049 | 0.2945916 | FALSE |
| FUT8-AS1 | 13874 | 1.417812 | 0.156246 | 0.2949274 | FALSE |
| B3GALT4 | 9688 | 1.417681 | 0.156284 | 0.294964 | FALSE |
| MRPL57 | 10449 | 1.416812 | 0.156538 | 0.2953046 | FALSE |
| POLI | 4661 | 1.416573 | 0.156608 | 0.2953969 | FALSE |
| CRIP1 | 12085 | 1.416489 | 0.156632 | 0.2954077 | FALSE |
| MUC2 | 155 | 1.416078 | 0.156753 | 0.2955989 | FALSE |
| POLE2 | 2248 | 1.415744 | 0.15685 | 0.2957119 | FALSE |
| STXBP4 | 4996 | 1.414919 | 0.157092 | 0.2960607 | FALSE |
| LINC00643 | 14501 | 1.413276 | 0.157575 | 0.2968623 | FALSE |
| SMG8 | 1130 | 1.412605 | 0.157772 | 0.2971707 | FALSE |
| NDUFA7 | 9574 | 1.412304 | 0.15786 | 0.2972576 | FALSE |
| TOX4 | 2867 | 1.412108 | 0.157918 | 0.2973304 | FALSE |
| FOXD3 | 1572 | 1.411562 | 0.158079 | 0.2975974 | FALSE |
| SCD | 2489 | 1.40963 | 0.158649 | 0.2985985 | FALSE |
| FCHSD2 | 6884 | 1.409334 | 0.158736 | 0.2987272 | FALSE |
| CTAGE1 | 12690 | 1.409207 | 0.158774 | 0.2987619 | FALSE |
| KRT9 | 13295 | 1.409136 | 0.158795 | 0.2987654 | FALSE |
| PPP6R1 | 2730 | 1.408916 | 0.15886 | 0.2988518 | FALSE |
| CCDC103 | 2275 | 1.407905 | 0.159159 | 0.2992706 | FALSE |
| NMBR | 11093 | 1.407836 | 0.15918 | 0.299273 | FALSE |
| ARNT2 | 2750 | 1.407127 | 0.15939 | 0.299596 | FALSE |
| ZFPL1 | 5736 | 1.406997 | 0.159428 | 0.2996311 | FALSE |
| LINC01116 | 12300 | 1.40687 | 0.159466 | 0.2996311 | FALSE |
| LRP3 | 6523 | 1.404748 | 0.160096 | 0.3006348 | FALSE |
| EFNA4 | 10639 | 1.404618 | 0.160135 | 0.3006713 | FALSE |
| GYPC | 13508 | 1.404296 | 0.160231 | 0.3007789 | FALSE |
| CASP8 | 12425 | 1.403869 | 0.160358 | 0.3009548 | FALSE |
| RBP1 | 13740 | 1.403852 | 0.160363 | 0.3009548 | FALSE |
| NR2F2-AS1 | 5136 | 1.40338 | 0.160504 | 0.3011464 | FALSE |
| TMEM206 | 2085 | 1.402932 | 0.160637 | 0.3012543 | FALSE |
| ZNF827 | 2367 | 1.402886 | 0.160651 | 0.3012543 | FALSE |
| C15orf40 | 5978 | 1.402868 | 0.160656 | 0.3012543 | FALSE |
| LINC01144 | 4974 | 1.402864 | 0.160657 | 0.3012543 | FALSE |
| ODC1 | 8546 | 1.402795 | 0.160678 | 0.3012568 | FALSE |
| DEDD2 | 362 | 1.402609 | 0.160733 | 0.3013247 | FALSE |
| BTBD2 | 5629 | 1.40253 | 0.160757 | 0.3013327 | FALSE |
| TRIM11 | 6256 | 1.402255 | 0.160839 | 0.3014143 | FALSE |
| HLA-B | 10573 | 1.401585 | 0.161039 | 0.3017531 | FALSE |
| MMP14 | 9605 | 1.401116 | 0.161179 | 0.3019763 | FALSE |
| PSEN1 | 2676 | 1.400928 | 0.161236 | 0.3019763 | FALSE |
| ADAT3 | 10063 | 1.399986 | 0.161518 | 0.302296 | FALSE |
| FLJ30901 | 8348 | 1.39997 | 0.161522 | 0.302296 | FALSE |
| OTUB1 | 6965 | 1.399443 | 0.16168 | 0.3025552 | FALSE |
| PDZRN4 | 1593 | 1.398453 | 0.161977 | 0.303002 | FALSE |
| IFI6 | 15386 | 1.397541 | 0.162251 | 0.3033764 | FALSE |
| TRIM5 | 4232 | 1.397528 | 0.162255 | 0.3033764 | FALSE |
| TMEM151A | 7509 | 1.397317 | 0.162318 | 0.3034587 | FALSE |
| PRSS58 | 1752 | 1.397251 | 0.162338 | 0.3034595 | FALSE |
| SYT5 | 10116 | 1.396794 | 0.162476 | 0.3036437 | FALSE |
| SPRR1A | 14581 | 1.396233 | 0.162644 | 0.3039229 | FALSE |
| PIK3AP1 | 6433 | 1.395775 | 0.162782 | 0.3041443 | FALSE |
| LYPD2 | 9840 | 1.395175 | 0.162963 | 0.3044457 | FALSE |
| RNF122 | 327 | 1.394935 | 0.163035 | 0.3045445 | FALSE |
| TBCD | 6289 | 1.394692 | 0.163109 | 0.3046037 | FALSE |
| SNX10 | 10302 | 1.394633 | 0.163127 | 0.3046037 | FALSE |
| TWIST1 | 11348 | 1.394559 | 0.163149 | 0.3046037 | FALSE |
| COL1A2 | 10414 | 1.394543 | 0.163154 | 0.3046037 | FALSE |
| HELZ2 | 13522 | 1.393901 | 0.163348 | 0.304836 | FALSE |
| DDIT4 | 8479 | 1.393009 | 0.163617 | 0.3053027 | FALSE |
| ARRDC1 | 12869 | 1.392573 | 0.163749 | 0.3055123 | FALSE |
| SIVA1 | 6649 | 1.392216 | 0.163857 | 0.3056409 | FALSE |
| CAPN10 | 5312 | 1.39159 | 0.164047 | 0.3059215 | FALSE |
| ZDHHC24 | 11419 | 1.391034 | 0.164215 | 0.3060898 | FALSE |
| TMEM218 | 8384 | 1.390728 | 0.164308 | 0.3061235 | FALSE |
| TNFRSF10B | 8798 | 1.390716 | 0.164312 | 0.3061235 | FALSE |
| ZMIZ1 | 13844 | 1.390133 | 0.164488 | 0.3062707 | FALSE |
| RAE1 | 7046 | 1.389041 | 0.16482 | 0.3068154 | FALSE |
| SLC18A2 | 4598 | 1.388749 | 0.164909 | 0.3069076 | FALSE |
| FAT1 | 3373 | 1.388669 | 0.164933 | 0.3069163 | FALSE |
| APCDD1L | 5339 | 1.388593 | 0.164957 | 0.3069228 | FALSE |
| IFNAR1 | 9178 | 1.388492 | 0.164987 | 0.3069435 | FALSE |
| LGALS3BP | 11028 | 1.38809 | 0.16511 | 0.307098 | FALSE |
| MCMBP | 12206 | 1.387671 | 0.165237 | 0.3072988 | FALSE |
| ACRBP | 14880 | 1.386299 | 0.165656 | 0.3078206 | FALSE |
| LOC105379807 | 1175 | 1.385953 | 0.165761 | 0.3079436 | FALSE |
| ZNF703 | 11201 | 1.384609 | 0.166172 | 0.3086619 | FALSE |
| C14orf144 | 9356 | 1.384559 | 0.166187 | 0.3086619 | FALSE |
| PSKH1 | 4944 | 1.383833 | 0.16641 | 0.3090379 | FALSE |
| UQCR10 | 14363 | 1.383578 | 0.166488 | 0.3090808 | FALSE |
| HSPD1 | 8979 | 1.383565 | 0.166492 | 0.3090808 | FALSE |
| RPL22 | 12378 | 1.383564 | 0.166492 | 0.3090808 | FALSE |
| FLT3LG | 7833 | 1.383209 | 0.166601 | 0.3091925 | FALSE |
| ENSA | 15130 | 1.380463 | 0.167444 | 0.3102726 | FALSE |
| DDC | 8601 | 1.380439 | 0.167452 | 0.3102726 | FALSE |
| ANKIB1 | 6042 | 1.380315 | 0.16749 | 0.3103065 | FALSE |
| TP53INP1 | 14743 | 1.379285 | 0.167807 | 0.3107469 | FALSE |
| HNRNPD | 12427 | 1.378892 | 0.167928 | 0.3108808 | FALSE |
| RAB8A | 12519 | 1.378498 | 0.16805 | 0.3110121 | FALSE |
| CYBA | 4809 | 1.37736 | 0.168401 | 0.3115518 | FALSE |
| TSPAN3 | 6196 | 1.376052 | 0.168806 | 0.3121173 | FALSE |
| RAB10 | 7211 | 1.376049 | 0.168806 | 0.3121173 | FALSE |
| PPAT | 2654 | 1.37597 | 0.168831 | 0.3121256 | FALSE |
| GDF15 | 4788 | 1.375378 | 0.169014 | 0.3124276 | FALSE |
| EIF2S2 | 4129 | 1.375038 | 0.16912 | 0.3125485 | FALSE |
| GSX1 | 13690 | 1.374597 | 0.169256 | 0.3126903 | FALSE |
| WBP11P1 | 1866 | 1.374388 | 0.169321 | 0.3127732 | FALSE |
| TRPV6 | 1368 | 1.373625 | 0.169558 | 0.3130627 | FALSE |
| TJP2 | 10257 | 1.373383 | 0.169633 | 0.3131073 | FALSE |
| SNRPF | 9886 | 1.373354 | 0.169642 | 0.3131073 | FALSE |
| EPHA10 | 15292 | 1.37228 | 0.169976 | 0.3136866 | FALSE |
| MSX1 | 13972 | 1.37159 | 0.170191 | 0.3139348 | FALSE |
| INCENP | 3341 | 1.371498 | 0.17022 | 0.3139506 | FALSE |
| ALKBH1 | 10695 | 1.371267 | 0.170292 | 0.3140401 | FALSE |
| VGF | 14474 | 1.371078 | 0.170351 | 0.314041 | FALSE |
| CSAG4 | 13371 | 1.370954 | 0.170389 | 0.314041 | FALSE |
| LALBA | 167 | 1.370541 | 0.170518 | 0.3142413 | FALSE |
| CLCC1 | 5732 | 1.370217 | 0.170619 | 0.3143535 | FALSE |
| LOC149373 | 8814 | 1.369299 | 0.170906 | 0.3148446 | FALSE |
| ZNF286A | 9187 | 1.368283 | 0.171224 | 0.3153555 | FALSE |
| GIT1 | 13351 | 1.367769 | 0.171384 | 0.3155982 | FALSE |
| C19orf48 | 12125 | 1.367507 | 0.171466 | 0.3156913 | FALSE |
| KRTAP6-3 | 6551 | 1.367111 | 0.171591 | 0.3158453 | FALSE |
| DHX30 | 10542 | 1.365636 | 0.172053 | 0.3165578 | FALSE |
| TSC22D4 | 7989 | 1.365619 | 0.172059 | 0.3165578 | FALSE |
| IFI27L1 | 5901 | 1.365234 | 0.17218 | 0.3167058 | FALSE |
| MPLKIP | 9906 | 1.364798 | 0.172317 | 0.316889 | FALSE |
| AGTPBP1 | 3071 | 1.364788 | 0.17232 | 0.316889 | FALSE |
| WDR44 | 228 | 1.364676 | 0.172355 | 0.3169165 | FALSE |
| FNDC3A | 6864 | 1.3642 | 0.172505 | 0.3171172 | FALSE |
| BRD3OS | 6261 | 1.363991 | 0.17257 | 0.3172008 | FALSE |
| MFSD4A | 13856 | 1.363909 | 0.172596 | 0.317211 | FALSE |
| RAD1 | 2962 | 1.363129 | 0.172842 | 0.3175933 | FALSE |
| CCL24 | 8962 | 1.363055 | 0.172865 | 0.3175933 | FALSE |
| OR52N2 | 12065 | 1.362523 | 0.173033 | 0.3177147 | FALSE |
| SSX5 | 10982 | 1.361671 | 0.173302 | 0.318171 | FALSE |
| NKX2-2 | 13563 | 1.361258 | 0.173432 | 0.3183047 | FALSE |
| POLR2H | 1694 | 1.361247 | 0.173436 | 0.3183047 | FALSE |
| SLC35G3 | 8693 | 1.361175 | 0.173458 | 0.3183091 | FALSE |
| SOD3 | 8980 | 1.36107 | 0.173492 | 0.3183326 | FALSE |
| TREX1 | 7799 | 1.36088 | 0.173552 | 0.3184054 | FALSE |
| DNAJC1 | 13555 | 1.360806 | 0.173575 | 0.3184109 | FALSE |
| NOL9 | 6525 | 1.360315 | 0.17373 | 0.3186413 | FALSE |
| ARFIP2 | 13340 | 1.36028 | 0.173741 | 0.3186413 | FALSE |
| BNIP2 | 14255 | 1.359787 | 0.173897 | 0.31889 | FALSE |
| NDFIP2 | 15496 | 1.359265 | 0.174063 | 0.3190515 | FALSE |
| KCTD5 | 2008 | 1.358918 | 0.174173 | 0.3191183 | FALSE |
| C2orf40 | 2448 | 1.357765 | 0.174538 | 0.3196528 | FALSE |
| LINC01278 | 4148 | 1.357624 | 0.174583 | 0.3196974 | FALSE |
| SRR | 7925 | 1.356387 | 0.174976 | 0.320267 | FALSE |
| ZNF488 | 12054 | 1.356179 | 0.175042 | 0.3203506 | FALSE |
| ATCAY | 9498 | 1.355918 | 0.175125 | 0.3204651 | FALSE |
| BCAR3 | 8567 | 1.355766 | 0.175174 | 0.3204943 | FALSE |
| TRNAU1AP | 1112 | 1.355459 | 0.175271 | 0.3206198 | FALSE |
| KLHL25 | 8615 | 1.355329 | 0.175313 | 0.3206581 | FALSE |
| CDKN1C | 13344 | 1.35513 | 0.175376 | 0.3206989 | FALSE |
| LPP | 1047 | 1.354839 | 0.175469 | 0.320831 | FALSE |
| MIEF1 | 9273 | 1.354208 | 0.17567 | 0.3211612 | FALSE |
| GLYCTK | 15490 | 1.353271 | 0.175969 | 0.321457 | FALSE |
| PINLYP | 14212 | 1.352931 | 0.176078 | 0.3216056 | FALSE |
| CAMSAP1 | 5550 | 1.35272 | 0.176145 | 0.3216912 | FALSE |
| NDUFB11 | 4 | 1.352606 | 0.176182 | 0.3217201 | FALSE |
| TMEM121 | 14652 | 1.352455 | 0.17623 | 0.3217586 | FALSE |
| SLC13A5 | 907 | 1.351868 | 0.176418 | 0.3219631 | FALSE |
| PRF1 | 12032 | 1.351459 | 0.176548 | 0.3221643 | FALSE |
| RHPN2 | 8754 | 1.351169 | 0.176641 | 0.3222577 | FALSE |
| CBX1 | 12649 | 1.350951 | 0.176711 | 0.3223 | FALSE |
| C1QBP | 12546 | 1.350905 | 0.176726 | 0.3223 | FALSE |
| PKD2L1 | 3969 | 1.350664 | 0.176803 | 0.3223578 | FALSE |
| DNAL1 | 9884 | 1.349296 | 0.177242 | 0.3230527 | FALSE |
| TSPO2 | 483 | 1.348898 | 0.17737 | 0.323248 | FALSE |
| SEC22B | 458 | 1.348649 | 0.17745 | 0.3233561 | FALSE |
| ATP5PF | 3398 | 1.348134 | 0.177615 | 0.3235823 | FALSE |
| LINC00294 | 11489 | 1.347995 | 0.17766 | 0.323626 | FALSE |
| TSGA10 | 15043 | 1.347924 | 0.177683 | 0.3236299 | FALSE |
| HAX1 | 12908 | 1.347666 | 0.177766 | 0.3237434 | FALSE |
| FAM45BP | 10249 | 1.347554 | 0.177802 | 0.3237713 | FALSE |
| LINC01197 | 6189 | 1.347247 | 0.177901 | 0.3238615 | FALSE |
| CRCT1 | 10370 | 1.347207 | 0.177914 | 0.3238615 | FALSE |
| YKT6 | 11194 | 1.347059 | 0.177961 | 0.3239106 | FALSE |
| SORCS3 | 9961 | 1.345868 | 0.178345 | 0.3244581 | FALSE |
| YIF1B | 4250 | 1.345605 | 0.17843 | 0.3245747 | FALSE |
| DCP2 | 6187 | 1.345467 | 0.178474 | 0.3245802 | FALSE |
| LCE3E | 3401 | 1.343384 | 0.179148 | 0.3256151 | FALSE |
| FAM129B | 9758 | 1.342852 | 0.17932 | 0.3258096 | FALSE |
| ARAP1 | 10218 | 1.342796 | 0.179338 | 0.3258096 | FALSE |
| L1CAM | 4878 | 1.342246 | 0.179516 | 0.3259818 | FALSE |
| MRPL41 | 6125 | 1.341548 | 0.179743 | 0.326317 | FALSE |
| CHST7 | 2808 | 1.341442 | 0.179777 | 0.3263416 | FALSE |
| PRELID2 | 2115 | 1.341352 | 0.179806 | 0.3263567 | FALSE |
| TRAM1L1 | 6683 | 1.341139 | 0.179875 | 0.3264443 | FALSE |
| C15orf65 | 5454 | 1.340868 | 0.179963 | 0.326566 | FALSE |
| SYT6 | 3202 | 1.340741 | 0.180005 | 0.326603 | FALSE |
| PCED1A | 5722 | 1.34064 | 0.180037 | 0.3266246 | FALSE |
| PGGHG | 14036 | 1.340375 | 0.180123 | 0.3267429 | FALSE |
| FRG1 | 10288 | 1.339977 | 0.180253 | 0.3269396 | FALSE |
| CCNB3 | 1427 | 1.339644 | 0.180361 | 0.3270607 | FALSE |
| PPBP | 11653 | 1.337066 | 0.181201 | 0.328338 | FALSE |
| SPRED3 | 7317 | 1.33681 | 0.181285 | 0.3284301 | FALSE |
| MPP6 | 14612 | 1.335037 | 0.181864 | 0.3292893 | FALSE |
| ENPP4 | 7290 | 1.333895 | 0.182238 | 0.3297374 | FALSE |
| CRYZL1 | 5612 | 1.333477 | 0.182375 | 0.329909 | FALSE |
| LTBP1 | 5677 | 1.33229 | 0.182765 | 0.3303844 | FALSE |
| SCD5 | 13156 | 1.332004 | 0.182859 | 0.330516 | FALSE |
| CD37 | 14325 | 1.331529 | 0.183015 | 0.3307217 | FALSE |
| LASP1 | 7494 | 1.331157 | 0.183137 | 0.3308281 | FALSE |
| HGD | 5301 | 1.330634 | 0.183309 | 0.3310132 | FALSE |
| PEX11A | 5199 | 1.330626 | 0.183312 | 0.3310132 | FALSE |
| CYP4X1 | 8490 | 1.330524 | 0.183346 | 0.3310132 | FALSE |
| MAST2 | 6931 | 1.330384 | 0.183392 | 0.3310582 | FALSE |
| NAT16 | 13635 | 1.330311 | 0.183416 | 0.3310633 | FALSE |
| CITED4 | 14508 | 1.330144 | 0.183471 | 0.3311244 | FALSE |
| SLCO2B1 | 13862 | 1.329395 | 0.183718 | 0.3314169 | FALSE |
| ITGAV | 6728 | 1.328458 | 0.184027 | 0.331898 | FALSE |
| PLPP5 | 3664 | 1.328295 | 0.184081 | 0.3319568 | FALSE |
| GFRAL | 7205 | 1.327928 | 0.184202 | 0.3320664 | FALSE |
| PITX3 | 9594 | 1.327821 | 0.184237 | 0.3320859 | FALSE |
| TAS2R60 | 1141 | 1.327295 | 0.184411 | 0.332361 | FALSE |
| KRT15 | 4136 | 1.326875 | 0.18455 | 0.3325347 | FALSE |
| SELENOH | 401 | 1.32655 | 0.184658 | 0.3326518 | FALSE |
| GHRL | 10748 | 1.325946 | 0.184858 | 0.3329353 | FALSE |
| FBXL7 | 4201 | 1.32464 | 0.185291 | 0.3336383 | FALSE |
| PPIH | 3252 | 1.32456 | 0.185317 | 0.3336477 | FALSE |
| PTPN22 | 13073 | 1.32396 | 0.185516 | 0.3339425 | FALSE |
| BAAT | 11993 | 1.323938 | 0.185524 | 0.3339425 | FALSE |
| TUSC1 | 5636 | 1.323832 | 0.185559 | 0.3339595 | FALSE |
| GNAO1 | 1214 | 1.323716 | 0.185597 | 0.3339599 | FALSE |
| LOC728989 | 7911 | 1.323596 | 0.185637 | 0.3339932 | FALSE |
| COL6A3 | 7349 | 1.32345 | 0.185686 | 0.3340421 | FALSE |
| GJB3 | 11871 | 1.32321 | 0.185766 | 0.3341428 | FALSE |
| HIST1H2AM | 14722 | 1.323153 | 0.185785 | 0.3341428 | FALSE |
| CD6 | 5228 | 1.322982 | 0.185841 | 0.3342066 | FALSE |
| FAM27E2 | 6091 | 1.322697 | 0.185936 | 0.3343386 | FALSE |
| INHBC | 11631 | 1.322416 | 0.18603 | 0.3344683 | FALSE |
| SUMO1P1 | 13452 | 1.322181 | 0.186108 | 0.3345344 | FALSE |
| GNG5 | 7352 | 1.321974 | 0.186177 | 0.3346174 | FALSE |
| LOC148709 | 7064 | 1.321165 | 0.186446 | 0.3349863 | FALSE |
| ZNF286B | 3069 | 1.320341 | 0.186721 | 0.3353645 | FALSE |
| CATSPERE | 2052 | 1.319941 | 0.186855 | 0.3355657 | FALSE |
| C20orf194 | 1942 | 1.319612 | 0.186965 | 0.3357245 | FALSE |
| IGSF11 | 9631 | 1.319339 | 0.187056 | 0.3357726 | FALSE |
| MYH3 | 7050 | 1.319126 | 0.187127 | 0.3358618 | FALSE |
| AQP2 | 12016 | 1.317767 | 0.187582 | 0.3365619 | FALSE |
| CTBS | 7890 | 1.317479 | 0.187678 | 0.3366963 | FALSE |
| RBSN | 5634 | 1.317193 | 0.187774 | 0.336791 | FALSE |
| FAIM | 11314 | 1.316841 | 0.187892 | 0.3369639 | FALSE |
| SEMA4B | 3689 | 1.316032 | 0.188163 | 0.3373393 | FALSE |
| AXL | 3107 | 1.316024 | 0.188166 | 0.3373393 | FALSE |
| WBP11 | 12482 | 1.315808 | 0.188238 | 0.3374306 | FALSE |
| EFCAB6 | 10634 | 1.315348 | 0.188393 | 0.3376301 | FALSE |
| TMEM105 | 13317 | 1.314722 | 0.188603 | 0.3378909 | FALSE |
| MRPS34 | 1877 | 1.314537 | 0.188666 | 0.3379249 | FALSE |
| YEATS2 | 5035 | 1.314204 | 0.188778 | 0.3380401 | FALSE |
| BLID | 14746 | 1.314153 | 0.188795 | 0.3380401 | FALSE |
| SNAI1 | 10532 | 1.313819 | 0.188907 | 0.3381639 | FALSE |
| GOSR2 | 15633 | 1.312707 | 0.189282 | 0.3386405 | FALSE |
| ARGLU1 | 3699 | 1.312376 | 0.189393 | 0.3387239 | FALSE |
| TPM1 | 13377 | 1.310861 | 0.189905 | 0.3394444 | FALSE |
| ZSCAN22 | 2963 | 1.310471 | 0.190037 | 0.3396024 | FALSE |
| EFTUD2 | 13345 | 1.310168 | 0.190139 | 0.339669 | FALSE |
| ISM2 | 9217 | 1.310095 | 0.190164 | 0.3396743 | FALSE |
| LSM10 | 8891 | 1.309899 | 0.19023 | 0.3397199 | FALSE |
| TMEM74 | 11681 | 1.30962 | 0.190324 | 0.3398151 | FALSE |
| VENTX | 12202 | 1.309242 | 0.190452 | 0.3398983 | FALSE |
| CLUL1 | 8027 | 1.309169 | 0.190477 | 0.3398983 | FALSE |
| REXO5 | 7558 | 1.309082 | 0.190507 | 0.3398983 | FALSE |
| LOC100128164 | 1240 | 1.30869 | 0.190639 | 0.3400965 | FALSE |
| AGER | 2744 | 1.308487 | 0.190708 | 0.3401804 | FALSE |
| PCYT1B | 1253 | 1.308306 | 0.19077 | 0.3402418 | FALSE |
| ARHGAP33 | 12939 | 1.307571 | 0.191019 | 0.3405403 | FALSE |
| RASA4CP | 15214 | 1.307038 | 0.1912 | 0.3407589 | FALSE |
| HBB | 8441 | 1.306757 | 0.191295 | 0.3408387 | FALSE |
| SCRT2 | 642 | 1.30665 | 0.191332 | 0.3408646 | FALSE |
| PPM1F | 14494 | 1.306425 | 0.191408 | 0.3409231 | FALSE |
| GPR78 | 7035 | 1.305662 | 0.191668 | 0.3413464 | FALSE |
| COL6A6 | 3161 | 1.305449 | 0.19174 | 0.3414365 | FALSE |
| SHANK2-AS3 | 9833 | 1.305143 | 0.191844 | 0.3415053 | FALSE |
| DCTN2 | 5103 | 1.304998 | 0.191894 | 0.3415543 | FALSE |
| GPAM | 2539 | 1.30487 | 0.191937 | 0.341593 | FALSE |
| NCCRP1 | 2189 | 1.304023 | 0.192226 | 0.3419899 | FALSE |
| TRIM69 | 4680 | 1.303073 | 0.19255 | 0.3425256 | FALSE |
| BMPR2 | 760 | 1.303012 | 0.192571 | 0.3425256 | FALSE |
| ZDHHC14 | 8820 | 1.302858 | 0.192623 | 0.3425801 | FALSE |
| TNFRSF8 | 617 | 1.302731 | 0.192667 | 0.3426183 | FALSE |
| FAM57A | 5669 | 1.301659 | 0.193033 | 0.3431917 | FALSE |
| MED4 | 11651 | 1.301428 | 0.193112 | 0.3432619 | FALSE |
| ATF7IP2 | 6618 | 1.30137 | 0.193132 | 0.3432619 | FALSE |
| WDTC1 | 3213 | 1.300269 | 0.193509 | 0.343803 | FALSE |
| KCTD21 | 7445 | 1.299099 | 0.19391 | 0.3443592 | FALSE |
| EEF1A1 | 6906 | 1.298366 | 0.194162 | 0.3446886 | FALSE |
| LINC00158 | 10977 | 1.298125 | 0.194244 | 0.3447346 | FALSE |
| LOC100130264 | 10831 | 1.298111 | 0.194249 | 0.3447346 | FALSE |
| LOC100132831 | 1434 | 1.298098 | 0.194254 | 0.3447346 | FALSE |
| BCORP1 | 15152 | 1.29795 | 0.194305 | 0.3447466 | FALSE |
| ARID3A | 11508 | 1.296825 | 0.194691 | 0.3452372 | FALSE |
| BRAT1 | 11580 | 1.296481 | 0.19481 | 0.3453688 | FALSE |
| GH2 | 3241 | 1.296137 | 0.194928 | 0.345452 | FALSE |
| LOC105374952 | 2395 | 1.296128 | 0.194931 | 0.345452 | FALSE |
| PPARA | 8173 | 1.296103 | 0.19494 | 0.345452 | FALSE |
| SLC25A42 | 13633 | 1.296088 | 0.194945 | 0.345452 | FALSE |
| VIPAS39 | 3970 | 1.295997 | 0.194977 | 0.3454684 | FALSE |
| LOC643201 | 12307 | 1.295923 | 0.195002 | 0.3454744 | FALSE |
| DCAF12L2 | 10326 | 1.295722 | 0.195071 | 0.3455512 | FALSE |
| PALM3 | 12893 | 1.295321 | 0.19521 | 0.3456854 | FALSE |
| SOCS1 | 12682 | 1.294713 | 0.195419 | 0.3460176 | FALSE |
| NAP1L6 | 3636 | 1.294024 | 0.195657 | 0.3463995 | FALSE |
| UBE2K | 1823 | 1.293757 | 0.195749 | 0.3465236 | FALSE |
| LINC01881 | 9169 | 1.293466 | 0.19585 | 0.3466231 | FALSE |
| TMEM63C | 5324 | 1.293038 | 0.195998 | 0.3468458 | FALSE |
| CAPN5 | 3432 | 1.292735 | 0.196103 | 0.346992 | FALSE |
| OTUD1 | 12271 | 1.292404 | 0.196217 | 0.3471161 | FALSE |
| CST5 | 5697 | 1.291831 | 0.196416 | 0.3474277 | FALSE |
| MRPL30 | 1978 | 1.291276 | 0.196608 | 0.3477257 | FALSE |
| HHEX | 5076 | 1.291205 | 0.196633 | 0.3477257 | FALSE |
| PLA2G12B | 2201 | 1.291051 | 0.196686 | 0.3477257 | FALSE |
| AKAP8 | 1643 | 1.290399 | 0.196912 | 0.3480696 | FALSE |
| PROCA1 | 3240 | 1.290329 | 0.196936 | 0.3480732 | FALSE |
| PITPNB | 5691 | 1.289377 | 0.197267 | 0.3485657 | FALSE |
| WASF3 | 8211 | 1.288717 | 0.197496 | 0.3488338 | FALSE |
| BUD13 | 4763 | 1.288705 | 0.197501 | 0.3488338 | FALSE |
| MFSD1 | 6667 | 1.287568 | 0.197896 | 0.3493748 | FALSE |
| HOMER1 | 11006 | 1.2872 | 0.198025 | 0.3495222 | FALSE |
| APEX2 | 375 | 1.287042 | 0.19808 | 0.3495799 | FALSE |
| PYY2 | 2354 | 1.286846 | 0.198148 | 0.3496554 | FALSE |
| CACYBP | 1031 | 1.28613 | 0.198398 | 0.3499832 | FALSE |
| PSD2 | 14718 | 1.286038 | 0.19843 | 0.3500003 | FALSE |
| ATP5MC1 | 2207 | 1.28574 | 0.198534 | 0.3500585 | FALSE |
| TMEM116 | 539 | 1.285687 | 0.198552 | 0.3500585 | FALSE |
| CYP51A1 | 10477 | 1.285517 | 0.198612 | 0.3500971 | FALSE |
| NUDT16 | 135 | 1.28526 | 0.198701 | 0.3502029 | FALSE |
| RNF151 | 141 | 1.285163 | 0.198735 | 0.3502231 | FALSE |
| SLC25A47 | 11080 | 1.284924 | 0.198819 | 0.3502913 | FALSE |
| NDUFAB1 | 7536 | 1.284639 | 0.198918 | 0.3503527 | FALSE |
| MMP17 | 1944 | 1.28419 | 0.199075 | 0.350546 | FALSE |
| PRSS21 | 2914 | 1.284039 | 0.199128 | 0.3505995 | FALSE |
| RHEB | 880 | 1.283773 | 0.199221 | 0.3507239 | FALSE |
| SLC26A2 | 12745 | 1.28364 | 0.199268 | 0.3507663 | FALSE |
| AHR | 9189 | 1.283454 | 0.199333 | 0.3508415 | FALSE |
| WFDC12 | 12197 | 1.283136 | 0.199444 | 0.350998 | FALSE |
| FAM50B | 1728 | 1.282653 | 0.199614 | 0.3511957 | FALSE |
| ZCCHC24 | 11724 | 1.282623 | 0.199624 | 0.3511957 | FALSE |
| USP7 | 7598 | 1.281808 | 0.19991 | 0.3516194 | FALSE |
| P4HB | 6922 | 1.281493 | 0.200021 | 0.3517346 | FALSE |
| SHANK2 | 1723 | 1.281438 | 0.20004 | 0.3517346 | FALSE |
| SURF2 | 1789 | 1.281429 | 0.200043 | 0.3517346 | FALSE |
| MYEOV | 7807 | 1.281296 | 0.20009 | 0.351774 | FALSE |
| PIR | 10861 | 1.281237 | 0.20011 | 0.351774 | FALSE |
| NSD2 | 83 | 1.280758 | 0.200279 | 0.3519411 | FALSE |
| PXN-AS1 | 7700 | 1.280172 | 0.200485 | 0.3521547 | FALSE |
| ZNF735 | 8896 | 1.280004 | 0.200544 | 0.3521793 | FALSE |
| CYP3A5 | 15058 | 1.279784 | 0.200621 | 0.3522484 | FALSE |
| GSN | 5868 | 1.279764 | 0.200628 | 0.3522484 | FALSE |
| C11orf58 | 6597 | 1.279429 | 0.200746 | 0.3523775 | FALSE |
| NUFIP2 | 13529 | 1.279427 | 0.200747 | 0.3523775 | FALSE |
| KLHL22 | 4935 | 1.279189 | 0.200831 | 0.3524454 | FALSE |
| UBAP2 | 740 | 1.278702 | 0.201002 | 0.3526276 | FALSE |
| RRP7BP | 7905 | 1.278472 | 0.201083 | 0.3527302 | FALSE |
| EVI5L | 6335 | 1.278124 | 0.201206 | 0.3529057 | FALSE |
| FOXK2 | 15384 | 1.27724 | 0.201518 | 0.353413 | FALSE |
| SPIN2B | 6299 | 1.27653 | 0.201768 | 0.3537336 | FALSE |
| TP53I13 | 13572 | 1.276351 | 0.201831 | 0.3538048 | FALSE |
| CCDC80 | 4542 | 1.276189 | 0.201889 | 0.3538655 | FALSE |
| NRG3 | 2257 | 1.275618 | 0.202091 | 0.3541455 | FALSE |
| GRIN2B | 1857 | 1.275609 | 0.202094 | 0.3541455 | FALSE |
| CNR2 | 2200 | 1.274142 | 0.202613 | 0.3548567 | FALSE |
| STRADB | 8799 | 1.273011 | 0.203014 | 0.3554128 | FALSE |
| SOGA1 | 14125 | 1.272867 | 0.203065 | 0.3554494 | FALSE |
| RTN1 | 1294 | 1.272568 | 0.203171 | 0.3555954 | FALSE |
| OPA1 | 7711 | 1.271804 | 0.203443 | 0.3559755 | FALSE |
| NUS1 | 13267 | 1.271692 | 0.203483 | 0.3559755 | FALSE |
| XK | 14707 | 1.27163 | 0.203505 | 0.3559755 | FALSE |
| CARMIL1 | 2529 | 1.271579 | 0.203523 | 0.3559755 | FALSE |
| WDR82 | 11394 | 1.271495 | 0.203553 | 0.3559755 | FALSE |
| GP9 | 15569 | 1.271426 | 0.203577 | 0.3559755 | FALSE |
| NT5C | 2836 | 1.271391 | 0.20359 | 0.3559755 | FALSE |
| LOC441204 | 11335 | 1.27138 | 0.203593 | 0.3559755 | FALSE |
| LMOD1 | 2210 | 1.270975 | 0.203738 | 0.3561875 | FALSE |
| ABCB8 | 14613 | 1.270338 | 0.203964 | 0.356544 | FALSE |
| TPTEP1 | 4454 | 1.269228 | 0.20436 | 0.356956 | FALSE |
| ADAM29 | 8977 | 1.268979 | 0.204449 | 0.3570712 | FALSE |
| ACP5 | 1985 | 1.268685 | 0.204553 | 0.3572145 | FALSE |
| LINC01783 | 13027 | 1.268429 | 0.204645 | 0.3572543 | FALSE |
| STMN1 | 11215 | 1.268222 | 0.204719 | 0.3573434 | FALSE |
| FIP1L1 | 9407 | 1.267005 | 0.205154 | 0.3579425 | FALSE |
| RAB5A | 8548 | 1.266267 | 0.205418 | 0.3583231 | FALSE |
| MEGF9 | 4883 | 1.264742 | 0.205964 | 0.3591158 | FALSE |
| TBL2 | 13465 | 1.264638 | 0.206001 | 0.3591239 | FALSE |
| DPF3 | 15329 | 1.264601 | 0.206014 | 0.3591239 | FALSE |
| FBXW4 | 12311 | 1.263753 | 0.206319 | 0.3595742 | FALSE |
| SPRYD7 | 9260 | 1.263569 | 0.206385 | 0.3596091 | FALSE |
| HEATR5A | 3796 | 1.263167 | 0.206529 | 0.3598206 | FALSE |
| GGN | 10589 | 1.262659 | 0.206712 | 0.3600585 | FALSE |
| GPR39 | 2975 | 1.262456 | 0.206785 | 0.3601455 | FALSE |
| CCT8 | 9031 | 1.262362 | 0.206819 | 0.3601643 | FALSE |
| EIF2B4 | 7565 | 1.262203 | 0.206876 | 0.3601836 | FALSE |
| CCR3 | 5671 | 1.261789 | 0.207025 | 0.3604015 | FALSE |
| PRKN | 14294 | 1.261686 | 0.207062 | 0.3604015 | FALSE |
| FBLN1 | 9118 | 1.261599 | 0.207093 | 0.3604015 | FALSE |
| HLA-DPB1 | 3256 | 1.260822 | 0.207373 | 0.360758 | FALSE |
| PGAM4 | 161 | 1.260812 | 0.207377 | 0.360758 | FALSE |
| HGSNAT | 3777 | 1.260774 | 0.20739 | 0.360758 | FALSE |
| LTA | 1565 | 1.260604 | 0.207452 | 0.3608245 | FALSE |
| SNRPG | 577 | 1.26026 | 0.207576 | 0.361 | FALSE |
| LINC01869 | 4518 | 1.259292 | 0.207925 | 0.3614165 | FALSE |
| EXOSC8 | 14567 | 1.259224 | 0.207949 | 0.3614165 | FALSE |
| NDUFV3 | 11563 | 1.258877 | 0.208075 | 0.3615464 | FALSE |
| LRRTM2 | 6381 | 1.258185 | 0.208325 | 0.3618848 | FALSE |
| SERPINA3 | 13424 | 1.25748 | 0.20858 | 0.3621825 | FALSE |
| SH3BP4 | 5902 | 1.257126 | 0.208708 | 0.3623648 | FALSE |
| DHH | 15313 | 1.256931 | 0.208779 | 0.3624069 | FALSE |
| TAOK3 | 4480 | 1.256831 | 0.208815 | 0.3624238 | FALSE |
| NMU | 6455 | 1.256776 | 0.208835 | 0.3624238 | FALSE |
| SSBP2 | 14720 | 1.256656 | 0.208878 | 0.362459 | FALSE |
| TNFAIP8 | 3932 | 1.255418 | 0.209327 | 0.3631975 | FALSE |
| PI3 | 3716 | 1.25489 | 0.209519 | 0.3634897 | FALSE |
| MYH14 | 13190 | 1.254539 | 0.209646 | 0.3636629 | FALSE |
| RIMKLB | 10584 | 1.254487 | 0.209665 | 0.3636629 | FALSE |
| ZNF677 | 12259 | 1.254096 | 0.209807 | 0.3637264 | FALSE |
| MYO10 | 1833 | 1.254077 | 0.209814 | 0.3637264 | FALSE |
| SCYL3 | 10186 | 1.254066 | 0.209818 | 0.3637264 | FALSE |
| GUCA2A | 1758 | 1.25329 | 0.2101 | 0.3639734 | FALSE |
| EPOP | 10771 | 1.252245 | 0.210481 | 0.3645113 | FALSE |
| MFSD14A | 12905 | 1.251826 | 0.210633 | 0.3647353 | FALSE |
| PAX9 | 5438 | 1.251678 | 0.210687 | 0.3647479 | FALSE |
| CA5A | 6687 | 1.251584 | 0.210721 | 0.3647668 | FALSE |
| ASB2 | 148 | 1.251448 | 0.210771 | 0.3648122 | FALSE |
| TDRD10 | 6941 | 1.250977 | 0.210943 | 0.3650692 | FALSE |
| CD86 | 8990 | 1.250767 | 0.211019 | 0.3651614 | FALSE |
| TEX26 | 13275 | 1.250695 | 0.211046 | 0.3651664 | FALSE |
| B3GNT6 | 3530 | 1.250191 | 0.21123 | 0.3654444 | FALSE |
| C11orf16 | 7456 | 1.250039 | 0.211285 | 0.3655 | FALSE |
| ATP5MD | 9012 | 1.249827 | 0.211363 | 0.3655935 | FALSE |
| PARVB | 6885 | 1.248851 | 0.21172 | 0.3660892 | FALSE |
| NSUN6 | 15123 | 1.247942 | 0.212052 | 0.3665429 | FALSE |
| CCT6A | 4008 | 1.246272 | 0.212665 | 0.3674387 | FALSE |
| SPPL2B | 4199 | 1.245868 | 0.212813 | 0.3676136 | FALSE |
| WDR31 | 6342 | 1.245595 | 0.212913 | 0.3676839 | FALSE |
| NOTCH2 | 5694 | 1.245538 | 0.212934 | 0.3676839 | FALSE |
| LOC107984035 | 9208 | 1.245529 | 0.212937 | 0.3676839 | FALSE |
| SARAF | 9695 | 1.245479 | 0.212956 | 0.3676839 | FALSE |
| KIF20A | 3094 | 1.245236 | 0.213045 | 0.3677708 | FALSE |
| TMEM222 | 2456 | 1.244766 | 0.213218 | 0.3679846 | FALSE |
| TAF9 | 4847 | 1.244689 | 0.213246 | 0.3679846 | FALSE |
| FXYD7 | 2773 | 1.243601 | 0.213646 | 0.3686056 | FALSE |
| ZNF233 | 3073 | 1.242837 | 0.213928 | 0.368969 | FALSE |
| UBE2B | 10335 | 1.241791 | 0.214314 | 0.3695528 | FALSE |
| GAS2L1P2 | 10003 | 1.241522 | 0.214413 | 0.3696833 | FALSE |
| MED10 | 9525 | 1.241215 | 0.214526 | 0.3698379 | FALSE |
| KIF17 | 4124 | 1.241117 | 0.214563 | 0.3698493 | FALSE |
| GPR142 | 3182 | 1.240192 | 0.214904 | 0.3702447 | FALSE |
| KCTD7 | 3905 | 1.239593 | 0.215126 | 0.3704829 | FALSE |
| YWHAE | 963 | 1.239564 | 0.215137 | 0.3704829 | FALSE |
| ELK1 | 4685 | 1.239562 | 0.215137 | 0.3704829 | FALSE |
| C18orf12 | 13245 | 1.23917 | 0.215283 | 0.3706319 | FALSE |
| ZSCAN10 | 4568 | 1.239072 | 0.215319 | 0.3706319 | FALSE |
| HSF1 | 3803 | 1.238705 | 0.215455 | 0.3707862 | FALSE |
| ZNF205-AS1 | 15558 | 1.238607 | 0.215491 | 0.3707913 | FALSE |
| STEAP1 | 11766 | 1.238248 | 0.215624 | 0.3708716 | FALSE |
| ORAI2 | 1433 | 1.238167 | 0.215654 | 0.3708824 | FALSE |
| HUWE1 | 13185 | 1.238082 | 0.215686 | 0.3708958 | FALSE |
| LAMP1 | 8523 | 1.237811 | 0.215786 | 0.3710278 | FALSE |
| S100A1 | 6165 | 1.237443 | 0.215923 | 0.3711809 | FALSE |
| ITGA8 | 8165 | 1.237344 | 0.215959 | 0.3711809 | FALSE |
| NIP7 | 3736 | 1.237319 | 0.215969 | 0.3711809 | FALSE |
| ATP6AP1 | 13867 | 1.236961 | 0.216102 | 0.3713251 | FALSE |
| TRMT10C | 6085 | 1.236598 | 0.216236 | 0.3715159 | FALSE |
| HLCS | 3582 | 1.236449 | 0.216292 | 0.3715701 | FALSE |
| MVB12B | 7630 | 1.23621 | 0.216381 | 0.3716819 | FALSE |
| LMNA | 678 | 1.235453 | 0.216662 | 0.3721244 | FALSE |
| CCNYL1 | 1060 | 1.234906 | 0.216865 | 0.3723922 | FALSE |
| TMEM126A | 14177 | 1.234535 | 0.217004 | 0.3725475 | FALSE |
| TSPYL2 | 12292 | 1.234227 | 0.217118 | 0.3726236 | FALSE |
| IGF2BP2 | 12132 | 1.234224 | 0.217119 | 0.3726236 | FALSE |
| XCR1 | 10315 | 1.233875 | 0.217249 | 0.3727598 | FALSE |
| MAPK8IP2 | 7361 | 1.233864 | 0.217254 | 0.3727598 | FALSE |
| PSIP1 | 9606 | 1.233625 | 0.217343 | 0.372843 | FALSE |
| ARHGAP42 | 9811 | 1.233028 | 0.217565 | 0.373143 | FALSE |
| CYP2A13 | 3819 | 1.232494 | 0.217765 | 0.3734029 | FALSE |
| ALG9 | 901 | 1.232282 | 0.217844 | 0.3734567 | FALSE |
| ANXA2R | 5945 | 1.232063 | 0.217926 | 0.373515 | FALSE |
| LGALS3 | 2143 | 1.231765 | 0.218037 | 0.3736648 | FALSE |
| RBCK1 | 13701 | 1.231379 | 0.218181 | 0.3738711 | FALSE |
| CLIC1 | 2167 | 1.231148 | 0.218268 | 0.3739781 | FALSE |
| C7orf50 | 10428 | 1.230837 | 0.218384 | 0.3740544 | FALSE |
| HIST1H3C | 622 | 1.230268 | 0.218597 | 0.3742961 | FALSE |
| SPON1 | 2068 | 1.229726 | 0.2188 | 0.3746026 | FALSE |
| DGKA | 12730 | 1.229457 | 0.218901 | 0.3747341 | FALSE |
| SERPINB9P1 | 9866 | 1.228842 | 0.219131 | 0.3750056 | FALSE |
| PRKX | 1332 | 1.228755 | 0.219164 | 0.3750203 | FALSE |
| DHDDS | 8763 | 1.228418 | 0.21929 | 0.3751393 | FALSE |
| MAGEA6 | 2766 | 1.228347 | 0.219317 | 0.3751393 | FALSE |
| ANXA4 | 2120 | 1.2282 | 0.219372 | 0.3751393 | FALSE |
| H3F3C | 9040 | 1.228186 | 0.219377 | 0.3751393 | FALSE |
| PCDHAC2 | 10870 | 1.227762 | 0.219536 | 0.3753499 | FALSE |
| PWP1 | 8119 | 1.22773 | 0.219548 | 0.3753499 | FALSE |
| ZNF382 | 2125 | 1.227503 | 0.219634 | 0.3754135 | FALSE |
| MTM1 | 10209 | 1.227157 | 0.219764 | 0.3755946 | FALSE |
| MAPKAPK5-AS1 | 11815 | 1.226282 | 0.220093 | 0.3760471 | FALSE |
| PPP2R5C | 15191 | 1.226261 | 0.2201 | 0.3760471 | FALSE |
| ZNF608 | 10433 | 1.226146 | 0.220144 | 0.3760799 | FALSE |
| RNF175 | 13264 | 1.225409 | 0.220421 | 0.3765127 | FALSE |
| NR2C2AP | 4689 | 1.225009 | 0.220572 | 0.3767289 | FALSE |
| CREG2 | 11588 | 1.224467 | 0.220776 | 0.3769542 | FALSE |
| ELMSAN1 | 7962 | 1.224301 | 0.220839 | 0.3770199 | FALSE |
| SLC41A3 | 4915 | 1.222931 | 0.221356 | 0.3778614 | FALSE |
| PDE3A | 9111 | 1.222829 | 0.221394 | 0.3778859 | FALSE |
| SUCLG2 | 5521 | 1.222608 | 0.221478 | 0.3779395 | FALSE |
| LCE1D | 12710 | 1.222554 | 0.221498 | 0.3779395 | FALSE |
| SRP72 | 8356 | 1.221924 | 0.221736 | 0.378222 | FALSE |
| MIR7515HG | 1439 | 1.220783 | 0.222168 | 0.3788346 | FALSE |
| MAS1L | 7824 | 1.220172 | 0.2224 | 0.3791879 | FALSE |
| OR2H1 | 8882 | 1.219732 | 0.222566 | 0.3793896 | FALSE |
| SLC22A18 | 10494 | 1.219003 | 0.222843 | 0.3797782 | FALSE |
| STMN3 | 863 | 1.217814 | 0.223295 | 0.3803405 | FALSE |
| CHST13 | 170 | 1.217739 | 0.223323 | 0.3803477 | FALSE |
| CYS1 | 4732 | 1.217257 | 0.223506 | 0.3805354 | FALSE |
| ADAR | 7654 | 1.216249 | 0.22389 | 0.3810641 | FALSE |
| GPT | 3852 | 1.215148 | 0.22431 | 0.3816535 | FALSE |
| CREB5 | 6904 | 1.214014 | 0.224742 | 0.3823066 | FALSE |
| ADCYAP1 | 5637 | 1.213766 | 0.224837 | 0.3823429 | FALSE |
| COLCA1 | 13471 | 1.213661 | 0.224877 | 0.3823695 | FALSE |
| KIFAP3 | 14616 | 1.213401 | 0.224977 | 0.3824552 | FALSE |
| RBM10 | 986 | 1.213198 | 0.225054 | 0.3825059 | FALSE |
| ATP8B5P | 9412 | 1.212528 | 0.22531 | 0.3828145 | FALSE |
| ACYP1 | 2075 | 1.211661 | 0.225642 | 0.3832117 | FALSE |
| TSR2 | 15082 | 1.211253 | 0.225798 | 0.3834355 | FALSE |
| ZNF746 | 7979 | 1.210572 | 0.226059 | 0.3838371 | FALSE |
| HMBOX1 | 5537 | 1.210199 | 0.226203 | 0.3839679 | FALSE |
| BCLAF3 | 16 | 1.210115 | 0.226235 | 0.3839679 | FALSE |
| SLC1A5 | 677 | 1.210024 | 0.22627 | 0.3839855 | FALSE |
| MIOX | 7312 | 1.209932 | 0.226305 | 0.3840037 | FALSE |
| MEFV | 1173 | 1.209689 | 0.226398 | 0.3841202 | FALSE |
| TPSG1 | 4113 | 1.209463 | 0.226485 | 0.3841333 | FALSE |
| LOC554206 | 8332 | 1.209457 | 0.226487 | 0.3841333 | FALSE |
| ARSF | 8797 | 1.209363 | 0.226523 | 0.3841333 | FALSE |
| ULBP1 | 11010 | 1.209336 | 0.226534 | 0.3841333 | FALSE |
| ZNF462 | 7025 | 1.209231 | 0.226574 | 0.3841333 | FALSE |
| PARD6A | 14899 | 1.209221 | 0.226578 | 0.3841333 | FALSE |
| RNASE13 | 9179 | 1.208998 | 0.226664 | 0.3842369 | FALSE |
| MPST | 2805 | 1.208541 | 0.226839 | 0.3843999 | FALSE |
| ARSJ | 8421 | 1.208457 | 0.226872 | 0.3843999 | FALSE |
| MGAT4C | 14697 | 1.208279 | 0.22694 | 0.3843999 | FALSE |
| KDM6A | 8588 | 1.207681 | 0.22717 | 0.3846781 | FALSE |
| FLNB | 9226 | 1.206738 | 0.227533 | 0.3851261 | FALSE |
| A3GALT2 | 1120 | 1.206257 | 0.227718 | 0.3853981 | FALSE |
| CCND3 | 13958 | 1.205683 | 0.22794 | 0.3857309 | FALSE |
| PRMT5 | 11129 | 1.205341 | 0.228072 | 0.3859124 | FALSE |
| TACR2 | 2494 | 1.204894 | 0.228244 | 0.3861208 | FALSE |
| JAG2 | 13857 | 1.204768 | 0.228293 | 0.3861613 | FALSE |
| RPN2 | 8968 | 1.203745 | 0.228688 | 0.3866625 | FALSE |
| MRE11 | 10034 | 1.20346 | 0.228798 | 0.386807 | FALSE |
| H2BFS | 968 | 1.202783 | 0.22906 | 0.3870825 | FALSE |
| JTB | 10873 | 1.202403 | 0.229207 | 0.3872473 | FALSE |
| BCKDHB | 4447 | 1.201994 | 0.229366 | 0.3874313 | FALSE |
| TTC32 | 10736 | 1.201635 | 0.229505 | 0.3875493 | FALSE |
| OR3A1 | 9719 | 1.201601 | 0.229518 | 0.3875493 | FALSE |
| PDIA4 | 41 | 1.201484 | 0.229564 | 0.3875493 | FALSE |
| FOS | 14095 | 1.19922 | 0.230442 | 0.3887949 | FALSE |
| TCF7 | 6045 | 1.199168 | 0.230463 | 0.3887949 | FALSE |
| FAM86DP | 5551 | 1.199145 | 0.230472 | 0.3887949 | FALSE |
| MTSS1L | 7106 | 1.198848 | 0.230587 | 0.3889477 | FALSE |
| IL21R | 15405 | 1.198744 | 0.230628 | 0.388974 | FALSE |
| CYP17A1 | 9601 | 1.198572 | 0.230694 | 0.3890448 | FALSE |
| OR11A1 | 3206 | 1.198252 | 0.230819 | 0.3892128 | FALSE |
| TIPIN | 8363 | 1.197824 | 0.230986 | 0.3894518 | FALSE |
| EVI2B | 793 | 1.197125 | 0.231258 | 0.3897428 | FALSE |
| TMEM184B | 5682 | 1.196654 | 0.231441 | 0.3900101 | FALSE |
| DDX11L2 | 7350 | 1.195709 | 0.23181 | 0.3904861 | FALSE |
| PECAM1 | 364 | 1.195674 | 0.231824 | 0.3904861 | FALSE |
| MYL9 | 12683 | 1.195416 | 0.231925 | 0.3906137 | FALSE |
| PDE11A | 2054 | 1.195176 | 0.232018 | 0.3907295 | FALSE |
| DUSP6 | 2727 | 1.195033 | 0.232074 | 0.3907815 | FALSE |
| TFDP2 | 12320 | 1.194684 | 0.232211 | 0.3908434 | FALSE |
| ZNF583 | 13748 | 1.194619 | 0.232236 | 0.3908434 | FALSE |
| PTAFR | 4040 | 1.194477 | 0.232291 | 0.3908947 | FALSE |
| HSD17B1 | 9308 | 1.194135 | 0.232425 | 0.3910777 | FALSE |
| TMA7 | 9322 | 1.193893 | 0.23252 | 0.3911948 | FALSE |
| SYNDIG1L | 9392 | 1.193357 | 0.23273 | 0.3915056 | FALSE |
| CSNK2B | 8767 | 1.193085 | 0.232836 | 0.391559 | FALSE |
| RNF187 | 2353 | 1.193084 | 0.232836 | 0.391559 | FALSE |
| RALGPS2 | 3964 | 1.192577 | 0.233035 | 0.3918213 | FALSE |
| RNASEH1 | 3359 | 1.192506 | 0.233063 | 0.3918213 | FALSE |
| ARSD | 5688 | 1.192366 | 0.233118 | 0.3918213 | FALSE |
| UBE2Z | 14027 | 1.191684 | 0.233385 | 0.3921864 | FALSE |
| TFRC | 7497 | 1.191251 | 0.233555 | 0.3923876 | FALSE |
| CBX6 | 9634 | 1.191044 | 0.233636 | 0.3924819 | FALSE |
| SOS2 | 11075 | 1.189689 | 0.234169 | 0.3932071 | FALSE |
| TEX264 | 2469 | 1.189528 | 0.234232 | 0.3932429 | FALSE |
| SPRYD3 | 7280 | 1.189075 | 0.23441 | 0.3934015 | FALSE |
| HS3ST3A1 | 12661 | 1.188998 | 0.23444 | 0.3934015 | FALSE |
| ETF1 | 4779 | 1.188947 | 0.234461 | 0.3934015 | FALSE |
| YBX1 | 14863 | 1.188632 | 0.234585 | 0.3934828 | FALSE |
| LINC02249 | 4708 | 1.187552 | 0.23501 | 0.3940696 | FALSE |
| RUFY3 | 14518 | 1.187465 | 0.235044 | 0.3940848 | FALSE |
| MND1 | 8358 | 1.186386 | 0.23547 | 0.3946741 | FALSE |
| USP32P2 | 14588 | 1.185834 | 0.235688 | 0.3949521 | FALSE |
| LHPP | 12568 | 1.185634 | 0.235767 | 0.3950144 | FALSE |
| DOCK1 | 8524 | 1.185612 | 0.235776 | 0.3950144 | FALSE |
| TRIM15 | 9990 | 1.185091 | 0.235981 | 0.3952323 | FALSE |
| R3HDM4 | 8612 | 1.184626 | 0.236165 | 0.3953708 | FALSE |
| CDK17 | 11114 | 1.184224 | 0.236324 | 0.3954677 | FALSE |
| CARD6 | 3811 | 1.183415 | 0.236645 | 0.395919 | FALSE |
| FAM155B | 12190 | 1.18307 | 0.236781 | 0.3960629 | FALSE |
| MIR205HG | 4171 | 1.182613 | 0.236963 | 0.3963235 | FALSE |
| ZC3H3 | 13223 | 1.182125 | 0.237156 | 0.3965922 | FALSE |
| BNIP3L | 11957 | 1.18208 | 0.237174 | 0.3965922 | FALSE |
| PLA2G16 | 10327 | 1.181735 | 0.237311 | 0.396771 | FALSE |
| TMEM41A | 3976 | 1.18146 | 0.23742 | 0.396771 | FALSE |
| VWA1 | 14311 | 1.18143 | 0.237432 | 0.396771 | FALSE |
| POLR2F | 15269 | 1.181326 | 0.237473 | 0.3967956 | FALSE |
| DUSP14 | 1176 | 1.181118 | 0.237556 | 0.3968912 | FALSE |
| LIMCH1 | 957 | 1.180655 | 0.23774 | 0.3971561 | FALSE |
| UNC45A | 4675 | 1.17963 | 0.238147 | 0.3977945 | FALSE |
| ACSS1 | 13778 | 1.179113 | 0.238353 | 0.3980531 | FALSE |
| FAS | 6473 | 1.178981 | 0.238406 | 0.3980984 | FALSE |
| COL22A1 | 127 | 1.178649 | 0.238538 | 0.3981504 | FALSE |
| PHB | 9492 | 1.178571 | 0.238569 | 0.3981584 | FALSE |
| ZNF215 | 9181 | 1.178129 | 0.238745 | 0.3983673 | FALSE |
| GNA13 | 13773 | 1.178062 | 0.238772 | 0.3983694 | FALSE |
| PAPSS1 | 404 | 1.177752 | 0.238895 | 0.398533 | FALSE |
| MIEN1 | 11019 | 1.177021 | 0.239187 | 0.3988919 | FALSE |
| THNSL2 | 3574 | 1.175979 | 0.239603 | 0.399458 | FALSE |
| TMEM150C | 3797 | 1.175833 | 0.239662 | 0.3995127 | FALSE |
| SEBOX | 13850 | 1.17569 | 0.239719 | 0.3995654 | FALSE |
| SGK1 | 5750 | 1.17558 | 0.239763 | 0.3995961 | FALSE |
| FGD1 | 9499 | 1.174856 | 0.240052 | 0.400036 | FALSE |
| POLR3C | 4452 | 1.174425 | 0.240225 | 0.4002382 | FALSE |
| NFE2L3 | 8723 | 1.172404 | 0.241035 | 0.4012457 | FALSE |
| PIGA | 2706 | 1.172253 | 0.241095 | 0.4013039 | FALSE |
| EFS | 6807 | 1.171998 | 0.241198 | 0.4013888 | FALSE |
| RHBDL1 | 8847 | 1.171677 | 0.241327 | 0.4015178 | FALSE |
| MMP28 | 7096 | 1.170891 | 0.241643 | 0.4019577 | FALSE |
| FLJ32255 | 869 | 1.170621 | 0.241751 | 0.4020034 | FALSE |
| H2AFY | 162 | 1.170577 | 0.241769 | 0.4020034 | FALSE |
| LRFN4 | 13731 | 1.170132 | 0.241948 | 0.4021232 | FALSE |
| ASCC1 | 6712 | 1.169743 | 0.242104 | 0.4022551 | FALSE |
| PSD | 8331 | 1.168667 | 0.242538 | 0.4029324 | FALSE |
| TMEM272 | 10480 | 1.168483 | 0.242612 | 0.4030128 | FALSE |
| GDNF | 2371 | 1.1684 | 0.242645 | 0.4030255 | FALSE |
| HIST1H2AK | 2249 | 1.168234 | 0.242712 | 0.4030511 | FALSE |
| OR10H2 | 12738 | 1.168054 | 0.242785 | 0.4031288 | FALSE |
| DCK | 6887 | 1.167183 | 0.243136 | 0.4035838 | FALSE |
| RPL23A | 12452 | 1.167064 | 0.243185 | 0.4035994 | FALSE |
| OR2T6 | 8947 | 1.16566 | 0.243752 | 0.4044337 | FALSE |
| NXT2 | 7997 | 1.165324 | 0.243888 | 0.4045733 | FALSE |
| BMF | 7230 | 1.164601 | 0.244181 | 0.4049903 | FALSE |
| TRMT2A | 14416 | 1.164575 | 0.244191 | 0.4049903 | FALSE |
| LINC00665 | 7735 | 1.163649 | 0.244566 | 0.4054836 | FALSE |
| NKX1-2 | 14753 | 1.163496 | 0.244628 | 0.4055434 | FALSE |
| OR7E14P | 75 | 1.16313 | 0.244777 | 0.4057465 | FALSE |
| RASSF3 | 2586 | 1.161992 | 0.245239 | 0.406426 | FALSE |
| ACBD4 | 10707 | 1.16175 | 0.245337 | 0.4065028 | FALSE |
| PP7080 | 7574 | 1.161491 | 0.245442 | 0.4066341 | FALSE |
| UBR1 | 14975 | 1.161427 | 0.245468 | 0.4066341 | FALSE |
| KRCC1 | 14891 | 1.161326 | 0.245509 | 0.406659 | FALSE |
| CCDC173 | 14687 | 1.161225 | 0.24555 | 0.4066839 | FALSE |
| SLC16A11 | 937 | 1.160908 | 0.245679 | 0.4067971 | FALSE |
| RIPPLY1 | 3045 | 1.160808 | 0.24572 | 0.4067971 | FALSE |
| MAZ | 2221 | 1.160801 | 0.245723 | 0.4067971 | FALSE |
| ZNF729 | 15583 | 1.160592 | 0.245808 | 0.4068948 | FALSE |
| OR52B6 | 12116 | 1.160399 | 0.245886 | 0.4069817 | FALSE |
| ANKRD35 | 7002 | 1.160042 | 0.246032 | 0.4071368 | FALSE |
| SRGN | 7362 | 1.159799 | 0.246131 | 0.4071368 | FALSE |
| PRSS2 | 15453 | 1.159753 | 0.246149 | 0.4071368 | FALSE |
| UPK1A-AS1 | 5964 | 1.159665 | 0.246185 | 0.4071368 | FALSE |
| GLRX5 | 14650 | 1.159657 | 0.246188 | 0.4071368 | FALSE |
| EIF3J-AS1 | 15421 | 1.159023 | 0.246447 | 0.4075209 | FALSE |
| OR4D2 | 12346 | 1.158429 | 0.246689 | 0.4078352 | FALSE |
| AGFG2 | 351 | 1.157682 | 0.246994 | 0.4082528 | FALSE |
| PTPRN | 10713 | 1.157381 | 0.247117 | 0.4084127 | FALSE |
| F3 | 2964 | 1.156843 | 0.247337 | 0.4086606 | FALSE |
| NOL6 | 3781 | 1.156755 | 0.247372 | 0.4086606 | FALSE |
| CDCA3 | 7818 | 1.156736 | 0.24738 | 0.4086606 | FALSE |
| PPP1R14C | 12689 | 1.156694 | 0.247397 | 0.4086606 | FALSE |
| CNTROB | 12563 | 1.155435 | 0.247912 | 0.4093381 | FALSE |
| CD40 | 15492 | 1.155127 | 0.248038 | 0.4094598 | FALSE |
| MINK1 | 14189 | 1.153787 | 0.248588 | 0.4102363 | FALSE |
| CCDC141 | 4784 | 1.15352 | 0.248697 | 0.4103737 | FALSE |
| OPHN1 | 15194 | 1.152845 | 0.248974 | 0.4107441 | FALSE |
| RPL22L1 | 13734 | 1.152491 | 0.249119 | 0.4109371 | FALSE |
| EID2 | 8308 | 1.152432 | 0.249144 | 0.4109371 | FALSE |
| TLK1 | 8395 | 1.152148 | 0.24926 | 0.4110862 | FALSE |
| KRT19 | 11873 | 1.152019 | 0.249313 | 0.4110997 | FALSE |
| ZNF812P | 875 | 1.152 | 0.249321 | 0.4110997 | FALSE |
| ZNF480 | 12875 | 1.151434 | 0.249554 | 0.4114399 | FALSE |
| CASZ1 | 699 | 1.149929 | 0.250173 | 0.4121568 | FALSE |
| CCT4 | 1630 | 1.149365 | 0.250406 | 0.4124901 | FALSE |
| DYRK2 | 11468 | 1.148353 | 0.250823 | 0.4130532 | FALSE |
| ZCCHC7 | 11089 | 1.147619 | 0.251126 | 0.4134651 | FALSE |
| TCP1 | 2356 | 1.147038 | 0.251366 | 0.4137296 | FALSE |
| MRPL3 | 14937 | 1.14687 | 0.251435 | 0.4138003 | FALSE |
| SH2B2 | 9431 | 1.146443 | 0.251612 | 0.4140472 | FALSE |
| SOX9 | 8319 | 1.146156 | 0.251731 | 0.4140941 | FALSE |
| IGF2-AS | 3489 | 1.146118 | 0.251746 | 0.4140941 | FALSE |
| THY1 | 943 | 1.145761 | 0.251894 | 0.4142935 | FALSE |
| COLQ | 13362 | 1.145096 | 0.252169 | 0.4146591 | FALSE |
| RAB1B | 13235 | 1.144783 | 0.252299 | 0.4148287 | FALSE |
| TULP3 | 13506 | 1.144699 | 0.252334 | 0.4148423 | FALSE |
| NEUROG1 | 5585 | 1.144566 | 0.252389 | 0.4148457 | FALSE |
| METRN | 2956 | 1.144365 | 0.252472 | 0.414939 | FALSE |
| TMEM100 | 3588 | 1.143959 | 0.252641 | 0.415172 | FALSE |
| HIST3H3 | 9657 | 1.142845 | 0.253103 | 0.4158006 | FALSE |
| PARD6G-AS1 | 13382 | 1.142437 | 0.253272 | 0.4160353 | FALSE |
| USF3 | 394 | 1.142067 | 0.253426 | 0.4162441 | FALSE |
| EPS8 | 4593 | 1.141885 | 0.253502 | 0.4163246 | FALSE |
| TFF1 | 15041 | 1.141319 | 0.253737 | 0.4165799 | FALSE |
| SLC1A1 | 10925 | 1.140585 | 0.254043 | 0.4169938 | FALSE |
| EXOC3L2 | 8 | 1.140048 | 0.254266 | 0.4173171 | FALSE |
| TCEA2 | 11976 | 1.139834 | 0.254355 | 0.4174196 | FALSE |
| DOLPP1 | 15409 | 1.139693 | 0.254414 | 0.4174564 | FALSE |
| HLA-L | 11205 | 1.139652 | 0.254431 | 0.4174564 | FALSE |
| EXOSC4 | 14040 | 1.139544 | 0.254476 | 0.4174761 | FALSE |
| OR6M1 | 6976 | 1.139431 | 0.254523 | 0.4174761 | FALSE |
| HLA-E | 10046 | 1.138695 | 0.25483 | 0.4179357 | FALSE |
| SMC3 | 14545 | 1.137982 | 0.255128 | 0.4183799 | FALSE |
| GDF7 | 2088 | 1.137662 | 0.255262 | 0.4185552 | FALSE |
| RGS13 | 9978 | 1.13741 | 0.255367 | 0.4186832 | FALSE |
| ORAI1 | 3033 | 1.137347 | 0.255393 | 0.4186832 | FALSE |
| TGIF1 | 12361 | 1.137195 | 0.255457 | 0.4187434 | FALSE |
| CDK20 | 4721 | 1.137011 | 0.255534 | 0.4188256 | FALSE |
| AADACL3 | 4628 | 1.136253 | 0.255851 | 0.4191693 | FALSE |
| GET4 | 12571 | 1.13606 | 0.255931 | 0.4192577 | FALSE |
| DALRD3 | 3029 | 1.135713 | 0.256077 | 0.4194482 | FALSE |
| LTBP2 | 9969 | 1.135654 | 0.256101 | 0.4194482 | FALSE |
| MPZL1 | 5187 | 1.13491 | 0.256413 | 0.4199001 | FALSE |
| CYSLTR1 | 6356 | 1.134803 | 0.256458 | 0.4199001 | FALSE |
| CACNA1E | 1931 | 1.134529 | 0.256573 | 0.4200441 | FALSE |
| TBC1D28 | 9425 | 1.134291 | 0.256673 | 0.4201635 | FALSE |
| S100A14 | 14671 | 1.134025 | 0.256784 | 0.4202897 | FALSE |
| GOLGA2P11 | 12644 | 1.133979 | 0.256803 | 0.4202897 | FALSE |
| CYP26C1 | 2596 | 1.133692 | 0.256924 | 0.4204427 | FALSE |
| TOB2 | 6037 | 1.133529 | 0.256992 | 0.4205037 | FALSE |
| CACNB3 | 6694 | 1.133475 | 0.257015 | 0.4205037 | FALSE |
| RBMX | 7669 | 1.133299 | 0.257089 | 0.4205806 | FALSE |
| SNX31 | 113 | 1.132761 | 0.257315 | 0.4208875 | FALSE |
| TTLL8 | 12059 | 1.132724 | 0.25733 | 0.4208875 | FALSE |
| PRELID1 | 8854 | 1.131444 | 0.257868 | 0.4215911 | FALSE |
| TNXB | 7318 | 1.129793 | 0.258563 | 0.4224985 | FALSE |
| KLK2 | 8956 | 1.129741 | 0.258585 | 0.4224985 | FALSE |
| ZNF331 | 10250 | 1.128556 | 0.259085 | 0.4231382 | FALSE |
| CBR3 | 14760 | 1.128484 | 0.259116 | 0.4231436 | FALSE |
| NPHP1 | 1351 | 1.128419 | 0.259143 | 0.4231442 | FALSE |
| BTRC | 4679 | 1.128335 | 0.259178 | 0.4231579 | FALSE |
| PTEN | 5702 | 1.128218 | 0.259228 | 0.4231944 | FALSE |
| LINC01138 | 12860 | 1.127853 | 0.259382 | 0.4233186 | FALSE |
| MMS19 | 11750 | 1.127722 | 0.259437 | 0.4233186 | FALSE |
| WDR41 | 5507 | 1.127707 | 0.259444 | 0.4233186 | FALSE |
| SEC24B-AS1 | 7087 | 1.1276 | 0.259489 | 0.4233186 | FALSE |
| EXOC1 | 9880 | 1.127525 | 0.259521 | 0.4233186 | FALSE |
| LOC100129434 | 5733 | 1.127327 | 0.259604 | 0.4233536 | FALSE |
| NAGLU | 5876 | 1.127133 | 0.259686 | 0.4234121 | FALSE |
| C5orf58 | 15577 | 1.127057 | 0.259718 | 0.4234203 | FALSE |
| PABPC3 | 3457 | 1.126829 | 0.259815 | 0.4234892 | FALSE |
| KCNJ5 | 7601 | 1.12676 | 0.259844 | 0.4234926 | FALSE |
| CCDC106 | 771 | 1.126419 | 0.259988 | 0.4236393 | FALSE |
| CBLN2 | 3066 | 1.125222 | 0.260495 | 0.4243766 | FALSE |
| TLE6 | 6058 | 1.124757 | 0.260692 | 0.4246077 | FALSE |
| SLC25A39 | 14439 | 1.124595 | 0.260761 | 0.4246325 | FALSE |
| ACSM1 | 1293 | 1.12446 | 0.260818 | 0.4246745 | FALSE |
| MRPL44 | 14191 | 1.124346 | 0.260866 | 0.4246745 | FALSE |
| CLYBL | 8485 | 1.123972 | 0.261025 | 0.4248857 | FALSE |
| PCNP | 13036 | 1.123657 | 0.261159 | 0.425059 | FALSE |
| C11orf94 | 14432 | 1.123505 | 0.261223 | 0.4251198 | FALSE |
| EAPP | 9009 | 1.122921 | 0.261471 | 0.4254457 | FALSE |
| DNAJC3 | 9516 | 1.122841 | 0.261505 | 0.4254457 | FALSE |
| FAM160A1 | 2555 | 1.122344 | 0.261716 | 0.4256564 | FALSE |
| TNFRSF1A | 4173 | 1.122256 | 0.261754 | 0.425673 | FALSE |
| SPN | 15277 | 1.121944 | 0.261886 | 0.4258444 | FALSE |
| CCL17 | 8009 | 1.121851 | 0.261926 | 0.4258644 | FALSE |
| TATDN2 | 3003 | 1.121475 | 0.262086 | 0.4260801 | FALSE |
| CACNA2D1 | 9320 | 1.120939 | 0.262314 | 0.4263878 | FALSE |
| NCBP2-AS2 | 8393 | 1.119996 | 0.262715 | 0.4269707 | FALSE |
| TFF3 | 11819 | 1.119407 | 0.262967 | 0.4272011 | FALSE |
| AGTRAP | 13178 | 1.118485 | 0.26336 | 0.4275735 | FALSE |
| TLN2 | 7679 | 1.11839 | 0.2634 | 0.427595 | FALSE |
| TIGD4 | 14793 | 1.117931 | 0.263596 | 0.4278687 | FALSE |
| COA7 | 5628 | 1.117515 | 0.263774 | 0.4279502 | FALSE |
| LINC00304 | 12953 | 1.117397 | 0.263825 | 0.4279502 | FALSE |
| FFAR1 | 5681 | 1.117366 | 0.263838 | 0.4279502 | FALSE |
| TMEM175 | 14485 | 1.117365 | 0.263838 | 0.4279502 | FALSE |
| FXYD1 | 12060 | 1.117152 | 0.263929 | 0.4280535 | FALSE |
| CALCRL | 5262 | 1.116216 | 0.26433 | 0.4285694 | FALSE |
| YPEL5 | 15011 | 1.115824 | 0.264497 | 0.4287538 | FALSE |
| NAMPT | 9457 | 1.115758 | 0.264526 | 0.4287538 | FALSE |
| WDR3 | 4901 | 1.115586 | 0.264599 | 0.428828 | FALSE |
| WFDC10B | 9790 | 1.115523 | 0.264626 | 0.428828 | FALSE |
| ARMC2 | 5516 | 1.115299 | 0.264722 | 0.4289064 | FALSE |
| AQP6 | 9235 | 1.115282 | 0.26473 | 0.4289064 | FALSE |
| ZFP90 | 11088 | 1.114346 | 0.265131 | 0.4293784 | FALSE |
| MEST | 2850 | 1.113734 | 0.265393 | 0.4297145 | FALSE |
| SLC1A7 | 10548 | 1.11357 | 0.265464 | 0.429784 | FALSE |
| LOC101928659 | 2765 | 1.112777 | 0.265804 | 0.4302907 | FALSE |
| MRPS18B | 15293 | 1.112649 | 0.265859 | 0.4303352 | FALSE |
| SHOX | 7941 | 1.112384 | 0.265973 | 0.4304393 | FALSE |
| PGBD5 | 3902 | 1.11231 | 0.266005 | 0.4304393 | FALSE |
| GALC | 9221 | 1.112307 | 0.266006 | 0.4304393 | FALSE |
| STARD3 | 12659 | 1.112092 | 0.266099 | 0.4305443 | FALSE |
| PCAT6 | 10708 | 1.111858 | 0.266199 | 0.4306626 | FALSE |
| PCDHGA7 | 6817 | 1.111484 | 0.26636 | 0.4307939 | FALSE |
| KDM1B | 9940 | 1.111477 | 0.266363 | 0.4307939 | FALSE |
| SLC22A4 | 1152 | 1.110479 | 0.266793 | 0.4313214 | FALSE |
| STRA6 | 10656 | 1.110463 | 0.2668 | 0.4313214 | FALSE |
| MYLIP | 14081 | 1.109169 | 0.267357 | 0.4320443 | FALSE |
| ZCWPW2 | 6084 | 1.109032 | 0.267416 | 0.4320951 | FALSE |
| KLHDC4 | 11825 | 1.108648 | 0.267582 | 0.4322735 | FALSE |
| PTCH2 | 14898 | 1.107951 | 0.267883 | 0.4324991 | FALSE |
| ENHO | 6653 | 1.107618 | 0.268027 | 0.4326343 | FALSE |
| SYF2 | 12735 | 1.107469 | 0.268091 | 0.4326544 | FALSE |
| HIST1H3J | 11530 | 1.107432 | 0.268107 | 0.4326544 | FALSE |
| PARP9 | 13130 | 1.107397 | 0.268122 | 0.4326544 | FALSE |
| PTOV1 | 6883 | 1.107311 | 0.268159 | 0.4326586 | FALSE |
| SNRPD1 | 2226 | 1.107131 | 0.268237 | 0.432692 | FALSE |
| FAM9B | 1913 | 1.107087 | 0.268256 | 0.432692 | FALSE |
| TIPARP | 12940 | 1.105974 | 0.268738 | 0.4333345 | FALSE |
| MYOF | 7433 | 1.105365 | 0.269001 | 0.4335723 | FALSE |
| ANAPC5 | 1066 | 1.105339 | 0.269013 | 0.4335723 | FALSE |
| SWSAP1 | 12488 | 1.105106 | 0.269114 | 0.4335723 | FALSE |
| INTS7 | 5983 | 1.105093 | 0.269119 | 0.4335723 | FALSE |
| LOC101927596 | 13547 | 1.103691 | 0.269727 | 0.4344167 | FALSE |
| HMX3 | 1509 | 1.103352 | 0.269874 | 0.4345396 | FALSE |
| TCTN1 | 6990 | 1.1028 | 0.270114 | 0.4347912 | FALSE |
| RABL3 | 2690 | 1.102541 | 0.270227 | 0.4348828 | FALSE |
| IL18BP | 5713 | 1.101561 | 0.270653 | 0.4355236 | FALSE |
| MFNG | 7756 | 1.101137 | 0.270837 | 0.4357756 | FALSE |
| CLEC12A | 4467 | 1.100368 | 0.271172 | 0.4361347 | FALSE |
| KBTBD13 | 6703 | 1.100076 | 0.271299 | 0.436192 | FALSE |
| S100A11 | 1020 | 1.099821 | 0.27141 | 0.4362122 | FALSE |
| SPIN1 | 11813 | 1.099809 | 0.271415 | 0.4362122 | FALSE |
| EFCC1 | 13018 | 1.099308 | 0.271634 | 0.4364153 | FALSE |
| LOC389831 | 6939 | 1.099213 | 0.271675 | 0.4364153 | FALSE |
| KIAA1328 | 3217 | 1.099196 | 0.271683 | 0.4364153 | FALSE |
| LDAH | 3927 | 1.099135 | 0.271709 | 0.4364153 | FALSE |
| SETD9 | 10962 | 1.098841 | 0.271837 | 0.4365764 | FALSE |
| SPRR2E | 8556 | 1.098356 | 0.272049 | 0.4368419 | FALSE |
| PTPN11 | 1651 | 1.098161 | 0.272134 | 0.4369183 | FALSE |
| LRRC15 | 7004 | 1.097972 | 0.272217 | 0.4370059 | FALSE |
| RAD51D | 405 | 1.097731 | 0.272322 | 0.437109 | FALSE |
| LINC02470 | 9514 | 1.097696 | 0.272337 | 0.437109 | FALSE |
| CACNA1I | 7358 | 1.097505 | 0.272421 | 0.437109 | FALSE |
| PIPOX | 13613 | 1.097043 | 0.272623 | 0.4373432 | FALSE |
| OLFM2 | 5592 | 1.096848 | 0.272708 | 0.4374168 | FALSE |
| KDELR3 | 6862 | 1.09681 | 0.272724 | 0.4374168 | FALSE |
| HDAC10 | 8770 | 1.096542 | 0.272842 | 0.4375599 | FALSE |
| ARMCX5 | 6492 | 1.096393 | 0.272907 | 0.4376195 | FALSE |
| ACBD7 | 7330 | 1.095772 | 0.273179 | 0.4379738 | FALSE |
| STX7 | 3120 | 1.095159 | 0.273447 | 0.4382483 | FALSE |
| TMEM86A | 12076 | 1.095113 | 0.273467 | 0.4382483 | FALSE |
| CAPZB | 13243 | 1.094847 | 0.273584 | 0.4383732 | FALSE |
| CYBRD1 | 3480 | 1.094807 | 0.273601 | 0.4383732 | FALSE |
| VARS | 3623 | 1.094176 | 0.273878 | 0.4386605 | FALSE |
| NKX2-5 | 9882 | 1.094125 | 0.2739 | 0.4386605 | FALSE |
| CRYBB3 | 6489 | 1.09395 | 0.273977 | 0.4386605 | FALSE |
| PPM1K | 5431 | 1.093479 | 0.274184 | 0.438933 | FALSE |
| KHDRBS1 | 316 | 1.093434 | 0.274203 | 0.438933 | FALSE |
| BTNL9 | 11745 | 1.093226 | 0.274295 | 0.4390342 | FALSE |
| AFF3 | 3721 | 1.093077 | 0.27436 | 0.4390399 | FALSE |
| ZNF420 | 1730 | 1.093026 | 0.274382 | 0.4390399 | FALSE |
| UPK3A | 10200 | 1.092666 | 0.27454 | 0.4392479 | FALSE |
| RPL26L1 | 3287 | 1.092505 | 0.274611 | 0.4392775 | FALSE |
| SOX3 | 9801 | 1.092496 | 0.274615 | 0.4392775 | FALSE |
| KCNK6 | 9458 | 1.09132 | 0.275132 | 0.4397992 | FALSE |
| ARX | 8361 | 1.091306 | 0.275138 | 0.4397992 | FALSE |
| NLRC3 | 13477 | 1.089994 | 0.275716 | 0.4404522 | FALSE |
| AMPH | 5067 | 1.089218 | 0.276058 | 0.4408633 | FALSE |
| GPR152 | 680 | 1.088855 | 0.276218 | 0.4410739 | FALSE |
| MADD | 5409 | 1.088097 | 0.276552 | 0.4413777 | FALSE |
| SWAP70 | 11083 | 1.088 | 0.276595 | 0.4413777 | FALSE |
| PATZ1 | 9124 | 1.087976 | 0.276606 | 0.4413777 | FALSE |
| MKL1 | 7557 | 1.087777 | 0.276694 | 0.4414728 | FALSE |
| CXCL14 | 13772 | 1.0875 | 0.276816 | 0.4415779 | FALSE |
| MESP2 | 7076 | 1.087403 | 0.276859 | 0.4416011 | FALSE |
| IRF2BPL | 13326 | 1.087172 | 0.276961 | 0.4417189 | FALSE |
| OR5V1 | 573 | 1.08709 | 0.276997 | 0.4417316 | FALSE |
| DTD1 | 13691 | 1.086812 | 0.27712 | 0.4418374 | FALSE |
| MAFIP | 10814 | 1.085286 | 0.277795 | 0.4427572 | FALSE |
| HIST1H2BJ | 2692 | 1.084846 | 0.27799 | 0.4429082 | FALSE |
| ZNF202 | 8857 | 1.084469 | 0.278157 | 0.4430841 | FALSE |
| PLD4 | 7193 | 1.08404 | 0.278347 | 0.4432938 | FALSE |
| RRP36 | 1851 | 1.08398 | 0.278374 | 0.4432938 | FALSE |
| PYCR2 | 7115 | 1.083827 | 0.278442 | 0.4433567 | FALSE |
| RHBDD3 | 160 | 1.08351 | 0.278582 | 0.4435354 | FALSE |
| LOC100240735 | 14886 | 1.083251 | 0.278697 | 0.4436732 | FALSE |
| DUSP9 | 11642 | 1.082939 | 0.278836 | 0.4438484 | FALSE |
| GKAP1 | 5171 | 1.08259 | 0.27899 | 0.4440499 | FALSE |
| BSND | 14442 | 1.082478 | 0.27904 | 0.444052 | FALSE |
| EPHX3 | 4943 | 1.082459 | 0.279049 | 0.444052 | FALSE |
| ANP32A-IT1 | 8212 | 1.081939 | 0.27928 | 0.4443229 | FALSE |
| ESYT3 | 8873 | 1.08182 | 0.279333 | 0.4443229 | FALSE |
| CHD7 | 1436 | 1.081463 | 0.279491 | 0.4445301 | FALSE |
| PYGB | 5631 | 1.081137 | 0.279636 | 0.4447154 | FALSE |
| TTTY15 | 12274 | 1.080572 | 0.279888 | 0.4449794 | FALSE |
| SP7 | 9213 | 1.080423 | 0.279954 | 0.4450395 | FALSE |
| FLJ37453 | 7033 | 1.080167 | 0.280068 | 0.4451302 | FALSE |
| ENOPH1 | 9391 | 1.080103 | 0.280096 | 0.4451302 | FALSE |
| SLC9A7 | 8048 | 1.079454 | 0.280385 | 0.4454085 | FALSE |
| MRPS15 | 11747 | 1.07928 | 0.280463 | 0.4454864 | FALSE |
| DOCK6 | 9942 | 1.078853 | 0.280653 | 0.4455645 | FALSE |
| CLCA4 | 13802 | 1.078786 | 0.280683 | 0.4455645 | FALSE |
| METTL23 | 14682 | 1.078197 | 0.280946 | 0.445891 | FALSE |
| NLRP11 | 9212 | 1.077964 | 0.28105 | 0.4459655 | FALSE |
| P2RY1 | 5689 | 1.076922 | 0.281515 | 0.4465678 | FALSE |
| CEP70 | 7101 | 1.076703 | 0.281613 | 0.4466777 | FALSE |
| CALCOCO2 | 14691 | 1.076283 | 0.281801 | 0.4469302 | FALSE |
| GPALPP1 | 10325 | 1.076167 | 0.281853 | 0.4469324 | FALSE |
| LTBR | 3 | 1.076152 | 0.281859 | 0.4469324 | FALSE |
| CPQ | 11406 | 1.07594 | 0.281954 | 0.4470374 | FALSE |
| LOC101927027 | 13824 | 1.075403 | 0.282194 | 0.4473368 | FALSE |
| SIRPG | 4879 | 1.075337 | 0.282224 | 0.4473368 | FALSE |
| C19orf53 | 14946 | 1.075326 | 0.282229 | 0.4473368 | FALSE |
| PCDH1 | 1988 | 1.074762 | 0.282481 | 0.4476222 | FALSE |
| NTN3 | 1817 | 1.074756 | 0.282484 | 0.4476222 | FALSE |
| TUT1 | 3348 | 1.074732 | 0.282495 | 0.4476222 | FALSE |
| WASF1 | 10287 | 1.074625 | 0.282543 | 0.4476386 | FALSE |
| SP110 | 4125 | 1.074395 | 0.282646 | 0.4476593 | FALSE |
| MED26 | 6413 | 1.07436 | 0.282661 | 0.4476593 | FALSE |
| PDZD7 | 2886 | 1.074178 | 0.282743 | 0.4477431 | FALSE |
| MPZ | 9399 | 1.073523 | 0.283037 | 0.4481173 | FALSE |
| NOTUM | 14126 | 1.07297 | 0.283285 | 0.4484646 | FALSE |
| TRABD2B | 14698 | 1.072895 | 0.283318 | 0.4484724 | FALSE |
| ZSCAN2 | 11746 | 1.072679 | 0.283415 | 0.4485805 | FALSE |
| FUT3 | 10448 | 1.072243 | 0.283611 | 0.4488448 | FALSE |
| LY6E | 8488 | 1.071789 | 0.283815 | 0.4491221 | FALSE |
| TAT | 9625 | 1.071687 | 0.283861 | 0.4491491 | FALSE |
| DYNC1LI2 | 3565 | 1.070808 | 0.284256 | 0.4495014 | FALSE |
| SPNS1 | 657 | 1.070533 | 0.284379 | 0.4496515 | FALSE |
| APOL6 | 3090 | 1.070338 | 0.284467 | 0.4496659 | FALSE |
| TPP1 | 4747 | 1.070257 | 0.284504 | 0.4496659 | FALSE |
| POU3F3 | 2158 | 1.070125 | 0.284563 | 0.4497143 | FALSE |
| C11orf49 | 5650 | 1.06992 | 0.284655 | 0.4497692 | FALSE |
| SUPT7L | 976 | 1.069432 | 0.284875 | 0.4499345 | FALSE |
| LIPT2 | 7065 | 1.068945 | 0.285094 | 0.4501638 | FALSE |
| BABAM1 | 15085 | 1.068763 | 0.285176 | 0.4502286 | FALSE |
| THOC6 | 1035 | 1.068124 | 0.285465 | 0.4505662 | FALSE |
| LY6D | 3901 | 1.068097 | 0.285477 | 0.4505662 | FALSE |
| FURIN | 11917 | 1.067039 | 0.285954 | 0.4511831 | FALSE |
| BMP10 | 5289 | 1.066761 | 0.28608 | 0.4512446 | FALSE |
| LOC729080 | 6899 | 1.066139 | 0.286361 | 0.4516423 | FALSE |
| CNOT7 | 14361 | 1.065815 | 0.286507 | 0.4518277 | FALSE |
| FAM163B | 13324 | 1.065718 | 0.286551 | 0.4518513 | FALSE |
| TMEM248 | 11013 | 1.06542 | 0.286686 | 0.4520182 | FALSE |
| CYGB | 7765 | 1.064953 | 0.286897 | 0.4522602 | FALSE |
| CASQ1 | 10312 | 1.064459 | 0.287121 | 0.4524758 | FALSE |
| TRIM8 | 9448 | 1.064279 | 0.287202 | 0.4525586 | FALSE |
| A2ML1 | 11359 | 1.063892 | 0.287378 | 0.4527436 | FALSE |
| CROCC | 3849 | 1.063636 | 0.287494 | 0.4528807 | FALSE |
| VPS18 | 9135 | 1.062329 | 0.288086 | 0.4536772 | FALSE |
| CNP | 14452 | 1.061805 | 0.288324 | 0.4540061 | FALSE |
| VAMP7 | 2322 | 1.060902 | 0.288734 | 0.4546063 | FALSE |
| BORCS5 | 9567 | 1.059554 | 0.289348 | 0.455434 | FALSE |
| SYNE3 | 4850 | 1.059399 | 0.289418 | 0.4554992 | FALSE |
| DCLRE1B | 12820 | 1.058981 | 0.289608 | 0.4556611 | FALSE |
| KRTAP4-1 | 14234 | 1.058753 | 0.289712 | 0.4557786 | FALSE |
| CACNA1B | 11761 | 1.05854 | 0.289809 | 0.4558804 | FALSE |
| TRO | 5108 | 1.058483 | 0.289835 | 0.4558804 | FALSE |
| PRKD1 | 14868 | 1.058331 | 0.289905 | 0.4559435 | FALSE |
| COL9A1 | 10157 | 1.056179 | 0.290886 | 0.4571665 | FALSE |
| OR7E2P | 10718 | 1.055912 | 0.291008 | 0.4572655 | FALSE |
| GAREM1 | 5933 | 1.055563 | 0.291168 | 0.4574055 | FALSE |
| VSIG8 | 4182 | 1.055405 | 0.29124 | 0.4574356 | FALSE |
| ZNF473 | 14232 | 1.055206 | 0.291331 | 0.4574816 | FALSE |
| TMEM44 | 2245 | 1.055163 | 0.291351 | 0.4574816 | FALSE |
| ADAMTS7 | 4358 | 1.054823 | 0.291506 | 0.4576798 | FALSE |
| CELSR3 | 474 | 1.054742 | 0.291543 | 0.457692 | FALSE |
| YBX3 | 7742 | 1.054366 | 0.291715 | 0.4578701 | FALSE |
| CDH1 | 6176 | 1.054108 | 0.291833 | 0.4579987 | FALSE |
| MTTP | 14685 | 1.054059 | 0.291856 | 0.4579987 | FALSE |
| FAAP20 | 14134 | 1.05384 | 0.291956 | 0.4581101 | FALSE |
| CYHR1 | 4470 | 1.053404 | 0.292156 | 0.4583214 | FALSE |
| TESK2 | 3005 | 1.053354 | 0.292179 | 0.4583214 | FALSE |
| CDK4 | 4356 | 1.053113 | 0.292289 | 0.458445 | FALSE |
| NKAIN1 | 10424 | 1.053054 | 0.292316 | 0.458445 | FALSE |
| PFDN5 | 6769 | 1.052973 | 0.292353 | 0.4584572 | FALSE |
| CHTF18 | 12536 | 1.05266 | 0.292497 | 0.4586362 | FALSE |
| DARS2 | 10470 | 1.052261 | 0.29268 | 0.4587082 | FALSE |
| KLHDC10 | 3520 | 1.051491 | 0.293033 | 0.4590714 | FALSE |
| SFPQ | 11868 | 1.051479 | 0.293039 | 0.4590714 | FALSE |
| TRAPPC4 | 9732 | 1.051306 | 0.293118 | 0.4591146 | FALSE |
| NDRG2 | 10469 | 1.051291 | 0.293125 | 0.4591146 | FALSE |
| FAM174A | 13661 | 1.051151 | 0.293189 | 0.4591693 | FALSE |
| PRKCZ | 480 | 1.050053 | 0.293694 | 0.4597701 | FALSE |
| INO80C | 10213 | 1.049996 | 0.29372 | 0.4597701 | FALSE |
| RTCB | 967 | 1.049513 | 0.293942 | 0.4600717 | FALSE |
| ELOF1 | 7862 | 1.049213 | 0.29408 | 0.4601956 | FALSE |
| ASIC1 | 3739 | 1.048946 | 0.294203 | 0.4603418 | FALSE |
| UQCC1 | 6619 | 1.048766 | 0.294286 | 0.4604254 | FALSE |
| HIST1H2BG | 10043 | 1.048496 | 0.29441 | 0.4605306 | FALSE |
| PELO | 11664 | 1.047579 | 0.294833 | 0.4610963 | FALSE |
| RPL17 | 2 | 1.046898 | 0.295147 | 0.4614027 | FALSE |
| MLLT1 | 12450 | 1.046125 | 0.295503 | 0.4617756 | FALSE |
| CSMD3 | 2093 | 1.045972 | 0.295574 | 0.4617937 | FALSE |
| DGKQ | 13743 | 1.045402 | 0.295837 | 0.4621588 | FALSE |
| TMEM40 | 9694 | 1.045291 | 0.295888 | 0.4621927 | FALSE |
| JAZF1 | 14848 | 1.04513 | 0.295963 | 0.462244 | FALSE |
| KLF3-AS1 | 7856 | 1.045092 | 0.29598 | 0.462244 | FALSE |
| LOC148413 | 10528 | 1.044449 | 0.296278 | 0.4625696 | FALSE |
| IFI44 | 8149 | 1.044288 | 0.296352 | 0.4626396 | FALSE |
| OR13H1 | 1996 | 1.043975 | 0.296497 | 0.4627733 | FALSE |
| CDK5R2 | 1513 | 1.043527 | 0.296704 | 0.4630044 | FALSE |
| PCYT2 | 3151 | 1.043362 | 0.296781 | 0.4630774 | FALSE |
| NRAS | 9157 | 1.043163 | 0.296873 | 0.4631749 | FALSE |
| CECR2 | 5672 | 1.04309 | 0.296907 | 0.4631814 | FALSE |
| ZNF45 | 10451 | 1.042753 | 0.297063 | 0.4633325 | FALSE |
| GPANK1 | 3808 | 1.042469 | 0.297194 | 0.4634452 | FALSE |
| SLC25A44 | 1797 | 1.042097 | 0.297367 | 0.4636317 | FALSE |
| CUEDC2 | 513 | 1.041323 | 0.297726 | 0.4640424 | FALSE |
| AJAP1 | 14156 | 1.041155 | 0.297804 | 0.4640895 | FALSE |
| FOXR1 | 8500 | 1.041103 | 0.297828 | 0.4640895 | FALSE |
| NRP1 | 11017 | 1.041066 | 0.297845 | 0.4640895 | FALSE |
| NANOS3 | 9464 | 1.040283 | 0.298208 | 0.4646096 | FALSE |
| RIN3 | 11526 | 1.039445 | 0.298598 | 0.4650772 | FALSE |
| LINC01126 | 4591 | 1.038785 | 0.298905 | 0.4653777 | FALSE |
| ATP6V0A1 | 8429 | 1.038769 | 0.298912 | 0.4653777 | FALSE |
| ZSWIM1 | 10175 | 1.038426 | 0.299072 | 0.4654444 | FALSE |
| ZBTB22 | 8628 | 1.038337 | 0.299113 | 0.4654625 | FALSE |
| SIGLEC6 | 6999 | 1.036621 | 0.299913 | 0.4665671 | FALSE |
| APH1B | 7899 | 1.036458 | 0.299989 | 0.4666222 | FALSE |
| ZNF253 | 11905 | 1.036417 | 0.300008 | 0.4666222 | FALSE |
| TONSL | 360 | 1.036059 | 0.300175 | 0.4668224 | FALSE |
| MAN2B1 | 12771 | 1.035665 | 0.300358 | 0.4670284 | FALSE |
| RTN4R | 7675 | 1.034949 | 0.300693 | 0.4673158 | FALSE |
| ZNF728 | 12801 | 1.034661 | 0.300827 | 0.4674784 | FALSE |
| VAMP5 | 3104 | 1.033906 | 0.30118 | 0.4679337 | FALSE |
| STT3B | 1078 | 1.033379 | 0.301427 | 0.4682701 | FALSE |
| ZG16B | 12865 | 1.033258 | 0.301483 | 0.4683115 | FALSE |
| CDC42SE2 | 8279 | 1.032994 | 0.301607 | 0.4684103 | FALSE |
| CDCA7L | 1044 | 1.032896 | 0.301653 | 0.468435 | FALSE |
| ALOX15B | 8127 | 1.032637 | 0.301774 | 0.4685301 | FALSE |
| CCDC15 | 7889 | 1.032335 | 0.301915 | 0.4687031 | FALSE |
| LOC643802 | 7446 | 1.032117 | 0.302017 | 0.4687823 | FALSE |
| PCDH18 | 7178 | 1.032098 | 0.302026 | 0.4687823 | FALSE |
| TXNRD3 | 4503 | 1.031561 | 0.302278 | 0.4691263 | FALSE |
| CANT1 | 14858 | 1.031135 | 0.302478 | 0.469343 | FALSE |
| VGLL3 | 7276 | 1.030947 | 0.302566 | 0.4694332 | FALSE |
| MON1A | 8443 | 1.029317 | 0.303331 | 0.4703664 | FALSE |
| PMP22 | 13032 | 1.028852 | 0.303549 | 0.4706323 | FALSE |
| FAM47C | 8254 | 1.028023 | 0.303939 | 0.4711431 | FALSE |
| VTI1A | 897 | 1.027795 | 0.304046 | 0.471216 | FALSE |
| GRM5 | 4638 | 1.027434 | 0.304216 | 0.4714288 | FALSE |
| HDGFL1 | 581 | 1.02732 | 0.30427 | 0.4714288 | FALSE |
| SNX6 | 14676 | 1.027311 | 0.304274 | 0.4714288 | FALSE |
| WDR11 | 15489 | 1.02637 | 0.304717 | 0.4720218 | FALSE |
| WARS | 8715 | 1.025933 | 0.304923 | 0.4721902 | FALSE |
| WBP2 | 2517 | 1.025883 | 0.304947 | 0.4721902 | FALSE |
| ACAT1 | 8133 | 1.025093 | 0.305319 | 0.4727203 | FALSE |
| AGAP2-AS1 | 5492 | 1.024717 | 0.305497 | 0.4729482 | FALSE |
| UBE2I | 5207 | 1.024453 | 0.305621 | 0.4730006 | FALSE |
| LSM5 | 1487 | 1.024259 | 0.305713 | 0.4730955 | FALSE |
| ST6GALNAC3 | 11484 | 1.02403 | 0.305821 | 0.4731706 | FALSE |
| RAB39A | 9644 | 1.023958 | 0.305855 | 0.473175 | FALSE |
| CACNG1 | 8126 | 1.023752 | 0.305952 | 0.4732787 | FALSE |
| LINC01949 | 11251 | 1.023607 | 0.306021 | 0.4733378 | FALSE |
| ACSS3 | 8590 | 1.023043 | 0.306288 | 0.4736564 | FALSE |
| RPS28 | 11505 | 1.022553 | 0.306519 | 0.4738741 | FALSE |
| ZNF91 | 3111 | 1.02217 | 0.3067 | 0.4740502 | FALSE |
| PDE6G | 10278 | 1.021657 | 0.306943 | 0.4742952 | FALSE |
| ACTN2 | 3054 | 1.021336 | 0.307095 | 0.4744831 | FALSE |
| HSPA9 | 3924 | 1.020682 | 0.307405 | 0.4749149 | FALSE |
| MYOM2 | 9024 | 1.020211 | 0.307628 | 0.4751828 | FALSE |
| GPR179 | 12231 | 1.019989 | 0.307734 | 0.4752347 | FALSE |
| CTRC | 3577 | 1.019797 | 0.307825 | 0.4753284 | FALSE |
| RP2 | 7872 | 1.019503 | 0.307964 | 0.4754725 | FALSE |
| AGBL5 | 1578 | 1.019289 | 0.308066 | 0.4755596 | FALSE |
| DBX1 | 9188 | 1.018856 | 0.308271 | 0.475783 | FALSE |
| ZIC1 | 3641 | 1.018409 | 0.308484 | 0.4759842 | FALSE |
| MUC1 | 3962 | 1.018351 | 0.308511 | 0.4759842 | FALSE |
| GAN | 8911 | 1.017772 | 0.308786 | 0.4763427 | FALSE |
| FGF8 | 9699 | 1.017605 | 0.308866 | 0.4764085 | FALSE |
| CXorf38 | 4009 | 1.017554 | 0.30889 | 0.4764085 | FALSE |
| HMGN3 | 952 | 1.017385 | 0.30897 | 0.4764854 | FALSE |
| KLHDC7A | 14641 | 1.017315 | 0.309004 | 0.4764898 | FALSE |
| MAPRE2 | 876 | 1.017219 | 0.309049 | 0.4765132 | FALSE |
| C10orf99 | 3578 | 1.01715 | 0.309082 | 0.4765168 | FALSE |
| FAM189A1 | 5229 | 1.016038 | 0.309611 | 0.4772856 | FALSE |
| GPR12 | 14252 | 1.015551 | 0.309843 | 0.477549 | FALSE |
| OLIG1 | 7374 | 1.015196 | 0.310012 | 0.4777626 | FALSE |
| RFTN2 | 7495 | 1.015119 | 0.310049 | 0.4777721 | FALSE |
| WDR78 | 11953 | 1.014357 | 0.310412 | 0.4781087 | FALSE |
| CDC42EP5 | 1135 | 1.014287 | 0.310446 | 0.4781087 | FALSE |
| BLM | 11052 | 1.014276 | 0.310451 | 0.4781087 | FALSE |
| APTX | 8437 | 1.013933 | 0.310615 | 0.4783136 | FALSE |
| HOXA5 | 14645 | 1.013521 | 0.310811 | 0.4785222 | FALSE |
| TIMM17A | 352 | 1.013066 | 0.311029 | 0.4788095 | FALSE |
| TTC3P1 | 15443 | 1.012902 | 0.311107 | 0.4788358 | FALSE |
| MAP3K2 | 14862 | 1.012783 | 0.311164 | 0.4788762 | FALSE |
| SMIM15 | 8631 | 1.011988 | 0.311544 | 0.4793627 | FALSE |
| KRTAP2-3 | 12942 | 1.011919 | 0.311577 | 0.4793627 | FALSE |
| IPO8 | 9210 | 1.011634 | 0.311713 | 0.4794383 | FALSE |
| MYADML | 10522 | 1.011241 | 0.311901 | 0.479664 | FALSE |
| EML1 | 5278 | 1.011199 | 0.311921 | 0.479664 | FALSE |
| MXD3 | 1325 | 1.011123 | 0.311958 | 0.4796727 | FALSE |
| HK1 | 2333 | 1.010865 | 0.312081 | 0.4797807 | FALSE |
| ATP7B | 12163 | 1.010848 | 0.312089 | 0.4797807 | FALSE |
| RPRM | 9165 | 1.010099 | 0.312448 | 0.4802849 | FALSE |
| GLCE | 11554 | 1.009419 | 0.312774 | 0.480644 | FALSE |
| SLC35E4 | 636 | 1.009315 | 0.312824 | 0.4806688 | FALSE |
| ZRANB3 | 13133 | 1.009253 | 0.312853 | 0.4806688 | FALSE |
| GRB7 | 14018 | 1.009048 | 0.312952 | 0.4807284 | FALSE |
| SRA1 | 13961 | 1.008573 | 0.313179 | 0.4810311 | FALSE |
| C11orf71 | 6508 | 1.008161 | 0.313377 | 0.4812402 | FALSE |
| DNLZ | 15381 | 1.007838 | 0.313532 | 0.481431 | FALSE |
| BHLHB9 | 1257 | 1.007179 | 0.313849 | 0.4818119 | FALSE |
| PCSK9 | 11431 | 1.007128 | 0.313873 | 0.4818119 | FALSE |
| PREX1 | 14499 | 1.007065 | 0.313904 | 0.4818119 | FALSE |
| CCNF | 14832 | 1.0066 | 0.314127 | 0.482013 | FALSE |
| DROSHA | 10495 | 1.005664 | 0.314577 | 0.482455 | FALSE |
| SNAI3-AS1 | 10199 | 1.005552 | 0.314631 | 0.482455 | FALSE |
| EMC4 | 5316 | 1.005442 | 0.314684 | 0.4824888 | FALSE |
| NUP88 | 13730 | 1.004871 | 0.314959 | 0.4827683 | FALSE |
| DUSP3 | 6570 | 1.004624 | 0.315078 | 0.4829033 | FALSE |
| SYNGAP1 | 4101 | 1.004271 | 0.315248 | 0.4830692 | FALSE |
| FBRSL1 | 5774 | 1.004048 | 0.315355 | 0.4831517 | FALSE |
| NADSYN1 | 14716 | 1.004031 | 0.315364 | 0.4831517 | FALSE |
| CHST11 | 5276 | 1.003684 | 0.315531 | 0.4832245 | FALSE |
| HNF1A | 7768 | 1.002874 | 0.315922 | 0.4836748 | FALSE |
| STEAP3 | 6065 | 1.002292 | 0.316203 | 0.4840483 | FALSE |
| PKMYT1 | 13004 | 1.002171 | 0.316261 | 0.4840483 | FALSE |
| ZER1 | 11375 | 1.002112 | 0.31629 | 0.4840483 | FALSE |
| ZNF18 | 14860 | 1.001538 | 0.316567 | 0.4843673 | FALSE |
| PEAR1 | 9787 | 1.001066 | 0.316795 | 0.4845846 | FALSE |
| MAP3K5 | 1054 | 1.000697 | 0.316973 | 0.4847315 | FALSE |
| UBXN11 | 12964 | 1.000675 | 0.316984 | 0.4847315 | FALSE |
| USP14 | 12873 | 1.000463 | 0.317086 | 0.4847475 | FALSE |
| CREB3L3 | 12485 | 1.000461 | 0.317087 | 0.4847475 | FALSE |
| TMEM186 | 4155 | 0.999628 | 0.317491 | 0.4851895 | FALSE |
| ZNF738 | 6470 | 0.999607 | 0.317501 | 0.4851895 | FALSE |
| RGS12 | 8796 | 0.99921 | 0.317693 | 0.4853884 | FALSE |
| DNASE2 | 1442 | 0.998797 | 0.317893 | 0.4856466 | FALSE |
| P3H4 | 6686 | 0.997923 | 0.318317 | 0.4861038 | FALSE |
| PLEKHF2 | 2383 | 0.997781 | 0.318386 | 0.486114 | FALSE |
| C2CD4A | 6259 | 0.997268 | 0.318634 | 0.4864072 | FALSE |
| KLF3 | 13578 | 0.997257 | 0.31864 | 0.4864072 | FALSE |
| DRICH1 | 503 | 0.997056 | 0.318737 | 0.4865086 | FALSE |
| PIWIL4 | 10729 | 0.99699 | 0.318769 | 0.48651 | FALSE |
| STARD7-AS1 | 4629 | 0.996169 | 0.319168 | 0.4870709 | FALSE |
| OTX1 | 6222 | 0.99574 | 0.319376 | 0.4873413 | FALSE |
| UTP20 | 14854 | 0.995676 | 0.319408 | 0.4873413 | FALSE |
| ADIPOR2 | 11543 | 0.994791 | 0.319838 | 0.4878669 | FALSE |
| HAGLROS | 6393 | 0.994775 | 0.319846 | 0.4878669 | FALSE |
| DNAJB7 | 13128 | 0.994606 | 0.319928 | 0.4879409 | FALSE |
| CBX2 | 7855 | 0.994547 | 0.319957 | 0.4879409 | FALSE |
| AGBL4 | 853 | 0.994468 | 0.319995 | 0.4879519 | FALSE |
| PIGP | 15203 | 0.993778 | 0.320331 | 0.4884165 | FALSE |
| TNFAIP6 | 3369 | 0.992863 | 0.320777 | 0.4887542 | FALSE |
| TMEM8A | 4183 | 0.992651 | 0.32088 | 0.4888246 | FALSE |
| SELENOF | 3606 | 0.992125 | 0.321137 | 0.489111 | FALSE |
| NUP133 | 8816 | 0.991833 | 0.321279 | 0.4892416 | FALSE |
| NKD2 | 13207 | 0.991621 | 0.321382 | 0.4893515 | FALSE |
| LOC145783 | 2607 | 0.990896 | 0.321736 | 0.4898427 | FALSE |
| MMP19 | 1311 | 0.990756 | 0.321805 | 0.4898603 | FALSE |
| OR4K2 | 11533 | 0.990624 | 0.321869 | 0.4899018 | FALSE |
| VSTM2B | 5222 | 0.990252 | 0.322051 | 0.4900211 | FALSE |
| BTD | 6672 | 0.990207 | 0.322073 | 0.4900211 | FALSE |
| GRIK1 | 607 | 0.989681 | 0.32233 | 0.4902214 | FALSE |
| LOC441666 | 5792 | 0.989479 | 0.322429 | 0.4902762 | FALSE |
| TSR1 | 8649 | 0.988748 | 0.322786 | 0.4906768 | FALSE |
| NDUFB2 | 10659 | 0.987691 | 0.323304 | 0.4913203 | FALSE |
| SEC14L2 | 11696 | 0.98753 | 0.323383 | 0.4913625 | FALSE |
| CKAP4 | 12493 | 0.987506 | 0.323395 | 0.4913625 | FALSE |
| MMP9 | 2414 | 0.987395 | 0.323449 | 0.4913868 | FALSE |
| ARRDC4 | 12120 | 0.987345 | 0.323474 | 0.4913868 | FALSE |
| IST1 | 3554 | 0.985851 | 0.324206 | 0.4922203 | FALSE |
| ELFN1 | 14303 | 0.985841 | 0.324211 | 0.4922203 | FALSE |
| ZNF843 | 2241 | 0.98555 | 0.324354 | 0.4922735 | FALSE |
| PKN1 | 9926 | 0.985525 | 0.324366 | 0.4922735 | FALSE |
| GCM1 | 10965 | 0.985516 | 0.324371 | 0.4922735 | FALSE |
| ATP6V0E2-AS1 | 7022 | 0.985513 | 0.324372 | 0.4922735 | FALSE |
| ANP32C | 13484 | 0.985406 | 0.324425 | 0.4923055 | FALSE |
| C20orf141 | 4894 | 0.985212 | 0.32452 | 0.4924022 | FALSE |
| FBXO3 | 682 | 0.984424 | 0.324907 | 0.4928885 | FALSE |
| MYO1C | 7083 | 0.984315 | 0.324961 | 0.4928885 | FALSE |
| ZNF626 | 5020 | 0.984303 | 0.324967 | 0.4928885 | FALSE |
| UMAD1 | 10143 | 0.984014 | 0.325109 | 0.4930562 | FALSE |
| PRRC2C | 2087 | 0.983272 | 0.325474 | 0.4934331 | FALSE |
| TMPRSS6 | 5263 | 0.983252 | 0.325483 | 0.4934331 | FALSE |
| ILVBL | 15166 | 0.983062 | 0.325577 | 0.4934792 | FALSE |
| GRIA3 | 12564 | 0.982997 | 0.325609 | 0.4934798 | FALSE |
| KRTAP12-1 | 10574 | 0.982827 | 0.325693 | 0.4935588 | FALSE |
| SGSM1 | 2382 | 0.982618 | 0.325795 | 0.4936147 | FALSE |
| JADE3 | 3458 | 0.982566 | 0.325821 | 0.4936147 | FALSE |
| CEP128 | 6890 | 0.982088 | 0.326056 | 0.4936795 | FALSE |
| LMAN1L | 5708 | 0.980973 | 0.326606 | 0.4943201 | FALSE |
| ZFHX4 | 5336 | 0.980838 | 0.326673 | 0.4943632 | FALSE |
| DEFB123 | 14014 | 0.980787 | 0.326698 | 0.4943632 | FALSE |
| PKP1 | 4588 | 0.98056 | 0.32681 | 0.4944848 | FALSE |
| GUCY1B1 | 11770 | 0.98021 | 0.326982 | 0.4946982 | FALSE |
| RAB2B | 11933 | 0.980019 | 0.327077 | 0.494778 | FALSE |
| KLF7 | 4230 | 0.979273 | 0.327445 | 0.4951586 | FALSE |
| ST7-AS1 | 12578 | 0.979161 | 0.3275 | 0.4951943 | FALSE |
| YIPF1 | 10224 | 0.978996 | 0.327582 | 0.4952697 | FALSE |
| TM2D1 | 2155 | 0.978775 | 0.327691 | 0.4953869 | FALSE |
| LIPC | 1062 | 0.978607 | 0.327774 | 0.4954645 | FALSE |
| HLA-DOB | 10391 | 0.978318 | 0.327917 | 0.4956326 | FALSE |
| HINT3 | 6426 | 0.977603 | 0.328271 | 0.4959954 | FALSE |
| FAM204A | 6998 | 0.977565 | 0.32829 | 0.4959954 | FALSE |
| PEX7 | 11768 | 0.977349 | 0.328396 | 0.4960693 | FALSE |
| USP37 | 8163 | 0.977156 | 0.328492 | 0.4961656 | FALSE |
| PLK2 | 9671 | 0.976393 | 0.32887 | 0.4966725 | FALSE |
| ZNF28 | 3847 | 0.976154 | 0.328988 | 0.4967232 | FALSE |
| AGXT | 13542 | 0.975786 | 0.329171 | 0.4969025 | FALSE |
| TLE1 | 15247 | 0.975628 | 0.329249 | 0.4969728 | FALSE |
| PSMD7 | 4144 | 0.974515 | 0.329801 | 0.4976573 | FALSE |
| ZNF724 | 493 | 0.974457 | 0.32983 | 0.4976573 | FALSE |
| OR2H2 | 6532 | 0.973948 | 0.330082 | 0.4977173 | FALSE |
| CHRAC1 | 1228 | 0.973928 | 0.330092 | 0.4977173 | FALSE |
| OR10A5 | 8021 | 0.973695 | 0.330208 | 0.4977957 | FALSE |
| EIF3G | 7408 | 0.973208 | 0.33045 | 0.4979684 | FALSE |
| LZTR1 | 5015 | 0.971947 | 0.331077 | 0.498717 | FALSE |
| ABCF3 | 5959 | 0.971834 | 0.331133 | 0.498717 | FALSE |
| SLC25A28 | 5668 | 0.971824 | 0.331138 | 0.498717 | FALSE |
| CST3 | 11164 | 0.970717 | 0.331689 | 0.4994094 | FALSE |
| MYH11 | 4778 | 0.970713 | 0.331691 | 0.4994094 | FALSE |
| TMEM198 | 4135 | 0.970708 | 0.331694 | 0.4994094 | FALSE |
| SMIM14 | 14546 | 0.970187 | 0.331953 | 0.4995597 | FALSE |
| ABI2 | 2841 | 0.969562 | 0.332265 | 0.4999323 | FALSE |
| ASTL | 11284 | 0.967928 | 0.33308 | 0.5007307 | FALSE |
| RANBP3 | 11570 | 0.967878 | 0.333105 | 0.5007307 | FALSE |
| DTWD1 | 10616 | 0.967857 | 0.333116 | 0.5007307 | FALSE |
| ANKLE2 | 4513 | 0.967269 | 0.33341 | 0.5010759 | FALSE |
| COX8A | 6567 | 0.966729 | 0.33368 | 0.5013209 | FALSE |
| RIN1 | 922 | 0.966725 | 0.333682 | 0.5013209 | FALSE |
| GPR157 | 5480 | 0.966617 | 0.333736 | 0.5013209 | FALSE |
| EXOSC1 | 13034 | 0.966557 | 0.333766 | 0.5013209 | FALSE |
| IGSF8 | 4293 | 0.966087 | 0.334001 | 0.5014053 | FALSE |
| CCDC158 | 3105 | 0.966021 | 0.334034 | 0.5014053 | FALSE |
| GPR83 | 7079 | 0.96543 | 0.334329 | 0.5017349 | FALSE |
| BTBD18 | 10375 | 0.965297 | 0.334396 | 0.5017867 | FALSE |
| SCGB2A2 | 9342 | 0.965176 | 0.334457 | 0.5018295 | FALSE |
| HIST3H2BB | 1038 | 0.965006 | 0.334542 | 0.5019044 | FALSE |
| RPAP1 | 8465 | 0.964886 | 0.334602 | 0.5019044 | FALSE |
| AKIP1 | 14984 | 0.964884 | 0.334603 | 0.5019044 | FALSE |
| ZNF577 | 12107 | 0.964175 | 0.334958 | 0.5023409 | FALSE |
| C19orf24 | 13684 | 0.964045 | 0.335023 | 0.5023904 | FALSE |
| STX18 | 11709 | 0.963915 | 0.335089 | 0.50244 | FALSE |
| CRK | 6714 | 0.963757 | 0.335168 | 0.5025106 | FALSE |
| MTA1 | 14086 | 0.963183 | 0.335456 | 0.5027494 | FALSE |
| RFLNB | 3175 | 0.962854 | 0.335621 | 0.5029486 | FALSE |
| GDAP1 | 7251 | 0.961573 | 0.336264 | 0.5036713 | FALSE |
| CDH2 | 12063 | 0.960665 | 0.336721 | 0.5041467 | FALSE |
| GLUL | 5010 | 0.960621 | 0.336743 | 0.5041467 | FALSE |
| SLC38A7 | 9836 | 0.960494 | 0.336807 | 0.5041608 | FALSE |
| VARS2 | 12917 | 0.960474 | 0.336817 | 0.5041608 | FALSE |
| CHRNA4 | 3318 | 0.960106 | 0.337002 | 0.5043414 | FALSE |
| ZAP70 | 7861 | 0.959013 | 0.337552 | 0.5049716 | FALSE |
| PPP2CB | 5036 | 0.958268 | 0.337928 | 0.5054365 | FALSE |
| FOXA2 | 11899 | 0.956885 | 0.338625 | 0.506321 | FALSE |
| TBC1D10C | 1146 | 0.956487 | 0.338826 | 0.5064898 | FALSE |
| SYNGR2 | 5508 | 0.955695 | 0.339226 | 0.506991 | FALSE |
| KDM4B | 13112 | 0.95499 | 0.339583 | 0.5074266 | FALSE |
| RHOB | 2064 | 0.954636 | 0.339762 | 0.5075637 | FALSE |
| CYP3A43 | 15178 | 0.954489 | 0.339836 | 0.5076112 | FALSE |
| CD2BP2 | 9299 | 0.954176 | 0.339995 | 0.5077023 | FALSE |
| CSAG3 | 13375 | 0.953866 | 0.340151 | 0.5078881 | FALSE |
| FLJ42393 | 552 | 0.953645 | 0.340263 | 0.5079596 | FALSE |
| TBC1D22A | 12122 | 0.952399 | 0.340895 | 0.5088 | FALSE |
| EFHC1 | 4266 | 0.951474 | 0.341364 | 0.5093091 | FALSE |
| ARMCX6 | 15141 | 0.950967 | 0.341621 | 0.5095471 | FALSE |
| IKZF2 | 2524 | 0.949127 | 0.342556 | 0.5108441 | FALSE |
| LOC728739 | 1937 | 0.948877 | 0.342683 | 0.5109849 | FALSE |
| FAM92A1P2 | 9197 | 0.948672 | 0.342787 | 0.5110429 | FALSE |
| MBD5 | 15081 | 0.947558 | 0.343355 | 0.5116932 | FALSE |
| ATP5MPL | 3981 | 0.946755 | 0.343764 | 0.5120367 | FALSE |
| COQ4 | 1759 | 0.946609 | 0.343838 | 0.5120367 | FALSE |
| NACAP1 | 7718 | 0.946527 | 0.34388 | 0.5120367 | FALSE |
| CD79A | 2927 | 0.946312 | 0.343989 | 0.5121512 | FALSE |
| ZBTB37 | 4048 | 0.945917 | 0.344191 | 0.5123465 | FALSE |
| FAM13C | 3943 | 0.94513 | 0.344593 | 0.5127735 | FALSE |
| EXD3 | 10367 | 0.945107 | 0.344604 | 0.5127735 | FALSE |
| PLVAP | 11250 | 0.944879 | 0.344721 | 0.5128979 | FALSE |
| MYO6 | 7313 | 0.944667 | 0.344829 | 0.5130101 | FALSE |
| TNNT3 | 15438 | 0.944205 | 0.345065 | 0.5133074 | FALSE |
| ECSCR | 2070 | 0.944126 | 0.345105 | 0.5133074 | FALSE |
| SYT16 | 15603 | 0.944083 | 0.345127 | 0.5133074 | FALSE |
| HNF1A-AS1 | 12069 | 0.943925 | 0.345208 | 0.5133471 | FALSE |
| ZNF56 | 11186 | 0.943838 | 0.345252 | 0.5133471 | FALSE |
| ANKRD13B | 2492 | 0.943451 | 0.34545 | 0.5135656 | FALSE |
| ZNF514 | 9152 | 0.943422 | 0.345465 | 0.5135656 | FALSE |
| CASD1 | 8207 | 0.943164 | 0.345597 | 0.5136448 | FALSE |
| RNF149 | 313 | 0.942583 | 0.345894 | 0.5138615 | FALSE |
| HRH3 | 3381 | 0.942193 | 0.346094 | 0.5141092 | FALSE |
| HSD17B7 | 12310 | 0.941676 | 0.346359 | 0.5144535 | FALSE |
| RNGTT | 13451 | 0.941447 | 0.346476 | 0.5145788 | FALSE |
| CDC123 | 4561 | 0.940952 | 0.346729 | 0.5149066 | FALSE |
| SAMM50 | 7384 | 0.940847 | 0.346783 | 0.5149376 | FALSE |
| CNGB1 | 5523 | 0.940697 | 0.34686 | 0.5150028 | FALSE |
| CERS2 | 10572 | 0.940435 | 0.346994 | 0.5151455 | FALSE |
| CALCA | 2737 | 0.940381 | 0.347022 | 0.5151455 | FALSE |
| ATG2A | 3371 | 0.940258 | 0.347085 | 0.5151824 | FALSE |
| IFITM2 | 5806 | 0.940064 | 0.347185 | 0.5152313 | FALSE |
| NT5E | 14359 | 0.93999 | 0.347223 | 0.5152313 | FALSE |
| KTN1 | 11304 | 0.939947 | 0.347245 | 0.5152313 | FALSE |
| TMEM129 | 4045 | 0.939568 | 0.347439 | 0.5154709 | FALSE |
| EYA1 | 1751 | 0.939361 | 0.347545 | 0.5155467 | FALSE |
| ACKR4 | 15013 | 0.938795 | 0.347836 | 0.5158638 | FALSE |
| GJB6 | 13383 | 0.937937 | 0.348277 | 0.5164195 | FALSE |
| LRRC63 | 10360 | 0.937692 | 0.348403 | 0.5165573 | FALSE |
| MYBL2 | 13066 | 0.93641 | 0.349062 | 0.5173478 | FALSE |
| COX6B2 | 6571 | 0.936398 | 0.349068 | 0.5173478 | FALSE |
| USP27X | 10643 | 0.936052 | 0.349246 | 0.5175627 | FALSE |
| C7orf43 | 15000 | 0.935875 | 0.349338 | 0.5176487 | FALSE |
| INTS3 | 1582 | 0.935589 | 0.349485 | 0.5178179 | FALSE |
| GGNBP2 | 1635 | 0.935229 | 0.34967 | 0.5180436 | FALSE |
| PLXNB2 | 2447 | 0.93447 | 0.350062 | 0.5184266 | FALSE |
| CD14 | 10972 | 0.933282 | 0.350674 | 0.5191574 | FALSE |
| PPP1R11 | 5739 | 0.932845 | 0.3509 | 0.5193729 | FALSE |
| INO80D | 11517 | 0.931718 | 0.351482 | 0.5201365 | FALSE |
| TLE3 | 925 | 0.931648 | 0.351518 | 0.5201409 | FALSE |
| SLC35G2 | 2897 | 0.931447 | 0.351622 | 0.5201962 | FALSE |
| MLLT6 | 1386 | 0.931196 | 0.351752 | 0.5202601 | FALSE |
| SPCS3 | 8516 | 0.931106 | 0.351799 | 0.5202601 | FALSE |
| RPAP3 | 6377 | 0.930432 | 0.352147 | 0.5206916 | FALSE |
| ATP5PO | 14306 | 0.930392 | 0.352168 | 0.5206916 | FALSE |
| BACE1 | 6700 | 0.93025 | 0.352242 | 0.5207092 | FALSE |
| SERPINE2 | 8627 | 0.930179 | 0.352278 | 0.5207092 | FALSE |
| LNPK | 8815 | 0.928196 | 0.353306 | 0.5217487 | FALSE |
| SEMA3E | 7074 | 0.927825 | 0.353498 | 0.5219835 | FALSE |
| LINC01480 | 6225 | 0.927652 | 0.353588 | 0.5220668 | FALSE |
| LIMS1 | 2138 | 0.927414 | 0.353712 | 0.5221066 | FALSE |
| LINC00626 | 3198 | 0.926184 | 0.35435 | 0.5228959 | FALSE |
| TAF7 | 9503 | 0.925953 | 0.35447 | 0.5229742 | FALSE |
| LIPN | 14398 | 0.925796 | 0.354552 | 0.5229742 | FALSE |
| HTRA3 | 1372 | 0.92576 | 0.354571 | 0.5229742 | FALSE |
| ZNF793 | 10296 | 0.92551 | 0.354701 | 0.5230672 | FALSE |
| HPRT1 | 1393 | 0.924969 | 0.354982 | 0.5233448 | FALSE |
| ALK | 12698 | 0.924955 | 0.354989 | 0.5233448 | FALSE |
| MPIG6B | 8978 | 0.924712 | 0.355116 | 0.5234818 | FALSE |
| ETV1 | 1963 | 0.923577 | 0.355707 | 0.5239575 | FALSE |
| TCF4 | 10102 | 0.922592 | 0.35622 | 0.5245159 | FALSE |
| MYPOP | 2033 | 0.921724 | 0.356673 | 0.5250835 | FALSE |
| PNPLA6 | 9846 | 0.920895 | 0.357105 | 0.5255767 | FALSE |
| CCDC40 | 11632 | 0.920889 | 0.357108 | 0.5255767 | FALSE |
| HEBP2 | 495 | 0.920699 | 0.357208 | 0.5256691 | FALSE |
| SCGB3A1 | 10688 | 0.919983 | 0.357582 | 0.5261246 | FALSE |
| TBX5 | 14927 | 0.919641 | 0.35776 | 0.5263381 | FALSE |
| SRP19 | 6464 | 0.919552 | 0.357807 | 0.526357 | FALSE |
| SNRPE | 15565 | 0.919353 | 0.357911 | 0.5264605 | FALSE |
| YTHDC1 | 8031 | 0.918898 | 0.358149 | 0.5266749 | FALSE |
| KRTAP6-1 | 6553 | 0.918882 | 0.358157 | 0.5266749 | FALSE |
| BCAM | 348 | 0.918881 | 0.358158 | 0.5266749 | FALSE |
| XRCC3 | 5293 | 0.918728 | 0.358238 | 0.5267431 | FALSE |
| PROS1 | 9158 | 0.918234 | 0.358496 | 0.5270337 | FALSE |
| BST2 | 6582 | 0.918192 | 0.358518 | 0.5270337 | FALSE |
| ATP5S | 3762 | 0.917816 | 0.358715 | 0.5272466 | FALSE |
| ZMYND19 | 3251 | 0.917716 | 0.358768 | 0.527274 | FALSE |
| OR4D10 | 2134 | 0.916993 | 0.359146 | 0.52757 | FALSE |
| MPND | 336 | 0.916957 | 0.359165 | 0.52757 | FALSE |
| LLPH | 4276 | 0.916706 | 0.359297 | 0.5277044 | FALSE |
| LMBRD1 | 6746 | 0.916452 | 0.35943 | 0.5278504 | FALSE |
| PTP4A2 | 2648 | 0.916287 | 0.359516 | 0.5279278 | FALSE |
| MRM3 | 2188 | 0.916167 | 0.359579 | 0.5279706 | FALSE |
| PYROXD2 | 7884 | 0.916074 | 0.359628 | 0.5279927 | FALSE |
| SNORD22 | 10781 | 0.91482 | 0.360286 | 0.5288341 | FALSE |
| FAM184B | 13233 | 0.914745 | 0.360326 | 0.5288341 | FALSE |
| CD248 | 6102 | 0.914724 | 0.360337 | 0.5288341 | FALSE |
| ABHD2 | 15533 | 0.914032 | 0.3607 | 0.5292682 | FALSE |
| PRIMA1 | 9209 | 0.913756 | 0.360845 | 0.5294017 | FALSE |
| METTL8 | 8146 | 0.91373 | 0.360859 | 0.5294017 | FALSE |
| GUF1 | 14150 | 0.913361 | 0.361053 | 0.5296366 | FALSE |
| ZNF85 | 12648 | 0.91317 | 0.361153 | 0.5296845 | FALSE |
| LOC440300 | 2904 | 0.912387 | 0.361565 | 0.5301891 | FALSE |
| STK31 | 6695 | 0.912205 | 0.361661 | 0.5302248 | FALSE |
| LRRC75A | 6657 | 0.912192 | 0.361668 | 0.5302248 | FALSE |
| TAP2 | 8047 | 0.912083 | 0.361725 | 0.5302248 | FALSE |
| IQGAP3 | 13694 | 0.91198 | 0.361779 | 0.5302545 | FALSE |
| ACTL6A | 7154 | 0.911324 | 0.362125 | 0.5306613 | FALSE |
| ADAM17 | 13968 | 0.911111 | 0.362237 | 0.5307512 | FALSE |
| SLC37A1 | 9810 | 0.911016 | 0.362287 | 0.5307512 | FALSE |
| DPH2 | 5298 | 0.910978 | 0.362307 | 0.5307512 | FALSE |
| CCDC88C | 4875 | 0.910898 | 0.362349 | 0.5307512 | FALSE |
| AACS | 2074 | 0.910822 | 0.362389 | 0.5307512 | FALSE |
| GRHPR | 5632 | 0.909858 | 0.362897 | 0.5313952 | FALSE |
| SH3YL1 | 14553 | 0.90967 | 0.362997 | 0.5314408 | FALSE |
| KLK3 | 1327 | 0.908155 | 0.363796 | 0.5322606 | FALSE |
| LRP2 | 1884 | 0.908073 | 0.36384 | 0.5322606 | FALSE |
| FAHD2CP | 4184 | 0.908065 | 0.363844 | 0.5322606 | FALSE |
| SIM2 | 11160 | 0.908029 | 0.363863 | 0.5322606 | FALSE |
| RPL14 | 9419 | 0.907652 | 0.364062 | 0.532396 | FALSE |
| TNNI1 | 4768 | 0.907368 | 0.364212 | 0.5325224 | FALSE |
| RFNG | 3771 | 0.907188 | 0.364307 | 0.5326117 | FALSE |
| AP3D1 | 14118 | 0.9067 | 0.364565 | 0.5328269 | FALSE |
| FLAD1 | 4326 | 0.906652 | 0.364591 | 0.5328269 | FALSE |
| TSTD3 | 7848 | 0.906375 | 0.364737 | 0.5329912 | FALSE |
| RTL4 | 11637 | 0.905825 | 0.365028 | 0.5332419 | FALSE |
| MRPL52 | 10939 | 0.905823 | 0.36503 | 0.5332419 | FALSE |
| RAB33B | 5791 | 0.905377 | 0.365266 | 0.533464 | FALSE |
| ATP6V1G2 | 5215 | 0.90476 | 0.365593 | 0.5338664 | FALSE |
| PSRC1 | 6006 | 0.904728 | 0.36561 | 0.5338664 | FALSE |
| DCD | 10010 | 0.904414 | 0.365776 | 0.5340595 | FALSE |
| BTN3A2 | 5057 | 0.903993 | 0.365999 | 0.5342856 | FALSE |
| SAMD4A | 8368 | 0.903643 | 0.366185 | 0.5343551 | FALSE |
| DMRTA1 | 3211 | 0.903581 | 0.366218 | 0.5343551 | FALSE |
| JPH1 | 11820 | 0.9033 | 0.366367 | 0.5344728 | FALSE |
| GLIPR1L2 | 4299 | 0.902993 | 0.36653 | 0.5346107 | FALSE |
| OR4D6 | 9183 | 0.902917 | 0.36657 | 0.5346196 | FALSE |
| MSH3 | 12169 | 0.902772 | 0.366647 | 0.5346399 | FALSE |
| COQ9 | 8285 | 0.902762 | 0.366652 | 0.5346399 | FALSE |
| FOXD4L5 | 12984 | 0.901851 | 0.367136 | 0.5351376 | FALSE |
| ARPC4 | 1057 | 0.901797 | 0.367165 | 0.5351376 | FALSE |
| PPP1R16A | 6727 | 0.901649 | 0.367243 | 0.5352023 | FALSE |
| RPUSD3 | 7592 | 0.901423 | 0.367363 | 0.5353275 | FALSE |
| RRAGD | 4334 | 0.901047 | 0.367563 | 0.5355688 | FALSE |
| CSPG5 | 676 | 0.900495 | 0.367857 | 0.5359466 | FALSE |
| CHTF8 | 1403 | 0.90023 | 0.367998 | 0.5360106 | FALSE |
| NFASC | 9983 | 0.900219 | 0.368004 | 0.5360106 | FALSE |
| COLGALT2 | 10004 | 0.899926 | 0.36816 | 0.5361788 | FALSE |
| SLC23A3 | 7904 | 0.899813 | 0.36822 | 0.5361788 | FALSE |
| CDK16 | 2516 | 0.899623 | 0.368321 | 0.5361788 | FALSE |
| SH3D19 | 4940 | 0.899574 | 0.368347 | 0.5361788 | FALSE |
| ST8SIA4 | 13487 | 0.899551 | 0.368359 | 0.5361788 | FALSE |
| WDR34 | 2928 | 0.899274 | 0.368507 | 0.5363435 | FALSE |
| RNF167 | 1203 | 0.898681 | 0.368823 | 0.5366797 | FALSE |
| THADA | 11382 | 0.898647 | 0.368841 | 0.5366797 | FALSE |
| WDR48 | 6078 | 0.89758 | 0.36941 | 0.5374073 | FALSE |
| KISS1 | 11640 | 0.897205 | 0.36961 | 0.5375482 | FALSE |
| LAMTOR1 | 5296 | 0.896733 | 0.369861 | 0.5378145 | FALSE |
| SLC9A9 | 15071 | 0.896518 | 0.369976 | 0.5379201 | FALSE |
| CFAP65 | 11059 | 0.896468 | 0.370003 | 0.5379201 | FALSE |
| INE1 | 11732 | 0.896031 | 0.370236 | 0.538205 | FALSE |
| CCDC177 | 11982 | 0.895972 | 0.370268 | 0.538205 | FALSE |
| SOX18 | 3783 | 0.895825 | 0.370346 | 0.5382691 | FALSE |
| KRTAP5-AS1 | 6067 | 0.895523 | 0.370508 | 0.5384536 | FALSE |
| MUC5B | 5647 | 0.895418 | 0.370564 | 0.538485 | FALSE |
| LSM6 | 8838 | 0.895074 | 0.370748 | 0.5386521 | FALSE |
| SLC5A10 | 4042 | 0.894785 | 0.370902 | 0.5387764 | FALSE |
| CWC27 | 14128 | 0.894364 | 0.371127 | 0.5390533 | FALSE |
| NPW | 110 | 0.893914 | 0.371368 | 0.5393529 | FALSE |
| STAT5A | 12465 | 0.893409 | 0.371638 | 0.5396953 | FALSE |
| MRPS18C | 2204 | 0.892765 | 0.371983 | 0.5400456 | FALSE |
| AKIRIN1 | 723 | 0.891515 | 0.372653 | 0.5407169 | FALSE |
| APCS | 4347 | 0.891366 | 0.372733 | 0.5407826 | FALSE |
| AP1M1 | 2702 | 0.890783 | 0.373046 | 0.5410857 | FALSE |
| CASP7 | 1058 | 0.890544 | 0.373174 | 0.5412215 | FALSE |
| MFSD4B | 647 | 0.890414 | 0.373244 | 0.5412725 | FALSE |
| PROCR | 10724 | 0.890206 | 0.373355 | 0.5413426 | FALSE |
| KLK8 | 9713 | 0.890161 | 0.373379 | 0.5413426 | FALSE |
| POLR1C | 4288 | 0.890088 | 0.373419 | 0.5413426 | FALSE |
| PTP4A3 | 2733 | 0.890066 | 0.37343 | 0.5413426 | FALSE |
| MRAP2 | 6709 | 0.889754 | 0.373598 | 0.541531 | FALSE |
| PMPCB | 9198 | 0.889633 | 0.373663 | 0.541531 | FALSE |
| C17orf51 | 1103 | 0.889597 | 0.373682 | 0.541531 | FALSE |
| KCTD10 | 1693 | 0.889309 | 0.373837 | 0.5415804 | FALSE |
| ASIC4 | 11235 | 0.888321 | 0.374368 | 0.5422649 | FALSE |
| NOC4L | 8872 | 0.888141 | 0.374465 | 0.5423202 | FALSE |
| ANKRD36BP2 | 9878 | 0.888101 | 0.374486 | 0.5423202 | FALSE |
| GRAMD2B | 11472 | 0.887575 | 0.374769 | 0.5426294 | FALSE |
| CFAP73 | 2381 | 0.887455 | 0.374834 | 0.5426727 | FALSE |
| COX4I1 | 8044 | 0.886999 | 0.375079 | 0.5429778 | FALSE |
| ZNHIT2 | 12969 | 0.884899 | 0.376211 | 0.5444048 | FALSE |
| LRRC1 | 2016 | 0.884744 | 0.376295 | 0.5444048 | FALSE |
| LOC100128079 | 2922 | 0.884685 | 0.376327 | 0.5444048 | FALSE |
| SYT4 | 5533 | 0.884653 | 0.376344 | 0.5444048 | FALSE |
| SECTM1 | 14034 | 0.884321 | 0.376523 | 0.5445128 | FALSE |
| SMUG1 | 9541 | 0.884103 | 0.376641 | 0.5446134 | FALSE |
| CARTPT | 6772 | 0.884063 | 0.376662 | 0.5446134 | FALSE |
| XIST | 1739 | 0.883638 | 0.376892 | 0.5448287 | FALSE |
| IAPP | 5749 | 0.883007 | 0.377232 | 0.5450636 | FALSE |
| EGR4 | 8116 | 0.882621 | 0.377441 | 0.5452857 | FALSE |
| TCF15 | 2704 | 0.881626 | 0.377979 | 0.5458107 | FALSE |
| DBF4B | 3661 | 0.881515 | 0.378039 | 0.545847 | FALSE |
| TOPBP1 | 5838 | 0.881406 | 0.378098 | 0.5458818 | FALSE |
| STXBP6 | 3488 | 0.881233 | 0.378192 | 0.5459665 | FALSE |
| POC5 | 2175 | 0.880786 | 0.378434 | 0.5461645 | FALSE |
| TREML1 | 7976 | 0.880311 | 0.378691 | 0.5463339 | FALSE |
| DHRS4L1 | 7126 | 0.880172 | 0.378766 | 0.5463921 | FALSE |
| GFPT1 | 10386 | 0.879833 | 0.37895 | 0.5466066 | FALSE |
| XKR8 | 13132 | 0.879539 | 0.379109 | 0.5467859 | FALSE |
| IFI35 | 9 | 0.877964 | 0.379963 | 0.5478662 | FALSE |
| ING5 | 11040 | 0.877808 | 0.380048 | 0.5478872 | FALSE |
| MMP16 | 7698 | 0.877477 | 0.380228 | 0.5480658 | FALSE |
| LOC107984974 | 13725 | 0.877321 | 0.380312 | 0.5480658 | FALSE |
| TMEM134 | 719 | 0.876679 | 0.380661 | 0.5484676 | FALSE |
| CBR1 | 10110 | 0.876415 | 0.380804 | 0.548599 | FALSE |
| CAPRIN2 | 5 | 0.876382 | 0.380822 | 0.548599 | FALSE |
| ATP6V1F | 12609 | 0.8762 | 0.380921 | 0.548691 | FALSE |
| BOK | 8214 | 0.875849 | 0.381112 | 0.5488647 | FALSE |
| PIGT | 5657 | 0.8757 | 0.381193 | 0.5489308 | FALSE |
| RDH14 | 3293 | 0.875614 | 0.38124 | 0.5489475 | FALSE |
| MAPKAPK2 | 2943 | 0.875538 | 0.381281 | 0.5489565 | FALSE |
| TRIM49B | 10850 | 0.874519 | 0.381836 | 0.5495523 | FALSE |
| LILRA1 | 6018 | 0.874381 | 0.381911 | 0.5496098 | FALSE |
| RAPGEF1 | 2330 | 0.873961 | 0.382139 | 0.5497871 | FALSE |
| HLA-DRB3 | 4152 | 0.873433 | 0.382427 | 0.5500133 | FALSE |
| EPHA1 | 1150 | 0.873414 | 0.382437 | 0.5500133 | FALSE |
| TMEM33 | 13110 | 0.873263 | 0.38252 | 0.5500305 | FALSE |
| LHX6 | 7829 | 0.873096 | 0.382611 | 0.5501107 | FALSE |
| RAB26 | 4564 | 0.872958 | 0.382686 | 0.5501683 | FALSE |
| B3GNT2 | 14853 | 0.872511 | 0.38293 | 0.5504174 | FALSE |
| ZFAND2A | 13490 | 0.872337 | 0.383025 | 0.5505032 | FALSE |
| CALR | 3604 | 0.872183 | 0.383109 | 0.5505227 | FALSE |
| CCDC9 | 7478 | 0.871691 | 0.383377 | 0.5505987 | FALSE |
| PPM1J | 5329 | 0.871672 | 0.383387 | 0.5505987 | FALSE |
| MAN1B1 | 14803 | 0.871593 | 0.38343 | 0.5505987 | FALSE |
| EPHX2 | 10437 | 0.871584 | 0.383435 | 0.5505987 | FALSE |
| FOXD1 | 10790 | 0.871505 | 0.383478 | 0.5505987 | FALSE |
| TMEM104 | 15223 | 0.871388 | 0.383542 | 0.5506399 | FALSE |
| CYB5R2 | 9170 | 0.87041 | 0.384076 | 0.5511535 | FALSE |
| LINC02014 | 14465 | 0.870134 | 0.384227 | 0.5512687 | FALSE |
| LLCFC1 | 3483 | 0.869412 | 0.384622 | 0.5517104 | FALSE |
| ZNF578 | 6723 | 0.869377 | 0.384641 | 0.5517104 | FALSE |
| COQ5 | 2441 | 0.868192 | 0.385289 | 0.5524375 | FALSE |
| ARHGEF4 | 8988 | 0.867544 | 0.385644 | 0.5527148 | FALSE |
| CDK2AP2 | 10058 | 0.867398 | 0.385724 | 0.5527567 | FALSE |
| PPP1R2 | 9524 | 0.867051 | 0.385914 | 0.5528771 | FALSE |
| RAET1K | 10251 | 0.866417 | 0.386262 | 0.5532215 | FALSE |
| HCG4B | 13975 | 0.866406 | 0.386268 | 0.5532215 | FALSE |
| NUTM2B-AS1 | 9796 | 0.866354 | 0.386296 | 0.5532215 | FALSE |
| CDHR2 | 3378 | 0.865774 | 0.386614 | 0.5535249 | FALSE |
| PSMB8 | 6726 | 0.865586 | 0.386717 | 0.5536218 | FALSE |
| SYNJ2BP | 6734 | 0.865305 | 0.386871 | 0.5537918 | FALSE |
| ZNF581 | 3095 | 0.865054 | 0.387009 | 0.5539052 | FALSE |
| RABGAP1L | 2651 | 0.865009 | 0.387034 | 0.5539052 | FALSE |
| SLITRK2 | 6718 | 0.864674 | 0.387218 | 0.554006 | FALSE |
| TMEM43 | 15616 | 0.864645 | 0.387234 | 0.554006 | FALSE |
| EPAS1 | 11886 | 0.864563 | 0.387279 | 0.5540197 | FALSE |
| CYFIP2 | 6163 | 0.864125 | 0.387519 | 0.5543131 | FALSE |
| RARS2 | 14837 | 0.863836 | 0.387678 | 0.5544132 | FALSE |
| PPP2R1B | 12825 | 0.863617 | 0.387798 | 0.5544728 | FALSE |
| SLC25A11 | 9965 | 0.863599 | 0.387808 | 0.5544728 | FALSE |
| LINC01023 | 6392 | 0.863526 | 0.387848 | 0.5544795 | FALSE |
| MYO7A | 13987 | 0.863299 | 0.387973 | 0.5545901 | FALSE |
| HNRNPUL1 | 2013 | 0.863243 | 0.388004 | 0.5545901 | FALSE |
| SMIM7 | 1338 | 0.86313 | 0.388066 | 0.5545901 | FALSE |
| GKN1 | 9680 | 0.862571 | 0.388373 | 0.5548187 | FALSE |
| F10 | 4692 | 0.862499 | 0.388413 | 0.5548187 | FALSE |
| CRIPT | 9281 | 0.862214 | 0.38857 | 0.5549451 | FALSE |
| PSMG1 | 7029 | 0.861836 | 0.388778 | 0.5551474 | FALSE |
| FAHD2B | 5765 | 0.86174 | 0.388831 | 0.5551474 | FALSE |
| DEFB132 | 3089 | 0.861618 | 0.388898 | 0.5551674 | FALSE |
| ZAN | 6017 | 0.861484 | 0.388972 | 0.5551714 | FALSE |
| MED8 | 12627 | 0.861129 | 0.389167 | 0.5553462 | FALSE |
| ZNF784 | 7310 | 0.860257 | 0.389647 | 0.5558822 | FALSE |
| USP32P1 | 13250 | 0.860091 | 0.389739 | 0.5559113 | FALSE |
| CD300LB | 14989 | 0.859846 | 0.389874 | 0.5560532 | FALSE |
| MTCH2 | 13403 | 0.859735 | 0.389935 | 0.5560898 | FALSE |
| ARHGAP25 | 6070 | 0.859623 | 0.389997 | 0.5561129 | FALSE |
| MSLN | 5727 | 0.859534 | 0.390046 | 0.5561129 | FALSE |
| TSSC2 | 14855 | 0.859275 | 0.390189 | 0.5561129 | FALSE |
| LONP2 | 10387 | 0.859254 | 0.3902 | 0.5561129 | FALSE |
| PDE6B | 376 | 0.858702 | 0.390505 | 0.5564082 | FALSE |
| ASB16 | 14804 | 0.858566 | 0.39058 | 0.5564082 | FALSE |
| FOXS1 | 13469 | 0.858556 | 0.390586 | 0.5564082 | FALSE |
| FIGNL1 | 5292 | 0.856389 | 0.391783 | 0.5577578 | FALSE |
| ATP6V1E1 | 3515 | 0.855821 | 0.392097 | 0.558086 | FALSE |
| PCMTD2 | 5712 | 0.855774 | 0.392123 | 0.558086 | FALSE |
| MTA3 | 842 | 0.855612 | 0.392212 | 0.5581155 | FALSE |
| EPHA5-AS1 | 5155 | 0.855172 | 0.392456 | 0.5584112 | FALSE |
| SMIM2-AS1 | 12980 | 0.854989 | 0.392557 | 0.5585045 | FALSE |
| SPANXA2-OT1 | 9794 | 0.854724 | 0.392704 | 0.5586624 | FALSE |
| PIGG | 8808 | 0.85421 | 0.392989 | 0.5590165 | FALSE |
| PRRC2A | 1506 | 0.854019 | 0.393094 | 0.5590653 | FALSE |
| CAMK1G | 12636 | 0.853878 | 0.393173 | 0.5591255 | FALSE |
| THBD | 6217 | 0.85373 | 0.393255 | 0.5591913 | FALSE |
| ZNF254 | 13158 | 0.852696 | 0.393828 | 0.5598538 | FALSE |
| GOLIM4 | 12899 | 0.852561 | 0.393903 | 0.5598584 | FALSE |
| AFDN-DT | 7983 | 0.851281 | 0.394613 | 0.5607662 | FALSE |
| ZBTB9 | 7117 | 0.85098 | 0.39478 | 0.5609528 | FALSE |
| PAXIP1-AS2 | 3818 | 0.850708 | 0.394932 | 0.5611165 | FALSE |
| LINC00471 | 6400 | 0.850593 | 0.394995 | 0.5611563 | FALSE |
| HIBCH | 6442 | 0.850492 | 0.395052 | 0.5611851 | FALSE |
| BCL2 | 15537 | 0.850053 | 0.395296 | 0.5614807 | FALSE |
| PGAM1 | 9676 | 0.849704 | 0.39549 | 0.5616225 | FALSE |
| CCDC102B | 1769 | 0.849649 | 0.39552 | 0.5616225 | FALSE |
| SNHG11 | 4445 | 0.849469 | 0.39562 | 0.5616868 | FALSE |
| OXT | 15510 | 0.849276 | 0.395728 | 0.5617814 | FALSE |
| PSMB9 | 2625 | 0.848883 | 0.395946 | 0.5618814 | FALSE |
| COMMD3 | 14265 | 0.848852 | 0.395964 | 0.5618814 | FALSE |
| ST14 | 13411 | 0.84868 | 0.396059 | 0.5619528 | FALSE |
| EFCAB12 | 14861 | 0.847692 | 0.39661 | 0.5624884 | FALSE |
| WDR38 | 8156 | 0.847603 | 0.396659 | 0.5624974 | FALSE |
| RAPGEF3 | 13046 | 0.846607 | 0.397214 | 0.5631825 | FALSE |
| BRCC3 | 195 | 0.846006 | 0.397549 | 0.563555 | FALSE |
| FGF13 | 451 | 0.845987 | 0.39756 | 0.563555 | FALSE |
| MROH1 | 4014 | 0.845942 | 0.397585 | 0.563555 | FALSE |
| RNF31 | 9572 | 0.845479 | 0.397843 | 0.5638701 | FALSE |
| AATF | 7078 | 0.844817 | 0.398213 | 0.5642404 | FALSE |
| RAP1GDS1 | 10824 | 0.844444 | 0.398421 | 0.5644844 | FALSE |
| ESRP2 | 14195 | 0.844329 | 0.398486 | 0.5645243 | FALSE |
| MRPL36 | 3167 | 0.842862 | 0.399306 | 0.565481 | FALSE |
| SLC30A1 | 6240 | 0.841958 | 0.399811 | 0.5661461 | FALSE |
| LOC103344931 | 11515 | 0.841774 | 0.399914 | 0.5662406 | FALSE |
| SMA5 | 3058 | 0.841573 | 0.400027 | 0.5663487 | FALSE |
| PELP1 | 15275 | 0.841146 | 0.400266 | 0.5665333 | FALSE |
| FAAP24 | 8406 | 0.840971 | 0.400364 | 0.5665695 | FALSE |
| TUBA3C | 9146 | 0.840669 | 0.400533 | 0.5666601 | FALSE |
| CLCN7 | 1809 | 0.840598 | 0.400573 | 0.5666601 | FALSE |
| SLC25A21-AS1 | 8392 | 0.840302 | 0.400739 | 0.5667465 | FALSE |
| LSM2 | 6688 | 0.840295 | 0.400743 | 0.5667465 | FALSE |
| GOLGA2P6 | 13752 | 0.839821 | 0.401009 | 0.567071 | FALSE |
| FAM214B | 741 | 0.839439 | 0.401223 | 0.56722 | FALSE |
| NRROS | 13643 | 0.839076 | 0.401427 | 0.5672832 | FALSE |
| C19orf44 | 6598 | 0.839049 | 0.401442 | 0.5672832 | FALSE |
| ZKSCAN2 | 5175 | 0.839037 | 0.401449 | 0.5672832 | FALSE |
| ADAMTSL5 | 9643 | 0.838706 | 0.401634 | 0.5674936 | FALSE |
| TCF20 | 12658 | 0.838632 | 0.401676 | 0.567501 | FALSE |
| APBB1 | 1806 | 0.837767 | 0.402162 | 0.5680846 | FALSE |
| RPA4 | 10393 | 0.837117 | 0.402527 | 0.5682924 | FALSE |
| GPHA2 | 8790 | 0.836954 | 0.402618 | 0.5683704 | FALSE |
| ZNF593 | 3916 | 0.836313 | 0.402979 | 0.5687251 | FALSE |
| MRGPRG-AS1 | 65 | 0.83527 | 0.403566 | 0.5694506 | FALSE |
| TRIQK | 4696 | 0.834657 | 0.403911 | 0.5697319 | FALSE |
| KCNB2 | 6659 | 0.834549 | 0.403972 | 0.5697663 | FALSE |
| KRTAP1-3 | 7461 | 0.834291 | 0.404117 | 0.5698684 | FALSE |
| ATP6V1D | 1776 | 0.833945 | 0.404312 | 0.5700405 | FALSE |
| SRPX | 10846 | 0.83342 | 0.404608 | 0.5704063 | FALSE |
| NSF | 5726 | 0.833307 | 0.404672 | 0.5704447 | FALSE |
| FZD3 | 5314 | 0.832951 | 0.404872 | 0.5705733 | FALSE |
| C22orf46 | 11368 | 0.832556 | 0.405095 | 0.5707695 | FALSE |
| RBM8A | 3589 | 0.83251 | 0.405121 | 0.5707695 | FALSE |
| MYMK | 3396 | 0.832426 | 0.405169 | 0.5707849 | FALSE |
| TBKBP1 | 6083 | 0.832241 | 0.405273 | 0.5708291 | FALSE |
| CTDSP1 | 6260 | 0.831412 | 0.405741 | 0.5713078 | FALSE |
| SLC6A3 | 7005 | 0.83138 | 0.405759 | 0.5713078 | FALSE |
| IMP4 | 12673 | 0.830591 | 0.406205 | 0.5717809 | FALSE |
| ZNF571 | 13662 | 0.83049 | 0.406262 | 0.5718097 | FALSE |
| VPS28 | 2525 | 0.829902 | 0.406594 | 0.572162 | FALSE |
| SMARCA5 | 2731 | 0.829403 | 0.406876 | 0.5724171 | FALSE |
| MEI1 | 15606 | 0.829325 | 0.406921 | 0.5724199 | FALSE |
| KRTAP10-11 | 2804 | 0.82927 | 0.406952 | 0.5724199 | FALSE |
| KCNJ16 | 2599 | 0.82877 | 0.407235 | 0.5727148 | FALSE |
| RPS4Y2 | 7227 | 0.828397 | 0.407446 | 0.5728834 | FALSE |
| ANKRD10 | 1115 | 0.828392 | 0.407449 | 0.5728834 | FALSE |
| HNRNPCL1 | 4067 | 0.828364 | 0.407464 | 0.5728834 | FALSE |
| ECHDC1 | 997 | 0.828152 | 0.407584 | 0.5729491 | FALSE |
| PDHA1 | 6676 | 0.827941 | 0.407704 | 0.5730656 | FALSE |
| KCNK5 | 8480 | 0.827733 | 0.407822 | 0.5731188 | FALSE |
| EEF1AKMT4 | 1764 | 0.827729 | 0.407824 | 0.5731188 | FALSE |
| DDX41 | 9473 | 0.827318 | 0.408057 | 0.5733554 | FALSE |
| TXNIP | 3557 | 0.827129 | 0.408164 | 0.5734544 | FALSE |
| GALNT10 | 7845 | 0.826965 | 0.408257 | 0.5735334 | FALSE |
| LINC01686 | 8434 | 0.82679 | 0.408356 | 0.5736213 | FALSE |
| SCARF2 | 3399 | 0.82582 | 0.408906 | 0.5742392 | FALSE |
| ZDHHC16 | 12597 | 0.825516 | 0.409079 | 0.5743673 | FALSE |
| FAIM2 | 5478 | 0.825328 | 0.409185 | 0.5744249 | FALSE |
| ATAD3A | 1449 | 0.825212 | 0.409251 | 0.5744657 | FALSE |
| RWDD3 | 9287 | 0.825133 | 0.409296 | 0.5744771 | FALSE |
| GPX1 | 1763 | 0.82497 | 0.409389 | 0.5745554 | FALSE |
| SEC61A1 | 9497 | 0.823464 | 0.410244 | 0.5752795 | FALSE |
| S1PR2 | 6588 | 0.822885 | 0.410573 | 0.5755692 | FALSE |
| AREL1 | 8502 | 0.822793 | 0.410626 | 0.5755692 | FALSE |
| PCLO | 7133 | 0.821821 | 0.411179 | 0.5761298 | FALSE |
| BCL6 | 4665 | 0.821765 | 0.411211 | 0.5761298 | FALSE |
| SLC24A1 | 12387 | 0.821185 | 0.411541 | 0.5764646 | FALSE |
| UBXN1 | 9510 | 0.821151 | 0.41156 | 0.5764646 | FALSE |
| LINC00608 | 12503 | 0.820965 | 0.411666 | 0.5765159 | FALSE |
| SOX2-OT | 197 | 0.820861 | 0.411725 | 0.5765159 | FALSE |
| AAMP | 5146 | 0.82086 | 0.411726 | 0.5765159 | FALSE |
| PSMA3-AS1 | 7547 | 0.820811 | 0.411754 | 0.5765159 | FALSE |
| THAP11 | 5140 | 0.820763 | 0.411781 | 0.5765159 | FALSE |
| MRPL49 | 10231 | 0.819953 | 0.412243 | 0.5770018 | FALSE |
| ERBB3 | 6736 | 0.819563 | 0.412465 | 0.5771437 | FALSE |
| PPP1R13L | 3102 | 0.818388 | 0.413136 | 0.5779092 | FALSE |
| CAPN2 | 2673 | 0.818354 | 0.413155 | 0.5779092 | FALSE |
| ATF7 | 7958 | 0.818305 | 0.413183 | 0.5779092 | FALSE |
| AMER2 | 1127 | 0.818036 | 0.413337 | 0.57807 | FALSE |
| SLC25A29 | 9261 | 0.817166 | 0.413834 | 0.5786003 | FALSE |
| HSPE1 | 8188 | 0.816587 | 0.414164 | 0.5788158 | FALSE |
| CISD1 | 13117 | 0.81652 | 0.414203 | 0.5788176 | FALSE |
| EXT2 | 5457 | 0.816103 | 0.414441 | 0.5789518 | FALSE |
| CLIC3 | 1938 | 0.815761 | 0.414637 | 0.5791045 | FALSE |
| FLOT1 | 4094 | 0.815688 | 0.414679 | 0.5791045 | FALSE |
| KCND1 | 4013 | 0.815578 | 0.414742 | 0.5791045 | FALSE |
| B4GALNT4 | 4081 | 0.815466 | 0.414806 | 0.5791321 | FALSE |
| NOTCH2NL | 12083 | 0.815414 | 0.414835 | 0.5791321 | FALSE |
| MTX2 | 7359 | 0.815214 | 0.41495 | 0.5792402 | FALSE |
| LOC100506544 | 12247 | 0.815038 | 0.415051 | 0.5793291 | FALSE |
| LRFN1 | 5878 | 0.814682 | 0.415254 | 0.57951 | FALSE |
| THAP4 | 11935 | 0.814394 | 0.415419 | 0.5796367 | FALSE |
| RAB3D | 5099 | 0.814273 | 0.415489 | 0.5796816 | FALSE |
| WDR93 | 15007 | 0.814014 | 0.415637 | 0.5797718 | FALSE |
| PRORSD1P | 10203 | 0.813987 | 0.415652 | 0.5797718 | FALSE |
| B2M | 10277 | 0.813966 | 0.415664 | 0.5797718 | FALSE |
| MFSD5 | 13842 | 0.813543 | 0.415907 | 0.5800063 | FALSE |
| EMD | 2735 | 0.813338 | 0.416024 | 0.5800819 | FALSE |
| TMEM62 | 10936 | 0.813319 | 0.416035 | 0.5800819 | FALSE |
| ETFA | 14823 | 0.81302 | 0.416207 | 0.5801531 | FALSE |
| SPATA25 | 3446 | 0.811533 | 0.41706 | 0.5810665 | FALSE |
| SEC22C | 5053 | 0.811483 | 0.417088 | 0.5810665 | FALSE |
| GID4 | 3871 | 0.811451 | 0.417107 | 0.5810665 | FALSE |
| NOS1AP | 10899 | 0.811114 | 0.4173 | 0.5811783 | FALSE |
| ZNF43 | 11127 | 0.810745 | 0.417512 | 0.5813634 | FALSE |
| PPP6C | 5142 | 0.81031 | 0.417762 | 0.5816596 | FALSE |
| KLHL26 | 1437 | 0.810219 | 0.417814 | 0.5816806 | FALSE |
| PRSS55 | 6057 | 0.810146 | 0.417856 | 0.5816872 | FALSE |
| PGS1 | 879 | 0.809629 | 0.418153 | 0.5819972 | FALSE |
| GANAB | 11323 | 0.809552 | 0.418198 | 0.582007 | FALSE |
| RBPMS2 | 7430 | 0.809322 | 0.41833 | 0.5821392 | FALSE |
| LOC202181 | 4681 | 0.809007 | 0.418511 | 0.5822877 | FALSE |
| COLEC12 | 4022 | 0.808651 | 0.418716 | 0.5825208 | FALSE |
| PIEZO2 | 11627 | 0.808399 | 0.418861 | 0.5826188 | FALSE |
| HIST1H4C | 3079 | 0.80831 | 0.418912 | 0.5826382 | FALSE |
| PLCXD3 | 143 | 0.807312 | 0.419487 | 0.5833336 | FALSE |
| PNRC1 | 11571 | 0.807218 | 0.419541 | 0.583357 | FALSE |
| PLA2G12A | 6776 | 0.806595 | 0.4199 | 0.5837523 | FALSE |
| ACOT7 | 10471 | 0.806453 | 0.419982 | 0.5837622 | FALSE |
| ZNF675 | 2206 | 0.806009 | 0.420238 | 0.5840142 | FALSE |
| HMGA1 | 9317 | 0.805509 | 0.420526 | 0.584311 | FALSE |
| NOS1 | 14979 | 0.805156 | 0.42073 | 0.5844901 | FALSE |
| AGRN | 8141 | 0.805073 | 0.420778 | 0.5845047 | FALSE |
| F2R | 629 | 0.804535 | 0.421088 | 0.584884 | FALSE |
| TBXA2R | 11213 | 0.804416 | 0.421157 | 0.58491 | FALSE |
| PPP4C | 1137 | 0.804373 | 0.421182 | 0.58491 | FALSE |
| CCL25 | 15388 | 0.803585 | 0.421637 | 0.585386 | FALSE |
| MCPH1-AS1 | 11893 | 0.803443 | 0.421719 | 0.5854479 | FALSE |
| FARSA | 4578 | 0.803318 | 0.421791 | 0.5854962 | FALSE |
| SDHAF1 | 5867 | 0.802849 | 0.422062 | 0.5857389 | FALSE |
| DEDD | 8108 | 0.802821 | 0.422078 | 0.5857389 | FALSE |
| RETSAT | 294 | 0.802561 | 0.422229 | 0.5858844 | FALSE |
| ACKR2 | 10460 | 0.80251 | 0.422258 | 0.5858844 | FALSE |
| CCT8L2 | 13880 | 0.802391 | 0.422327 | 0.5858847 | FALSE |
| PMEL | 10142 | 0.80238 | 0.422333 | 0.5858847 | FALSE |
| GOLGA5 | 12787 | 0.802145 | 0.422469 | 0.5859173 | FALSE |
| CTBP1-AS2 | 631 | 0.801222 | 0.423003 | 0.5864498 | FALSE |
| NAGS | 4257 | 0.800855 | 0.423216 | 0.5865883 | FALSE |
| ZNF589 | 12349 | 0.800752 | 0.423275 | 0.5866189 | FALSE |
| STK16 | 1682 | 0.800303 | 0.423535 | 0.5869273 | FALSE |
| C16orf92 | 8416 | 0.800237 | 0.423573 | 0.5869282 | FALSE |
| ORC6 | 5686 | 0.799207 | 0.42417 | 0.5874429 | FALSE |
| PAFAH1B1 | 4963 | 0.798902 | 0.424347 | 0.5875837 | FALSE |
| B9D2 | 1718 | 0.798768 | 0.424425 | 0.5876393 | FALSE |
| KIF26B | 13695 | 0.798474 | 0.424595 | 0.5878233 | FALSE |
| C11orf21 | 10238 | 0.797272 | 0.425293 | 0.5884068 | FALSE |
| RARRES2 | 8878 | 0.795768 | 0.426167 | 0.5892162 | FALSE |
| MRPL2 | 2723 | 0.795261 | 0.426462 | 0.5895195 | FALSE |
| SEMA4D | 11655 | 0.79504 | 0.42659 | 0.589645 | FALSE |
| TTF1 | 7554 | 0.794098 | 0.427138 | 0.5902461 | FALSE |
| DCST2 | 2875 | 0.793498 | 0.427488 | 0.5905723 | FALSE |
| CAPZA1 | 3669 | 0.793188 | 0.427668 | 0.5906651 | FALSE |
| PRORY | 10746 | 0.793063 | 0.427741 | 0.5907135 | FALSE |
| TAGLN3 | 7324 | 0.792963 | 0.427799 | 0.5907418 | FALSE |
| USP46-AS1 | 8083 | 0.792311 | 0.428179 | 0.5912143 | FALSE |
| RAMP2 | 9853 | 0.792172 | 0.42826 | 0.5912739 | FALSE |
| PHC1 | 14958 | 0.791922 | 0.428406 | 0.5913707 | FALSE |
| ADRA1A | 5376 | 0.791636 | 0.428573 | 0.5915078 | FALSE |
| SCGB1A1 | 11473 | 0.791622 | 0.428581 | 0.5915078 | FALSE |
| SCARA5 | 2062 | 0.791484 | 0.428662 | 0.5915297 | FALSE |
| RARRES1 | 717 | 0.791465 | 0.428673 | 0.5915297 | FALSE |
| CNTNAP2 | 1108 | 0.791227 | 0.428812 | 0.5915657 | FALSE |
| KRTAP23-1 | 2294 | 0.791116 | 0.428876 | 0.5915657 | FALSE |
| XPO6 | 7345 | 0.791003 | 0.428942 | 0.5915884 | FALSE |
| LOC731157 | 15303 | 0.789798 | 0.429646 | 0.5924018 | FALSE |
| IARS | 10625 | 0.788877 | 0.430184 | 0.5928301 | FALSE |
| RAB11FIP1 | 6895 | 0.788695 | 0.43029 | 0.5929245 | FALSE |
| YWHAZ | 14164 | 0.788456 | 0.43043 | 0.5930125 | FALSE |
| GALNT15 | 14203 | 0.787947 | 0.430728 | 0.593318 | FALSE |
| CCSAP | 5896 | 0.787627 | 0.430915 | 0.5935236 | FALSE |
| CLN8 | 13228 | 0.787313 | 0.431099 | 0.5937244 | FALSE |
| EIF5A | 2946 | 0.787089 | 0.43123 | 0.5938526 | FALSE |
| CRYBG3 | 904 | 0.786957 | 0.431307 | 0.5938899 | FALSE |
| ERBIN | 10932 | 0.786441 | 0.431609 | 0.5940233 | FALSE |
| GAPDHS | 12522 | 0.78644 | 0.43161 | 0.5940233 | FALSE |
| PPP1R14B | 11430 | 0.786423 | 0.43162 | 0.5940233 | FALSE |
| ADA | 10409 | 0.786068 | 0.431828 | 0.5942049 | FALSE |
| SEMA4G | 1712 | 0.785757 | 0.43201 | 0.5944033 | FALSE |
| LPIN3 | 13224 | 0.785589 | 0.432108 | 0.5944341 | FALSE |
| TELO2 | 11142 | 0.785491 | 0.432166 | 0.5944608 | FALSE |
| EIF3B | 4167 | 0.785174 | 0.432352 | 0.5946641 | FALSE |
| CR2 | 3926 | 0.785097 | 0.432397 | 0.5946738 | FALSE |
| GOLGA6C | 15137 | 0.785032 | 0.432435 | 0.5946739 | FALSE |
| RTCA | 7191 | 0.7849 | 0.432512 | 0.5947072 | FALSE |
| THAP9-AS1 | 11183 | 0.78444 | 0.432782 | 0.5949943 | FALSE |
| STXBP5 | 11423 | 0.783736 | 0.433195 | 0.5955098 | FALSE |
| FOXB1 | 4813 | 0.78358 | 0.433287 | 0.5955833 | FALSE |
| KIAA1755 | 1388 | 0.782687 | 0.433811 | 0.596042 | FALSE |
| LINC01590 | 2779 | 0.781776 | 0.434346 | 0.5966202 | FALSE |
| MARK1 | 1040 | 0.78109 | 0.43475 | 0.5970169 | FALSE |
| ZNF580 | 6821 | 0.780592 | 0.435042 | 0.5973667 | FALSE |
| ELP4 | 9246 | 0.780314 | 0.435206 | 0.5975388 | FALSE |
| ZNF431 | 4307 | 0.780054 | 0.435359 | 0.5976964 | FALSE |
| PDE6H | 9708 | 0.779599 | 0.435627 | 0.5980116 | FALSE |
| SATL1 | 10580 | 0.778642 | 0.436191 | 0.5986827 | FALSE |
| ZFC3H1 | 13021 | 0.778639 | 0.436192 | 0.5986827 | FALSE |
| NDST3 | 1619 | 0.778243 | 0.436426 | 0.5988978 | FALSE |
| DDX17 | 15304 | 0.77787 | 0.436646 | 0.5990944 | FALSE |
| ALPP | 8880 | 0.777705 | 0.436743 | 0.5991753 | FALSE |
| ELMO2 | 6209 | 0.776516 | 0.437444 | 0.5998744 | FALSE |
| ZNF704 | 5833 | 0.775524 | 0.43803 | 0.6005195 | FALSE |
| TMIGD2 | 7573 | 0.775354 | 0.438131 | 0.6005519 | FALSE |
| PRPS1L1 | 4686 | 0.775265 | 0.438183 | 0.6005713 | FALSE |
| DHX34 | 10221 | 0.77513 | 0.438263 | 0.6005753 | FALSE |
| OTUD6A | 6007 | 0.775034 | 0.43832 | 0.6006004 | FALSE |
| OSER1 | 5281 | 0.774501 | 0.438635 | 0.600874 | FALSE |
| SOX6 | 5581 | 0.773923 | 0.438976 | 0.6012619 | FALSE |
| CYB561D2 | 4626 | 0.773885 | 0.438999 | 0.6012619 | FALSE |
| CCDC110 | 11282 | 0.773762 | 0.439072 | 0.6012619 | FALSE |
| FAM24B | 9677 | 0.773616 | 0.439158 | 0.6013275 | FALSE |
| NPAS3 | 2399 | 0.773484 | 0.439236 | 0.601329 | FALSE |
| SCFD1 | 8497 | 0.773079 | 0.439476 | 0.6016044 | FALSE |
| ZNF720 | 5408 | 0.771907 | 0.44017 | 0.6024488 | FALSE |
| HAND2 | 1501 | 0.771517 | 0.440401 | 0.6026675 | FALSE |
| ANKRD36C | 4551 | 0.77146 | 0.440434 | 0.6026675 | FALSE |
| HIST1H2AH | 9538 | 0.771008 | 0.440702 | 0.6029407 | FALSE |
| MTURN | 15087 | 0.770975 | 0.440722 | 0.6029407 | FALSE |
| HMX2 | 12467 | 0.770555 | 0.440971 | 0.6032285 | FALSE |
| PRDM10 | 233 | 0.770403 | 0.441061 | 0.603299 | FALSE |
| MEX3D | 12640 | 0.77031 | 0.441116 | 0.6033217 | FALSE |
| RNF215 | 10148 | 0.769748 | 0.441449 | 0.6037248 | FALSE |
| NANS | 4202 | 0.769309 | 0.44171 | 0.6040282 | FALSE |
| PPP2R1A | 15204 | 0.769053 | 0.441862 | 0.6041832 | FALSE |
| SMIM1 | 12401 | 0.768497 | 0.442192 | 0.6045817 | FALSE |
| RMND5B | 3673 | 0.768388 | 0.442257 | 0.6046015 | FALSE |
| F11R | 2982 | 0.768299 | 0.44231 | 0.6046015 | FALSE |
| FRMD6 | 4374 | 0.768105 | 0.442425 | 0.6046015 | FALSE |
| URAD | 4989 | 0.767945 | 0.44252 | 0.6046598 | FALSE |
| POLR2E | 9679 | 0.767557 | 0.44275 | 0.6048691 | FALSE |
| MAPK12 | 12004 | 0.767345 | 0.442876 | 0.6049184 | FALSE |
| GDPD3 | 8492 | 0.767025 | 0.443067 | 0.6050896 | FALSE |
| PPM1G | 12386 | 0.766577 | 0.443333 | 0.6054006 | FALSE |
| C4BPB | 13755 | 0.766436 | 0.443417 | 0.6054093 | FALSE |
| YES1 | 11210 | 0.766019 | 0.443665 | 0.6056422 | FALSE |
| MCAT | 8600 | 0.765476 | 0.443988 | 0.6060305 | FALSE |
| RNF126 | 12758 | 0.765138 | 0.444189 | 0.6062047 | FALSE |
| ZNF789 | 7145 | 0.765069 | 0.44423 | 0.6062047 | FALSE |
| HDAC11 | 1211 | 0.765001 | 0.444271 | 0.6062047 | FALSE |
| ERICH2 | 548 | 0.764575 | 0.444525 | 0.6064451 | FALSE |
| SGMS1 | 14001 | 0.764149 | 0.444778 | 0.6064562 | FALSE |
| DCDC2B | 2295 | 0.764019 | 0.444856 | 0.6064562 | FALSE |
| NIPAL1 | 15380 | 0.763874 | 0.444942 | 0.6064562 | FALSE |
| SLC2A13 | 13168 | 0.763864 | 0.444948 | 0.6064562 | FALSE |
| LCA10 | 10379 | 0.763851 | 0.444956 | 0.6064562 | FALSE |
| C19orf18 | 3940 | 0.763716 | 0.445037 | 0.6064562 | FALSE |
| TWF1 | 13531 | 0.763715 | 0.445037 | 0.6064562 | FALSE |
| NARF | 165 | 0.76328 | 0.445296 | 0.6067567 | FALSE |
| LMF2 | 332 | 0.762804 | 0.44558 | 0.6069847 | FALSE |
| GRPR | 12711 | 0.762706 | 0.445639 | 0.6070115 | FALSE |
| LPCAT3 | 2951 | 0.762026 | 0.446044 | 0.6074273 | FALSE |
| TBL3 | 14406 | 0.761624 | 0.446284 | 0.6075846 | FALSE |
| SYNGR4 | 1496 | 0.761571 | 0.446316 | 0.6075846 | FALSE |
| PCDHB3 | 1562 | 0.761545 | 0.446332 | 0.6075846 | FALSE |
| TP53TG5 | 10842 | 0.760933 | 0.446697 | 0.6079234 | FALSE |
| ZNF674-AS1 | 6713 | 0.760819 | 0.446765 | 0.6079631 | FALSE |
| CCL21 | 9972 | 0.76026 | 0.447099 | 0.6082815 | FALSE |
| TIGD1 | 4323 | 0.759887 | 0.447322 | 0.6084219 | FALSE |
| GMPPB | 1158 | 0.759846 | 0.447347 | 0.6084219 | FALSE |
| ZNF471 | 1171 | 0.759511 | 0.447547 | 0.6086031 | FALSE |
| IMMP2L | 8346 | 0.759382 | 0.447624 | 0.6086509 | FALSE |
| TSPAN11 | 7044 | 0.759236 | 0.447711 | 0.6086679 | FALSE |
| CAPN13 | 14100 | 0.758918 | 0.447902 | 0.6087839 | FALSE |
| PARP6 | 5610 | 0.758898 | 0.447914 | 0.6087839 | FALSE |
| ZNF721 | 5735 | 0.758803 | 0.44797 | 0.6088083 | FALSE |
| LAMTOR5 | 9167 | 0.75808 | 0.448403 | 0.6091315 | FALSE |
| LINC02076 | 15064 | 0.757617 | 0.44868 | 0.6094022 | FALSE |
| PROSER2 | 14658 | 0.755834 | 0.449749 | 0.6105351 | FALSE |
| EPHB3 | 12055 | 0.755196 | 0.450131 | 0.6110015 | FALSE |
| RESP18 | 8756 | 0.754762 | 0.450392 | 0.6111957 | FALSE |
| TUSC2 | 9016 | 0.753887 | 0.450917 | 0.6116431 | FALSE |
| PRRX2 | 11727 | 0.753717 | 0.451019 | 0.6117285 | FALSE |
| CHRNA7 | 2797 | 0.753367 | 0.451229 | 0.6119606 | FALSE |
| TMED10P1 | 8077 | 0.75323 | 0.451312 | 0.6120191 | FALSE |
| C9orf62 | 10514 | 0.752582 | 0.451701 | 0.6123101 | FALSE |
| EMC2 | 3496 | 0.751236 | 0.452511 | 0.6132194 | FALSE |
| RALBP1 | 490 | 0.750623 | 0.45288 | 0.613613 | FALSE |
| HHLA3 | 728 | 0.750513 | 0.452946 | 0.6136495 | FALSE |
| IGFALS | 5961 | 0.750339 | 0.453051 | 0.6137382 | FALSE |
| ITGA1 | 13549 | 0.749761 | 0.453399 | 0.6140502 | FALSE |
| H1FX | 13421 | 0.749588 | 0.453503 | 0.6141381 | FALSE |
| HIST1H4K | 10235 | 0.749084 | 0.453807 | 0.6144962 | FALSE |
| CPNE8 | 3450 | 0.74888 | 0.45393 | 0.6145583 | FALSE |
| COPS8 | 9563 | 0.748747 | 0.45401 | 0.6145583 | FALSE |
| ECHDC2 | 3267 | 0.748484 | 0.454168 | 0.6146066 | FALSE |
| CLPTM1 | 15394 | 0.747971 | 0.454478 | 0.6148723 | FALSE |
| OR10Q1 | 9436 | 0.747253 | 0.454911 | 0.6154051 | FALSE |
| ELN | 9414 | 0.747076 | 0.455018 | 0.6154964 | FALSE |
| TRIM45 | 10940 | 0.74688 | 0.455136 | 0.6155979 | FALSE |
| WASHC5 | 5326 | 0.746756 | 0.455211 | 0.6155979 | FALSE |
| DLX2 | 15392 | 0.746291 | 0.455492 | 0.615871 | FALSE |
| MED29 | 11280 | 0.746127 | 0.455591 | 0.6159517 | FALSE |
| COX14 | 5168 | 0.746017 | 0.455657 | 0.6159883 | FALSE |
| EVPLL | 12691 | 0.744889 | 0.456339 | 0.6166865 | FALSE |
| PDLIM4 | 6948 | 0.744836 | 0.456371 | 0.6166865 | FALSE |
| TAGLN | 6724 | 0.744523 | 0.45656 | 0.6168158 | FALSE |
| NEK9 | 14199 | 0.744016 | 0.456867 | 0.6170366 | FALSE |
| ZNF676 | 13249 | 0.743802 | 0.456996 | 0.6171581 | FALSE |
| C5orf15 | 11187 | 0.743013 | 0.457474 | 0.6176191 | FALSE |
| BANF1 | 9968 | 0.742977 | 0.457496 | 0.6176191 | FALSE |
| BTF3L4 | 9484 | 0.742874 | 0.457558 | 0.6176499 | FALSE |
| BMP2K | 11715 | 0.742705 | 0.45766 | 0.6177347 | FALSE |
| CADPS | 9798 | 0.742569 | 0.457743 | 0.6177925 | FALSE |
| KRTAP10-12 | 9393 | 0.742347 | 0.457877 | 0.6179207 | FALSE |
| SAP130 | 13063 | 0.741947 | 0.458119 | 0.618141 | FALSE |
| KRT71 | 2502 | 0.74127 | 0.45853 | 0.6185353 | FALSE |
| MAPRE1 | 7224 | 0.741269 | 0.45853 | 0.6185353 | FALSE |
| LOC440040 | 10464 | 0.740831 | 0.458796 | 0.6187333 | FALSE |
| PNMA8B | 13057 | 0.740232 | 0.459159 | 0.6191165 | FALSE |
| CEACAM21 | 5630 | 0.739447 | 0.459636 | 0.6197053 | FALSE |
| OR2AG2 | 4984 | 0.738953 | 0.459936 | 0.6200298 | FALSE |
| ABCF1 | 4796 | 0.73892 | 0.459956 | 0.6200298 | FALSE |
| AUP1 | 13776 | 0.738825 | 0.460013 | 0.6200541 | FALSE |
| AP2A2 | 1402 | 0.738431 | 0.460253 | 0.6203232 | FALSE |
| RND3 | 14181 | 0.738195 | 0.460396 | 0.6203601 | FALSE |
| AFAP1 | 2006 | 0.737665 | 0.460718 | 0.6206341 | FALSE |
| IRF7 | 1431 | 0.737204 | 0.460998 | 0.6207707 | FALSE |
| OLIG2 | 11511 | 0.7372 | 0.461001 | 0.6207707 | FALSE |
| ZMAT5 | 12815 | 0.737166 | 0.461021 | 0.6207707 | FALSE |
| RRAS | 12438 | 0.737091 | 0.461067 | 0.6207786 | FALSE |
| FNBP1 | 4104 | 0.736459 | 0.461451 | 0.6211357 | FALSE |
| GPR26 | 11521 | 0.736076 | 0.461684 | 0.6213042 | FALSE |
| PLXND1 | 2089 | 0.73601 | 0.461725 | 0.6213042 | FALSE |
| OR1J4 | 6098 | 0.735992 | 0.461736 | 0.6213042 | FALSE |
| LINC01976 | 10392 | 0.735657 | 0.461939 | 0.6214716 | FALSE |
| LOC100505915 | 7876 | 0.73516 | 0.462242 | 0.621779 | FALSE |
| PDZD2 | 7817 | 0.735151 | 0.462248 | 0.621779 | FALSE |
| MNT | 2925 | 0.734743 | 0.462496 | 0.6219172 | FALSE |
| EPHA6 | 239 | 0.734721 | 0.462509 | 0.6219172 | FALSE |
| LGMN | 427 | 0.734496 | 0.462646 | 0.6220481 | FALSE |
| AOC2 | 267 | 0.734063 | 0.46291 | 0.6223493 | FALSE |
| PPP1R3C | 7677 | 0.73369 | 0.463138 | 0.6225479 | FALSE |
| CDKL3 | 11274 | 0.733067 | 0.463518 | 0.6229224 | FALSE |
| TPD52 | 10036 | 0.732564 | 0.463824 | 0.6231494 | FALSE |
| FANK1 | 6353 | 0.732223 | 0.464032 | 0.6233218 | FALSE |
| FHL3 | 12537 | 0.732078 | 0.464121 | 0.6233871 | FALSE |
| CFAP206 | 6623 | 0.731164 | 0.464679 | 0.6239222 | FALSE |
| ANKRD28 | 12449 | 0.730531 | 0.465066 | 0.624227 | FALSE |
| NOTCH1 | 6179 | 0.730236 | 0.465246 | 0.6243617 | FALSE |
| MIP | 11852 | 0.728536 | 0.466286 | 0.6255421 | FALSE |
| CHRM5 | 10007 | 0.728077 | 0.466566 | 0.6257578 | FALSE |
| DNM1P50 | 608 | 0.727976 | 0.466628 | 0.6257871 | FALSE |
| ANO2 | 3316 | 0.726942 | 0.467261 | 0.626475 | FALSE |
| IGSF21 | 5947 | 0.726642 | 0.467445 | 0.6266227 | FALSE |
| CPEB2 | 1181 | 0.726435 | 0.467572 | 0.6266228 | FALSE |
| AIRE | 4953 | 0.726339 | 0.467631 | 0.626648 | FALSE |
| GALNT7 | 436 | 0.725563 | 0.468107 | 0.6271779 | FALSE |
| AKR1C4 | 5955 | 0.725337 | 0.468245 | 0.6273099 | FALSE |
| EXOC3L1 | 12595 | 0.725042 | 0.468426 | 0.6274448 | FALSE |
| MRPS24 | 1766 | 0.724962 | 0.468475 | 0.6274567 | FALSE |
| LOC399815 | 12321 | 0.724851 | 0.468543 | 0.6274749 | FALSE |
| RBP2 | 7578 | 0.724809 | 0.468569 | 0.6274749 | FALSE |
| PDGFA | 2811 | 0.724242 | 0.468917 | 0.6278334 | FALSE |
| KIF3C | 8355 | 0.723992 | 0.469071 | 0.6279485 | FALSE |
| ITFG1 | 3292 | 0.723971 | 0.469084 | 0.6279485 | FALSE |
| NIT1 | 4348 | 0.723363 | 0.469457 | 0.6282332 | FALSE |
| RPS19BP1 | 10829 | 0.723238 | 0.469534 | 0.6282821 | FALSE |
| PNMA1 | 11920 | 0.722678 | 0.469878 | 0.6285155 | FALSE |
| SNX17 | 5958 | 0.722627 | 0.469909 | 0.6285155 | FALSE |
| TFE3 | 3896 | 0.722393 | 0.470053 | 0.6286002 | FALSE |
| LINC01146 | 1017 | 0.722325 | 0.470095 | 0.6286024 | FALSE |
| ELF1 | 12082 | 0.721432 | 0.470644 | 0.6291751 | FALSE |
| PCGF2 | 13150 | 0.720991 | 0.470915 | 0.629484 | FALSE |
| ZNF75D | 3544 | 0.720763 | 0.471055 | 0.6296177 | FALSE |
| LRRC75A-AS1 | 10945 | 0.719253 | 0.471985 | 0.6305369 | FALSE |
| BAP1 | 3121 | 0.719043 | 0.472114 | 0.6306558 | FALSE |
| LINC01233 | 1564 | 0.71858 | 0.4724 | 0.630983 | FALSE |
| RAB7A | 15457 | 0.718265 | 0.472594 | 0.6311884 | FALSE |
| CHAMP1 | 6012 | 0.717513 | 0.473058 | 0.6315676 | FALSE |
| EBI3 | 13212 | 0.717455 | 0.473093 | 0.6315676 | FALSE |
| COL4A5 | 12287 | 0.717406 | 0.473124 | 0.6315676 | FALSE |
| AQP7P1 | 14548 | 0.71736 | 0.473152 | 0.6315676 | FALSE |
| HCG18 | 158 | 0.717187 | 0.473259 | 0.6316446 | FALSE |
| DHRS7B | 4925 | 0.716174 | 0.473884 | 0.6320474 | FALSE |
| SYN1 | 2655 | 0.715884 | 0.474063 | 0.6322323 | FALSE |
| HAP1 | 13809 | 0.715781 | 0.474127 | 0.6322632 | FALSE |
| PLIN2 | 7268 | 0.715496 | 0.474303 | 0.6324139 | FALSE |
| CEP131 | 10084 | 0.715467 | 0.474321 | 0.6324139 | FALSE |
| NDNF | 15383 | 0.715373 | 0.474379 | 0.6324374 | FALSE |
| SLC18B1 | 15475 | 0.714852 | 0.474701 | 0.6328126 | FALSE |
| DPP3 | 11763 | 0.713715 | 0.475403 | 0.6335335 | FALSE |
| CSNK1A1P1 | 12888 | 0.713158 | 0.475748 | 0.6339387 | FALSE |
| SPI1 | 15454 | 0.712766 | 0.475991 | 0.6341818 | FALSE |
| NYAP1 | 8336 | 0.712732 | 0.476012 | 0.6341818 | FALSE |
| DCTN1 | 11392 | 0.712442 | 0.476191 | 0.6342546 | FALSE |
| NLRC5 | 14819 | 0.712363 | 0.47624 | 0.6342546 | FALSE |
| CDC26 | 9222 | 0.712317 | 0.476268 | 0.6342546 | FALSE |
| PSORS1C2 | 6297 | 0.712316 | 0.476269 | 0.6342546 | FALSE |
| GSN-AS1 | 15495 | 0.711824 | 0.476574 | 0.6346063 | FALSE |
| DNAJC14 | 14493 | 0.711665 | 0.476672 | 0.6346293 | FALSE |
| SPAG9 | 15341 | 0.710618 | 0.477321 | 0.635284 | FALSE |
| VWA3A | 1850 | 0.710609 | 0.477327 | 0.635284 | FALSE |
| MRPL11 | 5000 | 0.710141 | 0.477617 | 0.6353997 | FALSE |
| TMEM126B | 2027 | 0.709348 | 0.478109 | 0.6358917 | FALSE |
| RAB5B | 14572 | 0.708668 | 0.478531 | 0.6363988 | FALSE |
| ALDH16A1 | 3647 | 0.708454 | 0.478663 | 0.6365115 | FALSE |
| CLCN1 | 12733 | 0.708303 | 0.478757 | 0.6365115 | FALSE |
| KRTAP3-2 | 8246 | 0.707502 | 0.479255 | 0.6367728 | FALSE |
| ARRDC2 | 101 | 0.707443 | 0.479291 | 0.6367728 | FALSE |
| TAL1 | 1246 | 0.707065 | 0.479526 | 0.6370183 | FALSE |
| ZCWPW1 | 12156 | 0.706817 | 0.47968 | 0.6371689 | FALSE |
| FSTL3 | 8226 | 0.705772 | 0.48033 | 0.6378152 | FALSE |
| CYP11B1 | 6099 | 0.705543 | 0.480472 | 0.6378959 | FALSE |
| POLH | 1476 | 0.704775 | 0.48095 | 0.6383136 | FALSE |
| RGS11 | 7913 | 0.704598 | 0.48106 | 0.6383638 | FALSE |
| FAM180A | 13476 | 0.704583 | 0.48107 | 0.6383638 | FALSE |
| GGT6 | 14385 | 0.70449 | 0.481128 | 0.6383864 | FALSE |
| TKTL1 | 6417 | 0.703316 | 0.481859 | 0.6391895 | FALSE |
| CALHM1 | 596 | 0.70319 | 0.481937 | 0.6391895 | FALSE |
| NPLOC4 | 509 | 0.702967 | 0.482076 | 0.6393196 | FALSE |
| GREM1 | 13895 | 0.702568 | 0.482325 | 0.6395395 | FALSE |
| OR10H5 | 7841 | 0.702504 | 0.482365 | 0.6395395 | FALSE |
| LPL | 5267 | 0.702197 | 0.482556 | 0.6396848 | FALSE |
| ZNF274 | 15305 | 0.701953 | 0.482708 | 0.6398111 | FALSE |
| GAL | 5286 | 0.701913 | 0.482733 | 0.6398111 | FALSE |
| SFT2D1 | 1667 | 0.701795 | 0.482807 | 0.6398544 | FALSE |
| DEPDC4 | 6515 | 0.701037 | 0.48328 | 0.6404268 | FALSE |
| ATG12 | 1065 | 0.700008 | 0.483922 | 0.6411804 | FALSE |
| GLIS3-AS1 | 11986 | 0.699826 | 0.484036 | 0.6411804 | FALSE |
| TCEAL7 | 15551 | 0.699758 | 0.484078 | 0.6411804 | FALSE |
| TAPBPL | 3794 | 0.699745 | 0.484087 | 0.6411804 | FALSE |
| SNORA70 | 5741 | 0.699732 | 0.484095 | 0.6411804 | FALSE |
| DLK2 | 1858 | 0.698529 | 0.484846 | 0.6420129 | FALSE |
| IKBKG | 711 | 0.69843 | 0.484908 | 0.6420405 | FALSE |
| SNAPIN | 12855 | 0.697198 | 0.485679 | 0.6427219 | FALSE |
| CDA | 5931 | 0.697032 | 0.485783 | 0.6427628 | FALSE |
| ASPH | 4044 | 0.696797 | 0.48593 | 0.6429029 | FALSE |
| FAM220A | 2791 | 0.69668 | 0.486003 | 0.6429308 | FALSE |
| TNFSF9 | 10843 | 0.696421 | 0.486165 | 0.6429967 | FALSE |
| GHRHR | 7694 | 0.695686 | 0.486625 | 0.6433877 | FALSE |
| TXNL4B | 1903 | 0.694527 | 0.487352 | 0.6442527 | FALSE |
| ITPRIP | 12566 | 0.694464 | 0.487391 | 0.6442527 | FALSE |
| KMT2C | 13552 | 0.694379 | 0.487445 | 0.6442527 | FALSE |
| KCNJ4 | 10099 | 0.693533 | 0.487975 | 0.6448449 | FALSE |
| ACTC1 | 15021 | 0.693145 | 0.488219 | 0.6451121 | FALSE |
| KCNH2 | 9820 | 0.692797 | 0.488437 | 0.6452916 | FALSE |
| WDR83 | 3923 | 0.692468 | 0.488643 | 0.6455098 | FALSE |
| CRLF3 | 4845 | 0.692342 | 0.488723 | 0.6455598 | FALSE |
| WT1-AS | 12497 | 0.69178 | 0.489075 | 0.6459168 | FALSE |
| C15orf62 | 9813 | 0.691486 | 0.48926 | 0.6461061 | FALSE |
| ORMDL3 | 2209 | 0.691232 | 0.48942 | 0.6462077 | FALSE |
| CPSF6 | 4949 | 0.690951 | 0.489596 | 0.6463862 | FALSE |
| PLEKHA2 | 1960 | 0.690631 | 0.489797 | 0.646488 | FALSE |
| CMTM6 | 6147 | 0.69049 | 0.489886 | 0.6465504 | FALSE |
| SLC26A8 | 10092 | 0.690364 | 0.489965 | 0.6466003 | FALSE |
| PFDN6 | 1831 | 0.688959 | 0.490849 | 0.6474933 | FALSE |
| SRSF10 | 14856 | 0.688225 | 0.491311 | 0.6477202 | FALSE |
| NANOG | 13624 | 0.687887 | 0.491524 | 0.6478916 | FALSE |
| PKNOX1 | 14327 | 0.68624 | 0.492562 | 0.6490954 | FALSE |
| GTF2H3 | 11077 | 0.685703 | 0.4929 | 0.6494321 | FALSE |
| C7orf13 | 253 | 0.685252 | 0.493185 | 0.6496146 | FALSE |
| SENP2 | 411 | 0.68522 | 0.493205 | 0.6496146 | FALSE |
| FBLL1 | 6266 | 0.685067 | 0.493302 | 0.649687 | FALSE |
| CABP5 | 11426 | 0.68499 | 0.49335 | 0.6496962 | FALSE |
| LIFR-AS1 | 13467 | 0.684477 | 0.493674 | 0.6499584 | FALSE |
| S1PR4 | 1707 | 0.683857 | 0.494065 | 0.6502673 | FALSE |
| NOL12 | 9413 | 0.683776 | 0.494117 | 0.6502673 | FALSE |
| SLC20A2 | 9757 | 0.683152 | 0.494511 | 0.650567 | FALSE |
| C19orf73 | 2094 | 0.68308 | 0.494556 | 0.6505721 | FALSE |
| DHX37 | 4319 | 0.682091 | 0.495181 | 0.6511204 | FALSE |
| GORAB | 174 | 0.680661 | 0.496086 | 0.6518506 | FALSE |
| SCOC | 7283 | 0.680642 | 0.496098 | 0.6518506 | FALSE |
| SUSD2 | 9581 | 0.68062 | 0.496112 | 0.6518506 | FALSE |
| ZDHHC8P1 | 4159 | 0.680458 | 0.496215 | 0.6519305 | FALSE |
| ADAM33 | 2368 | 0.678102 | 0.497707 | 0.6536168 | FALSE |
| SRP14 | 7912 | 0.677698 | 0.497963 | 0.6538725 | FALSE |
| SLC22A31 | 9073 | 0.677499 | 0.498089 | 0.6539541 | FALSE |
| MAGOH | 7277 | 0.677031 | 0.498386 | 0.6541242 | FALSE |
| CDIPT | 10863 | 0.676656 | 0.498624 | 0.6543266 | FALSE |
| C9orf50 | 8198 | 0.676443 | 0.498759 | 0.6544274 | FALSE |
| HPR | 6804 | 0.67588 | 0.499117 | 0.6546982 | FALSE |
| MARK2 | 3273 | 0.6758 | 0.499168 | 0.6547099 | FALSE |
| SFXN4 | 1788 | 0.675654 | 0.49926 | 0.6547766 | FALSE |
| MVB12A | 10432 | 0.675011 | 0.499669 | 0.6551474 | FALSE |
| FGFR4 | 8281 | 0.674874 | 0.499756 | 0.6552065 | FALSE |
| CCDC159 | 11877 | 0.674582 | 0.499941 | 0.6553399 | FALSE |
| LCE2D | 5891 | 0.67396 | 0.500337 | 0.6557358 | FALSE |
| NSD3 | 4019 | 0.673898 | 0.500376 | 0.6557358 | FALSE |
| MYBPH | 11378 | 0.673826 | 0.500422 | 0.6557358 | FALSE |
| RNF128 | 15451 | 0.673424 | 0.500678 | 0.6558892 | FALSE |
| LSM14A | 11854 | 0.672801 | 0.501074 | 0.6562192 | FALSE |
| RGS9BP | 12108 | 0.67235 | 0.501361 | 0.6564301 | FALSE |
| LCE6A | 8369 | 0.67222 | 0.501444 | 0.6564834 | FALSE |
| AGPAT4-IT1 | 13114 | 0.672067 | 0.501541 | 0.6565559 | FALSE |
| POTEF | 10247 | 0.671158 | 0.50212 | 0.6572036 | FALSE |
| MALL | 11424 | 0.670945 | 0.502256 | 0.6573262 | FALSE |
| ALG14 | 11859 | 0.670855 | 0.502313 | 0.6573462 | FALSE |
| IDUA | 13290 | 0.670732 | 0.502391 | 0.6573937 | FALSE |
| SOX10 | 6640 | 0.670606 | 0.502472 | 0.6574022 | FALSE |
| TRIM29 | 13432 | 0.670409 | 0.502597 | 0.6574022 | FALSE |
| RASGRP3 | 9804 | 0.670304 | 0.502664 | 0.6574022 | FALSE |
| CYP2B6 | 5763 | 0.670281 | 0.502679 | 0.6574022 | FALSE |
| ADAMTSL1 | 14253 | 0.669931 | 0.502902 | 0.6574139 | FALSE |
| TMEM246 | 11589 | 0.669424 | 0.503225 | 0.6576342 | FALSE |
| METTL27 | 11173 | 0.669344 | 0.503276 | 0.6576342 | FALSE |
| KLF15 | 24 | 0.669231 | 0.503348 | 0.6576342 | FALSE |
| CDSN | 4256 | 0.669222 | 0.503354 | 0.6576342 | FALSE |
| MINDY1 | 402 | 0.669152 | 0.503399 | 0.6576342 | FALSE |
| HBA2 | 13794 | 0.669117 | 0.503421 | 0.6576342 | FALSE |
| SHISAL2A | 7827 | 0.669058 | 0.503458 | 0.6576342 | FALSE |
| RRAGA | 2320 | 0.668587 | 0.503759 | 0.6579168 | FALSE |
| ILDR1 | 6448 | 0.668413 | 0.50387 | 0.6580068 | FALSE |
| C11orf96 | 4160 | 0.667961 | 0.504158 | 0.6582595 | FALSE |
| ARSK | 2817 | 0.667907 | 0.504193 | 0.6582595 | FALSE |
| BCDIN3D | 10582 | 0.66778 | 0.504274 | 0.6582595 | FALSE |
| CAMK2N1 | 12945 | 0.667648 | 0.504358 | 0.6583146 | FALSE |
| CGB1 | 1349 | 0.667505 | 0.50445 | 0.6583788 | FALSE |
| KCTD2 | 9052 | 0.666546 | 0.505062 | 0.6590132 | FALSE |
| GABARAPL3 | 15190 | 0.666208 | 0.505278 | 0.659095 | FALSE |
| TRIB2 | 14063 | 0.665874 | 0.505492 | 0.6592984 | FALSE |
| KRBOX1 | 12988 | 0.665247 | 0.505893 | 0.6597112 | FALSE |
| PCDHGC4 | 13254 | 0.664209 | 0.506557 | 0.6603226 | FALSE |
| FAM200A | 6207 | 0.664172 | 0.50658 | 0.6603226 | FALSE |
| ENO3 | 2892 | 0.664052 | 0.506657 | 0.6603226 | FALSE |
| GRM2 | 10018 | 0.663842 | 0.506791 | 0.6604427 | FALSE |
| WNT5A | 2778 | 0.663407 | 0.50707 | 0.6607505 | FALSE |
| ATF7IP | 13472 | 0.662876 | 0.50741 | 0.6609733 | FALSE |
| MTMR10 | 4932 | 0.662725 | 0.507507 | 0.6610442 | FALSE |
| HNMT | 8991 | 0.662476 | 0.507666 | 0.6611418 | FALSE |
| PIN1P1 | 5041 | 0.662066 | 0.507929 | 0.6613186 | FALSE |
| CYP4A11 | 3447 | 0.661903 | 0.508033 | 0.6613996 | FALSE |
| FAM98A | 7935 | 0.661786 | 0.508108 | 0.6614421 | FALSE |
| ACAD8 | 13557 | 0.661359 | 0.508382 | 0.6616323 | FALSE |
| PTPN13 | 12347 | 0.661296 | 0.508423 | 0.6616323 | FALSE |
| CFP | 6246 | 0.660788 | 0.508748 | 0.6619995 | FALSE |
| NGB | 11740 | 0.660413 | 0.508989 | 0.6622023 | FALSE |
| TLN1 | 5147 | 0.660325 | 0.509045 | 0.6622206 | FALSE |
| SHPK | 10593 | 0.660184 | 0.509136 | 0.6622832 | FALSE |
| NCOA6 | 227 | 0.659149 | 0.5098 | 0.6629267 | FALSE |
| TXNL1 | 7689 | 0.658282 | 0.510357 | 0.6634217 | FALSE |
| CA8 | 8498 | 0.658279 | 0.510359 | 0.6634217 | FALSE |
| DAPK2 | 8040 | 0.658226 | 0.510393 | 0.6634217 | FALSE |
| PSMA3 | 10666 | 0.657929 | 0.510584 | 0.6635076 | FALSE |
| LINC01399 | 10741 | 0.657925 | 0.510586 | 0.6635076 | FALSE |
| FOXC2 | 11005 | 0.657722 | 0.510717 | 0.6636219 | FALSE |
| KIF13A | 11206 | 0.65761 | 0.510789 | 0.6636603 | FALSE |
| TMSB15B | 9182 | 0.656664 | 0.511397 | 0.6642849 | FALSE |
| CXorf67 | 14712 | 0.656481 | 0.511515 | 0.6643826 | FALSE |
| C22orf15 | 3528 | 0.656285 | 0.511641 | 0.6644217 | FALSE |
| SRSF3 | 2191 | 0.65617 | 0.511715 | 0.6644217 | FALSE |
| PDE4C | 5594 | 0.655311 | 0.512268 | 0.6649252 | FALSE |
| ASCL5 | 804 | 0.654556 | 0.512754 | 0.6654942 | FALSE |
| MBIP | 13847 | 0.653286 | 0.513572 | 0.6662712 | FALSE |
| NLGN2 | 10732 | 0.653254 | 0.513593 | 0.6662712 | FALSE |
| DNASE1L1 | 4592 | 0.65323 | 0.513608 | 0.6662712 | FALSE |
| THRB | 1136 | 0.653147 | 0.513662 | 0.6662853 | FALSE |
| BMP6 | 4569 | 0.653049 | 0.513725 | 0.666312 | FALSE |
| TTYH2 | 13252 | 0.65292 | 0.513808 | 0.6663193 | FALSE |
| FBXL5 | 3439 | 0.652908 | 0.513816 | 0.6663193 | FALSE |
| OTOP2 | 7885 | 0.652774 | 0.513902 | 0.6663761 | FALSE |
| SPRED1 | 1906 | 0.652402 | 0.514142 | 0.6665765 | FALSE |
| GADD45G | 4670 | 0.651923 | 0.514451 | 0.6669218 | FALSE |
| SNU13 | 7464 | 0.651676 | 0.51461 | 0.6669959 | FALSE |
| ZNF611 | 5900 | 0.65153 | 0.514704 | 0.6670293 | FALSE |
| SPRY4 | 12099 | 0.651205 | 0.514914 | 0.6672458 | FALSE |
| ESPNL | 4501 | 0.650638 | 0.51528 | 0.6676648 | FALSE |
| SPDYE3 | 7724 | 0.650397 | 0.515436 | 0.6677557 | FALSE |
| GLT1D1 | 2057 | 0.649973 | 0.51571 | 0.6680551 | FALSE |
| B4GALNT1 | 14434 | 0.64926 | 0.51617 | 0.6685411 | FALSE |
| LIN37 | 4404 | 0.648822 | 0.516453 | 0.6687414 | FALSE |
| KRTAP20-2 | 4168 | 0.647928 | 0.517032 | 0.6693363 | FALSE |
| HSPA2 | 12800 | 0.647913 | 0.517041 | 0.6693363 | FALSE |
| ZC4H2 | 2462 | 0.647463 | 0.517332 | 0.6695468 | FALSE |
| PANK3 | 156 | 0.646403 | 0.518018 | 0.6703238 | FALSE |
| C2 | 2518 | 0.646195 | 0.518153 | 0.6704426 | FALSE |
| CSTF3 | 5164 | 0.645722 | 0.518459 | 0.6706749 | FALSE |
| ZSCAN1 | 1799 | 0.645719 | 0.518461 | 0.6706749 | FALSE |
| KPNA3 | 10170 | 0.644837 | 0.519033 | 0.6712618 | FALSE |
| OPLAH | 5611 | 0.64482 | 0.519044 | 0.6712618 | FALSE |
| CD53 | 6645 | 0.64445 | 0.519284 | 0.6715164 | FALSE |
| CD247 | 9628 | 0.643626 | 0.519818 | 0.6719695 | FALSE |
| C1orf56 | 12418 | 0.643576 | 0.51985 | 0.6719695 | FALSE |
| MGME1 | 2650 | 0.643113 | 0.520151 | 0.6722484 | FALSE |
| TRIM49 | 10476 | 0.641671 | 0.521087 | 0.6733469 | FALSE |
| KRTAP10-10 | 13463 | 0.640175 | 0.522059 | 0.6742685 | FALSE |
| LHFPL5 | 14878 | 0.639814 | 0.522294 | 0.6744602 | FALSE |
| HIST1H3E | 82 | 0.639471 | 0.522517 | 0.674581 | FALSE |
| APOA1 | 9633 | 0.639212 | 0.522685 | 0.6746871 | FALSE |
| PRB3 | 6624 | 0.638694 | 0.523022 | 0.6749549 | FALSE |
| SQSTM1 | 14920 | 0.638542 | 0.523121 | 0.6750268 | FALSE |
| PARK7 | 7465 | 0.637992 | 0.523479 | 0.6754329 | FALSE |
| CCNY | 5540 | 0.637612 | 0.523726 | 0.675573 | FALSE |
| HIP1 | 4818 | 0.637427 | 0.523847 | 0.675573 | FALSE |
| INHA | 2531 | 0.637032 | 0.524104 | 0.675849 | FALSE |
| CCHCR1 | 11669 | 0.636871 | 0.524209 | 0.6759285 | FALSE |
| JAKMIP1 | 12141 | 0.636641 | 0.524359 | 0.6760102 | FALSE |
| DNER | 3320 | 0.636525 | 0.524434 | 0.6760323 | FALSE |
| ZMYM1 | 10662 | 0.63629 | 0.524587 | 0.6760904 | FALSE |
| RFX3 | 6707 | 0.63628 | 0.524594 | 0.6760904 | FALSE |
| IFITM5 | 5166 | 0.635802 | 0.524905 | 0.6763804 | FALSE |
| RPSAP9 | 11812 | 0.633534 | 0.526385 | 0.677657 | FALSE |
| CEBPD | 6388 | 0.633486 | 0.526416 | 0.677657 | FALSE |
| COG4 | 15408 | 0.63328 | 0.526551 | 0.6777077 | FALSE |
| ELOVL2 | 13303 | 0.632855 | 0.526828 | 0.6779083 | FALSE |
| CNTNAP4 | 935 | 0.632625 | 0.526979 | 0.6780457 | FALSE |
| MARS | 14226 | 0.63253 | 0.527041 | 0.6780698 | FALSE |
| GNAI1 | 2553 | 0.632021 | 0.527373 | 0.6782743 | FALSE |
| IK | 2142 | 0.63182 | 0.527505 | 0.6782744 | FALSE |
| STT3A | 2868 | 0.631782 | 0.527529 | 0.6782744 | FALSE |
| HIST1H2BB | 1310 | 0.631736 | 0.527559 | 0.6782744 | FALSE |
| GABBR1 | 8005 | 0.631554 | 0.527678 | 0.678332 | FALSE |
| KRTAP5-8 | 9280 | 0.6313 | 0.527844 | 0.6783405 | FALSE |
| NXT1 | 13805 | 0.631012 | 0.528033 | 0.678397 | FALSE |
| ZBTB12 | 9282 | 0.630853 | 0.528137 | 0.6784748 | FALSE |
| KCNK7 | 14994 | 0.630724 | 0.528221 | 0.6785274 | FALSE |
| NPAS2 | 3459 | 0.630345 | 0.528469 | 0.6787617 | FALSE |
| IMPG1 | 1899 | 0.630265 | 0.528521 | 0.6787617 | FALSE |
| PRR25 | 1888 | 0.630051 | 0.528661 | 0.678814 | FALSE |
| PRKAG2 | 8494 | 0.629364 | 0.529111 | 0.679232 | FALSE |
| COPZ2 | 9771 | 0.629288 | 0.529161 | 0.679232 | FALSE |
| ZNF816 | 8337 | 0.629203 | 0.529216 | 0.6792476 | FALSE |
| CILP | 15095 | 0.628847 | 0.529449 | 0.6794764 | FALSE |
| CREG1 | 125 | 0.628379 | 0.529756 | 0.679644 | FALSE |
| RYBP | 12100 | 0.627968 | 0.530025 | 0.6797834 | FALSE |
| C9orf40 | 1490 | 0.62759 | 0.530273 | 0.6800452 | FALSE |
| PRR15L | 15494 | 0.627495 | 0.530335 | 0.6800692 | FALSE |
| HIST1H3B | 4092 | 0.62722 | 0.530515 | 0.6801888 | FALSE |
| GORASP2 | 9046 | 0.627111 | 0.530586 | 0.6802246 | FALSE |
| AVL9 | 10481 | 0.626557 | 0.53095 | 0.6805228 | FALSE |
| KSR1 | 2614 | 0.626119 | 0.531237 | 0.6808351 | FALSE |
| CTU1 | 2449 | 0.625933 | 0.531359 | 0.6809356 | FALSE |
| CAND2 | 6092 | 0.62521 | 0.531833 | 0.6814876 | FALSE |
| PHLDB1 | 1131 | 0.624839 | 0.532077 | 0.6817438 | FALSE |
| ZFR | 13379 | 0.624728 | 0.53215 | 0.6817812 | FALSE |
| STK4 | 5737 | 0.624646 | 0.532203 | 0.6817943 | FALSE |
| KMT5C | 6432 | 0.624296 | 0.532433 | 0.681889 | FALSE |
| ANKRD11 | 4722 | 0.624266 | 0.532453 | 0.681889 | FALSE |
| HNRNPA1 | 8086 | 0.624079 | 0.532576 | 0.681889 | FALSE |
| DEFB135 | 9854 | 0.624058 | 0.532589 | 0.681889 | FALSE |
| LOC101929384 | 10786 | 0.624002 | 0.532626 | 0.681889 | FALSE |
| PLEKHG4 | 8902 | 0.623108 | 0.533214 | 0.6823403 | FALSE |
| ZNF585A | 8737 | 0.622844 | 0.533387 | 0.6823403 | FALSE |
| BDNF-AS | 11123 | 0.622532 | 0.533592 | 0.6824548 | FALSE |
| PRTFDC1 | 11500 | 0.622405 | 0.533676 | 0.6825058 | FALSE |
| ZC3HAV1L | 8487 | 0.621797 | 0.534075 | 0.6826988 | FALSE |
| TTC36 | 15014 | 0.621777 | 0.534089 | 0.6826988 | FALSE |
| NCAPH2 | 1224 | 0.620598 | 0.534864 | 0.683326 | FALSE |
| AXIN2 | 5380 | 0.620566 | 0.534885 | 0.683326 | FALSE |
| IFITM4P | 2131 | 0.620369 | 0.535015 | 0.6834358 | FALSE |
| RAD23A | 10418 | 0.620129 | 0.535173 | 0.6835818 | FALSE |
| YIPF6 | 7429 | 0.619715 | 0.535445 | 0.6838741 | FALSE |
| MRPL54 | 12389 | 0.619535 | 0.535564 | 0.6839507 | FALSE |
| COQ8B | 13241 | 0.617773 | 0.536725 | 0.6849487 | FALSE |
| TMEM123 | 3351 | 0.617608 | 0.536834 | 0.6850316 | FALSE |
| FHIT | 4575 | 0.617526 | 0.536888 | 0.6850447 | FALSE |
| TADA1 | 5627 | 0.617433 | 0.536949 | 0.685067 | FALSE |
| RAD23B | 6969 | 0.617228 | 0.537084 | 0.685142 | FALSE |
| NRBP2 | 204 | 0.617211 | 0.537096 | 0.685142 | FALSE |
| EVPL | 6391 | 0.616983 | 0.537246 | 0.685222 | FALSE |
| IGHMBP2 | 4346 | 0.616884 | 0.537311 | 0.6852494 | FALSE |
| LRP6 | 12072 | 0.616454 | 0.537595 | 0.6854838 | FALSE |
| TTC27 | 1003 | 0.616406 | 0.537627 | 0.6854838 | FALSE |
| MTMR11 | 9097 | 0.615716 | 0.538082 | 0.6859525 | FALSE |
| BEST1 | 7172 | 0.614976 | 0.538571 | 0.6864718 | FALSE |
| DGUOK | 6913 | 0.614644 | 0.53879 | 0.6866309 | FALSE |
| HMGB1 | 14604 | 0.613907 | 0.539277 | 0.6869715 | FALSE |
| NAIP | 2443 | 0.613505 | 0.539542 | 0.6872139 | FALSE |
| SF3B2 | 7234 | 0.613376 | 0.539628 | 0.6872139 | FALSE |
| GGCX | 10775 | 0.613353 | 0.539643 | 0.6872139 | FALSE |
| TMED5 | 242 | 0.612951 | 0.539909 | 0.6874964 | FALSE |
| PFKFB3 | 3114 | 0.611961 | 0.540564 | 0.6882181 | FALSE |
| LOC105376805 | 8311 | 0.611836 | 0.540646 | 0.6882674 | FALSE |
| RASL12 | 13061 | 0.611606 | 0.540798 | 0.6884051 | FALSE |
| DNAJC9-AS1 | 13782 | 0.611423 | 0.54092 | 0.6885032 | FALSE |
| CAT | 15455 | 0.6109 | 0.541266 | 0.6887756 | FALSE |
| KLHL12 | 9194 | 0.610727 | 0.54138 | 0.688837 | FALSE |
| TIMM23 | 9450 | 0.610548 | 0.541499 | 0.6888723 | FALSE |
| TEP1 | 9945 | 0.610519 | 0.541518 | 0.6888723 | FALSE |
| ATP2B2-IT2 | 13630 | 0.610308 | 0.541658 | 0.6889472 | FALSE |
| NAT6 | 12935 | 0.610297 | 0.541665 | 0.6889472 | FALSE |
| DUSP16 | 11429 | 0.609702 | 0.542059 | 0.6893645 | FALSE |
| TEAD3 | 10384 | 0.609339 | 0.5423 | 0.6895301 | FALSE |
| PI4K2B | 12558 | 0.609249 | 0.542359 | 0.6895498 | FALSE |
| IQSEC2 | 4711 | 0.609097 | 0.54246 | 0.6895657 | FALSE |
| SSRP1 | 13894 | 0.608978 | 0.542539 | 0.6895876 | FALSE |
| TMEM216 | 8803 | 0.608767 | 0.542679 | 0.6896756 | FALSE |
| MAL | 8931 | 0.608448 | 0.54289 | 0.6898659 | FALSE |
| CFLAR | 4465 | 0.608303 | 0.542987 | 0.6898983 | FALSE |
| ZNF474 | 705 | 0.60724 | 0.543692 | 0.6906819 | FALSE |
| CLDND1 | 9302 | 0.606925 | 0.543901 | 0.6908288 | FALSE |
| DUSP15 | 4615 | 0.606866 | 0.54394 | 0.6908288 | FALSE |
| DMRTC2 | 9946 | 0.606438 | 0.544224 | 0.6910773 | FALSE |
| NCOA7 | 7257 | 0.606361 | 0.544275 | 0.6910861 | FALSE |
| S100P | 9931 | 0.606106 | 0.544444 | 0.6911394 | FALSE |
| SCRIB | 2176 | 0.606098 | 0.54445 | 0.6911394 | FALSE |
| RDM1 | 2227 | 0.60482 | 0.545299 | 0.6920485 | FALSE |
| SCAMP2 | 3200 | 0.604618 | 0.545433 | 0.6921626 | FALSE |
| SCN2A | 8757 | 0.604374 | 0.545595 | 0.6922454 | FALSE |
| ANKRD18A | 10202 | 0.60432 | 0.545631 | 0.6922454 | FALSE |
| CDC14C | 9437 | 0.603705 | 0.54604 | 0.6925955 | FALSE |
| KRTAP13-4 | 14983 | 0.603374 | 0.54626 | 0.6927834 | FALSE |
| GREM2 | 585 | 0.603349 | 0.546277 | 0.6927834 | FALSE |
| CSAD | 1627 | 0.60308 | 0.546455 | 0.6929541 | FALSE |
| PAF1 | 1610 | 0.60269 | 0.546715 | 0.6932269 | FALSE |
| FAM71F2 | 11365 | 0.602337 | 0.54695 | 0.6933797 | FALSE |
| AHNAK2 | 5974 | 0.602336 | 0.546951 | 0.6933797 | FALSE |
| CD27 | 15142 | 0.602309 | 0.546968 | 0.6933797 | FALSE |
| NAGA | 4656 | 0.6019 | 0.547241 | 0.6936123 | FALSE |
| NEMP2 | 13655 | 0.601201 | 0.547706 | 0.6940075 | FALSE |
| DEFB118 | 12335 | 0.601165 | 0.54773 | 0.6940075 | FALSE |
| MXRA7 | 8800 | 0.600797 | 0.547975 | 0.6942055 | FALSE |
| LINC00842 | 9022 | 0.600597 | 0.548108 | 0.6942618 | FALSE |
| PARP8 | 12067 | 0.600261 | 0.548332 | 0.6944576 | FALSE |
| TCEANC2 | 11400 | 0.600052 | 0.548472 | 0.6944967 | FALSE |
| CDK5R1 | 4444 | 0.599758 | 0.548668 | 0.6946323 | FALSE |
| DRD4 | 750 | 0.599389 | 0.548914 | 0.694751 | FALSE |
| SH3KBP1 | 15442 | 0.599381 | 0.548919 | 0.694751 | FALSE |
| CBL | 1537 | 0.599131 | 0.549086 | 0.6947676 | FALSE |
| SLMAP | 7953 | 0.598951 | 0.549206 | 0.6948633 | FALSE |
| FNDC3B | 12677 | 0.598757 | 0.549335 | 0.6949575 | FALSE |
| ZDHHC19 | 15541 | 0.598459 | 0.549534 | 0.6951097 | FALSE |
| USP18 | 11105 | 0.5979 | 0.549907 | 0.6954689 | FALSE |
| AHDC1 | 9883 | 0.597665 | 0.550063 | 0.6956109 | FALSE |
| GFER | 8111 | 0.597447 | 0.550209 | 0.6957387 | FALSE |
| DNAJB13 | 2436 | 0.596742 | 0.55068 | 0.6962212 | FALSE |
| SFTPC | 13134 | 0.596565 | 0.550798 | 0.6963143 | FALSE |
| ZC2HC1C | 1412 | 0.596457 | 0.55087 | 0.6963492 | FALSE |
| ARMC6 | 7726 | 0.596164 | 0.551066 | 0.6964839 | FALSE |
| MAP2K4 | 14967 | 0.595736 | 0.551352 | 0.696789 | FALSE |
| OPALIN | 1558 | 0.595641 | 0.551415 | 0.6968129 | FALSE |
| UBE2DNL | 7984 | 0.595468 | 0.551531 | 0.6969026 | FALSE |
| MBOAT1 | 1198 | 0.595096 | 0.551779 | 0.6970609 | FALSE |
| DMAP1 | 873 | 0.594984 | 0.551854 | 0.6970609 | FALSE |
| SCN4A | 4870 | 0.594784 | 0.551988 | 0.6971422 | FALSE |
| EFNB3 | 4407 | 0.594494 | 0.552182 | 0.6972744 | FALSE |
| RAB3IL1 | 1854 | 0.594325 | 0.552295 | 0.6973608 | FALSE |
| EIF2B1 | 10020 | 0.593952 | 0.552544 | 0.6976194 | FALSE |
| MAFF | 781 | 0.593573 | 0.552798 | 0.6978832 | FALSE |
| C19orf33 | 883 | 0.59292 | 0.553235 | 0.6983664 | FALSE |
| SET | 13768 | 0.592784 | 0.553326 | 0.6983664 | FALSE |
| AKAP8L | 15024 | 0.592734 | 0.553359 | 0.6983664 | FALSE |
| DMD | 3145 | 0.592464 | 0.55354 | 0.6985381 | FALSE |
| MNX1-AS1 | 690 | 0.59209 | 0.55379 | 0.6986707 | FALSE |
| MITD1 | 14965 | 0.59204 | 0.553824 | 0.6986707 | FALSE |
| ERCC1 | 6953 | 0.591102 | 0.554452 | 0.6993504 | FALSE |
| TMBIM4 | 2350 | 0.590699 | 0.554722 | 0.6996346 | FALSE |
| C12orf66 | 905 | 0.590125 | 0.555107 | 0.7000634 | FALSE |
| TPO | 9588 | 0.58917 | 0.555747 | 0.7006449 | FALSE |
| COLCA2 | 6184 | 0.588643 | 0.556101 | 0.7010114 | FALSE |
| ZWILCH | 7602 | 0.588143 | 0.556436 | 0.7011178 | FALSE |
| LINC01351 | 5957 | 0.587063 | 0.557161 | 0.7018617 | FALSE |
| MGC34796 | 5460 | 0.58673 | 0.557385 | 0.7020869 | FALSE |
| POU2F1 | 544 | 0.586375 | 0.557624 | 0.7022456 | FALSE |
| ATRIP | 10719 | 0.586325 | 0.557657 | 0.7022456 | FALSE |
| GDI2 | 8989 | 0.586275 | 0.557691 | 0.7022456 | FALSE |
| EMP2 | 6014 | 0.585901 | 0.557942 | 0.7025055 | FALSE |
| HIST1H2AG | 15472 | 0.585495 | 0.558215 | 0.7027925 | FALSE |
| CIAO1 | 4156 | 0.585373 | 0.558297 | 0.7028391 | FALSE |
| DLGAP4 | 12470 | 0.585273 | 0.558364 | 0.7028671 | FALSE |
| PURG | 2048 | 0.585124 | 0.558464 | 0.7028852 | FALSE |
| SPG7 | 8782 | 0.585109 | 0.558474 | 0.7028852 | FALSE |
| STYXL1 | 14173 | 0.585051 | 0.558513 | 0.7028852 | FALSE |
| PPP1R37 | 11623 | 0.584626 | 0.558799 | 0.7030751 | FALSE |
| SLC30A9 | 13039 | 0.584431 | 0.55893 | 0.7031304 | FALSE |
| ASAP3 | 4927 | 0.584408 | 0.558946 | 0.7031304 | FALSE |
| FAM114A1 | 8850 | 0.583648 | 0.559457 | 0.7036764 | FALSE |
| UBTD2 | 1709 | 0.58303 | 0.559873 | 0.7040296 | FALSE |
| KRTAP5-11 | 5746 | 0.582729 | 0.560076 | 0.7042278 | FALSE |
| OTUD5 | 2915 | 0.582613 | 0.560154 | 0.7042694 | FALSE |
| CHST5 | 7015 | 0.582244 | 0.560402 | 0.7045251 | FALSE |
| KRTAP5-7 | 6546 | 0.582036 | 0.560542 | 0.7046252 | FALSE |
| EID1 | 12623 | 0.581483 | 0.560915 | 0.7048862 | FALSE |
| NDUFA1 | 6112 | 0.581341 | 0.561011 | 0.7049497 | FALSE |
| PPP3R2 | 10320 | 0.581017 | 0.561229 | 0.7050541 | FALSE |
| ZNF678 | 9876 | 0.580858 | 0.561336 | 0.705132 | FALSE |
| GRB2 | 11881 | 0.580044 | 0.561885 | 0.7056024 | FALSE |
| INCA1 | 7635 | 0.579945 | 0.561952 | 0.7056024 | FALSE |
| PARP4 | 2433 | 0.579868 | 0.562004 | 0.7056024 | FALSE |
| KDM5B | 7266 | 0.579839 | 0.562023 | 0.7056024 | FALSE |
| GRIN2D | 2527 | 0.579834 | 0.562027 | 0.7056024 | FALSE |
| NHSL1 | 1535 | 0.579736 | 0.562093 | 0.7056287 | FALSE |
| LBX1 | 5479 | 0.579658 | 0.562145 | 0.7056381 | FALSE |
| IL9R | 3619 | 0.579564 | 0.562209 | 0.705661 | FALSE |
| ZNF280C | 992 | 0.579089 | 0.562529 | 0.7058941 | FALSE |
| CCND2 | 2503 | 0.57883 | 0.562704 | 0.7060525 | FALSE |
| PCIF1 | 2408 | 0.578305 | 0.563058 | 0.7063304 | FALSE |
| KLK14 | 9039 | 0.578028 | 0.563245 | 0.7064695 | FALSE |
| RCOR2 | 15362 | 0.577902 | 0.56333 | 0.7064695 | FALSE |
| PLIN4 | 14790 | 0.57786 | 0.563359 | 0.7064695 | FALSE |
| APH1A | 2505 | 0.577806 | 0.563395 | 0.7064695 | FALSE |
| HYKK | 9371 | 0.577667 | 0.563489 | 0.7065306 | FALSE |
| PIK3C2B | 8780 | 0.576488 | 0.564285 | 0.7071888 | FALSE |
| KMO | 1553 | 0.575908 | 0.564677 | 0.7076234 | FALSE |
| NUDCD1 | 8647 | 0.574885 | 0.565369 | 0.7083766 | FALSE |
| IRX1 | 8680 | 0.574506 | 0.565625 | 0.7084707 | FALSE |
| NPY4R | 2366 | 0.574196 | 0.565835 | 0.7086613 | FALSE |
| PGM3 | 3731 | 0.573764 | 0.566128 | 0.7087148 | FALSE |
| TRUB2 | 7459 | 0.573743 | 0.566142 | 0.7087148 | FALSE |
| MFSD13A | 10220 | 0.573676 | 0.566187 | 0.7087148 | FALSE |
| QSOX2 | 915 | 0.572757 | 0.566809 | 0.7092147 | FALSE |
| CSNK2A1 | 12077 | 0.572646 | 0.566884 | 0.709252 | FALSE |
| DMRTA2 | 1248 | 0.572048 | 0.567289 | 0.709702 | FALSE |
| B3GNT7 | 8890 | 0.571356 | 0.567758 | 0.7101749 | FALSE |
| PRPF40A | 6519 | 0.570216 | 0.568531 | 0.7108004 | FALSE |
| ART5 | 7297 | 0.569927 | 0.568727 | 0.7109886 | FALSE |
| PRM3 | 12848 | 0.569666 | 0.568904 | 0.7110962 | FALSE |
| FAM111A-DT | 15597 | 0.569431 | 0.569064 | 0.7112386 | FALSE |
| HMG20B | 7424 | 0.569089 | 0.569296 | 0.7114691 | FALSE |
| CHL1 | 116 | 0.568955 | 0.569387 | 0.7114691 | FALSE |
| FAM110D | 13050 | 0.568891 | 0.56943 | 0.7114691 | FALSE |
| PCBD2 | 9859 | 0.568641 | 0.5696 | 0.7115673 | FALSE |
| LMO4 | 10478 | 0.568455 | 0.569726 | 0.7115731 | FALSE |
| CLSTN1 | 7196 | 0.568433 | 0.569741 | 0.7115731 | FALSE |
| ASB4 | 2590 | 0.567645 | 0.570276 | 0.7120459 | FALSE |
| MORN1 | 8577 | 0.5676 | 0.570307 | 0.7120459 | FALSE |
| NUCB1 | 12018 | 0.567561 | 0.570333 | 0.7120459 | FALSE |
| RUVBL2 | 3843 | 0.567406 | 0.570438 | 0.7120459 | FALSE |
| TCF12 | 2742 | 0.566343 | 0.571161 | 0.712663 | FALSE |
| NCKAP5L | 10318 | 0.565878 | 0.571477 | 0.7129435 | FALSE |
| LGR5 | 6380 | 0.565684 | 0.571609 | 0.7130028 | FALSE |
| RXFP1 | 6652 | 0.565674 | 0.571615 | 0.7130028 | FALSE |
| ARSA | 15374 | 0.565549 | 0.5717 | 0.7130519 | FALSE |
| PLN | 4886 | 0.564743 | 0.572249 | 0.7135648 | FALSE |
| MANEA | 15046 | 0.564565 | 0.57237 | 0.7136589 | FALSE |
| BTF3 | 8179 | 0.564478 | 0.572429 | 0.7136758 | FALSE |
| ZNF727 | 11404 | 0.564278 | 0.572565 | 0.7137885 | FALSE |
| CASC4 | 14836 | 0.56384 | 0.572863 | 0.7140462 | FALSE |
| FAM27E3 | 10764 | 0.563631 | 0.573005 | 0.7141666 | FALSE |
| FAM210A | 11780 | 0.562397 | 0.573846 | 0.7150999 | FALSE |
| LOC401357 | 4055 | 0.561067 | 0.574752 | 0.715942 | FALSE |
| GTF2F1 | 14838 | 0.561002 | 0.574796 | 0.715942 | FALSE |
| JAG1 | 8818 | 0.560874 | 0.574883 | 0.7159468 | FALSE |
| SPATA2L | 14998 | 0.560862 | 0.574892 | 0.7159468 | FALSE |
| FUT8 | 12327 | 0.560473 | 0.575157 | 0.7160684 | FALSE |
| CPSF4 | 3122 | 0.56043 | 0.575186 | 0.7160684 | FALSE |
| LCE3A | 14725 | 0.559923 | 0.575532 | 0.7162878 | FALSE |
| HCP5 | 4215 | 0.559045 | 0.576131 | 0.7168868 | FALSE |
| CSDC2 | 4402 | 0.55812 | 0.576762 | 0.7174194 | FALSE |
| RPRD1A | 2252 | 0.557536 | 0.577161 | 0.7177355 | FALSE |
| ALDH3B1 | 7821 | 0.557483 | 0.577197 | 0.7177355 | FALSE |
| SNRNP200 | 8069 | 0.557479 | 0.5772 | 0.7177355 | FALSE |
| PRELID3A | 13197 | 0.555928 | 0.57826 | 0.7186533 | FALSE |
| FBXW11 | 8059 | 0.555089 | 0.578834 | 0.7192519 | FALSE |
| JMJD8 | 3452 | 0.554793 | 0.579036 | 0.7194463 | FALSE |
| PTPRS | 8216 | 0.554546 | 0.579205 | 0.7195991 | FALSE |
| KIAA0087 | 15491 | 0.554385 | 0.579315 | 0.7196788 | FALSE |
| THYN1 | 3773 | 0.553904 | 0.579645 | 0.7198631 | FALSE |
| C11orf42 | 11593 | 0.553899 | 0.579648 | 0.7198631 | FALSE |
| PIGM | 5990 | 0.55329 | 0.580065 | 0.7202664 | FALSE |
| TBC1D9B | 2708 | 0.552652 | 0.580502 | 0.7206978 | FALSE |
| AK6 | 3368 | 0.552516 | 0.580595 | 0.7207528 | FALSE |
| ATN1 | 7581 | 0.551838 | 0.581059 | 0.7212721 | FALSE |
| NTMT1 | 6816 | 0.551765 | 0.581109 | 0.7212769 | FALSE |
| DENR | 518 | 0.551431 | 0.581338 | 0.7215037 | FALSE |
| CLDN19 | 6632 | 0.551227 | 0.581478 | 0.7215661 | FALSE |
| RAB28 | 9850 | 0.550973 | 0.581652 | 0.7217215 | FALSE |
| TTLL13P | 7409 | 0.550574 | 0.581926 | 0.7220036 | FALSE |
| ZNF66 | 14948 | 0.55025 | 0.582148 | 0.7220962 | FALSE |
| ARHGEF1 | 9743 | 0.550185 | 0.582192 | 0.7220962 | FALSE |
| NFU1 | 4631 | 0.550061 | 0.582278 | 0.7220962 | FALSE |
| ASNSP1 | 5836 | 0.549191 | 0.582874 | 0.7227146 | FALSE |
| MYOD1 | 7619 | 0.549132 | 0.582915 | 0.7227146 | FALSE |
| PTPN9 | 1461 | 0.548924 | 0.583058 | 0.7228342 | FALSE |
| USP12 | 5779 | 0.548456 | 0.583379 | 0.7231178 | FALSE |
| PDK4 | 10758 | 0.547903 | 0.583759 | 0.7234538 | FALSE |
| RPS6KA2 | 8430 | 0.547859 | 0.583789 | 0.7234538 | FALSE |
| RHOU | 6285 | 0.547648 | 0.583934 | 0.7235528 | FALSE |
| BCL2L12 | 1575 | 0.547576 | 0.583983 | 0.7235528 | FALSE |
| PHIP | 5760 | 0.547473 | 0.584054 | 0.7235528 | FALSE |
| KRTAP13-1 | 815 | 0.547377 | 0.58412 | 0.7235772 | FALSE |
| MED25 | 8366 | 0.547036 | 0.584354 | 0.72381 | FALSE |
| STOML2 | 7057 | 0.546967 | 0.584401 | 0.7238114 | FALSE |
| CHGB | 6406 | 0.546319 | 0.584847 | 0.7242384 | FALSE |
| MST1 | 12610 | 0.545479 | 0.585424 | 0.7247336 | FALSE |
| RAD9A | 1300 | 0.544934 | 0.585799 | 0.7251006 | FALSE |
| AP4B1 | 3884 | 0.544913 | 0.585813 | 0.7251006 | FALSE |
| CTU2 | 6811 | 0.54453 | 0.586077 | 0.7252957 | FALSE |
| DHX36 | 5090 | 0.544443 | 0.586137 | 0.7252957 | FALSE |
| TBC1D3P2 | 3548 | 0.544414 | 0.586157 | 0.7252957 | FALSE |
| TMEM259 | 8831 | 0.543574 | 0.586735 | 0.7258961 | FALSE |
| GEMIN2 | 2840 | 0.543484 | 0.586797 | 0.7259153 | FALSE |
| IGLON5 | 388 | 0.54328 | 0.586937 | 0.7260315 | FALSE |
| CHPF | 11292 | 0.543108 | 0.587055 | 0.7261031 | FALSE |
| PRX | 6897 | 0.543061 | 0.587088 | 0.7261031 | FALSE |
| C20orf144 | 4468 | 0.5429 | 0.587199 | 0.7261828 | FALSE |
| FAM124A | 3832 | 0.542605 | 0.587402 | 0.7263191 | FALSE |
| LY6H | 13116 | 0.542149 | 0.587716 | 0.7265925 | FALSE |
[truncated: 526,354 more chars]
