# Supplementary material for: Brain tissue electrical conductivity as a promising biomarker for dementia assessment using MRI
Source: Alzheimers Dement. 2025 Jun 23;21(6):e70270. doi: 10.1002/alz.70270 (PMC12185248; doi:10.1002/alz.70270)
Supplement: Supplementary file 4 — Supporting Information [file ALZ-21-e70270-s007.docx]

**Table S17.** The complete set of PLS2 gene weights and associated statistics for tau SUVRs difference between Dementia and cognitively normal participants.

| geneIDs | geneIDX | zscores | pvals | adj.pvals | significant |
| --- | --- | --- | --- | --- | --- |
| SCGN | 3232 | 9.381704 | 6.49E-21 | 1.01E-16 | TRUE |
| C5orf49 | 14560 | 8.983488 | 2.62E-19 | 2.05E-15 | TRUE |
| GRIN3A | 3420 | 8.925262 | 4.45E-19 | 2.32E-15 | TRUE |
| SLA | 8624 | 8.883447 | 6.48E-19 | 2.53E-15 | TRUE |
| SLN | 1997 | 8.53462 | 1.41E-17 | 3.66E-14 | TRUE |
| LINC01140 | 7147 | 8.515422 | 1.66E-17 | 3.71E-14 | TRUE |
| TMEM159 | 14213 | 8.477149 | 2.31E-17 | 4.51E-14 | TRUE |
| FAM71F1 | 2287 | 8.416216 | 3.89E-17 | 6.09E-14 | TRUE |
| SVOP | 4830 | 8.415933 | 3.90E-17 | 6.09E-14 | TRUE |
| CHMP1A | 11170 | 8.377918 | 5.39E-17 | 7.66E-14 | TRUE |
| SPON2 | 2337 | 8.266161 | 1.38E-16 | 1.80E-13 | TRUE |
| FSTL5 | 1450 | 8.226124 | 1.93E-16 | 2.16E-13 | TRUE |
| RBP4 | 9759 | 8.137567 | 4.03E-16 | 4.20E-13 | TRUE |
| CENPW | 9988 | 8.105767 | 5.24E-16 | 5.12E-13 | TRUE |
| TXNL4A | 6401 | 8.033219 | 9.49E-16 | 8.25E-13 | TRUE |
| RRP7A | 11298 | 7.972777 | 1.55E-15 | 1.28E-12 | TRUE |
| GMFB | 3372 | 7.954629 | 1.80E-15 | 1.39E-12 | TRUE |
| SCN3B | 3473 | 7.944317 | 1.95E-15 | 1.39E-12 | TRUE |
| FAM110C | 1632 | 7.859573 | 3.85E-15 | 2.51E-12 | TRUE |
| NDUFAF8 | 11462 | 7.733682 | 1.04E-14 | 5.83E-12 | TRUE |
| BATF3 | 9428 | 7.716559 | 1.20E-14 | 6.44E-12 | TRUE |
| ASCL2 | 12462 | 7.696186 | 1.40E-14 | 7.14E-12 | TRUE |
| TIMM8B | 6527 | 7.685526 | 1.52E-14 | 7.44E-12 | TRUE |
| PRKCD | 9266 | 7.674415 | 1.66E-14 | 7.87E-12 | TRUE |
| RIPOR2 | 1584 | 7.620246 | 2.53E-14 | 1.10E-11 | TRUE |
| SYT17 | 5315 | 7.604459 | 2.86E-14 | 1.21E-11 | TRUE |
| ATP2B4 | 3024 | 7.594677 | 3.09E-14 | 1.27E-11 | TRUE |
| FABP5P3 | 2878 | 7.514442 | 5.72E-14 | 2.18E-11 | TRUE |
| SPPL3 | 9332 | 7.428834 | 1.10E-13 | 3.64E-11 | TRUE |
| PID1 | 7463 | 7.415034 | 1.22E-13 | 3.88E-11 | TRUE |
| SLC7A4 | 12791 | 7.393713 | 1.43E-13 | 4.38E-11 | TRUE |
| HTR7 | 11112 | 7.383777 | 1.54E-13 | 4.49E-11 | TRUE |
| PCDH20 | 14748 | 7.382613 | 1.55E-13 | 4.49E-11 | TRUE |
| CXorf57 | 2813 | 7.375637 | 1.64E-13 | 4.58E-11 | TRUE |
| SLIT1 | 12013 | 7.375266 | 1.64E-13 | 4.58E-11 | TRUE |
| ACOT8 | 1990 | 7.357125 | 1.88E-13 | 5.15E-11 | TRUE |
| C1QL3 | 1179 | 7.318636 | 2.51E-13 | 6.75E-11 | TRUE |
| P2RY13 | 1897 | 7.301863 | 2.84E-13 | 7.48E-11 | TRUE |
| PPP1R1A | 7058 | 7.299024 | 2.90E-13 | 7.48E-11 | TRUE |
| SPHKAP | 266 | 7.275349 | 3.46E-13 | 8.57E-11 | TRUE |
| DDAH1 | 1521 | 7.261434 | 3.83E-13 | 9.34E-11 | TRUE |
| FKBP1A | 5085 | 7.259551 | 3.88E-13 | 9.34E-11 | TRUE |
| HTR1A | 7466 | 7.246437 | 4.28E-13 | 1.01E-10 | TRUE |
| LOXL1 | 8655 | 7.22914 | 4.86E-13 | 1.12E-10 | TRUE |
| SMARCD3 | 9449 | 7.222354 | 5.11E-13 | 1.16E-10 | TRUE |
| TUBB6 | 133 | 7.208328 | 5.66E-13 | 1.25E-10 | TRUE |
| CPAMD8 | 7410 | 7.20729 | 5.71E-13 | 1.25E-10 | TRUE |
| WDR86 | 13442 | 7.196034 | 6.20E-13 | 1.31E-10 | TRUE |
| GABRA5 | 3770 | 7.183644 | 6.79E-13 | 1.41E-10 | TRUE |
| AIF1 | 3077 | 7.181585 | 6.89E-13 | 1.42E-10 | TRUE |
| GTF2H5 | 13693 | 7.144117 | 9.06E-13 | 1.79E-10 | TRUE |
| WDFY4 | 4815 | 7.119425 | 1.08E-12 | 2.09E-10 | TRUE |
| PNCK | 14371 | 7.091155 | 1.33E-12 | 2.50E-10 | TRUE |
| GPR68 | 13756 | 7.077175 | 1.47E-12 | 2.74E-10 | TRUE |
| ADM5 | 5410 | 7.032237 | 2.03E-12 | 3.69E-10 | TRUE |
| POLR2L | 9466 | 6.991477 | 2.72E-12 | 4.83E-10 | TRUE |
| PTGER3 | 11200 | 6.977126 | 3.01E-12 | 5.28E-10 | TRUE |
| SPACA9 | 2329 | 6.975967 | 3.04E-12 | 5.28E-10 | TRUE |
| STUM | 10030 | 6.971486 | 3.14E-12 | 5.39E-10 | TRUE |
| GMFG | 9532 | 6.947281 | 3.72E-12 | 6.26E-10 | TRUE |
| DPYSL3 | 14244 | 6.920826 | 4.49E-12 | 7.24E-10 | TRUE |
| NKAIN4 | 5819 | 6.915979 | 4.65E-12 | 7.41E-10 | TRUE |
| SLC27A5 | 9599 | 6.90702 | 4.95E-12 | 7.82E-10 | TRUE |
| TMSB10 | 7775 | 6.903222 | 5.08E-12 | 7.88E-10 | TRUE |
| RIIAD1 | 7462 | 6.902988 | 5.09E-12 | 7.88E-10 | TRUE |
| OPRK1 | 9053 | 6.893142 | 5.46E-12 | 8.36E-10 | TRUE |
| IL17RD | 993 | 6.890444 | 5.56E-12 | 8.41E-10 | TRUE |
| GNB4 | 11644 | 6.881359 | 5.93E-12 | 8.66E-10 | TRUE |
| ERICH6-AS1 | 2883 | 6.881357 | 5.93E-12 | 8.66E-10 | TRUE |
| GDA | 12675 | 6.872849 | 6.29E-12 | 9.03E-10 | TRUE |
| SHISAL1 | 1122 | 6.870569 | 6.39E-12 | 9.09E-10 | TRUE |
| RNASEH2C | 8785 | 6.867221 | 6.55E-12 | 9.21E-10 | TRUE |
| WTIP | 11757 | 6.864316 | 6.68E-12 | 9.24E-10 | TRUE |
| TRAPPC2L | 7607 | 6.860733 | 6.85E-12 | 9.39E-10 | TRUE |
| PACRG | 6614 | 6.85798 | 6.98E-12 | 9.49E-10 | TRUE |
| GPC4 | 2880 | 6.852634 | 7.25E-12 | 9.71E-10 | TRUE |
| TIMM10 | 7406 | 6.848639 | 7.46E-12 | 9.79E-10 | TRUE |
| C1QA | 4795 | 6.845775 | 7.61E-12 | 9.91E-10 | TRUE |
| MEIS3 | 5641 | 6.812488 | 9.59E-12 | 1.24E-09 | TRUE |
| ABLIM3 | 11168 | 6.793018 | 1.10E-11 | 1.38E-09 | TRUE |
| OST4 | 3204 | 6.777952 | 1.22E-11 | 1.50E-09 | TRUE |
| SH3BGRL3 | 14874 | 6.765984 | 1.32E-11 | 1.62E-09 | TRUE |
| PNMT | 4787 | 6.759102 | 1.39E-11 | 1.68E-09 | TRUE |
| FOLR2 | 13985 | 6.750262 | 1.48E-11 | 1.77E-09 | TRUE |
| C1orf50 | 11246 | 6.746319 | 1.52E-11 | 1.81E-09 | TRUE |
| CETN2 | 7780 | 6.718814 | 1.83E-11 | 2.13E-09 | TRUE |
| FNBP1L | 7492 | 6.70948 | 1.95E-11 | 2.22E-09 | TRUE |
| LRRC36 | 2390 | 6.707926 | 1.97E-11 | 2.22E-09 | TRUE |
| BAIAP3 | 1554 | 6.677401 | 2.43E-11 | 2.64E-09 | TRUE |
| SRM | 3782 | 6.664701 | 2.65E-11 | 2.84E-09 | TRUE |
| ISOC1 | 12655 | 6.65798 | 2.78E-11 | 2.95E-09 | TRUE |
| PLPPR2 | 13352 | 6.648552 | 2.96E-11 | 3.08E-09 | TRUE |
| PKIA | 13528 | 6.645731 | 3.02E-11 | 3.11E-09 | TRUE |
| CPNE6 | 13234 | 6.644757 | 3.04E-11 | 3.11E-09 | TRUE |
| SPATA33 | 5111 | 6.644159 | 3.05E-11 | 3.11E-09 | TRUE |
| BCKDK | 12803 | 6.640911 | 3.12E-11 | 3.12E-09 | TRUE |
| TBCA | 1113 | 6.632649 | 3.30E-11 | 3.22E-09 | TRUE |
| HBQ1 | 3936 | 6.629112 | 3.38E-11 | 3.26E-09 | TRUE |
| DLL3 | 5156 | 6.61189 | 3.79E-11 | 3.62E-09 | TRUE |
| CAMK2D | 2610 | 6.604057 | 4.00E-11 | 3.77E-09 | TRUE |
| CADM1 | 8653 | 6.587218 | 4.48E-11 | 4.13E-09 | TRUE |
| SLC26A4-AS1 | 4789 | 6.576572 | 4.81E-11 | 4.36E-09 | TRUE |
| SIL1 | 13537 | 6.576189 | 4.83E-11 | 4.36E-09 | TRUE |
| F12 | 14200 | 6.556637 | 5.50E-11 | 4.86E-09 | TRUE |
| C2CD4C | 13992 | 6.550531 | 5.73E-11 | 5.01E-09 | TRUE |
| CASK | 4188 | 6.547344 | 5.86E-11 | 5.06E-09 | TRUE |
| WDR54 | 11097 | 6.543973 | 5.99E-11 | 5.15E-09 | TRUE |
| MDGA1 | 3068 | 6.532362 | 6.47E-11 | 5.47E-09 | TRUE |
| GSKIP | 11583 | 6.532133 | 6.48E-11 | 5.47E-09 | TRUE |
| KLK5 | 7336 | 6.523591 | 6.86E-11 | 5.72E-09 | TRUE |
| TUBB2A | 88 | 6.522856 | 6.90E-11 | 5.72E-09 | TRUE |
| TDRD9 | 11755 | 6.522609 | 6.91E-11 | 5.72E-09 | TRUE |
| DNAL4 | 4581 | 6.512508 | 7.39E-11 | 6.08E-09 | TRUE |
| KCNMB4 | 6419 | 6.510699 | 7.48E-11 | 6.12E-09 | TRUE |
| LMO3 | 14736 | 6.488789 | 8.65E-11 | 7.01E-09 | TRUE |
| RTBDN | 10518 | 6.478456 | 9.27E-11 | 7.43E-09 | TRUE |
| FABP6 | 14970 | 6.471521 | 9.70E-11 | 7.74E-09 | TRUE |
| SLC4A3 | 10789 | 6.460616 | 1.04E-10 | 8.27E-09 | TRUE |
| CSF1R | 13807 | 6.453437 | 1.09E-10 | 8.55E-09 | TRUE |
| SST | 3479 | 6.450768 | 1.11E-10 | 8.66E-09 | TRUE |
| SNRNP27 | 12924 | 6.43451 | 1.24E-10 | 9.49E-09 | TRUE |
| CD63 | 13955 | 6.432706 | 1.25E-10 | 9.53E-09 | TRUE |
| FABP7 | 14625 | 6.431634 | 1.26E-10 | 9.53E-09 | TRUE |
| RPL12 | 8062 | 6.39882 | 1.57E-10 | 1.13E-08 | TRUE |
| PRSS35 | 11445 | 6.393738 | 1.62E-10 | 1.16E-08 | TRUE |
| NR4A3 | 12612 | 6.384973 | 1.71E-10 | 1.22E-08 | TRUE |
| ERFE | 6575 | 6.348836 | 2.17E-10 | 1.53E-08 | TRUE |
| PCBD1 | 498 | 6.347946 | 2.18E-10 | 1.53E-08 | TRUE |
| KCNG1 | 5972 | 6.332769 | 2.41E-10 | 1.67E-08 | TRUE |
| YPEL1 | 15602 | 6.331528 | 2.43E-10 | 1.67E-08 | TRUE |
| DYNLL1 | 4070 | 6.328147 | 2.48E-10 | 1.70E-08 | TRUE |
| RNF181 | 1138 | 6.321206 | 2.60E-10 | 1.76E-08 | TRUE |
| NTSR1 | 11471 | 6.313978 | 2.72E-10 | 1.82E-08 | TRUE |
| LYPD8 | 7521 | 6.305442 | 2.87E-10 | 1.92E-08 | TRUE |
| RASL11B | 9048 | 6.29964 | 2.98E-10 | 1.98E-08 | TRUE |
| LINC02217 | 923 | 6.280155 | 3.38E-10 | 2.21E-08 | TRUE |
| LRRC3B | 656 | 6.256516 | 3.94E-10 | 2.54E-08 | TRUE |
| MMD | 13058 | 6.249887 | 4.11E-10 | 2.62E-08 | TRUE |
| HRASLS | 5785 | 6.226371 | 4.77E-10 | 3.02E-08 | TRUE |
| PNPLA3 | 5800 | 6.221975 | 4.91E-10 | 3.08E-08 | TRUE |
| IFT22 | 6584 | 6.221592 | 4.92E-10 | 3.08E-08 | TRUE |
| RPL29 | 13286 | 6.219943 | 4.97E-10 | 3.10E-08 | TRUE |
| VPS37D | 12919 | 6.215163 | 5.13E-10 | 3.16E-08 | TRUE |
| HACD4 | 14732 | 6.213579 | 5.18E-10 | 3.18E-08 | TRUE |
| FDPS | 14440 | 6.208358 | 5.35E-10 | 3.26E-08 | TRUE |
| C3orf14 | 3573 | 6.208147 | 5.36E-10 | 3.26E-08 | TRUE |
| HLA-DMB | 7924 | 6.202546 | 5.56E-10 | 3.37E-08 | TRUE |
| CIB1 | 14694 | 6.201331 | 5.60E-10 | 3.38E-08 | TRUE |
| HPCAL4 | 3143 | 6.195166 | 5.82E-10 | 3.50E-08 | TRUE |
| PLCB2 | 10139 | 6.180746 | 6.38E-10 | 3.78E-08 | TRUE |
| RAB36 | 8450 | 6.174656 | 6.63E-10 | 3.91E-08 | TRUE |
| DHRS2 | 416 | 6.16009 | 7.27E-10 | 4.25E-08 | TRUE |
| ALKAL2 | 14206 | 6.159024 | 7.32E-10 | 4.25E-08 | TRUE |
| KCTD12 | 12357 | 6.146763 | 7.91E-10 | 4.56E-08 | TRUE |
| PSORS1C1 | 5448 | 6.143298 | 8.08E-10 | 4.63E-08 | TRUE |
| CCT5 | 11327 | 6.133678 | 8.59E-10 | 4.90E-08 | TRUE |
| SORBS2 | 9050 | 6.122453 | 9.21E-10 | 5.22E-08 | TRUE |
| PGM2L1 | 14293 | 6.118575 | 9.44E-10 | 5.33E-08 | TRUE |
| DNAJA4 | 5270 | 6.114885 | 9.66E-10 | 5.40E-08 | TRUE |
| DKFZp779M0652 | 13583 | 6.114621 | 9.68E-10 | 5.40E-08 | TRUE |
| GABRB1 | 5973 | 6.10563 | 1.02E-09 | 5.64E-08 | TRUE |
| FARP1 | 12317 | 6.101876 | 1.05E-09 | 5.75E-08 | TRUE |
| GASAL1 | 6620 | 6.100348 | 1.06E-09 | 5.77E-08 | TRUE |
| LRRC42 | 1517 | 6.086918 | 1.15E-09 | 6.20E-08 | TRUE |
| ARHGDIG | 11001 | 6.083819 | 1.17E-09 | 6.30E-08 | TRUE |
| ARHGEF28 | 11654 | 6.078424 | 1.21E-09 | 6.48E-08 | TRUE |
| TAC1 | 12492 | 6.07351 | 1.25E-09 | 6.65E-08 | TRUE |
| STX17-AS1 | 10722 | 6.068262 | 1.29E-09 | 6.84E-08 | TRUE |
| IL13RA2 | 13674 | 6.068118 | 1.29E-09 | 6.84E-08 | TRUE |
| ZSCAN18 | 3692 | 6.062885 | 1.34E-09 | 7.01E-08 | TRUE |
| PPM1M | 3701 | 6.060247 | 1.36E-09 | 7.10E-08 | TRUE |
| S100Z | 6622 | 6.056962 | 1.39E-09 | 7.19E-08 | TRUE |
| CEBPA | 10486 | 6.047857 | 1.47E-09 | 7.52E-08 | TRUE |
| AMIGO2 | 9500 | 6.045793 | 1.49E-09 | 7.57E-08 | TRUE |
| NBDY | 14313 | 6.040507 | 1.54E-09 | 7.77E-08 | TRUE |
| CREM | 7792 | 6.039499 | 1.55E-09 | 7.77E-08 | TRUE |
| DAPL1 | 3607 | 6.036723 | 1.57E-09 | 7.88E-08 | TRUE |
| DIRAS3 | 9897 | 6.031837 | 1.62E-09 | 8.10E-08 | TRUE |
| DYRK3 | 7776 | 6.024694 | 1.69E-09 | 8.41E-08 | TRUE |
| NDUFA11 | 9849 | 6.023891 | 1.70E-09 | 8.42E-08 | TRUE |
| ELL3 | 6994 | 6.021439 | 1.73E-09 | 8.53E-08 | TRUE |
| PRSS23 | 4186 | 6.019792 | 1.75E-09 | 8.59E-08 | TRUE |
| RAP2B | 5120 | 6.016032 | 1.79E-09 | 8.73E-08 | TRUE |
| CCDC85C | 12399 | 6.011312 | 1.84E-09 | 8.96E-08 | TRUE |
| CRIP2 | 14341 | 6.009362 | 1.86E-09 | 9.04E-08 | TRUE |
| HES5 | 10314 | 6.005777 | 1.90E-09 | 9.22E-08 | TRUE |
| SP5 | 2358 | 5.998404 | 1.99E-09 | 9.56E-08 | TRUE |
| SH3BGR | 14093 | 5.994198 | 2.04E-09 | 9.78E-08 | TRUE |
| WNT10B | 4590 | 5.986431 | 2.14E-09 | 1.02E-07 | TRUE |
| SARNP | 1523 | 5.981179 | 2.22E-09 | 1.05E-07 | TRUE |
| PSME2 | 11347 | 5.978673 | 2.25E-09 | 1.06E-07 | TRUE |
| FZD1 | 4214 | 5.97634 | 2.28E-09 | 1.07E-07 | TRUE |
| DNAAF4 | 9569 | 5.975658 | 2.29E-09 | 1.07E-07 | TRUE |
| TUBA3D | 10115 | 5.963719 | 2.47E-09 | 1.15E-07 | TRUE |
| C8orf46 | 11051 | 5.950913 | 2.67E-09 | 1.23E-07 | TRUE |
| EFCAB1 | 12790 | 5.947832 | 2.72E-09 | 1.24E-07 | TRUE |
| CFD | 9032 | 5.944534 | 2.77E-09 | 1.26E-07 | TRUE |
| ENO1 | 126 | 5.925326 | 3.12E-09 | 1.40E-07 | TRUE |
| MAOB | 2780 | 5.917105 | 3.28E-09 | 1.47E-07 | TRUE |
| ATP5ME | 8777 | 5.912905 | 3.36E-09 | 1.50E-07 | TRUE |
| PCED1B | 4373 | 5.89792 | 3.68E-09 | 1.62E-07 | TRUE |
| MARCKS | 6398 | 5.896753 | 3.71E-09 | 1.63E-07 | TRUE |
| GLRA3 | 4885 | 5.894905 | 3.75E-09 | 1.64E-07 | TRUE |
| CENPVL3 | 4251 | 5.886855 | 3.94E-09 | 1.71E-07 | TRUE |
| MOB1B | 5118 | 5.877672 | 4.16E-09 | 1.80E-07 | TRUE |
| DHRS9 | 10866 | 5.871625 | 4.32E-09 | 1.86E-07 | TRUE |
| PSMB7 | 7093 | 5.868372 | 4.40E-09 | 1.89E-07 | TRUE |
| VWA5A | 13401 | 5.861816 | 4.58E-09 | 1.96E-07 | TRUE |
| RNF150 | 7508 | 5.844201 | 5.09E-09 | 2.13E-07 | TRUE |
| CALML3 | 2478 | 5.844165 | 5.09E-09 | 2.13E-07 | TRUE |
| TMEM130 | 2934 | 5.836386 | 5.33E-09 | 2.22E-07 | TRUE |
| GSS | 11930 | 5.835032 | 5.38E-09 | 2.23E-07 | TRUE |
| CRHBP | 12989 | 5.831653 | 5.49E-09 | 2.26E-07 | TRUE |
| KLK7 | 10101 | 5.828059 | 5.61E-09 | 2.30E-07 | TRUE |
| STK17A | 2121 | 5.826658 | 5.65E-09 | 2.31E-07 | TRUE |
| FAR2P1 | 11914 | 5.819599 | 5.90E-09 | 2.41E-07 | TRUE |
| SLC8A2 | 2123 | 5.814816 | 6.07E-09 | 2.47E-07 | TRUE |
| MESP1 | 14254 | 5.814287 | 6.09E-09 | 2.47E-07 | TRUE |
| GLRA2 | 10858 | 5.802736 | 6.52E-09 | 2.63E-07 | TRUE |
| DOK6 | 5086 | 5.799313 | 6.66E-09 | 2.68E-07 | TRUE |
| NAGK | 8180 | 5.79893 | 6.67E-09 | 2.68E-07 | TRUE |
| C1QB | 9047 | 5.798379 | 6.70E-09 | 2.68E-07 | TRUE |
| FOXF2 | 7506 | 5.793982 | 6.87E-09 | 2.74E-07 | TRUE |
| NBAS | 11464 | 5.78599 | 7.21E-09 | 2.86E-07 | TRUE |
| CPLX3 | 10594 | 5.780559 | 7.45E-09 | 2.94E-07 | TRUE |
| NUDT16L1 | 14192 | 5.779918 | 7.47E-09 | 2.94E-07 | TRUE |
| ATP6V1C1 | 5348 | 5.772312 | 7.82E-09 | 3.05E-07 | TRUE |
| PRDX4 | 11482 | 5.769238 | 7.96E-09 | 3.09E-07 | TRUE |
| DNAH2 | 7793 | 5.767014 | 8.07E-09 | 3.12E-07 | TRUE |
| RPL36 | 12439 | 5.763522 | 8.24E-09 | 3.18E-07 | TRUE |
| MYL12B | 2417 | 5.758058 | 8.51E-09 | 3.28E-07 | TRUE |
| PPP1R17 | 4422 | 5.754243 | 8.70E-09 | 3.30E-07 | TRUE |
| NUDT11 | 9664 | 5.754153 | 8.71E-09 | 3.30E-07 | TRUE |
| GUK1 | 14805 | 5.753243 | 8.75E-09 | 3.31E-07 | TRUE |
| LOC105372480 | 1056 | 5.747606 | 9.05E-09 | 3.40E-07 | TRUE |
| ARPC2 | 2760 | 5.746147 | 9.13E-09 | 3.42E-07 | TRUE |
| KLHDC8B | 11952 | 5.741588 | 9.38E-09 | 3.50E-07 | TRUE |
| FAM181A | 4673 | 5.727449 | 1.02E-08 | 3.78E-07 | TRUE |
| PEA15 | 11141 | 5.726528 | 1.03E-08 | 3.79E-07 | TRUE |
| SYNPR | 826 | 5.723027 | 1.05E-08 | 3.85E-07 | TRUE |
| SLC35F2 | 8230 | 5.72192 | 1.05E-08 | 3.87E-07 | TRUE |
| NELFE | 7305 | 5.71873 | 1.07E-08 | 3.93E-07 | TRUE |
| CACNA1H | 12146 | 5.714863 | 1.10E-08 | 4.00E-07 | TRUE |
| PCDH17 | 12643 | 5.713855 | 1.10E-08 | 4.02E-07 | TRUE |
| KLK10 | 5801 | 5.710822 | 1.12E-08 | 4.08E-07 | TRUE |
| RER1 | 14821 | 5.708844 | 1.14E-08 | 4.11E-07 | TRUE |
| PMVK | 11212 | 5.708382 | 1.14E-08 | 4.11E-07 | TRUE |
| GMIP | 4633 | 5.708223 | 1.14E-08 | 4.11E-07 | TRUE |
| TM2D3 | 4424 | 5.697484 | 1.22E-08 | 4.34E-07 | TRUE |
| RTP1 | 4185 | 5.696356 | 1.22E-08 | 4.36E-07 | TRUE |
| RTN4RL2 | 11295 | 5.691853 | 1.26E-08 | 4.45E-07 | TRUE |
| PEX19 | 7270 | 5.689725 | 1.27E-08 | 4.49E-07 | TRUE |
| PDE2A | 12192 | 5.687469 | 1.29E-08 | 4.51E-07 | TRUE |
| PCSK5 | 10880 | 5.680411 | 1.34E-08 | 4.69E-07 | TRUE |
| TXN | 7690 | 5.680079 | 1.35E-08 | 4.69E-07 | TRUE |
| MKL2 | 47 | 5.674264 | 1.39E-08 | 4.82E-07 | TRUE |
| LOC440434 | 12773 | 5.664323 | 1.48E-08 | 5.09E-07 | TRUE |
| KCNN3 | 2063 | 5.656118 | 1.55E-08 | 5.32E-07 | TRUE |
| GALNT14 | 10291 | 5.653859 | 1.57E-08 | 5.37E-07 | TRUE |
| STX1A | 1577 | 5.649935 | 1.61E-08 | 5.45E-07 | TRUE |
| SMIM29 | 8781 | 5.644471 | 1.66E-08 | 5.62E-07 | TRUE |
| SNHG8 | 11119 | 5.643305 | 1.67E-08 | 5.64E-07 | TRUE |
| SLIT3 | 4191 | 5.642389 | 1.68E-08 | 5.66E-07 | TRUE |
| NANOS1 | 11380 | 5.641509 | 1.69E-08 | 5.66E-07 | TRUE |
| NUDT4 | 4803 | 5.636933 | 1.73E-08 | 5.78E-07 | TRUE |
| FAM46A | 476 | 5.63372 | 1.76E-08 | 5.85E-07 | TRUE |
| LINC02482 | 10493 | 5.633311 | 1.77E-08 | 5.86E-07 | TRUE |
| RPL41 | 10566 | 5.628737 | 1.82E-08 | 5.98E-07 | TRUE |
| SEMA4F | 9173 | 5.621157 | 1.90E-08 | 6.19E-07 | TRUE |
| LST1 | 13632 | 5.616427 | 1.95E-08 | 6.33E-07 | TRUE |
| LINC00484 | 13556 | 5.616225 | 1.95E-08 | 6.33E-07 | TRUE |
| FAM120AOS | 5059 | 5.613387 | 1.98E-08 | 6.42E-07 | TRUE |
| RPA2 | 9309 | 5.612909 | 1.99E-08 | 6.43E-07 | TRUE |
| DTNBP1 | 9562 | 5.607611 | 2.05E-08 | 6.60E-07 | TRUE |
| NUTF2 | 12164 | 5.601118 | 2.13E-08 | 6.79E-07 | TRUE |
| FAM107A | 12382 | 5.591586 | 2.25E-08 | 7.08E-07 | TRUE |
| PIRT | 11981 | 5.587668 | 2.30E-08 | 7.22E-07 | TRUE |
| TSTD1 | 9989 | 5.586023 | 2.32E-08 | 7.28E-07 | TRUE |
| ADRB2 | 7936 | 5.585608 | 2.33E-08 | 7.28E-07 | TRUE |
| PTS | 5377 | 5.583602 | 2.36E-08 | 7.34E-07 | TRUE |
| BIRC3 | 3078 | 5.578817 | 2.42E-08 | 7.50E-07 | TRUE |
| ANKRD6 | 7846 | 5.575992 | 2.46E-08 | 7.59E-07 | TRUE |
| MRPL23 | 11137 | 5.565986 | 2.61E-08 | 7.96E-07 | TRUE |
| C12orf45 | 4225 | 5.565714 | 2.61E-08 | 7.96E-07 | TRUE |
| UBA52 | 12898 | 5.561421 | 2.68E-08 | 8.14E-07 | TRUE |
| RNASEH2A | 13198 | 5.55856 | 2.72E-08 | 8.24E-07 | TRUE |
| RIC8A | 10657 | 5.551244 | 2.84E-08 | 8.58E-07 | TRUE |
| DDAH2 | 12328 | 5.550356 | 2.85E-08 | 8.59E-07 | TRUE |
| DUSP26 | 14110 | 5.550304 | 2.85E-08 | 8.59E-07 | TRUE |
| TYROBP | 10354 | 5.5443 | 2.95E-08 | 8.83E-07 | TRUE |
| LINC00958 | 1951 | 5.54413 | 2.95E-08 | 8.83E-07 | TRUE |
| EIPR1 | 1296 | 5.543888 | 2.96E-08 | 8.83E-07 | TRUE |
| TCEA3 | 10031 | 5.542068 | 2.99E-08 | 8.90E-07 | TRUE |
| SOWAHA | 14603 | 5.539824 | 3.03E-08 | 8.98E-07 | TRUE |
| MSANTD1 | 8241 | 5.533913 | 3.13E-08 | 9.27E-07 | TRUE |
| PSTPIP1 | 3355 | 5.533197 | 3.14E-08 | 9.29E-07 | TRUE |
| CCDC90B | 11116 | 5.532786 | 3.15E-08 | 9.30E-07 | TRUE |
| LSM3 | 12136 | 5.524886 | 3.30E-08 | 9.66E-07 | TRUE |
| PLPPR4 | 6292 | 5.509712 | 3.59E-08 | 1.04E-06 | TRUE |
| NUDT18 | 13595 | 5.507171 | 3.65E-08 | 1.05E-06 | TRUE |
| BYSL | 9094 | 5.505846 | 3.67E-08 | 1.06E-06 | TRUE |
| MANF | 11523 | 5.500769 | 3.78E-08 | 1.08E-06 | TRUE |
| ME1 | 2579 | 5.494547 | 3.92E-08 | 1.12E-06 | TRUE |
| DERL2 | 14107 | 5.493938 | 3.93E-08 | 1.12E-06 | TRUE |
| COCH | 4544 | 5.493845 | 3.93E-08 | 1.12E-06 | TRUE |
| LINC01094 | 136 | 5.493683 | 3.94E-08 | 1.12E-06 | TRUE |
| HRK | 5062 | 5.492354 | 3.97E-08 | 1.12E-06 | TRUE |
| LRRN2 | 6917 | 5.490877 | 4.00E-08 | 1.13E-06 | TRUE |
| RTL8C | 686 | 5.489021 | 4.04E-08 | 1.14E-06 | TRUE |
| POLR2G | 10044 | 5.487983 | 4.07E-08 | 1.14E-06 | TRUE |
| KCNA3 | 6403 | 5.465786 | 4.61E-08 | 1.29E-06 | TRUE |
| CCDC24 | 1842 | 5.451048 | 5.01E-08 | 1.38E-06 | TRUE |
| NFKBIE | 9549 | 5.447085 | 5.12E-08 | 1.41E-06 | TRUE |
| SULF1 | 5394 | 5.44048 | 5.31E-08 | 1.45E-06 | TRUE |
| NUDT14 | 12394 | 5.437208 | 5.41E-08 | 1.48E-06 | TRUE |
| MSRB2 | 3177 | 5.437042 | 5.42E-08 | 1.48E-06 | TRUE |
| GPR34 | 12591 | 5.432318 | 5.56E-08 | 1.50E-06 | TRUE |
| TMEM132A | 7285 | 5.43064 | 5.62E-08 | 1.52E-06 | TRUE |
| HCST | 13429 | 5.429632 | 5.65E-08 | 1.52E-06 | TRUE |
| GOLM1 | 12966 | 5.423134 | 5.86E-08 | 1.57E-06 | TRUE |
| NUDT10 | 3472 | 5.407817 | 6.38E-08 | 1.71E-06 | TRUE |
| TSPAN33 | 7080 | 5.40762 | 6.39E-08 | 1.71E-06 | TRUE |
| TMEM263 | 10352 | 5.402344 | 6.58E-08 | 1.75E-06 | TRUE |
| TCF7L1 | 14549 | 5.395855 | 6.82E-08 | 1.81E-06 | TRUE |
| UNC5B-AS1 | 13535 | 5.383584 | 7.30E-08 | 1.92E-06 | TRUE |
| NIT2 | 8400 | 5.382536 | 7.34E-08 | 1.93E-06 | TRUE |
| GPRIN2 | 2288 | 5.382266 | 7.36E-08 | 1.93E-06 | TRUE |
| CD99 | 14706 | 5.382098 | 7.36E-08 | 1.93E-06 | TRUE |
| PAFAH1B3 | 7294 | 5.37919 | 7.48E-08 | 1.95E-06 | TRUE |
| ATOH7 | 6049 | 5.373136 | 7.74E-08 | 2.01E-06 | TRUE |
| ADAM28 | 8499 | 5.364923 | 8.10E-08 | 2.10E-06 | TRUE |
| DYNC1I1 | 13916 | 5.364153 | 8.13E-08 | 2.10E-06 | TRUE |
| UCHL3 | 6359 | 5.36278 | 8.20E-08 | 2.11E-06 | TRUE |
| CD74 | 8396 | 5.360377 | 8.30E-08 | 2.13E-06 | TRUE |
| RGS18 | 9905 | 5.358279 | 8.40E-08 | 2.15E-06 | TRUE |
| TOMM6 | 11465 | 5.355359 | 8.54E-08 | 2.18E-06 | TRUE |
| MYL6 | 1518 | 5.351601 | 8.72E-08 | 2.22E-06 | TRUE |
| HSPB3 | 15560 | 5.348956 | 8.85E-08 | 2.24E-06 | TRUE |
| CLPP | 8475 | 5.344059 | 9.09E-08 | 2.29E-06 | TRUE |
| STOML1 | 8166 | 5.343402 | 9.12E-08 | 2.30E-06 | TRUE |
| TMEM108 | 14132 | 5.343206 | 9.13E-08 | 2.30E-06 | TRUE |
| CCDC167 | 12943 | 5.340346 | 9.28E-08 | 2.32E-06 | TRUE |
| LAMTOR4 | 10484 | 5.333431 | 9.64E-08 | 2.40E-06 | TRUE |
| TFPT | 8783 | 5.330121 | 9.81E-08 | 2.44E-06 | TRUE |
| C1S | 4139 | 5.329368 | 9.86E-08 | 2.44E-06 | TRUE |
| FILIP1 | 10453 | 5.326646 | 1.00E-07 | 2.47E-06 | TRUE |
| MGST1 | 8928 | 5.326159 | 1.00E-07 | 2.47E-06 | TRUE |
| LRRC73 | 9869 | 5.321854 | 1.03E-07 | 2.52E-06 | TRUE |
| ARL3 | 5567 | 5.317899 | 1.05E-07 | 2.56E-06 | TRUE |
| KRT17 | 226 | 5.314444 | 1.07E-07 | 2.61E-06 | TRUE |
| PDE4DIP | 13775 | 5.306321 | 1.12E-07 | 2.72E-06 | TRUE |
| KLF6 | 2477 | 5.296757 | 1.18E-07 | 2.85E-06 | TRUE |
| GXYLT2 | 655 | 5.292584 | 1.21E-07 | 2.91E-06 | TRUE |
| RSPO3 | 670 | 5.292258 | 1.21E-07 | 2.91E-06 | TRUE |
| PLAC9 | 13349 | 5.281362 | 1.28E-07 | 3.07E-06 | TRUE |
| PPP1R32 | 14659 | 5.279578 | 1.29E-07 | 3.10E-06 | TRUE |
| MYO16 | 704 | 5.271689 | 1.35E-07 | 3.22E-06 | TRUE |
| CHST9 | 11900 | 5.264006 | 1.41E-07 | 3.33E-06 | TRUE |
| UCHL1 | 2372 | 5.258915 | 1.45E-07 | 3.42E-06 | TRUE |
| CFAP52 | 7974 | 5.255889 | 1.47E-07 | 3.47E-06 | TRUE |
| TNFAIP8L2 | 773 | 5.254347 | 1.49E-07 | 3.49E-06 | TRUE |
| DNAH5 | 898 | 5.252137 | 1.50E-07 | 3.52E-06 | TRUE |
| SHISA9 | 12176 | 5.245724 | 1.56E-07 | 3.63E-06 | TRUE |
| ALPL | 475 | 5.236607 | 1.64E-07 | 3.78E-06 | TRUE |
| MPV17L2 | 12041 | 5.226964 | 1.72E-07 | 3.97E-06 | TRUE |
| BCL2A1 | 12946 | 5.221717 | 1.77E-07 | 4.06E-06 | TRUE |
| RNASET2 | 8840 | 5.22162 | 1.77E-07 | 4.06E-06 | TRUE |
| VSTM2L | 7169 | 5.217039 | 1.82E-07 | 4.15E-06 | TRUE |
| MTCH1 | 3640 | 5.210986 | 1.88E-07 | 4.27E-06 | TRUE |
| TMEM42 | 5804 | 5.209558 | 1.89E-07 | 4.29E-06 | TRUE |
| HMGN2 | 3928 | 5.203705 | 1.95E-07 | 4.41E-06 | TRUE |
| NCAM2 | 862 | 5.196563 | 2.03E-07 | 4.56E-06 | TRUE |
| CHCHD6 | 1019 | 5.192124 | 2.08E-07 | 4.66E-06 | TRUE |
| ZPR1 | 5211 | 5.189355 | 2.11E-07 | 4.71E-06 | TRUE |
| FOXRED2 | 3581 | 5.186734 | 2.14E-07 | 4.76E-06 | TRUE |
| DHX58 | 13806 | 5.184209 | 2.17E-07 | 4.81E-06 | TRUE |
| ARHGAP4 | 3683 | 5.179728 | 2.22E-07 | 4.91E-06 | TRUE |
| APBB1IP | 442 | 5.176874 | 2.26E-07 | 4.98E-06 | TRUE |
| ETNPPL | 13830 | 5.173222 | 2.30E-07 | 5.07E-06 | TRUE |
| SUSD3 | 10167 | 5.171708 | 2.32E-07 | 5.11E-06 | TRUE |
| FAM86C1 | 2233 | 5.170091 | 2.34E-07 | 5.14E-06 | TRUE |
| FDXR | 12603 | 5.167057 | 2.38E-07 | 5.22E-06 | TRUE |
| TRPV2 | 14111 | 5.165315 | 2.40E-07 | 5.26E-06 | TRUE |
| GTF2F2 | 4062 | 5.158254 | 2.49E-07 | 5.42E-06 | TRUE |
| HEBP1 | 11325 | 5.15541 | 2.53E-07 | 5.48E-06 | TRUE |
| ZC3HAV1 | 14443 | 5.144593 | 2.68E-07 | 5.78E-06 | TRUE |
| POMGNT2 | 14212 | 5.143565 | 2.70E-07 | 5.80E-06 | TRUE |
| PET100 | 1215 | 5.13914 | 2.76E-07 | 5.91E-06 | TRUE |
| TRIB3 | 3083 | 5.131509 | 2.87E-07 | 6.14E-06 | TRUE |
| THTPA | 6944 | 5.11871 | 3.08E-07 | 6.52E-06 | TRUE |
| EMG1 | 4603 | 5.112002 | 3.19E-07 | 6.72E-06 | TRUE |
| NKAIN2 | 7611 | 5.088095 | 3.62E-07 | 7.51E-06 | TRUE |
| PTPRA | 914 | 5.085716 | 3.66E-07 | 7.58E-06 | TRUE |
| ACSL4 | 11667 | 5.085123 | 3.67E-07 | 7.60E-06 | TRUE |
| TKFC | 8451 | 5.083849 | 3.70E-07 | 7.64E-06 | TRUE |
| BCRP2 | 1 | 5.082974 | 3.72E-07 | 7.66E-06 | TRUE |
| MCUR1 | 1772 | 5.080045 | 3.77E-07 | 7.77E-06 | TRUE |
| CCDC189 | 10717 | 5.069388 | 3.99E-07 | 8.18E-06 | TRUE |
| ADAP2 | 10205 | 5.064034 | 4.10E-07 | 8.38E-06 | TRUE |
| TUBB8P12 | 12283 | 5.062599 | 4.14E-07 | 8.42E-06 | TRUE |
| HTR7P1 | 3388 | 5.061767 | 4.15E-07 | 8.43E-06 | TRUE |
| B9D1 | 7139 | 5.056456 | 4.27E-07 | 8.63E-06 | TRUE |
| HRAS | 1570 | 5.056066 | 4.28E-07 | 8.63E-06 | TRUE |
| ZFHX3 | 2687 | 5.054913 | 4.31E-07 | 8.66E-06 | TRUE |
| DCTPP1 | 2117 | 5.053513 | 4.34E-07 | 8.70E-06 | TRUE |
| CNTN3 | 11082 | 5.052654 | 4.36E-07 | 8.71E-06 | TRUE |
| NKAIN3 | 4831 | 5.050223 | 4.41E-07 | 8.80E-06 | TRUE |
| CORO1A | 4284 | 5.048708 | 4.45E-07 | 8.86E-06 | TRUE |
| LAMTOR2 | 10429 | 5.042036 | 4.61E-07 | 9.16E-06 | TRUE |
| ARHGEF40 | 7295 | 5.038001 | 4.70E-07 | 9.31E-06 | TRUE |
| HYI | 3227 | 5.035861 | 4.76E-07 | 9.38E-06 | TRUE |
| PRAF2 | 14280 | 5.033207 | 4.82E-07 | 9.47E-06 | TRUE |
| AKR7A3 | 4345 | 5.032086 | 4.85E-07 | 9.50E-06 | TRUE |
| THRA | 5871 | 5.032027 | 4.85E-07 | 9.50E-06 | TRUE |
| LRRC56 | 13753 | 5.028565 | 4.94E-07 | 9.63E-06 | TRUE |
| TPM3 | 12788 | 5.020347 | 5.16E-07 | 9.97E-06 | TRUE |
| LYN | 1205 | 5.020203 | 5.16E-07 | 9.97E-06 | TRUE |
| ACOT2 | 1760 | 5.013993 | 5.33E-07 | 1.03E-05 | TRUE |
| CMSS1 | 8489 | 5.010715 | 5.42E-07 | 1.04E-05 | TRUE |
| COL21A1 | 5087 | 5.006488 | 5.54E-07 | 1.06E-05 | TRUE |
| PLB1 | 2264 | 5.005115 | 5.58E-07 | 1.07E-05 | TRUE |
| TMEM176B | 8125 | 4.999825 | 5.74E-07 | 1.09E-05 | TRUE |
| ZCCHC18 | 9463 | 4.998693 | 5.77E-07 | 1.09E-05 | TRUE |
| GRP | 14211 | 4.997658 | 5.80E-07 | 1.10E-05 | TRUE |
| BEX1 | 4714 | 4.988485 | 6.09E-07 | 1.15E-05 | TRUE |
| AP3M2 | 13703 | 4.988127 | 6.10E-07 | 1.15E-05 | TRUE |
| RPS4X | 7053 | 4.98206 | 6.29E-07 | 1.18E-05 | TRUE |
| APOPT1 | 415 | 4.978032 | 6.42E-07 | 1.20E-05 | TRUE |
| CHID1 | 10549 | 4.975271 | 6.52E-07 | 1.21E-05 | TRUE |
| SWI5 | 1165 | 4.975143 | 6.52E-07 | 1.21E-05 | TRUE |
| AKR7A2 | 8529 | 4.974757 | 6.53E-07 | 1.22E-05 | TRUE |
| B3GALNT1 | 3668 | 4.971192 | 6.65E-07 | 1.23E-05 | TRUE |
| PCDH19 | 11163 | 4.968766 | 6.74E-07 | 1.24E-05 | TRUE |
| ATP2C2 | 14188 | 4.966559 | 6.82E-07 | 1.26E-05 | TRUE |
| ANGPT1 | 927 | 4.961287 | 7.00E-07 | 1.28E-05 | TRUE |
| PRDX6 | 9651 | 4.959353 | 7.07E-07 | 1.29E-05 | TRUE |
| TRAF3IP3 | 3471 | 4.958345 | 7.11E-07 | 1.30E-05 | TRUE |
| VAT1L | 3453 | 4.956811 | 7.17E-07 | 1.31E-05 | TRUE |
| MYL5 | 12584 | 4.956422 | 7.18E-07 | 1.31E-05 | TRUE |
| GLDC | 8032 | 4.956417 | 7.18E-07 | 1.31E-05 | TRUE |
| TUNAR | 3159 | 4.949063 | 7.46E-07 | 1.35E-05 | TRUE |
| GLT8D2 | 11345 | 4.946037 | 7.57E-07 | 1.37E-05 | TRUE |
| ADGRB3 | 9909 | 4.938182 | 7.89E-07 | 1.42E-05 | TRUE |
| ME3 | 11374 | 4.932547 | 8.12E-07 | 1.46E-05 | TRUE |
| PUSL1 | 12481 | 4.932364 | 8.12E-07 | 1.46E-05 | TRUE |
| PPEF1 | 10553 | 4.929959 | 8.22E-07 | 1.47E-05 | TRUE |
| POLE4 | 10081 | 4.924967 | 8.44E-07 | 1.51E-05 | TRUE |
| RFK | 3743 | 4.922497 | 8.54E-07 | 1.52E-05 | TRUE |
| FREM3 | 10472 | 4.922316 | 8.55E-07 | 1.52E-05 | TRUE |
| ARPP19 | 11057 | 4.919865 | 8.66E-07 | 1.54E-05 | TRUE |
| LYPD1 | 1463 | 4.917486 | 8.77E-07 | 1.55E-05 | TRUE |
| FUOM | 11863 | 4.915411 | 8.86E-07 | 1.56E-05 | TRUE |
| FABP5 | 3965 | 4.913541 | 8.94E-07 | 1.58E-05 | TRUE |
| GNG2 | 12732 | 4.909821 | 9.12E-07 | 1.60E-05 | TRUE |
| GRIA1 | 1608 | 4.9093 | 9.14E-07 | 1.61E-05 | TRUE |
| SOX11 | 8402 | 4.90715 | 9.24E-07 | 1.62E-05 | TRUE |
| CDH9 | 7114 | 4.905975 | 9.30E-07 | 1.63E-05 | TRUE |
| MYH15 | 15170 | 4.903961 | 9.39E-07 | 1.64E-05 | TRUE |
| GDPD2 | 14845 | 4.903593 | 9.41E-07 | 1.64E-05 | TRUE |
| RPS18 | 3467 | 4.898548 | 9.65E-07 | 1.67E-05 | TRUE |
| DYDC2 | 5485 | 4.898122 | 9.68E-07 | 1.67E-05 | TRUE |
| FKBP1B | 2231 | 4.894596 | 9.85E-07 | 1.70E-05 | TRUE |
| GOLT1A | 210 | 4.885129 | 1.03E-06 | 1.77E-05 | TRUE |
| C2orf80 | 1144 | 4.88107 | 1.06E-06 | 1.80E-05 | TRUE |
| ADAMTS9 | 5569 | 4.880714 | 1.06E-06 | 1.80E-05 | TRUE |
| TAF10 | 4521 | 4.876922 | 1.08E-06 | 1.83E-05 | TRUE |
| BRK1 | 7854 | 4.875475 | 1.09E-06 | 1.84E-05 | TRUE |
| DCAF11 | 2466 | 4.872915 | 1.10E-06 | 1.86E-05 | TRUE |
| SIDT2 | 14269 | 4.869096 | 1.12E-06 | 1.89E-05 | TRUE |
| SHF | 8371 | 4.868841 | 1.12E-06 | 1.89E-05 | TRUE |
| FZD8 | 13044 | 4.861491 | 1.17E-06 | 1.96E-05 | TRUE |
| DDRGK1 | 1241 | 4.860699 | 1.17E-06 | 1.96E-05 | TRUE |
| CABYR | 2421 | 4.859761 | 1.18E-06 | 1.97E-05 | TRUE |
| SLC17A8 | 14644 | 4.85918 | 1.18E-06 | 1.97E-05 | TRUE |
| JUN | 2844 | 4.857276 | 1.19E-06 | 1.99E-05 | TRUE |
| PLD6 | 11860 | 4.849026 | 1.24E-06 | 2.07E-05 | TRUE |
| TNIK | 6666 | 4.847565 | 1.25E-06 | 2.07E-05 | TRUE |
| STYK1 | 4312 | 4.845619 | 1.26E-06 | 2.09E-05 | TRUE |
| ST5 | 4930 | 4.832422 | 1.35E-06 | 2.21E-05 | TRUE |
| DIO2 | 1741 | 4.829456 | 1.37E-06 | 2.24E-05 | TRUE |
| RHOC | 10826 | 4.828458 | 1.38E-06 | 2.25E-05 | TRUE |
| TMEFF2 | 11562 | 4.826178 | 1.39E-06 | 2.27E-05 | TRUE |
| VWCE | 7901 | 4.824395 | 1.40E-06 | 2.28E-05 | TRUE |
| PTPRR | 701 | 4.824197 | 1.41E-06 | 2.28E-05 | TRUE |
| SRRD | 2007 | 4.824123 | 1.41E-06 | 2.28E-05 | TRUE |
| MAGED1 | 12785 | 4.819887 | 1.44E-06 | 2.32E-05 | TRUE |
| CYTH4 | 8602 | 4.81984 | 1.44E-06 | 2.32E-05 | TRUE |
| LOC642852 | 3521 | 4.814998 | 1.47E-06 | 2.37E-05 | TRUE |
| SEM1 | 176 | 4.80616 | 1.54E-06 | 2.47E-05 | TRUE |
| MGMT | 9057 | 4.803877 | 1.56E-06 | 2.49E-05 | TRUE |
| FAM241B | 8388 | 4.803787 | 1.56E-06 | 2.49E-05 | TRUE |
| MZT2B | 6830 | 4.801824 | 1.57E-06 | 2.51E-05 | TRUE |
| FAM149A | 5881 | 4.799539 | 1.59E-06 | 2.54E-05 | TRUE |
| TSPAN4 | 841 | 4.795985 | 1.62E-06 | 2.57E-05 | TRUE |
| SAMD9 | 2822 | 4.7947 | 1.63E-06 | 2.58E-05 | TRUE |
| PRRX1 | 6979 | 4.791022 | 1.66E-06 | 2.63E-05 | TRUE |
| SUMF1 | 12960 | 4.790111 | 1.67E-06 | 2.63E-05 | TRUE |
| LOC100129291 | 2301 | 4.777684 | 1.77E-06 | 2.78E-05 | TRUE |
| ST20 | 13890 | 4.77614 | 1.79E-06 | 2.80E-05 | TRUE |
| CNTN6 | 11278 | 4.772378 | 1.82E-06 | 2.84E-05 | TRUE |
| ANO3 | 13077 | 4.76818 | 1.86E-06 | 2.89E-05 | TRUE |
| PYGL | 4934 | 4.767986 | 1.86E-06 | 2.89E-05 | TRUE |
| FBXW9 | 3260 | 4.765027 | 1.89E-06 | 2.92E-05 | TRUE |
| ACKR3 | 5583 | 4.7633 | 1.90E-06 | 2.94E-05 | TRUE |
| CPT1C | 5061 | 4.761864 | 1.92E-06 | 2.95E-05 | TRUE |
| PLXDC2 | 10602 | 4.760198 | 1.93E-06 | 2.98E-05 | TRUE |
| ADCY2 | 14592 | 4.755161 | 1.98E-06 | 3.04E-05 | TRUE |
| MZT2A | 14084 | 4.747525 | 2.06E-06 | 3.12E-05 | TRUE |
| TUFM | 5784 | 4.741952 | 2.12E-06 | 3.19E-05 | TRUE |
| PRDX1 | 12483 | 4.741354 | 2.12E-06 | 3.20E-05 | TRUE |
| LOC100288911 | 1721 | 4.74077 | 2.13E-06 | 3.21E-05 | TRUE |
| LDHD | 5260 | 4.740468 | 2.13E-06 | 3.21E-05 | TRUE |
| RILPL2 | 5942 | 4.740181 | 2.14E-06 | 3.21E-05 | TRUE |
| CAPG | 2580 | 4.739015 | 2.15E-06 | 3.22E-05 | TRUE |
| MAPK1 | 13828 | 4.73638 | 2.18E-06 | 3.25E-05 | TRUE |
| RPS14 | 3049 | 4.735987 | 2.18E-06 | 3.26E-05 | TRUE |
| RAB3GAP1 | 12180 | 4.734742 | 2.19E-06 | 3.27E-05 | TRUE |
| SYNGR3 | 10420 | 4.734457 | 2.20E-06 | 3.27E-05 | TRUE |
| TRIM22 | 7939 | 4.731293 | 2.23E-06 | 3.32E-05 | TRUE |
| JMJD4 | 9096 | 4.730778 | 2.24E-06 | 3.32E-05 | TRUE |
| ENOX1 | 166 | 4.728118 | 2.27E-06 | 3.35E-05 | TRUE |
| TSPAN6 | 9122 | 4.727356 | 2.27E-06 | 3.36E-05 | TRUE |
| SLC16A2 | 2830 | 4.723805 | 2.31E-06 | 3.42E-05 | TRUE |
| GLOD4 | 13634 | 4.722762 | 2.33E-06 | 3.43E-05 | TRUE |
| LOC728392 | 14228 | 4.722507 | 2.33E-06 | 3.43E-05 | TRUE |
| PRKCG | 3935 | 4.719439 | 2.36E-06 | 3.46E-05 | TRUE |
| RNF7 | 11470 | 4.718522 | 2.38E-06 | 3.47E-05 | TRUE |
| SLC35A2 | 4802 | 4.718451 | 2.38E-06 | 3.47E-05 | TRUE |
| WDR6 | 2169 | 4.713904 | 2.43E-06 | 3.54E-05 | TRUE |
| MYH7 | 7790 | 4.713445 | 2.44E-06 | 3.54E-05 | TRUE |
| RAET1E-AS1 | 9789 | 4.711463 | 2.46E-06 | 3.57E-05 | TRUE |
| LRP1B | 11067 | 4.709916 | 2.48E-06 | 3.59E-05 | TRUE |
| STK25 | 8172 | 4.709558 | 2.48E-06 | 3.59E-05 | TRUE |
| SNX7 | 14785 | 4.707578 | 2.51E-06 | 3.62E-05 | TRUE |
| ADRA1B | 6486 | 4.704208 | 2.55E-06 | 3.67E-05 | TRUE |
| CPNE5 | 9898 | 4.702929 | 2.56E-06 | 3.68E-05 | TRUE |
| ALDH9A1 | 13758 | 4.696293 | 2.65E-06 | 3.79E-05 | TRUE |
| ASH2L | 5191 | 4.694647 | 2.67E-06 | 3.82E-05 | TRUE |
| PLAC9P1 | 3780 | 4.694288 | 2.68E-06 | 3.82E-05 | TRUE |
| TENM3 | 12556 | 4.693871 | 2.68E-06 | 3.82E-05 | TRUE |
| COMTD1 | 5159 | 4.693837 | 2.68E-06 | 3.82E-05 | TRUE |
| TMTC1 | 1664 | 4.688209 | 2.76E-06 | 3.91E-05 | TRUE |
| ANXA1 | 2749 | 4.687527 | 2.77E-06 | 3.92E-05 | TRUE |
| ACTG1 | 6583 | 4.684206 | 2.81E-06 | 3.98E-05 | TRUE |
| JPT1 | 10554 | 4.683511 | 2.82E-06 | 3.99E-05 | TRUE |
| SELENOW | 8089 | 4.679536 | 2.88E-06 | 4.05E-05 | TRUE |
| PERM1 | 13183 | 4.678169 | 2.89E-06 | 4.07E-05 | TRUE |
| B3GNT4 | 10543 | 4.677867 | 2.90E-06 | 4.08E-05 | TRUE |
| TMEM17 | 11483 | 4.677305 | 2.91E-06 | 4.08E-05 | TRUE |
| ALOX5 | 8271 | 4.677049 | 2.91E-06 | 4.08E-05 | TRUE |
| COPS6 | 1580 | 4.676564 | 2.92E-06 | 4.09E-05 | TRUE |
| PYCR3 | 1242 | 4.672563 | 2.97E-06 | 4.16E-05 | TRUE |
| RASL10A | 1357 | 4.671579 | 2.99E-06 | 4.18E-05 | TRUE |
| RTN4RL1 | 13908 | 4.670637 | 3.00E-06 | 4.19E-05 | TRUE |
| SUSD1 | 3476 | 4.669461 | 3.02E-06 | 4.21E-05 | TRUE |
| WDR66 | 7400 | 4.666979 | 3.06E-06 | 4.26E-05 | TRUE |
| C22orf39 | 3575 | 4.666629 | 3.06E-06 | 4.26E-05 | TRUE |
| B3GNT5 | 4541 | 4.663212 | 3.11E-06 | 4.32E-05 | TRUE |
| TBC1D24 | 2050 | 4.658994 | 3.18E-06 | 4.39E-05 | TRUE |
| ST6GALNAC6 | 7321 | 4.658654 | 3.18E-06 | 4.40E-05 | TRUE |
| NUPR1 | 9826 | 4.658385 | 3.19E-06 | 4.40E-05 | TRUE |
| CCBE1 | 10658 | 4.656304 | 3.22E-06 | 4.43E-05 | TRUE |
| CYB5D2 | 14665 | 4.65286 | 3.27E-06 | 4.50E-05 | TRUE |
| ADTRP | 10456 | 4.651027 | 3.30E-06 | 4.54E-05 | TRUE |
| CCR5 | 10534 | 4.649171 | 3.33E-06 | 4.56E-05 | TRUE |
| MRPS25 | 4294 | 4.639497 | 3.49E-06 | 4.76E-05 | TRUE |
| S100A10 | 517 | 4.638553 | 3.51E-06 | 4.77E-05 | TRUE |
| VEZT | 652 | 4.638399 | 3.51E-06 | 4.77E-05 | TRUE |
| C6orf52 | 11540 | 4.638388 | 3.51E-06 | 4.77E-05 | TRUE |
| C12orf75 | 13492 | 4.636643 | 3.54E-06 | 4.80E-05 | TRUE |
| SLC26A11 | 1455 | 4.635699 | 3.56E-06 | 4.81E-05 | TRUE |
| LOXL3 | 5748 | 4.63261 | 3.61E-06 | 4.87E-05 | TRUE |
| NOVA2 | 6668 | 4.631668 | 3.63E-06 | 4.89E-05 | TRUE |
| MT1B | 4525 | 4.629829 | 3.66E-06 | 4.92E-05 | TRUE |
| GRM1 | 2815 | 4.628305 | 3.69E-06 | 4.95E-05 | TRUE |
| CARMIL3 | 9654 | 4.626193 | 3.72E-06 | 4.99E-05 | TRUE |
| DNALI1 | 10709 | 4.626129 | 3.73E-06 | 4.99E-05 | TRUE |
| MARCO | 11581 | 4.624802 | 3.75E-06 | 5.02E-05 | TRUE |
| HDC | 11769 | 4.623916 | 3.77E-06 | 5.04E-05 | TRUE |
| PDYN | 4187 | 4.622476 | 3.79E-06 | 5.06E-05 | TRUE |
| SF3B3 | 5361 | 4.622368 | 3.79E-06 | 5.06E-05 | TRUE |
| SULF2 | 2615 | 4.619107 | 3.85E-06 | 5.13E-05 | TRUE |
| NSG2 | 770 | 4.618778 | 3.86E-06 | 5.13E-05 | TRUE |
| SMAD2 | 2998 | 4.618751 | 3.86E-06 | 5.13E-05 | TRUE |
| FAM102B | 13461 | 4.617774 | 3.88E-06 | 5.15E-05 | TRUE |
| ARMC10 | 9585 | 4.615879 | 3.91E-06 | 5.19E-05 | TRUE |
| AP3S1 | 14420 | 4.61566 | 3.92E-06 | 5.19E-05 | TRUE |
| CRYM | 5487 | 4.612612 | 3.98E-06 | 5.25E-05 | TRUE |
| PIM2 | 13872 | 4.611084 | 4.01E-06 | 5.29E-05 | TRUE |
| SPATA1 | 15033 | 4.608672 | 4.05E-06 | 5.35E-05 | TRUE |
| FAM207A | 10525 | 4.604896 | 4.13E-06 | 5.43E-05 | TRUE |
| FTL | 11525 | 4.603059 | 4.16E-06 | 5.47E-05 | TRUE |
| C11orf97 | 12811 | 4.60258 | 4.17E-06 | 5.47E-05 | TRUE |
| TMEM238 | 11332 | 4.59935 | 4.24E-06 | 5.54E-05 | TRUE |
| CD24 | 3231 | 4.595271 | 4.32E-06 | 5.64E-05 | TRUE |
| DNAJC30 | 12217 | 4.591573 | 4.40E-06 | 5.73E-05 | TRUE |
| SMIM4 | 6573 | 4.590171 | 4.43E-06 | 5.76E-05 | TRUE |
| C8orf34 | 5692 | 4.585879 | 4.52E-06 | 5.86E-05 | TRUE |
| HCLS1 | 7550 | 4.58053 | 4.64E-06 | 6.00E-05 | TRUE |
| LYRM9 | 11878 | 4.579689 | 4.66E-06 | 6.01E-05 | TRUE |
| DCAKD | 3194 | 4.578618 | 4.68E-06 | 6.03E-05 | TRUE |
| CYTOR | 3408 | 4.577788 | 4.70E-06 | 6.04E-05 | TRUE |
| GSTM3 | 11398 | 4.577318 | 4.71E-06 | 6.05E-05 | TRUE |
| PCOLCE2 | 8269 | 4.575068 | 4.76E-06 | 6.11E-05 | TRUE |
| RASAL3 | 2156 | 4.574769 | 4.77E-06 | 6.11E-05 | TRUE |
| PDGFRA | 2686 | 4.574245 | 4.78E-06 | 6.12E-05 | TRUE |
| CMTM3 | 941 | 4.573414 | 4.80E-06 | 6.14E-05 | TRUE |
| ARPC3 | 12393 | 4.570309 | 4.87E-06 | 6.22E-05 | TRUE |
| GRID2 | 10527 | 4.569916 | 4.88E-06 | 6.23E-05 | TRUE |
| NAXE | 1874 | 4.569382 | 4.89E-06 | 6.23E-05 | TRUE |
| TMEM256 | 9707 | 4.569313 | 4.89E-06 | 6.23E-05 | TRUE |
| AKR7L | 14624 | 4.568389 | 4.91E-06 | 6.25E-05 | TRUE |
| TG | 9987 | 4.566201 | 4.97E-06 | 6.31E-05 | TRUE |
| P2RY12 | 10475 | 4.565641 | 4.98E-06 | 6.32E-05 | TRUE |
| LUZP2 | 8994 | 4.562481 | 5.06E-06 | 6.40E-05 | TRUE |
| RAB34 | 5047 | 4.561904 | 5.07E-06 | 6.42E-05 | TRUE |
| CLU | 10654 | 4.558894 | 5.14E-06 | 6.50E-05 | TRUE |
| NUDT2 | 13602 | 4.556895 | 5.19E-06 | 6.55E-05 | TRUE |
| ZFP36L2 | 12527 | 4.553739 | 5.27E-06 | 6.64E-05 | TRUE |
| MIF | 5299 | 4.550429 | 5.35E-06 | 6.72E-05 | TRUE |
| AMZ1 | 14796 | 4.544078 | 5.52E-06 | 6.91E-05 | TRUE |
| LOC100506100 | 3564 | 4.541011 | 5.60E-06 | 6.99E-05 | TRUE |
| RAB27B | 8403 | 4.539013 | 5.65E-06 | 7.05E-05 | TRUE |
| CHMP4A | 3325 | 4.537015 | 5.71E-06 | 7.11E-05 | TRUE |
| ARHGAP45 | 870 | 4.534517 | 5.77E-06 | 7.17E-05 | TRUE |
| KRT86 | 11244 | 4.531532 | 5.86E-06 | 7.25E-05 | TRUE |
| CTSH | 8010 | 4.519231 | 6.21E-06 | 7.62E-05 | TRUE |
| CNRIP1 | 12680 | 4.519204 | 6.21E-06 | 7.62E-05 | TRUE |
| CAMKV | 1941 | 4.518527 | 6.23E-06 | 7.64E-05 | TRUE |
| COPZ1 | 3012 | 4.516515 | 6.29E-06 | 7.69E-05 | TRUE |
| NOL4 | 8458 | 4.515965 | 6.30E-06 | 7.70E-05 | TRUE |
| LOC101927420 | 7342 | 4.513473 | 6.38E-06 | 7.78E-05 | TRUE |
| RASAL1 | 1568 | 4.50817 | 6.54E-06 | 7.96E-05 | TRUE |
| DCLK2 | 13627 | 4.507895 | 6.55E-06 | 7.96E-05 | TRUE |
| UBL5 | 5382 | 4.507011 | 6.57E-06 | 7.98E-05 | TRUE |
| APTR | 3365 | 4.505091 | 6.63E-06 | 8.04E-05 | TRUE |
| RHBDF2 | 10516 | 4.500993 | 6.76E-06 | 8.16E-05 | TRUE |
| MAN1B1-AS1 | 3605 | 4.500417 | 6.78E-06 | 8.17E-05 | TRUE |
| TAB2 | 7418 | 4.497378 | 6.88E-06 | 8.29E-05 | TRUE |
| VIT | 10660 | 4.493337 | 7.01E-06 | 8.42E-05 | TRUE |
| CDC42P3 | 5579 | 4.490011 | 7.12E-06 | 8.54E-05 | TRUE |
| RSPH14 | 12542 | 4.488896 | 7.16E-06 | 8.57E-05 | TRUE |
| IL12RB2 | 1711 | 4.48789 | 7.19E-06 | 8.60E-05 | TRUE |
| CPNE7 | 6605 | 4.485606 | 7.27E-06 | 8.69E-05 | TRUE |
| SQLE | 8862 | 4.481005 | 7.43E-06 | 8.84E-05 | TRUE |
| PTCHD1 | 7338 | 4.478532 | 7.52E-06 | 8.93E-05 | TRUE |
| NEPRO | 1929 | 4.477581 | 7.55E-06 | 8.97E-05 | TRUE |
| PTN | 4198 | 4.474561 | 7.66E-06 | 9.09E-05 | TRUE |
| EIF1 | 7212 | 4.463947 | 8.05E-06 | 9.51E-05 | TRUE |
| APOM | 8806 | 4.462806 | 8.09E-06 | 9.54E-05 | TRUE |
| YJEFN3 | 7021 | 4.462283 | 8.11E-06 | 9.55E-05 | TRUE |
| CYB5R1 | 1839 | 4.454991 | 8.39E-06 | 9.85E-05 | TRUE |
| NPNT | 615 | 4.453839 | 8.43E-06 | 9.90E-05 | TRUE |
| ASB1 | 12726 | 4.452439 | 8.49E-06 | 9.95E-05 | TRUE |
| SELPLG | 7327 | 4.442766 | 8.88E-06 | 0.00010322 | TRUE |
| TUBA4A | 1269 | 4.437203 | 9.11E-06 | 0.00010561 | TRUE |
| TIMP1 | 13840 | 4.436588 | 9.14E-06 | 0.00010576 | TRUE |
| TMEM176A | 14178 | 4.435949 | 9.17E-06 | 0.00010599 | TRUE |
| WFDC1 | 12983 | 4.433242 | 9.28E-06 | 0.00010718 | TRUE |
| ACSM5 | 13292 | 4.433233 | 9.28E-06 | 0.00010718 | TRUE |
| DDA1 | 12827 | 4.428657 | 9.48E-06 | 0.00010932 | TRUE |
| RGS20 | 13270 | 4.425331 | 9.63E-06 | 0.00011077 | TRUE |
| TRIM24 | 11677 | 4.424991 | 9.64E-06 | 0.00011086 | TRUE |
| MS4A7 | 693 | 4.424526 | 9.67E-06 | 0.00011102 | TRUE |
| ATP6AP1 | 3511 | 4.424109 | 9.68E-06 | 0.00011115 | TRUE |
| LRTOMT | 15335 | 4.421537 | 9.80E-06 | 0.00011208 | TRUE |
| CNIH2 | 4287 | 4.419026 | 9.91E-06 | 0.00011322 | TRUE |
| A1BG | 3421 | 4.413869 | 1.02E-05 | 0.00011537 | TRUE |
| SERF2 | 4948 | 4.413504 | 1.02E-05 | 0.00011537 | TRUE |
| FCGBP | 9304 | 4.40992 | 1.03E-05 | 0.00011681 | TRUE |
| CX3CR1 | 800 | 4.409467 | 1.04E-05 | 0.00011688 | TRUE |
| TIMP4 | 8357 | 4.406293 | 1.05E-05 | 0.00011835 | TRUE |
| UQCC2 | 10618 | 4.4057 | 1.05E-05 | 0.00011859 | TRUE |
| NPB | 13051 | 4.404099 | 1.06E-05 | 0.00011938 | TRUE |
| CARD17 | 14231 | 4.400798 | 1.08E-05 | 0.00012087 | TRUE |
| DACT1 | 13256 | 4.395088 | 1.11E-05 | 0.00012364 | TRUE |
| PRR13 | 10650 | 4.392712 | 1.12E-05 | 0.00012483 | TRUE |
| EGFEM1P | 8276 | 4.391678 | 1.12E-05 | 0.00012532 | TRUE |
| C17orf67 | 9088 | 4.391534 | 1.13E-05 | 0.00012532 | TRUE |
| DNAJA1 | 13706 | 4.380172 | 1.19E-05 | 0.00013092 | TRUE |
| PLPPR3 | 12559 | 4.375075 | 1.21E-05 | 0.00013364 | TRUE |
| PGLS | 3542 | 4.374796 | 1.22E-05 | 0.00013372 | TRUE |
| TMSB4X | 1220 | 4.371089 | 1.24E-05 | 0.00013563 | TRUE |
| NIPSNAP1 | 260 | 4.365616 | 1.27E-05 | 0.00013878 | TRUE |
| SLC7A11 | 2762 | 4.363844 | 1.28E-05 | 0.00013971 | TRUE |
| RPL39L | 4749 | 4.3575 | 1.32E-05 | 0.00014342 | TRUE |
| TLR2 | 6389 | 4.357043 | 1.32E-05 | 0.00014362 | TRUE |
| SKAP2 | 4386 | 4.356487 | 1.32E-05 | 0.00014388 | TRUE |
| MRPS6 | 13969 | 4.354574 | 1.33E-05 | 0.00014494 | TRUE |
| PSENEN | 4793 | 4.350795 | 1.36E-05 | 0.00014695 | TRUE |
| ACTR10 | 1128 | 4.348755 | 1.37E-05 | 0.00014812 | TRUE |
| IGSF6 | 11941 | 4.348003 | 1.37E-05 | 0.00014831 | TRUE |
| NABP1 | 7555 | 4.347841 | 1.37E-05 | 0.00014831 | TRUE |
| HLA-DPA1 | 7692 | 4.347614 | 1.38E-05 | 0.00014831 | TRUE |
| NEURL1B | 461 | 4.347564 | 1.38E-05 | 0.00014831 | TRUE |
| ECHDC3 | 4602 | 4.346279 | 1.38E-05 | 0.00014877 | TRUE |
| BEGAIN | 6589 | 4.345781 | 1.39E-05 | 0.00014901 | TRUE |
| BEX2 | 8274 | 4.343902 | 1.40E-05 | 0.00014995 | TRUE |
| FAM229B | 12139 | 4.340634 | 1.42E-05 | 0.00015181 | TRUE |
| SCPEP1 | 7811 | 4.331949 | 1.48E-05 | 0.00015718 | TRUE |
| SEMA4A | 10311 | 4.3308 | 1.49E-05 | 0.00015778 | TRUE |
| IGFBP5 | 848 | 4.329419 | 1.50E-05 | 0.00015865 | TRUE |
| KXD1 | 9100 | 4.326156 | 1.52E-05 | 0.00016071 | TRUE |
| NOL4L | 6738 | 4.324194 | 1.53E-05 | 0.00016204 | TRUE |
| KIAA1024 | 3100 | 4.323435 | 1.54E-05 | 0.00016248 | TRUE |
| RPL32 | 1032 | 4.323037 | 1.54E-05 | 0.00016267 | TRUE |
| AEBP1 | 3412 | 4.322086 | 1.55E-05 | 0.00016304 | TRUE |
| FYB1 | 14370 | 4.321411 | 1.55E-05 | 0.00016343 | TRUE |
| BLVRB | 4196 | 4.319187 | 1.57E-05 | 0.00016497 | TRUE |
| FGD3 | 12472 | 4.314853 | 1.60E-05 | 0.00016752 | TRUE |
| HBM | 4204 | 4.314771 | 1.60E-05 | 0.00016752 | TRUE |
| UMPS | 10511 | 4.312799 | 1.61E-05 | 0.00016879 | TRUE |
| HMGCS1 | 157 | 4.312499 | 1.61E-05 | 0.00016891 | TRUE |
| C19orf70 | 6845 | 4.310225 | 1.63E-05 | 0.00017043 | TRUE |
| TPST1 | 14789 | 4.301791 | 1.69E-05 | 0.00017657 | TRUE |
| POC1A | 8533 | 4.301362 | 1.70E-05 | 0.0001768 | TRUE |
| ROBO2 | 10117 | 4.298653 | 1.72E-05 | 0.00017862 | TRUE |
| PLD3 | 11481 | 4.297385 | 1.73E-05 | 0.00017944 | TRUE |
| PLSCR4 | 10562 | 4.294211 | 1.75E-05 | 0.00018126 | TRUE |
| PRPH | 15350 | 4.293101 | 1.76E-05 | 0.00018205 | TRUE |
| PTGER4 | 10941 | 4.290613 | 1.78E-05 | 0.00018357 | TRUE |
| IMPACT | 8087 | 4.288813 | 1.80E-05 | 0.00018475 | TRUE |
| SERPINF1 | 9028 | 4.287639 | 1.81E-05 | 0.0001856 | TRUE |
| PPM1N | 1400 | 4.286944 | 1.81E-05 | 0.00018606 | TRUE |
| CRMP1 | 468 | 4.283092 | 1.84E-05 | 0.0001887 | TRUE |
| VPS37B | 6992 | 4.280168 | 1.87E-05 | 0.00019082 | TRUE |
| ZNF436 | 7761 | 4.275319 | 1.91E-05 | 0.00019451 | TRUE |
| TINCR | 10234 | 4.27464 | 1.91E-05 | 0.00019498 | TRUE |
| SCN7A | 12005 | 4.273154 | 1.93E-05 | 0.00019577 | TRUE |
| RAB32 | 13448 | 4.271589 | 1.94E-05 | 0.00019702 | TRUE |
| APLNR | 7499 | 4.26774 | 1.97E-05 | 0.0001998 | TRUE |
| EXOC6 | 5304 | 4.267076 | 1.98E-05 | 0.00020027 | TRUE |
| ZFP36L1 | 9055 | 4.261449 | 2.03E-05 | 0.00020459 | TRUE |
| SARDH | 2894 | 4.261192 | 2.03E-05 | 0.00020469 | TRUE |
| CERS1 | 2921 | 4.259938 | 2.04E-05 | 0.00020571 | TRUE |
| ALKBH7 | 7882 | 4.258382 | 2.06E-05 | 0.00020661 | TRUE |
| SNUPN | 8129 | 4.257753 | 2.06E-05 | 0.00020706 | TRUE |
| FBL | 1027 | 4.253177 | 2.11E-05 | 0.00021067 | TRUE |
| ZCCHC17 | 5720 | 4.248646 | 2.15E-05 | 0.00021429 | TRUE |
| ACTR3B | 944 | 4.247369 | 2.16E-05 | 0.00021524 | TRUE |
| GFAP | 3172 | 4.244473 | 2.19E-05 | 0.0002172 | TRUE |
| TUBB | 2168 | 4.242982 | 2.21E-05 | 0.00021851 | TRUE |
| ASTN1 | 12818 | 4.242576 | 2.21E-05 | 0.00021877 | TRUE |
| CTXN1 | 2452 | 4.240684 | 2.23E-05 | 0.00022035 | TRUE |
| FAM117A | 4169 | 4.237218 | 2.26E-05 | 0.00022321 | TRUE |
| GPRIN1 | 795 | 4.235347 | 2.28E-05 | 0.00022466 | TRUE |
| CDH13 | 10617 | 4.228419 | 2.35E-05 | 0.00023066 | TRUE |
| SNTG2 | 10624 | 4.22594 | 2.38E-05 | 0.00023292 | TRUE |
| PALMD | 11326 | 4.224468 | 2.40E-05 | 0.00023422 | TRUE |
| RAB8B | 14160 | 4.22441 | 2.40E-05 | 0.00023422 | TRUE |
| TUSC3 | 1814 | 4.219575 | 2.45E-05 | 0.00023855 | TRUE |
| RAVER2 | 9784 | 4.219351 | 2.45E-05 | 0.00023864 | TRUE |
| IGF1 | 6227 | 4.218738 | 2.46E-05 | 0.00023899 | TRUE |
| LOC440934 | 7163 | 4.218434 | 2.46E-05 | 0.00023917 | TRUE |
| C20orf27 | 730 | 4.21685 | 2.48E-05 | 0.0002407 | TRUE |
| KLHL35 | 5419 | 4.215881 | 2.49E-05 | 0.00024159 | TRUE |
| FKBP14 | 14036 | 4.212098 | 2.53E-05 | 0.00024495 | TRUE |
| MACROD2 | 11166 | 4.212062 | 2.53E-05 | 0.00024495 | TRUE |
| PCK2 | 5517 | 4.208988 | 2.57E-05 | 0.00024777 | TRUE |
| METTL1 | 4715 | 4.20895 | 2.57E-05 | 0.00024777 | TRUE |
| FBXO2 | 13199 | 4.20892 | 2.57E-05 | 0.00024777 | TRUE |
| NWD2 | 2632 | 4.208684 | 2.57E-05 | 0.00024787 | TRUE |
| XYLT1 | 283 | 4.207911 | 2.58E-05 | 0.00024857 | TRUE |
| GNB2 | 11384 | 4.20696 | 2.59E-05 | 0.00024946 | TRUE |
| LSM4 | 3185 | 4.204348 | 2.62E-05 | 0.00025219 | TRUE |
| NXPH1 | 1363 | 4.204224 | 2.62E-05 | 0.00025219 | TRUE |
| CLTCL1 | 1825 | 4.203917 | 2.62E-05 | 0.00025238 | TRUE |
| TUBB8 | 6869 | 4.203247 | 2.63E-05 | 0.00025297 | TRUE |
| FUCA1 | 10006 | 4.202919 | 2.63E-05 | 0.00025318 | TRUE |
| VAV1 | 9629 | 4.201267 | 2.65E-05 | 0.00025472 | TRUE |
| C11orf1 | 4291 | 4.200114 | 2.67E-05 | 0.00025571 | TRUE |
| LDOC1 | 5895 | 4.196615 | 2.71E-05 | 0.00025937 | TRUE |
| CYP26A1 | 10655 | 4.188434 | 2.81E-05 | 0.00026742 | TRUE |
| GLI1 | 4134 | 4.187697 | 2.82E-05 | 0.00026813 | TRUE |
| LIX1 | 12667 | 4.186915 | 2.83E-05 | 0.00026873 | TRUE |
| C1QTNF2 | 7368 | 4.183924 | 2.87E-05 | 0.00027179 | TRUE |
| THAP8 | 12426 | 4.182883 | 2.88E-05 | 0.00027255 | TRUE |
| KAT14 | 8466 | 4.181889 | 2.89E-05 | 0.00027344 | TRUE |
| IGSF22 | 14326 | 4.181861 | 2.89E-05 | 0.00027344 | TRUE |
| SSTR2 | 14113 | 4.18148 | 2.90E-05 | 0.00027374 | TRUE |
| GCNT1 | 7272 | 4.181128 | 2.90E-05 | 0.00027386 | TRUE |
| LINC01315 | 1462 | 4.180894 | 2.90E-05 | 0.00027386 | TRUE |
| NEDD8 | 3748 | 4.179683 | 2.92E-05 | 0.00027491 | TRUE |
| CNR1 | 11925 | 4.178887 | 2.93E-05 | 0.00027571 | TRUE |
| TMEM106C | 263 | 4.177058 | 2.95E-05 | 0.0002776 | TRUE |
| DHDH | 8684 | 4.172986 | 3.01E-05 | 0.0002821 | TRUE |
| CDO1 | 13005 | 4.166899 | 3.09E-05 | 0.00028818 | TRUE |
| PYCR1 | 4979 | 4.166697 | 3.09E-05 | 0.00028826 | TRUE |
| ANAPC11 | 12052 | 4.165642 | 3.10E-05 | 0.0002892 | TRUE |
| GJA1 | 650 | 4.16455 | 3.12E-05 | 0.00028999 | TRUE |
| FAM189A2 | 13304 | 4.1639 | 3.13E-05 | 0.00029057 | TRUE |
| CDKN2D | 7428 | 4.163716 | 3.13E-05 | 0.00029057 | TRUE |
| TFEC | 140 | 4.163605 | 3.13E-05 | 0.00029057 | TRUE |
| SYK | 4473 | 4.160942 | 3.17E-05 | 0.00029335 | TRUE |
| VSIG4 | 5505 | 4.157704 | 3.21E-05 | 0.00029701 | TRUE |
| TTC9B | 4258 | 4.15571 | 3.24E-05 | 0.00029908 | TRUE |
| TNNI3 | 15032 | 4.154782 | 3.26E-05 | 0.00029995 | TRUE |
| MED19 | 3765 | 4.153442 | 3.28E-05 | 0.00030118 | TRUE |
| COPS4 | 7091 | 4.149983 | 3.32E-05 | 0.00030487 | TRUE |
| SUB1 | 12381 | 4.148428 | 3.35E-05 | 0.00030676 | TRUE |
| BLNK | 5016 | 4.147313 | 3.36E-05 | 0.00030777 | TRUE |
| CD81 | 6205 | 4.14728 | 3.36E-05 | 0.00030777 | TRUE |
| MAN1A1 | 12085 | 4.139917 | 3.47E-05 | 0.00031652 | TRUE |
| AKIRIN2 | 7208 | 4.136227 | 3.53E-05 | 0.00032127 | TRUE |
| FIGNL2 | 11319 | 4.134872 | 3.55E-05 | 0.00032298 | TRUE |
| SEMA5A | 11341 | 4.132718 | 3.58E-05 | 0.00032584 | TRUE |
| HES4 | 6755 | 4.130525 | 3.62E-05 | 0.00032877 | TRUE |
| TMEM215 | 2591 | 4.129219 | 3.64E-05 | 0.00033045 | TRUE |
| LINC00937 | 9310 | 4.127713 | 3.66E-05 | 0.00033168 | TRUE |
| STAB1 | 8204 | 4.127458 | 3.67E-05 | 0.00033184 | TRUE |
| ADIRF | 1815 | 4.126046 | 3.69E-05 | 0.0003333 | TRUE |
| METTL6 | 12910 | 4.123585 | 3.73E-05 | 0.00033669 | TRUE |
| WNT7B | 8270 | 4.122051 | 3.76E-05 | 0.00033835 | TRUE |
| AHRR | 11068 | 4.118303 | 3.82E-05 | 0.00034311 | TRUE |
| GABRE | 8635 | 4.117915 | 3.82E-05 | 0.00034349 | TRUE |
| UBE2S | 13019 | 4.116831 | 3.84E-05 | 0.00034448 | TRUE |
| GGTA1P | 13407 | 4.11641 | 3.85E-05 | 0.00034475 | TRUE |
| SPICE1 | 12280 | 4.112951 | 3.91E-05 | 0.00034928 | TRUE |
| PDLIM3 | 1756 | 4.111848 | 3.93E-05 | 0.00035043 | TRUE |
| FARSB | 10537 | 4.111317 | 3.93E-05 | 0.00035104 | TRUE |
| CMTM8 | 762 | 4.110149 | 3.95E-05 | 0.00035262 | TRUE |
| RNF5 | 12874 | 4.107112 | 4.01E-05 | 0.00035647 | TRUE |
| JMJD7 | 6876 | 4.106457 | 4.02E-05 | 0.00035707 | TRUE |
| DKK3 | 2537 | 4.101818 | 4.10E-05 | 0.00036368 | TRUE |
| SNX3 | 1232 | 4.099577 | 4.14E-05 | 0.00036681 | TRUE |
| VAMP8 | 3113 | 4.096948 | 4.19E-05 | 0.00037058 | TRUE |
| RTL6 | 4512 | 4.096229 | 4.20E-05 | 0.00037152 | TRUE |
| SNAPC5 | 8926 | 4.090671 | 4.30E-05 | 0.00037905 | TRUE |
| RMI2 | 7203 | 4.090454 | 4.31E-05 | 0.00037905 | TRUE |
| ZNF831 | 13079 | 4.090413 | 4.31E-05 | 0.00037905 | TRUE |
| FDX1L | 10822 | 4.087905 | 4.35E-05 | 0.00038272 | TRUE |
| HSD17B8 | 10365 | 4.086168 | 4.39E-05 | 0.00038524 | TRUE |
| FAR2P2 | 7236 | 4.085396 | 4.40E-05 | 0.00038608 | TRUE |
| EFNB2 | 1041 | 4.084826 | 4.41E-05 | 0.00038653 | TRUE |
| CHCHD5 | 139 | 4.081196 | 4.48E-05 | 0.00039152 | TRUE |
| STK32C | 3147 | 4.080825 | 4.49E-05 | 0.00039172 | TRUE |
| UBE2D2 | 13480 | 4.079119 | 4.52E-05 | 0.00039371 | TRUE |
| SFMBT2 | 2281 | 4.077243 | 4.56E-05 | 0.00039605 | TRUE |
| FAM86FP | 13433 | 4.07698 | 4.56E-05 | 0.00039625 | TRUE |
| RRAS2 | 1034 | 4.0767 | 4.57E-05 | 0.00039651 | TRUE |
| ADGRV1 | 12569 | 4.073858 | 4.62E-05 | 0.00040049 | TRUE |
| RGS4 | 323 | 4.072592 | 4.65E-05 | 0.00040245 | TRUE |
| TBC1D26 | 12250 | 4.072284 | 4.66E-05 | 0.00040276 | TRUE |
| GABRA3 | 406 | 4.071552 | 4.67E-05 | 0.00040358 | TRUE |
| POP4 | 11765 | 4.070564 | 4.69E-05 | 0.00040485 | TRUE |
| ITGB2 | 12646 | 4.06478 | 4.81E-05 | 0.00041479 | TRUE |
| SLC2A12 | 13533 | 4.063639 | 4.83E-05 | 0.00041659 | TRUE |
| WLS | 12971 | 4.062448 | 4.86E-05 | 0.0004185 | TRUE |
| RPS10 | 9465 | 4.053693 | 5.04E-05 | 0.00043352 | TRUE |
| CHODL | 747 | 4.050661 | 5.11E-05 | 0.00043821 | TRUE |
| PPP2R3C | 4308 | 4.048185 | 5.16E-05 | 0.00044263 | TRUE |
| CNMD | 2515 | 4.046996 | 5.19E-05 | 0.00044464 | TRUE |
| KLHL13 | 14794 | 4.044142 | 5.25E-05 | 0.00044878 | TRUE |
| EIF6 | 4340 | 4.044101 | 5.25E-05 | 0.00044878 | TRUE |
| PAX6 | 8953 | 4.0441 | 5.25E-05 | 0.00044878 | TRUE |
| PLTP | 2148 | 4.043498 | 5.27E-05 | 0.00044912 | TRUE |
| TRMT112 | 3875 | 4.043189 | 5.27E-05 | 0.00044934 | TRUE |
| VAT1 | 4526 | 4.043123 | 5.27E-05 | 0.00044934 | TRUE |
| EMC3 | 6011 | 4.040027 | 5.34E-05 | 0.00045383 | TRUE |
| DBI | 13589 | 4.039764 | 5.35E-05 | 0.0004541 | TRUE |
| EIF4EBP1 | 6682 | 4.03951 | 5.36E-05 | 0.00045434 | TRUE |
| ACAT2 | 12338 | 4.038702 | 5.37E-05 | 0.00045551 | TRUE |
| WDR31 | 6823 | 4.038136 | 5.39E-05 | 0.00045627 | TRUE |
| CD68 | 3502 | 4.037084 | 5.41E-05 | 0.00045782 | TRUE |
| WRB | 3189 | 4.036281 | 5.43E-05 | 0.00045886 | TRUE |
| TUBB3 | 5718 | 4.036173 | 5.43E-05 | 0.00045886 | TRUE |
| C1orf61 | 1263 | 4.035111 | 5.46E-05 | 0.00046069 | TRUE |
| RPS2 | 5325 | 4.033936 | 5.49E-05 | 0.0004625 | TRUE |
| FAM171B | 11336 | 4.03335 | 5.50E-05 | 0.00046341 | TRUE |
| ATP5MF | 2154 | 4.02995 | 5.58E-05 | 0.00046991 | TRUE |
| VAMP2 | 9925 | 4.028106 | 5.62E-05 | 0.00047284 | TRUE |
| NHLRC1 | 594 | 4.026365 | 5.66E-05 | 0.0004761 | TRUE |
| CDH4 | 768 | 4.024737 | 5.70E-05 | 0.00047915 | TRUE |
| EFHB | 11076 | 4.024382 | 5.71E-05 | 0.00047935 | TRUE |
| ANKLE2 | 8929 | 4.023195 | 5.74E-05 | 0.00048152 | TRUE |
| SUMO3 | 7808 | 4.020033 | 5.82E-05 | 0.00048672 | TRUE |
| DOC2B | 11567 | 4.018455 | 5.86E-05 | 0.00048895 | TRUE |
| KLHL1 | 9175 | 4.018176 | 5.87E-05 | 0.00048926 | TRUE |
| LARP1 | 1129 | 4.016467 | 5.91E-05 | 0.0004923 | TRUE |
| DPF1 | 1303 | 4.014486 | 5.96E-05 | 0.00049619 | TRUE |
| UQCRFS1 | 10316 | 4.011763 | 6.03E-05 | 0.00050115 | TRUE |
| CASP1 | 2429 | 4.010919 | 6.05E-05 | 0.00050243 | TRUE |
| RHBDD2 | 12153 | 4.00859 | 6.11E-05 | 0.00050577 | TRUE |
| MCUB | 1168 | 4.0028 | 6.26E-05 | 0.00051647 | TRUE |
| B4GALT2 | 14205 | 4.00236 | 6.27E-05 | 0.00051709 | TRUE |
| EMID1 | 11243 | 4.00081 | 6.31E-05 | 0.00052022 | TRUE |
| E2F5 | 9121 | 4.000544 | 6.32E-05 | 0.00052053 | TRUE |
| LINC01750 | 10620 | 3.998166 | 6.38E-05 | 0.0005249 | TRUE |
| PTPRF | 4210 | 3.996942 | 6.42E-05 | 0.00052684 | TRUE |
| HSP90B1 | 8885 | 3.996567 | 6.43E-05 | 0.0005274 | TRUE |
| MAFG | 10601 | 3.994922 | 6.47E-05 | 0.00053052 | TRUE |
| FDPSP2 | 9344 | 3.993511 | 6.51E-05 | 0.00053341 | TRUE |
| ATOX1 | 10856 | 3.993224 | 6.52E-05 | 0.00053377 | TRUE |
| RPL27 | 13356 | 3.993084 | 6.52E-05 | 0.00053381 | TRUE |
| TREM2 | 14973 | 3.991977 | 6.55E-05 | 0.00053547 | TRUE |
| CDH8 | 14964 | 3.991676 | 6.56E-05 | 0.00053587 | TRUE |
| OPRM1 | 370 | 3.991538 | 6.56E-05 | 0.0005359 | TRUE |
| PRPH2 | 9637 | 3.9883 | 6.65E-05 | 0.00054213 | TRUE |
| TRIP12 | 7513 | 3.987368 | 6.68E-05 | 0.00054398 | TRUE |
| SIGLEC11 | 8019 | 3.986487 | 6.71E-05 | 0.00054572 | TRUE |
| FGGY | 1733 | 3.982972 | 6.81E-05 | 0.00055242 | TRUE |
| KLF8 | 7003 | 3.981479 | 6.85E-05 | 0.00055533 | TRUE |
| ASIC2 | 232 | 3.980772 | 6.87E-05 | 0.0005564 | TRUE |
| FAM160A2 | 6649 | 3.979941 | 6.89E-05 | 0.00055777 | TRUE |
| MT3 | 13599 | 3.979259 | 6.91E-05 | 0.00055909 | TRUE |
| B3GALT6 | 7443 | 3.97838 | 6.94E-05 | 0.00056087 | TRUE |
| B3GAT1 | 13312 | 3.977535 | 6.96E-05 | 0.00056257 | TRUE |
| TMEM255A | 13120 | 3.976435 | 7.00E-05 | 0.0005646 | TRUE |
| MCM6 | 5273 | 3.975137 | 7.03E-05 | 0.0005671 | TRUE |
| RPL7A | 2719 | 3.973542 | 7.08E-05 | 0.00057003 | TRUE |
| TCTA | 11321 | 3.973289 | 7.09E-05 | 0.00057034 | TRUE |
| MLC1 | 2068 | 3.973079 | 7.09E-05 | 0.00057055 | TRUE |
| HSBP1 | 7561 | 3.972722 | 7.11E-05 | 0.00057112 | TRUE |
| LRMDA | 13891 | 3.972172 | 7.12E-05 | 0.000572 | TRUE |
| DPYSL4 | 14987 | 3.972108 | 7.12E-05 | 0.000572 | TRUE |
| LINC01485 | 1512 | 3.97166 | 7.14E-05 | 0.00057278 | TRUE |
| CHRNA3 | 4341 | 3.970775 | 7.16E-05 | 0.00057462 | TRUE |
| MYB | 8692 | 3.970259 | 7.18E-05 | 0.00057528 | TRUE |
| CALHM6 | 300 | 3.969697 | 7.20E-05 | 0.00057634 | TRUE |
| PTPRZ1 | 4423 | 3.966469 | 7.29E-05 | 0.0005833 | TRUE |
| CACHD1 | 6793 | 3.965795 | 7.32E-05 | 0.00058465 | TRUE |
| FZD9 | 6783 | 3.965288 | 7.33E-05 | 0.0005856 | TRUE |
| PIFO | 452 | 3.963761 | 7.38E-05 | 0.00058876 | TRUE |
| MAPK1IP1L | 5250 | 3.963163 | 7.40E-05 | 0.00058993 | TRUE |
| RARA-AS1 | 13967 | 3.963041 | 7.40E-05 | 0.00058993 | TRUE |
| CSTB | 3608 | 3.961812 | 7.44E-05 | 0.00059268 | TRUE |
| C2orf73 | 11260 | 3.960356 | 7.48E-05 | 0.0005957 | TRUE |
| TYW5 | 7996 | 3.959734 | 7.50E-05 | 0.00059664 | TRUE |
| TMEM53 | 2265 | 3.958837 | 7.53E-05 | 0.00059858 | TRUE |
| SNCA | 3443 | 3.958025 | 7.56E-05 | 0.0005997 | TRUE |
| FOXN3-AS1 | 3585 | 3.95427 | 7.68E-05 | 0.00060788 | TRUE |
| PKIB | 12046 | 3.954181 | 7.68E-05 | 0.00060788 | TRUE |
| LGALS9C | 5493 | 3.953966 | 7.69E-05 | 0.00060812 | TRUE |
| OXCT1 | 486 | 3.951838 | 7.76E-05 | 0.00061281 | TRUE |
| LARGE1 | 2978 | 3.948895 | 7.85E-05 | 0.00061863 | TRUE |
| IFI27L2 | 10107 | 3.948782 | 7.85E-05 | 0.00061863 | TRUE |
| SNRPC | 13331 | 3.947463 | 7.90E-05 | 0.00062173 | TRUE |
| ZNF226 | 7174 | 3.94717 | 7.91E-05 | 0.00062186 | TRUE |
| HBG1 | 13166 | 3.943519 | 8.03E-05 | 0.00062983 | TRUE |
| PHPT1 | 14729 | 3.940683 | 8.12E-05 | 0.00063668 | TRUE |
| PSMD9 | 14956 | 3.93983 | 8.15E-05 | 0.00063863 | TRUE |
| PPHLN1 | 11339 | 3.939362 | 8.17E-05 | 0.00063899 | TRUE |
| SSSCA1 | 6841 | 3.939334 | 8.17E-05 | 0.00063899 | TRUE |
| INPP5K | 10334 | 3.937354 | 8.24E-05 | 0.00064332 | TRUE |
| POLR3GL | 6773 | 3.935535 | 8.30E-05 | 0.00064724 | TRUE |
| EZR | 1500 | 3.934969 | 8.32E-05 | 0.00064845 | TRUE |
| PMM1 | 3793 | 3.933469 | 8.37E-05 | 0.00065186 | TRUE |
| DPCD | 2831 | 3.93074 | 8.47E-05 | 0.00065775 | TRUE |
| STX12 | 9276 | 3.930589 | 8.47E-05 | 0.00065775 | TRUE |
| ARL15 | 14462 | 3.926673 | 8.61E-05 | 0.00066755 | TRUE |
| CHRNB1 | 2487 | 3.925036 | 8.67E-05 | 0.00067178 | TRUE |
| RPL29P2 | 3972 | 3.923743 | 8.72E-05 | 0.00067506 | TRUE |
| PDK2 | 921 | 3.92065 | 8.83E-05 | 0.00068277 | TRUE |
| C1orf53 | 3061 | 3.919594 | 8.87E-05 | 0.00068467 | TRUE |
| CD33 | 5482 | 3.918865 | 8.90E-05 | 0.00068615 | TRUE |
| ACOX2 | 14812 | 3.918493 | 8.91E-05 | 0.00068687 | TRUE |
| CA11 | 11951 | 3.915266 | 9.03E-05 | 0.00069441 | TRUE |
| PTPRC | 6982 | 3.914706 | 9.05E-05 | 0.00069568 | TRUE |
| SEZ6 | 12709 | 3.914583 | 9.06E-05 | 0.00069569 | TRUE |
| NETO2 | 13861 | 3.911829 | 9.16E-05 | 0.00070264 | TRUE |
| LOC257396 | 1337 | 3.910681 | 9.20E-05 | 0.00070564 | TRUE |
| XKR4 | 12523 | 3.908482 | 9.29E-05 | 0.0007114 | TRUE |
| ARL16 | 11491 | 3.906506 | 9.36E-05 | 0.00071607 | TRUE |
| LTBP4 | 15594 | 3.905824 | 9.39E-05 | 0.00071715 | TRUE |
| CELF5 | 4357 | 3.90549 | 9.40E-05 | 0.0007178 | TRUE |
| RPL31P11 | 8592 | 3.905261 | 9.41E-05 | 0.00071812 | TRUE |
| PLCH2 | 11793 | 3.904881 | 9.43E-05 | 0.0007189 | TRUE |
| RIMBP2 | 12000 | 3.904594 | 9.44E-05 | 0.00071932 | TRUE |
| CD83 | 5984 | 3.904505 | 9.44E-05 | 0.00071932 | TRUE |
| SPRN | 1479 | 3.903334 | 9.49E-05 | 0.00072246 | TRUE |
| EYA2 | 4767 | 3.901455 | 9.56E-05 | 0.00072703 | TRUE |
| RIOX2 | 5333 | 3.898667 | 9.67E-05 | 0.00073473 | TRUE |
| RPL23 | 4936 | 3.897089 | 9.74E-05 | 0.0007381 | TRUE |
| DNAH12 | 820 | 3.895941 | 9.78E-05 | 0.00074089 | TRUE |
| GAR1 | 9583 | 3.895737 | 9.79E-05 | 0.00074115 | TRUE |
| TBCB | 12613 | 3.895279 | 9.81E-05 | 0.0007422 | TRUE |
| CCR1 | 1344 | 3.894667 | 9.83E-05 | 0.00074335 | TRUE |
| LOH12CR2 | 14127 | 3.892175 | 9.93E-05 | 0.00075031 | TRUE |
| SAMD9L | 6191 | 3.891183 | 9.98E-05 | 0.00075302 | TRUE |
| MAPK3 | 12159 | 3.888682 | 0.0001008 | 0.00075966 | TRUE |
| NNAT | 5109 | 3.888593 | 0.0001008 | 0.00075966 | TRUE |
| LMO1 | 14780 | 3.888593 | 0.0001008 | 0.00075966 | TRUE |
| DDX25 | 12348 | 3.888584 | 0.0001008 | 0.00075966 | TRUE |
| MYL6B | 7785 | 3.887634 | 0.0001012 | 0.0007619 | TRUE |
| CTC1 | 7024 | 3.886136 | 0.0001019 | 0.00076618 | TRUE |
| C12orf73 | 12928 | 3.885862 | 0.000102 | 0.00076637 | TRUE |
| PYCARD | 305 | 3.884222 | 0.0001027 | 0.00077082 | TRUE |
| HIST1H2BI | 14520 | 3.883853 | 0.0001028 | 0.00077162 | TRUE |
| CDC42 | 5724 | 3.882844 | 0.0001032 | 0.00077409 | TRUE |
| CABCOCO1 | 11353 | 3.88241 | 0.0001034 | 0.0007751 | TRUE |
| PRR29 | 3338 | 3.878335 | 0.0001052 | 0.00078744 | TRUE |
| IL3RA | 626 | 3.873916 | 0.0001071 | 0.00080072 | TRUE |
| KRT14 | 2185 | 3.871094 | 0.0001083 | 0.00080889 | TRUE |
| MRPL51 | 561 | 3.86941 | 0.0001091 | 0.0008141 | TRUE |
| CMTM7 | 1370 | 3.864366 | 0.0001114 | 0.00082834 | TRUE |
| BORCS7 | 9033 | 3.863085 | 0.000112 | 0.00083191 | TRUE |
| ARHGAP6 | 11898 | 3.862272 | 0.0001123 | 0.00083389 | TRUE |
| TMEM70 | 2144 | 3.859422 | 0.0001137 | 0.00084247 | TRUE |
| LOC100507351 | 11971 | 3.859079 | 0.0001138 | 0.00084286 | TRUE |
| UBE2A | 12774 | 3.856516 | 0.000115 | 0.00085053 | TRUE |
| LOC100507516 | 14264 | 3.853204 | 0.0001166 | 0.00086131 | TRUE |
| GALM | 11492 | 3.853081 | 0.0001166 | 0.00086134 | TRUE |
| LOC441052 | 2829 | 3.852721 | 0.0001168 | 0.0008622 | TRUE |
| CEP83 | 7472 | 3.849512 | 0.0001184 | 0.00087275 | TRUE |
| NLE1 | 9995 | 3.846869 | 0.0001196 | 0.00088013 | TRUE |
| NEBL-AS1 | 780 | 3.845979 | 0.0001201 | 0.0008825 | TRUE |
| ADCYAP1R1 | 7328 | 3.845799 | 0.0001202 | 0.00088274 | TRUE |
| ADCY7 | 12221 | 3.843825 | 0.0001211 | 0.00088862 | TRUE |
| SNAI2 | 4349 | 3.840034 | 0.000123 | 0.00089992 | TRUE |
| CCL28 | 7451 | 3.838352 | 0.0001239 | 0.00090505 | TRUE |
| MNDA | 679 | 3.837024 | 0.0001245 | 0.00090932 | TRUE |
| DBX2 | 15023 | 3.836023 | 0.000125 | 0.00091234 | TRUE |
| AK1 | 14710 | 3.835676 | 0.0001252 | 0.00091304 | TRUE |
| C21orf2 | 8883 | 3.834741 | 0.0001257 | 0.00091567 | TRUE |
| MFSD12 | 14767 | 3.834519 | 0.0001258 | 0.00091607 | TRUE |
| C16orf95 | 545 | 3.832619 | 0.0001268 | 0.00092274 | TRUE |
| WDR25 | 11026 | 3.832241 | 0.000127 | 0.00092373 | TRUE |
| DACH2 | 10851 | 3.830078 | 0.0001281 | 0.00093102 | TRUE |
| C2CD2L | 9163 | 3.826341 | 0.0001301 | 0.00094395 | TRUE |
| ZMYND15 | 11381 | 3.824683 | 0.0001309 | 0.00094988 | TRUE |
| CCDC148 | 6008 | 3.824368 | 0.0001311 | 0.00095021 | TRUE |
| LMAN2L | 6341 | 3.824121 | 0.0001312 | 0.00095073 | TRUE |
| DENND1C | 7806 | 3.822572 | 0.0001321 | 0.00095539 | TRUE |
| PUF60 | 9912 | 3.818669 | 0.0001342 | 0.00096974 | TRUE |
| SCG3 | 13274 | 3.817343 | 0.0001349 | 0.00097316 | TRUE |
| KIF21B | 9093 | 3.816668 | 0.0001353 | 0.00097522 | TRUE |
| CTNND2 | 7705 | 3.816143 | 0.0001356 | 0.0009761 | TRUE |
| GATB | 10051 | 3.81593 | 0.0001357 | 0.00097649 | TRUE |
| RPL26 | 8775 | 3.814022 | 0.0001367 | 0.00098271 | TRUE |
| SMIM27 | 1385 | 3.806608 | 0.0001409 | 0.00100938 | TRUE |
| RSPO4 | 11061 | 3.805018 | 0.0001418 | 0.00101496 | TRUE |
| ZDHHC4 | 8218 | 3.804003 | 0.0001424 | 0.00101866 | TRUE |
| FZR1 | 8912 | 3.800688 | 0.0001443 | 0.00103097 | TRUE |
| HSD11B1L | 5690 | 3.799924 | 0.0001447 | 0.00103368 | TRUE |
| MT2A | 14208 | 3.799644 | 0.0001449 | 0.00103391 | TRUE |
| CCT7 | 6273 | 3.798626 | 0.0001455 | 0.00103721 | TRUE |
| CRELD2 | 15430 | 3.798398 | 0.0001456 | 0.00103769 | TRUE |
| CHST1 | 7036 | 3.79277 | 0.000149 | 0.00105909 | TRUE |
| VKORC1L1 | 12062 | 3.791984 | 0.0001494 | 0.00106149 | TRUE |
| CFAP61 | 12443 | 3.791033 | 0.00015 | 0.00106459 | TRUE |
| PPDPF | 2802 | 3.78972 | 0.0001508 | 0.00106926 | TRUE |
| ALOX5AP | 4992 | 3.782952 | 0.000155 | 0.00109334 | TRUE |
| CAPN12 | 8155 | 3.782946 | 0.000155 | 0.00109334 | TRUE |
| FNDC11 | 14615 | 3.780553 | 0.0001565 | 0.00110192 | TRUE |
| RPL31 | 7481 | 3.780241 | 0.0001567 | 0.0011028 | TRUE |
| CTGF | 8898 | 3.777666 | 0.0001583 | 0.00111326 | TRUE |
| HADH | 3927 | 3.775408 | 0.0001597 | 0.00112289 | TRUE |
| PCDH15 | 12093 | 3.775114 | 0.0001599 | 0.00112371 | TRUE |
| DISP2 | 6314 | 3.772995 | 0.0001613 | 0.00113166 | TRUE |
| ZNF521 | 9870 | 3.772166 | 0.0001618 | 0.00113402 | TRUE |
| SH3GLB1 | 1661 | 3.771793 | 0.0001621 | 0.0011347 | TRUE |
| BEX3 | 8532 | 3.77121 | 0.0001625 | 0.00113619 | TRUE |
| BCL7C | 8809 | 3.77113 | 0.0001625 | 0.00113619 | TRUE |
| SOX5 | 3304 | 3.765861 | 0.000166 | 0.00115577 | TRUE |
| NPY1R | 7037 | 3.765624 | 0.0001661 | 0.00115635 | TRUE |
| DIAPH3 | 13558 | 3.76218 | 0.0001684 | 0.00116875 | TRUE |
| MT1F | 6199 | 3.76142 | 0.000169 | 0.00117162 | TRUE |
| FABP3 | 1550 | 3.761345 | 0.000169 | 0.00117162 | TRUE |
| PTPRG | 3759 | 3.761208 | 0.0001691 | 0.00117175 | TRUE |
| PDRG1 | 14223 | 3.755448 | 0.000173 | 0.00119585 | TRUE |
| TENM1 | 4710 | 3.755091 | 0.0001733 | 0.00119702 | TRUE |
| HRH1 | 3560 | 3.742501 | 0.0001822 | 0.00124816 | TRUE |
| SLC1A4 | 13906 | 3.742002 | 0.0001826 | 0.00125009 | TRUE |
| SLC2A10 | 11415 | 3.741753 | 0.0001827 | 0.00125078 | TRUE |
| SLC2A4RG | 14547 | 3.740815 | 0.0001834 | 0.00125436 | TRUE |
| C1orf226 | 11490 | 3.736428 | 0.0001867 | 0.00127477 | TRUE |
| GTF3C6 | 11324 | 3.734835 | 0.0001878 | 0.00128174 | TRUE |
| ZCCHC12 | 2250 | 3.734712 | 0.0001879 | 0.00128181 | TRUE |
| PDLIM5 | 5788 | 3.733936 | 0.0001885 | 0.00128521 | TRUE |
| NECAB2 | 8284 | 3.732137 | 0.0001899 | 0.0012933 | TRUE |
| EEF1AKMT1 | 5860 | 3.729593 | 0.0001918 | 0.00130472 | TRUE |
| PROCA1 | 14460 | 3.724916 | 0.0001954 | 0.00132569 | TRUE |
| SUMO2 | 12056 | 3.723049 | 0.0001968 | 0.00133383 | TRUE |
| CC2D2A | 6284 | 3.723042 | 0.0001968 | 0.00133383 | TRUE |
| DOC2A | 479 | 3.721686 | 0.0001979 | 0.00133927 | TRUE |
| LOC93622 | 13092 | 3.715878 | 0.0002025 | 0.00136628 | TRUE |
| ANXA11 | 10483 | 3.715025 | 0.0002032 | 0.00136948 | TRUE |
| SPATA18 | 7222 | 3.714769 | 0.0002034 | 0.00136948 | TRUE |
| MLNR | 11064 | 3.712951 | 0.0002049 | 0.00137862 | TRUE |
| ARF3 | 1243 | 3.712741 | 0.000205 | 0.00137917 | TRUE |
| SNRPD3 | 302 | 3.71252 | 0.0002052 | 0.00137978 | TRUE |
| BCL11B | 11635 | 3.708929 | 0.0002081 | 0.00139589 | TRUE |
| CBFB | 3666 | 3.704646 | 0.0002117 | 0.00141544 | TRUE |
| MORN4 | 2077 | 3.704476 | 0.0002118 | 0.00141578 | TRUE |
| MINOS1 | 3799 | 3.703705 | 0.0002125 | 0.00141888 | TRUE |
| CLDND2 | 9830 | 3.701076 | 0.0002147 | 0.0014325 | TRUE |
| COG5 | 11344 | 3.701065 | 0.0002147 | 0.0014325 | TRUE |
| SOD1 | 8394 | 3.699256 | 0.0002162 | 0.00144152 | TRUE |
| SLC27A2 | 1688 | 3.698937 | 0.0002165 | 0.00144272 | TRUE |
| MAGEH1 | 8604 | 3.698272 | 0.0002171 | 0.00144588 | TRUE |
| FN3KRP | 14147 | 3.695088 | 0.0002198 | 0.00146163 | TRUE |
| RYR3 | 710 | 3.693952 | 0.0002208 | 0.00146756 | TRUE |
| KRT31 | 7863 | 3.693776 | 0.0002209 | 0.00146795 | TRUE |
| RAC1 | 9106 | 3.693029 | 0.0002216 | 0.00147102 | TRUE |
| PRMT6 | 4801 | 3.692351 | 0.0002222 | 0.00147314 | TRUE |
| SMAD1 | 4655 | 3.692081 | 0.0002224 | 0.00147339 | TRUE |
| PPCS | 12575 | 3.691664 | 0.0002228 | 0.00147518 | TRUE |
| PLXNC1 | 13445 | 3.690951 | 0.0002234 | 0.00147845 | TRUE |
| MAMLD1 | 2757 | 3.689202 | 0.000225 | 0.00148764 | TRUE |
| SLC24A3 | 14982 | 3.688991 | 0.0002251 | 0.00148824 | TRUE |
| RARS | 12515 | 3.68847 | 0.0002256 | 0.00149066 | TRUE |
| TSR3 | 11817 | 3.687002 | 0.0002269 | 0.00149802 | TRUE |
| ECHS1 | 14539 | 3.686372 | 0.0002275 | 0.0015011 | TRUE |
| HGH1 | 10821 | 3.685996 | 0.0002278 | 0.00150268 | TRUE |
| KLHDC8A | 8784 | 3.683873 | 0.0002297 | 0.00151271 | TRUE |
| RGMB | 5154 | 3.682969 | 0.0002305 | 0.00151744 | TRUE |
| KCNQ1 | 103 | 3.682652 | 0.0002308 | 0.00151869 | TRUE |
| PXMP4 | 8865 | 3.681942 | 0.0002315 | 0.00152167 | TRUE |
| RAN | 5445 | 3.681939 | 0.0002315 | 0.00152167 | TRUE |
| MUM1L1 | 8701 | 3.68128 | 0.0002321 | 0.00152432 | TRUE |
| BAIAP2 | 9509 | 3.672088 | 0.0002406 | 0.00157427 | TRUE |
| GPX4 | 7182 | 3.668437 | 0.000244 | 0.00159602 | TRUE |
| TGFBI | 2098 | 3.666627 | 0.0002458 | 0.00160423 | TRUE |
| TTYH1 | 14700 | 3.666327 | 0.0002461 | 0.00160544 | TRUE |
| KCNK2 | 3063 | 3.664059 | 0.0002483 | 0.00161716 | TRUE |
| CCKBR | 2319 | 3.662468 | 0.0002498 | 0.00162644 | TRUE |
| LCP1 | 1602 | 3.661978 | 0.0002503 | 0.00162837 | TRUE |
| CELF4 | 1606 | 3.659749 | 0.0002525 | 0.00164074 | TRUE |
| OTUB2 | 11331 | 3.659692 | 0.0002525 | 0.00164074 | TRUE |
| FAM86B3P | 6630 | 3.657819 | 0.0002544 | 0.00165003 | TRUE |
| UXT | 14197 | 3.657517 | 0.0002547 | 0.00165061 | TRUE |
| ARHGAP5-AS1 | 4589 | 3.656687 | 0.0002555 | 0.00165527 | TRUE |
| PSMA6 | 10317 | 3.654222 | 0.000258 | 0.00167016 | TRUE |
| NDUFA6-AS1 | 10479 | 3.6516 | 0.0002606 | 0.00168423 | TRUE |
| CCDC68 | 4752 | 3.650609 | 0.0002616 | 0.00168934 | TRUE |
| EDF1 | 3876 | 3.650153 | 0.0002621 | 0.00169095 | TRUE |
| LAPTM5 | 11568 | 3.649691 | 0.0002626 | 0.00169259 | TRUE |
| P2RX5 | 4321 | 3.649406 | 0.0002628 | 0.00169377 | TRUE |
| LINC01547 | 9994 | 3.649068 | 0.0002632 | 0.0016953 | TRUE |
| SLC39A4 | 8113 | 3.648102 | 0.0002642 | 0.00170099 | TRUE |
| OTOF | 4619 | 3.645976 | 0.0002664 | 0.0017137 | TRUE |
| CYP46A1 | 2616 | 3.645691 | 0.0002667 | 0.00171419 | TRUE |
| NPM1 | 10730 | 3.644711 | 0.0002677 | 0.00171862 | TRUE |
| L3MBTL4 | 2965 | 3.644277 | 0.0002681 | 0.00172081 | TRUE |
| PYDC1 | 13980 | 3.640232 | 0.0002724 | 0.00174664 | TRUE |
| DCUN1D5 | 15280 | 3.640061 | 0.0002726 | 0.00174709 | TRUE |
| PSMG3-AS1 | 10274 | 3.639915 | 0.0002727 | 0.00174736 | TRUE |
| ARL10 | 8404 | 3.639012 | 0.0002737 | 0.00175206 | TRUE |
| FAM184A | 3276 | 3.63842 | 0.0002743 | 0.00175537 | TRUE |
| RPS19 | 2450 | 3.636584 | 0.0002763 | 0.00176648 | TRUE |
| C9orf116 | 2611 | 3.635648 | 0.0002773 | 0.00176908 | TRUE |
| ORC4 | 39 | 3.632091 | 0.0002811 | 0.00179021 | TRUE |
| HIST1H2BM | 14187 | 3.629323 | 0.0002842 | 0.00180731 | TRUE |
| COA3 | 562 | 3.628264 | 0.0002853 | 0.00181179 | TRUE |
| FHOD3 | 3822 | 3.627929 | 0.0002857 | 0.00181218 | TRUE |
| NTNG2 | 2920 | 3.627894 | 0.0002857 | 0.00181218 | TRUE |
| FCGRT | 12337 | 3.626161 | 0.0002877 | 0.00182216 | TRUE |
| HMGCR | 14024 | 3.624807 | 0.0002892 | 0.00183025 | TRUE |
| MIF-AS1 | 4105 | 3.622376 | 0.0002919 | 0.0018453 | TRUE |
| COPE | 6865 | 3.620163 | 0.0002944 | 0.0018589 | TRUE |
| PTGR2 | 8616 | 3.618323 | 0.0002965 | 0.0018699 | TRUE |
| UBE3D | 9982 | 3.617747 | 0.0002972 | 0.00187255 | TRUE |
| DDX54 | 357 | 3.616176 | 0.000299 | 0.00188243 | TRUE |
| GBA | 9612 | 3.615811 | 0.0002994 | 0.00188432 | TRUE |
| NTSR2 | 13609 | 3.614332 | 0.0003011 | 0.00189359 | TRUE |
| DNAH14 | 6034 | 3.613962 | 0.0003016 | 0.00189477 | TRUE |
| NELL2 | 8260 | 3.60988 | 0.0003063 | 0.00192098 | TRUE |
| NOV | 9060 | 3.607818 | 0.0003088 | 0.0019332 | TRUE |
| EDNRB | 12881 | 3.604622 | 0.0003126 | 0.00195246 | TRUE |
| MED24 | 11092 | 3.602197 | 0.0003155 | 0.00196841 | TRUE |
| JAML | 7150 | 3.599941 | 0.0003183 | 0.00198398 | TRUE |
| DAP3 | 9099 | 3.599656 | 0.0003186 | 0.00198536 | TRUE |
| TRIM27 | 12878 | 3.595248 | 0.0003241 | 0.00201687 | TRUE |
| TNNT1 | 2627 | 3.593427 | 0.0003264 | 0.00202812 | TRUE |
| ELOB | 5684 | 3.593282 | 0.0003265 | 0.00202812 | TRUE |
| COL23A1 | 11356 | 3.591863 | 0.0003283 | 0.00203758 | TRUE |
| PSMB6 | 2854 | 3.590761 | 0.0003297 | 0.00204459 | TRUE |
| GNG4 | 13951 | 3.590595 | 0.0003299 | 0.00204508 | TRUE |
| CRYZ | 2389 | 3.590412 | 0.0003302 | 0.00204571 | TRUE |
| GSTM2 | 9335 | 3.588668 | 0.0003324 | 0.00205699 | TRUE |
| EBNA1BP2 | 14594 | 3.588119 | 0.0003331 | 0.00206039 | TRUE |
| HLA-DMA | 10242 | 3.587306 | 0.0003341 | 0.00206484 | TRUE |
| FHOD1 | 73 | 3.585873 | 0.000336 | 0.00207341 | TRUE |
| GNG3 | 8268 | 3.583435 | 0.0003391 | 0.00209122 | TRUE |
| ISG15 | 10060 | 3.582787 | 0.0003399 | 0.00209476 | TRUE |
| LOC644936 | 5943 | 3.581195 | 0.000342 | 0.00210593 | TRUE |
| C1QC | 5609 | 3.581192 | 0.000342 | 0.00210593 | TRUE |
| UFC1 | 1422 | 3.577959 | 0.0003463 | 0.00212712 | TRUE |
| WNT4 | 10635 | 3.575715 | 0.0003493 | 0.00214125 | TRUE |
| MRPL37 | 3547 | 3.575702 | 0.0003493 | 0.00214125 | TRUE |
| HPCAL1 | 11458 | 3.575612 | 0.0003494 | 0.00214125 | TRUE |
| TMEM234 | 5265 | 3.57352 | 0.0003522 | 0.00215504 | TRUE |
| BRD7 | 11991 | 3.573419 | 0.0003524 | 0.00215504 | TRUE |
| TNFRSF11A | 2124 | 3.571721 | 0.0003546 | 0.00216822 | TRUE |
| GPM6A | 7886 | 3.570962 | 0.0003557 | 0.00217366 | TRUE |
| DUSP28 | 9300 | 3.570199 | 0.0003567 | 0.00217915 | TRUE |
| TMEM59L | 14889 | 3.568559 | 0.000359 | 0.00219198 | TRUE |
| TPI1 | 8547 | 3.568414 | 0.0003591 | 0.00219234 | TRUE |
| METTL5 | 13211 | 3.564751 | 0.0003642 | 0.00221712 | TRUE |
| GINS2 | 1033 | 3.562369 | 0.0003675 | 0.00223637 | TRUE |
| SSC4D | 5858 | 3.562 | 0.000368 | 0.00223751 | TRUE |
| NPC2 | 5365 | 3.561941 | 0.0003681 | 0.00223751 | TRUE |
| ABCC6 | 4523 | 3.561634 | 0.0003686 | 0.00223926 | TRUE |
| THBS4 | 45 | 3.560404 | 0.0003703 | 0.0022489 | TRUE |
| ZMYND10 | 7969 | 3.555912 | 0.0003767 | 0.00228147 | TRUE |
| MILR1 | 1265 | 3.555651 | 0.000377 | 0.00228286 | TRUE |
| CISD3 | 14409 | 3.554777 | 0.0003783 | 0.00228894 | TRUE |
| NUDT17 | 11972 | 3.554748 | 0.0003783 | 0.00228894 | TRUE |
| NT5DC2 | 14542 | 3.553639 | 0.0003799 | 0.00229683 | TRUE |
| MCTP1 | 3168 | 3.553361 | 0.0003803 | 0.00229837 | TRUE |
| PDCD5 | 4110 | 3.551212 | 0.0003835 | 0.00231454 | TRUE |
| IQCG | 10181 | 3.550516 | 0.0003845 | 0.00231798 | TRUE |
| MGAT4B | 12047 | 3.549983 | 0.0003853 | 0.00232089 | TRUE |
| SOBP | 443 | 3.548842 | 0.0003869 | 0.00233004 | TRUE |
| JARID2-AS1 | 3215 | 3.548643 | 0.0003872 | 0.00233004 | TRUE |
| TPX2 | 5483 | 3.546812 | 0.0003899 | 0.00234449 | TRUE |
| LPAR6 | 278 | 3.546056 | 0.000391 | 0.00235032 | TRUE |
| ST6GALNAC5 | 2678 | 3.545567 | 0.0003918 | 0.00235378 | TRUE |
| TSPAN18 | 8169 | 3.544616 | 0.0003932 | 0.00236138 | TRUE |
| PLPP4 | 5696 | 3.542843 | 0.0003958 | 0.00237457 | TRUE |
| SCIN | 11735 | 3.541745 | 0.0003975 | 0.00238174 | TRUE |
| CDH10 | 1264 | 3.540352 | 0.0003996 | 0.00239343 | TRUE |
| HLA-DRA | 181 | 3.539444 | 0.000401 | 0.00239864 | TRUE |
| IGFN1 | 12316 | 3.539374 | 0.0004011 | 0.00239864 | TRUE |
| CRADD | 13757 | 3.538943 | 0.0004017 | 0.00240164 | TRUE |
| SFTA1P | 12422 | 3.534023 | 0.0004093 | 0.00244119 | TRUE |
| DERL3 | 4280 | 3.533369 | 0.0004103 | 0.00244351 | TRUE |
| LOC101928111 | 14382 | 3.532926 | 0.000411 | 0.00244668 | TRUE |
| CNN3 | 9764 | 3.53209 | 0.0004123 | 0.00245349 | TRUE |
| HTR2C | 8095 | 3.529902 | 0.0004157 | 0.00247011 | TRUE |
| RPL35 | 11711 | 3.526742 | 0.0004207 | 0.00249647 | TRUE |
| PPIL6 | 10690 | 3.52669 | 0.0004208 | 0.00249647 | TRUE |
| MFSD10 | 10105 | 3.52524 | 0.0004231 | 0.00250827 | TRUE |
| PON3 | 12010 | 3.524593 | 0.0004241 | 0.00251345 | TRUE |
| AK8 | 10413 | 3.519888 | 0.0004317 | 0.00255362 | TRUE |
| CBLN1 | 3180 | 3.518274 | 0.0004344 | 0.00256435 | TRUE |
| C1R | 533 | 3.51429 | 0.0004409 | 0.0025965 | TRUE |
| HLA-DQB1 | 2900 | 3.514171 | 0.0004411 | 0.0025965 | TRUE |
| SAP18 | 7423 | 3.513806 | 0.0004417 | 0.00259709 | TRUE |
| SIVA1 | 10412 | 3.513584 | 0.0004421 | 0.0025973 | TRUE |
| HERC5 | 1710 | 3.511883 | 0.0004449 | 0.00261197 | TRUE |
| TTLL1 | 7175 | 3.511788 | 0.0004451 | 0.00261197 | TRUE |
| RSPH9 | 1145 | 3.510365 | 0.0004475 | 0.00262304 | TRUE |
| TLL1 | 625 | 3.510083 | 0.000448 | 0.00262484 | TRUE |
| ARHGDIA | 11299 | 3.506593 | 0.0004539 | 0.00265254 | TRUE |
| WWC1 | 2864 | 3.506303 | 0.0004544 | 0.00265444 | TRUE |
| CENPP | 3810 | 3.504779 | 0.000457 | 0.00266769 | TRUE |
| LAGE3 | 2186 | 3.502852 | 0.0004603 | 0.00268305 | TRUE |
| ZMAT1 | 7816 | 3.502395 | 0.0004611 | 0.00268566 | TRUE |
| CACNG3 | 11790 | 3.501822 | 0.0004621 | 0.00268844 | TRUE |
| TUBA4B | 2628 | 3.498713 | 0.0004675 | 0.00271677 | TRUE |
| LIN7B | 7273 | 3.495478 | 0.0004732 | 0.00274601 | TRUE |
| SFTPD | 4511 | 3.49534 | 0.0004735 | 0.00274641 | TRUE |
| FRMD3 | 7688 | 3.494982 | 0.0004741 | 0.00274833 | TRUE |
| OXTR | 8273 | 3.494955 | 0.0004741 | 0.00274833 | TRUE |
| LOC729970 | 7670 | 3.494349 | 0.0004752 | 0.00275254 | TRUE |
| SF3B6 | 9732 | 3.492242 | 0.000479 | 0.00277127 | TRUE |
| SNAP29 | 14441 | 3.492126 | 0.0004792 | 0.00277144 | TRUE |
| RPS8 | 5695 | 3.490515 | 0.0004821 | 0.00278306 | TRUE |
| SSTR1 | 8043 | 3.488943 | 0.0004849 | 0.00279535 | TRUE |
| VBP1 | 2890 | 3.48816 | 0.0004864 | 0.00280251 | TRUE |
| FLJ20021 | 163 | 3.487663 | 0.0004873 | 0.00280669 | TRUE |
| BTC | 13866 | 3.486604 | 0.0004892 | 0.00281589 | TRUE |
| TANGO6 | 11629 | 3.486591 | 0.0004892 | 0.00281589 | TRUE |
| KCTD15 | 1362 | 3.486357 | 0.0004896 | 0.00281732 | TRUE |
| SPRY1 | 1958 | 3.48615 | 0.00049 | 0.00281846 | TRUE |
| RYR1 | 1116 | 3.485754 | 0.0004908 | 0.0028216 | TRUE |
| MT1A | 6833 | 3.484796 | 0.0004925 | 0.00283068 | TRUE |
| FXYD6 | 4056 | 3.484619 | 0.0004928 | 0.00283151 | TRUE |
| TMEM65 | 10134 | 3.484142 | 0.0004937 | 0.00283552 | TRUE |
| ADGRL3 | 4107 | 3.483634 | 0.0004947 | 0.00283986 | TRUE |
| RPL39 | 8643 | 3.4828 | 0.0004962 | 0.00284663 | TRUE |
| CORT | 7055 | 3.481541 | 0.0004985 | 0.00285795 | TRUE |
| VPS35 | 13219 | 3.480962 | 0.0004996 | 0.00286282 | TRUE |
| MRC1 | 3305 | 3.480889 | 0.0004998 | 0.00286282 | TRUE |
| NPTXR | 4861 | 3.479915 | 0.0005016 | 0.00287009 | TRUE |
| TOMM20 | 6397 | 3.479168 | 0.000503 | 0.00287495 | TRUE |
| ERH | 13982 | 3.478693 | 0.0005039 | 0.00287899 | TRUE |
| TMEM107 | 383 | 3.476768 | 0.0005075 | 0.00289445 | TRUE |
| BCR | 10276 | 3.476465 | 0.0005081 | 0.00289667 | TRUE |
| KLF10 | 14241 | 3.473465 | 0.0005138 | 0.00292498 | TRUE |
| RABAC1 | 12959 | 3.472644 | 0.0005154 | 0.0029316 | TRUE |
| TSPAN2 | 4828 | 3.472565 | 0.0005155 | 0.0029316 | TRUE |
| AZIN1-AS1 | 5042 | 3.471261 | 0.000518 | 0.00294373 | TRUE |
| SCUBE2 | 13929 | 3.470452 | 0.0005196 | 0.00295154 | TRUE |
| DPH5 | 11391 | 3.468772 | 0.0005228 | 0.0029679 | TRUE |
| DOK4 | 5290 | 3.468561 | 0.0005233 | 0.00296916 | TRUE |
| KCTD4 | 6221 | 3.463385 | 0.0005334 | 0.00302358 | TRUE |
| EFHC2 | 12792 | 3.462981 | 0.0005342 | 0.00302702 | TRUE |
| DDN | 14559 | 3.462114 | 0.000536 | 0.0030324 | TRUE |
| HIST1H2BO | 11357 | 3.459579 | 0.000541 | 0.00305335 | TRUE |
| CHPF2 | 3987 | 3.455708 | 0.0005489 | 0.00309195 | TRUE |
| FBXO15 | 514 | 3.454518 | 0.0005513 | 0.00310339 | TRUE |
| PSD3 | 11945 | 3.452565 | 0.0005553 | 0.00312257 | TRUE |
| GPNMB | 5536 | 3.446259 | 0.0005684 | 0.00318605 | TRUE |
| HSPB11 | 5721 | 3.444764 | 0.0005716 | 0.00320142 | TRUE |
| NUPR2 | 6027 | 3.442988 | 0.0005753 | 0.00321791 | TRUE |
| RBFA | 4592 | 3.442699 | 0.0005759 | 0.0032202 | TRUE |
| LOC102724156 | 11157 | 3.442256 | 0.0005769 | 0.00322432 | TRUE |
| IFT27 | 15094 | 3.44102 | 0.0005795 | 0.00323677 | TRUE |
| TP53I11 | 6975 | 3.440809 | 0.00058 | 0.00323787 | TRUE |
| VPS29 | 12608 | 3.438548 | 0.0005848 | 0.00325678 | TRUE |
| FAU | 9291 | 3.438483 | 0.000585 | 0.00325678 | TRUE |
| GNAQ | 13543 | 3.435471 | 0.0005915 | 0.00329086 | TRUE |
| TPI1P2 | 1576 | 3.434851 | 0.0005929 | 0.00329722 | TRUE |
| SLC39A12 | 6291 | 3.434484 | 0.0005937 | 0.00329934 | TRUE |
| SH3PXD2B | 11503 | 3.434036 | 0.0005947 | 0.00330245 | TRUE |
| MTHFD1 | 1340 | 3.433792 | 0.0005952 | 0.00330425 | TRUE |
| GPI | 292 | 3.433308 | 0.0005963 | 0.00330898 | TRUE |
| VOPP1 | 8132 | 3.429257 | 0.0006052 | 0.00335282 | TRUE |
| ZC3H15 | 6592 | 3.427895 | 0.0006083 | 0.00336611 | TRUE |
| BAIAP2L2 | 6837 | 3.426368 | 0.0006117 | 0.0033803 | TRUE |
| LHX6 | 289 | 3.422462 | 0.0006206 | 0.00342078 | TRUE |
| CDC42EP4 | 12987 | 3.421947 | 0.0006217 | 0.00342485 | TRUE |
| RNF2 | 3326 | 3.421849 | 0.000622 | 0.00342488 | TRUE |
| CYHR1 | 12138 | 3.421249 | 0.0006233 | 0.00343124 | TRUE |
| CABP7 | 10546 | 3.421017 | 0.0006239 | 0.00343296 | TRUE |
| TNFAIP8L1 | 1432 | 3.420898 | 0.0006241 | 0.00343325 | TRUE |
| ACOT13 | 13868 | 3.419505 | 0.0006274 | 0.00344724 | TRUE |
| MYBPC1 | 11851 | 3.419231 | 0.000628 | 0.00344829 | TRUE |
| JPH4 | 6430 | 3.416884 | 0.0006334 | 0.00347449 | TRUE |
| MRPS16 | 6298 | 3.416755 | 0.0006337 | 0.00347492 | TRUE |
| SAP30BP | 2113 | 3.416405 | 0.0006345 | 0.00347817 | TRUE |
| GSTO2 | 2338 | 3.416053 | 0.0006354 | 0.00348145 | TRUE |
| DCTN3 | 1596 | 3.414121 | 0.0006399 | 0.00350255 | TRUE |
| PLEKHA4 | 4514 | 3.41295 | 0.0006426 | 0.0035164 | TRUE |
| NAV3 | 9272 | 3.408008 | 0.0006544 | 0.00357445 | TRUE |
| FAM187A | 11204 | 3.406947 | 0.0006569 | 0.00358712 | TRUE |
| FDFT1 | 4905 | 3.405657 | 0.0006601 | 0.00360131 | TRUE |
| SEMA3D | 11572 | 3.405298 | 0.0006609 | 0.00360131 | TRUE |
| CDH11 | 1780 | 3.396947 | 0.0006814 | 0.00369628 | TRUE |
| SMARCA4 | 10832 | 3.396771 | 0.0006819 | 0.00369737 | TRUE |
| MAST3 | 5908 | 3.392635 | 0.0006922 | 0.00374455 | TRUE |
| LOC645166 | 3734 | 3.392405 | 0.0006928 | 0.0037464 | TRUE |
| SLC1A6 | 12817 | 3.391262 | 0.0006957 | 0.00375816 | TRUE |
| PLCG2 | 5582 | 3.389659 | 0.0006998 | 0.0037789 | TRUE |
| ARHGAP12 | 1559 | 3.387293 | 0.0007059 | 0.00380638 | TRUE |
| SBF1P1 | 4356 | 3.382676 | 0.0007178 | 0.00386561 | TRUE |
| MYRIP | 13839 | 3.381935 | 0.0007198 | 0.00387339 | TRUE |
| ASL | 11387 | 3.381575 | 0.0007207 | 0.00387713 | TRUE |
| NPY | 10615 | 3.38138 | 0.0007212 | 0.00387855 | TRUE |
| ITGAM | 14758 | 3.379641 | 0.0007258 | 0.00389647 | TRUE |
| MRPS21 | 9240 | 3.379392 | 0.0007265 | 0.00389866 | TRUE |
| PPP5D1 | 5834 | 3.379285 | 0.0007267 | 0.00389884 | TRUE |
| EIF3H | 1732 | 3.378359 | 0.0007292 | 0.00390797 | TRUE |
| FOLR1 | 3563 | 3.37263 | 0.0007445 | 0.00397656 | TRUE |
| EEF1G | 5045 | 3.372223 | 0.0007456 | 0.00397955 | TRUE |
| CDKL5 | 10047 | 3.372141 | 0.0007459 | 0.00397955 | TRUE |
| RGS22 | 14381 | 3.371042 | 0.0007488 | 0.00399409 | TRUE |
| EMC7 | 8272 | 3.369268 | 0.0007537 | 0.00401715 | TRUE |
| MAPKAPK3 | 10125 | 3.368952 | 0.0007545 | 0.00401902 | TRUE |
| MORN5 | 15375 | 3.36794 | 0.0007573 | 0.00403069 | TRUE |
| FAM212A | 4220 | 3.366998 | 0.0007599 | 0.00404179 | TRUE |
| HS2ST1 | 10616 | 3.366831 | 0.0007604 | 0.00404179 | TRUE |
| TOMM5 | 11686 | 3.363713 | 0.000769 | 0.00407941 | TRUE |
| HIST1H2BH | 5923 | 3.363507 | 0.0007696 | 0.00408107 | TRUE |
| MEIG1 | 11308 | 3.362017 | 0.0007738 | 0.0040992 | TRUE |
| NIPSNAP3A | 3400 | 3.355713 | 0.0007916 | 0.0041794 | TRUE |
| KIF2A | 10844 | 3.355389 | 0.0007925 | 0.00418288 | TRUE |
| DPYSL5 | 4161 | 3.355119 | 0.0007933 | 0.00418549 | TRUE |
| GABARAPL2 | 6156 | 3.354215 | 0.0007959 | 0.00419427 | TRUE |
| BLOC1S1 | 7629 | 3.352471 | 0.0008009 | 0.00421584 | TRUE |
| WDR13 | 10315 | 3.351723 | 0.0008031 | 0.00422582 | TRUE |
| NSDHL | 15143 | 3.34948 | 0.0008096 | 0.00425732 | TRUE |
| TATDN1 | 8459 | 3.34866 | 0.000812 | 0.00426631 | TRUE |
| POLR2J | 61 | 3.347629 | 0.0008151 | 0.00427722 | TRUE |
| UG0898H09 | 598 | 3.34752 | 0.0008154 | 0.00427747 | TRUE |
| DOK1 | 13702 | 3.345445 | 0.0008215 | 0.00430382 | TRUE |
| BCS1L | 8300 | 3.34496 | 0.0008229 | 0.00430847 | TRUE |
| MYDGF | 3131 | 3.343878 | 0.0008262 | 0.00432241 | TRUE |
| MR1 | 13654 | 3.342246 | 0.000831 | 0.00434355 | TRUE |
| PEG10 | 1646 | 3.341745 | 0.0008325 | 0.00434849 | TRUE |
| CCIN | 1599 | 3.339649 | 0.0008388 | 0.00437267 | TRUE |
| SV2B | 6236 | 3.338441 | 0.0008425 | 0.00438588 | TRUE |
| ACVR2A | 14317 | 3.335326 | 0.000852 | 0.00442499 | TRUE |
| NDUFAF1 | 13343 | 3.334578 | 0.0008543 | 0.00443536 | TRUE |
| LOC729683 | 11815 | 3.334459 | 0.0008547 | 0.00443536 | TRUE |
| PTDSS1 | 7853 | 3.333026 | 0.0008591 | 0.00445139 | TRUE |
| RASGEF1A | 4600 | 3.332848 | 0.0008596 | 0.00445276 | TRUE |
| PHYHD1 | 5366 | 3.332671 | 0.0008602 | 0.00445412 | TRUE |
| LDHA | 12844 | 3.332289 | 0.0008613 | 0.00445876 | TRUE |
| DUSP23 | 3448 | 3.331322 | 0.0008643 | 0.00447132 | TRUE |
| TMEM120A | 14742 | 3.330612 | 0.0008666 | 0.00447977 | TRUE |
| CCDC28A | 7973 | 3.33013 | 0.0008681 | 0.00448605 | TRUE |
| PHF24 | 3470 | 3.329103 | 0.0008713 | 0.00450114 | TRUE |
| FLJ33534 | 10258 | 3.328835 | 0.0008721 | 0.00450398 | TRUE |
| PSMD8 | 14580 | 3.328078 | 0.0008745 | 0.00451475 | TRUE |
| ASPHD2 | 13565 | 3.326314 | 0.00088 | 0.00453742 | TRUE |
| KPNA4 | 8275 | 3.323396 | 0.0008893 | 0.00457706 | TRUE |
| BBS4 | 424 | 3.323279 | 0.0008897 | 0.00457706 | TRUE |
| PTER | 11803 | 3.321722 | 0.0008946 | 0.00459894 | TRUE |
| SNRPD2 | 5823 | 3.321073 | 0.0008967 | 0.00460678 | TRUE |
| RAP1B | 12291 | 3.320845 | 0.0008975 | 0.00460903 | TRUE |
| ONECUT1 | 8853 | 3.314714 | 0.0009174 | 0.00470359 | TRUE |
| 3-Mar | 8278 | 3.314114 | 0.0009193 | 0.00471215 | TRUE |
| ITGB1BP1 | 12403 | 3.311084 | 0.0009294 | 0.00475723 | TRUE |
| GHR | 5451 | 3.310732 | 0.0009305 | 0.00475955 | TRUE |
| ARMCX1 | 11305 | 3.31049 | 0.0009313 | 0.00475955 | TRUE |
| BCAS2 | 8611 | 3.309296 | 0.0009353 | 0.00477529 | TRUE |
| C1QL1 | 11328 | 3.308724 | 0.0009372 | 0.00478341 | TRUE |
| SLC26A4 | 2419 | 3.308498 | 0.000938 | 0.00478571 | TRUE |
| CGREF1 | 2182 | 3.305841 | 0.0009469 | 0.00482031 | TRUE |
| PCBP3 | 1399 | 3.302712 | 0.0009575 | 0.00486257 | TRUE |
| FAM120C | 5810 | 3.302066 | 0.0009598 | 0.0048698 | TRUE |
| LINC00900 | 2129 | 3.301396 | 0.0009621 | 0.00487828 | TRUE |
| NADK2 | 15077 | 3.300524 | 0.000965 | 0.00489188 | TRUE |
| SERINC1 | 14778 | 3.29629 | 0.0009797 | 0.00495817 | TRUE |
| RAPH1 | 4660 | 3.296063 | 0.0009805 | 0.00496057 | TRUE |
| TRIB1 | 12553 | 3.295482 | 0.0009825 | 0.00496923 | TRUE |
| DOLK | 4438 | 3.293828 | 0.0009883 | 0.00499533 | TRUE |
| LOC101060391 | 3222 | 3.293461 | 0.0009896 | 0.00500024 | TRUE |
| APLN | 1556 | 3.290673 | 0.0009995 | 0.00503866 | TRUE |
| PLCD3 | 861 | 3.288569 | 0.001007 | 0.0050732 | TRUE |
| PRIM2 | 3363 | 3.287784 | 0.0010098 | 0.00508184 | TRUE |
| PEMT | 1529 | 3.287727 | 0.00101 | 0.00508184 | TRUE |
| RGS14 | 6631 | 3.287 | 0.0010126 | 0.00509234 | TRUE |
| CYB561 | 6536 | 3.285521 | 0.0010179 | 0.00510865 | TRUE |
| TSEN34 | 13603 | 3.28398 | 0.0010235 | 0.00512844 | TRUE |
| BRINP3 | 11333 | 3.283426 | 0.0010255 | 0.00513359 | TRUE |
| ZNF544 | 2592 | 3.279132 | 0.0010413 | 0.00519757 | TRUE |
| KCNJ5 | 11259 | 3.271898 | 0.0010683 | 0.00530678 | TRUE |
| TBX19 | 6223 | 3.270999 | 0.0010717 | 0.00532199 | TRUE |
| DCAF15 | 3566 | 3.266675 | 0.0010882 | 0.00538172 | TRUE |
| WDR1 | 11946 | 3.266109 | 0.0010904 | 0.00538907 | TRUE |
| SOX9-AS1 | 3904 | 3.264778 | 0.0010955 | 0.00540933 | TRUE |
| TYMP | 1524 | 3.264563 | 0.0010963 | 0.00541173 | TRUE |
| SF3B5 | 10766 | 3.26142 | 0.0011086 | 0.0054669 | TRUE |
| CRNDE | 3730 | 3.260149 | 0.0011135 | 0.005488 | TRUE |
| TTC7B | 14105 | 3.256448 | 0.0011282 | 0.0055448 | TRUE |
| FTH1 | 8444 | 3.256056 | 0.0011297 | 0.00555023 | TRUE |
| CYBB | 4657 | 3.255021 | 0.0011338 | 0.00556875 | TRUE |
| RPL38 | 6052 | 3.253715 | 0.0011391 | 0.0055909 | TRUE |
| RAP1GAP | 8039 | 3.253354 | 0.0011405 | 0.00559581 | TRUE |
| FAM49A | 8664 | 3.252145 | 0.0011454 | 0.00561482 | TRUE |
| LAT2 | 10330 | 3.251355 | 0.0011486 | 0.00562868 | TRUE |
| NDUFA13 | 11395 | 3.250488 | 0.0011521 | 0.00564057 | TRUE |
| KCNK3 | 10728 | 3.249597 | 0.0011557 | 0.00565441 | TRUE |
| CCDC3 | 9603 | 3.249524 | 0.001156 | 0.00565441 | TRUE |
| SIGIRR | 6901 | 3.246989 | 0.0011663 | 0.00569966 | TRUE |
| OAZ1 | 4863 | 3.237532 | 0.0012057 | 0.00587362 | TRUE |
| ABHD1 | 387 | 3.236175 | 0.0012114 | 0.00589654 | TRUE |
| EFNA2 | 3256 | 3.234375 | 0.0012191 | 0.00592971 | TRUE |
| LOC389895 | 3002 | 3.233978 | 0.0012208 | 0.00593242 | TRUE |
| FIBIN | 10496 | 3.233793 | 0.0012216 | 0.00593442 | TRUE |
| CCNH | 7246 | 3.232114 | 0.0012288 | 0.00596384 | TRUE |
| ANKRD50 | 6025 | 3.231015 | 0.0012335 | 0.00598173 | TRUE |
| RSPH1 | 1531 | 3.231012 | 0.0012335 | 0.00598173 | TRUE |
| 1-Mar | 2987 | 3.228568 | 0.0012441 | 0.00602517 | TRUE |
| VPS26B | 11973 | 3.22848 | 0.0012445 | 0.00602517 | TRUE |
| ASS1 | 7071 | 3.22556 | 0.0012573 | 0.00607756 | TRUE |
| OAZ3 | 414 | 3.223612 | 0.0012658 | 0.00611088 | TRUE |
| IDH2 | 10012 | 3.22361 | 0.0012659 | 0.00611088 | TRUE |
| MIR7-3HG | 15091 | 3.223552 | 0.0012661 | 0.00611088 | TRUE |
| CDKN2AIPNL | 3084 | 3.223081 | 0.0012682 | 0.00611905 | TRUE |
| CHMP2A | 12812 | 3.222359 | 0.0012714 | 0.0061326 | TRUE |
| DMAC1 | 11866 | 3.221265 | 0.0012763 | 0.00615226 | TRUE |
| ACSF2 | 12165 | 3.218155 | 0.0012902 | 0.00620597 | TRUE |
| CSGALNACT1 | 4442 | 3.217543 | 0.0012929 | 0.00621158 | TRUE |
| DMGDH | 4515 | 3.217367 | 0.0012937 | 0.00621348 | TRUE |
| SEC61G | 2208 | 3.216027 | 0.0012998 | 0.00623682 | TRUE |
| TXLNB | 512 | 3.213337 | 0.001312 | 0.00628974 | TRUE |
| TMEM136 | 5095 | 3.211367 | 0.0013211 | 0.0063239 | TRUE |
| RPL7 | 7665 | 3.211341 | 0.0013212 | 0.0063239 | TRUE |
| GALNT16 | 11281 | 3.209646 | 0.001329 | 0.00635936 | TRUE |
| RPS27 | 11337 | 3.20749 | 0.001339 | 0.00639921 | TRUE |
| RTL8A | 12494 | 3.20741 | 0.0013394 | 0.00639921 | TRUE |
| WDR7 | 1491 | 3.20689 | 0.0013418 | 0.00640883 | TRUE |
| IL33 | 995 | 3.205861 | 0.0013466 | 0.00642786 | TRUE |
| RGS10 | 13003 | 3.203891 | 0.0013558 | 0.00646608 | TRUE |
| CDC34 | 13300 | 3.20316 | 0.0013593 | 0.00647857 | TRUE |
| RPS3A | 8239 | 3.202017 | 0.0013647 | 0.00649838 | TRUE |
| LIMD2 | 10535 | 3.201402 | 0.0013676 | 0.00651029 | TRUE |
| IKBIP | 3846 | 3.200656 | 0.0013712 | 0.00652319 | TRUE |
| HIST1H2BK | 3894 | 3.199879 | 0.0013749 | 0.00653881 | TRUE |
| IDI1 | 10806 | 3.199386 | 0.0013772 | 0.00654801 | TRUE |
| PRKAA2 | 11811 | 3.198432 | 0.0013818 | 0.00656373 | TRUE |
| LOC101927752 | 11929 | 3.195498 | 0.0013959 | 0.00662546 | TRUE |
| AP3B2 | 14885 | 3.194434 | 0.0014011 | 0.00664436 | TRUE |
| APOE | 2056 | 3.192422 | 0.0014109 | 0.00668358 | TRUE |
| MAF | 3146 | 3.191114 | 0.0014173 | 0.00670985 | TRUE |
| PBX4 | 5354 | 3.189077 | 0.0014273 | 0.00674914 | TRUE |
| MAML2 | 111 | 3.188706 | 0.0014291 | 0.00675372 | TRUE |
| LINC01410 | 13310 | 3.18593 | 0.0014429 | 0.00680651 | TRUE |
| LCMT1 | 3191 | 3.185155 | 0.0014468 | 0.00682271 | TRUE |
| HNRNPC | 2510 | 3.181591 | 0.0014647 | 0.00689681 | TRUE |
| RSU1 | 5808 | 3.176809 | 0.0014891 | 0.00699679 | TRUE |
| PMF1 | 12607 | 3.176363 | 0.0014913 | 0.00700335 | TRUE |
| TRIM36 | 1289 | 3.176087 | 0.0014928 | 0.00700791 | TRUE |
| IPO5P1 | 4208 | 3.175107 | 0.0014978 | 0.00702951 | TRUE |
| MDK | 4428 | 3.175006 | 0.0014983 | 0.00702985 | TRUE |
| SPARC | 10131 | 3.174694 | 0.0014999 | 0.0070353 | TRUE |
| NPDC1 | 4854 | 3.174403 | 0.0015015 | 0.00703813 | TRUE |
| PRDM12 | 6248 | 3.170083 | 0.001524 | 0.00713079 | TRUE |
| C8orf58 | 3946 | 3.169329 | 0.0015279 | 0.00714504 | TRUE |
| MAPK7 | 8625 | 3.167883 | 0.0015355 | 0.00717423 | TRUE |
| WFS1 | 5789 | 3.166889 | 0.0015408 | 0.00719235 | TRUE |
| BLCAP | 9725 | 3.166484 | 0.0015429 | 0.00719694 | TRUE |
| RAB3C | 8852 | 3.165342 | 0.001549 | 0.00722208 | TRUE |
| PCDH10 | 10749 | 3.16374 | 0.0015576 | 0.00725762 | TRUE |
| ZFP69 | 7903 | 3.161551 | 0.0015693 | 0.00729734 | TRUE |
| KRT83 | 14314 | 3.159913 | 0.0015782 | 0.007329 | TRUE |
| TMEM54 | 3686 | 3.158303 | 0.0015869 | 0.00736364 | TRUE |
| TCTEX1D2 | 1139 | 3.157808 | 0.0015896 | 0.00736959 | TRUE |
| TOM1L1 | 4909 | 3.156015 | 0.0015994 | 0.00740845 | TRUE |
| ZSCAN5A | 10338 | 3.155523 | 0.0016021 | 0.00741538 | TRUE |
| LOC105378853 | 12043 | 3.154184 | 0.0016095 | 0.00744406 | TRUE |
| VANGL2 | 6784 | 3.154021 | 0.0016104 | 0.00744602 | TRUE |
| LY86 | 413 | 3.153827 | 0.0016114 | 0.00744877 | TRUE |
| SBF2 | 8754 | 3.152768 | 0.0016173 | 0.00747142 | TRUE |
| FAM129A | 8199 | 3.152428 | 0.0016192 | 0.00747792 | TRUE |
| CARD16 | 609 | 3.15161 | 0.0016237 | 0.00749668 | TRUE |
| TPPP3 | 8195 | 3.150042 | 0.0016325 | 0.00753036 | TRUE |
| FZD2 | 1793 | 3.149072 | 0.0016379 | 0.00755095 | TRUE |
| IL13RA1 | 9277 | 3.147005 | 0.0016495 | 0.00759334 | TRUE |
| C8orf82 | 6152 | 3.145581 | 0.0016576 | 0.00761694 | TRUE |
| ABCB6 | 9933 | 3.145368 | 0.0016588 | 0.00762025 | TRUE |
| SLC25A23 | 1520 | 3.144773 | 0.0016622 | 0.00763352 | TRUE |
| COG1 | 3230 | 3.143439 | 0.0016698 | 0.0076639 | TRUE |
| UHMK1 | 11756 | 3.143123 | 0.0016716 | 0.00766992 | TRUE |
| NDUFAB1 | 5870 | 3.142914 | 0.0016728 | 0.00767156 | TRUE |
| ARHGAP36 | 8732 | 3.14286 | 0.0016731 | 0.00767156 | TRUE |
| SLC4A4 | 9692 | 3.13994 | 0.0016898 | 0.00773559 | TRUE |
| NHP2 | 11312 | 3.1397 | 0.0016912 | 0.00773966 | TRUE |
| AKAP8 | 3133 | 3.134749 | 0.00172 | 0.00786457 | TRUE |
| PNPLA4 | 4456 | 3.13416 | 0.0017235 | 0.00787346 | TRUE |
| SRGAP1 | 4522 | 3.132223 | 0.0017349 | 0.00792328 | TRUE |
| TMEM9 | 9260 | 3.131611 | 0.0017385 | 0.00793749 | TRUE |
| FAM196A | 13380 | 3.131405 | 0.0017397 | 0.00794075 | TRUE |
| SSR2 | 13865 | 3.131257 | 0.0017406 | 0.00794243 | TRUE |
| IL34 | 5280 | 3.129885 | 0.0017487 | 0.00797264 | TRUE |
| PSEN2 | 14171 | 3.129764 | 0.0017495 | 0.0079736 | TRUE |
| LRRC27 | 3055 | 3.128011 | 0.0017599 | 0.00801196 | TRUE |
| TTPA | 11390 | 3.127742 | 0.0017615 | 0.00801696 | TRUE |
| LOC644189 | 9313 | 3.127399 | 0.0017636 | 0.00802399 | TRUE |
| SYT10 | 6231 | 3.12605 | 0.0017717 | 0.00805854 | TRUE |
| CACUL1 | 4877 | 3.124893 | 0.0017787 | 0.00808795 | TRUE |
| RPS11 | 5119 | 3.123693 | 0.001786 | 0.00811865 | TRUE |
| SYN2 | 6160 | 3.122633 | 0.0017924 | 0.0081432 | TRUE |
| FERMT2 | 7593 | 3.122403 | 0.0017938 | 0.00814703 | TRUE |
| SLC2A5 | 9117 | 3.12224 | 0.0017948 | 0.00814703 | TRUE |
| ZNF350 | 12144 | 3.122018 | 0.0017962 | 0.00814839 | TRUE |
| FAM45BP | 12009 | 3.119619 | 0.0018109 | 0.00819127 | TRUE |
| ARRB2 | 5359 | 3.118084 | 0.0018203 | 0.00822454 | TRUE |
| APEH | 3667 | 3.115316 | 0.0018375 | 0.00829253 | TRUE |
| PTRHD1 | 3394 | 3.114672 | 0.0018415 | 0.00830586 | TRUE |
| PPP4R4 | 10571 | 3.114117 | 0.001845 | 0.0083167 | TRUE |
| PDHB | 11385 | 3.113965 | 0.0018459 | 0.00831859 | TRUE |
| GPR107 | 5496 | 3.112676 | 0.001854 | 0.00834778 | TRUE |
| PGAM5 | 9620 | 3.111451 | 0.0018617 | 0.00837767 | TRUE |
| CCK | 12251 | 3.111263 | 0.0018629 | 0.00838059 | TRUE |
| DOCK8 | 10492 | 3.110089 | 0.0018703 | 0.00841155 | TRUE |
| BTNL9 | 15058 | 3.10997 | 0.0018711 | 0.00841252 | TRUE |
| RPS24 | 11343 | 3.106932 | 0.0018904 | 0.00848725 | TRUE |
| ATIC | 2204 | 3.105585 | 0.001899 | 0.00852111 | TRUE |
| TNS3 | 12908 | 3.105273 | 0.001901 | 0.00852766 | TRUE |
| EIF3K | 6976 | 3.10511 | 0.0019021 | 0.00852991 | TRUE |
| POR | 7604 | 3.104625 | 0.0019052 | 0.00854002 | TRUE |
| LRRTM1 | 9871 | 3.104505 | 0.001906 | 0.00854002 | TRUE |
| SLC16A8 | 6562 | 3.101856 | 0.0019231 | 0.00860941 | TRUE |
| SSR3 | 8757 | 3.101504 | 0.0019254 | 0.00861718 | TRUE |
| HES1 | 3091 | 3.101015 | 0.0019286 | 0.00862896 | TRUE |
| NWD1 | 9245 | 3.100201 | 0.0019339 | 0.00864928 | TRUE |
| LRRC61 | 9727 | 3.09961 | 0.0019378 | 0.00866008 | TRUE |
| CCDC8 | 13431 | 3.097846 | 0.0019493 | 0.00870204 | TRUE |
| DPP10-AS1 | 12993 | 3.095896 | 0.0019622 | 0.00875177 | TRUE |
| ABR | 4513 | 3.089849 | 0.0020026 | 0.00890651 | TRUE |
| LOC100506271 | 365 | 3.088945 | 0.0020087 | 0.00892951 | TRUE |
| SNTB1 | 5854 | 3.08797 | 0.0020153 | 0.00895283 | TRUE |
| MT1X | 11575 | 3.087715 | 0.002017 | 0.00895797 | TRUE |
| SAMD15 | 1671 | 3.085614 | 0.0020313 | 0.0090087 | TRUE |
| ANKRD19P | 4900 | 3.085349 | 0.0020331 | 0.00901262 | TRUE |
| SHC2 | 1440 | 3.08446 | 0.0020392 | 0.00903604 | TRUE |
| DNAJB5 | 2171 | 3.084226 | 0.0020408 | 0.00904058 | TRUE |
| LOC105374546 | 3218 | 3.082499 | 0.0020527 | 0.00908034 | TRUE |
| BPHL | 7014 | 3.08185 | 0.0020572 | 0.00909759 | TRUE |
| SCCPDH | 6196 | 3.080354 | 0.0020675 | 0.00913569 | TRUE |
| GPT2 | 3724 | 3.080106 | 0.0020693 | 0.0091381 | TRUE |
| XRCC6 | 13725 | 3.079687 | 0.0020722 | 0.00914669 | TRUE |
| EFEMP1 | 4556 | 3.079471 | 0.0020737 | 0.00914859 | TRUE |
| MAP1LC3A | 3779 | 3.079193 | 0.0020756 | 0.00915323 | TRUE |
| TMEM47 | 776 | 3.078687 | 0.0020792 | 0.009165 | TRUE |
| RPL21P44 | 2548 | 3.078374 | 0.0020813 | 0.00917066 | TRUE |
| TSTA3 | 5345 | 3.077746 | 0.0020857 | 0.00918225 | TRUE |
| SELENOM | 4470 | 3.077453 | 0.0020878 | 0.00918869 | TRUE |
| PTHLH | 14202 | 3.077255 | 0.0020892 | 0.00919221 | TRUE |
| TRAPPC6A | 7572 | 3.07659 | 0.0020938 | 0.00920756 | TRUE |
| BAG3 | 12456 | 3.076284 | 0.002096 | 0.00921442 | TRUE |
| ZNF525 | 5202 | 3.069038 | 0.0021475 | 0.0094144 | TRUE |
| CSNK2B | 5308 | 3.066687 | 0.0021645 | 0.00948078 | TRUE |
| TTN | 15614 | 3.065306 | 0.0021745 | 0.00951667 | TRUE |
| GALNT17 | 12019 | 3.064432 | 0.0021808 | 0.00953916 | TRUE |
| DNAJC2 | 9704 | 3.064349 | 0.0021814 | 0.00953916 | TRUE |
| MAPK8IP1P2 | 7606 | 3.06289 | 0.0021921 | 0.00958043 | TRUE |
| SND1 | 9014 | 3.060861 | 0.002207 | 0.00963751 | TRUE |
| PIGQ | 7683 | 3.059883 | 0.0022142 | 0.00966363 | TRUE |
| RPL27A | 15337 | 3.056064 | 0.0022426 | 0.00977044 | TRUE |
| ALG1L | 8833 | 3.056004 | 0.0022431 | 0.00977044 | TRUE |
| CIB2 | 6926 | 3.054969 | 0.0022508 | 0.00979876 | TRUE |
| CAPZB | 14806 | 3.054863 | 0.0022516 | 0.0097995 | TRUE |
| GLUD1 | 8897 | 3.054716 | 0.0022527 | 0.00980157 | TRUE |
| B4GAT1 | 3522 | 3.053106 | 0.0022649 | 0.00984349 | TRUE |
| OVOL2 | 13912 | 3.052672 | 0.0022681 | 0.00985212 | TRUE |
| ZNF229 | 5999 | 3.048628 | 0.0022989 | 0.00997462 | TRUE |
| SLC27A3 | 6786 | 3.048348 | 0.002301 | 0.00997838 | TRUE |
| TOX3 | 14779 | 3.047187 | 0.0023099 | 0.0100059 | TRUE |
| GULP1 | 987 | 3.046535 | 0.002315 | 0.01002207 | TRUE |
| GNPTAB | 13668 | 3.044029 | 0.0023343 | 0.01009754 | TRUE |
| ARHGAP28 | 11370 | 3.043688 | 0.002337 | 0.0101034 | TRUE |
| SMYD3 | 5246 | 3.043466 | 0.0023387 | 0.01010807 | TRUE |
| STOM | 6754 | 3.042056 | 0.0023497 | 0.01014992 | TRUE |
| WSCD1 | 13068 | 3.040739 | 0.00236 | 0.01019161 | TRUE |
| RFC3 | 14245 | 3.035508 | 0.0024013 | 0.01035291 | TRUE |
| TMEM208 | 11459 | 3.033791 | 0.002415 | 0.01040914 | TRUE |
| PDZRN4 | 12143 | 3.03334 | 0.0024186 | 0.01042184 | TRUE |
| DENND6B | 8546 | 3.033168 | 0.00242 | 0.01042491 | TRUE |
| SLC39A3 | 5634 | 3.030476 | 0.0024417 | 0.0105096 | TRUE |
| C1orf194 | 13960 | 3.030254 | 0.0024435 | 0.01051444 | TRUE |
| DIS3L | 81 | 3.028938 | 0.0024542 | 0.01054873 | TRUE |
| CA10 | 10716 | 3.027641 | 0.0024647 | 0.01058828 | TRUE |
| MRPL28 | 11383 | 3.02733 | 0.0024672 | 0.01059336 | TRUE |
| HLA-DRB5 | 2053 | 3.025728 | 0.0024804 | 0.01064672 | TRUE |
| TEX9 | 10845 | 3.025066 | 0.0024858 | 0.01066713 | TRUE |
| ROBO1 | 11147 | 3.024277 | 0.0024923 | 0.01068619 | TRUE |
| NRAP | 14331 | 3.024021 | 0.0024944 | 0.0106923 | TRUE |
| FAM86EP | 13779 | 3.023708 | 0.002497 | 0.01070043 | TRUE |
| RPAIN | 13714 | 3.023454 | 0.0024991 | 0.01070518 | TRUE |
| PDGFC | 7658 | 3.023159 | 0.0025015 | 0.01070812 | TRUE |
| MPP2 | 1046 | 3.022819 | 0.0025043 | 0.01071723 | TRUE |
| IQCK | 2442 | 3.021748 | 0.0025132 | 0.01074639 | TRUE |
| NAT6 | 9496 | 3.021346 | 0.0025165 | 0.01075479 | TRUE |
| ADGRG1 | 6282 | 3.017558 | 0.0025482 | 0.01087822 | TRUE |
| ARHGAP18 | 7968 | 3.016154 | 0.00256 | 0.01092276 | TRUE |
| GAL3ST4 | 8690 | 3.015291 | 0.0025673 | 0.01094496 | TRUE |
| NDN | 3337 | 3.01529 | 0.0025673 | 0.01094496 | TRUE |
| PIN4 | 6938 | 3.014981 | 0.00257 | 0.01095015 | TRUE |
| ENKUR | 7684 | 3.012955 | 0.0025872 | 0.01101451 | TRUE |
| GNAI2 | 9523 | 3.011477 | 0.0025998 | 0.01105924 | TRUE |
| SSBP4 | 1212 | 3.007922 | 0.0026304 | 0.01117726 | TRUE |
| TMEM68 | 10815 | 3.005864 | 0.0026483 | 0.01123863 | TRUE |
| NUMBL | 7518 | 3.005762 | 0.0026492 | 0.01123863 | TRUE |
| SLC25A14 | 4390 | 3.005531 | 0.0026512 | 0.01124412 | TRUE |
| ATP5IF1 | 9444 | 3.004994 | 0.0026559 | 0.01125788 | TRUE |
| AKR7A2P1 | 4624 | 3.004475 | 0.0026604 | 0.01127405 | TRUE |
| GPSM1 | 2446 | 3.004102 | 0.0026637 | 0.01128482 | TRUE |
| GOLGB1 | 15237 | 3.003016 | 0.0026732 | 0.0113221 | TRUE |
| NDUFB8 | 9928 | 3.000479 | 0.0026956 | 0.01140449 | TRUE |
| MXRA8 | 2712 | 2.999454 | 0.0027046 | 0.01143984 | TRUE |
| SLC19A1 | 13090 | 2.997599 | 0.0027212 | 0.01150035 | TRUE |
| TUBA1B | 14395 | 2.996111 | 0.0027345 | 0.0115493 | TRUE |
| FIBCD1 | 3578 | 2.996057 | 0.002735 | 0.0115493 | TRUE |
| ZDHHC12 | 3611 | 2.995525 | 0.0027397 | 0.01156635 | TRUE |
| PTRH1 | 10228 | 2.994878 | 0.0027455 | 0.01158778 | TRUE |
| APCDD1 | 6424 | 2.993961 | 0.0027538 | 0.01161952 | TRUE |
| RASGRP1 | 3373 | 2.993191 | 0.0027608 | 0.0116426 | TRUE |
| DHRSX | 2557 | 2.992357 | 0.0027683 | 0.01167131 | TRUE |
| HINT2 | 7769 | 2.989949 | 0.0027902 | 0.01175103 | TRUE |
| CALB1 | 11531 | 2.989754 | 0.002792 | 0.01175537 | TRUE |
| TTPAL | 2937 | 2.98885 | 0.0028003 | 0.01178386 | TRUE |
| KCNF1 | 12453 | 2.988241 | 0.0028059 | 0.01179288 | TRUE |
| ECH1 | 12534 | 2.988227 | 0.002806 | 0.01179288 | TRUE |
| SERTM1 | 4074 | 2.987586 | 0.0028119 | 0.01181361 | TRUE |
| CTSF | 9319 | 2.986464 | 0.0028222 | 0.0118475 | TRUE |
| SERPINB2 | 2363 | 2.985607 | 0.0028302 | 0.01187437 | TRUE |
| GABARAP | 13670 | 2.984033 | 0.0028448 | 0.01192603 | TRUE |
| BFSP1 | 4701 | 2.982969 | 0.0028547 | 0.01196115 | TRUE |
| MMAA | 11918 | 2.982575 | 0.0028583 | 0.01197014 | TRUE |
| ASIP | 503 | 2.982124 | 0.0028626 | 0.01198458 | TRUE |
| HCG23 | 14106 | 2.980832 | 0.0028747 | 0.01202559 | TRUE |
| TRUB1 | 7661 | 2.979013 | 0.0028918 | 0.01208916 | TRUE |
| HACD1 | 14643 | 2.977761 | 0.0029036 | 0.0121305 | TRUE |
| ADIPOR1 | 11022 | 2.977292 | 0.0029081 | 0.01214583 | TRUE |
| RNF175 | 2782 | 2.97679 | 0.0029128 | 0.01216249 | TRUE |
| E2F3 | 295 | 2.975916 | 0.0029211 | 0.01219069 | TRUE |
| CYP2R1 | 2575 | 2.975278 | 0.0029272 | 0.01221281 | TRUE |
| PPP1R3G | 11101 | 2.974859 | 0.0029312 | 0.01222624 | TRUE |
| ITGAX | 8056 | 2.974731 | 0.0029325 | 0.01222808 | TRUE |
| TMEM106A | 7651 | 2.974267 | 0.0029369 | 0.01224086 | TRUE |
| SMG8 | 5671 | 2.974247 | 0.0029371 | 0.01224086 | TRUE |
| CMAS | 12568 | 2.973254 | 0.0029466 | 0.01227399 | TRUE |
| RGS9 | 685 | 2.972594 | 0.0029529 | 0.0122971 | TRUE |
| NOP10 | 11749 | 2.972513 | 0.0029537 | 0.0122971 | TRUE |
| C2orf69 | 14298 | 2.971748 | 0.0029611 | 0.01231794 | TRUE |
| PPIAL4G | 11416 | 2.968645 | 0.0029912 | 0.01242647 | TRUE |
| FCGR2A | 2953 | 2.967822 | 0.0029992 | 0.01245648 | TRUE |
| UQCR11 | 4192 | 2.965995 | 0.0030171 | 0.01252739 | TRUE |
| GAST | 3853 | 2.964051 | 0.0030362 | 0.01260011 | TRUE |
| GSG1L | 2877 | 2.962975 | 0.0030468 | 0.01263417 | TRUE |
| GLO1 | 14131 | 2.962566 | 0.0030509 | 0.01264762 | TRUE |
| ARFGAP3 | 8589 | 2.961474 | 0.0030617 | 0.01268582 | TRUE |
| CREB3 | 1049 | 2.960819 | 0.0030682 | 0.01270857 | TRUE |
| CFAP70 | 14316 | 2.960759 | 0.0030688 | 0.01270857 | TRUE |
| TERF1 | 12001 | 2.959555 | 0.0030808 | 0.01275465 | TRUE |
| STOX1 | 10968 | 2.959481 | 0.0030816 | 0.01275465 | TRUE |
| MEIS2 | 13271 | 2.959109 | 0.0030853 | 0.01276329 | TRUE |
| RAB3IP | 2847 | 2.95776 | 0.0030988 | 0.0128125 | TRUE |
| UBXN8 | 10052 | 2.956553 | 0.003111 | 0.01285595 | TRUE |
| TMEM205 | 10888 | 2.955319 | 0.0031235 | 0.01289981 | TRUE |
| PBXIP1 | 10809 | 2.955258 | 0.0031241 | 0.01289981 | TRUE |
| BAD | 11527 | 2.954502 | 0.0031317 | 0.01292408 | TRUE |
| STRIP1 | 1982 | 2.954358 | 0.0031332 | 0.01292408 | TRUE |
| ICAM3 | 1901 | 2.953861 | 0.0031383 | 0.01293444 | TRUE |
| TOLLIP | 1427 | 2.952562 | 0.0031515 | 0.01298214 | TRUE |
| FLRT1 | 1021 | 2.950664 | 0.0031709 | 0.01305185 | TRUE |
| CAPNS1 | 102 | 2.949399 | 0.0031839 | 0.0130983 | TRUE |
| SERPINB8 | 10892 | 2.945629 | 0.003223 | 0.01322093 | TRUE |
| GPAA1 | 10401 | 2.945076 | 0.0032288 | 0.01323416 | TRUE |
| RILPL1 | 8662 | 2.944413 | 0.0032357 | 0.0132556 | TRUE |
| APRT | 14558 | 2.944177 | 0.0032381 | 0.01326224 | TRUE |
| SGSM2 | 867 | 2.943372 | 0.0032466 | 0.01328633 | TRUE |
| MPEG1 | 13289 | 2.942951 | 0.003251 | 0.01330093 | TRUE |
| ERI3 | 5645 | 2.939904 | 0.0032831 | 0.01341838 | TRUE |
| MT1M | 1063 | 2.937464 | 0.0033091 | 0.01350333 | TRUE |
| EBP | 4712 | 2.935552 | 0.0033295 | 0.01356904 | TRUE |
| CXCR4 | 1648 | 2.934382 | 0.0033421 | 0.01360965 | TRUE |
| FIS1 | 11621 | 2.934163 | 0.0033445 | 0.01361571 | TRUE |
| SNHG15 | 12480 | 2.932052 | 0.0033673 | 0.01369387 | TRUE |
| STUB1 | 6741 | 2.931982 | 0.0033681 | 0.01369387 | TRUE |
| TEK | 11049 | 2.92807 | 0.0034107 | 0.01383497 | TRUE |
| SLC9A6 | 7999 | 2.927058 | 0.0034219 | 0.01386927 | TRUE |
| GPR88 | 4754 | 2.925658 | 0.0034373 | 0.01392101 | TRUE |
| HTATSF1 | 220 | 2.92439 | 0.0034513 | 0.01396194 | TRUE |
| CACNA1F | 6902 | 2.924261 | 0.0034528 | 0.01396194 | TRUE |
| SH3RF1 | 11338 | 2.923814 | 0.0034577 | 0.01397477 | TRUE |
| UBE3A | 11310 | 2.92321 | 0.0034644 | 0.01399829 | TRUE |
| DCTD | 3609 | 2.92284 | 0.0034685 | 0.01401132 | TRUE |
| PACS1 | 12618 | 2.921836 | 0.0034797 | 0.01405293 | TRUE |
| CD2 | 5009 | 2.918752 | 0.0035144 | 0.01417074 | TRUE |
| TXN2 | 12901 | 2.915046 | 0.0035564 | 0.01432902 | TRUE |
| VPS25 | 3788 | 2.914413 | 0.0035636 | 0.01435329 | TRUE |
| PSMB1 | 2412 | 2.912791 | 0.0035821 | 0.0144069 | TRUE |
| ELAC2 | 963 | 2.912003 | 0.0035912 | 0.01443958 | TRUE |
| KCNMB1 | 11210 | 2.911259 | 0.0035998 | 0.01446657 | TRUE |
| LY86-AS1 | 11806 | 2.91091 | 0.0036038 | 0.01447902 | TRUE |
| GGCT | 5880 | 2.910273 | 0.0036111 | 0.01450111 | TRUE |
| TMEM164 | 3419 | 2.909222 | 0.0036233 | 0.01453502 | TRUE |
| SWSAP1 | 11495 | 2.906879 | 0.0036505 | 0.01462575 | TRUE |
| SLC40A1 | 2663 | 2.904995 | 0.0036726 | 0.01470259 | TRUE |
| HIST1H2AD | 3160 | 2.903477 | 0.0036904 | 0.01476271 | TRUE |
| GAP43 | 11601 | 2.903205 | 0.0036936 | 0.01477176 | TRUE |
| YWHAH | 5910 | 2.900914 | 0.0037208 | 0.01487257 | TRUE |
| PGAP1 | 10503 | 2.900383 | 0.0037271 | 0.01489399 | TRUE |
| MPDZ | 7502 | 2.899801 | 0.003734 | 0.01491786 | TRUE |
| NRXN2 | 6062 | 2.899535 | 0.0037372 | 0.01492289 | TRUE |
| LINC00260 | 821 | 2.898699 | 0.0037471 | 0.0149551 | TRUE |
| AQP4 | 12412 | 2.897759 | 0.0037584 | 0.01498851 | TRUE |
| FAM162A | 7007 | 2.897256 | 0.0037644 | 0.01500108 | TRUE |
| ARHGAP20 | 4285 | 2.895142 | 0.0037899 | 0.01507558 | TRUE |
| GS1-124K5.11 | 6615 | 2.894989 | 0.0037917 | 0.0150791 | TRUE |
| RALGPS2 | 10042 | 2.894244 | 0.0038007 | 0.01510724 | TRUE |
| STON2 | 2665 | 2.894073 | 0.0038028 | 0.01510942 | TRUE |
| RNF5P1 | 2895 | 2.894039 | 0.0038032 | 0.01510942 | TRUE |
| HPGDS | 11831 | 2.893063 | 0.003815 | 0.01514983 | TRUE |
| SPATC1L | 3729 | 2.891646 | 0.0038323 | 0.01520179 | TRUE |
| CXADR | 10165 | 2.890952 | 0.0038408 | 0.01523154 | TRUE |
| SMAD9 | 13610 | 2.890466 | 0.0038467 | 0.01524737 | TRUE |
| SCN9A | 6775 | 2.890167 | 0.0038504 | 0.01524959 | TRUE |
| EMP1 | 7062 | 2.890118 | 0.003851 | 0.01524959 | TRUE |
| TIMM17B | 12897 | 2.889898 | 0.0038537 | 0.01525175 | TRUE |
| LRCH2 | 11139 | 2.889629 | 0.003857 | 0.01526094 | TRUE |
| GNG10 | 2018 | 2.889486 | 0.0038587 | 0.01526346 | TRUE |
| HSPB1 | 5826 | 2.88937 | 0.0038601 | 0.01526346 | TRUE |
| PTTG1IP | 6522 | 2.889277 | 0.0038613 | 0.01526346 | TRUE |
| TVP23A | 5577 | 2.888869 | 0.0038663 | 0.01527854 | TRUE |
| PTRH2 | 11307 | 2.888617 | 0.0038694 | 0.01528306 | TRUE |
| USP51 | 6767 | 2.888326 | 0.003873 | 0.01529334 | TRUE |
| RAB23 | 2977 | 2.88803 | 0.0038766 | 0.01529615 | TRUE |
| RPL37A | 5633 | 2.88695 | 0.00389 | 0.01533714 | TRUE |
| RPS5 | 5666 | 2.886244 | 0.0038987 | 0.01536384 | TRUE |
| PPP1R14C | 3431 | 2.886132 | 0.0039001 | 0.01536543 | TRUE |
| DPM2 | 4534 | 2.882311 | 0.0039477 | 0.01553736 | TRUE |
| SUV39H1 | 8997 | 2.882096 | 0.0039504 | 0.01554014 | TRUE |
| LHFPL3 | 10474 | 2.881433 | 0.0039587 | 0.01556895 | TRUE |
| PPP1R18 | 11108 | 2.880825 | 0.0039664 | 0.01558834 | TRUE |
| RAC2 | 12406 | 2.880291 | 0.0039731 | 0.01560583 | TRUE |
| FAM174B | 2349 | 2.880099 | 0.0039755 | 0.01561141 | TRUE |
| CALB2 | 8893 | 2.879682 | 0.0039808 | 0.01562815 | TRUE |
| CCDC71L | 12379 | 2.879464 | 0.0039835 | 0.01563503 | TRUE |
| DENND2A | 5927 | 2.877859 | 0.0040038 | 0.01569903 | TRUE |
| CPE | 10869 | 2.876575 | 0.0040202 | 0.01575514 | TRUE |
| ROM1 | 5939 | 2.875584 | 0.0040328 | 0.01580073 | TRUE |
| ALDOA | 3709 | 2.875095 | 0.0040391 | 0.01582125 | TRUE |
| ADAMTS19 | 10733 | 2.874838 | 0.0040423 | 0.01583017 | TRUE |
| PDGFRL | 7370 | 2.8715 | 0.0040853 | 0.01599031 | TRUE |
| B3GNTL1 | 11351 | 2.870417 | 0.0040993 | 0.01603716 | TRUE |
| GS1-124K5.4 | 9977 | 2.86769 | 0.0041348 | 0.01612571 | TRUE |
| DLG2 | 5929 | 2.867648 | 0.0041354 | 0.01612571 | TRUE |
| ZNF517 | 13124 | 2.866099 | 0.0041556 | 0.01618061 | TRUE |
| NPY5R | 14013 | 2.865723 | 0.0041606 | 0.01619581 | TRUE |
| ZNF277 | 2668 | 2.865446 | 0.0041642 | 0.0162035 | TRUE |
| PRMT2 | 153 | 2.864654 | 0.0041747 | 0.01623845 | TRUE |
| DAP | 7842 | 2.860069 | 0.0042355 | 0.01644236 | TRUE |
| LILRP2 | 11750 | 2.859236 | 0.0042466 | 0.01647122 | TRUE |
| RDH10 | 1297 | 2.858972 | 0.0042502 | 0.01647887 | TRUE |
| HEYL | 12921 | 2.858045 | 0.0042626 | 0.0165148 | TRUE |
| NAA38 | 2086 | 2.857792 | 0.004266 | 0.01651978 | TRUE |
| PARD3B | 4065 | 2.8577 | 0.0042672 | 0.01652048 | TRUE |
| ACTG1P4 | 14437 | 2.855562 | 0.0042961 | 0.01661566 | TRUE |
| WISP2 | 12452 | 2.852534 | 0.0043372 | 0.01675821 | TRUE |
| TMEM126A | 12448 | 2.851484 | 0.0043516 | 0.01679291 | TRUE |
| MRPL30 | 12231 | 2.851351 | 0.0043534 | 0.01679492 | TRUE |
| CLPB | 2101 | 2.85063 | 0.0043633 | 0.01682273 | TRUE |
| C16orf58 | 13795 | 2.850606 | 0.0043636 | 0.01682273 | TRUE |
| FKRP | 6237 | 2.849809 | 0.0043745 | 0.01686 | TRUE |
| PLA2G15 | 12970 | 2.849135 | 0.0043838 | 0.01688405 | TRUE |
| PPID | 5072 | 2.849017 | 0.0043855 | 0.01688616 | TRUE |
| FIP1L1 | 7845 | 2.847904 | 0.0044008 | 0.01694115 | TRUE |
| FITM1 | 10177 | 2.846994 | 0.0044134 | 0.01698547 | TRUE |
| RPL19 | 9966 | 2.846139 | 0.0044253 | 0.01702277 | TRUE |
| ATXN10 | 7168 | 2.845647 | 0.0044321 | 0.0170407 | TRUE |
| MT1E | 6074 | 2.843715 | 0.0044591 | 0.01712331 | TRUE |
| FAM47E | 9793 | 2.843239 | 0.0044658 | 0.01714398 | TRUE |
| GDF10 | 1199 | 2.842429 | 0.0044771 | 0.01717717 | TRUE |
| ATP6V0C | 11070 | 2.84126 | 0.0044936 | 0.01723452 | TRUE |
| CCNQ | 14393 | 2.839912 | 0.0045126 | 0.01729283 | TRUE |
| SLC30A10 | 3246 | 2.83859 | 0.0045313 | 0.01734533 | TRUE |
| CLBA1 | 10104 | 2.838261 | 0.004536 | 0.01735897 | TRUE |
| IQCA1 | 8424 | 2.835646 | 0.0045733 | 0.01747606 | TRUE |
| CCS | 5340 | 2.834714 | 0.0045867 | 0.01751987 | TRUE |
| RPL18 | 12336 | 2.83458 | 0.0045886 | 0.01752163 | TRUE |
| GPR137B | 8173 | 2.834322 | 0.0045923 | 0.0175315 | TRUE |
| ANKRD30B | 7672 | 2.834133 | 0.004595 | 0.01753331 | TRUE |
| CD84 | 13641 | 2.833197 | 0.0046085 | 0.01757341 | TRUE |
| TEAD1 | 3295 | 2.83315 | 0.0046092 | 0.01757341 | TRUE |
| B4GALT3 | 1665 | 2.832991 | 0.0046115 | 0.01757341 | TRUE |
| C22orf23 | 10489 | 2.83215 | 0.0046236 | 0.01759946 | TRUE |
| MPV17 | 14752 | 2.83176 | 0.0046293 | 0.01761665 | TRUE |
| SF3A2 | 7318 | 2.831508 | 0.0046329 | 0.01762625 | TRUE |
| SIX3 | 5843 | 2.830921 | 0.0046414 | 0.01765004 | TRUE |
| CNIH3 | 4761 | 2.826075 | 0.0047122 | 0.01788448 | TRUE |
| DACT3 | 1702 | 2.825024 | 0.0047277 | 0.01793109 | TRUE |
| MAP2K1 | 4027 | 2.823314 | 0.004753 | 0.01800864 | TRUE |
| CORO1C | 2021 | 2.822745 | 0.0047614 | 0.01803189 | TRUE |
| IGSF21 | 7033 | 2.821286 | 0.0047832 | 0.0180922 | TRUE |
| DGCR6L | 13214 | 2.820747 | 0.0047912 | 0.01811385 | TRUE |
| PLCH1 | 13536 | 2.819727 | 0.0048065 | 0.01815397 | TRUE |
| RANBP3L | 4492 | 2.819384 | 0.0048116 | 0.01816786 | TRUE |
| RUBCNL | 12925 | 2.819064 | 0.0048164 | 0.01817394 | TRUE |
| NPFFR2 | 4166 | 2.818811 | 0.0048202 | 0.01818388 | TRUE |
| TSPOAP1 | 3144 | 2.818647 | 0.0048227 | 0.0181844 | TRUE |
| BRF2 | 12747 | 2.81773 | 0.0048364 | 0.01822807 | TRUE |
| FERMT1 | 14369 | 2.816502 | 0.004855 | 0.01829304 | TRUE |
| LYRM2 | 3546 | 2.816401 | 0.0048565 | 0.01829438 | TRUE |
| LY96 | 5643 | 2.814617 | 0.0048835 | 0.01838064 | TRUE |
| SYTL5 | 7442 | 2.814096 | 0.0048915 | 0.01839948 | TRUE |
| TUBB2B | 15160 | 2.813681 | 0.0048978 | 0.01841881 | TRUE |
| SPATA20 | 3562 | 2.813236 | 0.0049046 | 0.01843989 | TRUE |
| ADPRHL2 | 10607 | 2.812782 | 0.0049115 | 0.01845527 | TRUE |
| PELI1 | 4324 | 2.812203 | 0.0049203 | 0.01848144 | TRUE |
| GRIK5 | 3315 | 2.811849 | 0.0049258 | 0.0184929 | TRUE |
| GNA12 | 4895 | 2.808682 | 0.0049745 | 0.01864892 | TRUE |
| BOLA1 | 13440 | 2.807979 | 0.0049853 | 0.01868072 | TRUE |
| RCHY1 | 11120 | 2.806707 | 0.0050051 | 0.01874565 | TRUE |
| CCDC153 | 14057 | 2.805599 | 0.0050223 | 0.01880121 | TRUE |
| RCN2 | 8484 | 2.80478 | 0.0050351 | 0.01884453 | TRUE |
| PNMA8A | 14210 | 2.804526 | 0.0050391 | 0.01885487 | TRUE |
| CHST15 | 11376 | 2.804442 | 0.0050404 | 0.01885527 | TRUE |
| GPR25 | 11218 | 2.804309 | 0.0050425 | 0.01885854 | TRUE |
| CLIC5 | 8717 | 2.803035 | 0.0050624 | 0.01892867 | TRUE |
| RAPGEF4 | 10614 | 2.80265 | 0.0050685 | 0.01894675 | TRUE |
| HINT1 | 14119 | 2.80048 | 0.0051027 | 0.01906549 | TRUE |
| EMP3 | 11645 | 2.798584 | 0.0051327 | 0.01916404 | TRUE |
| CXCL3 | 13551 | 2.796534 | 0.0051654 | 0.01927684 | TRUE |
| MPPED1 | 9742 | 2.795963 | 0.0051745 | 0.01930171 | TRUE |
| CPVL | 5277 | 2.795652 | 0.0051795 | 0.01931568 | TRUE |
| CD52 | 14367 | 2.792437 | 0.0052313 | 0.01948544 | TRUE |
| YBX2 | 7356 | 2.789875 | 0.0052728 | 0.01963094 | TRUE |
| TMEM101 | 14800 | 2.788738 | 0.0052914 | 0.01968592 | TRUE |
| OR14I1 | 10861 | 2.788364 | 0.0052975 | 0.01969284 | TRUE |
| MYO1F | 15002 | 2.788183 | 0.0053005 | 0.01969284 | TRUE |
| CRLF1 | 7881 | 2.78683 | 0.0053226 | 0.01975049 | TRUE |
| NDP | 3556 | 2.786183 | 0.0053333 | 0.01978203 | TRUE |
| RPL8 | 2767 | 2.783465 | 0.0053782 | 0.01994233 | TRUE |
| MAOA | 3462 | 2.783009 | 0.0053857 | 0.01996091 | TRUE |
| NDUFS5 | 13497 | 2.782928 | 0.0053871 | 0.01996117 | TRUE |
| CLEC4G | 7680 | 2.781145 | 0.0054168 | 0.02005687 | TRUE |
| ORMDL2 | 3124 | 2.780595 | 0.0054259 | 0.02008137 | TRUE |
| SKP1 | 10174 | 2.779438 | 0.0054453 | 0.02013398 | TRUE |
| PHF20L1 | 8644 | 2.77855 | 0.0054602 | 0.02018432 | TRUE |
| NRCAM | 9587 | 2.776895 | 0.0054881 | 0.02028258 | TRUE |
| FLOT2 | 4200 | 2.77615 | 0.0055007 | 0.0203163 | TRUE |
| GRIK4 | 9628 | 2.776077 | 0.0055019 | 0.0203163 | TRUE |
| CX3CL1 | 4227 | 2.773503 | 0.0055456 | 0.02045043 | TRUE |
| TYRO3 | 12442 | 2.773447 | 0.0055466 | 0.02045043 | TRUE |
| ACTA1 | 3247 | 2.77264 | 0.0055604 | 0.02048671 | TRUE |
| SREK1IP1 | 849 | 2.772476 | 0.0055632 | 0.0204922 | TRUE |
| ITGB7 | 1123 | 2.770336 | 0.0055998 | 0.02061277 | TRUE |
| SSR4 | 7541 | 2.7694 | 0.005616 | 0.0206575 | TRUE |
| PITPNB | 5189 | 2.769237 | 0.0056188 | 0.02066298 | TRUE |
| HCFC1R1 | 9156 | 2.768937 | 0.005624 | 0.02067715 | TRUE |
| COMMD9 | 11322 | 2.768702 | 0.005628 | 0.0206872 | TRUE |
| LARS2 | 5100 | 2.764898 | 0.0056941 | 0.0208937 | TRUE |
| DDB1 | 6258 | 2.764851 | 0.0056949 | 0.0208937 | TRUE |
| MRPL20 | 14387 | 2.762642 | 0.0057336 | 0.02102575 | TRUE |
| ZCRB1 | 11520 | 2.761889 | 0.0057468 | 0.02106442 | TRUE |
| MAPKAPK5-AS1 | 14016 | 2.758968 | 0.0057984 | 0.02124865 | TRUE |
| LINC02381 | 6825 | 2.75875 | 0.0058023 | 0.02125784 | TRUE |
| ZNF706 | 8159 | 2.755861 | 0.0058538 | 0.02143144 | TRUE |
| CHAF1B | 8696 | 2.754664 | 0.0058752 | 0.02150496 | TRUE |
| TUBA1A | 12437 | 2.752882 | 0.0059073 | 0.02161224 | TRUE |
| NDUFA2 | 8615 | 2.749577 | 0.0059672 | 0.02177535 | TRUE |
| ISYNA1 | 9259 | 2.748815 | 0.0059811 | 0.02182094 | TRUE |
| PAK3 | 1956 | 2.747267 | 0.0060094 | 0.0219191 | TRUE |
| GSPT2 | 663 | 2.745291 | 0.0060457 | 0.02204124 | TRUE |
| AP5S1 | 3942 | 2.744342 | 0.0060632 | 0.02209163 | TRUE |
| FAH | 4571 | 2.744312 | 0.0060638 | 0.02209163 | TRUE |
| FAM181B | 9015 | 2.740222 | 0.0061398 | 0.02233202 | TRUE |
| MRPL14 | 7215 | 2.739922 | 0.0061454 | 0.02234721 | TRUE |
| LIMS2 | 229 | 2.738832 | 0.0061658 | 0.0224058 | TRUE |
| EFEMP2 | 11940 | 2.73776 | 0.0061859 | 0.02247374 | TRUE |
| EIF3F | 4672 | 2.736503 | 0.0062096 | 0.02254516 | TRUE |
| RNF11 | 4535 | 2.736404 | 0.0062115 | 0.02254561 | TRUE |
| TMEM258 | 4508 | 2.734779 | 0.0062422 | 0.02263653 | TRUE |
| CSE1L | 7530 | 2.734774 | 0.0062423 | 0.02263653 | TRUE |
| ODC1 | 9132 | 2.734474 | 0.006248 | 0.02264728 | TRUE |
| APEX1 | 254 | 2.732748 | 0.0062808 | 0.02274457 | TRUE |
| FAM105A | 245 | 2.731648 | 0.0063018 | 0.02281264 | TRUE |
| DHCR7 | 11996 | 2.731326 | 0.006308 | 0.02282709 | TRUE |
| FRA10AC1 | 7018 | 2.731006 | 0.0063141 | 0.02284398 | TRUE |
| PTGES | 5212 | 2.730818 | 0.0063177 | 0.02285172 | TRUE |
| RPS20 | 12708 | 2.730658 | 0.0063208 | 0.02285753 | TRUE |
| VASN | 9589 | 2.730412 | 0.0063255 | 0.02286931 | TRUE |
| CLEC9A | 8158 | 2.72967 | 0.0063398 | 0.02291554 | TRUE |
| SMIM10L2A | 5269 | 2.729197 | 0.0063489 | 0.02293783 | TRUE |
| DAD1 | 13159 | 2.727771 | 0.0063764 | 0.02303189 | TRUE |
| DNAJC12 | 11118 | 2.727158 | 0.0063882 | 0.02305874 | TRUE |
| ZFP64 | 11190 | 2.725871 | 0.0064132 | 0.02313282 | TRUE |
| CXXC4 | 3753 | 2.723691 | 0.0064557 | 0.02326458 | TRUE |
| YWHAB | 11875 | 2.723518 | 0.0064591 | 0.02327141 | TRUE |
| HOPX | 3929 | 2.723106 | 0.0064671 | 0.02329508 | TRUE |
| C9orf16 | 2012 | 2.721071 | 0.0065071 | 0.023423 | TRUE |
| BCAN | 7291 | 2.719633 | 0.0065354 | 0.02351405 | TRUE |
| COX6A2 | 6797 | 2.719121 | 0.0065456 | 0.02354506 | TRUE |
| ST6GAL1 | 3415 | 2.714285 | 0.0066419 | 0.02385864 | TRUE |
| MAGIX | 15162 | 2.71313 | 0.0066651 | 0.02393097 | TRUE |
| ALDH2 | 4275 | 2.711649 | 0.0066949 | 0.0240271 | TRUE |
| SLC15A2 | 4632 | 2.711368 | 0.0067006 | 0.02403347 | TRUE |
| STK17B | 4184 | 2.710169 | 0.0067249 | 0.02411244 | TRUE |
| OCA2 | 14512 | 2.709796 | 0.0067325 | 0.02413404 | TRUE |
| TIMM13 | 3243 | 2.70654 | 0.0067988 | 0.02434408 | TRUE |
| PPP1R1C | 8443 | 2.705323 | 0.0068238 | 0.02442227 | TRUE |
| RAB3B | 11985 | 2.704722 | 0.0068362 | 0.02443794 | TRUE |
| GBAP1 | 12064 | 2.704555 | 0.0068396 | 0.02443794 | TRUE |
| MB21D2 | 10485 | 2.703653 | 0.0068582 | 0.02448545 | TRUE |
| SLIRP | 7657 | 2.701495 | 0.0069029 | 0.02463188 | TRUE |
| HIST1H2BG | 6950 | 2.698773 | 0.0069596 | 0.02480601 | TRUE |
| PPP1CA | 14565 | 2.697969 | 0.0069764 | 0.02486026 | TRUE |
| PSMA5 | 4061 | 2.6971 | 0.0069946 | 0.02491337 | TRUE |
| TMEM63C | 7837 | 2.696992 | 0.0069969 | 0.02491337 | TRUE |
| SOWAHD | 12867 | 2.696955 | 0.0069977 | 0.02491337 | TRUE |
| PIH1D1 | 12870 | 2.696846 | 0.007 | 0.02491585 | TRUE |
| TNFRSF14 | 3233 | 2.696391 | 0.0070095 | 0.02493856 | TRUE |
| TBPL1 | 8375 | 2.694475 | 0.00705 | 0.0250425 | TRUE |
| SCN3A | 14947 | 2.69413 | 0.0070573 | 0.02505705 | TRUE |
| TECPR2 | 2705 | 2.69404 | 0.0070592 | 0.02505813 | TRUE |
| GPC5 | 9736 | 2.692581 | 0.0070901 | 0.0251509 | TRUE |
| HIST1H2BL | 8606 | 2.692381 | 0.0070944 | 0.02516028 | TRUE |
| ENC1 | 10286 | 2.690733 | 0.0071295 | 0.0252677 | TRUE |
| TRIL | 7900 | 2.69052 | 0.0071341 | 0.02527238 | TRUE |
| ALCAM | 1669 | 2.68967 | 0.0071523 | 0.02532736 | TRUE |
| HSD17B6 | 5551 | 2.689636 | 0.007153 | 0.02532736 | TRUE |
| CHRM4 | 7159 | 2.689568 | 0.0071545 | 0.02532736 | TRUE |
| PIP4P1 | 13046 | 2.687 | 0.0072097 | 0.02551713 | TRUE |
| SHC1 | 4151 | 2.68421 | 0.0072701 | 0.02570198 | TRUE |
| NFATC1 | 13617 | 2.683832 | 0.0072784 | 0.02572523 | TRUE |
| POLR2K | 9914 | 2.682229 | 0.0073133 | 0.02581382 | TRUE |
| RUNDC3A | 2578 | 2.682109 | 0.007316 | 0.02581725 | TRUE |
| TMC8 | 5339 | 2.677822 | 0.0074103 | 0.02611465 | TRUE |
| LINC01736 | 12570 | 2.677341 | 0.0074209 | 0.02614628 | TRUE |
| ZSWIM7 | 1488 | 2.676183 | 0.0074466 | 0.02621907 | TRUE |
| STIM1 | 3844 | 2.675703 | 0.0074573 | 0.02623403 | TRUE |
| LOC105376064 | 14586 | 2.675496 | 0.0074619 | 0.02624331 | TRUE |
| SIN3B | 4726 | 2.675019 | 0.0074725 | 0.02627477 | TRUE |
| SLC16A5 | 12160 | 2.673425 | 0.0075081 | 0.02638216 | TRUE |
| NDUFS6 | 8225 | 2.673047 | 0.0075166 | 0.02640597 | TRUE |
| DLEU7 | 8367 | 2.672965 | 0.0075184 | 0.02640649 | TRUE |
| MND1 | 3948 | 2.670297 | 0.0075784 | 0.02659933 | TRUE |
| GRB10 | 12299 | 2.670124 | 0.0075823 | 0.02660706 | TRUE |
| SLC39A10 | 2865 | 2.668768 | 0.007613 | 0.02669729 | TRUE |
| LINGO3 | 4817 | 2.667517 | 0.0076414 | 0.02678431 | TRUE |
| HPGD | 5352 | 2.665647 | 0.007684 | 0.02691564 | TRUE |
| RPLP1 | 715 | 2.663172 | 0.0077408 | 0.02708204 | TRUE |
| ROMO1 | 13718 | 2.662893 | 0.0077472 | 0.02709442 | TRUE |
| OAF | 13716 | 2.662436 | 0.0077577 | 0.0271191 | TRUE |
| ACOX3 | 5606 | 2.661964 | 0.0077686 | 0.02715109 | TRUE |
| FCGR3A | 13697 | 2.659066 | 0.0078358 | 0.02734296 | TRUE |
| AMZ2P1 | 194 | 2.658133 | 0.0078575 | 0.02740654 | TRUE |
| WASHC3 | 11862 | 2.656977 | 0.0078845 | 0.02747005 | TRUE |
| CYTL1 | 12484 | 2.656097 | 0.0079051 | 0.02752499 | TRUE |
| ERMARD | 4583 | 2.656078 | 0.0079055 | 0.02752499 | TRUE |
| KIAA0368 | 2381 | 2.655696 | 0.0079145 | 0.02755005 | TRUE |
| CCNA1 | 8077 | 2.654027 | 0.0079537 | 0.02767436 | TRUE |
| EXOSC1 | 13522 | 2.65385 | 0.0079579 | 0.02768272 | TRUE |
| PSMB10 | 4389 | 2.652933 | 0.0079796 | 0.02775186 | TRUE |
| MTMR3 | 12383 | 2.651192 | 0.0080208 | 0.02786913 | TRUE |
| LINC01088 | 4613 | 2.651148 | 0.0080219 | 0.02786913 | TRUE |
| TM2D2 | 9895 | 2.651134 | 0.0080222 | 0.02786913 | TRUE |
| CASTOR1 | 10556 | 2.650527 | 0.0080366 | 0.02790685 | TRUE |
| FBXO44 | 6271 | 2.648657 | 0.0080812 | 0.02802363 | TRUE |
| IDNK | 14972 | 2.648458 | 0.008086 | 0.02802778 | TRUE |
| BRMS1 | 7187 | 2.647077 | 0.0081191 | 0.02811823 | TRUE |
| SPATA24 | 3964 | 2.646572 | 0.0081312 | 0.02815401 | TRUE |
| SMPDL3A | 6342 | 2.646448 | 0.0081342 | 0.0281581 | TRUE |
| C3 | 7373 | 2.646345 | 0.0081367 | 0.02816044 | TRUE |
| LOC102723665 | 9054 | 2.645451 | 0.0081582 | 0.02821622 | TRUE |
| CHD3 | 6523 | 2.645266 | 0.0081627 | 0.02822541 | TRUE |
| FAM32A | 2494 | 2.645097 | 0.0081668 | 0.02823326 | TRUE |
| LIX1L | 8174 | 2.6449 | 0.0081715 | 0.02824346 | TRUE |
| RPL18A | 9337 | 2.644575 | 0.0081794 | 0.0282581 | TRUE |
| CD38 | 9640 | 2.644172 | 0.0081891 | 0.02827419 | TRUE |
| DNPEP | 3543 | 2.64133 | 0.0082581 | 0.02843596 | TRUE |
| HAVCR2 | 4623 | 2.640625 | 0.0082753 | 0.02848264 | TRUE |
| PHACTR3 | 10517 | 2.639575 | 0.008301 | 0.02856474 | TRUE |
| DCTN2 | 13637 | 2.6384 | 0.0083298 | 0.02863955 | TRUE |
| ZIC5 | 13062 | 2.63807 | 0.0083379 | 0.02866028 | TRUE |
| GRM3 | 2935 | 2.636515 | 0.0083763 | 0.02876595 | TRUE |
| LOC101927974 | 1975 | 2.636229 | 0.0083833 | 0.02877494 | TRUE |
| TCTE1 | 3242 | 2.636194 | 0.0083842 | 0.02877494 | TRUE |
| NUCB2 | 12746 | 2.635169 | 0.0084095 | 0.02883493 | TRUE |
| UBXN6 | 13886 | 2.633076 | 0.0084615 | 0.02897374 | TRUE |
| RAD17 | 13041 | 2.632426 | 0.0084777 | 0.02901963 | TRUE |
| AKR1C3 | 13299 | 2.632019 | 0.0084879 | 0.02904806 | TRUE |
| IVD | 4087 | 2.631828 | 0.0084927 | 0.02905803 | TRUE |
| AGA | 10206 | 2.630709 | 0.0085207 | 0.02913474 | TRUE |
| ACAD9 | 4983 | 2.630198 | 0.0085335 | 0.02917219 | TRUE |
| MASP1 | 6422 | 2.629684 | 0.0085464 | 0.02920356 | TRUE |
| PSMC1 | 956 | 2.627178 | 0.0086096 | 0.02937458 | TRUE |
| PRDX2 | 4827 | 2.625954 | 0.0086406 | 0.02944828 | TRUE |
| AMDHD1 | 273 | 2.625736 | 0.0086462 | 0.02946073 | TRUE |
| PSMB5 | 7517 | 2.624558 | 0.0086762 | 0.02954995 | TRUE |
| MT1JP | 14617 | 2.624334 | 0.0086819 | 0.02956295 | TRUE |
| FPR1 | 14069 | 2.62368 | 0.0086985 | 0.02961332 | TRUE |
| GRAMD1C | 9809 | 2.622888 | 0.0087188 | 0.02965642 | TRUE |
| CDH3 | 8131 | 2.622023 | 0.008741 | 0.0297124 | TRUE |
| GAS5 | 1557 | 2.621919 | 0.0087436 | 0.02971501 | TRUE |
| SH3BP5 | 7664 | 2.619255 | 0.0088122 | 0.02991557 | TRUE |
| CCDC158 | 4580 | 2.618369 | 0.0088351 | 0.02998193 | TRUE |
| OAZ2 | 6110 | 2.618206 | 0.0088393 | 0.02998816 | TRUE |
| USP35 | 2671 | 2.61757 | 0.0088558 | 0.03003758 | TRUE |
| STMN2 | 9103 | 2.615025 | 0.0089221 | 0.03024264 | TRUE |
| MON1A | 1536 | 2.614707 | 0.0089304 | 0.03026425 | TRUE |
| TRAPPC9 | 12695 | 2.614559 | 0.0089343 | 0.0302708 | TRUE |
| FUCA2 | 2438 | 2.614339 | 0.00894 | 0.03027293 | TRUE |
| DRC1 | 10322 | 2.614313 | 0.0089407 | 0.03027293 | TRUE |
| TIMM50 | 11427 | 2.613422 | 0.0089641 | 0.03033555 | TRUE |
| ACY3 | 8767 | 2.613385 | 0.008965 | 0.03033555 | TRUE |
| CETN3 | 9772 | 2.613091 | 0.0089727 | 0.03035509 | TRUE |
| CH25H | 5227 | 2.612647 | 0.0089844 | 0.03038797 | TRUE |
| RIPK2 | 10663 | 2.612453 | 0.0089895 | 0.03039865 | TRUE |
| LOC400710 | 5327 | 2.611744 | 0.0090082 | 0.030442 | TRUE |
| TCAP | 7548 | 2.611556 | 0.0090131 | 0.03045216 | TRUE |
| 3-Sep | 8385 | 2.611479 | 0.0090152 | 0.03045244 | TRUE |
| NCAN | 12038 | 2.610696 | 0.0090358 | 0.03051565 | TRUE |
| OLFML3 | 9550 | 2.609279 | 0.0090733 | 0.03061586 | TRUE |
| CIP2A | 9926 | 2.608467 | 0.0090949 | 0.03068197 | TRUE |
| SIRT3 | 1903 | 2.608293 | 0.0090995 | 0.03069095 | TRUE |
| C11orf65 | 8210 | 2.608201 | 0.0091019 | 0.03069257 | TRUE |
| ATP6V1H | 5563 | 2.60811 | 0.0091044 | 0.03069386 | TRUE |
| METTL26 | 2275 | 2.607848 | 0.0091113 | 0.03070437 | TRUE |
| AK4 | 13065 | 2.60765 | 0.0091166 | 0.03071552 | TRUE |
| RHOJ | 5642 | 2.607556 | 0.0091191 | 0.03071733 | TRUE |
| FAM167A | 6476 | 2.607395 | 0.0091234 | 0.03072516 | TRUE |
| SHISA5 | 5576 | 2.606724 | 0.0091413 | 0.0307788 | TRUE |
| ALDH6A1 | 12674 | 2.606196 | 0.0091554 | 0.03081965 | TRUE |
| UROD | 9916 | 2.605655 | 0.0091699 | 0.03086172 | TRUE |
| NPTX2 | 2679 | 2.604413 | 0.0092032 | 0.03096047 | TRUE |
| CDK5 | 2118 | 2.602964 | 0.0092422 | 0.03108494 | TRUE |
| NAA10 | 13181 | 2.602833 | 0.0092457 | 0.03109013 | TRUE |
| MIIP | 5837 | 2.601636 | 0.009278 | 0.03117872 | TRUE |
| HHEX | 1748 | 2.601103 | 0.0092925 | 0.03120038 | TRUE |
| HLA-A | 3588 | 2.599078 | 0.0093475 | 0.03133792 | TRUE |
| NDUFB7 | 11268 | 2.596831 | 0.0094088 | 0.03151663 | TRUE |
| UFD1 | 12599 | 2.59549 | 0.0094456 | 0.03162632 | TRUE |
| DRAIC | 13035 | 2.594566 | 0.009471 | 0.03169552 | TRUE |
| MRPL10 | 8123 | 2.594518 | 0.0094724 | 0.03169552 | TRUE |
| PSMA7 | 13744 | 2.59369 | 0.0094952 | 0.03175156 | TRUE |
| LAIR1 | 8258 | 2.592399 | 0.0095309 | 0.03185735 | TRUE |
| CLDN10 | 6937 | 2.592227 | 0.0095357 | 0.03186234 | TRUE |
| PRDM16 | 5855 | 2.592198 | 0.0095365 | 0.03186234 | TRUE |
| MRPS11 | 298 | 2.590544 | 0.0095824 | 0.03200219 | TRUE |
| SERHL2 | 5235 | 2.589324 | 0.0096165 | 0.03208838 | TRUE |
| OPTN | 4999 | 2.588427 | 0.0096415 | 0.03215835 | TRUE |
| CMTM4 | 8961 | 2.587409 | 0.0096701 | 0.03223298 | TRUE |
| HIST1H2BC | 10610 | 2.587408 | 0.0096701 | 0.03223298 | TRUE |
| NFIA | 5017 | 2.587202 | 0.0096759 | 0.03224539 | TRUE |
| FAHD2A | 8707 | 2.586178 | 0.0097047 | 0.03232055 | TRUE |
| KCNN2 | 926 | 2.586106 | 0.0097067 | 0.03232055 | TRUE |
| WFIKKN2 | 4864 | 2.584037 | 0.0097651 | 0.03250816 | TRUE |
| TRPS1 | 2701 | 2.581472 | 0.009838 | 0.03273678 | TRUE |
| PSMB4 | 11402 | 2.581349 | 0.0098415 | 0.03274148 | TRUE |
| NDUFA3 | 14870 | 2.579081 | 0.0099064 | 0.03293621 | TRUE |
| PRKG1 | 4507 | 2.578406 | 0.0099257 | 0.03298659 | TRUE |
| CCEPR | 10103 | 2.57689 | 0.0099694 | 0.0331105 | TRUE |
| ERICH3 | 5256 | 2.574792 | 0.01003 | 0.03329078 | TRUE |
| MVK | 7829 | 2.574699 | 0.0100327 | 0.03329267 | TRUE |
| PDGFB | 5829 | 2.572904 | 0.0100849 | 0.03344082 | TRUE |
| PON2 | 14448 | 2.57287 | 0.0100859 | 0.03344082 | TRUE |
| OSCP1 | 530 | 2.572869 | 0.0100859 | 0.03344082 | TRUE |
| ICAM5 | 13671 | 2.572554 | 0.0100951 | 0.03345708 | TRUE |
| FAM131A | 1292 | 2.571661 | 0.0101212 | 0.03353638 | TRUE |
| MPHOSPH6 | 8090 | 2.571133 | 0.0101366 | 0.03358044 | TRUE |
| NDUFB10 | 6358 | 2.569634 | 0.0101806 | 0.03370465 | TRUE |
| ADGRA3 | 14651 | 2.568574 | 0.0102118 | 0.03377682 | TRUE |
| GSTM5 | 10962 | 2.568526 | 0.0102132 | 0.03377682 | TRUE |
| GPR183 | 12319 | 2.567981 | 0.0102293 | 0.03382282 | TRUE |
| ALYREF | 14576 | 2.567018 | 0.0102577 | 0.03390461 | TRUE |
| EXOSC7 | 13518 | 2.566997 | 0.0102584 | 0.03390461 | TRUE |
| AGAP3 | 13170 | 2.566149 | 0.0102835 | 0.03397325 | TRUE |
| MZT1 | 1893 | 2.565343 | 0.0103074 | 0.0340307 | TRUE |
| LCTL | 7644 | 2.564707 | 0.0103263 | 0.03407155 | TRUE |
| GDAP1L1 | 1791 | 2.564223 | 0.0103407 | 0.0341047 | TRUE |
| COPRS | 7104 | 2.563741 | 0.0103551 | 0.03414489 | TRUE |
| TPT1 | 9038 | 2.562055 | 0.0104055 | 0.03428218 | TRUE |
| SYP | 333 | 2.5611 | 0.0104341 | 0.03436932 | TRUE |
| LRRN1 | 11906 | 2.560624 | 0.0104484 | 0.03440194 | TRUE |
| MOV10 | 7588 | 2.560361 | 0.0104563 | 0.03442074 | TRUE |
| PLPBP | 607 | 2.559904 | 0.0104701 | 0.03445878 | TRUE |
| REEP1 | 12555 | 2.558661 | 0.0105076 | 0.03455342 | TRUE |
| SMIM30 | 10377 | 2.558658 | 0.0105077 | 0.03455342 | TRUE |
| SEMA3C | 8626 | 2.558534 | 0.0105115 | 0.03455848 | TRUE |
| SLC44A3 | 13086 | 2.557313 | 0.0105484 | 0.03467274 | TRUE |
| PITPNM2 | 9960 | 2.556304 | 0.0105791 | 0.03476213 | TRUE |
| PCDHGC3 | 5588 | 2.556271 | 0.0105801 | 0.03476213 | TRUE |
| FLJ37035 | 9786 | 2.55561 | 0.0106002 | 0.03482091 | TRUE |
| DPYD | 3750 | 2.554974 | 0.0106196 | 0.03486 | TRUE |
| MEDAG | 13545 | 2.554711 | 0.0106276 | 0.034867 | TRUE |
| GSTP1 | 4236 | 2.554014 | 0.0106489 | 0.03492955 | TRUE |
| CLEC4A | 12236 | 2.553498 | 0.0106647 | 0.03497401 | TRUE |
| NUP85 | 328 | 2.55216 | 0.0107057 | 0.03509389 | TRUE |
| NECTIN2 | 5206 | 2.551804 | 0.0107167 | 0.03512008 | TRUE |
| 4-Mar | 1870 | 2.551318 | 0.0107316 | 0.03514931 | TRUE |
| LSM7 | 11911 | 2.550731 | 0.0107497 | 0.03519382 | TRUE |
| NENF | 8979 | 2.549748 | 0.0107801 | 0.03527104 | TRUE |
| PRRT1 | 1676 | 2.549159 | 0.0107983 | 0.03532327 | TRUE |
| AHCY | 13446 | 2.548394 | 0.010822 | 0.03539343 | TRUE |
| MED10 | 15331 | 2.54547 | 0.0109131 | 0.03565394 | TRUE |
| TMPRSS5 | 9176 | 2.543041 | 0.0109892 | 0.03587277 | TRUE |
| RPL9 | 8044 | 2.542541 | 0.011005 | 0.03590216 | TRUE |
| GPM6B | 4557 | 2.542536 | 0.0110051 | 0.03590216 | TRUE |
| FAHD2CP | 11834 | 2.542182 | 0.0110163 | 0.03592355 | TRUE |
| RPS29 | 5596 | 2.539826 | 0.0110908 | 0.03613629 | TRUE |
| ADSL | 13642 | 2.539595 | 0.0110981 | 0.03615262 | TRUE |
| HEY1 | 8750 | 2.538471 | 0.0111338 | 0.03625385 | TRUE |
| MEST | 9716 | 2.538158 | 0.0111438 | 0.03627129 | TRUE |
| SHISA4 | 1371 | 2.536745 | 0.0111888 | 0.0363649 | TRUE |
| NACA2 | 1984 | 2.536604 | 0.0111933 | 0.03636663 | TRUE |
| MANBAL | 6985 | 2.536563 | 0.0111947 | 0.03636663 | TRUE |
| KPNA2 | 11668 | 2.53651 | 0.0111964 | 0.03636663 | TRUE |
| PRODH | 3953 | 2.534621 | 0.0112569 | 0.03654812 | TRUE |
| SH2D5 | 10221 | 2.533875 | 0.0112809 | 0.03661081 | TRUE |
| LMCD1 | 13287 | 2.533198 | 0.0113027 | 0.03667398 | TRUE |
| ARHGEF26 | 9131 | 2.532619 | 0.0113214 | 0.03671937 | TRUE |
| DBNL | 7483 | 2.531833 | 0.0113468 | 0.03679414 | TRUE |
| CCDC110 | 3974 | 2.530412 | 0.0113929 | 0.03693585 | TRUE |
| TRAIP | 7394 | 2.529693 | 0.0114162 | 0.03699628 | TRUE |
| ATL1 | 89 | 2.529338 | 0.0114278 | 0.03702605 | TRUE |
| HSPBP1 | 7848 | 2.529051 | 0.0114371 | 0.03704866 | TRUE |
| KCNA4 | 7214 | 2.527427 | 0.0114902 | 0.03720283 | TRUE |
| LOC728715 | 6154 | 2.52687 | 0.0115084 | 0.03724094 | TRUE |
| C19orf38 | 13650 | 2.526444 | 0.0115224 | 0.03727841 | TRUE |
| NT5DC3 | 12666 | 2.526254 | 0.0115286 | 0.03729087 | TRUE |
| PLPP3 | 2744 | 2.524879 | 0.0115738 | 0.03742934 | TRUE |
| AIMP2 | 3851 | 2.524286 | 0.0115934 | 0.03747704 | TRUE |
| TP53BP1 | 4403 | 2.524119 | 0.0115989 | 0.03748709 | TRUE |
| PQBP1 | 3924 | 2.523895 | 0.0116063 | 0.03750323 | TRUE |
| RPS2P32 | 10837 | 2.520478 | 0.0117196 | 0.03780682 | TRUE |
| SLITRK1 | 2425 | 2.520213 | 0.0117284 | 0.03781188 | TRUE |
| GJB6 | 8658 | 2.518705 | 0.0117787 | 0.0379429 | TRUE |
| NAA11 | 12891 | 2.517735 | 0.0118112 | 0.0380397 | TRUE |
| C19orf25 | 14613 | 2.517122 | 0.0118318 | 0.03809811 | TRUE |
| KIF1BP | 1856 | 2.515788 | 0.0118767 | 0.03821112 | TRUE |
| MRPL55 | 10627 | 2.515092 | 0.0119001 | 0.03826301 | TRUE |
| NR1H3 | 2441 | 2.514991 | 0.0119035 | 0.03826611 | TRUE |
| S100A16 | 13651 | 2.514676 | 0.0119142 | 0.03828456 | TRUE |
| GPR6 | 5993 | 2.513608 | 0.0119503 | 0.03836914 | TRUE |
| CDC14A | 5058 | 2.513177 | 0.0119649 | 0.03840408 | TRUE |
| RLBP1 | 8163 | 2.513142 | 0.0119661 | 0.03840408 | TRUE |
| P2RY6 | 7772 | 2.512188 | 0.0119985 | 0.03850016 | TRUE |
| SSBP1 | 4720 | 2.511949 | 0.0120066 | 0.03851109 | TRUE |
| PSMB3 | 10166 | 2.510399 | 0.0120595 | 0.03864027 | TRUE |
| C19orf53 | 12879 | 2.510011 | 0.0120727 | 0.0386669 | TRUE |
| DOK5 | 10411 | 2.509531 | 0.0120892 | 0.03871156 | TRUE |
| TUBA1C | 3022 | 2.50918 | 0.0121012 | 0.03872625 | TRUE |
| SSBP2 | 8255 | 2.507843 | 0.0121471 | 0.03885718 | TRUE |
| LANCL2 | 13568 | 2.506261 | 0.0122016 | 0.03901552 | TRUE |
| CC2D2B | 2431 | 2.505888 | 0.0122144 | 0.03904871 | TRUE |
| LINC01351 | 9947 | 2.505266 | 0.0122359 | 0.03910946 | TRUE |
| PNP | 2402 | 2.503993 | 0.01228 | 0.03922639 | TRUE |
| PIANP | 8939 | 2.50383 | 0.0122857 | 0.03923645 | TRUE |
| FGL2 | 5164 | 2.503346 | 0.0123025 | 0.03928213 | TRUE |
| S100A6 | 1519 | 2.502735 | 0.0123238 | 0.03934197 | TRUE |
| FCGR1B | 13449 | 2.502187 | 0.0123429 | 0.03939488 | TRUE |
| TMEM200A | 8208 | 2.502042 | 0.0123479 | 0.03940297 | TRUE |
| OTUD1 | 3070 | 2.501347 | 0.0123722 | 0.03947233 | TRUE |
| CEP170B | 12756 | 2.501096 | 0.012381 | 0.0394842 | TRUE |
| FAM228B | 9582 | 2.500813 | 0.0123909 | 0.03949965 | TRUE |
| RPL36A | 4881 | 2.500646 | 0.0123967 | 0.03950262 | TRUE |
| CTU1 | 8496 | 2.500258 | 0.0124103 | 0.03953741 | TRUE |
| HSPA8 | 15482 | 2.497525 | 0.0125064 | 0.03979178 | TRUE |
| DGKB | 12343 | 2.496514 | 0.0125421 | 0.03989221 | TRUE |
| HSD17B14 | 11309 | 2.496397 | 0.0125462 | 0.03989725 | TRUE |
| LCN12 | 15626 | 2.495969 | 0.0125614 | 0.03993391 | TRUE |
| SIRPA | 5639 | 2.495626 | 0.0125735 | 0.03995968 | TRUE |
| C19orf12 | 9856 | 2.495115 | 0.0125916 | 0.04000914 | TRUE |
| ANOS1 | 10720 | 2.494936 | 0.012598 | 0.0400212 | TRUE |
| TAC3 | 13474 | 2.494711 | 0.012606 | 0.04003844 | TRUE |
| CUTA | 14383 | 2.494118 | 0.0126271 | 0.04008911 | TRUE |
| ABHD4 | 11653 | 2.492738 | 0.0126762 | 0.04022074 | TRUE |
| LRFN2 | 8825 | 2.491545 | 0.0127189 | 0.04033151 | TRUE |
| STIP1 | 2849 | 2.490723 | 0.0127483 | 0.04040035 | TRUE |
| FAM110B | 12088 | 2.490433 | 0.0127588 | 0.04042514 | TRUE |
| HLA-DRB4 | 14196 | 2.48976 | 0.0127829 | 0.04049355 | TRUE |
| EDN1 | 528 | 2.489125 | 0.0128058 | 0.04054952 | TRUE |
| ST8SIA4 | 11382 | 2.487804 | 0.0128535 | 0.04067572 | TRUE |
| SYTL4 | 12998 | 2.487723 | 0.0128564 | 0.04067675 | TRUE |
| PTBP2 | 7253 | 2.487139 | 0.0128775 | 0.0407271 | TRUE |
| HLA-DRB1 | 1505 | 2.484252 | 0.0129824 | 0.04099247 | TRUE |
| NSMCE1 | 1811 | 2.482539 | 0.013045 | 0.04115684 | TRUE |
| TLR3 | 8055 | 2.482282 | 0.0130544 | 0.04117823 | TRUE |
| HSPA6 | 14384 | 2.481761 | 0.0130735 | 0.04123016 | TRUE |
| RPL28 | 4041 | 2.481506 | 0.0130829 | 0.04124303 | TRUE |
| 1-Mar | 10457 | 2.480641 | 0.0131146 | 0.04133411 | TRUE |
| PSMD4 | 12228 | 2.47941 | 0.01316 | 0.04146114 | TRUE |
| PHGR1 | 10959 | 2.478857 | 0.0131804 | 0.04151711 | TRUE |
| ZNF217 | 13862 | 2.478526 | 0.0131927 | 0.04154728 | TRUE |
| HIST1H2BD | 10386 | 2.476281 | 0.0132759 | 0.0417674 | TRUE |
| PWP1 | 3409 | 2.475526 | 0.013304 | 0.04182967 | TRUE |
| SELENON | 386 | 2.47539 | 0.0133091 | 0.04182967 | TRUE |
| MLIP | 13346 | 2.474749 | 0.013333 | 0.04187114 | TRUE |
| KANSL1-AS1 | 6628 | 2.474406 | 0.0133458 | 0.04189454 | TRUE |
| CLNS1A | 12162 | 2.474308 | 0.0133495 | 0.04189762 | TRUE |
| PLD4 | 4012 | 2.473703 | 0.0133721 | 0.04194454 | TRUE |
| BSPRY | 13306 | 2.472664 | 0.013411 | 0.04204184 | TRUE |
| DLEC1 | 5193 | 2.471905 | 0.0134395 | 0.0421211 | TRUE |
| C1orf54 | 14334 | 2.470329 | 0.0134989 | 0.04227325 | TRUE |
| METAP1 | 13006 | 2.469039 | 0.0135476 | 0.04241745 | TRUE |
| USP9X | 6528 | 2.468679 | 0.0135613 | 0.04245164 | TRUE |
| MVD | 8638 | 2.468057 | 0.0135849 | 0.04249995 | TRUE |
| MCRIP1 | 1796 | 2.467074 | 0.0136222 | 0.04259975 | TRUE |
| WIZ | 5172 | 2.46695 | 0.0136269 | 0.04260599 | TRUE |
| RREB1 | 5223 | 2.465911 | 0.0136665 | 0.04269561 | TRUE |
| SMIM2-AS1 | 10191 | 2.464265 | 0.0137295 | 0.04284939 | TRUE |
| COX6A1 | 2732 | 2.464023 | 0.0137387 | 0.04286977 | TRUE |
| FBXL16 | 3031 | 2.462526 | 0.0137962 | 0.04301481 | TRUE |
| CDK20 | 7779 | 2.462172 | 0.0138098 | 0.04304871 | TRUE |
| LILRB4 | 9453 | 2.459975 | 0.0138947 | 0.04325276 | TRUE |
| UPP2 | 2107 | 2.457769 | 0.0139803 | 0.04347606 | TRUE |
| HIST3H2BB | 5528 | 2.457284 | 0.0139992 | 0.04352614 | TRUE |
| UBE2MP1 | 1937 | 2.45638 | 0.0140345 | 0.04362713 | TRUE |
| GPR26 | 4120 | 2.455019 | 0.0140877 | 0.04374046 | TRUE |
| SLCO1C1 | 14201 | 2.453655 | 0.0141413 | 0.04388057 | TRUE |
| ZNF438 | 11909 | 2.453399 | 0.0141513 | 0.0439031 | TRUE |
| TFG | 8214 | 2.451809 | 0.014214 | 0.04405385 | TRUE |
| ATF7IP2 | 1625 | 2.45171 | 0.0142179 | 0.04405667 | TRUE |
| SRSF12 | 4911 | 2.451578 | 0.0142231 | 0.04405667 | TRUE |
| UBB | 1900 | 2.451145 | 0.0142403 | 0.04410023 | TRUE |
| KIF1B | 11693 | 2.449893 | 0.0142899 | 0.04422758 | TRUE |
| CHP1 | 3487 | 2.449524 | 0.0143045 | 0.04426416 | TRUE |
| GRN | 5842 | 2.449296 | 0.0143136 | 0.04427466 | TRUE |
| LOC101929066 | 8235 | 2.448909 | 0.014329 | 0.04431349 | TRUE |
| RPL23AP82 | 1048 | 2.447259 | 0.0143947 | 0.04449049 | TRUE |
| COA6 | 12235 | 2.446595 | 0.0144213 | 0.04456372 | TRUE |
| SLC2A14 | 15555 | 2.443992 | 0.0145257 | 0.04484223 | TRUE |
| TMEM187 | 5363 | 2.442634 | 0.0145805 | 0.04499353 | TRUE |
| CROT | 8240 | 2.441933 | 0.0146089 | 0.0450632 | TRUE |
| POLR2I | 10982 | 2.441808 | 0.0146139 | 0.04506991 | TRUE |
| DZIP3 | 13832 | 2.44162 | 0.0146215 | 0.04508449 | TRUE |
| SLC44A2 | 5204 | 2.441193 | 0.0146388 | 0.04512453 | TRUE |
| GADD45GIP1 | 9711 | 2.441157 | 0.0146403 | 0.04512453 | TRUE |
| SCAMP1-AS1 | 8915 | 2.440087 | 0.0146837 | 0.04524058 | TRUE |
| PNOC | 11413 | 2.438365 | 0.0147539 | 0.04542088 | TRUE |
| NIP7 | 8135 | 2.436783 | 0.0148186 | 0.04557519 | TRUE |
| STMN1 | 5285 | 2.435291 | 0.0148798 | 0.04575456 | TRUE |
| UBE2Q1 | 6900 | 2.434749 | 0.0149021 | 0.04581414 | TRUE |
| TMEM18 | 5242 | 2.434052 | 0.0149309 | 0.04588441 | TRUE |
| RTN1 | 3029 | 2.433141 | 0.0149685 | 0.04597621 | TRUE |
| TMIGD3 | 15135 | 2.433115 | 0.0149695 | 0.04597621 | TRUE |
| FADS2 | 10015 | 2.432724 | 0.0149857 | 0.04601684 | TRUE |
| NMNAT3 | 5725 | 2.432289 | 0.0150037 | 0.0460631 | TRUE |
| RIT2 | 8686 | 2.43133 | 0.0150435 | 0.04614897 | TRUE |
| TTC23L | 1833 | 2.43106 | 0.0150547 | 0.04617431 | TRUE |
| SEC11C | 1713 | 2.42944 | 0.0151222 | 0.04635389 | TRUE |
| CHCHD2 | 11794 | 2.427691 | 0.0151953 | 0.04653239 | TRUE |
| LRP10 | 3501 | 2.425852 | 0.0152725 | 0.04674138 | TRUE |
| DRAM1 | 1803 | 2.425762 | 0.0152763 | 0.04674382 | TRUE |
| MDFIC | 3725 | 2.424537 | 0.0153279 | 0.04685596 | TRUE |
| EPB41L4A-AS1 | 11352 | 2.423595 | 0.0153677 | 0.04696848 | TRUE |
| 8-Mar | 11498 | 2.423409 | 0.0153756 | 0.04698335 | TRUE |
| ANGPT4 | 5144 | 2.423264 | 0.0153817 | 0.04699293 | TRUE |
| TNFAIP2 | 13820 | 2.421674 | 0.0154492 | 0.04715295 | TRUE |
| PFDN5 | 13577 | 2.420402 | 0.0155034 | 0.04729976 | TRUE |
| ITM2C | 9239 | 2.420085 | 0.0155169 | 0.04733178 | TRUE |
| CTSO | 9011 | 2.419938 | 0.0155232 | 0.04734168 | TRUE |
| NR2F2 | 8045 | 2.419577 | 0.0155386 | 0.04736098 | TRUE |
| ASH1L-AS1 | 10839 | 2.419158 | 0.0155565 | 0.04739892 | TRUE |
| MYBPC2 | 8193 | 2.41773 | 0.0156177 | 0.04754642 | TRUE |
| ZG16B | 275 | 2.417154 | 0.0156424 | 0.04760269 | TRUE |
| INHBB | 8954 | 2.416373 | 0.015676 | 0.04766832 | TRUE |
| POLR3K | 9693 | 2.415607 | 0.015709 | 0.04774084 | TRUE |
| SEC14L2 | 6005 | 2.415428 | 0.0157167 | 0.04775503 | TRUE |
| PACSIN1 | 14355 | 2.415164 | 0.0157281 | 0.04778037 | TRUE |
| C1orf122 | 7885 | 2.414977 | 0.0157362 | 0.04779562 | TRUE |
| ANKRD2 | 5316 | 2.414766 | 0.0157453 | 0.04781403 | TRUE |
| BBOX1 | 736 | 2.413648 | 0.0157937 | 0.04795166 | TRUE |
| WDR77 | 14146 | 2.41303 | 0.0158205 | 0.0480144 | TRUE |
| USE1 | 1492 | 2.412455 | 0.0158455 | 0.04807153 | TRUE |
| ZFAS1 | 14820 | 2.412337 | 0.0158506 | 0.04807776 | TRUE |
| RPL24 | 9645 | 2.4099 | 0.0159569 | 0.04836257 | TRUE |
| HLA-DOA | 5291 | 2.407901 | 0.0160445 | 0.04857168 | TRUE |
| PSMC6 | 8916 | 2.40739 | 0.016067 | 0.04863029 | TRUE |
| RPS15 | 14697 | 2.407037 | 0.0160825 | 0.04865399 | TRUE |
| MT1L | 1954 | 2.407 | 0.0160842 | 0.04865399 | TRUE |
| TRAPPC5 | 8107 | 2.406478 | 0.0161072 | 0.04869531 | TRUE |
| COL11A1 | 8165 | 2.406406 | 0.0161103 | 0.04869549 | TRUE |
| SNHG19 | 5866 | 2.406014 | 0.0161276 | 0.04873835 | TRUE |
| IQCD | 15419 | 2.405792 | 0.0161375 | 0.04875727 | TRUE |
| NICN1 | 9918 | 2.404517 | 0.0161939 | 0.04888174 | TRUE |
| KLRC3 | 8025 | 2.404204 | 0.0162077 | 0.04891417 | TRUE |
| INTU | 3612 | 2.402993 | 0.0162615 | 0.04903858 | TRUE |
| TBL2 | 749 | 2.402689 | 0.016275 | 0.04906044 | TRUE |
| CD200 | 6115 | 2.402556 | 0.0162809 | 0.04906882 | TRUE |
| MSRA | 11065 | 2.399973 | 0.0163963 | 0.04934984 | TRUE |
| MRPS24 | 5668 | 2.399805 | 0.0164038 | 0.04936299 | TRUE |
| MGAT4C | 3494 | 2.399641 | 0.0164112 | 0.0493756 | TRUE |
| BOP1 | 3848 | 2.399012 | 0.0164394 | 0.04945098 | TRUE |
| FAM241A | 10179 | 2.398259 | 0.0164732 | 0.04952418 | TRUE |
| RCN1 | 14260 | 2.397348 | 0.0165142 | 0.04961887 | TRUE |
| EEF1B2 | 6802 | 2.396311 | 0.016561 | 0.04972126 | TRUE |
| SEMA6B | 10596 | 2.3953 | 0.0166068 | 0.04983944 | TRUE |
| SYTL3 | 4938 | 2.394753 | 0.0166316 | 0.0498947 | TRUE |
| EPHA5 | 14274 | 2.393923 | 0.0166692 | 0.04997897 | TRUE |
| SLC16A9 | 11342 | 2.392149 | 0.01675 | 0.0501731 | FALSE |
| CDK4 | 11273 | 2.390963 | 0.0168042 | 0.05030655 | FALSE |
| ITGB8 | 14143 | 2.390714 | 0.0168156 | 0.0503214 | FALSE |
| ABCA1 | 13970 | 2.390033 | 0.0168469 | 0.05039551 | FALSE |
| ATP6V0A4 | 5625 | 2.38856 | 0.0169145 | 0.05057863 | FALSE |
| TUBA3FP | 11578 | 2.387963 | 0.016942 | 0.0506415 | FALSE |
| MFAP1 | 6047 | 2.386673 | 0.0170016 | 0.0508001 | FALSE |
| GORAB | 11297 | 2.386375 | 0.0170154 | 0.05083157 | FALSE |
| IARS2 | 14471 | 2.385276 | 0.0170663 | 0.05096421 | FALSE |
| RPS6 | 1368 | 2.384064 | 0.0171226 | 0.05111285 | FALSE |
| HSDL2 | 15019 | 2.382319 | 0.017204 | 0.05133612 | FALSE |
| CYS1 | 10487 | 2.381361 | 0.0172488 | 0.0514502 | FALSE |
| CRYBB2 | 5553 | 2.379361 | 0.0173427 | 0.05169078 | FALSE |
| MEX3A | 7241 | 2.379189 | 0.0173508 | 0.05169078 | FALSE |
| LOC101060091 | 1193 | 2.379103 | 0.0173548 | 0.05169078 | FALSE |
| RPS9 | 4363 | 2.378086 | 0.0174028 | 0.0518106 | FALSE |
| SPON1 | 14320 | 2.377696 | 0.0174212 | 0.05184003 | FALSE |
| RPL13AP3 | 11692 | 2.377187 | 0.0174452 | 0.05189752 | FALSE |
| LINC00467 | 8634 | 2.375772 | 0.0175123 | 0.05208704 | FALSE |
| KCNH5 | 9981 | 2.375066 | 0.0175458 | 0.05214709 | FALSE |
| BRI3 | 8101 | 2.374964 | 0.0175507 | 0.05215159 | FALSE |
| RAMP1 | 10788 | 2.37463 | 0.0175665 | 0.05218888 | FALSE |
| CFL1P1 | 14186 | 2.374559 | 0.0175699 | 0.052189 | FALSE |
| GPKOW | 6015 | 2.373626 | 0.0176144 | 0.05226146 | FALSE |
| RPL7L1 | 4762 | 2.372974 | 0.0176455 | 0.05232869 | FALSE |
| CALY | 2434 | 2.372575 | 0.0176646 | 0.05237063 | FALSE |
| MAPK8IP2 | 10243 | 2.37111 | 0.0177348 | 0.05253883 | FALSE |
| AP2S1 | 6060 | 2.369903 | 0.0177928 | 0.0526807 | FALSE |
| QSOX1 | 3615 | 2.368884 | 0.0178418 | 0.05279835 | FALSE |
| PRKD2 | 817 | 2.368206 | 0.0178746 | 0.05287291 | FALSE |
| KYNU | 6145 | 2.366791 | 0.0179431 | 0.05304537 | FALSE |
| LINC01176 | 14026 | 2.365818 | 0.0179903 | 0.05315482 | FALSE |
| CD163 | 1285 | 2.365638 | 0.017999 | 0.05316057 | FALSE |
| TAGLN2 | 9986 | 2.36461 | 0.0180491 | 0.05328821 | FALSE |
| TOMM7 | 1894 | 2.364304 | 0.018064 | 0.05332218 | FALSE |
| APOC1 | 6586 | 2.364096 | 0.0180741 | 0.05334205 | FALSE |
| ATP6V0D1 | 3863 | 2.363777 | 0.0180897 | 0.05337793 | FALSE |
| PTAFR | 2826 | 2.363287 | 0.0181136 | 0.05342839 | FALSE |
| SLC2A8 | 13838 | 2.362885 | 0.0181333 | 0.0534662 | FALSE |
| DRAP1 | 9247 | 2.362132 | 0.0181702 | 0.0535494 | FALSE |
| TMEM233 | 271 | 2.362099 | 0.0181718 | 0.0535494 | FALSE |
| C2orf81 | 15432 | 2.361671 | 0.0181928 | 0.05360115 | FALSE |
| DNAJB2 | 5591 | 2.361537 | 0.0181994 | 0.05361043 | FALSE |
| C1QTNF12 | 192 | 2.361159 | 0.0182179 | 0.05365499 | FALSE |
| CTBS | 12486 | 2.360985 | 0.0182265 | 0.05365996 | FALSE |
| C16orf89 | 9860 | 2.360477 | 0.0182515 | 0.05369306 | FALSE |
| LAMB1 | 15093 | 2.359629 | 0.0182932 | 0.05378558 | FALSE |
| CLPS | 7797 | 2.358549 | 0.0183465 | 0.05389166 | FALSE |
| SBF1 | 8104 | 2.357583 | 0.0183943 | 0.05402194 | FALSE |
| DUSP2 | 13447 | 2.356254 | 0.0184603 | 0.05417217 | FALSE |
| RPL35A | 13712 | 2.356203 | 0.0184628 | 0.05417217 | FALSE |
| KLRC2 | 2143 | 2.355471 | 0.0184992 | 0.05424823 | FALSE |
| UNC93B1 | 13071 | 2.355443 | 0.0185006 | 0.05424823 | FALSE |
| DNAJC15 | 13964 | 2.355169 | 0.0185143 | 0.05427223 | FALSE |
| HID1 | 4281 | 2.353293 | 0.018608 | 0.0545264 | FALSE |
| PIGF | 12482 | 2.352675 | 0.0186389 | 0.05459663 | FALSE |
| COPS9 | 6491 | 2.352538 | 0.0186458 | 0.05460651 | FALSE |
| LOC654780 | 5947 | 2.351535 | 0.0186961 | 0.0547232 | FALSE |
| MEA1 | 14233 | 2.351199 | 0.018713 | 0.05476239 | FALSE |
| DCAF7 | 11659 | 2.349365 | 0.0188055 | 0.05500202 | FALSE |
| NOP16 | 12155 | 2.34838 | 0.0188553 | 0.05512708 | FALSE |
| HIST2H2AB | 13935 | 2.347207 | 0.0189147 | 0.05526554 | FALSE |
| VSIG10L | 4959 | 2.347167 | 0.0189168 | 0.05526554 | FALSE |
| GABRA2 | 5638 | 2.345866 | 0.0189829 | 0.0554381 | FALSE |
| EMC6 | 4106 | 2.345792 | 0.0189867 | 0.05543875 | FALSE |
| C1QBP | 2601 | 2.345085 | 0.0190227 | 0.05553362 | FALSE |
| C10orf105 | 12105 | 2.343619 | 0.0190977 | 0.05572114 | FALSE |
| SFXN5 | 4594 | 2.340911 | 0.0192368 | 0.05607461 | FALSE |
| RAB13 | 1188 | 2.340051 | 0.0192811 | 0.05619343 | FALSE |
| HBZ | 689 | 2.339903 | 0.0192887 | 0.05620522 | FALSE |
| TM7SF2 | 7185 | 2.338425 | 0.0193652 | 0.05639649 | FALSE |
| CTR9 | 2126 | 2.338172 | 0.0193783 | 0.05641367 | FALSE |
| UBE2L6 | 11401 | 2.335694 | 0.0195072 | 0.05674656 | FALSE |
| COL10A1 | 12770 | 2.334355 | 0.0195771 | 0.05688849 | FALSE |
| CHRM3 | 889 | 2.334089 | 0.0195911 | 0.05691387 | FALSE |
| LYRM1 | 11079 | 2.333508 | 0.0196215 | 0.05698363 | FALSE |
| RPL10A | 10380 | 2.332813 | 0.019658 | 0.05706062 | FALSE |
| PTH2 | 9274 | 2.332564 | 0.019671 | 0.05707449 | FALSE |
| PYGO2 | 9690 | 2.331534 | 0.0197252 | 0.05721046 | FALSE |
| AFF2 | 8088 | 2.331392 | 0.0197327 | 0.05722153 | FALSE |
| PPIB | 7202 | 2.331207 | 0.0197424 | 0.05723918 | FALSE |
| RNF135 | 6232 | 2.330854 | 0.0197611 | 0.05728252 | FALSE |
| MRAP2 | 8527 | 2.32987 | 0.019813 | 0.05741186 | FALSE |
| PGRMC1 | 6534 | 2.329206 | 0.0198482 | 0.05749235 | FALSE |
| UQCC3 | 2347 | 2.328561 | 0.0198823 | 0.05757002 | FALSE |
| ABHD12B | 14073 | 2.327874 | 0.0199188 | 0.05766491 | FALSE |
| SLC25A18 | 3854 | 2.326584 | 0.0199874 | 0.05783144 | FALSE |
| SEC23IP | 13740 | 2.326423 | 0.019996 | 0.05784556 | FALSE |
| FYN | 14524 | 2.325988 | 0.0200192 | 0.05789123 | FALSE |
| GPS2 | 8524 | 2.325716 | 0.0200337 | 0.05792197 | FALSE |
| APMAP | 9355 | 2.32479 | 0.0200832 | 0.0580441 | FALSE |
| VAPA | 6377 | 2.324408 | 0.0201036 | 0.05809245 | FALSE |
| LOC101930085 | 4460 | 2.324322 | 0.0201082 | 0.05809502 | FALSE |
| TRIP6 | 4865 | 2.323574 | 0.0201483 | 0.05818935 | FALSE |
| HMGCL | 775 | 2.320333 | 0.0203229 | 0.05860679 | FALSE |
| COX7A2L | 13867 | 2.320206 | 0.0203297 | 0.05861578 | FALSE |
| MMP14 | 6617 | 2.318958 | 0.0203973 | 0.0587781 | FALSE |
| CERS6 | 4497 | 2.317468 | 0.0204783 | 0.05894615 | FALSE |
| CUL2 | 14203 | 2.316548 | 0.0205284 | 0.05905413 | FALSE |
| RXFP1 | 7283 | 2.315922 | 0.0205625 | 0.05911254 | FALSE |
| CRACR2B | 11044 | 2.31557 | 0.0205818 | 0.05915695 | FALSE |
| STARD7 | 8522 | 2.314503 | 0.0206401 | 0.05924959 | FALSE |
| MAN2B2 | 2810 | 2.311822 | 0.0207875 | 0.05959488 | FALSE |
| UBE2N | 2352 | 2.311436 | 0.0208088 | 0.05964498 | FALSE |
| ISLR2 | 2076 | 2.310994 | 0.0208332 | 0.05969905 | FALSE |
| PLPPR1 | 2069 | 2.310836 | 0.0208419 | 0.0597071 | FALSE |
| THNSL2 | 9146 | 2.309649 | 0.0209076 | 0.05983693 | FALSE |
| SRP14 | 2316 | 2.309639 | 0.0209081 | 0.05983693 | FALSE |
| UBA7 | 14737 | 2.309071 | 0.0209396 | 0.05991022 | FALSE |
| IFI35 | 14680 | 2.308087 | 0.0209943 | 0.06004205 | FALSE |
| SPINT2 | 14869 | 2.307261 | 0.0210403 | 0.06012111 | FALSE |
| CCDC77 | 5772 | 2.307074 | 0.0210507 | 0.0601399 | FALSE |
| NISCH | 4048 | 2.306074 | 0.0211065 | 0.06024427 | FALSE |
| HIF3A | 8119 | 2.304751 | 0.0211805 | 0.06042247 | FALSE |
| FADS1 | 6891 | 2.304579 | 0.0211902 | 0.06043894 | FALSE |
| SGSH | 8417 | 2.304376 | 0.0212015 | 0.06046037 | FALSE |
| METTL9 | 8932 | 2.303626 | 0.0212436 | 0.06055834 | FALSE |
| ALAS2 | 8553 | 2.303541 | 0.0212484 | 0.06055981 | FALSE |
| ATP5MG | 11987 | 2.303463 | 0.0212528 | 0.06055981 | FALSE |
| LOC105374952 | 712 | 2.30341 | 0.0212558 | 0.06055981 | FALSE |
| LINC00158 | 14134 | 2.302431 | 0.0213109 | 0.06067512 | FALSE |
| OSER1 | 1477 | 2.302415 | 0.0213118 | 0.06067512 | FALSE |
| NPM3 | 3074 | 2.302075 | 0.0213309 | 0.06071862 | FALSE |
| TIMP2 | 5572 | 2.301999 | 0.0213352 | 0.06071976 | FALSE |
| HMGN2P46 | 2289 | 2.301212 | 0.0213797 | 0.06081297 | FALSE |
| RBX1 | 12847 | 2.299658 | 0.0214676 | 0.06104093 | FALSE |
| ICE1 | 3286 | 2.299282 | 0.0214889 | 0.06109046 | FALSE |
| IFI30 | 2009 | 2.298785 | 0.0215172 | 0.06113734 | FALSE |
| TBC1D10A | 10853 | 2.298377 | 0.0215403 | 0.06119211 | FALSE |
| GRB14 | 11433 | 2.297839 | 0.021571 | 0.06126794 | FALSE |
| NEUROD2 | 12101 | 2.295782 | 0.0216883 | 0.06153429 | FALSE |
| TMEM86A | 1747 | 2.294706 | 0.02175 | 0.06169795 | FALSE |
| ARHGDIB | 8706 | 2.294523 | 0.0217605 | 0.06171653 | FALSE |
| UROS | 7719 | 2.294256 | 0.0217758 | 0.06174879 | FALSE |
| ZNF239 | 2681 | 2.293378 | 0.0218263 | 0.06188063 | FALSE |
| CYP11A1 | 1132 | 2.292978 | 0.0218493 | 0.06193466 | FALSE |
| CTBP2 | 2464 | 2.292887 | 0.0218545 | 0.06193829 | FALSE |
| EXOSC4 | 1119 | 2.291409 | 0.0219398 | 0.06214893 | FALSE |
| CASC10 | 11397 | 2.291358 | 0.0219427 | 0.06214893 | FALSE |
| LINC02361 | 675 | 2.291323 | 0.0219447 | 0.06214893 | FALSE |
| DRD2 | 14840 | 2.290906 | 0.0219689 | 0.06220595 | FALSE |
| RFXANK | 13069 | 2.290474 | 0.0219939 | 0.06226547 | FALSE |
| TAMM41 | 1333 | 2.288432 | 0.0221124 | 0.06256702 | FALSE |
| BIN1 | 6653 | 2.287827 | 0.0221476 | 0.06262731 | FALSE |
| ENY2 | 11733 | 2.287114 | 0.0221892 | 0.0627162 | FALSE |
| TRAF3IP2 | 4939 | 2.286263 | 0.0222389 | 0.06284533 | FALSE |
| HTR3B | 9978 | 2.285989 | 0.0222549 | 0.06287925 | FALSE |
| 11-Sep | 6026 | 2.285832 | 0.0222641 | 0.06289384 | FALSE |
| NME7 | 2223 | 2.285456 | 0.0222861 | 0.06294465 | FALSE |
| HIST1H2BJ | 9998 | 2.285067 | 0.0223089 | 0.06298566 | FALSE |
| REXO5 | 4238 | 2.282573 | 0.0224555 | 0.06333165 | FALSE |
| RPL13 | 6126 | 2.281983 | 0.0224903 | 0.06340695 | FALSE |
| LOC102723968 | 11109 | 2.281281 | 0.0225318 | 0.06350099 | FALSE |
| CHRDL1 | 2135 | 2.280693 | 0.0225666 | 0.06357613 | FALSE |
| RPL10 | 9676 | 2.280511 | 0.0225774 | 0.06359504 | FALSE |
| MAEA | 3153 | 2.280246 | 0.0225931 | 0.06362781 | FALSE |
| ISCA2 | 130 | 2.280062 | 0.022604 | 0.06364707 | FALSE |
| ESD | 15181 | 2.279489 | 0.022638 | 0.06373133 | FALSE |
| GLTPD2 | 597 | 2.278332 | 0.0227068 | 0.06387898 | FALSE |
| RAB11A | 1276 | 2.278025 | 0.0227251 | 0.06390741 | FALSE |
| GOSR2 | 3778 | 2.277599 | 0.0227505 | 0.06395581 | FALSE |
| DNAJC1 | 3618 | 2.276604 | 0.0228099 | 0.06408823 | FALSE |
| PRDX5 | 12605 | 2.27541 | 0.0228813 | 0.06424283 | FALSE |
| LOC105373383 | 778 | 2.275092 | 0.0229004 | 0.06428482 | FALSE |
| LOC344967 | 3616 | 2.274841 | 0.0229155 | 0.06430399 | FALSE |
| MMGT1 | 6874 | 2.274137 | 0.0229578 | 0.06438797 | FALSE |
| WDR26 | 2696 | 2.27349 | 0.0229967 | 0.06447398 | FALSE |
| PSMD7 | 4267 | 2.271522 | 0.0231154 | 0.06476039 | FALSE |
| MT1G | 5133 | 2.271108 | 0.0231404 | 0.06481894 | FALSE |
| UQCRQ | 7616 | 2.270753 | 0.0231619 | 0.06486753 | FALSE |
| ZNF185 | 15476 | 2.270384 | 0.0231843 | 0.06491538 | FALSE |
| ITPRIPL2 | 8100 | 2.270334 | 0.0231873 | 0.06491538 | FALSE |
| EVI2B | 426 | 2.269133 | 0.0232602 | 0.0650311 | FALSE |
| VIM | 5683 | 2.269105 | 0.0232619 | 0.0650311 | FALSE |
| PREB | 2082 | 2.268854 | 0.0232772 | 0.06505051 | FALSE |
| ABCC12 | 11821 | 2.26774 | 0.0233451 | 0.06520516 | FALSE |
| AMN1 | 2901 | 2.2666 | 0.0234147 | 0.06538693 | FALSE |
| NR2E1 | 6881 | 2.266469 | 0.0234227 | 0.06538693 | FALSE |
| MRPS18A | 10753 | 2.266311 | 0.0234323 | 0.06540223 | FALSE |
| LOC100049716 | 11529 | 2.265593 | 0.0234763 | 0.06547818 | FALSE |
| VEGFB | 1020 | 2.265176 | 0.0235019 | 0.0655378 | FALSE |
| CENPX | 5309 | 2.264905 | 0.0235185 | 0.06557248 | FALSE |
| NRP2 | 10443 | 2.263844 | 0.0235837 | 0.06573082 | FALSE |
| TMEM186 | 5922 | 2.261833 | 0.0237077 | 0.06602937 | FALSE |
| TMEM173 | 5608 | 2.261422 | 0.0237331 | 0.06608838 | FALSE |
| DCHS1 | 13554 | 2.261038 | 0.0237569 | 0.06614146 | FALSE |
| PLEKHO2 | 10836 | 2.260909 | 0.0237649 | 0.06614146 | FALSE |
| DIAPH2 | 11734 | 2.260406 | 0.0237961 | 0.06621643 | FALSE |
| MESD | 5114 | 2.260043 | 0.0238186 | 0.06624613 | FALSE |
| KIF3A | 14743 | 2.260029 | 0.0238195 | 0.06624613 | FALSE |
| UBR4 | 7678 | 2.259319 | 0.0238635 | 0.06633337 | FALSE |
| FAM131C | 5691 | 2.25883 | 0.023894 | 0.0663943 | FALSE |
| CD7 | 3707 | 2.258551 | 0.0239113 | 0.06641894 | FALSE |
| LEF1-AS1 | 13482 | 2.257482 | 0.023978 | 0.0665804 | FALSE |
| LINC01480 | 15576 | 2.256817 | 0.0240195 | 0.0666839 | FALSE |
| FAM200B | 14801 | 2.256209 | 0.0240575 | 0.06677763 | FALSE |
| MRPL52 | 6400 | 2.256126 | 0.0240627 | 0.06678019 | FALSE |
| UBE2M | 9301 | 2.254688 | 0.0241529 | 0.06694727 | FALSE |
| LINC00663 | 7875 | 2.254511 | 0.024164 | 0.0669648 | FALSE |
| SYCE3 | 9234 | 2.253589 | 0.024222 | 0.06710315 | FALSE |
| FAM111A | 5640 | 2.253133 | 0.0242508 | 0.06717084 | FALSE |
| EIF3J | 10361 | 2.252917 | 0.0242644 | 0.06719665 | FALSE |
| MRAS | 10209 | 2.252205 | 0.0243093 | 0.06730918 | FALSE |
| ZNF646 | 1252 | 2.251239 | 0.0243704 | 0.06742876 | FALSE |
| PI4KA | 5022 | 2.251234 | 0.0243707 | 0.06742876 | FALSE |
| FGFBP3 | 2187 | 2.250673 | 0.0244063 | 0.06750584 | FALSE |
| IFT74 | 3676 | 2.250476 | 0.0244187 | 0.06751455 | FALSE |
| TERF2IP | 14513 | 2.250215 | 0.0244353 | 0.06752644 | FALSE |
| GAMT | 3154 | 2.250111 | 0.0244419 | 0.06753274 | FALSE |
| IL1B | 8478 | 2.249038 | 0.0245101 | 0.06766799 | FALSE |
| PIN1 | 8350 | 2.247681 | 0.0245965 | 0.06787603 | FALSE |
| C17orf58 | 1442 | 2.247302 | 0.0246207 | 0.0679308 | FALSE |
| RARRES3 | 13511 | 2.24684 | 0.0246503 | 0.06800025 | FALSE |
| GPR143 | 1544 | 2.244738 | 0.024785 | 0.06833567 | FALSE |
| ARHGEF26-AS1 | 7227 | 2.244189 | 0.0248202 | 0.06840883 | FALSE |
| TMSB4Y | 5687 | 2.244008 | 0.0248319 | 0.06842885 | FALSE |
| LOC339803 | 4263 | 2.243327 | 0.0248757 | 0.0685186 | FALSE |
| YKT6 | 5490 | 2.243094 | 0.0248908 | 0.0685186 | FALSE |
| WDR83OS | 709 | 2.242668 | 0.0249182 | 0.06858217 | FALSE |
| VPS37C | 5476 | 2.242059 | 0.0249576 | 0.06867832 | FALSE |
| H3F3B | 5027 | 2.241003 | 0.0250259 | 0.06882393 | FALSE |
| DHRS7B | 716 | 2.239244 | 0.02514 | 0.0690832 | FALSE |
| MRPS18B | 11146 | 2.238971 | 0.0251578 | 0.06909057 | FALSE |
| GALNT11 | 4791 | 2.238931 | 0.0251604 | 0.06909057 | FALSE |
| URB1-AS1 | 11600 | 2.238659 | 0.0251781 | 0.06911491 | FALSE |
| ETFRF1 | 12952 | 2.235561 | 0.0253805 | 0.06956066 | FALSE |
| HPCA | 8211 | 2.235009 | 0.0254168 | 0.0696355 | FALSE |
| LGALS3 | 7586 | 2.234249 | 0.0254667 | 0.0697319 | FALSE |
| CASQ1 | 3051 | 2.234202 | 0.0254698 | 0.0697319 | FALSE |
| LBHD1 | 4009 | 2.233876 | 0.0254912 | 0.0697784 | FALSE |
| VTI1B | 13927 | 2.233093 | 0.0255428 | 0.0698892 | FALSE |
| RHEB | 928 | 2.232732 | 0.0255666 | 0.06993579 | FALSE |
| FANCL | 10795 | 2.23176 | 0.0256308 | 0.07009915 | FALSE |
| GPR27 | 5497 | 2.231416 | 0.0256536 | 0.07014912 | FALSE |
| ANGPTL6 | 13898 | 2.231259 | 0.025664 | 0.07015498 | FALSE |
| ATE1 | 1355 | 2.230528 | 0.0257124 | 0.07024854 | FALSE |
| DUSP3 | 3852 | 2.23036 | 0.0257236 | 0.0702667 | FALSE |
| SMIM10 | 10048 | 2.229881 | 0.0257553 | 0.07031668 | FALSE |
| MRPS33 | 2224 | 2.229026 | 0.0258122 | 0.07043965 | FALSE |
| PRICKLE2 | 10515 | 2.228909 | 0.02582 | 0.07044388 | FALSE |
| VCAN | 5623 | 2.228346 | 0.0258575 | 0.07052015 | FALSE |
| SMAD3 | 8477 | 2.226576 | 0.0259756 | 0.07079445 | FALSE |
| TRPC4 | 4770 | 2.226374 | 0.0259891 | 0.0708086 | FALSE |
| PSMC3 | 7349 | 2.226363 | 0.0259899 | 0.0708086 | FALSE |
| STARD3NL | 7590 | 2.225488 | 0.0260485 | 0.07094358 | FALSE |
| SURF1 | 9123 | 2.225359 | 0.0260571 | 0.07095174 | FALSE |
| ZWINT | 9364 | 2.225308 | 0.0260606 | 0.07095174 | FALSE |
| FADS3 | 11599 | 2.223559 | 0.0261781 | 0.07123459 | FALSE |
| CARHSP1 | 8779 | 2.223109 | 0.0262085 | 0.07130468 | FALSE |
| ADCK2 | 7469 | 2.222896 | 0.0262228 | 0.07133135 | FALSE |
| FGF2 | 10502 | 2.221432 | 0.0263217 | 0.07153416 | FALSE |
| NECAP2 | 11761 | 2.221386 | 0.0263248 | 0.07153416 | FALSE |
| NEK11 | 9418 | 2.22068 | 0.0263726 | 0.07165164 | FALSE |
| RBBP7 | 6038 | 2.219509 | 0.0264521 | 0.07185507 | FALSE |
| TMEM141 | 3643 | 2.219079 | 0.0264814 | 0.07191566 | FALSE |
| FGD4 | 434 | 2.218598 | 0.0265141 | 0.07196089 | FALSE |
| SYF2 | 6862 | 2.217539 | 0.0265863 | 0.07209628 | FALSE |
| EDARADD | 12430 | 2.216382 | 0.0266654 | 0.07228359 | FALSE |
| PLEKHH2 | 13520 | 2.215992 | 0.0266921 | 0.07234342 | FALSE |
| ANG | 4447 | 2.215835 | 0.0267028 | 0.07234748 | FALSE |
| FAM172A | 7925 | 2.215766 | 0.0267075 | 0.07234775 | FALSE |
| HEPH | 6768 | 2.215214 | 0.0267454 | 0.07243772 | FALSE |
| SMIM10L2B | 11657 | 2.213793 | 0.026843 | 0.07264428 | FALSE |
| FUT10 | 4216 | 2.21346 | 0.0268659 | 0.07268871 | FALSE |
| MS4A4A | 241 | 2.212473 | 0.026934 | 0.07283763 | FALSE |
| RTRAF | 11379 | 2.212459 | 0.026935 | 0.07283763 | FALSE |
| EIF3D | 4962 | 2.211116 | 0.0270278 | 0.07307604 | FALSE |
| RPL23AP7 | 4732 | 2.210376 | 0.0270791 | 0.07319085 | FALSE |
| RHEBL1 | 11355 | 2.209986 | 0.0271061 | 0.07323716 | FALSE |
| SEC61A2 | 1677 | 2.209569 | 0.0271351 | 0.07329006 | FALSE |
| LOC554206 | 6595 | 2.209235 | 0.0271583 | 0.07333502 | FALSE |
| BTBD9 | 14291 | 2.209127 | 0.0271658 | 0.07333502 | FALSE |
| PRR5 | 228 | 2.208586 | 0.0272035 | 0.07340518 | FALSE |
| SLC17A7 | 2229 | 2.208551 | 0.0272059 | 0.07340518 | FALSE |
| ENPP1 | 8114 | 2.20819 | 0.027231 | 0.07344767 | FALSE |
| TCEAL9 | 5421 | 2.20798 | 0.0272457 | 0.0734618 | FALSE |
| MSMO1 | 7009 | 2.20729 | 0.0272938 | 0.07356622 | FALSE |
| SLC29A3 | 306 | 2.206409 | 0.0273554 | 0.07370675 | FALSE |
| TSKU | 2659 | 2.206024 | 0.0273823 | 0.07376664 | FALSE |
| ASXL3 | 2482 | 2.205629 | 0.02741 | 0.07381573 | FALSE |
| COL16A1 | 3989 | 2.205245 | 0.0274369 | 0.07385308 | FALSE |
| ITFG1 | 2931 | 2.205229 | 0.027438 | 0.07385308 | FALSE |
| FBXL2 | 6051 | 2.203588 | 0.0275533 | 0.07411751 | FALSE |
| IFI6 | 5440 | 2.201819 | 0.0276781 | 0.07439678 | FALSE |
| DHX36 | 11947 | 2.201272 | 0.0277168 | 0.07447513 | FALSE |
| HLA-DPB1 | 13080 | 2.200821 | 0.0277487 | 0.07453529 | FALSE |
| B3GAT2 | 12292 | 2.200042 | 0.0278039 | 0.07464513 | FALSE |
| NECTIN3 | 15015 | 2.199912 | 0.0278131 | 0.07465708 | FALSE |
| SLC35B1 | 7197 | 2.199181 | 0.0278651 | 0.07477075 | FALSE |
| GLUD2 | 15621 | 2.198893 | 0.0278855 | 0.07481286 | FALSE |
| C14orf132 | 6437 | 2.198236 | 0.0279323 | 0.07489975 | FALSE |
| MED16 | 11774 | 2.198087 | 0.0279429 | 0.07491536 | FALSE |
| SNRPB2 | 2748 | 2.197599 | 0.0279777 | 0.07498291 | FALSE |
| ADORA2B | 793 | 2.197403 | 0.0279917 | 0.07500752 | FALSE |
| COP1 | 11431 | 2.196849 | 0.0280312 | 0.07507104 | FALSE |
| TAF9B | 2808 | 2.19672 | 0.0280405 | 0.07507387 | FALSE |
| BDNF | 13733 | 2.19622 | 0.0280762 | 0.07511772 | FALSE |
| ATP6V0CP3 | 5550 | 2.196155 | 0.0280809 | 0.07511772 | FALSE |
| LOC100130331 | 6396 | 2.195932 | 0.0280968 | 0.0751347 | FALSE |
| KLK15 | 14661 | 2.195055 | 0.0281597 | 0.07525183 | FALSE |
| SAMD4A | 4779 | 2.194348 | 0.0282104 | 0.07536111 | FALSE |
| ZBED6CL | 9674 | 2.191165 | 0.0284399 | 0.07590921 | FALSE |
| KIF22 | 11371 | 2.189894 | 0.0285319 | 0.07609 | FALSE |
| ZNF26 | 14763 | 2.189764 | 0.0285414 | 0.07610217 | FALSE |
| CGAS | 4954 | 2.189155 | 0.0285856 | 0.07620708 | FALSE |
| CACNB3 | 5599 | 2.188749 | 0.0286151 | 0.07627275 | FALSE |
| SYTL2 | 378 | 2.18846 | 0.0286361 | 0.07631578 | FALSE |
| PSMA2 | 11315 | 2.187824 | 0.0286824 | 0.07638711 | FALSE |
| COLEC11 | 11541 | 2.18706 | 0.0287381 | 0.07650944 | FALSE |
| HIST2H2BE | 12545 | 2.186182 | 0.0288023 | 0.07663424 | FALSE |
| DKFZP586I1420 | 12649 | 2.186083 | 0.0288095 | 0.07663424 | FALSE |
| WDR17 | 10233 | 2.185998 | 0.0288158 | 0.07663774 | FALSE |
| UTP11 | 2458 | 2.185241 | 0.0288712 | 0.07674327 | FALSE |
| ARHGAP22 | 6094 | 2.185188 | 0.0288751 | 0.07674327 | FALSE |
| RPL15 | 2691 | 2.183829 | 0.0289748 | 0.07692991 | FALSE |
| ANXA2 | 2754 | 2.183684 | 0.0289855 | 0.07694514 | FALSE |
| OCRL | 11428 | 2.181638 | 0.0291363 | 0.07730603 | FALSE |
| TRH | 14276 | 2.181569 | 0.0291414 | 0.07730644 | FALSE |
| PTBP3 | 9853 | 2.181312 | 0.0291603 | 0.07734369 | FALSE |
| KCNB2 | 5930 | 2.181123 | 0.0291743 | 0.0773545 | FALSE |
| ASB5 | 2386 | 2.179763 | 0.029275 | 0.07758934 | FALSE |
| GSTM1 | 6558 | 2.179653 | 0.0292832 | 0.07759054 | FALSE |
| KCNJ16 | 14198 | 2.178273 | 0.0293857 | 0.07780944 | FALSE |
| SMOC1 | 4563 | 2.177407 | 0.0294502 | 0.07795381 | FALSE |
| CRNKL1 | 5039 | 2.176706 | 0.0295025 | 0.0780658 | FALSE |
| SLC5A12 | 7958 | 2.176627 | 0.0295084 | 0.07806819 | FALSE |
| PHF10 | 8534 | 2.175816 | 0.029569 | 0.07820288 | FALSE |
| AHNAK2 | 11825 | 2.174979 | 0.0296317 | 0.07835457 | FALSE |
| PLPPR5 | 11484 | 2.174675 | 0.0296545 | 0.07838831 | FALSE |
| JADE2 | 14906 | 2.17447 | 0.0296699 | 0.0784157 | FALSE |
| SMO | 10230 | 2.174286 | 0.0296837 | 0.07843893 | FALSE |
| DENR | 3699 | 2.173797 | 0.0297204 | 0.07849616 | FALSE |
| S100A11 | 14747 | 2.173677 | 0.0297294 | 0.07850671 | FALSE |
| PLXNA1 | 14745 | 2.173502 | 0.0297426 | 0.07852818 | FALSE |
| PRPS1 | 7449 | 2.17188 | 0.0298647 | 0.07878419 | FALSE |
| MRPL18 | 1687 | 2.171654 | 0.0298818 | 0.07881588 | FALSE |
| FAAP24 | 2420 | 2.171532 | 0.029891 | 0.07882687 | FALSE |
| RNASE6 | 5046 | 2.171212 | 0.0299152 | 0.07887731 | FALSE |
| KCNMB2 | 2119 | 2.169725 | 0.0300277 | 0.07914733 | FALSE |
| DOCK9-AS2 | 4694 | 2.169173 | 0.0300696 | 0.07921761 | FALSE |
| NRL | 268 | 2.168654 | 0.030109 | 0.079268 | FALSE |
| CUL4A | 9359 | 2.167476 | 0.0301986 | 0.07947717 | FALSE |
| SUFU | 13231 | 2.166169 | 0.0302983 | 0.07964569 | FALSE |
| DCP2 | 3244 | 2.163529 | 0.0305005 | 0.0801369 | FALSE |
| SIGLEC14 | 2964 | 2.162517 | 0.0305783 | 0.08030091 | FALSE |
| PHLDB3 | 2036 | 2.161938 | 0.030623 | 0.08036587 | FALSE |
| LRP3 | 12969 | 2.161419 | 0.030663 | 0.08042861 | FALSE |
| NT5C3A | 7360 | 2.161171 | 0.0306821 | 0.08046532 | FALSE |
| ZIC1 | 10634 | 2.159644 | 0.0308002 | 0.08074797 | FALSE |
| ATP5PB | 4844 | 2.15926 | 0.03083 | 0.08076851 | FALSE |
| SLC25A26 | 15592 | 2.15924 | 0.0308316 | 0.08076851 | FALSE |
| MIR99AHG | 5685 | 2.158733 | 0.0308709 | 0.08083829 | FALSE |
| RPS27A | 7060 | 2.156971 | 0.0310079 | 0.08111561 | FALSE |
| DARS | 6680 | 2.156296 | 0.0310606 | 0.08120426 | FALSE |
| CHCHD1 | 11396 | 2.155893 | 0.031092 | 0.08126761 | FALSE |
| PLIN5 | 7302 | 2.155706 | 0.0311066 | 0.0812922 | FALSE |
| TMED3 | 12288 | 2.155508 | 0.0311221 | 0.08131904 | FALSE |
| TPM3P9 | 12723 | 2.1549 | 0.0311697 | 0.08141609 | FALSE |
| RHOA | 15037 | 2.154339 | 0.0312136 | 0.0814897 | FALSE |
| ARMCX2 | 7545 | 2.154274 | 0.0312187 | 0.0814897 | FALSE |
| FAS | 75 | 2.15405 | 0.0312363 | 0.08150832 | FALSE |
| ILF2 | 10227 | 2.153981 | 0.0312417 | 0.08150883 | FALSE |
| CIDEB | 12930 | 2.152273 | 0.0313759 | 0.0817823 | FALSE |
| MED8 | 7149 | 2.152244 | 0.0313781 | 0.0817823 | FALSE |
| RDH11 | 3555 | 2.151835 | 0.0314104 | 0.08183967 | FALSE |
| ILF3-AS1 | 3353 | 2.151696 | 0.0314213 | 0.08185457 | FALSE |
| ITGB4 | 6301 | 2.150339 | 0.0315284 | 0.08207767 | FALSE |
| GPR17 | 6229 | 2.150316 | 0.0315302 | 0.08207767 | FALSE |
| MYRFL | 8548 | 2.150273 | 0.0315336 | 0.08207767 | FALSE |
| ORC3 | 4344 | 2.150212 | 0.0315384 | 0.08207767 | FALSE |
| NMU | 10491 | 2.150212 | 0.0315384 | 0.08207767 | FALSE |
| BTF3P11 | 14177 | 2.149993 | 0.0315558 | 0.08210908 | FALSE |
| CHDH | 3271 | 2.147141 | 0.0317821 | 0.08261538 | FALSE |
| PIK3CD | 15417 | 2.145756 | 0.0318925 | 0.08288856 | FALSE |
| TCERG1L | 4265 | 2.145595 | 0.0319053 | 0.08290818 | FALSE |
| COMMD6 | 6050 | 2.145161 | 0.03194 | 0.08298449 | FALSE |
| LDHC | 4857 | 2.144388 | 0.0320018 | 0.08309676 | FALSE |
| ACADL | 5568 | 2.144355 | 0.0320045 | 0.08309676 | FALSE |
| LSAMP | 4835 | 2.143584 | 0.0320662 | 0.08322861 | FALSE |
| SYNPO | 907 | 2.142234 | 0.0321747 | 0.08345552 | FALSE |
| SCMH1 | 4545 | 2.141685 | 0.0322188 | 0.08355626 | FALSE |
| SMKR1 | 11349 | 2.141391 | 0.0322425 | 0.0836038 | FALSE |
| PIPSL | 9458 | 2.141075 | 0.032268 | 0.08365597 | FALSE |
| S1PR1 | 3342 | 2.141004 | 0.0322737 | 0.08365694 | FALSE |
| CCDC103 | 14766 | 2.140668 | 0.0323008 | 0.08371332 | FALSE |
| C3orf70 | 1673 | 2.140203 | 0.0323384 | 0.08379673 | FALSE |
| CNPY2 | 573 | 2.139534 | 0.0323924 | 0.08389682 | FALSE |
| DCAF5 | 11762 | 2.139154 | 0.0324232 | 0.08395746 | FALSE |
| SIDT1 | 7056 | 2.136735 | 0.0326196 | 0.08438548 | FALSE |
| LAMA1 | 5637 | 2.135543 | 0.0327167 | 0.08462275 | FALSE |
| NPTX1 | 10541 | 2.133654 | 0.0328711 | 0.08493787 | FALSE |
| TUBA3C | 11056 | 2.133469 | 0.0328863 | 0.084963 | FALSE |
| YDJC | 11926 | 2.131156 | 0.0330763 | 0.0854081 | FALSE |
| CHPT1 | 1448 | 2.131106 | 0.0330804 | 0.0854081 | FALSE |
| STX2 | 1804 | 2.13064 | 0.0331188 | 0.0854649 | FALSE |
| CRB1 | 15152 | 2.129911 | 0.033179 | 0.08560599 | FALSE |
| DGCR6 | 751 | 2.129679 | 0.0331981 | 0.0856413 | FALSE |
| TEDC1 | 9854 | 2.128477 | 0.0332976 | 0.0858553 | FALSE |
| SOX4 | 2116 | 2.128034 | 0.0333343 | 0.08592161 | FALSE |
| LTC4S | 13519 | 2.127405 | 0.0333864 | 0.08602775 | FALSE |
| CYBRD1 | 1176 | 2.125461 | 0.0335482 | 0.08633061 | FALSE |
| NALT1 | 4952 | 2.125102 | 0.0335781 | 0.08637921 | FALSE |
| FAM177A1 | 277 | 2.124971 | 0.033589 | 0.08639311 | FALSE |
| SQOR | 14564 | 2.12464 | 0.0336167 | 0.08644996 | FALSE |
| CDHR1 | 9462 | 2.124096 | 0.0336621 | 0.08652458 | FALSE |
| PHGDH | 8828 | 2.122636 | 0.0337844 | 0.08680983 | FALSE |
| KLHL25 | 794 | 2.122444 | 0.0338005 | 0.08683694 | FALSE |
| GSX1 | 8139 | 2.120811 | 0.0339377 | 0.08713224 | FALSE |
| MCFD2 | 14890 | 2.120425 | 0.0339702 | 0.08718815 | FALSE |
| MPL | 13479 | 2.11822 | 0.0341564 | 0.08762599 | FALSE |
| RASGRP4 | 9559 | 2.118201 | 0.034158 | 0.08762599 | FALSE |
| ITM2B | 13171 | 2.117155 | 0.0342467 | 0.08782457 | FALSE |
| ZNF622 | 9547 | 2.116699 | 0.0342854 | 0.08790941 | FALSE |
| EHBP1L1 | 14623 | 2.116317 | 0.0343179 | 0.0879782 | FALSE |
| PTP4A1 | 13112 | 2.115279 | 0.0344062 | 0.08818421 | FALSE |
| CRYZL1 | 14863 | 2.11524 | 0.0344095 | 0.08818421 | FALSE |
| TLR4 | 8700 | 2.1143 | 0.0344897 | 0.08833171 | FALSE |
| SSR4P1 | 2635 | 2.112924 | 0.0346073 | 0.08858941 | FALSE |
| RPS17 | 6426 | 2.112781 | 0.0346195 | 0.08860624 | FALSE |
| ZNF649 | 9439 | 2.111852 | 0.0346991 | 0.08879551 | FALSE |
| CHP2 | 9322 | 2.111618 | 0.0347192 | 0.08883235 | FALSE |
| RRP36 | 13115 | 2.110741 | 0.0347946 | 0.08898696 | FALSE |
| PA2G4 | 11 | 2.110716 | 0.0347967 | 0.08898696 | FALSE |
| SNHG16 | 5076 | 2.109756 | 0.0348794 | 0.08918373 | FALSE |
| MARK1 | 5090 | 2.109685 | 0.0348855 | 0.08918479 | FALSE |
| COL5A3 | 1765 | 2.109158 | 0.0349309 | 0.08928638 | FALSE |
| KCTD2 | 407 | 2.108944 | 0.0349494 | 0.08931898 | FALSE |
| WSB2 | 7200 | 2.108738 | 0.0349672 | 0.08934983 | FALSE |
| RXYLT1 | 5865 | 2.107071 | 0.0351114 | 0.08968904 | FALSE |
| ZC2HC1A | 12396 | 2.106067 | 0.0351985 | 0.08985592 | FALSE |
| TP53I3 | 5903 | 2.106053 | 0.0351997 | 0.08985592 | FALSE |
| LGI4 | 455 | 2.105491 | 0.0352486 | 0.08993876 | FALSE |
| LINGO1 | 937 | 2.105481 | 0.0352495 | 0.08993876 | FALSE |
| TSPAN17 | 7118 | 2.105121 | 0.0352808 | 0.08998929 | FALSE |
| TMEM220 | 2044 | 2.104876 | 0.0353021 | 0.09001432 | FALSE |
| CINP | 5857 | 2.104799 | 0.0353088 | 0.09001673 | FALSE |
| PJA1 | 12305 | 2.104568 | 0.0353289 | 0.09005334 | FALSE |
| SPSB4 | 2105 | 2.104481 | 0.0353365 | 0.09005798 | FALSE |
| AHNAK | 12731 | 2.104168 | 0.0353638 | 0.09008346 | FALSE |
| DRAXIN | 14207 | 2.103469 | 0.0354248 | 0.09020944 | FALSE |
| TUBB4B | 12489 | 2.103257 | 0.0354433 | 0.0902419 | FALSE |
| C12orf76 | 5313 | 2.103105 | 0.0354566 | 0.09026102 | FALSE |
| MRVI1 | 8724 | 2.102786 | 0.0354845 | 0.0903173 | FALSE |
| METTL27 | 3119 | 2.102508 | 0.0355088 | 0.09036449 | FALSE |
| PPP1R26 | 1539 | 2.102268 | 0.0355298 | 0.09040323 | FALSE |
| C11orf95 | 5168 | 2.102107 | 0.0355439 | 0.09042439 | FALSE |
| RACK1 | 8924 | 2.101743 | 0.0355758 | 0.09044664 | FALSE |
| CNTN1 | 6390 | 2.101082 | 0.0356338 | 0.09053514 | FALSE |
| LINC00595 | 2526 | 2.1009 | 0.0356497 | 0.09055078 | FALSE |
| LGR4 | 10362 | 2.100748 | 0.0356631 | 0.09055078 | FALSE |
| C1orf158 | 628 | 2.100748 | 0.0356631 | 0.09055078 | FALSE |
| CLPSL1 | 457 | 2.100515 | 0.0356836 | 0.09058604 | FALSE |
| RBM3 | 3090 | 2.099802 | 0.0357463 | 0.0906814 | FALSE |
| HILS1 | 1738 | 2.099796 | 0.0357468 | 0.0906814 | FALSE |
| MRPL36 | 12261 | 2.099015 | 0.0358156 | 0.09081994 | FALSE |
| DUSP14 | 11088 | 2.098782 | 0.0358361 | 0.09082965 | FALSE |
| CYB5R3 | 3604 | 2.098708 | 0.0358426 | 0.09082965 | FALSE |
| MTMR9LP | 1072 | 2.098621 | 0.0358503 | 0.09083438 | FALSE |
| SURF2 | 10088 | 2.097806 | 0.0359223 | 0.09094301 | FALSE |
| UBE2L5 | 280 | 2.097689 | 0.0359326 | 0.09095446 | FALSE |
| ZSWIM9 | 12449 | 2.097622 | 0.0359386 | 0.09095473 | FALSE |
| B9D2 | 13295 | 2.09748 | 0.0359511 | 0.09097178 | FALSE |
| FAM96B | 6251 | 2.09735 | 0.0359626 | 0.09098614 | FALSE |
| AQP5 | 4572 | 2.096053 | 0.0360775 | 0.09122538 | FALSE |
| TADA3 | 7260 | 2.096019 | 0.0360805 | 0.09122538 | FALSE |
| MORN2 | 10226 | 2.094753 | 0.036193 | 0.09143576 | FALSE |
| ATF5 | 11805 | 2.094685 | 0.036199 | 0.09143626 | FALSE |
| AKR1A1 | 3888 | 2.094594 | 0.0362071 | 0.09144194 | FALSE |
| DRICH1 | 4593 | 2.094077 | 0.0362531 | 0.09152859 | FALSE |
| PINLYP | 3052 | 2.093551 | 0.0363 | 0.09161735 | FALSE |
| NEK6 | 1035 | 2.092952 | 0.0363534 | 0.09173742 | FALSE |
| SAMD11 | 10498 | 2.092626 | 0.0363826 | 0.09179607 | FALSE |
| YWHAG | 11122 | 2.092411 | 0.0364018 | 0.09182972 | FALSE |
| HIST1H2AB | 1838 | 2.092265 | 0.0364148 | 0.09183481 | FALSE |
| FAM213B | 1298 | 2.09179 | 0.0364573 | 0.09189569 | FALSE |
| CAP1 | 8871 | 2.091069 | 0.0365219 | 0.09202878 | FALSE |
| ZFP36 | 7732 | 2.090889 | 0.036538 | 0.0920546 | FALSE |
| GRASP | 11949 | 2.089881 | 0.0366285 | 0.09223795 | FALSE |
| ANHX | 1338 | 2.089724 | 0.0366426 | 0.09225862 | FALSE |
| UGT2B10 | 1934 | 2.089235 | 0.0366866 | 0.09235228 | FALSE |
| MECR | 7179 | 2.087978 | 0.0367998 | 0.09260972 | FALSE |
| TP53BP2 | 11386 | 2.087749 | 0.0368205 | 0.09262267 | FALSE |
| XK | 1425 | 2.087312 | 0.0368599 | 0.09267151 | FALSE |
| NSD2 | 7799 | 2.084783 | 0.037089 | 0.09317249 | FALSE |
| HSD17B10 | 10312 | 2.082022 | 0.0373405 | 0.09366697 | FALSE |
| KLRG1 | 9847 | 2.081964 | 0.0373458 | 0.09366697 | FALSE |
| NRAV | 8649 | 2.080403 | 0.0374886 | 0.09397996 | FALSE |
| FAM189A1 | 6338 | 2.079814 | 0.0375426 | 0.09408519 | FALSE |
| TTC38 | 10947 | 2.079289 | 0.0375908 | 0.09417579 | FALSE |
| LRRC4C | 13954 | 2.079051 | 0.0376127 | 0.09420038 | FALSE |
| CD58 | 13481 | 2.077301 | 0.0377738 | 0.09454333 | FALSE |
| MYL3 | 8138 | 2.076953 | 0.0378059 | 0.09459341 | FALSE |
| FFAR4 | 13774 | 2.075566 | 0.0379341 | 0.09483831 | FALSE |
| HLA-L | 6309 | 2.075206 | 0.0379674 | 0.09490648 | FALSE |
| MBOAT2 | 8855 | 2.075107 | 0.0379766 | 0.09491423 | FALSE |
| HLA-DQB2 | 3033 | 2.073486 | 0.0381271 | 0.09521414 | FALSE |
| RUNX1T1 | 4131 | 2.073233 | 0.0381506 | 0.09524247 | FALSE |
| DYNLT1 | 2896 | 2.072921 | 0.0381796 | 0.09529381 | FALSE |
| MRM3 | 5192 | 2.072881 | 0.0381834 | 0.09529381 | FALSE |
| RAPSN | 12685 | 2.07038 | 0.0384168 | 0.09581516 | FALSE |
| ECEL1 | 5669 | 2.069355 | 0.0385128 | 0.09600809 | FALSE |
| LINC01116 | 3217 | 2.069292 | 0.0385187 | 0.09600809 | FALSE |
| RIBC1 | 2840 | 2.068577 | 0.0385858 | 0.09614469 | FALSE |
| CCL2 | 12938 | 2.068464 | 0.0385964 | 0.09615581 | FALSE |
| BTBD1 | 11096 | 2.067731 | 0.0386653 | 0.09631215 | FALSE |
| GRIK1 | 14948 | 2.067252 | 0.0387104 | 0.09639374 | FALSE |
| ACAT1 | 5814 | 2.066232 | 0.0388066 | 0.0965563 | FALSE |
| PIGP | 11720 | 2.065404 | 0.0388848 | 0.09673551 | FALSE |
| CITED4 | 6933 | 2.065242 | 0.0389001 | 0.09674282 | FALSE |
| PARVA | 11354 | 2.064848 | 0.0389374 | 0.09680473 | FALSE |
| FUT9 | 1821 | 2.064337 | 0.0389858 | 0.09690962 | FALSE |
| HHIPL1 | 10305 | 2.063453 | 0.0390696 | 0.09707666 | FALSE |
| WDR92 | 5332 | 2.062964 | 0.039116 | 0.09716376 | FALSE |
| CCT3 | 13113 | 2.061812 | 0.0392256 | 0.09738199 | FALSE |
| PRELP | 1052 | 2.061525 | 0.039253 | 0.0974344 | FALSE |
| DCAF4 | 2976 | 2.060963 | 0.0393066 | 0.09755193 | FALSE |
| BABAM2 | 9741 | 2.060616 | 0.0393397 | 0.09761863 | FALSE |
| CNTNAP3 | 1156 | 2.059295 | 0.039466 | 0.09788542 | FALSE |
| H2BFS | 15123 | 2.058261 | 0.0395651 | 0.09810008 | FALSE |
| AQP1 | 7784 | 2.058055 | 0.0395849 | 0.09813177 | FALSE |
| SLPI | 11974 | 2.057674 | 0.0396214 | 0.09819308 | FALSE |
| LOC285097 | 4068 | 2.057434 | 0.0396445 | 0.09823465 | FALSE |
| MSN | 864 | 2.057309 | 0.0396565 | 0.09824885 | FALSE |
| STRAP | 9126 | 2.055772 | 0.0398045 | 0.09859986 | FALSE |
| NDUFV3 | 3108 | 2.054606 | 0.0399171 | 0.0988161 | FALSE |
| COA4 | 4436 | 2.053928 | 0.0399827 | 0.09894712 | FALSE |
| FBLN2 | 2110 | 2.053554 | 0.0400189 | 0.09902107 | FALSE |
| SDF2L1 | 11882 | 2.053451 | 0.0400289 | 0.09903009 | FALSE |
| CPNE2 | 13618 | 2.05321 | 0.0400522 | 0.0990722 | FALSE |
| POLR2J4 | 1503 | 2.052949 | 0.0400775 | 0.09911912 | FALSE |
| MBD5 | 12794 | 2.052458 | 0.0401252 | 0.09920557 | FALSE |
| CLUL1 | 7045 | 2.051788 | 0.0401903 | 0.0993194 | FALSE |
| LPCAT2 | 11311 | 2.05095 | 0.0402718 | 0.09950519 | FALSE |
| RASA4CP | 11320 | 2.050513 | 0.0403144 | 0.09959465 | FALSE |
| SPA17 | 5704 | 2.050379 | 0.0403275 | 0.09961119 | FALSE |
| SH3GLB2 | 2857 | 2.049701 | 0.0403936 | 0.09974308 | FALSE |
| ABHD17C | 9445 | 2.048217 | 0.0405387 | 0.10006982 | FALSE |
| NACA | 5248 | 2.048106 | 0.0405496 | 0.10008085 | FALSE |
| LINC00320 | 13864 | 2.047683 | 0.0405911 | 0.10016655 | FALSE |
| KCNT2 | 2088 | 2.047621 | 0.0405972 | 0.10016655 | FALSE |
| DEDD2 | 2341 | 2.046139 | 0.0407427 | 0.1005098 | FALSE |
| PUM3 | 8449 | 2.045691 | 0.0407868 | 0.10058681 | FALSE |
| C11orf49 | 4836 | 2.045476 | 0.040808 | 0.10062314 | FALSE |
| RABGAP1 | 8627 | 2.045146 | 0.0408405 | 0.10067269 | FALSE |
| DERL1 | 4996 | 2.044938 | 0.040861 | 0.10069035 | FALSE |
| GDE1 | 13243 | 2.044422 | 0.0409119 | 0.10078401 | FALSE |
| EIF3J-AS1 | 2891 | 2.043814 | 0.0409719 | 0.10090014 | FALSE |
| LACTB | 12401 | 2.043007 | 0.0410517 | 0.10106487 | FALSE |
| HSP90AB1 | 12865 | 2.042367 | 0.0411151 | 0.10118907 | FALSE |
| CXCL16 | 8029 | 2.042163 | 0.0411354 | 0.10122291 | FALSE |
| GAL | 3742 | 2.041328 | 0.0412182 | 0.10139491 | FALSE |
| CPZ | 15031 | 2.040704 | 0.0412803 | 0.10153151 | FALSE |
| FAM50B | 2060 | 2.038703 | 0.0414797 | 0.1019418 | FALSE |
| ZNF883 | 6701 | 2.038571 | 0.0414929 | 0.10195541 | FALSE |
| CDKN1A | 12574 | 2.038517 | 0.0414983 | 0.10195541 | FALSE |
| NOL7 | 12313 | 2.037414 | 0.0416086 | 0.10217075 | FALSE |
| TTC9C | 7649 | 2.037336 | 0.0416164 | 0.10217075 | FALSE |
| TBC1D13 | 2853 | 2.037314 | 0.0416186 | 0.10217075 | FALSE |
| WDR78 | 3878 | 2.037232 | 0.0416268 | 0.10217487 | FALSE |
| NQO1 | 10680 | 2.036546 | 0.0416956 | 0.10229546 | FALSE |
| SEC11A | 2015 | 2.036416 | 0.0417086 | 0.1023114 | FALSE |
| BTG3 | 7566 | 2.035978 | 0.0417526 | 0.10240318 | FALSE |
| WDR82 | 9671 | 2.035414 | 0.0418092 | 0.10252608 | FALSE |
| USP5 | 9392 | 2.035197 | 0.0418311 | 0.10255969 | FALSE |
| NPHS1 | 5272 | 2.035102 | 0.0418406 | 0.10255969 | FALSE |
| LPP | 4322 | 2.03474 | 0.041877 | 0.10261188 | FALSE |
| EN2 | 2725 | 2.032324 | 0.0421209 | 0.1031511 | FALSE |
| EPHA10 | 12147 | 2.029589 | 0.0423983 | 0.1037916 | FALSE |
| MAP3K19 | 1415 | 2.029008 | 0.0424575 | 0.10392011 | FALSE |
| ANXA2P1 | 3485 | 2.02587 | 0.0427781 | 0.10459027 | FALSE |
| KLRC1 | 8236 | 2.025237 | 0.042843 | 0.10473263 | FALSE |
| ASB6 | 8401 | 2.02336 | 0.043036 | 0.10513869 | FALSE |
| CRSP8P | 9885 | 2.022775 | 0.0430964 | 0.10526957 | FALSE |
| FAM151B | 13783 | 2.022549 | 0.0431197 | 0.10527736 | FALSE |
| MAD2L2 | 11934 | 2.022541 | 0.0431205 | 0.10527736 | FALSE |
| JAG2 | 1913 | 2.022519 | 0.0431228 | 0.10527736 | FALSE |
| ARHGAP31 | 14546 | 2.022483 | 0.0431265 | 0.10527736 | FALSE |
| NCAM1 | 4148 | 2.022416 | 0.0431334 | 0.10527781 | FALSE |
| RITA1 | 11647 | 2.022083 | 0.0431678 | 0.10533086 | FALSE |
| ZMAT2 | 10452 | 2.021975 | 0.0431789 | 0.10533962 | FALSE |
| CALM3 | 10490 | 2.021533 | 0.0432246 | 0.10543462 | FALSE |
| NMI | 9022 | 2.020863 | 0.0432939 | 0.10553786 | FALSE |
| IGSF1 | 14497 | 2.019981 | 0.0433854 | 0.10571124 | FALSE |
| S100A13 | 7334 | 2.019683 | 0.0434163 | 0.10575362 | FALSE |
| RPL19P12 | 575 | 2.019514 | 0.0434338 | 0.10576465 | FALSE |
| SPATA4 | 1134 | 2.019213 | 0.0434651 | 0.10582303 | FALSE |
| OS9 | 1011 | 2.018796 | 0.0435084 | 0.10591206 | FALSE |
| LIPT2 | 2542 | 2.01871 | 0.0435174 | 0.10591734 | FALSE |
| FCER1G | 7309 | 2.01834 | 0.0435559 | 0.10594505 | FALSE |
| ATP5PF | 3050 | 2.018187 | 0.0435718 | 0.10595081 | FALSE |
| RRAGB | 3140 | 2.017553 | 0.0436378 | 0.10606268 | FALSE |
| DDX60 | 2988 | 2.01645 | 0.0437529 | 0.10630863 | FALSE |
| PNKP | 14236 | 2.015045 | 0.0438999 | 0.10663264 | FALSE |
| NANOS3 | 7221 | 2.014975 | 0.0439073 | 0.10663389 | FALSE |
| TSR1 | 5995 | 2.014473 | 0.0439599 | 0.10672854 | FALSE |
| DSEL | 6214 | 2.014265 | 0.0439817 | 0.10676494 | FALSE |
| ATP5MD | 3775 | 2.014001 | 0.0440094 | 0.10679904 | FALSE |
| ABCA3 | 6189 | 2.011108 | 0.0443141 | 0.10740489 | FALSE |
| TSHB | 4427 | 2.010375 | 0.0443915 | 0.10755929 | FALSE |
| UBXN11 | 14066 | 2.008989 | 0.0445383 | 0.10788151 | FALSE |
| TMEM117 | 11771 | 2.007546 | 0.0446916 | 0.10820244 | FALSE |
| GPBP1 | 13239 | 2.00607 | 0.0448488 | 0.10856289 | FALSE |
| TYRP1 | 12320 | 2.006018 | 0.0448543 | 0.10856289 | FALSE |
| MAPK8IP1 | 12693 | 2.005727 | 0.0448854 | 0.10862124 | FALSE |
| RGS19 | 1759 | 2.005488 | 0.0449109 | 0.10866617 | FALSE |
| TCP1 | 7601 | 2.004765 | 0.0449882 | 0.10881946 | FALSE |
| HOMER1 | 8146 | 2.004049 | 0.0450648 | 0.10896591 | FALSE |
| ERCC1 | 906 | 2.003893 | 0.0450815 | 0.1089778 | FALSE |
| FRMPD3 | 6890 | 2.003539 | 0.0451195 | 0.10905266 | FALSE |
| TSACC | 3866 | 2.00339 | 0.0451354 | 0.10907442 | FALSE |
| GTPBP4 | 4224 | 2.003102 | 0.0451663 | 0.10913221 | FALSE |
| HIST1H2BF | 1159 | 2.00225 | 0.0452579 | 0.10933642 | FALSE |
| CNOT7 | 2033 | 2.002062 | 0.0452781 | 0.10936835 | FALSE |
| ZNF593 | 14172 | 2.00165 | 0.0453224 | 0.1094585 | FALSE |
| COMMD7 | 8990 | 2.00123 | 0.0453676 | 0.10955079 | FALSE |
| LZTR1 | 9829 | 2.00099 | 0.0453935 | 0.10959631 | FALSE |
| GPD2 | 7598 | 2.000667 | 0.0454283 | 0.10966344 | FALSE |
| PDE1A | 4958 | 2.000276 | 0.0454705 | 0.10973137 | FALSE |
| UBE2K | 5219 | 2.000111 | 0.0454883 | 0.10975741 | FALSE |
| DHRS12 | 9753 | 1.999662 | 0.0455368 | 0.10984052 | FALSE |
| MATN2 | 6413 | 1.999034 | 0.0456047 | 0.10995341 | FALSE |
| ERAP2 | 5875 | 1.996928 | 0.045833 | 0.11045281 | FALSE |
| MYOM2 | 3723 | 1.994728 | 0.0460726 | 0.11096483 | FALSE |
| CBLN2 | 191 | 1.994716 | 0.0460739 | 0.11096483 | FALSE |
| RAE1 | 4385 | 1.994039 | 0.0461478 | 0.11110865 | FALSE |
| DNAJC19 | 14804 | 1.993733 | 0.0461812 | 0.11117206 | FALSE |
| ENSA | 14108 | 1.993606 | 0.0461951 | 0.11118837 | FALSE |
| PIGV | 13920 | 1.993483 | 0.0462086 | 0.11120363 | FALSE |
| HEMK1 | 9435 | 1.99315 | 0.046245 | 0.11126919 | FALSE |
| PSMD1 | 10132 | 1.993039 | 0.0462572 | 0.11126919 | FALSE |
| CRIM1 | 15579 | 1.991385 | 0.0464386 | 0.11156768 | FALSE |
| UAP1 | 7980 | 1.991322 | 0.0464455 | 0.11156768 | FALSE |
| TBC1D14 | 7445 | 1.991166 | 0.0464626 | 0.11157932 | FALSE |
| ACP1 | 12262 | 1.989935 | 0.0465981 | 0.11186549 | FALSE |
| CD44 | 9800 | 1.989809 | 0.046612 | 0.11188163 | FALSE |
| NTMT1 | 12405 | 1.989462 | 0.0466502 | 0.11190472 | FALSE |
| C9orf163 | 6905 | 1.98922 | 0.0466769 | 0.11193532 | FALSE |
| NUBPL | 14701 | 1.989058 | 0.0466948 | 0.11193532 | FALSE |
| EXOC1 | 1649 | 1.98899 | 0.0467023 | 0.11193532 | FALSE |
| TMED10 | 2333 | 1.986624 | 0.0469641 | 0.11248498 | FALSE |
| SCRG1 | 15584 | 1.986448 | 0.0469836 | 0.1125145 | FALSE |
| NBEA | 4446 | 1.986279 | 0.0470024 | 0.11252493 | FALSE |
| ADH5 | 4213 | 1.986154 | 0.0470162 | 0.11254091 | FALSE |
| NUS1 | 9403 | 1.985951 | 0.0470388 | 0.11256039 | FALSE |
| TIMP3 | 1904 | 1.985549 | 0.0470834 | 0.11265001 | FALSE |
| FAT1 | 6492 | 1.985419 | 0.0470979 | 0.11266734 | FALSE |
| MELTF | 10727 | 1.985337 | 0.047107 | 0.11267191 | FALSE |
| PCDH18 | 7734 | 1.98523 | 0.0471189 | 0.11268312 | FALSE |
| PAXIP1-AS1 | 5604 | 1.985027 | 0.0471415 | 0.11271988 | FALSE |
| GCDH | 2479 | 1.984779 | 0.0471691 | 0.11275138 | FALSE |
| POLR3C | 11019 | 1.984339 | 0.0472181 | 0.11284304 | FALSE |
| SLC7A7 | 8838 | 1.983805 | 0.0472776 | 0.11295896 | FALSE |
| RPS21 | 14263 | 1.983133 | 0.0473526 | 0.11310356 | FALSE |
| RPL11 | 3211 | 1.98275 | 0.0473954 | 0.11317118 | FALSE |
| LYPLAL1 | 10772 | 1.980272 | 0.047673 | 0.11378192 | FALSE |
| MAPK11 | 13661 | 1.979957 | 0.0477084 | 0.113849 | FALSE |
| SLC25A22 | 4641 | 1.979751 | 0.0477315 | 0.11387781 | FALSE |
| HCK | 12157 | 1.978885 | 0.047829 | 0.11403234 | FALSE |
| LILRA4 | 115 | 1.978472 | 0.0478755 | 0.11411008 | FALSE |
| GNG12 | 3711 | 1.978466 | 0.0478762 | 0.11411008 | FALSE |
| TSGA10 | 3198 | 1.978133 | 0.0479137 | 0.11418216 | FALSE |
| KLK12 | 13465 | 1.977657 | 0.0479674 | 0.11423196 | FALSE |
| POLM | 4299 | 1.975389 | 0.048224 | 0.11474674 | FALSE |
| SNCG | 13067 | 1.974399 | 0.0483364 | 0.11495144 | FALSE |
| SRR | 4028 | 1.974372 | 0.0483395 | 0.11495144 | FALSE |
| IFI16 | 2657 | 1.974253 | 0.048353 | 0.11496611 | FALSE |
| ARPC1A | 9828 | 1.973466 | 0.0484425 | 0.11516142 | FALSE |
| PPIH | 11346 | 1.972378 | 0.0485665 | 0.11536843 | FALSE |
| STX18 | 4980 | 1.971901 | 0.0486209 | 0.11544621 | FALSE |
| CCDC102A | 6375 | 1.971897 | 0.0486214 | 0.11544621 | FALSE |
| DDX20 | 4849 | 1.971635 | 0.0486513 | 0.11548217 | FALSE |
| LPIN2 | 10293 | 1.971361 | 0.0486826 | 0.11553895 | FALSE |
| PNMA1 | 7791 | 1.969548 | 0.0488902 | 0.11596123 | FALSE |
| CXADRP3 | 11301 | 1.968891 | 0.0489656 | 0.11610486 | FALSE |
| SLBP | 10698 | 1.967656 | 0.0491076 | 0.11641149 | FALSE |
| STX7 | 9433 | 1.96693 | 0.0491913 | 0.11656924 | FALSE |
| CXADRP2 | 10803 | 1.966213 | 0.049274 | 0.11671222 | FALSE |
| ZFAND2A | 15568 | 1.965893 | 0.049311 | 0.11678207 | FALSE |
| PRPF31 | 474 | 1.965707 | 0.0493325 | 0.11679758 | FALSE |
| RHOBTB3 | 7191 | 1.96503 | 0.0494108 | 0.11696525 | FALSE |
| RAB24 | 13018 | 1.964599 | 0.0494607 | 0.11706565 | FALSE |
| TPTE2P3 | 11267 | 1.961181 | 0.0498579 | 0.11788094 | FALSE |
| RGL3 | 11098 | 1.961023 | 0.0498763 | 0.11790667 | FALSE |
| GLG1 | 4360 | 1.960712 | 0.0499126 | 0.11795678 | FALSE |
| MRTO4 | 2181 | 1.959905 | 0.0500069 | 0.11814384 | FALSE |
| SLFN12 | 4235 | 1.958636 | 0.0501554 | 0.11844106 | FALSE |
| INTS4 | 10767 | 1.95816 | 0.0502112 | 0.11855495 | FALSE |
| PAF1 | 3112 | 1.957611 | 0.0502757 | 0.11865333 | FALSE |
| HMOX2 | 3955 | 1.956522 | 0.0504037 | 0.11888367 | FALSE |
| TTC19 | 2550 | 1.956015 | 0.0504634 | 0.11898856 | FALSE |
| PTCH1 | 14282 | 1.955438 | 0.0505314 | 0.11911298 | FALSE |
| B3GALT4 | 8023 | 1.955263 | 0.050552 | 0.11914367 | FALSE |
| RPL6 | 9116 | 1.95496 | 0.0505878 | 0.11920997 | FALSE |
| VCP | 5554 | 1.954854 | 0.0506003 | 0.11922149 | FALSE |
| DMWD | 9008 | 1.953048 | 0.0508139 | 0.11967066 | FALSE |
| TMEM181 | 9802 | 1.952569 | 0.0508707 | 0.11978633 | FALSE |
| GLI3 | 2743 | 1.951847 | 0.0509564 | 0.11995196 | FALSE |
| HK2 | 4190 | 1.950719 | 0.0510905 | 0.12021335 | FALSE |
| NDUFB11 | 5902 | 1.950039 | 0.0511715 | 0.12036768 | FALSE |
| PDIA3P1 | 11284 | 1.949838 | 0.0511954 | 0.12039482 | FALSE |
| ATP6V0B | 1988 | 1.949171 | 0.051275 | 0.12053867 | FALSE |
| MED14OS | 3093 | 1.946282 | 0.0516209 | 0.12126058 | FALSE |
| OR10G2 | 1985 | 1.945115 | 0.0517611 | 0.12151703 | FALSE |
| SOX9 | 13530 | 1.944138 | 0.0518788 | 0.12175671 | FALSE |
| FRRS1L | 4901 | 1.94284 | 0.0520355 | 0.12206946 | FALSE |
| ANKRD30BP2 | 7729 | 1.942723 | 0.0520496 | 0.12208431 | FALSE |
| ZNF219 | 649 | 1.94149 | 0.0521989 | 0.12238779 | FALSE |
| ATP6V1E1 | 7964 | 1.94146 | 0.0522025 | 0.12238779 | FALSE |
| OLFML2B | 4103 | 1.940811 | 0.0522812 | 0.12251718 | FALSE |
| WDR34 | 4279 | 1.940677 | 0.0522975 | 0.12253692 | FALSE |
| MBLAC2 | 12034 | 1.940488 | 0.0523204 | 0.12257044 | FALSE |
| RPL36AL | 110 | 1.940115 | 0.0523657 | 0.12264168 | FALSE |
| 2-Sep | 9071 | 1.939957 | 0.0523849 | 0.12266827 | FALSE |
| WIF1 | 5438 | 1.939519 | 0.0524382 | 0.12273783 | FALSE |
| ZNHIT1 | 6888 | 1.936707 | 0.0527812 | 0.12344824 | FALSE |
| INPP4A | 5253 | 1.936015 | 0.0528659 | 0.12360934 | FALSE |
| EPHX1 | 2801 | 1.935708 | 0.0529035 | 0.12367878 | FALSE |
| A1BG-AS1 | 3241 | 1.934535 | 0.0530474 | 0.1239596 | FALSE |
| GPC6 | 11856 | 1.933877 | 0.0531283 | 0.12412699 | FALSE |
| PAMR1 | 4490 | 1.933424 | 0.053184 | 0.12422311 | FALSE |
| PXDC1 | 14075 | 1.93321 | 0.0532103 | 0.12426608 | FALSE |
| HLCS | 2581 | 1.931735 | 0.0533922 | 0.12461755 | FALSE |
| BHMT2 | 10565 | 1.931538 | 0.0534166 | 0.12465458 | FALSE |
| EMC8 | 12126 | 1.931081 | 0.053473 | 0.12474914 | FALSE |
| ZNHIT3 | 15067 | 1.930259 | 0.0535748 | 0.12492314 | FALSE |
| FLVCR1-AS1 | 8064 | 1.930059 | 0.0535995 | 0.124951 | FALSE |
| C2orf40 | 9192 | 1.928181 | 0.0538326 | 0.12541957 | FALSE |
| TP53TG1 | 12102 | 1.927609 | 0.0539038 | 0.12552924 | FALSE |
| LOC101927811 | 8935 | 1.923746 | 0.0543864 | 0.12652129 | FALSE |
| GALR3 | 3647 | 1.923637 | 0.0544001 | 0.12652185 | FALSE |
| DCN | 9710 | 1.923256 | 0.0544479 | 0.12658896 | FALSE |
| SDHAF1 | 10208 | 1.921743 | 0.0546381 | 0.12701226 | FALSE |
| ENOPH1 | 15166 | 1.921569 | 0.05466 | 0.1270443 | FALSE |
| SCRN3 | 2 | 1.921119 | 0.0547167 | 0.12713827 | FALSE |
| ASMTL | 599 | 1.920394 | 0.0548082 | 0.1273129 | FALSE |
| SRI | 11444 | 1.920314 | 0.0548182 | 0.12731744 | FALSE |
| KIAA1191 | 2737 | 1.920247 | 0.0548267 | 0.12731817 | FALSE |
| PDIA3 | 5108 | 1.918467 | 0.0550518 | 0.12772709 | FALSE |
| RGS1 | 1394 | 1.915921 | 0.0553752 | 0.12835897 | FALSE |
| SDC3 | 5680 | 1.915136 | 0.0554752 | 0.12849951 | FALSE |
| SPAST | 11870 | 1.914942 | 0.0554999 | 0.12851653 | FALSE |
| HIST2H2AC | 5636 | 1.914885 | 0.0555072 | 0.12851653 | FALSE |
| SAT1 | 345 | 1.914747 | 0.0555248 | 0.12851922 | FALSE |
| ZNF202 | 8677 | 1.913693 | 0.0556594 | 0.12879267 | FALSE |
| MRPL3 | 2707 | 1.913185 | 0.0557244 | 0.1288929 | FALSE |
| ADAMTSL2 | 8020 | 1.913161 | 0.0557274 | 0.1288929 | FALSE |
| RAB39A | 10652 | 1.912474 | 0.0558154 | 0.12903912 | FALSE |
| FBLN5 | 8714 | 1.911257 | 0.0559716 | 0.12937905 | FALSE |
| TRIM17 | 883 | 1.911199 | 0.055979 | 0.12937905 | FALSE |
| DOCK6 | 14311 | 1.910538 | 0.056064 | 0.12949884 | FALSE |
| RAD18 | 6809 | 1.909508 | 0.0561966 | 0.12972718 | FALSE |
| HS3ST4 | 401 | 1.909448 | 0.0562043 | 0.12972718 | FALSE |
| LOC101927318 | 2746 | 1.90869 | 0.0563021 | 0.12991449 | FALSE |
| MORN3 | 1525 | 1.908126 | 0.0563749 | 0.12999527 | FALSE |
| TMEM218 | 11511 | 1.907912 | 0.0564026 | 0.13003122 | FALSE |
| ACVR2B | 3659 | 1.905593 | 0.056703 | 0.13055059 | FALSE |
| RIMKLB | 13267 | 1.905239 | 0.056749 | 0.13063592 | FALSE |
| HSPD1 | 161 | 1.903643 | 0.0569567 | 0.13094177 | FALSE |
| PALD1 | 4472 | 1.903008 | 0.0570395 | 0.1310869 | FALSE |
| KLC1 | 1755 | 1.901525 | 0.0572333 | 0.13150028 | FALSE |
| DDR1 | 9801 | 1.900877 | 0.0573181 | 0.13167586 | FALSE |
| LRSAM1 | 9155 | 1.900495 | 0.0573682 | 0.13175216 | FALSE |
| MGEA5 | 3038 | 1.900109 | 0.0574188 | 0.13181033 | FALSE |
| NDUFB1 | 627 | 1.898737 | 0.0575991 | 0.13214641 | FALSE |
| CD248 | 10724 | 1.898673 | 0.0576075 | 0.13214641 | FALSE |
| SERPINA1 | 2787 | 1.896905 | 0.0578405 | 0.13260305 | FALSE |
| TSPAN10 | 14857 | 1.896182 | 0.057936 | 0.13278305 | FALSE |
| CALU | 14451 | 1.895941 | 0.0579678 | 0.13279614 | FALSE |
| ENTPD6 | 11350 | 1.895665 | 0.0580044 | 0.13279614 | FALSE |
| GIMAP2 | 2004 | 1.895206 | 0.0580651 | 0.13290365 | FALSE |
| LYL1 | 852 | 1.894888 | 0.0581072 | 0.13296114 | FALSE |
| EID2B | 9289 | 1.894575 | 0.0581487 | 0.1330366 | FALSE |
| SLC41A3 | 5369 | 1.894134 | 0.0582072 | 0.13315095 | FALSE |
| ARHGAP42 | 6207 | 1.893013 | 0.0583561 | 0.13342866 | FALSE |
| RBM24 | 10917 | 1.892899 | 0.0583713 | 0.13342866 | FALSE |
| PSME1 | 8943 | 1.891912 | 0.0585027 | 0.13366912 | FALSE |
| CDC42SE2 | 12151 | 1.891852 | 0.0585107 | 0.13366912 | FALSE |
| EFCAB6 | 13542 | 1.891234 | 0.0585931 | 0.13383134 | FALSE |
| MDH2 | 5074 | 1.890904 | 0.0586372 | 0.13387088 | FALSE |
| FPGS | 6647 | 1.890554 | 0.0586839 | 0.13394397 | FALSE |
| EML3 | 4875 | 1.890303 | 0.0587174 | 0.13396525 | FALSE |
| CHST14 | 13834 | 1.889976 | 0.0587612 | 0.13404544 | FALSE |
| LOC145783 | 15013 | 1.889415 | 0.0588362 | 0.1341815 | FALSE |
| LRCOL1 | 6217 | 1.888255 | 0.0589917 | 0.13447326 | FALSE |
| KCTD17 | 14934 | 1.887644 | 0.0590738 | 0.13458177 | FALSE |
| LY6E | 8872 | 1.887223 | 0.0591303 | 0.13469104 | FALSE |
| ELFN2 | 790 | 1.88593 | 0.0593044 | 0.13501617 | FALSE |
| SESN3 | 3696 | 1.884555 | 0.0594899 | 0.13533269 | FALSE |
| FAM163A | 12156 | 1.8843 | 0.0595244 | 0.13536615 | FALSE |
| TMEM196 | 3015 | 1.88278 | 0.0597302 | 0.13571905 | FALSE |
| CYP4Z1 | 6806 | 1.8826 | 0.0597546 | 0.13571905 | FALSE |
| YAF2 | 8130 | 1.88249 | 0.0597695 | 0.13571905 | FALSE |
| 2-Mar | 7510 | 1.882467 | 0.0597726 | 0.13571905 | FALSE |
| CRB2 | 2158 | 1.882325 | 0.0597919 | 0.13574124 | FALSE |
| RMND5B | 6065 | 1.882243 | 0.059803 | 0.13574124 | FALSE |
| GEMIN8 | 11535 | 1.881807 | 0.0598622 | 0.13584354 | FALSE |
| MBNL3 | 13504 | 1.881431 | 0.0599133 | 0.13593974 | FALSE |
| WFDC3 | 9643 | 1.880433 | 0.0600491 | 0.13622804 | FALSE |
| SIPA1 | 1472 | 1.880164 | 0.0600857 | 0.13629138 | FALSE |
| MLLT11 | 5175 | 1.880092 | 0.0600955 | 0.13629386 | FALSE |
| FRG1 | 5911 | 1.879478 | 0.0601793 | 0.13644413 | FALSE |
| MPI | 1050 | 1.875774 | 0.0606863 | 0.13747417 | FALSE |
| C9orf131 | 6972 | 1.875686 | 0.0606984 | 0.13748163 | FALSE |
| CCDC106 | 5406 | 1.874479 | 0.0608644 | 0.13781774 | FALSE |
| CRIP1 | 1509 | 1.873758 | 0.0609638 | 0.13800274 | FALSE |
| PDCD2L | 389 | 1.8735 | 0.0609994 | 0.1380633 | FALSE |
| COX7C | 13011 | 1.87124 | 0.0613118 | 0.13870772 | FALSE |
| FAM220A | 6581 | 1.871184 | 0.0613196 | 0.13870772 | FALSE |
| TMEM128 | 532 | 1.869067 | 0.0616135 | 0.13921015 | FALSE |
| FAM19A1 | 13751 | 1.866291 | 0.0620007 | 0.13982348 | FALSE |
| SH2B3 | 13022 | 1.86586 | 0.062061 | 0.13991908 | FALSE |
| DDX49 | 14003 | 1.865652 | 0.0620901 | 0.13994436 | FALSE |
| RNF149 | 9068 | 1.864848 | 0.0622027 | 0.14011744 | FALSE |
| DPYSL2 | 10463 | 1.864056 | 0.0623138 | 0.14032732 | FALSE |
| ACTB | 4582 | 1.863009 | 0.062461 | 0.14061821 | FALSE |
| TWIST1 | 697 | 1.862797 | 0.0624908 | 0.14064961 | FALSE |
| SMIM10L2B-AS1 | 4274 | 1.862782 | 0.0624929 | 0.14064961 | FALSE |
| SEL1L3 | 3429 | 1.862323 | 0.0625576 | 0.14073429 | FALSE |
| ANP32E | 7242 | 1.862005 | 0.0626024 | 0.14077432 | FALSE |
| PARP1 | 11748 | 1.861429 | 0.0626836 | 0.14093672 | FALSE |
| MRPL57 | 83 | 1.861129 | 0.062726 | 0.14101164 | FALSE |
| TMEM9B | 1898 | 1.859226 | 0.0629951 | 0.1414562 | FALSE |
| KLHL2 | 8217 | 1.858805 | 0.0630548 | 0.14156763 | FALSE |
| GABRG1 | 1603 | 1.858676 | 0.0630731 | 0.14158837 | FALSE |
| STK10 | 2778 | 1.858438 | 0.0631068 | 0.14162604 | FALSE |
| TEX26 | 10576 | 1.857956 | 0.0631753 | 0.14167 | FALSE |
| CCDC86 | 8301 | 1.857954 | 0.0631755 | 0.14167 | FALSE |
| NDUFAF3 | 1617 | 1.857716 | 0.0632094 | 0.14171115 | FALSE |
| CHMP2B | 3496 | 1.857064 | 0.063302 | 0.14189389 | FALSE |
| ABI1 | 15038 | 1.856882 | 0.0633279 | 0.14191596 | FALSE |
| SEC13 | 10054 | 1.856574 | 0.0633718 | 0.14199386 | FALSE |
| DEAF1 | 1658 | 1.856056 | 0.0634456 | 0.14213882 | FALSE |
| UBC | 2008 | 1.855463 | 0.0635301 | 0.14228749 | FALSE |
| ADD3 | 4040 | 1.854608 | 0.0636522 | 0.14249968 | FALSE |
| SLC3A2 | 3427 | 1.85379 | 0.0637692 | 0.14274113 | FALSE |
| SCGB3A2 | 15005 | 1.853167 | 0.0638584 | 0.14289991 | FALSE |
| NEDD4 | 5876 | 1.852822 | 0.0639079 | 0.1429901 | FALSE |
| TRIM68 | 9775 | 1.851343 | 0.0641202 | 0.14337355 | FALSE |
| SLC29A4 | 12831 | 1.849919 | 0.0643252 | 0.1437783 | FALSE |
| TMEM74 | 3819 | 1.849281 | 0.0644172 | 0.14394437 | FALSE |
| RNF165 | 15293 | 1.849129 | 0.0644392 | 0.14397282 | FALSE |
| NME1 | 11744 | 1.847895 | 0.0646175 | 0.1442682 | FALSE |
| MRPL44 | 12487 | 1.846229 | 0.064859 | 0.14470389 | FALSE |
| CHMP6 | 3048 | 1.846093 | 0.0648787 | 0.14472728 | FALSE |
| HEXIM2 | 11294 | 1.845408 | 0.0649782 | 0.1449079 | FALSE |
| RNF157-AS1 | 8988 | 1.844973 | 0.0650415 | 0.14498692 | FALSE |
| ZMYND19 | 14695 | 1.844752 | 0.0650736 | 0.14503792 | FALSE |
| NGB | 6045 | 1.844344 | 0.065133 | 0.14512893 | FALSE |
| TRIM71 | 13771 | 1.844252 | 0.0651464 | 0.1451381 | FALSE |
| UFL1 | 13572 | 1.843935 | 0.0651926 | 0.14522031 | FALSE |
| SLC8A3 | 4899 | 1.843179 | 0.0653029 | 0.14542449 | FALSE |
| CREB3L1 | 14152 | 1.842669 | 0.0653774 | 0.14554887 | FALSE |
| RPS3 | 1407 | 1.841935 | 0.0654847 | 0.1457255 | FALSE |
| ZNF710-AS1 | 3934 | 1.840906 | 0.0656353 | 0.14599846 | FALSE |
| C6orf99 | 1715 | 1.839759 | 0.0658036 | 0.14633117 | FALSE |
| ZNF215 | 1980 | 1.838468 | 0.0659935 | 0.1467116 | FALSE |
| LINC01138 | 11724 | 1.836332 | 0.0663086 | 0.14728643 | FALSE |
| ZDHHC7 | 368 | 1.835586 | 0.0664189 | 0.14746866 | FALSE |
| SMPD1 | 3235 | 1.835447 | 0.0664395 | 0.14747968 | FALSE |
| P4HA2 | 2858 | 1.835103 | 0.0664904 | 0.14756458 | FALSE |
| MRPL24 | 8414 | 1.834968 | 0.0665104 | 0.14758802 | FALSE |
| TBC1D20 | 1167 | 1.834855 | 0.0665272 | 0.14759972 | FALSE |
| LY9 | 5324 | 1.834805 | 0.0665346 | 0.14759972 | FALSE |
| ZNF93 | 10157 | 1.834487 | 0.0665817 | 0.14768335 | FALSE |
| MOSPD2 | 12061 | 1.833708 | 0.0666974 | 0.14787685 | FALSE |
| MRPL43 | 2356 | 1.832525 | 0.0668732 | 0.14820377 | FALSE |
| PAXBP1-AS1 | 2697 | 1.831662 | 0.0670018 | 0.14844658 | FALSE |
| TDRKH | 5693 | 1.83051 | 0.0671737 | 0.1487853 | FALSE |
| ALG5 | 5268 | 1.830178 | 0.0672233 | 0.1488741 | FALSE |
| CKS1B | 132 | 1.830083 | 0.0672375 | 0.14888026 | FALSE |
| IL2RB | 4232 | 1.829605 | 0.067309 | 0.14897947 | FALSE |
| PTPA | 4833 | 1.829054 | 0.0673915 | 0.14909873 | FALSE |
| SEC61B | 3630 | 1.828949 | 0.0674072 | 0.1491026 | FALSE |
| TIGD6 | 11423 | 1.828915 | 0.0674123 | 0.1491026 | FALSE |
| DEFB124 | 9880 | 1.828488 | 0.0674763 | 0.14922305 | FALSE |
| NPPA | 5104 | 1.828389 | 0.0674912 | 0.14923477 | FALSE |
| RASGEF1C | 3733 | 1.828134 | 0.0675294 | 0.14927712 | FALSE |
| RIT1 | 3857 | 1.827728 | 0.0675904 | 0.1493644 | FALSE |
| MAGEE2 | 12258 | 1.82768 | 0.0675976 | 0.1493644 | FALSE |
| TRMT10C | 2375 | 1.827382 | 0.0676424 | 0.14941456 | FALSE |
| KIFAP3 | 14878 | 1.826197 | 0.0678206 | 0.14973017 | FALSE |
| RBM42 | 5158 | 1.825184 | 0.0679733 | 0.15000368 | FALSE |
| SLC22A18 | 14099 | 1.824373 | 0.0680957 | 0.15023146 | FALSE |
| DDT | 678 | 1.82406 | 0.068143 | 0.15027986 | FALSE |
| GAREM2 | 7251 | 1.823391 | 0.0682442 | 0.15047411 | FALSE |
| TMEM147 | 3736 | 1.822604 | 0.0683634 | 0.15069443 | FALSE |
| CFAP44 | 1171 | 1.822473 | 0.0683832 | 0.15071695 | FALSE |
| PRSS3 | 14849 | 1.822097 | 0.0684403 | 0.15082136 | FALSE |
| CHST11 | 7565 | 1.821996 | 0.0684556 | 0.15083387 | FALSE |
| NCBP2-AS2 | 14453 | 1.821636 | 0.0685102 | 0.15093299 | FALSE |
| TGFBR3L | 1766 | 1.821215 | 0.0685742 | 0.15104603 | FALSE |
| HSP90AA1 | 7497 | 1.820494 | 0.0686838 | 0.15123014 | FALSE |
| TMED1 | 353 | 1.820222 | 0.0687252 | 0.15129997 | FALSE |
| JUP | 15556 | 1.818988 | 0.0689133 | 0.15164972 | FALSE |
| ARRB1 | 1978 | 1.818925 | 0.0689229 | 0.15164972 | FALSE |
| DIRC1 | 10455 | 1.818117 | 0.0690463 | 0.15188079 | FALSE |
| TNIP3 | 4388 | 1.817922 | 0.0690761 | 0.15191066 | FALSE |
| CFAP20 | 1597 | 1.817183 | 0.0691891 | 0.15208565 | FALSE |
| DRG2 | 13626 | 1.816987 | 0.0692191 | 0.15213022 | FALSE |
| NDUFB2-AS1 | 410 | 1.816623 | 0.0692749 | 0.1522103 | FALSE |
| C11orf71 | 8904 | 1.816622 | 0.069275 | 0.1522103 | FALSE |
| SCN2A | 10116 | 1.816309 | 0.069323 | 0.15225151 | FALSE |
| ANKRD65 | 1054 | 1.814988 | 0.0695258 | 0.15261108 | FALSE |
| PRRG3 | 14117 | 1.814618 | 0.0695826 | 0.15271449 | FALSE |
| KLHL22 | 8798 | 1.814493 | 0.0696019 | 0.15273524 | FALSE |
| CKAP4 | 2345 | 1.814004 | 0.0696771 | 0.15285746 | FALSE |
| CHRNA6 | 11712 | 1.813391 | 0.0697715 | 0.15304314 | FALSE |
| TSNARE1 | 3855 | 1.812866 | 0.0698525 | 0.15319921 | FALSE |
| HGF | 8531 | 1.812358 | 0.0699309 | 0.15332815 | FALSE |
| GH2 | 4615 | 1.812289 | 0.0699416 | 0.15333001 | FALSE |
| IGFBP7 | 13351 | 1.811637 | 0.0700423 | 0.15350783 | FALSE |
| TRMT12 | 14086 | 1.811573 | 0.0700522 | 0.153508 | FALSE |
| ATF7 | 5935 | 1.810901 | 0.0701562 | 0.1537143 | FALSE |
| SLC30A3 | 7858 | 1.810741 | 0.070181 | 0.15372126 | FALSE |
| SPSB2 | 7072 | 1.810704 | 0.0701867 | 0.15372126 | FALSE |
| LOC100506125 | 14037 | 1.81069 | 0.0701889 | 0.15372126 | FALSE |
| OSCAR | 7170 | 1.81025 | 0.070257 | 0.15382747 | FALSE |
| HAUS7 | 3621 | 1.810058 | 0.0702868 | 0.15387109 | FALSE |
| PABPC1L | 12424 | 1.809637 | 0.0703521 | 0.15397738 | FALSE |
| LOC729732 | 6490 | 1.809618 | 0.070355 | 0.15397738 | FALSE |
| SNRPG | 9205 | 1.808982 | 0.0704538 | 0.15415035 | FALSE |
| SSTR4 | 14199 | 1.808837 | 0.0704763 | 0.15417807 | FALSE |
| FBXO41 | 7564 | 1.807637 | 0.070663 | 0.15447838 | FALSE |
| RNF139 | 4429 | 1.806857 | 0.0707846 | 0.15471348 | FALSE |
| HIST1H2BN | 11643 | 1.806428 | 0.0708515 | 0.15482551 | FALSE |
| ZDHHC23 | 534 | 1.80584 | 0.0709433 | 0.15498283 | FALSE |
| LINC01197 | 1464 | 1.805182 | 0.0710462 | 0.15518588 | FALSE |
| POLR1D | 14125 | 1.804229 | 0.0711954 | 0.1554328 | FALSE |
| MLF1 | 8659 | 1.803676 | 0.0712821 | 0.15554904 | FALSE |
| ACOT7 | 10864 | 1.803554 | 0.0713013 | 0.15556908 | FALSE |
| DDIT4 | 8974 | 1.803203 | 0.0713563 | 0.15564583 | FALSE |
| PDXP | 779 | 1.803059 | 0.0713789 | 0.15567343 | FALSE |
| LOC101926934 | 2612 | 1.802387 | 0.0714845 | 0.15588196 | FALSE |
| SLC27A6 | 7015 | 1.802262 | 0.0715042 | 0.15590308 | FALSE |
| CAPNS2 | 11348 | 1.80144 | 0.0716336 | 0.15609805 | FALSE |
| RPL4 | 11008 | 1.801314 | 0.0716534 | 0.15611953 | FALSE |
| SLC16A11 | 14486 | 1.801178 | 0.0716748 | 0.15614445 | FALSE |
| CALHM2 | 10538 | 1.800361 | 0.0718037 | 0.15638152 | FALSE |
| SZRD1 | 10643 | 1.799225 | 0.0719831 | 0.15661362 | FALSE |
| JAZF1 | 6840 | 1.799031 | 0.0720138 | 0.15662095 | FALSE |
| FGFR1 | 11975 | 1.797789 | 0.0722105 | 0.15702684 | FALSE |
| GGCX | 9755 | 1.797355 | 0.0722793 | 0.15715384 | FALSE |
| DBN1 | 439 | 1.796615 | 0.0723968 | 0.15730278 | FALSE |
| SYT4 | 11715 | 1.794579 | 0.0727208 | 0.15791697 | FALSE |
| IBA57 | 7002 | 1.793939 | 0.0728229 | 0.15809478 | FALSE |
| GALNT18 | 6675 | 1.792064 | 0.0731227 | 0.15861349 | FALSE |
| PITPNC1 | 948 | 1.791772 | 0.0731695 | 0.15869201 | FALSE |
| HLA-H | 3011 | 1.79169 | 0.0731826 | 0.15869201 | FALSE |
| KRT17P5 | 11424 | 1.791407 | 0.073228 | 0.15875377 | FALSE |
| CENPF | 12786 | 1.789945 | 0.0734628 | 0.15917439 | FALSE |
| TTYH3 | 12916 | 1.788807 | 0.0736459 | 0.15945074 | FALSE |
| SLC2A13 | 10442 | 1.788709 | 0.0736617 | 0.15945074 | FALSE |
| STAG3 | 12256 | 1.788633 | 0.0736739 | 0.15945517 | FALSE |
| MRPL41 | 9203 | 1.78818 | 0.073747 | 0.15956905 | FALSE |
| ACO2 | 7453 | 1.787842 | 0.0738015 | 0.15966495 | FALSE |
| MPP3 | 6756 | 1.787154 | 0.0739126 | 0.15988318 | FALSE |
| PAAF1 | 4776 | 1.78709 | 0.0739229 | 0.15988342 | FALSE |
| HES7 | 10275 | 1.786404 | 0.0740339 | 0.16005681 | FALSE |
| PLK2 | 7905 | 1.784982 | 0.0742642 | 0.16043655 | FALSE |
| DDX10 | 4303 | 1.78494 | 0.0742711 | 0.16043655 | FALSE |
| EEF1AKMT4 | 14089 | 1.784743 | 0.074303 | 0.16048342 | FALSE |
| WTAP | 13958 | 1.784275 | 0.074379 | 0.16058474 | FALSE |
| RPS7 | 1247 | 1.784229 | 0.0743865 | 0.16058474 | FALSE |
| ADAMTSL3 | 12784 | 1.784097 | 0.0744079 | 0.16059903 | FALSE |
| PHLPP2 | 3636 | 1.783768 | 0.0744614 | 0.16069224 | FALSE |
| DUSP18 | 14459 | 1.782861 | 0.0746089 | 0.16098847 | FALSE |
| TP53I13 | 12718 | 1.782628 | 0.0746469 | 0.16104206 | FALSE |
| VAMP7 | 1294 | 1.782455 | 0.0746751 | 0.16104225 | FALSE |
| GYG2 | 6961 | 1.782237 | 0.0747106 | 0.16109665 | FALSE |
| ZNF205-AS1 | 12733 | 1.78205 | 0.0747411 | 0.16114016 | FALSE |
| NCAPH2 | 12355 | 1.779941 | 0.0750856 | 0.16179372 | FALSE |
| CBR3 | 3606 | 1.77739 | 0.0755041 | 0.1626058 | FALSE |
| AAED1 | 3622 | 1.776899 | 0.0755849 | 0.16269009 | FALSE |
| KRTAP6-3 | 8376 | 1.775949 | 0.0757413 | 0.1629748 | FALSE |
| SLC15A3 | 2014 | 1.775906 | 0.0757484 | 0.1629748 | FALSE |
| TRIM35 | 5841 | 1.775472 | 0.07582 | 0.16310635 | FALSE |
| NEXMIF | 1620 | 1.774597 | 0.0759645 | 0.16337217 | FALSE |
| ST14 | 7332 | 1.774263 | 0.0760197 | 0.16346841 | FALSE |
| GUCY1B1 | 3835 | 1.772264 | 0.0763507 | 0.16409007 | FALSE |
| CHMP1B | 5116 | 1.772121 | 0.0763745 | 0.16411851 | FALSE |
| ZMYND12 | 3057 | 1.77188 | 0.0764145 | 0.16418191 | FALSE |
| B4GALT5 | 1555 | 1.770891 | 0.0765788 | 0.1644815 | FALSE |
| DHDDS | 3432 | 1.769938 | 0.0767375 | 0.16469479 | FALSE |
| MFSD1 | 10706 | 1.769825 | 0.0767563 | 0.16471259 | FALSE |
| COMMD5 | 14454 | 1.767723 | 0.0771072 | 0.16535216 | FALSE |
| RPLP0 | 1719 | 1.766178 | 0.077366 | 0.16581384 | FALSE |
| FAM122C | 3790 | 1.765731 | 0.077441 | 0.16590859 | FALSE |
| MPND | 14620 | 1.765394 | 0.0774976 | 0.16600706 | FALSE |
| ERBB2 | 14168 | 1.765043 | 0.0775565 | 0.16608786 | FALSE |
| FLAD1 | 6471 | 1.764426 | 0.0776603 | 0.16626448 | FALSE |
| RPS23 | 12227 | 1.76417 | 0.0777034 | 0.16633393 | FALSE |
| DPH2 | 12389 | 1.763676 | 0.0777865 | 0.16648919 | FALSE |
| TAL2 | 9992 | 1.763064 | 0.0778897 | 0.16664152 | FALSE |
| GINS3 | 2278 | 1.762882 | 0.0779204 | 0.16668439 | FALSE |
| YBX3 | 6176 | 1.762619 | 0.0779648 | 0.16673369 | FALSE |
| RAB31 | 8660 | 1.762107 | 0.0780512 | 0.166887 | FALSE |
| LINC00909 | 691 | 1.761703 | 0.0781195 | 0.16699199 | FALSE |
| AKAP5 | 11340 | 1.761509 | 0.0781523 | 0.16701427 | FALSE |
| ERC2 | 6848 | 1.761339 | 0.078181 | 0.16703625 | FALSE |
| RENBP | 11192 | 1.761239 | 0.078198 | 0.16704956 | FALSE |
| TMEM246 | 13177 | 1.761026 | 0.078234 | 0.1670809 | FALSE |
| PDCD6 | 10447 | 1.760695 | 0.07829 | 0.1671549 | FALSE |
| MRPL13 | 11089 | 1.759978 | 0.0784115 | 0.16739145 | FALSE |
| ACOT4 | 9109 | 1.759278 | 0.0785303 | 0.16762209 | FALSE |
| CELF3 | 2776 | 1.759082 | 0.0785636 | 0.16767024 | FALSE |
| MRPL2 | 11508 | 1.75868 | 0.0786319 | 0.16779308 | FALSE |
| SYPL2 | 440 | 1.758516 | 0.0786598 | 0.16781727 | FALSE |
| EFNB1 | 14735 | 1.758487 | 0.0786647 | 0.16781727 | FALSE |
| OR2A14 | 13694 | 1.758401 | 0.0786793 | 0.16782556 | FALSE |
| MFAP4 | 9594 | 1.756722 | 0.0789652 | 0.16834352 | FALSE |
| PPIAL4A | 6241 | 1.756086 | 0.0790737 | 0.16850589 | FALSE |
| CCDC112 | 11593 | 1.756023 | 0.0790845 | 0.16850589 | FALSE |
| HSPA13 | 11516 | 1.755755 | 0.0791303 | 0.16854978 | FALSE |
| CDC42BPB | 167 | 1.755499 | 0.079174 | 0.16856386 | FALSE |
| MLH1 | 3125 | 1.755485 | 0.0791764 | 0.16856386 | FALSE |
| FGF3 | 11737 | 1.753934 | 0.0794418 | 0.1690829 | FALSE |
| RETREG1 | 507 | 1.753379 | 0.079537 | 0.16926239 | FALSE |
| MRFAP1 | 8315 | 1.749513 | 0.0802024 | 0.17038794 | FALSE |
| SCAPER | 3188 | 1.748733 | 0.0803372 | 0.17059375 | FALSE |
| CDH22 | 1038 | 1.747436 | 0.0805617 | 0.17088489 | FALSE |
| ADI1 | 10850 | 1.746168 | 0.0807818 | 0.17128188 | FALSE |
| XRCC4 | 12196 | 1.74541 | 0.0809135 | 0.1714531 | FALSE |
| PCDH8 | 14999 | 1.745388 | 0.0809174 | 0.1714531 | FALSE |
| FAM86DP | 432 | 1.745273 | 0.0809374 | 0.17147225 | FALSE |
| AIG1 | 7069 | 1.745096 | 0.0809682 | 0.17151426 | FALSE |
| TMEM61 | 3170 | 1.74488 | 0.0810058 | 0.17157066 | FALSE |
| RPLP0P2 | 2374 | 1.744027 | 0.0811544 | 0.17183888 | FALSE |
| AVPR2 | 2200 | 1.743291 | 0.0812828 | 0.17201685 | FALSE |
| ETFB | 8277 | 1.742162 | 0.0814801 | 0.17236515 | FALSE |
| RORC | 2923 | 1.741866 | 0.0815319 | 0.1723951 | FALSE |
| NXN | 7500 | 1.741829 | 0.0815384 | 0.1723951 | FALSE |
| MBOAT7 | 6320 | 1.739188 | 0.0820017 | 0.17321072 | FALSE |
| DPY19L3 | 7663 | 1.73868 | 0.0820911 | 0.17336714 | FALSE |
| TRIP10 | 13611 | 1.737206 | 0.0823508 | 0.17380725 | FALSE |
| CSTF1 | 6810 | 1.736996 | 0.0823879 | 0.173862 | FALSE |
| PGAM2 | 920 | 1.736532 | 0.0824698 | 0.17398793 | FALSE |
| PNPLA6 | 4763 | 1.735972 | 0.0825688 | 0.17414973 | FALSE |
| ACAA2 | 7751 | 1.735369 | 0.0826755 | 0.1743277 | FALSE |
| TWISTNB | 9356 | 1.734177 | 0.0828867 | 0.17467882 | FALSE |
| IMP4 | 13578 | 1.733877 | 0.0829399 | 0.17474687 | FALSE |
| RAB29 | 6922 | 1.733492 | 0.0830083 | 0.17486432 | FALSE |
| WDR62 | 10269 | 1.733284 | 0.0830452 | 0.17490698 | FALSE |
| HLA-G | 13226 | 1.733252 | 0.0830509 | 0.17490698 | FALSE |
| HSD3B7 | 1245 | 1.733016 | 0.0830928 | 0.17497173 | FALSE |
| ZNF572 | 12783 | 1.732156 | 0.0832458 | 0.17516352 | FALSE |
| B4GALNT1 | 4915 | 1.732145 | 0.0832477 | 0.17516352 | FALSE |
| ATP2C1 | 9840 | 1.731534 | 0.0833566 | 0.17529099 | FALSE |
| SATL1 | 13012 | 1.731404 | 0.0833797 | 0.17531612 | FALSE |
| GRIK3 | 10229 | 1.731151 | 0.0834248 | 0.17536378 | FALSE |
| HIKESHI | 14194 | 1.73095 | 0.0834607 | 0.17541555 | FALSE |
| EPCAM | 15371 | 1.73081 | 0.0834857 | 0.17544078 | FALSE |
| MIR4435-2HG | 9817 | 1.730744 | 0.0834974 | 0.17544078 | FALSE |
| SLC25A42 | 2936 | 1.730346 | 0.0835685 | 0.17552411 | FALSE |
| SPPL2C | 27 | 1.72901 | 0.0838073 | 0.17595482 | FALSE |
| SMARCA1 | 2418 | 1.727963 | 0.0839949 | 0.17625391 | FALSE |
| BDKRB2 | 5355 | 1.727267 | 0.0841197 | 0.17644487 | FALSE |
| SELENOS | 4292 | 1.727091 | 0.0841513 | 0.17648747 | FALSE |
| PWP2 | 8196 | 1.726891 | 0.0841872 | 0.1765162 | FALSE |
| LCAT | 5868 | 1.726889 | 0.0841876 | 0.1765162 | FALSE |
| STRADB | 117 | 1.726757 | 0.0842113 | 0.17654224 | FALSE |
| ACTR3C | 1031 | 1.72644 | 0.0842683 | 0.17659515 | FALSE |
| C1orf52 | 3249 | 1.726112 | 0.0843273 | 0.17664319 | FALSE |
| WWTR1 | 2150 | 1.725711 | 0.0843994 | 0.17672329 | FALSE |
| ARX | 4858 | 1.724629 | 0.0845944 | 0.17703662 | FALSE |
| CNTNAP2 | 5358 | 1.724396 | 0.0846364 | 0.17710086 | FALSE |
| EXOC3-AS1 | 3132 | 1.72421 | 0.0846699 | 0.17712367 | FALSE |
| MEGF11 | 8914 | 1.723719 | 0.0847586 | 0.17724966 | FALSE |
| KIFC3 | 11902 | 1.723688 | 0.0847642 | 0.17724966 | FALSE |
| GGNBP1 | 15444 | 1.72351 | 0.0847963 | 0.17727996 | FALSE |
| SYT5 | 9778 | 1.723294 | 0.0848354 | 0.17727996 | FALSE |
| HSPH1 | 9261 | 1.722182 | 0.0850366 | 0.17765287 | FALSE |
| PI16 | 863 | 1.722049 | 0.0850606 | 0.17765572 | FALSE |
| UFM1 | 7086 | 1.721568 | 0.0851478 | 0.17779025 | FALSE |
| RUVBL2 | 14357 | 1.721261 | 0.0852035 | 0.17781263 | FALSE |
| LBX2-AS1 | 4742 | 1.72113 | 0.0852272 | 0.17783735 | FALSE |
| HIST2H2BC | 6832 | 1.720205 | 0.0853952 | 0.17814024 | FALSE |
| SPIN2B | 11558 | 1.717534 | 0.0858816 | 0.17898784 | FALSE |
| ZFAND3 | 12220 | 1.717118 | 0.0859576 | 0.17912229 | FALSE |
| GEM | 8830 | 1.716893 | 0.0859987 | 0.17918407 | FALSE |
| KCNJ12 | 14363 | 1.716046 | 0.0861536 | 0.17945298 | FALSE |
| SCAMP2 | 1135 | 1.715622 | 0.0862312 | 0.1795489 | FALSE |
| ZBTB48 | 15025 | 1.715074 | 0.0863317 | 0.1797101 | FALSE |
| TTC23 | 11296 | 1.714786 | 0.0863845 | 0.17977215 | FALSE |
| DNPH1 | 10388 | 1.714441 | 0.0864478 | 0.1798579 | FALSE |
| RAB18 | 10969 | 1.714205 | 0.0864911 | 0.1798743 | FALSE |
| NAT14 | 9265 | 1.713914 | 0.0865445 | 0.17996149 | FALSE |
| POLR3H | 1637 | 1.713632 | 0.0865963 | 0.18004528 | FALSE |
| ZNF667-AS1 | 11672 | 1.71259 | 0.086788 | 0.18039579 | FALSE |
| SNX2 | 6142 | 1.712362 | 0.08683 | 0.18045472 | FALSE |
| SNHG10 | 2454 | 1.712293 | 0.0868427 | 0.18045472 | FALSE |
| HAGH | 12823 | 1.712248 | 0.086851 | 0.18045472 | FALSE |
| TATDN2 | 10374 | 1.711563 | 0.0869772 | 0.18069303 | FALSE |
| COMT | 10433 | 1.711384 | 0.0870102 | 0.18073761 | FALSE |
| MIEN1 | 14265 | 1.710928 | 0.0870944 | 0.18088838 | FALSE |
| KIAA1147 | 6762 | 1.710589 | 0.087157 | 0.18089824 | FALSE |
| SMIM19 | 14049 | 1.710349 | 0.0872014 | 0.18096626 | FALSE |
| MRPL54 | 3587 | 1.710175 | 0.0872335 | 0.18098495 | FALSE |
| C10orf82 | 9605 | 1.709176 | 0.0874184 | 0.1813203 | FALSE |
| GPR152 | 2395 | 1.708623 | 0.0875208 | 0.18146058 | FALSE |
| RPL10L | 13074 | 1.708487 | 0.087546 | 0.18148878 | FALSE |
| CRCT1 | 2606 | 1.708139 | 0.0876106 | 0.18157995 | FALSE |
| COL8A1 | 12408 | 1.708068 | 0.0876237 | 0.18157995 | FALSE |
| S1PR2 | 10608 | 1.708062 | 0.0876248 | 0.18157995 | FALSE |
| RTN4IP1 | 9413 | 1.707954 | 0.0876449 | 0.1815974 | FALSE |
| CELF6 | 10162 | 1.707738 | 0.087685 | 0.18163539 | FALSE |
| PPP4C | 9771 | 1.70773 | 0.0876865 | 0.18163539 | FALSE |
| LRRIQ1 | 1260 | 1.707089 | 0.0878055 | 0.18180974 | FALSE |
| TRAPPC4 | 8710 | 1.706458 | 0.0879228 | 0.18197592 | FALSE |
| ORAI1 | 2622 | 1.70438 | 0.0883101 | 0.1826852 | FALSE |
| CD4 | 6910 | 1.704268 | 0.088331 | 0.18270429 | FALSE |
| LINC00461 | 5207 | 1.703577 | 0.0884601 | 0.18289874 | FALSE |
| CDC27 | 3497 | 1.703295 | 0.0885129 | 0.18297369 | FALSE |
| PRM2 | 11778 | 1.703258 | 0.0885198 | 0.18297369 | FALSE |
| EBLN3P | 5644 | 1.702737 | 0.0886173 | 0.18315101 | FALSE |
| ZNF846 | 14726 | 1.701935 | 0.0887676 | 0.1834373 | FALSE |
| WDR49 | 12199 | 1.700766 | 0.0889869 | 0.18380356 | FALSE |
| TMEM121 | 11614 | 1.70074 | 0.0889918 | 0.18380356 | FALSE |
| AHSG | 11542 | 1.699783 | 0.0891718 | 0.18404057 | FALSE |
| PODXL2 | 14567 | 1.699757 | 0.0891766 | 0.18404057 | FALSE |
| RUBCN | 10530 | 1.699754 | 0.0891772 | 0.18404057 | FALSE |
| IL4I1 | 7389 | 1.699581 | 0.0892098 | 0.18407586 | FALSE |
| PRELID1 | 1028 | 1.697251 | 0.0896492 | 0.18481949 | FALSE |
| EMILIN1 | 8358 | 1.696883 | 0.0897188 | 0.18491414 | FALSE |
| NAV1 | 6381 | 1.696883 | 0.0897188 | 0.18491414 | FALSE |
| TNKS2 | 15431 | 1.696557 | 0.0897804 | 0.18501683 | FALSE |
| EPN1 | 5678 | 1.696193 | 0.0898493 | 0.1851344 | FALSE |
| PRKX | 3823 | 1.695596 | 0.0899624 | 0.18534297 | FALSE |
| COL4A2 | 9315 | 1.6946 | 0.0901513 | 0.18563431 | FALSE |
| DNAJB1 | 11039 | 1.693382 | 0.0903828 | 0.18603739 | FALSE |
| WNT11 | 7019 | 1.692892 | 0.090476 | 0.18620481 | FALSE |
| C6orf203 | 5432 | 1.692074 | 0.0906319 | 0.18643324 | FALSE |
| CSPG5 | 1942 | 1.691913 | 0.0906626 | 0.18646597 | FALSE |
| RBP1 | 7255 | 1.690962 | 0.0908441 | 0.18676554 | FALSE |
| ARPC5 | 771 | 1.689537 | 0.0911166 | 0.18725191 | FALSE |
| PLEKHF2 | 9324 | 1.689385 | 0.0911457 | 0.18728711 | FALSE |
| LINC01554 | 3973 | 1.688726 | 0.091272 | 0.18749729 | FALSE |
| DCD | 3240 | 1.688393 | 0.0913358 | 0.18760383 | FALSE |
| CAMK4 | 12858 | 1.687072 | 0.0915895 | 0.1880508 | FALSE |
| LAMC2 | 3970 | 1.686657 | 0.0916693 | 0.18816723 | FALSE |
| AGTPBP1 | 106 | 1.68652 | 0.0916957 | 0.18819465 | FALSE |
| PRSS1 | 3850 | 1.686401 | 0.0917186 | 0.18819738 | FALSE |
| NEURL1 | 10575 | 1.685756 | 0.0918428 | 0.18840891 | FALSE |
| ARHGEF19 | 13458 | 1.685673 | 0.0918588 | 0.18840891 | FALSE |
| GPANK1 | 2862 | 1.685665 | 0.0918603 | 0.18840891 | FALSE |
| ZNF341 | 10288 | 1.685197 | 0.0919506 | 0.1885445 | FALSE |
| EMC9 | 2080 | 1.685096 | 0.0919701 | 0.18855971 | FALSE |
| CCDC85A | 11613 | 1.683381 | 0.0923014 | 0.18918935 | FALSE |
| DTX1 | 12875 | 1.682698 | 0.0924336 | 0.18941067 | FALSE |
| TEKT2 | 4689 | 1.681692 | 0.0926286 | 0.18976291 | FALSE |
| MAPRE2 | 10488 | 1.681686 | 0.0926297 | 0.18976291 | FALSE |
| SOWAHC | 8984 | 1.68158 | 0.0926503 | 0.18977678 | FALSE |
| RPS4Y2 | 10700 | 1.681526 | 0.0926608 | 0.18977678 | FALSE |
| HDDC2 | 15153 | 1.681249 | 0.0927146 | 0.18983716 | FALSE |
| DROSHA | 8565 | 1.680813 | 0.0927992 | 0.18998567 | FALSE |
| TRABD2A | 5398 | 1.680625 | 0.0928358 | 0.19003558 | FALSE |
| ZNF826P | 5411 | 1.680554 | 0.0928496 | 0.19003894 | FALSE |
| ZBTB44 | 10961 | 1.679404 | 0.0930733 | 0.19040177 | FALSE |
| CELA2B | 2391 | 1.679061 | 0.0931402 | 0.19048405 | FALSE |
| KCNG3 | 10093 | 1.678763 | 0.0931982 | 0.19057791 | FALSE |
| C4orf19 | 2807 | 1.678103 | 0.093327 | 0.19081623 | FALSE |
| CD151 | 12902 | 1.676328 | 0.093674 | 0.19140048 | FALSE |
| NR0B1 | 14656 | 1.67614 | 0.0937108 | 0.19145067 | FALSE |
| NOS1 | 4480 | 1.675955 | 0.093747 | 0.19148665 | FALSE |
| GEMIN8P4 | 12918 | 1.675223 | 0.0938905 | 0.19170098 | FALSE |
| ACTG2 | 2954 | 1.675202 | 0.0938946 | 0.19170098 | FALSE |
| FZD3 | 3886 | 1.674238 | 0.0940838 | 0.19201209 | FALSE |
| DDOST | 9063 | 1.673774 | 0.094175 | 0.19216407 | FALSE |
| RPL21 | 8321 | 1.673734 | 0.0941829 | 0.19216407 | FALSE |
| PCYOX1L | 14712 | 1.673452 | 0.0942383 | 0.19225213 | FALSE |
| SOX3 | 14937 | 1.672529 | 0.0944201 | 0.19252233 | FALSE |
| IGDCC4 | 11710 | 1.671978 | 0.0945287 | 0.19261816 | FALSE |
| TOX4 | 3679 | 1.671583 | 0.0946066 | 0.19275181 | FALSE |
| NECTIN1 | 2599 | 1.671469 | 0.0946291 | 0.19277253 | FALSE |
| R3HCC1 | 10757 | 1.671056 | 0.0947106 | 0.19288836 | FALSE |
| HIGD1A | 6302 | 1.670985 | 0.0947246 | 0.1928918 | FALSE |
| DYRK1B | 4138 | 1.670661 | 0.0947887 | 0.19297188 | FALSE |
| GLRX5 | 213 | 1.670067 | 0.0949061 | 0.19318583 | FALSE |
| INSM1 | 2061 | 1.66901 | 0.0951154 | 0.19356145 | FALSE |
| FARSA | 3080 | 1.668733 | 0.0951703 | 0.19364798 | FALSE |
| CCDC80 | 14984 | 1.668601 | 0.0951965 | 0.19367603 | FALSE |
| HLA-F-AS1 | 5041 | 1.668227 | 0.0952707 | 0.19380175 | FALSE |
| SERPINI2 | 642 | 1.667805 | 0.0953544 | 0.1939215 | FALSE |
| RPAP1 | 1513 | 1.667743 | 0.0953668 | 0.1939215 | FALSE |
| ATP5PO | 7653 | 1.667653 | 0.0953846 | 0.19393262 | FALSE |
| GLIPR2 | 9657 | 1.666845 | 0.0955452 | 0.19413291 | FALSE |
| ACCS | 155 | 1.66668 | 0.0955781 | 0.19417436 | FALSE |
| BASP1 | 3614 | 1.66567 | 0.0957792 | 0.19446733 | FALSE |
| TIMM17A | 2367 | 1.665581 | 0.0957969 | 0.19446733 | FALSE |
| CSF2RA | 12602 | 1.665457 | 0.0958216 | 0.19449225 | FALSE |
| S100A2 | 7824 | 1.66525 | 0.0958629 | 0.19455077 | FALSE |
| PPP1R42 | 3999 | 1.664994 | 0.095914 | 0.19462913 | FALSE |
| ZMYM3 | 13160 | 1.664533 | 0.096006 | 0.19479055 | FALSE |
| GRIK2 | 2449 | 1.664081 | 0.0960962 | 0.19492313 | FALSE |
| CACNA2D1 | 10449 | 1.662544 | 0.0964037 | 0.19544544 | FALSE |
| STT3A | 1642 | 1.662133 | 0.0964861 | 0.19558705 | FALSE |
| TREX1 | 2805 | 1.661737 | 0.0965655 | 0.19569727 | FALSE |
| SELENBP1 | 9621 | 1.660913 | 0.0967309 | 0.19600706 | FALSE |
| NOTCH2NL | 4953 | 1.660743 | 0.0967651 | 0.19605085 | FALSE |
| LINC00982 | 135 | 1.660286 | 0.0968569 | 0.19621153 | FALSE |
| IDS | 10186 | 1.660118 | 0.0968907 | 0.19623482 | FALSE |
| SHANK2 | 7076 | 1.660104 | 0.0968935 | 0.19623482 | FALSE |
| TRPC1 | 12346 | 1.659934 | 0.0969277 | 0.19627866 | FALSE |
| RPL23AP32 | 1036 | 1.65975 | 0.0969648 | 0.1963282 | FALSE |
| EME2 | 10053 | 1.659683 | 0.0969782 | 0.19633008 | FALSE |
| PLA1A | 3908 | 1.65938 | 0.0970392 | 0.19642814 | FALSE |
| RPL3 | 3820 | 1.658609 | 0.0971946 | 0.19661533 | FALSE |
| ZAP70 | 13701 | 1.658186 | 0.0972799 | 0.19676247 | FALSE |
| PUS7L | 14550 | 1.657604 | 0.0973974 | 0.19697463 | FALSE |
| NETO1 | 6367 | 1.657401 | 0.0974384 | 0.19703207 | FALSE |
| NR4A2 | 4786 | 1.656086 | 0.0977044 | 0.19749327 | FALSE |
| DDR2 | 10644 | 1.655542 | 0.0978146 | 0.19769046 | FALSE |
| TCTN1 | 3763 | 1.655327 | 0.0978582 | 0.19772741 | FALSE |
| PRSS36 | 8091 | 1.655158 | 0.0978925 | 0.19777109 | FALSE |
| JTB | 6392 | 1.654787 | 0.0979677 | 0.19789756 | FALSE |
| PRMT1 | 11642 | 1.654 | 0.0981275 | 0.19816915 | FALSE |
| MTHFD1L | 1643 | 1.653594 | 0.09821 | 0.19831018 | FALSE |
| HARS2 | 4276 | 1.653358 | 0.098258 | 0.19837926 | FALSE |
| KRTAP2-1 | 1300 | 1.65302 | 0.0983268 | 0.19845739 | FALSE |
| SNX10 | 3558 | 1.652986 | 0.0983337 | 0.19845739 | FALSE |
| PSMB2 | 635 | 1.652358 | 0.0984616 | 0.19866418 | FALSE |
| CYSLTR1 | 10533 | 1.652233 | 0.0984871 | 0.19868993 | FALSE |
| RPL13A | 5964 | 1.651491 | 0.0986384 | 0.19892473 | FALSE |
| LOC105379807 | 13048 | 1.651475 | 0.0986416 | 0.19892473 | FALSE |
| EIF2B4 | 6000 | 1.651083 | 0.0987216 | 0.19905859 | FALSE |
| ACBD6 | 12332 | 1.651025 | 0.0987335 | 0.19905859 | FALSE |
| BRD3OS | 7876 | 1.650271 | 0.0988875 | 0.19931777 | FALSE |
| STXBP6 | 13720 | 1.64974 | 0.0989961 | 0.19951095 | FALSE |
| TDRD10 | 15046 | 1.649392 | 0.0990674 | 0.19962876 | FALSE |
| PRKD1 | 13360 | 1.649227 | 0.0991011 | 0.1996711 | FALSE |
| CCT2 | 3371 | 1.648948 | 0.0991583 | 0.19976051 | FALSE |
| TACR2 | 9757 | 1.648707 | 0.0992077 | 0.1998085 | FALSE |
| INO80C | 12720 | 1.64862 | 0.0992255 | 0.19981868 | FALSE |
| SAR1A | 9806 | 1.648195 | 0.0993127 | 0.19991693 | FALSE |
| SNRPF | 12907 | 1.648086 | 0.099335 | 0.1999362 | FALSE |
| BLOC1S2 | 13394 | 1.647685 | 0.0994173 | 0.20002983 | FALSE |
| ZNF462 | 622 | 1.647153 | 0.0995266 | 0.20011567 | FALSE |
| HEXA | 1891 | 1.646231 | 0.0997162 | 0.20041958 | FALSE |
| RPS19BP1 | 2488 | 1.645918 | 0.0997806 | 0.2005233 | FALSE |
| SKIV2L | 8179 | 1.645646 | 0.0998367 | 0.20058887 | FALSE |
| GNA13 | 9719 | 1.645147 | 0.0999395 | 0.20076513 | FALSE |
| MRGPRD | 13412 | 1.6447 | 0.1000317 | 0.20088698 | FALSE |
| MYOM1 | 5703 | 1.641679 | 0.1006565 | 0.20194607 | FALSE |
| CALR | 11364 | 1.641406 | 0.1007132 | 0.20203374 | FALSE |
| SREBF1 | 13036 | 1.641315 | 0.100732 | 0.20204568 | FALSE |
| FTMT | 1273 | 1.638638 | 0.1012887 | 0.20300588 | FALSE |
| BRPF1 | 12257 | 1.638315 | 0.101356 | 0.20311477 | FALSE |
| OTUB1 | 5024 | 1.6374 | 0.1015469 | 0.20347129 | FALSE |
| BCHE | 2081 | 1.636204 | 0.1017969 | 0.20381544 | FALSE |
| ZNF480 | 13365 | 1.635869 | 0.101867 | 0.20392943 | FALSE |
| SDC4 | 2742 | 1.635181 | 0.1020111 | 0.20411446 | FALSE |
| ZNF827 | 10632 | 1.635179 | 0.1020115 | 0.20411446 | FALSE |
| MFSD5 | 12588 | 1.634845 | 0.1020815 | 0.20422841 | FALSE |
| SAMM50 | 8633 | 1.634597 | 0.1021336 | 0.20430632 | FALSE |
| ANKRD28 | 1236 | 1.632535 | 0.1025668 | 0.20501564 | FALSE |
| RALA | 7474 | 1.632441 | 0.1025866 | 0.20502897 | FALSE |
| HAX1 | 11455 | 1.631896 | 0.1027014 | 0.20517967 | FALSE |
| EIF3M | 4551 | 1.631755 | 0.1027311 | 0.20520427 | FALSE |
| WNT7A | 666 | 1.631713 | 0.10274 | 0.20520427 | FALSE |
| CDHR2 | 4702 | 1.631191 | 0.10285 | 0.20537162 | FALSE |
| ACSS3 | 3422 | 1.630024 | 0.1030964 | 0.20581106 | FALSE |
| MOB3A | 11225 | 1.628625 | 0.1033924 | 0.20624395 | FALSE |
| AASS | 8436 | 1.62805 | 0.1035143 | 0.20644322 | FALSE |
| NDUFB2 | 15603 | 1.628029 | 0.1035187 | 0.20644322 | FALSE |
| CCL22 | 14848 | 1.6269 | 0.1037583 | 0.20686827 | FALSE |
| CD8A | 13452 | 1.626049 | 0.1039392 | 0.2072025 | FALSE |
| CCNB3 | 5297 | 1.625389 | 0.1040797 | 0.20745607 | FALSE |
| NIPSNAP2 | 8952 | 1.623469 | 0.1044892 | 0.20821918 | FALSE |
| ARFGEF3 | 7352 | 1.623267 | 0.1045323 | 0.20827862 | FALSE |
| ZNF233 | 13778 | 1.622999 | 0.1045896 | 0.20833964 | FALSE |
| RPP40 | 6428 | 1.621681 | 0.1048717 | 0.20879321 | FALSE |
| FAM201A | 13827 | 1.621217 | 0.1049711 | 0.20891322 | FALSE |
| ETFDH | 14425 | 1.621035 | 0.1050101 | 0.20896428 | FALSE |
| TMEM43 | 8500 | 1.619936 | 0.105246 | 0.20938039 | FALSE |
| LSM10 | 11921 | 1.619542 | 0.1053307 | 0.20952218 | FALSE |
| TLR9 | 11164 | 1.618475 | 0.1055603 | 0.20984533 | FALSE |
| SLC1A3 | 5399 | 1.61609 | 0.1060749 | 0.2106311 | FALSE |
| IL17RB | 12825 | 1.616081 | 0.1060768 | 0.2106311 | FALSE |
| SUCLG2 | 7932 | 1.61599 | 0.1060965 | 0.21064341 | FALSE |
| FAM92A1P2 | 3772 | 1.615832 | 0.1061306 | 0.21068448 | FALSE |
| AK5 | 4144 | 1.615643 | 0.1061715 | 0.21073888 | FALSE |
| SLC1A1 | 7942 | 1.613501 | 0.1066357 | 0.21151461 | FALSE |
| DLX2 | 4768 | 1.612932 | 0.1067593 | 0.21161902 | FALSE |
| PPAT | 1878 | 1.612911 | 0.1067638 | 0.21161902 | FALSE |
| LOC286359 | 7913 | 1.611764 | 0.1070133 | 0.21200596 | FALSE |
| KRTAP17-1 | 3491 | 1.610814 | 0.1072203 | 0.21236215 | FALSE |
| NLGN3 | 6749 | 1.610026 | 0.1073922 | 0.21264876 | FALSE |
| LSP1 | 5635 | 1.609714 | 0.1074603 | 0.21272977 | FALSE |
| GDAP1 | 5694 | 1.608737 | 0.1076739 | 0.21309855 | FALSE |
| RPL23A | 3383 | 1.607956 | 0.1078448 | 0.21335716 | FALSE |
| STYXL1 | 13635 | 1.607953 | 0.1078455 | 0.21335716 | FALSE |
| NTAN1 | 1047 | 1.60779 | 0.1078812 | 0.21340079 | FALSE |
| ZC2HC1C | 5096 | 1.607706 | 0.1078996 | 0.2134102 | FALSE |
| PSMF1 | 11215 | 1.6069 | 0.1080763 | 0.21365161 | FALSE |
| HIST1H2BE | 986 | 1.606793 | 0.1080998 | 0.213671 | FALSE |
| SPIN1 | 10924 | 1.605714 | 0.1083368 | 0.21408528 | FALSE |
| DHCR24 | 10647 | 1.605638 | 0.1083535 | 0.21409123 | FALSE |
| ALDH4A1 | 2774 | 1.605396 | 0.1084067 | 0.2141693 | FALSE |
| TMEM150B | 3201 | 1.604716 | 0.1085563 | 0.21438365 | FALSE |
| MLEC | 11634 | 1.604097 | 0.1086927 | 0.21457161 | FALSE |
| PELO | 13387 | 1.604001 | 0.1087138 | 0.21458628 | FALSE |
| TTL | 11753 | 1.60271 | 0.1089987 | 0.2150671 | FALSE |
| GJA8 | 10972 | 1.602089 | 0.1091359 | 0.21528353 | FALSE |
| RPL17 | 8198 | 1.601624 | 0.1092388 | 0.21542058 | FALSE |
| B3GNT8 | 11532 | 1.600957 | 0.1093864 | 0.21564165 | FALSE |
| LRRC8B | 3354 | 1.600281 | 0.1095363 | 0.21582813 | FALSE |
| CCDC70 | 8 | 1.600191 | 0.1095562 | 0.21583883 | FALSE |
| LSM2 | 11719 | 1.599639 | 0.1096787 | 0.21591827 | FALSE |
| GLCE | 1764 | 1.598729 | 0.1098808 | 0.2162073 | FALSE |
| APBA1 | 6225 | 1.598188 | 0.1100011 | 0.2164168 | FALSE |
| NEUROD6 | 14556 | 1.598077 | 0.1100258 | 0.21643815 | FALSE |
| AK3 | 12457 | 1.597122 | 0.1102385 | 0.21677468 | FALSE |
| TSPAN3 | 15282 | 1.595676 | 0.1105612 | 0.21727246 | FALSE |
| LOC101927027 | 799 | 1.594451 | 0.1108351 | 0.21770128 | FALSE |
| EIF3L | 5832 | 1.592865 | 0.1111905 | 0.21826228 | FALSE |
| WDHD1 | 7101 | 1.592606 | 0.1112486 | 0.21834896 | FALSE |
| MGC16275 | 1345 | 1.591959 | 0.1113939 | 0.21860672 | FALSE |
| FBXO6 | 11300 | 1.590712 | 0.1116744 | 0.21893729 | FALSE |
| DECR2 | 12376 | 1.590532 | 0.1117149 | 0.21896184 | FALSE |
| KIAA1211 | 6729 | 1.590461 | 0.1117309 | 0.21896573 | FALSE |
| LY6G5C | 11496 | 1.589215 | 0.1120119 | 0.21939646 | FALSE |
| ANKIB1 | 5780 | 1.587904 | 0.112308 | 0.21990377 | FALSE |
| GSTA4 | 3930 | 1.587455 | 0.1124096 | 0.22004755 | FALSE |
| TFRC | 9275 | 1.587268 | 0.112452 | 0.22006894 | FALSE |
| ETF1 | 5701 | 1.586653 | 0.1125913 | 0.22021007 | FALSE |
| HEY2 | 4942 | 1.585237 | 0.1129125 | 0.22078312 | FALSE |
| DNAJC5 | 10036 | 1.584083 | 0.1131748 | 0.22121307 | FALSE |
| DCK | 11145 | 1.583871 | 0.1132231 | 0.22127971 | FALSE |
| F3 | 711 | 1.58355 | 0.1132962 | 0.22136719 | FALSE |
| AGBL4 | 2334 | 1.582939 | 0.1134354 | 0.22152843 | FALSE |
| PSPH | 4887 | 1.582844 | 0.113457 | 0.22154305 | FALSE |
| NRDC | 1485 | 1.582419 | 0.113554 | 0.22170462 | FALSE |
| GADD45G | 8850 | 1.581887 | 0.1136754 | 0.22185856 | FALSE |
| SELENOH | 1607 | 1.581761 | 0.1137041 | 0.22188701 | FALSE |
| DDIT4L | 6261 | 1.581427 | 0.1137804 | 0.22200819 | FALSE |
| ACSBG1 | 10639 | 1.580918 | 0.1138968 | 0.22220607 | FALSE |
| SHANK1 | 7701 | 1.580859 | 0.1139103 | 0.22220607 | FALSE |
| SPTY2D1 | 385 | 1.580743 | 0.1139368 | 0.22223009 | FALSE |
| RHCE | 9257 | 1.580678 | 0.1139517 | 0.22223137 | FALSE |
| SELENOK | 10707 | 1.579782 | 0.1141568 | 0.22260362 | FALSE |
| CD14 | 6230 | 1.578027 | 0.1145594 | 0.22324947 | FALSE |
| ZNRF2 | 11330 | 1.576749 | 0.1148533 | 0.22376639 | FALSE |
| SYT6 | 14786 | 1.576495 | 0.1149118 | 0.22385242 | FALSE |
| SNRPD1 | 9968 | 1.575416 | 0.1151605 | 0.22422511 | FALSE |
| ABCG8 | 5592 | 1.575098 | 0.1152338 | 0.22434002 | FALSE |
| ITGA8 | 4721 | 1.575011 | 0.1152539 | 0.22435118 | FALSE |
| TIPIN | 14508 | 1.574933 | 0.1152719 | 0.22435829 | FALSE |
| ZSCAN10 | 12493 | 1.574631 | 0.1153416 | 0.22446607 | FALSE |
| PNMA8B | 1723 | 1.574464 | 0.1153802 | 0.22451319 | FALSE |
| NORAD | 8629 | 1.574373 | 0.1154012 | 0.22451844 | FALSE |
| MRPS23 | 3287 | 1.5742 | 0.1154412 | 0.22454804 | FALSE |
| TMEM150C | 14034 | 1.573899 | 0.1155108 | 0.22465543 | FALSE |
| SCG2 | 11306 | 1.5734 | 0.1156262 | 0.22485195 | FALSE |
| PRAM1 | 6958 | 1.572826 | 0.1157591 | 0.22508237 | FALSE |
| LONRF2 | 8384 | 1.572078 | 0.1159325 | 0.22539139 | FALSE |
| PALM3 | 4921 | 1.571692 | 0.116022 | 0.22553742 | FALSE |
| ATP1B2 | 4005 | 1.571394 | 0.1160912 | 0.2256438 | FALSE |
| SFRP2 | 3352 | 1.571311 | 0.1161104 | 0.2256532 | FALSE |
| EIF1AD | 3623 | 1.571104 | 0.1161585 | 0.22566245 | FALSE |
| CES4A | 9999 | 1.56937 | 0.1165618 | 0.22619373 | FALSE |
| POLR2F | 145 | 1.569341 | 0.1165685 | 0.22619373 | FALSE |
| LRIG1 | 11632 | 1.569306 | 0.1165767 | 0.22619373 | FALSE |
| HDLBP | 6706 | 1.568473 | 0.1167708 | 0.22648607 | FALSE |
| GPC1 | 7085 | 1.568056 | 0.1168681 | 0.22664663 | FALSE |
| PAPLN | 15062 | 1.567644 | 0.1169642 | 0.22678502 | FALSE |
| LKAAEAR1 | 1682 | 1.566796 | 0.1171624 | 0.22713288 | FALSE |
| RAB15 | 10781 | 1.566303 | 0.1172777 | 0.2272355 | FALSE |
| DTD1 | 2713 | 1.566259 | 0.117288 | 0.2272355 | FALSE |
| AP1M1 | 8605 | 1.565457 | 0.1174758 | 0.22734667 | FALSE |
| TAC4 | 2142 | 1.564272 | 0.1177537 | 0.22779899 | FALSE |
| MMP28 | 14899 | 1.564131 | 0.1177868 | 0.22783484 | FALSE |
| MS4A15 | 8152 | 1.563007 | 0.1180509 | 0.22825827 | FALSE |
| SULT1C4 | 13784 | 1.561808 | 0.1183332 | 0.22872198 | FALSE |
| TMEM219 | 13953 | 1.561569 | 0.1183896 | 0.22880257 | FALSE |
| SRA1 | 5578 | 1.56127 | 0.1184601 | 0.22888223 | FALSE |
| PDLIM7 | 10708 | 1.561199 | 0.1184768 | 0.22888629 | FALSE |
| ELOC | 2355 | 1.56103 | 0.1185167 | 0.22890674 | FALSE |
| RAB33A | 15582 | 1.559713 | 0.1188277 | 0.22936581 | FALSE |
| STAU1 | 13469 | 1.559357 | 0.1189119 | 0.22949998 | FALSE |
| YAP1 | 7416 | 1.558994 | 0.1189978 | 0.2296374 | FALSE |
| NDUFC2 | 13885 | 1.558122 | 0.1192043 | 0.22991829 | FALSE |
| MYCN | 2881 | 1.555828 | 0.119749 | 0.2307168 | FALSE |
| SMARCD1 | 5688 | 1.554656 | 0.120028 | 0.23122588 | FALSE |
| SLC22A12 | 3554 | 1.554103 | 0.1201599 | 0.23142281 | FALSE |
| PHF13 | 10304 | 1.553985 | 0.120188 | 0.2314485 | FALSE |
| RPN2 | 12204 | 1.553871 | 0.1202152 | 0.23147141 | FALSE |
| CFDP1 | 1991 | 1.553811 | 0.1202295 | 0.23147141 | FALSE |
| ST13P4 | 5026 | 1.553641 | 0.1202701 | 0.231521 | FALSE |
| OSBPL8 | 14087 | 1.55278 | 0.1204757 | 0.23185974 | FALSE |
| ATG12 | 6801 | 1.552653 | 0.1205061 | 0.23187763 | FALSE |
| SPACA3 | 3398 | 1.552126 | 0.1206321 | 0.23204642 | FALSE |
| ULK2 | 13158 | 1.55174 | 0.1207244 | 0.23216697 | FALSE |
| LOC101928424 | 4253 | 1.551291 | 0.120832 | 0.23234515 | FALSE |
| CHST7 | 2547 | 1.550888 | 0.1209285 | 0.23250223 | FALSE |
| ZDHHC22 | 14632 | 1.550266 | 0.1210777 | 0.23273176 | FALSE |
| DDC | 10302 | 1.549944 | 0.121155 | 0.23285167 | FALSE |
| IRF5 | 14377 | 1.549812 | 0.1211866 | 0.23288394 | FALSE |
| C5 | 4334 | 1.548945 | 0.1213949 | 0.23319822 | FALSE |
| RAB5A | 958 | 1.547906 | 0.1216449 | 0.23362103 | FALSE |
| SNHG20 | 3849 | 1.547641 | 0.1217088 | 0.23368619 | FALSE |
| NKX2-3 | 4745 | 1.546794 | 0.1219129 | 0.23402073 | FALSE |
| BTBD2 | 14055 | 1.54593 | 0.1221215 | 0.23432219 | FALSE |
| RRAGC | 9326 | 1.544251 | 0.1225275 | 0.23488326 | FALSE |
| SMIM7 | 459 | 1.544067 | 0.1225721 | 0.23493988 | FALSE |
| RPL5 | 9040 | 1.543913 | 0.1226094 | 0.23495378 | FALSE |
| SYT9 | 5075 | 1.543847 | 0.1226254 | 0.23495562 | FALSE |
| KANK1 | 12661 | 1.54378 | 0.1226416 | 0.23495793 | FALSE |
| DOCK7 | 5944 | 1.543413 | 0.1227306 | 0.23509956 | FALSE |
| ELF4 | 3120 | 1.543109 | 0.1228043 | 0.23521197 | FALSE |
| TSG101 | 8725 | 1.542907 | 0.1228533 | 0.23527702 | FALSE |
| FGFR3 | 12771 | 1.542443 | 0.122966 | 0.23546388 | FALSE |
| NACAD | 6711 | 1.541994 | 0.1230751 | 0.23558617 | FALSE |
| PNPLA7 | 7501 | 1.540512 | 0.1234356 | 0.23614527 | FALSE |
| PARP6 | 301 | 1.539125 | 0.1237738 | 0.23660498 | FALSE |
| OTOS | 14130 | 1.538542 | 0.1239161 | 0.23684816 | FALSE |
| CDC123 | 11746 | 1.538274 | 0.1239816 | 0.23694436 | FALSE |
| UQCC1 | 2263 | 1.538055 | 0.1240352 | 0.23701255 | FALSE |
| FAM126A | 3958 | 1.537878 | 0.1240784 | 0.23704245 | FALSE |
| SNHG11 | 7862 | 1.536794 | 0.1243438 | 0.23740426 | FALSE |
| SPIN4 | 10993 | 1.536348 | 0.1244531 | 0.23755489 | FALSE |
| ECI2 | 11400 | 1.535193 | 0.1247364 | 0.23792149 | FALSE |
| PCYT2 | 11388 | 1.534709 | 0.1248553 | 0.23811922 | FALSE |
| KCMF1 | 8957 | 1.534089 | 0.1250078 | 0.23838086 | FALSE |
| LOR | 11316 | 1.532847 | 0.1253136 | 0.23884746 | FALSE |
| BRMS1L | 8111 | 1.531533 | 0.1256377 | 0.23928274 | FALSE |
| LDAH | 15193 | 1.531487 | 0.1256491 | 0.23928274 | FALSE |
| SKAP1 | 11505 | 1.531158 | 0.1257303 | 0.23937499 | FALSE |
| LINC00886 | 4755 | 1.53114 | 0.1257348 | 0.23937499 | FALSE |
| GPR132 | 13640 | 1.53105 | 0.125757 | 0.23937499 | FALSE |
| HUWE1 | 8591 | 1.530953 | 0.125781 | 0.23938818 | FALSE |
| LFNG | 4021 | 1.530194 | 0.1259687 | 0.23960008 | FALSE |
| CYP3A5 | 5632 | 1.53014 | 0.1259821 | 0.23960008 | FALSE |
| ILVBL | 394 | 1.530131 | 0.1259843 | 0.23960008 | FALSE |
| ADRA2C | 10732 | 1.528629 | 0.1263564 | 0.24019094 | FALSE |
| LINC00643 | 11534 | 1.528024 | 0.1265066 | 0.24037452 | FALSE |
| CCDC173 | 4001 | 1.527998 | 0.126513 | 0.24037452 | FALSE |
| CSPG4 | 12796 | 1.527631 | 0.1266042 | 0.24051563 | FALSE |
| CLEC18B | 13061 | 1.526737 | 0.1268264 | 0.24085003 | FALSE |
| RWDD4 | 15095 | 1.525628 | 0.1271025 | 0.24122787 | FALSE |
| CH17-340M24.3 | 7516 | 1.5242 | 0.1274588 | 0.24178653 | FALSE |
| DLG3 | 10065 | 1.523419 | 0.1276539 | 0.24206861 | FALSE |
| CAMLG | 14582 | 1.522754 | 0.1278203 | 0.24229589 | FALSE |
| AOAH | 11563 | 1.521262 | 0.1281941 | 0.24279845 | FALSE |
| USP14 | 5521 | 1.520506 | 0.1283839 | 0.24309893 | FALSE |
| ACADVL | 8185 | 1.519779 | 0.1285665 | 0.24336882 | FALSE |
| GABRB3 | 11176 | 1.519691 | 0.1285887 | 0.24336882 | FALSE |
| RAD51D | 4784 | 1.519581 | 0.1286163 | 0.2433917 | FALSE |
| PLAC4 | 257 | 1.519374 | 0.1286684 | 0.2434561 | FALSE |
| CRK | 1148 | 1.518944 | 0.1287766 | 0.24357707 | FALSE |
| RAD21 | 14718 | 1.518716 | 0.128834 | 0.24365617 | FALSE |
| BDH2 | 3981 | 1.516655 | 0.1293538 | 0.24452094 | FALSE |
| LRFN3 | 2266 | 1.515487 | 0.1296491 | 0.24501991 | FALSE |
| ZNF18 | 381 | 1.515059 | 0.1297575 | 0.24516539 | FALSE |
| IARS | 9942 | 1.51429 | 0.1299523 | 0.24547898 | FALSE |
| ZWILCH | 4416 | 1.51428 | 0.1299548 | 0.24547898 | FALSE |
| TAGLN3 | 7735 | 1.514137 | 0.1299911 | 0.2455178 | FALSE |
| LRRC47 | 13648 | 1.514047 | 0.1300139 | 0.24553124 | FALSE |
| GMPPB | 13798 | 1.513647 | 0.1301154 | 0.24559902 | FALSE |
| NAA35 | 14474 | 1.513645 | 0.1301159 | 0.24559902 | FALSE |
| MAB21L2 | 4680 | 1.513557 | 0.1301382 | 0.24559902 | FALSE |
| CBX2 | 7134 | 1.512997 | 0.1302804 | 0.24579699 | FALSE |
| SLC7A6OS | 3669 | 1.510489 | 0.1309187 | 0.24679271 | FALSE |
| AKR1C4 | 2354 | 1.510248 | 0.1309802 | 0.2468788 | FALSE |
| ZNRF4 | 6629 | 1.509946 | 0.1310572 | 0.24699425 | FALSE |
| DPY30 | 1124 | 1.50943 | 0.1311889 | 0.24705622 | FALSE |
| RPS15A | 10391 | 1.509412 | 0.1311935 | 0.24705622 | FALSE |
| ZDHHC24 | 741 | 1.509332 | 0.131214 | 0.24705622 | FALSE |
| P2RX2 | 3664 | 1.509322 | 0.1312165 | 0.24705622 | FALSE |
| TMED10P1 | 7929 | 1.508629 | 0.1313936 | 0.24727055 | FALSE |
| FOXO1 | 12122 | 1.508019 | 0.1315497 | 0.24747484 | FALSE |
| DRG1 | 3620 | 1.507915 | 0.1315763 | 0.24749513 | FALSE |
| AKAP11 | 9593 | 1.507764 | 0.1316149 | 0.24752562 | FALSE |
| CDC16 | 1141 | 1.507728 | 0.1316242 | 0.24752562 | FALSE |
| HBA2 | 3351 | 1.507057 | 0.131796 | 0.24781905 | FALSE |
| GARNL3 | 8050 | 1.505301 | 0.1322467 | 0.24854686 | FALSE |
| ZNF512B | 4584 | 1.505169 | 0.1322806 | 0.24858074 | FALSE |
| FABP1 | 10250 | 1.504285 | 0.132508 | 0.24888833 | FALSE |
| BEST4 | 11199 | 1.503937 | 0.1325976 | 0.24900983 | FALSE |
| CALD1 | 13915 | 1.50391 | 0.1326045 | 0.24900983 | FALSE |
| MAGEF1 | 8925 | 1.503627 | 0.1326774 | 0.24911678 | FALSE |
| GNG8 | 5050 | 1.503303 | 0.1327609 | 0.24921369 | FALSE |
| DVL1 | 13116 | 1.502051 | 0.1330839 | 0.24973007 | FALSE |
| ALDH7A1 | 5477 | 1.501636 | 0.1331911 | 0.24990124 | FALSE |
| GNB1L | 1114 | 1.501524 | 0.1332201 | 0.24992555 | FALSE |
| ATAT1 | 12051 | 1.501221 | 0.1332984 | 0.25004248 | FALSE |
| KIF26B | 6348 | 1.499593 | 0.1337199 | 0.25074279 | FALSE |
| SHISA7 | 10996 | 1.499247 | 0.1338096 | 0.2508809 | FALSE |
| FUK | 2336 | 1.498654 | 0.1339634 | 0.25112421 | FALSE |
| PPP1R3C | 13797 | 1.498623 | 0.1339715 | 0.25112421 | FALSE |
| DPP10 | 7344 | 1.498457 | 0.1340146 | 0.25117487 | FALSE |
| CRLF2 | 10882 | 1.498298 | 0.1340558 | 0.25119202 | FALSE |
| FSIP1 | 12418 | 1.497845 | 0.1341735 | 0.25132216 | FALSE |
| PPP2R3B | 14888 | 1.497584 | 0.1342414 | 0.25141911 | FALSE |
| MOCS2 | 7358 | 1.497246 | 0.1343293 | 0.25152346 | FALSE |
| MYBL2 | 4022 | 1.496452 | 0.1345359 | 0.25178976 | FALSE |
| SBDSP1 | 2011 | 1.496381 | 0.1345544 | 0.25179422 | FALSE |
| RPS12 | 2670 | 1.496106 | 0.134626 | 0.25188699 | FALSE |
| AMOT | 9341 | 1.495564 | 0.1347673 | 0.25204042 | FALSE |
| USP11 | 525 | 1.495549 | 0.1347712 | 0.25204042 | FALSE |
| CCDC124 | 9407 | 1.495351 | 0.1348229 | 0.25208537 | FALSE |
| NFIB | 2747 | 1.495131 | 0.1348803 | 0.25210516 | FALSE |
| NME3 | 6884 | 1.495125 | 0.1348818 | 0.25210516 | FALSE |
| RHOD | 13066 | 1.494145 | 0.1351377 | 0.25248176 | FALSE |
| ELAC1 | 6730 | 1.494106 | 0.1351479 | 0.25248176 | FALSE |
| SMOX | 1883 | 1.493711 | 0.1352512 | 0.25258411 | FALSE |
| RASSF3 | 13484 | 1.493451 | 0.1353192 | 0.25268091 | FALSE |
| FOXA3 | 2138 | 1.493309 | 0.1353563 | 0.25271288 | FALSE |
| OARD1 | 3662 | 1.492967 | 0.1354458 | 0.25280005 | FALSE |
| AXL | 8766 | 1.492537 | 0.1355584 | 0.2529162 | FALSE |
| EPN2 | 6574 | 1.491269 | 0.1358909 | 0.25350623 | FALSE |
| ARMCX5 | 7910 | 1.491087 | 0.1359387 | 0.25353484 | FALSE |
| C15orf40 | 8257 | 1.490558 | 0.1360776 | 0.25370313 | FALSE |
| CTH | 13375 | 1.488435 | 0.1366362 | 0.25462319 | FALSE |
| HDGFL3 | 7430 | 1.485936 | 0.1372961 | 0.25563949 | FALSE |
| CTXN2 | 15130 | 1.484793 | 0.1375987 | 0.25610561 | FALSE |
| RPL14 | 4309 | 1.484743 | 0.1376119 | 0.25610561 | FALSE |
| GRIA3 | 1582 | 1.484391 | 0.1377052 | 0.25621825 | FALSE |
| ATP9A | 10106 | 1.484158 | 0.137767 | 0.25630271 | FALSE |
| PKIG | 250 | 1.484006 | 0.1378073 | 0.25631671 | FALSE |
| PPP1R13L | 2087 | 1.483127 | 0.1380407 | 0.25665911 | FALSE |
| TMEM182 | 11809 | 1.4823 | 0.1382605 | 0.25694558 | FALSE |
| GRINA | 11648 | 1.481815 | 0.1383895 | 0.25702109 | FALSE |
| TLE3 | 8116 | 1.481653 | 0.1384326 | 0.25702109 | FALSE |
| FXYD5 | 4016 | 1.481244 | 0.1385416 | 0.25719277 | FALSE |
| TXNL1 | 6857 | 1.481003 | 0.1386058 | 0.25728141 | FALSE |
| AGFG2 | 13471 | 1.480649 | 0.1387001 | 0.25742599 | FALSE |
| ENOX2 | 398 | 1.480429 | 0.1387588 | 0.2575043 | FALSE |
| TLCD1 | 12604 | 1.480023 | 0.1388671 | 0.25767472 | FALSE |
| S100A4 | 8187 | 1.479337 | 0.1390503 | 0.25798396 | FALSE |
| CPNE8 | 5474 | 1.479059 | 0.1391245 | 0.25806448 | FALSE |
| RPS25 | 7890 | 1.479051 | 0.1391267 | 0.25806448 | FALSE |
| AMPD2 | 11743 | 1.478918 | 0.1391622 | 0.25809718 | FALSE |
| UNC45A | 5387 | 1.478794 | 0.1391954 | 0.25809718 | FALSE |
| ANO10 | 8170 | 1.478218 | 0.1393494 | 0.25829378 | FALSE |
| MFSD14A | 5307 | 1.478106 | 0.1393794 | 0.25831871 | FALSE |
| TJP3 | 10684 | 1.477958 | 0.139419 | 0.25836149 | FALSE |
| CTXND1 | 11372 | 1.477796 | 0.1394624 | 0.25839152 | FALSE |
| PDIA4 | 10711 | 1.477712 | 0.1394849 | 0.25839165 | FALSE |
| PTPRN | 1218 | 1.477566 | 0.139524 | 0.25843346 | FALSE |
| SSC5D | 669 | 1.477389 | 0.1395714 | 0.25849065 | FALSE |
| SPATA13 | 5197 | 1.4771 | 0.1396488 | 0.25860344 | FALSE |
| DIRC2 | 7413 | 1.47697 | 0.1396837 | 0.2586067 | FALSE |
| SCML1 | 6102 | 1.476519 | 0.1398046 | 0.25876931 | FALSE |
| PAOX | 7495 | 1.474917 | 0.1402349 | 0.25950421 | FALSE |
| VANGL1 | 3446 | 1.47445 | 0.1403605 | 0.25964445 | FALSE |
| CACYBP | 15151 | 1.47323 | 0.140689 | 0.26012911 | FALSE |
| MOXD1 | 7536 | 1.472927 | 0.1407707 | 0.26024937 | FALSE |
| TTC3 | 12287 | 1.472682 | 0.1408368 | 0.26034075 | FALSE |
| MYCNOS | 1070 | 1.471806 | 0.1410733 | 0.2606854 | FALSE |
| PSAT1 | 3569 | 1.471717 | 0.1410973 | 0.26069901 | FALSE |
| SNRPE | 12076 | 1.471356 | 0.1411949 | 0.26082024 | FALSE |
| GATM | 4183 | 1.471141 | 0.141253 | 0.2608365 | FALSE |
| UBTD2 | 753 | 1.470904 | 0.1413171 | 0.26092003 | FALSE |
| FAM89A | 8600 | 1.469919 | 0.1415837 | 0.26126401 | FALSE |
| OR2B11 | 141 | 1.469907 | 0.1415869 | 0.26126401 | FALSE |
| COPG2 | 4126 | 1.469444 | 0.1417124 | 0.26140295 | FALSE |
| C8G | 1722 | 1.469376 | 0.1417308 | 0.2614061 | FALSE |
| PLS3 | 3284 | 1.469138 | 0.1417954 | 0.26149427 | FALSE |
| CSF3R | 10748 | 1.468799 | 0.1418873 | 0.26163298 | FALSE |
| APTX | 8127 | 1.468703 | 0.1419134 | 0.26165015 | FALSE |
| NXT2 | 96 | 1.467988 | 0.1421075 | 0.26191538 | FALSE |
| S100A5 | 8880 | 1.467579 | 0.1422186 | 0.26205842 | FALSE |
| RRAGD | 6132 | 1.466514 | 0.1425083 | 0.26246847 | FALSE |
| WAC-AS1 | 4217 | 1.466285 | 0.1425707 | 0.2625365 | FALSE |
| TLCD2 | 5467 | 1.466002 | 0.1426477 | 0.26253969 | FALSE |
| TMEM231 | 5624 | 1.465461 | 0.1427952 | 0.26275371 | FALSE |
| AGBL5 | 14237 | 1.465452 | 0.1427976 | 0.26275371 | FALSE |
| FLOT1 | 7827 | 1.465113 | 0.1428901 | 0.26289288 | FALSE |
| STK26 | 6098 | 1.464437 | 0.1430746 | 0.26320134 | FALSE |
| SOX2 | 12800 | 1.463849 | 0.1432352 | 0.26337286 | FALSE |
| SERP1 | 6757 | 1.463388 | 0.1433612 | 0.26353574 | FALSE |
| ACTA2 | 13708 | 1.463003 | 0.1434666 | 0.2636432 | FALSE |
| B3GALT2 | 12097 | 1.462486 | 0.1436081 | 0.26381023 | FALSE |
| SYCE1L | 9884 | 1.461565 | 0.1438605 | 0.26418424 | FALSE |
| PGAP3 | 6013 | 1.461447 | 0.1438928 | 0.2642091 | FALSE |
| RPL13AP6 | 8109 | 1.461273 | 0.1439405 | 0.2642657 | FALSE |
| ITPKB | 12016 | 1.460809 | 0.1440679 | 0.26446841 | FALSE |
| HEPACAM | 134 | 1.460502 | 0.1441522 | 0.26453973 | FALSE |
| EIF4A3 | 2448 | 1.460458 | 0.1441642 | 0.26453973 | FALSE |
| KDELR3 | 10176 | 1.460445 | 0.1441678 | 0.26453973 | FALSE |
| ACTC1 | 1923 | 1.460421 | 0.1441744 | 0.26453973 | FALSE |
| MMP16 | 9380 | 1.459992 | 0.1442923 | 0.26466281 | FALSE |
| ANGPTL4 | 9633 | 1.459277 | 0.1444889 | 0.26489911 | FALSE |
| ADGRG6 | 1910 | 1.45867 | 0.144656 | 0.26517432 | FALSE |
| ADAM33 | 6679 | 1.457953 | 0.1448535 | 0.26541195 | FALSE |
| GTF2IRD2 | 1753 | 1.457844 | 0.1448835 | 0.26542146 | FALSE |
| RPS14P3 | 8834 | 1.456617 | 0.1452221 | 0.2659627 | FALSE |
| CBX1 | 6751 | 1.45577 | 0.1454562 | 0.26635178 | FALSE |
| ILF3 | 10478 | 1.455725 | 0.1454687 | 0.26635178 | FALSE |
| GUCY1A1 | 13977 | 1.455341 | 0.1455749 | 0.26648386 | FALSE |
| FAM57A | 3282 | 1.454741 | 0.145741 | 0.2666462 | FALSE |
| FAIM2 | 11329 | 1.454732 | 0.1457435 | 0.2666462 | FALSE |
| ST7-AS1 | 13247 | 1.454688 | 0.1457557 | 0.2666462 | FALSE |
| ATP6V1G2 | 9769 | 1.454651 | 0.1457659 | 0.2666462 | FALSE |
| HIST1H3C | 6548 | 1.454432 | 0.1458266 | 0.26669476 | FALSE |
| VPS18 | 543 | 1.454135 | 0.1459089 | 0.26681408 | FALSE |
| TPM1 | 13081 | 1.453032 | 0.1462149 | 0.26727982 | FALSE |
| PRSS3P2 | 1515 | 1.452442 | 0.1463787 | 0.2675481 | FALSE |
| RSF1 | 481 | 1.452096 | 0.1464749 | 0.26764082 | FALSE |
| PFDN2 | 4182 | 1.452046 | 0.1464888 | 0.26764082 | FALSE |
| TMEM199 | 13168 | 1.45189 | 0.1465322 | 0.26764082 | FALSE |
| PGRMC2 | 11501 | 1.451651 | 0.1465987 | 0.26773096 | FALSE |
| GLUL | 3358 | 1.451573 | 0.1466204 | 0.26773931 | FALSE |
| TLR1 | 9464 | 1.450613 | 0.1468877 | 0.26813343 | FALSE |
| MPP6 | 7675 | 1.449534 | 0.1471885 | 0.26848711 | FALSE |
| PLEKHG4B | 9917 | 1.449487 | 0.1472016 | 0.26848711 | FALSE |
| RAB3IL1 | 31 | 1.4491 | 0.1473097 | 0.26859523 | FALSE |
| ALK | 7433 | 1.448743 | 0.1474094 | 0.2687406 | FALSE |
| BMS1P14 | 10389 | 1.448623 | 0.1474429 | 0.26874261 | FALSE |
| RAB33B | 1039 | 1.448494 | 0.1474789 | 0.26877341 | FALSE |
| ZNF286A | 14253 | 1.448113 | 0.1475855 | 0.26892112 | FALSE |
| RELN | 2371 | 1.448081 | 0.1475944 | 0.26892112 | FALSE |
| TMSB15B | 9468 | 1.447586 | 0.1477329 | 0.26914205 | FALSE |
| DGKG | 12059 | 1.446038 | 0.1481665 | 0.26980636 | FALSE |
| HAND2 | 8347 | 1.44589 | 0.1482081 | 0.26983697 | FALSE |
| PKP3 | 3288 | 1.445272 | 0.1483815 | 0.2701034 | FALSE |
| SORCS2 | 1404 | 1.445172 | 0.1484096 | 0.27012306 | FALSE |
| CFAP97 | 1372 | 1.444683 | 0.1485469 | 0.27034161 | FALSE |
| SCYL3 | 4809 | 1.444258 | 0.1486664 | 0.27046379 | FALSE |
| CD40 | 1053 | 1.444198 | 0.1486833 | 0.27046379 | FALSE |
| STOML2 | 9701 | 1.444079 | 0.1487168 | 0.2704932 | FALSE |
| G6PC3 | 1508 | 1.443631 | 0.1488428 | 0.27065947 | FALSE |
| TMEM174 | 13235 | 1.44307 | 0.1490008 | 0.27091519 | FALSE |
| PGPEP1 | 11570 | 1.442925 | 0.1490416 | 0.27095794 | FALSE |
| RFX3 | 3306 | 1.442012 | 0.149299 | 0.27133121 | FALSE |
| SNU13 | 2529 | 1.44179 | 0.1493616 | 0.27138195 | FALSE |
| PLCE1 | 4946 | 1.438345 | 0.1503362 | 0.27296336 | FALSE |
| PEX7 | 2596 | 1.438343 | 0.1503368 | 0.27296336 | FALSE |
| ADGRF5 | 10741 | 1.438063 | 0.1504162 | 0.27307585 | FALSE |
| NMRAL1 | 10528 | 1.437976 | 0.1504409 | 0.27308895 | FALSE |
| FAM167A-AS1 | 7188 | 1.43721 | 0.1506583 | 0.27330643 | FALSE |
| ENHO | 8776 | 1.437154 | 0.1506742 | 0.27330643 | FALSE |
| TMEM100 | 3474 | 1.437123 | 0.150683 | 0.27330643 | FALSE |
| MPZL1 | 3480 | 1.436506 | 0.1508584 | 0.27359275 | FALSE |
| TMA7 | 14854 | 1.435119 | 0.1512532 | 0.27421328 | FALSE |
| KCNH7 | 4790 | 1.434879 | 0.1513216 | 0.27430546 | FALSE |
| CTNNA2 | 15327 | 1.43477 | 0.1513526 | 0.2743116 | FALSE |
| AGPAT1 | 10977 | 1.434255 | 0.1514995 | 0.27434169 | FALSE |
| NME4 | 1388 | 1.433888 | 0.1516042 | 0.27446773 | FALSE |
| CNDP2 | 4965 | 1.433568 | 0.1516956 | 0.27455066 | FALSE |
| DNAJC3-AS1 | 3410 | 1.433387 | 0.1517473 | 0.27459304 | FALSE |
| COX6C | 15141 | 1.433273 | 0.1517798 | 0.27459486 | FALSE |
| H2AFJ | 2383 | 1.432395 | 0.1520308 | 0.27501708 | FALSE |
| BAALC-AS2 | 1875 | 1.431513 | 0.1522832 | 0.27544186 | FALSE |
| DNAJC14 | 547 | 1.431373 | 0.1523233 | 0.27548224 | FALSE |
| BORCS5 | 65 | 1.431312 | 0.1523408 | 0.27548224 | FALSE |
| KRBOX4 | 8083 | 1.430586 | 0.1525489 | 0.27573769 | FALSE |
| ZZZ3 | 5765 | 1.430452 | 0.1525873 | 0.27576853 | FALSE |
| PRRC2B | 4392 | 1.430146 | 0.1526751 | 0.27589529 | FALSE |
| EMX2 | 9592 | 1.430007 | 0.152715 | 0.27589815 | FALSE |
| NT5C3B | 12953 | 1.429219 | 0.1529413 | 0.27621665 | FALSE |
| HIST1H2APS1 | 9705 | 1.428523 | 0.1531414 | 0.27648218 | FALSE |
| SUMO1P1 | 4181 | 1.428428 | 0.1531687 | 0.27649958 | FALSE |
| AZIN1 | 8612 | 1.428255 | 0.1532185 | 0.27655749 | FALSE |
| TNFSF12 | 10480 | 1.427964 | 0.1533022 | 0.27664633 | FALSE |
| GPRC5C | 2359 | 1.427821 | 0.1533434 | 0.27668712 | FALSE |
| DNAH7 | 15409 | 1.426046 | 0.1538551 | 0.27748226 | FALSE |
| RSRC2 | 4104 | 1.425603 | 0.153983 | 0.27764888 | FALSE |
| CCL24 | 2603 | 1.424665 | 0.1542541 | 0.27800942 | FALSE |
| BLM | 7317 | 1.424558 | 0.154285 | 0.27803315 | FALSE |
| SLC39A6 | 1776 | 1.424447 | 0.1543171 | 0.2780358 | FALSE |
| EXOSC8 | 7992 | 1.424342 | 0.1543475 | 0.2780358 | FALSE |
| SSUH2 | 15623 | 1.424307 | 0.1543576 | 0.2780358 | FALSE |
| HMGA1 | 5289 | 1.423734 | 0.1545235 | 0.27827044 | FALSE |
| TRMT2A | 8227 | 1.423334 | 0.1546394 | 0.27844702 | FALSE |
| ANKRD35 | 5305 | 1.423096 | 0.1547083 | 0.27853913 | FALSE |
| PIEZO1 | 7244 | 1.422999 | 0.1547364 | 0.27853917 | FALSE |
| ARFIP2 | 7505 | 1.422849 | 0.1547799 | 0.27857181 | FALSE |
| HIST1H2AG | 10824 | 1.422506 | 0.1548794 | 0.27871876 | FALSE |
| LRRC75A-AS1 | 11389 | 1.422394 | 0.1549119 | 0.27874514 | FALSE |
| MRPS15 | 4963 | 1.421551 | 0.1551566 | 0.27900799 | FALSE |
| MRGPRE | 1437 | 1.421522 | 0.1551651 | 0.27900799 | FALSE |
| DAB2 | 859 | 1.420719 | 0.1553985 | 0.27933129 | FALSE |
| SEMA3A | 8628 | 1.420429 | 0.1554828 | 0.27941867 | FALSE |
| HLA-DPB2 | 14646 | 1.420091 | 0.1555812 | 0.2795633 | FALSE |
| KHDRBS1 | 14838 | 1.418454 | 0.1560583 | 0.28025948 | FALSE |
| MRO | 2010 | 1.418293 | 0.1561052 | 0.28027945 | FALSE |
| CROCC | 882 | 1.417521 | 0.1563307 | 0.2805553 | FALSE |
| NDUFA6 | 12035 | 1.415038 | 0.1570574 | 0.28166544 | FALSE |
| SLC25A48 | 12939 | 1.414843 | 0.1571145 | 0.28173566 | FALSE |
| GAS8-AS1 | 12202 | 1.414207 | 0.1573011 | 0.28200037 | FALSE |
| OR13A1 | 1526 | 1.414119 | 0.157327 | 0.28200037 | FALSE |
| G6PC | 1120 | 1.413842 | 0.1574083 | 0.28206831 | FALSE |
| UBR7 | 1641 | 1.41354 | 0.157497 | 0.28217101 | FALSE |
| ALG9 | 2133 | 1.413404 | 0.157537 | 0.28220184 | FALSE |
| NXPH4 | 3700 | 1.412906 | 0.1576834 | 0.28243172 | FALSE |
| PROKR1 | 9366 | 1.412682 | 0.1577492 | 0.28248499 | FALSE |
| LINC01993 | 12673 | 1.411921 | 0.1579732 | 0.28278889 | FALSE |
| C15orf65 | 9095 | 1.411618 | 0.1580625 | 0.28281911 | FALSE |
| USP39 | 11651 | 1.411129 | 0.1582066 | 0.28300881 | FALSE |
| ZCWPW2 | 11704 | 1.410818 | 0.1582983 | 0.28311144 | FALSE |
| OR10Q1 | 14128 | 1.409943 | 0.1585565 | 0.28348813 | FALSE |
| CDA | 13792 | 1.40992 | 0.1585633 | 0.28348813 | FALSE |
| DAB1 | 1459 | 1.409793 | 0.1586008 | 0.28352096 | FALSE |
| CD247 | 13244 | 1.409735 | 0.1586179 | 0.28352096 | FALSE |
| TUSC2 | 1884 | 1.409398 | 0.1587175 | 0.28361856 | FALSE |
| RNF187 | 1510 | 1.409366 | 0.158727 | 0.28361856 | FALSE |
| KCTD10 | 2191 | 1.409264 | 0.1587571 | 0.28364001 | FALSE |
| TPM2 | 643 | 1.408669 | 0.1589331 | 0.28387956 | FALSE |
| CHST13 | 8508 | 1.407752 | 0.1592045 | 0.2842012 | FALSE |
| EFCC1 | 5004 | 1.407711 | 0.1592167 | 0.2842012 | FALSE |
| PHB | 2499 | 1.407598 | 0.1592501 | 0.2842285 | FALSE |
| HLA-DRB3 | 6067 | 1.407134 | 0.1593877 | 0.28437654 | FALSE |
| FAM212B | 9834 | 1.406807 | 0.1594846 | 0.28451708 | FALSE |
| GFM2 | 11681 | 1.406475 | 0.1595831 | 0.28466031 | FALSE |
| PPOX | 9180 | 1.406129 | 0.1596858 | 0.28474603 | FALSE |
| CILP2 | 15183 | 1.403799 | 0.1603787 | 0.28591634 | FALSE |
| TFDP2 | 177 | 1.403305 | 0.1605259 | 0.28614613 | FALSE |
| ATP6V0E2 | 11317 | 1.403076 | 0.1605942 | 0.28620255 | FALSE |
| MAZ | 1416 | 1.402884 | 0.1606514 | 0.28623933 | FALSE |
| CPSF4 | 4564 | 1.402427 | 0.1607878 | 0.28636287 | FALSE |
| ACTR1A | 3378 | 1.402241 | 0.1608433 | 0.28639702 | FALSE |
| RUSC1-AS1 | 11478 | 1.401863 | 0.1609562 | 0.28652103 | FALSE |
| SSPN | 12935 | 1.401226 | 0.1611465 | 0.2868272 | FALSE |
| LINC01551 | 13004 | 1.400604 | 0.1613325 | 0.28709293 | FALSE |
| LRRC2 | 375 | 1.400041 | 0.161501 | 0.2872947 | FALSE |
| FANK1 | 15307 | 1.399807 | 0.1615711 | 0.28735396 | FALSE |
| ASB2 | 14043 | 1.39954 | 0.1616511 | 0.28740826 | FALSE |
| ZNRF3 | 6998 | 1.399521 | 0.1616568 | 0.28740826 | FALSE |
| PFN1P2 | 10319 | 1.399264 | 0.1617338 | 0.28751251 | FALSE |
| PI3 | 7724 | 1.398918 | 0.1618376 | 0.2876292 | FALSE |
| COL24A1 | 3952 | 1.39888 | 0.161849 | 0.2876292 | FALSE |
| RNF128 | 2856 | 1.398711 | 0.1618997 | 0.28767646 | FALSE |
| KRTAP20-1 | 1593 | 1.39834 | 0.162011 | 0.28784155 | FALSE |
| GLB1L | 10163 | 1.396861 | 0.1624554 | 0.28842603 | FALSE |
| GDF5OS | 1971 | 1.396785 | 0.1624782 | 0.28842603 | FALSE |
| PPP2R5C | 11723 | 1.396754 | 0.1624876 | 0.28842603 | FALSE |
| COL9A1 | 3951 | 1.396528 | 0.1625556 | 0.28848121 | FALSE |
| SGTA | 5271 | 1.396118 | 0.162679 | 0.28866745 | FALSE |
| TALDO1 | 1745 | 1.395358 | 0.1629079 | 0.2890081 | FALSE |
| TOMM22 | 9018 | 1.395168 | 0.1629652 | 0.2890769 | FALSE |
| ARNT2 | 10522 | 1.39497 | 0.1630249 | 0.28914999 | FALSE |
| CHCHD7 | 5978 | 1.393546 | 0.1634547 | 0.28979626 | FALSE |
| PRPSAP2 | 5983 | 1.393456 | 0.1634819 | 0.28979626 | FALSE |
| GOLGA3 | 10110 | 1.393341 | 0.1635167 | 0.289825 | FALSE |
| HIST1H2AC | 8069 | 1.393038 | 0.1636083 | 0.28995449 | FALSE |
| TMOD3 | 11729 | 1.392367 | 0.1638113 | 0.29018266 | FALSE |
| SCGB3A1 | 7889 | 1.391218 | 0.1641593 | 0.29070036 | FALSE |
| KHDC1L | 2562 | 1.390842 | 0.1642733 | 0.29085947 | FALSE |
| GEMIN4 | 4230 | 1.390799 | 0.1642864 | 0.29085947 | FALSE |
| KIRREL3 | 9193 | 1.389378 | 0.1647178 | 0.29149128 | FALSE |
| KPTN | 10100 | 1.388311 | 0.1650424 | 0.29199423 | FALSE |
| NFU1 | 9412 | 1.387285 | 0.1653549 | 0.29235385 | FALSE |
| USP27X-AS1 | 12409 | 1.386838 | 0.1654912 | 0.29244832 | FALSE |
| KLHDC4 | 4631 | 1.386724 | 0.1655259 | 0.29244834 | FALSE |
| EXOC5 | 8348 | 1.386619 | 0.165558 | 0.29244834 | FALSE |
| SLC1A2 | 11497 | 1.385974 | 0.1657548 | 0.29271936 | FALSE |
| HBA1 | 11010 | 1.385907 | 0.1657753 | 0.29271936 | FALSE |
| RPL37 | 12150 | 1.385672 | 0.1658471 | 0.29279361 | FALSE |
| WDR44 | 9695 | 1.383731 | 0.1664408 | 0.29370918 | FALSE |
| NCOA1 | 5247 | 1.382898 | 0.1666961 | 0.29409331 | FALSE |
| EPB41L4B | 3762 | 1.382835 | 0.1667155 | 0.29409421 | FALSE |
| COQ8B | 3316 | 1.380483 | 0.167438 | 0.29530212 | FALSE |
| ITGB5 | 262 | 1.379811 | 0.1676448 | 0.29555856 | FALSE |
| ZFR | 9376 | 1.379107 | 0.1678618 | 0.29588262 | FALSE |
| MORC4 | 2075 | 1.378896 | 0.1679268 | 0.29593056 | FALSE |
| KIF17 | 5890 | 1.377973 | 0.1682116 | 0.29634965 | FALSE |
| GDNF | 4908 | 1.37785 | 0.1682496 | 0.29635793 | FALSE |
| CNPY3 | 7560 | 1.377803 | 0.1682641 | 0.29635793 | FALSE |
| DENND2C | 11304 | 1.377399 | 0.1683889 | 0.29651093 | FALSE |
| ARHGAP27P1-BPTFP1-KPNA2P3 | 14416 | 1.377162 | 0.1684622 | 0.29657309 | FALSE |
| PCED1A | 13303 | 1.376753 | 0.1685886 | 0.29672889 | FALSE |
| C16orf70 | 5667 | 1.374992 | 0.1691339 | 0.29758062 | FALSE |
| PIN1P1 | 1058 | 1.374792 | 0.1691959 | 0.29759677 | FALSE |
| FHL3 | 968 | 1.374293 | 0.1693507 | 0.29776854 | FALSE |
| BANF1 | 5889 | 1.373877 | 0.1694799 | 0.29786393 | FALSE |
| P3H2 | 2177 | 1.373853 | 0.1694873 | 0.29786393 | FALSE |
| EIF5B | 15064 | 1.37375 | 0.1695193 | 0.29786393 | FALSE |
| RNF215 | 10911 | 1.373154 | 0.1697045 | 0.29815578 | FALSE |
| LEPROT | 8992 | 1.372883 | 0.1697887 | 0.29827027 | FALSE |
| PPP5C | 3593 | 1.372585 | 0.1698814 | 0.29839954 | FALSE |
| PARP8 | 11394 | 1.371999 | 0.1700637 | 0.29858565 | FALSE |
| ZFYVE21 | 4359 | 1.371676 | 0.1701643 | 0.29872868 | FALSE |
| AK6 | 4647 | 1.370946 | 0.1703918 | 0.29909442 | FALSE |
| ESPL1 | 6598 | 1.370389 | 0.1705655 | 0.29933212 | FALSE |
| EPS8 | 8758 | 1.369935 | 0.1707072 | 0.29954715 | FALSE |
| EMX2OS | 13282 | 1.369185 | 0.1709415 | 0.29981518 | FALSE |
| RUFY3 | 1060 | 1.369139 | 0.1709558 | 0.29981518 | FALSE |
| PLGRKT | 9571 | 1.368421 | 0.1711803 | 0.30011187 | FALSE |
| ZFHX4-AS1 | 903 | 1.368351 | 0.1712022 | 0.30011187 | FALSE |
| PUS3 | 966 | 1.36818 | 0.1712557 | 0.30011187 | FALSE |
| CRAT | 6042 | 1.368107 | 0.1712786 | 0.30011187 | FALSE |
| FCHSD2 | 1308 | 1.367459 | 0.1714815 | 0.30037425 | FALSE |
| TAF9 | 8946 | 1.3674 | 0.1715 | 0.30037425 | FALSE |
| NRP1 | 12945 | 1.366393 | 0.1718156 | 0.30074952 | FALSE |
| FXYD1 | 10370 | 1.366067 | 0.1719179 | 0.3008612 | FALSE |
| ZNF91 | 13368 | 1.365806 | 0.1719999 | 0.30096851 | FALSE |
| SLC25A44 | 490 | 1.365749 | 0.1720178 | 0.30096851 | FALSE |
| KLF7 | 13485 | 1.365287 | 0.1721629 | 0.30118868 | FALSE |
| IPO13 | 13600 | 1.365117 | 0.1722163 | 0.30124842 | FALSE |
| APOL4 | 10726 | 1.36499 | 0.1722562 | 0.30128452 | FALSE |
| GALNT10 | 5909 | 1.364842 | 0.1723027 | 0.30128566 | FALSE |
| ZNF613 | 3937 | 1.364842 | 0.1723027 | 0.30128566 | FALSE |
| NFKBIL1 | 13773 | 1.364263 | 0.1724848 | 0.30146518 | FALSE |
| TLE6 | 7429 | 1.36386 | 0.1726116 | 0.30160251 | FALSE |
| CLYBL | 1146 | 1.363679 | 0.1726686 | 0.30163465 | FALSE |
| FMNL1 | 13432 | 1.363185 | 0.1728242 | 0.30177158 | FALSE |
| THAP4 | 5408 | 1.362832 | 0.1729355 | 0.30189839 | FALSE |
| LGALS1 | 8096 | 1.362059 | 0.1731793 | 0.30218902 | FALSE |
| IFI27L1 | 11086 | 1.361792 | 0.1732635 | 0.30226859 | FALSE |
| ZSCAN2 | 5458 | 1.361079 | 0.1734887 | 0.30259391 | FALSE |
| RETNLB | 8715 | 1.360905 | 0.1735437 | 0.30265605 | FALSE |
| CACNG4 | 2750 | 1.360325 | 0.1737271 | 0.30290829 | FALSE |
| ALOX15B | 1083 | 1.36002 | 0.1738236 | 0.30300895 | FALSE |
| CCT8 | 6652 | 1.359922 | 0.1738546 | 0.30302922 | FALSE |
| CHRNB2 | 7728 | 1.359517 | 0.1739828 | 0.30321889 | FALSE |
| KRTAP6-2 | 10504 | 1.35911 | 0.1741117 | 0.30337026 | FALSE |
| FOXG1 | 6007 | 1.358961 | 0.1741589 | 0.30337662 | FALSE |
| ZNF449 | 1739 | 1.358818 | 0.1742043 | 0.30340189 | FALSE |
| COX14 | 6795 | 1.358661 | 0.174254 | 0.30345476 | FALSE |
| HLA-C | 4912 | 1.358244 | 0.1743863 | 0.30357353 | FALSE |
| SNN | 13324 | 1.357007 | 0.174779 | 0.30418167 | FALSE |
| KCTD6 | 3961 | 1.356446 | 0.1749573 | 0.30424607 | FALSE |
| LAMC3 | 8618 | 1.356433 | 0.1749614 | 0.30424607 | FALSE |
| METRNL | 7181 | 1.354794 | 0.1754832 | 0.30505158 | FALSE |
| P4HB | 4019 | 1.3545 | 0.1755769 | 0.30518055 | FALSE |
| GPR39 | 5060 | 1.354141 | 0.1756914 | 0.30534559 | FALSE |
| SAA1 | 8238 | 1.353431 | 0.175918 | 0.30567142 | FALSE |
| FBRSL1 | 4568 | 1.35328 | 0.1759662 | 0.30572122 | FALSE |
| NCF1C | 7694 | 1.35304 | 0.1760429 | 0.30578643 | FALSE |
| METTL7A | 11488 | 1.352711 | 0.176148 | 0.30593504 | FALSE |
| COL8A2 | 3122 | 1.352292 | 0.1762819 | 0.30606567 | FALSE |
| ARPC4 | 3324 | 1.352067 | 0.1763539 | 0.30615661 | FALSE |
| AACS | 15087 | 1.351827 | 0.1764307 | 0.3061879 | FALSE |
| TLX1 | 2065 | 1.351325 | 0.1765913 | 0.30636472 | FALSE |
| PPP1R1B | 2489 | 1.350095 | 0.1769855 | 0.30689318 | FALSE |
| ADAM7 | 6464 | 1.350027 | 0.1770073 | 0.30689318 | FALSE |
| FUT7 | 12506 | 1.350007 | 0.1770137 | 0.30689318 | FALSE |
| BTRC | 13768 | 1.349814 | 0.1770757 | 0.30693244 | FALSE |
| TAAR5 | 14022 | 1.349266 | 0.1772515 | 0.30715518 | FALSE |
| GFER | 8561 | 1.34923 | 0.1772631 | 0.30715518 | FALSE |
| IGSF23 | 13850 | 1.348766 | 0.1774121 | 0.3073453 | FALSE |
| ABHD2 | 5301 | 1.348625 | 0.1774574 | 0.3073834 | FALSE |
| ENDOU | 6932 | 1.348349 | 0.1775462 | 0.30744119 | FALSE |
| C19orf57 | 13119 | 1.347514 | 0.1778147 | 0.30783809 | FALSE |
| YIPF1 | 8093 | 1.346944 | 0.1779983 | 0.30812169 | FALSE |
| SYT13 | 10593 | 1.346744 | 0.1780627 | 0.30819908 | FALSE |
| COL4A1 | 7210 | 1.346626 | 0.1781007 | 0.30823076 | FALSE |
| MRPS35 | 3441 | 1.345767 | 0.1783777 | 0.30867591 | FALSE |
| TSFM | 12329 | 1.345364 | 0.1785077 | 0.30886675 | FALSE |
| HSPA5 | 7509 | 1.344617 | 0.178749 | 0.30918149 | FALSE |
| RIN3 | 8479 | 1.344227 | 0.178875 | 0.30933106 | FALSE |
| CYB5A | 4399 | 1.343862 | 0.178993 | 0.30950092 | FALSE |
| SEC63 | 7456 | 1.343126 | 0.1792312 | 0.30984419 | FALSE |
| PPARA | 4202 | 1.343003 | 0.179271 | 0.3098698 | FALSE |
| FOXJ3 | 11589 | 1.342929 | 0.179295 | 0.3098698 | FALSE |
| KHDC1 | 2188 | 1.342743 | 0.1793552 | 0.3098698 | FALSE |
| RAB8A | 11545 | 1.342713 | 0.1793649 | 0.3098698 | FALSE |
| RTL10 | 13625 | 1.34232 | 0.1794923 | 0.31005553 | FALSE |
| CREG1 | 423 | 1.341214 | 0.179851 | 0.31060654 | FALSE |
| TGOLN2 | 6091 | 1.340958 | 0.1799341 | 0.31071574 | FALSE |
| ARSE | 1752 | 1.339989 | 0.1802489 | 0.31119066 | FALSE |
| TSPO | 5699 | 1.33879 | 0.180639 | 0.3117609 | FALSE |
| STXBP3 | 581 | 1.338092 | 0.1808664 | 0.31208446 | FALSE |
| RETREG2 | 12279 | 1.33774 | 0.1809812 | 0.31224801 | FALSE |
| NR2C2AP | 14490 | 1.33758 | 0.1810334 | 0.31226911 | FALSE |
| B4GALT1 | 14548 | 1.337214 | 0.1811528 | 0.31236873 | FALSE |
| CGNL1 | 6546 | 1.336586 | 0.1813578 | 0.31265623 | FALSE |
| CORO1B | 3428 | 1.336499 | 0.1813862 | 0.31267073 | FALSE |
| FSCN2 | 6304 | 1.335969 | 0.1815594 | 0.31293475 | FALSE |
| RPS26 | 6683 | 1.334989 | 0.1818799 | 0.31340769 | FALSE |
| ALDH1L1 | 6274 | 1.334885 | 0.181914 | 0.31340769 | FALSE |
| SRD5A3 | 14707 | 1.333862 | 0.1822491 | 0.31384666 | FALSE |
| EPHX2 | 4598 | 1.333509 | 0.1823648 | 0.31397679 | FALSE |
| BAMBI | 10178 | 1.33308 | 0.1825056 | 0.31418448 | FALSE |
| OGG1 | 12651 | 1.33238 | 0.1827354 | 0.31451082 | FALSE |
| UBA1 | 5986 | 1.332237 | 0.1827823 | 0.31455703 | FALSE |
| CRYBB1 | 1851 | 1.331772 | 0.1829351 | 0.31475069 | FALSE |
| RGL1 | 11162 | 1.331681 | 0.182965 | 0.31476751 | FALSE |
| ICAM4 | 15081 | 1.331285 | 0.1830953 | 0.31495687 | FALSE |
| NCKAP1 | 4972 | 1.330552 | 0.1833365 | 0.3152199 | FALSE |
| PSMA1 | 8695 | 1.330514 | 0.183349 | 0.3152199 | FALSE |
| NPL | 5187 | 1.328453 | 0.1840285 | 0.31617953 | FALSE |
| VAMP5 | 15381 | 1.328332 | 0.1840685 | 0.31621342 | FALSE |
| LLPH | 11302 | 1.327079 | 0.1844826 | 0.31670676 | FALSE |
| ARSK | 869 | 1.326102 | 0.1848059 | 0.31713182 | FALSE |
| SEC22B | 2912 | 1.325664 | 0.184951 | 0.31731116 | FALSE |
| PPIAP30 | 3726 | 1.325045 | 0.1851562 | 0.31754431 | FALSE |
| ARHGAP1 | 8896 | 1.324293 | 0.1854058 | 0.31788201 | FALSE |
| TOGARAM2 | 8581 | 1.322774 | 0.1859105 | 0.31867758 | FALSE |
| MYORG | 7025 | 1.322188 | 0.1861056 | 0.31890696 | FALSE |
| CMIP | 12963 | 1.320768 | 0.1865787 | 0.31961268 | FALSE |
| UMOD | 11977 | 1.320581 | 0.1866411 | 0.31964075 | FALSE |
| TNC | 7265 | 1.320352 | 0.1867175 | 0.3197103 | FALSE |
| LHX3 | 6365 | 1.319941 | 0.1868547 | 0.31991017 | FALSE |
| C1QTNF3 | 14407 | 1.319676 | 0.1869432 | 0.32002664 | FALSE |
| ZNF648 | 2882 | 1.319512 | 0.186998 | 0.32008538 | FALSE |
| PARVB | 7778 | 1.319394 | 0.1870374 | 0.32011781 | FALSE |
| CYP4X1 | 14009 | 1.319024 | 0.1871611 | 0.32025934 | FALSE |
| MX2 | 5778 | 1.318606 | 0.1873009 | 0.32034323 | FALSE |
| F10 | 1408 | 1.317712 | 0.1876001 | 0.32065952 | FALSE |
| PSMG1 | 4452 | 1.317452 | 0.1876872 | 0.32066813 | FALSE |
| KDELR1 | 10740 | 1.316825 | 0.1878973 | 0.32095699 | FALSE |
| GLIS3-AS1 | 11406 | 1.316598 | 0.1879734 | 0.32104435 | FALSE |
| DUSP9 | 13464 | 1.315344 | 0.1883943 | 0.3216654 | FALSE |
| CHRNA7 | 3544 | 1.314991 | 0.1885129 | 0.32179762 | FALSE |
| PLBD1 | 14088 | 1.314991 | 0.1885129 | 0.32179762 | FALSE |
| LYPD2 | 7669 | 1.314343 | 0.1887308 | 0.32212905 | FALSE |
| RB1 | 3905 | 1.314291 | 0.1887483 | 0.32212905 | FALSE |
| EDA | 12215 | 1.313421 | 0.1890411 | 0.3224528 | FALSE |
| CYP2A13 | 7742 | 1.31302 | 0.1891762 | 0.32261282 | FALSE |
| MRPS34 | 5835 | 1.312817 | 0.1892446 | 0.32269428 | FALSE |
| THY1 | 8962 | 1.312208 | 0.18945 | 0.32297397 | FALSE |
| THADA | 3245 | 1.310767 | 0.1899365 | 0.32369751 | FALSE |
| YPEL5 | 8661 | 1.310508 | 0.190024 | 0.3237859 | FALSE |
| SHISAL2A | 10559 | 1.310232 | 0.1901174 | 0.32386625 | FALSE |
| OR2A2 | 8839 | 1.310229 | 0.1901184 | 0.32386625 | FALSE |
| PTPN9 | 3418 | 1.310021 | 0.1901887 | 0.32393683 | FALSE |
| NDUFB6 | 113 | 1.309984 | 0.1902012 | 0.32393683 | FALSE |
| NDUFV1 | 236 | 1.309785 | 0.1902686 | 0.32396003 | FALSE |
| SMUG1 | 5216 | 1.30976 | 0.190277 | 0.32396003 | FALSE |
| SWAP70 | 3602 | 1.309192 | 0.1904693 | 0.32421678 | FALSE |
| CLIC6 | 2781 | 1.308973 | 0.1905435 | 0.32430773 | FALSE |
| CENPM | 1106 | 1.308793 | 0.1906045 | 0.32437621 | FALSE |
| TMEM184B | 6645 | 1.308428 | 0.1907282 | 0.32451607 | FALSE |
| AP2M1 | 11528 | 1.308172 | 0.190815 | 0.32460694 | FALSE |
| RCBTB2 | 5055 | 1.307757 | 0.1909557 | 0.32479721 | FALSE |
| DIEXF | 9525 | 1.307038 | 0.1911998 | 0.32517696 | FALSE |
| PRSS8 | 5001 | 1.30692 | 0.1912399 | 0.32520973 | FALSE |
| CACNA1B | 10099 | 1.306794 | 0.1912827 | 0.32522274 | FALSE |
| STAT5A | 7324 | 1.306775 | 0.1912891 | 0.32522274 | FALSE |
| SLAMF7 | 1316 | 1.30602 | 0.1915458 | 0.32558821 | FALSE |
| C2orf50 | 195 | 1.305836 | 0.1916083 | 0.32565918 | FALSE |
| PAK4 | 6163 | 1.304994 | 0.1918949 | 0.32611075 | FALSE |
| COX7A2 | 5901 | 1.304848 | 0.1919446 | 0.32615979 | FALSE |
| MRPS18C | 162 | 1.304545 | 0.1920478 | 0.32626426 | FALSE |
| LAMB2 | 8571 | 1.304269 | 0.1921419 | 0.32637569 | FALSE |
| USP30-AS1 | 12949 | 1.304076 | 0.1922077 | 0.32642939 | FALSE |
| TMEM116 | 11674 | 1.303762 | 0.1923147 | 0.32651987 | FALSE |
| USP46-AS1 | 2024 | 1.302943 | 0.1925942 | 0.3269438 | FALSE |
| BAG1 | 10719 | 1.302533 | 0.1927342 | 0.32711162 | FALSE |
| TRMT5 | 12801 | 1.302261 | 0.1928272 | 0.32719714 | FALSE |
| CSF1 | 10431 | 1.301741 | 0.1930049 | 0.3274277 | FALSE |
| ANKRD54 | 11047 | 1.30148 | 0.1930942 | 0.32750805 | FALSE |
| GPS1 | 327 | 1.301353 | 0.1931377 | 0.32754621 | FALSE |
| ANO7 | 9016 | 1.301222 | 0.1931825 | 0.32758669 | FALSE |
| FEZF2 | 1071 | 1.300722 | 0.1933536 | 0.32784135 | FALSE |
| SPRYD3 | 2172 | 1.300603 | 0.1933944 | 0.32787489 | FALSE |
| DRD4 | 6658 | 1.300292 | 0.1935009 | 0.32801992 | FALSE |
| TUBG1 | 14118 | 1.298916 | 0.1939728 | 0.32871286 | FALSE |
| UBE2I | 11745 | 1.297786 | 0.1943609 | 0.32920322 | FALSE |
| PXN-AS1 | 13486 | 1.297482 | 0.1944654 | 0.32929185 | FALSE |
| NTRK3 | 12950 | 1.297439 | 0.1944802 | 0.32929185 | FALSE |
| LOC439933 | 4649 | 1.297431 | 0.1944829 | 0.32929185 | FALSE |
| COMP | 3839 | 1.296959 | 0.1946453 | 0.32952742 | FALSE |
| CASTOR2 | 6763 | 1.296493 | 0.1948057 | 0.32965981 | FALSE |
| SDK2 | 13426 | 1.296104 | 0.1949397 | 0.32978176 | FALSE |
| CYP7B1 | 13225 | 1.2961 | 0.194941 | 0.32978176 | FALSE |
| NDOR1 | 14255 | 1.295236 | 0.1952388 | 0.33021407 | FALSE |
| CLTA | 3027 | 1.295151 | 0.1952681 | 0.33022792 | FALSE |
| CEP112 | 11871 | 1.295056 | 0.1953009 | 0.33024761 | FALSE |
| LOC145694 | 12894 | 1.294767 | 0.1954006 | 0.33038048 | FALSE |
| FBXO17 | 3510 | 1.294599 | 0.1954586 | 0.33044277 | FALSE |
| GBE1 | 5238 | 1.294243 | 0.1955815 | 0.33057832 | FALSE |
| LOC101928370 | 13888 | 1.294183 | 0.1956022 | 0.33057832 | FALSE |
| KLHL12 | 11598 | 1.293369 | 0.1958835 | 0.33092489 | FALSE |
| BTBD17 | 2897 | 1.293322 | 0.1958997 | 0.33092489 | FALSE |
| MEGF9 | 7067 | 1.293266 | 0.1959191 | 0.33092489 | FALSE |
| ACBD4 | 8336 | 1.293061 | 0.19599 | 0.33098316 | FALSE |
| DNAJB6 | 5791 | 1.290365 | 0.196924 | 0.33227069 | FALSE |
| OPA1 | 2134 | 1.290275 | 0.1969552 | 0.33227069 | FALSE |
| LRRTM2 | 3524 | 1.290058 | 0.1970305 | 0.33232839 | FALSE |
| GTPBP3 | 4796 | 1.289808 | 0.1971173 | 0.3324202 | FALSE |
| PIAS3 | 739 | 1.289695 | 0.1971566 | 0.33243458 | FALSE |
| ME2 | 2471 | 1.289661 | 0.1971684 | 0.33243458 | FALSE |
| URB1 | 5719 | 1.28897 | 0.1974085 | 0.33280355 | FALSE |
| ATP6V1A | 3877 | 1.288559 | 0.1975514 | 0.3330086 | FALSE |
| MAPKAPK2 | 13087 | 1.288302 | 0.1976409 | 0.3331234 | FALSE |
| TMEM198 | 13802 | 1.287521 | 0.1979128 | 0.33340195 | FALSE |
| LCE1A | 6906 | 1.286547 | 0.1982522 | 0.33390186 | FALSE |
| CDC26 | 14991 | 1.28614 | 0.1983942 | 0.33403302 | FALSE |
| BPNT1 | 15394 | 1.285322 | 0.1986798 | 0.33429782 | FALSE |
| KPNB1 | 7376 | 1.284927 | 0.1988178 | 0.33449404 | FALSE |
| INTS7 | 12464 | 1.284596 | 0.1989335 | 0.33465268 | FALSE |
| COX8A | 6896 | 1.284386 | 0.1990069 | 0.33474019 | FALSE |
| PAPSS1 | 10014 | 1.284042 | 0.1991273 | 0.33490656 | FALSE |
| NYAP1 | 313 | 1.28358 | 0.199289 | 0.33514245 | FALSE |
| NAGLU | 7079 | 1.282974 | 0.1995012 | 0.33542719 | FALSE |
| VPS28 | 7454 | 1.282605 | 0.1996305 | 0.33560851 | FALSE |
| GREM2 | 9681 | 1.282044 | 0.1998272 | 0.3359031 | FALSE |
| ZNF45 | 2966 | 1.281794 | 0.1999149 | 0.3360144 | FALSE |
| ZMAT5 | 5600 | 1.279303 | 0.2007904 | 0.33734788 | FALSE |
| ZHX2 | 5797 | 1.279058 | 0.2008766 | 0.33744943 | FALSE |
| GPR153 | 7211 | 1.278452 | 0.2010901 | 0.33770449 | FALSE |
| NCAPG2 | 14998 | 1.278045 | 0.2012336 | 0.33782986 | FALSE |
| YWHAQ | 14315 | 1.277704 | 0.2013538 | 0.33796051 | FALSE |
| FAM111A-DT | 8824 | 1.276491 | 0.201782 | 0.33857015 | FALSE |
| RNASE13 | 3267 | 1.276393 | 0.2018166 | 0.33859191 | FALSE |
| HIST1H4K | 5629 | 1.275595 | 0.2020987 | 0.33895605 | FALSE |
| COPS8 | 6432 | 1.275285 | 0.2022084 | 0.33906722 | FALSE |
| MYO10 | 3208 | 1.275128 | 0.2022639 | 0.339124 | FALSE |
| RPUSD3 | 11375 | 1.274248 | 0.2025755 | 0.33943692 | FALSE |
| RARRES2 | 14465 | 1.274233 | 0.2025809 | 0.33943692 | FALSE |
| ZNF516 | 1150 | 1.273915 | 0.2026935 | 0.33951581 | FALSE |
| POLE2 | 5459 | 1.273608 | 0.2028024 | 0.33958971 | FALSE |
| C2orf70 | 7194 | 1.270932 | 0.2037528 | 0.34092563 | FALSE |
| EIF3IP1 | 13241 | 1.270616 | 0.2038653 | 0.34105984 | FALSE |
| EPHB3 | 9951 | 1.270521 | 0.2038991 | 0.34106085 | FALSE |
| TBC1D10C | 3777 | 1.269521 | 0.2042553 | 0.34143416 | FALSE |
| KCTD21 | 7361 | 1.269488 | 0.2042671 | 0.34143416 | FALSE |
| C2CD4A | 14442 | 1.269428 | 0.2042885 | 0.34143416 | FALSE |
| NCSTN | 1202 | 1.268158 | 0.2047415 | 0.34206541 | FALSE |
| DTWD1 | 10919 | 1.267996 | 0.2047994 | 0.3420909 | FALSE |
| BHLHE23 | 5051 | 1.267586 | 0.2049458 | 0.34226657 | FALSE |
| WASF2 | 12533 | 1.267579 | 0.2049483 | 0.34226657 | FALSE |
| TCEA2 | 1854 | 1.26751 | 0.204973 | 0.34227118 | FALSE |
| ZNF624 | 12069 | 1.26731 | 0.2050445 | 0.34234678 | FALSE |
| IER5 | 23 | 1.26722 | 0.2050766 | 0.34234678 | FALSE |
| DYRK2 | 6360 | 1.267086 | 0.2051245 | 0.34234678 | FALSE |
| FAM171A2 | 7679 | 1.267084 | 0.2051253 | 0.34234678 | FALSE |
| OLFM3 | 13118 | 1.267077 | 0.2051278 | 0.34234678 | FALSE |
| SOCS7 | 577 | 1.266779 | 0.2052343 | 0.34248806 | FALSE |
| AKIP1 | 11494 | 1.266359 | 0.2053846 | 0.34262909 | FALSE |
| STXBP4 | 10417 | 1.265229 | 0.2057893 | 0.34323091 | FALSE |
| RPUSD4 | 3139 | 1.263462 | 0.2064232 | 0.34413822 | FALSE |
| HEPN1 | 2922 | 1.262845 | 0.2066449 | 0.34437171 | FALSE |
| POLR1C | 14793 | 1.262771 | 0.2066715 | 0.34437171 | FALSE |
| SLC37A1 | 60 | 1.262546 | 0.2067524 | 0.34446979 | FALSE |
| LOC541472 | 2933 | 1.262385 | 0.2068103 | 0.34452954 | FALSE |
| C6orf118 | 1466 | 1.261477 | 0.207137 | 0.3449702 | FALSE |
| NOL12 | 10432 | 1.261466 | 0.207141 | 0.3449702 | FALSE |
| FAM213A | 10514 | 1.260262 | 0.2075749 | 0.34550871 | FALSE |
| CFAP74 | 2456 | 1.259983 | 0.2076755 | 0.34563941 | FALSE |
| PALLD | 2340 | 1.259613 | 0.207809 | 0.34581381 | FALSE |
| DNAH9 | 8956 | 1.25932 | 0.2079148 | 0.34592716 | FALSE |
| TSPYL2 | 332 | 1.258877 | 0.2080748 | 0.34615651 | FALSE |
| NDUFS3 | 13013 | 1.258683 | 0.2081449 | 0.34619944 | FALSE |
| ARL8A | 12711 | 1.258474 | 0.2082204 | 0.34626581 | FALSE |
| SMPD3 | 14851 | 1.25845 | 0.2082291 | 0.34626581 | FALSE |
| KAAG1 | 1781 | 1.258299 | 0.2082836 | 0.34631974 | FALSE |
| TIGD5 | 10414 | 1.257578 | 0.2085444 | 0.34667761 | FALSE |
| SLC7A10 | 15405 | 1.25752 | 0.2085654 | 0.34667761 | FALSE |
| COX4I1 | 12869 | 1.255649 | 0.2092433 | 0.3475826 | FALSE |
| LOC100507557 | 5010 | 1.255448 | 0.2093162 | 0.34762985 | FALSE |
| GLIS2 | 2013 | 1.255328 | 0.2093597 | 0.34766522 | FALSE |
| GPR182 | 8052 | 1.254684 | 0.2095935 | 0.34794256 | FALSE |
| OR2M7 | 4808 | 1.251703 | 0.2106781 | 0.34955751 | FALSE |
| SIGMAR1 | 7931 | 1.251242 | 0.2108462 | 0.34976216 | FALSE |
| ACTN1 | 147 | 1.250953 | 0.2109516 | 0.34978858 | FALSE |
| CNTN4 | 10548 | 1.250565 | 0.2110932 | 0.34998626 | FALSE |
| GJA4 | 15204 | 1.250331 | 0.2111787 | 0.35005365 | FALSE |
| H2AFY | 13178 | 1.250147 | 0.2112459 | 0.35012791 | FALSE |
| HIST1H2BB | 1590 | 1.24923 | 0.211581 | 0.35049753 | FALSE |
| OSR2 | 10773 | 1.249109 | 0.2116252 | 0.35053368 | FALSE |
| REEP4 | 14545 | 1.248263 | 0.2119348 | 0.35090498 | FALSE |
| GPHB5 | 9882 | 1.248162 | 0.2119717 | 0.35092176 | FALSE |
| SPPL2B | 2457 | 1.246976 | 0.2124063 | 0.35149229 | FALSE |
| TGFBR3 | 5209 | 1.245923 | 0.2127927 | 0.35194538 | FALSE |
| PCDHAC2 | 7975 | 1.245463 | 0.2129616 | 0.35215029 | FALSE |
| KIAA0319 | 5454 | 1.244551 | 0.2132968 | 0.35266732 | FALSE |
| UHRF2 | 10689 | 1.244334 | 0.2133767 | 0.35276199 | FALSE |
| AMPH | 11886 | 1.244123 | 0.2134543 | 0.35285302 | FALSE |
| STEAP3 | 10770 | 1.243984 | 0.2135055 | 0.35290027 | FALSE |
| PBLD | 5200 | 1.242391 | 0.2140923 | 0.35368334 | FALSE |
| CAMSAP1 | 8047 | 1.241585 | 0.2143897 | 0.35406726 | FALSE |
| C3AR1 | 14772 | 1.240895 | 0.2146445 | 0.35440833 | FALSE |
| CST3 | 5479 | 1.240642 | 0.214738 | 0.35452525 | FALSE |
| SEC24D | 9183 | 1.240059 | 0.2149536 | 0.35480617 | FALSE |
| ATP5MPL | 8207 | 1.239537 | 0.2151467 | 0.35500125 | FALSE |
| EIF4G1 | 8790 | 1.23937 | 0.2152085 | 0.35500125 | FALSE |
| LDHB | 15028 | 1.23931 | 0.2152307 | 0.35500125 | FALSE |
| RGS8 | 5287 | 1.238643 | 0.2154777 | 0.35529622 | FALSE |
| C2CD4D | 12819 | 1.237987 | 0.2157209 | 0.35561151 | FALSE |
| LIMK2 | 7574 | 1.237377 | 0.2159472 | 0.35586399 | FALSE |
| ERGIC2 | 4152 | 1.236546 | 0.2162557 | 0.3561282 | FALSE |
| MRPL49 | 15304 | 1.236236 | 0.2163709 | 0.3562053 | FALSE |
| LOC101929122 | 11526 | 1.235517 | 0.2166382 | 0.3566078 | FALSE |
| PPBP | 3393 | 1.2353 | 0.2167189 | 0.35670311 | FALSE |
| NUDT1 | 6597 | 1.233754 | 0.2172946 | 0.3573497 | FALSE |
| PCMT1 | 2694 | 1.233216 | 0.2174952 | 0.35764198 | FALSE |
| PTOV1 | 8780 | 1.233128 | 0.217528 | 0.35765833 | FALSE |
| CCDC191 | 6999 | 1.232988 | 0.2175802 | 0.35770658 | FALSE |
| ST8SIA2 | 160 | 1.232886 | 0.2176183 | 0.35773153 | FALSE |
| AGAP2 | 10703 | 1.232488 | 0.2177668 | 0.35793808 | FALSE |
| HPS6 | 15053 | 1.232094 | 0.217914 | 0.35810461 | FALSE |
| ING5 | 14 | 1.231037 | 0.218309 | 0.3584442 | FALSE |
| IK | 9046 | 1.230925 | 0.2183509 | 0.35844586 | FALSE |
| HPDL | 13653 | 1.230293 | 0.2185874 | 0.35875873 | FALSE |
| TMEM209 | 3072 | 1.229617 | 0.2188406 | 0.35901326 | FALSE |
| C6orf89 | 10701 | 1.229518 | 0.2188777 | 0.35901326 | FALSE |
| CYB561D1 | 11414 | 1.229511 | 0.2188803 | 0.35901326 | FALSE |
| SH3D21 | 5571 | 1.229511 | 0.2188803 | 0.35901326 | FALSE |
| RPS6KA4 | 2850 | 1.229314 | 0.2189541 | 0.359059 | FALSE |
| TMEM131 | 2811 | 1.229222 | 0.2189886 | 0.35907788 | FALSE |
| TMIE | 2939 | 1.228895 | 0.2191112 | 0.35916588 | FALSE |
| RAD23B | 4788 | 1.22788 | 0.219492 | 0.35950983 | FALSE |
| MTX2 | 12848 | 1.22788 | 0.219492 | 0.35950983 | FALSE |
| GLIPR1L2 | 99 | 1.227801 | 0.2195217 | 0.35950983 | FALSE |
| FYCO1 | 5376 | 1.227784 | 0.2195281 | 0.35950983 | FALSE |
| C1orf21 | 8226 | 1.227723 | 0.219551 | 0.35950983 | FALSE |
| DNAL1 | 13790 | 1.22746 | 0.2196497 | 0.3596339 | FALSE |
| PSMA3 | 2875 | 1.227107 | 0.2197824 | 0.35970098 | FALSE |
| DGKH | 1546 | 1.226967 | 0.219835 | 0.35970893 | FALSE |
| CCNYL1 | 8910 | 1.226811 | 0.2198936 | 0.35970893 | FALSE |
| CFAP97D1 | 9125 | 1.226651 | 0.2199538 | 0.35971914 | FALSE |
| CCDC120 | 13889 | 1.226548 | 0.2199925 | 0.35974301 | FALSE |
| LOC101928659 | 7893 | 1.226286 | 0.2200911 | 0.35982887 | FALSE |
| ANKRD12 | 8344 | 1.226201 | 0.220123 | 0.35984352 | FALSE |
| TRIM62 | 12248 | 1.225786 | 0.2202792 | 0.36002352 | FALSE |
| LOC731157 | 13255 | 1.225507 | 0.2203843 | 0.36005939 | FALSE |
| HADHB | 12648 | 1.225483 | 0.2203933 | 0.36005939 | FALSE |
| TPRG1L | 1326 | 1.22529 | 0.220466 | 0.36006587 | FALSE |
| LHFPL6 | 680 | 1.225289 | 0.2204664 | 0.36006587 | FALSE |
| LOC148709 | 12431 | 1.225026 | 0.2205654 | 0.36019004 | FALSE |
| CXCL6 | 7861 | 1.223942 | 0.2209741 | 0.36074439 | FALSE |
| EIF1AY | 5053 | 1.223812 | 0.2210232 | 0.36078678 | FALSE |
| MID1 | 8200 | 1.221026 | 0.2220762 | 0.36193879 | FALSE |
| LINC00319 | 9659 | 1.220786 | 0.2221671 | 0.36204916 | FALSE |
| PRKCZ | 6588 | 1.220579 | 0.2222455 | 0.36210144 | FALSE |
| UBQLN2 | 10469 | 1.219237 | 0.2227543 | 0.36274138 | FALSE |
| EIF2S2 | 14095 | 1.219122 | 0.2227979 | 0.36277465 | FALSE |
| C7orf50 | 13063 | 1.218843 | 0.2229038 | 0.36290928 | FALSE |
| HOXB7 | 6551 | 1.218726 | 0.2229482 | 0.3629438 | FALSE |
| CPNE3 | 3202 | 1.218572 | 0.2230067 | 0.36300119 | FALSE |
| GBP3 | 10034 | 1.218176 | 0.2231571 | 0.36317041 | FALSE |
| CLDN14 | 8308 | 1.217901 | 0.2232616 | 0.36326484 | FALSE |
| TCEAL5 | 4268 | 1.217726 | 0.2233281 | 0.36333525 | FALSE |
| SIGLEC15 | 12032 | 1.217418 | 0.2234452 | 0.36348794 | FALSE |
| PSTK | 8212 | 1.217348 | 0.2234718 | 0.36349342 | FALSE |
| PPP6C | 10142 | 1.216764 | 0.223694 | 0.36377911 | FALSE |
| ITGA5 | 7933 | 1.21636 | 0.2238478 | 0.3639535 | FALSE |
| PPM1G | 15137 | 1.216027 | 0.2239746 | 0.36408397 | FALSE |
| LOC389831 | 5846 | 1.215095 | 0.2243299 | 0.36451351 | FALSE |
| C1GALT1 | 11099 | 1.214221 | 0.2246633 | 0.36499936 | FALSE |
| ELOVL2 | 9685 | 1.213575 | 0.2249101 | 0.36533863 | FALSE |
| COMMD1 | 6778 | 1.213072 | 0.2251023 | 0.36553694 | FALSE |
| AIM2 | 8556 | 1.212408 | 0.2253562 | 0.36587331 | FALSE |
| HGSNAT | 10846 | 1.212286 | 0.2254029 | 0.36589422 | FALSE |
| KCNK1 | 2048 | 1.212252 | 0.2254159 | 0.36589422 | FALSE |
| PDE5A | 13321 | 1.212042 | 0.2254963 | 0.36598668 | FALSE |
| ATP6V1F | 10609 | 1.211168 | 0.225831 | 0.36645385 | FALSE |
| CMKLR1 | 1149 | 1.210928 | 0.225923 | 0.36656505 | FALSE |
| FAM174A | 173 | 1.210553 | 0.2260668 | 0.36672219 | FALSE |
| RETN | 15078 | 1.210276 | 0.226173 | 0.36685646 | FALSE |
| EPHA8 | 13681 | 1.21013 | 0.226229 | 0.36687865 | FALSE |
| RPAP3 | 5783 | 1.210118 | 0.2262336 | 0.36687865 | FALSE |
| NT5C | 10891 | 1.209596 | 0.226434 | 0.36716544 | FALSE |
| COG6 | 6036 | 1.209477 | 0.2264796 | 0.36719012 | FALSE |
| TMEM30B | 5853 | 1.20891 | 0.2266974 | 0.36747831 | FALSE |
| PEX11G | 8021 | 1.208649 | 0.2267977 | 0.36752657 | FALSE |
| MAGEA6 | 6746 | 1.208494 | 0.2268573 | 0.36756996 | FALSE |
| SMYD5 | 4090 | 1.206568 | 0.2275986 | 0.36859507 | FALSE |
| TSTD3 | 2432 | 1.206426 | 0.2276533 | 0.3686455 | FALSE |
| MGST3 | 9012 | 1.205937 | 0.2278418 | 0.3687866 | FALSE |
| BAAT | 7223 | 1.205909 | 0.2278526 | 0.3687866 | FALSE |
| PSMD10 | 1549 | 1.205906 | 0.2278537 | 0.3687866 | FALSE |
| CANT1 | 7126 | 1.205326 | 0.2280775 | 0.36910302 | FALSE |
| GLT1D1 | 5336 | 1.205253 | 0.2281056 | 0.36910717 | FALSE |
| ENO3 | 12978 | 1.205197 | 0.2281273 | 0.36910717 | FALSE |
| DNAJC3 | 12798 | 1.204848 | 0.228262 | 0.36922665 | FALSE |
| PTPN18 | 437 | 1.204807 | 0.2282778 | 0.36922665 | FALSE |
| PPP2CB | 13438 | 1.204761 | 0.2282956 | 0.36922665 | FALSE |
| TOPBP1 | 12860 | 1.204208 | 0.2285092 | 0.36949058 | FALSE |
| SAP130 | 12163 | 1.204155 | 0.2285297 | 0.36949058 | FALSE |
| DTX3L | 9185 | 1.203826 | 0.2286568 | 0.36964025 | FALSE |
| KAT7 | 6439 | 1.203732 | 0.2286932 | 0.36964025 | FALSE |
| CASD1 | 4293 | 1.201331 | 0.2296228 | 0.37087444 | FALSE |
| FAM120A | 1857 | 1.201181 | 0.229681 | 0.37088225 | FALSE |
| LOC100132831 | 4503 | 1.201135 | 0.2296988 | 0.37088225 | FALSE |
| POLR2E | 1480 | 1.201022 | 0.2297427 | 0.37091398 | FALSE |
| REXO2 | 14052 | 1.200962 | 0.2297659 | 0.37091398 | FALSE |
| IQGAP3 | 1351 | 1.200181 | 0.2300691 | 0.37136495 | FALSE |
| CLIP3 | 9184 | 1.199698 | 0.2302567 | 0.37155266 | FALSE |
| SEZ6L | 12899 | 1.198754 | 0.2306236 | 0.372068 | FALSE |
| SLC9A7 | 13901 | 1.197645 | 0.2310552 | 0.372649 | FALSE |
| TNFSF13B | 2797 | 1.197519 | 0.2311043 | 0.37265126 | FALSE |
| CMC1 | 4046 | 1.196389 | 0.2315448 | 0.37313058 | FALSE |
| ADRM1 | 12503 | 1.196067 | 0.2316704 | 0.37324903 | FALSE |
| SFRP1 | 7856 | 1.195462 | 0.2319066 | 0.37347744 | FALSE |
| BLOC1S4 | 504 | 1.194974 | 0.2320972 | 0.37362418 | FALSE |
| EGLN1 | 15625 | 1.194597 | 0.2322445 | 0.3737958 | FALSE |
| PARP14 | 12589 | 1.193145 | 0.2328126 | 0.37455585 | FALSE |
| HAMP | 2433 | 1.192741 | 0.2329708 | 0.37477185 | FALSE |
| ZRANB3 | 10375 | 1.192281 | 0.2331511 | 0.37498466 | FALSE |
| TST | 2451 | 1.191926 | 0.2332903 | 0.37513132 | FALSE |
| PML | 2765 | 1.191723 | 0.2333699 | 0.37522074 | FALSE |
| SOX6 | 458 | 1.19148 | 0.2334652 | 0.37533541 | FALSE |
| SMARCA5 | 9159 | 1.191218 | 0.233568 | 0.37542348 | FALSE |
| SPTSSA | 1246 | 1.190951 | 0.2336728 | 0.37551472 | FALSE |
| AKR1B1 | 2791 | 1.189668 | 0.2341769 | 0.37620878 | FALSE |
| NPAS2 | 3401 | 1.189269 | 0.2343338 | 0.37635622 | FALSE |
| CCL19 | 212 | 1.189251 | 0.2343409 | 0.37635622 | FALSE |
| PTP4A3 | 15600 | 1.188863 | 0.2344936 | 0.37652405 | FALSE |
| CASP5 | 5518 | 1.188794 | 0.2345207 | 0.37652899 | FALSE |
| ZFAND6 | 451 | 1.188641 | 0.234581 | 0.37654506 | FALSE |
| SLC52A2 | 10376 | 1.188601 | 0.2345967 | 0.37654506 | FALSE |
| CD19 | 2996 | 1.188585 | 0.234603 | 0.37654506 | FALSE |
| DKK4 | 2709 | 1.188123 | 0.234785 | 0.37675972 | FALSE |
| IFNL2 | 12528 | 1.187288 | 0.235114 | 0.37705558 | FALSE |
| ACACB | 1170 | 1.187124 | 0.2351787 | 0.37712061 | FALSE |
| TPTEP1 | 12296 | 1.187001 | 0.2352272 | 0.37715972 | FALSE |
| A2M | 12704 | 1.185412 | 0.2358546 | 0.3780493 | FALSE |
| C19orf73 | 7917 | 1.185098 | 0.2359787 | 0.37820946 | FALSE |
| ZNF19 | 1214 | 1.184818 | 0.2360894 | 0.37834811 | FALSE |
| HIST1H4C | 9931 | 1.184628 | 0.2361646 | 0.37842974 | FALSE |
| ANKDD1A | 15066 | 1.184527 | 0.2362045 | 0.37845497 | FALSE |
| PVT1 | 7115 | 1.183055 | 0.2367874 | 0.3792722 | FALSE |
| RTN4R | 9962 | 1.182637 | 0.236953 | 0.37945984 | FALSE |
| MHENCR | 576 | 1.181191 | 0.2375269 | 0.38022295 | FALSE |
| COL1A2 | 10046 | 1.180483 | 0.2378082 | 0.38055636 | FALSE |
| GLP2R | 9363 | 1.180398 | 0.237842 | 0.38056072 | FALSE |
| FAM129B | 11009 | 1.180345 | 0.237863 | 0.38056072 | FALSE |
| H1FX | 7840 | 1.180216 | 0.2379143 | 0.38056072 | FALSE |
| LOC390705 | 764 | 1.179896 | 0.2380416 | 0.38069607 | FALSE |
| HLA-F | 4199 | 1.179634 | 0.2381458 | 0.38070698 | FALSE |
| VGLL3 | 13529 | 1.178668 | 0.2385304 | 0.38116588 | FALSE |
| KDM1B | 13475 | 1.177643 | 0.238939 | 0.38165937 | FALSE |
| CST5 | 5648 | 1.176895 | 0.2392374 | 0.3819053 | FALSE |
| GP9 | 15499 | 1.176375 | 0.2394451 | 0.38219773 | FALSE |
| CYGB | 5941 | 1.175886 | 0.2396404 | 0.38235343 | FALSE |
| SSNA1 | 9691 | 1.175518 | 0.2397875 | 0.38253685 | FALSE |
| RESP18 | 888 | 1.175476 | 0.2398043 | 0.38253685 | FALSE |
| TRNAU1AP | 13024 | 1.17494 | 0.2400187 | 0.38280072 | FALSE |
| TUBB4A | 6032 | 1.174683 | 0.2401216 | 0.38287884 | FALSE |
| ADHFE1 | 165 | 1.174571 | 0.2401664 | 0.38287999 | FALSE |
| NFAM1 | 11468 | 1.17328 | 0.2406835 | 0.38354799 | FALSE |
| ZFR2 | 7704 | 1.172987 | 0.240801 | 0.38365698 | FALSE |
| HDGFL1 | 9906 | 1.17189 | 0.2412412 | 0.38420172 | FALSE |
| HMGN5 | 1013 | 1.171595 | 0.2413597 | 0.38422667 | FALSE |
| STBD1 | 7365 | 1.171132 | 0.2415457 | 0.38441114 | FALSE |
| EFNA4 | 1944 | 1.171073 | 0.2415694 | 0.38441114 | FALSE |
| CHPF | 4622 | 1.170003 | 0.2419998 | 0.38497963 | FALSE |
| ADGRB2 | 8437 | 1.170001 | 0.2420006 | 0.38497963 | FALSE |
| OBSL1 | 5825 | 1.16974 | 0.2421056 | 0.38506838 | FALSE |
| RTCB | 11607 | 1.169095 | 0.2423654 | 0.38544228 | FALSE |
| ESYT3 | 12460 | 1.168517 | 0.2425983 | 0.38577347 | FALSE |
| MOS | 11187 | 1.167906 | 0.2428447 | 0.38608674 | FALSE |
| SLC22A31 | 411 | 1.167804 | 0.2428858 | 0.3861129 | FALSE |
| EBPL | 15591 | 1.167401 | 0.2430485 | 0.38633215 | FALSE |
| CREG2 | 13801 | 1.166412 | 0.2434479 | 0.38676003 | FALSE |
| PEX11A | 10465 | 1.166367 | 0.2434661 | 0.38676003 | FALSE |
| BNIP3 | 5228 | 1.166106 | 0.2435716 | 0.38685092 | FALSE |
| THOC7 | 11910 | 1.166103 | 0.2435728 | 0.38685092 | FALSE |
| NSUN6 | 2207 | 1.165848 | 0.2436759 | 0.38697536 | FALSE |
| STARD7-AS1 | 3520 | 1.165741 | 0.2437192 | 0.38700477 | FALSE |
| SUSD2 | 9299 | 1.164461 | 0.2442372 | 0.38766989 | FALSE |
| SLC30A1 | 2832 | 1.164367 | 0.2442753 | 0.38769096 | FALSE |
| LINC01881 | 6473 | 1.164138 | 0.2443681 | 0.38775948 | FALSE |
| SGCE | 4348 | 1.162891 | 0.2448737 | 0.38840412 | FALSE |
| DPRX | 11687 | 1.161883 | 0.245283 | 0.38893489 | FALSE |
| SMIM24 | 10092 | 1.161317 | 0.245513 | 0.38926013 | FALSE |
| SENP2 | 2199 | 1.161171 | 0.2455724 | 0.38928881 | FALSE |
| LINC01126 | 10901 | 1.16115 | 0.2455809 | 0.38928881 | FALSE |
| ECHDC1 | 10393 | 1.161053 | 0.2456203 | 0.38931186 | FALSE |
| SLC9A3R1 | 3089 | 1.160596 | 0.2458062 | 0.38949159 | FALSE |
| RHPN2 | 14359 | 1.160588 | 0.2458095 | 0.38949159 | FALSE |
| LYPD3 | 8674 | 1.160501 | 0.2458449 | 0.38949159 | FALSE |
| RNF167 | 7046 | 1.160468 | 0.2458583 | 0.38949159 | FALSE |
| SLC37A4 | 15510 | 1.160171 | 0.2459792 | 0.38956465 | FALSE |
| PIWIL4 | 6772 | 1.159733 | 0.2461575 | 0.38976812 | FALSE |
| IRF2BPL | 14209 | 1.157498 | 0.247069 | 0.39105285 | FALSE |
| HVCN1 | 9768 | 1.157152 | 0.2472103 | 0.39119729 | FALSE |
| LALBA | 1583 | 1.157002 | 0.2472715 | 0.39125466 | FALSE |
| SSX5 | 9689 | 1.156798 | 0.2473549 | 0.39134694 | FALSE |
| CIB3 | 8516 | 1.1562 | 0.2475994 | 0.39165444 | FALSE |
| TNNT3 | 5453 | 1.15515 | 0.248029 | 0.39217533 | FALSE |
| CADPS | 8901 | 1.154987 | 0.2480958 | 0.39224119 | FALSE |
| HRH4 | 766 | 1.154814 | 0.2481666 | 0.39231353 | FALSE |
| FGF13 | 8188 | 1.154562 | 0.2482698 | 0.39239737 | FALSE |
| LITAF | 3889 | 1.154397 | 0.2483374 | 0.39246454 | FALSE |
| RNASEH1-AS1 | 5700 | 1.154325 | 0.248367 | 0.3924715 | FALSE |
| CIPC | 4843 | 1.153762 | 0.2485978 | 0.39279652 | FALSE |
| CDYL2 | 10249 | 1.153507 | 0.2487023 | 0.39292206 | FALSE |
| IL27 | 10345 | 1.153108 | 0.2488661 | 0.39306154 | FALSE |
| RPL30 | 9009 | 1.152838 | 0.2489769 | 0.39319685 | FALSE |
| MORN1 | 12933 | 1.152635 | 0.2490602 | 0.39328875 | FALSE |
| ROBO3 | 12578 | 1.15229 | 0.2492019 | 0.39341144 | FALSE |
| SCOC | 10451 | 1.152067 | 0.2492935 | 0.39341388 | FALSE |
| METTL23 | 1782 | 1.15197 | 0.2493334 | 0.39341388 | FALSE |
| PDE3A | 13411 | 1.151952 | 0.2493408 | 0.39341388 | FALSE |
| DNAJC9-AS1 | 2115 | 1.151791 | 0.249407 | 0.39347812 | FALSE |
| KRTAP10-9 | 2648 | 1.151668 | 0.2494575 | 0.39347812 | FALSE |
| OR2W3 | 6532 | 1.151608 | 0.2494822 | 0.39347812 | FALSE |
| KLHL26 | 425 | 1.151489 | 0.2495311 | 0.39351558 | FALSE |
| IAH1 | 14862 | 1.151075 | 0.2497014 | 0.39370466 | FALSE |
| DDX41 | 9112 | 1.15082 | 0.2498063 | 0.39383036 | FALSE |
| APAF1 | 10718 | 1.150512 | 0.2499331 | 0.39391102 | FALSE |
| SLC26A2 | 11052 | 1.149565 | 0.2503231 | 0.3943667 | FALSE |
| RPS28 | 15011 | 1.149387 | 0.2503964 | 0.39444252 | FALSE |
| RGN | 3825 | 1.148521 | 0.2507535 | 0.39480615 | FALSE |
| ATG2A | 2708 | 1.146176 | 0.2517223 | 0.39609213 | FALSE |
| ALAS1 | 10458 | 1.145509 | 0.2519984 | 0.39636689 | FALSE |
| FLJ32255 | 4167 | 1.14487 | 0.252263 | 0.39674323 | FALSE |
| GPR176 | 41 | 1.144748 | 0.2523136 | 0.39678281 | FALSE |
| USF3 | 8558 | 1.143809 | 0.2527029 | 0.39731507 | FALSE |
| TBXAS1 | 856 | 1.143193 | 0.2529585 | 0.39763698 | FALSE |
| ZNF677 | 3059 | 1.142994 | 0.2530411 | 0.39772685 | FALSE |
| RPL34 | 15577 | 1.142924 | 0.2530701 | 0.39773254 | FALSE |
| PLPP5 | 7330 | 1.142767 | 0.2531353 | 0.39775503 | FALSE |
| IMMT | 2740 | 1.14248 | 0.2532546 | 0.39786237 | FALSE |
| ZNF608 | 10573 | 1.141976 | 0.253464 | 0.39807139 | FALSE |
| MAEL | 10200 | 1.141847 | 0.2535176 | 0.39810566 | FALSE |
| CALCRL | 1374 | 1.14151 | 0.2536578 | 0.39819854 | FALSE |
| ZDHHC16 | 8968 | 1.141475 | 0.2536723 | 0.39819854 | FALSE |
| CLEC2D | 355 | 1.141253 | 0.2537647 | 0.3983035 | FALSE |
| BORCS8-MEF2B | 15203 | 1.140915 | 0.2539053 | 0.3984258 | FALSE |
| DEFB123 | 5731 | 1.140697 | 0.253996 | 0.39847839 | FALSE |
| STN1 | 1572 | 1.140679 | 0.2540035 | 0.39847839 | FALSE |
| PTGIR | 6461 | 1.139234 | 0.2546056 | 0.39922974 | FALSE |
| LLCFC1 | 6084 | 1.139223 | 0.2546102 | 0.39922974 | FALSE |
| AGAP2-AS1 | 3206 | 1.138582 | 0.2548775 | 0.39956885 | FALSE |
| MGC34796 | 2558 | 1.138021 | 0.2551117 | 0.39985577 | FALSE |
| CCDC6 | 745 | 1.137376 | 0.2553811 | 0.40020959 | FALSE |
| MYLIP | 8918 | 1.137095 | 0.2554986 | 0.40034171 | FALSE |
| AMZ2 | 13742 | 1.136631 | 0.2556926 | 0.40056539 | FALSE |
| SOCS2 | 11852 | 1.136518 | 0.2557398 | 0.40057061 | FALSE |
| TKTL1 | 9189 | 1.13642 | 0.2557808 | 0.40057061 | FALSE |
| PMPCB | 8610 | 1.136378 | 0.2557984 | 0.40057061 | FALSE |
| STEAP1 | 2155 | 1.136177 | 0.2558825 | 0.40066216 | FALSE |
| KLHL29 | 13142 | 1.136098 | 0.2559156 | 0.40067379 | FALSE |
| BRCC3 | 4753 | 1.135576 | 0.2561341 | 0.40093559 | FALSE |
| A4GALT | 5123 | 1.135226 | 0.2562806 | 0.40107965 | FALSE |
| SLC35G2 | 4935 | 1.135119 | 0.2563255 | 0.40107965 | FALSE |
| COL25A1 | 8480 | 1.135093 | 0.2563364 | 0.40107965 | FALSE |
| CHTOP | 1319 | 1.134133 | 0.2567388 | 0.40148015 | FALSE |
| MUL1 | 1151 | 1.133256 | 0.2571068 | 0.40190072 | FALSE |
| ATP13A4 | 11080 | 1.133101 | 0.2571718 | 0.40195635 | FALSE |
| PLEKHA7 | 3926 | 1.132768 | 0.2573117 | 0.40212341 | FALSE |
| GPATCH4 | 1078 | 1.132724 | 0.2573302 | 0.40212341 | FALSE |
| C19orf48 | 5435 | 1.132302 | 0.2575075 | 0.40236028 | FALSE |
| NUDT7 | 13857 | 1.132142 | 0.2575747 | 0.40242514 | FALSE |
| LYRM4 | 6990 | 1.131325 | 0.2579183 | 0.40284116 | FALSE |
| FOS | 13383 | 1.130709 | 0.2581776 | 0.40316555 | FALSE |
| TMEM62 | 5689 | 1.130367 | 0.2583216 | 0.40322048 | FALSE |
| PTPRO | 1655 | 1.130309 | 0.258346 | 0.40322048 | FALSE |
| LINC02470 | 4940 | 1.130179 | 0.2584008 | 0.4032215 | FALSE |
| NDNF | 10327 | 1.128914 | 0.2589341 | 0.40382253 | FALSE |
| DEF8 | 10985 | 1.128629 | 0.2590544 | 0.40396978 | FALSE |
| FURIN | 326 | 1.128386 | 0.2591569 | 0.40404911 | FALSE |
| UBE2R2 | 7461 | 1.126239 | 0.2600644 | 0.40514064 | FALSE |
| AMIGO3 | 388 | 1.125971 | 0.2601778 | 0.4052669 | FALSE |
| RAP1GDS1 | 13359 | 1.125925 | 0.2601973 | 0.4052669 | FALSE |
| COMMD10 | 14258 | 1.12497 | 0.2606017 | 0.40581603 | FALSE |
| EMC4 | 2722 | 1.123689 | 0.261145 | 0.40654048 | FALSE |
| GEMIN2 | 1137 | 1.123387 | 0.2612732 | 0.40665688 | FALSE |
| SNHG12 | 3121 | 1.122457 | 0.2616682 | 0.40719278 | FALSE |
| REC8 | 13275 | 1.122328 | 0.261723 | 0.40723756 | FALSE |
| RAB28 | 6875 | 1.121864 | 0.2619203 | 0.40742284 | FALSE |
| NID2 | 13809 | 1.121408 | 0.2621142 | 0.40764343 | FALSE |
| CYP51A1 | 15464 | 1.121228 | 0.2621908 | 0.40772199 | FALSE |
| PCP2 | 8577 | 1.119957 | 0.2627321 | 0.40840118 | FALSE |
| SELENOI | 15633 | 1.119341 | 0.2629947 | 0.40876874 | FALSE |
| CDC42EP5 | 8502 | 1.119199 | 0.2630553 | 0.40882222 | FALSE |
| PTPRN2 | 1728 | 1.11755 | 0.2637592 | 0.40963124 | FALSE |
| NYNRIN | 4894 | 1.116352 | 0.2642715 | 0.41034527 | FALSE |
| POLR2H | 11165 | 1.115968 | 0.2644358 | 0.41055968 | FALSE |
| LIPN | 9499 | 1.11454 | 0.2650476 | 0.41142777 | FALSE |
| HMGN3 | 5278 | 1.113509 | 0.2654899 | 0.41190982 | FALSE |
| ARHGAP25 | 9186 | 1.113325 | 0.2655689 | 0.41199149 | FALSE |
| FKBP10 | 13939 | 1.112372 | 0.2659782 | 0.41258561 | FALSE |
| THYN1 | 15548 | 1.112299 | 0.2660096 | 0.41259334 | FALSE |
| LTA4H | 10448 | 1.111886 | 0.2661872 | 0.41282778 | FALSE |
| KNTC1 | 10873 | 1.111585 | 0.2663166 | 0.41293634 | FALSE |
| SLC35G6 | 9111 | 1.111452 | 0.2663738 | 0.41295342 | FALSE |
| HIST1H2AM | 14652 | 1.111075 | 0.2665361 | 0.41312298 | FALSE |
| IDH1-AS1 | 11148 | 1.11084 | 0.2666372 | 0.4132388 | FALSE |
| HMGCLL1 | 837 | 1.110741 | 0.2666798 | 0.41326389 | FALSE |
| MAGOHB | 9039 | 1.110642 | 0.2667225 | 0.41326736 | FALSE |
| TMC4 | 2502 | 1.110523 | 0.2667737 | 0.41328645 | FALSE |
| ZNF704 | 13075 | 1.10931 | 0.2672965 | 0.41401424 | FALSE |
| CDK16 | 2690 | 1.109078 | 0.2673965 | 0.4141282 | FALSE |
| KIAA0100 | 7226 | 1.108608 | 0.2675993 | 0.41440121 | FALSE |
| DEDD | 2331 | 1.107323 | 0.2681543 | 0.41513728 | FALSE |
| TAT | 11205 | 1.10692 | 0.2683285 | 0.41532473 | FALSE |
| SLC52A1 | 14612 | 1.106653 | 0.268444 | 0.4153901 | FALSE |
| EID2 | 2260 | 1.106638 | 0.2684505 | 0.4153901 | FALSE |
| P2RY1 | 13624 | 1.106449 | 0.2685322 | 0.41547549 | FALSE |
| IMMP2L | 9188 | 1.10618 | 0.2686486 | 0.41557331 | FALSE |
| RPS7P5 | 976 | 1.104625 | 0.2693221 | 0.4164915 | FALSE |
| STX5 | 4888 | 1.103564 | 0.2697823 | 0.41703816 | FALSE |
| COX6B1 | 509 | 1.103225 | 0.2699295 | 0.41718313 | FALSE |
| DALRD3 | 3151 | 1.102831 | 0.2701006 | 0.4172856 | FALSE |
| LINC00471 | 6061 | 1.10278 | 0.2701227 | 0.4172856 | FALSE |
| GID4 | 8465 | 1.102354 | 0.2703078 | 0.41745515 | FALSE |
| ARHGEF4 | 4366 | 1.102328 | 0.2703191 | 0.41745515 | FALSE |
| PCYT1B | 1801 | 1.10219 | 0.2703791 | 0.41749818 | FALSE |
| ESS2 | 1685 | 1.101574 | 0.2706469 | 0.41778594 | FALSE |
| RBM12 | 3082 | 1.101528 | 0.2706669 | 0.41778594 | FALSE |
| TRIM15 | 9936 | 1.101413 | 0.270717 | 0.41782191 | FALSE |
| MGC70870 | 10521 | 1.100718 | 0.2710194 | 0.41822764 | FALSE |
| ATP6V0E1 | 14619 | 1.100432 | 0.2711439 | 0.41835701 | FALSE |
| DDX60L | 8481 | 1.100126 | 0.2712772 | 0.41852136 | FALSE |
| IGSF9 | 13233 | 1.099816 | 0.2714123 | 0.41864711 | FALSE |
| ZNF569 | 11334 | 1.099744 | 0.2714437 | 0.4186542 | FALSE |
| SULT2B1 | 13643 | 1.099484 | 0.271557 | 0.41878768 | FALSE |
| SYNC | 12222 | 1.098757 | 0.2718741 | 0.41911127 | FALSE |
| CRYZL2P | 2733 | 1.098446 | 0.2720098 | 0.41927913 | FALSE |
| CPSF6 | 3025 | 1.098313 | 0.2720678 | 0.41932727 | FALSE |
| GRAMD4 | 14480 | 1.098072 | 0.2721731 | 0.41944808 | FALSE |
| FAM187B | 4578 | 1.097592 | 0.2723827 | 0.41969244 | FALSE |
| NCOA7 | 13623 | 1.097586 | 0.2723853 | 0.41969244 | FALSE |
| IRX5 | 14855 | 1.09751 | 0.2724185 | 0.41970224 | FALSE |
| TIMM29 | 10462 | 1.097115 | 0.2725911 | 0.41992679 | FALSE |
| ARVCF | 5628 | 1.094702 | 0.2736472 | 0.4211387 | FALSE |
| VIPAS39 | 11083 | 1.094499 | 0.2737362 | 0.42123416 | FALSE |
| OR52B6 | 11040 | 1.09436 | 0.2737971 | 0.42124499 | FALSE |
| PHOSPHO2 | 5067 | 1.094216 | 0.2738603 | 0.42130067 | FALSE |
| ZNF471 | 6044 | 1.092797 | 0.2744829 | 0.42208739 | FALSE |
| MBLAC1 | 8490 | 1.092743 | 0.2745067 | 0.42208739 | FALSE |
| CUEDC2 | 3792 | 1.092556 | 0.2745888 | 0.42217216 | FALSE |
| GALNT2 | 2131 | 1.092399 | 0.2746578 | 0.422179 | FALSE |
| DICER1-AS1 | 4968 | 1.092132 | 0.2747751 | 0.42225095 | FALSE |
| SLC25A1 | 6014 | 1.091658 | 0.2749835 | 0.42252962 | FALSE |
| METRN | 12392 | 1.091459 | 0.275071 | 0.42262254 | FALSE |
| SEC22A | 2841 | 1.091002 | 0.275272 | 0.42281893 | FALSE |
| FIBP | 11992 | 1.090984 | 0.2752799 | 0.42281893 | FALSE |
| RAB26 | 12137 | 1.090092 | 0.2756726 | 0.42333891 | FALSE |
| UQCR10 | 14675 | 1.090025 | 0.2757021 | 0.42334264 | FALSE |
| IGHMBP2 | 11517 | 1.089778 | 0.2758109 | 0.42346813 | FALSE |
| TJP2 | 3176 | 1.089703 | 0.275844 | 0.42347728 | FALSE |
| ATP8B3 | 13547 | 1.088054 | 0.2765713 | 0.42442708 | FALSE |
| NCBP2 | 7711 | 1.0869 | 0.277081 | 0.42512586 | FALSE |
| LOC728739 | 11429 | 1.086704 | 0.2771676 | 0.42521706 | FALSE |
| RNF40 | 12273 | 1.086469 | 0.2772715 | 0.42533472 | FALSE |
| HNRNPR | 10257 | 1.085945 | 0.2775033 | 0.42548355 | FALSE |
| GFRA3 | 1418 | 1.085759 | 0.2775856 | 0.42552424 | FALSE |
| HPR | 1981 | 1.08553 | 0.277687 | 0.42559614 | FALSE |
| GRIN2B | 214 | 1.085246 | 0.2778127 | 0.42566738 | FALSE |
| ZIC4 | 9990 | 1.085241 | 0.2778149 | 0.42566738 | FALSE |
| SLC6A19 | 6187 | 1.085184 | 0.2778402 | 0.42566738 | FALSE |
| LASP1 | 3102 | 1.084909 | 0.277962 | 0.42576713 | FALSE |
| ZNF579 | 12942 | 1.08481 | 0.2780058 | 0.42579259 | FALSE |
| LOC100268168 | 5963 | 1.084744 | 0.2780351 | 0.42579565 | FALSE |
| TOR3A | 4418 | 1.084537 | 0.2781268 | 0.42589439 | FALSE |
| NDUFA7 | 1712 | 1.084472 | 0.2781556 | 0.42589678 | FALSE |
| APBB1 | 13508 | 1.083934 | 0.2783941 | 0.42622019 | FALSE |
| NDUFA10 | 10774 | 1.083723 | 0.2784876 | 0.42629489 | FALSE |
| DNM1P50 | 2704 | 1.083701 | 0.2784974 | 0.42629489 | FALSE |
| LINC01664 | 12892 | 1.083091 | 0.278768 | 0.42664974 | FALSE |
| SLC25A38 | 14216 | 1.083021 | 0.2787991 | 0.42664974 | FALSE |
| WDR41 | 12571 | 1.082994 | 0.2788111 | 0.42664974 | FALSE |
| ACSM1 | 265 | 1.082408 | 0.2790713 | 0.42700609 | FALSE |
| SUPT4H1 | 334 | 1.081765 | 0.279357 | 0.42740139 | FALSE |
| TMEM198B | 12325 | 1.081245 | 0.2795881 | 0.42770743 | FALSE |
| SPNS3 | 15000 | 1.080915 | 0.2797349 | 0.42782375 | FALSE |
| CDCA3 | 4203 | 1.080898 | 0.2797425 | 0.42782375 | FALSE |
| TROAP | 11005 | 1.08077 | 0.2797994 | 0.42786898 | FALSE |
| ZNF691 | 10843 | 1.079927 | 0.2801747 | 0.42831712 | FALSE |
| PPIA | 7718 | 1.079116 | 0.280536 | 0.42880708 | FALSE |
| C9orf62 | 2382 | 1.079064 | 0.2805592 | 0.42880708 | FALSE |
| ATF3 | 1051 | 1.078198 | 0.2809454 | 0.42928546 | FALSE |
| RHOF | 2106 | 1.076541 | 0.2816854 | 0.43020589 | FALSE |
| TIMM23 | 276 | 1.076135 | 0.2818669 | 0.43044105 | FALSE |
| RFX1 | 9712 | 1.075665 | 0.2820771 | 0.4306755 | FALSE |
| CHAMP1 | 3847 | 1.075509 | 0.2821469 | 0.43069209 | FALSE |
| VSIG8 | 7478 | 1.075422 | 0.2821858 | 0.43069209 | FALSE |
| NDUFB4 | 4662 | 1.075398 | 0.2821966 | 0.43069209 | FALSE |
| TNFAIP8L3 | 9308 | 1.074678 | 0.2825189 | 0.43097368 | FALSE |
| MINCR | 5023 | 1.073854 | 0.2828881 | 0.4314106 | FALSE |
| LRRC7 | 12706 | 1.073519 | 0.2830383 | 0.43155545 | FALSE |
| FAM173A | 13734 | 1.073245 | 0.2831612 | 0.43165862 | FALSE |
| EXD3 | 5422 | 1.070625 | 0.2843381 | 0.43324144 | FALSE |
| RGS13 | 1693 | 1.069776 | 0.2847201 | 0.43365453 | FALSE |
| GRPR | 3341 | 1.069643 | 0.28478 | 0.43370155 | FALSE |
| FBXW4 | 8040 | 1.069461 | 0.284862 | 0.43370155 | FALSE |
| TTC32 | 12787 | 1.068862 | 0.2851319 | 0.43394955 | FALSE |
| DKFZP434K028 | 1401 | 1.068478 | 0.2853049 | 0.43411049 | FALSE |
| TBC1D22A | 12367 | 1.068425 | 0.2853288 | 0.43411049 | FALSE |
| ABI3 | 8387 | 1.068095 | 0.2854777 | 0.43425827 | FALSE |
| MARK4 | 10287 | 1.06799 | 0.285525 | 0.43428806 | FALSE |
| PRPS1L1 | 11533 | 1.067716 | 0.2856486 | 0.43443381 | FALSE |
| ACTN2 | 12410 | 1.067576 | 0.2857118 | 0.43448763 | FALSE |
| CA4 | 760 | 1.066863 | 0.2860337 | 0.43489252 | FALSE |
| ANAPC15 | 12855 | 1.066739 | 0.2860897 | 0.43489865 | FALSE |
| C2CD4B | 971 | 1.066424 | 0.286232 | 0.43489865 | FALSE |
| COMMD3 | 13457 | 1.066423 | 0.2862325 | 0.43489865 | FALSE |
| PGGHG | 8601 | 1.064982 | 0.2868841 | 0.43567697 | FALSE |
| TNR | 10049 | 1.064483 | 0.28711 | 0.4359353 | FALSE |
| RAB3A | 11747 | 1.064067 | 0.2872983 | 0.43617899 | FALSE |
| ALB | 15382 | 1.063542 | 0.2875362 | 0.43643226 | FALSE |
| ZNF473 | 8933 | 1.063514 | 0.2875489 | 0.43643226 | FALSE |
| SLC4A1AP | 2999 | 1.06336 | 0.2876187 | 0.4364851 | FALSE |
| LAMTOR5 | 2102 | 1.062953 | 0.2878033 | 0.43669111 | FALSE |
| B3GLCT | 14684 | 1.062808 | 0.287869 | 0.43674603 | FALSE |
| LSG1 | 7293 | 1.062202 | 0.288144 | 0.43686888 | FALSE |
| DCLRE1B | 5958 | 1.062118 | 0.2881821 | 0.43688432 | FALSE |
| SUZ12P1 | 12327 | 1.061372 | 0.2885209 | 0.43718589 | FALSE |
| ALG14 | 12781 | 1.061226 | 0.2885872 | 0.43724402 | FALSE |
| SEC61A1 | 12300 | 1.061081 | 0.2886531 | 0.43730146 | FALSE |
| ALG8 | 15231 | 1.060811 | 0.2887758 | 0.43744497 | FALSE |
| LOC728989 | 4365 | 1.060475 | 0.2889286 | 0.43761498 | FALSE |
| HIST1H4J | 1769 | 1.060291 | 0.2890122 | 0.43767589 | FALSE |
| RBMX | 11232 | 1.059917 | 0.2891824 | 0.4378487 | FALSE |
| TRO | 2706 | 1.05947 | 0.2893858 | 0.43811427 | FALSE |
| HIST1H2AH | 8614 | 1.059387 | 0.2894236 | 0.43812521 | FALSE |
| VPS33B | 143 | 1.059331 | 0.2894491 | 0.43812521 | FALSE |
| ZER1 | 7419 | 1.058993 | 0.289603 | 0.43831573 | FALSE |
| PRTG | 9538 | 1.058518 | 0.2898193 | 0.43855831 | FALSE |
| NUCB1 | 9535 | 1.057528 | 0.2902707 | 0.43912549 | FALSE |
| PIPOX | 13776 | 1.057511 | 0.2902784 | 0.43912549 | FALSE |
| RBM11 | 10238 | 1.056637 | 0.2906773 | 0.43960124 | FALSE |
| CLTC | 10572 | 1.056537 | 0.2907229 | 0.43962776 | FALSE |
| APEX2 | 14156 | 1.056344 | 0.2908111 | 0.43967598 | FALSE |
| BMP2 | 4586 | 1.055907 | 0.2910107 | 0.43989271 | FALSE |
| CHST6 | 11953 | 1.055533 | 0.2911816 | 0.43999609 | FALSE |
| ZNF467 | 2888 | 1.053953 | 0.2919044 | 0.44090261 | FALSE |
| MTM1 | 2093 | 1.053533 | 0.2920968 | 0.44115051 | FALSE |
| BMPR2 | 11732 | 1.053446 | 0.2921366 | 0.44116808 | FALSE |
| POTEF | 2655 | 1.053383 | 0.2921655 | 0.44116905 | FALSE |
| IKBKG | 10367 | 1.052751 | 0.2924551 | 0.44156375 | FALSE |
| SINHCAF | 13425 | 1.05216 | 0.2927261 | 0.44175962 | FALSE |
| RNF182 | 7257 | 1.051812 | 0.2928858 | 0.44195791 | FALSE |
| PRELID2 | 13424 | 1.050716 | 0.293389 | 0.44254641 | FALSE |
| RABL3 | 10218 | 1.050524 | 0.2934773 | 0.44263676 | FALSE |
| GSTO1 | 14398 | 1.049718 | 0.2938478 | 0.44315285 | FALSE |
| PRORSD1P | 2766 | 1.049248 | 0.294064 | 0.44335685 | FALSE |
| FAM27E3 | 13931 | 1.049239 | 0.2940681 | 0.44335685 | FALSE |
| MYH11 | 12488 | 1.04856 | 0.2943807 | 0.44371634 | FALSE |
| LSM5 | 8733 | 1.048362 | 0.2944719 | 0.44377098 | FALSE |
| PDHA1 | 5899 | 1.048334 | 0.2944848 | 0.44377098 | FALSE |
| SNX17 | 807 | 1.046814 | 0.2951854 | 0.44452682 | FALSE |
| NSFL1C | 2486 | 1.046635 | 0.295268 | 0.44460835 | FALSE |
| CAMK1G | 12497 | 1.046087 | 0.2955209 | 0.44490348 | FALSE |
| BCAR3 | 8555 | 1.04577 | 0.2956672 | 0.44506245 | FALSE |
| MIEF2 | 7050 | 1.045735 | 0.2956834 | 0.44506245 | FALSE |
| PRRX2 | 3695 | 1.045457 | 0.2958118 | 0.44508387 | FALSE |
| C17orf51 | 7691 | 1.045445 | 0.2958173 | 0.44508387 | FALSE |
| TLL2 | 3962 | 1.045192 | 0.2959342 | 0.44510829 | FALSE |
| CHAT | 13185 | 1.045186 | 0.295937 | 0.44510829 | FALSE |
| ZNF703 | 15071 | 1.045176 | 0.2959416 | 0.44510829 | FALSE |
| SLC5A2 | 15474 | 1.044624 | 0.2961968 | 0.44544919 | FALSE |
| OR1D5 | 12310 | 1.043915 | 0.2965247 | 0.44581371 | FALSE |
| SDF2 | 10318 | 1.043739 | 0.2966062 | 0.44589327 | FALSE |
| CTNNBIP1 | 10079 | 1.043319 | 0.2968006 | 0.44601397 | FALSE |
| EIF3I | 15506 | 1.043224 | 0.2968446 | 0.44601547 | FALSE |
| ANKRD53 | 12529 | 1.043132 | 0.2968872 | 0.44601547 | FALSE |
| FNDC3B | 11664 | 1.042916 | 0.2969872 | 0.4461169 | FALSE |
| CPSF2 | 10937 | 1.042863 | 0.2970117 | 0.4461169 | FALSE |
| PRSS2 | 11060 | 1.042057 | 0.2973853 | 0.44642125 | FALSE |
| CCT6B | 10246 | 1.042056 | 0.2973857 | 0.44642125 | FALSE |
| TLR6 | 14325 | 1.041841 | 0.2974854 | 0.44651229 | FALSE |
| ZNF667 | 7229 | 1.039853 | 0.2984082 | 0.44767826 | FALSE |
| CAVIN3 | 6578 | 1.03982 | 0.2984235 | 0.44767826 | FALSE |
| SMIM15 | 9360 | 1.039729 | 0.2984658 | 0.44769874 | FALSE |
| OR2W5 | 8588 | 1.03947 | 0.2985862 | 0.44783634 | FALSE |
| TCEANC | 6070 | 1.039237 | 0.2986945 | 0.44795583 | FALSE |
| USP20 | 5807 | 1.038763 | 0.298915 | 0.44809243 | FALSE |
| RTP5 | 14439 | 1.038733 | 0.2989289 | 0.44809243 | FALSE |
| EPHA5-AS1 | 1823 | 1.037838 | 0.2993455 | 0.44863081 | FALSE |
| LMF1 | 7282 | 1.036278 | 0.3000725 | 0.44941875 | FALSE |
| PCSK9 | 3346 | 1.035938 | 0.3002311 | 0.44957014 | FALSE |
| ABHD10 | 5184 | 1.035638 | 0.3003711 | 0.44973669 | FALSE |
| TUSC1 | 6717 | 1.035415 | 0.3004751 | 0.44981825 | FALSE |
| HSD17B1 | 6654 | 1.035398 | 0.3004831 | 0.44981825 | FALSE |
| ZNF793 | 11857 | 1.033888 | 0.3011885 | 0.4507448 | FALSE |
| KRTAP3-2 | 12564 | 1.031446 | 0.3023317 | 0.45206617 | FALSE |
| C16orf87 | 4961 | 1.030877 | 0.3025985 | 0.45237854 | FALSE |
| MYOF | 5070 | 1.030485 | 0.3027824 | 0.45261017 | FALSE |
| SPR | 1203 | 1.029658 | 0.3031706 | 0.45313257 | FALSE |
| BTD | 1392 | 1.029617 | 0.3031898 | 0.45313257 | FALSE |
| TMEM222 | 12745 | 1.028333 | 0.3037932 | 0.45373071 | FALSE |
| KLHL41 | 15597 | 1.028191 | 0.30386 | 0.45374374 | FALSE |
| NPC1L1 | 11377 | 1.028077 | 0.3039136 | 0.45378046 | FALSE |
| MROH7 | 13557 | 1.027792 | 0.3040477 | 0.45389394 | FALSE |
| TRAF5 | 8890 | 1.027221 | 0.3043164 | 0.45425174 | FALSE |
| PRKAG1 | 2473 | 1.026878 | 0.3044779 | 0.45440604 | FALSE |
| KRTAP8-1 | 12485 | 1.026759 | 0.304534 | 0.4544463 | FALSE |
| PRR36 | 4518 | 1.02629 | 0.3047549 | 0.45464461 | FALSE |
| MARCKSL1 | 11867 | 1.026241 | 0.304778 | 0.45464461 | FALSE |
| TCF7 | 10584 | 1.02623 | 0.3047832 | 0.45464461 | FALSE |
| SMCO3 | 9427 | 1.026014 | 0.304885 | 0.45469654 | FALSE |
| PLA2G7 | 13975 | 1.025971 | 0.3049053 | 0.45469654 | FALSE |
| MAP7D3 | 5713 | 1.025762 | 0.3050038 | 0.45475671 | FALSE |
| RBM4 | 3345 | 1.025482 | 0.3051358 | 0.45491018 | FALSE |
| OPHN1 | 8836 | 1.02468 | 0.3055142 | 0.45530062 | FALSE |
| EFR3B | 14645 | 1.022665 | 0.3064663 | 0.45650187 | FALSE |
| LOC643802 | 690 | 1.021525 | 0.3070058 | 0.45714978 | FALSE |
| PPP2R1A | 6694 | 1.021439 | 0.3070465 | 0.45714978 | FALSE |
| ACP5 | 4800 | 1.021437 | 0.3070474 | 0.45714978 | FALSE |
| NEU1 | 6 | 1.019846 | 0.3078015 | 0.45796716 | FALSE |
| MEX3D | 4323 | 1.019648 | 0.3078954 | 0.45806331 | FALSE |
| BCLAF3 | 13755 | 1.019539 | 0.3079471 | 0.45809666 | FALSE |
| DCAF12L2 | 13483 | 1.019171 | 0.3081218 | 0.45831284 | FALSE |
| SNTA1 | 12565 | 1.017529 | 0.3089018 | 0.45934201 | FALSE |
| ANAPC5 | 13613 | 1.017361 | 0.3089817 | 0.4594171 | FALSE |
| LBH | 6442 | 1.017207 | 0.309055 | 0.45948229 | FALSE |
| C12orf60 | 13057 | 1.016622 | 0.3093333 | 0.45980862 | FALSE |
| SYNGR4 | 5555 | 1.016447 | 0.3094166 | 0.45987752 | FALSE |
| VWA7 | 14053 | 1.016401 | 0.3094385 | 0.45987752 | FALSE |
| ABI2 | 5523 | 1.015774 | 0.309737 | 0.46023371 | FALSE |
| ARF5 | 13760 | 1.01546 | 0.3098866 | 0.46041221 | FALSE |
| IL17REL | 5077 | 1.015381 | 0.3099242 | 0.46042438 | FALSE |
| IGF2-AS | 9345 | 1.015247 | 0.3099881 | 0.46043315 | FALSE |
| ZNF75D | 7205 | 1.015106 | 0.3100553 | 0.4604878 | FALSE |
| RNF24 | 9846 | 1.014659 | 0.3102684 | 0.460673 | FALSE |
| MAP6 | 315 | 1.014285 | 0.3104468 | 0.46089407 | FALSE |
| ARSJ | 7172 | 1.013589 | 0.3107789 | 0.46122966 | FALSE |
| TWF2 | 6417 | 1.013289 | 0.3109221 | 0.46138071 | FALSE |
| RPRM | 14137 | 1.013223 | 0.3109537 | 0.46138369 | FALSE |
| MMAB | 10343 | 1.012911 | 0.3111027 | 0.46156099 | FALSE |
| TMEM91 | 13963 | 1.012414 | 0.3113401 | 0.46186947 | FALSE |
| MFSD3 | 11038 | 1.012252 | 0.3114176 | 0.46194051 | FALSE |
| R3HDM4 | 12893 | 1.01213 | 0.3114759 | 0.46197977 | FALSE |
| PGAP2 | 11393 | 1.012073 | 0.3115032 | 0.46197977 | FALSE |
| FUBP1 | 5627 | 1.01059 | 0.3122127 | 0.46290034 | FALSE |
| LAMTOR1 | 8438 | 1.010145 | 0.3124258 | 0.46314623 | FALSE |
| PRMT5 | 9892 | 1.01012 | 0.3124378 | 0.46314623 | FALSE |
| ZNF559 | 7048 | 1.008749 | 0.313095 | 0.46406234 | FALSE |
| NCCRP1 | 10668 | 1.008707 | 0.3131152 | 0.46406234 | FALSE |
| STMN4 | 4519 | 1.008552 | 0.3131895 | 0.46408455 | FALSE |
| CARTPT | 1062 | 1.008232 | 0.3133431 | 0.4642681 | FALSE |
| C7 | 5500 | 1.007532 | 0.3136792 | 0.46467798 | FALSE |
| NARF | 3104 | 1.007079 | 0.3138968 | 0.46491226 | FALSE |
| ANKRA2 | 8583 | 1.006018 | 0.3144069 | 0.46553545 | FALSE |
| TTC1 | 7057 | 1.005714 | 0.3145532 | 0.46566379 | FALSE |
| COQ5 | 14230 | 1.004733 | 0.3150254 | 0.46626119 | FALSE |
| CIAO1 | 6519 | 1.004274 | 0.3152466 | 0.46642528 | FALSE |
| BCAR1 | 7059 | 1.004055 | 0.3153521 | 0.46646553 | FALSE |
| ZNF319 | 6037 | 1.003985 | 0.3153858 | 0.46646553 | FALSE |
| SNX33 | 14798 | 1.002949 | 0.3158855 | 0.46710533 | FALSE |
| ENGASE | 3619 | 1.002312 | 0.3161929 | 0.46734686 | FALSE |
| ERICH2 | 1861 | 1.002269 | 0.3162137 | 0.46734686 | FALSE |
| CENPU | 6489 | 1.00199 | 0.3163484 | 0.46746188 | FALSE |
| SLF1 | 12517 | 1.001647 | 0.3165141 | 0.46755715 | FALSE |
| TELO2 | 14350 | 1.001552 | 0.31656 | 0.46755715 | FALSE |
| ZNF578 | 8142 | 1.001518 | 0.3165764 | 0.46755715 | FALSE |
| PLP2 | 77 | 1.000869 | 0.3168901 | 0.46782304 | FALSE |
| NOS1AP | 14308 | 1.000839 | 0.3169047 | 0.46782304 | FALSE |
| NFASC | 9457 | 1.000829 | 0.3169095 | 0.46782304 | FALSE |
| CACFD1 | 12981 | 1.000601 | 0.3170197 | 0.46794162 | FALSE |
| HS3ST2 | 13695 | 1.000476 | 0.3170802 | 0.46798668 | FALSE |
| CSH1 | 5951 | 1.00039 | 0.3171218 | 0.46800389 | FALSE |
| NECAP1 | 11660 | 1.000303 | 0.3171639 | 0.46802182 | FALSE |
| ENAH | 9118 | 1.000013 | 0.3173042 | 0.46818469 | FALSE |
| UCP3 | 12438 | 0.999729 | 0.3174417 | 0.46825492 | FALSE |
| FIG4 | 4883 | 0.999459 | 0.3175724 | 0.46835945 | FALSE |
| SRP9 | 11250 | 0.99944 | 0.3175816 | 0.46835945 | FALSE |
| PP7080 | 12658 | 0.999053 | 0.317769 | 0.46856094 | FALSE |
| UBE2E2 | 15298 | 0.998282 | 0.3181426 | 0.46896565 | FALSE |
| TXNL4B | 1630 | 0.998239 | 0.3181635 | 0.46896565 | FALSE |
| WBP11 | 13507 | 0.997763 | 0.3183943 | 0.46926162 | FALSE |
| CCDC87 | 6687 | 0.997517 | 0.3185136 | 0.46939324 | FALSE |
| UBE2J1 | 4035 | 0.997127 | 0.3187029 | 0.46957232 | FALSE |
| NT5E | 13905 | 0.997051 | 0.3187398 | 0.46957232 | FALSE |
| HMGN1 | 9694 | 0.997019 | 0.3187553 | 0.46957232 | FALSE |
| ALPPL2 | 13709 | 0.995873 | 0.3193119 | 0.4703479 | FALSE |
| NRAS | 11489 | 0.995617 | 0.3194363 | 0.47048683 | FALSE |
| IFIT3 | 13728 | 0.995415 | 0.3195345 | 0.4705428 | FALSE |
| ERP29 | 7362 | 0.995071 | 0.3197017 | 0.47074477 | FALSE |
| RDH12 | 14472 | 0.993935 | 0.3202545 | 0.47146989 | FALSE |
| KRTAP22-1 | 6289 | 0.99384 | 0.3203008 | 0.47149359 | FALSE |
| DLEU1 | 12820 | 0.993638 | 0.3203991 | 0.47154959 | FALSE |
| UPRT | 1109 | 0.992252 | 0.3210746 | 0.47232139 | FALSE |
| KRAS | 14588 | 0.992038 | 0.321179 | 0.47243048 | FALSE |
| RBCK1 | 596 | 0.991878 | 0.321257 | 0.47250083 | FALSE |
| TMEM40 | 11473 | 0.991493 | 0.3214449 | 0.47273267 | FALSE |
| ABCB8 | 404 | 0.990983 | 0.3216939 | 0.47300982 | FALSE |
| CAPN5 | 12477 | 0.990764 | 0.3218008 | 0.47312258 | FALSE |
| DHX9 | 15361 | 0.990064 | 0.3221428 | 0.47350188 | FALSE |
| CYBA | 12120 | 0.989063 | 0.3226323 | 0.47390225 | FALSE |
| NAP1L6 | 10900 | 0.988026 | 0.3231399 | 0.47451121 | FALSE |
| GRK6 | 5698 | 0.986594 | 0.3238417 | 0.47527389 | FALSE |
| CCDC170 | 6708 | 0.984978 | 0.3246349 | 0.47616974 | FALSE |
| IKZF5 | 5241 | 0.983783 | 0.3252222 | 0.47676284 | FALSE |
| ATP8B5P | 15400 | 0.982948 | 0.325633 | 0.47727557 | FALSE |
| LRFN4 | 6713 | 0.982687 | 0.3257615 | 0.47741328 | FALSE |
| NAGA | 5098 | 0.981735 | 0.3262304 | 0.47770182 | FALSE |
| BOD1L2 | 5035 | 0.981609 | 0.3262925 | 0.47770471 | FALSE |
| TXNRD3 | 8332 | 0.98138 | 0.3264054 | 0.47778047 | FALSE |
| NUFIP2 | 1342 | 0.980035 | 0.3270688 | 0.47852758 | FALSE |
| SLC52A3 | 9207 | 0.979414 | 0.3273755 | 0.47875722 | FALSE |
| KLF3 | 14595 | 0.979407 | 0.3273789 | 0.47875722 | FALSE |
| VLDLR | 14327 | 0.978407 | 0.3278731 | 0.47924918 | FALSE |
| AP1S1 | 363 | 0.97831 | 0.327921 | 0.47924918 | FALSE |
| LIME1 | 7807 | 0.978028 | 0.3280605 | 0.47939516 | FALSE |
| SOCS1 | 7963 | 0.977244 | 0.3284484 | 0.47987228 | FALSE |
| PROSER2 | 5306 | 0.976565 | 0.3287846 | 0.4802346 | FALSE |
| TMEM237 | 15386 | 0.976557 | 0.3287885 | 0.4802346 | FALSE |
| SEMA3E | 10202 | 0.976254 | 0.3289386 | 0.48040894 | FALSE |
| ACPP | 2956 | 0.975895 | 0.3291165 | 0.48062384 | FALSE |
| CHURC1 | 3475 | 0.973436 | 0.3303367 | 0.48202361 | FALSE |
| PCDHGC5 | 6939 | 0.973379 | 0.330365 | 0.48202361 | FALSE |
| DLK2 | 12378 | 0.973342 | 0.3303834 | 0.48202361 | FALSE |
| SUPT6H | 5262 | 0.973139 | 0.3304842 | 0.48212578 | FALSE |
| SLURP1 | 13491 | 0.972956 | 0.3305752 | 0.48216847 | FALSE |
| NRG3 | 1103 | 0.972685 | 0.3307099 | 0.48224158 | FALSE |
| OR11A1 | 11814 | 0.972443 | 0.3308302 | 0.48231546 | FALSE |
| LOC644794 | 5670 | 0.972087 | 0.3310073 | 0.48252859 | FALSE |
| RNASEH1 | 7073 | 0.971078 | 0.3315094 | 0.4829004 | FALSE |
| CLCC1 | 4332 | 0.970565 | 0.3317649 | 0.48322755 | FALSE |
| TMEM177 | 5792 | 0.970383 | 0.3318556 | 0.4833146 | FALSE |
| EYA1 | 11041 | 0.970175 | 0.3319593 | 0.48337549 | FALSE |
| PRPF6 | 2303 | 0.970043 | 0.3320251 | 0.48341752 | FALSE |
| CCND2 | 2654 | 0.969677 | 0.3322075 | 0.48356407 | FALSE |
| PNO1 | 13340 | 0.969324 | 0.3323836 | 0.48376801 | FALSE |
| UCN2 | 10764 | 0.969208 | 0.3324414 | 0.48380718 | FALSE |
| FOXK2 | 4543 | 0.968218 | 0.3329355 | 0.48430226 | FALSE |
| TPD52 | 9280 | 0.967849 | 0.3331198 | 0.48447871 | FALSE |
| MYD88 | 12689 | 0.967655 | 0.3332167 | 0.48457457 | FALSE |
| TXNDC17 | 10438 | 0.96672 | 0.333684 | 0.48507501 | FALSE |
| ATP6V1D | 14309 | 0.966652 | 0.333718 | 0.48507501 | FALSE |
| LRRC24 | 2332 | 0.966186 | 0.3339511 | 0.48519125 | FALSE |
| ANKRD33 | 4945 | 0.966062 | 0.3340132 | 0.48523629 | FALSE |
| CYP2J2 | 14616 | 0.965966 | 0.3340612 | 0.48526098 | FALSE |
| NMT1 | 14709 | 0.965696 | 0.3341963 | 0.48536707 | FALSE |
| MDFI | 3182 | 0.96563 | 0.3342294 | 0.48536995 | FALSE |
| TNFRSF1A | 499 | 0.964721 | 0.3346846 | 0.48580829 | FALSE |
| ZNF263 | 14433 | 0.964717 | 0.3346866 | 0.48580829 | FALSE |
| PDLIM4 | 7573 | 0.964106 | 0.3349928 | 0.48611735 | FALSE |
| PLA2G12B | 4866 | 0.964038 | 0.3350269 | 0.48612169 | FALSE |
| TSPO2 | 14303 | 0.963686 | 0.3352034 | 0.4862475 | FALSE |
| CES1 | 5230 | 0.963679 | 0.3352069 | 0.4862475 | FALSE |
| ATG101 | 15330 | 0.963308 | 0.335393 | 0.48642717 | FALSE |
| ARRDC4 | 10154 | 0.962936 | 0.3355796 | 0.48665199 | FALSE |
| EFTUD2 | 3450 | 0.962499 | 0.335799 | 0.48683538 | FALSE |
| MYL9 | 7872 | 0.962084 | 0.3360074 | 0.48700203 | FALSE |
| BNIP3L | 5478 | 0.961858 | 0.3361209 | 0.48712141 | FALSE |
| LOC100507487 | 7654 | 0.961765 | 0.3361677 | 0.48714396 | FALSE |
| NCL | 9214 | 0.961545 | 0.3362782 | 0.48721383 | FALSE |
| LOC107984035 | 15380 | 0.960873 | 0.336616 | 0.48762601 | FALSE |
| TADA1 | 9196 | 0.960312 | 0.3368982 | 0.48790134 | FALSE |
| NKD2 | 14292 | 0.960291 | 0.3369088 | 0.48790134 | FALSE |
| PROS1 | 9419 | 0.959982 | 0.3370643 | 0.48808131 | FALSE |
| ZNF577 | 4759 | 0.959175 | 0.3374706 | 0.48855058 | FALSE |
| SPRYD4 | 7464 | 0.958972 | 0.3375729 | 0.48855058 | FALSE |
| PGAM1 | 3574 | 0.95891 | 0.3376041 | 0.48855058 | FALSE |
| TEX10 | 14639 | 0.958904 | 0.3376071 | 0.48855058 | FALSE |
| GET4 | 961 | 0.958813 | 0.337653 | 0.48857171 | FALSE |
| TBC1D22B | 7446 | 0.958189 | 0.3379675 | 0.48893627 | FALSE |
| MINK1 | 15566 | 0.957661 | 0.3382337 | 0.48923093 | FALSE |
| PRDM10 | 7041 | 0.957401 | 0.3383649 | 0.48937537 | FALSE |
| WBP2 | 3390 | 0.957098 | 0.3385178 | 0.48955122 | FALSE |
| PLEKHB2 | 4023 | 0.956754 | 0.3386914 | 0.48975703 | FALSE |
| LIG1 | 183 | 0.956356 | 0.3388924 | 0.489957 | FALSE |
| RIDA | 12984 | 0.95604 | 0.339052 | 0.49005181 | FALSE |
| SLC29A2 | 1616 | 0.955226 | 0.3394634 | 0.49051037 | FALSE |
| RPL22 | 5229 | 0.954215 | 0.3399748 | 0.4910224 | FALSE |
| TMEM44 | 8425 | 0.953596 | 0.3402882 | 0.49134465 | FALSE |
| NXT1 | 3283 | 0.953588 | 0.3402922 | 0.49134465 | FALSE |
| S100A1 | 5431 | 0.953283 | 0.3404467 | 0.49150382 | FALSE |
| EIF3G | 523 | 0.953244 | 0.3404665 | 0.49150382 | FALSE |
| NDFIP2 | 519 | 0.953086 | 0.3405465 | 0.49150382 | FALSE |
| MAST1 | 8049 | 0.953074 | 0.3405526 | 0.49150382 | FALSE |
| SEMA6C | 11986 | 0.952244 | 0.3409733 | 0.49205528 | FALSE |
| L3MBTL1 | 336 | 0.951748 | 0.3412248 | 0.49232739 | FALSE |
| CNPY1 | 8488 | 0.951365 | 0.3414191 | 0.4925623 | FALSE |
| CD6 | 8361 | 0.950169 | 0.3420264 | 0.49325632 | FALSE |
| WDR83 | 15194 | 0.949866 | 0.3421803 | 0.49343283 | FALSE |
| TEX30 | 15384 | 0.948992 | 0.3426247 | 0.49384581 | FALSE |
| MIP | 330 | 0.9485 | 0.342875 | 0.49411545 | FALSE |
| MAST2 | 6068 | 0.948373 | 0.3429396 | 0.49416304 | FALSE |
| HIST3H2A | 12862 | 0.947757 | 0.3432532 | 0.49447815 | FALSE |
| ZSCAN22 | 5838 | 0.94733 | 0.3434706 | 0.49474584 | FALSE |
| NCR2 | 11282 | 0.947193 | 0.3435404 | 0.49480079 | FALSE |
| OR7E2P | 95 | 0.947004 | 0.3436367 | 0.49489389 | FALSE |
| GIMAP4 | 8882 | 0.946516 | 0.3438855 | 0.49516086 | FALSE |
| CCR3 | 14438 | 0.945825 | 0.3442379 | 0.49541505 | FALSE |
| ELMSAN1 | 8737 | 0.94523 | 0.3445415 | 0.49578578 | FALSE |
| C15orf39 | 9795 | 0.944905 | 0.3447074 | 0.49590375 | FALSE |
| LUC7L3 | 502 | 0.944553 | 0.3448871 | 0.49605488 | FALSE |
| ACAA1 | 9 | 0.944102 | 0.3451175 | 0.4962706 | FALSE |
| CALN1 | 8697 | 0.94363 | 0.3453588 | 0.49650978 | FALSE |
| KCNE4 | 8216 | 0.943596 | 0.3453761 | 0.49650978 | FALSE |
| PGM1 | 7193 | 0.94327 | 0.3455428 | 0.49663242 | FALSE |
| PEBP4 | 13991 | 0.942927 | 0.3457182 | 0.49683888 | FALSE |
| FAHD1 | 2201 | 0.942581 | 0.3458953 | 0.4970019 | FALSE |
| ZNF733P | 14235 | 0.942142 | 0.3461199 | 0.49714196 | FALSE |
| SEMG2 | 14040 | 0.941438 | 0.3464804 | 0.49744301 | FALSE |
| SCNN1B | 6570 | 0.941422 | 0.3464886 | 0.49744301 | FALSE |
| ZNF157 | 3874 | 0.940961 | 0.3467248 | 0.49769071 | FALSE |
| AGER | 13326 | 0.939039 | 0.3477107 | 0.49882944 | FALSE |
| MUC6 | 6657 | 0.938545 | 0.3479644 | 0.49910336 | FALSE |
| ACAD10 | 5173 | 0.938169 | 0.3481576 | 0.49928206 | FALSE |
| BECN1 | 9852 | 0.938116 | 0.3481848 | 0.49928206 | FALSE |
| SCAF1 | 3798 | 0.937873 | 0.3483097 | 0.49941532 | FALSE |
| PCSK1N | 14229 | 0.937552 | 0.3484747 | 0.49960609 | FALSE |
| TPSG1 | 7070 | 0.937452 | 0.3485261 | 0.49963398 | FALSE |
| SYT16 | 4277 | 0.937305 | 0.3486017 | 0.49969652 | FALSE |
| NR2F2-AS1 | 11129 | 0.937116 | 0.3486989 | 0.4997442 | FALSE |
| SCRT2 | 7697 | 0.936657 | 0.348935 | 0.50003678 | FALSE |
| MTX1 | 8406 | 0.936051 | 0.3492469 | 0.50038144 | FALSE |
| FBXO3 | 8823 | 0.935956 | 0.3492958 | 0.50038144 | FALSE |
| DMAP1 | 6618 | 0.935941 | 0.3493036 | 0.50038144 | FALSE |
| TRIM5 | 148 | 0.93586 | 0.3493453 | 0.50039533 | FALSE |
| ACYP1 | 7488 | 0.935342 | 0.3496121 | 0.50068575 | FALSE |
| NKIRAS2 | 4995 | 0.935277 | 0.3496456 | 0.50068784 | FALSE |
| SLC13A5 | 1157 | 0.934796 | 0.3498934 | 0.50095102 | FALSE |
| BABAM1 | 3297 | 0.934449 | 0.3500723 | 0.50111535 | FALSE |
| ZSWIM1 | 6553 | 0.934205 | 0.3501982 | 0.50124957 | FALSE |
| KCNK6 | 2756 | 0.933676 | 0.350471 | 0.50154192 | FALSE |
| MMRN2 | 13451 | 0.933438 | 0.3505939 | 0.50154192 | FALSE |
| TP53INP1 | 11537 | 0.933436 | 0.3505949 | 0.50154192 | FALSE |
| PFKL | 11359 | 0.932785 | 0.350931 | 0.50188493 | FALSE |
| PIK3AP1 | 6083 | 0.9319 | 0.3513882 | 0.50247276 | FALSE |
| LOC399815 | 3968 | 0.931865 | 0.3514063 | 0.50247276 | FALSE |
| CDIPT | 8873 | 0.931337 | 0.3516793 | 0.5027711 | FALSE |
| PLA2G4A | 875 | 0.929169 | 0.3528015 | 0.50432937 | FALSE |
| EPB41L4A | 4846 | 0.929018 | 0.3528798 | 0.50437566 | FALSE |
| CYP4F8 | 5728 | 0.928844 | 0.3529699 | 0.50438565 | FALSE |
| DOK7 | 15283 | 0.928688 | 0.3530508 | 0.50445509 | FALSE |
| SNAP47 | 4684 | 0.928545 | 0.3531249 | 0.5045014 | FALSE |
| SRPX | 10954 | 0.928156 | 0.3533267 | 0.50471085 | FALSE |
| LMAN2 | 6552 | 0.928051 | 0.3533811 | 0.50474253 | FALSE |
| C1orf220 | 6669 | 0.92752 | 0.3536566 | 0.50508988 | FALSE |
| S100B | 11323 | 0.927168 | 0.3538393 | 0.50528518 | FALSE |
| DNASE1L1 | 12293 | 0.927025 | 0.3539136 | 0.50531835 | FALSE |
| RAB1B | 1558 | 0.926877 | 0.3539904 | 0.50538191 | FALSE |
| HSPA9 | 14352 | 0.926616 | 0.3541259 | 0.50552926 | FALSE |
| TBXT | 3117 | 0.926116 | 0.3543857 | 0.50578857 | FALSE |
| RAB11FIP4 | 1005 | 0.925955 | 0.3544694 | 0.50578857 | FALSE |
| PI4K2B | 7549 | 0.925728 | 0.3545874 | 0.50587645 | FALSE |
| SUGCT | 5664 | 0.925331 | 0.3547938 | 0.50611286 | FALSE |
| RARRES1 | 9141 | 0.924463 | 0.3552453 | 0.50666453 | FALSE |
| ZNF627 | 8441 | 0.924126 | 0.3554207 | 0.50677971 | FALSE |
| TMEM87B | 6912 | 0.924121 | 0.3554233 | 0.50677971 | FALSE |
| PLXND1 | 11075 | 0.924029 | 0.3554712 | 0.50680178 | FALSE |
| CERS4 | 11813 | 0.923827 | 0.3555764 | 0.50690551 | FALSE |
| ADAM8 | 8231 | 0.923605 | 0.355692 | 0.50702409 | FALSE |
| PITX3 | 5657 | 0.922398 | 0.356321 | 0.50762123 | FALSE |
| KDM8 | 1325 | 0.922365 | 0.3563382 | 0.50762123 | FALSE |
| C6orf48 | 8847 | 0.922023 | 0.3565166 | 0.50773651 | FALSE |
| MAN2B1 | 12735 | 0.920751 | 0.3571804 | 0.50833454 | FALSE |
| ZNF737 | 9181 | 0.920373 | 0.3573779 | 0.50854618 | FALSE |
| PFKFB3 | 11186 | 0.92002 | 0.3575623 | 0.50875745 | FALSE |
| MISP3 | 13871 | 0.919923 | 0.357613 | 0.50875745 | FALSE |
| MSX1 | 15550 | 0.919902 | 0.357624 | 0.50875745 | FALSE |
| C5orf30 | 6178 | 0.919689 | 0.3577353 | 0.50879254 | FALSE |
| CCT4 | 2291 | 0.919194 | 0.3579941 | 0.50905238 | FALSE |
| AAMDC | 6804 | 0.918728 | 0.3582379 | 0.50921372 | FALSE |
| SUPT7L | 5630 | 0.91822 | 0.3585037 | 0.50945263 | FALSE |
| GHRL | 13398 | 0.917573 | 0.3588425 | 0.50988767 | FALSE |
| CDCA7L | 242 | 0.917505 | 0.3588781 | 0.50989193 | FALSE |
| ELK1 | 4982 | 0.91696 | 0.3591636 | 0.51025123 | FALSE |
| SSRP1 | 14173 | 0.915514 | 0.3599219 | 0.51123554 | FALSE |
| OR5B17 | 4445 | 0.914997 | 0.3601932 | 0.51155113 | FALSE |
| RFX4 | 5585 | 0.914966 | 0.3602095 | 0.51155113 | FALSE |
| DUSP6 | 8059 | 0.914874 | 0.3602578 | 0.51157325 | FALSE |
| AGMAT | 217 | 0.914627 | 0.3603875 | 0.51161702 | FALSE |
| LRRC57 | 10545 | 0.914601 | 0.3604011 | 0.51161702 | FALSE |
| PGM2 | 7482 | 0.914566 | 0.3604195 | 0.51161702 | FALSE |
| NPPC | 11114 | 0.914372 | 0.3605214 | 0.51170751 | FALSE |
| MUC4 | 13377 | 0.91432 | 0.3605487 | 0.51170751 | FALSE |
| RARS2 | 6776 | 0.914257 | 0.3605818 | 0.51170802 | FALSE |
| GPALPP1 | 7473 | 0.914034 | 0.360699 | 0.51182783 | FALSE |
| UBE2T | 13156 | 0.91373 | 0.3608587 | 0.51196158 | FALSE |
| SNRNP25 | 7225 | 0.912376 | 0.3615708 | 0.51278569 | FALSE |
| PRSS21 | 11160 | 0.912027 | 0.3617545 | 0.51299965 | FALSE |
| GKAP1 | 14720 | 0.911259 | 0.3621589 | 0.51344886 | FALSE |
| TMTC3 | 8647 | 0.911176 | 0.3622026 | 0.51344886 | FALSE |
| SNX31 | 14501 | 0.910888 | 0.3623544 | 0.51361739 | FALSE |
| PPM1J | 12964 | 0.910065 | 0.3627882 | 0.5140925 | FALSE |
| LOC101927596 | 1173 | 0.909706 | 0.3629776 | 0.51426758 | FALSE |
| ADSS | 11313 | 0.909508 | 0.363082 | 0.51436896 | FALSE |
| MTTP | 2827 | 0.909307 | 0.3631881 | 0.51447259 | FALSE |
| LRP4 | 9206 | 0.90913 | 0.3632815 | 0.51448225 | FALSE |
| PQLC3 | 4734 | 0.909107 | 0.3632936 | 0.51448225 | FALSE |
| PSME3 | 1029 | 0.908445 | 0.3636432 | 0.51472438 | FALSE |
| ASNSP1 | 7935 | 0.90793 | 0.3639152 | 0.51494265 | FALSE |
| MAGOH | 728 | 0.907696 | 0.3640389 | 0.51496856 | FALSE |
| GFPT1 | 7377 | 0.905488 | 0.3652069 | 0.5163965 | FALSE |
| GTF2F1 | 1848 | 0.905423 | 0.3652413 | 0.51639846 | FALSE |
| RNF122 | 13399 | 0.905198 | 0.3653605 | 0.51652023 | FALSE |
| PRSS58 | 14837 | 0.905089 | 0.3654182 | 0.51655514 | FALSE |
| RAB10 | 10588 | 0.90333 | 0.3663508 | 0.51759257 | FALSE |
| LINC02004 | 8390 | 0.903181 | 0.3664298 | 0.51765643 | FALSE |
| FAAP20 | 15018 | 0.90312 | 0.3664622 | 0.51765643 | FALSE |
| GOLPH3L | 550 | 0.902209 | 0.3669459 | 0.51819509 | FALSE |
| NDUFA5 | 14746 | 0.902152 | 0.3669761 | 0.51819509 | FALSE |
| YARS2 | 13054 | 0.901998 | 0.3670579 | 0.51826379 | FALSE |
| ZNF514 | 8339 | 0.900233 | 0.3679963 | 0.51935413 | FALSE |
| SPRED1 | 13813 | 0.89978 | 0.3682373 | 0.51964744 | FALSE |
| SF3B2 | 7310 | 0.898348 | 0.3690001 | 0.52044188 | FALSE |
| ADRA2A | 14250 | 0.897803 | 0.3692906 | 0.52080467 | FALSE |
| SLMAP | 5723 | 0.896715 | 0.369871 | 0.52146066 | FALSE |
| JUNB | 6703 | 0.896681 | 0.3698892 | 0.52146066 | FALSE |
| THAP11 | 10776 | 0.896572 | 0.3699473 | 0.52149566 | FALSE |
| COQ7 | 7038 | 0.895332 | 0.3706096 | 0.52233506 | FALSE |
| PAFAH2 | 3396 | 0.894881 | 0.3708507 | 0.52253349 | FALSE |
| GDI2 | 8004 | 0.894133 | 0.3712507 | 0.52295573 | FALSE |
| WDR73 | 3107 | 0.893937 | 0.3713556 | 0.52299373 | FALSE |
| SCFD1 | 9498 | 0.89355 | 0.3715627 | 0.5231478 | FALSE |
| PKD2L1 | 14861 | 0.893503 | 0.3715879 | 0.5231478 | FALSE |
| NDUFA9 | 11935 | 0.892673 | 0.3720323 | 0.52372635 | FALSE |
| RNF141 | 665 | 0.892438 | 0.3721582 | 0.52380925 | FALSE |
| NANS | 10379 | 0.892344 | 0.3722086 | 0.5238186 | FALSE |
| HIST1H2AK | 13202 | 0.892238 | 0.3722654 | 0.5238186 | FALSE |
| PAX4 | 14191 | 0.891816 | 0.3724916 | 0.52402479 | FALSE |
| ZNF583 | 3716 | 0.891784 | 0.3725087 | 0.52402479 | FALSE |
| ZNF812P | 6299 | 0.891015 | 0.3729211 | 0.52455245 | FALSE |
| TNFRSF10B | 362 | 0.89067 | 0.3731062 | 0.52476368 | FALSE |
| MFNG | 2517 | 0.890307 | 0.3733011 | 0.52494518 | FALSE |
| GALNT9 | 7899 | 0.889658 | 0.3736496 | 0.52529348 | FALSE |
| USP12 | 13690 | 0.889374 | 0.3738021 | 0.52536127 | FALSE |
| CASP8 | 7519 | 0.886773 | 0.3752011 | 0.52683796 | FALSE |
| AGT | 11183 | 0.886738 | 0.37522 | 0.52683796 | FALSE |
| PPP1R2 | 14122 | 0.886135 | 0.3755448 | 0.52719932 | FALSE |
| CCND3 | 12425 | 0.88565 | 0.3758062 | 0.52731802 | FALSE |
| DUSP19 | 9169 | 0.88561 | 0.3758277 | 0.52731802 | FALSE |
| MAP3K2 | 14669 | 0.885362 | 0.3759614 | 0.52740532 | FALSE |
| TMEM52 | 9320 | 0.885276 | 0.3760078 | 0.52742304 | FALSE |
| PRF1 | 285 | 0.885135 | 0.3760838 | 0.52743504 | FALSE |
| TRAM1L1 | 1331 | 0.883194 | 0.3771315 | 0.52866716 | FALSE |
| ATP5MC1 | 6135 | 0.88224 | 0.377647 | 0.52910523 | FALSE |
| GAPDHS | 11011 | 0.881286 | 0.378163 | 0.52973323 | FALSE |
| CARMIL1 | 10043 | 0.880939 | 0.3783508 | 0.52985386 | FALSE |
| MAPRE1 | 9688 | 0.880847 | 0.3784006 | 0.52986535 | FALSE |
| WIPI2 | 9983 | 0.880784 | 0.3784347 | 0.52986535 | FALSE |
| RFX2 | 11696 | 0.879582 | 0.3790858 | 0.53050293 | FALSE |
| UBXN1 | 5171 | 0.878152 | 0.3798612 | 0.53125597 | FALSE |
| VDAC3 | 657 | 0.877956 | 0.3799676 | 0.5313089 | FALSE |
| VPS51 | 8551 | 0.877738 | 0.3800859 | 0.5313762 | FALSE |
| ERGIC3 | 4234 | 0.877502 | 0.380214 | 0.5313762 | FALSE |
| DYNLRB2 | 9031 | 0.877499 | 0.3802157 | 0.5313762 | FALSE |
| HSF1 | 4725 | 0.877429 | 0.3802537 | 0.5313762 | FALSE |
| ZNF599 | 13579 | 0.877168 | 0.3803954 | 0.53147924 | FALSE |
| CCM2L | 6198 | 0.877053 | 0.3804579 | 0.531519 | FALSE |
| EVI5L | 2553 | 0.876782 | 0.3806051 | 0.53167714 | FALSE |
| MTMR8 | 1160 | 0.87616 | 0.3809431 | 0.53210175 | FALSE |
| ABCF3 | 7105 | 0.875868 | 0.3811018 | 0.53227592 | FALSE |
| FAM161B | 11923 | 0.875681 | 0.3812035 | 0.53237037 | FALSE |
| RTTN | 8942 | 0.875594 | 0.3812508 | 0.53238889 | FALSE |
| PQLC1 | 11194 | 0.874973 | 0.3815886 | 0.53276543 | FALSE |
| OXLD1 | 14875 | 0.87484 | 0.381661 | 0.53281889 | FALSE |
| COL6A6 | 13585 | 0.874679 | 0.3817486 | 0.53284605 | FALSE |
| CCL17 | 8086 | 0.874129 | 0.382048 | 0.53312117 | FALSE |
| FTHL17 | 13463 | 0.87386 | 0.3821945 | 0.5332304 | FALSE |
| RDH16 | 14014 | 0.873756 | 0.3822511 | 0.53326184 | FALSE |
| MON1B | 1112 | 0.872205 | 0.3830966 | 0.53425053 | FALSE |
| FEM1A | 5738 | 0.872017 | 0.3831991 | 0.53425057 | FALSE |
| COLEC12 | 3488 | 0.870619 | 0.3839622 | 0.53507589 | FALSE |
| LHX8 | 149 | 0.870481 | 0.3840376 | 0.53513324 | FALSE |
| PRKN | 12004 | 0.869555 | 0.3845436 | 0.53574287 | FALSE |
| GNPAT | 14252 | 0.869266 | 0.3847017 | 0.53586979 | FALSE |
| RAB5B | 2803 | 0.869263 | 0.3847033 | 0.53586979 | FALSE |
| KRTAP19-2 | 10395 | 0.868678 | 0.3850233 | 0.53607717 | FALSE |
| BUD13 | 9939 | 0.868562 | 0.3850867 | 0.53607717 | FALSE |
| NDUFA1 | 8267 | 0.868561 | 0.3850873 | 0.53607717 | FALSE |
| BCKDHB | 15299 | 0.868552 | 0.3850922 | 0.53607717 | FALSE |
| TXNIP | 10184 | 0.868347 | 0.3852044 | 0.53618559 | FALSE |
| PLA2G12A | 3565 | 0.867339 | 0.3857563 | 0.53681041 | FALSE |
| PINK1 | 1327 | 0.866674 | 0.3861207 | 0.537174 | FALSE |
| ACTR3BP2 | 13384 | 0.86592 | 0.386534 | 0.5376534 | FALSE |
| PYGO1 | 1952 | 0.864784 | 0.3871574 | 0.53832882 | FALSE |
| LNPK | 7439 | 0.864621 | 0.3872468 | 0.53840536 | FALSE |
| IQCJ | 10519 | 0.864232 | 0.3874605 | 0.53860656 | FALSE |
| LSM6 | 904 | 0.864103 | 0.3875313 | 0.5386169 | FALSE |
| DGUOK | 6728 | 0.862925 | 0.3881787 | 0.53931725 | FALSE |
| HSPB8 | 12775 | 0.862456 | 0.3884366 | 0.53962765 | FALSE |
| GGT8P | 1329 | 0.86215 | 0.388605 | 0.53976558 | FALSE |
| MEI1 | 8374 | 0.860976 | 0.3892513 | 0.54047271 | FALSE |
| CACNG1 | 1869 | 0.860974 | 0.3892524 | 0.54047271 | FALSE |
| RNF126 | 3796 | 0.860397 | 0.3895702 | 0.540818 | FALSE |
| C1orf56 | 495 | 0.859653 | 0.3899804 | 0.5411951 | FALSE |
| AZIN2 | 6297 | 0.858721 | 0.3904945 | 0.54167077 | FALSE |
| LOC100129434 | 2463 | 0.858675 | 0.3905198 | 0.54167077 | FALSE |
| IFITM5 | 7029 | 0.858592 | 0.3905657 | 0.54167077 | FALSE |
| CRLF3 | 11721 | 0.85825 | 0.3907544 | 0.54188451 | FALSE |
| ILDR2 | 12980 | 0.858095 | 0.39084 | 0.5419551 | FALSE |
| CC2D1A | 3513 | 0.857928 | 0.3909322 | 0.54203489 | FALSE |
| STMN3 | 9588 | 0.857039 | 0.3914233 | 0.5426677 | FALSE |
| U2AF1L4 | 10725 | 0.856131 | 0.3919253 | 0.5432673 | FALSE |
| CERS5 | 2874 | 0.855968 | 0.3920155 | 0.54334409 | FALSE |
| XIAP | 7979 | 0.855528 | 0.3922589 | 0.54349808 | FALSE |
| STT3B | 5774 | 0.854535 | 0.3928086 | 0.54402773 | FALSE |
| CTNS | 6681 | 0.854511 | 0.3928219 | 0.54402773 | FALSE |
| GORASP2 | 14655 | 0.853962 | 0.393126 | 0.54430427 | FALSE |
| HYKK | 303 | 0.853504 | 0.3933799 | 0.54455924 | FALSE |
| WARS | 5677 | 0.852833 | 0.3937519 | 0.54502601 | FALSE |
| ETFA | 168 | 0.85267 | 0.3938423 | 0.54510289 | FALSE |
| FAHD2B | 4067 | 0.852502 | 0.3939355 | 0.54512933 | FALSE |
| LINC02550 | 7290 | 0.85237 | 0.3940088 | 0.54514019 | FALSE |
| ZNF721 | 5199 | 0.851676 | 0.3943939 | 0.5455467 | FALSE |
| FMO2 | 9317 | 0.851652 | 0.3944073 | 0.5455467 | FALSE |
| LINC01783 | 3294 | 0.851477 | 0.3945044 | 0.54563283 | FALSE |
| CARD6 | 6407 | 0.85125 | 0.3946305 | 0.54566235 | FALSE |
| PMPCA | 4778 | 0.85113 | 0.3946971 | 0.54570624 | FALSE |
| BHLHB9 | 12519 | 0.850305 | 0.3951555 | 0.54619509 | FALSE |
| HMGN4 | 7248 | 0.850038 | 0.395304 | 0.54633844 | FALSE |
| PMEL | 11775 | 0.849867 | 0.395399 | 0.54633844 | FALSE |
| GPR179 | 1831 | 0.849729 | 0.3954758 | 0.54639618 | FALSE |
| HMOX1 | 8345 | 0.84845 | 0.3961874 | 0.54706402 | FALSE |
| DOPEY1 | 8411 | 0.84842 | 0.3962041 | 0.54706402 | FALSE |
| SNRPA | 13731 | 0.847441 | 0.3967494 | 0.54772013 | FALSE |
| GPN2 | 10922 | 0.846796 | 0.3971088 | 0.54816799 | FALSE |
| LOC441204 | 11446 | 0.846109 | 0.3974919 | 0.54858846 | FALSE |
| DNTTIP1 | 3901 | 0.846061 | 0.3975187 | 0.54858846 | FALSE |
| ASB18 | 4628 | 0.845956 | 0.3975773 | 0.54862087 | FALSE |
| ATP6V0E2-AS1 | 9541 | 0.845206 | 0.3979958 | 0.54885629 | FALSE |
| SLC25A11 | 4528 | 0.845147 | 0.3980288 | 0.54885629 | FALSE |
| PGAM1P5 | 1789 | 0.844873 | 0.3981818 | 0.54895278 | FALSE |
| C6orf226 | 5631 | 0.843592 | 0.3988974 | 0.54974352 | FALSE |
| C9orf78 | 14518 | 0.843374 | 0.3990193 | 0.54974352 | FALSE |
| PRPF40A | 9888 | 0.843303 | 0.399059 | 0.54974352 | FALSE |
| N4BP3 | 6462 | 0.843227 | 0.3991015 | 0.54975361 | FALSE |
| ZNF844 | 2723 | 0.842788 | 0.399347 | 0.54999489 | FALSE |
| TAB1 | 14574 | 0.842698 | 0.3993974 | 0.55001577 | FALSE |
| DBX1 | 1940 | 0.842505 | 0.3995053 | 0.55011601 | FALSE |
| PRM3 | 9858 | 0.842108 | 0.3997275 | 0.55032497 | FALSE |
| LOC100289361 | 11824 | 0.84185 | 0.3998719 | 0.55042685 | FALSE |
| PYGB | 5103 | 0.841653 | 0.3999822 | 0.55048173 | FALSE |
| IFNL3 | 8569 | 0.841094 | 0.4002953 | 0.55071865 | FALSE |
| FAM219B | 4013 | 0.840508 | 0.4006236 | 0.55104186 | FALSE |
| MCCC2 | 4307 | 0.840254 | 0.400766 | 0.55114301 | FALSE |
| YIF1B | 6707 | 0.840229 | 0.40078 | 0.55114301 | FALSE |
| CA5A | 3641 | 0.840045 | 0.4008832 | 0.55123638 | FALSE |
| KIAA1328 | 8110 | 0.839876 | 0.4009779 | 0.55131818 | FALSE |
| GH1 | 2727 | 0.839674 | 0.4010912 | 0.55137695 | FALSE |
| POP7 | 4358 | 0.839571 | 0.401149 | 0.55140788 | FALSE |
| KLK2 | 14920 | 0.838669 | 0.4016551 | 0.55186095 | FALSE |
| USP7 | 4045 | 0.838417 | 0.4017965 | 0.55195085 | FALSE |
| NACAP1 | 2027 | 0.83834 | 0.4018398 | 0.55195085 | FALSE |
| SLITRK6 | 7883 | 0.838288 | 0.401869 | 0.55195085 | FALSE |
| TRIM10 | 9713 | 0.838238 | 0.401897 | 0.55195085 | FALSE |
| ENPP4 | 12780 | 0.83733 | 0.4024071 | 0.55255426 | FALSE |
| KISS1 | 14671 | 0.83546 | 0.4034588 | 0.5537065 | FALSE |
| PCDHGA12 | 1600 | 0.835143 | 0.4036372 | 0.55390277 | FALSE |
| ARHGAP5 | 10074 | 0.835056 | 0.4036862 | 0.55392136 | FALSE |
| CCDC141 | 613 | 0.833295 | 0.4046784 | 0.55494185 | FALSE |
| HPRT1 | 6726 | 0.833164 | 0.4047522 | 0.55499446 | FALSE |
| TEX264 | 3438 | 0.832223 | 0.4052831 | 0.55562799 | FALSE |
| BMP6 | 6095 | 0.832157 | 0.4053203 | 0.55562799 | FALSE |
| BAG4 | 8876 | 0.831108 | 0.4059126 | 0.5562928 | FALSE |
| EVPLL | 10295 | 0.829941 | 0.4065721 | 0.55705016 | FALSE |
| HELZ2 | 2384 | 0.829577 | 0.406778 | 0.55723451 | FALSE |
| MGME1 | 1585 | 0.829317 | 0.4069251 | 0.55734915 | FALSE |
| MED15 | 1886 | 0.82916 | 0.4070139 | 0.55736772 | FALSE |
| MCAT | 8744 | 0.829153 | 0.4070178 | 0.55736772 | FALSE |
| GPR78 | 5190 | 0.82863 | 0.4073138 | 0.55760709 | FALSE |
| FAM102A | 13782 | 0.828019 | 0.4076597 | 0.55790464 | FALSE |
| LBX1 | 13179 | 0.827614 | 0.4078891 | 0.55816972 | FALSE |
| SEC23B | 13329 | 0.827518 | 0.4079435 | 0.55819528 | FALSE |
| ACRBP | 3731 | 0.827168 | 0.4081418 | 0.55841777 | FALSE |
| FXYD7 | 6824 | 0.826979 | 0.408249 | 0.55851545 | FALSE |
| CSMD3 | 56 | 0.826545 | 0.408495 | 0.55870537 | FALSE |
| ADAMTS8 | 6667 | 0.825968 | 0.4088222 | 0.55905512 | FALSE |
| RBM43 | 15443 | 0.825607 | 0.4090271 | 0.55928628 | FALSE |
| ARGLU1 | 4122 | 0.824501 | 0.4096549 | 0.56004686 | FALSE |
| EFCAB12 | 9152 | 0.82434 | 0.4097464 | 0.56012289 | FALSE |
| LOC103344931 | 2666 | 0.823519 | 0.4102129 | 0.56061356 | FALSE |
| CATSPERE | 1471 | 0.823377 | 0.4102936 | 0.56064062 | FALSE |
| WDFY2 | 6385 | 0.82314 | 0.4104284 | 0.56076096 | FALSE |
| PPP1R9A | 2692 | 0.822621 | 0.4107236 | 0.56111521 | FALSE |
| AFDN-DT | 4098 | 0.8219 | 0.4111338 | 0.56147942 | FALSE |
| PRKDC | 4030 | 0.821536 | 0.411341 | 0.56171334 | FALSE |
| TAAR8 | 12691 | 0.820911 | 0.411697 | 0.56206524 | FALSE |
| COL18A1 | 6017 | 0.820326 | 0.4120303 | 0.56235983 | FALSE |
| CLDN4 | 14017 | 0.820225 | 0.4120879 | 0.5623893 | FALSE |
| LINC00665 | 3423 | 0.820014 | 0.4122081 | 0.56250433 | FALSE |
| BMP7 | 2064 | 0.818745 | 0.4129319 | 0.56331314 | FALSE |
| ERC1 | 7312 | 0.818664 | 0.4129781 | 0.56331314 | FALSE |
| NAT16 | 8319 | 0.818659 | 0.412981 | 0.56331314 | FALSE |
| NLN | 10946 | 0.818286 | 0.4131939 | 0.56345606 | FALSE |
| IL21R | 2658 | 0.817722 | 0.4135159 | 0.56374758 | FALSE |
| DNAJB13 | 9581 | 0.81772 | 0.4135171 | 0.56374758 | FALSE |
| PHOX2A | 14540 | 0.817659 | 0.4135519 | 0.56374758 | FALSE |
| AJAP1 | 9859 | 0.81716 | 0.413837 | 0.564087 | FALSE |
| RAB6D | 8980 | 0.816667 | 0.4141188 | 0.56430619 | FALSE |
| CDHR3 | 2120 | 0.816626 | 0.4141422 | 0.56430619 | FALSE |
| SFXN4 | 15303 | 0.815975 | 0.4145144 | 0.56476418 | FALSE |
| CYP26C1 | 2726 | 0.815887 | 0.4145648 | 0.56478353 | FALSE |
| PRR35 | 1992 | 0.815767 | 0.4146334 | 0.56482782 | FALSE |
| WDR46 | 14723 | 0.815614 | 0.4147209 | 0.56489784 | FALSE |
| CCL21 | 14792 | 0.815373 | 0.4148588 | 0.56499086 | FALSE |
| RBMX2 | 1126 | 0.815242 | 0.4149338 | 0.56499086 | FALSE |
| ZNF286B | 15292 | 0.814641 | 0.4152778 | 0.56541007 | FALSE |
| KSR1 | 2779 | 0.814429 | 0.4153992 | 0.56543775 | FALSE |
| TMEM151A | 9473 | 0.813207 | 0.4160994 | 0.56611299 | FALSE |
| TTTY15 | 9166 | 0.812716 | 0.4163809 | 0.56624726 | FALSE |
| C2orf74 | 10526 | 0.812704 | 0.4163878 | 0.56624726 | FALSE |
| NDUFS8 | 1158 | 0.812683 | 0.4163998 | 0.56624726 | FALSE |
| FAM83H | 4038 | 0.812202 | 0.4166757 | 0.56639483 | FALSE |
| LIN28B-AS1 | 12793 | 0.812178 | 0.4166895 | 0.56639483 | FALSE |
| SDK1 | 4757 | 0.812086 | 0.4167423 | 0.56641733 | FALSE |
| PTCRA | 2189 | 0.811523 | 0.4170654 | 0.56675793 | FALSE |
| LINC00842 | 10010 | 0.811304 | 0.4171911 | 0.56686912 | FALSE |
| SYCN | 13076 | 0.811207 | 0.4172468 | 0.56686912 | FALSE |
| PNPLA1 | 732 | 0.810895 | 0.417426 | 0.56705077 | FALSE |
| GJB3 | 7228 | 0.810816 | 0.4174713 | 0.56706313 | FALSE |
| CDKL3 | 15273 | 0.810066 | 0.4179022 | 0.56759911 | FALSE |
| KRT81 | 11859 | 0.808685 | 0.4186964 | 0.5685295 | FALSE |
| SLC7A2 | 7819 | 0.808598 | 0.4187464 | 0.56854809 | FALSE |
| TMBIM6 | 12115 | 0.807784 | 0.4192149 | 0.56907972 | FALSE |
| NINL | 9114 | 0.807728 | 0.4192472 | 0.56907972 | FALSE |
| GPR156 | 5057 | 0.806821 | 0.4197696 | 0.56955647 | FALSE |
| C6orf201 | 4129 | 0.806563 | 0.4199183 | 0.56964446 | FALSE |
| DPH6 | 8947 | 0.805599 | 0.4204741 | 0.57034895 | FALSE |
| AXIN2 | 5146 | 0.805388 | 0.4205958 | 0.57042992 | FALSE |
| GOLGA5 | 12522 | 0.805059 | 0.4207856 | 0.57062299 | FALSE |
| FGD2 | 1730 | 0.804485 | 0.4211169 | 0.57101604 | FALSE |
| ARRDC1 | 13971 | 0.803781 | 0.4215235 | 0.57122714 | FALSE |
| GPR155 | 3803 | 0.803605 | 0.4216251 | 0.57127449 | FALSE |
| OGFOD1 | 13114 | 0.802729 | 0.4221314 | 0.57185268 | FALSE |
| LOC100240735 | 647 | 0.802654 | 0.4221747 | 0.57186187 | FALSE |
| FLVCR1 | 3983 | 0.802589 | 0.4222123 | 0.57186322 | FALSE |
| ATP6V0A1 | 10807 | 0.802171 | 0.422454 | 0.57199141 | FALSE |
| LINC01399 | 6734 | 0.802109 | 0.4224899 | 0.57199141 | FALSE |
| TBCD | 9383 | 0.801869 | 0.4226287 | 0.57208028 | FALSE |
| INCENP | 6864 | 0.801757 | 0.4226935 | 0.57211845 | FALSE |
| CYFIP2 | 7426 | 0.801492 | 0.4228469 | 0.5722269 | FALSE |
| NIT1 | 408 | 0.801096 | 0.4230761 | 0.57243796 | FALSE |
| CAV2 | 3323 | 0.799371 | 0.4240753 | 0.57357236 | FALSE |
| PAWR | 9135 | 0.799332 | 0.4240979 | 0.57357236 | FALSE |
| LPCAT3 | 2245 | 0.799228 | 0.4241582 | 0.57360428 | FALSE |
| CEP19 | 11709 | 0.799034 | 0.4242707 | 0.57361458 | FALSE |
| LTA | 14691 | 0.799025 | 0.4242759 | 0.57361458 | FALSE |
| PLIN2 | 4696 | 0.797658 | 0.425069 | 0.57448806 | FALSE |
| STARD3 | 15421 | 0.796604 | 0.4256811 | 0.57522525 | FALSE |
| SMIM1 | 11630 | 0.796592 | 0.425688 | 0.57522525 | FALSE |
| VSX1 | 4626 | 0.796287 | 0.4258652 | 0.57536525 | FALSE |
| RAB12 | 1670 | 0.795849 | 0.4261198 | 0.57551416 | FALSE |
| ENTPD2 | 14761 | 0.795844 | 0.4261227 | 0.57551416 | FALSE |
| HMHB1 | 8048 | 0.793677 | 0.4273835 | 0.57701755 | FALSE |
| CAPZA1 | 5130 | 0.793581 | 0.4274394 | 0.57704319 | FALSE |
| SIRPB1 | 2992 | 0.793324 | 0.4275891 | 0.57714558 | FALSE |
| SERPINB1 | 12090 | 0.793014 | 0.4277697 | 0.57728964 | FALSE |
| DYTN | 1430 | 0.792659 | 0.4279765 | 0.57751895 | FALSE |
| SRPRB | 14657 | 0.79251 | 0.4280634 | 0.57753721 | FALSE |
| CXCL14 | 7034 | 0.792509 | 0.4280639 | 0.57753721 | FALSE |
| CBFA2T3 | 844 | 0.792372 | 0.4281438 | 0.57754526 | FALSE |
| CLIC1 | 15502 | 0.791915 | 0.4284102 | 0.57767375 | FALSE |
| TP53RK | 9866 | 0.791856 | 0.4284446 | 0.57767375 | FALSE |
| MRPL40 | 6965 | 0.791765 | 0.4284977 | 0.57767375 | FALSE |
| SRP19 | 2163 | 0.79162 | 0.4285823 | 0.57773793 | FALSE |
| SP110 | 14403 | 0.791272 | 0.4287853 | 0.57791192 | FALSE |
| GYG1 | 3483 | 0.790754 | 0.4290876 | 0.57821962 | FALSE |
| SERPINI1 | 5020 | 0.790096 | 0.4294717 | 0.5786874 | FALSE |
| PGS1 | 2020 | 0.789935 | 0.4295657 | 0.5787642 | FALSE |
| HBB | 5735 | 0.789104 | 0.4300512 | 0.57936839 | FALSE |
| PRSS27 | 10842 | 0.788984 | 0.4301214 | 0.57941294 | FALSE |
| PLEKHA2 | 9575 | 0.788588 | 0.4303528 | 0.57966032 | FALSE |
| OAS1 | 11780 | 0.788543 | 0.4303792 | 0.57966032 | FALSE |
| PDE6H | 12996 | 0.787745 | 0.4308459 | 0.58018896 | FALSE |
| NME9 | 12149 | 0.787351 | 0.4310764 | 0.58043488 | FALSE |
| LOC108783654 | 5129 | 0.787175 | 0.4311794 | 0.58048812 | FALSE |
| TMEM185B | 8523 | 0.786441 | 0.4316092 | 0.58096661 | FALSE |
| GAS2L1P2 | 10745 | 0.786069 | 0.4318271 | 0.58120986 | FALSE |
| PCDHB4 | 3871 | 0.785736 | 0.4320222 | 0.58141277 | FALSE |
| MYADML | 7016 | 0.785476 | 0.4321745 | 0.58141277 | FALSE |
| CHAD | 1420 | 0.785463 | 0.4321822 | 0.58141277 | FALSE |
| TIPARP | 14898 | 0.785329 | 0.4322607 | 0.58141277 | FALSE |
| CYB561A3 | 1003 | 0.785304 | 0.4322754 | 0.58141277 | FALSE |
| BNIP1 | 1310 | 0.785198 | 0.4323375 | 0.58144631 | FALSE |
| SHOX | 5239 | 0.784764 | 0.432592 | 0.58168775 | FALSE |
| RC3H2 | 6931 | 0.784667 | 0.4326488 | 0.58168775 | FALSE |
| EHD1 | 9229 | 0.78456 | 0.4327116 | 0.58169223 | FALSE |
| RBSN | 14573 | 0.784244 | 0.432897 | 0.58179832 | FALSE |
| CCDC74B | 4101 | 0.784156 | 0.4329486 | 0.58181769 | FALSE |
| STK31 | 4679 | 0.78395 | 0.4330694 | 0.58193009 | FALSE |
| ASPH | 3811 | 0.783108 | 0.4335637 | 0.5822439 | FALSE |
| DPEP3 | 3969 | 0.782995 | 0.43363 | 0.58225742 | FALSE |
| LMNA | 12132 | 0.782964 | 0.4336483 | 0.58225742 | FALSE |
| LONP2 | 13601 | 0.782884 | 0.4336952 | 0.58227049 | FALSE |
| BMP2K | 13016 | 0.782787 | 0.4337522 | 0.58229697 | FALSE |
| AVL9 | 4173 | 0.782477 | 0.4339343 | 0.58248161 | FALSE |
| LOC100126784 | 9758 | 0.781424 | 0.4345532 | 0.58303325 | FALSE |
| AKR1C1 | 10409 | 0.781394 | 0.4345708 | 0.58303325 | FALSE |
| VAV2 | 8829 | 0.781346 | 0.434599 | 0.58303325 | FALSE |
| SLC25A28 | 5312 | 0.780992 | 0.4348072 | 0.58323137 | FALSE |
| GNAO1 | 12676 | 0.780968 | 0.4348213 | 0.58323137 | FALSE |
| MYMK | 11663 | 0.78071 | 0.4349731 | 0.58327342 | FALSE |
| MNX1-AS1 | 13422 | 0.780434 | 0.4351355 | 0.58340246 | FALSE |
| MPG | 2268 | 0.780011 | 0.4353844 | 0.58368616 | FALSE |
| ATAD3A | 14836 | 0.779857 | 0.4354751 | 0.58370251 | FALSE |
| VSTM4 | 98 | 0.7798 | 0.4355086 | 0.58370251 | FALSE |
| AMN | 6909 | 0.779295 | 0.435806 | 0.58400091 | FALSE |
| SNHG5 | 6009 | 0.778457 | 0.4362996 | 0.58448225 | FALSE |
| SLC25A33 | 5663 | 0.778368 | 0.4363521 | 0.58448225 | FALSE |
| OXT | 11571 | 0.777911 | 0.4366215 | 0.58471534 | FALSE |
| MGRN1 | 952 | 0.7764 | 0.4375128 | 0.58540395 | FALSE |
| GFRA4 | 2085 | 0.776376 | 0.437527 | 0.58540395 | FALSE |
| LTBP1 | 13679 | 0.775212 | 0.4382144 | 0.58612301 | FALSE |
| CR2 | 2838 | 0.774398 | 0.4386955 | 0.58661588 | FALSE |
| C1QL2 | 5862 | 0.773843 | 0.4390236 | 0.5869543 | FALSE |
| MED4 | 879 | 0.773757 | 0.4390745 | 0.5869721 | FALSE |
| COL6A3 | 2653 | 0.773575 | 0.4391822 | 0.58706582 | FALSE |
| UBR1 | 8793 | 0.77312 | 0.4394514 | 0.58737545 | FALSE |
| PLK3 | 14817 | 0.772483 | 0.4398284 | 0.5877789 | FALSE |
| C11orf74 | 7141 | 0.771746 | 0.4402649 | 0.58821134 | FALSE |
| DKC1 | 9958 | 0.771661 | 0.4403152 | 0.58822834 | FALSE |
| TNIP2 | 10360 | 0.770309 | 0.4411166 | 0.58909757 | FALSE |
| ZFP2 | 4246 | 0.769022 | 0.4418803 | 0.5899158 | FALSE |
| VSIR | 4690 | 0.768886 | 0.441961 | 0.5899732 | FALSE |
| PGAM4 | 11017 | 0.767034 | 0.4430613 | 0.5909374 | FALSE |
| WRNIP1 | 8369 | 0.76528 | 0.4441048 | 0.59205235 | FALSE |
| XRCC1 | 12111 | 0.765247 | 0.4441245 | 0.59205235 | FALSE |
| PPP6R1 | 5231 | 0.76492 | 0.4443192 | 0.59216043 | FALSE |
| AADACL3 | 82 | 0.764611 | 0.4445032 | 0.59229946 | FALSE |
| MFSD9 | 871 | 0.76459 | 0.4445157 | 0.59229946 | FALSE |
| OLMALINC | 2738 | 0.764554 | 0.4445372 | 0.59229946 | FALSE |
| WDR3 | 10261 | 0.764476 | 0.4445836 | 0.59231089 | FALSE |
| ATP5MC2 | 7164 | 0.763447 | 0.4451969 | 0.5930673 | FALSE |
| SFN | 14572 | 0.763396 | 0.4452273 | 0.5930673 | FALSE |
| EPHX3 | 182 | 0.761808 | 0.4461746 | 0.59413864 | FALSE |
| SLC22A4 | 9706 | 0.761793 | 0.4461836 | 0.59413864 | FALSE |
| NOTUM | 5441 | 0.760656 | 0.4468626 | 0.59478948 | FALSE |
| KRBOX1 | 10364 | 0.760185 | 0.447144 | 0.59506277 | FALSE |
| ST8SIA3 | 11077 | 0.757706 | 0.448627 | 0.59667345 | FALSE |
| TSSC4 | 13355 | 0.757524 | 0.448736 | 0.59667345 | FALSE |
| SLC8B1 | 8078 | 0.757182 | 0.4489408 | 0.59679752 | FALSE |
| PIGH | 11515 | 0.756811 | 0.4491631 | 0.59690373 | FALSE |
| SRGN | 14226 | 0.756725 | 0.4492146 | 0.59690373 | FALSE |
| TEX44 | 9529 | 0.756629 | 0.4492722 | 0.59692943 | FALSE |
| COG4 | 6356 | 0.756168 | 0.4495485 | 0.59719505 | FALSE |
| SUCO | 9110 | 0.755941 | 0.4496846 | 0.59722996 | FALSE |
| APOA1 | 15616 | 0.755933 | 0.4496894 | 0.59722996 | FALSE |
| PYY2 | 10326 | 0.754955 | 0.450276 | 0.59785667 | FALSE |
| SEC22C | 5836 | 0.754658 | 0.4504542 | 0.59798159 | FALSE |
| MUC12 | 8205 | 0.754514 | 0.4505406 | 0.59800491 | FALSE |
| SEBOX | 6962 | 0.753963 | 0.4508714 | 0.59829159 | FALSE |
| SCD | 7392 | 0.753457 | 0.4511753 | 0.59864404 | FALSE |
| KRT85 | 9212 | 0.753114 | 0.4513814 | 0.59881582 | FALSE |
| ADARB2-AS1 | 10949 | 0.752961 | 0.4514734 | 0.59882939 | FALSE |
| LINC00674 | 2492 | 0.752858 | 0.4515352 | 0.59882939 | FALSE |
| SH3GL2 | 464 | 0.752593 | 0.4516945 | 0.59896212 | FALSE |
| ZNF428 | 11889 | 0.751517 | 0.4523416 | 0.59968248 | FALSE |
| PSMG4 | 3290 | 0.751409 | 0.4524065 | 0.59971776 | FALSE |
| MRPS12 | 1490 | 0.751269 | 0.4524908 | 0.5997317 | FALSE |
| ARL14EP | 9606 | 0.751264 | 0.4524938 | 0.5997317 | FALSE |
| LINC01672 | 12471 | 0.749425 | 0.4536011 | 0.60109738 | FALSE |
| STX3 | 11408 | 0.749241 | 0.453712 | 0.60119335 | FALSE |
| RHBDD3 | 68 | 0.749154 | 0.4537644 | 0.60121186 | FALSE |
| CD2BP2 | 5727 | 0.748777 | 0.4539916 | 0.6013482 | FALSE |
| LINC01023 | 4170 | 0.748728 | 0.4540212 | 0.6013482 | FALSE |
| IRX3 | 12614 | 0.74821 | 0.4543335 | 0.60165994 | FALSE |
| JPH1 | 5214 | 0.74795 | 0.4544903 | 0.60181663 | FALSE |
| PARK7 | 1066 | 0.747631 | 0.4546828 | 0.601953 | FALSE |
| SYNPO2L | 10936 | 0.747569 | 0.4547202 | 0.601953 | FALSE |
| SLC22A3 | 5293 | 0.747067 | 0.4550231 | 0.60208969 | FALSE |
| DHH | 14739 | 0.747024 | 0.4550491 | 0.60208969 | FALSE |
| FBLN1 | 1645 | 0.74697 | 0.4550817 | 0.60208969 | FALSE |
| LHX2 | 10020 | 0.74689 | 0.45513 | 0.60210262 | FALSE |
| GPRASP1 | 8245 | 0.746806 | 0.4551807 | 0.60211876 | FALSE |
| SELENOF | 6185 | 0.746737 | 0.4552223 | 0.60212291 | FALSE |
| LTBR | 14689 | 0.746422 | 0.4554125 | 0.60222323 | FALSE |
| BNIP2 | 10542 | 0.74642 | 0.4554137 | 0.60222323 | FALSE |
| AFAP1 | 8330 | 0.746064 | 0.4556288 | 0.60233012 | FALSE |
| EVX1 | 10662 | 0.745532 | 0.4559502 | 0.60262674 | FALSE |
| PSORS1C2 | 12876 | 0.745373 | 0.4560463 | 0.60266781 | FALSE |
| DEFB132 | 12710 | 0.744032 | 0.4568571 | 0.60351929 | FALSE |
| WNT6 | 8456 | 0.743802 | 0.4569963 | 0.60357648 | FALSE |
| RIPPLY1 | 1953 | 0.743219 | 0.4573491 | 0.60391406 | FALSE |
| STK19 | 2773 | 0.742933 | 0.4575222 | 0.60404064 | FALSE |
| MYH3 | 5682 | 0.742588 | 0.4577312 | 0.60426346 | FALSE |
| ATP23 | 14126 | 0.742419 | 0.4578335 | 0.60426346 | FALSE |
| MYPOP | 7841 | 0.742299 | 0.4579062 | 0.60429238 | FALSE |
| IL26 | 7465 | 0.741933 | 0.4581279 | 0.60442952 | FALSE |
| SERPINA6 | 9563 | 0.741894 | 0.4581516 | 0.60442952 | FALSE |
| TCEAL1 | 14379 | 0.741617 | 0.4583194 | 0.60442952 | FALSE |
| FAM98A | 13164 | 0.741231 | 0.4585534 | 0.60468706 | FALSE |
| ZFAND2B | 6457 | 0.740906 | 0.4587504 | 0.60489588 | FALSE |
| EPHA6 | 5136 | 0.740769 | 0.4588335 | 0.6049544 | FALSE |
| TAPT1-AS1 | 5813 | 0.74033 | 0.4590998 | 0.60520338 | FALSE |
| TMEM272 | 13506 | 0.740224 | 0.4591641 | 0.60523711 | FALSE |
| KIRREL2 | 819 | 0.740131 | 0.4592205 | 0.60526045 | FALSE |
| LIPC | 1807 | 0.739965 | 0.4593212 | 0.60534217 | FALSE |
| PTCH2 | 8657 | 0.739387 | 0.459672 | 0.60565132 | FALSE |
| HSDL1 | 2614 | 0.738864 | 0.4599896 | 0.60592466 | FALSE |
| LCMT2 | 9961 | 0.738811 | 0.4600218 | 0.60592466 | FALSE |
| STXBP5 | 7938 | 0.73879 | 0.4600345 | 0.60592466 | FALSE |
| THBD | 8434 | 0.738657 | 0.4601153 | 0.60597999 | FALSE |
| RNGTT | 1271 | 0.737761 | 0.4606597 | 0.60639045 | FALSE |
| TEX13B | 8102 | 0.736815 | 0.4612349 | 0.60704534 | FALSE |
| FRMD1 | 8392 | 0.73553 | 0.4620168 | 0.60797208 | FALSE |
| MYO3A | 8590 | 0.735419 | 0.4620844 | 0.60800982 | FALSE |
| KRTAP5-10 | 7855 | 0.73505 | 0.462309 | 0.60820309 | FALSE |
| GRIN2C | 11970 | 0.734846 | 0.4624333 | 0.60831535 | FALSE |
| CHTF18 | 8175 | 0.734424 | 0.4626904 | 0.60849991 | FALSE |
| HHLA3 | 11239 | 0.733851 | 0.4630396 | 0.6088055 | FALSE |
| CFAP161 | 5215 | 0.733273 | 0.4633919 | 0.60905056 | FALSE |
| COX6B2 | 15490 | 0.733205 | 0.4634334 | 0.60905056 | FALSE |
| PPIF | 8148 | 0.733098 | 0.4634987 | 0.60905056 | FALSE |
| CLTB | 8320 | 0.732796 | 0.4636829 | 0.6091902 | FALSE |
| HLA-E | 6969 | 0.732667 | 0.4637616 | 0.60924239 | FALSE |
| HRH3 | 12880 | 0.732363 | 0.463947 | 0.60937491 | FALSE |
| KRTAP5-AS1 | 5945 | 0.73231 | 0.4639794 | 0.60937491 | FALSE |
| GIT1 | 4991 | 0.732022 | 0.4641551 | 0.60950334 | FALSE |
| KRT9 | 11206 | 0.731931 | 0.4642107 | 0.60952508 | FALSE |
| SLC25A47 | 8487 | 0.731803 | 0.4642888 | 0.60957648 | FALSE |
| THOC6 | 9165 | 0.731509 | 0.4644683 | 0.60974659 | FALSE |
| PCDH1 | 376 | 0.731463 | 0.4644964 | 0.60974659 | FALSE |
| SLC25A39 | 9197 | 0.729795 | 0.4655155 | 0.61093048 | FALSE |
| VAC14 | 1817 | 0.729551 | 0.4656647 | 0.61102367 | FALSE |
| MYEOV | 8122 | 0.728769 | 0.466143 | 0.6115486 | FALSE |
| HOXD8 | 12388 | 0.728568 | 0.466266 | 0.61160729 | FALSE |
| TNFRSF13C | 13869 | 0.727742 | 0.4667715 | 0.6122191 | FALSE |
| SLC25A53 | 6908 | 0.727563 | 0.4668811 | 0.61226741 | FALSE |
| PPP6R2 | 740 | 0.727501 | 0.4669191 | 0.61226741 | FALSE |
| RPL26L1 | 14361 | 0.72678 | 0.4673607 | 0.61268347 | FALSE |
| MAPK12 | 6608 | 0.726562 | 0.4674943 | 0.6128072 | FALSE |
| MMP17 | 13998 | 0.726251 | 0.4676849 | 0.61295425 | FALSE |
| LINC00304 | 3691 | 0.726134 | 0.4677566 | 0.61299685 | FALSE |
| MIEF1 | 8888 | 0.726035 | 0.4678173 | 0.61302499 | FALSE |
| CDH2 | 12707 | 0.725527 | 0.4681288 | 0.61333032 | FALSE |
| DHX30 | 513 | 0.72482 | 0.4685625 | 0.61379563 | FALSE |
| VSTM2B | 7941 | 0.724482 | 0.4687699 | 0.61401587 | FALSE |
| FCN3 | 1240 | 0.724156 | 0.46897 | 0.61417505 | FALSE |
| BOD1 | 11261 | 0.723506 | 0.4693691 | 0.61460359 | FALSE |
| GPX1 | 11758 | 0.723229 | 0.4695392 | 0.61471455 | FALSE |
| ST7 | 2285 | 0.723161 | 0.469581 | 0.61471775 | FALSE |
| COL22A1 | 9294 | 0.723018 | 0.4696688 | 0.61473221 | FALSE |
| INCA1 | 12194 | 0.723015 | 0.4696707 | 0.61473221 | FALSE |
| TRIM69 | 4160 | 0.721637 | 0.4705177 | 0.61563466 | FALSE |
| CAMKMT | 5155 | 0.721039 | 0.4708855 | 0.61601283 | FALSE |
| SNAPIN | 4081 | 0.720946 | 0.4709427 | 0.61603362 | FALSE |
| CWC27 | 2943 | 0.7208 | 0.4710326 | 0.61603362 | FALSE |
| INAFM1 | 10199 | 0.72079 | 0.4710387 | 0.61603362 | FALSE |
| MAP3K5 | 714 | 0.720539 | 0.4711932 | 0.61615753 | FALSE |
| RRAS | 7756 | 0.719772 | 0.4716654 | 0.61652278 | FALSE |
| PREX2 | 2710 | 0.719765 | 0.4716697 | 0.61652278 | FALSE |
| RHBDL1 | 10688 | 0.71884 | 0.4722395 | 0.61705552 | FALSE |
| FUT8-AS1 | 12690 | 0.718833 | 0.4722438 | 0.61705552 | FALSE |
| NMUR1 | 2607 | 0.71849 | 0.4724552 | 0.6171883 | FALSE |
| PLEKHB1 | 9963 | 0.717724 | 0.4729275 | 0.6176445 | FALSE |
| PPM1F | 12099 | 0.717604 | 0.4730015 | 0.6176445 | FALSE |
| TRIM72 | 970 | 0.717578 | 0.4730175 | 0.6176445 | FALSE |
| DHRS4 | 4675 | 0.717366 | 0.4731483 | 0.6176445 | FALSE |
| GPR119 | 10279 | 0.716769 | 0.4735166 | 0.61790365 | FALSE |
| PPIL1 | 3 | 0.716307 | 0.4738018 | 0.61822416 | FALSE |
| TCTE3 | 7770 | 0.716207 | 0.4738635 | 0.61825311 | FALSE |
| SARS | 5326 | 0.715075 | 0.4745627 | 0.61911364 | FALSE |
| CXCL2 | 930 | 0.714597 | 0.4748581 | 0.61929229 | FALSE |
| VRK1 | 1650 | 0.713764 | 0.4753731 | 0.61980885 | FALSE |
| CCNF | 12349 | 0.711958 | 0.4764908 | 0.6210589 | FALSE |
| APH1B | 4941 | 0.711843 | 0.476562 | 0.62109994 | FALSE |
| TMEM63B | 3781 | 0.711708 | 0.4766456 | 0.62115712 | FALSE |
| TBC1D31 | 7012 | 0.711616 | 0.4767026 | 0.6211796 | FALSE |
| FAM53C | 10175 | 0.711112 | 0.4770148 | 0.62148287 | FALSE |
| C10orf95 | 13133 | 0.710996 | 0.4770867 | 0.62152472 | FALSE |
| TMIGD2 | 10066 | 0.710801 | 0.4772076 | 0.62163035 | FALSE |
| TMEM37 | 7674 | 0.710599 | 0.4773328 | 0.62174163 | FALSE |
| ISM2 | 6899 | 0.710526 | 0.477378 | 0.62174877 | FALSE |
| NDST3 | 3917 | 0.710221 | 0.4775671 | 0.62189142 | FALSE |
| C14orf119 | 5502 | 0.710065 | 0.4776638 | 0.62195818 | FALSE |
| ZNF814 | 9540 | 0.71001 | 0.4776979 | 0.62195818 | FALSE |
| CDK15 | 4125 | 0.709461 | 0.4780384 | 0.62234969 | FALSE |
| SRD5A1 | 2544 | 0.709343 | 0.4781117 | 0.62239316 | FALSE |
| PSIP1 | 224 | 0.708741 | 0.4784852 | 0.62277574 | FALSE |
| SLC35F5 | 8395 | 0.708664 | 0.478533 | 0.62278609 | FALSE |
| BAP1 | 7074 | 0.708461 | 0.478659 | 0.62279697 | FALSE |
| RAB3D | 3902 | 0.708092 | 0.4788881 | 0.62301075 | FALSE |
| SF3A3 | 9512 | 0.708065 | 0.4789049 | 0.62301075 | FALSE |
| OR2Z1 | 22 | 0.706666 | 0.4797741 | 0.62388188 | FALSE |
| FAM114A2 | 11763 | 0.706203 | 0.4800619 | 0.62411822 | FALSE |
| KIAA1143 | 10973 | 0.705966 | 0.4802093 | 0.62418822 | FALSE |
| MRPS7 | 7630 | 0.705763 | 0.4803355 | 0.62428797 | FALSE |
| GGN | 15047 | 0.705464 | 0.4805215 | 0.62435393 | FALSE |
| PTEN | 5726 | 0.70544 | 0.4805365 | 0.62435393 | FALSE |
| LOC389641 | 8641 | 0.70528 | 0.480636 | 0.62437948 | FALSE |
| COA7 | 4944 | 0.705129 | 0.48073 | 0.62444965 | FALSE |
| ARID3A | 12917 | 0.7043 | 0.481246 | 0.62486032 | FALSE |
| CDK5RAP1 | 8167 | 0.70407 | 0.4813892 | 0.62499436 | FALSE |
| RGS9BP | 3932 | 0.703534 | 0.481723 | 0.62522737 | FALSE |
| NUBP1 | 9883 | 0.703445 | 0.4817785 | 0.62524015 | FALSE |
| ZNF746 | 3367 | 0.703235 | 0.4819093 | 0.62527444 | FALSE |
| FAM234B | 1467 | 0.702236 | 0.482532 | 0.62595824 | FALSE |
| KCNQ2 | 1694 | 0.702073 | 0.4826336 | 0.62603814 | FALSE |
| DLGAP3 | 14687 | 0.701899 | 0.4827422 | 0.626075 | FALSE |
| PTPN22 | 1136 | 0.701692 | 0.4828713 | 0.62613856 | FALSE |
| DERA | 8927 | 0.701399 | 0.483054 | 0.62632362 | FALSE |
| CA8 | 9490 | 0.700883 | 0.483376 | 0.62667843 | FALSE |
| CD79A | 352 | 0.700832 | 0.4834079 | 0.62667843 | FALSE |
| TIGD4 | 6781 | 0.700669 | 0.4835096 | 0.62669713 | FALSE |
| NUP88 | 5697 | 0.700597 | 0.4835546 | 0.62669713 | FALSE |
| AQP7P1 | 10095 | 0.700552 | 0.4835826 | 0.62669713 | FALSE |
| KCTD5 | 2248 | 0.700298 | 0.4837412 | 0.62674677 | FALSE |
| VTI1A | 4382 | 0.699322 | 0.4843508 | 0.62732862 | FALSE |
| EXOC3L1 | 12739 | 0.699219 | 0.4844152 | 0.62735999 | FALSE |
| XRCC3 | 4907 | 0.698793 | 0.4846814 | 0.62765278 | FALSE |
| THAP6 | 6600 | 0.69863 | 0.4847833 | 0.62769376 | FALSE |
| HOXA5 | 462 | 0.698508 | 0.4848596 | 0.62769376 | FALSE |
| TOR1A | 4625 | 0.698484 | 0.4848746 | 0.62769376 | FALSE |
| PCMTD2 | 4058 | 0.698431 | 0.4849077 | 0.62769376 | FALSE |
| NOSIP | 1449 | 0.698145 | 0.4850865 | 0.62781336 | FALSE |
| LY6H | 2682 | 0.697199 | 0.4856783 | 0.62841951 | FALSE |
| NES | 12621 | 0.696783 | 0.4859386 | 0.62860391 | FALSE |
| CUTC | 8497 | 0.69628 | 0.4862535 | 0.62893389 | FALSE |
| COG8 | 11957 | 0.69609 | 0.4863725 | 0.629009 | FALSE |
| WDCP | 15606 | 0.695559 | 0.4867051 | 0.6292309 | FALSE |
| POU3F3 | 14273 | 0.695196 | 0.4869325 | 0.62942082 | FALSE |
| FOXS1 | 2892 | 0.694469 | 0.4873881 | 0.62985358 | FALSE |
| CISD1 | 6433 | 0.694202 | 0.4875555 | 0.63001783 | FALSE |
| SETD9 | 2052 | 0.693806 | 0.4878039 | 0.63028664 | FALSE |
| AASDHPPT | 9604 | 0.693301 | 0.4881207 | 0.63053962 | FALSE |
| CABLES1 | 13949 | 0.692985 | 0.488319 | 0.63074365 | FALSE |
| TAGLN | 2590 | 0.692776 | 0.4884501 | 0.63080617 | FALSE |
| GINM1 | 10309 | 0.691634 | 0.4891672 | 0.63152621 | FALSE |
| CEP89 | 15329 | 0.690842 | 0.4896648 | 0.63206427 | FALSE |
| ERLIN2 | 12072 | 0.690445 | 0.4899144 | 0.63230875 | FALSE |
| PAXIP1-AS2 | 7962 | 0.689803 | 0.4903181 | 0.63253432 | FALSE |
| SOX18 | 2914 | 0.689748 | 0.4903527 | 0.63253432 | FALSE |
| ZIC3 | 5610 | 0.688658 | 0.4910385 | 0.63323599 | FALSE |
| FXR2 | 4973 | 0.68849 | 0.4911443 | 0.63329415 | FALSE |
| CEP72 | 7026 | 0.68823 | 0.491308 | 0.63343645 | FALSE |
| IRF7 | 10736 | 0.687419 | 0.4918187 | 0.6338745 | FALSE |
| NAMPT | 2165 | 0.687389 | 0.4918376 | 0.6338745 | FALSE |
| LOC100130264 | 8184 | 0.686978 | 0.4920966 | 0.63411019 | FALSE |
| EXT1 | 12125 | 0.686354 | 0.4924899 | 0.63450591 | FALSE |
| HK1 | 4014 | 0.68627 | 0.4925429 | 0.63452184 | FALSE |
| BUD31 | 1211 | 0.685848 | 0.492809 | 0.63460687 | FALSE |
| C11orf58 | 9887 | 0.685677 | 0.4929168 | 0.63460687 | FALSE |
| TNFAIP8 | 13748 | 0.685627 | 0.4929484 | 0.63460687 | FALSE |
| MTMR9 | 2804 | 0.683519 | 0.4942789 | 0.63581526 | FALSE |
| YWHAE | 4008 | 0.683119 | 0.4945317 | 0.63603565 | FALSE |
| PSMB8 | 7703 | 0.682536 | 0.4949001 | 0.6363505 | FALSE |
| TAS1R1 | 13616 | 0.682474 | 0.4949393 | 0.6363505 | FALSE |
| CLCA4 | 3417 | 0.681984 | 0.4952491 | 0.6365394 | FALSE |
| GRAMD1A | 7984 | 0.68101 | 0.4958652 | 0.63725819 | FALSE |
| FNDC3A | 10899 | 0.680492 | 0.496193 | 0.63754294 | FALSE |
| P3H4 | 2588 | 0.679857 | 0.496595 | 0.6379022 | FALSE |
| DEPDC4 | 10441 | 0.679398 | 0.4968857 | 0.63817075 | FALSE |
| SNAI1 | 12446 | 0.679296 | 0.4969503 | 0.63820131 | FALSE |
| FBXO25 | 14581 | 0.679177 | 0.4970257 | 0.63824569 | FALSE |
| LIN37 | 15 | 0.678728 | 0.4973102 | 0.63850613 | FALSE |
| LGMN | 6929 | 0.678258 | 0.4976081 | 0.63883171 | FALSE |
| GPD1 | 15036 | 0.67728 | 0.4982283 | 0.63936983 | FALSE |
| MTSS1L | 4783 | 0.676457 | 0.4987505 | 0.63988608 | FALSE |
| TBC1D28 | 4073 | 0.675555 | 0.4993232 | 0.64045946 | FALSE |
| INSIG1 | 6684 | 0.674274 | 0.5001371 | 0.64134556 | FALSE |
| NACC1 | 9679 | 0.674034 | 0.5002897 | 0.64148859 | FALSE |
| LIMS1 | 309 | 0.673598 | 0.5005669 | 0.64173879 | FALSE |
| FAM138E | 11231 | 0.672861 | 0.5010357 | 0.64212916 | FALSE |
| ATF7IP | 2470 | 0.672601 | 0.5012012 | 0.64223589 | FALSE |
| CREB3L3 | 9170 | 0.672376 | 0.5013444 | 0.64231362 | FALSE |
| FOXD1 | 6839 | 0.672114 | 0.5015111 | 0.6423698 | FALSE |
| RRP7BP | 12611 | 0.671909 | 0.5016416 | 0.64243169 | FALSE |
| GPR157 | 1779 | 0.67135 | 0.5019976 | 0.64283489 | FALSE |
| FAM110D | 15475 | 0.67125 | 0.5020613 | 0.64286379 | FALSE |
| TRIM45 | 9798 | 0.671032 | 0.5022001 | 0.64293628 | FALSE |
| GTF2H3 | 1221 | 0.670811 | 0.5023409 | 0.64295856 | FALSE |
| AAMP | 10007 | 0.670501 | 0.5025385 | 0.64315873 | FALSE |
| GDF11 | 4197 | 0.670384 | 0.502613 | 0.6431986 | FALSE |
| POFUT2 | 5452 | 0.669485 | 0.5031861 | 0.64372414 | FALSE |
| RWDD3 | 935 | 0.669119 | 0.5034196 | 0.64397006 | FALSE |
| SPRR2C | 3976 | 0.669029 | 0.503477 | 0.64399081 | FALSE |
| LAPTM4A | 1057 | 0.668729 | 0.5036684 | 0.6441302 | FALSE |
| TONSL | 13880 | 0.668032 | 0.5041132 | 0.64459358 | FALSE |
| KRT78 | 15541 | 0.667895 | 0.5042006 | 0.64465268 | FALSE |
| AP2A2 | 12387 | 0.667814 | 0.5042523 | 0.64466607 | FALSE |
| AP4M1 | 15214 | 0.666994 | 0.504776 | 0.64517723 | FALSE |
| ATRIP | 13604 | 0.666811 | 0.5048929 | 0.64522114 | FALSE |
| LRRC75A | 10939 | 0.665277 | 0.5058733 | 0.64631562 | FALSE |
| AGBL3 | 3704 | 0.663639 | 0.5069214 | 0.64723147 | FALSE |
| KRTCAP2 | 8646 | 0.663313 | 0.5071301 | 0.64735488 | FALSE |
| CCDC32 | 9514 | 0.663108 | 0.5072614 | 0.6474012 | FALSE |
| GOLIM4 | 3364 | 0.662933 | 0.5073735 | 0.64749138 | FALSE |
| TMEM74B | 10970 | 0.662616 | 0.5075765 | 0.64764477 | FALSE |
| KRTAP10-11 | 5802 | 0.662148 | 0.5078764 | 0.64781751 | FALSE |
| IL1RAPL2 | 9724 | 0.661881 | 0.5080475 | 0.64798127 | FALSE |
| YWHAEP1 | 5343 | 0.661757 | 0.508127 | 0.64802977 | FALSE |
| MESP2 | 6742 | 0.661092 | 0.5085533 | 0.6484677 | FALSE |
| COA1 | 7527 | 0.660504 | 0.5089305 | 0.64878984 | FALSE |
| C21orf58 | 8631 | 0.660392 | 0.5090023 | 0.6487977 | FALSE |
| MMADHC | 12 | 0.659854 | 0.5093475 | 0.64911439 | FALSE |
| PIGO | 5733 | 0.659783 | 0.5093931 | 0.64911439 | FALSE |
| KCND1 | 9969 | 0.659744 | 0.5094181 | 0.64911439 | FALSE |
| LINC01590 | 10695 | 0.659719 | 0.5094342 | 0.64911439 | FALSE |
| IL15 | 10454 | 0.659268 | 0.5097237 | 0.64937359 | FALSE |
| ZKSCAN2 | 15589 | 0.659208 | 0.5097622 | 0.64937359 | FALSE |
| NOTCH2 | 7178 | 0.65907 | 0.5098508 | 0.64943354 | FALSE |
| COL26A1 | 11213 | 0.658021 | 0.5105246 | 0.65013291 | FALSE |
| HINT3 | 4621 | 0.657392 | 0.5109289 | 0.65054173 | FALSE |
| MMS19 | 14271 | 0.656789 | 0.5113166 | 0.65098236 | FALSE |
| TAF1A | 7345 | 0.656368 | 0.5115874 | 0.65122103 | FALSE |
| RHBDF1 | 14777 | 0.656051 | 0.5117913 | 0.65142758 | FALSE |
| CD3D | 5420 | 0.655964 | 0.5118473 | 0.65144536 | FALSE |
| PACRGL | 8446 | 0.655843 | 0.5119251 | 0.65144536 | FALSE |
| GNA15 | 13685 | 0.655835 | 0.5119303 | 0.65144536 | FALSE |
| NRROS | 2970 | 0.655487 | 0.5121543 | 0.65167731 | FALSE |
| ABHD16A | 676 | 0.654989 | 0.5124748 | 0.65202742 | FALSE |
| KCNQ3 | 744 | 0.65493 | 0.5125128 | 0.65202742 | FALSE |
| HMX2 | 7909 | 0.654836 | 0.5125734 | 0.65205136 | FALSE |
| TBL1X | 7350 | 0.654694 | 0.5126648 | 0.65211462 | FALSE |
| FTSJ1 | 15097 | 0.654475 | 0.5128058 | 0.65224095 | FALSE |
| LOC729930 | 10497 | 0.653602 | 0.5133683 | 0.65279697 | FALSE |
| C14orf144 | 6498 | 0.65332 | 0.51355 | 0.65292186 | FALSE |
| TLR5 | 13043 | 0.652284 | 0.514218 | 0.65361165 | FALSE |
| FZD7 | 11580 | 0.651574 | 0.514676 | 0.65390603 | FALSE |
| CACNA1I | 8407 | 0.651418 | 0.5147767 | 0.65394962 | FALSE |
| HDAC9 | 2474 | 0.649882 | 0.5157684 | 0.65513257 | FALSE |
| KIF3C | 62 | 0.648646 | 0.5165672 | 0.65585116 | FALSE |
| KRTAP2-3 | 539 | 0.648532 | 0.5166409 | 0.65589147 | FALSE |
| ZSCAN30 | 1108 | 0.648426 | 0.5167095 | 0.65592521 | FALSE |
| FLJ42393 | 10476 | 0.647962 | 0.5170095 | 0.65614875 | FALSE |
| PDGFA | 15422 | 0.647959 | 0.5170115 | 0.65614875 | FALSE |
| DACH1 | 1545 | 0.647871 | 0.5170684 | 0.65616772 | FALSE |
| SIRPG | 1251 | 0.64779 | 0.5171208 | 0.65618094 | FALSE |
| MFSD4A | 7287 | 0.647507 | 0.5173039 | 0.65635998 | FALSE |
| EFCAB11 | 6112 | 0.647284 | 0.5174482 | 0.65648978 | FALSE |
| ABHD14B | 2867 | 0.646916 | 0.5176863 | 0.65663206 | FALSE |
| ERBB4 | 11584 | 0.646779 | 0.517775 | 0.65669125 | FALSE |
| PIGM | 1698 | 0.646668 | 0.5178468 | 0.6567291 | FALSE |
| C5orf58 | 7641 | 0.646175 | 0.518166 | 0.6570273 | FALSE |
| TMEM160 | 467 | 0.645925 | 0.5183279 | 0.65712598 | FALSE |
| TRIM11 | 1668 | 0.644867 | 0.5190134 | 0.65788828 | FALSE |
| DCTN6 | 8141 | 0.64442 | 0.5193031 | 0.65820218 | FALSE |
| TCEAL7 | 2963 | 0.644282 | 0.5193926 | 0.65823185 | FALSE |
| CDK17 | 3906 | 0.644254 | 0.5194107 | 0.65823185 | FALSE |
| HNF1A-AS1 | 3918 | 0.643474 | 0.5199166 | 0.65855258 | FALSE |
| MUC1 | 5839 | 0.642478 | 0.5205629 | 0.65931778 | FALSE |
| DMD | 4404 | 0.641938 | 0.5209134 | 0.6596352 | FALSE |
| OR7E14P | 816 | 0.641856 | 0.5209667 | 0.6596352 | FALSE |
| TNKS1BP1 | 3535 | 0.641633 | 0.5211115 | 0.65967966 | FALSE |
| USP31 | 7089 | 0.641583 | 0.521144 | 0.65967966 | FALSE |
| TMEM167A | 13544 | 0.640909 | 0.5215818 | 0.66011383 | FALSE |
| WDR48 | 11557 | 0.64086 | 0.5216136 | 0.66011383 | FALSE |
| UTP20 | 8485 | 0.640438 | 0.5218879 | 0.66037371 | FALSE |
| SARAF | 14002 | 0.640414 | 0.5219035 | 0.66037371 | FALSE |
| RALBP1 | 1484 | 0.640292 | 0.5219828 | 0.66042059 | FALSE |
| OXER1 | 2206 | 0.640199 | 0.5220432 | 0.66042473 | FALSE |
| UNC5D | 2062 | 0.639862 | 0.5222623 | 0.66050699 | FALSE |
| ELP6 | 5088 | 0.639593 | 0.5224372 | 0.6606649 | FALSE |
| UQCRH | 5283 | 0.639186 | 0.5227019 | 0.66090011 | FALSE |
| NPAP1 | 2644 | 0.639124 | 0.5227423 | 0.66090011 | FALSE |
| EAPP | 1444 | 0.638746 | 0.5229882 | 0.66105064 | FALSE |
| YPEL2 | 6677 | 0.638076 | 0.5234242 | 0.66154497 | FALSE |
| AMFR | 5019 | 0.637958 | 0.523501 | 0.66154497 | FALSE |
| MED27 | 3757 | 0.637802 | 0.5236026 | 0.66161325 | FALSE |
| ANKRD13B | 1307 | 0.637536 | 0.5237758 | 0.66175967 | FALSE |
| CD37 | 7008 | 0.636968 | 0.5241457 | 0.66213895 | FALSE |
| LINC02014 | 9436 | 0.636551 | 0.5244174 | 0.66227054 | FALSE |
| GPHA2 | 4548 | 0.636548 | 0.5244193 | 0.66227054 | FALSE |
| YES1 | 69 | 0.636405 | 0.5245125 | 0.66231412 | FALSE |
| CTXN3 | 9426 | 0.635824 | 0.5248911 | 0.66265228 | FALSE |
| FOXA2 | 10124 | 0.635534 | 0.5250802 | 0.66283743 | FALSE |
| LINC00294 | 14270 | 0.635446 | 0.5251376 | 0.66285634 | FALSE |
| HOOK1 | 9922 | 0.635158 | 0.5253254 | 0.66295089 | FALSE |
| HADHA | 2589 | 0.635126 | 0.5253462 | 0.66295089 | FALSE |
| SNX22 | 13272 | 0.635071 | 0.5253821 | 0.66295089 | FALSE |
| IL4R | 12698 | 0.63469 | 0.5256306 | 0.66315741 | FALSE |
| AGBL2 | 10278 | 0.634334 | 0.5258629 | 0.66338444 | FALSE |
| CEBPD | 6431 | 0.634263 | 0.5259092 | 0.66338444 | FALSE |
| RDH14 | 4060 | 0.634119 | 0.5260032 | 0.66338444 | FALSE |
| MMP19 | 12509 | 0.634089 | 0.5260227 | 0.66338444 | FALSE |
| PLCXD2 | 2677 | 0.63378 | 0.5262244 | 0.66343208 | FALSE |
| SNX6 | 12730 | 0.633203 | 0.5266011 | 0.66379252 | FALSE |
| TRAPPC1 | 4113 | 0.633074 | 0.5266853 | 0.66384517 | FALSE |
| C9orf106 | 1044 | 0.632858 | 0.5268264 | 0.66396943 | FALSE |
| OSTC | 4711 | 0.632408 | 0.5271203 | 0.6641503 | FALSE |
| F13A1 | 11142 | 0.632193 | 0.5272608 | 0.6641503 | FALSE |
| GPR12 | 9226 | 0.632155 | 0.5272856 | 0.6641503 | FALSE |
| ALDH16A1 | 13338 | 0.632141 | 0.5272947 | 0.6641503 | FALSE |
| ATP8A1 | 11881 | 0.632088 | 0.5273294 | 0.6641503 | FALSE |
| GRB7 | 2211 | 0.632064 | 0.5273451 | 0.6641503 | FALSE |
| ALKBH3 | 12988 | 0.630259 | 0.5285251 | 0.6652523 | FALSE |
| CAPS | 9940 | 0.629975 | 0.5287109 | 0.66532546 | FALSE |
| ZNF469 | 13930 | 0.629903 | 0.5287581 | 0.66533118 | FALSE |
| ARFIP1 | 719 | 0.629546 | 0.5289917 | 0.66557156 | FALSE |
| GNG5 | 1175 | 0.629204 | 0.5292155 | 0.66574604 | FALSE |
| GLMP | 8693 | 0.628808 | 0.5294748 | 0.66591142 | FALSE |
| AOC2 | 4814 | 0.628719 | 0.529533 | 0.66593114 | FALSE |
| RAD23A | 637 | 0.628621 | 0.5295972 | 0.66594905 | FALSE |
| EXOSC5 | 7145 | 0.628391 | 0.5297478 | 0.66594905 | FALSE |
| TOR2A | 9211 | 0.628391 | 0.5297478 | 0.66594905 | FALSE |
| LOC400684 | 14400 | 0.627719 | 0.530188 | 0.66637961 | FALSE |
| FLNA | 13371 | 0.62648 | 0.5310002 | 0.66714616 | FALSE |
| TSR2 | 12843 | 0.626463 | 0.5310113 | 0.66714616 | FALSE |
| WFDC12 | 10406 | 0.626297 | 0.5311202 | 0.6672293 | FALSE |
| MRPL21 | 4219 | 0.62607 | 0.531269 | 0.66736269 | FALSE |
| VPS72 | 10775 | 0.625852 | 0.531412 | 0.66748868 | FALSE |
| POMGNT1 | 1809 | 0.625408 | 0.5317033 | 0.66772081 | FALSE |
| MRGPRX4 | 3721 | 0.625284 | 0.5317847 | 0.66774216 | FALSE |
| HAPLN1 | 2074 | 0.624939 | 0.5320111 | 0.66797281 | FALSE |
| OR10H2 | 12579 | 0.624823 | 0.5320872 | 0.66801475 | FALSE |
| ATP13A2 | 9218 | 0.62437 | 0.5323846 | 0.66817348 | FALSE |
| LCE3E | 158 | 0.624112 | 0.532554 | 0.66829692 | FALSE |
| CDK2AP2 | 5856 | 0.62409 | 0.5325685 | 0.66829692 | FALSE |
| LYZL1 | 5389 | 0.623887 | 0.5327018 | 0.66841056 | FALSE |
| IFI44 | 5195 | 0.623779 | 0.5327727 | 0.66844592 | FALSE |
| ERAL1 | 2377 | 0.623399 | 0.5330224 | 0.66868557 | FALSE |
| CD226 | 1424 | 0.623196 | 0.5331557 | 0.66876543 | FALSE |
| CDH16 | 10277 | 0.622795 | 0.5334192 | 0.66893156 | FALSE |
| IL18BP | 297 | 0.622779 | 0.5334298 | 0.66893156 | FALSE |
| CKB | 3039 | 0.622666 | 0.533504 | 0.66893395 | FALSE |
| ELOF1 | 12940 | 0.622433 | 0.5336572 | 0.66907232 | FALSE |
| GRM2 | 13212 | 0.622328 | 0.5337262 | 0.66908443 | FALSE |
| LINC01184 | 13097 | 0.621602 | 0.5342036 | 0.66954261 | FALSE |
| ARID5A | 2100 | 0.621319 | 0.5343898 | 0.66961147 | FALSE |
| GPC2 | 932 | 0.621258 | 0.5344299 | 0.66961147 | FALSE |
| CEP70 | 2378 | 0.621258 | 0.5344299 | 0.66961147 | FALSE |
| 5-Mar | 8743 | 0.620829 | 0.5347121 | 0.66985775 | FALSE |
| RPS6KA6 | 754 | 0.620259 | 0.5350873 | 0.670274 | FALSE |
| IAPP | 13833 | 0.619919 | 0.5353111 | 0.67044776 | FALSE |
| TUB | 6406 | 0.619918 | 0.5353118 | 0.67044776 | FALSE |
| RPL22L1 | 10550 | 0.619456 | 0.535616 | 0.67077505 | FALSE |
| VWA1 | 1553 | 0.618973 | 0.5359341 | 0.67101221 | FALSE |
| SHOC2 | 3663 | 0.618159 | 0.5364705 | 0.67163 | FALSE |
| PRKAG2-AS1 | 8337 | 0.617505 | 0.5369017 | 0.67211595 | FALSE |
| ARHGAP23 | 4661 | 0.617192 | 0.5371081 | 0.67232051 | FALSE |
| KCNQ1DN | 10589 | 0.61596 | 0.5379209 | 0.67301463 | FALSE |
| PKNOX1 | 8723 | 0.615378 | 0.5383051 | 0.67338752 | FALSE |
| CHAC2 | 9267 | 0.615176 | 0.5384385 | 0.67350048 | FALSE |
| COLCA2 | 14760 | 0.614459 | 0.5389121 | 0.6740389 | FALSE |
| SAMD10 | 10729 | 0.614233 | 0.5390614 | 0.67411778 | FALSE |
| CALHM1 | 6913 | 0.614067 | 0.5391711 | 0.67420102 | FALSE |
| TFIP11 | 14491 | 0.612682 | 0.5400866 | 0.67529187 | FALSE |
| BAG2 | 12107 | 0.612065 | 0.5404948 | 0.67569408 | FALSE |
| ENO4 | 7647 | 0.611477 | 0.5408838 | 0.67607237 | FALSE |
| TTF1 | 1324 | 0.610899 | 0.5412664 | 0.67641897 | FALSE |
| FAM69B | 12075 | 0.609286 | 0.5423349 | 0.67756518 | FALSE |
| MGAT2 | 15014 | 0.608832 | 0.5426358 | 0.67782864 | FALSE |
| ERG28 | 1311 | 0.607376 | 0.5436014 | 0.67889364 | FALSE |
| EID1 | 4730 | 0.607351 | 0.543618 | 0.67889364 | FALSE |
| SPATA32 | 6452 | 0.60696 | 0.5438775 | 0.67916258 | FALSE |
| SGK1 | 11430 | 0.606128 | 0.5444298 | 0.67960156 | FALSE |
| MPST | 11081 | 0.605548 | 0.5448149 | 0.67990292 | FALSE |
| HIST1H4L | 1866 | 0.605153 | 0.5450773 | 0.68011765 | FALSE |
| TMBIM4 | 2533 | 0.604878 | 0.5452601 | 0.68023706 | FALSE |
| TRMT1 | 3028 | 0.603677 | 0.5460584 | 0.68106998 | FALSE |
| PPP1R11 | 13944 | 0.603497 | 0.5461781 | 0.68116493 | FALSE |
| ZNF747 | 14195 | 0.603287 | 0.5463178 | 0.68128477 | FALSE |
| C6orf62 | 9297 | 0.602551 | 0.5468074 | 0.68167787 | FALSE |
| NAE1 | 5851 | 0.602389 | 0.5469152 | 0.68170354 | FALSE |
| SLCO2B1 | 11754 | 0.60114 | 0.5477467 | 0.68252231 | FALSE |
| SPRYD7 | 6704 | 0.600836 | 0.5479492 | 0.68252909 | FALSE |
| RBM34 | 7597 | 0.600721 | 0.5480258 | 0.68252909 | FALSE |
| CCT6A | 10187 | 0.600673 | 0.5480578 | 0.68252909 | FALSE |
| HCP5 | 3567 | 0.600486 | 0.5481824 | 0.68256261 | FALSE |
| NOC4L | 5166 | 0.600436 | 0.5482157 | 0.68256261 | FALSE |
| CMTM6 | 5121 | 0.600207 | 0.5483683 | 0.68269821 | FALSE |
| ZNF581 | 9399 | 0.599855 | 0.5486029 | 0.68293588 | FALSE |
| PHB2 | 5806 | 0.598954 | 0.5492036 | 0.68352866 | FALSE |
| ZNF28 | 774 | 0.59816 | 0.5497332 | 0.68390727 | FALSE |
| SPTBN1 | 11090 | 0.597786 | 0.5499827 | 0.68413044 | FALSE |
| MUC5AC | 4215 | 0.59776 | 0.5500001 | 0.68413044 | FALSE |
| EGR4 | 14860 | 0.59681 | 0.5506342 | 0.68455458 | FALSE |
| FLJ30901 | 8486 | 0.59679 | 0.5506476 | 0.68455458 | FALSE |
| SSTR3 | 12573 | 0.595268 | 0.5516643 | 0.6856005 | FALSE |
| ZNF580 | 101 | 0.593982 | 0.5525242 | 0.68650533 | FALSE |
| TMEM161A | 9699 | 0.592992 | 0.5531865 | 0.68700651 | FALSE |
| HIC2 | 7117 | 0.591812 | 0.5539765 | 0.68781784 | FALSE |
| KCNIP1 | 1708 | 0.591109 | 0.5544474 | 0.68818389 | FALSE |
| CD86 | 12450 | 0.589442 | 0.5555648 | 0.68935191 | FALSE |
| MCRIP2 | 10019 | 0.589288 | 0.5556681 | 0.68938478 | FALSE |
| LOC148413 | 10710 | 0.589271 | 0.5556795 | 0.68938478 | FALSE |
| RAB7A | 9373 | 0.588586 | 0.556139 | 0.68971949 | FALSE |
| TULP3 | 11846 | 0.588054 | 0.556496 | 0.69001449 | FALSE |
| C3orf67 | 5812 | 0.587796 | 0.5566692 | 0.69011975 | FALSE |
| LOC91548 | 8060 | 0.587476 | 0.5568841 | 0.69033134 | FALSE |
| MBD4 | 13730 | 0.587161 | 0.5570956 | 0.69047195 | FALSE |
| ARL1 | 12307 | 0.587044 | 0.5571742 | 0.69047195 | FALSE |
| GIPR | 5769 | 0.586664 | 0.5574294 | 0.69056927 | FALSE |
| GATD1 | 13713 | 0.5857 | 0.5580771 | 0.6912074 | FALSE |
| TMEM8A | 6819 | 0.585601 | 0.5581437 | 0.69123505 | FALSE |
| SELENOT | 14596 | 0.584766 | 0.5587051 | 0.69170358 | FALSE |
| EPHA1 | 4262 | 0.584736 | 0.5587252 | 0.69170358 | FALSE |
| EBI3 | 3998 | 0.584377 | 0.5589667 | 0.69170358 | FALSE |
| TMEM11 | 6695 | 0.584298 | 0.5590198 | 0.69170358 | FALSE |
| PSMC2 | 6020 | 0.584256 | 0.5590481 | 0.69170358 | FALSE |
| GUCA2A | 9636 | 0.584183 | 0.5590972 | 0.69170358 | FALSE |
| PRDX3 | 12577 | 0.583668 | 0.5594437 | 0.69202274 | FALSE |
| BCKDHA | 2951 | 0.58273 | 0.5600751 | 0.69269412 | FALSE |
| KMT5C | 11554 | 0.582175 | 0.5604488 | 0.69285995 | FALSE |
| CSNK2A1 | 9393 | 0.582157 | 0.5604609 | 0.69285995 | FALSE |
| NPHP1 | 4343 | 0.582136 | 0.5604751 | 0.69285995 | FALSE |
| OPA3 | 15383 | 0.580789 | 0.5613827 | 0.69388339 | FALSE |
| TRAPPC2 | 6028 | 0.58067 | 0.5614629 | 0.69388339 | FALSE |
| C8orf76 | 5595 | 0.580582 | 0.5615222 | 0.69388339 | FALSE |
| LCE1F | 13561 | 0.580212 | 0.5617717 | 0.69402374 | FALSE |
| TRIM49B | 12116 | 0.58014 | 0.5618202 | 0.69402887 | FALSE |
| ZIK1 | 8053 | 0.57932 | 0.5623733 | 0.69449257 | FALSE |
| ADNP-AS1 | 3045 | 0.57914 | 0.5624947 | 0.6945328 | FALSE |
| SQSTM1 | 10017 | 0.578696 | 0.5627943 | 0.69474895 | FALSE |
| PANX1 | 4149 | 0.578519 | 0.5629138 | 0.69483072 | FALSE |
| KLHDC10 | 6887 | 0.57795 | 0.5632979 | 0.69506608 | FALSE |
| LOC391322 | 5732 | 0.57794 | 0.5633046 | 0.69506608 | FALSE |
| CDC6 | 967 | 0.577892 | 0.563337 | 0.69506608 | FALSE |
| GSTM2P1 | 13883 | 0.577803 | 0.5633971 | 0.69506608 | FALSE |
| ASIC4 | 12536 | 0.577638 | 0.5635085 | 0.69506608 | FALSE |
| CAPN10 | 5364 | 0.577578 | 0.5635491 | 0.69506608 | FALSE |
| SPATA5L1 | 1795 | 0.577344 | 0.5637071 | 0.69517629 | FALSE |
| AREL1 | 9036 | 0.576324 | 0.5643962 | 0.69589131 | FALSE |
| PLA2G2D | 3273 | 0.576178 | 0.5644949 | 0.69595807 | FALSE |
| TRPV6 | 9437 | 0.575277 | 0.565104 | 0.69659915 | FALSE |
| B4GALNT4 | 7498 | 0.575051 | 0.5652568 | 0.69673261 | FALSE |
| LINC01506 | 8800 | 0.574826 | 0.565409 | 0.6968103 | FALSE |
| TMEM230 | 4135 | 0.574203 | 0.5658304 | 0.69706662 | FALSE |
| SLC12A4 | 7065 | 0.573846 | 0.566072 | 0.69719607 | FALSE |
| TOMM20L | 5461 | 0.573836 | 0.5660788 | 0.69719607 | FALSE |
| ERLIN1 | 6626 | 0.57366 | 0.5661979 | 0.69728784 | FALSE |
| OR2H1 | 5882 | 0.573558 | 0.5662669 | 0.69731793 | FALSE |
| SPN | 3318 | 0.573459 | 0.566334 | 0.69734552 | FALSE |
| BCL2L1 | 4819 | 0.573363 | 0.5663989 | 0.69737061 | FALSE |
| CSAG3 | 3452 | 0.573292 | 0.566447 | 0.69737486 | FALSE |
| ECT2 | 6353 | 0.571684 | 0.5675361 | 0.69855062 | FALSE |
| SPCS1 | 885 | 0.571124 | 0.5679156 | 0.69896272 | FALSE |
| FGD6 | 2322 | 0.570837 | 0.5681101 | 0.69914712 | FALSE |
| MED29 | 6538 | 0.570493 | 0.5683434 | 0.69932404 | FALSE |
| STK16 | 13856 | 0.570352 | 0.568439 | 0.69938663 | FALSE |
| C15orf62 | 24 | 0.570247 | 0.5685102 | 0.69941919 | FALSE |
| ADAMTS10 | 13735 | 0.570108 | 0.5686044 | 0.69945094 | FALSE |
| GABBR1 | 4539 | 0.569052 | 0.5693209 | 0.69997585 | FALSE |
| PSD2 | 7256 | 0.568563 | 0.5696527 | 0.70032882 | FALSE |
| NTN1 | 10797 | 0.568023 | 0.5700194 | 0.70066933 | FALSE |
| LOC100505915 | 11936 | 0.567496 | 0.5703772 | 0.70094391 | FALSE |
| VDAC1 | 225 | 0.567282 | 0.5705226 | 0.70106743 | FALSE |
| LGALS14 | 10697 | 0.566296 | 0.5711926 | 0.70162491 | FALSE |
| VLDLR-AS1 | 6331 | 0.566208 | 0.5712524 | 0.70162491 | FALSE |
| TOP1MT | 10428 | 0.566185 | 0.571268 | 0.70162491 | FALSE |
| C11orf96 | 14958 | 0.566086 | 0.5713353 | 0.70162491 | FALSE |
| SNHG18 | 8820 | 0.563851 | 0.5728555 | 0.70317125 | FALSE |
| KAT5 | 11106 | 0.563621 | 0.5730121 | 0.70327796 | FALSE |
| MIR7515HG | 1384 | 0.562796 | 0.5735738 | 0.70371803 | FALSE |
| TIGD2 | 13711 | 0.562022 | 0.574101 | 0.70413631 | FALSE |
| WNT5A | 13972 | 0.561693 | 0.5743252 | 0.70428979 | FALSE |
| POLD2 | 12744 | 0.56164 | 0.5743613 | 0.70428979 | FALSE |
| GYG2P1 | 659 | 0.561312 | 0.5745849 | 0.70445339 | FALSE |
| PLN | 15454 | 0.560375 | 0.5752237 | 0.70501543 | FALSE |
| OTUD5 | 2485 | 0.560226 | 0.5753253 | 0.70502942 | FALSE |
| KRT15 | 6059 | 0.559478 | 0.5758355 | 0.705142 | FALSE |
| CSRNP1 | 1228 | 0.559394 | 0.5758929 | 0.705142 | FALSE |
| TMEM254 | 925 | 0.559152 | 0.576058 | 0.70526388 | FALSE |
| ADAT3 | 7022 | 0.558366 | 0.5765945 | 0.7056444 | FALSE |
| TRAM2 | 2825 | 0.558108 | 0.5767706 | 0.70580473 | FALSE |
| GNPDA1 | 7635 | 0.557776 | 0.5769974 | 0.70597999 | FALSE |
| CNOT3 | 6081 | 0.557358 | 0.5772829 | 0.70615516 | FALSE |
| TMEM175 | 2029 | 0.556853 | 0.5776279 | 0.70652191 | FALSE |
| YIPF5 | 9089 | 0.556502 | 0.5778677 | 0.70675999 | FALSE |
| GNRH2 | 13743 | 0.556354 | 0.5779689 | 0.7068284 | FALSE |
| TMEM94 | 5380 | 0.556141 | 0.5781145 | 0.70692762 | FALSE |
| ZNF10 | 13852 | 0.556103 | 0.5781404 | 0.70692762 | FALSE |
| ACSS1 | 340 | 0.555782 | 0.5783599 | 0.70707267 | FALSE |
| GTF3C1 | 7911 | 0.555731 | 0.5783948 | 0.70707267 | FALSE |
| HRASLS5 | 3433 | 0.554878 | 0.5789781 | 0.70767512 | FALSE |
| TCF20 | 2958 | 0.554557 | 0.5791977 | 0.70784037 | FALSE |
| ADCK5 | 12896 | 0.55444 | 0.5792778 | 0.70787534 | FALSE |
| LILRA1 | 682 | 0.554097 | 0.5795125 | 0.7081068 | FALSE |
| DGKA | 331 | 0.552573 | 0.5805558 | 0.70921539 | FALSE |
| MRPL32 | 6369 | 0.552021 | 0.580934 | 0.70962187 | FALSE |
| AQP2 | 1666 | 0.551542 | 0.5812622 | 0.7099068 | FALSE |
| ETV1 | 2886 | 0.551354 | 0.581391 | 0.7099068 | FALSE |
| PWWP2B | 1863 | 0.551019 | 0.5816207 | 0.71001684 | FALSE |
| CPNE1 | 1127 | 0.550124 | 0.5822343 | 0.710655 | FALSE |
| SNF8 | 13188 | 0.54994 | 0.5823605 | 0.71075354 | FALSE |
| PTPN23 | 10136 | 0.549794 | 0.5824607 | 0.71081264 | FALSE |
| FAM24B | 14650 | 0.549698 | 0.5825265 | 0.71081264 | FALSE |
| C1orf198 | 14653 | 0.54956 | 0.5826212 | 0.71081264 | FALSE |
| ZSCAN1 | 2731 | 0.549538 | 0.5826363 | 0.71081264 | FALSE |
| RAB39B | 3582 | 0.548438 | 0.5833912 | 0.71162254 | FALSE |
| SMCR5 | 9644 | 0.548362 | 0.5834434 | 0.71163066 | FALSE |
| KIAA1671 | 14886 | 0.54765 | 0.5839323 | 0.71206029 | FALSE |
| ZAN | 4141 | 0.546688 | 0.5845931 | 0.71264183 | FALSE |
| BTG2 | 2469 | 0.546624 | 0.5846371 | 0.71264183 | FALSE |
| LOC105373876 | 12060 | 0.546034 | 0.5850426 | 0.7130249 | FALSE |
| TAS2R39 | 10096 | 0.545878 | 0.5851498 | 0.7131 | FALSE |
| RETSAT | 6030 | 0.545698 | 0.5852735 | 0.71310666 | FALSE |
| IGF2BP2 | 3741 | 0.545671 | 0.5852921 | 0.71310666 | FALSE |
| DDX50 | 14816 | 0.545412 | 0.5854702 | 0.71326804 | FALSE |
| H3F3C | 10102 | 0.545069 | 0.5857061 | 0.71338862 | FALSE |
| HGD | 14129 | 0.544877 | 0.5858381 | 0.71343829 | FALSE |
| ELP5 | 10213 | 0.543904 | 0.5865075 | 0.71397542 | FALSE |
| VDAC2 | 11707 | 0.542649 | 0.5873715 | 0.71470631 | FALSE |
| TRIB2 | 5266 | 0.542571 | 0.5874252 | 0.71470631 | FALSE |
| SLC39A11 | 4640 | 0.542228 | 0.5876614 | 0.71493474 | FALSE |
| ACBD7 | 7230 | 0.54136 | 0.5882595 | 0.71543958 | FALSE |
| IGSF11 | 2160 | 0.541006 | 0.5885034 | 0.71559192 | FALSE |
| SPI1 | 8340 | 0.540979 | 0.5885221 | 0.71559192 | FALSE |
| C8orf44 | 1502 | 0.540903 | 0.5885744 | 0.71559996 | FALSE |
| SOD3 | 1439 | 0.540523 | 0.5888364 | 0.71573855 | FALSE |
| ASGR2 | 5516 | 0.539624 | 0.5894564 | 0.71628226 | FALSE |
| OR13H1 | 2997 | 0.539501 | 0.5895412 | 0.71632933 | FALSE |
| FAM210A | 3360 | 0.539033 | 0.5898641 | 0.7166106 | FALSE |
| SPEM1 | 6179 | 0.538332 | 0.5903479 | 0.71708688 | FALSE |
| SAMD5 | 8331 | 0.536587 | 0.5915529 | 0.71816004 | FALSE |
| ENOSF1 | 5598 | 0.536159 | 0.5918487 | 0.71835172 | FALSE |
| PPCDC | 2221 | 0.535184 | 0.5925226 | 0.71889906 | FALSE |
| NMB | 5581 | 0.535174 | 0.5925295 | 0.71889906 | FALSE |
| GAB4 | 14485 | 0.534763 | 0.5928138 | 0.71907646 | FALSE |
| C19orf24 | 14725 | 0.532758 | 0.5942011 | 0.72064748 | FALSE |
| ARMC6 | 15500 | 0.532433 | 0.5944261 | 0.72080855 | FALSE |
| EPOP | 11819 | 0.531894 | 0.5947994 | 0.72116611 | FALSE |
| CSPG4P1Y | 13721 | 0.531874 | 0.5948133 | 0.72116611 | FALSE |
| KRTAP19-5 | 2927 | 0.531253 | 0.5952435 | 0.72158081 | FALSE |
| CCDC166 | 9302 | 0.531247 | 0.5952476 | 0.72158081 | FALSE |
| ANP32A-IT1 | 7997 | 0.531115 | 0.5953391 | 0.72163573 | FALSE |
| TBATA | 10494 | 0.530556 | 0.5957265 | 0.72197434 | FALSE |
| PLG | 9832 | 0.530512 | 0.595757 | 0.72197434 | FALSE |
| DSN1 | 3334 | 0.529922 | 0.596166 | 0.72221866 | FALSE |
| LINC02076 | 13322 | 0.529892 | 0.5961868 | 0.72221866 | FALSE |
| SYDE1 | 11873 | 0.529631 | 0.5963678 | 0.72227588 | FALSE |
| ASNA1 | 14554 | 0.52962 | 0.5963754 | 0.72227588 | FALSE |
| DLG4 | 13472 | 0.528984 | 0.5968165 | 0.72264721 | FALSE |
| KRT19 | 360 | 0.528978 | 0.5968207 | 0.72264721 | FALSE |
| HIF1AN | 12873 | 0.528805 | 0.5969407 | 0.72273656 | FALSE |
| MAP4K3 | 1406 | 0.527942 | 0.5975396 | 0.72329357 | FALSE |
| AP2A1 | 4956 | 0.527239 | 0.5980276 | 0.72359354 | FALSE |
| NSMF | 13584 | 0.526202 | 0.5987479 | 0.72419539 | FALSE |
| PTPRU | 6672 | 0.525996 | 0.598891 | 0.72426987 | FALSE |
| SLITRK4 | 11812 | 0.52598 | 0.5989021 | 0.72426987 | FALSE |
| DBIL5P | 7685 | 0.525595 | 0.5991696 | 0.72453735 | FALSE |
| LMO4 | 198 | 0.524735 | 0.5997674 | 0.72514804 | FALSE |
| BDNF-AS | 10251 | 0.524372 | 0.6000198 | 0.72534101 | FALSE |
| MPLKIP | 11956 | 0.5225 | 0.6013222 | 0.72635378 | FALSE |
| CCDC22 | 6827 | 0.522322 | 0.6014462 | 0.72644733 | FALSE |
| SASH1 | 2283 | 0.521825 | 0.6017922 | 0.72678512 | FALSE |
| SEC14L4 | 1097 | 0.521727 | 0.6018604 | 0.72678512 | FALSE |
| KDM6A | 1758 | 0.521646 | 0.6019168 | 0.72679121 | FALSE |
| GDF15 | 5987 | 0.52142 | 0.6020742 | 0.72692511 | FALSE |
| ZBTB22 | 13630 | 0.521129 | 0.6022769 | 0.72704447 | FALSE |
| AOC3 | 12369 | 0.521073 | 0.6023159 | 0.72704447 | FALSE |
| ZFP62 | 2911 | 0.518855 | 0.6038619 | 0.72863326 | FALSE |
| CDK5R1 | 4878 | 0.518533 | 0.6040864 | 0.72883213 | FALSE |
| SHANK2-AS3 | 1610 | 0.518198 | 0.6043201 | 0.72901741 | FALSE |
| ADAMTSL5 | 11703 | 0.517889 | 0.6045357 | 0.72910869 | FALSE |
| IFNAR1 | 11368 | 0.517603 | 0.6047353 | 0.72923616 | FALSE |
| CD109 | 12931 | 0.517601 | 0.6047367 | 0.72923616 | FALSE |
| AGRN | 14628 | 0.517537 | 0.6047813 | 0.72923616 | FALSE |
| SGF29 | 2252 | 0.517411 | 0.6048693 | 0.72927514 | FALSE |
| USP27X | 1763 | 0.517357 | 0.604907 | 0.72927514 | FALSE |
| UBAP2 | 15301 | 0.516797 | 0.6052979 | 0.72946513 | FALSE |
| DHRS13 | 10203 | 0.516587 | 0.6054445 | 0.72958558 | FALSE |
| LOC149373 | 7619 | 0.516506 | 0.605501 | 0.7295975 | FALSE |
| RAB40B | 2955 | 0.516431 | 0.6055534 | 0.72960436 | FALSE |
| QPRT | 10907 | 0.516296 | 0.6056477 | 0.72960548 | FALSE |
| TMEM64 | 12563 | 0.516114 | 0.6057748 | 0.72970237 | FALSE |
| ALKBH6 | 6159 | 0.514724 | 0.6067459 | 0.73070323 | FALSE |
| COPS7A | 14289 | 0.51437 | 0.6069933 | 0.7308886 | FALSE |
| ZC3H3 | 174 | 0.51364 | 0.6075037 | 0.73139049 | FALSE |
| PDXDC1 | 9024 | 0.513445 | 0.6076401 | 0.73149833 | FALSE |
| CHRAC1 | 8599 | 0.51271 | 0.6081542 | 0.73192211 | FALSE |
| HTRA3 | 13539 | 0.512455 | 0.6083326 | 0.7320056 | FALSE |
| OR4D6 | 5959 | 0.512441 | 0.6083424 | 0.7320056 | FALSE |
| CDAN1 | 8978 | 0.51202 | 0.608637 | 0.73217271 | FALSE |
| NOL9 | 7745 | 0.511902 | 0.6087196 | 0.73217271 | FALSE |
| PSMD13 | 940 | 0.511841 | 0.6087623 | 0.73217271 | FALSE |
| LOC643201 | 8708 | 0.511382 | 0.6090836 | 0.73249029 | FALSE |
| RBM10 | 2702 | 0.511263 | 0.6091669 | 0.73249029 | FALSE |
| AATF | 7326 | 0.510505 | 0.6096977 | 0.73295942 | FALSE |
| NKX2-5 | 6455 | 0.510233 | 0.6098882 | 0.73313209 | FALSE |
| RTCA | 3954 | 0.510057 | 0.6100115 | 0.73322392 | FALSE |
| BCORP1 | 4155 | 0.509842 | 0.6101622 | 0.73331663 | FALSE |
| GRHPR | 10829 | 0.509813 | 0.6101825 | 0.73331663 | FALSE |
| EXOC7 | 7083 | 0.509597 | 0.6103338 | 0.73344213 | FALSE |
| PRB1 | 3054 | 0.509523 | 0.6103857 | 0.73344807 | FALSE |
| U2AF2 | 7268 | 0.508684 | 0.6109737 | 0.73409826 | FALSE |
| CTAGE1 | 1213 | 0.508491 | 0.611109 | 0.73420441 | FALSE |
| KBTBD6 | 11588 | 0.50821 | 0.6113061 | 0.73426596 | FALSE |
| BZW2 | 10925 | 0.508202 | 0.6113117 | 0.73426596 | FALSE |
| COQ4 | 9396 | 0.50815 | 0.6113482 | 0.73426596 | FALSE |
| FAM173B | 2516 | 0.507388 | 0.6118826 | 0.73462566 | FALSE |
| SOX8 | 7921 | 0.506717 | 0.6123534 | 0.73507799 | FALSE |
| FLJ37453 | 2125 | 0.506633 | 0.6124123 | 0.73509231 | FALSE |
| PRLH | 7821 | 0.506094 | 0.6127907 | 0.73540903 | FALSE |
| AHDC1 | 316 | 0.506056 | 0.6128173 | 0.73540903 | FALSE |
| ART5 | 4319 | 0.505714 | 0.6130574 | 0.73543006 | FALSE |
| FBXL7 | 11716 | 0.505694 | 0.6130715 | 0.73543006 | FALSE |
| ACAD8 | 6006 | 0.505629 | 0.6131171 | 0.73543006 | FALSE |
| ZNF735 | 11013 | 0.504459 | 0.6139389 | 0.73635924 | FALSE |
| FAM200A | 3369 | 0.504347 | 0.6140176 | 0.73639283 | FALSE |
| ARSA | 4477 | 0.503764 | 0.6144272 | 0.73671889 | FALSE |
| DGCR5 | 14887 | 0.503674 | 0.6144905 | 0.73673822 | FALSE |
| MARS | 1859 | 0.503087 | 0.6149031 | 0.73711987 | FALSE |
| SLC1A7 | 2525 | 0.502979 | 0.614979 | 0.73715437 | FALSE |
| TAPT1 | 11472 | 0.502766 | 0.6151288 | 0.73727477 | FALSE |
| CBX4 | 12677 | 0.502528 | 0.6152962 | 0.73728967 | FALSE |
| MSI2 | 9720 | 0.502097 | 0.6155993 | 0.7374455 | FALSE |
| ADAMTS7 | 12824 | 0.501403 | 0.6160875 | 0.73791728 | FALSE |
| PHF1 | 4871 | 0.501102 | 0.6162993 | 0.73811442 | FALSE |
| EFHC1 | 15583 | 0.500955 | 0.6164028 | 0.73818177 | FALSE |
| HIPK1-AS1 | 14164 | 0.500083 | 0.6170166 | 0.73874711 | FALSE |
| C1orf43 | 13968 | 0.499989 | 0.6170828 | 0.73876978 | FALSE |
| CGB1 | 11887 | 0.499587 | 0.6173659 | 0.73899551 | FALSE |
| PFN2 | 230 | 0.499333 | 0.6175448 | 0.73903989 | FALSE |
| ARF1 | 12757 | 0.498901 | 0.6178491 | 0.73923431 | FALSE |
| CSAD | 9448 | 0.498368 | 0.6182247 | 0.73956527 | FALSE |
| FAM9B | 12635 | 0.498067 | 0.6184368 | 0.73969066 | FALSE |
| PYCR2 | 14734 | 0.498024 | 0.6184671 | 0.73969066 | FALSE |
| BMP10 | 15253 | 0.497917 | 0.6185425 | 0.73972427 | FALSE |
| LRRC29 | 3637 | 0.497729 | 0.6186751 | 0.73982615 | FALSE |
| TNFSF9 | 15161 | 0.497557 | 0.6187963 | 0.73991455 | FALSE |
| STK4 | 9342 | 0.497181 | 0.6190614 | 0.74017493 | FALSE |
| PITPNA | 10148 | 0.496733 | 0.6193773 | 0.74049603 | FALSE |
| CNR2 | 12077 | 0.496655 | 0.6194324 | 0.74050517 | FALSE |
| SDS | 8609 | 0.49572 | 0.620092 | 0.74102879 | FALSE |
| XPNPEP3 | 14071 | 0.495631 | 0.6201548 | 0.74102879 | FALSE |
| RHNO1 | 14031 | 0.495497 | 0.6202493 | 0.74103342 | FALSE |
| PPP2R1B | 4396 | 0.495464 | 0.6202726 | 0.74103342 | FALSE |
| RAMP2 | 8813 | 0.495424 | 0.6203009 | 0.74103342 | FALSE |
| SCO2 | 756 | 0.495095 | 0.6205331 | 0.74113376 | FALSE |
| MCM3 | 5786 | 0.495074 | 0.6205479 | 0.74113376 | FALSE |
| MOAP1 | 6941 | 0.494997 | 0.6206022 | 0.74113376 | FALSE |
| SNHG17 | 10656 | 0.494902 | 0.6206693 | 0.74113376 | FALSE |
| ITPKA | 13436 | 0.494692 | 0.6208175 | 0.74117428 | FALSE |
| WFDC10B | 9023 | 0.494458 | 0.6209828 | 0.74117428 | FALSE |
| SPRED3 | 521 | 0.494451 | 0.6209877 | 0.74117428 | FALSE |
| EPS8L2 | 6448 | 0.494234 | 0.6211409 | 0.74122593 | FALSE |
| APCDD1L | 14185 | 0.494187 | 0.6211741 | 0.74122593 | FALSE |
| PRRG2 | 409 | 0.494054 | 0.621268 | 0.74122593 | FALSE |
| PMP2 | 5403 | 0.493364 | 0.6217554 | 0.7416376 | FALSE |
| SERPINB9P1 | 1704 | 0.492234 | 0.6225539 | 0.7423711 | FALSE |
| UPK2 | 1465 | 0.491393 | 0.6231485 | 0.74296086 | FALSE |
| GDI1 | 7161 | 0.491156 | 0.6233161 | 0.74308192 | FALSE |
| KDM5B | 6863 | 0.489906 | 0.6242004 | 0.74385502 | FALSE |
| YIF1A | 10659 | 0.48986 | 0.624233 | 0.74385502 | FALSE |
| MAP7D1 | 13736 | 0.489592 | 0.6244226 | 0.74402432 | FALSE |
| TAS2R60 | 3071 | 0.489023 | 0.6248254 | 0.74438549 | FALSE |
| GPAM | 12054 | 0.488962 | 0.6248686 | 0.74438549 | FALSE |
| PCLO | 5480 | 0.488857 | 0.6249429 | 0.74438823 | FALSE |
| OBSCN | 4201 | 0.48877 | 0.6250045 | 0.74438823 | FALSE |
| PGBD5 | 9811 | 0.488474 | 0.6252141 | 0.74457017 | FALSE |
| HAGLROS | 2210 | 0.487914 | 0.6256108 | 0.74487228 | FALSE |
| L1CAM | 6240 | 0.487435 | 0.6259501 | 0.74521955 | FALSE |
| METTL22 | 8249 | 0.487326 | 0.6260273 | 0.74523426 | FALSE |
| TCL6 | 3818 | 0.486996 | 0.6262612 | 0.74541958 | FALSE |
| CDK5R2 | 11392 | 0.486823 | 0.6263838 | 0.74550767 | FALSE |
| CDK7 | 11899 | 0.486403 | 0.6266815 | 0.74562142 | FALSE |
| NTN3 | 8221 | 0.485713 | 0.6271707 | 0.74599516 | FALSE |
| NADSYN1 | 12717 | 0.484716 | 0.6278778 | 0.74666164 | FALSE |
| OPLAH | 6640 | 0.483676 | 0.6286158 | 0.74747644 | FALSE |
| ALKBH1 | 7833 | 0.483481 | 0.6287542 | 0.74747644 | FALSE |
| DYNC2LI1 | 5900 | 0.483003 | 0.6290936 | 0.74782301 | FALSE |
| OLFM2 | 7562 | 0.48262 | 0.6293656 | 0.74808942 | FALSE |
| RAD1 | 4171 | 0.482062 | 0.6297619 | 0.7483898 | FALSE |
| CCDC30 | 12361 | 0.481619 | 0.6300766 | 0.74864459 | FALSE |
| PARD3 | 7133 | 0.481558 | 0.63012 | 0.74864459 | FALSE |
| ULBP1 | 13667 | 0.481461 | 0.6301889 | 0.74866958 | FALSE |
| PKP1 | 9330 | 0.481066 | 0.6304696 | 0.74888923 | FALSE |
| PARD6G-AS1 | 11627 | 0.480806 | 0.6306544 | 0.74901602 | FALSE |
| PRDM6 | 7589 | 0.480781 | 0.6306722 | 0.74901602 | FALSE |
| NDUFAF4P1 | 8959 | 0.480507 | 0.6308669 | 0.74913351 | FALSE |
| DIO3 | 9972 | 0.480145 | 0.6311243 | 0.74930737 | FALSE |
| LINC00475 | 811 | 0.480045 | 0.6311954 | 0.74930737 | FALSE |
| CECR2 | 2436 | 0.480029 | 0.6312068 | 0.74930737 | FALSE |
| PTGDS | 4043 | 0.479964 | 0.631253 | 0.74930737 | FALSE |
| MFSD2B | 7983 | 0.479756 | 0.6314009 | 0.74934601 | FALSE |
| MSH2 | 9510 | 0.479718 | 0.6314279 | 0.74934601 | FALSE |
| RAPGEF1 | 5350 | 0.479299 | 0.6317259 | 0.74947598 | FALSE |
| FAIM | 15565 | 0.479247 | 0.6317629 | 0.74947598 | FALSE |
| MAP2K4 | 1522 | 0.478329 | 0.6324161 | 0.75011838 | FALSE |
| ORAI2 | 9822 | 0.477049 | 0.6333272 | 0.7510721 | FALSE |
| BCL6 | 3056 | 0.477045 | 0.6333301 | 0.7510721 | FALSE |
| TMEM203 | 8799 | 0.476997 | 0.6333643 | 0.7510721 | FALSE |
| ODF3L1 | 5015 | 0.476637 | 0.6336206 | 0.75131912 | FALSE |
| PLEKHN1 | 4326 | 0.475744 | 0.6342568 | 0.75195936 | FALSE |
| FAM47C | 1635 | 0.47495 | 0.6348226 | 0.75245901 | FALSE |
| APH1A | 7396 | 0.474456 | 0.6351748 | 0.75281934 | FALSE |
| DKKL1 | 14529 | 0.473573 | 0.6358044 | 0.75339428 | FALSE |
| FMO5 | 13150 | 0.47342 | 0.6359136 | 0.7534613 | FALSE |
| NAF1 | 14330 | 0.47335 | 0.6359635 | 0.7534613 | FALSE |
| MAGED2 | 15223 | 0.473291 | 0.6360056 | 0.7534613 | FALSE |
| PKMYT1 | 13794 | 0.473151 | 0.6361055 | 0.75346626 | FALSE |
| KCTD7 | 10231 | 0.47315 | 0.6361062 | 0.75346626 | FALSE |
| GAS2L1 | 13328 | 0.472874 | 0.6363031 | 0.7535853 | FALSE |
| UBE2Z | 9124 | 0.472546 | 0.6365371 | 0.75380537 | FALSE |
| KIAA1217 | 8764 | 0.472163 | 0.6368104 | 0.75407194 | FALSE |
| TEP1 | 6209 | 0.47204 | 0.6368982 | 0.75411877 | FALSE |
| OTOP2 | 5137 | 0.471407 | 0.6373501 | 0.75448239 | FALSE |
| KRTAP1-3 | 8891 | 0.471011 | 0.6376329 | 0.75464568 | FALSE |
| HS3ST3A1 | 1504 | 0.469685 | 0.6385801 | 0.7555951 | FALSE |
| DTYMK | 13134 | 0.469072 | 0.6390182 | 0.75599902 | FALSE |
| ZNF488 | 10671 | 0.468723 | 0.6392676 | 0.7561225 | FALSE |
| C9orf24 | 1563 | 0.468473 | 0.6394464 | 0.75627668 | FALSE |
| PRIMA1 | 405 | 0.467445 | 0.6401815 | 0.75699491 | FALSE |
| SMC3 | 9874 | 0.467354 | 0.6402466 | 0.75699491 | FALSE |
| BARX1 | 1654 | 0.467353 | 0.6402473 | 0.75699491 | FALSE |
| OXSM | 6582 | 0.46716 | 0.6403854 | 0.75704729 | FALSE |
| INHBC | 14449 | 0.467088 | 0.6404369 | 0.75704729 | FALSE |
| GMNN | 5665 | 0.466529 | 0.6408369 | 0.75735785 | FALSE |
| SLC38A7 | 11513 | 0.466483 | 0.6408698 | 0.75735785 | FALSE |
| GSN-AS1 | 13250 | 0.46645 | 0.6408934 | 0.75735785 | FALSE |
| MAD2L1BP | 8620 | 0.466184 | 0.6410838 | 0.75744488 | FALSE |
| EEF1A1 | 7259 | 0.466144 | 0.6411124 | 0.75744488 | FALSE |
| CCDC159 | 11631 | 0.465986 | 0.6412255 | 0.75752124 | FALSE |
| DUSP5P1 | 552 | 0.465469 | 0.6415956 | 0.7579012 | FALSE |
| ALPP | 11853 | 0.465202 | 0.6417868 | 0.75806975 | FALSE |
| ZNF626 | 3771 | 0.465125 | 0.6418419 | 0.7580776 | FALSE |
| PLA2G16 | 12598 | 0.464325 | 0.6424149 | 0.7586397 | FALSE |
| TMEM132D | 2315 | 0.464123 | 0.6425596 | 0.75873447 | FALSE |
| HCN3 | 12025 | 0.463984 | 0.6426592 | 0.75873447 | FALSE |
| DBET | 5298 | 0.463942 | 0.6426893 | 0.75873447 | FALSE |
| GSDME | 239 | 0.463522 | 0.6429902 | 0.75897597 | FALSE |
| HTRA1 | 4467 | 0.463147 | 0.643259 | 0.75916439 | FALSE |
| RPS27L | 10372 | 0.463118 | 0.6432798 | 0.75916439 | FALSE |
| PDE6D | 2472 | 0.462386 | 0.6438045 | 0.75970685 | FALSE |
| FRMD6 | 13347 | 0.461453 | 0.6444736 | 0.76020949 | FALSE |
| TSSC2 | 12644 | 0.460723 | 0.6449974 | 0.76060023 | FALSE |
| STAT6 | 646 | 0.460611 | 0.6450777 | 0.76063509 | FALSE |
| YBX3P1 | 8514 | 0.459525 | 0.6458572 | 0.76117518 | FALSE |
| FAM214B | 6335 | 0.459524 | 0.6458579 | 0.76117518 | FALSE |
| RHOV | 13021 | 0.459498 | 0.6458766 | 0.76117518 | FALSE |
| TTC3P1 | 7276 | 0.458858 | 0.6463361 | 0.76148714 | FALSE |
| IGBP1 | 8857 | 0.458614 | 0.6465114 | 0.7616362 | FALSE |
| CHTF8 | 4278 | 0.458427 | 0.6466457 | 0.76173704 | FALSE |
| RRAGA | 6811 | 0.455876 | 0.6484791 | 0.76325025 | FALSE |
| CDV3 | 12524 | 0.455853 | 0.6484957 | 0.76325025 | FALSE |
| RNF222 | 9293 | 0.455642 | 0.6486474 | 0.76325025 | FALSE |
| LINC01144 | 13524 | 0.455318 | 0.6488805 | 0.7633613 | FALSE |
| LCN6 | 729 | 0.455286 | 0.6489035 | 0.7633613 | FALSE |
| MYT1 | 4072 | 0.455169 | 0.6489877 | 0.76337081 | FALSE |
| C11orf21 | 6404 | 0.454761 | 0.6492812 | 0.76357578 | FALSE |
| GEMIN6 | 1248 | 0.452979 | 0.6505639 | 0.76479658 | FALSE |
| FAM155B | 5402 | 0.452702 | 0.6507633 | 0.76491604 | FALSE |
| CRYBB3 | 13264 | 0.452182 | 0.6511379 | 0.76518367 | FALSE |
| CUL7 | 5174 | 0.451887 | 0.6513504 | 0.76531835 | FALSE |
| FBXW2 | 10387 | 0.451595 | 0.6515608 | 0.76550801 | FALSE |
| SERPINA3 | 9115 | 0.451367 | 0.6517251 | 0.76558596 | FALSE |
| KCNN1 | 9334 | 0.451186 | 0.6518555 | 0.76568165 | FALSE |
| PECAM1 | 5896 | 0.450626 | 0.6522591 | 0.76597211 | FALSE |
| MAMSTR | 10771 | 0.450571 | 0.6522988 | 0.76597211 | FALSE |
| KDM4B | 12682 | 0.450227 | 0.6525468 | 0.76620577 | FALSE |
| TMEM119 | 6820 | 0.449743 | 0.6528958 | 0.76646744 | FALSE |
| NDUFB3 | 13344 | 0.449692 | 0.6529325 | 0.76646744 | FALSE |
| PAXX | 9574 | 0.449554 | 0.6530321 | 0.76646744 | FALSE |
| RBPMS2 | 1663 | 0.44951 | 0.6530638 | 0.76646744 | FALSE |
| PINK1-AS | 347 | 0.449413 | 0.6531338 | 0.76647512 | FALSE |
| C8orf59 | 636 | 0.449347 | 0.6531814 | 0.76647512 | FALSE |
| RRM1 | 2095 | 0.449133 | 0.6533357 | 0.76649886 | FALSE |
| SPX | 14828 | 0.447975 | 0.6541712 | 0.76730635 | FALSE |
| RASGEF1B | 12323 | 0.44786 | 0.6542542 | 0.76734613 | FALSE |
| MALL | 10437 | 0.447553 | 0.6544758 | 0.76754844 | FALSE |
| RANGRF | 876 | 0.445687 | 0.6558234 | 0.76896757 | FALSE |
| DHRS4L1 | 2243 | 0.445673 | 0.6558335 | 0.76896757 | FALSE |
| TMEM95 | 1486 | 0.445301 | 0.6561022 | 0.76916733 | FALSE |
| ACTL6A | 9886 | 0.445095 | 0.6562511 | 0.76928415 | FALSE |
| PLEKHJ1 | 6041 | 0.444949 | 0.6563566 | 0.76935015 | FALSE |
| LOC645752 | 11983 | 0.444597 | 0.656611 | 0.76941759 | FALSE |
| AP4B1 | 13647 | 0.44398 | 0.657057 | 0.76988253 | FALSE |
| LCE5A | 9177 | 0.443565 | 0.6573571 | 0.77006098 | FALSE |
| DNAJB9 | 9145 | 0.442749 | 0.6579473 | 0.77046368 | FALSE |
| GHITM | 14676 | 0.442232 | 0.6583213 | 0.77084394 | FALSE |
| C3orf22 | 5013 | 0.441663 | 0.6587331 | 0.77115279 | FALSE |
| SP100 | 12321 | 0.441091 | 0.6591471 | 0.7715797 | FALSE |
| BTN2A1 | 2435 | 0.4403 | 0.6597198 | 0.7719091 | FALSE |
| NFYC-AS1 | 9037 | 0.440266 | 0.6597445 | 0.7719091 | FALSE |
| AAGAB | 14856 | 0.439614 | 0.6602167 | 0.7721957 | FALSE |
| NLRP11 | 14865 | 0.43934 | 0.6604152 | 0.77237007 | FALSE |
| ARL6IP4 | 11069 | 0.438944 | 0.6607021 | 0.77247447 | FALSE |
| SCAND1 | 9735 | 0.438854 | 0.6607673 | 0.77249295 | FALSE |
| HDGFL2 | 11399 | 0.438709 | 0.6608724 | 0.77249501 | FALSE |
| CILP | 1328 | 0.438415 | 0.6610855 | 0.77257601 | FALSE |
| OR10V1 | 3519 | 0.43823 | 0.6612196 | 0.77267495 | FALSE |
| OGFRL1 | 1270 | 0.438019 | 0.6613725 | 0.77273815 | FALSE |
| LAMP1 | 3631 | 0.437224 | 0.6619489 | 0.77329601 | FALSE |
| ZNF439 | 5761 | 0.436997 | 0.6621135 | 0.77343053 | FALSE |
| NOP14-AS1 | 12351 | 0.436767 | 0.6622803 | 0.77350688 | FALSE |
| DOLPP1 | 1647 | 0.43666 | 0.6623579 | 0.77350688 | FALSE |
| MEFV | 12636 | 0.436634 | 0.6623768 | 0.77350688 | FALSE |
| SNX8 | 8879 | 0.436229 | 0.6626706 | 0.77368335 | FALSE |
| UACA | 10164 | 0.436221 | 0.6626764 | 0.77368335 | FALSE |
| POLR2B | 14636 | 0.435618 | 0.6631139 | 0.77407854 | FALSE |
| PI4KB | 9634 | 0.435363 | 0.663299 | 0.77408453 | FALSE |
| GYPC | 15507 | 0.435344 | 0.6633128 | 0.77408453 | FALSE |
| XPO6 | 11240 | 0.43429 | 0.6640779 | 0.77468318 | FALSE |
| LCE1C | 2408 | 0.433461 | 0.6646799 | 0.7752382 | FALSE |
| UBE2B | 4346 | 0.432704 | 0.6652298 | 0.77567971 | FALSE |
| CHL1 | 9833 | 0.432435 | 0.6654253 | 0.77584975 | FALSE |
| LINC01278 | 5292 | 0.432209 | 0.6655895 | 0.77598032 | FALSE |
| SLC41A2 | 9178 | 0.432136 | 0.6656426 | 0.77598032 | FALSE |
| CUEDC1 | 10335 | 0.431684 | 0.6659711 | 0.77617779 | FALSE |
| SLC4A7 | 3843 | 0.431653 | 0.6659936 | 0.77617779 | FALSE |
| TMEM105 | 910 | 0.431638 | 0.6660045 | 0.77617779 | FALSE |
| GNAI1 | 54 | 0.431149 | 0.66636 | 0.77653421 | FALSE |
| POC5 | 12688 | 0.430197 | 0.6670523 | 0.77710927 | FALSE |
| GRIA2 | 6266 | 0.428872 | 0.6680164 | 0.77782746 | FALSE |
| TFCP2 | 731 | 0.428669 | 0.6681641 | 0.7779407 | FALSE |
| SOX17 | 15068 | 0.427473 | 0.6690348 | 0.77860706 | FALSE |
| CEACAM20 | 14674 | 0.427404 | 0.6690851 | 0.77860706 | FALSE |
| TMEM216 | 7614 | 0.427232 | 0.6692104 | 0.77869486 | FALSE |
| SLITRK5 | 8126 | 0.426537 | 0.6697166 | 0.77905198 | FALSE |
| XCR1 | 1938 | 0.426249 | 0.6699264 | 0.7791801 | FALSE |
| THBS1 | 12455 | 0.426152 | 0.6699971 | 0.77920432 | FALSE |
| VGF | 8353 | 0.425845 | 0.6702208 | 0.77934853 | FALSE |
| ARF4 | 12282 | 0.424794 | 0.6709869 | 0.7799194 | FALSE |
| HECTD3 | 2257 | 0.42444 | 0.671245 | 0.7799194 | FALSE |
| NDUFA12 | 14294 | 0.42438 | 0.6712887 | 0.7799194 | FALSE |
| C19orf47 | 9151 | 0.424348 | 0.671312 | 0.7799194 | FALSE |
| TAF7 | 3986 | 0.424072 | 0.6715133 | 0.77992329 | FALSE |
| PPP1R16A | 2350 | 0.423943 | 0.6716074 | 0.77996325 | FALSE |
| CABP4 | 1959 | 0.423435 | 0.6719779 | 0.78028899 | FALSE |
| TCF15 | 116 | 0.422217 | 0.6728666 | 0.78093611 | FALSE |
| LIG3 | 10965 | 0.422192 | 0.6728849 | 0.78093611 | FALSE |
| ZNF816 | 6866 | 0.421993 | 0.6730301 | 0.78098872 | FALSE |
| TBRG4 | 2209 | 0.421281 | 0.6735499 | 0.78137173 | FALSE |
| LMBRD1 | 6317 | 0.421267 | 0.6735601 | 0.78137173 | FALSE |
| PRR25 | 1754 | 0.420147 | 0.6743781 | 0.78208845 | FALSE |
| HSPA4 | 3336 | 0.41902 | 0.6752015 | 0.78269504 | FALSE |
| PHF23 | 1447 | 0.418907 | 0.6752841 | 0.78273273 | FALSE |
| ANKRD34A | 579 | 0.4187 | 0.6754354 | 0.78279202 | FALSE |
| COQ9 | 9617 | 0.417757 | 0.6761248 | 0.7832426 | FALSE |
| SH2D6 | 3509 | 0.41765 | 0.676203 | 0.7832752 | FALSE |
| WASF3 | 3221 | 0.417416 | 0.6763742 | 0.78336918 | FALSE |
| NUDC | 5362 | 0.41728 | 0.6764736 | 0.78341448 | FALSE |
| ITGA1 | 9042 | 0.416831 | 0.676802 | 0.78373675 | FALSE |
| CSNK2A2 | 13208 | 0.416751 | 0.6768605 | 0.78374646 | FALSE |
| SEZ6L2 | 2504 | 0.416064 | 0.6773632 | 0.78421228 | FALSE |
| FAM90A7P | 11618 | 0.415891 | 0.6774898 | 0.78426725 | FALSE |
| ZNF433 | 427 | 0.415862 | 0.677511 | 0.78426725 | FALSE |
| RPS4Y1 | 1960 | 0.415665 | 0.6776552 | 0.78437606 | FALSE |
| PPM1K | 14790 | 0.415548 | 0.6777408 | 0.78441709 | FALSE |
| GZMB | 3916 | 0.41532 | 0.6779077 | 0.78455215 | FALSE |
| MAP1S | 15065 | 0.414968 | 0.6781653 | 0.78479226 | FALSE |
| PNRC1 | 10819 | 0.414184 | 0.6787394 | 0.78518393 | FALSE |
| ENTPD5 | 8285 | 0.414083 | 0.6788133 | 0.78518393 | FALSE |
| AFF3 | 13940 | 0.414077 | 0.6788177 | 0.78518393 | FALSE |
| RBM18 | 10674 | 0.413974 | 0.6788931 | 0.78518393 | FALSE |
| MTSS1 | 4468 | 0.413793 | 0.6790257 | 0.78526475 | FALSE |
| SLC18A2 | 12580 | 0.412321 | 0.6801042 | 0.78628436 | FALSE |
| FBXL8 | 9996 | 0.412315 | 0.6801086 | 0.78628436 | FALSE |
| RPH3AL | 9224 | 0.412084 | 0.6802779 | 0.7863731 | FALSE |
| GNS | 10294 | 0.411365 | 0.6808049 | 0.78691484 | FALSE |
| NFE2L3 | 15313 | 0.410953 | 0.681107 | 0.7871397 | FALSE |
| EIF5A | 5423 | 0.410931 | 0.6811231 | 0.7871397 | FALSE |
| KLHDC7A | 7004 | 0.410878 | 0.681162 | 0.7871397 | FALSE |
| IFT140 | 12324 | 0.410825 | 0.6812009 | 0.7871397 | FALSE |
| MRGPRG-AS1 | 10613 | 0.410565 | 0.6813915 | 0.78730184 | FALSE |
| HMG20A | 5884 | 0.410049 | 0.68177 | 0.78756451 | FALSE |
| ZNF391 | 2368 | 0.409612 | 0.6820906 | 0.78781841 | FALSE |
| RWDD2A | 14422 | 0.409183 | 0.6824054 | 0.78806553 | FALSE |
| DYNLRB1 | 6053 | 0.408892 | 0.6826189 | 0.7881957 | FALSE |
| BCAS3 | 7753 | 0.408685 | 0.6827708 | 0.78831289 | FALSE |
| SPANXA2-OT1 | 10929 | 0.407831 | 0.6833978 | 0.78897844 | FALSE |
| COX11 | 4974 | 0.407658 | 0.6835248 | 0.78906682 | FALSE |
| FNDC9 | 14641 | 0.407013 | 0.6839984 | 0.78955531 | FALSE |
| AURKAIP1 | 9416 | 0.406918 | 0.6840682 | 0.78957756 | FALSE |
| NPW | 4407 | 0.406732 | 0.6842048 | 0.78967694 | FALSE |
| SLITRK2 | 10058 | 0.406044 | 0.6847103 | 0.79000598 | FALSE |
| MERTK | 10084 | 0.404946 | 0.6855172 | 0.79050776 | FALSE |
| VRK3 | 170 | 0.404891 | 0.6855576 | 0.79050776 | FALSE |
| ANKRD17 | 14578 | 0.404516 | 0.6858333 | 0.79068084 | FALSE |
| SLC25A20 | 1309 | 0.404182 | 0.6860789 | 0.79090563 | FALSE |
| CYB561D2 | 3142 | 0.40395 | 0.6862495 | 0.79104396 | FALSE |
| MUC2 | 3097 | 0.403415 | 0.686643 | 0.79132832 | FALSE |
| BRD4 | 7852 | 0.403025 | 0.6869298 | 0.79132832 | FALSE |
| NKX2-6 | 2175 | 0.40275 | 0.6871321 | 0.79132832 | FALSE |
| CALM2 | 13591 | 0.402728 | 0.6871483 | 0.79132832 | FALSE |
| PELP1 | 11908 | 0.40272 | 0.6871542 | 0.79132832 | FALSE |
| NAPSA | 3673 | 0.401851 | 0.6877937 | 0.79194805 | FALSE |
| POLH | 3672 | 0.40144 | 0.6880962 | 0.79217969 | FALSE |
| PRKG2 | 4300 | 0.401175 | 0.6882913 | 0.79234593 | FALSE |
| GGA1 | 11066 | 0.400337 | 0.6889083 | 0.79293946 | FALSE |
| SFT2D1 | 6813 | 0.399673 | 0.6893974 | 0.79344396 | FALSE |
| ARSF | 5099 | 0.39921 | 0.6897385 | 0.79371966 | FALSE |
| DHPS | 15599 | 0.398755 | 0.6900737 | 0.79404702 | FALSE |
| ARHGAP33 | 13842 | 0.398416 | 0.6903236 | 0.79421757 | FALSE |
| EMD | 9953 | 0.397647 | 0.6908904 | 0.79469427 | FALSE |
| ELN | 7320 | 0.39723 | 0.6911979 | 0.79493093 | FALSE |
| PARS2 | 5522 | 0.396699 | 0.6915894 | 0.79526427 | FALSE |
| OR4D2 | 12454 | 0.396098 | 0.6920327 | 0.79571548 | FALSE |
| HDHD2 | 15551 | 0.395861 | 0.6922076 | 0.79580115 | FALSE |
| PCAT6 | 11021 | 0.395689 | 0.6923345 | 0.79586052 | FALSE |
| GAPDH | 723 | 0.395608 | 0.6923942 | 0.79586052 | FALSE |
| NEUROG1 | 9235 | 0.395211 | 0.6926872 | 0.79593347 | FALSE |
| TRAF4 | 9485 | 0.395151 | 0.6927315 | 0.79593347 | FALSE |
| NR2F1-AS1 | 6012 | 0.395151 | 0.6927315 | 0.79593347 | FALSE |
| AGXT | 11993 | 0.394949 | 0.6928805 | 0.79604625 | FALSE |
| POLG | 7723 | 0.394549 | 0.6931758 | 0.79632691 | FALSE |
| RHOG | 12944 | 0.394343 | 0.6933278 | 0.7964083 | FALSE |
| HIST1H4F | 11426 | 0.392354 | 0.6947967 | 0.79783725 | FALSE |
| PLBD2 | 7297 | 0.392099 | 0.6949851 | 0.79794824 | FALSE |
| ELP4 | 5541 | 0.391709 | 0.6952732 | 0.79814999 | FALSE |
| QKI | 12216 | 0.38966 | 0.696788 | 0.79953659 | FALSE |
| DCX | 14880 | 0.389299 | 0.697055 | 0.79972556 | FALSE |
| PHF20 | 617 | 0.389213 | 0.6971186 | 0.79973985 | FALSE |
| EXTL3 | 15458 | 0.388653 | 0.6975329 | 0.80003897 | FALSE |
| CEP128 | 7765 | 0.38811 | 0.6979346 | 0.80037455 | FALSE |
| PPP1R8 | 6977 | 0.38805 | 0.697979 | 0.80037455 | FALSE |
| A3GALT2 | 13249 | 0.387275 | 0.6985526 | 0.8009148 | FALSE |
| MRPL17 | 1631 | 0.386699 | 0.6989791 | 0.80128618 | FALSE |
| SP140L | 11435 | 0.38621 | 0.6993411 | 0.80158371 | FALSE |
| MRPS9 | 5249 | 0.385628 | 0.6997722 | 0.80196866 | FALSE |
| MTCH2 | 13230 | 0.384865 | 0.7003374 | 0.80237268 | FALSE |
| SALL1 | 3649 | 0.384607 | 0.7005286 | 0.80253288 | FALSE |
| SH2D3C | 1206 | 0.384315 | 0.700745 | 0.80266313 | FALSE |
| FAM180A | 4998 | 0.383736 | 0.7011741 | 0.80296786 | FALSE |
| PLEKHG2 | 10247 | 0.383385 | 0.7014343 | 0.80298204 | FALSE |
| C19orf33 | 13844 | 0.382909 | 0.7017872 | 0.80332722 | FALSE |
| YIPF3 | 9876 | 0.382005 | 0.7024577 | 0.8039769 | FALSE |
| CSRP2 | 2280 | 0.381733 | 0.7026594 | 0.80414895 | FALSE |
| PARP9 | 448 | 0.380318 | 0.7037094 | 0.80523267 | FALSE |
| COPS5 | 9492 | 0.379804 | 0.7040909 | 0.80549577 | FALSE |
| B3GNT2 | 5981 | 0.3798 | 0.7040939 | 0.80549577 | FALSE |
| CLEC12A | 5524 | 0.378382 | 0.7051468 | 0.80622837 | FALSE |
| GIMAP8 | 9873 | 0.37785 | 0.705542 | 0.80656223 | FALSE |
| CRTC1 | 8945 | 0.37772 | 0.7056386 | 0.80661366 | FALSE |
| SOS2 | 3794 | 0.377492 | 0.705808 | 0.80674831 | FALSE |
| LRFN1 | 9273 | 0.376992 | 0.7061796 | 0.80711397 | FALSE |
| RNF41 | 5360 | 0.376877 | 0.706265 | 0.80712336 | FALSE |
| ZNF682 | 2539 | 0.376607 | 0.7064657 | 0.80720495 | FALSE |
| SRSF2 | 9686 | 0.376203 | 0.706766 | 0.80741184 | FALSE |
| FAM204A | 10692 | 0.376107 | 0.7068374 | 0.80741184 | FALSE |
| GTPBP8 | 11087 | 0.376083 | 0.7068552 | 0.80741184 | FALSE |
| SIMC1 | 11100 | 0.376016 | 0.706905 | 0.80741184 | FALSE |
| RASGRF2 | 11133 | 0.375921 | 0.7069756 | 0.80742423 | FALSE |
| GAN | 13532 | 0.375793 | 0.7070708 | 0.80742423 | FALSE |
| JHDM1D-AS1 | 12683 | 0.374696 | 0.7078866 | 0.80811967 | FALSE |
| NDRG2 | 9944 | 0.374418 | 0.7080934 | 0.80823769 | FALSE |
| RAB11FIP1 | 7970 | 0.37426 | 0.7082109 | 0.80827208 | FALSE |
| PPP1R9B | 3095 | 0.374124 | 0.7083121 | 0.80827208 | FALSE |
| RGS2 | 10082 | 0.373636 | 0.7086751 | 0.80848855 | FALSE |
| GSTM4 | 4370 | 0.373444 | 0.708818 | 0.80855475 | FALSE |
| KLLN | 11314 | 0.373419 | 0.7088366 | 0.80855475 | FALSE |
| RIOK1 | 9878 | 0.372834 | 0.709272 | 0.80887771 | FALSE |
| TLK1 | 5614 | 0.371774 | 0.7100611 | 0.80953804 | FALSE |
| NFS1 | 5028 | 0.371363 | 0.7103672 | 0.80976886 | FALSE |
| MIF4GD | 4782 | 0.371118 | 0.7105496 | 0.8099178 | FALSE |
| AKIRIN1 | 2763 | 0.370545 | 0.7109765 | 0.81028613 | FALSE |
| CHUK | 12888 | 0.370312 | 0.71115 | 0.810339 | FALSE |
| POC1B-AS1 | 1259 | 0.370274 | 0.7111783 | 0.810339 | FALSE |
| TNNI1 | 6277 | 0.370102 | 0.7113065 | 0.81036688 | FALSE |
| SYN1 | 12679 | 0.369413 | 0.7118199 | 0.81066321 | FALSE |
| WDR61 | 5660 | 0.369405 | 0.7118259 | 0.81066321 | FALSE |
| ACP2 | 3368 | 0.368799 | 0.7122776 | 0.81111206 | FALSE |
| EIF3B | 6870 | 0.368129 | 0.712777 | 0.81140996 | FALSE |
| NINJ1 | 6399 | 0.368038 | 0.7128449 | 0.81140996 | FALSE |
| IGSF8 | 3971 | 0.367654 | 0.7131312 | 0.8116177 | FALSE |
| ZNF808 | 13934 | 0.366935 | 0.7136675 | 0.81199156 | FALSE |
| DHRS4-AS1 | 4256 | 0.366358 | 0.714098 | 0.81235943 | FALSE |
| SP140 | 1434 | 0.36626 | 0.7141711 | 0.81235943 | FALSE |
| SPEM2 | 12121 | 0.366223 | 0.7141987 | 0.81235943 | FALSE |
| RFC2 | 480 | 0.36604 | 0.7143352 | 0.81245563 | FALSE |
| TMEM41A | 142 | 0.365852 | 0.7144755 | 0.81249696 | FALSE |
| CEBPE | 15533 | 0.36561 | 0.7146561 | 0.81258496 | FALSE |
| C11orf94 | 1507 | 0.365609 | 0.7146569 | 0.81258496 | FALSE |
| KLF3-AS1 | 12857 | 0.364435 | 0.7155332 | 0.81334477 | FALSE |
| HEBP2 | 6092 | 0.364317 | 0.7156213 | 0.81338577 | FALSE |
| HSF2 | 8042 | 0.363984 | 0.71587 | 0.81355009 | FALSE |
| LPCAT1 | 2559 | 0.363461 | 0.7162605 | 0.81385578 | FALSE |
| EFL1 | 12586 | 0.36335 | 0.7163434 | 0.81385578 | FALSE |
| OR10A5 | 8232 | 0.363345 | 0.7163472 | 0.81385578 | FALSE |
| MBD1 | 7039 | 0.363207 | 0.7164503 | 0.81391373 | FALSE |
| CAT | 5401 | 0.363 | 0.7166049 | 0.81397109 | FALSE |
| CYP1B1 | 2625 | 0.362361 | 0.7170823 | 0.81445418 | FALSE |
| TPGS1 | 3652 | 0.361359 | 0.7178311 | 0.81512701 | FALSE |
| CYP4A11 | 11093 | 0.360914 | 0.7181637 | 0.81538628 | FALSE |
| ZNHIT2 | 12212 | 0.360788 | 0.7182579 | 0.81543401 | FALSE |
| CRIPT | 6732 | 0.360504 | 0.7184703 | 0.81555661 | FALSE |
| TBL3 | 7698 | 0.36024 | 0.7186677 | 0.81568515 | FALSE |
| YIPF6 | 9281 | 0.35964 | 0.7191164 | 0.81603723 | FALSE |
| GDPD3 | 4818 | 0.359576 | 0.7191642 | 0.81603723 | FALSE |
| INTS3 | 14702 | 0.359519 | 0.7192069 | 0.81603723 | FALSE |
| ZNF227 | 681 | 0.359257 | 0.7194028 | 0.8160865 | FALSE |
| BNIPL | 2952 | 0.359141 | 0.7194896 | 0.8160865 | FALSE |
| HIST3H3 | 12932 | 0.359114 | 0.7195098 | 0.8160865 | FALSE |
| STRA6 | 11606 | 0.357572 | 0.7206636 | 0.81699231 | FALSE |
| DYNC1H1 | 9222 | 0.357486 | 0.720728 | 0.81699231 | FALSE |
| ZDHHC8P1 | 6659 | 0.357275 | 0.7208859 | 0.81705285 | FALSE |
| DLD | 901 | 0.35707 | 0.7210394 | 0.81716754 | FALSE |
| SLC25A21-AS1 | 3073 | 0.356551 | 0.721428 | 0.81754863 | FALSE |
| DPM3 | 13531 | 0.355892 | 0.7219214 | 0.81796308 | FALSE |
| STK33 | 4893 | 0.355853 | 0.7219507 | 0.81796308 | FALSE |
| PROCR | 8149 | 0.35569 | 0.7220727 | 0.81801095 | FALSE |
| NEK3 | 4862 | 0.355645 | 0.7221064 | 0.81801095 | FALSE |
| LGALSL | 8782 | 0.35517 | 0.7224622 | 0.81830548 | FALSE |
| KBTBD13 | 9774 | 0.354825 | 0.7227207 | 0.81847962 | FALSE |
| MUT | 10945 | 0.354646 | 0.7228548 | 0.81851291 | FALSE |
| ZNF589 | 2594 | 0.354453 | 0.7229994 | 0.81858523 | FALSE |
| NPY4R | 10580 | 0.354124 | 0.723246 | 0.8187779 | FALSE |
| CTNNBL1 | 8341 | 0.353746 | 0.7235292 | 0.81902496 | FALSE |
| ADGRB1 | 15279 | 0.353693 | 0.723569 | 0.81902496 | FALSE |
| LPIN3 | 13805 | 0.353197 | 0.7239408 | 0.81937975 | FALSE |
| PNRC2 | 9907 | 0.353135 | 0.7239872 | 0.81937975 | FALSE |
| MAFK | 14054 | 0.352906 | 0.7241589 | 0.81944613 | FALSE |
| SNAI3-AS1 | 13608 | 0.352847 | 0.7242031 | 0.81944613 | FALSE |
| AGPAT4 | 2904 | 0.352716 | 0.7243014 | 0.81949124 | FALSE |
| PEF1 | 3036 | 0.352232 | 0.7246643 | 0.81969764 | FALSE |
| PABPC4 | 12595 | 0.352131 | 0.72474 | 0.81969764 | FALSE |
| SDC2 | 4502 | 0.351766 | 0.7250138 | 0.81988861 | FALSE |
| LOC440300 | 2222 | 0.3509 | 0.7256634 | 0.82038585 | FALSE |
| BBS12 | 4092 | 0.350756 | 0.7257714 | 0.82044865 | FALSE |
| MIOX | 4650 | 0.35059 | 0.725896 | 0.82047079 | FALSE |
| CFAP65 | 12191 | 0.349276 | 0.7268821 | 0.82128853 | FALSE |
[truncated: 561,182 more chars]
