# Supplementary material for: Brain tissue electrical conductivity as a promising biomarker for dementia assessment using MRI
Source: Alzheimers Dement. 2025 Jun 23;21(6):e70270. doi: 10.1002/alz.70270 (PMC12185248; doi:10.1002/alz.70270)
Supplement: Supplementary file 5 — Supporting Information [file ALZ-21-e70270-s009.docx]

**Tables S18.** Full list of the GO terms associated with upweighted genes from PLS2 of conductivity difference between Dementia and cognitively normal participants.

| **term_name** | **term_id** | **adjusted_p_value** | **term_size** | **query_size** | **effective_domain_size** |
| --- | --- | --- | --- | --- | --- |
| regulation of localization | GO:0032879 | 1.96E-11 | 2018 | 2403 | 21031 |
| cell-cell signaling | GO:0007267 | 4.17E-10 | 1704 | 2403 | 21031 |
| organonitrogen compound biosynthetic process | GO:1901566 | 6.55E-10 | 1761 | 2403 | 21031 |
| regulation of cellular component organization | GO:0051128 | 8.76E-10 | 2425 | 2403 | 21031 |
| trans-synaptic signaling | GO:0099537 | 1.74E-09 | 756 | 2403 | 21031 |
| synaptic signaling | GO:0099536 | 2.77E-09 | 780 | 2403 | 21031 |
| cell projection organization | GO:0030030 | 4.27E-09 | 1613 | 2403 | 21031 |
| regulation of transport | GO:0051049 | 5.20E-09 | 1594 | 2403 | 21031 |
| anterograde trans-synaptic signaling | GO:0098916 | 7.67E-09 | 750 | 2403 | 21031 |
| chemical synaptic transmission | GO:0007268 | 7.67E-09 | 750 | 2403 | 21031 |
| plasma membrane bounded cell projection organization | GO:0120036 | 1.25E-08 | 1570 | 2403 | 21031 |
| carbohydrate derivative metabolic process | GO:1901135 | 3.17E-08 | 1091 | 2403 | 21031 |
| regulation of protein localization | GO:0032880 | 9.05E-08 | 889 | 2403 | 21031 |
| organic substance transport | GO:0071702 | 1.79E-07 | 2358 | 2403 | 21031 |
| regulation of cellular localization | GO:0060341 | 2.10E-07 | 995 | 2403 | 21031 |
| establishment of localization in cell | GO:0051649 | 2.75E-07 | 1972 | 2403 | 21031 |
| cellular response to organic cyclic compound | GO:0071407 | 2.78E-07 | 535 | 2403 | 21031 |
| neurogenesis | GO:0022008 | 4.71E-07 | 1742 | 2403 | 21031 |
| regulation of trans-synaptic signaling | GO:0099177 | 5.73E-07 | 490 | 2403 | 21031 |
| nitrogen compound transport | GO:0071705 | 6.28E-07 | 1911 | 2403 | 21031 |
| modulation of chemical synaptic transmission | GO:0050804 | 1.10E-06 | 489 | 2403 | 21031 |
| organelle assembly | GO:0070925 | 1.10E-06 | 1001 | 2403 | 21031 |
| neuron development | GO:0048666 | 1.39E-06 | 1167 | 2403 | 21031 |
| generation of neurons | GO:0048699 | 2.06E-06 | 1514 | 2403 | 21031 |
| head development | GO:0060322 | 3.92E-06 | 787 | 2403 | 21031 |
| organic substance catabolic process | GO:1901575 | 4.36E-06 | 2074 | 2403 | 21031 |
| regulation of establishment of protein localization | GO:0070201 | 6.50E-06 | 537 | 2403 | 21031 |
| regulation of developmental process | GO:0050793 | 8.14E-06 | 2455 | 2403 | 21031 |
| small molecule metabolic process | GO:0044281 | 8.30E-06 | 1813 | 2403 | 21031 |
| regulation of protein metabolic process | GO:0051246 | 8.56E-06 | 2102 | 2403 | 21031 |
| carbohydrate derivative biosynthetic process | GO:1901137 | 9.97E-06 | 661 | 2403 | 21031 |
| intracellular transport | GO:0046907 | 1.10E-05 | 1371 | 2403 | 21031 |
| protein transport | GO:0015031 | 1.21E-05 | 1424 | 2403 | 21031 |
| central nervous system development | GO:0007417 | 1.51E-05 | 1035 | 2403 | 21031 |
| cytoskeleton organization | GO:0007010 | 1.81E-05 | 1512 | 2403 | 21031 |
| brain development | GO:0007420 | 1.86E-05 | 736 | 2403 | 21031 |
| neuron differentiation | GO:0030182 | 1.87E-05 | 1431 | 2403 | 21031 |
| response to endogenous stimulus | GO:0009719 | 1.99E-05 | 1678 | 2403 | 21031 |
| neuron projection development | GO:0031175 | 2.63E-05 | 1014 | 2403 | 21031 |
| cellular response to organic substance | GO:0071310 | 2.66E-05 | 1971 | 2403 | 21031 |
| signal release | GO:0023061 | 2.91E-05 | 492 | 2403 | 21031 |
| negative regulation of response to stimulus | GO:0048585 | 3.06E-05 | 1648 | 2403 | 21031 |
| macromolecule catabolic process | GO:0009057 | 3.56E-05 | 1338 | 2403 | 21031 |
| regulation of anatomical structure morphogenesis | GO:0022603 | 4.93E-05 | 838 | 2403 | 21031 |
| regulation of secretion by cell | GO:1903530 | 5.29E-05 | 564 | 2403 | 21031 |
| regulation of molecular function | GO:0065009 | 6.96E-05 | 1799 | 2403 | 21031 |
| export from cell | GO:0140352 | 0.000139211 | 894 | 2403 | 21031 |
| secretion by cell | GO:0032940 | 0.000187478 | 827 | 2403 | 21031 |
| cell morphogenesis | GO:0000902 | 0.000213544 | 986 | 2403 | 21031 |
| positive regulation of cell communication | GO:0010647 | 0.000237878 | 1777 | 2403 | 21031 |
| positive regulation of signaling | GO:0023056 | 0.000250307 | 1778 | 2403 | 21031 |
| response to metal ion | GO:0010038 | 0.000403946 | 360 | 2403 | 21031 |
| cellular response to endogenous stimulus | GO:0071495 | 0.000423739 | 1432 | 2403 | 21031 |
| regulation of intracellular signal transduction | GO:1902531 | 0.000434273 | 1766 | 2403 | 21031 |
| telencephalon development | GO:0021537 | 0.000448098 | 279 | 2403 | 21031 |
| regulation of protein transport | GO:0051223 | 0.000498221 | 440 | 2403 | 21031 |
| response to organic cyclic compound | GO:0014070 | 0.000513704 | 905 | 2403 | 21031 |
| cellular response to oxygen-containing compound | GO:1901701 | 0.000666841 | 1179 | 2403 | 21031 |
| organophosphate metabolic process | GO:0019637 | 0.000755748 | 1034 | 2403 | 21031 |
| response to oxygen-containing compound | GO:1901700 | 0.00077687 | 1663 | 2403 | 21031 |
| ribonucleotide metabolic process | GO:0009259 | 0.000777433 | 471 | 2403 | 21031 |
| ribose phosphate metabolic process | GO:0019693 | 0.000823643 | 485 | 2403 | 21031 |
| regulation of plasma membrane bounded cell projection organization | GO:0120035 | 0.001030547 | 652 | 2403 | 21031 |
| cellular catabolic process | GO:0044248 | 0.001031786 | 1600 | 2403 | 21031 |
| amide biosynthetic process | GO:0043604 | 0.001086281 | 887 | 2403 | 21031 |
| translation | GO:0006412 | 0.001100248 | 723 | 2403 | 21031 |
| cell junction organization | GO:0034330 | 0.001151871 | 759 | 2403 | 21031 |
| purine ribonucleotide metabolic process | GO:0009150 | 0.001365248 | 450 | 2403 | 21031 |
| regulation of neuron projection development | GO:0010975 | 0.001406954 | 457 | 2403 | 21031 |
| cytoplasmic translation | GO:0002181 | 0.001497845 | 156 | 2403 | 21031 |
| regulation of organelle organization | GO:0033043 | 0.001746573 | 1173 | 2403 | 21031 |
| organonitrogen compound catabolic process | GO:1901565 | 0.001862863 | 1399 | 2403 | 21031 |
| regulation of cell projection organization | GO:0031344 | 0.001946739 | 667 | 2403 | 21031 |
| negative regulation of nitrogen compound metabolic process | GO:0051172 | 0.001999338 | 2202 | 2403 | 21031 |
| regulation of secretion | GO:0051046 | 0.002109112 | 619 | 2403 | 21031 |
| response to calcium ion | GO:0051592 | 0.002280274 | 147 | 2403 | 21031 |
| neuron projection morphogenesis | GO:0048812 | 0.002284882 | 655 | 2403 | 21031 |
| actin filament-based process | GO:0030029 | 0.002420144 | 805 | 2403 | 21031 |
| cell death | GO:0008219 | 0.002448283 | 1988 | 2403 | 21031 |
| positive regulation of protein localization | GO:1903829 | 0.002477298 | 490 | 2403 | 21031 |
| regulation of catabolic process | GO:0009894 | 0.002880001 | 1026 | 2403 | 21031 |
| microtubule-based transport | GO:0099111 | 0.002929267 | 213 | 2403 | 21031 |
| cell-cell adhesion | GO:0098609 | 0.003011673 | 946 | 2403 | 21031 |
| intracellular signaling cassette | GO:0141124 | 0.003066444 | 1861 | 2403 | 21031 |
| positive regulation of cellular component organization | GO:0051130 | 0.003103156 | 1116 | 2403 | 21031 |
| negative regulation of response to external stimulus | GO:0032102 | 0.003120239 | 405 | 2403 | 21031 |
| peptide biosynthetic process | GO:0043043 | 0.003285979 | 752 | 2403 | 21031 |
| regulation of cellular component biogenesis | GO:0044087 | 0.003643428 | 971 | 2403 | 21031 |
| positive regulation of establishment of protein localization | GO:1904951 | 0.003770212 | 328 | 2403 | 21031 |
| cell projection morphogenesis | GO:0048858 | 0.003877218 | 676 | 2403 | 21031 |
| programmed cell death | GO:0012501 | 0.004061361 | 1984 | 2403 | 21031 |
| cellular anatomical entity morphogenesis | GO:0032989 | 0.004446138 | 785 | 2403 | 21031 |
| response to inorganic substance | GO:0010035 | 0.004493399 | 531 | 2403 | 21031 |
| plasma membrane bounded cell projection morphogenesis | GO:0120039 | 0.004532042 | 671 | 2403 | 21031 |
| negative regulation of molecular function | GO:0044092 | 0.004883765 | 672 | 2403 | 21031 |
| synapse organization | GO:0050808 | 0.005382903 | 485 | 2403 | 21031 |
| cellular response to metal ion | GO:0071248 | 0.006175047 | 200 | 2403 | 21031 |
| metal ion transport | GO:0030001 | 0.006209319 | 877 | 2403 | 21031 |
| glycoprotein metabolic process | GO:0009100 | 0.006216578 | 372 | 2403 | 21031 |
| regulation of membrane potential | GO:0042391 | 0.006691121 | 433 | 2403 | 21031 |
| cellular response to stress | GO:0033554 | 0.006738846 | 1770 | 2403 | 21031 |
| regulation of neurotransmitter transport | GO:0051588 | 0.007451476 | 98 | 2403 | 21031 |
| protein catabolic process | GO:0030163 | 0.007469029 | 968 | 2403 | 21031 |
| cell adhesion | GO:0007155 | 0.008163153 | 1512 | 2403 | 21031 |
| negative regulation of protein metabolic process | GO:0051248 | 0.008706895 | 766 | 2403 | 21031 |
| cellular response to hormone stimulus | GO:0032870 | 0.008729598 | 609 | 2403 | 21031 |
| non-membrane-bounded organelle assembly | GO:0140694 | 0.008859864 | 409 | 2403 | 21031 |
| regulation of locomotion | GO:0040012 | 0.009111445 | 1038 | 2403 | 21031 |
| leukocyte cell-cell adhesion | GO:0007159 | 0.009495932 | 403 | 2403 | 21031 |
| actin cytoskeleton organization | GO:0030036 | 0.009578923 | 717 | 2403 | 21031 |
| response to steroid hormone | GO:0048545 | 0.010007098 | 337 | 2403 | 21031 |
| transmembrane transport | GO:0055085 | 0.011184324 | 1534 | 2403 | 21031 |
| homeostatic process | GO:0042592 | 0.011386574 | 1712 | 2403 | 21031 |
| leukocyte activation | GO:0045321 | 0.011729839 | 946 | 2403 | 21031 |
| negative regulation of multicellular organismal process | GO:0051241 | 0.011971477 | 1110 | 2403 | 21031 |
| response to hormone | GO:0009725 | 0.012925992 | 874 | 2403 | 21031 |
| monoatomic ion transport | GO:0006811 | 0.013925251 | 1256 | 2403 | 21031 |
| potassium ion transmembrane transport | GO:0071805 | 0.014042018 | 212 | 2403 | 21031 |
| response to abiotic stimulus | GO:0009628 | 0.0141008 | 1128 | 2403 | 21031 |
| pallium development | GO:0021543 | 0.014872238 | 194 | 2403 | 21031 |
| synaptic vesicle exocytosis | GO:0016079 | 0.015404474 | 96 | 2403 | 21031 |
| negative regulation of signaling | GO:0023057 | 0.017765385 | 1398 | 2403 | 21031 |
| negative regulation of cell communication | GO:0010648 | 0.017765385 | 1398 | 2403 | 21031 |
| forebrain development | GO:0030900 | 0.018602852 | 417 | 2403 | 21031 |
| positive regulation of response to stimulus | GO:0048584 | 0.019007238 | 2274 | 2403 | 21031 |
| cell motility | GO:0048870 | 0.019712889 | 1709 | 2403 | 21031 |
| membrane organization | GO:0061024 | 0.019910528 | 815 | 2403 | 21031 |
| vesicle-mediated transport | GO:0016192 | 0.02036455 | 1547 | 2403 | 21031 |
| apoptotic process | GO:0006915 | 0.020500992 | 1913 | 2403 | 21031 |
| secretion | GO:0046903 | 0.02059872 | 963 | 2403 | 21031 |
| glycoprotein biosynthetic process | GO:0009101 | 0.020612082 | 311 | 2403 | 21031 |
| regulation of response to stress | GO:0080134 | 0.020696975 | 1340 | 2403 | 21031 |
| dephosphorylation | GO:0016311 | 0.020816151 | 298 | 2403 | 21031 |
| regulation of transmembrane transport | GO:0034762 | 0.021529141 | 446 | 2403 | 21031 |
| response to organonitrogen compound | GO:0010243 | 0.021818042 | 964 | 2403 | 21031 |
| mitochondrion organization | GO:0007005 | 0.022649958 | 495 | 2403 | 21031 |
| regulation of response to external stimulus | GO:0032101 | 0.023018393 | 1077 | 2403 | 21031 |
| nucleotide metabolic process | GO:0009117 | 0.023528755 | 601 | 2403 | 21031 |
| nucleoside phosphate metabolic process | GO:0006753 | 0.025101085 | 609 | 2403 | 21031 |
| response to mechanical stimulus | GO:0009612 | 0.027048783 | 217 | 2403 | 21031 |
| negative regulation of cellular component organization | GO:0051129 | 0.029203947 | 719 | 2403 | 21031 |
| regulation of binding | GO:0051098 | 0.029565814 | 243 | 2403 | 21031 |
| positive regulation of transport | GO:0051050 | 0.03063511 | 844 | 2403 | 21031 |
| response to nitrogen compound | GO:1901698 | 0.031004063 | 1060 | 2403 | 21031 |
| regulation of cell migration | GO:0030334 | 0.032884478 | 934 | 2403 | 21031 |
| cell migration | GO:0016477 | 0.033132231 | 1496 | 2403 | 21031 |
| cell activation | GO:0001775 | 0.034301202 | 1092 | 2403 | 21031 |
| response to lipid | GO:0033993 | 0.034548971 | 920 | 2403 | 21031 |
| negative regulation of locomotion | GO:0040013 | 0.034841427 | 336 | 2403 | 21031 |
| regulation of cell morphogenesis | GO:0022604 | 0.037455189 | 245 | 2403 | 21031 |
| regulation of cell motility | GO:2000145 | 0.037456518 | 996 | 2403 | 21031 |
| cerebellum development | GO:0021549 | 0.038436024 | 106 | 2403 | 21031 |
| amide metabolic process | GO:0043603 | 0.04122073 | 1194 | 2403 | 21031 |
| monoatomic ion transmembrane transport | GO:0034220 | 0.041804182 | 1013 | 2403 | 21031 |
| cellular response to lipid | GO:0071396 | 0.041843303 | 609 | 2403 | 21031 |
| locomotion | GO:0040011 | 0.045325339 | 1234 | 2403 | 21031 |
| cellular response to steroid hormone stimulus | GO:0071383 | 0.046465021 | 215 | 2403 | 21031 |
| endosomal transport | GO:0016197 | 0.04720811 | 286 | 2403 | 21031 |
| regulation of cell population proliferation | GO:0042127 | 0.047563147 | 1683 | 2403 | 21031 |
| peptide metabolic process | GO:0006518 | 0.048292215 | 911 | 2403 | 21031 |
| positive regulation of multicellular organismal process | GO:0051240 | 0.049196874 | 1637 | 2403 | 21031 |
| transport along microtubule | GO:0010970 | 0.049325184 | 172 | 2403 | 21031 |
