# Supplementary material for: Brain tissue electrical conductivity as a promising biomarker for dementia assessment using MRI
Source: Alzheimers Dement. 2025 Jun 23;21(6):e70270. doi: 10.1002/alz.70270 (PMC12185248; doi:10.1002/alz.70270)
Supplement: Supplementary file 7 — Supporting Information [file ALZ-21-e70270-s012.docx]

**Tables S20.** Full list of the GO terms associated with upweighted genes from PLS2 of tau SUVRs difference between Dementia and cognitively normal participants.

| term_name | term_id | adjusted_p_value | term_size | query_size | effective_domain_size |
| --- | --- | --- | --- | --- | --- |
| regulation of localization | GO:0032879 | 2.70E-27 | 2018 | 3825 | 21031 |
| cell-cell signaling | GO:0007267 | 2.53E-26 | 1704 | 3825 | 21031 |
| regulation of transport | GO:0051049 | 7.31E-23 | 1594 | 3825 | 21031 |
| neurogenesis | GO:0022008 | 1.02E-21 | 1742 | 3825 | 21031 |
| positive regulation of cell communication | GO:0010647 | 5.46E-21 | 1777 | 3825 | 21031 |
| positive regulation of signaling | GO:0023056 | 6.23E-21 | 1778 | 3825 | 21031 |
| organic substance transport | GO:0071702 | 6.64E-21 | 2358 | 3825 | 21031 |
| neuron development | GO:0048666 | 8.39E-21 | 1167 | 3825 | 21031 |
| generation of neurons | GO:0048699 | 2.24E-20 | 1514 | 3825 | 21031 |
| regulation of cellular component organization | GO:0051128 | 3.79E-20 | 2425 | 3825 | 21031 |
| cell projection organization | GO:0030030 | 4.69E-20 | 1613 | 3825 | 21031 |
| regulation of developmental process | GO:0050793 | 7.73E-20 | 2455 | 3825 | 21031 |
| plasma membrane bounded cell projection organization | GO:0120036 | 1.24E-18 | 1570 | 3825 | 21031 |
| intracellular signaling cassette | GO:0141124 | 2.25E-18 | 1861 | 3825 | 21031 |
| neuron differentiation | GO:0030182 | 4.04E-18 | 1431 | 3825 | 21031 |
| establishment of localization in cell | GO:0051649 | 7.00E-18 | 1972 | 3825 | 21031 |
| response to endogenous stimulus | GO:0009719 | 2.10E-17 | 1678 | 3825 | 21031 |
| cell morphogenesis | GO:0000902 | 6.43E-17 | 986 | 3825 | 21031 |
| regulation of intracellular signal transduction | GO:1902531 | 7.31E-17 | 1766 | 3825 | 21031 |
| neuron projection development | GO:0031175 | 1.84E-16 | 1014 | 3825 | 21031 |
| nitrogen compound transport | GO:0071705 | 2.63E-16 | 1911 | 3825 | 21031 |
| positive regulation of response to stimulus | GO:0048584 | 2.22E-15 | 2274 | 3825 | 21031 |
| cellular response to endogenous stimulus | GO:0071495 | 3.14E-15 | 1432 | 3825 | 21031 |
| regulation of cellular localization | GO:0060341 | 4.06E-15 | 995 | 3825 | 21031 |
| cellular response to organic substance | GO:0071310 | 5.24E-15 | 1971 | 3825 | 21031 |
| organonitrogen compound biosynthetic process | GO:1901566 | 1.42E-14 | 1761 | 3825 | 21031 |
| positive regulation of signal transduction | GO:0009967 | 3.42E-14 | 1561 | 3825 | 21031 |
| protein transport | GO:0015031 | 3.43E-14 | 1424 | 3825 | 21031 |
| regulation of protein localization | GO:0032880 | 4.08E-14 | 889 | 3825 | 21031 |
| positive regulation of multicellular organismal process | GO:0051240 | 3.54E-13 | 1637 | 3825 | 21031 |
| regulation of molecular function | GO:0065009 | 5.13E-13 | 1799 | 3825 | 21031 |
| regulation of protein metabolic process | GO:0051246 | 5.50E-13 | 2102 | 3825 | 21031 |
| regulation of anatomical structure morphogenesis | GO:0022603 | 6.66E-13 | 838 | 3825 | 21031 |
| export from cell | GO:0140352 | 1.24E-12 | 894 | 3825 | 21031 |
| transmembrane transport | GO:0055085 | 2.42E-12 | 1534 | 3825 | 21031 |
| positive regulation of developmental process | GO:0051094 | 2.63E-12 | 1328 | 3825 | 21031 |
| small molecule metabolic process | GO:0044281 | 3.48E-12 | 1813 | 3825 | 21031 |
| intracellular transport | GO:0046907 | 9.45E-12 | 1371 | 3825 | 21031 |
| vesicle-mediated transport | GO:0016192 | 9.92E-12 | 1547 | 3825 | 21031 |
| central nervous system development | GO:0007417 | 1.46E-11 | 1035 | 3825 | 21031 |
| metal ion transport | GO:0030001 | 1.69E-11 | 877 | 3825 | 21031 |
| homeostatic process | GO:0042592 | 1.86E-11 | 1712 | 3825 | 21031 |
| response to organic cyclic compound | GO:0014070 | 2.15E-11 | 905 | 3825 | 21031 |
| response to organonitrogen compound | GO:0010243 | 2.69E-11 | 964 | 3825 | 21031 |
| response to oxygen-containing compound | GO:1901700 | 3.91E-11 | 1663 | 3825 | 21031 |
| positive regulation of transport | GO:0051050 | 4.22E-11 | 844 | 3825 | 21031 |
| monoatomic ion transport | GO:0006811 | 7.36E-11 | 1256 | 3825 | 21031 |
| monoatomic ion transmembrane transport | GO:0034220 | 1.55E-10 | 1013 | 3825 | 21031 |
| secretion by cell | GO:0032940 | 1.59E-10 | 827 | 3825 | 21031 |
| cell population proliferation | GO:0008283 | 2.57E-10 | 2006 | 3825 | 21031 |
| response to nitrogen compound | GO:1901698 | 3.50E-10 | 1060 | 3825 | 21031 |
| cellular response to oxygen-containing compound | GO:1901701 | 3.89E-10 | 1179 | 3825 | 21031 |
| cytoskeleton organization | GO:0007010 | 6.11E-10 | 1512 | 3825 | 21031 |
| regulation of protein modification process | GO:0031399 | 6.26E-10 | 1229 | 3825 | 21031 |
| regulation of multicellular organismal development | GO:2000026 | 7.37E-10 | 1411 | 3825 | 21031 |
| cellular response to stress | GO:0033554 | 8.03E-10 | 1770 | 3825 | 21031 |
| positive regulation of intracellular signal transduction | GO:1902533 | 1.84E-09 | 1033 | 3825 | 21031 |
| cell migration | GO:0016477 | 2.05E-09 | 1496 | 3825 | 21031 |
| negative regulation of cell communication | GO:0010648 | 3.42E-09 | 1398 | 3825 | 21031 |
| negative regulation of signaling | GO:0023057 | 3.42E-09 | 1398 | 3825 | 21031 |
| negative regulation of response to stimulus | GO:0048585 | 3.49E-09 | 1648 | 3825 | 21031 |
| inorganic ion transmembrane transport | GO:0098660 | 4.15E-09 | 900 | 3825 | 21031 |
| monoatomic cation transport | GO:0006812 | 5.40E-09 | 1033 | 3825 | 21031 |
| regulation of cell population proliferation | GO:0042127 | 6.31E-09 | 1683 | 3825 | 21031 |
| cell motility | GO:0048870 | 7.66E-09 | 1709 | 3825 | 21031 |
| inorganic cation transmembrane transport | GO:0098662 | 9.09E-09 | 808 | 3825 | 21031 |
| response to lipid | GO:0033993 | 9.40E-09 | 920 | 3825 | 21031 |
| membrane organization | GO:0061024 | 1.27E-08 | 815 | 3825 | 21031 |
| locomotion | GO:0040011 | 1.31E-08 | 1234 | 3825 | 21031 |
| response to hormone | GO:0009725 | 1.44E-08 | 874 | 3825 | 21031 |
| negative regulation of nitrogen compound metabolic process | GO:0051172 | 2.52E-08 | 2202 | 3825 | 21031 |
| regulation of locomotion | GO:0040012 | 2.74E-08 | 1038 | 3825 | 21031 |
| monoatomic cation transmembrane transport | GO:0098655 | 2.78E-08 | 830 | 3825 | 21031 |
| phosphorylation | GO:0016310 | 3.79E-08 | 1579 | 3825 | 21031 |
| regulation of cell development | GO:0060284 | 5.58E-08 | 849 | 3825 | 21031 |
| negative regulation of multicellular organismal process | GO:0051241 | 6.33E-08 | 1110 | 3825 | 21031 |
| import into cell | GO:0098657 | 6.89E-08 | 923 | 3825 | 21031 |
| actin filament-based process | GO:0030029 | 1.09E-07 | 805 | 3825 | 21031 |
| cellular homeostasis | GO:0019725 | 1.68E-07 | 831 | 3825 | 21031 |
| positive regulation of cellular component organization | GO:0051130 | 1.93E-07 | 1116 | 3825 | 21031 |
| circulatory system development | GO:0072359 | 1.95E-07 | 1130 | 3825 | 21031 |
| positive regulation of cell differentiation | GO:0045597 | 1.99E-07 | 864 | 3825 | 21031 |
| cell adhesion | GO:0007155 | 2.03E-07 | 1512 | 3825 | 21031 |
| secretion | GO:0046903 | 2.91E-07 | 963 | 3825 | 21031 |
| organic substance catabolic process | GO:1901575 | 3.21E-07 | 2074 | 3825 | 21031 |
| negative regulation of signal transduction | GO:0009968 | 3.37E-07 | 1290 | 3825 | 21031 |
| protein phosphorylation | GO:0006468 | 4.92E-07 | 1346 | 3825 | 21031 |
| regulation of cell motility | GO:2000145 | 5.26E-07 | 996 | 3825 | 21031 |
| regulation of cell differentiation | GO:0045595 | 5.41E-07 | 1576 | 3825 | 21031 |
| regulation of phosphate metabolic process | GO:0019220 | 6.13E-07 | 1132 | 3825 | 21031 |
| regulation of phosphorus metabolic process | GO:0051174 | 6.77E-07 | 1133 | 3825 | 21031 |
| regulation of catalytic activity | GO:0050790 | 6.96E-07 | 1227 | 3825 | 21031 |
| cell death | GO:0008219 | 7.81E-07 | 1988 | 3825 | 21031 |
| protein modification by small protein conjugation | GO:0032446 | 8.19E-07 | 858 | 3825 | 21031 |
| regulation of cell migration | GO:0030334 | 1.12E-06 | 934 | 3825 | 21031 |
| positive regulation of protein metabolic process | GO:0051247 | 1.36E-06 | 1253 | 3825 | 21031 |
| carbohydrate derivative metabolic process | GO:1901135 | 1.72E-06 | 1091 | 3825 | 21031 |
| programmed cell death | GO:0012501 | 1.72E-06 | 1984 | 3825 | 21031 |
| regulation of cellular component biogenesis | GO:0044087 | 1.86E-06 | 971 | 3825 | 21031 |
| cell-cell adhesion | GO:0098609 | 2.48E-06 | 946 | 3825 | 21031 |
| cellular catabolic process | GO:0044248 | 2.63E-06 | 1600 | 3825 | 21031 |
| regulation of catabolic process | GO:0009894 | 4.47E-06 | 1026 | 3825 | 21031 |
| enzyme-linked receptor protein signaling pathway | GO:0007167 | 5.19E-06 | 967 | 3825 | 21031 |
| organophosphate metabolic process | GO:0019637 | 6.21E-06 | 1034 | 3825 | 21031 |
| response to abiotic stimulus | GO:0009628 | 6.32E-06 | 1128 | 3825 | 21031 |
| macromolecule catabolic process | GO:0009057 | 7.06E-06 | 1338 | 3825 | 21031 |
| positive regulation of molecular function | GO:0044093 | 7.59E-06 | 1097 | 3825 | 21031 |
| regulation of response to stress | GO:0080134 | 8.37E-06 | 1340 | 3825 | 21031 |
| establishment of protein localization | GO:0045184 | 9.33E-06 | 1924 | 3825 | 21031 |
| post-translational protein modification | GO:0043687 | 1.70E-05 | 1021 | 3825 | 21031 |
| protein modification by small protein conjugation or removal | GO:0070647 | 1.78E-05 | 970 | 3825 | 21031 |
| regulation of organelle organization | GO:0033043 | 1.87E-05 | 1173 | 3825 | 21031 |
| apoptotic process | GO:0006915 | 2.42E-05 | 1913 | 3825 | 21031 |
| chemical homeostasis | GO:0048878 | 4.36E-05 | 1031 | 3825 | 21031 |
| organonitrogen compound catabolic process | GO:1901565 | 6.59E-05 | 1399 | 3825 | 21031 |
| positive regulation of nucleobase-containing compound metabolic process | GO:0045935 | 6.67E-05 | 2052 | 3825 | 21031 |
| protein catabolic process | GO:0030163 | 0.000136797 | 968 | 3825 | 21031 |
| positive regulation of RNA metabolic process | GO:0051254 | 0.000158857 | 1852 | 3825 | 21031 |
| mitotic cell cycle | GO:0000278 | 0.000167864 | 895 | 3825 | 21031 |
| amide metabolic process | GO:0043603 | 0.000169006 | 1194 | 3825 | 21031 |
| regulation of phosphorylation | GO:0042325 | 0.000175399 | 966 | 3825 | 21031 |
| tube development | GO:0035295 | 0.000203397 | 1091 | 3825 | 21031 |
| amide biosynthetic process | GO:0043604 | 0.000297665 | 887 | 3825 | 21031 |
| growth | GO:0040007 | 0.000303524 | 939 | 3825 | 21031 |
| organelle assembly | GO:0070925 | 0.000316783 | 1001 | 3825 | 21031 |
| regulation of protein phosphorylation | GO:0001932 | 0.000437509 | 910 | 3825 | 21031 |
| cell cycle | GO:0007049 | 0.000465619 | 1819 | 3825 | 21031 |
| lipid metabolic process | GO:0006629 | 0.000537533 | 1388 | 3825 | 21031 |
| leukocyte activation | GO:0045321 | 0.000567921 | 946 | 3825 | 21031 |
| negative regulation of developmental process | GO:0051093 | 0.000617232 | 928 | 3825 | 21031 |
| positive regulation of cell population proliferation | GO:0008284 | 0.000663301 | 962 | 3825 | 21031 |
| regulation of response to external stimulus | GO:0032101 | 0.000693139 | 1077 | 3825 | 21031 |
| tissue development | GO:0009888 | 0.000701628 | 2010 | 3825 | 21031 |
| peptide metabolic process | GO:0006518 | 0.001113307 | 911 | 3825 | 21031 |
| carboxylic acid metabolic process | GO:0019752 | 0.002042392 | 899 | 3825 | 21031 |
| anatomical structure formation involved in morphogenesis | GO:0048646 | 0.002134266 | 1183 | 3825 | 21031 |
| cell activation | GO:0001775 | 0.00332061 | 1092 | 3825 | 21031 |
| organic acid metabolic process | GO:0006082 | 0.004325305 | 927 | 3825 | 21031 |
| negative regulation of DNA-templated transcription | GO:0045892 | 0.005469128 | 1333 | 3825 | 21031 |
| multicellular organismal-level homeostasis | GO:0048871 | 0.005485508 | 806 | 3825 | 21031 |
| oxoacid metabolic process | GO:0043436 | 0.00578979 | 921 | 3825 | 21031 |
| positive regulation of RNA biosynthetic process | GO:1902680 | 0.005962144 | 1706 | 3825 | 21031 |
| regulation of immune system process | GO:0002682 | 0.005965707 | 1512 | 3825 | 21031 |
| positive regulation of DNA-templated transcription | GO:0045893 | 0.006710909 | 1703 | 3825 | 21031 |
| supramolecular fiber organization | GO:0097435 | 0.006942125 | 842 | 3825 | 21031 |
| microtubule-based process | GO:0007017 | 0.007482673 | 953 | 3825 | 21031 |
| negative regulation of RNA biosynthetic process | GO:1902679 | 0.009988478 | 1347 | 3825 | 21031 |
| tube morphogenesis | GO:0035239 | 0.011509282 | 872 | 3825 | 21031 |
| cellular lipid metabolic process | GO:0044255 | 0.014405381 | 1005 | 3825 | 21031 |
| negative regulation of nucleobase-containing compound metabolic process | GO:0045934 | 0.028759318 | 1588 | 3825 | 21031 |
| negative regulation of RNA metabolic process | GO:0051253 | 0.038314635 | 1463 | 3825 | 21031 |
| hemopoiesis | GO:0030097 | 0.038435812 | 965 | 3825 | 21031 |
| negative regulation of transcription by RNA polymerase II | GO:0000122 | 0.042895467 | 986 | 3825 | 21031 |
| positive regulation of gene expression | GO:0010628 | 0.048806133 | 1189 | 3825 | 21031 |
