# Supplementary material for: Brain tissue electrical conductivity as a promising biomarker for dementia assessment using MRI
Source: Alzheimers Dement. 2025 Jun 23;21(6):e70270. doi: 10.1002/alz.70270 (PMC12185248; doi:10.1002/alz.70270)
Supplement: Supplementary file 8 — Supporting Information [file ALZ-21-e70270-s004.docx]

**Tables S21.** Full list of the GO terms associated with the upweighted genes from the control spatial-spin null analyses of conductivity.

| term_name | term_id | adjusted_p_value | term_size | query_size | effective_domain_size |
| --- | --- | --- | --- | --- | --- |
| smoothened signaling pathway | GO:0007224 | 0.00452 | 151 | 305 | 21031 |
| cell-cell signaling | GO:0007267 | 0.013918 | 1704 | 305 | 21031 |
