# Supplementary material for: Brain tissue electrical conductivity as a promising biomarker for dementia assessment using MRI
Source: Alzheimers Dement. 2025 Jun 23;21(6):e70270. doi: 10.1002/alz.70270 (PMC12185248; doi:10.1002/alz.70270)
Supplement: Supplementary file 9 — Supporting Information [file ALZ-21-e70270-s003.docx]

**Tables S22.** Full list of the GO terms associated with the upweighted genes from the control spatial-spin null analyses of Aβ SUVRs.

| term_name | term_id | adjusted_p_value | term_size | query_size | effective_domain_size |
| --- | --- | --- | --- | --- | --- |
| regulation of biological quality | GO:0065008 | 0.005944 | 2846 | 84 | 21031 |
