# Supplementary material for: Brain tissue electrical conductivity as a promising biomarker for dementia assessment using MRI
Source: Alzheimers Dement. 2025 Jun 23;21(6):e70270. doi: 10.1002/alz.70270 (PMC12185248; doi:10.1002/alz.70270)
Supplement: Supplementary file 10 — Supporting Information [file ALZ-21-e70270-s005.docx]

**Tables S23.** Full list of the GO terms associated with the upweighted genes from the control spatial-spin null analyses of tau SUVRs.

| term_name | term_id | adjusted_p_value | term_size | query_size | effective_domain_size |
| --- | --- | --- | --- | --- | --- |
| system development | GO:0048731 | 0.001429 | 3973 | 129 | 21031 |
| developmental process | GO:0032502 | 0.003947 | 6453 | 129 | 21031 |
| multicellular organism development | GO:0007275 | 0.004113 | 4643 | 129 | 21031 |
| nervous system development | GO:0007399 | 0.004258 | 2531 | 129 | 21031 |
| regulation of cation channel activity | GO:2001257 | 0.004938 | 100 | 129 | 21031 |
| lipid modification | GO:0030258 | 0.044247 | 192 | 129 | 21031 |
| regulation of monoatomic ion transmembrane transporter activity | GO:0032412 | 0.044247 | 192 | 129 | 21031 |
| transport | GO:0006810 | 0.046036 | 4350 | 129 | 21031 |
| regulation of growth | GO:0040008 | 0.049911 | 620 | 129 | 21031 |
