# Supplementary material for: Brain tissue electrical conductivity as a promising biomarker for dementia assessment using MRI
Source: Alzheimers Dement. 2025 Jun 23;21(6):e70270. doi: 10.1002/alz.70270 (PMC12185248; doi:10.1002/alz.70270)
Supplement: Supplementary file 11 — Supporting Information [file ALZ-21-e70270-s001.docx]

**Supplementary Figure**

**
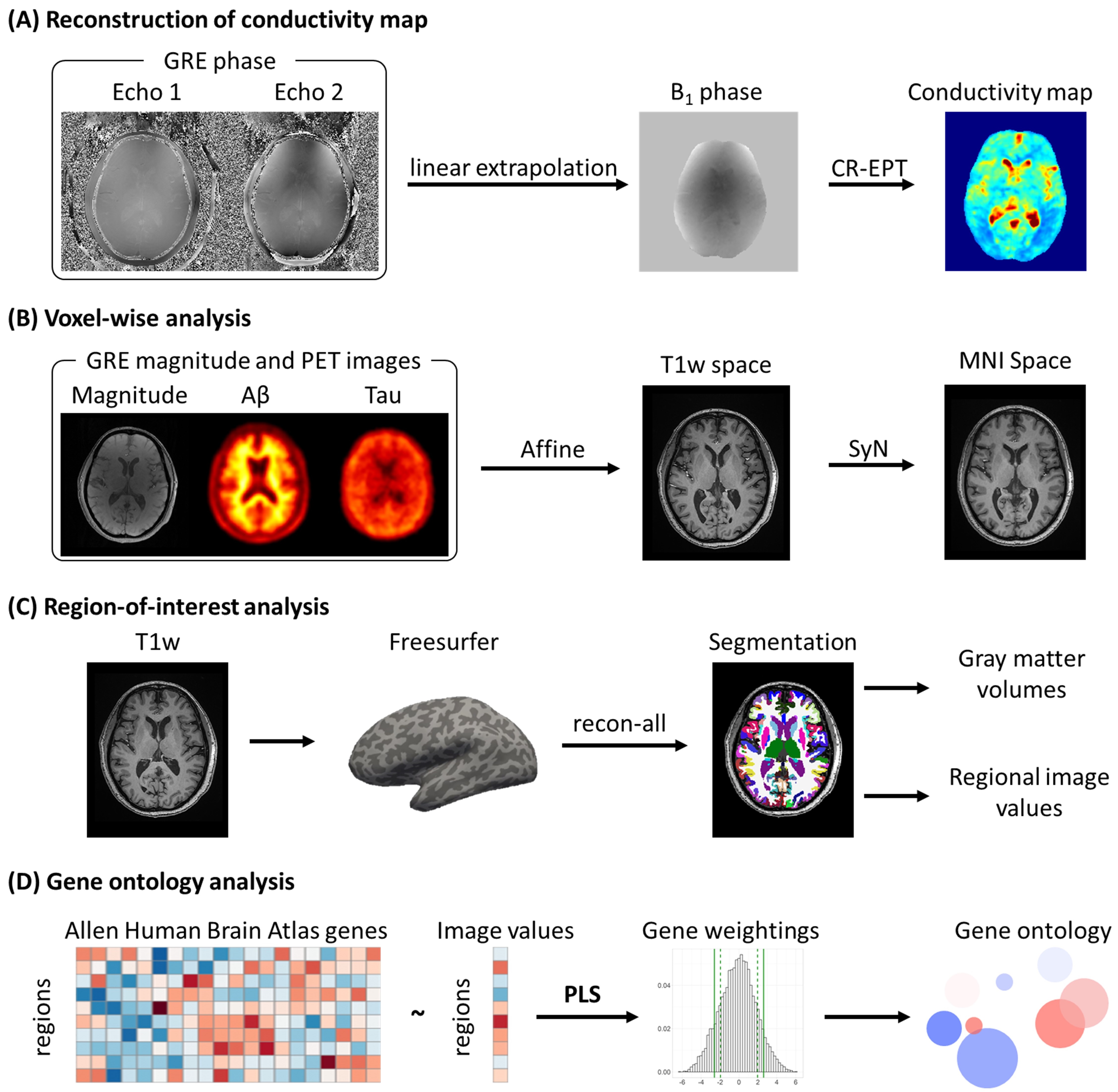
**

**Supplementary Figure 1. Methodology approach of this study. A,** The major steps for brain electrical conductivity reconstruction; **B,** The major steps of image preprocessing for voxel-wise analysis in MNI space; **C,** The major steps of image preprocessing for region-of-interest group analysis; **D,** The major steps for gene ontology analysis.

*Notes*. CR-EPT, convection-reaction electrical properties tomography; GRE, gradient-recalled echo; PET, positron emission computed tomography; PLS, partial least squares.
